# Comparative Genomics of Syntrophic Branched-Chain Fatty Acid Degrading Bacteria

**DOI:** 10.1264/jsme2.ME16057

**Published:** 2016-07-16

**Authors:** Takashi Narihiro, Masaru K. Nobu, Hideyuki Tamaki, Yoichi Kamagata, Yuji Sekiguchi, Wen-Tso Liu

**Affiliations:** 1 Bioproduction Research Institute, National Institute of Advanced Industrial Science and Technology (AIST)Central 6, Higashi 1–1–1, Tsukuba, Ibaraki 305–8566Japan; 2 Department of Civil and Environmental Engineering, University of Illinois at Urbana-Champaign205 North Mathews Ave, Urbana, IL 61801USA; 3 Biotechnology Research Institute, The University of Tokyo1–1–1 Yayoi, Bunkyo-ku, Tokyo 113–8657Japan; 4 Biomedical Research Institute, National Institute of Advanced Industrial Science and Technology (AIST)Central 6, Higashi 1–1–1, Tsukuba, Ibaraki 305–8566Japan

**Keywords:** syntroph, branched-chain fatty acid, genomics, energy conservation

## Abstract

The syntrophic degradation of branched-chain fatty acids (BCFAs) such as 2-methylbutyrate and isobutyrate is an essential step in the production of methane from proteins/amino acids in anaerobic ecosystems. While a few syntrophic BCFA-degrading bacteria have been isolated, their metabolic pathways in BCFA and short-chain fatty acid (SCFA) degradation as well as energy conservation systems remain unclear. In an attempt to identify these pathways, we herein performed comparative genomics of three syntrophic bacteria: 2-methylbutyrate-degrading “*Syntrophomonas wolfei* subsp. *methylbutyratica*” strain JCM 14075^T^ (=4J5^T^), isobutyrate-degrading *Syntrophothermus lipocalidus* strain TGB-C1^T^, and non-BCFA-metabolizing *S. wolfei* subsp. *wolfei* strain Göttingen^T^. We demonstrated that 4J5 and TGB-C1 both encode multiple genes/gene clusters involved in β-oxidation, as observed in the Göttingen genome, which has multiple copies of genes associated with butyrate degradation. The 4J5 genome possesses phylogenetically distinct β-oxidation genes, which may be involved in 2-methylbutyrate degradation. In addition, these *Syntrophomonadaceae* strains harbor various hydrogen/formate generation systems (*i.e.*, electron-bifurcating hydrogenase, formate dehydrogenase, and membrane-bound hydrogenase) and energy-conserving electron transport systems, including electron transfer flavoprotein (ETF)-linked acyl-CoA dehydrogenase, ETF-linked iron-sulfur binding reductase, ETF dehydrogenase (FixABCX), and flavin oxidoreductase-heterodisulfide reductase (Flox-Hdr). Unexpectedly, the TGB-C1 genome encodes a nitrogenase complex, which may function as an alternative H_2_ generation mechanism. These results suggest that the BCFA-degrading syntrophic strains 4J5 and TGB-C1 possess specific β-oxidation-related enzymes for BCFA oxidation as well as appropriate energy conservation systems to perform thermodynamically unfavorable syntrophic metabolism.

Under methanogenic conditions, the degradation of amino acids and proteinaceous materials inevitably generates fatty acids as byproducts (26). Fatty acid-oxidizing bacteria and methanogens are known to form syntrophic interactions in order to accomplish the endergonic oxidation of these fatty acids (9, 19, 26). Although the biochemical pathways and genes involved in the syntrophic degradation of short-chain fatty acids (SCFA; *e.g.*, propionate and butyrate) have already been described, they have not yet been elucidated for branched-chain fatty acids (BCFAs; *e.g.*, isobutyrate, isovalerate, and 2-methylbutyrate) derived from branched-chain amino acids (13, 26). Syntrophic BCFA degradation to acetate and propionate has been observed in isolates and mixed cultures (14, 26, 34, 35, 41). Only three strains of the family *Syntrophomonadaceae* are currently known to syntrophically degrade 2-methylbutyrate (“*Syntrophomonas wolfei* subsp. *methylbutyratica*” strain JCM 14075^T^ (=4J5^T^) and *S. bryantii* strain CuCal^T^) and isobutyrate (*Syntrophothermus lipocalidus* strain TGB-C1^T^) (29, 33, 40). These *Syntrophomonadaceae* species are considered to be important for fatty acid degradation in anaerobic ecosystems, including the sludge digestion process (19), rice paddy fields (12), and the termite gut (42). Furthermore, an uncultivated *Syntrophaceae* member has been proposed to degrade BCFA syntrophically in a methanogenic bioreactor through metagenomic and metatranscriptomic approaches (21). However, the key catabolic enzymes and energy conservation systems necessary to drive thermodynamically unfavorable BCFA and SCFA degradation remain unclear.

In the present study, the genomes of strains 4J5 (20) and TGB-C1 (4) were investigated in order to identify the metabolic pathways for 2-methylbutyrate and isobutyrate catabolism and energy conservation systems for syntrophic metabolism. A comparative genomic analysis between BCFA-and non-BCFA-degrading syntrophs within the family *Syntrophomonadaceae* (*i.e.*, *S. wolfei* subsp. *wolfei* strain Göttingen^T^ [30]) provides genomic insights into the degradation of BCFA in methanogenic ecosystems.

## Materials and Methods

### Genome sequencing and annotation

This study analyzed the “*S. wolfei* subsp. *methylbutyratica*” strain JCM 14075^T^ (=4J5^T^) draft genome (DDBJ/GenBank/EMBL accession: BBQT01000001–BBQT01000092) (20), *S. lipocalidus* strain TGB-C1^T^ complete genome (CP002048) (4), and *S. wolfei* strain Göttingen complete genome (CP000448) (30). As reported previously (20), the genomic DNA of strain 4J5 was provided by the RIKEN BRC through the National Bio-Resource Project of MEXT, Japan, and sequenced using the Illumina MiSeq platform (Illumina, San Diego, CA, USA) at FASMAC (Atsugi, Japan). Briefly, we constructed and sequenced a 300-bp paired-end library totaling *ca.* 2.2 Gb of MiSeq data. Assemblies were performed using SPAdes version 3.1.1 (2). The strain 4J5 draft genome comprises 89 scaffolds and has an estimated genome size of 3.2 Mbp with an average G+C content of 45.55%. The quality of the genome sequence was evaluated using the Check M version 1.0.5 program with a marker gene set of the class *Clostridia* (23). A total of 2,964 protein coding genes were annotated with Prokka version 1.11 (see Supplemental Information) (28). Basic local alignment search tool (BLAST) (ver. 2.2.30) with a non-redundant protein sequence database (nr) and the protein sequence database of Göttingen and TGB-C1 (11) and BLASTKoala of Kyoto Encyclopedia of Genes and Genomes (KEGG) (8) were used to search for functional domains and characterize potential protein functions. Proteins associated with energy conservation systems were identified by criteria based on the genomic and physiological information of previously reported energy conservation pathways (21). Transport systems were identified using TransportDB (25).

## Results and Discussion

### BCFA degradation

**Fig. 1:**
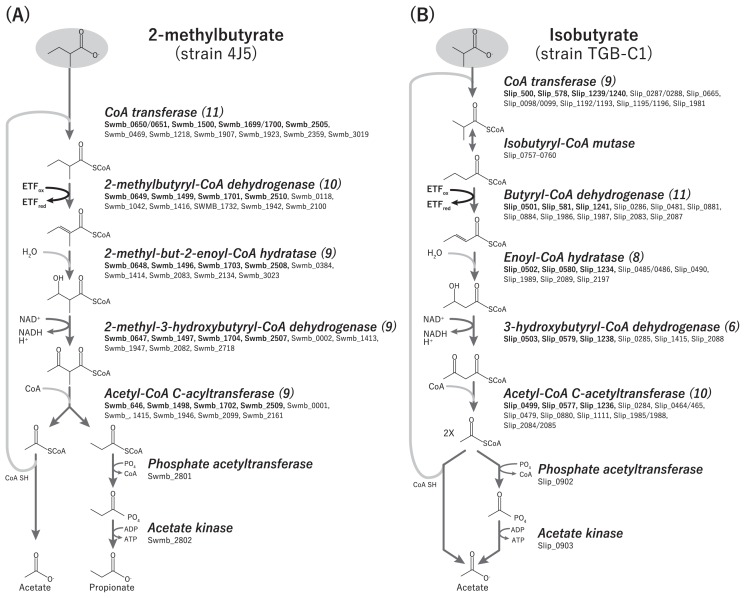
Branched-chain fatty acid metabolism in (A) “*Syntrophomonas wolfei* subsp. *methylbutyratica*” strain 4J5 and (B) *Syntrophothermus lipocalidus* strain TGB-C1. Each reaction is labeled with the protein name (italicized) of the gene encoding the function and locus tag(s). Figures in brackets indicate the number of multiple gene copies. Four (Swmb_00646–00651, Swmb1699–1704, Swmb_1496–1500, and Swmb_2505–02510) and three (Slip_0499–0503, Slip_0577–0581, and Slip_1236–1241) β-oxidation gene clusters were identified in strains 4J5 and TGB-C1, respectively (shown in bold face).

The *Syntrophomonadaceae* strains 4J5 (40), TGB-C1 (29), and Göttingen (1, 15, 16) have very similar genetic compositions (see Supplemental information, Table S1, S2, and S3), reflecting previously observed physiological similarities, such as central metabolic pathways (*e.g.*, glycolysis and the TCA cycle) and pilus and flagellum formation. Syntrophic BCFA degradation is considered to proceed through β-oxidation (13, 21), as observed for butyrate degradation by the strain Göttingen (16, 27, 30, 38). These *Syntrophomonadaceae* genomes all encode multiple genes for β-oxidation (Fig. 1, Table S4), potentially with varying specificities to particular fatty acid structures (*i.e.*, alkyl branching, chain length, and saturation), affinities to specific concentration ranges, or adaptation to other environmental conditions (30). Strain 4J5 possesses several β-oxidation genes with high similarities (>90%) to those in Göttingen, presumably involved in butyrate metabolism (Table S4, Fig. S1) (27, 32). However, we also identified several strain 4J5 β-oxidation-related genes with relatively low similarities to those of Göttingen (42.3%–62.6% amino acid identity), which may specifically be involved in 2-methylbutyrate degradation—acyl-CoA dehydrogenase (Swmb_1942), enoyl-CoA hydratases (Swmb_01703 and Swmb_03023), 3-hydroxybutyryl-CoA dehydrogenase (Swmb_01947), and acetyl-CoA C-acetyltransferase (Swmb_02509). The TGB-C1 genome encodes several β-oxidation genes not only with relatively high similarities (30–85%), but also with no significant similarity (<30%) to those in the mesophilic strains Göttingen and 4J5 (Table S5), implying that TGB-C1 drives the β-oxidation pathway by using thermostable/thermophilic enzymes. Further biochemical, transcriptomic, and/or proteomic analyses are needed in order to clarify the activities and specificities of the β-oxidation-related enzymes of these strains.

In syntrophic isobutyrate degradation, strain TGB-C1 has been reported to perform alkyl isomerization in order to form butyrate for subsequent β-oxidation (29). Strain TGB-C1 encodes an isobutyryl-CoA mutase gene cluster including the C- and N-terminal domain proteins, cob(I)alamin adenosyltransferase and MeaB-like protein (Slip_0757–0760) to facilitate the rearrangement step (Fig. 1B, Table S5). Homologous gene clusters have been found in “*Ca.* Caldisyntrophus multiacidovorans”, a syntrophic BCFA degrader in a lab-scale anaerobic bioreactor (21) and other prokaryotes (Fig. S2). The isobutyryl-CoA mutase genes of TGB-C1 and “*Ca.* Caldisyntrophus” are phylogenetically related to a group associated with sulfate-reducing bacteria (Fig. S2), suggesting potential evolutionary relationships between isobutyrate-isomerizing syntrophs and sulfate reducers.

As final products of the predicted β-oxidation pathways, the syntrophic degradation of isobutyrate generates two acetyl-CoA and 2-methylbutyrate produces acetyl-CoA and propionyl-CoA (Fig. 1A). Acetyl-CoA yields ATP through dethiolation to acetate by phosphate acetyltransferase (Swmb_02801 and Slip_0902) and acetate kinase (Swmb_02802 and Slip_0903). Regarding strain 4J5, these enzymes may perform the dethiolation of 2-methylbutyrate-derived propionyl-CoA because the active site structure of previously known propionate kinase resembles those of acetate kinase and butyrate kinase (7). AMP-dependent acyl-CoA synthetases found in the 4J5 and TGB-C1 genomes (Swmb_02363 and Swmb_02710; Slip_0475, Slip_0583, and Slip_1686) potentially serve as an alternative acyl-CoA degradation pathway, as suggested by McInerney *et al.* (17). However, Swmb_02710 and Slip_1686 have high identities (>64% by amino acid sequences) to that of strain Göttingen (Swol_1180), which has been predicted to function in biosynthesis (30). The other homolog found in 4J5 (Swmb_02363) may be involved in poly-β-hydroxybutyrate metabolism due to an association with the poly-β-hydroxybutyrate polymerase gene, as observed in strain Göttingen (Swol_1144). The remaining TGB-C1 homologs (Slip_0475 and Slip_0583) have low amino acid sequence identities (<32%) with the biosynthesis-associated acyl-CoA synthetase (Table S4), implying that these acyl-CoA synthetase genes are responsible for the production of acetate from acetyl-CoA through the degradation of isobutyrate.

### Energy conservation and electron flow

**Fig. 2:**
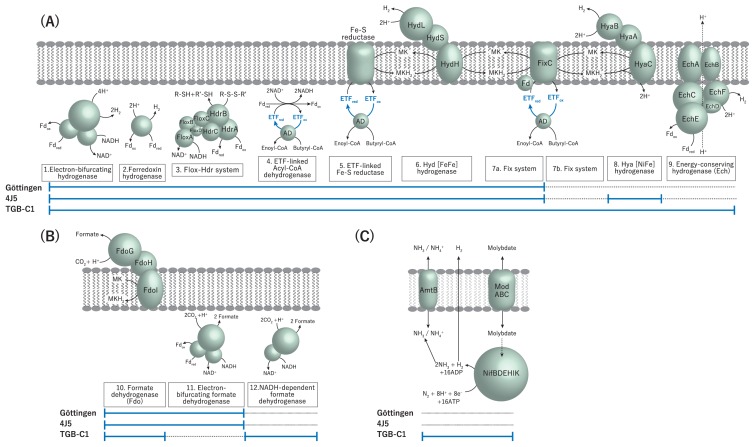
Hydrogenases, formate dehydrogenases, and energy conservation systems of *Syntrophomonas wolfei* subsp. *wolfei* strain Göttingen, “*Syntrophomonas wolfei* subsp. *methylbutyratica*” strain 4J5, and *Syntrophothermus lipocalidus* strain TGB-C1. (A) Hydrogenases and reverse electron transport systems including (1) electron-bifurcating hydrogenase, (2) ferredoxin hydrogenase, (3) flavin oxidoreductase-heterodisulfide reductase (flox-Hdr), (4) electron-transfer-flavoprotein (ETF)-linked acyl-CoA dehydrogenase, (5) ETF-linked iron-sulfur binding (Fe-S) reductase, (6) [FeFe] hydrogenase (Hyd), (7) ETF-oxidizing hydrogenase complex (FixABCX), (8) [NiFe] hydrogenase (Hya), and (9) energy-conserving hydrogenase (Ech). (B) Formate dehydrogenases including (10) formate dehydrogenase (Fdo), (11) electron-bifurcating formate dehydrogenase, and (12) NADH-dependent formate dehydrogenase. (C) A possible nitrogenase-mediated hydrogen generation system. The solid line indicates the existence of corresponding genes in the strains, while the dotted line indicates the absence of these genes.

A syntrophic substrate metabolizer uses energy conservation systems, such as reverse election transfer and electron bifurcation, to conduct thermodynamically unfavorable proton (H^+^) reduction to hydrogen (H_2_) (26, 31). Since the 4J5, Göttingen, and TGB-C1 genomes lack the *Rhodobacter* nitrogen fixation (Rnf) complex, these *Syntrophomonadaceae* syntrophs may not perform reverse electron transport from NADH to oxidized ferredoxin (Fd_ox_) through the Rnf found in several syntrophs (*e.g.*, *Syntrophus* and *Syntrophobacter*) (17, 39). Instead, we identified other types of reverse electron transport systems employing electron transfer flavoprotein (ETF) dehydrogenases described in the Göttingen genome: ETF-linked iron-sulfur binding (Fe-S) reductase and ETF-oxidizing hydrogenase complex (FixABCX) systems (6, 10, 22, 30) (Fig. 2A, Table S6). These results support a previous finding on the expression of Fe-S reductase with strain Göttingen during syntrophic butyrate degradation (18, 27). Although these *Syntrophomonadaceae* genomes also encode an electron-bifurcating ETF-associated butyryl-CoA dehydrogenase (Bcd/EtfAB), this system may support the capacities of these organisms to perform crotonate reduction (10, 18). In addition, the *Syntrophomonadaceae* syntrophs encode a syntroph-associated flavin oxidoreductase-heterodisulfide reductase (Flox-Hdr) system (22, 24) expressed by the BCFA-degrading syntroph “*Ca.* Caldisyntrophus multiacidovorans” and aromatic compound-degrading syntrophs (*Pelotomaculum* spp. and *Syntrophorhabdus* spp.) along with FixABCX (21), suggesting the involvement of these energy conservation systems in the syntrophic degradation of various organic compounds including BCFAs.

There may be multiple hydrogenase and formate dehydrogenase genes in the 4J5 and TGB-C1 genomes (Fig. 2, Table S6). Both strains harbor systems for hydrogen generation through electron-bifurcating Fe-only hydrogenase (NADH/Fd-oxidizing), Hyd hydrogenase (quinol), and cytoplasmic hydrogenase (Fd) with relatively high similarities (>68%) to those of strain Göttingen (30). In addition, the genomes of strains 4J5 and TGB-C1 encode the Hya hydrogenase gene cluster (Swmb_01823–01933 and Slip_0383–0392), which are lacking in the Göttingen genome. Strain TGB-C1 also has an energy-conserving hydrogenase (Ech; Fd-oxidizing) gene cluster (Slip_0655–0660). Regarding formate generation, we identified putative electron-bifurcating formate dehydrogenases (37) in strain 4J5, cytoplasmic formate dehydrogenase (NADH-oxidizing) in strain TGB-C1, and Fdo formate dehydrogenase (quinol-oxidizing) in both. These results suggest that BCFA-degrading syntrophs flexibly employ multiple energy conservation systems to maintain a syntrophic lifestyle in thermodynamically limited environments.

**Fig. 3:**
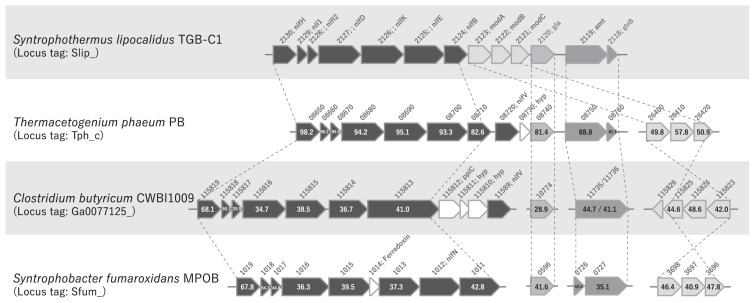
Comparison of nitrogenase-like gene cassettes found in *Syntrophothermus lipocalidus* strain TGB-C1, *Thermacetogenium phaeum* strain PB, *Clostridium butyricum* strain CWBI1009, and *Syntrophobacter fumaroxidans* strain MPOB. Strain TGB-C1 nitrogenase-associated cassette encodes nitrogen fixation proteins (NifBDEHIK), glutamine amidotransferase class-I (gla), a molybdate transporter (ModABC), and ammonia transporter (amt) with a nitrogen regulatory protein P-II family (glnB). Other strains harbor NifV (strains PB and CWBI1009), peptidyl-prolyl cis-trans isomerase C (ppiC) (strain PB), ferredoxin (strain MPOB), and hypothetical protein (hyp) (strains PB and CWBI1009) within the cassette. Abbreviated locus tags are shown (*e.g.*, ‘Slip_2130’ as ‘2130’ in the row of strain TGB-C1). Figures in gene boxes indicate amino acid sequence identity to the corresponding gene of strain TGB-C1.

A nitrogenase gene cluster (NifBDEHIK) was detected in the genome of strain TGB-C1 (Slip_2124–2130) along with a molybdate transporter (ModABC, Slip_2121–2123) and ammonia transporter (AmtB, Slip_2119) (Fig. 2C, Table S7). A previous microbial genome survey revealed that nitrogen fixation-related proteins (Nif) are distributed in phylogenetically diverse microbes including some fermentative bacteria and syntrophic substrate metabolizers (5). The nitrogenase activity of the fermentative H_2_-producing organism *Clostridium butyricum* strain CWBI1009 has been proposed to generate H_2_ and enhance tolerance to acidification through the consumption of protons in the reaction, which produces ammonia as a base (3). We observed high amino acid sequence similarities (up to 98%) between the nitrogenase, molybdate transporter, and ammonia transporter genes of TGB-C1 and CWBI1009, and also moderate similarities (up to 68%) with two other Nif-encoding syntrophs (*Thermacetogenium phaeum* strain PB and *Syntrophobacter fumaroxidans* strain MPOB) (Fig. 3, Table S7). Among syntrophs, nitrogen fixation may serve as a mechanism to tolerate acidification and provide hydrogen and ammonia for partner hydrogenotrophic methanogens to survive under hydrogen/ammonia-limited conditions.

In summary, a comparative genomic analysis of the *Syntrophomonadaceae* strains revealed multiple genes for the β-oxidation of BCFA/SCFA and energy conservation systems employing various types of electron carriers for electron disposal. In theory, the syntrophic degradation of 2-methylbutyrate and isobutyrate have Gibbs free energy values identical to butyrate under standard conditions (calculations based on Thauer *et al.* [36]):

$$\begin{array}{l} {\text{Butyrate}}^{-}+2{\text{H}}_{2}\text{O}\to 2\;{\text{Acetate}}^{-}+2{\text{H}}_{2}+{\text{H}}^{+}\\ 2-{\text{methylbutyrate}}^{-}+2{\text{H}}_{2}\text{O}\to {\text{Acetate}}^{-}+{\text{Propionate}}^{-}+2{\text{H}}_{2}+{\text{H}}^{+}\\ {\text{Isobutyrate}}^{-}+2{\text{H}}_{2}\text{O}\to 2\;{\text{Acetate}}^{-}+2{\text{H}}_{2}+{\text{H}}^{+}\\ \Delta {\text{G}}_{0}’=+48.3\;\text{kJ }{\text{mol}}^{-1} \end{array}$$

Thus, while *Syntrophomonadaceae* strains degrading the above fatty acids have unique substrate-specific genes (*e.g.*, β-oxidation), they share similar energy conservation systems reflecting the thermodynamic similarity shown above. TGB-C1 has several distinct energy conservation pathways from Göttingen and 4J5 potentially due to differences in their temperature preferences. These genome-based results further provide insights into how syntrophic BCFA-degraders interact with H_2_/formate-utilizing methanogen in an anaerobic ecosystem.

## Supplementary Information
