# Supplementary material for: Comparative Genomics of Syntrophic Branched-Chain Fatty Acid Degrading Bacteria
Source: Microbes Environ. 2016 Jul 16;31(3):288–92. doi: 10.1264/jsme2.ME16057 (PMC5017805; doi:10.1264/jsme2.ME16057)
Supplement: Supplementary file 1 [file 31_288_s1.pdf]

**Supplemental information**

# Comparative Genomics of Syntrophic Branched-Chain Fatty Acid Degrading Bacteria

Takashi Narihiro[1,2†*], Masaru K. Nobu[2†], Hideyuki Tamaki[1], Yoichi Kamagata[1], Yuji Sekiguchi[3], and Wen-Tso Liu[2]

[1]*Bioproduction Research Institute, National Institute of Advanced Industrial Science and Technology (AIST), Central 6, Higashi 1-1-1, Tsukuba, Ibaraki 305-8566, Japan*; [2]*Department of Civil and Environmental Engineering, University of Illinois at Urbana-Champaign, 205 North Mathews Ave, Urbana, IL 61801, USA;* [3]*Biomedical Research Institute, National Institute of Advanced Industrial Science and Technology (AIST), Central 6, Higashi 1-1-1, Tsukuba, Ibaraki 305-8566, Japan*

* Corresponding author: E-mail t.narihiro@aist.go.jp, Tel: +81-29-861-6591; Fax: +81-29-861-6587.

† These authors contributed equally to this work.

## Materials and Methods

Clustered regularly interspaced short palindromic repeats (CRISPRs) were identified using the CRISPRs web server (6). Alignment of the translated amino acid sequences and construction of maximum likelihood (ML) trees of acyl-CoA dehydrogenase, enoyl-CoA hydratase, 3-hydroxyacyl-CoA dehydrogenase, acetyl-CoA acetyltransferase, and isobutyrly-CoA mutase were performed with the MEGA version 6.06 (5, 15). For phylogenetic comparison of genes involved in β–oxidation pathway, we tentatively annotated acyl-CoA dehydrogenase, enoyl-CoA hydratase, 3-hydroxyacyl-CoA dehydrogenase, and acetyl-CoA acetyltransferase genes from draft genome sequences of *Syntrophomonas palmitatica* strain

JCM 14374$^{T}$ (accession no. NZ_BBCE00000000) and *Syntrophomonas zehnderi* strain OL-4$^{T}$ (NZ_CGIH00000000) by using Prokka version 1.11 (10). The topology of the trees was estimated by 1,000 bootstrap replicates (4). The Jones-Taylor-Thornton (JTT) model and nearest-neighbor-interchange (NNI) were used for substitution model and heuristic method, respectively (7, 14).

**Results and Discussion**

*Genome statistics*

The quality of the genome of "*Syntrophomonas wolfei* subsp. *methylbutyratica*" strain 4J5, *Syntrophomonas wolfei* subsp. *wolfei* strain Göttingen, and *Syntrophothermus lipocalidus* strain TGB-C1 seems to be high (>97%) enough for the comparative genomics (Table S1). Approximately 1,800 genes of the strain 4J5 were highly homologous (>90% amino acid identity) to Göttingen genes, whereas approximately 1,000 genes of the strains 4J5 and Göttingen have significant amino acid identity (>70%) with the genes of strain TGB-C1 (Fig. S3).

*Central metabolism, physiology, and transport systems*

In accordance with the genome of strain Göttingen (11), the 4J5 and TGB-C1 genomes encode the Embden–Meyerhof pathway, pentose phosphate pathway, partial tricarboxylic acid (TCA) cycle, citrate (Re)-synthase gene cluster, and co-factor biosynthesis for central metabolism (Table S2). Bacterial core RNA polymerase (*rpoA*, *rpoB*, *rpoC*, and *rpoZ*) with sigma factors including general primary 70 factors (*rpoD*), heat stress factors (*rpoE*), a flagella biogenesis factor (*fliA, rpoF*), and a nitrogen-limitation 54 factor (*rpoN*) are identified in both of the strains. The RNA genes (rRNA and tRNA) were also found (Table S7). To cope with disturbances of environmental conditions, the strain 4J5 genome encodes

thioredoxin, rubrerythrin, superoxide dismutase, and molecular chaperone systems. The strain TGB-C1 genome harbors these systems, except for superoxide dismutase.

The genes encoding flagellum components, type IV pilus synthesis, chemotaxis, and sporulation are found in the genomes. In addition, all of the genes required for synthesis and metabolize poly-β-hydroxyalkanoate (PHA) are found in the 4J5 genome, but not in the TGB-C1 genome. The PHA granules have been observed in the cells of the strain 4J5 under electron microscopic observation (16), implying that the strain 4J5 may utilize PHA under thermodynamically unfavorable conditions for the degradation of VFAs and BCFAs as suggested by previous culture- and genome-based studies on the strain Göttingen (1, 2, 8, 11). We found the genes associated with clustered regularly interspaced short palindromic repeat (CRISPR) within the genomes (Table S7). Although CRISPR is known as acquired immune system against phages (12, 13), the ecological role of the CRISPRs observed in the syntroph genomes remains obscure due to the absence of comprehensive study on CRISPR dynamics in methanogenic ecosystem.

A total of 105 and 68 genes/gene clusters involved in transportation, secretion, and drug resistance are found in the strain 4J5 and TGB-C1 genomes, respectively (Table S3). For example, there are tungstate, molybdate, ferrous iron, zinc, cobalt, phosphate, sodium, potassium, amino acids, and monosaccharides transporters. The strains Göttingen, 4J5, and TGB-C1 shared almost all of the transport/secretion systems with some exceptions; for example, ammonia transporters (Slip_1571 and Slip_2119) were found only in the strain TGB-C1, suggesting that *Syntrophothermus lipocalidus* possesses nitrogen-associated physiological function (discussed in main text).

Table S1. Genome statistics

| Attribute | *Syntrophomonas wolfei* subsp. *metylbutyratica* strain 4J5 | *Syntrophomonas wolfei* subsp. *wolfei* strain Göttingen | *Syntrophothermus lipocalidus* strain TGB-C1 |
|---|---|---|---|
| Reference | This study | (11) | (3) |
| Status | Draft | Complete | Complete |
| DNA scaffold | 89 | 1 | 1 |
| DNA, total (bp) | 3,194,001 | 2,936,195 | 2,405,559 |
| DNA, G+C content | 45.55% | 44.87% | 50.99% |
| Genes total number | 3,073 | 2,677 | 2,440 |
| Protein coding genes | 2,964 (96.5%) | 2,574 (96.2%) | 2,385 (97.8%) |
| RNA genes | 54 | 65 | 55 |
| rRNA genes | 11 | 19 | 6 |
| tRNA genes | 43 | 46 | 46 |
| Genes with function prediction | 2,184 (73.7%) | 1,507 (57.1%) | 1,726 (70.74%) |
| CRISPR | | | |
| Site number | 2 | 5 | 2 |
| Spacer number | 63, 126 | 3, 15, 36, 61, 185 | 119, 152 |
| Genome quality | | | |
| Completeness | 97.35% | 97.35% | 98.81% |
| Contamination | 0.04% | 0.21% | 0.51% |

Table S2. *Syntrophomonas wolfei* subsp. *metylbutyratica* strain 4J5$^T$ genes relevant to general function.

| Locus Tag | Gene Product Name | BLAST top hit in *Syntrophomonas wolfei* strain Goettingen | | | BLAST top hit in *Syntrophothermus lipocalidus* strain TGB-C1 | | |
|---|---|---|---|---|---|---|---|
| | | Gene ID and annotation | Identity (%) | e-Value | Gene ID and annotation | Identity (%) | e-Value |
| Glycolysis / TCA cycle | | | | | | | |
| SWMB_00127 | glk; glucokinase | 638137153 Swol_0259 glucokinase (EC 2712) | 93.2 | 0 | 646854887 Slip_1859 glucokinase (EC 2.7.1.2) | 43.6 | 2E-61 |
| SWMB_00126 | pgi-pmi; glucose/mannose-6-phosphate isomerase | 638137154 Swol_0260 bifunctional phosphoglucose/phosphomannose isomerase (EC 5318; EC 531 | 95.6 | 0 | 646854886 Slip_1858 bifunctional phosphoglucose/phosphomannose isomerase (EC 5.3.1.8; EC 5.3.1 | 59.1 | 8E-146 |
| SWMB_00515 | glpX; fructose-1,6-bisphosphatase II | 638139293 Swol_2409 hypothetical protein | 98.2 | 0 | 646855333 Slip_2308 fructose-1,6-bisphosphatase, class II | 76.9 | 0 |
| SWMB_02090 | pfkA, PFK; 6-phosphofructokinase 1 | 638138923 pfkA 6-phosphofructokinase (EC 27111) | 97.8 | 0 | 646854733 pfkA 6-phosphofructokinase (EC 2.7.1.11) | 60.8 | 6E-133 |
| SWMB_02719 | pfkA, PFK; 6-phosphofructokinase 1 | 638138043 pfkA 6-phosphofructokinase (EC 27111) | 98.1 | 0 | 646854145 pfkA 6-phosphofructokinase (EC 2.7.1.11) | 67.9 | 2E-166 |
| SWMB_00513 | FBA, fbaA; fructose-bisphosphate aldolase, class II | 638139295 Swol_2411 fructose-bisphosphate aldolase (EC 41213) | 97.9 | 0 | 646855335 Slip_2310 fructose-bisphosphate aldolase (EC 4.1.2.13) | 78.8 | 6E-166 |
| SWMB_00112 | TPI, tpiA; triosephosphate isomerase (TIM) | 638137167 tpiA triosephosphate isomerase (EC 5311) | 92.8 | 0 | 646854878 tpiA triosephosphate isomerase (EC 5.3.1.1) | 54.4 | 8E-93 |
| SWMB_00114 | GAPDH, gapA; glyceraldehyde 3-phosphate dehydrogenase | 638137165 Swol_0272 glyceraldehyde-3-phosphate dehydrogenase (NAD+) (EC 12112) | 98.5 | 0 | 646854880 Slip_1852 glyceraldehyde-3-phosphate dehydrogenase (NAD+) (EC 1.2.1.12) | 58.5 | 3E-147 |
| SWMB_00113 | PGK, pgk; phosphoglycerate kinase | 638137166 pgk phosphoglycerate kinase (EC 2723) | 96.1 | 0 | 646854879 pgk phosphoglycerate kinase (EC 2.7.2.3) | 59.5 | 4E-175 |
| SWMB_00111 | gpmI; 2,3-bisphosphoglycerate-independent phosphoglycerate mutase | 638137168 gpmI phosphoglycerate mutase (EC 5421) | 95.5 | 0 | 646854877 gpmI phosphoglycerate mutase (EC 5.4.2.1) | 68.4 | 0 |
| SWMB_00845 | apgM; 2,3-bisphosphoglycerate-independent phosphoglycerate mutase | 638138180 Swol_1301 phosphoglycerate mutase (EC 5421) | 94.8 | 0 | 646853321 Slip_0290 phosphoglycerate mutase (EC 5.4.2.1) | 56.1 | 3E-166 |
| SWMB_00110 | ENO, eno; enolase; phosphopyruvate hydratase | 638137169 eno enolase (EC 42111) | 97.7 | 0 | 646854876 eno enolase | 73.5 | 0 |
| SWMB_02089 | PK, pyk; pyruvate kinase | 638138922 Swol_2040 pyruvate kinase (EC 27140) | 97.9 | 0 | 646854732 Slip_1701 pyruvate kinase (EC 2.7.1.40) | 61.7 | 0 |
| SWMB_02328 | Pyruvate, phosphate dikinase | 638138392 Swol_1512 pyruvate phosphate dikinase (EC 2791) | 96.5 | 0 | 646854654 Slip_1625 pyruvate phosphate dikinase (EC 2.7.9.1) | 75.2 | 0 |
| SWMB_02463 | pyruvate carboxylase | 638137403 Swol_0519 pyruvate carboxylase, PYKA | 98.6 | 0 | 646853453 Slip_0423 acetyl-CoA carboxylase, biotin carboxylase | 48.6 | 6E-129 |
| SWMB_02193 | hydro-lyases, Fe-S type, tartrate/fumarate subfamily, beta region | 638138505 Swol_1628 Fumarate hydratase beta subunit | 98.1 | 1E-150 | 646855078 Slip_2051 hydro-lyase, Fe-S type, tartrate/fumarate subfamily, beta subunit | 35.0 | 3E-29 |
| SWMB_02194 | hydro-lyases, Fe-S type, tartrate/fumarate subfamily, alpha region | 638138506 Swol_1629 Fumarate hydratase alpha subunit | 95.1 | 0 | 646855079 Slip_2052 hydro-lyase, Fe-S type, tartrate/fumarate subfamily, alpha subunit | 30.6 | 2E-36 |
| SWMB_01460 | DLD, lpd, pdhD; dihydrolipoamide dehydrogenase | 638138860 Swol_1978 dihydrolipoamide dehydrogenase (EC 1814) | 94.4 | 0 | 646855207 Slip_2184 dihydrolipoamide dehydrogenase | 39.4 | 3E-102 |
| SWMB_00330 | korD, oorD; 2-oxoglutarate ferredoxin oxidoreductase subunit delta | 638138281 Swol_1401 4Fe-4S ferredoxin, iron-sulfur binding | 37.1 | 5E-11 | 646853091 Slip_0043 4Fe-4S ferredoxin iron-sulfur binding domain protein | 69.0 | 7E-40 |
| SWMB_00331 | korC, oorC; 2-oxoglutarate ferredoxin oxidoreductase subunit gamma | 638136935 Swol_0038 2-oxoglutarate ferredoxin oxidoreductase, gamma subunit (EC 1273) | 95.9 | 6E-135 | 646853090 Slip_0042 Pyruvate/ketoisovalerate oxidoreductase | 65.1 | 6E-87 |
| SWMB_00332 | korB, oorB; 2-oxoglutarate ferredoxin oxidoreductase subunit beta | 638136934 Swol_0037 2-oxoglutarate ferredoxin oxidoreductase, beta subunit (EC 1273) | 100.0 | 0 | 646853089 Slip_0041 thiamine pyrophosphate protein domain protein TPP-binding protein | 79.3 | 7E-159 |
| SWMB_00333 | korA, oorA; 2-oxoglutarate ferredoxin oxidoreductase subunit alpha | 638136933 Swol_0036 putative keto/oxoacid ferredoxin oxidoreductase, alpha subunit | 98.3 | 0 | 646853088 Slip_0040 pyruvate flavodoxin/ferredoxin oxidoreductase domain protein | 77.4 | 0 |
| SWMB_00728 | korD, oorD; 2-oxoglutarate ferredoxin oxidoreductase subunit delta | 638138281 Swol_1401 4Fe-4S ferredoxin, iron-sulfur binding | 93.0 | 3E-45 | 646854802 Slip_1773 4Fe-4S ferredoxin iron-sulfur binding domain protein | 59.2 | 1E-26 |
| SWMB_00729 | korA, oorA; 2-oxoglutarate ferredoxin oxidoreductase subunit alpha | 638138280 Swol_1400 2-oxoisovalerate ferredoxin oxidoreductase, alpha subunit | 97.7 | 0 | 646854801 Slip_1772 pyruvate flavodoxin/ferredoxin oxidoreductase domain protein | 67.6 | 0 |
| SWMB_00730 | korB, oorB; 2-oxoglutarate ferredoxin oxidoreductase subunit beta | 638138279 Swol_1399 putative keto/oxoacid ferredoxin oxidoreductase, beta subunit | 98.4 | 0 | 646854800 Slip_1771 thiamine pyrophosphate protein domain protein TPP-binding protein | 75.1 | 3E-145 |
| SWMB_00731 | korC, oorC; 2-oxoglutarate ferredoxin oxidoreductase subunit gamma | 638138278 Swol_1398 2-oxoglutarate oxidoreductase gamma subunit | 93.4 | 1E-127 | 646854799 Slip_1770 2-oxoglutarate ferredoxin oxidoreductase, gamma subunit (EC 1.2.7.3) | 62.2 | 2E-82 |
| | | | | | 646853257 Slip_0223 fumarate reductase/succinate dehydrogenase flavoprotein domain protein | | |
| re-citrate synthase gene cluster | | | | | | | |
| SWMB_01738 | Isocitrate dehydrogenase [NADP] | 638137267 Swol_0378 isocitrate dehydrogenase (NADP) (EC 11142) | 97.9 | 0 | 646853542 Slip_0511 isocitrate dehydrogenase (NADP) (EC 1.1.1.42) | 81.3 | 0 |
| SWMB_01739 | 2,3-dimethylmalate dehydratase small subunit | 638137266 Swol_0377 leuD 3-isopropylmalate dehydratase, small subunit (EC 42133) | 98.8 | 6E-121 | 646853541 Slip_0510 leuD 3-isopropylmalate dehydratase, small subunit | 80.1 | 2E-99 |
| SWMB_01740 | 2,3-dimethylmalate dehydratase large subunit | 638137265 Swol_0376 leuC 3-isopropylmalate dehydratase, large subunit (EC 42133) | 97.8 | 0 | 646853540 Slip_0509 leuC homoaconitate hydratase family protein | 78.7 | 0 |
| SWMB_01741 | putative re-citrate synthase | 638137264 Swol_0375 hypothetical protein | 97.4 | 0 | 646853539 Slip_0508 homocitrate synthase | 76.3 | 0 |
| Pentose phosphate pathway | | | | | | | |
| SWMB_00140 | tktA, tktB; transketolase | 638137140 Swol_0244 transketolase subunit B (EC 2211) | 96.5 | 0 | 646854901 Slip_1873 transketolase subunit B (EC 2.2.1.1) | 67.1 | 3E-157 |
| SWMB_00141 | tktA, tktB; transketolase | 638137139 Swol_0243 transketolase subunit A (EC 2211) | 95.6 | 0 | 646854902 Slip_1874 transketolase subunit A (EC 2.2.1.1) | 70.6 | 6E-138 |
| SWMB_00514 | talA, talB; transaldolase | 638139294 tal transaldolase (EC 2212) | 98.1 | 2E-155 | 646855334 tal transaldolase (EC 2.2.1.2) | 72.5 | 1E-116 |
| SWMB_02744 | rpe, RPE; ribulose-phosphate 3-epimerase | 638138098 Swol_1223 ribulose-5-phosphate 3-epimerase (EC 5131) | 94.0 | 4E-151 | 646853901 Slip_0868 ribulose-5-phosphate 3-epimerase (EC 5.1.3.1) | 58.7 | 2E-93 |
| SWMB_00294 | PRPS, prsA; ribose-phosphate pyrophosphokinase | 638136971 prs Ribose-phosphate diphosphokinase | 97.8 | 0 | 646853149 prs ribose-phosphate pyrophosphokinase | 76.6 | 5E-170 |
| SWMB_01013 | pmm-pgm; phosphomannomutase / phosphoglucomutase | 638139034 Swol_2154 phosphomannomutase (EC 5428) | 94.0 | 0 | 646853797 Slip_0765 phosphomannomutase (EC 5.4.2.8) | 69.4 | 0 |
| SWMB_02559 | deoB; phosphopentomutase | 638137487 deoB Phosphopentomutase | 93.3 | 0 | 646853524 deoB phosphopentomutase | 60.8 | 9E-171 |
| SWMB_01013 | pmm-pgm; phosphomannomutase / phosphoglucomutase | 638139034 Swol_2154 phosphomannomutase (EC 5428) | 94.0 | 0 | 646853797 Slip_0765 phosphomannomutase (EC 5.4.2.8) | 69.4 | 0 |
| SWMB_02972 | pmm-pgm; phosphomannomutase / phosphoglucomutase | 638138797 Swol_1915 phosphomannomutase (EC 5428) | 96.0 | 0 | 646853797 Slip_0765 phosphomannomutase (EC 5.4.2.8) | 68.3 | 0 |
| SWMB_00294 | PRPS, prsA; ribose-phosphate pyrophosphokinase | 638136971 prs Ribose-phosphate diphosphokinase | 97.8 | 0 | 646853149 prs ribose-phosphate pyrophosphokinase | 76.6 | 5E-170 |
| SWMB_02011 | deoC, DERA; deoxyribose-phosphate aldolase | 638138440 deoC Deoxyribose-phosphate aldolase | 93.2 | 2E-150 | 646854663 deoC deoxyribose-phosphate aldolase | 48.6 | 9E-62 |
| | | | | | 646855321 Slip_2294 sugar-phosphate isomerase, RpiB/LacA/LacB family | | |
| | | | | | 646853351 Slip_0321 PfkB domain protein | | |
| Anaplerotic reactions | | | | | | | |
| SWMB_02089 | pyk; pyruvate kinase | 638138922 Swol_2040 pyruvate kinase (EC 27140) | 97.9 | 0 | 646854732 Slip_1701 pyruvate kinase (EC 2.7.1.40) | 61.7 | 0 |
| SWMB_02328 | ppdK; pyruvate, orthophosphate dikinase | 638138392 Swol_1512 pyruvate phosphate dikinase (EC 2791) | 96.5 | 0 | 646854654 Slip_1625 pyruvate phosphate dikinase (EC 2.7.9.1) | 75.2 | 0 |
| SWMB_00006 | porB; pyruvate ferredoxin oxidoreductase beta subunit | 638138254 Swol_1374 pyruvate synthase subunit porB | 96.5 | 0 | | | |
| SWMB_00007 | porA; pyruvate ferredoxin oxidoreductase alpha subunit | 638138255 Swol_1375 pyruvate ferredoxin oxidoreductase, alpha subunit (EC 1271) | 96.5 | 0 | | | |
| SWMB_00008 | porD; pyruvate ferredoxin oxidoreductase delta subunit | 638138256 Swol_1376 pyruvate ferredoxin oxidoreductase, delta subunit (EC 1271) | 94.1 | 2E-70 | | | |
| SWMB_00009 | porG; pyruvate ferredoxin oxidoreductase gamma subunit | 638138257 Swol_1377 pyruvate ferredoxin oxidoreductase, gamma subunit (EC 1271) | 93.8 | 1E-137 | | | |
| SWMB_00921 | porG; pyruvate ferredoxin oxidoreductase gamma subunit | 638139117 Swol_2237 pyruvate ferredoxin oxidoreductase, gamma subunit (EC 1271) | 95.7 | 2E-121 | | | |
| SWMB_00922 | porD; pyruvate ferredoxin oxidoreductase delta subunit | 638139116 Swol_2236 pyruvate synthase delta chain | 96.5 | 7E-58 | | | |
| SWMB_00923 | porA; pyruvate ferredoxin oxidoreductase alpha subunit | 638139115 Swol_2235 2-oxoisovalerateferredoxin oxidoreductase, alpha subunit | 99.2 | 0 | | | |
| SWMB_00924 | porB; pyruvate ferredoxin oxidoreductase beta subunit | 638139114 Swol_2234 2-ketoacid ferredoxin oxidoreductase beta subunit | 99.0 | 0 | | | |
| SWMB_02955 | aldehyde dehydrogenase (NAD+) | 639331138 Swol_0319 Aldehyde dehydrogenase | 88.5 | 0 | | | |
| SWMB_01418 | Phosphoenolpyruvate synthase (2.7.9.2) | | | | | | |
| SWMB_01420 | Phosphoenolpyruvate synthase (2.7.9.2) | | | | | | |
| SWMB_02538 | Phosphoenolpyruvate synthase (2.7.9.2) | | | | | | |
| SWMB_02598 | Phosphoenolpyruvate synthase (2.7.9.2) | | | | | | |
| RNA polymerase | | | | | | | |
| SWMB_00505 | DNA-directed RNA polymerase subunit delta | 638139304 Swol_2420 hypothetical protein | 90.9 | 6E-50 | 646855343 Slip_2318 hypothetical protein | 43.6 | 1E-17 |
| SWMB_00589 | DNA-directed RNA polymerase subunit beta | 638139222 rpoB DNA-directed RNA polymerase subunit beta (EC 2776) | 98.3 | 0 | 646855267 rpoB DNA-directed RNA polymerase subunit beta (EC 2.7.7.6) | 81.4 | 0 |
| SWMB_00590 | DNA-directed RNA polymerase subunit beta' | 638139221 rpoC DNA-directed RNA polymerase subunit beta' (EC 2776) | 98.9 | 0 | 646855266 rpoC DNA-directed RNA polymerase subunit beta' (EC 2.7.7.6) | 79.5 | 0 |
| SWMB_00625 | DNA-directed RNA polymerase subunit alpha | 638139185 rpoA DNA-directed RNA polymerase subunit alpha (EC 2776) | 99.7 | 0 | 646855230 rpoA DNA-directed RNA polymerase subunit alpha (EC 2.7.7.6) | 75.6 | 5E-177 |
| SWMB_02757 | DNA-directed RNA polymerase subunit omega | 638138111 rpoZ DNA-directed RNA polymerase subunit omega (EC 2776) | 95.8 | 6E-35 | 646853888 rpoZ DNA-directed RNA polymerase subunit omega (EC 2.7.7.6) | 64.4 | 9E-22 |
| Sigma factor / anti-sigma factor | | | | | | | |
| SWMB_00121 | rpoN; RNA polymerase sigma-54 factor | 638137158 Swol_0265 RNA polymerase, sigma 54 subunit, RpoN/SigL | 96.8 | 0 | 646854882 Slip_1854 RNA polymerase, sigma 54 subunit, RpoN/SigL | 53.7 | 0 |
| SWMB_00232 | rpoE; RNA polymerase sigma-70 factor, ECF subfamily | 638137032 Swol_0132 RNA polymerase, sigma-24 subunit, RpoE | 89.2 | 1E-129 | 646854990 Slip_1965 RNA polymerase, sigma-24 subunit, RpoE | 58.2 | 1E-71 |
| SWMB_00233 | mycothiol system anti-sigma-R factor | 638137031 Swol_0131 putative transmembrane anti-sigma factor | 91.3 | 0 | 646854992 Slip_1967 putative transmembrane anti-sigma factor | 33.2 | 1E-41 |
| SWMB_00465 | Sigma-W factor | 638137032 Swol_0132 RNA polymerase, sigma-24 subunit, RpoE | 34.8 | 7E-25 | 646854430 Slip_1410 RNA polymerase, sigma-24 subunit, ECF subfamily | 35.0 | 3E-22 |
| SWMB_00576 | RNA polymerase sporulation-specific sigma factor | 638139237 Swol_2351 RNA polymerase, sigma 30 subunit, SigH | 100.0 | 1E-159 | 646855281 Slip_2254 RNA polymerase, sigma 30 subunit, SigH | 84.4 | 8E-130 |
| SWMB_00798 | rpoE; RNA polymerase sigma-70 factor, ECF subfamily | 638137032 Swol_0132 RNA polymerase, sigma-24 subunit, RpoE | 27.3 | 1E-08 | 646854430 Slip_1410 RNA polymerase, sigma-24 subunit, ECF subfamily | 21.8 | 6E-07 |
| SWMB_01098 | Putative anti-sigma factor antagonist BtrV | 638138944 Swol_2062 anti-sigma F factor antagonist (stage II sporulation protein AA) | 96.0 | 1E-64 | 646853556 Slip_0525 anti-anti-sigma regulatory factor, SpoIIAA | 31.5 | 9E-09 |
| SWMB_01104 | rpoE; RNA polymerase sigma-70 factor, ECF subfamily | 638138940 Swol_2058 RNA polymerase, sigma-24 subunit, RpoE | 97.1 | 1E-121 | 646854430 Slip_1410 RNA polymerase, sigma-24 subunit, ECF subfamily | 26.5 | 1E-08 |
| SWMB_01123 | sigma-70 RNA polymerase sigma factor | 638137818 Swol_0944 RNA polymerase sigma factor | 91.8 | 1E-162 | 646853625 Slip_0594 putative RNA polymerase, sigma 28 subunit, FliA/WhiG subfamily | 32.4 | 1E-19 |
| SWMB_01321 | RNA polymerase sigma factor for flagellar operon FliA | 638137746 Swol_0878 RNA polymerase, sigma 28 subunit, SigD/FliA/WhiG | 97.3 | 0 | 646854014 Slip_0982 RNA polymerase, sigma 28 subunit, SigD/FliA/WhiG | 63.1 | 6E-107 |
| SWMB_01365 | RNA polymerase sporulation-specific sigma factor | 638137706 Swol_0836 RNA polymerase, sigma subunit, RpsG/SigG | 99.6 | 3E-167 | 646853841 Slip_0808 RNA polymerase, sigma subunit, RpsG/SigG | 80.2 | 6E-134 |
| SWMB_01367 | RNA polymerase sigma-35 factor precursor | 638137705 Swol_0835 RNA polymerase, sigma 29 subunit, SigE | 98.8 | 7E-173 | 646853840 Slip_0807 RNA polymerase, sigma 29 subunit, SigE | 79.3 | 3E-124 |
| SWMB_01423 | rpoE; RNA polymerase sigma-70 factor, ECF subfamily | 638138901 Swol_2019 RNA polymerase, sigma-24 subunit, RpoE | 99.5 | 9E-142 | 646854990 Slip_1965 RNA polymerase, sigma-24 subunit, RpoE | 45.0 | 3E-47 |
| SWMB_01424 | mycothiol system anti-sigma-R factor | 638138900 Swol_2018 putative transmembrane anti-sigma factor | 91.2 | 0 | 646854431 Slip_1411 hypothetical protein | 27.2 | 0.0001 |
| SWMB_01571 | Putative anti-sigma factor antagonist BtrV | 638138678 Swol_1795 anti-anti-sigma regulatory factor, SpoIIAA | 90.0 | 3E-65 | 646853556 Slip_0525 anti-anti-sigma regulatory factor, SpoIIAA | 22.3 | 0.0008 |
| SWMB_01574 | rpoE; RNA polymerase sigma-70 factor, ECF subfamily | 638138674 Swol_1791 RNA polymerase, sigma-24 subunit, RpoE | 93.1 | 2E-127 | 646854430 Slip_1410 RNA polymerase, sigma-24 subunit, ECF subfamily | 27.2 | 1E-09 |
| SWMB_01607 | Anti-sigma-V factor RsiV | 638138645 Swol_1762 hypothetical protein | 92.5 | 3E-173 | 646853840 Slip_0807 RNA polymerase, sigma 29 subunit, SigE | 44.7 | 0.13 |
| SWMB_01650 | RNA polymerase sigma-28 factor precursor | 638137385 Swol_0501 sigma 28 (flagella/sporulation) | 95.2 | 1E-86 | 646853423 Slip_0393 RNA polymerase, sigma 27/28 subunit, RpsK/SigK | 67.8 | 1E-53 |
| SWMB_01951 | Anti-sigma-V factor RsiV | 639331177 Swol_1624 hypothetical protein | 94.9 | 7E-155 | 646855347 Slip_2322 S-layer domain protein | 25.9 | 0.00008 |
| SWMB_02033 | Putative anti-sigma factor antagonist BtrV | 638138679 Swol_1796 hypothetical protein | 68.3 | 0 | 646853556 Slip_0525 anti-anti-sigma regulatory factor, SpoIIAA | 26.2 | 3E-07 |
| SWMB_02334 | rpoD; RNA polymerase primary sigma factor | 638138387 sigA RNA polymerase, sigma 70 subunit, RpoD | 98.6 | 0 | 646854649 sigA RNA polymerase, sigma 70 subunit, RpoD | 84.7 | 0 |
| SWMB_02464 | RNA polymerase sigma-28 factor precursor | 638137401 Swol_0517 RNA polymerase, sigma 27/28 subunit, RpsK/SigK | 98.8 | 7E-177 | 646853423 Slip_0393 RNA polymerase, sigma 27/28 subunit, RpsK/SigK | 70.6 | 2E-120 |
| SWMB_02498 | rpoE; RNA polymerase sigma-70 factor, ECF subfamily | 638138674 Swol_1791 RNA polymerase, sigma-24 subunit, RpoE | 28.6 | 7E-14 | 646854990 Slip_1965 RNA polymerase, sigma-24 subunit, RpoE | 26.9 | 3E-10 |
| SWMB_02549 | RNA polymerase sporulation-specific sigma factor | 638137494 Swol_0615 RNA polymerase, sigma subunit, RpoX/SigF | 97.1 | 2E-172 | 646853558 Slip_0527 RNA polymerase, sigma subunit, RpoX/SigF | 60.5 | 4E-102 |
| SWMB_02550 | Anti-sigma F factor | 638137493 Swol_0614 putative anti-sigma regulatory factor, serine/threonine protein kinase | 95.3 | 1E-99 | 646853557 Slip_0526 putative anti-sigma regulatory factor, serine/threonine protein kinase | 56.1 | 2E-61 |
| SWMB_02732 | rpoE; RNA polymerase sigma-70 factor, ECF subfamily | 638138086 Swol_1212 RNA polymerase, sigma-24 subunit, RpoE | 95.8 | 1E-128 | 646854430 Slip_1410 RNA polymerase, sigma-24 subunit, ECF subfamily | 51.9 | 6E-67 |
| SWMB_02911 | rpoE; RNA polymerase sigma-70 factor, ECF subfamily | 638138901 Swol_2019 RNA polymerase, sigma-24 subunit, RpoE | 26.0 | 7E-14 | 646854430 Slip_1410 RNA polymerase, sigma-24 subunit, ECF subfamily | 27.9 | 2E-14 |
| SWMB_00061 | RNA polymerase sigma factor, sigma-70 family | | | | | | |
| SWMB_02520 | RNA polymerase sigma factor SigW | | | | | | |
| | | 638138387 Swol_1507 sigA RNA polymerase, sigma 70 subunit, RpoD | | | | | |
| | | | | | 646853160 Slip_0116 RNA polymerase, sigma-24 subunit, ECF subfamily | | |
| Histidine-kinase signal transduction | | | | | | | |
| SWMB_00042 | Signal transduction histidine-protein kinase BaeS | 638138557 Swol_1680 sensor histidine kinase | 23.4 | 1E-14 | 646853140 Slip_0096 multi-sensor signal transduction histidine kinase | 22.8 | 2E-17 |
| SWMB_00194 | Signal transduction histidine-protein kinase/phosphatase DegS | 638137082 Swol_0185 histidine kinase (EC 27133) | 96.2 | 0 | 648021255 Slip_1928 histidine kinase (EC 2.7.13.3) | 45.3 | 5E-107 |
| SWMB_00523 | Sensor histidine kinase CitA | 638139285 Swol_2401 signal transduction histidine kinase regulating citrate/malate metabolism (EC | 96.5 | 0 | 646854511 Slip_1489 signal transduction histidine kinase regulating citrate/malate metabolism | 28.4 | 1E-52 |
| SWMB_00765 | Sensor histidine kinase GraS | 638137826 Swol_0952 PAS/PAC sensor signal transduction histidine kinase (EC 27133) | 24.6 | 9E-14 | 646853398 Slip_0368 multi-sensor signal transduction histidine kinase | 23.1 | 2E-15 |
| SWMB_00858 | Sensor histidine kinase DpiB | 639331170 Swol_1291 hypothetical protein | 69.5 | 1E-178 | 646854511 Slip_1489 signal transduction histidine kinase regulating citrate/malate metabolism | 29.2 | 4E-64 |
| SWMB_01735 | Autoinducer 2 sensor kinase/phosphatase LuxQ | 638137269 Swol_0381 Hpt sensor hybrid histidine kinase (EC 27133) | 88.7 | 0 | 646853140 Slip_0096 multi-sensor signal transduction histidine kinase | 31.1 | 2E-31 |
| SWMB_01961 | Sensory histidine kinase DcuS | 638138489 Swol_1610 signal transduction histidine kinase regulating citrate/malate metabolism (EC | 91.3 | 1E-97 | 646853735 Slip_0708 signal transduction histidine kinase regulating citrate/malate metabolism | 31.4 | 7E-09 |
| SWMB_02255 | Signal transduction histidine-protein kinase BaeS | 638138557 Swol_1680 sensor histidine kinase | 91.9 | 0 | 646853140 Slip_0096 multi-sensor signal transduction histidine kinase | 28.0 | 9E-06 |
| SWMB_02564 | Signal transduction histidine-protein kinase BarA | 638137480 Swol_0601 multi-sensor hybrid histidine kinase (EC 27133) | 89.2 | 0 | 646853140 Slip_0096 multi-sensor signal transduction histidine kinase | 31.1 | 9E-30 |
| SWMB_02780 | putative signal transduction histidine kinase | 638137826 Swol_0952 PAS/PAC sensor signal transduction histidine kinase (EC 27133) | 26.0 | 4E-21 | 646853398 Slip_0368 multi-sensor signal transduction histidine kinase | 29.3 | 6E-26 |
| Stress response | | | | | | | |
| SWMB_00444 | Thioredoxin reductase | 638139355 Swol_2471 thioredoxin reductase (NADPH) (EC 1819) | 94.7 | 0 | 646855355 Slip_2331 thioredoxin reductase | 57.0 | 2E-121 |
| SWMB_01677 | Thioredoxin-M | 638137347 Swol_0462 thioredoxin | 97.3 | 5E-75 | 646853380 Slip_0350 thioredoxin | 54.6 | 2E-42 |
| SWMB_02155 | Rubrerythrin | 638137547 Swol_0670 rubrerythrin | 97.2 | 8E-130 | 646853273 Slip_0242 Rubrerythrin | 72.1 | 1E-86 |
| SWMB_01882 | Superoxide dismutase [Mn/Fe] | 638138588 Swol_1711 Superoxide dismutase | 99.0 | 7E-148 | | | |
| Chaperone / heat shock proteins | | | | | | | |
| SWMB_00278 | Heat shock protein 15 | 638136986 Swol_0084 hypothetical protein | 97.5 | 1E-50 | 646853157 Slip_0113 RNA-binding S4 domain protein | 63.8 | 3E-35 |
| SWMB_00671 | Heat shock protein 70 | 638138332 Swol_1452 molecular chaperone DnaK | 99.6 | 0 | 646854762 Slip_1731 Heat shock protein 70 | 63.9 | 0 |
| SWMB_01143 | General stress protein 16O | 638137838 Swol_0964 transcriptional regulator, TraR/DksA family | 94.6 | 3E-101 | 646853866 Slip_0833 transcriptional regulator, TraR/DksA family | 48.3 | 9E-42 |
| SWMB_01657 | Chaperone protein ClpB 1 | 638137378 clpB putative ATPase with chaperone activity, two ATP-binding domains | 99.2 | 0 | 646855295 Slip_2268 ATPase AAA-2 domain protein | 45.4 | 0 |
| SWMB_01659 | Heat shock protein J | 638137376 dnaJ dnaJ domain protein | 97.1 | 0 | 646854679 dnaJ chaperone protein DnaJ | 37.1 | 4E-58 |
| SWMB_01660 | Heat shock protein 70-2 | 638137375 dnaK chaperone protein DnaK | 98.9 | 0 | 646854680 dnaK chaperone protein DnaK | 58.9 | 0 |
| SWMB_01729 | Heat shock protein 70 | 638137274 Swol_0386 Molecular chaperone-like protein | 94.4 | 0 | 646853333 Slip_0302 molecular chaperone-like protein | 35.1 | 5E-100 |
| SWMB_01869 | Copper chaperone CopZ | 638138574 Swol_1697 copper ion-binding | 92.8 | 5E-41 | 646854691 Slip_1662 copper-translocating P-type ATPase | 35.8 | 2E-08 |
| SWMB_01990 | Heat-inducible transcription repressor HrcA | 638138460 hrcA heat-inducible transcription repressor HrcA | 95.6 | 0 | 646854682 hrcA heat-inducible transcription repressor HrcA | 55.4 | 1E-130 |
| SWMB_01991 | Stress protein H5 | 639331175 Swol_1580 hypothetical protein | 96.7 | 0 | 646853604 groL chaperonin GroEL | 24.6 | 4E-30 |
| SWMB_01992 | HSP-70 cofactor | 638138459 grpE GrpE protein | 94.0 | 6E-113 | 646854681 grpE GrpE protein | 50.9 | 2E-54 |
| SWMB_01993 | Heat shock protein 70 | 638138458 dnaK chaperone DnaK | 98.5 | 0 | 646854680 dnaK chaperone protein DnaK | 77.8 | 0 |
| SWMB_01994 | Chaperone protein DnaJ | 638138457 dnaJ chaperone DnaJ | 99.7 | 0 | 646854679 dnaJ chaperone protein DnaJ | 59.0 | 1E-144 |
| SWMB_02833 | chaperonin GroES | 638138739 groS groes | 97.9 | 3E-64 | 646853603 groS Chaperonin Cpn10 | 70.5 | 2E-45 |
| SWMB_02834 | chaperonin GroEL | 638138738 groL hypothetical protein | 99.3 | 0 | 646853604 groL chaperonin GroEL | 78.6 | 0 |
| | | | | | 646853969 Slip_0937 heat shock protein Hsp20 | | |
| | | | | | 646853980 Slip_0948 hslU heat shock protein HslVU, ATPase subunit HslU | | |
| | | | | | 646854387 Slip_1368 heat shock protein DnaJ domain protein | | |
| Flagellum | | | | | | | |
| SWMB_00150 | Flagellar protein FliS | 638137129 Swol_0232 Flagellin-specific chaperone FliS-like protein | 74.7 | 5E-88 | 646854910 Slip_1882 flagellar protein FliS | 46.1 | 2E-42 |
| SWMB_00153 | Flagellar protein FlaG | 638137130 Swol_0233 hypothetical protein | 50.0 | 2E-34 | 646854911 Slip_1883 flagellar protein FlaG protein | 35.9 | 7E-19 |
| SWMB_00154 | Flagellar protein FlaG | 638137130 Swol_0233 hypothetical protein | 40.6 | 8E-12 | 646854911 Slip_1883 flagellar protein FlaG protein | 58.9 | 3E-25 |
| SWMB_00181 | fliC; flagellin | 638137095 Swol_0198 Flagellin and related hook-associated proteins-like protein | 56.7 | 1E-120 | 646854930 Slip_1903 flagellin domain protein | 51.3 | 4E-36 |

| | | | | | | |
|---|---|---|---|---|---|---|
| SWMB_00182 fliD; flagellar hook-associated protein 2 | 638137094 Swol_0197 Flagellar capping protein-like protein | 36.8 | 4E-25 | 646854931 Slip_1904 flagellar hook-associated 2 domain protein | 42.6 | 6E-28 |
| SWMB_00184 Flagellar assembly factor FliW | 638137092 fliW hypothetical protein | 54.5 | 2E-50 | 646854933 fliW protein of unknown function DUF180 | 47.7 | 1E-53 |
| SWMB_00186 Flagellin | 638137090 Swol_0193 Flagellin and related hook-associated proteins-like protein | 35.9 | 3E-13 | 646854936 Slip_1909 flagellar hook-associated protein 3 | 48.0 | 6E-51 |
| SWMB_00187 flgK; flagellar hook-associated protein 1 FlgK | 638137089 Swol_0192 Flagellar hook-associated protein-like protein | 83.0 | 0 | 646854937 Slip_1910 flagellar hook-associated protein FlgK | 43.6 | 8E-150 |
| SWMB_00189 Flagellar biosynthesis anti-sigma factor FlgM | 638137087 Swol_0190 anti-sigma-28 factor, FlgM family | 95.9 | 3E-54 | 646854939 Slip_1912 anti-sigma-28 factor, FlgM family | 46.5 | 5E-19 |
| SWMB_00190 Flagellar operon protein | 638137086 Swol_0189 flagellar protein | 98.6 | 1E-99 | 646854940 Slip_1913 flagellar protein | 50.4 | 1E-42 |
| SWMB_00551 flgG; flagellar basal-body rod protein FlgG | 638139257 Swol_2373 flagellar basal-body rod protein (flgG-2) | 94.7 | 4E-171 | 646855301 Slip_2274 flagellar basal-body rod protein FlgF | 52.9 | 2E-82 |
| SWMB_00656 Flagellar biosynthetic protein FlhB | 638138354 Swol_1476 flagellar biosynthesis | 98.9 | 8E-57 | 646853949 Slip_0916 type III secretion exporter | 68.6 | 9E-36 |
| SWMB_01125 Flagellar rod assembly protein/muramidase FlgJ | 638137820 Swol_0946 hypothetical protein | 92.4 | 3E-79 | 646853757 Slip_0725 Flagellar protein FlgJ | 49.3 | 1E-23 |
| SWMB_01126 flgG; flagellar basal-body rod protein FlgG | 638137821 Swol_0947 flagellar basal-body rod protein FlgG | 95.8 | 0 | 646853758 Slip_0726 flagellar basal-body rod protein FlgG | 63.2 | 1E-122 |
| SWMB_01321 fliA; RNA polymerase sigma factor for flagellar operon FliA | 638137746 Swol_0878 RNA polymerase, sigma 28 subunit, SigD/FliA/WhiG | 97.3 | 0 | 646854014 Slip_0982 RNA polymerase, sigma 28 subunit, SigD/FliA/WhiG | 63.1 | 6E-107 |
| SWMB_01328 Flagellum site-determining protein YlxH | 638137739 Swol_0871 ParA protein | 92.0 | 0 | 646854007 Slip_0975 Cobyrinic acid ac-diamide synthase | 47.8 | 4E-94 |
| SWMB_01329 Flagella-associated GTP-binding protein | 638137738 Swol_0870 hypothetical protein | 93.8 | 0 | 646854006 Slip_0974 flagellar biosynthetic protein FlhF | 43.1 | 4E-113 |
| SWMB_01330 Flagellar biosynthesis protein FlhA | 638137737 Swol_0869 flagella-associated protein | 96.9 | 0 | 646854005 Slip_0973 flagellar biosynthesis protein FlhA | 70.0 | 0 |
| SWMB_01331 Flagellar biosynthetic protein FlhB | 638137736 Swol_0868 flagellar biosynthesis/type III secretory pathway protein | 96.8 | 0 | 646854004 Slip_0972 flagellar biosynthetic protein FlhB | 58.7 | 2E-155 |
| SWMB_01332 Flagellar biosynthetic protein FliR | 638137735 Swol_0867 flagellar biosynthetic protein FliR | 96.1 | 8E-172 | 646854003 Slip_0971 flagellar biosynthetic protein FliR | 50.6 | 2E-89 |
| SWMB_01333 Flagellar biosynthetic protein FliQ | 638137734 Swol_0866 hypothetical protein | 96.7 | 3E-55 | 646854002 Slip_0970 flagellar biosynthetic protein FliQ | 57.8 | 2E-25 |
| SWMB_01334 Flagellar biosynthetic protein FliP precursor | 638137733 Swol_0865 flagellar biosynthetic protein | 97.1 | 4E-166 | 646854001 Slip_0969 flagellar biosynthetic protein FliP | 69.8 | 4E-118 |
| SWMB_01335 flagella biosynthesis protein FliZ | 638137732 Swol_0864 hypothetical protein | 91.9 | 1E-138 | 646854000 Slip_0968 hypothetical protein | 37.4 | 7E-32 |
| SWMB_01337 Flagellar motor switch protein FliN | 638137730 Swol_0862 CheC, inhibitor of MCP methylation | 91.5 | 0 | 646853998 Slip_0966 CheC, inhibitor of MCP methylation | 57.3 | 2E-151 |
| SWMB_01338 Flagellar motor switch protein FliM | 638137729 Swol_0861 hypothetical protein | 99.1 | 0 | 646853997 Slip_0965 flagellar motor switch protein FliM | 62.5 | 9E-161 |
| SWMB_01339 Flagellar basal body-associated protein FliL | 638137728 Swol_0860 flagellar protein (FliL)-like protein | 96.9 | 2E-109 | 646853996 Slip_0964 flagellar basal body-associated protein FliL | 51.9 | 4E-51 |
| SWMB_01340 Flagellar protein (FlbD) | 638137727 Swol_0859 hypothetical protein | 95.3 | 1E-38 | 646853995 Slip_0963 flagellar FlbD family protein | 61.9 | 6E-24 |
| SWMB_01341 Flagellar hook protein FlgE | 638137726 Swol_0858 flagellar hook protein | 59.4 | 8E-130 | 646853994 Slip_0962 fagellar hook-basal body protein | 40.1 | 9E-49 |
| SWMB_01342 Flagellar operon protein | 638137725 Swol_0857 hypothetical protein | 93.8 | 4E-61 | 646853993 Slip_0961 flagellar operon protein | 54.3 | 9E-39 |
| SWMB_01343 Flagellar basal body rod modification protein | 638137724 Swol_0856 Flagellar hook capping protein-like protein | 83.3 | 9E-106 | 646853992 Slip_0960 flagellar hook capping protein | 43.0 | 5E-34 |
| SWMB_01344 Flagellar hook-length control protein FliK | 638137723 Swol_0855 Flagellar hook-length control protein-like protein | 57.2 | 3E-94 | 646853991 Slip_0959 flagellar hook-length control protein | 40.3 | 2E-21 |
| SWMB_01346 Flagellar export protein FliJ | 638137721 Swol_0853 hypothetical protein | 96.0 | 3E-82 | 646853989 Slip_0957 flagellar export protein FliJ | 35.4 | 7E-18 |
| SWMB_01347 fliI; flagellum-specific ATP synthase | 638137720 Swol_0852 type III secretion system ATPase, FliI/YscN (EC 36315) | 96.6 | 0 | 646853988 Slip_0956 type III secretion system ATPase, FliI/YscN (EC 3.6.3.15) | 70.7 | 0 |
| SWMB_01348 fliH; flagellar assembly protein FliH | 638137719 Swol_0851 Flagellar biosynthesis/type III secretory pathway protein-like protein | 92.1 | 0 | 646853987 Slip_0955 Flagellar assembly protein FliH/Type III secretion system HrpE | 40.0 | 1E-38 |
| SWMB_01349 Flagellar motor switch protein FliG | 639331161 Swol_0850 | 92.1 | 0 | 646853986 Slip_0954 flagellar motor switch protein FliG | 75.1 | 8E-173 |
| SWMB_01350 Flagellar M-ring protein | 638137718 Swol_0849 flagellar M-ring protein FliF | 93.9 | 0 | 646853985 Slip_0953 flagellar M-ring protein FliF | 45.3 | 3E-137 |
| SWMB_01351 Flagellar hook-basal body complex protein FliE | 638137717 fliE flagellar hook-basal body protein | 85.3 | 9E-58 | 646853984 fliE flagellar hook-basal body complex subunit FliE | 52.4 | 7E-29 |
| SWMB_01353 Flagellar basal body rod protein FlgB | 638137715 Swol_0846 hypothetical protein | 91.3 | 8E-88 | 646853982 Slip_0950 flagellar basal-body rod protein FlgB | 48.2 | 1E-38 |
| SWMB_01934 Flagellum site-determining protein YlxH | 638138085 Swol_1211 nucleotide-binding protein | 47.5 | 5E-82 | 646853371 Slip_0341 ATPase-like, ParA/MinD | 52.0 | 1E-85 |
| SWMB_02893 Flagellar motor switch protein | 638137221 Swol_0335 CheC, inhibitor of MCP methylation | 95.8 | 5E-139 | 646854012 Slip_0980 CheC, inhibitor of MCP methylation | 25.3 | 2E-09 |
| SWMB_02606 Flagellar assembly protein H | 638137151 Swol_0257 hypothetical protein | 82.1 | 0 | | | |
| | | | | 646853757 Slip_0725 Flagellar protein FlgJ | | |
| | | | | 646853758 Slip_0726 flagellar basal-body rod protein FlgG | | |
| | | | | 646853983 Slip_0951 flagellar basal-body rod protein FlgC | | |
| | | | | 646853984 Slip_0952 fliE flagellar hook-basal body complex subunit FliE | | |
| | | | | 646854926 Slip_1898 flagellin domain protein | | |
| | | | | 646854932 Slip_1905 carbon storage regulator, CsrA (could also regulate swarming and quorum sensing) | | |
| Pilin | | | | | | |
| SWMB_02048 cpaF, tadA; pilus assembly protein CpaF | 638137415 Swol_0531 type II secretion system protein E | 34.9 | 8E-10 | 646854946 Slip_1919 type II secretion system protein E | 31.0 | 5E-40 |
| SWMB_02057 Flp pilus assembly protein, protease CpaA | 638139006 Swol_2126 butyryl-CoA dehydrogenase (EC 13992) | 27.7 | 0.009 | 646854215 Slip_1187 Prepilin peptidase | 29.6 | 0.0001 |
| SWMB_02441 Fimbrial assembly protein (PilN) | 638137424 Swol_0540 hypothetical protein | 94.6 | 4E-98 | 646854206 Slip_1178 Fimbrial assembly family protein | 25.7 | 0.00002 |
| SWMB_02442 type IV pilus assembly protein PilM | 638137423 Swol_0539 Tfp pilus assembly protein ATPase PilM-like protein | 89.4 | 0 | 646853433 Slip_0403 Tfp pilus assembly protein ATPase PilM | 26.3 | 1E-24 |
| SWMB_02443 pilD, pppA; leader peptidase (prepilin peptidase) / N-methyltransferase | 638137422 Swol_0538 type 4 prepilin peptidase 1 (EC342343) Aspartic peptidase MEROPS family | 93.4 | 5E-135 | 646854215 Slip_1187 Prepilin peptidase | 38.6 | 4E-47 |
| SWMB_02447 putative major pilin subunit | 638137418 Swol_0534 hypothetical protein | 100.0 | 3E-99 | 646854214 Slip_1186 hypothetical protein | 40.0 | 2E-09 |
| SWMB_02448 type IV pilus assembly protein PilC | 638137417 Swol_0533 type II secretion system protein | 99.8 | 0 | 646853295 Slip_0264 Type II secretion system F domain protein | 35.6 | 1E-97 |
| SWMB_02449 twitching motility protein PilT | 638137416 Swol_0532 twitching motility protein | 99.1 | 0 | 646854216 Slip_1188 twitching motility protein | 48.6 | 1E-115 |
| SWMB_02450 type IV pilus assembly protein PilB | 638137415 Swol_0531 type II secretion system protein E | 92.3 | 0 | 646853431 Slip_0401 type II secretion system protein E (GspE) | 51.7 | 2E-146 |
| SWMB_00028 putative protein encoded in hypervariable junctions of pilus gene clusters | 638137455 Swol_0572 hypothetical protein | 42.7 | 5E-24 | | | |
| SWMB_01569 putative protein encoded in hypervariable junctions of pilus gene clusters | 638137455 Swol_0572 hypothetical protein | 30.2 | 0.000007 | | | |
| SWMB_01647 putative protein encoded in hypervariable junctions of pilus gene clusters | 638137455 Swol_0572 hypothetical protein | 39.3 | 5E-20 | | | |
| SWMB_02440 type IV pilus assembly protein PilO | 638137425 Swol_0541 hypothetical protein | 84.2 | 4E-159 | | | |
| SWMB_02444 Tfp pilus assembly protein PilX | 638137421 Swol_0537 hypothetical protein | 91.2 | 4E-89 | | | |
| SWMB_02445 Type II secretory pathway, component PulJ | 638137420 Swol_0536 hypothetical protein | 97.4 | 6E-110 | | | |
| SWMB_02446 putative type IV pilin N-term methylation site GFxxxE | 638137419 Swol_0535 hypothetical protein | 99.3 | 2E-101 | | | |
| SWMB_03047 putative protein encoded in hypervariable junctions of pilus gene clusters | 638137455 Swol_0572 hypothetical protein | 23.7 | 0.0004 | | | |
| SWMB_02458 Verru_Chthon cassette protein D | | | | 646853426 Slip_0396 pilin assembly protein | 37.0 | 2E-23 |
| | | | | 646854951 Slip_1924 peptidase A24A prepilin type IV | | |
| | | | | 646854952 Slip_1925 Flp/Fap pilin component | | |
| | | | | 646854008 Slip_0976 type IV pilus assembly PilZ | | |
| | | | | 646854046 Slip_1014 type IV pilus assembly PilZ | | |
| | | | | 646853435 Slip_0405 Pilus assembly protein, PilO | | |
| | | | | 646853434 Slip_0404 Fimbrial assembly family protein | | |
| | | | | 646853432 Slip_0402 Type 4 fimbrial biogenesis protein PilX, N-terminal domain | | |
| | | | | 646854218 Slip_1190 PilT protein domain protein | | |
| Sporulation | | | | | | |
| SWMB_00101 Sporulation-specific N-acetylmuramoyl-L-alanine amidase | 638137026 Swol_0126 N-acetylmuramoyl-L-alanine amidase | 36.6 | 3E-20 | 646853299 Slip_0268 cell wall hydrolase/autolysin | 35.0 | 1E-20 |
| SWMB_00122 Sporulation transcription regulator WhiA | 638137157 whiA hypothetical protein | 95.9 | 0 | 646854883 whiA protein of unknown function DUF199 | 50.8 | 5E-100 |
| SWMB_00269 Stage II sporulation protein E | 638136995 Swol_0093 Phosphoprotein phosphatase | 94.8 | 0 | 646853170 Slip_0126 protein serine/threonine phosphatase | 42.6 | 0 |
| SWMB_00274 spore cortex biosynthesis protein YabQ | 638136988 Swol_0086 hypothetical protein | 95.1 | 4E-83 | 646853159 Slip_0115 Spore cortex biosynthesis protein, YabQ | 39.8 | 5E-19 |
| SWMB_00275 Spore protein YabP | 638136987 Swol_0085 hypothetical protein | 100.0 | 4E-59 | 646853158 Slip_0114 sporulation protein YabP | 44.4 | 1E-23 |
| SWMB_00281 Stage III sporulation protein F | 638136983 Swol_0081 stage V sporulation protein B | 97.7 | 0 | 646853154 Slip_0110 polysaccharide biosynthesis protein | 66.7 | 0 |
| SWMB_00282 Stage V sporulation protein T | 638136982 Swol_0080 transcriptional regulator, AbrB family | 98.9 | 2E-130 | 646853153 Slip_0109 transcriptional regulator, AbrB family | 73.9 | 8E-92 |
| SWMB_00283 Spore germination protein YndE | 638136981 Swol_0079 hypothetical protein | 93.6 | 0 | 646853328 Slip_0297 spore germination protein | 28.4 | 7E-23 |
| SWMB_00284 Spore germination protein B1 | 638136980 Swol_0078 germination protein | 95.4 | 0 | 646853329 Slip_0298 GerA spore germination protein | 49.9 | 0 |
| SWMB_00285 Spore germination protein B3 precursor | 638136979 Swol_0077 hypothetical protein | 91.9 | 0 | 646853327 Slip_0296 germination protein, Ger(x)C family | 36.4 | 1E-86 |
| SWMB_00286 Spore germination protein B3 precursor | 638136977 Swol_0075 hypothetical protein | 88.7 | 0 | 646853327 Slip_0296 germination protein, Ger(x)C family | 33.8 | 1E-67 |
| SWMB_00288 spore germination protein (amino acid permease) | 638136976 Swol_0074 hypothetical protein | 88.0 | 0 | 646854095 Slip_1063 spore germination protein | 23.6 | 1E-14 |
| SWMB_00299 stage II sporulation protein R | 638136966 Swol_0063 pro-sigma-E processing factor spoIIR | 96.8 | 1E-134 | 646853144 Slip_0100 stage II sporulation protein R | 58.3 | 2E-56 |
| SWMB_00313 sporulation lipoprotein, YhcN/YlaJ family | 638136955 Swol_0052 hypothetical protein | 95.1 | 2E-141 | 646853108 Slip_0057 Sporulation lipoprotein YhcN/YlaJ | 29.5 | 2E-14 |
| SWMB_00376 Stage III sporulation protein J | 639331191 Swol_2574 protein translocase subunit yidC | 98.1 | 6E-156 | 646855407 Slip_2382 protein translocase subunit yidC | 54.2 | 9E-95 |
| SWMB_00382 Sporulation initiation inhibitor protein soj | 638139452 Swol_2568 chromosome segregation ATPase | 97.3 | 0 | 646855401 Slip_2376 chromosome segregation ATPase | 65.6 | 1E-124 |
| SWMB_00387 putative sporulation protein YyaC | 638139447 Swol_2563 YyaC | 93.1 | 5E-142 | 646855394 Slip_2369 sporulation protein YyaC | 55.7 | 3E-66 |
| SWMB_00404 Sporulation kinase E | 638139431 Swol_2546 Signal transduction histidine kinase-like protein | 20.3 | 0.0007 | 646854150 Slip_1119 putative PAS/PAC sensor protein | 23.4 | 0.00005 |
| SWMB_00512 Sporulation initiation phosphotransferase F | 638139296 Swol_2412 response regulator receiver protein | 98.4 | 6E-85 | 646855336 Slip_2311 response regulator receiver protein | 56.6 | 2E-45 |
| SWMB_00576 Stage 0 sporulation protein H | 638139237 Swol_2351 RNA polymerase, sigma 30 subunit, SigH | 100.0 | 1E-159 | 646855281 Slip_2254 RNA polymerase, sigma 30 subunit, SigH | 84.4 | 8E-130 |
| SWMB_00669 Sporulation initiation inhibitor protein soj | 638138334 Swol_1454 chromosome partitioning protein | 93.5 | 0 | 646854760 Slip_1729 Cobyrinic acid ac-diamide synthase | 68.1 | 5E-131 |
| SWMB_00717 putative spore protein YtfJ | 638138294 Swol_1414 hypothetical protein | 100.0 | 3E-98 | 646853583 Slip_0552 sporulation protein YtfJ | 66.1 | 1E-55 |
| SWMB_00718 Spore maturation protein A | 638138293 Swol_1413 nucleoside recognition proteinn | 97.5 | 2E-137 | 646853584 Slip_0553 nucleoside recognition domain protein | 71.4 | 3E-103 |
| SWMB_00719 Spore maturation protein B | 638138292 Swol_1412 nucleoside recognition proteinn | 98.3 | 4E-124 | 646853585 Slip_0554 nucleoside recognition domain protein | 74.2 | 5E-99 |
| SWMB_00815 stage II sporulation protein P | 638138220 Swol_1339 putative stage II sporulation protein P | 97.7 | 0 | 646854498 Slip_1476 stage II sporulation protein P | 47.6 | 3E-106 |
| SWMB_00833 Stage IV sporulation protein A | 638138203 Swol_1322 hypothetical protein | 98.8 | 0 | 646854492 Slip_1470 stage IV sporulation protein A | 77.6 | 0 |
| SWMB_00886 Stage III sporulation protein E | 638138140 Swol_1265 DNA translocase FtsK | 95.7 | 0 | 646854070 Slip_1038 DNA translocase FtsK | 53.4 | 0 |
| SWMB_01127 sporulation protein, YlmC/YmxH family | 638137822 Swol_0948 hypothetical protein | 97.9 | 5E-63 | 646853843 Slip_0810 sporulation protein, YlmC/YmxH family | 64.5 | 4E-32 |
| SWMB_01186 Stage V sporulation protein K | 638137856 Swol_0983 SpoVK | 99.3 | 0 | 646854111 Slip_1080 AAA ATPase central domain protein | 63.5 | 4E-122 |
| SWMB_01255 Spore germination protein | 638137923 Swol_1052 Spore germination protein-like protein | 95.1 | 0 | 646853068 Slip_0022 Spore germination protein-like Gmad2 | 58.6 | 1E-106 |
| SWMB_01258 Sporulation kinase E | 638137925 Swol_1054 PAS/PAC sensor signal transduction histidine kinase (EC 27133) | 89.2 | 0 | 646853849 Slip_0816 PAS/PAC sensor signal transduction histidine kinase (EC 2.7.13.3) | 24.9 | 4E-21 |
| SWMB_01267 Sporulation protein YunB | 638137797 Swol_0931 hypothetical protein | 98.2 | 4E-155 | 646853750 Slip_0723 sporulation protein YunB | 51.7 | 4E-66 |
| SWMB_01281 Small, acid-soluble spore proteins, alpha/beta type | 638137784 Swol_0918 hypothetical protein | 98.3 | 5E-36 | 646854098 Slip_1067 hypothetical protein | 63.2 | 5E-25 |
| SWMB_01287 sporulation protein, YlmC/YmxH family | 638137778 Swol_0912 hypothetical protein | 97.8 | 2E-58 | 646854048 Slip_1016 sporulation protein, YlmC/YmxH family | 67.1 | 9E-42 |
| SWMB_01310 sporulation integral membrane protein YtvI | 638137756 Swol_0888 protein of unknown function UPF0118 | 95.1 | 0 | 646854024 Slip_0992 sporulation integral membrane protein YtvI | 44.0 | 8E-87 |
| SWMB_01365 Stage II sporulation protein AC | 638137706 Swol_0836 RNA polymerase, sigma subunit, RpsG/SigG | 99.6 | 3E-167 | 646853841 Slip_0808 RNA polymerase, sigma subunit, RpsG/SigG | 80.2 | 6E-134 |
| SWMB_01368 sigma-E processing peptidase SpoIIGA | 638137704 Swol_0834 sporulation factor SpoIIGA Unknown type peptidase MEROPS family U04 | 95.9 | 0 | 646853839 Slip_0806 sporulation factor SpoIIGA. Unknown type peptidase. MEROPS family U04 | 47.2 | 3E-96 |
| SWMB_01382 Sporulation-specific penicillin-binding protein | 638137690 Swol_0820 Peptidoglycan glycosyltransferase | 97.9 | 0 | 646853822 Slip_0789 stage V sporulation protein D | 63.5 | 0 |
| SWMB_01383 Septum formation initiator | 638137689 Swol_0819 hypothetical protein | 94.6 | 2E-115 | 646853821 ftsL cell division protein FtsL | 30.8 | 1E-14 |
| SWMB_01427 putative sporulation protein YtaF | 638138897 Swol_2015 integral membrane protein | 95.7 | 7E-140 | 646854723 Slip_1693 sporulation protein YtaF | 50.7 | 1E-51 |
| SWMB_01661 Sporulation-specific penicillin-binding protein | 638137374 Swol_0489 Peptidoglycan glycosyltransferase | 92.3 | 0 | 646853396 Slip_0366 Peptidoglycan glycosyltransferase | 42.5 | 2E-146 |
| SWMB_01691 Spore germination protein GerM | 638137334 Swol_0447 putative germination protein GerM | 92.8 | 1E-132 | 646855084 Slip_2057 Lipoprotein LpqB, GerMN domain protein | 43.1 | 1E-45 |
| SWMB_01787 putative sporulation protein YtxC | 638137957 Swol_1087 hypothetical protein | 98.1 | 0 | 646854523 Slip_1501 Sporulation protein YtxC | 44.0 | 2E-92 |
| SWMB_01847 Putative sporulation-specific glycosylase YdhD | 638138754 Swol_1871 glycosyl hydrolase-like protein | 33.8 | 1E-08 | 646854175 Slip_1146 copper amine oxidase domain protein | 28.5 | 8E-06 |
| SWMB_01962 Spo0B-associated GTP-binding protein | 638138488 obg spo0B-associated GTP-binding protein | 96.7 | 0 | 646853736 obg GTP-binding protein Obg/CgtA | 67.4 | 0 |
| SWMB_01985 stage II sporulation protein P | 638138465 Swol_1586 hypothetical protein | 94.5 | 0 | 646854686 Slip_1657 stage II sporulation protein P | 41.1 | 3E-87 |
| SWMB_02003 sporulation protein YqfC | 638138448 Swol_1568 hypothetical protein | 100.0 | 1E-60 | 646854671 Slip_1642 sporulation protein YqfC | 56.0 | 3E-34 |
| SWMB_02004 sporulation protein YqfD | 638138447 Swol_1567 putative stage IV sporulation YqfD | 93.1 | 0 | 646854670 Slip_1641 sporulation protein YqfD | 40.7 | 2E-111 |
| SWMB_02028 Spore coat protein SA | 638137839 Swol_0965 1,2-diacylglycerol 3-glucosyltransferase (EC 241157) | 34.2 | 0.0004 | 646853663 Slip_0632 glycosyl transferase group 1 | 28.3 | 9E-12 |
| SWMB_02044 Sporulation initiation inhibitor protein soj | 638139452 Swol_2568 chromosome segregation ATPase | 36.6 | 6E-58 | 646855401 Slip_2376 chromosome segregation ATPase | 41.1 | 9E-65 |
| SWMB_02108 stage V sporulation protein AC | 638137505 Swol_0626 spore formation protein spoVAC | 95.5 | 1E-106 | 646853559 Slip_0528 stage V sporulation protein AC | 56.4 | 9E-60 |
| SWMB_02109 Stage V sporulation protein AD | 638137506 Swol_0627 stage V sporulation AD | 96.7 | 0 | 646853560 Slip_0529 stage V sporulation protein AD | 62.0 | 8E-152 |
| SWMB_02110 stage V sporulation protein AE | 638137507 Swol_0628 SpoVA protein | 95.8 | 3E-60 | 646853561 Slip_0530 stage V sporulation protein AE | 70.3 | 4E-44 |
| SWMB_02111 Spore germination protein B1 | 638137508 Swol_0629 GerA spore germination protein | 97.6 | 0 | 646853329 Slip_0298 GerA spore germination protein | 31.5 | 3E-72 |
| SWMB_02375 stage II sporulation protein P | 638138000 Swol_1127 putative stage II sporulation protein P | 93.7 | 0 | 646854257 Slip_1229 stage II sporulation protein P | 46.5 | 6E-97 |
| SWMB_02422 Stage III sporulation protein AH | 638137441 Swol_0557 hypothetical protein | 87.0 | 1E-116 | 646853451 Slip_0421 hypothetical protein | 36.7 | 2E-21 |
| SWMB_02423 stage III sporulation protein AG | 638137440 Swol_0556 hypothetical protein | 97.4 | 2E-135 | 646853450 Slip_0420 hypothetical protein | 41.1 | 9E-36 |
| SWMB_02424 stage III sporulation protein AF | 638137439 Swol_0555 hypothetical protein | 92.3 | 2E-122 | 646853449 Slip_0419 Sporulation stage III protein AF | 36.2 | 2E-43 |
| SWMB_02425 Stage III sporulation protein AE precursor | 638137438 Swol_0554 hypothetical protein | 97.2 | 0 | 646853448 Slip_0418 stage III sporulation protein AE | 43.6 | 7E-112 |
| SWMB_02426 stage III sporulation protein AD | 638137437 Swol_0553 putative sporulation protein | 99.2 | 4E-77 | 646853447 Slip_0417 stage III sporulation protein AD | 60.3 | 3E-54 |
| SWMB_02427 stage III sporulation protein AC | 638137436 Swol_0552 hypothetical protein | 100.0 | 9E-32 | 646853446 Slip_0416 stage III sporulation protein AC | 78.1 | 1E-29 |
| SWMB_02428 stage III sporulation protein SpoAB | 638137435 Swol_0551 hypothetical protein | 94.3 | 4E-107 | 646853445 Slip_0415 Sporulation stage III protein AB | 41.9 | 7E-39 |
| SWMB_02532 Sporulation initiation inhibitor protein soj | 638139452 Swol_2568 chromosome segregation ATPase | 35.2 | 3E-55 | 646855401 Slip_2376 chromosome segregation ATPase | 40.2 | 3E-63 |
| SWMB_02549 Stage II sporulation protein AC | 638137494 Swol_0615 RNA polymerase, sigma subunit, RpoX/SigF | 97.1 | 2E-172 | 646853558 Slip_0527 RNA polymerase, sigma subunit, RpoX/SigF | 60.5 | 4E-102 |
| SWMB_02550 stage II sporulation protein AB (anti-sigma F factor) | 638137493 Swol_0614 putative anti-sigma regulatory factor, serine/threonine protein kinase | 95.3 | 1E-99 | 646853557 Slip_0526 putative anti-sigma regulatory factor, serine/threonine protein kinase | 56.1 | 2E-61 |
| SWMB_02551 Stage II sporulation protein AA | 638137492 Swol_0613 anti-anti-sigma regulatory factor, SpoIIAA | 96.4 | 8E-70 | 646853556 Slip_0525 anti-anti-sigma regulatory factor, SpoIIAA | 50.0 | 2E-37 |
| SWMB_02562 Stage II sporulation protein M | 638137484 Swol_0605 uncharacterized membrane protein | 96.1 | 5E-136 | 646853521 Slip_0491 stage II sporulation protein M | 50.3 | 3E-64 |
| SWMB_02576 Stage 0 sporulation protein A | 638137469 Swol_0590 response regulator receiver protein | 98.5 | 0 | 646853471 Slip_0441 response regulator receiver protein | 69.6 | 2E-136 |
| SWMB_02577 SpoIVB peptidase precursor | 638137468 Swol_0589 SpoIVB peptidase Serine peptidase MEROPS family S55 | 97.1 | 0 | 646853470 Slip_0440 SpoIVB peptidase. Serine peptidase. MEROPS family S55 | 55.6 | 5E-174 |
| SWMB_02649 polysaccharide deacetylase family sporulation protein PdaB | 638137774 Swol_0908 xylanase/chitin deacetylase | 33.3 | 0.002 | 646853651 Slip_0620 polysaccharide deacetylase | 28.4 | 0.00007 |
| SWMB_02684 putative sporulation protein YyaC | 638138070 Swol_1197 hypothetical protein | 88.7 | 2E-115 | 646854398 Slip_1379 sporulation protein YyaC | 43.1 | 1E-44 |
| SWMB_02687 Spore germination protein B1 | 638138067 Swol_1194 grkA; spore germination protein, GrkA | 88.3 | 0 | 646853329 Slip_0298 GerA spore germination protein | 44.8 | 2E-145 |
| SWMB_02688 Spore germination protein A3 precursor | 638138066 Swol_1193 hypothetical protein | 87.2 | 0 | 646853327 Slip_0296 germination protein, Ger(x)C family | 29.9 | 6E-44 |
| SWMB_02689 Spore germination protein YndE | 638138065 Swol_1192 hypothetical protein | 92.3 | 0 | 646854095 Slip_1063 spore germination protein | 31.0 | 7E-38 |
| SWMB_02772 Stage V sporulation protein S | 638138125 spoVS stage V sporulation protein S | 98.8 | 1E-54 | 646854085 spoVS2 Stage V sporulation protein S | 96.5 | 3E-53 |
| SWMB_02796 Sporulation integral membrane protein YlbJ | 638137635 Swol_0764 hypothetical protein | 95.9 | 0 | 646853932 Slip_0899 sporulation integral membrane protein YlbJ | 47.6 | 2E-112 |
| SWMB_02943 Spore coat protein | 638138779 Swol_1896 hypothetical protein | 97.6 | 1E-57 | 646854397 Slip_1378 Coat F domain protein | 65.8 | 2E-34 |
| SWMB_02969 Sporulation kinase E | 638138800 Swol_1918 PAS/PAC sensor signal transduction histidine kinase (EC 27133) | 85.2 | 0 | 646854150 Slip_1119 putative PAS/PAC sensor protein | 20.7 | 6E-08 |
| SWMB_02999 Putative sporulation-specific glycosylase YdhD | 638138754 Swol_1871 glycosyl hydrolase-like protein | 92.0 | 0 | 646854968 Slip_1942 copper amine oxidase domain protein | 28.1 | 2E-29 |
| SWMB_00549 Sporulation initiation inhibitor protein soj | 638139259 Swol_2375 stage III sporulation protein D | 96.3 | 6E-54 | 646855303 Slip_2276 sporulation transcriptional regulator SpoIIID | 83.5 | 7E-45 |

| | | | | | | |
|---|---|---|---|---|---|---|
| SWMB_00452 Stage IV sporulation protein H | 638139344 Swol_2460 hypothetical protein | 92.8 | 8E-61 | | | |
| SWMB_02858 Spore photoproduct lyase | 638138721 Swol_1838 hypothetical protein | 98.5 | 0 | | | |
| SWMB_01848 Spore germination protein YaaH | 638138754 Swol_1871 glycosyl hydrolase-like protein | 32.8 | 4E-18 | | | |
| SWMB_00979 Inner spore coat protein H | 638139060 Swol_2180 hypothetical protein | 96.9 | 0 | | | |
| SWMB_01097 spore cortex-lytic enzyme | 638138945 Swol_2063 hypothetical protein | 91.3 | 0 | | | |
| SWMB_02715 Endospore coat-associated protein YheD | 638138047 Swol_1174 hypothetical protein | 95.7 | 0 | | | |
| SWMB_02721 Endospore coat-associated protein YheD | 638138041 Swol_1168 YheD | 95.1 | 0 | | | |
| SWMB_02722 Endospore coat-associated protein YheD | 638138038 Swol_1165 hypothetical protein | 92.6 | 0 | | | |
| SWMB_01409 Spo0E like sporulation regulatory protein | | | | | | |
| SWMB_02394 acid-soluble spore protein H | | | | | | |
| | | | | 646853322 Slip_0291 spore germination protein | | |
| | | | | 646853323 Slip_0292 germination protein, Ger(x)C family | | |
| | | | | 646853324 Slip_0293 spore germination protein | | |
| | | | | 646853326 Slip_0295 germination protein, Ger(x)C family | | |
| | | | | 646853444 Slip_0414 stage III sporulation protein AA | | |
| | | | | 646853926 Slip_0893 small acid-soluble spore protein alpha/beta type | | |
| | | | | 646853927 Slip_0894 spore protease | | |
| | | | | 646854085 Slip_1053 Stage V sporulation protein S | | |
| | | | | 646854094 Slip_1062 GerA spore germination protein | | |
| | | | | 646854534 Slip_1511 sporulation lipoprotein, YhcN/YlaJ family | | |
| | | | | 646854536 Slip_1513 polysaccharide deacetylase family sporulation protein PdaB | | |
| | | | | 646854736 Slip_1705 small acid-soluble spore protein | | |
| Chemotaxis | | | | | | |
| SWMB_00143 Chemotaxis protein MotB | 638137137 Swol_0240 hypothetical protein | 94.2 | 0 | 646854904 Slip_1876 OmpA/MotB domain protein | 61.1 | 8E-117 |
| SWMB_00144 Chemotaxis protein PomA | 638137136 Swol_0239 hypothetical protein | 96.2 | 0 | 646854905 Slip_1877 MotA/TolQ/ExbB proton channel | 66.5 | 3E-123 |
| SWMB_00666 Chemotaxis protein CheA | 638138337 Swol_1457 MCP methyltransferase, CheR-type | 99.6 | 0 | 646854757 Slip_1726 MCP methyltransferase, CheR-type | 70.3 | 9E-150 |
| SWMB_00667 two-component system, chemotaxis family, response regulator CheY | 638138336 Swol_1456 response regulator receiver protein | 98.4 | 6E-86 | 646854758 Slip_1727 response regulator receiver protein | 65.6 | 5E-58 |
| SWMB_00668 two-component system, chemotaxis family, sensor kinase CheA | 638138335 Swol_1455 CheA signal transduction histidine kinase | 92.3 | 0 | 646854759 Slip_1728 CheA signal transduction histidine kinase | 51.8 | 0 |
| SWMB_00672 Methyl-accepting chemotaxis protein 4 | 638138331 Swol_1451 putative methyl-accepting chemotaxis sensory transducer | 26.1 | 3.2 | 646854770 Slip_1739 methyl-accepting chemotaxis sensory transducer | 32.9 | 5E-19 |
| SWMB_00673 Serine chemoreceptor protein | 638138331 Swol_1451 putative methyl-accepting chemotaxis sensory transducer | 23.9 | 6.2 | 646854770 Slip_1739 methyl-accepting chemotaxis sensory transducer | 27.2 | 6E-20 |
| SWMB_00674 Chemotaxis protein CheW | 638138330 Swol_1450 CheW protein | 99.8 | 0 | 646854764 Slip_1733 CheW protein | 36.6 | 4E-56 |
| SWMB_00675 Chemotaxis response regulator protein-glutamate methylesterase | 638138329 cheB3 Protein-glutamate methylesterase | 100.0 | 0 | 646854765 cheB response regulator receiver modulated CheB methylesterase | 72.8 | 0 |
| SWMB_00676 Chemotaxis protein CheY | 638138328 Swol_1448 response regulator receiver protein | 100.0 | 6E-90 | 646854766 Slip_1735 response regulator receiver protein | 71.4 | 4E-63 |
| SWMB_00677 CheY-P phosphatase CheX | 638138327 Swol_1447 chemotaxis operon protein | 100.0 | 1E-112 | 646854767 Slip_1736 chemotaxis operon protein | 62.4 | 9E-72 |
| SWMB_00678 CheY-P phosphatase CheX | 638138326 Swol_1446 CheX protein (uncharacterized ORF in chemotaxis operon) | 98.7 | 2E-107 | 646854768 Slip_1737 CheC domain protein | 60.5 | 4E-64 |
| SWMB_00761 mcp; methyl-accepting chemotaxis protein | 638137793 Swol_0927 putative methyl-accepting chemotaxis sensory transducer | 27.2 | 7E-23 | 646854384 Slip_1365 methyl-accepting chemotaxis sensory transducer | 24.5 | 1E-19 |
| SWMB_00821 Chemotaxis protein CheY | 638138214 Swol_1333 response regulator receiver protein | 93.2 | 5E-77 | 646853999 Slip_0967 response regulator receiver protein | 61.7 | 7E-48 |
| SWMB_00822 Chemotaxis protein CheA | 638138213 Swol_1332 putative CheA signal transduction histidine kinases | 95.0 | 0 | 646854010 Slip_0978 CheA signal transduction histidine kinase | 29.4 | 7E-99 |
| SWMB_00823 Chemotaxis protein CheW | 638138330 Swol_1450 CheW protein | 35.6 | 3E-26 | 646854818 Slip_1789 CheW protein | 39.4 | 2E-27 |
| SWMB_00824 Dipeptide chemoreceptor protein | 638138212 Swol_1331 methyl-accepting chemotaxis sensory transducer | 25.5 | 0.5 | 646854384 Slip_1365 methyl-accepting chemotaxis sensory transducer | 28.7 | 1E-21 |
| SWMB_00825 Chemotaxis protein methyltransferase | 638138211 Swol_1330 MCP methyltransferase, CheR-type | 93.0 | 0 | 646854440 Slip_1419 MCP methyltransferase, CheR-type | 35.4 | 4E-44 |
| SWMB_00826 Chemotaxis response regulator protein-glutamate methylesterase | 638138210 cheB2 Protein-glutamate methylesterase | 95.9 | 0 | 646854009 cheB response regulator receiver modulated CheB methylesterase | 37.1 | 7E-70 |
| SWMB_00827 Sensory/regulatory protein RpfC | 638138209 Swol_1328 PAS/PAC sensor hybrid histidine kinase (EC 27133) | 93.2 | 0 | 646853849 Slip_0816 PAS/PAC sensor signal transduction histidine kinase (EC 2.7.13.3) | 24.2 | 0.008 |
| SWMB_00828 Sensory/regulatory protein RpfC | 638138208 Swol_1327 response regulator receiver protein | 98.5 | 5E-91 | 646854758 Slip_1727 response regulator receiver protein | 30.2 | 5E-12 |
| SWMB_01322 Chemoreceptor glutamine deamidase CheD | 638137745 cheD CheD, stimulates methylation of MCP proteins | 98.8 | 3E-115 | 646854013 cheD CheD, stimulates methylation of MCP proteins | 68.3 | 1E-82 |
| SWMB_01323 CheY-P phosphatase CheC | 638137744 Swol_0876 CheC, inhibitor of MCP methylation | 95.2 | 1E-141 | 646854012 Slip_0980 CheC, inhibitor of MCP methylation | 65.7 | 1E-94 |
| SWMB_01324 Chemotaxis protein CheW | 638137743 Swol_0875 CheW protein | 87.7 | 5E-84 | 646854011 Slip_0979 CheW protein | 53.2 | 4E-52 |
| SWMB_01325 Chemotaxis protein CheA | 638137742 Swol_0874 putative CheA signal transduction histidine kinases | 90.7 | 0 | 646854010 Slip_0978 CheA signal transduction histidine kinase | 57.9 | 0 |
| SWMB_01326 Chemotaxis response regulator protein-glutamate methylesterase | 638137741 cheB1 Protein-glutamate methylesterase | 85.2 | 0 | 646854009 cheB response regulator receiver modulated CheB methylesterase | 52.5 | 8E-133 |
| SWMB_01336 Chemotaxis protein CheY | 638137731 Swol_0863 response regulator receiver protein | 96.7 | 5E-79 | 646853999 Slip_0967 response regulator receiver protein | 84.9 | 2E-71 |
| SWMB_01758 Chemotaxis protein CheY | 638137245 Swol_0355 response regulator receiver protein | 93.2 | 3E-162 | 646853999 Slip_0967 response regulator receiver protein | 49.6 | 2E-36 |
| SWMB_01759 Chemotaxis protein CheA | 638137244 Swol_0353 putative CheA signal transduction histidine kinases | 91.9 | 0 | 646854759 Slip_1728 CheA signal transduction histidine kinase | 37.9 | 6E-35 |
| SWMB_02179 Chemotaxis protein methyltransferase | 638137569 Swol_0695 MCP methyltransferase, CheR-type | 95.4 | 0 | 646854440 Slip_1419 MCP methyltransferase, CheR-type | 61.8 | 1E-118 |
| SWMB_02511 Methyl-accepting chemotaxis protein | 638138723 Swol_1840 putative methyl-accepting chemotaxis sensory transducer | 21.2 | 0.0008 | 646854088 Slip_1056 methyl-accepting chemotaxis sensory transducer | 27.7 | 3E-10 |
| SWMB_02894 Chemotaxis protein CheY | 638137220 Swol_0334 response regulator receiver protein | 87.8 | 1E-77 | 646854766 Slip_1735 response regulator receiver protein | 36.6 | 5E-19 |
| SWMB_02923 Chemotaxis protein CheW | 638138769 Swol_1886 CheW protein | 98.7 | 4E-108 | 646854818 Slip_1789 CheW protein | 70.7 | 3E-74 |
| SWMB_01016 putative methyl-accepting chemotaxis protein (MCP), signaling domain | 638139031 Swol_2151 methyl-accepting chemotaxis sensory transducer with Cache sensor | 93.1 | 0 | 646854279 Slip_1250 methyl-accepting chemotaxis sensory transducer with Cache sensor | 28.2 | 1E-61 |
| SWMB_01001 putative methyl-accepting chemotaxis protein (MCP), signaling domain | 638139042 Swol_2162 methyl-accepting chemotaxis sensory transducer with Cache sensor | 92.9 | 0 | 646854821 Slip_1792 methyl-accepting chemotaxis sensory transducer | 42.2 | 2E-94 |
| | | | | 646853503 Slip_0473 methyl-accepting chemotaxis sensory transducer | | |
| | | | | 646853618 Slip_0587 methyl-accepting chemotaxis sensory transducer | | |
| | | | | 646854067 Slip_1035 methyl-accepting chemotaxis sensory transducer | | |
| | | | | 646854217 Slip_1189 methyl-accepting chemotaxis sensory transducer | | |
| | | | | 646854763 Slip_1732 methyl-accepting chemotaxis sensory transducer | | |
| | | | | 646854765 Slip_1734 response regulator receiver modulated CheB methylesterase | | |
| | | | | 646855119 Slip_2092 methyl-accepting chemotaxis sensory transducer | | |
| | | | | 646855154 Slip_2131 methyl-accepting chemotaxis sensory transducer with Cache sensor | | |
| | | | | 646854009 Slip_0977 response regulator receiver modulated CheB methylesterase | | |
| PHA biosynthesis | | | | | | |
| SWMB_02134 putative enoyl-CoA hydratase echA8 | 638137527 Swol_0650 putative crotonase | 98.4 | 0 | 646853532 Slip_0502 short chain enoyl-CoA hydratase (EC 4.2.1.17) | 32.8 | 1E-42 |
| SWMB_00384 putative enoyl-CoA hydratase echA8 | 638139450 Swol_2566 3-hydroxybutyryl-CoA dehydratase | 87.4 | 3E-161 | 646855116 Slip_2089 Enoyl-CoA hydratase/isomerase | 37.2 | 2E-44 |
| SWMB_02135 putative acetoacetyl-CoA reductase | 638137528 Swol_0651 oxidoreductase, short chain dehydrogenase/reductase family | 94.8 | 6E-159 | 646853238 Slip_0202 short-chain dehydrogenase/reductase SDR | 39.1 | 7E-54 |
| SWMB_02626 putative acetoacetyl-CoA reductase | 638138792 Swol_1910 3-oxoacyl-acyl-carrier-protein reductase (EC 111100) | 29.6 | 7E-29 | 646854468 Slip_1447 3-oxoacyl-acyl-carrier-protein reductase (EC 1.1.1.100) | 31.0 | 4E-34 |
| SWMB_02933 putative acetoacetyl-CoA reductase | 638138792 Swol_1910 3-oxoacyl-acyl-carrier-protein reductase (EC 111100) | 96.8 | 2E-179 | 646854468 Slip_1447 3-oxoacyl-acyl-carrier-protein reductase (EC 1.1.1.100) | 39.3 | 3E-58 |
| SWMB_02672 poly(hydroxyalkanoate) granule-associated protein | 638138081 Swol_1208 hypothetical protein | 99.0 | 9E-66 | 646854419 Slip_1400 hypothetical protein | 37.9 | 3E-21 |
| SWMB_01774 Poly-beta-hydroxybutyrate polymerase | 638137969 Swol_1099 poly(R)-hydroxyalkanoic acid synthase, class III, PhaC subunit | 97.8 | 0 | | | |
| SWMB_02363 Long-chain-fatty-acid--CoA ligase | 638138017 Swol_1144 acyl-CoA synthase | 93.6 | 0 | | | |
| SWMB_02362 Poly-beta-hydroxybutyrate polymerase | 638138018 Swol_1145 hypothetical protein | 95.2 | 0 | | | |
| SWMB_02763 Poly-beta-hydroxybutyrate polymerase | 638138116 Swol_1241 poly(R)-hydroxyalkanoic acid synthase, class III, PhaC subunit | 95.7 | 1E-130 | | | |
| SWMB_02764 (R)-specific enoyl-CoA hydratase | 638138117 Swol_1242 acyl dehydratase | 100.0 | 2E-104 | 646855117 Slip_2090 MaoC domain protein dehydratase | 47.4 | 3E-40 |
| SWMB_02765 polyhydroxyalkanoic acid synthase, PhaR subunit | 638138118 Swol_1243 hypothetical protein | 91.4 | 4E-119 | | | |
| SWMB_02766 poly(hydroxyalkanoate) granule-associated protein | 638138119 Swol_1244 hypothetical protein | 98.3 | 1E-78 | | | |
| CRISPR-associated proteins | | | | | | |
| SWMB_01201 putative CRISPR-associated RAMP protein, Csm4 family | 638138635 Swol_1753 CRISPR-associated protein, Csm4 family | 38.1 | 0.16 | | | |
| SWMB_01202 CRISPR type III-A/MTUBE-associated RAMP protein Csm4 | 638138635 Swol_1753 CRISPR-associated protein, Csm4 family | 29.2 | 1E-15 | | | |
| SWMB_01203 CRISPR type III-A/MTUBE-associated RAMP protein Csm5 | 638138634 Swol_1752 CRISPR-associated protein, Csm5 family | 25.7 | 0.0007 | | | |
| SWMB_01204 putative CRISPR-associated protein Cas6 | 638139398 Swol_2514 conserved hypothetical cytosolic protein | 31.6 | 3E-15 | | | |
| SWMB_01205 putative CRISPR-associated protein Cas6 | 638139398 Swol_2514 conserved hypothetical cytosolic protein | 46.3 | 4E-09 | | | |
| SWMB_01206 CRISPR/Cas system-associated protein Csx1 | | | | | | |
| | | | | | | |
| SWMB_01259 CRISPR-associated endoribonuclease Cas6 | | | | 646854360 Slip_1341 CRISPR-associated protein, Cas6 family | 27.8 | 3E-29 |
| SWMB_01260 CRISPR-associated protein, family | 638139406 Swol_2522 CRISPR-associated protein, Csh1 family | 22.0 | 0.0001 | | | |
| SWMB_01261 CRISPR-associated protein Cas7/Csh2, subtype I-B/HMARI | 638139404 Swol_2520 CRISPR-associated protein, Csh2 family | 26.8 | 4E-30 | 646854568 Slip_1545 CRISPR-associated protein, Csd2 family | 26.1 | 0.00002 |
| SWMB_01262 CRISPR-associated protein Cas5, subtype I-B/HMARI | 638139403 Swol_2519 CRISPR-associated protein, Cas5h family | 36.5 | 8E-16 | | | |
| SWMB_01263 CRISPR-associated nuclease/helicase Cas3 | 638139402 Swol_2518 CRISPR-associated helicase, Cas3 family | 25.8 | 1E-50 | 646854571 Slip_1548 CRISPR-associated helicase, Cas3 family | 30.0 | 6E-55 |
| | | | | | | |
| SWMB_01552 CRISPR-associated endoribonuclease Cas6 | 638138692 Swol_1809 hypothetical protein, CRISPR-associated protein, Cas6 (Pfam PF01881) | 93.0 | 5E-163 | 646854360 Slip_1341 CRISPR-associated protein, Cas6 family | 28.8 | 0.17 |
| SWMB_01553 CRISPR-associated protein Csh1 | 638138691 Swol_1808 hypothetical protein, csh1 CRISPR-associated protein Csh1 | 91.5 | 0 | | | |
| SWMB_01554 CRISPR-associated protein Csh2 | 638138690 Swol_1807 hypothetical protein, CRISPR-associated protein TM1801 (Pfam PF05107) | 97.5 | 0 | | | |
| SWMB_01555 CRISPR-associated protein Cas5h | 638138689 Swol_1806 CRISPR-associated protein, Cas5h family | 94.3 | 3E-175 | | | |
| SWMB_01556 CRISPR-associated endonuclease/helicase Cas3 | 638138688 Swol_1805 CRISPR-associated helicase, Cas3 family | 93.3 | 0 | 646854571 Slip_1548 CRISPR-associated helicase, Cas3 family | 24.3 | 8E-24 |
| SWMB_01557 CRISPR-associated exonuclease Cas4 | 638138687 Swol_1804 CRISPR-associated exonuclease, Cas4 family | 98.8 | 1E-118 | 646854364 Slip_1345 CRISPR-associated protein Cas4 | 31.9 | 2E-17 |
| SWMB_01558 CRISP-associated protein Cas1 | 638138686 Swol_1803 cas1 CRISPR-associated protein, Cas1 family | 97.0 | 0 | 646854363 Slip_1344 cas1 CRISPR-associated protein Cas1 | 36.9 | 3E-62 |
| SWMB_01559 CRISPR-associated protein Cas2 | 638138685 Swol_1802 cas2 CRISPR-associated protein, Cas2 family | 96.8 | 2E-64 | 646854565 cas2 CRISPR-associated protein, Cas2 family | 32.6 | 2E-10 |
| | | | | | | |
| SWMB_01849 CRISPR-associated endoribonuclease Cas2 | 638139399 cas2 CRISPR-associated protein, Cas2 family | 74.7 | 3E-44 | 646854362 Slip_1343 cas2 CRISPR-associated protein, Cas2 family | 41.5 | 5E-23 |
| SWMB_01850 CRISPR-associated endonuclease Cas1 | 638139400 cas1 CRISPR-associated protein, Cas1 family | 74.2 | 1E-178 | 646854363 Slip_1344 cas1 CRISPR-associated protein Cas1 | 43.6 | 2E-94 |
| SWMB_01851 CRISPR-associated protein Cas4 | 638139401 Swol_2517 CRISPR-associated exonuclease, Cas4 family | 55.4 | 6E-67 | 646854364 Slip_1345 CRISPR-associated protein Cas4 | 39.3 | 1E-29 |

The gray bars indicate that there are no corresponding genes with significant similarity (<20%).

Table S3. *Syntrophomonas wolfei* subsp. *metylbutyratica* strain 4J5$^T$ genes relevant to transport systems.

| | | | BLAST top hit in *Syntrophomonas wolfei* strain Goettingen | | | BLAST top hit in *Syntrophothermus lipocalidus* strain TGB-C1 | | | |
|---|---|---|---|---|---|---|---|---|---|
| Locus Tag | Gene Product Name | TransportDB identifier | Gene ID and annotation | Identity (%) | e-Value | Gene ID and annotation | TransportDB identifier | Identity (%) | e-Value |
| Tungstate | | | | | | | | | |
| SWMB_02195 | tupA, vupA; tungstate transport system substrate-binding protein | ABC(binding protein)\|3.A.1 | 638138507 Swol_1630 ABC-type tungstate transport system permease component-like protein | 97.8 | 0 | 646854788 Slip_1758 extracellular solute-binding protein family 1 | ABC(binding protein)\|3.A.1 | 59.2 | 8E-107 |
| SWMB_02196 | tupC, vupC; tungstate transport system ATP-binding protein | ABC(ABC)\|3.A.1 | 638138508 Swol_1631 phosphate ABC transporter ATP-binding protein | 95.5 | 9E-167 | 646854789 Slip_1759 ABC transporter related protein | ABC(ABC)\|3.A.1 | 63.0 | 1E-102 |
| SWMB_02197 | tupB, vupB; tungstate transport system permease protein | ABC(membrane)\|3.A.1 | 638138509 Swol_1632 sulfate/tungstate uptake family ABC transporter, permease protein | 98.7 | 4E-155 | 646854790 Slip_1760 binding-protein-dependent transport systems inner membrane component | ABC(membrane)\|3.A.1 | 66.4 | 2E-104 |
| Molybdate | | | | | | | | | |
| SWMB_00891 | modA; molybdate transport system substrate-binding protein | ABC(binding protein)\|3.A.1 | 638138436 Swol_1556 ABC-type molybdate transport system periplasmic component-like protein | 24.8 | 3E-12 | | | | |
| SWMB_00892 | modB; molybdate transport system permease protein | ABC(membrane)\|3.A.1 | 638138435 Swol_1555 molybdenum ABC transporter, permease protein | 33.3 | 6E-34 | | | | |
| SWMB_00893 | wtpC; molybdate/tungstate transport system ATP-binding protein | ABC(ABC)\|3.A.1 | 638138434 Swol_1554 molybdenum ABC transporter, ATP-binding protein | 36.1 | 5.00E-43 | | | | |
| SWMB_02016 | putative molybdopterin biosynthesis protein MoeA/LysR substrate binding protein | – | 638138437 Swol_1557 molybdate-binding protein | 92.1 | 0 | | | | |
| SWMB_02017 | Molybdate-binding periplasmic protein precursor | ABC(binding protein)\|3.A.1 | 638138436 Swol_1556 ABC-type molybdate transport system periplasmic component-like protein | 92.4 | 0 | 646855146 Slip_2123 molybdenum ABC transporter, periplasmic molybdate-binding protein | ABC(binding protein)\|3.A.1 | 50.7 | 1E-67 |
| SWMB_02018 | Molybdenum transport system permease protein ModB | ABC(membrane)\|3.A.1 | 638138435 Swol_1555 molybdenum ABC transporter, permease protein | 96.7 | 2E-168 | 646855145 Slip_2122 molybdate ABC transporter, inner membrane subunit | ABC(membrane)\|3.A.1 | 54.3 | 2E-63 |
| SWMB_02019 | Sulfate/thiosulfate import ATP-binding protein CysA | ABC(ABC)\|3.A.1 | 638138434 Swol_1554 molybdenum ABC transporter, ATP-binding protein | 93.8 | 0 | 646855144 Slip_2121 ABC transporter related protein | ABC(ABC)\|3.A.1 | 44.6 | 2E-55 |
| Iron | | | | | | | | | |
| SWMB_02242 | ABC.FEV.S; iron complex transport system substrate-binding protein | ABC(binding protein)\|3.A.1 | 638138550 Swol_1673 ferrichrome-binding periplasmic protein | 97.5 | 0 | | | | |
| SWMB_02243 | bioB; biotin synthase | – | 638138551 Swol_1674 hypothetical protein | 96.8 | 0 | | | | |
| SWMB_02244 | ABC.FEV.S; iron complex transport system substrate-binding protein | ABC(binding protein)\|3.A.1 | 638138552 Swol_1675 hypothetical protein | 96.5 | 0 | | | | |
| SWMB_02245 | ABC.FEV.S; iron complex transport system substrate-binding protein | ABC(binding protein)\|3.A.1 | 638138556 Swol_1679 periplasmic binding protein | 69.7 | 0 | | | | |
| SWMB_02246 | ABC.FEV.S; iron complex transport system substrate-binding protein | ABC(binding protein)\|3.A.1 | 638138550 Swol_1673 ferrichrome-binding periplasmic protein | 34.4 | 4E-54 | | | | |
| SWMB_02247 | ABC.FEV.A; iron complex transport system ATP-binding protein | ABC(ABC)\|3.A.1 | | | | | | | |
| SWMB_02248 | ABC.FEV.P; iron complex transport system permease protein | ABC(membrane)\|3.A.1 | 638138553 Swol_1676 transport system permease protein | 95.7 | 0 | | | | |
| SWMB_02249 | bioB; biotin synthase | – | 638138551 Swol_1674 hypothetical protein | 31.8 | 3E-61 | | | | |
| SWMB_02145 | Ferric uptake regulation protein | GBP\|1.B.1 | 638137537 Swol_0660 Fe2+/Zn2+ uptake regulation proteins | 93.8 | 3E-92 | 646854586 Slip_1558 ferric uptake regulator, Fur family | – | 52.9 | 2E-50 |
| SWMB_02146 | feoB; ferrous iron transport protein B | FeoB\|9.A.8 | 638137538 Swol_0661 Fe2+ transport system protein B-like protein | 93.8 | 0 | 646854585 Slip_1557 small GTP-binding protein | FeoB\|9.A.8 | 46.9 | 0 |
| SWMB_02147 | feoA; ferrous iron transport protein A | FeoB\|9.A.8 | 638137539 Swol_0662 putative Fe2+ transport protein | 96.2 | 1E-50 | 646854584 Slip_1556 FeoA family protein | Q7MV19_PORGI | 38.0 | 4E-14 |
| SWMB_02148 | antiporter inner membrane protein | ABC(membrane)\|3.A.1 | 638137540 Swol_0663 ATPases involved in chromosome partitioning-like protein | 96.3 | 0 | | | | |
| SWMB_02149 | putative ABC transporter ATP-binding protein | ABC(ABC)\|3.A.1 | 638137541 Swol_0664 ABC transporter ATP-binding protein | 98.4 | 0 | 646854583 Slip_1555 ABC transporter related protein | ABC(ABC)\|3.A.1 | 62.8 | 2E-115 |
| SWMB_02150 | sufC; Fe-S cluster assembly ATP-binding protein | ABC(membrane)\|3.A.1 | 638137542 Swol_0665 hypothetical protein | 96.1 | 0 | 646854582 Slip_1554 SufBD protein | ABC(membrane)\|3.A.1 | 61.9 | 9E-137 |
| SWMB_02282 | Ferrous iron transport protein B | FeoB\|9.A.8 | 638138419 Swol_1539 nucleoside recognition proteinn | 94.8 | 0 | | | | |
| SWMB_02283 | Ferrous iron transport protein B | FeoB\|9.A.8 | 638138418 Swol_1538 GTP-binding protein, HSR1-related | 95.4 | 0 | | | | |
| SWMB_02284 | FeoA domain protein | ABC(ABC)\|3.A.1 | 638138417 Swol_1537 FeoA | 79.5 | 1E-31 | | | | |
| SWMB_00434 | Phosphoglycerate transport regulatory protein PgtC precursor | ABC(binding protein)\|3.A.1 | 638139364 Swol_2479 ABC transporter, periplasmic substrate-binding protein | 98.9 | 0 | | | | |
| SWMB_00435 | sn-glycerol-3-phosphate import ATP-binding protein UgpC | ABC(ABC)\|3.A.1 | 638139363 Swol_2478 thiamine transport system ATP-binding protein | 99.4 | 0 | | | | |
| SWMB_00436 | Molybdenum transport system permease protein ModB | ABC(membrane)\|3.A.1 | 638139362 Swol_2477 ABC-type transporter permease protein | 99.8 | 0 | | | | |
| SWMB_00437 | Phosphoglycerate transport regulatory protein PgtC precursor | ABC(binding protein)\|3.A.1 | 638139361 Swol_2476 ABC-type iron(III)-binding periplasmic protein precursor | 97.5 | 0 | | | | |
| SWMB_01856 | putative iron(III)\|ABC-type Fe3+ transport system periplasmic component-like protein | ABC(binding protein)\|3.A.1 | 638138562 Swol_1685 ABC-type Fe3+ transport system periplasmic component-like protein | 94.4 | 0 | | | | |
| SWMB_02863 | iron complex transport system permease protein | ABC(membrane)\|3.A.1 | | | | 646854481 Slip_1459 transport system permease protein | ABC(membrane)\|3.A.1 | 40.1 | 8E-60 |
| SWMB_02864 | iron complex transport system substrate-binding protein | ABC(binding protein)\|3.A.1 | | | | 646854482 Slip_1460 periplasmic binding protein | ABC(binding protein)\|3.A.1 | 24.9 | 1E-18 |
| SWMB_02865 | iron complex transport system ATP-binding protein | ABC(ABC)\|3.A.1 | | | | 646854480 Slip_1458 ABC transporter related protein | ABC(ABC)\|3.A.1 | 35.8 | 8E-49 |
| SWMB_01927 | Ferrous iron transport protein B | FeoB\|9.A.8 | | | | | | | |
| SWMB_01928 | ferrous iron transport protein A | FeoB\|9.A.8 | | | | | | | |
| SWMB_03066 | ferrous iron transport protein B | FeoB\|9.A.8 | | | | | | | |
| Zinc | | | | | | | | | |
| SWMB_02367 | TC.BAT2; bacterial/archaeal transporter family-2 protein | Q6M0K5_METMP | 638138013 Swol_1140 protein of unknown function DUF606 | 95.9 | 6E-97 | 646854244 Slip_1216 protein of unknown function DUF606 | Q6M0K5_METMP | 60.1 | 1E-55 |
| SWMB_02368 | TC.ZIP, zupT, ZRT3, ZIP2; zinc transporter, ZIP family | ZIP\|2.A.5 | 638138012 Swol_1139 hypothetical protein | 96.2 | 6E-144 | 646854245 Slip_1217 zinc/iron permease | ZIP\|2.A.5 | 47.8 | 3E-58 |
| SWMB_02926 | High-affinity zinc uptake system membrane protein ZnuB | ABC(membrane)\|3.A.1 | 638138772 Swol_1889 ABC 3 transport family protein | 96.2 | 1E-173 | 646854777 Slip_1747 ABC-3 protein | ABC(membrane)\|3.A.1 | 33.3 | 6E-42 |
| SWMB_02927 | putative zinc transport system ATP-binding protein AdcC | ABC(ABC)\|3.A.1 | 638138773 Swol_1890 ABC-type zinc transport system ATP-binding protein | 95.4 | 0 | 646854776 Slip_1746 ABC transporter related protein | ABC(ABC)\|3.A.1 | 36.3 | 1E-39 |
| SWMB_02928 | putative zinc transport system zinc-binding lipoprotein AdcA precursor | ABC(binding protein)\|3.A.1 | 638138774 Swol_1891 cation ABC transporter, periplasmc-binding protein | 95.1 | 0 | 646854775 Slip_1745 periplasmic solute binding protein | ABC(binding protein)\|3.A.1 | 33.8 | 1E-48 |
| Cobalt/nickel/magnesium | | | | | | | | | |
| SWMB_00627 | ecfA1; energy-coupling factor transport system ATP-binding protein [EC:3.6.3.-] | ABC(ABC)\|3.A.1 | 638139183 Swol_2302 ecfA putative ABC transporter | 94.1 | 2E-180 | 646855228 Slip_2206 ABC transporter related protein | ABC(ABC)\|3.A.1 | 42.1 | 9E-79 |
| SWMB_00628 | ecfA2; energy-coupling factor transport system ATP-binding protein [EC:3.6.3.-] | ABC(ABC)\|3.A.1 | 638139182 Swol_2301 putative ABC transporter | 91.6 | 0 | 646855227 Slip_2205 ABC transporter related protein | ABC(ABC)\|3.A.1 | 41.3 | 1E-61 |
| SWMB_00629 | ecfT; energy-coupling factor transport system permease protein | ABC(membrane)\|3.A.1 | 638139181 Swol_2300 cobalt ABC transporter permease protein | 92.0 | 6E-151 | 646855226 Slip_2204 cobalt transport protein | ABC(membrane)\|3.A.1 | 31.6 | 5E-41 |
| SWMB_01248 | ecfT; energy-coupling factor transport system permease protein | ABC(membrane)\|3.A.1 | 638137916 Swol_1045 cobalt transport protein | 90.3 | 0 | 646853359 Slip_0329 cobalt transport protein | ABC(membrane)\|3.A.1 | 45.1 | 7E-82 |
| SWMB_01249 | ecfA1; energy-coupling factor transport system ATP-binding protein [EC:3.6.3.-] | ABC(ABC)\|3.A.1 | 638137917 Swol_1046 ABC transporter related | 90.2 | 0 | 646853360 Slip_0330 ABC transporter related protein | ABC(ABC)\|3.A.1 | 54.0 | 0 |
| SWMB_01282 | putative Mg(2+) transport ATPase | P-ATPase\|3.A.3 | 638137783 Swol_0917 cation transport ATPase | 94.2 | 2E-102 | 646854093 Slip_1061 MgtC/SapB transporter | P-ATPase\|3.A.3 | 40.9 | 9E-28 |
| SWMB_01480 | ecfA1; energy-coupling factor transport system ATP-binding protein [EC:3.6.3.-] | ABC(ABC)\|3.A.1 | 638138835 Swol_1952 ABC transporter, ATP-binding protein | 96.3 | 5E-175 | | | | |
| SWMB_01481 | ecfA2; energy-coupling factor transport system ATP-binding protein [EC:3.6.3.-] | ABC(ABC)\|3.A.1 | 638138834 Swol_1951 ABC-type cobalt transport system, ATPase component | 96.9 | 2E-163 | | | | |
| SWMB_01482 | ecfT; energy-coupling factor transport system permease protein | ABC(membrane)\|3.A.1 | 638138833 Swol_1950 ABC-type cobalt transport system permease component CbiQ | 98.3 | 2E-168 | | | | |
| SWMB_01483 | ecfA1; energy-coupling factor transport system ATP-binding protein [EC:3.6.3.-] | ABC(ABC)\|3.A.1 | 638138832 Swol_1949 ABC-type cobalt transport system, ATPase component | 98.2 | 0 | 646853254 Slip_0220 ABC transporter related protein | ABC(ABC)\|3.A.1 | 50.9 | 2E-95 |
| SWMB_01484 | ecfA2; energy-coupling factor transport system ATP-binding protein [EC:3.6.3.-] | ABC(ABC)\|3.A.1 | 638138831 Swol_1948 ABC-type transporter, ATPase component | 95.5 | 0 | 646853253 Slip_0219 ABC transporter related protein | ABC(ABC)\|3.A.1 | 43.9 | 3E-84 |
| SWMB_01485 | ecfT; energy-coupling factor transport system permease protein | ABC(ABC)\|3.A.1 | 638138830 Swol_1947 cobalt ABC transporter permease protein | 96.9 | 0 | 646853255 Slip_0221 cobalt transport protein | ABC(membrane)\|3.A.1 | 39.9 | 5E-61 |
| SWMB_01799 | Magnesium transporter MgtE | MgtE\|9.A.19 | 638137951 Swol_1082 divalent cation transporter | 98.2 | 0 | | | | |
| SWMB_02015 | Magnesium transport protein CorA | MIT\|1.A.35 | | | | 646854392 corA magnesium and cobalt transport protein CorA | MIT\|1.A.35 | 63.3 | 4E-153 |
| SWMB_01835 | cbiO; cobalt/nickel transport system ATP-binding protein | ABC(ABC)\|3.A.1 | | | | 646853404 Slip_0374 ABC transporter related protein | ABC(ABC)\|3.A.1 | 64.5 | 4E-123 |
| SWMB_01836 | cbiQ; cobalt/nickel transport system permease protein | ABC(membrane)\|3.A.1 | | | | 646853405 Slip_0375 cobalt ABC transporter, inner membrane subunit CbiQ | ABC(membrane)\|3.A.1 | 42.6 | 5E-68 |
| SWMB_01837 | cbiN; cobalt/nickel transport protein | Nickel permease | | | | 646853406 Slip_0376 hypothetical protein | Nickel permease | 58.8 | 7E-39 |
| SWMB_01838 | cbiM; cobalt/nickel transport system permease protein | Nickel permease | | | | 646853407 Slip_0377 cobalamin (vitamin B12) biosynthesis CbiM protein | Nickel permease | 65.2 | 5E-102 |
| | | | | | | 646853961 Slip_0929 cobalt ABC transporter, ATPase subunit | ABC(ABC)\|3.A.1 | | |
| | | | | | | 646853962 Slip_0930 cobalt ABC transporter, inner membrane subunit CbiQ | ABC(membrane)\|3.A.1 | | |
| | | | | | | 646853963 Slip_0931 cobalt transport protein | ABC(binding protein)\|3.A.1 | | |
| | | | | | | 646854029 Slip_0997 MgtC/SapB transporter | P-ATPase\|3.A.3 | | |
| Phosphate | | | | | | | | | |
| SWMB_00438 | Phosphate transport system permease protein PstC | ABC(membrane)\|3.A.1 | | | | | | | |
| SWMB_01279 | Putative PhoU protein | F-ATPase\|3.A.2 | 638137786 Swol_0920 hypothetical protein | 98.1 | 1E-150 | 646854101 Slip_1070 Putitive phosphate transport regulator | IISP\|3.A.5 | 61.1 | 2E-95 |
| SWMB_02212 | Low-affinity inorganic phosphate transporter 1 | PiT\|2.A.20 | 638138523 Swol_1646 phosphate transporter | 97.6 | 0 | 646853713 Slip_0683 phosphate transporter | PiT\|2.A.20 | 70.4 | 8E-157 |
| | | | | | | 646853712 Slip_0682 Putitive phosphate transport regulator | ABC(membrane)\|3.A.1 | | |
| SWMB_00754 | pstS; phosphate transport system substrate-binding protein | ABC(binding protein)\|3.A.1 | | | | | | | |
| SWMB_00755 | pstC; phosphate transport system permease protein | ABC(membrane)\|3.A.1 | | | | | | | |
| SWMB_00756 | pstA; phosphate transport system permease protein | ABC(membrane)\|3.A.1 | | | | | | | |
| SWMB_00757 | pstB; phosphate transport system ATP-binding protein [EC:3.6.3.27] | ABC(ABC)\|3.A.1 | | | | | | | |
| SWMB_01762 | phoU; phosphate transport system protein | PNaS\|2.A.58 | | | | 646853402 Slip_0372 phosphate uptake regulator, PhoU | PNaS\|2.A.58 | 56.5 | 1E-86 |
| SWMB_01763 | pstB; phosphate transport system ATP-binding protein [EC:3.6.3.27] | ABC(ABC)\|3.A.1 | | | | 646853401 Slip_0371 pstB phosphate ABC transporter ATP-binding protein | ABC(ABC)\|3.A.1 | 71.9 | 3E-134 |
| SWMB_01764 | pstA; phosphate transport system permease protein | ABC(membrane)\|3.A.1 | | | | 646853400 Slip_0370 phosphate ABC transporter membrane protein 2 | ABC(membrane)\|3.A.1 | 58.2 | 2E-87 |
| SWMB_01765 | pstC; phosphate transport system permease protein | ABC(membrane)\|3.A.1 | | | | 646853399 Slip_0369 phosphate ABC transporter membrane protein 1 | ABC(membrane)\|3.A.1 | 65.7 | 9E-122 |
| SWMB_01766 | pstS; phosphate transport system substrate-binding protein | ABC(binding protein)\|3.A.1 | | | | 646853395 Slip_0365 phosphate ABC transporter substrate-binding protein | ABC(binding protein)\|3.A.1 | 55.9 | 2E-107 |
| SWMB_01767 | phoR; two-component system | SSS\|2.A.21 | | | | 646853398 Slip_0368 multi-sensor signal transduction histidine kinase | SSS\|2.A.21 | 45.2 | 4E-158 |
| SWMB_01768 | phoB1, phoP; two-component system | ABC(membrane)\|3.A.1 | | | | 646853397 Slip_0367 two component transcriptional regulator | ABC(membrane)\|3.A.1 | 57.9 | 6E-87 |
| Calcium/sodium | | | | | | | | | |
| SWMB_02139 | putative calcium/sodium:proton antiporter | CaCA\|2.A.19 | 638137531 Swol_0654 sodium/calcium exchanger membrane region | 97.9 | 0 | 646854607 Slip_1580 sodium/calcium exchanger membrane region | CaCA\|2.A.19 | 63.8 | 2E-141 |
| SWMB_02947 | Sodium:dicarboxylate symporter family | DAACS\|2.A.23 | 638137199 Swol_0311 Na+/H+-dicarboxylate symporters-like protein | 94.1 | 0 | | | | |
| Potassium | | | | | | | | | |
| SWMB_01981 | potassium/proton antiporter | CPA1\|2.A.36 | 638138469 Swol_1590 potassium/proton antiporter, CPA1 family (TC 2A36) | 95.5 | 0 | | | | |
| SWMB_01919 | Ktr system potassium uptake protein B | Trk\|2.A.38 | 638137779 Swol_0913 K+ transporter trk | 24.9 | 7E-07 | 646854516 Slip_1494 potassium uptake protein, TrkH family | Trk\|2.A.38 | 45.8 | 3E-121 |
| SWMB_01920 | Ktr system potassium uptake protein A | VIC\|1.A.1, Trk\|2.A.38 | 638137780 Swol_0914 hypothetical protein | 23.1 | 1E-07 | 646854515 Slip_1493 TrkA-N domain protein | VIC\|1.A.1 | 47.0 | 2E-70 |
| SWMB_01285 | Trk system potassium uptake protein TrkA | VIC\|1.A.1 | 638137780 Swol_0914 hypothetical protein | 96.7 | 0 | | | | |
| SWMB_01286 | Trk system potassium uptake protein TrkG | Trk\|2.A.38 | 638137779 Swol_0913 K+ transporter trk | 97.9 | 0 | | | | |
| SWMB_02711 | Inner membrane protein YbaL | CPA2\|2.A.37 | 638138051 Swol_1178 Kef-type potassium/proton antiporter | 96.3 | 0 | 646854137 Slip_1106 Kef-type potassium/proton antiporter | CPA2\|2.A.37 | 53.9 | 0 |
| Nitrate/sulfonate/taurine | | | | | | | | | |
| SWMB_02887 | Putative aliphatic sulfonates-binding protein precursor | ABC(binding protein)\|3.A.1 | 638137227 Swol_0341 putative sulfonate transport system substrate-binding protein | 95.4 | 0 | 646854054 Slip_1022 NMT1/THI5 like domain protein | ABC(binding protein)\|3.A.1 | 30.3 | 7E-45 |
| SWMB_02888 | Putative aliphatic sulfonates transport permease protein SsuC | ABC(membrane)\|3.A.1 | 638137226 Swol_0340 putative sulfonate transport system permease protein | 97.2 | 3E-168 | 646854053 Slip_1021 binding-protein-dependent transport systems inner membrane component | ABC(membrane)\|3.A.1 | 28.5 | 2E-29 |
| SWMB_02889 | Aliphatic sulfonates import ATP-binding protein SsuB | ABC(ABC)\|3.A.1 | 638137225 Swol_0339 putative sulfonate transport system ATP-binding protein | 94.8 | 4E-156 | 646854052 Slip_1020 ABC transporter related protein | ABC(ABC)\|3.A.1 | 39.1 | 3.00E-44 |
| SWMB_00497 | Taurine import ATP-binding protein TauB | ABC(ABC)\|3.A.1 | | | | 646854349 Slip_1326 ABC transporter related protein | ABC(ABC)\|3.A.1 | 48.4 | 1E-83 |
| SWMB_00498 | Putative aliphatic sulfonates transport permease protein SsuC | ABC(membrane)\|3.A.1 | | | | 646854350 Slip_1327 binding-protein-dependent transport systems inner membrane component | ABC(membrane)\|3.A.1 | 30.0 | 7E-32 |
| SWMB_00499 | ABC transporter, substrate-binding protein, aliphatic sulfonates family | ABC(binding protein)\|3.A.1 | | | | 646854351 Slip_1328 aliphatic sulfonates family ABC transporter, periplasmic ligand-binding protein | ABC(binding protein)\|3.A.1 | 25.0 | 0.001 |
| Ammonium | | | | | | | | | |
| | | | | | | 646854598 Slip_1571 ammonium transporter | Amt\|1.A.11 | | |
| | | | | | | 646855142 Slip_2119 ammonium transporter | Amt\|1.A.11 | | |
| Glycine betaine/proline | | | | | | | | | |
| | | | | | | 646853664 Slip_0633 Substrate-binding region of ABC-type glycine betaine transport system | ABC(membrane)\|3.A.1 | | |
| SWMB_01908 | proW; glycine betaine/proline transport system permease protein | ABC(membrane)\|3.A.1 | 638138613 Swol_1736 binding-protein-dependent transport systems inner membrane component | 97.9 | 0 | 646853665 Slip_0634 binding-protein-dependent transport systems inner membrane component | ABC(membrane)\|3.A.1 | 36.4 | 6E-25 |
| | | | | | | 646853666 Slip_0635 binding-protein-dependent transport systems inner membrane component | ABC(membrane)\|3.A.1 | | |
| SWMB_01909 | proV; glycine betaine/proline transport system ATP-binding protein | ABC(ABC)\|3.A.1 | 638138614 Swol_1737 putative glycine betaine ABC transporter ATP-binding protein | 96.6 | 0 | 646853667 Slip_0636 glycine betaine/L-proline ABC transporter, ATPase subunit | ABC(ABC)\|3.A.1 | 44.2 | 5E-60 |
| SWMB_01910 | proX; glycine betaine/proline transport system substrate-binding protein | ABC(binding protein)\|3.A.1 | 638138615 Swol_1738 ABC transporter substrate-binding protein | 96.0 | 0 | 646853668 Slip_0638 hypothetical protein | ABC(binding protein)\|3.A.1 | 43.1 | 0.000001 |
| Amino acids | | | | | | | | | |
| SWMB_00394 | livK; branched-chain amino acid transport system substrate-binding protein | ABC(binding protein)\|3.A.1 | 638139440 Swol_2556 amino acid/amide ABC transporter substrate-binding protein | 96.9 | 0 | 646855387 Slip_2362 amino acid/amide ABC transporter substrate-binding protein | ABC(binding protein)\|3.A.1 | 56.1 | 4E-144 |
| SWMB_00395 | livH; branched-chain amino acid transport system permease protein | ABC(membrane)\|3.A.1 | 638139439 Swol_2555 amino acid/amide ABC transporter membrane protein 1 | 99.0 | 0 | 646855386 Slip_2361 amino acid/amide ABC transporter membrane protein 1 | ABC(membrane)\|3.A.1 | 74.9 | 1E-155 |
| SWMB_00396 | livM; branched-chain amino acid transport system permease protein | ABC(membrane)\|3.A.1 | 638139438 Swol_2554 amino acid/amide ABC transporter membrane protein 2 | 98.8 | 0 | 646855385 Slip_2360 amino acid/amide ABC transporter membrane protein 2 | ABC(membrane)\|3.A.1 | 63.1 | 3E-143 |
| SWMB_00397 | livG; branched-chain amino acid transport system ATP-binding protein | ABC(ABC)\|3.A.1 | 638139437 Swol_2553 amino acid/amide ABC transporter ATP-binding protein 1 | 99.2 | 8E-173 | 646855384 Slip_2359 amino acid/amide ABC transporter ATP-binding protein 1 | ABC(ABC)\|3.A.1 | 67.0 | 4E-110 |
| SWMB_00398 | livF; branched-chain amino acid transport system ATP-binding protein | ABC(ABC)\|3.A.1 | 638139436 Swol_2552 amino acid/amide ABC transporter ATP-binding protein 2 | 97.9 | 2E-163 | 646855383 Slip_2358 amino acid/amide ABC transporter ATP-binding protein 2 | ABC(ABC)\|3.A.1 | 76.9 | 1E-132 |
| SWMB_01054 | livK; branched-chain amino acid transport system substrate-binding protein | ABC(binding protein)\|3.A.1 | 638138994 Swol_2113 amino acid/amide ABC transporter substrate-binding protein | 96.5 | 0 | 646853698 Slip_0668 amino acid/amide ABC transporter substrate-binding protein | ABC(binding protein)\|3.A.1 | 77.2 | 0 |
| SWMB_01055 | livH; branched-chain amino acid transport system permease protein | ABC(membrane)\|3.A.1 | 638138993 Swol_2112 amino acid/amide ABC transporter membrane protein 1 | 99.3 | 0 | 646853699 Slip_0669 amino acid/amide ABC transporter membrane protein 1 | ABC(membrane)\|3.A.1 | 76.3 | 2E-156 |
| SWMB_01056 | livM; branched-chain amino acid transport system permease protein | ABC(membrane)\|3.A.1 | 638138992 Swol_2111 amino acid/amide ABC transporter membrane protein 2 | 98.3 | 0 | 646853700 Slip_0670 amino acid/amide ABC transporter membrane protein 2 | ABC(membrane)\|3.A.1 | 72.4 | 6E-154 |
| SWMB_01057 | livG; branched-chain amino acid transport system ATP-binding protein | ABC(ABC)\|3.A.1 | 638138991 Swol_2110 amino acid/amide ABC transporter ATP-binding protein 1 | 98.0 | 1E-180 | 646853701 Slip_0671 amino acid/amide ABC transporter ATP-binding protein 1 | ABC(ABC)\|3.A.1 | 64.8 | 2E-113 |
| SWMB_01058 | carboxylate/amino acid/amine transporter | DMT\|2.A.7 | 638138990 Swol_2109 hypothetical protein | 96.9 | 0 | 646853702 Slip_0672 protein of unknown function DUF6 transmembrane | DMT\|2.A.7 | 58.5 | 2E-105 |
| SWMB_00992 | Inner membrane protein YbiR | DASS\|2.A.47 | 638139046 Swol_2166 possible tyrosine transporter P-protein (TC 2A4521) | 97.6 | 0 | 646853277 Slip_0246 possible tyrosine transporter P-protein | DASS\|2.A.47 | 64.8 | 0 |
| SWMB_00489 | metQ; D-methionine transport system substrate-binding protein | ABC(binding protein)\|3.A.1 | 638139316 Swol_2432 hypothetical protein | 93.2 | 0 | | | | |
| SWMB_00490 | metI; D-methionine transport system permease protein | ABC(membrane)\|3.A.1 | 638139315 Swol_2431 ABC metal ion transporter, inner membrane subunit | 99.2 | 9E-167 | | | | |
| SWMB_00491 | metN; D-methionine transport system ATP-binding protein | ABC(ABC)\|3.A.1 | 638139314 Swol_2430 metN hypothetical protein | 96.5 | 0 | | | | |
| SWMB_02034 | Inner membrane amino-acid ABC transporter permease protein YecS | ABC(membrane)\|3.A.1 | 638138616 Swol_1739 amino acid ABC transporter membrane protein | 52.3 | 1E-56 | | | | |
| SWMB_02035 | Arginine transport ATP-binding protein ArtM | ABC(ABC)\|3.A.1 | 638138617 Swol_1740 amino acid ABC transporter ATP-binding protein | 63.2 | 2E-104 | | | | |
| SWMB_02489 | Glutamine transport ATP-binding protein GlnQ | ABC(ABC)\|3.A.1 | 638137941 Swol_1072 amino acid ABC transporter ATP-binding protein | 48.5 | 8E-79 | | | | |
| SWMB_02490 | Inner membrane amino-acid ABC transporter permease protein YecS | ABC(membrane)\|3.A.1 | 638137940 Swol_1071 amino acid ABC transporter membrane protein | 42.7 | 9E-44 | | | | |
| SWMB_02491 | ABC transporter arginine-binding protein 1 precursor | ABC(binding protein)\|3.A.1 | 638137939 Swol_1070 amino acid ABC transporter substrate-binding protein | 31.5 | 5E-30 | | | | |
| SWMB_02950 | Glutamine transport ATP-binding protein GlnQ | ABC(ABC)\|3.A.1 | 638137202 Swol_0314 amino acid ABC transporter ATP-binding protein | 96.9 | 0 | | | | |
| SWMB_02951 | Arginine transport system permease protein ArtQ | ABC(membrane)\|3.A.1 | 638137203 Swol_0315 amino acid ABC transporter membrane protein | 98.6 | 7E-152 | | | | |
| SWMB_02952 | Polar amino acids-binding domain of ATP-binding cassette transporter-like systems | ABC(binding protein)\|3.A.1 | 638137204 Swol_0316 amino acid ABC transporter substrate-binding protein | 96.1 | 7E-180 | | | | |
| SWMB_01844 | Glutamine transport ATP-binding protein GlnQ | ABC(ABC)\|3.A.1 | 638137928 Swol_1059 amino acid ABC transporter ATP-binding protein | 97.6 | 7E-178 | | | | |
| SWMB_01845 | Inner membrane amino-acid ABC transporter permease protein YecS | ABC(membrane)\|3.A.1 | | | | | | | |
| SWMB_01846 | Arginine-binding extracellular protein ArtP precursor | ABC(membrane)\|3.A.1 | 639331168 Swol_1057 amino acid ABC transporter substrate-binding protein | 98.2 | 0 | | | | |
| SWMB_01078 | Inner membrane protein YbiR | DASS\|2.A.47 | 638138972 Swol_2091 possible tyrosine transporter P-protein | 96.9 | 0 | | | | |
| | | | | | | 646853655 Slip_0624 Lysine exporter protein (LYSE/YGGA) | RhtB\|2.A.76 | | |
| | | | | | | 648021225 Slip_0690 amino acid ABC transporter ATP-binding protein | ABC(ABC)\|3.A.1 | | |
| | | | | | | 648021263 Slip_2115 amino acid/polyamine/organocation transporter | APC\|2.A.3 | | |
| Monosaccharide | | | | | | | | | |
| SWMB_01710 | Ribose transport system permease protein RbsC | ABC(membrane)\|3.A.1 | 638137304 Swol_0416 monosaccharide ABC transporter membrane protein | 97.0 | 0 | | | | |
| SWMB_01711 | ribose ABC transporter permease protein | ABC(membrane)\|3.A.1 | 638137303 Swol_0415 monosaccharide ABC transporter membrane protein | 96.5 | 0 | | | | |
| SWMB_01712 | Xylose import ATP-binding protein XylG | ABC(ABC)\|3.A.1 | 638137302 Swol_0414 monosaccharide ABC transporter ATP-binding protein | 94.7 | 0 | | | | |
| | | | 638137311 Swol_0423 monosaccharide ABC transporter substrate-binding protein | | | 646853352 Slip_0322 monosaccharide ABC transporter substrate-binding protein | ABC(binding protein)\|3.A.1 | 22.8 | 5E-10 |
| | | | 638137314 Swol_0426 monosaccharide ABC transporter ATP-binding protein | | | 646853354 Slip_0324 monosaccharide ABC transporter ATP-binding protein | ABC(ABC)\|3.A.1 | 34.7 | 3E-44 |
| | | | 638137313 Swol_0425 monosaccharide ABC transporter membrane protein | | | 646853355 Slip_0325 monosaccharide ABC transporter membrane protein | ABC(membrane)\|3.A.1 | 30.5 | 2E-32 |
| | | | 638137312 Swol_0424 monosaccharide ABC transporter membrane protein | | | | | | |
| Biotin | | | | | | | | | |
| SWMB_00255 | bioY; biotin transport system substrate-specific component | – | 638137009 Swol_0108 hypothetical protein | 99.5 | 4E-137 | 646853186 Slip_0142 BioY protein | BUT\|2.A.88 | 50.0 | 1E-52 |
| Cell division | | | | | | | | | |
| SWMB_00138 | ftsX; cell division transport system permease protein | ABC(membrane)\|3.A.1 | 638137142 Swol_0246 cell division protein FtsX | 95.9 | 0 | 646854899 Slip_1871 cell division protein FtsX | ABC(membrane)\|3.A.1 | 54.7 | 5E-109 |
| SWMB_00139 | ftsE; cell division transport system ATP-binding protein | ABC(ABC)\|3.A.1 | 638137141 Swol_0245 cell division ATP-binding protein FtsE | 97.4 | 7E-159 | 646854900 Slip_1872 cell division ATP-binding protein FtsE | ABC(ABC)\|3.A.1 | 69.6 | 4E-113 |
| Secretion system | | | | | | | | | |
| SWMB_00705 | secD; preprotein translocase subunit SecD | RND\|2.A.6 | 638138305 secD protein-export membrane protein SecD | 98.0 | 0 | 646853784 secD protein-export membrane protein SecD | RND\|2.A.6 | 63.9 | 3E-179 |
| SWMB_00706 | secF; preprotein translocase subunit SecF | RND\|2.A.6 | 638138304 secF protein translocase subunit secF | 95.4 | 0 | 646853785 secF protein translocase subunit secF | RND\|2.A.6 | 55.7 | 1E-97 |
| SWMB_00581 | secE; preprotein translocase subunit SecE | IISP\|3.A.5 | 638139229 secE protein translocase subunit secE/sec61 gamma | 97.5 | 6E-52 | 646855274 secE preprotein translocase, SecE subunit | IISP\|3.A.5 | 60.7 | 2E-20 |
| SWMB_00109 | secG; preprotein translocase subunit SecG | IISP\|3.A.5 | 638137170 Swol_0277 preprotein translocase, SecG subunit | 96.1 | 3E-45 | 646854875 Slip_1846 preprotein translocase, SecG subunit | IISP\|3.A.5 | 65.8 | 3E-26 |
| SWMB_00617 | secY; preprotein translocase subunit SecY | IISP\|3.A.5 | 638139194 secY protein translocase subunit secY/sec61 alpha | 98.1 | 0 | 646855239 secY protein translocase subunit secY/sec61 alpha | IISP\|3.A.5 | 77.0 | 0 |
| SWMB_00703 | yajC; preprotein translocase subunit YajC | IISP\|3.A.5 | 638138307 Swol_1427 protein translocase subunit yajC | 91.6 | 1E-42 | 646853782 Slip_0750 protein translocase subunit yajC | IISP\|3.A.5 | 52.4 | 3E-27 |
| SWMB_00376 | yidC, spoIIIJ, OXA1; YidC/Oxa1 family membrane protein insertase | Oxa1\|2.A.9 | 639331191 Swol_2574 protein translocase subunit yidC | 98.1 | 6E-156 | 646855407 Slip_2382 protein translocase subunit yidC | Oxa1\|2.A.9 | 54.2 | 9E-95 |
| SWMB_00145 | secA; preprotein translocase subunit SecA | IISP\|3.A.5 | 638137135 secA protein translocase subunit secA | 97.1 | 0 | 646854906 secA protein translocase subunit secA | IISP\|3.A.5 | 74.5 | 0 |
| SWMB_02811 | ftsY; fused signal recognition particle receptor | IISP\|3.A.5 | 638137648 ftsY signal recognition particle-docking protein FtsY | 97.1 | 0 | 646854312 ftsY signal recognition particle-docking protein FtsY | IISP\|3.A.5 | 65.9 | 7E-125 |
| SWMB_00634 | ffh; signal recognition particle subunit SRP54 | IISP\|3.A.5 | 638138374 ffh signal recognition particle subunit FFH/SRP54 (srp54) | 98.9 | 0 | 646853939 ffh signal recognition particle subunit FFH/SRP54 (srp54) | IISP\|3.A.5 | 76.2 | 0 |
| Twin arginine targeting family | | | | | | | | | |
| SWMB_00685 | tatA; sec-independent protein translocase protein TatA | Tat\|2.A.64 | 638138323 tatA Sec-independent protein translocase TatA | 97.3 | 3E-72 | 646853765 tatA Sec-independent protein translocase TatA | Tat\|2.A.64 | 63.5 | 4E-19 |
| SWMB_02167 | tatC; sec-independent protein translocase protein TatC | Tat\|2.A.64 | 638137558 tatC Sec-independent protein translocase TatC | 97.2 | 0 | 646854456 tatC Sec-independent protein translocase TatC | Tat\|2.A.64 | 56.8 | 2E-95 |

| | | | | | | | | | |
|---|---|---|---|---|---|---|---|---|---|
| SWMB_02168 | tatA; sec-independent protein translocase protein TatA | Tat\|2.A.64 | 638137559 tatA twin-arginine translocation protein, TatA/E family | 96.7 | 1E-35 | 646854451 tatA sec-independent translocation protein mttA/Hcf106 | Tat\|2.A.64 | 72.1 | 5E-18 |
| Drug resistance-related | | | | | | | | | |
| SWMB_00197 | Inner membrane transport permease YbhR | ABC(membrane)\|3.A.1 | 638137079 Swol_0182 ABC-type multidrug transport system permease component | 46.7 | 2E-128 | | | | |
| SWMB_00198 | Inner membrane transport permease YbhR | ABC(membrane)\|3.A.1 | 638137078 Swol_0181 ABC-type multidrug transport system permease component | 51.0 | 2E-131 | 646853267 Slip_0236 ABC-2 type transporter | ABC(membrane)\|3.A.1 | 31.3 | 4E-44 |
| SWMB_00199 | putative ABC transporter ATP-binding protein YbhF | ABC(ABC)\|3.A.1 | 638137077 Swol_0180 ABC transporter related | 50.0 | 1E-75 | 646853268 Slip_0237 ABC transporter related protein | ABC(ABC)\|3.A.1 | 30.8 | 1.00E-29 |
| SWMB_00200 | Multidrug resistance protein MdtN | OMF\|1.B.17 | 638137076 Swol_0179 secretion protein HlyD | 65.1 | 8E-150 | 646853269 Slip_0238 secretion protein HlyD family protein | Q4VSJ3_BURGL | 40.3 | 5E-61 |
| SWMB_00737 | putative ABC transporter permease YknZ | ABC(membrane)\|3.A.1 | 638139342 Swol_2458 hypothetical protein | 52.5 | 7E-137 | 646853259 Slip_0226 protein of unknown function DUF214 | ABC(ABC)\|3.A.1 | 48.1 | 3E-123 |
| SWMB_00738 | Macrolide export ATP-binding/permease protein MacB | ABC(ABC)\|3.A.1 | 638139341 Swol_2457 ABC-type transport systems, involved in lipoprotein release | 58.6 | 2E-90 | 646853260 Slip_0227 ABC transporter related protein | ABC(ABC)\|3.A.1 | 56.9 | 3E-95 |
| SWMB_00739 | Macrolide-specific efflux protein MacA precursor | OMF\|1.B.17 | 638139340 Swol_2456 hypothetical protein | 32.6 | 1E-42 | 646853261 Slip_0228 efflux transporter, RND family, MFP subunit | OMF\|1.B.17 | 31.7 | 4E-50 |
| SWMB_00800 | Metal-tetracycline/H(+) antiporter | MFS\|2.A.1 | 638138235 Swol_1354 multidrug-efflux transporter | 96.7 | 0 | 646853312 Slip_0281 major facilitator superfamily MFS 1 | MFS\|2.A.1 | 27.2 | 0.0000007 |
| SWMB_00230 | outer membrane channel protein | OMF\|1.B.17 | 638137034 Swol_0134 hypothetical protein | 31.4 | 2E-57 | 646854984 Slip_1958 outer membrane efflux protein | OMF\|1.B.17 | 46.9 | 3E-105 |
| SWMB_00508 | Lipid II:glycine glycyltransferase | RND\|2.A.6 | 638139301 Swol_2417 methicillin resistance protein | 94.3 | 0 | 646855340 Slip_2315 Methicillin resistance protein | ABC(membrane)\|3.A.1 | 60.7 | 2E-161 |
| SWMB_01886 | Lipoprotein-releasing system ATP-binding protein LolD | ABC(ABC)\|3.A.1 | 638138592 Swol_1715 ATPase | 93.2 | 8E-163 | 646854550 Slip_1527 ABC transporter related protein | ABC(ABC)\|3.A.1 | 53.0 | 4E-86 |
| SWMB_01885 | lipoprotein releasing system, transmembrane protein, LolC/E family | ABC(membrane)\|3.A.1 | 638138591 Swol_1714 hypothetical protein | 95.6 | 0 | 646854551 Slip_1528 protein of unknown function DUF214 | ABC(membrane)\|3.A.1 | 29.9 | 2E-124 |
| SWMB_01884 | Macrolide-specific efflux protein MacA precursor | OMF\|1.B.17 | 638138590 Swol_1713 Multidrug resistance efflux pump-like protein | 92.6 | 0 | 646854552 Slip_1529 efflux transporter, RND family, MFP subunit | OMF\|1.B.17 | 28.0 | 7E-35 |
| SWMB_00472 | Inner membrane transport permease YbhS | ABC(membrane)\|3.A.1 | 638139333 Swol_2449 ABC-type multidrug transport system permease component | 98.4 | 0 | 646853762 Slip_0730 ABC-2 type transporter | ABC(membrane)\|3.A.1 | 79.4 | 0 |
| SWMB_00473 | putative ABC transporter ATP-binding protein YbhF | ABC(ABC)\|3.A.1 | 638139332 Swol_2448 ABC transporter-related protein | 95.2 | 0 | 646853761 Slip_0729 ABC transporter related protein | ABC(ABC)\|3.A.1 | 56.5 | 7E-126 |
| SWMB_00474 | putative ABC transporter ATP-binding protein YbhF | ABC(ABC)\|3.A.1 | 638139331 Swol_2447 ABC multidrug efflux pump, ATPase subunit | 94.8 | 0 | 646853760 Slip_0728 ABC transporter related protein | ABC(ABC)\|3.A.1 | 57.1 | 1E-120 |
| SWMB_00475 | Cation efflux system protein CzcB | P-ATPase\|3.A.3 | 638139330 Swol_2446 secretion protein HlyD | 93.4 | 0 | 646853759 Slip_0727 secretion protein HlyD family protein | OMF\|1.B.17 | 51.8 | 3E-106 |
| SWMB_00745 | ABC-type transport system involved in multi-copper enzyme maturation, permease component | ABC(membrane)\|3.A.1 | 638138264 Swol_1384 hypothetical protein | 88.7 | 8E-92 | 646854320 Slip_1296 hypothetical protein | ABC(membrane)\|3.A.1 | 30.5 | 0.032 |
| SWMB_00746 | ABC transporter ATP-binding protein YtrB | ABC(ABC)\|3.A.1 | 638138263 Swol_1383 putative ABC transporter ATP-binding protein; RBL01943 | 94.1 | 0 | 646854321 Slip_1297 ABC transporter related protein | ABC(ABC)\|3.A.1 | 31.3 | 8E-42 |
| SWMB_00203 | putative multidrug efflux system subunit MdtC | RND\|2.A.6 | 638137073 Swol_0176 acriflavin resistance protein | 99.6 | 0 | 646854982 Slip_1956 acriflavin resistance protein | RND\|2.A.6 | 56.0 | 0 |
| SWMB_00204 | Macrolide-specific efflux protein MacA precursor | MFS\|2.A.1 | 638137072 Swol_0175 hypothetical protein | 95.3 | 0 | 646854983 Slip_1957 efflux transporter, RND family, MFP subunit | CNRB_ALCEU | 38.9 | 4E-82 |
| SWMB_00205 | sodium/calcium exchanger 1 | P-ATPase\|3.A.3 | 638137071 Swol_0174 hypothetical protein | 98.6 | 0 | | | | |
| SWMB_01122 | Macrolide export ATP-binding/permease protein MacB | ABC(membrane)\|3.A.1 | 638137817 Swol_0943 ABC transporter component-like protein | 92.3 | 0 | | | | |
| SWMB_02076 | Bacillibactin exporter | GPH\|2.A.2 | 638137998 Swol_1125 drug resistance transporter, EmrB/QacA family protein | 24.0 | 5E-06 | | | | |
| SWMB_02079 | Multidrug-efflux transporter 1 regulator | ABC(ABC)\|3.A.1 | 638138244 Swol_1364 hypothetical protein | 22.6 | 4E-06 | | | | |
| SWMB_00952 | Lactococcin-G-processing and transport ATP-binding protein LagD | ABC(membrane)\|3.A.1 | 638139110 Swol_2230 hypothetical protein | 97.2 | 0 | | | | |
| SWMB_00953 | NHLM bacteriocin system secretion protein | ABC(membrane)\|3.A.1 | 638139109 Swol_2229 hypothetical protein | 97.0 | 5E-117 | | | | |
| SWMB_02518 | Cation efflux system protein CzcA | RND\|2.A.6 | 638137990 Swol_1118 multidrug efflux pump subunit AcrB | 37.2 | 2E-44 | | | | |
| SWMB_02519 | Multidrug transporter MdtC | RND\|2.A.6 | 638137990 Swol_1118 multidrug efflux pump subunit AcrB | 22.4 | 3E-06 | | | | |
| SWMB_02257 | hypothetical protein | ABC(binding protein)\|3.A.1 | 638138559 Swol_1682 hypothetical protein | 91.06 | 2.00E-91 | | | | |
| SWMB_02258 | Macrolide export ATP-binding/permease protein MacB | ABC(ABC)\|3.A.1 | 638138560 Swol_1683 SN-glycerol-3-phosphate transport ATP-binding protein (UgpC) | 93.4 | 9E-179 | | | | |
| SWMB_02949 | Multidrug export protein MepA | MOP(MOP)\|2.A.66 | 638137201 Swol_0313 hypothetical protein | 95.1 | 0 | | | | |
| SWMB_02407 | Na(+)/drug antiporter | MOP(MOP)\|2.A.66 | 638137457 Swol_0575 MATE efflux family protein | 97.6 | 0 | | | | |
| SWMB_00763 | Bacitracin export permease protein BceB | ABC(membrane)\|3.A.1 | | | | | | | |
| SWMB_00764 | Bacitracin export permease protein BceB | ABC(membrane)\|3.A.1 | | | | | | | |
| SWMB_02781 | Macrolide export ATP-binding/permease protein MacB | ABC(ABC)\|3.A.1 | | | | | | | |
| SWMB_02782 | ABC exporter transmembrane subunit, DevC protein | ABC(membrane)\|3.A.1 | | | | | | | |
| SWMB_01636 | cysteine/glutathione ABC transporter membrane/ATP-binding component | ABC(membrane)\|3.A.1 | | | | | | | |
| SWMB_01637 | Iron import ATP-binding/permease protein IrtA | ABC(membrane)\|3.A.1 | | | | | | | |
| SWMB_01638 | Putative multidrug export ATP-binding/permease protein | ABC(membrane)\|3.A.1 | | | | | | | |
| SWMB_02494 | ABC-type transport system involved in multi-copper enzyme maturation, permease component | MFS\|2.A.1 | | | | | | | |
| SWMB_02495 | putative ABC transporter ATP-binding protein YxlF | ABC(ABC)\|3.A.1 | | | | | | | |
| SWMB_02599 | Daunorubicin/doxorubicin resistance ABC transporter permease protein DrrB | ABC(membrane)\|3.A.1 | | | | 646854525 Slip_1503 ABC-2 type transporter | ABC(membrane)\|3.A.1 | 21.8 | 0.00003 |
| SWMB_02600 | Daunorubicin/doxorubicin resistance ATP-binding protein DrrA | ABC(ABC)\|3.A.1 | | | | 646854526 Slip_1504 ABC transporter related protein | ABC(ABC)\|3.A.1 | 41.2 | 2E-53 |
| | | | | | | 646854062 Slip_1030 drug resistance transporter, EmrB/QacA subfamily | MFS\|2.A.1 | | |
| | | | | | | 646855366 Slip_2340 drug resistance transporter, EmrB/QacA subfamily | MFS\|2.A.1 | | |
| | | | | | | 646854250 Slip_1222 ABC-2 type transporter | ABC(membrane)\|3.A.1 | | |
| | | | | | | 646854251 Slip_1223 daunorubicin resistance ABC transporter ATPase subunit | ABC(ABC)\|3.A.1 | | |
| Other transporters | | | | | | | | | |
| SWMB_01088 | Sulfite exporter TauE/SafE | TSUP\|9.A.29 | 638138964 Swol_2083 permease | 94.3 | 1E-143 | 646855214 Slip_2191 protein of unknown function DUF81 | TSUP\|9.A.29 | 39.9 | 1E-41 |
| SWMB_01120 | ABC transporter ATP-binding protein YtrE | ABC(ABC)\|3.A.1 | 638137815 Swol_0941 ATP binding protein of ABC transporter | 96.3 | 8E-154 | 646853136 Slip_0092 ABC transporter related protein | ABC(ABC)\|3.A.1 | 46.0 | 4E-62 |
| SWMB_01814 | Putative K(+)-stimulated pyrophosphate-energized sodium pump | ABC(ABC)\|3.A.1 | 638137933 hppA Inorganic diphosphatase | 97.6 | 0 | 646853507 hppA Inorganic diphosphatase | H+-PPase\|3.A.10 | 87.8 | 0 |
| SWMB_01918 | Arsenic efflux pump protein | DASS\|2.A.47, ArsB\|2.A.45 | 638139046 Swol_2166 possible tyrosine transporter P-protein (TC 2A4521) | 23.2 | 2E-07 | 646854717 Slip_1687 possible tyrosine transporter P-protein | DASS\|2.A.47, ArsB\|2.A.45 | 58.1 | 2E-168 |
| SWMB_02574 | Mycobacterial copper transport protein B | ABC(membrane)\|3.A.1 | 638137471 Swol_0592 hypothetical protein | 98.3 | 0 | 646853474 Slip_0444 hypothetical protein | ABC(membrane)\|3.A.1 | 47.1 | 8E-98 |
| SWMB_02731 | antiporter inner membrane protein | ABC(binding protein)\|3.A.1 | 638138085 Swol_1211 nucleotide-binding protein | 95.7 | 0 | 646853371 Slip_0341 ATPase-like, ParA/MinD | CAPB_STAAU | 60.2 | 3E-115 |
| SWMB_02901 | ABC-type uncharacterized transport system, periplasmic component | ABC(binding protein)\|3.A.1 | 638137212 Swol_0325 hypothetical protein | 97.9 | 8E-26 | | | | |
| SWMB_02902 | hypothetical protein | MC\|2.A.29 | 638136994 Swol_0092 hypothetical protein | 72.73 | 4.00E-05 | | | | |
| SWMB_02903 | ABC-type uncharacterized transport system, periplasmic component | ABC(binding protein)\|3.A.1 | 638137212 Swol_0325 hypothetical protein | 98.7 | 1E-163 | 646854202 Slip_1174 protein of unknown function DUF534 | ABC(binding protein)\|3.A.1 | 77.1 | 8E-129 |
| SWMB_02904 | Autoinducer 2 import system permease protein LsrCS | ABC(membrane)\|3.A.1 | 638137211 Swol_0324 hypothetical protein | 96.4 | 2E-156 | 646854201 Slip_1173 inner-membrane translocator | ABC(membrane)\|3.A.1 | 70.0 | 7E-99 |
| SWMB_02905 | Spermidine/putrescine import ATP-binding protein PotA | ABC(ABC)\|3.A.1 | 638137210 Swol_0323 hypothetical protein | 95.8 | 0 | 646854200 Slip_1172 ABC transporter related protein | ABC(ABC)\|3.A.1 | 71.5 | 1E-139 |
| SWMB_01254 | putative cation efflux system protein c/MT2084 | CDF\|2.A.4 | 638137922 Swol_1051 putative cation efflux system protein | 96.2 | 0 | 646853798 Slip_0766 cation diffusion facilitator family transporter | CDF\|2.A.4 | 26.4 | 3E-22 |
| SWMB_02352 | MscS family inner membrane protein YnaI | MscS\|1.A.23 | 638138027 Swol_1154 hypothetical protein | 90.4 | 0 | 646853293 Slip_0262 MscS Mechanosensitive ion channel | MscS\|1.A.23 | 48.2 | 1E-96 |
| SWMB_00237 | putative bicarbonate transporter, IctB family | CPA2\|2.A.37 | 638137027 Swol_0127 hypothetical protein | 94.0 | 0 | 646853204 Slip_0160 putative bicarbonate transporter, IctB family | Putative membrane protein | 43.2 | 1E-169 |
| SWMB_01893 | gliding motility-associated ABC transporter permease protein GldF | ABC(membrane)\|3.A.1 | 638138599 Swol_1722 hypothetical protein | 94.2 | 0 | | | | |
| SWMB_01894 | putative ABC transporter ATP-binding protein YbhF | ABC(ABC)\|3.A.1 | 638138600 Swol_1723 ABC transporter ATP-binding protein | 98.1 | 0 | 646853067 Slip_0021 ABC transporter related protein | ABC(ABC)\|3.A.1 | 33.8 | 3E-54 |
| SWMB_01074 | ABC transporter, ATP-binding protein | ABC(ABC)\|3.A.1 | 638138975 Swol_2094 ABC transporter, ATP-binding protein | 96.7 | 0 | | | | |
| SWMB_02087 | ABC-type transporter ATP-binding protein EcsA | ABC(ABC)\|3.A.1 | 638138920 Swol_2038 ABC transporter ATP-binding protein | 98.8 | 2E-179 | | | | |
| SWMB_00659 | putative ABC transporter permease protein | ABC(membrane)\|3.A.1 | 638138351 Swol_1473 iron(III) ABC transporter, permease protein | 99.7 | 0 | | | | |
| SWMB_00660 | putative ABC transporter ATP-binding protein | ABC(ABC)\|3.A.1 | 638138350 Swol_1472 iron(III) ABC transporter, ATP-binding protein | 98.1 | 0 | | | | |
| SWMB_01626 | putative ABC transporter permease protein | ABC(membrane)\|3.A.1 | 638138351 Swol_1473 iron(III) ABC transporter, permease protein | 52.5 | 4E-110 | | | | |
| SWMB_01627 | putative ABC transporter ATP-binding protein | ABC(ABC)\|3.A.1 | 638138350 Swol_1472 iron(III) ABC transporter, ATP-binding protein | 44.0 | 3E-69 | | | | |
| SWMB_01865 | putative ABC transporter ATP-binding protein | ABC(ABC)\|3.A.1 | 638138571 Swol_1694 ABC transporter related | 96.0 | 0 | | | | |
| SWMB_01866 | putative ABC transporter permease protein | ABC(membrane)\|3.A.1 | 638138572 Swol_1695 transport system permease protein | 96.6 | 0 | | | | |
| SWMB_00426 | corrinoid ABC transporter substrate-binding protein | ABC(binding protein)\|3.A.1 | 638139371 Swol_2486 ferrichrome-binding periplasmic protein | 93.2 | 0 | | | | |
| SWMB_00431 | Spermidine/putrescine import ATP-binding protein PotA | ABC(ABC)\|3.A.1 | 638139366 Swol_2481 ABC transporter, ATP-binding protein | 93.9 | 0 | | | | |
| SWMB_00432 | Spermidine/putrescine transport system permease protein PotB | ABC(membrane)\|3.A.1 | 638139365 Swol_2480 ABC transporter, permease protein, putative | 95.6 | 0 | | | | |
| SWMB_00855 | putative ABC transporter ATP-binding protein YbhF | ABC(ABC)\|3.A.1 | 638139332 Swol_2448 ABC transporter-related protein | 33.3 | 5E-51 | | | | |
| SWMB_00856 | gliding motility-associated ABC transporter permease protein GldF | ABC(membrane)\|3.A.1 | 638139333 Swol_2449 ABC-type multidrug transport system permease component | 38.6 | 0.0008 | | | | |
| SWMB_00915 | ABC transporter ATP-binding protein YtrB | ABC(ABC)\|3.A.1 | 638139123 Swol_2243 ABC-type multidrug transport system ATPase component-like protein | 94.9 | 0 | | | | |
| SWMB_00916 | ABC-type transport system involved in multi-copper enzyme maturation, permease component | ABC(membrane)\|3.A.1 | 638139122 Swol_2242 putative ABC-2 type transport system permease protein | 96.6 | 0 | | | | |
| SWMB_00917 | Putative heavy-metal-binding domain YbjQ_1 | ABC(membrane)\|3.A.1 | 638139121 Swol_2241 hypothetical protein | 95.2 | 3E-66 | | | | |
| SWMB_00918 | putative sulfoacetate transporter SauU | MFS\|2.A.1 | 638139120 Swol_2240 putative permease | 95.9 | 0 | | | | |
| SWMB_01071 | ferrichrome/ferrioxamine B periplasmic transporter | ABC(binding protein)\|3.A.1 | 638138978 Swol_2097 periplasmic binding protein | 95.0 | 0 | | | | |
| SWMB_01139 | putative DMT superfamily transporter inner membrane protein | DMT\|2.A.7 | 638137834 Swol_0960 drug/metabolite transporter (DMT) superfamily permease | 95.3 | 1E-179 | | | | |
| SWMB_01858 | vitamin B12-transporter protein BtuF | ABC(binding protein)\|3.A.1 | 638138564 Swol_1687 periplasmic binding protein | 95.3 | 0 | | | | |
| SWMB_01860 | iron-dicitrate transporter substrate-binding subunit | ABC(binding protein)\|3.A.1 | 638138567 Swol_1690 periplasmic binding protein | 64.8 | 0 | | | | |
| SWMB_01862 | ferrichrome/ferrioxamine B periplasmic transporter | ABC(binding protein)\|3.A.1 | 638138567 Swol_1690 periplasmic binding protein | 94.5 | 0 | | | | |
| SWMB_02185 | ABC-2 family transporter protein | ABC(ABC)\|3.A.1 | 638138498 Swol_1619 hypothetical protein | 94.8 | 2E-148 | | | | |
| SWMB_02186 | SkfA peptide export ATP-binding protein SkfE | ABC(ABC)\|3.A.1 | 638138499 Swol_1620 ABC transporter related | 96.1 | 1E-166 | | | | |
| SWMB_02252 | corrinoid ABC transporter substrate-binding protein | ABC(binding protein)\|3.A.1 | 638138556 Swol_1679 periplasmic binding protein | 97.5 | 0 | | | | |
| SWMB_02867 | Neu5Ac permease | TRAP-T\|2.A.56 | 638137215 Swol_0329 TRAP dicarboxylate transporter-DctM subunit | 76.0 | 1E-130 | | | | |
| SWMB_02868 | L-dehydroascorbate transporter large permease subunit | TRAP-T\|2.A.56 | 638137215 Swol_0329 TRAP dicarboxylate transporter-DctM subunit | 81.3 | 1E-41 | | | | |
| SWMB_02897 | TRAP-type C4-dicarboxylate transport system, small permease component | TRAP-T\|2.A.56 | 638137216 Swol_0330 tripartite ATP-independent periplasmic transporters, DctQ component | 98.8 | 3E-113 | | | | |
| SWMB_02898 | Neu5Ac permease | TRAP-T\|2.A.56 | 638137215 Swol_0329 TRAP dicarboxylate transporter-DctM subunit | 98.2 | 0 | | | | |
| SWMB_03030 | putative ABC transporter ATP-binding protein YheS | ABC(ABC)\|3.A.1 | 638138834 Swol_1951 ABC-type cobalt transport system, ATPase component | 32.4 | 3E-07 | | | | |
| SWMB_00954 | urea ABC transporter, permease protein UrtB | ABC(membrane)\|3.A.1 | 638139108 Swol_2228 nucleoside ABC transporter membrane protein | 97.7 | 1E-151 | | | | |
| SWMB_00955 | L-arabinose transporter permease protein | ABC(membrane)\|3.A.1 | | | | | | | |
| SWMB_00956 | Galactose/methyl galactoside import ATP-binding protein MglA | ABC(ABC)\|3.A.1 | | | | | | | |
| SWMB_01511 | putative ABC transporter ATP-binding protein | ABC(membrane)\|3.A.1 | | | | | | | |
| SWMB_01512 | putative ABC transporter ATP-binding protein | ABC(membrane)\|3.A.1 | | | | | | | |
| SWMB_00040 | ABC transporter permease YtrF precursor | ABC(membrane)\|3.A.1 | | | | | | | |
| SWMB_00041 | Lipoprotein-releasing system ATP-binding protein LolD | ABC(ABC)\|3.A.1 | | | | | | | |
| SWMB_02479 | putative ABC-type exoprotein transport system, permease component | DMT\|2.A.7 | | | | | | | |
| SWMB_02480 | ABC-type transporter ATP-binding protein EcsA | ABC(ABC)\|3.A.1 | | | | | | | |
| SWMB_01565 | ABC-2 type transport system permease protein | APC\|2.A.3 | | | | 646854787 Slip_1757 hypothetical protein | ABC(membrane)\|3.A.1 | 32.0 | 1E-52 |
| SWMB_01566 | ABC transporter ATP-binding protein YtrB | ABC(ABC)\|3.A.1 | | | | 646854786 Slip_1756 ABC transporter related protein | ABC(ABC)\|3.A.1 | 50.8 | 7E-111 |
| | | | | | | 646853066 Slip_0020 ABC-2 type transporter | ABC(membrane)\|3.A.1 | | |
| | | | | | | 646853137 Slip_0093 efflux transporter, RND family, MFP subunit | OMF\|1.B.17 | | |
| | | | | | | 646853138 Slip_0094 outer membrane efflux protein | OMF\|1.B.17 | | |
| | | | | | | 646854195 Slip_1168 ABC transporter, ATPase, predicted | ABC(ABC)\|3.A.1 | | |
| | | | | | | 646853949 Slip_0916 type III secretion exporter | FLHB_SALTY | | |
| | | | | | | 646853613 Slip_0582 putative succinate-acetate transporter protein | DMT\|2.A.7 | | |
| | | | | | | 646853657 Slip_0626 LAO/AO transport system ATPase | CDF\|2.A.4 | | |
| | | | | | | 646853792 Slip_0760 LAO/AO transport system ATPase | CDF\|2.A.4 | | |

The gray bars indicate that there are no corresponding genes with significant similarity (<20%).

Table S4. *Syntrophomonas wolfei* subsp. *metylbutyratica* strain 4J5$^T$ genes encoding β-oxidation pathway.

| Locus tag | Gene product | Best BLAST hit in: *Syntrophomonas wolfei* Goettingen | | | *Syntrophothermus lipocalidus* DSM 12680 | | |
|---|---|---|---|---|---|---|---|
| | | Locus tag | %ID | e-value | Locus tag | %ID | e-value |
| Acyl-CoA dehydrogenase | | | | | | | |
| SWMB_00118 | Acyl-CoA dehydrogenase | Swol_0268 | 99.2 | 0 | Slip_0874 | 82.6 | 0 |
| SWMB_00649 | Acyl-CoA dehydrogenase | Swol_1483 | 98.7 | 0 | Slip_0286 | 83.5 | 0 |
| SWMB_01042 | Acyl-CoA dehydrogenase | Swol_2126 | 98.7 | 0 | Slip_0456 | 63.1 | 0 |
| SWMB_01416 | Acyl-CoA dehydrogenase | Swol_0788 | 99.7 | 0 | Slip_0501 | 70.2 | 0 |
| SWMB_01499 | Acyl-CoA dehydrogenase, short-chain specific | Swol_1933 | 99.2 | 0 | Slip_0881 | 72.0 | 0 |
| SWMB_01701 | Acyl-CoA dehydrogenase | Swol_0788 | 66.5 | 0 | Slip_0501 | 73.9 | 0 |
| SWMB_01732 | Acyl-CoA dehydrogenase, short-chain specific | Swol_0384 | 97.7 | 0 | Slip_0481 | 56.4 | 0 |
| SWMB_01942 | Acyl-CoA dehydrogenase | Swol_0788 | 44.3 | 1E-105 | Slip_1241 | 75.3 | 0 |
| SWMB_02100 | Acyl-CoA dehydrogenase | Swol_2052 | 94.9 | 0 | Slip_0881 | 71.6 | 0 |
| SWMB_02510 | Acyl-CoA dehydrogenase | Swol_0384 | 72.2 | 0 | Slip_0481 | 54.9 | 0 |
| Enoyl-CoA hydratase | | | | | | | |
| SWMB_00384 | putative enoyl-CoA hydratase echA8 | Swol_2566 | 87.4 | 3.00E-161 | | | |
| SWMB_00648 | 2-methyl-but-2-enoyl-CoA hydratase | Swol_1484 | 97.3 | 0 | Slip_2197 | 63.5 | 1.00E-120 |
| SWMB_01414 | putative enoyl-CoA hydratase echA8 | Swol_0790 | 96.5 | 0 | Slip_2197 | 63.1 | 1E-113 |
| SWMB_01496 | putative enoyl-CoA hydratase echA8 | Swol_1936 | 98.9 | 0 | Slip_2197 | 66.5 | 3E-122 |
| SWMB_01703 | 2-methyl-but-2-enoyl-CoA hydratase | Swol_0790 | 42.3 | 1E-59 | Slip_0502 | 65.6 | 1E-115 |
| SWMB_02083 | putative enoyl-CoA hydratase echA8 | Swol_2031 | 97.7 | 0 | Slip_2197 | 64.6 | 1E-115 |
| SWMB_02134 | putative enoyl-CoA hydratase echA8 | Swol_0650 | 98.4 | 0 | | | |
| SWMB_02508 | 2-methyl-but-2-enoyl-CoA hydratase | Swol_2031 | 67.3 | 9.00E-129 | Slip_2197 | 56.9 | 8.00E-100 |
| SWMB_03023 | putative enoyl-CoA hydratase echA8 | Swol_2031 | 45.8 | 9E-76 | Slip_0502 | 68.0 | 2E-125 |
| 3-hydroxybutyryl-CoA dehydrogenase | | | | | | | |
| SWMB_00002 | putative 3-hydroxybutyryl-CoA dehydrogenase | Swol_0307 | 99.3 | 0 | Slip_0503 | 54.6 | 9E-107 |
| SWMB_00647 | 2-methyl-3-hydroxybutyryl-CoA dehydrogenase | Swol_1485 | 97.5 | 0 | Slip_0285 | 82.9 | 6E-168 |
| SWMB_01413 | putative 3-hydroxybutyryl-CoA dehydrogenase | Swol_0791 | 98.6 | 0 | Slip_1415 | 56.8 | 2E-112 |
| SWMB_01497 | putative 3-hydroxybutyryl-CoA dehydrogenase | Swol_1935 | 99.3 | 0 | Slip_1415 | 82.4 | 5E-172 |
| SWMB_01704 | 2-methyl-3-hydroxybutyryl-CoA dehydrogenase | Swol_0435 | 93.0 | 7.00E-129 | Slip_0503 | 71.3 | 7E-123 |
| SWMB_01947 | putative 3-hydroxybutyryl-CoA dehydrogenase | Swol_0435 | 61.8 | 1.00E-84 | Slip_1238 | 73.2 | 2E-145 |
| SWMB_02082 | putative 3-hydroxybutyryl-CoA dehydrogenase | Swol_2030 | 99.3 | 0 | Slip_0503 | 55.6 | 5E-109 |
| SWMB_02507 | 2-methyl-3-hydroxybutyryl-CoA dehydrogenase | Swol_0791 | 68.8 | 9E-139 | Slip_0503 | 54.6 | 5E-103 |
| SWMB_02718 | putative 3-hydroxybutyryl-CoA dehydrogenase | Swol_1171 | 96.8 | 0 | Slip_1415 | 53.4 | 5E-107 |
| Acetyl-CoA acetyltransferase | | | | | | | |
| SWMB_00001 | Acetyl-CoA acetyltransferase | Swol_0308 | 97.5 | 0 | Slip_0499 | 59.9 | 9E-172 |
| SWMB_00646 | 2-methyl-acetoacetyl-CoA acetyltransferase | Swol_1486 | 97.8 | 0 | Slip_0284 | 62.8 | 1E-163 |
| SWMB_01415 | Acetyl-CoA acetyltransferase | Swol_0789 | 98.0 | 0 | Slip_0499 | 66.8 | 0 |
| SWMB_01498 | Acetyl-CoA acetyltransferase | Swol_1934 | 98.0 | 0 | Slip_0880 | 79.8 | 0 |
| SWMB_01702 | 2-methyl-acetoacetyl-CoA acetyltransferase | Swol_0789 | 74.5 | 0 | Slip_0499 | 66.5 | 0 |
| SWMB_01946 | Acetyl-CoA acetyltransferase | Swol_0789 | 72.7 | 0 | Slip_0499 | 68.4 | 0 |
| SWMB_02099 | Acetyl-CoA acetyltransferase | Swol_2051 | 95.7 | 0 | Slip_0880 | 81.1 | 0 |
| SWMB_02161 | Acetyl-CoA acetyltransferase | Swol_0675 | 99.5 | 0 | Slip_0499 | 67.3 | 0 |
| SWMB_02509 | 2-methyl-acetoacetyl-CoA acetyltransferase | Swol_0675 | 62.6 | 0 | Slip_0499 | 65.8 | 0 |
| CoA transferase | | | | | | | |
| SWMB_00469 | Succinyl-CoA:coenzyme A transferase | Swol_0436 | 72.0 | 0 | Slip_0882 | 62.0 | 0 |
| SWMB_00650 | Acyl-CoA:acetate/3-ketoacid CoA transferase | Swol_1482 | 96.0 | 0 | Slip_0287 | 80.9 | 0 |
| SWMB_00651 | Acyl-CoA:acetate/3-ketoacid CoA transferase | Swol_1481 | 99.3 | 0 | Slip_0288 | 80.8 | 8E-167 |
| SWMB_01218 | Succinyl-CoA:coenzyme A transferase | Swol_1014 | 96.2 | 0 | Slip_0882 | 57.3 | 0 |
| SWMB_01500 | Succinyl-CoA:coenzyme A transferase | Swol_1932 | 99.8 | 0 | Slip_0882 | 56.5 | 0 |
| SWMB_01699 | Acyl-CoA:acetate/3-ketoacid CoA transferase | Swol_0436 | 97.3 | 0 | Slip_0882 | 60.8 | 0 |
| SWMB_01700 | Acyl-CoA:acetate/3-ketoacid CoA transferase | Swol_0309 | 50.3 | 3.00E-162 | Slip_0500 | 50.3 | 9E-164 |
| SWMB_01907 | Succinyl-CoA:coenzyme A transferase | Swol_1735 | 98.6 | 0 | Slip_0665 | 40.2 | 2E-108 |
| SWMB_01923 | butyryl-CoA:acetate CoA-transferase | Swol_1744 | 95.9 | 1E-119 | Slip_0665 | 31.9 | 1E-24 |
| SWMB_02359 | Succinyl-CoA:coenzyme A transferase | Swol_1147 | 96.7 | 0 | Slip_0500 | 62.6 | 0 |
| SWMB_02505 | Acyl-CoA:acetate/3-ketoacid CoA transferase | Swol_1147 | 63.1 | 0 | Slip_0500 | 59.2 | 0 |
| SWMB_03019 | butyryl-CoA:acetate CoA-transferase | Swol_1932 | 34.3 | 2E-11 | Slip_0882 | 29.1 | 0.0000007 |
| SWMB_03052 | Succinyl-CoA:coenzyme A transferase | Swol_0787 | 98.2 | 4E-123 | Slip_0500 | 73.1 | 0 |
| Acetate kinase/phosphotransacetylase gene cassette | | | | | | | |
| SWMB_02801 | Phosphate acetyltransferase (phosphotransacetylase) | Swol_0767 | 98.48 | 0 | Slip_0902 | 77.9 | 0 |
| SWMB_02802 | Acetate kinase | Swol_0768 | 98.5 | 0 | Slip_0903 | 82.1 | 0 |
| Acyl-CoA synthetase | | | | | | | |
| SWMB_02363 | Long-chain-fatty-acid--CoA ligase (for PHA metabolism?) | Swol_1144 | 93.6 | 0 | Slip_0583 | 25.9 | 4E-26 |
| SWMB_02710 | Long-chain-fatty-acid--CoA ligase FadD13 (for coenzyme A biosynthesis?) | Swol_1180 | 99.3 | 0 | Slip_1686 | 64.4 | 0 |

The gray bars indicate that there are no corresponding genes with significant similarity (<20%).

Table S5. *Syntrophothermus lipocalidus strain* TGB-C1 genes encoding β-oxidation pathway.

| Locus tag | Gene product | Best BLAST hit in: *Syntrophomonas wolfei* Goettingen | | | *Syntrophomonas wolfei* subsp.*metylbutyratica* JCM14075T | | |
|---|---|---|---|---|---|---|---|
| | | Locus tag | %ID | e-value | Locus tag | %ID | e-value |
| Acyl-CoA dehydrogenase | | | | | | | |
| Slip_0286 | acyl-CoA dehydrogenase | Swol_1483 | 84.3 | 0 | SWMB_00649 | 83.5 | 0 |
| Slip_0481 | acyl-CoA dehydrogenase | Swol_0384 | 57.0 | 0 | SWMB_02510 | 54.9 | 0 |
| Slip_0501 | acyl-CoA dehydrogenase | Swol_0788 | 70.2 | 0 | SWMB_01701 | 73.9 | 0 |
| Slip_0581 | acyl-CoA dehydrogenase domain protein | Swol_0788 | 34.8 | 4.00E-66 | SWMB_01701 | 37.4 | 9.00E-72 |
| Slip_0881 | acyl-CoA dehydrogenase | Swol_1933 | 72.1 | 0 | SWMB_01499 | 72.0 | 0 |
| Slip_0884 | acyl-CoA dehydrogenase | | | | | | |
| Slip_1241 | Butyryl-CoA dehydrogenase | Swol_0788 | 44.5 | 9.00E-112 | SWMB_01942 | 75.3 | 0 |
| Slip_1986 | acyl-CoA dehydrogenase | | | | | | |
| Slip_1987 | short-chain dehydrogenase/reductase | | | | | | |
| Slip_2083 | acyl-CoA dehydrogenase | | | | | | |
| Slip_2087 | short-chain dehydrogenase/reductase | | | | | | |
| Enoyl-CoA hydratase | | | | | | | |
| Slip_0485 | short chain enoyl-CoA hydratase | | | | | | |
| Slip_0486 | Enoyl-CoA hydratase/isomerase | | | | | | |
| Slip_0490 | Enoyl-CoA hydratase | | | | | | |
| Slip_0502 | short chain enoyl-CoA hydratase | Swol_2031 | 50.4 | 2.00E-83 | SWMB_03023 | 68.0 | 2.00E-125 |
| Slip_0580 | enoyl-CoA hydratase/isomerase | Swol_2031 | 47.7 | 9.00E-79 | SWMB_01498 | 48.1 | 2.00E-77 |
| Slip_1234 | 3-hydroxybutyryl-CoA dehydratase | Swol_1936 | 43.5 | 4.00E-72 | SWMB_01090 | 30.1 | 7.00E-22 |
| Slip_1989 | Enoyl-CoA hydratase/isomerase | | | | | | |
| Slip_2089 | Enoyl-CoA hydratase/isomerase | Swol_2566 | 36.6 | 5.00E-41 | SWMB_00384 | 37.2 | 2.00E-44 |
| Slip_2197 | short chain enoyl-CoA hydratase | Swol_1936 | 67.3 | 3.00E-123 | SWMB_01496 | 66.5 | 3.00E-122 |
| 3-hydroxybutyryl-CoA dehydrogenase | | | | | | | |
| Slip_0285 | 3-hydroxyacyl-CoA dehydrogenase | Swol_1485 | 83.7 | 1.00E-180 | SWMB_00647 | 82.6 | 7.00E-180 |
| Slip_0503 | 3-hydroxyacyl-CoA dehydrogenase | Swol_1935 | 55.6 | 4.00E-109 | SWMB_01704 | 71.3 | 1.00E-150 |
| Slip_0579 | 3-hydroxyacyl-CoA dehydrogenase NAD-binding protein | Swol_1935 | 47.3 | 3.00E-92 | SWMB_01497 | 47.3 | 5.00E-92 |
| Slip_1238 | 3-hydroxybutyryl-CoA dehydrogenase | Swol_1935 | 54.7 | 1.00E-110 | SWMB_01947 | 73.2 | 2.00E-101 |
| Slip_1415 | 3-hydroxybutyryl-CoA dehydrogenase | Swol_1935 | 82.8 | 2.00E-172 | SWMB_01497 | 82.4 | 5.00E-172 |
| Slip_2088 | putative 3-hydroxybutyryl-CoA dehydrogenase | | | | | | |
| Acetyl-CoA acetyltransferase | | | | | | | |
| Slip_0284 | acetyl-CoA acetyltransferase | Swol_1486 | 62.8 | 2.00E-177 | SWMB_00646 | 62.8 | 8.00E-178 |
| Slip_0464 | Propanoyl-CoA C-acyltransferase | | | | | | |
| Slip_0465 | Acetyl-CoA acetyltransferase | | | | | | |
| Slip_0479 | Acetyl-CoA acetyltransferase | | | | | | |
| Slip_0499 | Acetyl-CoA acetyltransferase | Swol_0675 | 67.0 | 0 | SWMB_02161 | 67.3 | 0 |
| Slip_0577 | putative acetyl-CoA acetyltransferase | Swol_2051 | 28.2 | 1.00E-33 | SWMB_01702 | 30.0 | 1.00E-34 |
| Slip_0880 | Acetyl-CoA acetyltransferase | Swol_2051 | 80.3 | 0 | SWMB_02099 | 81.1 | 0 |
| Slip_1111 | Acetyl-CoA acetyltransferase | | | | SWMB_01090 | 64.2 | 1.00E-180 |
| Slip_1236 | Acetyl-CoA acetyltransferase | Swol_2051 | 22.9 | 8.00E-11 | SWMB_01703 | 47.1 | 7.00E-85 |
| Slip_1985 | acetyl-CoA acetyltransferase | | | | | | |
| Slip_1988 | acetyl-CoA acetyltransferase | | | | | | |
| Slip_2084 | Crotonobetainyl-CoA:carnitine CoA-transferase CaiB and related acyl-CoA transferases | | | | | | |
| Slip_2085 | acetyl-CoA acetyltransferase | | | | | | |
| CoA transferase | | | | | | | |
| Slip_0098 | putative acyl CoA:acetate/3-ketoacid CoA transferase, alpha subunit | | | | SWMB_01948 | 72.4 | 6.00E-67 |
| Slip_0099 | putative acyl CoA:acetate/3-ketoacid CoA transferase, beta subunit | | | | | | |
| Slip_0287 | coenzyme A transferase | Swol_1482 | 78.5 | 0 | SWMB_00650 | 80.9 | 0 |
| Slip_0288 | putative acyl CoA:acetate/3-ketoacid CoA transferase | Swol_1481 | 80.4 | 4.00E-166 | SWMB_00651 | 80.8 | 8.00E-167 |
| Slip_0500 | acetyl-CoA hydrolase/transferase | Swol_0309 | 74.7 | 0 | SWMB_03052 | 73.1 | 0 |
| Slip_0578 | putative CoA-transferase family III | | | | | | |
| Slip_0665 | putative 4-hydroxybutyrate CoA-transferase | Swol_1744 | 40.1 | 9.00E-109 | SWMB_01907 | 40.2 | 2.00E-108 |
| Slip_1192 | coenzyme A transferase | | | | | | |
| Slip_1193 | coenzyme A transferase | | | | | | |
| Slip_1195 | putative acyl CoA:acetate/3-ketoacid CoA transferase beta subunit | | | | | | |
| Slip_1196 | putative acyl CoA:acetate/3-ketoacid CoA transferase alpha subunit | | | | | | |
| Slip_1239 | Acyl-CoA:acetate/3-ketoacid CoA transferase | Swol_1481 | 41.4 | 7.00E-54 | SWMB_00651 | 41.4 | 9.00E-54 |
| Slip_1240 | Acyl-CoA:acetate/3-ketoacid CoA transferase | Swol_1482 | 29.1 | 3.00E-29 | SWMB_01944 | 30.7 | 2.00E-35 |
| Slip_1981 | CoA-transferase family III domain | | | | | | |
| Acetate kinase/phosphotransacetylase gene cassette | | | | | | | |
| Slip_0902 | Phosphate acetyltransferase (phosphotransacetylase) | Swol_0767 | 76.7 | 0 | SWMB_02801 | 77.9 | 0 |
| Slip_0903 | Acetate kinase | Swol_0768 | 82.12 | 0 | SWMB_02802 | 82.1 | 0 |
| Isomerization | | | | | | | |
| Slip_0757 | Cob(I)alamin adenosyltransferase | | | | | | |
| Slip_0758 | Isobutyryl-CoA mutase, N-terminal domain subunit | | | | | | |
| Slip_0759 | Isobutyryl-CoA mutase, C-terminal domain subunit (cobalamin B12-binding domain protein) | | | | | | |
| Slip_0760 | LAO/AO transport system ATPase, MeaB-like protein | | | | | | |
| Acyl-CoA synthetase | | | | | | | |
| Slip_1686 | Acyl-CoA synthetase (AMP-forming)/AMP-acid ligase II (for coenzyme A biosynthesis?) | Swol_1180 | 64.4 | 0 | SWMB_02710 | 64.4 | 0 |
| Slip_0583 | Acyl-CoA synthetase (AMP-forming)/AMP-acid ligase II | Swol_1180 | 31.1 | 6.00E-67 | SWMB_02710 | 30.6 | 5.00E-72 |
| Slip_0475 | Acyl-CoA synthetase (AMP-forming)/AMP-acid ligase II | | | | | | |

The gray bars indicate that there are no corresponding genes with significant similarity (<20%).

Table S6. *Syntrophomonas wolfei* subsp. *metylbutyratica* strain 4J5$^T$ genes encoding syntrophic electron flow.

| Locus tag in JCM14075 | Predicted product | *Syntrophomonas wolfei* Goettingen | | | *Syntrophothermus lipocalidus* TGB-C1 | | | Locus tag in TGB-C1 | Predicted product | Closely related strain | | |
|---|---|---|---|---|---|---|---|---|---|---|---|---|
| | | Locus Tag | %ID | e-value | Locus Tag | %ID | e-value | | | Locus Tag | %ID | e-value |
| Electron-bifurcating [FeFe] hydrogenase (H$^+$ + NADH + Fd$_{red}$ <=> H$_2$ +NAD$^+$ +Fd$_{ox}$) | | | | | | | | | | | | |
| SWMB_01221 | Fe-only hydrogenase | Swol_1017 | 98.1 | 0 | Slip_0125 | 81.4 | 0 | | | | | |
| SWMB_01222 | NADH:UQ oxidoreductase, 51 kDa subunit | Swol_1018 | 98.5 | 0 | Slip_0124 | 76.6 | 0 | | | | | |
| SWMB_01223 | NADH:UQ oxidoreductase, 24 kDa subunit | Swol_1019 | 98.0 | 8E-104 | Slip_0123 | 73.5 | 2E-72 | | | | | |
| Ferredoxin [FeFe] hydrogenase (H$^+$ + Fd$_{red}$ <=> H$_2$ + Fd$_{ox}$) | | | | | | | | | | | | |
| SWMB_00486 | NADP-reducing hydrogenase subunit HndC | Swol_2436 | 97.5 | 0 | Slip_0543 | 82.4 | 0 | | | | | |
| Periplasmic [FeFe] hydrogenase (MQ <=> H$_2$) | | | | | | | | | | | | |
| SWMB_01505 | Cytochrome b(N-terminal)/b6/petB | Swol_1927 | 95.6 | 3E-154 | Slip_1532 | 71.2 | 3E-120 | | | | | |
| SWMB_01506 | Periplasmic [Fe] hydrogenase small subunit precursor | Swol_1926 | 92.3 | 1E-79 | Slip_1533 | 68.7 | 5E-57 | | | | | |
| SWMB_01507 | Periplasmic [Fe] hydrogenase large subunit | Swol_1925 | 93.6 | 0 | Slip_1534 | 76.6 | 0 | | | | | |
| Periplasmic [NiFe] hydrogenase (MQ <=> H$_2$) | | | | | | | | | | | | |
| SWMB_01823 | Hydrogenase expression/formation protein HypE | | | | Slip_0392 | 64.6 | 1E-158 | | | | | |
| SWMB_01824 | Hydrogenase expression/formation protein HypD | | | | Slip_0391 | 67.4 | 1E-179 | | | | | |
| SWMB_01825 | Hydrogenase isoenzymes formation protein HypC | | | | Slip_0390 | 65.3 | 3E-32 | | | | | |
| SWMB_01826 | [NiFe] hydrogenase maturation protein HypF | | | | Slip_0389 | 63.9 | 0 | | | | | |
| SWMB_01827 | Hydrogenase 2 maturation protease | | | | Slip_0388 | 47.4 | 3E-45 | | | | | |
| SWMB_01828 | putative Ni/Fe-hydrogenase 1 B-type cytochrome subunit, hyaC | | | | Slip_0387 | 71.2 | 2E-107 | | | | | |
| SWMB_01829 | Periplasmic [NiFeSe] hydrogenase large subunit, hyaB | | | | Slip_0386 | 70.5 | 0 | | | | | |
| SWMB_01830 | Periplasmic [NiFe] hydrogenase large subunit precursor, hyaA | | | | Slip_0385 | 73.0 | 4E-18 | | | | | |
| SWMB_01831 | Periplasmic [NiFeSe] hydrogenase small subunit precursor | | | | Slip_0384 | 76.9 | 0 | | | | | |
| SWMB_01832 | Hydrogenase isoenzymes nickel incorporation protein HypB | | | | Slip_0384 | 59.7 | 1E-92 | | | | | |
| SWMB_01833 | Hydrogenase nickel incorporation protein HypA/HybF | | | | Slip_0383 | 49.1 | 2E-37 | | | | | |
| Energy conserving hydrogenase, Ech (H$^+$ + Fd$_{red}$ <=> H$_2$ + Fd$_{ox}$) | | | | | | | | | | *Thermacetogenium phaeum* PB | | |
| | | | | | | | | Slip_0655 | echA ech hydrogenase subunit A | Tph_c21360 | 41.6 | 3E-141 |
| | | | | | | | | Slip_0656 | echB ech hydrogenase subunit B | Tph_c21350 | 50.4 | 5E-87 |
| | | | | | | | | Slip_0657 | echC ech hydrogenase subunit C | Tph_c21340 | 66.2 | 4E-65 |
| | | | | | | | | Slip_0658 | echD ech hydrogenase subunit D | Tph_c21330 | 34.8 | 1E-15 |
| | | | | | | | | Slip_0659 | echE ech hydrogenase subunit E | Tph_c21320 | 54.4 | 5E-139 |
| | | | | | | | | Slip_0660 | NADH:ubiquinone reductase (H(+)-translocating) | Tph_c21310 | 41.5 | 1E-20 |
| Formate dehydrogenase O (Formate <=> MQ) | | | | | | | | | | | | |
| SWMB_01402 | Molybdopterin molybdenumtransferase | Swol_0802 | 95.5 | 0 | Slip_0566 | 52.8 | 7E-152 | | | | | |
| SWMB_01403 | Molybdopterin molybdenumtransferase | Swol_0801 | 95.5 | 0 | Slip_0567 | 56.9 | 0 | | | | | |
| SWMB_01404 | Formate dehydrogenase subunit alpha precursor, fdoG | Swol_0800 | 96.2 | 1E-152 | Slip_1434 | 77.5 | 9E-115 | | | | | |
| SWMB_01405 | Formate dehydrogenase subunit alpha precursor, fdoG | Swol_0799 | 96.9 | 0 | Slip_1434 | 70.4 | 0 | | | | | |
| SWMB_01406 | Formate dehydrogenase-N subunit beta, fdoH | Swol_0798 | 98.5 | 0 | Slip_1433 | 58.1 | 5E-121 | | | | | |
| SWMB_01407 | Formate dehydrogenase-N subunit gamma, fdoI | Swol_0797 | 99.6 | 5E-169 | Slip_1432 | 74.0 | 7E-125 | | | | | |
| SWMB_01408 | formate dehydrogenase accessory protein FdhE | Swol_0796 | 95.3 | 0 | Slip_1431 | 35.4 | 1E-67 | | | | | |
| SWMB_01533 | Formate dehydrogenase subunit alpha precursor, fdoG | Swol_1826 | 98.5 | 8E-145 | | | | | | | | |
| SWMB_01535 | Formate dehydrogenase, nitrate-inducible, major subunit precursor, fdoG | Swol_1825 | 92.5 | 0 | | | | | | | | |
| SWMB_01536 | Formate dehydrogenase-N subunit beta, fdoH | Swol_1824 | 93.3 | 0 | | | | | | | | |
| SWMB_01537 | Formate dehydrogenase-O subunit gamma, fdoI | Swol_1823 | 96.4 | 3E-158 | | | | | | | | |
| SWMB_01538 | formate dehydrogenase accessory protein FdhE | Swol_1822 | 93.3 | 4E-180 | | | | | | | | |
| SWMB_01539 | putative molybdopterin biosynthesis protein MoeA/LysR substrate binding-d[illegible] | Swol_1821 | 94.9 | 0 | | | | | | | | |
| Electron-bifurcating formate dehydrogenase (CO$_2$ + NADH + Fd$_{red}$ <=> Formate +NAD$^+$ +Fd$_{ox}$) | | | | | | | | | | | | |
| SWMB_02818 | NADH-quinone oxidoreductase subunit E | Swol_0783 | 96.0 | 4E-103 | | | | | | | | |
| SWMB_02819 | NADP-reducing hydrogenase subunit HndC | Swol_0784 | 98.3 | 0 | | | | | | | | |
| SWMB_02820 | Putative formate dehydrogenase/NADH-ubiquinone oxidoreductase-G | Swol_0785 | 97.2 | 0 | | | | | | | | |
| SWMB_02821 | Formate dehydrogenase H | Swol_0786 | 98.3 | 0 | | | | | | | | |
| SWMB_01527 | Putative formate dehydrogenase | Swol_1831 | 98.6 | 0 | | | | | | | | |
| SWMB_01528 | Formate dehydrogenase H | Swol_1830 | 98.0 | 0 | | | | | | | | |
| SWMB_01529 | NADP-reducing hydrogenase subunit HndA | Swol_1829 | 96.6 | 2E-103 | | | | | | | | |
| SWMB_01530 | NADP-reducing hydrogenase subunit HndC | Swol_1828 | 98.8 | 0 | | | | | | | | |
| SWMB_01228 | NADP-reducing hydrogenase subunit HndC | Swol_1024 | 98.5 | 0 | | | | | | | | |
| SWMB_01229 | NADP-reducing hydrogenase subunit HndA | Swol_1025 | 97.3 | 3E-106 | | | | | | | | |
| SWMB_01230 | Molybdopterin molybdenumtransferase | Swol_1026 | 94.5 | 0 | | | | | | | | |
| SWMB_01231 | bifunctional molybdenum cofactor biosynthesis protein MoaC/MogA | Swol_1027 | 96.5 | 0 | | | | | | | | |
| SWMB_01232 | Formate dehydrogenase H | Swol_1028 | 98.7 | 0 | | | | | | | | |
| SWMB_01233 | Putative formate dehydrogenase | Swol_1029 | 98.9 | 0 | | | | | | | | |
| NAD-dependent formate dehydrogenase (CO$_2$ + NADH <=> Formate +NAD$^+$) | | | | | | | | | | *S. wolfei* subsp. *metylbutyratica* JCM14075 | | |
| | | | | | | | | Slip_1031 | Formate dehydrogenase (NADP) alpha subunit | SWMB_02820 | 66.7 | 0 |
| | | | | | | | | Slip_1032 | Formate dehydrogenase (NADP) alpha subunit | SWMB_02821 | 69.4 | 0 |
| Flox-Hdr system | | | | | | | | | | | | |
| SWMB_01721 | Heterodisulfide reductase subunit C (HdrC) | Swol_0394 | 100.0 | 4E-139 | Slip_2075 | 63.4 | 2E-86 | | | | | |
| SWMB_01720 | Heterodisulfide reductase subunit B (HdrB) | Swol_0395 | 92.1 | 0 | Slip_2074 | 65.9 | 6E-142 | | | | | |
| SWMB_01719 | Heterodisulfide reductase subunit A (HdrA) | Swol_0396 | 99.0 | 5E-144 | | | | | | | | |
| SWMB_01718 | Heterodisulfide reductase subunit A (HdrA) | Swol_0397 | 98.9 | 0 | Slip_2073 | 75.5 | 0 | | | | | |
| SWMB_01717 | Hydrogenase subunit D (HydD) | Swol_0398-0399 | 97.1 | 4E-47 | Slip_2072 | 74.7 | 1E-34 | | | | | |
| SWMB_01716 | Hydrogenase subunit B (HydB) | Swol_0400 | 98.3 | 0 | Slip_2071 | 61.2 | 2E-143 | | | | | |
| SWMB_01715 | Flavoprotein oxidoreductase subunit A (FloxA) | Swol_0401 | 97.6 | 0 | Slip_2070 | 55.5 | 2E-134 | | | | | |
| SWMB_01714 | Flavoprotein oxidoreductase subunit B (FloxB) | Swol_0402 | 98.2 | 0 | Slip_2069 | 76.4 | 5E-162 | | | | | |
| Fix system (+AD) | | | | | | | | | | | | |
| SWMB_01042 | acyl-CoA dehydrogenase | Swol_2126 | 98.7 | 0 | Slip_0874 | 38.6 | 3E-87 | | | | | |
| SWMB_01043 | ETF-ubiquinone oxidoreductase (FixC) | Swol_2124 | 98.4 | 0 | Slip_0877 | 47.9 | 3E-127 | | | | | |
| SWMB_01044 | Ferredoxin (FixX) | Swol_2123 | 95.7 | 2E-63 | Slip_0878 | 42.4 | 4E-16 | | | | | |
| SWMB_01045 | Electron transfer flavoprotein β subunit (FixA) | Swol_2122 | 94.4 | 2E-148 | Slip_0875 | 35.5 | 2E-38 | | | | | |
| SWMB_01046 | Electron transfer flavoprotein α subunit (FixB) | Swol_2121 | 96.5 | 0 | Slip_0876 | 36.6 | 4E-58 | | | | | |
| Fix system (+AD/EH/CT) | | | | | | | | | | *S. wolfei* subsp. *metylbutyratica* JCM14075 | | |
| | | | | | | | | Slip_0455 | acyl-CoA dehydrogenase | | | |
| | | | | | | | | Slip_0456 | acyl-CoA dehydrogenase | SWMB_01042 | 63.1 | 2E-178 |
| | | | | | | | | Slip_0457 | ETF-ubiquinone oxidoreductase (FixC) | SWMB_01043 | 64.9 | 0 |
| | | | | | | | | Slip_0458 | Ferredoxin (FixX) | SWMB_01044 | 64.9 | 4E-41 |
| | | | | | | | | Slip_0459 | Electron transfer flavoprotein β subunit (FixA) | SWMB_01045 | 59.3 | 1E-96 |
| | | | | | | | | Slip_0460 | Electron transfer flavoprotein α subunit (FixB) | SWMB_01046 | 51.1 | 3E-101 |
| | | | | | | | | Slip_0461 | enoyl-CoA hydratase | | | |
| | | | | | | | | Slip_0462 | butyryl-CoA:acetate CoA-transferase | | | |
| Acyl-CoA dehydrogenase linked to ETF | | | | | | | | | | | | |
| SWMB_00118 | Acyl-CoA dehydrogenase | Swol_0268 | 99.2 | 0 | Slip_2109 | 40.1 | 1E-85 | | | | | |
| SWMB_00119 | fixA, etfB electron transfer flavoprotein beta subunit | Swol_0267 | 96.5 | 0 | Slip_2110 | 33.0 | 6E-36 | | | | | |
| SWMB_00120 | fixB, etfA electron transfer flavoprotein alpha subunit | Swol_0266 | 95.6 | 0 | Slip_2111 | 33.9 | 5E-43 | | | | | |
| Iron-sulfur-binding reductase linked to ETF | | | | | | | | | | | | |
| SWMB_02180 | Electron transfer flavoprotein small subunit | Swol_0696 | 97.2 | 2E-158 | Slip_1418 | 74.8 | 2E-121 | | | | | |
| SWMB_02181 | Electron transfer flavoprotein large subunit | Swol_0697 | 99.7 | 0 | Slip_1417 | 79.8 | 3E-176 | | | | | |
| SWMB_02182 | Fe-S oxidoreductase | Swol_0698 | 97.1 | 0 | Slip_1416 | 72.6 | 0 | | | | | |
| ATP synthase | | | | | | | | | | | | |
| SWMB_00535 | Putative F0F1-ATPase subunit (ATPase_gene1) | Swol_2389 | 98.9 | 4E-58 | Slip_2290 | 51.6 | 2E-15 | | | | | |
| SWMB_00536 | F-ATPase subunit 6 | Swol_2388 | 98.7 | 3E-162 | Slip_2288 | 57.3 | 1E-93 | | | | | |
| SWMB_00537 | Lipid-binding protein | Swol_2387 | 98.6 | 3E-41 | Slip_2287 | 77.1 | 1E-23 | | | | | |
| SWMB_00538 | F-type ATPase subunit b | Swol_2386 | 98.8 | 4E-122 | Slip_2286 | 60.8 | 1E-83 | | | | | |
| SWMB_00539 | F-type ATPase subunit delta | Swol_2385 | 97.3 | 1E-128 | Slip_2285 | 51.1 | 4E-64 | | | | | |
| SWMB_00540 | ATP synthase subunit alpha | Swol_2384 | 99.6 | 0 | Slip_2284 | 81.3 | 0 | | | | | |
| SWMB_00541 | F-ATPase gamma subunit | Swol_2383 | 97.0 | 0 | Slip_2283 | 69.9 | 6E-147 | | | | | |
| SWMB_00542 | ATP synthase subunit beta | Swol_2382 | 100.0 | 0 | Slip_2282 | 81.7 | 0 | | | | | |
| SWMB_00543 | F-ATPase epsilon subunit | Swol_2381 | 100.0 | 1E-96 | Slip_2281 | 71.6 | 1E-67 | | | | | |

The gray bars indicate that there are no corresponding genes with significant similarity (<20%).

Table S7. *Syntrophothermus lipocalidus* strain TGB-C1$^T$ genes encoding nitrogenase complex

| Locus tag in TGB-C1 | Predicted product | *Thermacetogenium phaeum* PB | | | *Clostridium butyricum* CWBI1009 | | | *Syntrophobacter fumaroxidans* MPOB | | |
|---|---|---|---|---|---|---|---|---|---|---|
| | | Locus Tag | %ID | e-value | Locus Tag | %ID | e-value | Locus Tag | %ID | e-value |
| Slip_2118 | nitrogen regulatory protein P-II family | Tph_c08760 | 81.3 | 2E-50 | Ga0077125_11736 | 41.1 | 1E-26 | Sfum_0726 | 42.0 | 1E-25 |
| Slip_2119 | ammonium transporter | Tph_c08750 | 88.8 | 0 | Ga0077125_11735 | 44.7 | 8E-132 | Sfum_0727 | 35.1 | 3E-58 |
| Slip_2120 | glutamine amidotransferase class-I | Tph_c08740 | 81.4 | 7E-146 | Ga0077125_10774 | 28.9 | 3.E-08 | Sfum_0596 | 41.6 | 2E-39 |
| Slip_2121 | modC molybdate transport system ATP-binding protein | Tph_c26420 | 50.9 | 2E-64 | Ga0077125_115825 | 44.6 | 3E-53 | Sfum_3696 | 47.8 | 5E-54 |
| Slip_2122 | modB molybdate transport system permease protein | Tph_c26410 | 57.8 | 5E-87 | Ga0077125_115824 | 48.6 | 2E-65 | Sfum_3698 | 46.4 | 1E-63 |
| Slip_2123 | modA molybdate transport system substrate-binding protein | Tph_c26400 | 49.8 | 5E-74 | Ga0077125_115823 | 42.0 | 1E-56 | Sfum_3697 | 40.9 | 2E-53 |
| | nifV homocitrate synthase | Tph_c08720 | | | | | | | | |
| Slip_2124 | nifB nitrogen fixation protein NifB | Tph_c08710 | 82.6 | 5E-167 | Ga0077125_115813 | 41.0 | 2E-66 | Sfum_1011 | 42.8 | 4E-69 |
| | Nitrogenase FeMo-cofactor scaffold and assembly protein NifN | | | | | | | Sfum_1012 | | |
| Slip_2125 | nifE nitrogenase molybdenum-cofactor synthesis protein | Tph_c08700 | 93.3 | 0 | Ga0077125_115814 | 36.7 | 2E-91 | Sfum_1013 | 37.3 | 4E-84 |
| | ferredoxin, 2fe-2s | | | | | | | Sfum_1014 | | |
| Slip_2126 | nifK nitrogenase molybdenum-iron protein beta chain | Tph_c08690 | 95.1 | 0 | Ga0077125_115815 | 38.5 | 2E-101 | Sfum_1015 | 39.5 | 4E-105 |
| Slip_2127 | nifD nitrogenase molybdenum-iron protein alpha chain | Tph_c08680 | 94.2 | 0 | Ga0077125_115816 | 34.7 | 6E-97 | Sfum_1016 | 36.3 | 3E-103 |
| Slip_2128 | nifI2 nitrogen regulatory protein PII 2 | Tph_c08670 | 95.9 | 1E-81 | Ga0077125_115817 | 39.5 | 1E-24 | Sfum_1017 | 44.0 | 2E-23 |
| Slip_2129 | nifI1 nitrogen regulatory protein PII 1 | Tph_c08660 | 96.2 | 2E-70 | Ga0077125_115818 | 55.1 | 5E-34 | Sfum_1018 | 54.7 | 2E-34 |
| Slip_2130 | nifH nitrogenase iron protein NifH | Tph_c08650 | 98.2 | 0 | Ga0077125_115819 | 68.1 | 5E-139 | Sfum_1019 | 67.8 | 5E-136 |

The gray bars indicate that there are no corresponding genes with significant similarity (<20%).

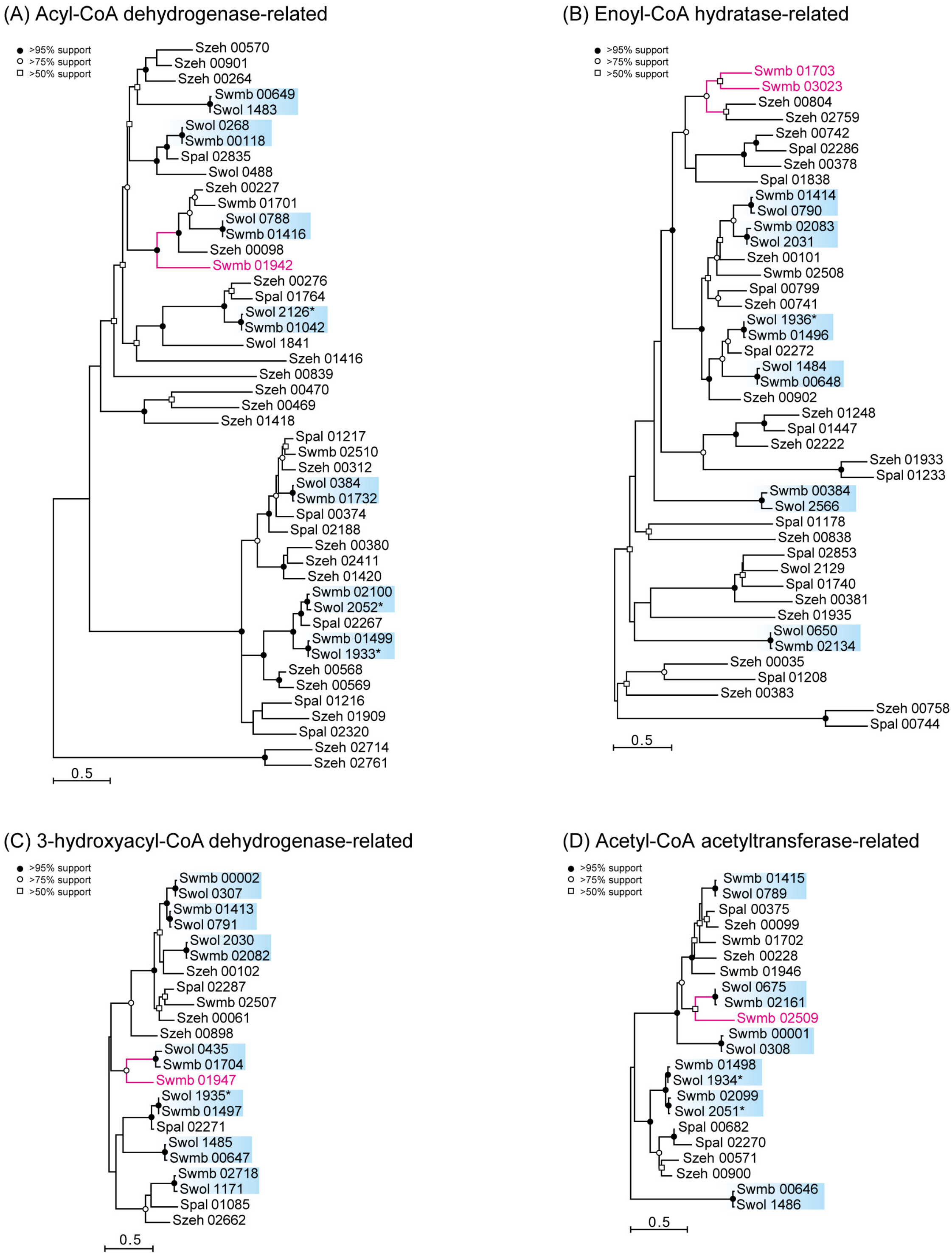


**Fig. S1.** Maximum likelihood trees of (A) acyl-CoA dehydrogenase, (B) enoyl-CoA dehydratase, (C) 3-hydroxyacyl-CoA dehydrogenase, and (D) acetyl-CoA acetyltransferase of genome-sequenced mesophilic *Syntrophomonadaceae* strains: *Syntrophomonas wolfei* subsp.

*methylbutyratica* strain 4J5 (locus tag: Swmb), *Syntrophomonas wolfei* subsp. *wolfei* strain Göttingen (Swol), *Syntrophomonas palmitatica* strain JCM 14374 (Spal), and *Syntrophomonas zehnderi* strain OL-4 (Szeh). The topology of the tree was estimated by bootstrap analysis with 1,000 replicates. Bars indicate the change per amino acid site. Locus tags of *Syntrophomonas wolfei* subsp. *wolfei* strain Göttingen with asterisk indicate significant expressed enzymes based on proteomic study (9). Cluster with blue color indicates a set of homologous gene of the strain 4J5 and Göttingen that has amino acid sequence identity range 87.4%–99.7%.

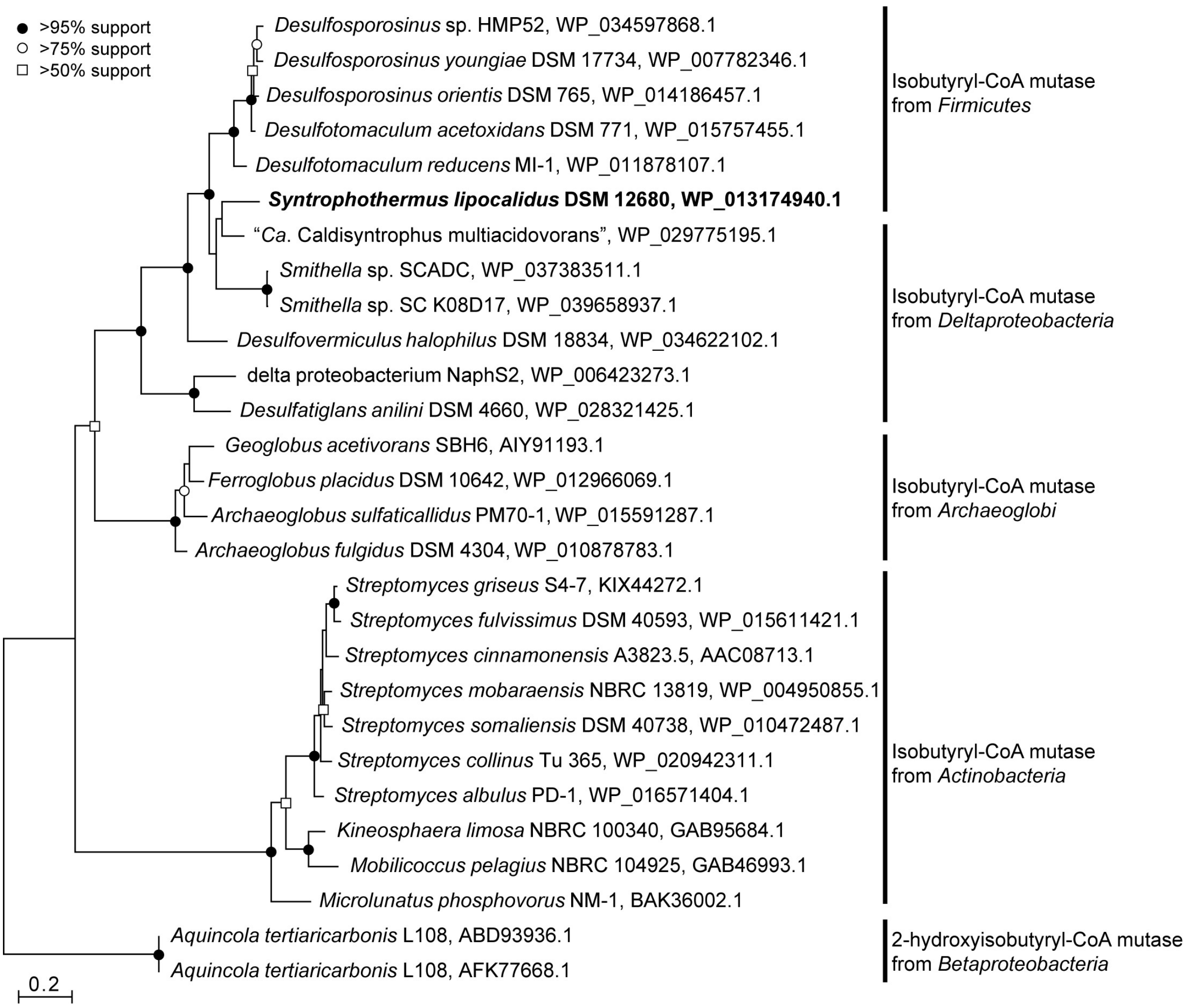


**Fig. S2.** A maximum likelihood tree of isobutyryl-CoA mutase. The topology of the tree was estimated by bootstrap analysis with 1,000 replicates. Bar indicates 0.2 change per amino acid site.

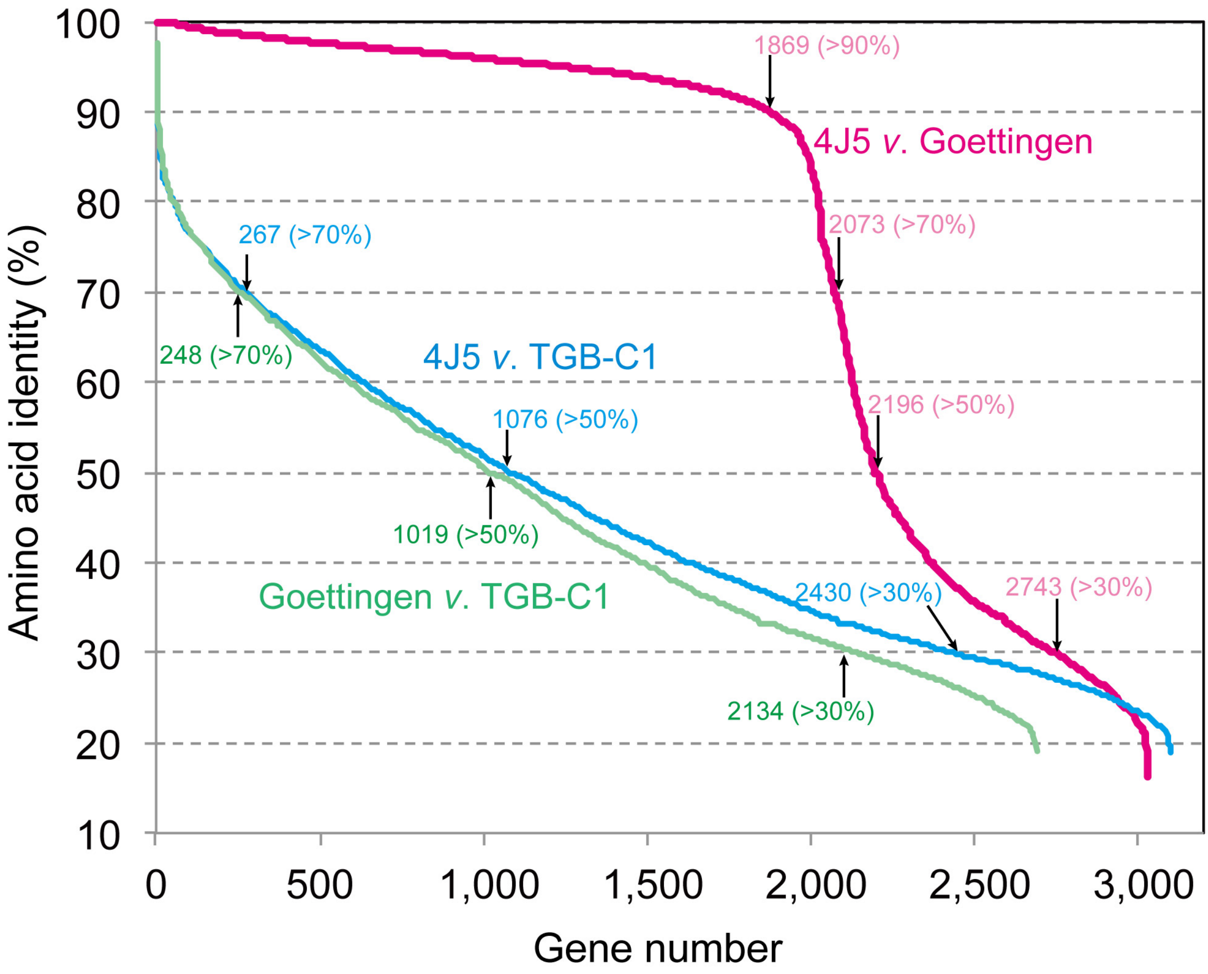


**Fig. S3.** Comparison of amino acid identities between the genome of "*Syntrophomonas wolfei* subsp. *methylbutyratica*" strain 4J5, *Syntrophomonas wolfei* subsp. *wolfei* strain Göttingen, and *Syntrophothermus lipocalidus* strain TGB-C1.

**Supplemental material**. Amino acid sequences of annotated proteins within the genome of *Syntrophomonas wolfei* subsp. *metylbutyratica* strain 4J5.

## Supplemental references


1. Amos, D.A., and M.J. McInerney. 1989. Poly-β-hydroxyalkanoate in *Syntrophomonas wolfei*. Arch. Microbiol. 152:172-177.
2. Beaty, P.S., and M.J. McInerney. 1989. Effects of organic-acid anions on the growth and metabolism of *Syntrophomonas wolfei* in pure culture and in defined consortia. Appl. Environ. Microbiol. 55:977-983.
3. Djao, O.D.N., X.J. Zhang, S. Lucas*, et al.* 2010. Complete genome sequence of *Syntrophothermus lipocalidus* type strain (TGB-C1(T)). Stand. Genomic Sci. 3:267-275.
4. Felsenstein, J. 1985. Confidence-limits on phylogenies - an approach using the bootstrap. Evolution. 39:783-791.
5. Felsenstein, J. 1981. Evolutionary trees from DNA sequences: a maximum likelihood approach. J. Mol. Evol. 17:368-376.
6. Grissa, I., G. Vergnaud, and C. Pourcel. 2007. The CRISPRdb database and tools to display CRISPRs and to generate dictionaries of spacers and repeats. BMC Bioinformatics. 8:172.
7. Jones, D.T., W.R. Taylor, and J.M. Thornton. 1992. The rapid generation of mutation data matrices from protein sequences. Comput Appl Biosci. 8:275-82.
8. McInerney, M.J., and N.Q. Wofford. 1992. Enzymes involved in crotonate metabolism in *Syntrophomonas wolfei*. Arch. Microbiol. 158:344-349.
9. Schmidt, A., N. Muller, B. Schink, and D. Schleheck. 2013. A proteomic view at the biochemistry of syntrophic butyrate oxidation in *Syntrophomonas wolfei*. PLoS One. 8:e56905.
10. Seemann, T. 2014. Prokka: rapid prokaryotic genome annotation. Bioinformatics. 30:2068-2069.

11. Sieber, J.R., D.R. Sims, C. Han, *et al.* 2010. The genome of *Syntrophomonas wolfei*: new insights into syntrophic metabolism and biohydrogen production. Environ. Microbiol. 12:2289-2301.

12. Sorek, R., V. Kunin, and P. Hugenholtz. 2008. CRISPR--a widespread system that provides acquired resistance against phages in bacteria and archaea. Nat. Rev. Microbiol. 6:181-186.

13. Sorek, R., C.M. Lawrence, and B. Wiedenheft. 2013. CRISPR-mediated adaptive immune systems in bacteria and archaea. Annu. Rev. Biochem. 82:237-266.

14. Takahashi, K., and M. Nei. 2000. Efficiencies of fast algorithms of phylogenetic inference under the criteria of maximum parsimony, minimum evolution, and maximum likelihood when a large number of sequences are used. Mol. Biol. Evol. 17:1251-8.

15. Tamura, K., G. Stecher, D. Peterson, A. Filipski, and S. Kumar. 2013. MEGA6: Molecular Evolutionary Genetics Analysis version 6.0. Mol. Biol. Evol. 30:2725-2729.

16. Wu, C., X. Dong, and X. Liu. 2007. *Syntrophomonas wolfei* subsp. *methylbutyratica* subsp. nov., and assignment of *Syntrophomonas wolfei* subsp. *saponavida* to *Syntrophomonas saponavida* sp. nov. comb. nov. Syst. Appl. Microbiol. 30:376-380.

**Supplemental material . Amino acid sequences of annotated proteins within the genome of " Syntrophomonas wolfei subsp. metylbutyratica strain 4J5.**

>Syntrophomonas_wolfei_subsp.metylbutyratica_JCM14075T_00001_Acetyl-CoA_acetyltransferase
MGLNEVVMVSACRTPIGKFLGQFKDVPAKDLAVTAGKEAIKRAGIPAEIIDEIVMGQVYQGLQKSLPARIVGLACGLPERSNACVVNQNCTSSMRALEIASRNIMLGVTDIALIVGVENMTQAPYMVPS[illegible]
NASGINDGASAAVIMSKQKAAELGITPLMKLVASVGVGVDPQIMGIGPAEAIPVALEKAGWKFDDVDYWELHEAFAPQFIGCKIRLKEKYGMDLSMDKVNHNGSGIALGHPIGSTGLRIVVSLYYELER[illegible]
>Syntrophomonas_wolfei_subsp.metylbutyratica_JCM14075T_00002_putative_3-hydroxybutyryl-CoA_dehydrogenase
MAINKIGMLGAGTMGQGIAWALASAGKKVVLYDIKKEFADRAISSIGKSLARAEEKGKAAPGTKDTIVGNIFGTDKFEDLKDVDFIVEGVFENMDVKKDVYTKLDALLAPEVIIATNTSSLSITEIAAITKR[illegible]
ETGDPKYRPSGLLKQYVRAGWLGMKTKKGFYNY*
>Syntrophomonas_wolfei_subsp.metylbutyratica_JCM14075T_00003_hypothetical_protein
MTLLQKTLFVAWGCINMQSERWRSMDATPFTLRVRLVFHPTGVTIKVASHGREIGAVPLEPRDKPLVLPMRSALSGTTDAPFGRY*
>Syntrophomonas_wolfei_subsp.metylbutyratica_JCM14075T_00004_(S)-limonene_6-monooxygenase
MSKSVDSFNLYKVSEAILANPLMACIVVDEHGIIRFINQAYLEWLHKDKDEVMGKHILEVTPHSKLPEVLRSGKSDLVDLWSVNDRETIITRYPLLKDEQVIGAVGQTIFLDKAGAKILMKKLQTMEKEY[illegible]
YREEFRQLYQARWQFDDLIGVNPEFTMVKAMAQQLAHTRSTILITGESGTGKELLAQSIHNQGGQEGPFVRVNCAALPENLLESELFGYEEGAFTGAKKGGKPGKFELAKGGTIFLDEIGDMPIGMQ[illegible]
LASLKQATRLGVQHFPSLTGEALPPAVNENEHSTLNDSVQRLEKELIEQMLKSTNYNKSQTAKELGLNKSVLYSKMRKYGLFN*
>Syntrophomonas_wolfei_subsp.metylbutyratica_JCM14075T_00005_precorrin-6B_methylase
VEQTMFTNEVILSWLQYFSRNAPINLAKLKMLDITGKNKNLIPTVMSNRAVLVFTDAGHPDIFYDMWNAELGDCDIWYNEGSDPSGEIKHDKVSDMINRGINASAAMLILNPDAATNYHIGMENSRFS[illegible]
DFEVLMSLPALFRENGLERTEAIHVAVSRVNSKKMMEPFPAPWLISGKRVKG*
>Syntrophomonas_wolfei_subsp.metylbutyratica_JCM14075T_00006_Pyruvate_synthase_subunit_PorB
MSTVYNLKREVEKPIRLTGGHRLCAGCSTGVVVSGVLRALDREDRAVVVNATSCLQVSTFMYPYTAYMDSYIHSAFENSAATCSGVEAAYNALKRRGKVEGTVKFITFAGDGGTYDIGFQSLSGAMER[illegible]
CVWPLYEVEEGVWRLSYMPKKKLPVEDFLRPQGRFRHMFRQGNEWMIEEAQSYVDQKWERLLEHSGA*
>Syntrophomonas_wolfei_subsp.metylbutyratica_JCM14075T_00007_Pyruvate_synthase_subunit_PorA
MSSRLSGNEAVAYAMKQINPDVMGAFPITPSTEIPEYFAAYVANGEVNTEYVAVESEHSSMSVCIAAQAAGARAVSATSSCGLALMYELLYVAASCRLPITLACVNRALSGPININHDYSDSMGARDSGW[illegible]
FEEYRMEDAQLALVLIGSTAGTAKAAIDKLREDGVKAGLVKIRVFRPFPGEELARALAGTKAVAVMDKSEGFSANGGPLFAETCSALYNLKERPYLINIVYGLGGRDVRTEDIEKVFGRLADIAVTGETH[illegible]
>Syntrophomonas_wolfei_subsp.metylbutyratica_JCM14075T_00008_Pyruvic-ferredoxin_oxidoreductase_subunit_delta
MIRAKDINELTPWQDLTPGGEIYEAGTARAFNTGTWRTNTPVFHEEACKHCMLCVPFCPDSAIPVKDGKRLDFDYMYCKGCGVCENVCPFPAITMMIGGVAR*
>Syntrophomonas_wolfei_subsp.metylbutyratica_JCM14075T_00009_Pyruvate_synthase_subunit_PorC
[illegible]
>Syntrophomonas_wolfei_subsp.metylbutyratica_JCM14075T_00011_Glutaredoxin-3
[illegible]
>Syntrophomonas_wolfei_subsp.metylbutyratica_JCM14075T_00012_hypothetical_protein
[illegible]
>Syntrophomonas_wolfei_subsp.metylbutyratica_JCM14075T_00013_Fatty_acid-binding_protein
[illegible]
>Syntrophomonas_wolfei_subsp.metylbutyratica_JCM14075T_00014_hypothetical_protein
[illegible]
>Syntrophomonas_wolfei_subsp.metylbutyratica_JCM14075T_00015_hypothetical_protein
[illegible]
>Syntrophomonas_wolfei_subsp.metylbutyratica_JCM14075T_00016_Endonuclease_YhcR_precursor
[illegible]
>Syntrophomonas_wolfei_subsp.metylbutyratica_JCM14075T_00017_hypothetical_protein
[illegible]
>Syntrophomonas_wolfei_subsp.metylbutyratica_JCM14075T_00018_hypothetical_protein
[illegible]
>Syntrophomonas_wolfei_subsp.metylbutyratica_JCM14075T_00019_Endo-1,4-beta-xylanase_A_precursor
[illegible]
>Syntrophomonas_wolfei_subsp.metylbutyratica_JCM14075T_00020_Methylphosphotriester-DNA--protein-cysteine_S-methyltransferase
[illegible]
>Syntrophomonas_wolfei_subsp.metylbutyratica_JCM14075T_00021_Penicillin-binding_protein_E
[illegible]
>Syntrophomonas_wolfei_subsp.metylbutyratica_JCM14075T_00022_Endo-1,4-beta-xylanase_A_precursor
[illegible]
>Syntrophomonas_wolfei_subsp.metylbutyratica_JCM14075T_00023_hypothetical_protein
[illegible]
>Syntrophomonas_wolfei_subsp.metylbutyratica_JCM14075T_00024_ATP-dependent_transcriptional_activator_MalT
[illegible]
>Syntrophomonas_wolfei_subsp.metylbutyratica_JCM14075T_00025_Penicillin-binding_protein_E
[illegible]
>Syntrophomonas_wolfei_subsp.metylbutyratica_JCM14075T_00026_hypothetical_protein
[illegible]
>Syntrophomonas_wolfei_subsp.metylbutyratica_JCM14075T_00027_multifunctional_acyl-CoA_thioesterase_I_and_protease_I_and_lysophospholipase_L1
[illegible]
>Syntrophomonas_wolfei_subsp.metylbutyratica_JCM14075T_00028_putative_protein_encoded_in_hypervariable_junctions_of_pilus_gene_clusters
[illegible]
>Syntrophomonas_wolfei_subsp.metylbutyratica_JCM14075T_00029_hypothetical_protein
[illegible]
>Syntrophomonas_wolfei_subsp.metylbutyratica_JCM14075T_00030_hypothetical_protein
[illegible]
>Syntrophomonas_wolfei_subsp.metylbutyratica_JCM14075T_00031_hypothetical_protein
[illegible]
>Syntrophomonas_wolfei_subsp.metylbutyratica_JCM14075T_00032_CAAX_amino_terminal_protease_self-_immunity
[illegible]
>Syntrophomonas_wolfei_subsp.metylbutyratica_JCM14075T_00033_MarR_family_protein
[illegible]
>Syntrophomonas_wolfei_subsp.metylbutyratica_JCM14075T_00034_hypothetical_protein
[illegible]
>Syntrophomonas_wolfei_subsp.metylbutyratica_JCM14075T_00035_Endoglucanase_precursor
[illegible]
>Syntrophomonas_wolfei_subsp.metylbutyratica_JCM14075T_00036_hypothetical_protein
[illegible]
>Syntrophomonas_wolfei_subsp.metylbutyratica_JCM14075T_00037_Bacillibactin_transport_regulator
[illegible]
>Syntrophomonas_wolfei_subsp.metylbutyratica_JCM14075T_00038_hypothetical_protein
[illegible]
>Syntrophomonas_wolfei_subsp.metylbutyratica_JCM14075T_00039_hypothetical_protein
[illegible]
>Syntrophomonas_wolfei_subsp.metylbutyratica_JCM14075T_00040_ABC_transporter_permease_YtrF_precursor
[illegible]
>Syntrophomonas_wolfei_subsp.metylbutyratica_JCM14075T_00041_Lipoprotein-releasing_system_ATP-binding_protein_LolD
[illegible]
>Syntrophomonas_wolfei_subsp.metylbutyratica_JCM14075T_00042_Signal_transduction_histidine-protein_kinase_BaeS
[illegible]
>Syntrophomonas_wolfei_subsp.metylbutyratica_JCM14075T_00043_Sensory_transduction_protein_regX3
[illegible]
>Syntrophomonas_wolfei_subsp.metylbutyratica_JCM14075T_00044_DNA_binding_domain,_excisionase_family
[illegible]
>Syntrophomonas_wolfei_subsp.metylbutyratica_JCM14075T_00045_hypothetical_protein
[illegible]
>Syntrophomonas_wolfei_subsp.metylbutyratica_JCM14075T_00046_Methicillin_resistance_mecR1_protein
[illegible]
>Syntrophomonas_wolfei_subsp.metylbutyratica_JCM14075T_00047_hypothetical_protein
[illegible]
>Syntrophomonas_wolfei_subsp.metylbutyratica_JCM14075T_00048_hypothetical_protein
[illegible]
>Syntrophomonas_wolfei_subsp.metylbutyratica_JCM14075T_00049_hypothetical_protein
[illegible]
>Syntrophomonas_wolfei_subsp.metylbutyratica_JCM14075T_00050_putative_S-adenosyl-L-methionine-dependent_methyltransferase_TehB
[illegible]
>Syntrophomonas_wolfei_subsp.metylbutyratica_JCM14075T_00051_hypothetical_protein
[illegible]
>Syntrophomonas_wolfei_subsp.metylbutyratica_JCM14075T_00052_Piwi_domain_protein
[illegible]
>Syntrophomonas_wolfei_subsp.metylbutyratica_JCM14075T_00053_type_III_restriction-modification_system_StyLTI_enzyme_res
[illegible]
>Syntrophomonas_wolfei_subsp.metylbutyratica_JCM14075T_00054_putative_methyltransferase
[illegible]
>Syntrophomonas_wolfei_subsp.metylbutyratica_JCM14075T_00055_hypothetical_protein
[illegible]
>Syntrophomonas_wolfei_subsp.metylbutyratica_JCM14075T_00056_ATP-dependent_helicase_HepA
[illegible]
>Syntrophomonas_wolfei_subsp.metylbutyratica_JCM14075T_00057_DNA_binding_domain,_excisionase_family
[illegible]
>Syntrophomonas_wolfei_subsp.metylbutyratica_JCM14075T_00058_hypothetical_protein
[illegible]
>Syntrophomonas_wolfei_subsp.metylbutyratica_JCM14075T_00059_putative_NTPase_(NACHT_family)
[illegible]
>Syntrophomonas_wolfei_subsp.metylbutyratica_JCM14075T_00060_hypothetical_protein
[illegible]
>Syntrophomonas_wolfei_subsp.metylbutyratica_JCM14075T_00061_RNA_polymerase_sigma_factor_sigma-70_family
[illegible]
>Syntrophomonas_wolfei_subsp.metylbutyratica_JCM14075T_00062_hypothetical_protein
[illegible]
>Syntrophomonas_wolfei_subsp.metylbutyratica_JCM14075T_00063_hypothetical_protein
[illegible]
>Syntrophomonas_wolfei_subsp.metylbutyratica_JCM14075T_00064_PD-(D/E)XK_nuclease_superfamily_protein
[illegible]
>Syntrophomonas_wolfei_subsp.metylbutyratica_JCM14075T_00065_hypothetical_protein
[illegible]
>Syntrophomonas_wolfei_subsp.metylbutyratica_JCM14075T_00066_DNA_polymerase_I_thermostable
[illegible]
>Syntrophomonas_wolfei_subsp.metylbutyratica_JCM14075T_00067_hypothetical_protein
[illegible]
>Syntrophomonas_wolfei_subsp.metylbutyratica_JCM14075T_00068_putative_phage-encoded_protein
[illegible]
>Syntrophomonas_wolfei_subsp.metylbutyratica_JCM14075T_00069_hypothetical_protein
[illegible]
>Syntrophomonas_wolfei_subsp.metylbutyratica_JCM14075T_00070_putative_P-loop_ATPase

MKISYGNSRMDKKWKNSDISWEDFCMRVSSTVRTTETVEEYRKLKKGQQDAIKDVGGYVAGHLREGRRKKGFVLCRSMILLDMDYGTPGIWDAVIMKYSFKCCAYSTHKHTPEKPRIRLAIPLSRDVSEAEYPAVARVVAKDIGIDLFDDTTYEAHRLMYWPSTSMNGEFWFQKKDGSNLDPDIYLARYDDWQDESTWPRSSRQSEVARSGVAEAGNPLTKPGIIGAFNRAYTVEDAIDTFLSD
VYEPSAMNGRYDYIPADSSAGVVIYDSAFAYSHHATDPVCGKLLNAFDLVRLHKFRSLDDKSAEDTPVNKLPSYKAMSELAVNDERVKLLLAEERRTQASAEFAVVDTDWEKKLEYEPRSTVLKNSLGN[illegible]
KTLAAAVARIYQPGIKFDSMLVLNGRTDLGKSTFFARLAGEWFSDSLNFTDMGKGKDAAEKIQGVWIVEIPELAGLSKMDVNNIKGFLSRQDDQYRPSYGRTVESHPRQCIIVGSTNAENAGFLRDTT[illegible]
MEIWCECFGNDRGKFERQADSYKIKLIMQKIGGWVYSGQKKKIKGYGAQYVWVRTIDGTENLNHGTS*
>Syntrophomonas_wolfei_subsp.metylbutyratica_JCM14075T_00071_VRR-NUC_domain_protein
MREKTIEIKLTNTVKSMGGVALKLISPGFDGVPDRLVLLPHGKLAFVELKAPGKRLRPLQEKRKRQLEALGYLVFCIDGAEQIGEMIDEILSS*
>Syntrophomonas_wolfei_subsp.metylbutyratica_JCM14075T_00072_hypothetical_protein
MKYCPHDYQTYATNFILQHPISAVFLDMGLGKSVITLTAIFDLVLDSFLIRKVLVIAPLRVARDTWPFEIEKWDHLKGMTYSVAIGTEQERKFALMRNVDVYIINRENVDWLVNKSGLPFDFDMVVVDE[illegible]
KEQKAYDTIKRDLILSLEGRDIDAANAAALSGKLLQMANGAVYDENGGVAHLHDRKLDALEDIIEAANGKPVLVAYWFKHDLERILYRFPAVKLDSAASIRRWNDGKIPLAVIHPASAGHGLNLQAGG[illegible]
>Syntrophomonas_wolfei_subsp.metylbutyratica_JCM14075T_00073_phage_transcriptional_regulator,_ArpU_family
MIALKYINKNAATIAAIRDYNNMRFIINNTPEEIKSVYEKMIAPRTPKLSRMPSARNPQAGADKLAAQIDKLDILRERYSQAIEYMAWFEPAWSSLTDTEQHILSEFYMGDNQKSGATYRLMSELSYSESHIE[illegible]
>Syntrophomonas_wolfei_subsp.metylbutyratica_JCM14075T_00074_HNH_endonuclease
MPFKPKRPCSYPGCPKLTHDRFCEEHQKLTDKQYEKYQRDPIVKKRYNRTWKRIRDRYIAAHPLCEKCASLGRVTPAQEVHHIKPLSQGGTNDVDNLMSLCTSCHSEITAREGGRWRK*
>Syntrophomonas_wolfei_subsp.metylbutyratica_JCM14075T_00075_Phage_terminase,_small_subunit
VAKDGTNRGGRRVRAGDKPQPLADKISAGKAAKVLAAPDLHPESMLEASDLDDAADLYGEDMPTPSDYLSARQKDGKPLGADVLFKETWKWLKERGCEKFVNPRLIEAYAQAFTRYIQCEEAISTY[illegible]
>Syntrophomonas_wolfei_subsp.metylbutyratica_JCM14075T_00076_DNA_adenine_methyltransferase_YhdJ
MNTTERFEKVNIDRLVPYARNARTHSKEQILQLRASLREFGFVNPVIVDKDLNVIAGHGRILAAKEEGIAEIPCVFAEHLTEAQKRAYIIADNRLALNAGWDAEMLSVELADLQATDFDVSLLGFDDAEL[illegible]
ADTEGLNFRKAFLEAGFYLSGTCIWKKQSLVLGRSPYQWQHEPILFGWKKAGKHAWYSDRKQSTIWEFDKPRKNADHPTMKPVPLIAYPILNSSMTGCIVLDPFGGSGSTLIACEQTGRICYTVELDEK[illegible]
>Syntrophomonas_wolfei_subsp.metylbutyratica_JCM14075T_00077_hypothetical_protein
[illegible]
>Syntrophomonas_wolfei_subsp.metylbutyratica_JCM14075T_00078_hypothetical_protein
[illegible]
>Syntrophomonas_wolfei_subsp.metylbutyratica_JCM14075T_00079_hypothetical_protein
[illegible]
>Syntrophomonas_wolfei_subsp.metylbutyratica_JCM14075T_00080_Phage_terminase-like_protein,_large_subunit
[illegible]
>Syntrophomonas_wolfei_subsp.metylbutyratica_JCM14075T_00081_hypothetical_protein
[illegible]
>Syntrophomonas_wolfei_subsp.metylbutyratica_JCM14075T_00082_phage_portal_protein,_HK97_family
[illegible]
>Syntrophomonas_wolfei_subsp.metylbutyratica_JCM14075T_00083_ATP-dependent_Clp_protease_proteolytic_subunit_1
[illegible]
>Syntrophomonas_wolfei_subsp.metylbutyratica_JCM14075T_00084_phage_major_capsid_protein,_HK97_family
[illegible]
>Syntrophomonas_wolfei_subsp.metylbutyratica_JCM14075T_00085_hypothetical_protein
[illegible]
>Syntrophomonas_wolfei_subsp.metylbutyratica_JCM14075T_00086_putative_phage_protein_(possible_DNA_packaging)
[illegible]
>Syntrophomonas_wolfei_subsp.metylbutyratica_JCM14075T_00087_putative_phage_head-tail_adaptor
[illegible]
>Syntrophomonas_wolfei_subsp.metylbutyratica_JCM14075T_00088_phage_protein,_HK97_gp10_family
[illegible]
>Syntrophomonas_wolfei_subsp.metylbutyratica_JCM14075T_00089_hypothetical_protein
[illegible]
>Syntrophomonas_wolfei_subsp.metylbutyratica_JCM14075T_00090_phage_major_tail_protein,_phi13_family
[illegible]
>Syntrophomonas_wolfei_subsp.metylbutyratica_JCM14075T_00091_hypothetical_protein
[illegible]
>Syntrophomonas_wolfei_subsp.metylbutyratica_JCM14075T_00092_Phage-related_minor_tail_protein
[illegible]
>Syntrophomonas_wolfei_subsp.metylbutyratica_JCM14075T_00093_hypothetical_protein
[illegible]
>Syntrophomonas_wolfei_subsp.metylbutyratica_JCM14075T_00094_hypothetical_protein
[illegible]
>Syntrophomonas_wolfei_subsp.metylbutyratica_JCM14075T_00095_hypothetical_protein
[illegible]
>Syntrophomonas_wolfei_subsp.metylbutyratica_JCM14075T_00096_hypothetical_protein
[illegible]
>Syntrophomonas_wolfei_subsp.metylbutyratica_JCM14075T_00097_hypothetical_protein
[illegible]
>Syntrophomonas_wolfei_subsp.metylbutyratica_JCM14075T_00098_hypothetical_protein
[illegible]
>Syntrophomonas_wolfei_subsp.metylbutyratica_JCM14075T_00099_hypothetical_protein
[illegible]
>Syntrophomonas_wolfei_subsp.metylbutyratica_JCM14075T_00100_toxin_secretion/phage_lysis_holin
[illegible]
>Syntrophomonas_wolfei_subsp.metylbutyratica_JCM14075T_00101_Sporulation-specific_N-acetylmuramoyl-L-alanine_amidase
[illegible]
>Syntrophomonas_wolfei_subsp.metylbutyratica_JCM14075T_00102_hypothetical_protein
[illegible]
>Syntrophomonas_wolfei_subsp.metylbutyratica_JCM14075T_00103_Transposon_Tn3_resolvase
[illegible]
>Syntrophomonas_wolfei_subsp.metylbutyratica_JCM14075T_00104_Transposon_resolvase
[illegible]
>Syntrophomonas_wolfei_subsp.metylbutyratica_JCM14075T_00105_SszA-binding_protein
[illegible]
>Syntrophomonas_wolfei_subsp.metylbutyratica_JCM14075T_00106_Ribonuclease_R
[illegible]
>Syntrophomonas_wolfei_subsp.metylbutyratica_JCM14075T_00107_phosphodiesterase
[illegible]
>Syntrophomonas_wolfei_subsp.metylbutyratica_JCM14075T_00108_Thermostable_monoacylglycerol_lipase
[illegible]
>Syntrophomonas_wolfei_subsp.metylbutyratica_JCM14075T_00109_Preprotein_translocase_band_1_subunit
[illegible]
>Syntrophomonas_wolfei_subsp.metylbutyratica_JCM14075T_00110_Enolase
[illegible]
>Syntrophomonas_wolfei_subsp.metylbutyratica_JCM14075T_00111_2,3-bisphosphoglycerate-independent_phosphoglycerate_mutase
[illegible]
>Syntrophomonas_wolfei_subsp.metylbutyratica_JCM14075T_00112_Bifunctional_PGK/TIM
[illegible]
>Syntrophomonas_wolfei_subsp.metylbutyratica_JCM14075T_00113_Phosphoglycerate_kinase
[illegible]
>Syntrophomonas_wolfei_subsp.metylbutyratica_JCM14075T_00114_Glyceraldehyde-3-phosphate_dehydrogenase
[illegible]
>Syntrophomonas_wolfei_subsp.metylbutyratica_JCM14075T_00115_Central_glycolytic_genes_regulator
[illegible]
>Syntrophomonas_wolfei_subsp.metylbutyratica_JCM14075T_00116_Membrane-bound_metallopeptidase
[illegible]
>Syntrophomonas_wolfei_subsp.metylbutyratica_JCM14075T_00117_Organic_hydroperoxide_resistance_transcriptional_regulator
[illegible]
>Syntrophomonas_wolfei_subsp.metylbutyratica_JCM14075T_00118_Acyl-CoA_dehydrogenase
[illegible]
>Syntrophomonas_wolfei_subsp.metylbutyratica_JCM14075T_00119_Electron_transfer_flavoprotein_small_subunit
[illegible]
>Syntrophomonas_wolfei_subsp.metylbutyratica_JCM14075T_00120_Electron_transfer_flavoprotein_large_subunit
[illegible]
>Syntrophomonas_wolfei_subsp.metylbutyratica_JCM14075T_00121_RNA_polymerase_sigma-54_factor_1
[illegible]
>Syntrophomonas_wolfei_subsp.metylbutyratica_JCM14075T_00122_Sporulation_transcription_regulator_WhiA
[illegible]
>Syntrophomonas_wolfei_subsp.metylbutyratica_JCM14075T_00123_LPPG:FO_2-phospho-L-lactate_transferase
[illegible]
>Syntrophomonas_wolfei_subsp.metylbutyratica_JCM14075T_00124_glmZ(sRNA)-inactivating_NTPase
[illegible]
>Syntrophomonas_wolfei_subsp.metylbutyratica_JCM14075T_00125_putative_phosphatase_YcdX
[illegible]
>Syntrophomonas_wolfei_subsp.metylbutyratica_JCM14075T_00126_bifunctional_phosphoglucose/phosphomannose_isomerase
[illegible]
>Syntrophomonas_wolfei_subsp.metylbutyratica_JCM14075T_00127_Glucokinase
[illegible]
>Syntrophomonas_wolfei_subsp.metylbutyratica_JCM14075T_00128_hypothetical_protein
[illegible]
>Syntrophomonas_wolfei_subsp.metylbutyratica_JCM14075T_00129_Sugar_phosphatase_YidA
[illegible]
>Syntrophomonas_wolfei_subsp.metylbutyratica_JCM14075T_00130_Membrane_protein_of_unknown_function
[illegible]
>Syntrophomonas_wolfei_subsp.metylbutyratica_JCM14075T_00131_Excinuclease_ABC_subunit_C
[illegible]
>Syntrophomonas_wolfei_subsp.metylbutyratica_JCM14075T_00132_Excinuclease_ABC_subunit_B
[illegible]
>Syntrophomonas_wolfei_subsp.metylbutyratica_JCM14075T_00133_Excinuclease_ABC_subunit_A
[illegible]
>Syntrophomonas_wolfei_subsp.metylbutyratica_JCM14075T_00134_AAA_domain_(dynein-related_subfamily)
[illegible]
>Syntrophomonas_wolfei_subsp.metylbutyratica_JCM14075T_00135_Cell_division_topological_determinant_MinJ
[illegible]
>Syntrophomonas_wolfei_subsp.metylbutyratica_JCM14075T_00136_Carboxy-terminal_processing_protease_CtpB_precursor
[illegible]
>Syntrophomonas_wolfei_subsp.metylbutyratica_JCM14075T_00137_Septal_ring_factor
[illegible]
>Syntrophomonas_wolfei_subsp.metylbutyratica_JCM14075T_00138_Cell_division_protein_FtsX
[illegible]
>Syntrophomonas_wolfei_subsp.metylbutyratica_JCM14075T_00139_Cell_division_ATP-binding_protein_FtsE
[illegible]
>Syntrophomonas_wolfei_subsp.metylbutyratica_JCM14075T_00140_1-deoxy-D-xylulose-5-phosphate_synthase
[illegible]
>Syntrophomonas_wolfei_subsp.metylbutyratica_JCM14075T_00141_Transketolase_1
[illegible]
>Syntrophomonas_wolfei_subsp.metylbutyratica_JCM14075T_00142_Peptide_chain_release_factor_2
[illegible]
>Syntrophomonas_wolfei_subsp.metylbutyratica_JCM14075T_00143_Chemotaxis_protein_MotB
[illegible]
>Syntrophomonas_wolfei_subsp.metylbutyratica_JCM14075T_00144_Chemotaxis_protein_PomA
[illegible]

AEQERARMGM*
>Syntrophomonas_wolfei_subsp.metylbutyratica_JCM14075T_00145_preprotein_translocase_subunit_SecA
[illegible]
>Syntrophomonas_wolfei_subsp.metylbutyratica_JCM14075T_00146_SigL_modulation_protein
[illegible]
>Syntrophomonas_wolfei_subsp.metylbutyratica_JCM14075T_00147_CxxC-x17-CxxC_domain_protein
MFQDKTLVCQDCGQEFVFTAGEQEFYSEKGFENEPKRCRECRVNRRNSRSNSRGPREMFAAVCAGCGAETEVPFRPVEGRPVYCMECFQKQKEAAY*
>Syntrophomonas_wolfei_subsp.metylbutyratica_JCM14075T_00148_hypothetical_protein
[illegible]
>Syntrophomonas_wolfei_subsp.metylbutyratica_JCM14075T_00149_hypothetical_protein
[illegible]
>Syntrophomonas_wolfei_subsp.metylbutyratica_JCM14075T_00150_Flagellar_protein_FliS
[illegible]
>Syntrophomonas_wolfei_subsp.metylbutyratica_JCM14075T_00151_UDP-4-amino-4-deoxy-L-arabinose--oxoglutarate_aminotransferase
[illegible]
>Syntrophomonas_wolfei_subsp.metylbutyratica_JCM14075T_00152_UDP-glucose_4-epimerase
[illegible]
>Syntrophomonas_wolfei_subsp.metylbutyratica_JCM14075T_00153_flagellar_protein_FlaG
[illegible]
>Syntrophomonas_wolfei_subsp.metylbutyratica_JCM14075T_00154_flagellar_protein_FlaG
[illegible]
>Syntrophomonas_wolfei_subsp.metylbutyratica_JCM14075T_00155_hypothetical_protein
[illegible]
>Syntrophomonas_wolfei_subsp.metylbutyratica_JCM14075T_00156_UDP-N-acetylglucosamine_4,6-dehydratase_(inverting)
[illegible]
>Syntrophomonas_wolfei_subsp.metylbutyratica_JCM14075T_00157_hypothetical_protein
[illegible]
>Syntrophomonas_wolfei_subsp.metylbutyratica_JCM14075T_00158_YcfA-like_protein
[illegible]
>Syntrophomonas_wolfei_subsp.metylbutyratica_JCM14075T_00159_hypothetical_protein
[illegible]
>Syntrophomonas_wolfei_subsp.metylbutyratica_JCM14075T_00160_putative_nucleotidyltransferase
[illegible]
>Syntrophomonas_wolfei_subsp.metylbutyratica_JCM14075T_00161_hypothetical_protein
[illegible]
>Syntrophomonas_wolfei_subsp.metylbutyratica_JCM14075T_00162_hypothetical_protein
[illegible]
>Syntrophomonas_wolfei_subsp.metylbutyratica_JCM14075T_00163_undecaprenyldiphospho-muramoylpentapeptide_beta-N-_acetylglucosaminyltransferase
[illegible]
>Syntrophomonas_wolfei_subsp.metylbutyratica_JCM14075T_00164_hypothetical_protein
[illegible]
>Syntrophomonas_wolfei_subsp.metylbutyratica_JCM14075T_00165_hypothetical_protein
[illegible]
>Syntrophomonas_wolfei_subsp.metylbutyratica_JCM14075T_00166_Pseudaminic_acid_synthase
[illegible]
>Syntrophomonas_wolfei_subsp.metylbutyratica_JCM14075T_00167_Spermidine_N(1)-acetyltransferase
[illegible]
>Syntrophomonas_wolfei_subsp.metylbutyratica_JCM14075T_00168_pseudaminic_acid_biosynthesis-associated_methylase
[illegible]
>Syntrophomonas_wolfei_subsp.metylbutyratica_JCM14075T_00169_3-deoxy-manno-octulosonate_cytidylyltransferase
[illegible]
>Syntrophomonas_wolfei_subsp.metylbutyratica_JCM14075T_00170_hypothetical_protein
[illegible]
>Syntrophomonas_wolfei_subsp.metylbutyratica_JCM14075T_00171_Acyl_carrier_protein
[illegible]
>Syntrophomonas_wolfei_subsp.metylbutyratica_JCM14075T_00172_FkbH_domain_protein
[illegible]
>Syntrophomonas_wolfei_subsp.metylbutyratica_JCM14075T_00173_3-demethylubiquinone-9_3-methyltransferase
[illegible]
>Syntrophomonas_wolfei_subsp.metylbutyratica_JCM14075T_00174_Putative_ribosomal_N-acetyltransferase_YdaF
[illegible]
>Syntrophomonas_wolfei_subsp.metylbutyratica_JCM14075T_00175_L-glutamine:2-deoxy-scyllo-inosose_aminotransferase
[illegible]
>Syntrophomonas_wolfei_subsp.metylbutyratica_JCM14075T_00176_UDP-N-acetylglucosamine_2-epimerase
[illegible]
>Syntrophomonas_wolfei_subsp.metylbutyratica_JCM14075T_00177_putative_oxidoreductase_YcjS
[illegible]
>Syntrophomonas_wolfei_subsp.metylbutyratica_JCM14075T_00178_3-deoxy-manno-octulosonate_cytidylyltransferase
[illegible]
>Syntrophomonas_wolfei_subsp.metylbutyratica_JCM14075T_00179_hypothetical_protein
[illegible]
>Syntrophomonas_wolfei_subsp.metylbutyratica_JCM14075T_00180_hypothetical_protein
[illegible]
>Syntrophomonas_wolfei_subsp.metylbutyratica_JCM14075T_00181_Flagellin
[illegible]
>Syntrophomonas_wolfei_subsp.metylbutyratica_JCM14075T_00182_flagellar_capping_protein
[illegible]
>Syntrophomonas_wolfei_subsp.metylbutyratica_JCM14075T_00183_hypothetical_protein
[illegible]
>Syntrophomonas_wolfei_subsp.metylbutyratica_JCM14075T_00184_Flagellar_assembly_factor_FliW
[illegible]
>Syntrophomonas_wolfei_subsp.metylbutyratica_JCM14075T_00185_hypothetical_protein
[illegible]
>Syntrophomonas_wolfei_subsp.metylbutyratica_JCM14075T_00186_Flagellin
[illegible]
>Syntrophomonas_wolfei_subsp.metylbutyratica_JCM14075T_00187_Flagellar_hook-associated_protein_1
[illegible]
>Syntrophomonas_wolfei_subsp.metylbutyratica_JCM14075T_00188_FlgN_protein
[illegible]
>Syntrophomonas_wolfei_subsp.metylbutyratica_JCM14075T_00189_flagellar_biosynthesis_anti-sigma_factor_FlgM
[illegible]
>Syntrophomonas_wolfei_subsp.metylbutyratica_JCM14075T_00190_flagellar_operon_protein
[illegible]
>Syntrophomonas_wolfei_subsp.metylbutyratica_JCM14075T_00191_DNA_utilization_protein_GntX
[illegible]
>Syntrophomonas_wolfei_subsp.metylbutyratica_JCM14075T_00192_hypothetical_protein
[illegible]
>Syntrophomonas_wolfei_subsp.metylbutyratica_JCM14075T_00193_Transcriptional_regulatory_protein_LiaR
[illegible]
>Syntrophomonas_wolfei_subsp.metylbutyratica_JCM14075T_00194_Signal_transduction_histidine-protein_kinase/phosphatase_DegS
[illegible]
>Syntrophomonas_wolfei_subsp.metylbutyratica_JCM14075T_00195_UDP-N-acetylglucosamine_2-epimerase
[illegible]
>Syntrophomonas_wolfei_subsp.metylbutyratica_JCM14075T_00196_hypothetical_protein
[illegible]
>Syntrophomonas_wolfei_subsp.metylbutyratica_JCM14075T_00197_Inner_membrane_transport_permease_YbhR
[illegible]
>Syntrophomonas_wolfei_subsp.metylbutyratica_JCM14075T_00198_Inner_membrane_transport_permease_YbhR
[illegible]
>Syntrophomonas_wolfei_subsp.metylbutyratica_JCM14075T_00199_putative_ABC_transporter_ATP-binding_protein_YbhF
[illegible]
>Syntrophomonas_wolfei_subsp.metylbutyratica_JCM14075T_00200_Multidrug_resistance_protein_MdtN
[illegible]
>Syntrophomonas_wolfei_subsp.metylbutyratica_JCM14075T_00201_Fatty_acid_metabolism_regulator_protein
[illegible]
>Syntrophomonas_wolfei_subsp.metylbutyratica_JCM14075T_00202_Fatty_acid_metabolism_regulator_protein
[illegible]
>Syntrophomonas_wolfei_subsp.metylbutyratica_JCM14075T_00203_Swarming_motility_protein_SwrC
[illegible]
>Syntrophomonas_wolfei_subsp.metylbutyratica_JCM14075T_00204_Macrolide-specific_efflux_protein_MacA_precursor
[illegible]
>Syntrophomonas_wolfei_subsp.metylbutyratica_JCM14075T_00205_sodium/calcium_exchanger_1
[illegible]
>Syntrophomonas_wolfei_subsp.metylbutyratica_JCM14075T_00206_Serine/threonine-protein_kinase_PknK
[illegible]
>Syntrophomonas_wolfei_subsp.metylbutyratica_JCM14075T_00207_hypothetical_protein
[illegible]
>Syntrophomonas_wolfei_subsp.metylbutyratica_JCM14075T_00208_putative_protein_involved_in_cytokinesis,_contains_TGc_(transglutaminase/protease-like)_domain
[illegible]
>Syntrophomonas_wolfei_subsp.metylbutyratica_JCM14075T_00209_putative_CtpA-like_serine_protease
[illegible]
>Syntrophomonas_wolfei_subsp.metylbutyratica_JCM14075T_00210_Transposase
[illegible]
>Syntrophomonas_wolfei_subsp.metylbutyratica_JCM14075T_00211_Outer_membrane_biogenesis_protein_BamB
[illegible]
>Syntrophomonas_wolfei_subsp.metylbutyratica_JCM14075T_00212_hypothetical_protein
[illegible]
>Syntrophomonas_wolfei_subsp.metylbutyratica_JCM14075T_00213_Cell_cycle_control_protein
[illegible]
>Syntrophomonas_wolfei_subsp.metylbutyratica_JCM14075T_00214_phage_major_capsid_protein,_HK97_family
[illegible]
>Syntrophomonas_wolfei_subsp.metylbutyratica_JCM14075T_00215_hypothetical_protein
[illegible]
>Syntrophomonas_wolfei_subsp.metylbutyratica_JCM14075T_00216_hypothetical_protein
[illegible]
>Syntrophomonas_wolfei_subsp.metylbutyratica_JCM14075T_00217_Tyrosine_recombinase_XerD

MKKAKTEAVDIAKYISLFLREYAPNHLTGSPHTLRSYENALTLYVGFLEQSGITLECFEASCFDKEKIERWLVWLTEIRNCSPETCNNRLASLRAFLKYLASRDVKYSDLSNGAATIRLRKRKNRKVNGLSRS[illegible]
HAKNPDIPEDLHAHQFRHAKASHWLEDGMNIVQISFLLGHAQLQTTMIYMDITIEQEAKAMATLESENDKKVTAKWKNTDTSISSLCGISKLSKK*
>Syntrophomonas_wolfei_subsp.metylbutyratica_JCM14075T_00218_site-specific_tyrosine_recombinase_XerC
MFEISASRKVLIQPFRDFRIASDRWNKANDKLMLYFDRHCLENFPKIEGITQDMIDSWCIQRKTEKSISCYCRTYIIAKLVEFLNQRKICSLVVPELIKAECHRTYIPHAFTDEELSSFFAECDNEVSKAPNQLT[illegible]
RNINSWTHEGFGFSDKFLYLSKSMGHMQLESTKYYYSLIPALADTIASHSQQGFDDIIPEVTEYEES*
>Syntrophomonas_wolfei_subsp.metylbutyratica_JCM14075T_00219_Tyrosine_recombinase_XerD
MDINYLLIHHDELLTYLREHDYAETYVNRYKTTIKQITDNASNHNWTTYEDVYQWYVDQSYSPTYLHEVRAIIGKLEQFHLHGNFPDNKKSYSSFWKIKSAYTKLSSEYKQLYEDFETQTCKGLKSSTVA[illegible]
ACMQLEFIDWNNDRIRIVQQKTSQPLEIPLLPVVGNAIYDYCINERPMSASPYLFLDSRAPHNNLTTDGIGYSVKKIMSAANIRQEKGRRKGTHIFRHHLAIKLLSNGVPQPVITRTMGHTAPESLSSYLHA[illegible]
>Syntrophomonas_wolfei_subsp.metylbutyratica_JCM14075T_00220_Transposase
MPRVRRALSSTGIYHIIVRGINKQNIFVENGDNERFIDTLAGYQKEIEYEIYAYCLMGNHVHLLMKEGNEEIGNTMRRIGISYAYWYNWQYNRIGHLFQDRFKSEPVEDDAYFLTVLRYIHQNPVKAGLSS[illegible]
VGQVPGT*
>Syntrophomonas_wolfei_subsp.metylbutyratica_JCM14075T_00221_putative_transposase
MKIQNPHDRFFKETLGKVEVAKDFLNNYLPGNIIKVIDVDTLEPQKDSFINKELQEGFSDLLFKANINNREGYIYFLFEHKSYTRKDIAFQLLRYMMEIWETKSKKEKADELPVIIPLVVYHGKDRWKIGTT[illegible]
VMTLAEIFREEGKEEGILKGMEVGAKQGKIEVAKNAIREGMELGLIEKLTGLPKKEIGKIAKEIEN*
>Syntrophomonas_wolfei_subsp.metylbutyratica_JCM14075T_00222_Tyrosine_recombinase_XerC
MNTIQQFEQHLLADGKAKKTIQSYLADVSSFHRYLQGQGISQADKINRSHITGFRNWMLEQDYKPATVNKAVNSLSSYTTYLVQTGVLPAGPPIVKPKQDRVKLDSDSEHQVEVFSEAELEQLLDYVD[illegible]
FCTRLVQAGIPITTVSKLAGHADISTTANYYINTSQKDKAQAVALL*
>Syntrophomonas_wolfei_subsp.metylbutyratica_JCM14075T_00224_hypothetical_protein
[illegible]
>Syntrophomonas_wolfei_subsp.metylbutyratica_JCM14075T_00225_hypothetical_protein
[illegible]
>Syntrophomonas_wolfei_subsp.metylbutyratica_JCM14075T_00226_Endoglucanase_precursor
[illegible]
>Syntrophomonas_wolfei_subsp.metylbutyratica_JCM14075T_00227_hypothetical_protein
[illegible]
>Syntrophomonas_wolfei_subsp.metylbutyratica_JCM14075T_00228_hypothetical_protein
[illegible]
>Syntrophomonas_wolfei_subsp.metylbutyratica_JCM14075T_00229_Serine/threonine-protein_kinase_AfsK
[illegible]
>Syntrophomonas_wolfei_subsp.metylbutyratica_JCM14075T_00230_outer_membrane_channel_protein
[illegible]
>Syntrophomonas_wolfei_subsp.metylbutyratica_JCM14075T_00231_hypothetical_protein
[illegible]
>Syntrophomonas_wolfei_subsp.metylbutyratica_JCM14075T_00232_Sigma-W_factor
[illegible]
>Syntrophomonas_wolfei_subsp.metylbutyratica_JCM14075T_00233_mycothiol_system_anti-sigma-R_factor
[illegible]
>Syntrophomonas_wolfei_subsp.metylbutyratica_JCM14075T_00234_Serine_protease_AprX
[illegible]
>Syntrophomonas_wolfei_subsp.metylbutyratica_JCM14075T_00235_putative_membrane_protein
[illegible]
>Syntrophomonas_wolfei_subsp.metylbutyratica_JCM14075T_00236_UDP-N-acetyl-alpha-D-glucosamine_C6_dehydratase
[illegible]
>Syntrophomonas_wolfei_subsp.metylbutyratica_JCM14075T_00237_putative_bicarbonate_transporter,_IctB_family
[illegible]
>Syntrophomonas_wolfei_subsp.metylbutyratica_JCM14075T_00238_N-acetylmuramoyl-L-alanine_amidase_LytC_precursor
[illegible]
>Syntrophomonas_wolfei_subsp.metylbutyratica_JCM14075T_00239_Putative_transcriptional_regulator_YvhJ
[illegible]
>Syntrophomonas_wolfei_subsp.metylbutyratica_JCM14075T_00240_putative_undecaprenyl-phosphate_N-acetylglucosaminyl_1-phosphate_transferase
[illegible]
>Syntrophomonas_wolfei_subsp.metylbutyratica_JCM14075T_00241_colanic_acid_biosynthesis_protein
[illegible]
>Syntrophomonas_wolfei_subsp.metylbutyratica_JCM14075T_00242_putative_peptidoglycan_biosynthesis_protein_MurJ
[illegible]
>Syntrophomonas_wolfei_subsp.metylbutyratica_JCM14075T_00243_hypothetical_protein
[illegible]
>Syntrophomonas_wolfei_subsp.metylbutyratica_JCM14075T_00244_hypothetical_protein
[illegible]
>Syntrophomonas_wolfei_subsp.metylbutyratica_JCM14075T_00245_hypothetical_protein
[illegible]
>Syntrophomonas_wolfei_subsp.metylbutyratica_JCM14075T_00246_hypothetical_protein
[illegible]
>Syntrophomonas_wolfei_subsp.metylbutyratica_JCM14075T_00247_D-alanyl-lipoteichoic_acid_biosynthesis_protein_DltB
[illegible]
>Syntrophomonas_wolfei_subsp.metylbutyratica_JCM14075T_00248_Lysine--tRNA_ligase
[illegible]
>Syntrophomonas_wolfei_subsp.metylbutyratica_JCM14075T_00249_Transcript_cleavage_factor_GreA
[illegible]
>Syntrophomonas_wolfei_subsp.metylbutyratica_JCM14075T_00250_hypothetical_protein
[illegible]
>Syntrophomonas_wolfei_subsp.metylbutyratica_JCM14075T_00251_hypothetical_protein
[illegible]
>Syntrophomonas_wolfei_subsp.metylbutyratica_JCM14075T_00252_hypothetical_protein
[illegible]
>Syntrophomonas_wolfei_subsp.metylbutyratica_JCM14075T_00253_putative_tRNA-dihydrouridine_synthase
[illegible]
>Syntrophomonas_wolfei_subsp.metylbutyratica_JCM14075T_00254_Type_III_pantothenate_kinase
[illegible]
>Syntrophomonas_wolfei_subsp.metylbutyratica_JCM14075T_00255_Biotin_ECF_transporter_S_component_BioY
[illegible]
>Syntrophomonas_wolfei_subsp.metylbutyratica_JCM14075T_00256_putative_nicotinate-nucleotide_pyrophosphorylase_[carboxylating]
[illegible]
>Syntrophomonas_wolfei_subsp.metylbutyratica_JCM14075T_00257_L-aspartate_oxidase
[illegible]
>Syntrophomonas_wolfei_subsp.metylbutyratica_JCM14075T_00258_Quinolinate_synthase_A
[illegible]
>Syntrophomonas_wolfei_subsp.metylbutyratica_JCM14075T_00259_Aspartate_1-decarboxylase_precursor
[illegible]
>Syntrophomonas_wolfei_subsp.metylbutyratica_JCM14075T_00260_Pantothenate_synthetase
[illegible]
>Syntrophomonas_wolfei_subsp.metylbutyratica_JCM14075T_00261_3-methyl-2-oxobutanoate_hydroxymethyltransferase
[illegible]
>Syntrophomonas_wolfei_subsp.metylbutyratica_JCM14075T_00262_arogenate_dehydrogenase
[illegible]
>Syntrophomonas_wolfei_subsp.metylbutyratica_JCM14075T_00263_Bifunctional_folate_synthesis_protein
[illegible]
>Syntrophomonas_wolfei_subsp.metylbutyratica_JCM14075T_00264_Dihydroneopterin_aldolase
[illegible]
>Syntrophomonas_wolfei_subsp.metylbutyratica_JCM14075T_00265_Dihydropteroate_synthase
[illegible]
>Syntrophomonas_wolfei_subsp.metylbutyratica_JCM14075T_00266_PII_uridylyl-transferase
[illegible]
>Syntrophomonas_wolfei_subsp.metylbutyratica_JCM14075T_00267_ATP-dependent_zinc_metalloprotease_FtsH
[illegible]
>Syntrophomonas_wolfei_subsp.metylbutyratica_JCM14075T_00268_tRNA(Ile)-lysidine_synthase
[illegible]
>Syntrophomonas_wolfei_subsp.metylbutyratica_JCM14075T_00269_Stage_II_sporulation_protein_E
[illegible]
>Syntrophomonas_wolfei_subsp.metylbutyratica_JCM14075T_00270_Formate_channel_1
[illegible]
>Syntrophomonas_wolfei_subsp.metylbutyratica_JCM14075T_00271_Guanosine-5'-triphosphate,3'-diphosphate_pyrophosphatase
[illegible]
>Syntrophomonas_wolfei_subsp.metylbutyratica_JCM14075T_00272_hypothetical_protein
[illegible]
>Syntrophomonas_wolfei_subsp.metylbutyratica_JCM14075T_00273_cell_division_protein_FtsL
[illegible]
>Syntrophomonas_wolfei_subsp.metylbutyratica_JCM14075T_00274_spore_cortex_biosynthesis_protein_YabQ
[illegible]
>Syntrophomonas_wolfei_subsp.metylbutyratica_JCM14075T_00275_Spore_protein_YabP
[illegible]
>Syntrophomonas_wolfei_subsp.metylbutyratica_JCM14075T_00276_hypothetical_protein
[illegible]
>Syntrophomonas_wolfei_subsp.metylbutyratica_JCM14075T_00278_Heat_shock_protein_15
[illegible]
>Syntrophomonas_wolfei_subsp.metylbutyratica_JCM14075T_00279_hypothetical_protein
[illegible]
>Syntrophomonas_wolfei_subsp.metylbutyratica_JCM14075T_00280_Nucleoside_triphosphate_pyrophosphohydrolase
[illegible]
>Syntrophomonas_wolfei_subsp.metylbutyratica_JCM14075T_00281_Stage_III_sporulation_protein_F
[illegible]
>Syntrophomonas_wolfei_subsp.metylbutyratica_JCM14075T_00282_Stage_V_sporulation_protein_T
[illegible]
>Syntrophomonas_wolfei_subsp.metylbutyratica_JCM14075T_00283_Spore_germination_protein_YndE
[illegible]
>Syntrophomonas_wolfei_subsp.metylbutyratica_JCM14075T_00284_Spore_germination_protein_B1
[illegible]
>Syntrophomonas_wolfei_subsp.metylbutyratica_JCM14075T_00285_Spore_germination_protein_B3_precursor
[illegible]

SRSLAQITPKLQEGAIIMRIEIKAEGNFYEQNEAGELFTRERFQAIEELANQEIKRQILLSIDKAQELNSDVFGWGQMIKQNYPQQWGEMEGNWEELFPTVDKQIKVDFSLRRSYLADKSFNFQ*
>Syntrophomonas_wolfei_subsp.metylbutyratica_JCM14075T_00286_Spore_germination_protein_B3_precursor
MPRRLLLFIVIFLLPSCSACWDIRELNNSAVCTGAGVELSREGKFIFSGQMVKPSAPSESGTQTSTAVVLSASGSGVADAARRFMLSLSRRPFWSSISTFVIGEELARED[illegible]
QFTGKTTPKLEQDQLKMKVTIRAELVFYEKSKSGELLTLSWKEEMERLAAQKIKQEISACIKKSQLLGSDILGWGYILQKHEPQLWESFSANWDDFFPTIESDIEVETLIVNSMLSQKSFRFR*
>Syntrophomonas_wolfei_subsp.metylbutyratica_JCM14075T_00287_hypothetical_protein
MYYNLILSIVLFFLLFLLREGRSWLKNPLGIEYCCAVLLFLLSLAYTLDLVQGWHLMPNPGAIFAKLKPLSDLLEII*
>Syntrophomonas_wolfei_subsp.metylbutyratica_JCM14075T_00288_spore_germination_protein_(amino_acid_permease)
MENKTLSSQQIAFMIFLFLTGSSLVYIPEIAARQSAWIACLLASLIGVYIIIILLALFTRFPDMSITRISELLLGKIAGSVLNILLFFSLFLIAQ[illegible]
FIVGFTALTVYYQGLVLNLKELLRLENPRPLILPLGLFLMVFTSYMYPSDLNYITTRLRYVPFYTISVNLLYLTIIFIAASLSKRRLSRHSRGEG*
>Syntrophomonas_wolfei_subsp.metylbutyratica_JCM14075T_00289_Transcription-repair-coupling_factor
LQENFWDLLCGVLSKRQNILLTGLSGSAKAFMLAEILKKQNRKILCLLPEEEKAYDLARDLQAFIEPGRLFMFLARDFLFMKENLST[illegible]
RRYRESLKEARKDGKIIREIKLLDSEYLKKEIEAHSVIYHAYFSSTIPQVAVASLQHISQKEMEPFYRQYETLFARIKEWQGKGLKVILAV[illegible]
LQLYAHRESREGYAFSPDTPWQSQFEDEFPFRETPDQLKAVNEVKKDMESRRPMDRLVCGDVGYGKTEVFLRAAFKAIMDGKQ[illegible]
DIYRVKEQLDELFPGIKIAVGHGRMKEDELARVMMDFVNGKYPLFLCTTIIESGLDMPNVNTIIVDEADKMGLAQLYQLRGRVG[illegible]
SLRRKGRQIEIQTLQRLPSNLSERFRGIRRVNDHTLIIQKYENSSLPALAEILQML*
>Syntrophomonas_wolfei_subsp.metylbutyratica_JCM14075T_00290_hypothetical_protein
MTTYYICEYCEQVFSTVETEGQDGSVEVKGICEECAMEMGLLESASLGGKQYYN*
>Syntrophomonas_wolfei_subsp.metylbutyratica_JCM14075T_00291_hypothetical_protein
MQHPAKVSRRKGASDRTSRERFTLRVLLVFHPAGVTIKVGDHGWEISAVYLCKLMNSKNFAVESKRYICLPQKRKYIKRWKAGQAYDYLLHL*
>Syntrophomonas_wolfei_subsp.metylbutyratica_JCM14075T_00292_Peptidyl-tRNA_hydrolase
MKMLVGLGNPGKKYSHTRHNIGFKVLEELARRHRIEKEKESRYDAIVGHLRINQEKLLVKPLTFMNLSGKAVRPLFNWFKLELSELLVVYDDMDLPPGAVRIRASGGTGGHKGMQSICESLGSDRFPRIRIGIGRPPGGAIDWVLGEFSESEKPLMQDAVEKAASAIECWIKSGIDACMNAYN*
>Syntrophomonas_wolfei_subsp.metylbutyratica_JCM14075T_00293_50S_ribosomal_protein_L25
MLGQKLNARKREIKNRGYLNQLKRSEQVPAVIYGKGEEAVPISVENLRQKLRNIFNVHGSRGIFSLEIEGESQPMMTLIREIQRNPVSGQLIHLDFLSVNLNEKINSNVGVLLGGEEVMKKGGILQAGLKEVEVLCFPQDLPEYLSADISSLEIGDTLHVADLIVPAEVEILTEAEAVIASILAPSKATAGEEEGVEGAGEGEAEEKPEQ*
>Syntrophomonas_wolfei_subsp.metylbutyratica_JCM14075T_00294_Ribose-phosphate_pyrophosphokinase
MQASRHWRMKLFSGNANLQLAEEIARYLGIPVGDARVSRFSDGEIQVNIEESVRGVDVFVVQPTCTPVNENLMELLIMIDAFRRASATRINAVIPYYGYARQDRKSRARDPITAKLVSNLIVQAGAQRVVAVDLHATQIQGFFDIPVDHLPGVPTLAEYFKTKGLEEDAVVVSPDVGGVTRARDLATKLRAPLAIVDKRRPAPNVSEIMNVIGEVKDKSVIIVDDIIDTAGTLCTAAEVMLEKGARDVYS
CCTHAVFSGPALDRLSRAPLKEIVITNTIPIDESKKLPNMIILSIAPLVGEAILRIHEDLEVSKLFEE*
>Syntrophomonas_wolfei_subsp.metylbutyratica_JCM14075T_00295_Bifunctional_protein_GlmU
VKLSAVILAAGKGLRMRSDLPKVAHKVAGKPILHVKAVKEAGIEDIAVVVGHGREIVQEICSGEKIRFVLQEEQLGTGHALMQAEAVVAPEDTILVLAGDIPLIQAETLRKLIKNHRQKQATATVLSVNMRNPSGYGRILRDKQGAFLRIIEEKDANDEEKGIKEINSGIYCFSARKVFSALHSTSTRNAQGEYYLTEALELLKNQQESIGIFLSDGEEDIYGINDRVQLVQAENILRQRKNRELMLSGVSL
MDPASTFIDSDVLIGHDTIIEPFSIIEGNSRIGRECEIGPTRISDSRIGNEVKIESSILIQSAVADRCSIGPFAYLRPETTLQEGVKVGDFVEIKKSTIGSGSKIPHLSYVGDATIGQGVNVGAGTITCNYDGKNKYQTVLEDRVFIGSNTNLVAPVRIGENSITGAGSTISRDVPPHTLAVERAGQKHIPRG*
>Syntrophomonas_wolfei_subsp.metylbutyratica_JCM14075T_00296_bifunctional_N-acetylglucosamine-1-phosphate_uridyltransferase/glucosamine-1-phosphate_acetyltransferase
MQYDAVILAGGVNSSELKIAPYDNEALIIIGKYPMIYYVVNALRSSQYIDKIVVVGPKEIMTSRIAFEKPIVVEGGKNAVESLACAVKLLKREKGTTERLLVLPTDIPFITTTAIDDFANQCRNYEADFYFAITSREVNELKFGVVRTYVKLKDGVFTGGNLFIRSEIIDQVLEKASQIVERRKNPLAIASLFGFGLMWRYLTRRLSIAAAEKRFFKVLGIKGKAIISPYAEVGVDVDKPSDLEIAQKYLLDLTL
*
>Syntrophomonas_wolfei_subsp.metylbutyratica_JCM14075T_00297_putative_HTH-type_transcriptional_regulator_YdfH
VIFNTGEKRLSPVDLLSYKPLRELVLEAIREAIKNGVLKPGERLMEIQLAEELGVSRTPIREALRKLELEGHVMVPRKGAYVADISLKDVADVFEIARALEALAAGLAAERITDEELEEMERLLVEKVEAISSNDMDKLVEVDTKFHEAIYRASRNQRLFAIINNLREQIQRFRSTSLSYPGRMQQSMQEHRDIVEAIQSRDVQLSRQLAQEHIENAEQSMIDSIKKNGLPWA*
>Syntrophomonas_wolfei_subsp.metylbutyratica_JCM14075T_00298_4-diphosphocytidyl-2-C-methyl-D-erythritol_kinase
MRKNIILKAPAKVNLTLDVKGKRSDGYHELETVMHQVNLLDIIISQGTGGIQIKSNNSLIPTNEENLAYQAAEMILGEYAHKGEVEIYIEKNIPVGAGLAGGSTDAAAVIQGINQLCDLGLEEELLEMAARIGSDVAFCLAGGSKLARGRGEILSNLPQRMIPYIILVKPHFQLSTAEVYRELDLTQVEEFPDNVAFLAAWEAYDIINIAGNMRNVLETVSIRKYPEIAAIKAELIRTGALNALMSGSGPSV
MGIFMEEQQALKAREQFRTRYQEVFLLSSYVSTKSEE*
>Syntrophomonas_wolfei_subsp.metylbutyratica_JCM14075T_00299_stage_II_sporulation_protein_R
MRKYLGIIMIALILLGSILCLQEKPLNKSVLRLHVIANSDSLADQALKIQVKDAVVEMMKKEFAGMDNMEQARQAALEDIAEIKRTAEAVVQANGYNYPVQVSLGEYQFPTKSYGNLVLPQGEYQAVRVTIGGGEGKNWWCVLFPPLCMVSSSEQGLSLHSPEEAKVSFKCLELIPKGAIFRTSNH*
>Syntrophomonas_wolfei_subsp.metylbutyratica_JCM14075T_00300_Ribbon-helix-helix_protein_copG_family
MATDKFEHATFYLTKNQVDLIKELARKNQISRSALVRMIIREYLARQEGNKSE*
>Syntrophomonas_wolfei_subsp.metylbutyratica_JCM14075T_00301_NTE_family_protein_RssA
MKSLGLALGGGGLKGLAHIGVLQVLEENSIPISAISGTSAGSIVAALYASGLSPWRMQEIAMQLEVKDYIDYDIVALTKFFWALLLPGVKTRLDGLVKGDKLEKLLYKWTRGKTLAESRIPLSIIACDIDSGQEIIFTNQNMEVTGPLVIKEALLSEAVRASTSIPATFVPLQFRGMQMVDGGVKEMVPVEVQKVMGVKGYILSVNLGRESYAEKVTGITQIVSRSLNIMSYETSATAEALFADLVIHPRVK
AVRLDDIEQAERIIRAGRRAAKAKIDEIKRGLED*
>Syntrophomonas_wolfei_subsp.metylbutyratica_JCM14075T_00302_hypothetical_protein
LSSLKINRQDKNLMRKQLEEILSIQKDLDKKINNYQQESVVEEYRGFWQELGQNNKDSIQRVSRYMVRKCNR*
>Syntrophomonas_wolfei_subsp.metylbutyratica_JCM14075T_00303_hypothetical_protein
VSRLLKCEPEGCMLDLWAKVLKAKYIEKQVKVLDTVFCVPDEQVGGKVSCVEDIKVKVIKAHEDLCFNKVKIFIDYEVILFVLVEGEYQITVTDRYEQSIELDEFDPPLTSEEFRIEIEQSEILKNWSFDFEIKGNCEDSSNPCKLTTPIKGTCIGLKVYVDIIDKLGKFHDVVVYGELDPVDY*
>Syntrophomonas_wolfei_subsp.metylbutyratica_JCM14075T_00304_Anp1
MADTRRGEQAKGDESSTMIDMAEKIMIGCPVNRRAWSLGYYLTSIRKLAYPESRLEFCFIINDCIDETPQILEDFARRENGRVRLIYYDLGQKYGHLRGQYNFSQLARLRNRLLEAFLESDAYLFSVDSDILVPVDSLSLLQDDCDIVSALVCNGEEVGDNSIYNILVKDAAGNLVHCRDPRDRVFPVDCTGAAYLIKRKVIASLGIRYSSDDGAEDIGFCRQAQEKGIGIFCDGRIECIHLMREDTTL
LSSKKTKIP*
>Syntrophomonas_wolfei_subsp.metylbutyratica_JCM14075T_00305_Putative_teichuronic_acid_biosynthesis_glycosyltransferase_TuaH
LSNINCILYPPTVDFHYLVQRPQHLIKNFSELGVLSFFLNNPHLHEHQRRGIERINPYLFLFNGVEPTEFLRDIRPVVYYSATAHVDMIKKYRPSLVVFDSVDEPSDEFAGWRPYIHRAVATADIVITTSDKLYKMAKSINPRSVLVPNACDYEHFSPSTHLPIPADMRGINSPVIGYIGAIATWVDLELLDRVAARLPHFNFVMLGPLYNVSELPQRPNLHWLGFKDYKDLPAYAQAFDVGLIPFKSIEN
TESVNPIKMWEYMAVGMPVVATALPEVYKFQELIYYSENEEEFIENIGKALVTDNPFRQKRQMELARQNSWSARAVEIIDIIENTLAEKGIKKTEYRVGDIMDTLLPDSAYTDFNEPFRASLSPFQQVLVSPGSGFRYSIYPGKESRDRDRVRNVRWSRADSAFPAGDIAVVQKSGFRYYTQRHQKELGLYYG*
>Syntrophomonas_wolfei_subsp.metylbutyratica_JCM14075T_00306_hypothetical_protein
MGESKLMEGKNLGILKVNTQKYQATQLAAGDDEGQFMISTAKKSSLKANKICPRCQERVNKQEMKSCDHCGSELCARCLKEYQKKHLPKTVPRRDSSIQGN*
>Syntrophomonas_wolfei_subsp.metylbutyratica_JCM14075T_00307_hypothetical_protein
LGEALSIWSPVVMAELSTPRSFDHHQIYYLDERFQGLEDLRVKLREYIVRCQGNALETLFKLEVICLLENMDGKMQLVKKEENLRDRVLLQEFDRNLDFKEPRIDFIGDIKKINWQGGLDGNQLHVILFIDYILLAIRQQVIRLLEQEAELKSSDLRVEGAAQDETARMEEKNLELRRQLRFYEKNIASLKKGIKKAESRNAVLSRESQQHQLLIEELRRAIQEKEQRLSRYETRLYYEDNQDNVYEEIYS
NNEENERISGNSLGSRVKRLFLNSI*
>Syntrophomonas_wolfei_subsp.metylbutyratica_JCM14075T_00308_hypothetical_protein
LNSPRAISDIKKDLESFVGSKIRLKANRGRNRIIEKGVLESIYPNIFVVKLDERKIERRVSYTYADVLTETVELFVYDTEDSEIRIAAANH*
>Syntrophomonas_wolfei_subsp.metylbutyratica_JCM14075T_00309_Putative_PBSX_repressor
LHNLIGGRIRHIRKTLKMTQEALAAQLGVSQQHISHVENGTKQASQLLIKHICLLFDTTETWLTTGKGDVFLSNEEIIKNQILHIGERAFAEALRKLLNDDSLEVLGTLLGPRLGEKPYDPSLEPMIDFLVDLWSLRDEKLKSWALVQFERAFPEDMKEKVKNSCANNHNRESIA*
>Syntrophomonas_wolfei_subsp.metylbutyratica_JCM14075T_00310_Diguanylate_cyclase_DosC
MNKAVKILVIEHNAEDISFIQRVFSLDKGKSELIFAERIAQALDLLKSNDFDLIMLNLALPDLSGIRIFEEILDKYTELPVIILSNPADEAIALEAVRKGAQDYIIKSETDFYPIKRIINCSIERNRLRINMAQLSITDELTKLYNRRGFFIMTQQLALARRLCQPVGFFFIDIDGMKDINDFWGHHQGDQALIDVSKILRESCRATDVIGRIGGDEFAISFIKEMNCIENIENRIRNKIELHNKSRLQKHMLSVSIGC
YCINVQCVDIQRALAEADKRMYLEKRNKKCLYRRISNQS*
>Syntrophomonas_wolfei_subsp.metylbutyratica_JCM14075T_00311_hypothetical_protein
MIFNSPSKGKLDLEEVVIDILNYIAEDLTRSYKLIVGSDSHIPGAQGTCYVSAIIIHRMGKGARYYYNKILDRRSSSLRQRIFYEASLSLDLADRLSQCLKKHGKDDMKVEIHLDVGIQGDTKDLVKEVTGMIVGSGFAAKIKPDACAASTVADRYTK*
>Syntrophomonas_wolfei_subsp.metylbutyratica_JCM14075T_00312_Ribosomal_RNA_small_subunit_methyltransferase_A
MKRIDLSLGRYYMEKYGIHPRKKWGQNFLVDGNILRKIAHLCNPGCKELVLEIGPGLGGLTRELTGISKGVLAIEIDFGLREALAESLQGLNNIHLLFADILQIELEELRKAFGEEDISGYKVCANIPYNITTPIIFKLLETCPQMESATLMMQKEVAGRILASPGSKEYGLLTLTTAYYAEVEYLMPVSRNCFYPRPEVDSSVIQLRPIKGKRVQVKDESNFKKLLRVSFQKRRKTILNICVDLFSAEKAEIH
SILNSIGIDPKSRPENLAIEQFARISDTLVMRRFGND I*
>Syntrophomonas_wolfei_subsp.metylbutyratica_JCM14075T_00313_sporulation_lipoprotein_YhcN/YlaJ_family
MKRKLLYLLCFSLMFMLLASGCQKTTAKKPMAPSAKKPTQTSQMTPSERRVLASKLSKMAEQVKGVQKATVVVADIGIANNITGNTKTNAQMNRATAKNLNDNTIGSDAGINRSEPKGMIVMVGLTLNPGNKDMNKSNQIKSKVMNKLKASDRRISQVLVTTDPNLVKRITDVAAGILEGKPIKTFEKDIKNIDSSLRQQKTTY*
>Syntrophomonas_wolfei_subsp.metylbutyratica_JCM14075T_00314_Cell_wall-binding_protein_YocH
MGIWVKMSKRAYLAAVLGLVLLSVLLFFAUQKPKSIEVDGKLISSVFFSGTVREVLDKNHITLRDRDKIVPSLNAPVKKMKKITVVRAFKVKVVADGCSREVLSIPVSIKEAIKLAGFQLGEKDIIKTTATSLTIPNQEIEIRVTQEQEVKEPIAFQVERTSDNTLERGLSRTIKQGKNGVALNTFKVTYHNGKEAKREVIGSKTLLEPVNKLVALGTITSVSRAGQRLDFREAKYMNASAYTYTGRNTATG
RQPAVGLVAVDPSVVPMGSKLYIEGYGYAFAADTGGAIKGNRLDLFMEERSQCLNWGNRTVKVYILE*
>Syntrophomonas_wolfei_subsp.metylbutyratica_JCM14075T_00315_putative_deoxyribonuclease_YcfH
MLIDTHAHLQDPAFNRDRKEILERAEAAGLEKIICIGYDYETSAEAVQLARQFPEVYAVVGVHPHDAKTLSTEVLAQLYDLARDPRVVAIGETGLDYYRNLSPEEQRKAFKEQIKLAHELYKPLVIHDRDAHQEVLDIIKKEKAGKNEGIMHCYSGHLPLAVELMKEGFYISFAGPLTFKNAKKTQEVAARVALERILIETDCPYLTPEPFRGKRNEPALVVHTARKLAELRHKTEEEIAYITSQNARRIY
RIKD*
>Syntrophomonas_wolfei_subsp.metylbutyratica_JCM14075T_00316_Methionine--tRNA_ligase
VQRKKSYITTPIYYPSDNLHIGHAYTTVAADCMARFKRMQGYDVFYLTGTDEHGQKIEKAAREKGMEPIKYVDGIVESIRQLWETMLITHTDFIRTTEARHEQLVQKIFNKVYEQGDIFISQYEGWYCTPCETFFTERQLAEGNCPDCGREVELLKEESYFFNMAKYADRLLEHIETHPEFIQPESRRNEVVNFIKMGLEDLCVSRTTFQWGVPVPFNQKHVVYVWFDALINYLTAIGYMDDEEKF
KQYWPADVHLMAKDIIRFHAIIWPIMLMALDLPLPKKVCAHGWIMLEGGKMSKSKGNVVDPVVLVKKYGVDAVRYYLVREIPFGADGSYSEEMLVNRINSDLANDLGNLISRTIAMIEYFDGVIPAPGVAEALDEDLINAAERYREATEKLDAFASYIVATRKLVSRANKYIDETAPWLLAKEEAKKERLTSVMYNLAESIRIALILLAPSMPTTSRANRQMEIFDLSQNPSWDEAAEWGRCKA
GSKVSRGEPLFPRIDLKSLEGEQQPVESPEGKKKVEKSQPAVPELVDIEEFARIDLRVAEVIACEKMKKADKLLVLRLRVGEEERTVVSGIAQYYSPEELLGKKVVLVANLKPVKLRGVLSQGMILAAGGDKVEVLLVDQDVPSGSRVK*
>Syntrophomonas_wolfei_subsp.metylbutyratica_JCM14075T_00317_Transition_state_regulatory_protein_AbrB
MLKSTGVVRKVDELGRIVIPIELRRTMGIEEKDALEIYVDSEKIILKKYEPACIFCGNAEEVVNYKGKNLCKSCLTELSKQVI*
>Syntrophomonas_wolfei_subsp.metylbutyratica_JCM14075T_00318_Ribosomal_RNA_small_subunit_methyltransferase_I
LKTGKLYICGTPIGNLDDVSIRLKTILKRVDLIACEDTRMTKILLNRFEIKTPLVSYHEHSEKREKEDYIAIQLLQQGNIALVSDAGMPTISDPGEELIRRALQTGIKVEIPGPSAATAALASGMDTSAFTFAGFLPQRRSRRREELEQLQSEQKTIILYEAPHRIRECLLDIVEIMGAERKLAIARELTKKHEEVRRGTAGELLHHFSSSPPRGEICLLIAPAEDKAAETNWELIIAETEELINLGIDKKEAFKIKA
KDYGIKKNELYNYFIKKRPGTQD*
>Syntrophomonas_wolfei_subsp.metylbutyratica_JCM14075T_00319_tRNA1(Val)_(adenine(37)-N6)-methyltransferase
VDKVNMMPDDQEESLDLVLGGMKLFQSREGYRFSLDAILLAHFPVLDGIKQAVDLGTGNGVIALILSRRGYARKIMGIEIQEKMIKRARKNIAFNHLEERIDLIQADIKNPKEYLPPGAELVVSNPPFWKKGEGKLSLNPEKAIARHELEVELADLVRAAAYILLPGCFCLIQRAERIQEIARLFSANGLVLRRIRPVYPLPERFARMILLEGQKGGAGGLTILPPLFVYESPGVYSAELKQLYGFSK*
>Syntrophomonas_wolfei_subsp.metylbutyratica_JCM14075T_00320_Initiation-control_protein_YabA
MEKTVRELKMEIIELLEVGQLKERVTLERASSPGQPPQLPEPPSWPDIEVESYENLGKVYKEGYHICPAAFGQSRNEECLFCLAFMEKE*
>Syntrophomonas_wolfei_subsp.metylbutyratica_JCM14075T_00321_hypothetical_protein
LAWVVGVRFKPAGKIYHFACEGHDLEAGDPVIVETVRGVIAMGKRDLDGEELQRPLRNVIRKAKPEDSKTWEKNQQKGREALDICKQKIKEHQLPMRLIDVEYTFDLGKIIFYFTAEGRVDFRELVKDLAAIFKTRIELRQIGVRDEAKMLGGIGACGRILCCNCFLGEFQPVSIRMAKEQNLSLNPTKISGICGRLMCCLKYESELYDTATNGENQHGKNSSRAKNDNSGTDS*
>Syntrophomonas_wolfei_subsp.metylbutyratica_JCM14075T_00322_DNA_polymerase_III_subunit_gamma/tau
MDIFFSIVGQERASSLLQKSLETGTISHAYLFLGPAGVGKMSCAQAFAQAILGRDDNDAMVFFRERIHPDLLILEKEGRSVISKEQISQLEPWLGLKPYRASHRVAIIRDSQLMSNEAANALLKNLEEPPEYAVIILIADQNILETIVSRCQLVRFNSLADKDIEQLLIRNREVEPLKAAQAASLQGGSVATAIRFAQQEDYEKIWETSRNIITELSGEEIVEVYKSAERMLEAPELIVNLLISLLRDINIYQQTRR
EELLVLPGSSELASSRRSVQPQHLTAALDKIMNLKNYYRSNVNSLIIVNNISYEIWQAFNS*
>Syntrophomonas_wolfei_subsp.metylbutyratica_JCM14075T_00323_hypothetical_protein
MKIEQGKKELRGFSLLGKADAREIKRGNASSFEQFLSQKREVESQLRNVQEIIKKLDKLEDQLKHKLNLNDLMLYRKLIRNFLQEATAKAYLLKKERGRNRRGRTMLTTISIVDQEAEQLINDFIGKKREAVEILNTLDKIRGMLVDLMI*
>Syntrophomonas_wolfei_subsp.metylbutyratica_JCM14075T_00324_Thymidylate_kinase
MENKGLFISLEGIDGCGKTSVKQGLLQYFSEFPVLSIREPGGTLISEKIREILLNEENRE ITARTEAFLYAAARSQLVEELILPALLRGKIVLADRYLDSTIAYQGYGRGLDLGFLTTLNQLGTSGLKPRLTLLLDIDPAEAQRRKKKDIPDRLEKEGIEFQSRVREGYLKIAREEPERIKLLDAGKDFEKVLQEAIKYIEDIWPAKSWP*
>Syntrophomonas_wolfei_subsp.metylbutyratica_JCM14075T_00325_Arginine_decarboxylase
LQHKATPIYSALKSYMAENNLRLHMPGHSGGKGFKVEELRAVAAMDLSEVPGLDDLHLPQAIKSARLLAADAFGAGQSLFLVNGSTSGIHALFSLPLPEGTKVIVPRNAHRSFYGGMVLSGALPVYLPGCLAPGLGVVLANSPEDISHLLQKRPNVEAVLLVDEAHGGHFPHPAYPRPALQCGADAVVNGLHKTLPVLNQGACLHLSAEFTEWEQVLA
AWSLLTTTSPSYPILASIDLARQLMVEKGFSLLERAACLSQNYKSRINQIKGLHFCFEEEELKALPGVSGVDPLKLLISTQELTIDGLAVAKVLRQKYRIQVELAAPGIILAMMSIFHEGDDWERLYRALEELARDYRAPVTLAEGEVPPLLIAPREAYLAAKSLPLEECRGAIAGEIIAAYPPGVPCLLPGEIDDTMLDYLRYLKKSGLGLQGPQDPALNYVKIIE*
>Syntrophomonas_wolfei_subsp.metylbutyratica_JCM14075T_00326_Inhibitor_of_sigma-G_Gin
MLTQNAFAPLEKKGILPCCICNEVPAEGICGGMKIKRNFLCQKCEEAIVKLTAGSADYHEILSRIKALFG*
>Syntrophomonas_wolfei_subsp.metylbutyratica_JCM14075T_00327_hypothetical_protein
MDATPGEGEQAKGGEREQEGRESAISHGREIGAVPLEPRDKPLVLPMRLRAVYLCKP*
>Syntrophomonas_wolfei_subsp.metylbutyratica_JCM14075T_00330_2-oxoglutarate-acceptor_oxidoreductase_subunit_OorD
MPKEMEAISTRMEKAIFHIFPDVCKGCGLCKEKCPNQTLVVWSEKLGVYGTPTVKPKDEDSCIACMICEMVCPDCAIYIERLRKKKAVG*
>Syntrophomonas_wolfei_subsp.metylbutyratica_JCM14075T_00331_Pyruvate_synthase_subunit_PorC
MSLIKIALAGEGGQGVQSVAEILAEAAYNENKQTIYIPNFGLEQRGGVSIAFIQVSDERIGAPKFNKADVVVALSERAIGRTSGYSDMNTLFVYDSGFMVKPEELPRAKKIIGIPAVDTANRQLHPKVRNIIIMGAVIGLTNVVSFEAAKEALESKLAYKFEKNPDLRELNFKALAIGKEMAEQSLKEGVGNNA*
>Syntrophomonas_wolfei_subsp.metylbutyratica_JCM14075T_00332_2-oxoglutarate_oxidoreductase_subunit_KorB
MGMIAPAIPKSWYLPSKPHKFCPGCGHGIVLKALGETIDELDIQDQVVFGCDIGCSLLSWNFFACDSTQTHHGRTTPVMVGIKRSRPELITIGYMGDGGGYSIGSQHLLNSAVRNEKVTLILVNNTNYGMTGGQMAPTTLPGQKTETSPFGRDVDLTGYPTRGPEFIAELTPDSAYVARATVSKYKQLRRFLRNALRNQMEGNGFSFVEVLSTCPTNWRTNAQQTWHFLEDEMEQYFPIGEIKT
PFGKGDK*
>Syntrophomonas_wolfei_subsp.metylbutyratica_JCM14075T_00333_2-oxoglutarate_oxidoreductase_subunit_KorA
MPQIFEASKKAFITGNEAIAWAALAAGAEIMFGYPITPQNEVMHYWTRLAPEYGKGFLQTEDELSAGFTVCGAVQSGKKAFTATAGPGNVLMQEPFGMAEAMRLPLFAVIQQRGGPSSGTVVYSQQEVTLTTYGGNGEGHRIVYSTATHQEIYDYTIKGFNTAWKYRFPTFLLGDGYQAKMREPLEMYNPEEHGIEMVKTYPLVGLGDGERPQHLICLRNEILEEVYVLFAAEYEAITP
ELVEYEEKGCEDAEIIILSHGVVSRAADDAVKILRAQGIKAGYFRPITLRPFPQKQLREAINRAKRPAKLLIAESAYGQLGRLITKQEIYGETVAIENLFMPGVGIIDYDIINKVKSIM*
>Syntrophomonas_wolfei_subsp.metylbutyratica_JCM14075T_00334_hypothetical_protein
VGEIYASGKSEVAINRALELEKRGQIEFTPLYLRTLKNDLEKGLAKLVAFTRAQSFLGLETGAMLNPAARWALKQEGDIILDVGYGVFGAQTLNLVEGAYESSIKMAVINYSRPMTGNRERIRSYIKNLGRVDAIVANTHMGDESTIDIIKAGNQEIFIVAAELGIPIAYQVLIDEIAKLKDSGEKNWEIPVKYIKRFMPTAIW*
>Syntrophomonas_wolfei_subsp.metylbutyratica_JCM14075T_00335_pro-sigmaK_processing_inhibitor_BofA
MEWVNVIIAALFFLAIFYIILQVLFKPVKLLWKLAINSLIGLVLLLISNYIGAYFQFDLPINIITVLVAGFLGLPGILLICFQLLVR*
>Syntrophomonas_wolfei_subsp.metylbutyratica_JCM14075T_00336_hypothetical_protein
VKICSSIWNWACKYLVLKEEDESKKEVSLEDLLQDAHREVQEAENLFDRVEDEEMIDYAILNLQAAEKRYNYLLTKIRQGKAEEFLES*
>Syntrophomonas_wolfei_subsp.metylbutyratica_JCM14075T_00337_Recombination_protein_RecR
MRLARPLENLIDQLLKLPGIGPKTAQRLALYLKPETEARQLAQAIVDCRQKVFPCSRCGYLTSDPCIICSDESRDKSLICIGEESDVIAIERTGRYQGVFHVLNKDFNLMEDKGLEDLNLQPLEERLAGSEIKEVVLALNPDMDGEVMSRFLAAVAGSFGVKATRLAHGLPVGGDIEFADEITLSRALEGRKEL*
>Syntrophomonas_wolfei_subsp.metylbutyratica_JCM14075T_00338_Nucleoid-associated_protein
MKFNPGGGSMNKMMKQAKQVQEKIVKMQEELRERREIEASSGGGVVTVKVNGKGELISIQIKPEAVDPEDLEMLEDLVLAAVNEGIRKSQEMVSGEMAKITGGFNIPGL*
>Syntrophomonas_wolfei_subsp.metylbutyratica_JCM14075T_00339_DNA_polymerase_III_subunit_gamma/tau
VAYLALYRAWRPRRFAEVVGQEKTVTALRNAVREGKLSHAYLFSGPRGTGKTSIAKIIAKAVNCENLSEGEPCNQCSSCQDINNGNFMDVIEIDAASNRGIDEIRDLREKVRYLPAQGKNKVYIIDEVHMLTTEAFNALLKTIEEPPDAVIFILATTESHKIPATIRSRCQVFNFRRLTTAEIMERLSDVAANDGIMLEPEALLLLARRANGGMRDALSMLDQIYSYKGNKISKQDVLEVMGLVDDAFM
AQLIAAVLSQDTPSIIGILGTAFSQGKEAQQLAEAAMFLRDFLLYLAKDDALEVVAGESQAFFEQKKEQVEAQSQLLRFLKLIELLSEAQRIMRLLRLIMMDTADKLRFSEQQKLLELIAFMEIATLFSEEKSAQPSFLEEKKPKKEVSPRAKASSNEAQDALWNKILAAVEKKKIPTHALLVQGKLLGIKDDTVYIAYKKGKAYHKEKFVKEREGNRAEIIESLKKLLGGRGSLRGERGKKFVEGKIKARLLGKEL*
>Syntrophomonas_wolfei_subsp.metylbutyratica_JCM14075T_00343_putative_xanthine_dehydrogenase_subunit_A
LQSELMRILAEKQFLKENIALITVISSSNPSPRGCRAGEMMLVDRFGYIGDGIGDSLIEARRQAIGEISGIRRVILPSSEEGMTEVFINAEPLIIDRQLIIVGSGNVANVVYRIASIVGYSITVIDNRAETLSRRFEAEILIGDIVELLYMVKNDENTSIVLSYHHEFDEAALLTVVYSPARYIGILGNKHKVFAYFSKLNELNIAEELINRVHVPVGIDIGGERASEIALAIVAEMQAIKYGRAGGFRILQSETIK
VERDELP*
>Syntrophomonas_wolfei_subsp.metylbutyratica_JCM14075T_00344_Ribosomal_RNA_large_subunit_methyltransferase_L
MDKIELIATIRTNAALEEQLVERELGSLGVLKPFLAPEIREKVVENARVKKALFADICRANLWLRSADRVLVKMGEFPARSFTELFDQTRELPWSDWLPANANFPVQGKSIRSKLFSVPDCQAIVKKAVVESLKGRYHQEWFEESGPRYTIEVALLKDIATLTLSLDTSGAGLHKRGYRQLSSAAPLKETLAAAMIDLSRWHPDRLMFDPFCGSGTIPIEAAMIALNRAPGLKREFAAEKWPVIPQKHWLDARSEANDLLRK
DLTLKIRGTDINKEVLSLARYHARQAGLEGHIDFHQQMPVDQLQTREKYGFLVCNPPYGQRLQDLDLSSVEKLYRDMASVFKKNLQTWSFYILTAHKDFEHIFGRRADKRRKLYNGRIETHFYQFFGPKPPRKHEAEMLANDY*
>Syntrophomonas_wolfei_subsp.metylbutyratica_JCM14075T_00345_hypothetical_protein
MFFDCGRARTAQEANYASLAGDVGISEPAAKQWLSILVSSGIVYLLYRVKPGLRILRPAMKKFKNTLKRLIKRPKMYFLDTGLCAYLTKWTSAETLEAGAMSGVFFETWVVTEIIKSYYQGRNLYGNVYYLRDSNGEEIDLLIIEDNKVYPLDIKKSANPGREAIKHFAVLAKTPELIAGGGIICLCNDLIPINELYWFIPVKVL QLLG*
>Syntrophomonas_wolfei_subsp.metylbutyratica_JCM14075T_00346_HTH-type_transcriptional_repressor_Bm3R1
MPISLEKQNKERRQRIIQCSLDLFIEKGYNTSIRDIIALSGVGTGTFYNYFVDKEDILKNLILEDFAKQIISSISEYVLVEKDLYERIETKRLTMEVFAQNETLSEIYSRNIEYGKIKFSNPIVSPIAHSILAMEKFSIHKWVVLKAITKEMIEMVLSFHKTLAVRGMTKPLLLVVND*
>Syntrophomonas_wolfei_subsp.metylbutyratica_JCM14075T_00347_NADPH-dependent_FMN_reductase
MKKVLGLVGSNRKLGNSELVKEIMSNIPEKCKRELRLTDLKIAPCKACYRCLQPDKACPVRDDFNFVIEKIRAADALIGVPVYFLGPHGYYKMLTDRLIGQNHSPDTQGKPCIIVMPYGSEGWYGKIACMVMPKLLRMKLVDCWQIYAPLPGESLLNPENICPARTLGERGHAGSKECPICGSDLFRLINENQVECPICGNRIKNNFNPDFSDSDCDRFSDHEMDEHFKGWLLEM
KDRFSVEKNHLKELQKGYRNQNWWIKP*
>Syntrophomonas_wolfei_subsp.metylbutyratica_JCM14075T_00349_hypothetical_protein
VLTISEVAGRFNISNRQVHELMDYGYLTVAQVERKDNRGISFKIEEIEETLDIPSLLADIKEKRERNEKPRYQGSSDLRKIIKAFKKNYYDRFEKIEEYPEAELLKACFYLFHLNHYAKTYPEISKSLYQLKARVLEKVYRENQAKFKVIYLLGADKKKVWLCDCKEAHSIIGPFSLSYNRFIREEAYCSKCYIQSVEKEYYSLMEFILRVGDYRFIFHSPRSLAAAWVDNLPELPCEVRREGFYEDMNFQVLYGRRVTA
VEERVFPLEIKGKLMEVLGIRPQIND*
>Syntrophomonas_wolfei_subsp.metylbutyratica_JCM14075T_00350_Laccase_domain_protein_YfiH
MPHWQWADDSEIPYITIPHWREAGVKIAFSSRWGGSSSPPFSELNLGLHVGDEREKVLDNRSRFLASLGQKLENLVCCQQVHGDRVLRVDESLAGSGAWDYESALPGFDAMVSHGPGILGSFYADCIPLFFFDPKKRVVAMAHSGWKGTMGKIAAESVKEMLKLGCLPRDIEVFLGPGIGPCCFQIQPELVLRVKTAFHHHQAILRENAHGYYWDLRESIKQSLQESGIQEAHIDSHLCTACNC
HLFFSYRAEKGRTGRMGAFIGLED*
>Syntrophomonas_wolfei_subsp.metylbutyratica_JCM14075T_00351_RmuC_family_protein
MMEIALIVLVLLGILLFLLPLQLQRMNTLRRDLDKLGAGNLLLLEKNGRREMIGQNLREEIGRNRREEEMNATQRAQGREELSNSFKTFSDSLSARMAEMGNLQQVQLEGFSQQMAHLTQSNEQKLEQMRGTIEERVRELQEKNAQKLDQMRTVVDEKLHATLEQRLGESFKLVSEELQQVHKGLGEMQALASGVGDLKKVLSNVKSRGTWGEVQLGNILEDILAPEQYSGNVAVKKGVERVEFA
VKLPGPDKEGSPLWLPIDAKFPLEDYHRLLEAYDGADLAAIEQAGKNLENRIKAQAKEICSKYIEPPATTDFAIMFLPTEGLYAEVLRRPGLFENLQRDYRITVAGPTTLAALLNSLSMGFRTLAIQKRSGEVWAILGAVKSEFGKFGAVLEKTRKKLQEASNSIEEAARRSRVMERKLKDVEELPEKQANELTSAEGFGEEIAASGDG*
>Syntrophomonas_wolfei_subsp.metylbutyratica_JCM14075T_00354_hypothetical_protein
LRIEQKLWRKTVGSWDHGIPRVDGLMPQLLIRVFGTHKLCQEEVIEEIRGFYPDAHIFGCSTAGEICATSIEDISIAITAEEFEHTRIEGQQQINLNQVQNSFQAGEYLASSLTHKDLSHVLVLSDGLHVNGSELLQGLTSSLPPGIAITGGMAGDGDGFSFEETLVFWDAPPAPDTIAVLGFYGNHLKVGYGSMGGFDPFGPQRLITRSVANVLYEMDGRSALELYKKYLGEHAAGLPATGVFFPLSISGQ
VAGSLVRALVNEEEQSLTFGGDVPTGAYARMMKANLDRLIDGAMSAMDSLAMSRPELAILISCTGRRILKPEGYAGEVEAVRRALGASVSMGGFYSYGEISPFKPGQKPKLHNQTMTITTFKEV*
>Syntrophomonas_wolfei_subsp.metylbutyratica_JCM14075T_00355_Cyclic_di-GMP_phosphodiesterase_response_regulator_RpfG
LENMHSLLRRQLRKQFGNHIPDIPGWDAFLQQVNDAYREFDADRNMLERALELESSELLETLNSQLRAVLEAFPDVFLLLDSQGIIIELKAGSSTELYMSPEQLLGHHIEVIHPDTINSPFAMAVEQVQQGRSKILTFEYPVLLNQQEHYFEARLLPVSGDHVFVIIRNVTERRIMEDQLRYLSMHDALTGLYNRSYFKLETAHLEKLSHRPIGLIVGDIDGLKLVNDTLGHEAGDDLLIKAAHVLKESLRD
CDFVARIGGDEFAVLLSRTSQTGIEGVITRIKNNIVLLNSSNPTPLPLSISLGYAERTDASMSLVELFKEADDNMYKEKLDHRKNFNANLVSTLMSSMDERHFFNQDHVEHMQELVCIMAEALDMSPQISCIKLLAQYHDIGQVGISDAVLFKKGKLSQEEQLQIERHPEIGYRIARLSPKLLPIADWILRHHEWWNGQGYPCGLAGEDIPLECRILALVDAYNAMTSNRPYRKAMSHEEAIEEIKSAG
TQFDPELVRLFLSKIETKFPSPRSKA*
>Syntrophomonas_wolfei_subsp.metylbutyratica_JCM14075T_00356_hypothetical_protein
MKEPFLIPPEQYARELLHTAAAGYLQTGL*
>Syntrophomonas_wolfei_subsp.metylbutyratica_JCM14075T_00357_DNA_binding_domain,_excisionase_family
MKCVYCNKEMDSVVTAHEAKWGKYRLLLDGINLYKCPDCGRKVFSPEEAAMVQNLTKALENLSENKPDFLNILEVADVLRVSKQTVYNMIRDGRINAFKCGREWRFRKEDILDISEGNYNVAARGDTVSTKDPELVRELLGQK*
>Syntrophomonas_wolfei_subsp.metylbutyratica_JCM14075T_00358_hypothetical_protein
LSFPITWVKALAKANRIHVSAHAFDRMGERMIPLDDVMRAIEYGEVIEIDQDGPWHFDVKVLFQKATPDKPQFYVVVAASYPEAEIVTVILPDDDEWELCGTMLRRKKQ*
>Syntrophomonas_wolfei_subsp.metylbutyratica_JCM14075T_00359_prevent-host-death_family_protein
MPQIRPISDLRNNFTEISRVHEGKEPVFLTKNGYGDMVVMSIEQYEAFRTAKHIDSALLEAQQQADSDPTRLEFDKVSVSLRERLTEKMGQTNA*
>Syntrophomonas_wolfei_subsp.metylbutyratica_JCM14075T_00363_Serine--tRNA_ligase
MLDAKLIRTNAEAVLEQLKRRGLSGVLKPFLAPEDEWRKILVEERKNPEKRKSIEDKLRSLVIGNRPLMEEVRQAGQVIEKELDEELAREAPLIRNLIPNLPHESVPVGEDEKSNQEVRRWGEPRQFEFEPRAHWDIGPALDLIDFERAAKLSGARFTVYKGAGARLERAVINFYLDIHCGEHGYREILPPFMVIADCMVGTGQLPKFAEDMFKLEGKEMYLIPTAEVPLTNLYREIEAKDL
PLYLTAYTPCFRAEAGSHGRDTRGVIRQHQFNKVELVKLCEPSNSYEELEKLTQDAERVLQLLGLPYRVVVLSTGDMGFSAAKTYDIEVWMPGYQDYREISSCSNCEDFQARRANIRYRPDPKARLQYVHTLNGSGVAIGRTVAAILENYQQADGSVIIPEALRPYMGGLERINRSEA*
>Syntrophomonas_wolfei_subsp.metylbutyratica_JCM14075T_00364_D-3-phosphoglycerate_dehydrogenase
MERKKVIVSERIADEGIELLKTELEVDYRDGISREELLEIIKDYDQVDALIVRSVTDVDEELISRAKRLKVVGRAGNGIDNIDVDVCTRYGVIVANTPDSNTISAAEQTISLLLSSIRNTAWASEYLKGGTWDRKPFRGVELYGKTVGIVGLGRIGSMVATRLKAFNMRVIAYDPYITDERFTRFGAEKMESLEELVNEADFITVHTPRNEETMHMIDERDLGLAKEGVRVVNCARGGIIKEEALLKGLESKKVLSA
GLDVFEKEPAIDYNPLFQFKNVVTPHLGADTFEAQKRVAQEVAEQVIKALPHNLGNLPYRNVLNIPMLSPEAAAFMRPYMAAAEHLGRLLAHLQEDMSKVIDIRCGIFVAIRPNQWTEEVAAKVLNLGRYNMALLEKDIPSSIMVEKANGMLRLAAEALEIQKRYMSPILRVEKTIEGGSTGERVL LKEIFAVQTIRYLEEALDKYGWIMVDVEIATHAMIHKITIAEKEGAFLGIDNIAKWS
VEEPVLEKLSQLDGVIGVKPILLQL*
>Syntrophomonas_wolfei_subsp.metylbutyratica_JCM14075T_00365_Soluble_hydrogenase_42_kDa_subunit
MIGEQYLLLPGPTPVPERVVRAMSRPMVNHRGSEFKELINVTEGIKQVFRTKNDVIIYPASGTGALEAAVVNFISPGDKVLAVSIGVFGDRFATIAREFGAQVEKLDFPWGEAPDPQVLKERAQDINQEIKAVLITHNETSTGVFNDIAAIKAAVGEHPALFMVDAVSGLAAVGLKMDEWKLDVVVSGSQKAFMIPPGLSFLAFNDRALRVLKQGENKNHKYYWDVSAGLKYLEKGQNPYTPAISLY
FGLEEALKMMLEEGLENILLRHLNYREMVRAAVRALGLKLLAADDYASPALTSVIAPQGIGGNKIRQYMRERFNIAIAGGQQKLDDVFRIGHLGYVREIDLVTALAALEIALLNCGYPIELGKGLKKAQEYISSI*
>Syntrophomonas_wolfei_subsp.metylbutyratica_JCM14075T_00367_Pyridoxal_biosynthesis_lyase_PdxS
MEEKANFTPKTALAQRLKGGVIMDVTTPEQAKIAEEAGACAVMALERVPADIRAAGGIARMADPSVILRIKETVAIPVMAKCRIGHFVEAQILEALGIDFIDESEVLTPADDKHHINKQAFKVPFVCGARNLGEALRRIAEGAAMIRTKGEAGTGNIVEAVRHIRQVNEQLRWVSSFRPVEELMAKAKELGAPYELVQEVARLGRLPVVNFAAGGIATPADAAMMMQLGCDGIFVGSGIFKSGDPMK
RARAIVTATAYYQDPQVLAEVSRDLGEAMVGIDIRSTPEERMQERGW*
>Syntrophomonas_wolfei_subsp.metylbutyratica_JCM14075T_00368_DNA_gyrase_subunit_A
LAQKEIPVIPIDIEDEVKKSFLEYSMSVIVSRALPDVRDGLKPVHRRILYAMHDLGMTHDKPHKKSARIVGEVIGKYHPHGDSAVYDAMVRMAQDFSIRYMLVDGHGNFGSVDGDAAAAMRYTESRMSRIAAELLRDIDKDTVDWRPNYDESREEPQVLPARIPNLLINGSAGIAVGMATNIPPHNLREIIDGIKAVIENPDIIEEELMQIIPGPDFPTGGIILGREGIRQAYRTGRGTIRTRARVEIEEK
KSGKFSIVVSEIPYQVNKARLVEKIAELVRDKKVEGIVDLRDESDRKGIRIVIELRKDVNVNVVLNKLFRHTQMEDTFGIIMLSLVNGEPRILNLKEMIKYYVEHRQEVINRRTIYELAKNRKRAHILEGLKIALDHLDEIIDIIRSSRDRENARGELQSRFNLSEIQANAILDMRLQRLTGLERSKVENEYEEVIKAIADYEDILARPERVLAMIKEDLEDIKRYGDDRRTEISGRDGEISFEDLIVEEDVVITTSHEGYIKRLPLS
NRGVYKRQNPLQTLYRSQKRGGKGHDLGVSATTIREDFADHFAVTMSKDRGRLLVVTMKGHTLAQDWRGRITAAGVKAIDLVEDLAEDSDIIVRRTGNRLLVLKHEIARGWTASRKGRGVRALEVRKQGGTGGIFVKEDREMFLVTQTNSIVRLKGSGIAMGWLKNQRLFINSAEEGAIKPV EMSEHIEKATTFNTAAIEKIDRGTEKSAGNSQKRLVTDIITKGEDSRSLGNFGRTYKAIGRVVRNKRMAK
EDELVILTSEGNIIRLEVEDISQQKRYSRGVLLMRLPEEDRIVGVARFKEKED*
>Syntrophomonas_wolfei_subsp.metylbutyratica_JCM14075T_00369_DNA_gyrase_subunit_B
LLKKEYNASDIQVLEGLEAVRKRPGMYIGSTGPKGLHHLVFEIVDNSIDEALAGYCTEIEVTINQDNSITVCDDGRGIPVDIHPAMGIPAVEVILTTLHSGGKFGGGGYNVSGGLHGVGLSVVNALSRRMEVRVKRDGHHQQDYERGTPVNELRIESGEEQETGTSIHFFADEEIFEEIIFEEIRLLQRLRELSFLNRNLKIALRDEREEEFVSQEFHFYEGKHFLKDFVAYINKEKEKVINDNPIYFEKKEGDMVDIVQA
IQYNSSYSENILYSFANNIRTQEGGTHEIGFKNALTRVINDYAKKNNLKENDSNLSGEDIREGIAAIISVLVKDPQFEGQTKTKLGNTYIRGIVESIVFEHVEDYLNENPAAARKLLDKAISARRAREAAKKARELTRRKNALESITALPGKLADCSNKNPAESELFIVEGESAGGSAKQGRDRYTQAILPLRGKILNVEKARLDRVLSSEEIRNLITAMGTGIGEDFDIKRARYHKIIIMTDADVDGSHIRVLL
TFFYRYMKELIEEGYLYIAQPPLYLGKRGKEIKYFYNDADLNAFLSQTEKWSIQRYKGLGEMDPEQLWETTMDAETRTVLQVTVEDAIKANDIFSALMGDNVEQRRDFIESNARYVANLDI*
>Syntrophomonas_wolfei_subsp.metylbutyratica_JCM14075T_00370_hypothetical_protein
YVYHILGNNYIISYSDIIAILNIEEYPLISHLDLKDVIDVMARIEKCLVSISEKGKESLVICNDLKYLSPISSNTLFKRAANR*
>Syntrophomonas_wolfei_subsp.metylbutyratica_JCM14075T_00371_DNA_replication_and_repair_protein_RecF
LKILNFQIKDFRNLKIKEFQPSPGLNFIYGENAQGKTNILEAIFYLSTGISFRSNKEKTLISYDSSSLQVQARYNNLQHERIIDSSITYGLDAKLLRINKKKTSYNHSDHLRILFTPDDLYLVKGAPYKRRFFLDFLLGQISNEYLFKLDNYRKILKKRNLLLKEEDNSRSSAIINDIFIEIAAQLLISRLNLINVLDEAIQEIYPQINDGDGQLKIRYALSFPVNSGKINLDILKDSLRKQVESEKEKEKRKITLGPHL
DDMHIYLNEQMARLFGSQGQQRNIAICLKLAEIMTFRKIKGFYPVFLLDEVLAELDSERAEKLLKDLILQKSQFFISPTLGFQIFLKPDVLEALLDELRAAKSDAISSSLVKDGCLLIGQ*
>Syntrophomonas_wolfei_subsp.metylbutyratica_JCM14075T_00372_DNA_polymerase_III_subunit_beta
MKFMIEKRDLLATVNTVIRAASNKNTVIPVLSGLLIEAEDPTGLTMTATDMEMGIRASTDVRIEVMEVGAIVLSVKSLVNAHYFPDGSILLPLFPSQIFIENQEISADLDIKYGRSSGFINTYRDYEYEPALPLLKKKIPCFSISQEVLKEALKKTVFAAGTHFRQVFTGVLFDLEGGGKLNVVASDTHRLARYSCNINPDAPEPLSFIIPTRTANEIIRLLSDSPEEINIGLSNNNVSFYKRNFLLLSRLIEGQYPNYEQVIPRSFS
YKVKLDTQVLADTLERARVIPSDDKLKIQYTRFKFEENEVTVNSVTVEEGQEEFRIVNSIKIQVNTITNNTEFVVKDKEGEKELTTEPPVNEKVLTLSIAIQRGTAYFKE[illegible]
>Syntrophomonas_wolfei_subsp.metylbutyratica_JCM14075T_00373_Chromosomal_replication_initiator_protein_DnaA
MSNYKDYSLVWNEVLDSMYQEIGSTSFEIWFSRVKTSSFHDINFCVTVPSDLTKEWIESRYLDIMQNKLRRIDPEVLSMDVRAPEIYSSRTREIIDLTQLVSIKSVLVFEVKSPAE ASNPKYTFDTFVVGNSNRFAHAACYAVAEGSPFKSYNPLFIYGGVGLGKTHLMQAIGHHILSKKPNYLVMYVSSEQFTNDMISSIKDDTTSLFRNKYRSIDVLLVDDIQFLAGKERTQEEFFHTFNTLHESSKQIVISSDRPPNNIPTLEDRLRSRFEWGLI
TDIQPPDLETRIAILRKKAQAGNLNIPEEVGKFIANYDGNNIRELEGALIRVLAFATINNKPLDMNTAVEALKDIMPPPRPRKITIETIQKTVAAHYHLEINEIVSKKRNKQLVYPRHIAMYLCRKLTDASFPQIGEQFGGRDHTTVMHAHEKLVNELLSDRELVAIEELKCKKIDPNIV*
>Syntrophomonas_wolfei_subsp.metylbutyratica_JCM14075T_00374_Ribonuclease_P_protein_component
MLDKKARIRTSREYRNVFEKGKRFSGRFLIVYYVENALNDNRFGFTISKKIGIAVIRNRIKRQLRAIVRKYQGQVRTKHDIVLVARAGIGKSSFEGMEKDYISIMKKAGLFARNANNIN*

>Syntrophomonas_wolfei_subsp.metylbutyratica_JCM14075T_00375_Putative_membrane_protein_insertion_efficiency_factor
LQEMLIILIKIYQKATFFKPASCRFYPSCSNYSIQALKKYGIVKGGWLTVKRLARCHPYNPGGYDPVP*
>Syntrophomonas_wolfei_subsp.metylbutyratica_JCM14075T_00376_Stage_III_sporulation_protein_J
LWHSFVQWFASVIQFMYGITDSLGVANYGLAIILMTIVIKIVLFPITHKQLKSMRAMQEIQPRMKYIQEKYKDDPQTMQLKVMELYKEHGVNPLGGCLPLVIQMPIFIAFYQSLFNFKFAVEEHARFLWIPNIGSSDPWYILAVLAAATTFIQQRVSMVDTKDPTQKSMLYVMPLFMAWIAATMPAGLPLYWVTFNILGILQQLYINWSSKKEKLAVATGTGFQLEMEKETLEESNGGHNTLTKEKV
AGREKGGKKVNGSPNSRKKGKKRG*
>Syntrophomonas_wolfei_subsp.metylbutyratica_JCM14075T_00377_R3H_domain_protein
MEVQIVEKKGKNVDEAIKAALDELECAIEDVSIEVLEEPSKGILGLVGKKPAVVRVSLVKKPEEEVRQVVEDLLNRMKIDYQINQVECEGGRVRVNIIGKDMGLLIGRKGETLNAVQYIIGLIINRQREERIRVLLDVEDYRKKREESLEALALRLSDKVKKTKKNVVMRPMSSQERRIVHTILQTDPQITTFSLGDEPNRKVVISLKK*
>Syntrophomonas_wolfei_subsp.metylbutyratica_JCM14075T_00378_tRNA_modification_GTPase_MnmE
MIGDDIAAISTAPGEGGIAIVRLSGNSVIEKVEQIFQPYRAGIKLSDKEGYSLSLGWLCDEKMEIIDEVLLGLMRAPRSYTGEDVVEINCHGGTLPARRCLETVMRQGIRLAQPGEFTRRAFLNGRLDVSQAEAVIEVIRAKTERGMNLALKQLAGRNSREISLLEDQLIEVNAMLEASLDFPDEVGDLDYSVAQGKLREVKNRIDKLLLAGERAEIYREGINVAICGKPNVGKSSLLNALLRKEKAIVTSVP
GTTRDIIEDYINIRGIPVKLRDTAGIRFTEDLVERIGIERSQEVISEADLVLFILDVGTGIDQEDREIYEKIEKKNKIVLVNKEDLEEKNISETELEQLFPGVKIVRGSIIEETGLEELEESMEKAVLSGQLQSDDMEVMINLRQKNALLTAKRHIEDSLAVMGKVSLDCLGVDIWGALEALGEINGKNLKEEVIERIFHDFCIGK*
>Syntrophomonas_wolfei_subsp.metylbutyratica_JCM14075T_00379_Glucose-inhibited_division_protein_A
MIYKAGSYEVIVIGAGHAGCEAALASARMGCQTLLVTMSIDHIALMPCNPSIGGPAKAQVVREIDALGGEMALNIDKANLQIRTINTGKGPAVQALRAQADKRQYHLEMLKTLFNQKKLDILMAEVEDIELSAARVKAIVTRTGARFECQALVLTTGTYLKGRIIVGDISFDGGPGNQFPAARLSESLKRMGLRLGRFKTGTPPRIDSKSVDFSEMIEQPGDIRPLRFSFISPLINRPQLSCWLTHSNKK
THQIVMDNLDRAPMYTGIIKGIGTRYCPSFEDKVVRFSHKDSHQLFIEPEGRDTDEMYVQGLNTSLPEDVQIEVLKSIPGLENVRIMRTGYAIEYDIIYPSQLKLSLECKTVEGLFTAGQINGTSGYEEAAAQGLIAGINAALQVKDREPLVLKRSESYIGVMIDDLINKEIEEPYRLLTSRAEYRLLLRQDNADLRLTEKGRQIGLVDDNRWMAYQQKEEILEVESNELKFSSFTPADEEMAEFLSYKNTA
AIRDRVSLWELLRRPELSIYDYVGKGWLRDNDPDILEQLEIQAKYEGYIEKQKEQVKRFEKLENKMLPPDLNYDEVYGLSQEAVQKLKIVLPASVGQASRIAGVNPADINVLLIFLEKKRRK*
>Syntrophomonas_wolfei_subsp.metylbutyratica_JCM14075T_00380_Ribosomal_RNA_small_subunit_methyltransferase_G
VEYNVRTFKEMLIEENSRQNLVSRKSLPDELEKHIEDSRSLLKFMDLKGSRVVDIGSGAGFPGLVLAIYCPEGEFLLLESDLKKTEFLQAVINRSGLKNCQVLRKRIEEVGRSELRSSFNFCSCRALAMMNVVLEYGLPLLRPGGKLLLWKGKNYLREIEQAANALDILGGKVVDIFTYSLMAERDRAIVVVEKERDTPAKYPRRVGIPAKRPL*
>Syntrophomonas_wolfei_subsp.metylbutyratica_JCM14075T_00381_Nucleoid_occlusion_protein
MVFKQLERLLGKSSEVKVTGIPVEKLYPNPFQPRKDFDEREMMELAQSIEAYGIIQPIIVRPRDDGFQIIAGERRFRACCLLGIREIPAIIQEMDDERVATVSLIENLQRRELNYFEEANAYNRLINGFGLTQEELARKIGKSQAAIANKIRLLKLPLPVRSLILPERISERHARALLKLNSAEMQLEVVKQIYEKELTVKETEELVEGISRNNIPQEIGRKPKGQSVSMVIRDARIFLNTIRETVKRARQTGIDIVMV
ERDVDSEYEITIRIGKHRKNQQLAL*
>Syntrophomonas_wolfei_subsp.metylbutyratica_JCM14075T_00382_Sporulation_initiation_inhibitor_protein_soj
MAKIIAIANQKGGVGKTTTAINLAASIAMGEQKVLLVDCDPQGNASSGLGINRKRLKNCVYNCLVEGLPAKEVIAATEVDGLEIIPATIQLAGAEVELATRENREHYMANVLKPIKEAYDYIFLDCPPSLGLLTINALAASNSVLIPLQCEYYALEGLSQLMDTILLVRKRLNRSLKLEGIALTMFDGRTNLAIQVVDEVKRYFPKEIYQTIIPRNVRLSEAPSHGQPAVIYDPRSRGAEEYRELAMEVLERAK
KGKRSW*
>Syntrophomonas_wolfei_subsp.metylbutyratica_JCM14075T_00383_putative_chromosome-partitioning_protein_ParB
VQKKERGLGRGLEALLSSDLGFDETQEVTQIEVDEIVLRKEQPRKRFDDKSLRELADSIKEHGLLQPLIVRPRQDGFELVAGERRWRAAQMAGLKQVPALLREMDDVQAAEVSLVENIQRDDLSAIEEAMAYKYMMDNYGYTQEVLAEKLGKSRSHIANMVRMLALPEQVLTMLESQEITAGHARAILSLPGSTEQIAAAEEIVAGRLSVREAEKKARSIIKRKRVEKKRDPVILELEERMESFFSSRTK
VLQKGRGGTIEISFYSLEDLERIVEIIGLD*
>Syntrophomonas_wolfei_subsp.metylbutyratica_JCM14075T_00384_putative_enoyl-CoA_hydratase_echA8
MSDRKLDLLTLDIADSIATVTLHAPPVNALSQLMLDEISSCFSNLSQNKLKVVVLTGNMNCGFSAGANIRELVSQNPQGNRQFFASLYQLFNQLEDIPFPLIAPINRFAMGAGLELALCADIRVMDDNARVAAAGVNMGLVFGTQRLSRLVGLGQAKNMVLTGRQITAHEAYDIGLVQYLSPPGQALNQAWKLAELIAQKSSLSVLGAKKVLNAGFDIPLDKGLHLEFEQLEKMLASEDFQQCTR
AFLNKTAKL*
>Syntrophomonas_wolfei_subsp.metylbutyratica_JCM14075T_00385_Septal_ring_factor
VKIRKKSPRKKTDNNFTFIIIPGKTGKALHLGLARKYIAGSLLLSSVLLSVMITYLFIQSQSEIRSVEKIKMESLKKDETIEILGEEIQNMRKQQEGIGQTQNEIKKLMGIMPEAEVNAKPSRGTMVSGMGGAENEVENILAATRDIKRDINRQENEINSLLSEVSQRGTYYRSRPNQWPVKGEISSPYGWRKSPFRSSKSSFHDGIDIVEAVGTEVLAAGDGKVIFAGWKAVYGKTVEIDHGYGFISRYGH
NSAILVNEGDKVKKGEAIARLGNTGKSTGPHLHFTIMKNNESVNPKNYLP*
>Syntrophomonas_wolfei_subsp.metylbutyratica_JCM14075T_00386_putative_acyltransferase
LWKKARRQNIESLISHGVQVKGEIRSSSSIRIDGNVEGKVEVKGDLIVGEKGKIKGEILVDNLILAGRIEGNIEARGRLEITATGSIMGDASCSLISIEEGGFIDGTSKMIRPQEKAEMKKGEKVSVARSVSS*
>Syntrophomonas_wolfei_subsp.metylbutyratica_JCM14075T_00387_putative_sporulation_protein_YyaC
VELPRLNRINCKDSFHCDDPLCFSKIERSISFFLKDLNQDYSREIVYLCIGTDRATGDCLGPLVGTRLQSLLRSAHIYGTLEKPVHAANLDHFLNIINAEHQNPLLVAIDACLGNADRIGYINIKKGSLKPGTALRKVLPEVGDFHISGVVNIGGFLEHMVLQNTRLYTVYKMADIIAKSLCLAHSSFKRHSLAACQASTLQE*
>Syntrophomonas_wolfei_subsp.metylbutyratica_JCM14075T_00388_putative_membrane_protein
MGYLGAVIGAGFASGQEIIQFFVVYGNDGLKGTLMATFAFAICGGMLLYLAHRHQVSNYQDLLAGLMGNKGGKLIDWLLALFLFLGISTMLSASGAVFEEHLYLPKSLGILLTYLMVIFFLFSGKKGLIFSFNMLVPIKILFLLIVTSYVAFFMESQALGSYSLYLCPVDSRLWIMSGILYVAYNFSLAMVVLSEYQSLSNCREGIAGAVWGGLVLGILVVLSYLSLCKFLPAVMHYEVPMLYLMGRISPGV
KQLYTLILWIGILTTAIANAYGFTQRIADFSGLSFGSCLLWCMTLSLPLAMQSFSALVGKIYPVFGLLGIIISVLFYQGIKDMALLLYYNIRN*
>Syntrophomonas_wolfei_subsp.metylbutyratica_JCM14075T_00389_L-seryl-tRNA(Sec)_selenium_transferase
MNNLKNIPSIDEMLKNERIKQLIEVYQRDFVVDVVRSATEELRRELFLKEEEIKKEKLMQAILVKVEHMVSRLAGGSLKEVINGTGVVLHTNLGRAPLGKGAIDYMKRMASSYNNLEFDLEKGERGERYGHVEEVLTRLTGAEAALVVNNNAAAVLLGLNTLARERIEVIVSRGQLVEIGGAFRIPEVMKLSGARLVEVGTTNKTYISDFAAAINEETALLFAAHTSNYKILGFAQEVPLEELVKLGQEKE
LPVMQDLGSGIFYDKADWGLREEPAVKNCVAAGADIVTFSGDKLLGGPQAGIIGKKPLVEAMKKNQLTRALRVDKLTIAALEGTLLEYLLGSPKKNIPVIEMLALSREELREKAERLLELLEKRTDGLSGIRRLKLVELEDMVGGGAYPTYKIPGFGVEIEFSQPGLEKAAKILRLGNPALLTRRQEDRMLLSVRTLLPGDEIKAADLIAGVVRQKGDI*
>Syntrophomonas_wolfei_subsp.metylbutyratica_JCM14075T_00390_SelB_translation_factor
MKRLIIGTAGHIDHGKTTLVRALTGVDTDRLKEEKQRGISIELGFAPFMLPGGQKAAIVDVPGHERFIRHMLAGAFGIDMVVFVIAADEGIMPQTREHLDIIELLGVKQGVVAITKKDLVDEEWLLLMEEIKEYLAGTALKNSPMIAVSAISGEGIEQLLEEIENMAEQVEEKPVMGQARLPIDRVFTIAGFGTVVTGTLWSGQIKTGESLELMPVQRPVKVRSLQVHGARVTEALAGQRVAVNLQ
GIEVAEIKRGYLLSTPDYLHPSYRVDTRLRLLSSSKRTLKNWNRIRFHLGTEEALGRVVLLDRDELQPGQESYAQIVMEKAVVCQKGDPFVIRYYSPVATIGGGSIIDPHAPKQKRFREEVLEQLVMKEEGSLYDLILQEMEAAEATFTSSDLARKTGHEEEHIKEELEQLLLDEKVGDLKNGEYMSTRSLEKINDEIVKRLQEYHRQYPLRAGYPREEMRSRFFKSINPRSFNAIIKYLEDRGSINSQNNQL
RLAGYSPEPGVKEKHAIEKIQELMDKELFAPPSLEELQQQLELNVEDFGEVVSYLLNQGLLIKLSGDIYFSTQALEEGKKILEEHFGREKELSLATARDLFNTSRRYTLPLLEHYDKTRFTRRIGDIRVKA*
>Syntrophomonas_wolfei_subsp.metylbutyratica_JCM14075T_00391_hypothetical_protein
MSKIGIPRTLAYFIYFPLWKTFFEELGHEVVLSPATSKAILDRGVKEAVNDACIPIKLYHGHAAALAGKADYIFCPRLVSVRSHGDFGTETFCPKFLGLPDMLRLAMDDLPEIIDIRVDLKQGKDELWQLSQKIGKSLGNSGEEVKRAFNRARKRQRQYNSLLCQEMLPLEALRIIEKKIPVPRKEKSNNNYDLQVAVVGYPYLLYDAYINAGLLAILEKEKVKVYTQDMISDRKMNRQAKTLPKSTFWY
FSNRVIYGALHFMQRPEIEGVIHLTAFACGPDSMVDRLLEIESRRRGNRPYLSIAVDEHTGEAGVRTRIEAFVDMLRYRRERK*
>Syntrophomonas_wolfei_subsp.metylbutyratica_JCM14075T_00392_hypothetical_protein
MKISFPHMGYSYIALKWLVNHLGHECIVPPEPSKKTLDLGVRYSPEFACIPFKILTGTYLEVAEKGAEIILTSGGMGPCRAGYYWVMHQYLLDEIGKKVKVIAFEPPLCDLRDFWRKLHRLRVSGGLSVKAFIQVIKVAWEKIKAIDDMEMQSHSIRPYEEKRGDTTRALWEALQFVDEANSEKEVDEARQEGLRVLQEVRRDESRDPVKIGIIGEIYVVLEPAANHYLQIMLGEMGVYTDRSIYLSSYTR
KNTIVNLEGDIFQVARPYLNEAPIGGHGVNSIGETILYARHGFDGVVQLAPFACIPEIVAKSIIPAVSRDFNIPVLTLFIDEQTGKAGVQTRLEAFVDLLEKRRELKRQRERWAI*
>Syntrophomonas_wolfei_subsp.metylbutyratica_JCM14075T_00393_2-hydroxyglutaryl-CoA_dehydratase_component_A
MKKAYLGVDVGSVSTNLVVLDESNEVVAAEYLRTSGQPIATVQKGLYSIGQKLKGQLEIAGVGTTGSARYLSSMIIGADIVKNEITAHAIAASSVVPGVNTIIEIGGQDSKIIILREGVVTDFAMNTVCAAGTGSFLDQQASRLTIAIEDFGNLALQAKNPVRIAGRCAVFAESDMIHKQQLGYNNEDIIAGLCEALVRNYMNNVGKGKEILPPVLFQGGVAANQGLVEAFKKYLEMDVIVPPWYDVMG
AIGAALLARRAVDKKQKSSFKGFNIREITFKTRSFECQGCSNACEVVQFFENDDLIARWGDRCGKWELQAV*
>Syntrophomonas_wolfei_subsp.metylbutyratica_JCM14075T_00394_Leucine-,_isoleucine-,_valine-,_threonine-,_and_alanine-binding_protein_precursor
MRVSKRLVLLVLMLFALGVLAGCGSKQEGGDKAGGDKTAVETIPVGINVELSGDLASYGSNSRNGMVLAIEEINQAGGVLGKELKYIERDCQSKPDEAMNNSAALAGEKIIAQLGPLTSGAVAGSTPVMMQNKIALIAPAATAENVTVEEKTGKTLDYIFRVCYLDADQAKRMAEFALNDLKVKNAAIFGSTSDDYAKGLAKYFEEAFIAGGGKIVAKEGYVNGDKDFKATLTKLRSKNPEFIYVPG
YYTEVALLIKQARDLDIKVPIGGGDGWDSPDMVKVAGVEALNNTYFANHYSVEDPSPAISSFVKAYEAKYKKLPDSFAALGYDSAKILAQAIKDAGEADSAKVAQALAQIKDFPGITGTMSINEKHNPVKKIVVIEYKDGKLVSR*
>Syntrophomonas_wolfei_subsp.metylbutyratica_JCM14075T_00395_LIV-I_protein_H
LLEFLQQLINGLSLGAIYALIALGYTMVYGIIMLINFAHGDIMMVGAYVGFFSIAVLGSNIFAALIFAMLSCAVLGVLIEKIAYKPLRNSSRIAALITAIGVSLFLEYFTLFLLTPQQRVFPEAAFPLHKYQIGGLLISNKDIFIFALAVILMIALQYIIKRTRTGKAMRAVSLDREAAILMGISVDRTISITFAIGSSLAAAAGVMIGIYYNTINPLMGIIPGLKAFVAAVLGGIGIIPGAMVGGFLMGILETMVSGYGSTL
YRDAVAFGVLILILLVKPTGLFGKDTGEKV*
>Syntrophomonas_wolfei_subsp.metylbutyratica_JCM14075T_00396_leucine/isoleucine/valine_transporter_permease_subunit
MEKKRFVPRSFLIEVLVLAGIFTLVQYLIVSGLLNEYYQINLSSMCINIILAVSLNLINGFTGQLSLGHAGFMAVGAYAAVVVTTIYSQPFILGLLAACLAAGLAGLIIGVPTLRLKGDYLAIATLGFGEIIRVILQNIDFIGGPAGILGIPRYSTWPWLFGLTIFTIVIIVNLINSSYGRAIISVREDEVASELMGINTTNYKVLAFVIGALFAGLAGALYAHYFYIIKPSTFNFLKSFDILVMVVLGGLGSTTGAVIAAIF
VTMLTAALQTFPAIRMVLYAVTLVVVMVYRPQGLMGNKEWGRRTWGRIWGEKNEPLANK*
>Syntrophomonas_wolfei_subsp.metylbutyratica_JCM14075T_00397_Lipopolysaccharide_export_system_ATP-binding_protein_LptB
VLDFNFHLEDGELLGLIGPNGAGKTTVFNLITGVYKPDEGNIIFSGRDITGLAPYTICQQGIARTFQNIRLFKDISVLDNVRIALHKDAPYGLLKTLLRTPGFFQGEIEINKKAREYLQIFNLEDKAGEIASSLPYGEQRRLEIARALATKPTLLILDEPAAGMNPNETRELMDLIRWIRKDFGLSILLIEHDMSLVMGVCERIYVLDYGQVIAEGLPEEIRRNKRVIEAYLGEEVG*
>Syntrophomonas_wolfei_subsp.metylbutyratica_JCM14075T_00398_LIV-I_protein_F
VLEVKGIDVYYGAIHALKGLSLEVEEGSIVTLIGANGAGKTTTLKSISGLLRLRNGAIMFKGSDISKVAPEKIVGLGISQVPEGRRVFSTMTVLENLEMGAYLRKDKKGIESDLENVFTRFPRLKERRKQTAGTLSGGEQQMLAIGRALMARPELMLMDEPSMGLAPLLVKEIFEIIKDINARGTTILLVEQNANMALSIANRAYVIETGEIVLEGTAEQLINSEEVKKAYLGG*
>Syntrophomonas_wolfei_subsp.metylbutyratica_JCM14075T_00399_Hypoxic_response_protein_1
MKVKDRMSEDVITVEMNTSLTEAFRLMKENNIRRLPVVDKGRLTGIITLTDLNQAAPSSATSLSIHELNYLLARTKIKDIVPKKQKVLTIGPENYIETAAKIMRENKVSGLPVLEQDKLVGIVTETDIFDALIDILGVNRAHSRIDCFVKDRPGSLAEVTGLIAEKSINILNAVVYFDNKSQRYKMILRIEDLKYEPLLEELQKRGYEIESVIVRESGEE*
>Syntrophomonas_wolfei_subsp.metylbutyratica_JCM14075T_00401_hypothetical_protein
MCYFIGIRSNFLLENYRFIEDTLVLVTYAKHQLQKEHDKYYYLLTLGGCSCDMVKSKDNALNQRIRSFLSKFPGNQESEVMLHWEGGEVIYEIGNNYEKLRPLMKQHIISKEDFLNLYPSLERDSLYVLT*
>Syntrophomonas_wolfei_subsp.metylbutyratica_JCM14075T_00402_hypothetical_protein
LAKTSDLISNFYSISSPDKKWSMIKDFTRAKYSAGLFWEVKSLVKKAKSVARNRMLDPSVLEDIPAKDPHAEYAKKDALFKQRRGAQR*
>Syntrophomonas_wolfei_subsp.metylbutyratica_JCM14075T_00403_Hydrogenase_transcriptional_regulatory_protein_hupR1
MNKKILLVDDDEKILASYRRQLGLRLELFTANNAEKGWETLQKHGPMAVVISDYKMPGVDGIAFLERVKEIAPDTVRIMLTGYADIDMAINAVNQGNIFRFLTKPCPIKDFIYAIAAALEQYRLICSERELLEQTLKGSIKLLIDILSISKPQEFNQLSRLRNLAKRTALHLNVEKPWEVDLALMLSPIGLITIPEYILQKQESGEYLSPDEYKMYRSHPATAQDLLANIPRLENVAEAIACQMKNYPKQDAH
SGLAEKEIPLLGRILKIVLDFNALIKKSWSATYALEYLQKYAENYDPEVLSALIIEVQNLKEWSQEDADGRLVKIKDIEVGMVLLNDLQDDKGLVLLPQGQEITPVLQKRLLNFAEKSRIRETVRVLEPLGDKAAGNPFGEN*
>Syntrophomonas_wolfei_subsp.metylbutyratica_JCM14075T_00404_Sporulation_kinase_E
LIFSKSSEYIKKPFFISSELSGKFLASIIDSLPDAVLVIDIDQKIIAWNRALEKMTGITAAQMLPSPTPFLKYNPEELMGKPIYNLLHSYDIMLLTTAFKKIIKNKESESLEIRIKDSAGDYHWVELVGNPRFNDNSEVDGAIFVGRVVTERKLVEEALKASEERYRELFENANDIVYIQDPGGQYLDFNKAAQAITGYSREELLNISMEHLVAPEYLDLSKKMMKQKINQGGTTCYELEIIGKNRNKIPVEVSSRLI
RYSDQSIAIQGTARDNTKRRQAEKALAAEKERLALILSSIGHGVTTTDNYGIITFINTEAERFFEWKNEYARGKRLLEVVPLVEGYTHLNHSLFFASKDEPHPVDIRIINNLGQISLLSTTVTHIPGNDGNDHGMVFVFRDVTELRKAESQLALSQKLESIGQLAAGIAHEINNPMQYIGDNLSFLNEAFASTFSILDQVKALASFEDISSCILGLEKLLEKSQRELDFFRNEIPQAIQQSLEGVTGVSDIVTALR
NFAHPGSGEKQIANVNHAIMGTVSIAKNEWKHLADLELNLGNDLPAVKMVVNQINQVLLNMIVNSSHSIQNAIEKGLYPKGRIVISSQKNGSYVEIRISDNGMGIPDTIINKVFDPFFTTKEVGKGTGQGLAISHDIIVNKHNGLIELESEVGRGTTFYIRLPIKGPI*
>Syntrophomonas_wolfei_subsp.metylbutyratica_JCM14075T_00405_TVP38/TMEM64_family_inner_membrane_protein_YdjZ
MDLHSLDSITAISSFLKSYGLLAPAVASALFIIQAVFPVFPYVILAAAGGLLFGFKLGFFLAWSGALAGACLAYWICRLAGSEWVIKTIQSRFSYDIREIDTRLAFWSILIARVLPVVPTPIINAAAALAGVPFWTFFFSSAIGKLPTALLYTGLGLCLFQVKDIRLTLLLLGLILLLLVAGRYLGKKHLLSP*
>Syntrophomonas_wolfei_subsp.metylbutyratica_JCM14075T_00406_Colicin_V_production_protein
MHFNLLDYLFIGIILLSAWAGYRQGLVDAIGGLLGTLGGLILAVLYREELAFYLDRHYGFTQALGDLLAERMPAPVCSPEWGAVGKGLGLPEILLDPTAFLAQLLLSLLCFLLILFTSSFIIKLVFAAFNRLLSCGGLDWLNRVLGLLLVTAKNIIIMIFLTGLLYPAINLAARMGSAWAVSINNSLSMSLLAPWFLELFTYGKTLLGIAALC*
>Syntrophomonas_wolfei_subsp.metylbutyratica_JCM14075T_00407_hypothetical_protein
MSRERLTLRVRLVFHPTGVTIKVASHGREIGAVPLEPQDKPQVLPMRSALSGTTDAPFGRY*
>Syntrophomonas_wolfei_subsp.metylbutyratica_JCM14075T_00408_putative_M18_family_aminopeptidase_1
LSEQKENLKKNAWLRIKPADLPAVHDFNQGYRDFLNQVKTEREAVAYIKQAAQGHGFVDLDRVASLEPGQKLFFQQKGKICALVVIGQEAMENGVNMVVSHIDSPRLDLKANPLYEADGLALFKTHYYGGIKKYQWLAIPLALHGVIKKDGQVVSVVIGEENDDFVLSIADLLPHLAKEQMEKKMSEAIPAENLNLLVGSQPLGDTADNPIKKKVLQILQEKYAIEEEDFSSAELQAVPAFQAR
DIGFDRSMIGAYGQDDRVCAYTSLRAALELEAPKRTAICLFADKEEIGSNGNTGLQSLIIENLMAELMSKAGYNNYLTLRKSLANSCSLSADVNAAVDPNYPEVFEKMNCSFLSRGVVLTKYTGSRGKSNSNDANPEFLARIRRLFDDNEVFWQVGELGKVDIGGGGTVAHYMARYGMEVVDLGVALLGMHSPFEVSSKVDVFLAYKAYRVFMQSFCNQ*
>Syntrophomonas_wolfei_subsp.metylbutyratica_JCM14075T_00409_Selenide,_water_dikinase
LIQTIDFFTPMVDDPFIFGQIAATNALNDVYAMGGTPILALNVVCFPECADLQVLRSILDGGLSKVLEAGALLVGGHTVDDNEPKYGLAVSGLVHPQKIIANNGAQPGDLLFLTKPLGNGVIATSIKAEMASGEAYKEAIKWMSMLNREGSQAMIEVGVNAATDITGFGLMGHLYEMAWGSDVQVEVFAEQIPFMEGTLEYAGLGLIPGGAYNNRDYLKDKVEYAGDIDPLIRDLLFSPETAGGL
LIAVAEKKAGELLRVMEKRGSFCNLIGRVRSEHFSPIRVHP*
>Syntrophomonas_wolfei_subsp.metylbutyratica_JCM14075T_00410_hypothetical_protein
MERKSFGLGDIVRMKKQHPCGSFNWEVTRMGADIKIKCQGCGRIVMLPRLQFEKRMIRVEKEAH*
>Syntrophomonas_wolfei_subsp.metylbutyratica_JCM14075T_00411_hypothetical_protein
VDAKNPGNQGFTAGDLVTVKSMPQDERFCIIGFKESAAGEKMAILKALFNDTYIIEKPLSLLQTLLIKGIL*
>Syntrophomonas_wolfei_subsp.metylbutyratica_JCM14075T_00412_30S_ribosomal_protein_S6
MRTYEMMFVLRPDLEEEQVSETKERLQKIIADFGGEFINAADGWGKKRLAYAIDDYVEGIYSLWYFKGKPETVDELDRIIKISENFLRHIIIRQDEK*
>Syntrophomonas_wolfei_subsp.metylbutyratica_JCM14075T_00413_Helix-destabilizing_protein
MLNRVILIGRLTKDPELRYTQNGTAVATFTLAVDRKFKREETDFIPIVVWAKQAENCANYLNKGSLVAIDGRIQVRTYDNQEGQRRWVTEVVAEDVRFMGKPAGSRSSTPDYADKNSSKNFDDNWSDLGREVNMDNIDLVDQHEDEDIPF*
>Syntrophomonas_wolfei_subsp.metylbutyratica_JCM14075T_00414_hypothetical_protein
LKKDRRKKRRQCNWCADKIEVVDYKEIPRLRRYITERGKILPRRITGNCAHHQRQLTVAIKRARIMALLPYTSE*
>Syntrophomonas_wolfei_subsp.metylbutyratica_JCM14075T_00415_hypothetical_protein
MLYGDDCCVLAGLQEKIGLMKKSTAGGVIT*
>Syntrophomonas_wolfei_subsp.metylbutyratica_JCM14075T_00416_MazG-like_family_protein
LKGKVKELDIAQNLKTIEWLKAELVDSVAALFKALLQKGNDAIQDAASSLVIITYLLARRVGVTFSVLDQAIKEKLRTSIKESEEIGSWSGDLSELYKYLESKKRW*
>Syntrophomonas_wolfei_subsp.metylbutyratica_JCM14075T_00417_putative_membrane_protein
LSAFIKLSLISSLLLCLTVLLPPLAFIIAIGWGALLIRGSLTLSQRHIGLIYLLNFSLLYGLAGAGTLLFYAAFFGLPALLMSLLLNRHQGYYSIQRWGVVAAVLGVSLFLGLLYWNNGQIGMEQMESQINNYVQESLKAYEDSGFIGFYESQGLSRAELEKSFKAMTRSIVRHLPALYYVQAIMEVFFMLLLASFLSRKSPDERLKRKAYTEEMMPWPLVWVLIVGLALWLWGRDEMGSLYYLGSNIMVVM
TPIAVYYGLAVLLFEFKRILPSKRKLFATLLIILSLFFPLSAIIFLSLMGLFDSLLNFRKIALEREV*
>Syntrophomonas_wolfei_subsp.metylbutyratica_JCM14075T_00418_hypothetical_protein
MKVILIQEVKNLGKAGEIKEVSDGYARNFLMPRGLAEEATKGKVKESEEKKLKMQRKKEKELSEAEKLKERLQGKTISIKARAGGADRLFGAVTSREISEVLQQEWGIVIDKKKIELPQPIKQLGEYKVKVKVYPSIQAEIKVIVAAE*
>Syntrophomonas_wolfei_subsp.metylbutyratica_JCM14075T_00419_Lon_protease_2
VKQYSVTTEQSQETQLHWRIECLYKNLGLIYGTQGLILRASKLDAIDLMGSQKLEERVVALQRILQEDPTINEVDPLGNLGLALDRLGEKVAEVFARKSIEEQIQQRIQEKMDEKYSEYIRDLKLEVLKEQNSSPENAQTLKKLGRLEKMERSNLSRSALEILRPSCREDIIGQGKALSSIISKLNNPYPQHILLYGPPGVGKTSCARLALEMVKGRKGNPFKEDAPFIEVDGTTLRWDPRESTNPLLGSVH
DPIYQGAKRELAEEGIPEPKLGLVSDAHGGILFIDELGEMDPLLQNKLLKVLEDKRVFFESSYYDPHNERIPQYIKQLFEKGVPADFILIGATTKSKEEINPAFRSRCMEVFFEPLRAEHIRQMVVNSAKKLNINMEDGVAGIIAEYTTEGRGANKILVDAYALAVNENGGEETPLEVRCQHIYQAIRNSRITPRILSRASDSNEVGKIFGVGVSGYQASLIELEAIAFPAEEQGKGNIRFNDTAGSMARDSLFN
ATSLFRKETGENLKNYDLHINIVGGGNVDGPSAGAAIYLAILSAIKGRALRQDLAITGEVSIQGRVKAVGGICEKIYGARQAGIKKLLIPRDNLKDVPDDIKALKIVPISYIKEAYPHVFSPSQAESGGELPICKLAGMGD*
>Syntrophomonas_wolfei_subsp.metylbutyratica_JCM14075T_00420_Adenylosuccinate_synthetase
MSAIVLVGAQWGDEGKGKITDFLAEKANCVVRYQGGSNAGHTVEVSQEKFMLHLIPSGILYPETLCVIGNGVVVDMGKLIEEIDGLQKRGIDTSNLRISLRSPVVMPYHKRIDELEDRYTRIGTTKRGIGPTYADKINRIGFRMGDILTGGECFRERFKAQIEYKNRIMEEIYQEDGFDCQQMLNEVLGQAEHLKKYLADTSYLVYTAIKEGKKVLFEGAQGTLLDIDHGTYPYVTSSHPIAGGACVG
TGIGPTNISKVLGVAKAYTTRVGEGPFPTELNDFQGEILRDKGQEYGTTTGRPRRCGWLDTVILRYAVRINGLTDLAITKLDVLDSFSTIKICVAYRYKGQLLTEFPDNIAILDDCEPEYIELPGWQEDISAASSIEELPPNARAYVAKIEELTGVKAAIIAVGPKRKQTIVNSPLFT*
>Syntrophomonas_wolfei_subsp.metylbutyratica_JCM14075T_00422_hypothetical_protein
MSTIRFKVFCLVLFFTLFISTSTLAASTEYKTFLREYNIEINKEWTIKFNKPLAQEPLNMGQIKEIKVIKNSDGQTVPNPAPEIQPDGASVKLKPPAGGYEYNTAYTLVVEGVKSQQGKVLAQPVKKEFVTAASPQSTLSPENTSVTTDSGITVSLGDYVLEKETELVVTPKQTEENVETGYKIEPYDINLGDMTQLDDFITIRIPYDDSYCEAGQDASRCVGAKYYNETTGRWEDVLYEVDAAKKELVIY
TDHLSRYGVFHVKNEGLRYAYISDVYADLLKVDTETATNALSEYVAKGGADGEIAVGLAASLVSDVFDSGSLSSGLDIVTNPTILASLGDPVFDTALADKAYKKLGNLGKAVAVAVVKIGSTMLKSDASKADILNLYKDVATFALGFSESAALGLAMSGIWVFDYTISTMFEQGMEIKMENIGMVYKHFNDDFRSGPYQARTLKDWRQVLIKIVEDNPNDEAAIKAAIEAEVDRYARVFWDIGADTQA
EVASELGIIRMSYPTSAEIEALVADYKANLYNRLYPVSTSVRNYMQQKAHAEYVKSLNDMRAFYNQYIDFTIVEDVAKGAQPKYAGYVVRFEPLSDAAVKKNWTGILNKDGAISTRFTMLGFIQAGCPNTIALYAPGDKPGEDEPKLRIGFTLKAPQTEVRLGGPDGNFTFVNGSESKIVDWAMATALKQIGSIELKPDGSFSALVAFADDGRQAVNRSNKIQVNNFELSGTFDDFLGTGTATVSFNS
FYFRKDISPLQPANPDDVKEYVTTYEYTDAVTGDLNLRKEDHVVIMSGQLGHERVGFSKLQFHEINLGKEYWGDNPTITDKSGKYSTNVSYNFTLNN*
>Syntrophomonas_wolfei_subsp.metylbutyratica_JCM14075T_00423_Internalin-A_precursor
MKKGFCHNSKLLISIVLAVSLLMTSVPDAFAATINTTSISGVVTGQGGTPLPGILVLLYKNYERISTANTDTNGQYSFGGLSSGSYRMLFSPTSYNQKNGTTYAYQRSPYSSTSLSSPITLSDRQSLSINTQLENAGFISGKVYDPNGKPLTGIMVCEVYEGSTYGRNYTNTSGEYIITQLPAENYKVHFDPAEHNAQYGTDYLEQWYNNKPGSNLADEVAVIQGQTINADAYLQISSGTVSGLKAEACG
NQISLSWSAFPGIQSYSIYRTSHFFGINYQKIDTTTSPSFTDSNLPLSTGYWYKVKPVTAIGEGSFSAPVCTKTGDTIITDPGVIISTVAGNGTAGYSGDGGPATSAQLSTPRGVVCDGAGNLYIVDGGNQRIRKVDTSGKISTVAGNGNHWFEGDGGPATAAGLYDPIGVACDSSGNLYIAESNSNCIRKVDSSGKISVVAGIVTQGWATYEGDGGPATSARLNYPFGVACDGSGNLYIADRGNHRIR
KVNTSGIISTVAGNGTAGYSGDGGLATSAQLKDPVSVACDRNGNLYIVDKDNNRIRKVDNTGKISTVAGNGTGGYAGDGGSPATSAQIWVPYGVTFDNNGNMYIADMNNKRIRKVDPLGIITTVAGNGSWKYSGDGGPAEAAGLCNAVGVACDSSGNLYIADSHSNCIRKVVLGTTPNNTITFPDPNLEAAIRETLDKPTEDILKTDIENITELALNFKGIASLEGIQYFTSLQTLYLAGNGITDLT
PLQSLRNLQHLDISNNAITDLGPLTKLSNLQGLDFSHNQLTDIQALANLTDLCYLDFSYNDGVGVLEPLRNLIGLTDLFMAGIRDSNPAQVALCSVSSNQSTDIGAILAGLKNLENLDISNNELPDITFVNQLPNLKTIDASNNTIVDTTPLETLSNLEKVSLYNNNITSISSLVKIPSLQEINISGNQVGGISQIEQLPNLTKLDLTANPISDLTPLTTLQDTVEVNHEEFTEPYTSWEERTDIPINKTWKI
EFSHAVDTSTINPDTIVVKEQNNQPVTVNISRGTDTNTILLEPPADGYKAGQCYYLFIKKGIGSSDGKVLQNSIRMKFTIAGTDSLAGHKLQSLPIVIQLVGGVLTLRDFCIFTNNILKICDQNKFIEHFIHKTNFTDDLPLAKAIDNPTMLNTARNSPHMFRQTLKVLNLEDKVPASATNDYLQDIAKEIGRFKLCSFPTETQIMGAISDTVEELKQGFVSRNTLTEYQDEKNNVNYLVKNRWVILPG
LRIEGTKTVKVGQTTKLSALANVHYSMKDNQNKWRTVKTNIDASDLTDSLWAAYNNLKADNYNPSDIIATVTQDGTVTGYNPGKAKIAVMYGGKLAICTIKVVK*
>Syntrophomonas_wolfei_subsp.metylbutyratica_JCM14075T_00424_Cellulosome-anchoring_protein_precursor
MPRSLKNIFICSFTFLLLLILTGIAAAAPQQEAIVTAFYDVSSSDANLVYINYMAKRGIISGFPDGSFHPSEGLTRAQAAVLVSKLSGQKANPGNSSFSDIDSSHWAANYINSAAAAKYIAGFPDGSFHPDEILTRAQGISLLLRLSEQSLTEAELPQLQDVDPNHWAAAIATALAAGMIDTENNTFKPDQAFNRGDLTRSLSLLLTKAPDLNRQELIGHLIIKTGSVKHKRDGSEKTQPINTTTTVRAGDT
LITANDGEAEINYPDGSGILLKPNSQLIIKTASGRAYIVKGGKPTAVDQLEIELSSGQVFGALASKYTASNKDTDEETIADNKFSKLVSRDNHSNLAAAQSSPQPWYKSAKKKKNIKVAMPWGEVAINGSFWSNYSSNSSSGMSLLQGEGSLTSGGVTRNLSPGQSSVSSSDGRPPSLPHLMSPSEARDWTQQRSWLGERAAEMQGQQEAIEPGSGSQEPGADPGSHLLTYKLGQALAQTEQTAA
ATASGSSGGGGKQTPDSFIDMESFLKMAIPYITFEQGEDPGYIAKKQGFIKDELRGEVYRDDILYSLLRMTYVPIPPDADLLQLAKEYGIISENDKTLQEENIKITTKTALQMMVNARNAWYERLEAEKPAEQLNLINYYISNSTYTYPQFMFSDDGQVLWYTVRGELKAINVSDASKSDRIPSNGSPKEEYPAQGGESAINIYDNSQVLRAVSIEVAPRRNYVTDKYILYRESPEKPWKRLIEFDLSLTRDY
EIIGFSADNTKLIVKTNFYDNYASLYQVDPETMSKELIYHNPHADAAVELVSVLAGIPVFSLKNPETGELLTAIYVDDKTRLVCLREEMATIMDHVRADLGENHFPVAISPDFDYLVLLHRDDRDYGSYRLYNVESRKDTLLLASDIPVKDVGHTYPVSFKASDGQTVFGYLTLPPGKSPRNLPLIVNVHGGPQARYVWTPNEYALLVSNLDIGVLSVDFRFSIGYGNEYSDSASKNMPLAQQDVFESV
NWAINSGIADPECIGIMGHSYGGYISFYQAAVHPDTYKAVISLMGVWDWTDLGLELIEDDPVPDYHRCSAPEPGTELAQLLSPSSYADKLKSPILIIYAGQDDAVYPSQNIRAIKELTEAGNTPQALYLPDSGHTPDTLENITAVFEEIKSFLNEQMKANP*
>Syntrophomonas_wolfei_subsp.metylbutyratica_JCM14075T_00425_hypothetical_protein
VFSKMKNKSSLWAIIVLAVLACALLVLPAMAEGDGDGSGGGKGVPLGLTSSNPADGQKDVPVKGDIQLTFNKNVIFLGIRDANKTCFSLSAADGSQVPIEVIMADDQLEFEKRRDIALRPLEELKPGTAYVVRISPQLQAKNGSSLGHEVTVNFVTAGKAAAVEKPVSEPVVEKNPAEVKKPVTNLPDNEVKKPAANLTDNASGVSSGDSQAVTEAAGETENKAAAENNSKTGSEDNKEVKSEESR
TVKEPTASAEKAPLKEAEEAEQSPFPVYAVIAGVVLVAGVGYIFLRKGKMK*
>Syntrophomonas_wolfei_subsp.metylbutyratica_JCM14075T_00426_corrinoid_ABC_transporter_substrate-binding_protein
MKMLMKYNTKAILRSLLTVFLLLSLILSSACSKEENRETAPSANTRTITDCIGREVKVPAQISRVACLCPESAYTMAMFGQGDKMVAVVGGVKRDLILTEMYPGIKDLPVPKSSAVINIEELVKTDPDVVFVKKDSFSSEAEVQKLNKSKLSFLVVDFNSMKEQQYAIEMMGLVLGADDKAQKYNNYYQNCVERVQKRVELIPQGERVKVYHAINEATRTDIKGSLSADWLEAAGADNVSINKDLKT
LEGKYFASLEQILLWNPEVILVNETGVANYILTNNQWAPLQAVKNKKVWQLPNGISRWGHPSSPETPLAILWTAQTLYPEKFLDLDMAAETRSFYKDFFGLELSEEVVDRIIAGEGMRIARPQ*
>Syntrophomonas_wolfei_subsp.metylbutyratica_JCM14075T_00427_methanogenesis_imperfect_marker_protein_11
VEIYVAIDDTDSLEFGATGQSANELRRLIEDKGWGKSEAVTRHQLLLHPDIPYTSHNSSMCFKAVIGGHNLDEFITSAAKFLRETSAAGSDPGLCVAVKERIKNLEELLSFGHKAKVEVLSKAEAYKTAENCGLHLSEHGGTGLGIIGALAGIGLRITGNDGRFQGRVQLGNSGETIAVGEIIAQTEIDEVQSMDGQALANDETMVIGEKIKTVLLEGKRILLVYREGGAWHSCNNKQLKNY*
>Syntrophomonas_wolfei_subsp.metylbutyratica_JCM14075T_00428_methanogenesis_imperfect_marker_protein_11
MKIFAAIDDSRQLDGGKAGETASLLGKFIEENGWGKCSIPSRHRLYPHPDTACKKHNTARSFSADIDEQYLKDFIDYACKLVRSTGTPDSNAGLVVAIPELMENLDELIEYAYRVKEGLVSQDEALKFKNKPGLYLFELNGSGRGIIGALAAAGLRITGNDGQFRGKLQIGTGEDYIASVKEIIDKSYVEQIKNMDFENISDSEYVRMGEKVKVVLLDNKYTLMVFPTGIEHPKWQTSTTHMLRVF*
>Syntrophomonas_wolfei_subsp.metylbutyratica_JCM14075T_00429_Cyclic_di-GMP_phosphodiesterase_response_regulator_RpfG
MGNKDKPSTALPNEIKQLQEELAQAREYCQALLWEQAQYQELEKTLQDSRQQFRNIINLLPDATFVVDKQGKVISWNRAMEDLTGVKAEDIAVKGNYAYSLPFYGERRPVLIDLILNPDITLPRQYDCLIKKEETLTAEAFCPKIGKKEGIFVRISASPLYDFTGAKAGAIESFHDISDFRQTLEALRDSEARFRGLAESSPALIFVLQNNRLRYVNARFSPLTEYDEEEYYMDFWDFIHPDFRDPVKSKA
LAWQRGENLFEKYEMKLLTRSGKEFWVEIYANPIQYEGQPALIGSLFDISERKHFEEALRQSEVLYRTIFETTGTAMMIFDKDLMTSLVNSEFEVLSGYSKEEIENKTKWPIFVHPDDRERMKRYHQQRRIDPGLAPSHYEFKLLDKEQKIKDIFITVDLIPGTSDSVVSFMDITERKKAEAQVKYLSFNDKLTGLYNRAFFEEELSRLDTERQLPLSLIIGDVNGLKLINDALGHESGDRLLRKAAEILRNS
CRKEDIIARWGGDEFIILLPKTKPHCAERICERIQEKSRSSSDFPIPISISLGTASKNESLQDVQAIIKEAEDKMYRNKLLESKSARSSFLLSLQQTLWAKSHETKEHCQNVKELAEQLGSRLGLPESELDDLRLLAVLHDVGNIAIPNEILDKPAPLSPNEWEIVKKHPETGYRIALTLPELAPIAGAILAHHERWDGKGYPLGLKAEEIPLISRILAIIDAFDVMTSGRPYKKAMSIAEARSEIRKCAGTQFDPELV
KTFCKLM*
>Syntrophomonas_wolfei_subsp.metylbutyratica_JCM14075T_00430_putative_hydrolase
MIIDTHIHAYPTSDDSKMTLEEIVAQARIIGLDGICITDHDSQEIMARAHEYRKQMGYPIFVGAEVFTWEGDVIVFGLERLPEERVSARYLLDLVNKANGVAISAHPFRQNNRGMGNSIREHKDLHGVEAFNGSTDHANNMKARDLAMELGIPVFGASDAHNTEALGKFATVFPNGLRDEKDLIEAIKMGKVYPVAYNHSADVKWTVIPPIG*
>Syntrophomonas_wolfei_subsp.metylbutyratica_JCM14075T_00431_Spermidine/putrescine_import_ATP-binding_protein_PotA
MLLELKGITKLFDKDNGVRDFNLTVSEGEFITLLGPSGCGKTTTLNLIGGFLQPGRGEIRIEGRDITHLPPEKRPVSTVFQSYALFPHLNVLENVAYGIRFYRKEKKKQALSLAREYINIVRLEGYEKSKVGNLSGGQQQRVALARALVIEPAMLLLDEPLSNLDASLRHSLREELKSLQRQLGITMIFVTHDQSEALSLSDRIVVMDKGYAIQIGTPREIYFYPISDYVAGFIGKSNILTRVGETIMVRPEDI
KMAKNAQGDYTISSIVFMGQHTEYIISAEDTTLEVVVVGKENNNFMQGDRVNVTISHQLGQMGNEDISLVPELK*
>Syntrophomonas_wolfei_subsp.metylbutyratica_JCM14075T_00432_Spermidine/putrescine_transport_system_permease_protein_PotB
MQYSRNMALLKNKIIWKERGNKSPLSFDVLIDRIMLFLVVGSLLLFILYPIFMVISTSVYRHGVFSLDNYRDLLSPVNLKLIKNSLFVSTLSSVLGTFLALCIALYTFLQKERIGALIYRLLMTTLISPPFVSALALIMLFGRRGLITYNLLGLSVNPYGWQGIVLLQTLGLISLAAIMLISALNAIDKRLLLASRDLGASALQTLHRVILPSIYPAILSVLFLLFTINMADFGTPIIIGGKFKVLATEAYMTIFSAADLGK
AAAMSVLLIPPAIIAFYFYRKNLEKNNSLADGVKALGGEGLDFRLPLFIRIALGTITGLFFFLMLLKGYNIFLSAVTINVSGKLTFTWKHIEAFQFAYLPAVYRSLSMAVIAGILASVIGILLSYYTHRRQLPLMKPIEFIASLPYIIPGIFFGLGYIVGFNNHPLLLTGTTAILVLNCTFRHISVGNKAANAAFENLDRRLEMAARDLGASPVRILTTVIFPLLKSTFLLSFINTFTACMTTVGAIIFLVSPRNVVASVL
MFKDITNGHYGLGAVVASVLIIVTVAVNLVAIKYLGREGGKPYVA*
>Syntrophomonas_wolfei_subsp.metylbutyratica_JCM14075T_00433_hypothetical_protein
MVWKGNPLELISEIPAVYYIVEYRGREISHSCNIQGIWRY*
>Syntrophomonas_wolfei_subsp.metylbutyratica_JCM14075T_00434_Phosphoglycerate_transport_regulatory_protein_PgtC_precursor
MMKNWKPLILAMILVMALALTACGKTEPTEQGKTDEKPFAGTTLELLVAYGGADKSFDAFTEATGIKIEWLEMSTGSALAKLQAEDGKTTADIWFGGGVDSYLNARDLGYLEAYKSPELSAIDPQYVDKDGYFSGLALVPAGFIVNEDILKEKGLEAPKTWEDLADPKYRGEISMPNPAISGTSYAIVSGLIQAWGEEKAWKYFEAFDSNIDFYAKGGGEPLEKVGAGEFAVGVVAITGGTFNVGE
TRPTRVVYPQDLIPWTPAPIAIFKNTENLDAAKALIDWYLSYDGQNILREADARIMARSDVDAPEIMSGLDKSKLVKFDLERMGKEREVILEKWKDLVGEK*
>Syntrophomonas_wolfei_subsp.metylbutyratica_JCM14075T_00435_sn-glycerol-3-phosphate_import_ATP-binding_protein_UgpC
MGIELKEITQKYGDLIAVNNANLFIEDGEMISLLGPSGCGKTTLLKTIAGLLPLHQGSILSNEKDISNLPPQARNAAMVFQNYALFPHMTVAENVAYGLKINKCALSLIKKKVEEILDIVELPGLNQRKISELSGGQQQRVALARALVIEPAMLLFDEPLSNLDQNLRVSMRRMIRKIQQEYRITSIYVTHDQEEAMSISDRIVVMKDGVIQQIGLPGEVYLNPRNRFVAEFMGTANLLELNEDDEIIQLLE
CGKWASSRDNCPGGESNNRQKRYLMFRPEDVEVGAEGNFEGTVIWHETIGSVTRVSIDFAGNEIIAEIRNYYKSGINCTIGEKIRFNIDLAGDGHIVPD*
>Syntrophomonas_wolfei_subsp.metylbutyratica_JCM14075T_00436_Molybdenum_transport_system_permease_protein_ModB
MQSRQSFIFKLFYILISLTIIYFVLLPIVSVFVYGLLSGSGTDLSNQILPRIWKYMKNSLQVALFVTVIATSFGLLASITLWRVNFWGRKLMQVLVLMPLISPPFVGTVAFIMLFGKRGLITHSLLGLEISPYGFHGIVTMQSIGLATLAYLLISSAIRKTDVSPEEAARNLGAPEKKVFLTITLPLMIPEITVAAMLVFLTSMADFATPIIIGGAYQTLASDLYIQITGLYNVQMASITGIFLFIPCFAAFFIQRHYSGK
NLVYSQYVSPERIEYQQVKPVVKFMLIGLSSLFVIFVVFQYAFIVIGAFTEHWGYNYAFSLRHLENILSKNLASFVNSVKLAVTVGFISSLLGVLLAYIIKTKDYPLTRQLDLVVTLPAATPGILLGIGYLVTFRYPLLGIGKYVLPGFEPVILLGTGIIIYLICIYRYIYIGMKAGYALMEHVNPNLELAAINLGASERRVFFDIMFPLLKPAFNTAFLKNFTSTMTTLGAIVFLLLPKNKVAVQQIFQIMTSSEIGAAA
MMALLLSLLSLVFLAAFQLLLNYEEIFKRLREGRQWVSSLRK*
>Syntrophomonas_wolfei_subsp.metylbutyratica_JCM14075T_00437_Phosphoglycerate_transport_regulatory_protein_PgtC_precursor
VKKFVSLMLSLVLVFSLAGCGAKTDTNKDNGDVKSTAYVGQDWSSEYYDESMKGTTLNLYGVTDNVRPIVDVFQEDTGIKVEHLTMQNGEILQRLENEGKSGVSIADLWFTGGADTFINASQKDLLLPYKSPSGEVLDAAMKDADGYWHGTSLTLVNWVVNKDLIAQKGLKMPETWDDLLQSGLKGEVSMPNPASSGTAYNVVTAMLEVKGEDGGWKYLDELIDQVPFFTPRGSDPANL
VVQGEAIVGINASNGDRDLEANNPHVALVYPTDGTGWWPQPVAIVKGTKNEDAAKVFIDWILSKRGMETIAKERNAAVARTDVERPEGIIDINTVKLFATDFKANAENRDIILAKWSEYVTAAGK*
>Syntrophomonas_wolfei_subsp.metylbutyratica_JCM14075T_00438_Phosphate_transport_system_permease_protein_PstC
VLIKFFELKFDLASGESLFAASLILAIMILPFITSNSEAALRSVAPEYRQAGMALGVSREYLALRVVVPLARKGMMGALVLAFGRAMGTGTKMF*
>Syntrophomonas_wolfei_subsp.metylbutyratica_JCM14075T_00439_transcriptional_regulator,_AbrB_family
MLTVMGAIKVTQKGQVVIPAEFRKKYGIKAPGHVLLAEREGHLVIMPMPDDPVAGARGMLKPKQPLAVSCAAFKKEEMMLEGEDNE*
>Syntrophomonas_wolfei_subsp.metylbutyratica_JCM14075T_00440_putative_nucleic_acid-binding_protein,_contains_PIN_domain
MSKSYIFDSYAVLSYLQDEPCADEIFALLRQAQDEKIKLYMTWINVGEVYYRVWREYGKIEAERVLDILSAWPLEILVADSELTIAAAAVKAQNKLAYADAFAIGAAILKQAELVTGDPEIKMASQKLGFILYWIDNQKGL*
>Syntrophomonas_wolfei_subsp.metylbutyratica_JCM14075T_00441_six-cysteine_peptide_SCIFF
MKKHLVTVVKPVLFRDGQETNCRECATSCQSACKTSCTVGNQHCKKH*
>Syntrophomonas_wolfei_subsp.metylbutyratica_JCM14075T_00442_hypothetical_protein
LRGLREYDFAAGIHLYRYKEQNILLDVNTGAIHLLDELAYHLVEKLAVLQGDWERALAELEAEFPAEEIAEAAQEIEDAWRNGSLFSEFAVPVVELSRLSVKALCLNMAHSCNMKCSYCFASQGNFGLRPALMSLETAQKALDFLLEQSGERQNLEVDFFGGEPLLVFPVIKDLVAYARERKETGKRFSFTLTTNCLLLNEEVQDFVIDHGIGVIMSLDGRKETHDRHRLLNNGQGSYELVLPKIQE
MLTKEPVSYYVRGTFTRDNLDFSEDLRHMLELGFTCVSLEPAVGADNPLAISRDDLPRVLKEYDRLTDILLEYRESGQDIHFFHYNLDLQKGPCLAKRVTGCGAGIEYLAVTPEGDLYPCHQFVGEARFYMGNVENGEIDYAVRRVFEENQLVNKECRRCWARYFCGGGCHAGAYFQGDISKPHAVSCTMHKKRIEGAIYLDLLKHLPPR*
>Syntrophomonas_wolfei_subsp.metylbutyratica_JCM14075T_00443_Murein_hydrolase_activator_NlpD_precursor
MLNLLQQHRRYTPLGLILGGLIFLFVMSSLGVQMPAYSVFIDQQEKFQTDDPEAVNLALQRIIAQERKRCQQDIDLVNQVEIEKVFAKRENIIPAEEVEKELRKNLQFESLATSIVVDGKAVASVKNRELAEKILNNLKREYSWLDEGEELLEVTFAEKVELRDTKVPSQEIIEPPQAYQLIKIGTNNPEKYLVKEGDSLWLIARRNDMYVNDIVVANNLNSDKLSLGQELLLVKSKPYITVMTQVKGERV
EAIPFQTKVVVDTSAGTSVRVKQEGKNGEKQVVYQATKLNGVLQEKDVKNEKILREAVDKIIVKGNRVVQVASRSGSGNLEWPTYGPINQYYKGRGHTGLDIGARSGTSIRAADAGYVASACYQGNYGKFIVINHNNGLVTRYAHCSSIAVSEGQSVSRGEVIGTVGSTGRSSGPHLHFEVLSGGSFQNPLNYLP*
>Syntrophomonas_wolfei_subsp.metylbutyratica_JCM14075T_00444_Thioredoxin_reductase
MYDVVIIGSGPAGLTAAIYTSRAKLKTLVLESSVFGGNAALIDHIDNYPGFPFGVSGADLMESFRLQAERFGAELRMEEVIGLKNASGAIKTLVTNMQEYQAKSVIIAMGAKRRELEVDGEKEYLGRGVSYCATCDGAFFQGTSVAVVGGGDSAVKEALYLAGIADKVYLIHRREGFRANQTALEKMMNHEKIELRLNQVVKRVEGDALMKSLLLKNLKTGEEERLEVEGLFVSIGLVAGTDLLGGM
LESREGYIVTDDNMMSSIPGIFAAGDVRAKKERQVATAVGEGALAGIAASEYLQE*
>Syntrophomonas_wolfei_subsp.metylbutyratica_JCM14075T_00445_hypothetical_protein
MWGIKSLDAGKGLILLLLLVLLLTLLINKHGYSIMRVMVGQEVNLKTAGFERMQSEHFDIRYMAQDSAYIEMIVQVSEEAYSAVAERFGREPAGKTVVVVYPDTASLAGSIGWNRNESAMGVYWAGSIRILSPGEWIKSDDLEKQFKQEGPMVHEFTHLMVDEITRGNYNRWWTEGIAQYLEKKITGFEFADPFARGRELEYYEFMTLEQKFDELDQQIAYWESLQAVQYIVDIYGEEKLFTVLEEQ
GKGSRLNTALEICLGISCQEFEQGFYQYLQKK*
>Syntrophomonas_wolfei_subsp.metylbutyratica_JCM14075T_00446_UDP-N-acetylglucosamine_1-carboxyvinyltransferase_2
LKDRLIIEGGHPLNGRVRVSGAKNAGLVLLVASTLADGETILDNMPRIRDVEVMVQILNELGVNARWNEDGSLSICPPPAGSMKVKTSYELSKKLRASNLLLGALLGREREAVISLPGGCDIGSRPMDLHIKGIQALGAEVDIEHGFIYARSKTPSGARVYLDFPSVGASENIMMMASRTPGQSVIENAAKEPEIVDLANFLNSMGARIRGAGTDLIKIEGVPELKACRYAIIPDRIEAGTYMVGAAISGGD
VQVENVIPTHLHPIVAKLQETGAIVEETDQGVRVRAGKQVLPVDIKTLPYPGFPTDMQSQMMALLSLAEGSSVIVENVFENRFQIVDELKRMGASIQVEGHTAVVRGVKTLFGARVKATDLRAGAGLILGALAARGETTIEDAIHIFRGYENIEKKLSQLGARVKTK*
>Syntrophomonas_wolfei_subsp.metylbutyratica_JCM14075T_00447_hypothetical_protein
MAVPGQKPLNFVSSVPANGARNVSTRLKEIKLVFDKNVVNDLVWNNNRKQIRMFKGSERIAIRVRRIPDTVDFSKRRNIYVRPVNALAPNSKYTIVILADLASKAGEKLGKSVVISFTTGKKAAVTPTVVVQE*
>Syntrophomonas_wolfei_subsp.metylbutyratica_JCM14075T_00448_Ribosomal_RNA_large_subunit_methyltransferase_H
LKYRIISVGKIRESFYMEGVREYMKRLGPYTSMEFIDGLEEKISPRAREKEIQAILQKEAEKIKRWLDKDEILVVLDLEGQVRSSEEIARQLEKWNASGKSRVTFLLGAAHGLANEIKQQAQESVSLSRLTFPHQMAVLILAEQIYRGFKILKGEPYHR*
>Syntrophomonas_wolfei_subsp.metylbutyratica_JCM14075T_00449_hypothetical_protein

[illegible]
>Syntrophomonas_wolfei_subsp.methylbutyratica_JCM14075T_00450_Putative_electron_transport_protein_YccM
[illegible]
>Syntrophomonas_wolfei_subsp.methylbutyratica_JCM14075T_00451_hypothetical_protein
[illegible]
>Syntrophomonas_wolfei_subsp.methylbutyratica_JCM14075T_00452_Stage_IV_sporulation_protein_H
[illegible]
>Syntrophomonas_wolfei_subsp.methylbutyratica_JCM14075T_00453_putative_nucleotidyltransferases
[illegible]
>Syntrophomonas_wolfei_subsp.methylbutyratica_JCM14075T_00454_Glutamine--tRNA_ligase
[illegible]
>Syntrophomonas_wolfei_subsp.methylbutyratica_JCM14075T_00455_hypothetical_protein
[illegible]
>Syntrophomonas_wolfei_subsp.methylbutyratica_JCM14075T_00456_hypothetical_protein
[illegible]
>Syntrophomonas_wolfei_subsp.methylbutyratica_JCM14075T_00457_NADH_dehydrogenase
[illegible]
>Syntrophomonas_wolfei_subsp.methylbutyratica_JCM14075T_00458_HTH-type_transcriptional_repressor_KstR2
[illegible]
>Syntrophomonas_wolfei_subsp.methylbutyratica_JCM14075T_00459_Macrolide_export_ATP-binding/permease_protein_MacB
[illegible]
>Syntrophomonas_wolfei_subsp.methylbutyratica_JCM14075T_00460_Macrolide_export_ATP-binding/permease_protein_MacB
[illegible]
>Syntrophomonas_wolfei_subsp.methylbutyratica_JCM14075T_00461_Macrolide-specific_efflux_protein_MacA_precursor
[illegible]
>Syntrophomonas_wolfei_subsp.methylbutyratica_JCM14075T_00462_hypothetical_protein
[illegible]
>Syntrophomonas_wolfei_subsp.methylbutyratica_JCM14075T_00463_hypothetical_protein
[illegible]
>Syntrophomonas_wolfei_subsp.methylbutyratica_JCM14075T_00464_hypothetical_protein
[illegible]
>Syntrophomonas_wolfei_subsp.methylbutyratica_JCM14075T_00465_Sigma-W_factor
[illegible]
>Syntrophomonas_wolfei_subsp.methylbutyratica_JCM14075T_00466_hypothetical_protein
[illegible]
>Syntrophomonas_wolfei_subsp.methylbutyratica_JCM14075T_00467_Molybdenum_storage_protein_subunit_beta
[illegible]
>Syntrophomonas_wolfei_subsp.methylbutyratica_JCM14075T_00468_Major_cardiolipin_synthase_ClsA
[illegible]
>Syntrophomonas_wolfei_subsp.methylbutyratica_JCM14075T_00469_Succinyl-CoA:coenzyme_A_transferase
[illegible]
>Syntrophomonas_wolfei_subsp.methylbutyratica_JCM14075T_00470_Carbonic_anhydrase_2
[illegible]
>Syntrophomonas_wolfei_subsp.methylbutyratica_JCM14075T_00471_transcriptional_regulator_BetI
[illegible]
>Syntrophomonas_wolfei_subsp.methylbutyratica_JCM14075T_00472_Inner_membrane_transport_protein_YbhS
[illegible]
>Syntrophomonas_wolfei_subsp.methylbutyratica_JCM14075T_00473_putative_ABC_transporter_ATP-binding_protein_YbhF
[illegible]
>Syntrophomonas_wolfei_subsp.methylbutyratica_JCM14075T_00474_putative_ABC_transporter_ATP-binding_protein_YbhF
[illegible]
>Syntrophomonas_wolfei_subsp.methylbutyratica_JCM14075T_00475_Cation_efflux_system_protein_CzcB
[illegible]
>Syntrophomonas_wolfei_subsp.methylbutyratica_JCM14075T_00476_glucosamine-6-phosphate_deaminase-like_protein
[illegible]
>Syntrophomonas_wolfei_subsp.methylbutyratica_JCM14075T_00477_Putative_glycosyltransferase_EpsD
[illegible]
>Syntrophomonas_wolfei_subsp.methylbutyratica_JCM14075T_00478_Putative_metallo-hydrolase_YycJ
[illegible]
>Syntrophomonas_wolfei_subsp.methylbutyratica_JCM14075T_00479_hypothetical_protein
[illegible]
>Syntrophomonas_wolfei_subsp.methylbutyratica_JCM14075T_00480_Transcriptional_regulatory_protein_WalR
[illegible]
>Syntrophomonas_wolfei_subsp.methylbutyratica_JCM14075T_00481_HEAT_repeat
[illegible]
>Syntrophomonas_wolfei_subsp.methylbutyratica_JCM14075T_00482_Poly-beta-1,6-N-acetyl-D-glucosamine_synthase
[illegible]
>Syntrophomonas_wolfei_subsp.methylbutyratica_JCM14075T_00483_hypothetical_protein
[illegible]
>Syntrophomonas_wolfei_subsp.methylbutyratica_JCM14075T_00484_Protein-L-isoaspartate_O-methyltransferase
[illegible]
>Syntrophomonas_wolfei_subsp.methylbutyratica_JCM14075T_00485_hypothetical_protein
[illegible]
>Syntrophomonas_wolfei_subsp.methylbutyratica_JCM14075T_00486_NADP-reducing_hydrogenase_subunit_HndC
[illegible]
>Syntrophomonas_wolfei_subsp.methylbutyratica_JCM14075T_00487_putative_domain_1
[illegible]
>Syntrophomonas_wolfei_subsp.methylbutyratica_JCM14075T_00488_Peptidyl-prolyl_cis-trans_isomerase_B
[illegible]
>Syntrophomonas_wolfei_subsp.methylbutyratica_JCM14075T_00489_D-methionine-binding_lipoprotein_MetQ_precursor
[illegible]
>Syntrophomonas_wolfei_subsp.methylbutyratica_JCM14075T_00490_D-methionine_transport_system_permease_protein_MetI
[illegible]
>Syntrophomonas_wolfei_subsp.methylbutyratica_JCM14075T_00491_Methionine_import_ATP-binding_protein_MetN
[illegible]
>Syntrophomonas_wolfei_subsp.methylbutyratica_JCM14075T_00492_putative_cysteine_desulfurase
[illegible]
>Syntrophomonas_wolfei_subsp.methylbutyratica_JCM14075T_00493_addiction_module_toxin,_Txe/YoeB_family
[illegible]
>Syntrophomonas_wolfei_subsp.methylbutyratica_JCM14075T_00494_Antitoxin_YefM
[illegible]
>Syntrophomonas_wolfei_subsp.methylbutyratica_JCM14075T_00495_GIY-YIG_nuclease_superfamily_protein
[illegible]
>Syntrophomonas_wolfei_subsp.methylbutyratica_JCM14075T_00496_hypothetical_protein
[illegible]
>Syntrophomonas_wolfei_subsp.methylbutyratica_JCM14075T_00497_Taurine_import_ATP-binding_protein_TauB
[illegible]
>Syntrophomonas_wolfei_subsp.methylbutyratica_JCM14075T_00498_Putative_aliphatic_sulfonates_transport_permease_protein_SsuC
[illegible]
>Syntrophomonas_wolfei_subsp.methylbutyratica_JCM14075T_00499_ABC_transporter,_substrate-binding_protein,_aliphatic_sulfonates_family
[illegible]
>Syntrophomonas_wolfei_subsp.methylbutyratica_JCM14075T_00500_hypothetical_protein
[illegible]
>Syntrophomonas_wolfei_subsp.methylbutyratica_JCM14075T_00501_Cyclic_di-GMP_phosphodiesterase_response_regulator_RpfG
[illegible]
>Syntrophomonas_wolfei_subsp.methylbutyratica_JCM14075T_00502_Aminopeptidase_N
[illegible]
>Syntrophomonas_wolfei_subsp.methylbutyratica_JCM14075T_00503_hypothetical_protein
[illegible]
>Syntrophomonas_wolfei_subsp.methylbutyratica_JCM14075T_00504_Arginine--tRNA_ligase
[illegible]
>Syntrophomonas_wolfei_subsp.methylbutyratica_JCM14075T_00505_DNA-directed_RNA_polymerase_subunit_delta
[illegible]
>Syntrophomonas_wolfei_subsp.methylbutyratica_JCM14075T_00506_metal-dependent_hydrolase
[illegible]
>Syntrophomonas_wolfei_subsp.methylbutyratica_JCM14075T_00507_CTP_synthase
[illegible]
>Syntrophomonas_wolfei_subsp.methylbutyratica_JCM14075T_00508_Lipid_II:glycine_glycyltransferase
[illegible]
>Syntrophomonas_wolfei_subsp.methylbutyratica_JCM14075T_00509_Alanine_racemase
[illegible]
>Syntrophomonas_wolfei_subsp.methylbutyratica_JCM14075T_00510_Putative_signal_peptide_peptidase_SppA
[illegible]
>Syntrophomonas_wolfei_subsp.methylbutyratica_JCM14075T_00511_Yip1_domain_protein
[illegible]
>Syntrophomonas_wolfei_subsp.methylbutyratica_JCM14075T_00512_Sporulation_initiation_phosphotransferase_F
[illegible]
>Syntrophomonas_wolfei_subsp.methylbutyratica_JCM14075T_00513_Fructose-bisphosphate_aldolase
[illegible]
>Syntrophomonas_wolfei_subsp.methylbutyratica_JCM14075T_00514_Transaldolase
[illegible]
>Syntrophomonas_wolfei_subsp.methylbutyratica_JCM14075T_00515_Fructose-1,6-bisphosphatase_class_2
[illegible]
>Syntrophomonas_wolfei_subsp.methylbutyratica_JCM14075T_00516_hypothetical_protein
[illegible]
>Syntrophomonas_wolfei_subsp.methylbutyratica_JCM14075T_00517_Murein_hydrolase_activator_NlpD_precursor
[illegible]
>Syntrophomonas_wolfei_subsp.methylbutyratica_JCM14075T_00518_molybdenum_cofactor_biosynthesis_protein_A
[illegible]
>Syntrophomonas_wolfei_subsp.methylbutyratica_JCM14075T_00519_3'-5'_exoribonuclease_YhaM
[illegible]
>Syntrophomonas_wolfei_subsp.methylbutyratica_JCM14075T_00520_molybdenum_cofactor_biosynthesis_protein_MoaC/MOSC-domain-containing_protein
[illegible]
>Syntrophomonas_wolfei_subsp.methylbutyratica_JCM14075T_00521_hypothetical_protein
[illegible]
>Syntrophomonas_wolfei_subsp.methylbutyratica_JCM14075T_00522_hypothetical_protein
[illegible]
>Syntrophomonas_wolfei_subsp.methylbutyratica_JCM14075T_00523_Sensor_histidine_kinase_CitA
[illegible]
>Syntrophomonas_wolfei_subsp.methylbutyratica_JCM14075T_00524_Transcriptional_regulatory_protein_YpdB
[illegible]
>Syntrophomonas_wolfei_subsp.methylbutyratica_JCM14075T_00525_50S_ribosomal_protein_L31
[illegible]
>Syntrophomonas_wolfei_subsp.methylbutyratica_JCM14075T_00526_putative_metal-dependent_enzyme
[illegible]
>Syntrophomonas_wolfei_subsp.methylbutyratica_JCM14075T_00527_Peptide_chain_release_factor_1
[illegible]

>Syntrophomonas_wolfei_subsp.metylbutyratica_JCM14075T_00528_Release_factor_glutamine_methyltransferase
[illegible]
>Syntrophomonas_wolfei_subsp.metylbutyratica_JCM14075T_00529_t(6)A37_threonylcarbamoyladenosine_biosynthesis_protein_RimN
[illegible]
>Syntrophomonas_wolfei_subsp.metylbutyratica_JCM14075T_00530_putative_manganese_efflux_pump_MntP
[illegible]
>Syntrophomonas_wolfei_subsp.metylbutyratica_JCM14075T_00531_Low_molecular_weight_protein-tyrosine-phosphatase_YwlE
[illegible]
>Syntrophomonas_wolfei_subsp.metylbutyratica_JCM14075T_00532_Serine_hydroxymethyltransferase
[illegible]
>Syntrophomonas_wolfei_subsp.metylbutyratica_JCM14075T_00533_deoxycytidylate_deaminase
[illegible]
>Syntrophomonas_wolfei_subsp.metylbutyratica_JCM14075T_00534_hypothetical_protein
[illegible]
>Syntrophomonas_wolfei_subsp.metylbutyratica_JCM14075T_00535_Putative_F0F1-ATPase_subunit_(ATPase_gene1)
[illegible]
>Syntrophomonas_wolfei_subsp.metylbutyratica_JCM14075T_00536_F-ATPase_subunit_6
[illegible]
>Syntrophomonas_wolfei_subsp.metylbutyratica_JCM14075T_00537_Lipid-binding_protein
[illegible]
>Syntrophomonas_wolfei_subsp.metylbutyratica_JCM14075T_00538_F-type_ATPase_subunit_b
[illegible]
>Syntrophomonas_wolfei_subsp.metylbutyratica_JCM14075T_00539_F-type_ATPase_subunit_delta
[illegible]
>Syntrophomonas_wolfei_subsp.metylbutyratica_JCM14075T_00540_ATP_synthase_subunit_alpha
[illegible]
>Syntrophomonas_wolfei_subsp.metylbutyratica_JCM14075T_00541_F-ATPase_gamma_subunit
[illegible]
>Syntrophomonas_wolfei_subsp.metylbutyratica_JCM14075T_00542_ATP_synthase_subunit_beta
[illegible]
>Syntrophomonas_wolfei_subsp.metylbutyratica_JCM14075T_00543_F-ATPase_epsilon_subunit
[illegible]
>Syntrophomonas_wolfei_subsp.metylbutyratica_JCM14075T_00544_hypothetical_protein
[illegible]
>Syntrophomonas_wolfei_subsp.metylbutyratica_JCM14075T_00545_hypothetical_protein
[illegible]
>Syntrophomonas_wolfei_subsp.metylbutyratica_JCM14075T_00546_hypothetical_protein
[illegible]
>Syntrophomonas_wolfei_subsp.metylbutyratica_JCM14075T_00547_UDP-N-acetylglucosamine_1-carboxyvinyltransferase_1
[illegible]
>Syntrophomonas_wolfei_subsp.metylbutyratica_JCM14075T_00548_hypothetical_protein
[illegible]
>Syntrophomonas_wolfei_subsp.metylbutyratica_JCM14075T_00549_14_kDa_transcription_factor
[illegible]
>Syntrophomonas_wolfei_subsp.metylbutyratica_JCM14075T_00550_Rod_shape-determining_protein_MreB
[illegible]
>Syntrophomonas_wolfei_subsp.metylbutyratica_JCM14075T_00551_Distal_rod_protein
[illegible]
>Syntrophomonas_wolfei_subsp.metylbutyratica_JCM14075T_00552_Putative_N-acetylmuramoyl-L-alanine_amidase
[illegible]
>Syntrophomonas_wolfei_subsp.metylbutyratica_JCM14075T_00553_Cell_division_protein_FtsA
[illegible]
>Syntrophomonas_wolfei_subsp.metylbutyratica_JCM14075T_00554_Transcriptional_regulator_CtsR
[illegible]
>Syntrophomonas_wolfei_subsp.metylbutyratica_JCM14075T_00555_hypothetical_protein
[illegible]
>Syntrophomonas_wolfei_subsp.metylbutyratica_JCM14075T_00556_Putative_ATP:guanido_phosphotransferase
[illegible]
>Syntrophomonas_wolfei_subsp.metylbutyratica_JCM14075T_00557_Negative_regulator_of_genetic_competence_ClpC/MecB
[illegible]
>Syntrophomonas_wolfei_subsp.metylbutyratica_JCM14075T_00558_Lon_protease_2
[illegible]
>Syntrophomonas_wolfei_subsp.metylbutyratica_JCM14075T_00559_DNA_integrity_scanning_protein_DisA
[illegible]
>Syntrophomonas_wolfei_subsp.metylbutyratica_JCM14075T_00560_hypothetical_protein
[illegible]
>Syntrophomonas_wolfei_subsp.metylbutyratica_JCM14075T_00561_H(2)O-forming_NADH_oxidase
[illegible]
>Syntrophomonas_wolfei_subsp.metylbutyratica_JCM14075T_00562_putative_PIN_and_TRAM-domain_containing_protein_precursor
[illegible]
>Syntrophomonas_wolfei_subsp.metylbutyratica_JCM14075T_00563_2-C-methyl-D-erythritol_4-phosphate_cytidylyltransferase
[illegible]
>Syntrophomonas_wolfei_subsp.metylbutyratica_JCM14075T_00564_2-C-methyl-D-erythritol_2,4-cyclodiphosphate_synthase
[illegible]
>Syntrophomonas_wolfei_subsp.metylbutyratica_JCM14075T_00565_putative_secreted_protein
[illegible]
>Syntrophomonas_wolfei_subsp.metylbutyratica_JCM14075T_00566_Glutamate--tRNA_ligase
[illegible]
>Syntrophomonas_wolfei_subsp.metylbutyratica_JCM14075T_00568_Serine_acetyltransferase
[illegible]
>Syntrophomonas_wolfei_subsp.metylbutyratica_JCM14075T_00569_Cysteine--tRNA_ligase
[illegible]
>Syntrophomonas_wolfei_subsp.metylbutyratica_JCM14075T_00570_Mini-ribonuclease_3
[illegible]
>Syntrophomonas_wolfei_subsp.metylbutyratica_JCM14075T_00571_Thymidylate_synthase_1
[illegible]
>Syntrophomonas_wolfei_subsp.metylbutyratica_JCM14075T_00572_Dihydrofolate_reductase
[illegible]
>Syntrophomonas_wolfei_subsp.metylbutyratica_JCM14075T_00573_hypothetical_protein
[illegible]
>Syntrophomonas_wolfei_subsp.metylbutyratica_JCM14075T_00574_Phosphomethylpyrimidine_synthase
[illegible]
>Syntrophomonas_wolfei_subsp.metylbutyratica_JCM14075T_00575_Putative_TrmH_family_tRNA/rRNA_methyltransferase
[illegible]
>Syntrophomonas_wolfei_subsp.metylbutyratica_JCM14075T_00576_Stage_0_sporulation_protein_H
[illegible]
>Syntrophomonas_wolfei_subsp.metylbutyratica_JCM14075T_00579_Elongation_factor_Tu-B
[illegible]
>Syntrophomonas_wolfei_subsp.metylbutyratica_JCM14075T_00580_50S_ribosomal_protein_L33_2
[illegible]
>Syntrophomonas_wolfei_subsp.metylbutyratica_JCM14075T_00581_Preprotein_translocase_subunit_SecE
[illegible]
>Syntrophomonas_wolfei_subsp.metylbutyratica_JCM14075T_00582_hypothetical_protein
[illegible]
>Syntrophomonas_wolfei_subsp.metylbutyratica_JCM14075T_00583_50S_ribosomal_protein_L11
[illegible]
>Syntrophomonas_wolfei_subsp.metylbutyratica_JCM14075T_00584_50S_ribosomal_protein_L1
[illegible]
>Syntrophomonas_wolfei_subsp.metylbutyratica_JCM14075T_00586_Vegetative_protein_300
[illegible]
>Syntrophomonas_wolfei_subsp.metylbutyratica_JCM14075T_00587_hypothetical_protein
[illegible]
>Syntrophomonas_wolfei_subsp.metylbutyratica_JCM14075T_00588_hypothetical_protein
[illegible]
>Syntrophomonas_wolfei_subsp.metylbutyratica_JCM14075T_00589_DNA-directed_RNA_polymerase_subunit_beta
[illegible]
>Syntrophomonas_wolfei_subsp.metylbutyratica_JCM14075T_00590_DNA-directed_RNA_polymerase_subunit_beta'
[illegible]
>Syntrophomonas_wolfei_subsp.metylbutyratica_JCM14075T_00591_Ribosome-associated_protein_L7Ae-like_protein
[illegible]
>Syntrophomonas_wolfei_subsp.metylbutyratica_JCM14075T_00592_30S_ribosomal_protein_S12
[illegible]
>Syntrophomonas_wolfei_subsp.metylbutyratica_JCM14075T_00593_30S_ribosomal_protein_S7
[illegible]
>Syntrophomonas_wolfei_subsp.metylbutyratica_JCM14075T_00594_Vegetative_protein_19
[illegible]
>Syntrophomonas_wolfei_subsp.metylbutyratica_JCM14075T_00595_Elongation_factor_Tu-B
[illegible]
>Syntrophomonas_wolfei_subsp.metylbutyratica_JCM14075T_00596_hypothetical_protein
[illegible]
>Syntrophomonas_wolfei_subsp.metylbutyratica_JCM14075T_00597_50S_ribosomal_protein_L3
[illegible]
>Syntrophomonas_wolfei_subsp.metylbutyratica_JCM14075T_00598_50S_ribosomal_protein_L4
[illegible]
>Syntrophomonas_wolfei_subsp.metylbutyratica_JCM14075T_00599_50S_ribosomal_protein_L23
[illegible]
>Syntrophomonas_wolfei_subsp.metylbutyratica_JCM14075T_00600_hypothetical_protein
[illegible]
>Syntrophomonas_wolfei_subsp.metylbutyratica_JCM14075T_00601_hypothetical_protein
[illegible]
>Syntrophomonas_wolfei_subsp.metylbutyratica_JCM14075T_00602_50S_ribosomal_protein_L22
[illegible]
>Syntrophomonas_wolfei_subsp.metylbutyratica_JCM14075T_00603_hypothetical_protein
[illegible]
>Syntrophomonas_wolfei_subsp.metylbutyratica_JCM14075T_00604_50S_ribosomal_protein_L16
[illegible]
>Syntrophomonas_wolfei_subsp.metylbutyratica_JCM14075T_00605_50S_ribosomal_protein_L29
[illegible]
>Syntrophomonas_wolfei_subsp.metylbutyratica_JCM14075T_00606_hypothetical_protein
[illegible]
>Syntrophomonas_wolfei_subsp.metylbutyratica_JCM14075T_00607_50S_ribosomal_protein_L14
[illegible]
>Syntrophomonas_wolfei_subsp.metylbutyratica_JCM14075T_00608_50S_ribosomal_protein_L24
[illegible]
>Syntrophomonas_wolfei_subsp.metylbutyratica_JCM14075T_00609_50S_ribosomal_protein_L5
[illegible]
>Syntrophomonas_wolfei_subsp.metylbutyratica_JCM14075T_00610_hypothetical_protein
[illegible]
>Syntrophomonas_wolfei_subsp.metylbutyratica_JCM14075T_00611_30S_ribosomal_protein_S8
[illegible]
>Syntrophomonas_wolfei_subsp.metylbutyratica_JCM14075T_00612_hypothetical_protein
[illegible]

>Syntrophomonas_wolfei_subsp.metylbutyratica_JCM14075T_00613_hypothetical_protein
LIKKPDKKTLRQGKHKRVRRKVAGTGERPRLCIFKSLHHIYAQIIDDEKGVTLAAASTLEEGLKGLESKSNVAAAQEVGANIAERAREKGITEVVFDRNGYKYHGSVAALANAARDKGLVF*
>Syntrophomonas_wolfei_subsp.metylbutyratica_JCM14075T_00614_30S_ribosomal_protein_S5
MIDKEQAAPEMIEKVVTIRRVAKVVKGGRRFSFSALVVVGDGEGKVGTGKGKATEVPEAIRKAIENAKKNMLSVPLIDGRTIPHEILGIFGAGQVLLKPASEGTGVIAGGPVRAVLEAAGIKDILTKSLGSDNATNIVHATMEGLRSLKRVEDVARQRGKTIDEIMN*
>Syntrophomonas_wolfei_subsp.metylbutyratica_JCM14075T_00615_50S_ribosomal_protein_L30
LAKHLKLTWTKSFIGCPETQRATIKSLGFKKLQQSLIKEDCPQIRGQINKVQHLLTVEEID*
>Syntrophomonas_wolfei_subsp.metylbutyratica_JCM14075T_00616_50S_ribosomal_protein_L15
VRLEELKAPAGANKRTKRVGRGTGSGHGKTSTRGHKGQKSRSGGGVRPGFEGGQMPLQRRLPKRGFNNIFKKVYAIVNVEDLNIFPDGSEVNPDTLQEAGLVKAIKDGVKILGNGVLEKRLQVKAHKISQQAEEKITAKGGKVEVI*
>Syntrophomonas_wolfei_subsp.metylbutyratica_JCM14075T_00617_preprotein_translocase_subunit_SecY
MLDSFQQAFKVGDLRKKLLFTLFMLLVFRLGAHIAVPGVNPGAMEKLLSGQLFGFFDIISGGAFKRFSIFAMSITPYINATIIMQLLTVVVPRLEELQKEGEEGRKVIVQYTRYGTVVLGFIQAMGMSIALGSRGALMHTGFLSYIIAISLTAGTALLMWIGETITEKGIGNGISLIIFAGIVSRLPGQIAGVGQELAGGIIGYFNILLFIVLALIAISIIAVNEGQRRLPVQYAKRVVGRKVYGGQSTFLPLRVN
AGGVIPIIFAMSLMMFPATIGSWMDPASGLNRFLQEYFSFSSVAYNMVYAALIVFFTYFYVAIIFNPMDVADNIKKYGGFIPGIRPGRPTAEYIDRVLSRLTLAGGVFLALIAILPNFIIGLTGITSLWFGGTALLIVVGVALDTMKQIESHLLLRSYEGFVK*
>Syntrophomonas_wolfei_subsp.metylbutyratica_JCM14075T_00618_Adenylate_kinase
MNIILMGPPGAGKGTQAELIKARFPIPHISTGDIFRDAVNQGSELGQEAQKYMSSGQLVPDEITTAIVKERITQADCEQGFLLDGFPRTTAQAQALDQSLTELGKKIDLAVNIDVPTELLIERLSGRISCQECKRVYNLNFNPPREQGKCDSCGSELVQRNDDRGETVAKRLEVYSQQTRPLLQYYEAQGVLFNIEGSRGSNQVFADIQEKLESL*
>Syntrophomonas_wolfei_subsp.metylbutyratica_JCM14075T_00619_Methionine_aminopeptidase_1
MICLKTRDEIAKMRKAGKLVARVLKMLENKVQPGITTGALNALAEEECRKNSAIPVFKNYPHSRGGRAFPAVICTSLNEEVVHGIPGSRLLKEGDIISIDFAVILDGFAGDAAITVPVGQVDPRVRKLISTTEESLMKGIEQARAGRRLGAVSRAIQEHAEKNCFSVVRDFVGHGIGRDMHEQPPVPNYGKRDRGPVLKEGMTIAIEPMLNMGSCQVYTRSDEWTVVTRDGGWSAHFEHSIAITAGEP
EILTLR*
>Syntrophomonas_wolfei_subsp.metylbutyratica_JCM14075T_00620_hypothetical_protein
VVYSKSGRDKGRMFVVVKLVNERFVLVADGEQRKIENPKLKNIKHLQWTRIKAEEVESYLARGEVPENHIIRKNLKRIQDRGEGGLVDG*
>Syntrophomonas_wolfei_subsp.metylbutyratica_JCM14075T_00621_Translation_initiation_factor_IF-1
MAKEDVIEVEGTVVEPLPNTMFKVELDNGHKVLAHISGKMRMNFIRILPGDRVMVELSPYDLTKGRIIYRFK*
>Syntrophomonas_wolfei_subsp.metylbutyratica_JCM14075T_00622_hypothetical_protein
LARIAGVDLPRDKRIEVSLTYIFGIGRSSSRKILAEAGINPDTRVKDLTEEEVSRLREIIEKEYHVEGDLRRQVNMDIKRLMDLGCYRGIRHRRGLPVRGQNTKTNARTRKGPKRTAGGKKK*
>Syntrophomonas_wolfei_subsp.metylbutyratica_JCM14075T_00623_30S_ribosomal_protein_S11
VARRTTTRVKRREKKNIEHGIAHIKSTFNNTIISITDRHGNAISWSSAGTIGFKGSRKSTPFAAQQAAENAAKAAMEHGLKEVECYVKGPGAGREAAIRSLQAAGLEVSVIKDVTPIPHNGCRPPKRRRV*
>Syntrophomonas_wolfei_subsp.metylbutyratica_JCM14075T_00624_30S_ribosomal_protein_S4
VGRYTDSVCRQCRREGEKLFLKGDRCYSEKCSVSRRAYPPGAHGQGRRQKPSEYGIQLREKQKTRRIYGIQEKQFRNYFRKADRKQGITGDNLLVLLERRLDNVVYRLGFASSRKEARQLVNHGHFFINGHKANIPSMLVRVGDIIEVRESSRESNKFQEMKEQAAYKTPPEWLSVDAEKMTGTVLAYPRREQIDTLVNEQLIVELYSR*
>Syntrophomonas_wolfei_subsp.metylbutyratica_JCM14075T_00625_DNA-directed_RNA_polymerase_subunit_alpha
MLEMEKPRIECVEKNSSSYNGRFVIEPLERGYGTTLGNSLRRVLLSSLPGAAVTSIKIDGVLHEFSTIPGVLEDTTEILNIKKLVLSYTSERKIIRLEQQGPKEVKASDITPDAEVEILNPDLHLASLDEDGKVEIEMTVERGRGYVSSDQQPQKNDDIVGLIPIDSIFTPVSRVNYTVENARVGKRTDYDRLNLEVWTNGSISPEEAISLSAQILIEYLKLFTEIDDTYAEVEIVEKEEEKKDKILEMSIEELELS
VRASNGLKRASINTVGDLIAKNREEMSKIRNLGQKSLEEIERKLELNLSFRKSED*
>Syntrophomonas_wolfei_subsp.metylbutyratica_JCM14075T_00626_hypothetical_protein
VSYRRLGRSDHRRSVLRNSVTSLLKEGKISTTETRAKEIRKLAEKMITLGKRGDLHARRQAAAYIMSDEVIQKLFSDIAARYEERNGGYTRLIKTGYRKGDGAPMVLIELVE*
>Syntrophomonas_wolfei_subsp.metylbutyratica_JCM14075T_00627_Energy-coupling_factor_transporter_ATP-binding_protein_EcfA1
MISLRDLTYFYPGGELPALKNISLDIRENEFLVILGRNGSGKSTLARLLNGLLLPSQGKVEVDGWNTADANQLQIRQKVGMVFQNPDNQIVATIVEEDVAFGPENLGLNPAEIRRRVNDSLRLVSMESYKDSAPAFLSGGQKQKIAIAGVMAMKPRYLVLDEALSMIDPRGKREIMESIRSLHKNEGVAVIMITHDLEEAREADRVVVLEEGKVKTISTPEELFPGHASLLALGLAPLEISHIIAEINRQGIL
LRTDLDMDKLVEEICH*
>Syntrophomonas_wolfei_subsp.metylbutyratica_JCM14075T_00628_Energy-coupling_factor_transporter_ATP-binding_protein_EcfA2
MSLYLENISFTYQSGTPFEKTALSGVSLSINDGDFVGITGGSGSGKSTLLQVLSGLLFPFAGKLCLDTEAINPGQKKSLNRLRRIVGLVFQFPEEQIFELQVYDEIAFGPRNLGFSRSEVDERVREAMNHMGLGFEKYSLRHCHSLSSGEKRRLAIAAILAMQPQILLLDEPAAGLDYEGWQALLQRLNLYNREKGISIVMVSHHYQQLLASCPRIILLDKGRILIDAASSSWLFYLDFLMQQGWELTASQ
ELVYRLMQKGWEFASSPRSPEETAWAVVDFIRRSK*
>Syntrophomonas_wolfei_subsp.metylbutyratica_JCM14075T_00629_Energy-coupling_factor_transporter_transmembrane_protein_EcfT
MNTLILGQYIPEKSLIHELDPRSKIIGTALLSLAVFLSAFPEEQLFILFLVMIIITSKISMRIHYLALKPFLFILALALLIQMLMTPGQELIEFYFIKITVEGLQAAGSLGLRLLTLILANLLSLTTTPMAFTAALERMFRPLKGWGLPVHELVMVMNISLRFIPLLLEEAERIRKAQLGRGADFRQGKLLGRIRRLGAVIIPVFRLSIYRAEMIATAMESRGYCGAEGRTQLNELRMKAFDYVIIILSLGMCLLVT
MTWGRGC*
>Syntrophomonas_wolfei_subsp.metylbutyratica_JCM14075T_00630_tRNA_pseudouridine_synthase_A
MTRIKLLLEYDGRNYHGFQLQKNANTVQAELEKAIYRLSGEQATLVCAGRTDAGVHAWGQVVAFDSCSTIPGDRWAYALNSQLPEDIQVLESQETRAEFNPRFDALKKCYGYLIYRQKKKATFYRHYALCSTEPLKLEAMQEAAGILKGRQNFRSFCASGSSARTFEREIFRCCLSEKGPYLLLRIEADGFLYNMVRIITGTLLEVGRGKYPPQHLREIIASQTRAGPTAPPQGLYLLQVFYP*
>Syntrophomonas_wolfei_subsp.metylbutyratica_JCM14075T_00631_hypothetical_protein
MPKRICLLLITVLTFAHSTARDGLSIPVYPVNQGSNEVQQAFAAMVKDFAIYSSLLEDVDSSGYLISQHANDSDEAINFLSPGFEPQLASDIVEAYTQWIPGNEKLAIVPCDIPVLTQADYNECSFYQPNHSSIIFRSYSGCYSEQDSFLFTVHLSYADKSWKIEKLLELLESPD*
>Syntrophomonas_wolfei_subsp.metylbutyratica_JCM14075T_00632_DegV_domain-containing_protein
LQEKIALLCDSMCDLPRSFIEKFNIKVVSARVIYPHQEYADRVDIQPEEVYARMPQEIPTTAMPPLQEIQEIFDKIRSEGFTHVLAVHISSGLSGTFQAVKLIAKDIQDMKVEVIDTKTLSMGTGWLVLDAARNIANGWSFDKILENQKKLQSKMHLYYVIETLEYLRRGGRIGLVASMLGEFLHLKPIISVNLEGKYFTFCKAKGRKKSIDKLIELVEQSMKDKPFNLAILHGGAKAECFAILERMRNLPNI
REVITSDISPALGVHTGPGLLGVCFHEV*
>Syntrophomonas_wolfei_subsp.metylbutyratica_JCM14075T_00633_putative_DNA-binding_protein
MLEKTEHMIMLKDFYGPLLTLTQQDLMSLYYEDDWSLSEIAENRGTSRQAVYDVLKRAETIALQEYEERLGLVDKFVLTRRGLEEAYALLKKTGCLQGKEIEKAIEILHEIMESV*
>Syntrophomonas_wolfei_subsp.metylbutyratica_JCM14075T_00634_hypothetical_protein
VAFEGLSDRLQDFKKLRGKGKIGEEDVKTAMREVRLALLEADVNFKVVKDFVERVRQRAVGQEVLQSLTPGQQIIKVVHDEMTALMGGSESKLNLAAKPPSIYMLVGLQGAGKTTTVAKLGRNLLKQGRRPVLVAGDIYRPAAIKQLQVLGEQVGIPVFSMGQSNPVDIARASLEYARSQNRDVILDTAGRLHINEELMDELKQIKSAINPHEILLVVDAMTGQDAVNVAESFNRDLGLTGVIL
TKLDGDTRGGAALSVKAVTGCPIKYVGMGEKLDALETFFPDRMASRILGMGDILSLVEKAQANIDQEKAREMERKIRKQEFTLEDFLEQMQQVRSMGPLDELLGMIPGIGKQMKGFKGEFDEREILRIEAIIKSMTQERRDTSIINGSRKKRIARGSGTKVQEVNRLLKQFEESRKLMKQFADLGGKKGKRGGMPPIRFPF*
>Syntrophomonas_wolfei_subsp.metylbutyratica_JCM14075T_00635_hypothetical_protein
VATRIRLRRMGAKKSPFYRLVVADSRYPRDGRFIEELGYYNPSTTPATVNIDEEKALKWLASGAKPSDTAKSLLQKQGIMARFAEIRK*
>Syntrophomonas_wolfei_subsp.metylbutyratica_JCM14075T_00636_putative_RNA-binding_protein_(contains_KH_domain)
VKELVEYIAKSLVDNPDEVEVREEGEREVTSVLELKVAADDMGKVIGKQGKIAKAIRTLTKATAAKEGKRVAVEIVKEG*
>Syntrophomonas_wolfei_subsp.metylbutyratica_JCM14075T_00637_hypothetical_protein
LNSDKLVAIGKIAGTYGYAGWLKVIPLTDFPERFYKLDKVILNQGGKLGTFLVDGLKAHNNFYLFKFSGIDTVEVAQGFQNGILQIDESELYPLPEGYFYHFQLQGLSVYDEEKGLLGELTDIIETGANDVYVVDSPQYGEILIPAIKDVILAVKLEEKRMEIKLLPGLIED*
>Syntrophomonas_wolfei_subsp.metylbutyratica_JCM14075T_00638_tRNA_(guanine-N(1)-)-methyltransferase
MKIDILTLFPEMFVSPFNESIIKRAREQGIITINTNIRDFAQDRQQVDDYPYGGGAGMVLKANVLGTAIEEVKGPDTWVLYMSPQGKPLNQDRVWELARRKELLIVCGHYEGIDERVMAMVDEEISGIDYILTGGELPAMVLVDAIARLIPGVLGDENSAMEESFSCSLLEYPQYTRPASYGEQEVPEVLLSGHHENIRRWRKKQSLLRTLLKRPDLLKKCFDEEEKSLLEEILFHRENKR*
>Syntrophomonas_wolfei_subsp.metylbutyratica_JCM14075T_00639_SpoU_rRNA_Methylase_family_protein
LRKAKVYIALLHYPMYNKRMEITTSITNLDLHDISRVARTYDVDAFFLVHPSNPQQELIDIISYWQDGYGGQYNPDRKDAFNIIKSAETLREGVQEYLESNMGEKVFTVVTTARPLDKTVAYLDARYLANTISYSQLKNMVFNEEKSFLLLFGTGWGIQKEIMESCDYILQPIEAKRNYNHLSVRSAVSIILDRLLGEYWFNL*
>Syntrophomonas_wolfei_subsp.metylbutyratica_JCM14075T_00640_50S_ribosomal_protein_L19
MQNIIKSIEEEYYKKDLPRFGPGDTVKVHVKVVEGTRERLQVFEGTVIKIRGTGLSQTFTVRRTTSGVGVERTFPLHSPKIARIEVTRRGKVRRGRLYYLRQLTGKKARVKEGRY*
>Syntrophomonas_wolfei_subsp.metylbutyratica_JCM14075T_00641_Ribosome_biogenesis_GTPase_A
LTINWFPGHMVKARREIENNLKLVDIALILLDARAPFSCRNSDLEAKINRKKVVMVLNKADLASPEAIRHYMQALEQEGFLVVAMDSLSGKGRQAVLRMAKSAFQEKAEELQRKGRRPRAIRAMIMGVPNIGKSTFLNSLVGKIAQTGAKPGVTRGKQWVRLREDIELLDTPGLMWPRVESEEQGLKLALLDIVGENAYSEYEVGLYLLSILKDKHPQILEKLGIGDAGNQLEEEMLAKIAGKRGYL
LKGGVPDLDKTCQVLLQEFRRGKLGNISLD*
>Syntrophomonas_wolfei_subsp.metylbutyratica_JCM14075T_00642_Capsule_biosynthesis_protein_CapA
VLRRFAIVFIFSLGSLASYYIYQATIGQQEPAEQCTASPVSKIKNTFTPTKKATITLTAAGDCLMHNTQIWSGLQADGSYCFDSFFPEVRELIMEGDYSSINFEAAMAGPDSAYTGYPLFNSPDAMAQTLKEAGFDLVATANNHCLDRGVKGAIRTLKVLEQAGLDTLGTYKSEEDSHTYLIKDIKGVKVAYLAYTYGTNGIPLPDEYSFLINFLDEKEILADIKAVRPQVDVLVLVLHWGLEYNPRPSD
MQRKQAYSFLEAGADVILGSHPHVIQTMEKIRAEKDKFVIYSIGNFISHQIGQERNSGIVLKMKFTKDFSSGNTLLDEVSYTPTFSHSYQEAGKMKFRVVPVEKTIEKIEKGEERILSKNHLPLLQAVLASTRSQLGEGFSRK*
>Syntrophomonas_wolfei_subsp.metylbutyratica_JCM14075T_00643_hypothetical_protein
MQDLYWECKVCEACGVCGKSEEEECRLYDEYYGFGKSPGAKERGSLAVLERDARKLNQANQGVINRLVNGSHFYISSMMPLSRFRESGKKIGDLKVAFISIDVKVMN*
>Syntrophomonas_wolfei_subsp.metylbutyratica_JCM14075T_00644_Nitrogen_assimilation_regulatory_protein
MNLGENSPNPVLLRPEQSIRETVTLFLERNIDGAPVVDENQIIGLFTKTHVYRAIKESMDMLQPIENIMKRDIIGHPDEEIEDVLYPGLGRLPIADETGIVGMITRTDLARVFLESYRNISEEDALINSTHNMIVSVDKQSRIRVFNRAAEKLLKQAENVKGKLIDDVYPTSRLTNIIKSGQVETLQKIMLNNHYFISNRSPIIKDGEIIGAVGVLQDISELEELSRELKYVKELNEELDAIISSYDGLYITD
GNGMTLRLNQAFERITGVNGRRFLGRYVDDIMDIAQEGIVSESVSLLARKETVTIIQETRSGKTILATGSPVFDKNGNIFRVVCNVRDITELNMLKQKLEKVQGLSQHYESQLRTELIRYSGTDKISKSSEMREKLLETVVRLAEVDSTILINGESGTGKELIAETIHNHSARAKKPFIKVNCGAIPENLMESELFGYDYGAFTGAKKEGKAGYFELAHEGTLFLDEIGDLPFNLQVKLLRVLQSKEINRVGGR
QALKVDTRILAATNRNLLEMVQKKQFREDLFYRLHVIPVIIPPLRDRKEDIPSLIVHFIALFNRKYRLNKRICPDVVDILMAYDWPGNVRELENLIERLVVISSDIISRDELPIHLGSAVLDSPQVSVSAIVPLKEAVESVEKQLIERAFAQYRTTRQIAKKLEVNASTVVRKAAKYGISSSSNSNKRT*
>Syntrophomonas_wolfei_subsp.metylbutyratica_JCM14075T_00645_Mu-like_prophage_protein_Com
VNDFRCNKCHKLLGKYRDCLELEIKCPRCGSRNSLAQKLKNENENEREKELCLASIEKRRG*
>Syntrophomonas_wolfei_subsp.metylbutyratica_JCM14075T_00646_Acetyl-CoA_acetyltransferase
MSDYVQKLEVKRAIYPKIAVSGFGKRPDYSGMGRNVGNVKNFREVVCVEACRTPYGVMGGKLKEYDAPQLGALAIKEVLRRTNVSGKVKPEDVDYVYMGQVVPAGNGQIPGRQATILAGLPHTVPTISVNKVCSSGIKTIDLGVQAIMLGKADIVIAGGQESMSNCPYSLPEMRYGRRMGLLNANCVDLMVYDGLWDAFYNRHMAIHGSETDSEFGFTREDNDEWGYNSQMKAVAAMEA
GRLDDEIFPVETNRGKDVLSKDEGPRPQTKMEALAKLPPVFNHKSTVTGKPGSVTAGNAPGVNDGGDVCLLMSRQKADELGLKPLFSIVDYAEVSQQTKDIATVPGLSIKKVLEQNGMTYKDVDLIEINEAFAAVVLVSARTILGMSKEEMFEKVNVNGSAIAFGHPIGATGARIVMTLAYELRRRGGIGVCGICSGHAQGDAMLIKVEA*
>Syntrophomonas_wolfei_subsp.metylbutyratica_JCM14075T_00647_putative_3-hydroxybutyryl-CoA_dehydrogenase
MAIKKIMVIGAGQMGGGIAQVSAQAGYDTVLYQDINMDLVKKGIAIVEKNLTRDVSKGKKSKEDKAAILGRIKPSIKLEDAADCDLVIEAIVENFDIKKQVFSQLDQIAPAHAILASNTSSLPITQIAATTKRPEKVIGMHFMNPVPVMKLVEIIRGLATTDEVYKIIEEASIKMGKTPVWCKDVPGFISNRVLQVMINEALWLYEGVAPAENIDNIMKLGMNHPMGPCELADLIGLDTILSILNVMYTG
YGDPKYRPSPLLKQYVQAGWLGRKTGKGIYDYRK*
>Syntrophomonas_wolfei_subsp.metylbutyratica_JCM14075T_00648_putative_enoyl-CoA_hydratase_echA8
MEFENLILKKEERIAHLVINRPKALNALNQDTLIEIKTAVEDVRDDQDIDVLIISGAGGKAFVAGADITEMMEISALEGREFGLLGQGVMRLIEAMEKPVIAAIDGFCLGGGCELAMACDFRICSEKSKFGQPEVGLGVTPGFGGTQRLPRIVGTGIAKQLLYTGDIINADEAMRVGLVNYIVPSEQLMGNVEKLAQKIALRGQKSVRFCKIAVNEGMQTDIDRAMTVEADLFGLCFATEDQKEGMR
AFVEKRKANFIGK*
>Syntrophomonas_wolfei_subsp.metylbutyratica_JCM14075T_00649_Acyl-CoA_dehydrogenase
MDFSLSDESLMMRDMFRKFSKNEVEPLAAETDRTHEFPMGTFKKMADLGLTGLPIPEEWGGAGASYLDFAMFVEELAKCCASTAVIMSVHTGLGCMSTYLYGSQRVKEKYLRALATGEIVGAYALTEPNAGSDAASLKCKAEDKGDYFLVNGSKIFITNGIVATTYCTFVKSDPTQGSGRGITCLVIEKDTPGFTMSKPVAKMGMNGSPTVELYFEDVKVPKENVLGEINDGFKVAMGLLAGG
RVTIAAQGLGIGEGALDYTVQYIKERQQFGKPLAANQGVQFMVADMAAALDAAQLLVYRAAWLKDHGLPHNKEASMAKLFATDTGMEVCTNQLMGGYGYCNEFPVERMMRDVKITQIYEGSNQIQRMIIGRETIGRF*
>Syntrophomonas_wolfei_subsp.metylbutyratica_JCM14075T_00650_Glutaconate_CoA-transferase_subunit_A
MGKAIRTLKEAGALIQDGDMLSFSGFTIWRRPMALVYELVRQGKKNLHLHVFNGGTHTEVLAGGAVKIWESCWVGHELYGKLGEGVARGQCEKSMIVEDYSHGQVARLLAGAYGLPFFPTALSMGTDILNPQYDMLKRAGLRDGSNPKIPRQKYTVSQDPFYDMGEILLMPAANPNWALVASQVGDEGTMRVNAQTYTDAEVIKAADKVIVAEEIVPDSYIRSPEKNIATGYSIDYV
VEIPWVSAHPTGSQGYYDYDADFIRNFYSKSKACDDWAEIWIRGVDSHEQYLERLGSNLEKLRANKVLGYSTRVKRGSR*
>Syntrophomonas_wolfei_subsp.metylbutyratica_JCM14075T_00651_Glutaconate_CoA-transferase_subunit_B
MTAKNNKLAKAGEYKPSELLAVAAREVVDGDIVFAGTGLPMLAIMLAQRVYAPDISRGIDLPTSVGDARCAHQASMAAGLTEAFYGQLHCGYVDLAFLGGAEIDKYGNVNTTVVGDYLSPEKRFTGSGGNPDINALARRTVFIMVQEKRRFREHVDYITSPGWRLPKWPGGEVFHKREVYGKFFRGGVEAVITNMGVFRFDEEGIMYLDTVHPGFTPQQVKDNCSFDLNISR
VSGETKPPTYYELELLYKEVDPEGIFLP*
>Syntrophomonas_wolfei_subsp.metylbutyratica_JCM14075T_00652_metallo-beta-lactamase/flavodoxin_domain-containing_protein
VQLSENVLLLCGNRHPNYVLVGKKEAAIIECGVSGYYSFQKQWEKLHQKPEVKYLVAMHAHFDHVCGIPALQKMFPAAVLLASPEAVLKKAKIMENFFAQDEKMLDLLADEGIIPRGLQSPNVTEIALDKLIAEGDEIKLGGDLNIKVLDAPGHSPCSLACYLPAEQVMFSDACGFQISDEDIFPVFFQAYDIYLETIKRLMSYPSKLLAIPHERIWTGRNIDAFYRRALSSAEQAFNIIRDLLDQGWE
DEKIEKELFARYYKGSLKIYTPENIQQCVQILLRRVKEL*
>Syntrophomonas_wolfei_subsp.metylbutyratica_JCM14075T_00653_NADH_oxidase
MKTTPELFSPAYIGKLVLKNRIVMAPMLVSYASSDGEVSERLLDYYEARAGGAGLIIVEAACVDAPTGREIFRQINIDDSLRYVAGLQQLAQHIKAYGCRTFIQLFHAGRQTNSLFTGVQPVAPSPLACPMVGEIPRELTEEEIEAIKFSQAAAYAFMAGFDGVELHAAHGYLINQFLSPHSNLRQDEYGGSLENRMRFLLNIIKKIKTSSPGLIISVRINMDDFVPGGLEKESVEICQHLEQAGVELIN
CSSGTYESLKSIEPASYKEGWRVYLAGELKKWIHIPVIAGGMLNNPAFANQLIAGGQADFIFLGRSLLADSEWPHKVRESGTEDIRPCIRCNNCIDNNFRGMMVDCSVNPLTGRERHFSQEGKKKLLHKANVMVVGGGPAGMQAALSKRGLNVTLYEKEKSLGLLNLACLPPHKHRVAYLRDYLANQVEKSGVEAFLNCEISLPDLNPDYILLATGSKPLLPQIPGWDQSYCLGREVLR
PEIKGKRVVIIGGGSNGCEVADFLLAGNNQITVEQNKLYADMEKKNRRDLMNRLEEGKVNKKTSSQVMEIKDQQLQLKSQQGAYECLEADYIVLATGFTPNNALYIEAQNRHKNVYPIGDAFQVKGFKHAIQEGAEVAHMIATGLKK*
>Syntrophomonas_wolfei_subsp.metylbutyratica_JCM14075T_00654_Ribonuclease_HII
VRSEQPLYNKRLFSDIDKSTHFKEGLGLDKQKYLLDEVKREDYLREYEAYNNGFRYIAGIDEAGRGPIAGPVMAAAVILSRDFFCPGINDSKKLTALKREKIAEEIKKQAITWSVAAVFPSYLDKVNILNATREAMLLAVKNLSPRPEFLLIDAVQLPLPIDIDKQYPLIKGDSLSVSIAAASILAKVERDRVMEAFDSLYPGYGFSKHKGYATREHLQSLLRLGTCALHRVSFEPVKSMVLGARYGEQPALF
F*
>Syntrophomonas_wolfei_subsp.metylbutyratica_JCM14075T_00655_hypothetical_protein
MENSQLYLNKIMLNITTKSDQNLNFVKGEVLRGMVQDILAGLQKVSLFIKGQMIDAVNSLPEVSELVQPGQQLQLMVEDFRDGRAYLKLTPQLMERIENSNISLNLTEIGVPAKEENIMMARKLLHYKLPVTSNNLNLMSKAMVLGGANEKNAEIVAFSMARKLPLDAPTLKSLAQFLMPANDVSKLAGNLDKGLLQLEQTVARMVETSGSSHIASSRPVGEMGTEATRPQRGISGDKEVKFSATAV
ANKGSESMQVSSKGPEIQGFTGTKSGEVLLPGEGRPALGQASSAPEAKIANVEQNYGKIKLEALLSGNSKNISLPVEPADARRNITLTGEKAAAVSSRVTTSSSPTQASPQSENVTSTSPAVNPAKAGGETSAVEKTLLGQITATINSKESTLASETGKNLGGSPAMDQGKSVSSPVLVNEKATLEGGKSPGVQTLESTINKKEPASGIKNVYTPTATSATSGRERQPLVTNLPVTDGKTVNVPELLPG
NISDKALSSGGMPMDSYKQISGQGAIAQVSPPIIAEGVTEVFEKAATIQTDHKEPATLLTNPNNDWTAPEQRELLRHLPLLTLLETLHLNLGREIGNRLQNAFSQERDIIRGLNLLQIIKNDSTSNKPLMADLFNRIEGMERELSQNPLNFSRGAVDSSSNYFYFAFPVQGEKYRLGQLRINREIGRKGLNKQDNIRFIVSLDTVQMGVVLFHVNWKRLGELEVQGVVERQIEIEYLNRNLGELFQGLES
LGYRIKNLGIKVVEQEEEMRLRLALEEIPLPKLGIDVRV*
>Syntrophomonas_wolfei_subsp.metylbutyratica_JCM14075T_00656_Flagellar_biosynthetic_protein_FlhB
VKEKDMEKAVALRYDMEKDEVPVVVAKGQGFIAEKIKKVAREESGVPIKEDRELADYLMALDLYEEIPPELYAVVAEILAFIYRMDKRFI*
>Syntrophomonas_wolfei_subsp.metylbutyratica_JCM14075T_00657_hypothetical_protein
MNRELGLWGEELAAQYLKKKGYKILERNFFTRYGELDLVCEKDDNIVFVEVKTRRSTRFGSPEEAVTPRKIGNLKKAAILYLKSSPRFFPEISFDVVSILVEDGKSRINHIINAF*
>Syntrophomonas_wolfei_subsp.metylbutyratica_JCM14075T_00658_hypothetical_protein
MLIYGKDIREQIKERVRQTAIEVKMGLAIIRVGDDPSSLAYVRGIRKFAEETGVKAEIVNLPDSVGERELLNTIGDLNNNSVITGIMLPTPLPAGLNASRLINAIDFDKDVEGIHNYNLGKLISKEEGVRPCTPKAVISMLKAHDIPIEGQKVTIIGRSMTVGSPLALMMTAENATVTVCHTRTRKLKEEALRADILVAAVGKREFVTADMVHKDMVVIDVGINFDENGKMLGDVHEEAWNHCRVASA
VPGGIGVITVAELFDNLRISQKA*
>Syntrophomonas_wolfei_subsp.metylbutyratica_JCM14075T_00659_putative_ABC_transporter_permease_protein
LLLISPLLCIFASLFKGRYPLSIDEVIRILWCKTAQLPCEVLDIKQTIIWNIDIRLPRAILGALVGGGLCLAISGAAFQGLFRNPLVSSGILGVSSGAGFGAALAIVMFSSVSSATYIFAFAFGTLAVFLSYIIGRVYDTTPTTIMLVLGGVIVSSVFSALISLLKYVADPYQQLPAIVFWLMGSLATARYSDIVAAGLPMLVGIAGLLAIRWRINVLSMGDKEAHTLGVNLAVKGLVIISATLATAAAVSVSGIIGWVGLVIP
HICRMLVGNDNRVLIPASLMSGACFLILVDDLGRIITGSEMPLGILTALVGGPFFVWLLIKTKGGNW*
>Syntrophomonas_wolfei_subsp.metylbutyratica_JCM14075T_00660_putative_ABC_transporter_ATP-binding_protein
MSLLQVIEAQGFWYGEKRIKKLNFQVEKGEVFCLLGPNGCGKTTLLDCILGVRQLHQGNIYIQGKEIKSMPAEKIAKILTFRSGQIARYIAYVPQSHERTFPYTVMDIVKMGRAAYTGRFSAPSKLDKAIAREALEMVGLFHLRHRPYTQLSGGEAQLVMVARALAQKTPMIIMDEPTAHLDFKHELEVLETIVELVGDSQISIIMATHFPNHAFYFENKKLPTRVALMHDHNFLELGRPSEVIAEESMKTLYGVNTRL
ISCATGERNELKQLVPISTS*
>Syntrophomonas_wolfei_subsp.metylbutyratica_JCM14075T_00661_methanogenesis_imperfect_marker_protein_11
MKILVAIDDTDNIDSRGTGELADLLSKYLEEKGWKGGAVTRHQLLVHPDIPYTSHNSSMCFMAEIEASYLAAYIEYAGHFLEKERAEGSDPGLCVAAIEQITFPDELIDFGYRAKQVVLTKNNAYDLAKKLRIDIHLSEHGGSGQGVIGALAGVALRLTANDGRFKGKLKVKSRDDLLSVREIKKQTPVTVVKSLDDMLVLDDDSIILMGEKLKAVLLEGECTLLVVPTGMNDPKWQSCATSQRLKF*
>Syntrophomonas_wolfei_subsp.metylbutyratica_JCM14075T_00662_hypothetical_protein
LSHWIFTEEGKYEFRQGKDAWKEAAQIADRCPHFQPDEEEEQVADENCSCYNCRYRRWTQKSFLCLKL*
>Syntrophomonas_wolfei_subsp.metylbutyratica_JCM14075T_00663_Cyclic_di-GMP_phosphodiesterase_response_regulator_RpfG
MPEDIKNKNLLDDFLEFFRLSEEALPSSEQKYMTMVENANDIISHIHLSDLSYLINSTGPKILGWSKEELMRKNPMNLIHPEDLTRALETIQAIGEIDGCTCECRFLKKDNQYLWLESSGKKIQFDADTVALLLYSRDISERKALEETVRQRLNLEAIATASQFFLAADNVDLIHIMSIVSAAVNADRVYIFEFFDEGKKSSNTYEWCATGIEPAQEMLQDLDSSDLFPWWLGRLKNGENIVIEDVNALPA
EANNEKFLLQSQGIKSLLVVPLCSEHRLLGFLGFDDNQSTRHWLAEDIKTLRMLAEMIALYWTRCQVKNALRDSEQRLASIIDFLPDATFAIDKQGRVIIWNRAMEEMSGIKGEDILGKDNYEYALPFFATKRPMLIDLVMNPDHPLKRYSLSEKRDETLTVGEIFSPQGSYMFGTASPLYDANGRRVGAIESIRDVTERRRAEEKYRHLSHFDKLTGLYNRSFFEEEVKRLDRSRLFPISIIGDVNGLK
LVNDAFGHREGDRLLRATQILKDSCRQEDIIARWGGDEFIILLPHTHPQQALQILCQRIKNACQPQSESEPIQVSIALGTASKETVDENIFTIIKETDDRMYSNKVQESKNTRNSLHSSLENSLRTRTRKSFYVVHKQETAKNIGMLGLSSKNELQNLLLLASLHDIGIITISPEILFKEAKLTPGEWEIIKKHPETGYRIAQSTPELLEIAEAILAHHERWDGTGYPQGIAGEQIPLGARILAIISDYNAMISGRP
YKAISPSLALQEIHRCAGSQFDPELVRLFLEKIGIRNE*
>Syntrophomonas_wolfei_subsp.metylbutyratica_JCM14075T_00664_Aminoglycoside_acetyltransferase_regulator
MRLSGFSAWSRFFRVLFLFLSIFWAFYSLKFKKIWHRRNWIETKKEKLYISEAERFRKTAVRMGGILKLQFFSTRVDILPSDISIDLAGLQDEVPGVAFAEIKKVAEEEFSRPIEIFYQVDESPLASASIQVHRGVLAGGQVAIKILRPGIEELIAIDLKALRQVLNWIISFTNWESFIDLEAIYQEFEETVLEELDYIREAHNAETIAENSQNDPELIVPGIFWDYTRRRVLTMEFKEGIKINNLAAFDQVGVD
RKAIARRLIEIYVKQLLFHDPVHPGLNPLFVTNDGRIIMVDYGMVGTITHELRDQLLRLLVEFALVKRDPYSVVDYLKEIGFLRPDANETVTRAVAQFIYLPLPVILFQRYPRTIRDRIGKGKNRRLIGLTLYEQPFQIPANFTFLGRALGTLYGICIALDPQIDFLEVSKPYVDQLAPAKEIWSVKEKTTNFFASWIEIPPLMEKVLLRVERGEIKLKLDLRSRLNEKIQENTRAQKMQAAWAIVGFSLLSSIYLAN
DFVFLSRYTFVFSALSFLLLLYQNRSPKAWKAPHPPLGKKGRKKQNKKS*
>Syntrophomonas_wolfei_subsp.metylbutyratica_JCM14075T_00665_putative_oxidoreductase/HEAT_repeat-containing_protein
MIQCLLSLVGGDKLSNKEEFKRNLSSEDEREAFAVEDIVYEGFRSGSSEFVELLQAFQEIISSQFVREIINRLRTLSGNVSVEKTIPLLHSDDAFIRNAVIECLSSEGESNLLISHEILLQKEKDSFKEDLFNIGPLDAFIFSREEYGDTLAEEKQKDELGLKEEAASESGTFELLKEAIAEGGLEGSIQGINNSLFFEQLPFLLDNPDKDIRKFAVDILFQLQSPHSAAVIARGLDDSDINIKITAAEYLGKLEAKAHIGGINRLLRLFSNRNLLLRCTCLETMAILGDEESARLVAEFYPQPEQINELELYSYLKYLARQKGEEYLPFVFSLMSKSGKLMAKEIINTIE
GILRRSGAVYLPAGMVEDLALYIASDINVINKYELLVFELGHFKNSEIGPLLLASYACSAEKLLCLGAVEGLGRLGLGDPEQLPLLLELKQIGDDEDLEAVGKALIQLAPVR*
>Syntrophomonas_wolfei_subsp.metylbutyratica_JCM14075T_00666_Chemotaxis_protein_methyltransferase
MDLSTEDFIARINLIYERTGMHFGENKIYYFKKRLQKREMEINQLQKVSEYIKHLKYDRDNREFQELINLITTNETYFFREFSQLEIFAEVCLPEVLEKKRAKSDPNLRIFSAACSSGEEPYTLAILKEMLEDFHNWNVVVKGADIDENVIKQAQRGIYDSRSVKDVPPPYLEKYFSNPSPGRFMVNPEIRNMVEYLQLNLMERRALRERNYDFIFCRNMLIYFDEYSRKQLVEKFYAMLNKGGYIFIGHAE
SLSRISTAFKIKRMNGHLAYQA*
>Syntrophomonas_wolfei_subsp.metylbutyratica_JCM14075T_00667_hypothetical_protein
VTKKRIIVVDDSEMIRNFHCYILKTAGFQVMSAVDGADALEKIYSVEEIPDIMVVTDINMPNMDGYTFIERLREDEQYEEIPIIIISTEDELKDMERGYEAGANIYIVKPTEPEVMVENVRMLME*
>Syntrophomonas_wolfei_subsp.metylbutyratica_JCM14075T_00668_Chemotaxis_protein_CheA
MKPVHFFDIDLEILQGVIEESSEHLNGIEGGLKELEAEFDSPDLNAVFRAMHSVKGVAAFLDLIPIKDTAHSLESFMTDMKKGLYPVTSEMIDILLKGVDILQQLRQLGEHVKKLEVNPPREEKELCHEVEPVANVVEKSSFREQIPEGQLTKEEIQEIYEKGTE...

MKNRVVCYFSAIQTEQPVVYRLIKNYDLIINILKADINPQKEGYLVVEMEGEKDKYEGGIAFLKNLGIIVEPLSETVLWQEDICIQCGACASFCPTESLSIDRDSMLVSFENSKCVVCGMCLDCCPTRAIKLHFEVKEAV*
>Syntrophomonas_wolfei_subsp.methylbutyratica_JCM14075T_00691_hypothetical_protein
MNNAANNGYGERYYRVLHAANDLKYPSVKIKESDLAIAVDRKSYSDSLLSLCQQELHGVRRQLEDYIKRHPEFMTSFVPLPLMLGAPEIACRMAAAAEKAGVGPMAAVAGAIAQSLGQALENQVEEVMVENGGDIYLRSKSDRIIAIFAGNSPFTYKIGIRVEPGESPLGICTSSATVGPSISLGRADAAVIKAYPAELADAVATQAGNLVQSKDDLIKAIDYAKAIQGVSGILLIQGDSMAAWGKIEIIPL
KGRQKNEGSK*
>Syntrophomonas_wolfei_subsp.methylbutyratica_JCM14075T_00692_Cysteine_synthase
MKVVNNVLELIGETPVIKLNRMHGADQAEVYVKLEGFNPGGSIKDRPGLYMIEKAEARGLLKKDSIILEATSGNTGIGLAMAASIKGYRILIVMPENMSEERKKILRAYGAELLLTPAAEGMPGAVRRARELAESSYFLVQQFENQDNAESHEMTTAQEILRQMEHKLDILICGVGSGGTLSGTGKVLKSSLPGIKIVAVEPAQSAVLSGKSAGVHKIQGIGAGFIPPVLNIEMVDQIIPVSDEDAIETC
RQIAKKEALLLGISSGAAIFAALNLARDMDPSQKILAIAPDGAEKYMSTELFD*
>Syntrophomonas_wolfei_subsp.methylbutyratica_JCM14075T_00693_NH(3)-dependent_NAD(+)_synthetase
MNPEAVVNYLVEWSRQKVRDAGALGLVLGVSGGVDSAVAAIIAKKAFPENCMTLLLPCESDVVDRMDSQALVEKFNIPYRIIDLDNAYHLLSTQFESYLKFEGLKGKLLRGNIKSRLRMMALYYSAQARNYLVLGTSNKSELYVGYSTKYGDAGVDLQLLGDLLKREVYELAQFLDVPESIINKPPSGGLWSGQTDEGEMGLTYEELDNYLASGEGSLEVINKIEGMMAGSLHKRNMPPVAIIFPEL
RD*
>Syntrophomonas_wolfei_subsp.methylbutyratica_JCM14075T_00694_Putative_protein-S-isoprenylcysteine_methyltransferase
LSPRKRQVAIRMLIFLAVFSYIGYIASQRLPSFDFWVIMGFFALFFTWTIIETIIYREPQTRVVEDDDQRSYLYLQLSSMLVLFVALLDFLEFKCTRVTLLEPGITILGFIFFIIYTVRYQAIIHLGSYYNPRVALYEEHSLVKEGPYRKIRHPFYLSALLSVLSLSCIFNSGAALLLTVLAVIPAIVYRINIEEEFLLEHFGEEYKQYMEQSSRIIPGIW*
>Syntrophomonas_wolfei_subsp.methylbutyratica_JCM14075T_00695_putative_transcriptional_regulatory_protein
MAGHSKWANIKHKKARSDEKRGKEFTKIAKEITIAVRSGGSGDPEANSKLKLAIQKAKAINMPNENINRAVKKGTGEIDSETIEEIIYEGYAPGGIAVMLEIATDNRNRTASEIRHLFSKNNGNLGESGCVAWMFKRVGLISIKKDKLNLDEEEFMLMVLEAGAEDVREEDEEYEVLTLPESFMQVKEAMEEENLPIEEADIVMLPENTVDITDVDMAGKIIKLIELLEDHDDVQNVYTNMSIPDEIITAL*
>Syntrophomonas_wolfei_subsp.methylbutyratica_JCM14075T_00696_hypothetical_protein
MKKTFTGITLGILIMVFLSGYFIGSHKVLEVRQKPVGIDDKSKIEFQRINEKSQIVMEKEYKRCQHVVISDFEPKEQLLGKSYSEISRIYSPQNGFTVSLQDDTLCIHQIIDDWCPLDKEKYRLKEYQGKLAVYQGPDEDNEVLRVTSISMETLPAEIQSAIREGKYEFNNEELLNDALENLDEY*
>Syntrophomonas_wolfei_subsp.methylbutyratica_JCM14075T_00697_Crossover_junction_endodeoxyribonuclease_RuvC
MKVLGIDPGTATTGYGLIRSEAGRVMAITYGTIVTPAQLEMPLRLNQIYEELQDLLMEYKPDAVAVEEIFYHRNSKTVITVAQSRGVILMTAANAGMPVAEYTPLQVKQAVVGYGKAEKKQVQLMVQKILGLQQLPRPDDAADALAVAICHLHSYRLSFLTGSSATKAGEKR*
>Syntrophomonas_wolfei_subsp.methylbutyratica_JCM14075T_00698_Holliday_junction_ATP-dependent_DNA_helicase_RuvA
MIAFLKGEVAESRERPSDIIDVNGVGYEVNIPISRLLPEQGEPILIHTFLQVLENEFKLFGFLDQDELRLFKTLLSVSGIGSKGALAVLSTMEPLVFYRAIASQDEKTLLRIPGVGKKTAQRMIFELQDKVPELKLVEVEKEQRPLLDELMEALEILGYSRSEVLPVIMDLNRNKQLGTSVEENIKLVLKAKAQEMRR*
>Syntrophomonas_wolfei_subsp.methylbutyratica_JCM14075T_00699_Holliday_junction_ATP-dependent_DNA_helicase_RuvB
MLEERLISSHLLDEDDNNENSIRPGRLSEYIGQEKVKENVEVFITAARERKESLDHVLLSGPPGLGKTTLASIIANELGIKPIRKTSGPAIERPGDLAAILTNLEPGEVLFIDEIHRLNRNVEEIMYPAMEDFVIDIIGKGPAARTLRLDLPPFTLIGATTRPGLLSSPLRDRFGINCRLDFYTPLELGEIILRAARILEISLDKEGAAEIAGRSRGTPRVANRLLRRVRDYALVKGNGIDFSLAKWALEMLEIDECG
LDVMDRMILEAIIKKFSGGPVGLDTLAASVSEESDTISDVYEPYLLKLGFIQKTPRGRMATEHAYHHLGYPLKGNLQGKGLFSDE*
>Syntrophomonas_wolfei_subsp.methylbutyratica_JCM14075T_00700_hypothetical_protein
MSKILLHICCGPCASYPVPRLREEGFELMGYFYNPNIHPFTEYQKRKEALEDYARQVELKVIFEPDYKPVDYPQNITYRESRRCLLCYQMRLEQAAQIARKGKFDYFTTTLLVSKYQKHELIREIGEAIGEKYELPFLYRDFREGFRQTGIIAREMGLYRQQYCGCLYSEMERYQSSRKKA*
>Syntrophomonas_wolfei_subsp.methylbutyratica_JCM14075T_00701_S-adenosylmethionine:tRNA_ribosyltransferase-isomerase
MLHDKNDDIYRLSTYHYDLPPELIAQYPAEYRDSSRLLVLDRKSGDLKDRVFKDIGQYLKKGDALVLNETRVIPARFFAYKESGARVEILLLKKLGDDWEALVKPARRLKVGSRVFLSPDQPLLIEIVEELEFAGGRRVRFLNGINEDTLLQEQGHIPLPPYINRPDEELDRERYQTVYASKSGSVAAPTAGLHFTPDLLKDIALQGINIVRIVLHVGMGTFRPVKNKDIRQHTMHLEYFEVAEEAAALL
NLTRESGGQIVAVGTTVVRTLESVYNEDCGFVAGKGETNKYIFPGYQFRAIDKLLTNFHLPASSLLMLVAAFAGTESTLAAYRHAVENRYRFFSYGDAMLIK*
>Syntrophomonas_wolfei_subsp.methylbutyratica_JCM14075T_00702_Queuine_tRNA-ribosyltransferase
MFKFELLQEDKTSKARLGRLSTAHTTVDTPIFMPVGTQATVKTLTPEDLYEIGAGIILANAYHLYLRPGCELIQQAGGLHRFMNWKKGILTDSGGFQVFSLSKLRDINDDGVWFHSHIDGSRHFLTPEKVMEIEAKLGADIAMCFDECAPYPCSYEEAFKAVKRTSLWAGRCKKAHNNDKQVLFGIIQGSVYPELRERSVGELVALDFPGYAIGGLSVGEPKEMLYATLEVTTRILPREKPRYLMGV
GAPEDLLEGVRLGVDMFDCVLPTRLARHGTAYTAGGKITVRNALYAADFSPLDAGCSCYVCQNYSRAYIRHLIKAEEILAQRLISYHNLFFLQLMAGIRAAIAKGEFPSFREFFHNYISV*
>Syntrophomonas_wolfei_subsp.methylbutyratica_JCM14075T_00703_preprotein_translocase_subunit_YajC
LAAQGSSEWMTIAIYFGFLAIFYFFLIIPRKKQEKKHKIMVEELKKGDEVVTIGGIRGEIGRVKEDTIMLKVADNMEIEMVKKAVYKVEE*
>Syntrophomonas_wolfei_subsp.methylbutyratica_JCM14075T_00704_hypothetical_protein
MVTLKDVRSSPIINTFIQKGNEHILGVMGYTDHGQLHLSLVSALSKDIMIKLGYDERLAELAGIAGYMHDIGNVINRIGHSQSGALMAMEILRRMGMVPEEAAIITAAIGNHDEGSGHPVNEVAAALILADKSHVHRNRVRNPDVATFDIHDRVNYAVERSTLNIDENEKVTIMELVIDTSICPVMEYFEIFMSRMLLCRRAAGFLGCEFELVINGVQLL*
>Syntrophomonas_wolfei_subsp.methylbutyratica_JCM14075T_00705_preprotein_translocase_subunit_SecD
LQKNFSVAVILAIIVALGALTYFAYHPILKNLNLGLDLRGGLHVVLEAQEKEGQKISADTINKAVGIIRNRVDKLGVSEPLIYPQGERRVVVELAGIDDPEEAVNIIKNTAQLEFWDESGKVLVTGKHLKEAQARASGEGQGFEVAIDFDREGAKLFADATTANVNKPLMIVIDGKVISAPTVSGPITDGSARITGNFTAKEAENLAVLLRSGALPVSFKILEKRTVGPTLGSDSLSKSVKAGIAGLIAILIFM
LGYRLPGFIADISVLVSILVLGTMTLFGAVLTLPGIAAFVLSIGMAVDANIIIYERIKEELRWGKTLKAAIEAGFNRAFWTIFDANLTTLIAAVVLFFFGTGPIKGFAVTLSIGIIASMLVVLTFTRYFLVLSADLSKNHKLYG*
>Syntrophomonas_wolfei_subsp.methylbutyratica_JCM14075T_00706_preprotein_translocase_subunit_SecF
VQFIQNRKYSFIFSTILILGLISIFVQGFKLGIDFEGGSLLRFKMDAAVSSAEVRSTIEEFKFIDQASIQKSGNEFTIRTRELNQEQTEQINKAFAAKFKNVEYLSAESVGATMGSELTRNAVLSVLIAMLLMLVYISFRFEWTFGVAAVVALFHNVLIVLGIFSIFQWELDGAFIAAILTIVGYSINDTIVIFDRIRENLRLKTRDDLTTLLNKSIMQTMNRSVNTVLTVLFPLVTLYLFGGSTIKIFVLTMLIGFVVG
AYSSIFIASPLYYDIKQRA*
>Syntrophomonas_wolfei_subsp.methylbutyratica_JCM14075T_00707_putative_integral_membrane_protein
MRAYLFIALFIALITGVTIFQNTAVVKVHFLNWVSPEISLALVILLTLCSGALLTTLVFILVRVFQIASKIRELTVSNKKLQMEIDRLKLQQKDEEGKKEM*
>Syntrophomonas_wolfei_subsp.methylbutyratica_JCM14075T_00708_Diaminopimelate_decarboxylase
LTGTMKINSQGHLEIGGMDTVDLAREFGTPLWIIDEEGFRRNCRNIKDAFSLYGDSQVLYASKTLATMAIYRIVEQEGLGLDVVSGGELYTALKAGFPMEKVYFHGNNKSREELRMAIRVGVARIVVDNFYELELLNQLCTEENCQQEILLRVTPGVEAHTHEYIQTGQIDSKFGFTLTDGQALEGVKKALSMPGIKLVGLHCHIGSQIFAMQSFRYTSSAMMDFWAEIKRSGCQLEELNLGGGL
GIYYAGDEPRKAEEWAKAVMPVVQEKARQHGLKVPLIVEPGRAIAGPAGLTLYTLGSIKEVKGIRKYVAVDGGMTDNPRPALYAAKYQALLANKAEEKPVETVSIAGKCCESGDMLIWDIKLPREVAGDILAVLATGAYNYSMSSNYNRLPRPAMVLVKQGQAELIIKREDYEDLLRNDLIPERLKGACQGDVSFVL*
>Syntrophomonas_wolfei_subsp.methylbutyratica_JCM14075T_00709_hypothetical_protein
MSRERLTRVRLVFHPTGVTIKVASHGREIGAVPLEPRDKPLVPMHRLRAEYLCKLMHNYFLQWNHIYTS*
>Syntrophomonas_wolfei_subsp.methylbutyratica_JCM14075T_00710_hypothetical_protein
MRSEAEKLLFLIRNPPIIMGKHFFRQGVTGKVYEIEKYSVEAVFNTGFEVKGQWKDDLHNIHCRLVFDFDSFKIIEAEAWAESTPFPICPQGLKSIDRIVGSTVGPGFNRIVTENIMEKEGCVHLGELVMNSVKALVQAASRDKPEWVETADYTQRWNDWIRMYQDQCIFFSQPGVFENSQEEIQTAFRSKK*
>Syntrophomonas_wolfei_subsp.methylbutyratica_JCM14075T_00711_Multifunctional_CCA_protein
VRIITSHNALDFDGLAAMVAAGKLYPGAVKVFSGTLSKNIRQFMALYKDLISIKYPKEIDLKQVKRMIVVDTASANRLGHLKEALQSEGIDVVIYDHHPPSLDDLPASISEVEQVGAVTTLLVEKIAERSILLSRGANLSVVANFMDQPFSEQEFRELLHVLLNSSQHHLIKNIDVVIALWDSNEFIPGLDTVTYRLLEIENCDVAFAVALMQGKVN
VVGRSRSNNVKVNEVLRKLGGRGHDRAASAIVKGKSPDEVREIIMAELEKAVQPGLLARDIMSTPVKTIPQNISMEEAGRIMLRYGHTGMPVVEGEKMIGVISRRDVDKAIHDLGHAPIKGFMSSGVLSVAPDTPVGEIQRMMVEYDVGRLPVTDNNRLMGIVSRTDILRTLHGDDYPEDHEVLYSFTGEENQNCLAIMQERMPSRILATLRLAGEMAESIGSRVYCVGGFVRDFFLRVPNFDVD
LVVEGDGEELAKMARHLGGKARIHERFRTAALILPDGTKIDIATSRTEYYEFPAALPKVEASIREDMYRRDFTINTLVIALNPDSFGDLIDYFGGRKDLKEGLIRILYNLSFVEDPTRIIRAIRFEQRYKFTIEDDTLRFAKDAIERRLLGKLSRYRIMQEILILLSEIDPLPALDRIMEIGVWEYMIPEIDLNKVSRTMIKRTPIITWWEERYYGKNIKGWLVYLMLLLAGLNEERVIQIIKRYHLDNYARKAIQE
SRQVPQIVEYLRENREILPGDMNQRLDGWSHESMVELLISLKDELLWEKLVNYLDLKEQVKVEINGFDLKKMGLKEGPEFRLIFDELYQKLNGEIKNREEELQMAEKWIMEGKFNNDPVAE*
>Syntrophomonas_wolfei_subsp.methylbutyratica_JCM14075T_00712_Zn-dependent_proteases
MIQSLSIMLIALPAIIIAITFHEYAHGKVADLLGDSTPYYQGRLTLNPLPHIDWIGFMMLFLFHFGWAKPVQVNPYNFKNVSPKQGMMLVSLAGPGMNILLAFLGMLAYKYLLPFQEQFEWAIMTNLIHPIIYINLILAAFNLIPLPPLDGSKVIAGLLPDSGAEIVYSLEPYGPILLLIITGAAAYIFLPLASFLNTLLYAIVF*
>Syntrophomonas_wolfei_subsp.methylbutyratica_JCM14075T_00713_Segregation_and_condensation_protein_A
MNYVVDLESFHGPLDLLLYLLEEQQLEIYDIPIATITDQYMEYLRQTGEFNLEQMGDFLIMATYLLNLKSRMLLPSRVELSEEDSDNPREELVRQLLDYKKYKKLAECLLSVGNQDFPRVFFRSDEEIGSIEELVGGADAAQLWRSFQVLLEPLQEEKFRLPQGDVNSIEKMEEIVERLEQKAGLVLQDLFQGVISREALAFFLALLELIRLQRVEAIQEQSFGEIKIRLQVAV*
>Syntrophomonas_wolfei_subsp.methylbutyratica_JCM14075T_00714_Segregation_and_condensation_protein_B
MLMLRDQIKAAVEAVLFVRAGRVGMDELVEILDLPLLEIKEILEILEYNNNIRSGLQIVEIKGGYLLCTRPAYSDLIARMEKPQKKRLSAAALDTLAIVAYRQPVTRLEIEQIRGVKSDRVVNTLLEKGLIQEAGHKAVLGKPILYATTEEFLKVFGLTSLKELPDLEEL*
>Syntrophomonas_wolfei_subsp.methylbutyratica_JCM14075T_00715_hypothetical_protein
VAERSIEEEIKIKAQQGRKLARYMSSTQDLVENQIRKAQERGEFNNLEGSGKPISLEENPFEPPELRMTFKILKDNDFAPYWIELGKEIDHEIDKFRKEVEFFKRYTAIFYSQGHSSPAEKRFDSKKALFYAESRFTLQRIDKKISDYNLHCPFFRMGRPNMKIDDEIYKVISSVEKLIEELKQNPNKQ*
>Syntrophomonas_wolfei_subsp.methylbutyratica_JCM14075T_00716_hypothetical_protein
MLALYICLFLFALGLALILFIYLKVEIIIELLPAENKSNDKSKLLIQLKPLFRPFRSPLIHYRFDSQLKPSVEDICKKAALFISDLQQYDESGYFSRIGIFDVVLAGHKKLIQIEKLEWKTIGLENAMDTAICTGGLWAIKGSFIGFISARSRLEEMKIEVQPDFSEIKLASHLDCIVKMRTVHIMIIVVRLLLHSFIKNDRAKRKRASFKGRKVLAFLKSMMGV*
>Syntrophomonas_wolfei_subsp.methylbutyratica_JCM14075T_00717_putative_spore_protein_YtfJ
MELQDKKQNHPIESLMQTAMQSIKDMVDVNTVVGDPVETNDGTVIIPISQVACGFAAGGGEYELTATSKNTELPFAGGSGAGVSVRPMGFLVVRMNDVRLVSLTGNHLAERVIDLAPQIIDKMGFLKKKNKFSI*
>Syntrophomonas_wolfei_subsp.methylbutyratica_JCM14075T_00718_Spore_maturation_protein_A
MVDLLNIVWFLLASGIIVAALNGNIEVVSEAISAAQGAVKICIDLVGIMALWLGIAKGGLIQALARLMKPLMLRLFPGIPPEHPALGAILLNSANILGLGNAATPFGMRAMQEMKNTDSSEASTAMCTFLALNTSCITLIPATIIGVRVSLGSTNPTEIVGACIFATSIAMLMAVSLDYWFRTRSPWGGKG*
>Syntrophomonas_wolfei_subsp.methylbutyratica_JCM14075T_00719_Spore_maturation_protein_B
VLLSILTLISQWAIPFLLLAVPLYGMLKKVPVYESFVEGAEQGFYTAVKIIPFLVGMMVAISVFRASGAMDYLSRAVAPLTALIGAPSEILPLAVMRPLSGSAVLGMSTELMNTYGPDSHIGRLASVMQGTTDTTFFVLTVYFGSVGIKKYRYALLTGLTADISGFLASVYICNLLFR*
>Syntrophomonas_wolfei_subsp.methylbutyratica_JCM14075T_00720_Formate--tetrahydrofolate_ligase
MKTDVEIAQSAKMQPITEIAARIGIEPNELELYGPYKAKISLDIMKRIEDKPQGKLVLVTAISPTPAGEGKTTTTIGLGQALNKIGKKAIVALREPSLGPVFGIKGGATGGGYAQVVPMEDINLHFTGDIHAVTTAHNLLAALMDNHLHQGNELDIDSRQIVWRRVMDLNERVLRNVVIGLGGRINGVPRESGFDISVASEVMAALCLATDLMDLKERFAKIVVAYSQQGQAITAGDLKAAGSMALL
MKDAIKPNLVQTLENTPAFIHGGPFANIAHGANSIVATRLGVKLADYVVTEAGFGADLGAEKFFNIVCRYGQLVPDAVVLVASIRALKHHGGVKKDKLGEENLEALAQGMENLKKHLENIQKFGVPAVVAINEFPSDTSKEIDYVRSCQAMQTAVSISRVWAQGGEGGIDLAEKLVLATEQGSQFKFLYDLNQPLKNKIDTICREIYGAGSVKFSAEANRMLQKYEESGFGDLPVCIAKTQYSLS
DNAQLLGRPTGFEVNIRQVKLSAGAGFVVALAGDIMTMPGLGKVPAAENIDIKPDGEIVGLF*
>Syntrophomonas_wolfei_subsp.methylbutyratica_JCM14075T_00721_GTP_cyclohydrolase_1
MQKKVIDEAKIEEAVHMILEAIGEDPEREGLQNTPKRVARMYSEIFAGMNSNPREFLKVSFTEFHDELVLVKDIPLYSMCEHHLLPFYGKAHVAYIPRGGKVVGISKLTRVAEAYARLPQLQERLTSQIADCINESLKPFGVAVVIEAEHMCMTMRGVRKPGASTVTSAVRGIFETRPETRAEVFSLINQKS*
>Syntrophomonas_wolfei_subsp.methylbutyratica_JCM14075T_00722_hypothetical_protein
VAKNKKESRKDESIYPDGIERFQAQKDEMIGAPVRKKKKQKQ*
>Syntrophomonas_wolfei_subsp.methylbutyratica_JCM14075T_00723_Ribosomal_large_subunit_pseudouridine_synthase_B
LRLAKYLAESGISSRRQAEKLITQGKVKVNGKTISEPAFTVKPGDRVEFAGKIIEQTDKIYLLLHKPAGYISSLADPQGRPRVIDLLSAVNTRVFPVGRLDYDTEGLLLLSNDGEFSNLMTHPRYLIPKKYEAWVQGLVKPDTIRQLEQGVALEDGITAPSRVRLLSSKKGQSLLELEIHEGKKRQIKRMCLAVGHRVIKLKRTALAFLTLEGVPQGHFRFLTASEVERLIMLARQSGK*
>Syntrophomonas_wolfei_subsp.methylbutyratica_JCM14075T_00724_Nicotinate-nucleotide--dimethylbenzimidazole_phosphoribosyltransferase
MNSLHLGKFAVLNNKIEQSLPQGINDYAVLIFAGDNGVSLENFSSYPPLTSRDIVISHLQNGGAAINILCRLVNAEIKVVDVGLCHKVNDDKILQRNIARGSNNFLYNDALEKAQVEKGIEVGLSAWEDVAAEKFDIIGVGEIGIGNTLCAAGLAAIITGLELEYLVGSGASDKVINKKIDILSKAINLRSLDPNDVIDLLARFGGLELAALTGFILAAVKNKKIIMLDGYVTAVAALLASRIDSGVPSFIAPST
TGEPGHGHVFEQLGLKPVFDFAVNYGEGLVAAIAFLAEVLFQ*
>Syntrophomonas_wolfei_subsp.methylbutyratica_JCM14075T_00725_hypothetical_protein
LTKLQMRVRFDFMGKSKSGRLLWGSKNPEQLAEELRQHKASLIRNIPVQGIHIDDIDMSQDVYSIYDEITGKQICFAPVRTFTASSMEDAIRFTMKEEFRTVELIEPEEITLSRSEIEKLLLRISEELIDYRDYIEKRLDNWK*
>Syntrophomonas_wolfei_subsp.methylbutyratica_JCM14075T_00726_phosphodiesterase
LNKITLDSIVEAVNELPALPHIVIKIMQLSEDPDSTVQDISNVLNQDQAMTARVLRLANSAFFGFPRRISTVTDAIVFLGFKTIRSIVLAVSVSNILDREIEGYALEHGELWRHSQCSAIAARMIARKCKFGSLDLAYTAALLHDIGKVILNDHMKEAYHEVVARVDENNISFMEAENEVFGFNHAQVGARVAEKWNLPPELVESIALHHSPQDALLNQRLTSIVHLADAICVSMGIGIGIDGMLYPLSDEA
LKLLDFDEIEVEKTISELVDVFADQQSF*
>Syntrophomonas_wolfei_subsp.methylbutyratica_JCM14075T_00727_hypothetical_protein
LGKIAVFAGGLGSGKSELAINFALEEAKRAAGVVLADLDMVNPFFASREVGQVLERAGVKLFGPGGDLSFGDVPSIPAEIIALIQQENYLFIDLGGDEVGSLVMGYLSRFIKRWEEMEVFLVLNPYRPFSSELAGVSKVKQLIELAARVRITGIISNPNLVEETNLKTIIEGHRQVQEFADFLQLPVKFLCVEERFWDQLLPRYGISLKKLTLHLRPTWLQDT*
>Syntrophomonas_wolfei_subsp.methylbutyratica_JCM14075T_00728_2-oxoglutarate-acceptor_oxidoreductase_subunit_OorD
MARGRVIFAERCKACGLCVEFCPKNLVFDDHTINVLGFHPVMLDPDECTGCGICGLMCPDLVIQVERE*
>Syntrophomonas_wolfei_subsp.methylbutyratica_JCM14075T_00729_2-oxoglutarate_oxidoreductase_subunit_KorA
MKKVLMKGNEALAEGAIIAGCRGFFGYPITPSSEIAEYMARKLPKIGGAFIQAESELAAINMVYGAAAAGKRVMTSSSGPGISLKQEGISFISEAELPCLVVNRSGGPGLGGIQPSQADYPQATRGGGHGDYRIIVLAPSSSQEMMDLAILAFELADKYRTPVMIMAEGTLGNLMEPVIRVEAVTEFLDKPWATTGCVGRKPNVINTLHLSQEELEGRNRHLTEKYKMISKNEKRAESFLLDDAEIVVVAF
GLLARIKKSVMEARKKGVKAGLIRPVTLWPFPDEHIQAVSRTARTILSVEINMGQMVEDIRLAAGGKIPVEFYGRIGFTPNPEEITRQLLFIAEREGLL*
>Syntrophomonas_wolfei_subsp.methylbutyratica_JCM14075T_00730_2-oxoglutarate_oxidoreductase_subunit_KorB
VKTVFKKPESLTDAKFHYCPGCSHGIIHRLIAECLDELKIREQTIGICPVGCSVLAYEYFNCDMLEAAHGRAPAIATAMKRLLPDRYLFTYQGDGDLASIGMGEIVHASLRGEKITVIFVNNTLYGMTGGQMAPTTLLGQVTTTSPYGRSKDWEGYPAKMAELLAQNEGSAYICRTAVNDPPNVVKTKRAIKKALQTQARGLGFSMVEVLSTCPTNWGLSPVEAMKWVEENMIPYYPLGEFKVKEE
*
>Syntrophomonas_wolfei_subsp.methylbutyratica_JCM14075T_00731_Pyruvate_synthase_subunit_PorC
MFSEKQILFAGFGGQGVLSMSKFLAYAAMGAGKNVTWVPSYGAEMRGGTANCLVTIANEEISSPLTENPLMAVIMNKPSLERFENKVQPDGTLFVNSSMVDRKCRRDDLTVLEVPVNQLAEELDNLRGANMILLGAYLEKTRVVEVEDALEHFEQIFSGKRKEIIQKNRQAFLAGVEYARNNW*
>Syntrophomonas_wolfei_subsp.methylbutyratica_JCM14075T_00732_hypothetical_protein
LKRFVEKVEKFLRAIILTVLIIVVVQGAMTKDTWRFYLSLGERLGGTYFPPVNNLPADSRKNSLNLNGQALQPSHAEITLQVEKFSSLPHAIIMVNVEEREKFVNGEVALNQLNAGDVVVENIASYYNFPLSFLVKDCSPNLAYPKGKTFTSNQSIAMIGKIIVK*
>Syntrophomonas_wolfei_subsp.methylbutyratica_JCM14075T_00733_HutP
VEIGSKIVSTAAIKMALSASRQEETRLKKELENWEVKAVAVDYGGEFISSVGKIVERAVVAAKREGVHDAHADEAVGAGAERAIAQMIKALGLNVGGKIGVARHKDHVSVAIFLGIGLLHLDEVAIGLGHRAVS*
>Syntrophomonas_wolfei_subsp.methylbutyratica_JCM14075T_00734_Chorismate_mutase_AroH
MQLRGIRGATTVNEDRPEEILRATRELLQQMQKENAFTVEEVASLFTTVTADIRSAFPAAAARAIGWDKVPLLCFQEIEVPDSLPRCIRVLLHVNTVKSQEIKHIYLKAARSLRRDLNR*
>Syntrophomonas_wolfei_subsp.methylbutyratica_JCM14075T_00735_(R)-stereoselective_amidase
MDKTLSLSICQMKTGKDKDANLKKAEKMIAAAALEGAEMVILPEVFNSPYQAELFPRYAESFPGPSTDFLAAAAHKHGLCVVGGTIIERDSQGKIYNSSFVFDERGELIGRHRKAHLFDINIPGRISFHESDTLAAGENITIVRYRSHLFALMICYDCRFPELARAAALEGAELLVIPAAFNTTTGPAHWQLLMRCRAVDNQLFVAASPARNPSASYQAWGHSLVVDPWGDILAEAGSGEEIIHARLDF
SRLEQVRQELPLLRQRRKDLYRLDYKAPGNGIV*
>Syntrophomonas_wolfei_subsp.methylbutyratica_JCM14075T_00736_hypothetical_protein
VKTDTVIRNEGMKVLIKHLGKVDAERFISLIRREPFDYTKWQKDLWKDKTVKEISEAAMEYRRTDSI*
>Syntrophomonas_wolfei_subsp.methylbutyratica_JCM14075T_00737_putative_ABC_transporter_permease_protein_YknZ
MSLSESIRVALDSIRANLLRSILTTLGIMIGIGTVIAVVAVGQGGQQSIMKEMEKFGSNLFQIRLDWRRDELPSGTEFTMQDIEIIKANVPELEYLLPCADTYSQVKAGNKAKSAQISGTEAGYACLHPLESTGRFFSDSDCQSGRRVAVIDEELARELFPRSSALGKKILVGNQRYTVVGLFKAESSPFAMGIKFVYIPTRVWLDIYPDGGISYIEGGTRNPAEVKTAMQSSKILERRHRSENLYQGVDMEQ
QIQAVNKVTGVMTLVLAGIASISLLVGGIGVMNVMLVSVTERTREIGIRKAIGARRRDILIQFLIEAVILCLLGGIGTGMALGVGGAIVKLLGWPPLVSWATVLLAFSASAIIGIVFGLYPAIKAANLDPIEALRRD*
>Syntrophomonas_wolfei_subsp.methylbutyratica_JCM14075T_00738_Macrolide_export_ATP-binding/permease_protein_MacB
MIKMERINKIYRPQSNPVHALQEVSLHIEGGDFVAVMGPSGSGKSTLMNVLGCLDTPSSGTYWLGGIEVSSMNSDQLASIRNRKIGFVFQNYNLLPRMTALRNVELPMLYAGVENKKRQARALDCIEQVGMLDRIHHRPAELSGGQCQRIAIARALVNSPDIILADEPTGNLDSASSLEIMHIFQSLHRNNTTIILVTHEAEIAAFARRVLHFRDGRLTADEILSPQHLKKTPLPKGGEDI*
>Syntrophomonas_wolfei_subsp.methylbutyratica_JCM14075T_00739_Macrolide-specific_efflux_protein_MacA_precursor
MPAPNIRTQTTIFKIALIIAVALSIGLNLWRNHKSGTLVHTTKVEEKTLGEKVYASGRIEQKKKVDVYSELPGKVNKLLVKTGEKVKKGQLLAVLDSPNLQSEMRTIDALLHYTRESNFIQLKNTALQADLQRGEKLFKNKVVTRSELEEKRAQQDKLESALKLAEIKSNGEQLAAAKTLLQKTELTSPFSGIITRLSVEEGEFVSAGAPLVTIGNEQHLLIQAEVDEADIGQIKVGQAVNITSEAFPEKTFSGQVVSIKPEATQKNGIRLVEVKLLLKSDNLKLGMSVDVSIRTGAKTKALLVPLSAVVSAE
DIFSKGQPERQVKVGQVEGNKAVIEEVASLPEKIFIFSKGKTGGEVEKVNLVVLSEINQVNALSLQEVRLDAFNQTAGHLVLQLKTVASLQSIAV*
>Syntrophomonas_wolfei_subsp.methylbutyratica_JCM14075T_00740_Yip1_domain_protein
MQQMPDNPALSSPEALDMAANAAIIGGIAGALLTPLLFALIVALLLKLFNALAGDKVPYRKLFKSVVSYAYLPLLLSSLIGLALGLTTPAENFGSVSSSPLLFPPDTYTFSALLAAQINPFILWSLYLAALGGMLLMSKEVKKVGSYIFLWLIYALASSYFGAASISNLYG*
>Syntrophomonas_wolfei_subsp.methylbutyratica_JCM14075T_00741_hypothetical_protein
MELESDSGTLSLGQKFSLIFTSPSRVFTELERNHSWLALLVISLVSLAATLPTLSKIKHMAS*
>Syntrophomonas_wolfei_subsp.methylbutyratica_JCM14075T_00742_hypothetical_protein
MAWRKLALLVLGVIMLCSLAACGNKVDSKAVREYADEITEKMLLAYNEDNYEEYIANTNSQFKTVVSEDMAKEFNEKGKIGDYVPGSKEKKFKDAVKTSQKEQKYLVVRYNAKFTDESEDVLVIMFDDNEAHQVGGIFFNSPKLREQSEK*
>Syntrophomonas_wolfei_subsp.methylbutyratica_JCM14075T_00743_putative_membrane_protein
MNYEMIWAIVIVFILIFIRKLVTPQIVRREIFFRGVRLPENLLGDLRLDDFKKAYLRNLSELCGFYTLLFCVILAKFPNNAILLGVVLLLLSAWLYYLSHQRVMQFKQANEAHMLNRKQVVMVDTSFRSDKSKRPLPAKTWFIIPLFIIGFNLLLAGYLAYDELPIVVPSHWNAQGEIDGGVFKTWGLVFLPLVQIFTGFMFLLYQAVGWSKLQIGTNPADSRERNRIRFRFRWGANIIFLNILILLVISLLNL
FVLQLPNVNVPVLLVFQLLIILVLLIDLIFMAVWTGGGGSRLNTGSVTYCQDMDWLDDDKDWIGGLLYFNPRDPALFVEKRFGIAWGLNYGNIKAYILIASIIAAFFLLGSLMELLLFY*
>Syntrophomonas_wolfei_subsp.methylbutyratica_JCM14075T_00744_HTH-type_transcriptional_repressor_YtrA
MFLAIDFQSDIPIYIQLKQQIIAGIARGELKEGEELPSVRQLAQDIGINLHTVNKAYSLLKDDGFIIRNRKGVIVANEEDDFKFRLREEINPLGDLAYQAFCKGMTEEELQLEIGEIYRSYQGAER*
>Syntrophomonas_wolfei_subsp.methylbutyratica_JCM14075T_00745_ABC-type_transport_system_involved_in_multi-copper_enzyme_maturation,_permease_component
MFKLLLKDLVLLVKSSIIWAMTLYLAIIVFSMTTPFDKYIYMAGYGATYILMAGLMAEFKNSSNDILLNSLPAKRHDIVLSKYLTALILTVLAVILGLIINLLPLLLIPIJLSRISMGDAAIVLLMAALTLTIVMPVNLIFSNQAARVGIVIVFMLLFFAPVWIKGFIMDRCEAEWAQFLINAAVSQPAVLLAAGAGLLLLLLALSLIISLRWYEARDF*
>Syntrophomonas_wolfei_subsp.methylbutyratica_JCM14075T_00746_ABC_transporter_ATP-binding_protein_YtrB
MENVLEIKGLSKNLGSFRLDKVSFSLPRGYIMGFIGPNGAGKTTTIKLLMNLMKPDGGDIRVFGLDNRHYEKEIKQHIAFVYDESHYYENLSLLDNRRIVARFYRDWDEKAFQQHLRDFDLPPHKKLKNLSRGMRTKFGLAIALSHAAEFLLMDEPTSGLDPVFRSELMDILQQLLVEENKSVLFSTHITTDLERVADYICFINQGRIVLCQSKDEMLQGHVLVKGALEQLSPEAEKMFIGLRKNHY
GFEGLSADPRGIRQFFGDGVLLESPGLDEIMVFYVRGHHHV*
>Syntrophomonas_wolfei_subsp.methylbutyratica_JCM14075T_00747_HTH-type_transcriptional_repressor_YtrA
LNIVRSNRSEPIYHQIVQQLIRGAIVREELKAGEPLPSIRGLARDLQVSVITTRRAYEDLERDGFIVTVAGKGSFVAPQNAELLREKRVNLVEEKLLAIIQEARSLGLSLPELQRIVELLYEEGL*
>Syntrophomonas_wolfei_subsp.methylbutyratica_JCM14075T_00748_Phospho-2-dehydro-3-deoxyheptonate_aldolase
MKPFKLASWDKKGSYTHIKIDSGAEPLLIGEGYCTIIAGPCAVESEEGYLQIARFLREAGAHALRGGLFKPRTSPYSFKGLGREGLEIMLEARRITGLPLVTEIMDIRDLELLGDNIDLIQVGSRNMQNYSLLEELGKLHKPILLKRGLSATIEEWLLAAEYVLDGGNPNVILCERGIRTFESYLRNTVDIGAVALLKDLSHLPILVDPSHATGKWKMVTPVARAAIAAGADGLMVEHPDPDRALSDGK
QSLTPENFLKMVEQVSIRLVKNL*
>Syntrophomonas_wolfei_subsp.methylbutyratica_JCM14075T_00749_hypothetical_protein
MPSHTIENERPSQERALKVLEKLGYTRISRGDAEQKRGSRKNVLFEGELEETILSKLTYPYGSEQRFFSGGSLATAIHAIHVQHAAGLYAANKEIYEMLCSGKSLEESLPDSTRHIWNTRRLKLVQGVKNQDTAVYEYMIYK*
>Syntrophomonas_wolfei_subsp.methylbutyratica_JCM14075T_00750_DNA-damage-inducible_protein_D
MSGIVKYSDQTFESIKHINEFGQEFWFARELQGVLEYAQWRRFHEAIERAKSACMNSGYTVEDHFADVGKMVEIGSGAERQVDDIMLSRYRASFSLKEVPACQATLYLTSGLSHREP*
>Syntrophomonas_wolfei_subsp.methylbutyratica_JCM14075T_00751_type_I_restriction-modification_system,_M_subunit
VTAYKNRQDAPKFAHVASLDEIKENDYNLNIPRYVDTFEEEPLMDIDQVNANIARLKTEIAEAEKKMDEYLKGLL*
>Syntrophomonas_wolfei_subsp.methylbutyratica_JCM14075T_00752_putative_type_I_restriction_enzymeP_M_protein
MIHSLAVGGRIAAIAPYGVLFRGASEGLIRKRVIEENLLDAVIGLPENIFYGTSIPACILVFKKNRENKSDVLFIDASKKDEDGNFRYVKGSNQNELTEKHRIL*
>Syntrophomonas_wolfei_subsp.methylbutyratica_JCM14075T_00753_putative_membrane_protein_YjcC
MFKPAKKALVDRITKLPTTLISVEPLDLMTNIAVGMVLLGISAFKYHRGCKSILYRKALLLQKIQIDFYGQRNLAACSIGGDDFAVFIESPHTMDAFEEAYLILKKQLEDGINAANQSLGLNQPLHLHMGYVDLQQIPDYHIESLVYKAIKEATYMAKQYDQACEHASWHSLKKMLEQRSIEVLYQPIVSLKSGDILGFEALSRGPGETALESPTIMFKDAERFRCLLELETLCHDKAVYNAANH
LGQKRVLFINTNPAILSNNEYRLGIPSLEGVRCGRNFSNVVVELTEKNSIRDYQQFREVLLYYRRLGFMMAIDDAGAGYSSLQAIAELQPEFVKIDMSLVQGVDRNPTKKALLETFVDFSHKLNFRLIAEGVETLAAMGCDYGQGYLLARPGSLKQEIQPHIREKILLSSRRNGHQFQSEPSKIADIAMFNNCIAPDMPVEEVIEIFNNNKSINGVICVQEDIPKGLIMREKLFARLGSRYGYDLFIRRSVED
LMDRRPLILPWFTALEEAARQVAERLDQGITDYIVTTREQRYFGVVSTAKMLDTMAKLQIVQAKDANPLTGLPGNRCIFTLESSRRDSVLYFDLDNFKAFNDYYGFAQGDRALTMLSQILVETLKSWGNSDDLVGHIGGDDFVIFTGDKADLIAANVIARFEEQIIELYDKSSLEQGYIVAADRRGQIKHYPIMSLSVAGVRNHANHSYANHLQLGEAAAELKKIAKAQQGSAYISDHGD*
>Syntrophomonas_wolfei_subsp.methylbutyratica_JCM14075T_00754_Phosphate-binding_protein_PstS_1_precursor
VLRKIRKMVVLVLALSMLLSVSIPAMGCADAIAVKVDGKKIELDVAPVIEKSRTLIPIRAVVEGLGNVIFNNASTQAISINTADQEVKLTLNSKQALVNGASQEVPAKAVDGRTLVPLRFVGEALGADVAYDAASKTVNVKYFSNMSGTLKIGGSTTIQPLAQAAADKLMAGNQGLSIMTIAGGGSGEGIKGVHAGTFNIGNVSRDLEAADNVSGYPGLISHRIGTDGIAVIVNPKNTVENLTKQQVIDIFT
GKIKNWKEVGGKDAAIVPVQREAGSGTREAFLKLLKKKVIATATPYTSTNSTGAVRANIENAKGAISFGSLGYMDGGAKYLVLDGVAPTNDNVLSGKYPISRILYMYTKDKPTGLAKAFLDFVLSKEGQKLVEDEGFIPVKKA*
>Syntrophomonas_wolfei_subsp.methylbutyratica_JCM14075T_00755_Phosphate_transport_system_permease_protein_PstC
LQSFETSSPEEKLFAVRIETKNRLISVRDSISEKLVFLLTTASAIVIFIFIFVIVKEAAAVLQVNSINFVTTSGFDQQINAYNAPADKPIWQFGALSLGLGTLTTTLGALLLAVPMGMSTAIFLSEIAPNAWRDLLKPAIEVTAAIPSVVLGFFGLLVLVPFIQELFLVNISGYAVYTGVLILSIMAVPTIVSLSEDAIRSVPNSLKEGSLALGATHWQTIVRVVLPAALSGITAAVILGMSRAIGETMAVLMVAGNVPLIPEGFLSVTRPLTSTIALEMDYA
VSGGIGNIPQPGIGMVFLLTPVTLASAIVNKSENISVPTGRRAIGEIAVFLLLVLVTCVGLSLCARLVYLPVKRGGRA*
>Syntrophomonas_wolfei_subsp.methylbutyratica_JCM14075T_00756_Phosphate_transport_system_permease_protein_PstA
VLDQKLRGTGIVLAWILPSSLSIVAVLSIFYLLFRGFAQINPDFILTTPRPTNLVNQDMSGGISTPIAGTFLLTLTGILLAAPWALATAIYLAEYGGSQAMVQIFRLAIDVLAGVPTIVIAIFGLAIFSLPQLGFLSSMVEGVDGVERSFGRSFLVAGITMAIMILPFVIKICEEALKAVPNYYREAALGMGASKWQSIVTVVLPSARQGILTGIILGMGRIIGDTAIVWLTLGGTIRMTGIQPWWYPVHWWSTLC
NTGSTLTSYIYFASPAGEGNMPDKAFGAALVLLIIIVLNIFTDLLERFGANKKGN*
>Syntrophomonas_wolfei_subsp.methylbutyratica_JCM14075T_00757_Phosphate_import_ATP-binding_protein_PstB_3
VEKESFIDGVLVHSQSSTTFSLDSSIISQDLNAWYGQTQALKQVNLKIYKNQITAFIGPSGCGKTTLLRCLNRMNDLIPGFDLKGSIELQGENIYAPGYSVNKLRKQVGMVFQQANPFPQTVLNNLKLPLEENFRKLKAQLKQIAIDKLKDTGLLEEVKDRLQQSALKLSGGQQQRLCVARALAIDPEILLLDEPCSALDPISTSKIEALILELRKERYSIVIVTHNLQQAARIADYVVFFYQGEIVEQGLP
QEIFTNPRMKLTSQYLQGMF*
>Syntrophomonas_wolfei_subsp.methylbutyratica_JCM14075T_00758_hypothetical_protein
LRISRFFPLLQNSDLAVIRQVGVLLQLARSLDRSMGSGIINDLVKHVDIRGDVIIKLSVQIMRSWRIFAPVISSLMRSKYSIRIFLLCKGSRVSAFSVFEPYTAHISRGQRKEGNYRR*
>Syntrophomonas_wolfei_subsp.methylbutyratica_JCM14075T_00759_putative_phosphatase_YcdX
MLKEADLHIHTTASGHGYSTIKEIADIAEKGLKMIAITDHGPAMHWGPHIWYFYNLRSLPRVMQGVEVLRGVEANILDLEGNVDMPEIVMSDYLDIVLAGFHDETGYSGRGVEENTQAMVAAIKNPYVHIIVHPGNPRFPIDVEQVVLAAKQYNKAIELNSSSFVVRPGSQSLCSQFAKAAKKHNALVSINSDAHICYNVGENAKAMDLALDAGIRNSQILNSSSRKIDEYLSWHRQQKPVLQAQ*
>Syntrophomonas_wolfei_subsp.methylbutyratica_JCM14075T_00760_hypothetical_protein
MKYLKFLSYLIRHKWFVFLECLIGVPWRGITHDLSKFLPGEFIAYANCFYANQHCSEQMEAPNSKESYMLAWLVHQKRNRHHWQWWILYGEENEPYATAMPDQYIKEMVADWWGAGRAQGKPDIIAWYQANRNKMVLHRATRRRVEYLLLHYRITGVGYEQGSAGIVNGLLINAMENHSLSI*
>Syntrophomonas_wolfei_subsp.methylbutyratica_JCM14075T_00761_hypothetical_protein
MNKLSIRKQIITFGCIIFTVGLLTVVAVNLQKEQFLMAEQLQDAAGNIVEKISVMAQLLDSRQFDSKLGYYLGNQRSSFLQRGYHLSQYMVSKQGTAVKAYGDLKTMPLTQNEINNMLQKERGILHVKVVEQMYTVAYDYSLERQSLIVLIIPELDYLKPVCRINTIVGIGLLSLFVSYLAAMFIIGITRPIDRIVEQVQRVEKGHLDLSLPKSGTAPELLVLAATINRMLQSIRYFLTKVKEMVAR
LQLSESNELAHQAGKSIDTIDRQIEDVSKAISEMSAAVTETAQMSSQMGATIKQVSQTVEAMAFQLESQSVGIEQVASSVETIAKLSQEMSHTSQELRRLTESVRRAVLSSQEITGRLTAVAGKAHAIIEQINEIADQTSLLALNAAIEAARAGEKGRGFAVVAEEVRKLAEQTAQSANEIAHIITSLYNKIQNVTEHTAKIVNINVQNADLTEGVLKHIDGIASQSKPMVDNFIHQISAGMEQVSGSLEQAISEVHNQAVESNMSSQAL
SHTIVNSIPEILQSSYNQAENTSRMLEHSSHLKEALVEHQVTNFLRSEL*
>Syntrophomonas_wolfei_subsp.methylbutyratica_JCM14075T_00762_hypothetical_protein
MKRFKSAIKLKFLGSAVLRFPLTVFFLICCTTLTCYMSILHKSFHDIMIYQLKQLMFLVPLLFGFIGVTAQFACERFPFNRLARLRWALYALSTVIFISPAPAIDYAGVGARTSVAVISMFCAFIWLPSYQGKFDVNSFVAALIHFKSSAVVSAVLGLIAIALAALGISLIIIRPMMGGNLLKYSSDAYGYMMAIVWILFATTYYLSLLPRFNSETELDQTYAQEASQCPHFLEILVSTSYLYLAISPLVAGYTLYLLAYFVKGIVTMNWPSG
QLQFIMLAYSAAGLIIYLLSSLENRFAVLYQRIIFPKALPVTVMQLSNVVIRLSAYGVTESRYYIALFGIIFSIVIAMLSLSFTPVKKNGLIALLAAGFAILSILPPADAFTVSRNSQITRLENYLQTEGVLVNGKINPKSGDADMTLRLETTSINLYLERRGYLKEVAWLPADFQTNRDMEKTLGFPAYKYLGVDQNNFFAHLDMQKPINIEGYDVLLQAGTYRGMDPKIEMTYNFEVHGFSYKILILDPVSAKEAR
YSVQNIGKKLISTGLYDFVSVSTGSNLPKSKKSLDAEDLTLDVENNNKRMRIFQSNIGITYSTGDGADYNMFIILSPAQR*
>Syntrophomonas_wolfei_subsp.methylbutyratica_JCM14075T_00763_Bacitracin_export_permease_protein_BceB
MGHFVQVYFAISPQAIFNTVTVFLIFPVIS LHAYSLIYRFKLIVDLFRAGQSGEGTEPKASLVLALVSMALIVAGYSLAFHLTENFLLYAPMVLALVIIGSYGLFHSFMVFVRLSKKNTSQYYRGMNLISVNLLYRIKSNATLLATIAVLSATTVTAVGMSYGIYYDINNAVNYHNPFSYVYWSNADRDQKVNQTLLQYPQNQLLHSVEIEFISARGSLPGLDTHFALISESNYNKLAGQKIKNLKPGEVI
AVIVHSDYDFRGDFVDLLDKPEFLVDRRPKILTVDNFSYLFGTAKEKNLMLLYVLEPQRLLMGDLGRGALSLLVIKNRALLAIPELLRNQKRKVDGTSSMDITATIIKKCVYLWLQDLGYTCRIAKQMSSQKFLDDAKSFMWLIFPITIFFIACYYVGLAQRLNLVAYLYFRLLDAVSQRPSLIA
>Syntrophomonas_wolfei_subsp.methylbutyratica_JCM14075T_00764_Bacitracin_export_permease_protein_BceB
VILFSITAKSIKKNFQNYFLFYVSMVFSIVIFYVFTSIQYNEQVAQTVCGTTTRYMLKTCLSVAVFSTIFMGYSNSFFIRKRKKEFGLYSLLGLQKRQVARMLFYENLTNGYCSTWCRHYSGEYIFKTLCHAFLQTYGTFCTGIFRHIPTSYI*
>Syntrophomonas_wolfei_subsp.methylbutyratica_JCM14075T_00765_Sensor_histidine_kinase_GraS
MQIRAIHTEKEALYSIPAAQNYEHKLFKKLLFIKYDEQNSRLEKIHAIEKKNEFFISSWIHEIKTPIAVSKLLLENNSCKDEGGLLNSLEEIEELIERLVEQVLYYSKIDDFSRDFYVQELELNRVLRESIKKHAKIFAKNRIIEMENLDITVTTDKKWLLFILDQIISNSLKYTPPGGLIKVYTEKDEREKRLIIMDNGIGIRPEDIDRVFERGFTGYNGREFNKSTGMGLYLARRLARKLGHDMSIDSVGYETRV
MIHFLKLSKGGRLS*
>Syntrophomonas_wolfei_subsp.methylbutyratica_JCM14075T_00766_ribosomal-protein-alanine_N-acetyltransferase
MVEIIRTPETKIQRLAEMETQAFEGGGLNEWTFVPFIRHGLVPCIRQGEVVGYAMFRMDWEQPSIAYLIGISIDESHRGKGLGTTLLRDSMEELKRYNITNIELTVSPENHSAVAVYQKLGFEVIEHRKNEYGDGKDRLAMSIIL*
>Syntrophomonas_wolfei_subsp.methylbutyratica_JCM14075T_00767_HTH-type_transcriptional_activator_TipA
LEKQLKIGEFAKLTGATVKTVLHYHKMGLLEAEKRSPGGYRLYGVAELNRMRSIKRLKYLGLSLEQIRGILGEPGGHKSFRSVLLAMQTELLNQIQTLTERVEKIQILLQEEQFNLLECTNESPSFKMVVAILGEEARFQYLDLCPEIYEQFRKLYEIIDDLQWGVDYEDILREVAEYFRDNPDKYYDSLVFGTKISAIAELLPDSPAVKELASEYAEFIKSLFPYQKLMSHESALQKSLNSLWRDMFAEVYSP
AQIKFVELLEKGSKSGQNRD*
>Syntrophomonas_wolfei_subsp.methylbutyratica_JCM14075T_00768_hypothetical_protein
MCRKAVQLNRLRRYTLPIMIVVVGGIIAHNTGAVSKDNLNWYGLLPAVLELLFIVICLINISKIVRRYRRLKSEGWEVLDAWGNSIPRIARMLIEPRLYHTLYLSIKGKAVANGEPGFGTCRDSYAFFVKVFIFLCLLEIIVVTILLPHRWMVWKMLHLLGLWAIMFWADYRAMDYNHSLSLTGMNLRGLRLRCNWQICWDDIARVQRISLTAPNGTMSPGMVKSHPGLFYLGLGSICNVELI
LKGPRCFQGMINEIPGVARIMLSLKEPDDFTQQIISIKPELYATEKDKSDK*
>Syntrophomonas_wolfei_subsp.methylbutyratica_JCM14075T_00769_hypothetical_protein
MNLSRYVLPGGSPFRSKKSDSNTGQRKKNRATQMSNMDILFAFLLAAILFCSFSLILSYAQTVVKSKATT*
>Syntrophomonas_wolfei_subsp.methylbutyratica_JCM14075T_00770_putative_ATPase_(AAA+_superfamily)
MSNDGLNLQRLLLYRQILKDETIRKAQELVLMMDTPSQKLRSVEKCYFAELQSLIKAAERNKWNGDLWKNHVLELILDDENIFSLACEKNGEKISAGLYQSALHDIAVLKELFNFNLPEIAEKLGMNTSVFSFNFQSDGEDHFHTPYIFKFHQLKELFTQDESPRALLSSLAEFYHVAGCGTMRKFHAFSWNKGLQGIENPDPVMLEDLVGYEYQKKILLGNTEAFISGKKANNILLYGERGTGKSS
SVKALLNQFGDRGLRMIELGKRQLLELPDIKTVSRGKNYKFILFIDDLSFEEFEDDYKEIKSAIDGGLQASPDNMLIYVTSNRRHLISENYRDQKEDNLRRSDEEVHISDTYQEKLSFADRFGIMISYQSPEQEEYLQIINELARRNKVAMPPKELEKLALQWEMQRKGRSGRTAQQFITHIMASNRPNAETWYS*
>Syntrophomonas_wolfei_subsp.methylbutyratica_JCM14075T_00771_Phd_YefM
MEAAIRSADLRNHYSEISKQCKESRNAVIITVNGRGDTAVLGLQYYQMKSELLRLTLAEAEDDVKNGRLAPMQDSFDDLRASLLRKNG*
>Syntrophomonas_wolfei_subsp.methylbutyratica_JCM14075T_00772_Plasmid_stabilisation_system_protein

MKYKILRTDKAEEQLREIIFYIAEDSGDIDIALGYLDKIETAINRLQEFPESGSVPRYSILKKQGYRVVIVERHLVFYKINDADKLVIIYAIVDGRREYRNLI*
>Syntrophomonas_wolfei_subsp.metylbutyratica_JCM14075T_00773_hypothetical_protein
MRKQIVAIKAVVFMLLLLAFLFLANKPEQHATYDLNHDGIMESYHLTRGKLTVTQPGGIDWSSPPEWNVQSFALDDATGDGTPELIMVLWKRGSFDRHKPLWYEQEENNYSCHLFVHRLIGNKLIPRWCSSALDRPIKSFTVEKDPSGKTFLAVEEGFYSSYCAGRPIIIGKEYSNWTWKQWGFYRI*
>Syntrophomonas_wolfei_subsp.metylbutyratica_JCM14075T_00774_hypothetical_protein
MNHRRLGPQLWCGLLVAALLMMAGCHAGSSTGDKDDKDANPGAIALAQEFAPYKEISVQDTAIVKPYKVAPDLSNVINAERFDFSDNARESLIKNGFVVLPAMGHEFFMTYEINRYDNVPNFITTDAVVHNYHLFYSHLLQNVEQQALIPELKKLNASLLQRAETDYKDLKGSDWENAARRNLAFFAVGSKLLNPDTRIPAAVKTEAEQEISFIKKHDSTKLSPVMAMGTKPDLIEGLKEDYTQY
IPRGHYAKTDDLKEYFQSMMWYGRITFRLKDADETRSAILMTLALHNGIGAREWEKIYTTTDFMVGKSDDIGYHQYIELLEQAYGKNLTPKKIAEDSQSFDKFRQLAKDLQPPVINSIPIFDATIQPDRDKEVLGFRFMGQRYTLDAEIFQHLIYREVTENPAGERRMLPSGLDVPAAMGSDTAETILKKQGAFEFKKYSTNMAKMQKYIAGLNDEWHQNLYWSWLYTLLPLTVDKSDGYPSFML
NEAWNHKELATFLGSWAELKHDTILYTKQNYAEMGGGGMEEIDDRGYVEPNPHLYARLASLTAMTRDGLKMRGLIDQKDSQNMDQLNELVLQLKVISEKELANKALTEEEYELIRTFGGQLEHFWYEVYRGDGLESRSQISDFPAMLIADVATNPPAEVLEVGTGYVDNIYAVVPVAGTLRIAKGGVYSYYEFPWNAADRLTDQKWQEMIYNDKAPALPEWTANYRIKGTASVDLLQIFN*
>Syntrophomonas_wolfei_subsp.metylbutyratica_JCM14075T_00775_prevent-host-death_family_protein
MLQIRPVSDLRNNFADISKIVHETAQPVFLTKNGYGDMVVMSFEAYEKLQFESEIYFKLKEAEMQARLTNQRFSHKEVFNRLRQTLMEKADNENV*
>Syntrophomonas_wolfei_subsp.metylbutyratica_JCM14075T_00776_Plasmid_stabilisation_system_protein
MYEITYLPIAEQDIAEIIIYISNQLKAPKAAIELLDAFDHSIFLLQEFPYAHKIYRLTKPLEEEYRLLPVKNYAVFYVVREQDKSVEVHRVVYAKMDLSQILK*
>Syntrophomonas_wolfei_subsp.metylbutyratica_JCM14075T_00777_hypothetical_protein
MRTGTANLPLHGGKCPAWLFTHMKALSRAIVEVILQDDGPDEVLRRLSHPYWFQALGCVVGFDWHSSGVTTTVCGALKEGLVEIGPQAGLFVAGGKGRTARQTPLEIMNHADRHGLSCSAEDLVYASKMSAKVDSAAVQDGYDIYHHCFFFTKDGHWAVIQQGMNEDLRQARRYHWLGDAVESFTTEPQAAICCDFTTDTLNLVANENENIREISTHLVQENPDRLLAEIKRLQESSLTLP
TCHPIPRTGYLNKALYAAYDKHPQDFETLLNVSGVGAGTLRALCLVAEVAYGARPSFSDPVRYSFAHGGKDGFPFPVNEADIEQSYITLRRALRKAKAGQREQLDALRKLARWHSEAIKVQASPISPSPPQPASLEKSPVRQPGLFD*
>Syntrophomonas_wolfei_subsp.metylbutyratica_JCM14075T_00778_hypothetical_protein
MQYEQVLLKLAPCGLDCSRCAGYRDGEIARLSRDLLSRLGNYRRLAPIRARTDYEFAGYEQFEKY*
>Syntrophomonas_wolfei_subsp.metylbutyratica_JCM14075T_00779_4-oxalocrotonate_tautomerase
MPLVKIEILQGKNREYKKALMDGVHNALVEALKIPDYDHNQRLYELDAENFEYIDTRTECNTIIEIAMFAGRSRAAKVDLYSAICRNLETNPGIPATDITIVLNEIPLHNWGIRGGKPADEVDLGFTVKI*
>Syntrophomonas_wolfei_subsp.metylbutyratica_JCM14075T_00780_DNA_polymerase_IV
MAELTNILHCDLDAFFASVEQLDNPSLRGKPVVVGGSIHSRGVVSTCSYEARNFGGLRIITESEALELLRPLPVSRIWGIGQKTERRLDKLGIKTIGDIQDLPPEWLEDKIGSAGRLFWELAHGIDKRAVEPEYERKSLGREETFPEDINDTAYLEKLIAQFAAELCRKLRQEALFAATITIKLRYSNFKAITRSKTINPCNSDIIVTQIASELLHHSYNNKHPLRLFGLSLGQLSSVTSLEQGILFEPQLNADYK
NIDRLMDEIRDRFGPGAIKRANLLPHKDIE*
>Syntrophomonas_wolfei_subsp.metylbutyratica_JCM14075T_00781_hypothetical_protein
VNTQSLKSHAEEIKTNLGVLFLAFKDKRTPWYAKALIFIVISYAFSPIDLIPDFIPVLGYLDDLILIPAGIALAIKLIPDEVFLEYKEKQNGLYHITIKGIYAGIIIFSIWLILFYLIVKQLAF*
>Syntrophomonas_wolfei_subsp.metylbutyratica_JCM14075T_00782_hypothetical_protein
MLVSQAHRKNCTRNRLYCQRRGVRDVDPGNTKKNILRRDIVQSQVGVD*
>Syntrophomonas_wolfei_subsp.metylbutyratica_JCM14075T_00783_putative_acetyltransferase
MIITKCDDSAELSSFLRLDYTNNLYFFTYLNEIFHNSSVEVLIAKYKSKIVLALLLTPIHCCISSSDINYVNAIADQLPIIHSVHVVGRKDLVEHLLRISKGPERDKHIYSFCEYTPAIVSNNPATVSQKAAESNLKDLIKFYSNNDMLIDAASRLPAILSWGKAYFIYKDHEIVSCALTTTQTNDAAMIGSVYTIPGYRNHGYAKDCVSNLCRDLTSNHIKPYLFYKSDDDFLAEFYKSLGFRETNTWILATSNRSF
CSRRKRTYRPSDPCRNYESC*
>Syntrophomonas_wolfei_subsp.metylbutyratica_JCM14075T_00784_Cd1
MRILVLNGSPRKNGTVANLLKSVIDGIPNKYEVEWINVYELKIEPCIACMKCRQDQECILPQDDAHIVGRKINEADGLIIGTPTHWGNMSAQLKLLFDRNVPVMMGEKRNGMPYPRQKGKPAVIVTACSTPWPLNFIVAESRGAIRAVNHVLNYGGYRILGKIIKPGTKSRPQISNRLLKKCRIVSNRF*
>Syntrophomonas_wolfei_subsp.metylbutyratica_JCM14075T_00785_putative_regulator_PrlF
MNLAKVSANGQVTVPIEIRRKLNLKEGDKILFIERENGEIVINNASATAILKAQKAFAGVAEKAGIQDEDQVQILIDEVRYGKDSK*
>Syntrophomonas_wolfei_subsp.metylbutyratica_JCM14075T_00786_putative_toxin-antitoxin_system_toxin_component,_PIN_family
MKILVDTNILISAILWPNSKPAAALLHVARFHELVLCDRNIFELRDVLRRKAPQALADVGVFLAELAYVLVPAPEYPQKLISDPKDQPILNAAIVADVDIIISGDKHFLMLDMERPRTMTAAQYLEWIVSGG*
>Syntrophomonas_wolfei_subsp.metylbutyratica_JCM14075T_00787_mycothiol_synthase
MEKALNSRLTDDNKIVPLPMEQIVIREAQPSDAPAMARINVATWRDSYGKFLPRQLMEELSCSQKQSEIENFFARWEENQAVALVAENKSGEMMAFIMAGKNHRQDLRYMGEIYNLHIAPAFQSRGIGCCLVYRVSQVFRQRNWNTMMVWALAENPSRRFYEKLGGRIIAQDIDKYRGFTAPVIAYGWNKLPICF*
>Syntrophomonas_wolfei_subsp.metylbutyratica_JCM14075T_00788_hypothetical_protein
MDAIVILIGKRMESAVKVQQILTDNGDIIRTRLGINHELSKDENAAGFIFLELCGDEKRVENLCDVLNAIGDVKAECLKMEIPGNGCGCSC*
>Syntrophomonas_wolfei_subsp.metylbutyratica_JCM14075T_00789_DNA_polymerase_IV
MAEPTNILHCDLDAFFASVEQLDNPSLRGKPVVVGGSIHSRGVASTCSYEARKFGIRSAMPTAQAYRLCPQAVFLPVNMPRYLEMSKQVFSILSRYSPIMEVISIDEAFLDISGCSGLYGSQEKIGCLIKEQVYSELGLTISVGISYNKFLAKLASDMDKPDGLRIITESEALELLRPLPVSRIWGIGQKTEQRLVRLGIKTIGDIQDLPPEWLEDKIGSAGRLFWELAHGIDKRVVEPEHERKSLGREETFPEDI
NDTVYLEKLIAQFAAELCRKLRQEELLAGSVTIKLRYSNFKTITRSKTITPCDSDIIVTQIAGELLHHFYNNKRPLRLFGLSLGHLSPAASLEQGSLFEPQLNADYKSIDRLMDEIRDRFGPGAIKRANLLPHKDIE*
>Syntrophomonas_wolfei_subsp.metylbutyratica_JCM14075T_00790_hypothetical_protein
MPVKISELIDEMDMQMDEYHTYLNKETNIIVTVSNEGLSIAEESDEGDDFSQYPDWQKQNIMEALDIIVNWDKYVELPDKYDINEYNIMENFCFSLDNEKIMNELLYAIRGRGAFRRFKDKVQRLGIQQQWDKFREETFKEIAIEWCEYNNIPYI*
>Syntrophomonas_wolfei_subsp.metylbutyratica_JCM14075T_00791_Cypemycin_methyltransferase
MGYYIDFNKITLEEFQRMLESTYLLPSQQIIAEDISERFQVLQSLGINNMQQLQQTLKNKDAVNRFSEKTSLPVDYLTVLRRMVNSYHPQPRIIKDYTVLSIEITDKLERMGIKTTPQLYDKVAAKADRNVLKKELDINDEEALLLARLADISRIRYVNPAFASLLLNSAYDTVIKIRNADYREMYEQLVKLNKDRRYYKGRINLKDMEFLINDTDYIPLDIEYEYDIMRIIKDNKINLMAMQESIRKPALYG
KSTAKFWDDEYISEQMLKLHLDPEVESASKTEETIAAETSFIINWTGMDEEKAVLDLGCGPGLYVKKFAETGAKVKGIDLSQRSIDYANRYIKAGHENIYFTRMNYLDIDYAESFDIATLIFYDFCALNSTEQNRLLDKIHRSLKSNGVFIFDVVTVNRESSESTSISIWESGFWSPDPYVEIMNTFIYEKPRTEGTQYTIIAEDGSIKIIRLYHRLFEMAEIQKLLNDHHFKVENIYNNLKGESLTENSETYGIIA
RKAKKL*
>Syntrophomonas_wolfei_subsp.metylbutyratica_JCM14075T_00792_putative_permeases
MVIWLALVGMLCWGIAPIFAKLGLQNVNPATGLVLRTIIAVVLVSSWMGVSGGITQFRQITLHQSILIAVEAILATVIGDLAYFAAIKGGNVSIVSMIMAASPLVTMICAALFLGEPITAWKCLGAGYIIMGIFLLL*
>Syntrophomonas_wolfei_subsp.metylbutyratica_JCM14075T_00793_Vancomycin_B-type_resistance_protein_VanW
LYIRYIRIGLISLLILLLGTINHAIYTDNLSDRPGINLDFLPYATSKERIIYSTPSASSSVTLLPWENDPDFQAAVAENEAMIQLANYCTVLQDPLPGEETNVHIAARLLKGTVVKSGQIFSQNEKIGPYSQDKGFQHGPVYIGYQLKTTVGGGVCKIASTLYNVTILSNLPVIERYAHSMPVPYVPLGQDATVCYGVKDFKFLNNTPYPILIWAESIENRLYIAFYGQTTPPRVKWHHEIVKEIKTYNIYRKNYA
VEAGKEKVILQGMDGAIVRSWLTIEDPQQGKIITKKLGKDYYNPMPSIVEKGMKKATSIPLH*
>Syntrophomonas_wolfei_subsp.metylbutyratica_JCM14075T_00794_Arginine_decarboxylase
MDQERTPLLDALKSYVEENVIPFHVPGHKQGRGNPELRKYIGSNVLKMDVNGMKELDYIGNPSGVIAEAQHLCAEAFGAEQAYFLVNGTSSGVQTMIMSACNPGNEIIIPRNAHKSTIGAIILSGAIPVYVQPQVNKMLGIATGVSAESIQKAIEEHPQAKSVFIINPSYYGIHSDLKSIVEISHQANMTVLADEAHGAHLYFHPEFSLNAIEAGADMSAVSMHKTGGSMTQSSVLLHQGDRISVDRVK
QVLQLSYTSSASYILMLSIDIARKQLATRGQLLLNKTLALARQVRQELNDIEGIYAFGKELVGSPGCADFDETKLGIHVRKLGLTGYQIERILRERYKIQIEFSDYNALAILSLGDRRENLRKLVAAFREIAGERESKDASKWFNIPFCPEMIVTPRDAFYSPKRIVPLELSRGEISGEMIMAYPPGIPVICMGERITQEIIEYIQLLRVEGCELQGATDAGINHIKVLGSALPQASVSKSVSQPRSAYQEIPVYLSP*
>Syntrophomonas_wolfei_subsp.metylbutyratica_JCM14075T_00795_D-alanyl-D-alanine_carboxypeptidase
LSHAAELLDYGFNRYTKKILLPQGAICGIVQVKNGSKDYVQVKTAEAVCSVVLKGQEKNLNSEKKLESAVKAPVKQGQKLGEVLVYNNQGLLRKVNLLASQDVPATNPSIISKDSLTAYGFPALLAIIAIILYLAHISRTQYKIGIIKKKQDGKYHNRR*
>Syntrophomonas_wolfei_subsp.metylbutyratica_JCM14075T_00796_(R)-specific_enoyl-CoA_hydratase
MLGKTIDEINVGDSADFTKTVTGYDVYSFAGVTGDFNPAHIDNEFASETSFGKTIAHGILSIGFISNVLGTQLPGPGSITVGLECDFKKPVYIGDTITATVEVTKKDEARNRVWLRTYCTNQRGEIVVDGEAKMMPVTKKP*
>Syntrophomonas_wolfei_subsp.metylbutyratica_JCM14075T_00797_(R)-specific_enoyl-CoA_hydratase
MLGKTVQEINVGDTASFNKSVTEYDVYSFAGITGDFNPAHINAVYASETSFGRIIAHGILSIGFISNVLGTQLPGPGSIYIHQECDFKKPVFIGDTITATVKVTKKDEAKNRVWLHTYCTNQRDEIVVDGEAIMMPTRKKPA*
>Syntrophomonas_wolfei_subsp.metylbutyratica_JCM14075T_00798_RNA_polymerase_sigma_factor_CnrH
LEYSQLELVRGIKNKDSGAYEYMISKYSKPVYYLAHSILSALHSKEDIEECVADVFLDAWIKIAEFNEEQASFKTWLLILTKYKAFSYKRKRRRHLDNIVDIADIEARDNINLEKQVILRQEQEQVMDIINNFAETDREIFFRRFLLGEKINDLARAFNLSRAAVDNRLLRGRKAIKEGLKKGGFEI*
>Syntrophomonas_wolfei_subsp.metylbutyratica_JCM14075T_00799_hypothetical_protein
MNEDRIILMLSRLDDDRWEKEIDKLMEGVDFNMESIKRKAVQKLAQHNTSIIKKSSLRKRLAYAAAVCACFVCLNVVYADEISETIKSFFNKTPVYSTMVDGKAYYLPQSLKLDDKTTVNSFMVSADRMEMELTSDLSPELLCSMKIVPKNDPDTEYTIGEYSEDGEKKYCLGLINAKEKNYKFKPAKEFNLLVGGQTYAITLEEARSLDGTQKLTESGPSSNKIDLVTVGANSIEKDGQRAVQLIASFK
DQDMKLSAFGERLDKISKLTVEDLGKDGIVSSGTAPKLADIYATDEKGNKYKLEKPQGARTMPLTTFVTGAPKDSSLTVNLPALQACYKKKVGSFNINIPKDGKESLNREVDLFAQKAVVKSVTRISPTSAKLVMQLNTGDQKFVKIRSFCIHSKDIRKIESEFSGDRAIMTLEFNKDVNVADLEISWPEFEMNGNWTINLK*
>Syntrophomonas_wolfei_subsp.metylbutyratica_JCM14075T_00800_Metal-tetracycline/H(+)_antiporter
LTEERIFNRSSATLFFSIFMVGIGFSIIMPILPYYAESMGASAFQLGLLMTVYALCQFIFAPFWGSYSDRVGRKPVLLVGMFGFTLTFYIFALANSLWVLFVARIAGGALSCATVPTAMAVMGDTSSPEKRGAGMGMVGASMGMGMIFGPAIGSGLAHISLAAPFVVAGSLSLLICFCILFLVKESLPVEDRVSEAQKIDRAPLLKGLKSPLAFLFTAMFLASMAEATNMGTFALFAEGKLGFGPTSMGLIFS
CAGLASVLVQGFVVGRSINKWGEEKTSGAGIILMAASFALFLQAKSLLELIIYMGIFSAGTGLIRPSISAATSKRTTGSQGTAMGVLQGYDSLGRVIGPSLGGYLLDMNLSYAYISAIFFSGLAFITLLFNRSRGENKEHEFS*
>Syntrophomonas_wolfei_subsp.metylbutyratica_JCM14075T_00801_Gamma-glutamylputrescine_oxidoreductase
MAAKLEFPDNTNPYWIASTEETNYPMLNEDINVDVAIIGGGIVGISSAYLLNQAGLKTAVIEADRILNGTTGHTTAKITSQHGIIYSNIKQQMGEDSARQYAEANQSAIQHIAETISMNGIDCDFKWCPAFVYTCSDEYISELEEETQVALSLGIKASYLDSLPLPFAVKAAMRFDEQAQFHPLKYLKALAQKFTSEGGLIYENTEAVDIDHNDRPVVVTRKGNKVTAEKVIIASHFPFFDGGGLFFTKIYP
EKSYIVAAQIEEQFPEGMFISAEGPGRSLRSQPFKNREIVLFAGEHHRTGHGEDTNVHYQNLFNFAQENFNVQNVLYRWSTQDCMTIDGVPYVGQLTPQNPNLYVATGFGKWGMSNGTAASMILKDLISEGDNPWARVYSPSRFFLNLSSVKSFIIQNATVAKDLVAGKLEGLPGCSELDKGEAQTINFEGDRVGIYRDENDKLHMVDTTCTHLGCELLWNNAEKTWDCPCHGSRFSYKGEIV
EGPAIYQLHQANDEANIVEAKIFK*
>Syntrophomonas_wolfei_subsp.metylbutyratica_JCM14075T_00802_Isoaspartyl_dipeptidase
VFKLLKNGHCFAPDDMGIKDILLAYDKICDIKDKILPADLWDVEVIDCQDCLVCPGIIDQHVHITGGGGEGQGPISRIPEIMFSKIVEAGVTTVVGVLGFDSITRSIAGLLAKARGLEAEGITTYIYTGSYGSPTETLTGRVLTDIALLDKVIGVGEIAIADYRSNHPSLQDLRTLASEANAGGMLGAKAGVLHLHVGDGEEGLQSLFRLVEESDFPINMFVPTHINRNATLFDQGIEFLKKGGQIDLTAGE
TKGYSVAEALAMLVAQGINLEKVTVSSDGNGSASNESGISNIDQLFYDIRACILENNLQLSMVIKTVTLNPARILKLYPQKGCLLPGSDADILILRENDLSIYRLLARGQVVVEDQKAVKKGKYEN*
>Syntrophomonas_wolfei_subsp.metylbutyratica_JCM14075T_00803_Cyanophycinase
LGHYKKGELVIIGGAEDKYGDSRILEEVARIIGGEDARVGIMTTATQHPEEVGEEYRNVFLRLGVRETDIISINSREEANLDDNVRRIRDVSGFFFTGGDQLRITSILGGTKVYEALLHSFYHGTPVIGTSAGASVMCSTMIVEGNSNDAARKCTLKMAPGLRLLEGVIIDQHFDQRGRLGRLLCGIAENPGILGIGIDEDTAIRVYPEEYFEVLGNNAVTVIDGRSIKYTNVSELEPDQILTITNASLHVLSK
GYGFDFKRRELISIN*
>Syntrophomonas_wolfei_subsp.metylbutyratica_JCM14075T_00804_Cyanophycin_synthetase
MEVVSMRNYSGRNIYSHKSVVKMIVELGDWAEITTNEIAGFNDRLLSCFPGLGSHHCSPGYEGGFVQRLKKGTLVSHVTEHLALELQCILGYDVYFGKTRLIKEPDRYCIIYEYINESCAMEVGNAAAEIVLALARNEAIAIDKVLKRLQQISFDSKLGPSTQAIYDEARRRHIPVRQLGGDSLLQLGYGKQMRFIEASLPGSTSSIAVDLAKNKQMVKDLLKENNIPVPCGGIVDSEDGAVVLAEQIGYPI
VVKPWDANQGKGVTTNIKDENMLRKAYRLANSFAKRIIVEKYIRGKDYRILVVGNKVAAVAERRPPYVIGDGLHSISELLEEENKNPNRGIGHEKALTRIHLDSVAKEFLARSELNENSIPKIGELVYLRENGNLSTGGSARDCTRDIHPVNKELAIKAAKIIGLNVAGIDMVAENISEPLTLQNGAIIEVNAAPGLRMHLYPSKGEGRNVAADILDQMYPAGSMSSIPVISITGTNGKTTVARLIRHVLSLA
GQKVGMTCSSGTYIGKECIAEGDNTGPLSAHSILYNRDVEVAVLETARGGIIRRGLGYDLADVGVITNISGDHLGMDDINTLEDMAFVKSLVVEAIKPDGYAVLNADDGMTEHIVRKASCNLVLFSLNRDNILVEYHIKQDGMAVVIEEDLIVIYQNKIRDEIVSINEIPITFNGKAICNIENSLAAVASLSAFGLAKNVIRLGLMSFTPDPTANEGRFNLFDMGSFRVLLDYGHNCSAYQSVMQFINNM
NAARLVGIIGVPGDRLDKTIFDIGQISGQAFSKIYIKEDEDLRGRSAGEVAGILYNGAVSGGADRKNMEIVLSETEALKAAINNALTGDLIVMFYEKFDKAYELVESFIKGQTQPIPLLPDTDTCFIYQSIY*
>Syntrophomonas_wolfei_subsp.metylbutyratica_JCM14075T_00805_Phospho-2-dehydro-3-deoxyheptonate_aldolase
LIIVMERNAAPEQLQVVKERLQDKGFKVHLSQGVERTIIGAIGDKTPALMELFETMPGVEKVVPVMQPFKMASREFQMEQSIIKVGNTEIGSGQVQIIAGPCAVESREKFLEIAKLVKESGATMLRGGAFKPRTSPYSFQGLEKEGLEILAEARQITGLPVVTEIMDTRLIEMVAGFADILQVGARNMQNFFLLRELGKINKPVLLKRGASATIEEWIMAAEYIISSGNNQVIMCERGIRTFENYTRNTLDL
SAVPLIKQLTHLPVMVDPSHGTGKWKLVEPMAMAAVAAGCDAIMVEVHQNPSEALSDGPQSLTPERFGKMVRKIRTIASSLGDNNY*
>Syntrophomonas_wolfei_subsp.metylbutyratica_JCM14075T_00806_Arogenate_dehydrogenase
MQADVLIVGVGLIGGSLGLALKDSPLVNRILGSDRDTASLDKALEMGVIDQAVSLEEGARQARLVFLCTPLRFYPEIINSIKPHLKPGSIVSDVGSTKEEVCRLLAALPEGIWAIGGHPMAGAETRGVQGADRYLFENAVYALTPLPDVPSPVLDFMVELLQNTGARIRFMEATLHDQLVATVSHIPHLTAAALVSLTGGRNEKLMMAAGGFRDTTRIASSNPELWEDILFSNREQIVPQLEQLISSLTII
KEALAGGDHDNILGLLHEAKAIRDKIPRVRRGLIPEFCDIICIVPDRPGIIGSLGQILGCEGVNIVDIEILRVREGDGGTIRLGVPRLEDAERAVIALQKQEIKAWTR*
>Syntrophomonas_wolfei_subsp.metylbutyratica_JCM14075T_00807_3-phosphoshikimate_1-carboxyvinyltransferase_1
MRRINSASKPLKGEVSVDADKSISHRAVIFSALAQGKSVIKNFLPASDTLSSCSCLGQLGIKIAARDSEMQVYGRGLTGFSEPHTVLDCGNSGTTMRLMAGLLSGQLFLSILNGDESLNQRPMKRIIEPLGFMGARIQARENGNYPPLVIIGNRLSGLRYQLPVASAQVKSALMLAALNADSETLLSEPQKSRDHSERMLTAMGADIAVNGLEIRLKPGKELQATEFLVPGDISSAAFFMVAASIIPGSELLI
RHVGVNPSRAGIIEILKEMGARIKLENERIISGEPVADLIVSHSRLQAVNLDAAIIPRLIDEIPILAVAMALAEGESTVKGAAELRVKETDRIAAISSELAKMGVDIREQPDGFVIKGKPDSLQGARVNSQGDHRIAMSLAVAALASKRESVIEDSEVVNISFPRFWDLLNTVTS*
>Syntrophomonas_wolfei_subsp.metylbutyratica_JCM14075T_00808_Cytidylate_kinase
MKIAIDGPAGAGKSTVAKTLSQLMSYIYIDTGAMYRSLTWKALQTGTNLDDEKALYELATNTNIHFQKDDSQQKVFCDSFDISEEIRSPQVSSYVSKLASYPLIRKLMVQRQQEMACNHSVVMDGRDIGECVLPDADYKFFLTASIAERARRRLEEMKSQGYEQQLEAIIDGIRKRDDDDARREVGALKVLPDSIVIDTSNMTIEQVIEKILSIIRED*
>Syntrophomonas_wolfei_subsp.metylbutyratica_JCM14075T_00809_1-acyl-sn-glycerol-3-phosphate_acyltransferase
MLYSFLRAIFRVLFFLFGLKSEGLHNIAEKGPLILAANHVSYWDAIVVATAIKRPINFMGKAELFDYPLLGYFIKRLNAFPVRRGTADRNAIKKALLLLEEGKILGIFPEGMRNLKGEDLKAQAGVAMIALKSGVPVVPVACIGTNRILPLGWFRPLVVRLGDPIGLEEYQGQKVNSAALEKLSGEIMTKINLLLEK*
>Syntrophomonas_wolfei_subsp.metylbutyratica_JCM14075T_00810_hypothetical_protein
LYVLLARNAGFCAGVRKAVKIAEEAAREPGKWFSLGPLVHNDEVVNYFRQKGIETVDTLERIGEPQTGIIIRSHGTSPELIKQAWELGLEVRDATCVLVKKIHEIVTRLRGEDYDIVVFGDAKHPEVKGILGWCGQRASVVASADEVGDLPVMQKVALLSQTTKDEKEFQKVAGALSNKCRELKVFNTICSATRSRQEAARELCRKVDLMLVIGDLKSSNTRTLSKECQNTGVRTFQIQKASEIKPEW
LDGVSKIGITAGASTPDWIIKEVLDRMTLYEEEKMLDEKQETSMETMQETSVETMQETSLESLEPEVKEETPANEESFAKLEAEMADFTTPNKGDVIKGKVIQVLDDEVMVDVGGKSEGIIPLREMSLQDIDSAKELVEVGDEIEVMVLKWDDDGTILLSKKKVDAKILLDELEESFKEGKVITGTVTGSVKGGLLVDVGIVAFLPASHVEDGYVKNLDEYINRELSFKIIEFNRNKRRGSQVVLSRKEL
VAEEKAKQKKQFWEEIEEGQVRKGRIKRLVDYGAFIDLGGYEGLLHVSEIAHTRIDQPSDVLQEGEEIDVYILALDREKERVSLSRKKLLKSPWEIVMEKFQEGDIIEGTVVRIAPFGAFVEIEAGVDGLVHISQLANHRVEKPEDVVQVNEKIKIKILSIDPEEKRIGLSLKEALNEMENQEVQNYMEKQEENE*
>Syntrophomonas_wolfei_subsp.metylbutyratica_JCM14075T_00811_putative_regulatory_protein,_FmdB_family
MPIFDFECQDCGKSFDKMLSYIDKDKVRCPECDSSNIKQLLSSFNTTRGGTKANKLPSPDSCGGCSNSGCPMRF*
>Syntrophomonas_wolfei_subsp.metylbutyratica_JCM14075T_00812_putative_molybdenum_cofactor_guanylyltransferase
MKSSGIILAGGKSSRMKFNKAFARITSQTAIEIINKFQAFFSETIIISNNPEEYLYLGLPVFTDVYPRQGPVSGIHSALYHASNETIFLLGCDMPFIEMQVVEYMLQKLGEYDSVVPCIDSQIQPTSAVYHRKCLPLFTSCLENQRLKLTRIFYEEINALLIQEKEIVQFGNPREIFFNVNDPGALNIAREMAGRLIPLESSENQNTW*
>Syntrophomonas_wolfei_subsp.metylbutyratica_JCM14075T_00813_Isopentenyl-diphosphate_delta-isomerase
LSLDEIDLSLNFLGKELQYPLMVNALTGGTNQSLAINRALARMALKYSLPMAVGSQSIALESPEAGPSFSIVRDINPNGIILANMNAATRVEEALEAVRMISADALQLHFNVVQELAMAEGDRNFKGIVDNVRQIVHECPVPVIAKEVGFGFSREAAQCLWEAGIEIFDCGGQGGTNFIVIEDQRGGNFAGELDTWGIPTAISLMEILQLPVKQVIASGGIRSALDATKALALGADLVGMAAPLLKAFVS
GGLEALDQSLSGFFFRLKSVFLMCGARNLQEIRRKPLIILGETAEYLRLRGIDPSCWARR*
>Syntrophomonas_wolfei_subsp.metylbutyratica_JCM14075T_00814_putative_membrane_protein
MANFPLGLIALVVVSILIYFGVAHRVLDRLRLSDRAALGLIALIIVGSFIDIPLSTRVTINLGGIAVGLALYVIIGAGTAFEKMRAVLAAVVTALALFLAARFLGAEPEKIFIDPIYIYPLVAGVVGYLAGRSRRGAFFAAVMGVLALDVGQYLYLTRSGIRGVVHVGGAGAFDSLILAGIIAVLLAELIGETLERVQGGPETEDRPQELVKNLQEPEAAHKPLPTEDYEKNGKNEKSSGENGGAEDA*
>Syntrophomonas_wolfei_subsp.metylbutyratica_JCM14075T_00815_stage_II_sporulation_protein_P
MLKRMLILFLMVLIGSCVGGGTFLAAYEEQPLDKCFTLLDENNNVIHQTGSKVYTGDEYISASNGRYKVVEIIGNTARCKYQGDEKMPVLSYDKKSNAWNFNRKEQPVVAGKRPAIAVYHTHSDESYVPSDGKESIEGNGGIYDVGQVMVNKLQKLGFEVQYNKNNHNPHDINAYNRSRRTAASLLKKSPDTIIDVHRDAVPATQYQAQVKGKDVTKVKLVVGRSNPNMKSNLEYAKKIKSVM
DKKTPGLSNGIFIGKGDYNQDLSPRAMLVEVGAHTNKKEDAEKGISLFAEALPTVLGYSNNPVNQAPVAKKPLSGNENQAAGTTILMIVIVVAAAVGGYYLLNRGTNK*
>Syntrophomonas_wolfei_subsp.metylbutyratica_JCM14075T_00816_hypothetical_protein
MERYLVAIVVGIAAGFLDRLIMLRSDYRFYPTYPHGYLTHLALGFIAAGLGAVAIPAIAKPDYVAVTFLALAAQQFREIRSMERETLSKLEESKLIPRGPDYIEGIARVFEARNYLVILTAMASSAVAYWWRWEYAVIAALLCIVFSTKLMGGSYIGEIAQVEKGEIIFDGSILKVNDVIIMNVGLTETRKKIMREGMGIIIKPRDDNARLTLDAPGQRMAIIHDVVSILGSKVDVSETELRPLARKQADKGYLAL
FLVANEPDQEYFMEIVKRVPVIESTRGTALKSFFGRKAAD*
>Syntrophomonas_wolfei_subsp.metylbutyratica_JCM14075T_00817_hypothetical_protein
VNKGFKGGILAIVTMDKDKVTGGGCPVFLAVDQKEQEKLGLFLARILDGIVHDLENGVYFICRH*
>Syntrophomonas_wolfei_subsp.metylbutyratica_JCM14075T_00818_hypothetical_protein
MSEMMKIPYFDKTTNADFGSIRLMGIDEYHNEIYVLGKKNLGKRYGNILMGIAEILGVKEELLTVDCMSRVNWSMKIGGFTSRRMGWVSLGRPVLGKGTEAAFLDLVNLVEITRMKVMKKVQVR*
>Syntrophomonas_wolfei_subsp.metylbutyratica_JCM14075T_00819_hypothetical_protein
MKIVFLGTTGVQHSLIAAHIYLGQLGSEDFRSLEYFCDLSKESSGFPIFIGDDARGNQVYSLGVGKDLQMGQKTIENLLTILGFSSQDLMLRPVSIKAERLLACLGKIPRYLGGTKINLFFSIIILRQELAAIKENVEQFCN*
>Syntrophomonas_wolfei_subsp.metylbutyratica_JCM14075T_00820_division_inhibitor_protein
MANNYMKRKDRIILTAVEIIDQLGVQGLTIREIASRQEISEAAIYRHFANKTEILLGTIDYFSSYDALLFRTIENQNMKAWEGITFLFASYMEYYENYPAISAVNFTQEAFQLEPEARQRMENVIDDRLRFINHLVIKGQKEGEFTRDICSESMVDVIFGTIQFIILRWRAKNYNFALKEHVLSTLSILIEAFSTVKLENRFAWGGVFNQEVSASAFDSVLTRPGLFLEKGKDQVNEKSEG*
>Syntrophomonas_wolfei_subsp.metylbutyratica_JCM14075T_00821_Chemotaxis_protein_CheY
LKSVLIVDDAMFMRYTLRKILEKNGFLVVGEAENGLLGAQKYGELLPDIVTMDITMPELNGIGAVQLIKQINPQANIVMISAMGQQHLVREAILAGASNFLVKPFSEERVVEVLNSI*
>Syntrophomonas_wolfei_subsp.metylbutyratica_JCM14075T_00822_Chemotaxis_protein_CheA
MDKSLSKEPMLEMFIFENLQLLEQLEQTILAHEKERCLENAVINEIFRIMHTIKGAAAMMMYDGISSLAHAIEDLFFYIREFHPDEMDYSKICDIVLEGIDFIKNEIGKLEQDQEADGDSSPFVENIGQYLLQIKEASRNSKLINEAAARPAGHRKKFYISTSKSPPTESIKRYHALIYFTPGCEMENIRAFNLVHKLKDQAEQIDYIPADIIENNDSVEFIRENGFAVIFSTHLQQEAINELFMKEAFIDSLDLRV
MEDEEWEIEQLRARNQIVLEEEPSVRNNEVREIGANLHSGSTRQSLISVNVDKLDRLMDMVGELVISETMVAFNPDLRTAAKLDNFHKATRQHRKIINELQDIVMSIRMVPLSMTFQKMNRLVRDMSKKLKKNVELVIIGEETEVDKNIIEHLSDPIMHLIRNAIDHGVESPEKRASLGKSPAARVYLEAKNEGGDVYIIIRDDGAGLNKEKILERARARGLIQKPDSNLSDGEIYSTILLPGFSTCEEISEF
SGRGVGMDVVLKNIEEVGGTVLVDSTPGQGTTISLKIPLTLAIINGMRVKVGNSIYILPITSIKESFKAKPDNIIVDPDGNEMLMVRGECYPVLRLHQKFQLKSALKKIAEGIIVIVQNDSGSICLFVDALLGEQQVVVKALPKFIGKIAGISACTLLGDGRACLIIDVANLINPSKG*
>Syntrophomonas_wolfei_subsp.metylbutyratica_JCM14075T_00823_Chemotaxis_protein_CheW
VSEILDEAVLQEEDTQKGKFLTFLVGKEEYGIEIKYVTEIIGGIQAITEIPDVPEYVKGIINLRGKIIPVMDVNLRFNKEARDYNDRTCIIVIDIDELSVGLIVDNVAEVISIADADIVPPPDVRSGFHNRYIKGIGKIGSEVKLILDCQRLLSTEELENLDYIN*
>Syntrophomonas_wolfei_subsp.metylbutyratica_JCM14075T_00824_Dipeptide_chemoreceptor_protein
MAGKKSNMDSSEEKNKEEVIGKGSVQKQDMGRALPERTTGELQEEINLLVEAIKAGRLDVRANVGKASGLEKECLLGINQMLDAVIGPLNVAADYVERISRGDIPPIITDSYNGDFNTIKNNLNTCIEAVNLLIADANLLARAAVEGRLDTRADAARHHGDFRAIVEGVNHTLDAVIGPLNVAADYVERISRGDIPPIITDSYNGDFNTIKNNLNTCIEAVNLLIADANLLARAAVEGRLDTRADAAR
HHGDFRAIVEGVNHTLDAVIGPLNVAADYVERISRGDIPPMITDSYNGDFNAIKNNLNTCIEAVNLLIADANLLARAAVEGRLDTRADAARHHGDFRAIVEGVNHTLDAVIGPMKEVSAVMREMEQGHLGVNVRGDYKGDFKLVKDTLNSTIEALKNYVNEISIVLTQMARNNLDVEISQDFRGDFSAIKDSLNYIITSWNETMSGINMASDQVANGSAQVARSSQFLAQGAAEQSSAIEQLTASIT
QIAAQTNQNAAYANQANDLALTARDNAIKGNEEMKNMLEAMEAINESSNNISKIIKVIDEIAFQTNILALNAAVEAARAGQHGKGFAVVAEEVRNLAARSASAARETTDMIEGSIKKTEAGTRIANETAEALGKIVEGVTRMAALVGDITVASNDQATGISQIDKGVEQISQVVQSNAATAEESASAAEELSSQAVLMKDEVGKFTLKKRNERAVQLSDLSPEMLALVKELLQKQKIMQAVDSRETRAI
ETASRGNSRLQISLSDNEFDKY*
>Syntrophomonas_wolfei_subsp.metylbutyratica_JCM14075T_00825_Chemotaxis_protein_methyltransferase
MINLSQQEFHWLADYVYNYCGIDLKQKKHLIESRLSNHLLETGFSSFAGYYKHLLQDASGESITAMLDRITTNHTYFMRESQHFDFFRDRVLPELLSKVKNRDLRIWSAGCSSGEEAYTLAMVISDFFSSKQAAWDTRILATDISARALQKAKEAVYTEEQIEALPAGWKLWYFQEINRNSFRIREKIKRQVIFRRFNLMEIPFPFKQGFQVIFCRNVLIYFDSSTRSKLIQRFYDSIVDGGYFFIGQAESINW
DNNRFSYVMPAVYRKE*
>Syntrophomonas_wolfei_subsp.metylbutyratica_JCM14075T_00826_Chemotaxis_response_regulator_protein-glutamate_methylesterase
LRLRRIKTLIVDDSLLFRETLARGISRDPAIEVVATAADAFMARDMIIKHKPDVMTLDVEMPRMSGIEFLRRLMPQYPLPVVMISALSESVFDALNAGAIDFVAKPDMNPNRDLDAFFSELILKIKIASVARLGRRAEVSKLLPTGKSQTDQGWVIAIGASTGGPEAIYQIVKNFNQDTPGAVIVQHMPAGFTRMYAERLNDTCLVDVKEAQNYDRVKMGQVLIAPGEYQMRLKQKAGDYMVECHP
GEKVNGHCPAVDVLFDSVAQSAGARAVGVILTGMGSDGARGLLAMRKAGARTIGQDEASCVVYGMPKVAYELGAVEKQYSLNYIGQALYKLINQL*
>Syntrophomonas_wolfei_subsp.metylbutyratica_JCM14075T_00827_Sensory/regulatory_protein_RpfC
MLEADKREYAKTIARIQEERDNLQIILESIGDGIITTDMDGYISMMNPVAEKLTGWSREEALGKHLDEVFRIIDNTSKQPLENPFDNVFTRGSSQGLLRNSALLDRDGKTRFASACASPIIDKNQKMAGMVVVFRDIDRIKKNEDEIRCLSWVLQLSSNAIIIFDTQGIIQFINSRFTQLTGYEACEVLNRNLSLINNLPLDVEKIFARLRAGEEWHDEVFFHKRQLEPCWWSIYCTAVRDEEDKITSYCSIIQ
DISEHKLAEENLKKAKEAAEVANRAKAEFLANMSHEIRTPLNAIIGMTSLSLLSNPEPDQKENLEIIKMAGDTLLSIIDNILDFSKIEAGKLTIEKIDFNLHELVEKNITIHKARAQEKGLLLLYCIDPAIPVQVNGDPYRLQQVFNNLIGNAIKFTDQGEVEIRLNLLDILEDRIVLQASVADTGRGITAGQQEKMFESFYQGDSSISRTYGGTGLGLNISKKIVEMMQGKIWLESDIESGSTFYFTVELAIPEPS
KLETESRQLINQKADAEKIPFLSILVVEDNEVNQMVLSNLLRAQGHQVEVAANGVEALELLDKNNYDLVFMDILMPVMDGLETTRRIREQEEITGFHIPIVAITAHAIYGDREKFLAAGMDDYLAKPVQMEELNQAISKMIKKARSTTTTNHMDIEPLSPKDLFFKVDSSFLEQVEKAILKIDLALLSLDLQQVEKQAHEIKELAYQMDEITIKNLAFKIELGARKGDLDQCWSLFRKLQEIYAYLSSLG
S*
>Syntrophomonas_wolfei_subsp.metylbutyratica_JCM14075T_00828_Sensory/regulatory_protein_RpfC
MKVLIAEDDLASRKFLYKFLSRYGNCDITVDGIETIDAFLLAHEEGQPYDLICLDIMMPKLDGIKTLKTIRDLEKQRGLQDSELVKIIMTTALNEPETVFNCFDSGCEAYAAKPIDTDKLLEAIKRLGLLD*
>Syntrophomonas_wolfei_subsp.metylbutyratica_JCM14075T_00829_putative_FeS-containing_Cyanobacterial-specific_oxidoreductase
MDNGFQDSAAEEKILTAPIKGIMENSIAGEMDIEPGDILYSINGHEINDILDYEFYSQEDNLLVEIEKANSGELWVLEIEKEYDEELGLLFDGLIFDKMKLCQNRCLFCFVDQLPRKMRKTLYVKDDDYRYSFLLGNFISLTNLSEKDWHKILSMRLSPLYVSIHCMQPELRARLFNNPRAANIKSELKRLQEAGIEVHTQIVLCPGINDAEVLQHSINELAQFFPTVASVGVVPVGLSGHRQGLPPLRGFT
VDEAIKTIELVDSFQDEFRPRLRRGFVYAADEFYLKAGRNFPPALYYDDYCQVENGIGLARIFVDDFAEIEASLPLVVERQEVYLITGVSAGPVLEKVVSRLNRIEGLELKIIRVTNRFFGGGVTVTGLLTGADIIATLSRDFAGKKLIIPEIVFKEGQDIMLDDTSREELEEKTGAEIITVDGSARSLVRTVLSLTEV*
>Syntrophomonas_wolfei_subsp.metylbutyratica_JCM14075T_00830_GTP-binding_protein_EngA
MKPVVAIVGRPNVGKSTLFNRLAGARKAIVEDIPGVTRDRLYDSSDWNGRDFIIIDTGGIRFNEGDIFAREIKLQAELAIEEADVIILVLDSREGITAEDEQVANLLRKSNKPVVLAANKVEEFKKQLDYYEFYKLGLGDPIPVSAMHGYNTNELLDAVISYFKPAREYEEEGDAIKIAIVGRPNVGKSSLVNALLGEERVIVSDVPGTTRDAIDTPFQYKGQNYLLIDTAGMRRKSRIKETTERYSVIRALKAI
ERADVVLIMLDATEGVAEQDKKIAGYVHEQSRANIIVVNKWDLVEKDGQTMNRFDKDIREELKFLSYSPIMYVSALTKKRIFQVLEIVNFVAEQHHRRVNTAELNQVINEAMMLNPLPGGGGKRIKILYSTQVRVAPPTFVFFSNKPEMVHFSYLRYLENALRKNFGFEGTPIRLLLRQNTEHDN*
>Syntrophomonas_wolfei_subsp.metylbutyratica_JCM14075T_00831_G3P_acyltransferase
LQEAALLFLCYLLGSIPFSYLFTRMLTRQDIRSKGSGNVGATNVFRTSGPGVALLAFLGDLLKGLLAAWIGQSTGSQFLLIFCAVLAVIGHIYPVFLGFKGGKGVTTSAGIILFLMPDVTGILLLIFLAIVYFTRYVSLGSISVACLLPLVSLAMGKPWSYIVIGILVAVLVVYHHRENIDRLRNGSEAKITDKA*
>Syntrophomonas_wolfei_subsp.metylbutyratica_JCM14075T_00832_Glycerol-3-phosphate_dehydrogenase_[NAD(P)+]
MPKICILGSGSWGTAQALLLSSKGFQVILWGRVEDGVDSLQQERENRRFLPGIRLSDSIVATSDLAQALKGADMVVMAVPSQSLREVLEKARPYLEKDSCLVNTAKGLEISSGMRMSQVVEDVLGSQSRERFSVLSGPSHAEEVARNIPTAITAASYHKEIAFLVQDLYMTPFFRVYTNPDVAGVELGGALKNIIALGTGIASGLGYGDNTQAALLTRGLHEIIRMGEAMGGEARTFSGLSGIGDLVVT
CSSRHSRNRQAGILVGQGYSLEETLMQIGMVVEGAHTIRVVHRLATQLKIDMPICTACYNVLYANRKARDEVDDLMRRQKKHEIEEIVKRKKEW*
>Syntrophomonas_wolfei_subsp.metylbutyratica_JCM14075T_00833_Stage_IV_sporulation_protein_A
VEGNNILKDIAERTGGDIYLGVVGPVRTGKSTFVKRFMELMILPNIQDIFERDRANDELPQSGSGKTITTTEPKFVPNEAVEITTQNGISLKVRLIDCVGYSVDGALGYEEDEEPRMVRTPWFDYEVPFEEAAEIGTRKVITDHSTIGIVITTDGSISDIPRDNYVSAEERVINELKELNKPFIVLLNSTRPYEKETLNLAEELSEKYEVSIIPVDAARMSTEQVYGILEEALYEFPVQEVNIKLPQWVDELEEDF
WLRQNMETSIREILNAIRKVRDIDRAVEQLSDMENVSYVSLEEMNLGTGTARIEVNVPEELFYQALSEVSGFGVEGTHDIMRIMKDLSVAKREFDKIASALDEVKESGYGVVTPRLEEMFLEEPELIRQGSRFGVKLKAKAPSLHIIRADITTEITPIIGTEKQCEELVRYMLDEFEEDPGKIWESNIFGKSLHDLVREGIQNKLHRMPENAQHKLQDTLQRIVNEGSGGLICIII*
>Syntrophomonas_wolfei_subsp.metylbutyratica_JCM14075T_00834_Cyclic_di-GMP_phosphodiesterase_response_regulator_RpfG
MPKDRPAMEFTNNSKEIKLESNNGGLYILENINGLILCCNSSGSLTFANKKAREMMGIKVEEVPELNIFDLVTPQEHNLLEKKVFTALQEHVSLSFESCILDAKKIPRLHRLNISVPVDGKKDEDVVLILLEDLSERRQMEEAFVESEHRLVHIIDFLPDATLAIDCNGRVITWNRAAEEMTGVRAREIIGKGNYEYALPFYGYRRPLLLDQVLSTSTEWEQSYTLLEKKARSLRAENFCPAVGESGAYLRLT
AAPLIDADGKRIGAIESIQNVTRKKRAEENTAIQRDLGISMIAINGMEEALSICLNSALKISGMHAGAIFYVDNGGDKTLSVHRGLSPGFVEQVKTCAFTNNSLYAQALYSSADKLTAYFGDIVLQEGLKSMAIIPIYHKDKMLASLKLSSRISVVPEHSRQAIENVANYIGSNIARLQVEEELRESEERYRQLVEVSPDAIAVHINGRIVFINHAGARLLGAEKVEDILGRIIFEHIPESRQIYIDLMEPLAANGV
FPLTEMKVILINGKIIYTEVTGAALVFEGTPAVQIIVRDITVRKKAQYDLSREVYKLRVLYDLAVAMSSSQSMDETLKLTADKSRELFAADCIFIFLIDESKNEAIPHTISGNKLTDWLEVRIPYGKGLIGMVAASESGYILEDYFSSTLFTHIYDELVLQEELLCGLAAPIITGNSRLGVLVVGHRKRTVLTEANLDTLTLIANLLAVEITRDRAEESLQKSLEHLKLTVFECTVNALSITAEKKDPYTAGHQRRV
ASLACAIAQEMKLPQYYIDNVRTASILHDIGKLQIPTDILTKPGGLTEIERLLVKTHPQAGYEIVKTIPFAQAISTTLLQHHERMDGSGYPQGLSGEEILLEARILAVADVVEAMASHRPYGLH*
>Syntrophomonas_wolfei_subsp.metylbutyratica_JCM14075T_00835_Cyclic_di-GMP_phosphodiesterase_response_regulator_RpfG
MINRALINNAKKFLNILASIEDGYYEVDKAGNLVFFNQSLQDMLGYSKEELLGMNYKHYMTNEDAKKVFKTYNLVFRTGEAAKGFDWQLISKSGKKLYVETSVSPIFSADGEITGFLGIIRDISERKRAEQQIRESERRLAGIISFLPDATFVIDISGKVIIWNQAAEEMTGIKAEDIIGKDDYEYSIPFYGYKKPMLVDIVMEESTYAHNLIADVEKKGHTLIKEVFFPAVGDSGLYCWAATAPLFDSGGNQ
TGAIISLRDISRRKQTENALQESLKKAQTILRSTVNALAITTEKRDLYTAGHQRRVAQLATAIAREMQFPDNITRGIYMAATLHDIGKMQISSDILSKPGELNDIETLLVRTHTQVGYEIVKTIPFELPVADMVIQHHERMDGSGYPQGLAGSQIMLEARILAVADVVEAMSSHRPYRPALGINLAMEEIRRHRGILYDPDVVDACLRLFARGQIDFL*
>Syntrophomonas_wolfei_subsp.metylbutyratica_JCM14075T_00836_Homoserine_dehydrogenase
MIGVALLGCGTVGSGVIKLLQKNSEIIDKRIGESIVIKKVLERDTEKCRLLGIKDEAITQDIQEILDDQEIAIVVELIGGIEPAFSYIMAALARGKHVVTANKDLIAIKGKDLFDQAKEKRVDFYFEASVAGGIPIVYPMKQSLAANQIQEVIGILNGTTNYILSKMSREGRDFNEVLAEAQQLGYAESDPSADVDGLDAARKIAILSSIAFNSRVTLDDVYVEGIRSITADDIQYAKELGYVIKLLAIAKENPQQKI
EVRVHPAFLPKTHPMAGVNDVFNAVFLRGDAVGEIMHYGRGAGQMPTASAVFGDIIEIARNILYHSNARIGCSCYDNKAIMDIKELEGEYYIRMIVEDRPGVLAGIAAVFGNNNVSIATVLQKTSKQCWAELILITHRVREMHLRDSLTLLKGLSIVGEIKNVIRLEGTDREAD*
>Syntrophomonas_wolfei_subsp.metylbutyratica_JCM14075T_00837_Homoserine_kinase
MFTVKVPATSANLGPGFDTLGLALQLYLEIKVKIIPPSGGIRFFVDGEEYPEEIFGDNLLYQAMKIVFAEAHVVEVPGLELTINSSIPPGKGLGSSAAAIVAGLYAANEVLEKRFAQEDLISWAVEMEGHADNVVPAVAGGLTTAMLYRKKVYYQQFSFPPELKLVVAVPDIPISTDESRQLLPKKINFKDMVNNLQRASYLLASLIKHDFRHLPAAMDDAIFQPRRKQLIPGFDRVFSQALGNGALGVAL
SGSGSSIIAFCQQEEKRVAQAMQDAFAVSGVQSQLMILAADPDGVKVLR*
>Syntrophomonas_wolfei_subsp.metylbutyratica_JCM14075T_00838_Aspartokinase
LSLIVAKFGGSSVASIDKIKHIAGRIKEIRDKGDDIVVVVSAMGDTTDELIELASLTGHDLCKREMDMLLATGEQQSAALLALTLNNYACPSVSLTGWQAGINSDDSHSKARITGIKTDRIKNEIKRGNAVIIAGFQGLSPFGDITTLGRGGSDTTAVALAASLDALRCMIFTDVEGVFTADPRIVKEAQKLPQVSYEEMLEMASLGAGVLQPRAAEFAMLYNVDVEILSSFSRNPGTIITEVVNMENHRA
VSGIAHDLNCARFALFDVPDQPGIAKTLFKTLAAESINVDIIIQSAMRDGRNDIAFTIEENDLSRAVPVVKELVESICASGMAYGKDVAKVSIVGAGMQSNIGVAAAMFEALADEGINIHMISTSEIRVSCVIDECHIKTAVKALHRKFELDKIE*
>Syntrophomonas_wolfei_subsp.metylbutyratica_JCM14075T_00839_Transposase
[illegible]
>Syntrophomonas_wolfei_subsp.metylbutyratica_JCM14075T_00840_Transposase
MLKDILDYDSIYLACGATDLIRRSVDGLAIIYEVKQGFFKMDPFGGIYFILFCNRSRNRLKALSWDRNGFVLYYKRLDGAGARFKWPNEPIEVRNITVKQLRLLMEGMSIDPPRGFGDITARAFY*
>Syntrophomonas_wolfei_subsp.metylbutyratica_JCM14075T_00841_hypothetical_protein
VTYEEQKALVTECRASGMKAKTWCEAKGIEYRRYVNWATRVNRKAQDKSQQWADITMAIAADKIANDEIKLKCGKWTICVATGFSPTLLADILKVVDTVC*
>Syntrophomonas_wolfei_subsp.metylbutyratica_JCM14075T_00842_PD-(D/E)XK_nuclease_family_transposase
[illegible]
>Syntrophomonas_wolfei_subsp.metylbutyratica_JCM14075T_00843_hypothetical_protein
[illegible]
>Syntrophomonas_wolfei_subsp.metylbutyratica_JCM14075T_00844_Threonine_synthase
[illegible]
>Syntrophomonas_wolfei_subsp.metylbutyratica_JCM14075T_00845_cofactor-independent_phosphoglycerate_mutase
[illegible]
>Syntrophomonas_wolfei_subsp.metylbutyratica_JCM14075T_00846_Glucosamine_ammonia-lyase
[illegible]

>Syntrophomonas_wolfei_subsp.metylbutyratica_JCM14075T_00852_HTH-type_transcriptional_repressor_NsrR
[illegible]
>Syntrophomonas_wolfei_subsp.metylbutyratica_JCM14075T_00853_Hydroxylamine_reductase
[illegible]
ILLSLLYLGIKNIRLGPSLPAFVSPGVLQFLVDNFGIKPISSAEEDLRAILAG*
>Syntrophomonas_wolfei_subsp.metylbutyratica_JCM14075T_00854_light-independent_protochlorophyllide_reductase_subunit_B
[illegible]
>Syntrophomonas_wolfei_subsp.metylbutyratica_JCM14075T_00855_putative_ABC_transporter_ATP-binding_protein_YbhF
[illegible]
QVAEALHKIPGVNRIEESGDRLYINATKQEELRPRIMQEALAHDARVLSFGLQESSLEDILLRLVKSDVSDLPERRSGFISSFFRLNRKSR*
>Syntrophomonas_wolfei_subsp.metylbutyratica_JCM14075T_00856_gliding_motility-associated_ABC_transporter_permease_protein_GldF
[illegible]
RQNVTFKN*
>Syntrophomonas_wolfei_subsp.metylbutyratica_JCM14075T_00857_metallo-beta-lactamase/flavodoxin_domain-containing_protein
[illegible]
KGFEYIKNCLNEGVGEKDLADRLFKRYIKGGLAYYPEAMMLGAMQLLIASVKLKM*
>Syntrophomonas_wolfei_subsp.metylbutyratica_JCM14075T_00858_Sensor_histidine_kinase_DpiB
[illegible]
>Syntrophomonas_wolfei_subsp.metylbutyratica_JCM14075T_00859_putative_accessory_gene_regulator_protein
[illegible]
>Syntrophomonas_wolfei_subsp.metylbutyratica_JCM14075T_00860_cyclic_lactone_autoinducer_peptide
[illegible]
>Syntrophomonas_wolfei_subsp.metylbutyratica_JCM14075T_00861_Lipoprotein_signal_peptidase
[illegible]
>Syntrophomonas_wolfei_subsp.metylbutyratica_JCM14075T_00862_Ribosomal_large_subunit_pseudouridine_synthase_D
[illegible]
>Syntrophomonas_wolfei_subsp.metylbutyratica_JCM14075T_00863_Bifunctional_protein_PyrR
[illegible]
>Syntrophomonas_wolfei_subsp.metylbutyratica_JCM14075T_00865_Aspartate_carbamoyltransferase
[illegible]
>Syntrophomonas_wolfei_subsp.metylbutyratica_JCM14075T_00866_Dihydroorotase
[illegible]
>Syntrophomonas_wolfei_subsp.metylbutyratica_JCM14075T_00867_Carbamoyl-phosphate_synthase_small_chain
[illegible]
>Syntrophomonas_wolfei_subsp.metylbutyratica_JCM14075T_00868_Carbamoyl-phosphate_synthase_large_chain
[illegible]
>Syntrophomonas_wolfei_subsp.metylbutyratica_JCM14075T_00869_hypothetical_protein
[illegible]
>Syntrophomonas_wolfei_subsp.metylbutyratica_JCM14075T_00870_Dihydroorotate_oxidase_B,_electron_transfer_subunit
[illegible]
>Syntrophomonas_wolfei_subsp.metylbutyratica_JCM14075T_00871_Dihydroorotate_dehydrogenase_B_(NAD(+)),_catalytic_subunit
[illegible]
>Syntrophomonas_wolfei_subsp.metylbutyratica_JCM14075T_00872_Orotidine_5'-phosphate_decarboxylase
[illegible]
>Syntrophomonas_wolfei_subsp.metylbutyratica_JCM14075T_00873_Orotate_phosphoribosyltransferase
[illegible]
>Syntrophomonas_wolfei_subsp.metylbutyratica_JCM14075T_00874_hypothetical_protein
[illegible]
>Syntrophomonas_wolfei_subsp.metylbutyratica_JCM14075T_00875_hypothetical_protein
[illegible]
>Syntrophomonas_wolfei_subsp.metylbutyratica_JCM14075T_00876_hypothetical_protein
[illegible]
>Syntrophomonas_wolfei_subsp.metylbutyratica_JCM14075T_00877_4-hydroxy-tetrahydrodipicolinate_reductase
[illegible]
>Syntrophomonas_wolfei_subsp.metylbutyratica_JCM14075T_00878_Dipicolinate_synthase_subunit_A
[illegible]
>Syntrophomonas_wolfei_subsp.metylbutyratica_JCM14075T_00879_Dipicolinate_synthase_subunit_B
[illegible]
>Syntrophomonas_wolfei_subsp.metylbutyratica_JCM14075T_00880_Aspartate-semialdehyde_dehydrogenase
[illegible]
>Syntrophomonas_wolfei_subsp.metylbutyratica_JCM14075T_00881_4-hydroxy-tetrahydrodipicolinate_synthase
[illegible]
>Syntrophomonas_wolfei_subsp.metylbutyratica_JCM14075T_00882_Ribonuclease_J_1
[illegible]
>Syntrophomonas_wolfei_subsp.metylbutyratica_JCM14075T_00883_Translocation-enhancing_protein_TepA
[illegible]
>Syntrophomonas_wolfei_subsp.metylbutyratica_JCM14075T_00884_hypothetical_protein
[illegible]
>Syntrophomonas_wolfei_subsp.metylbutyratica_JCM14075T_00885_Undecaprenyl-diphosphatase
[illegible]
>Syntrophomonas_wolfei_subsp.metylbutyratica_JCM14075T_00886_Stage_III_sporulation_protein_E
[illegible]
>Syntrophomonas_wolfei_subsp.metylbutyratica_JCM14075T_00887_putative_kinase
[illegible]
>Syntrophomonas_wolfei_subsp.metylbutyratica_JCM14075T_00888_cytoskeletal_protein_RodZ
[illegible]
>Syntrophomonas_wolfei_subsp.metylbutyratica_JCM14075T_00889_Ribosomal_protein_S12_methylthiotransferase_RimO
[illegible]
>Syntrophomonas_wolfei_subsp.metylbutyratica_JCM14075T_00890_CDP-diacylglycerol--glycerol-3-phosphate_3-phosphatidyltransferase
[illegible]
>Syntrophomonas_wolfei_subsp.metylbutyratica_JCM14075T_00891_Putative_binding_protein_precursor
[illegible]
>Syntrophomonas_wolfei_subsp.metylbutyratica_JCM14075T_00892_Sulfate_transport_system_permease_protein_CysW
[illegible]
>Syntrophomonas_wolfei_subsp.metylbutyratica_JCM14075T_00893_Sulfate/thiosulfate_import_ATP-binding_protein_CysA
[illegible]
>Syntrophomonas_wolfei_subsp.metylbutyratica_JCM14075T_00894_Cellulosome-anchoring_protein_precursor
[illegible]
>Syntrophomonas_wolfei_subsp.metylbutyratica_JCM14075T_00895_hypothetical_protein
[illegible]
>Syntrophomonas_wolfei_subsp.metylbutyratica_JCM14075T_00896_ATP-dependent_zinc_metalloprotease_FtsH
[illegible]
>Syntrophomonas_wolfei_subsp.metylbutyratica_JCM14075T_00897_Nicotinamide-nucleotide_amidohydrolase_PncC
[illegible]
>Syntrophomonas_wolfei_subsp.metylbutyratica_JCM14075T_00898_2'-5'_RNA_ligase
[illegible]
>Syntrophomonas_wolfei_subsp.metylbutyratica_JCM14075T_00899_hypothetical_protein
[illegible]
>Syntrophomonas_wolfei_subsp.metylbutyratica_JCM14075T_00900_hypothetical_protein
[illegible]
>Syntrophomonas_wolfei_subsp.metylbutyratica_JCM14075T_00901_Death_on_curing_protein
[illegible]
>Syntrophomonas_wolfei_subsp.metylbutyratica_JCM14075T_00902_hypothetical_protein
[illegible]
>Syntrophomonas_wolfei_subsp.metylbutyratica_JCM14075T_00903_Anaerobic_ribonucleoside-triphosphate_reductase
[illegible]
>Syntrophomonas_wolfei_subsp.metylbutyratica_JCM14075T_00904_tRNA-modifying_enzyme
[illegible]
>Syntrophomonas_wolfei_subsp.metylbutyratica_JCM14075T_00905_hopanoid_biosynthesis_associated_protein_HpnK
[illegible]
>Syntrophomonas_wolfei_subsp.metylbutyratica_JCM14075T_00906_hypothetical_protein
[illegible]
>Syntrophomonas_wolfei_subsp.metylbutyratica_JCM14075T_00907_hypothetical_protein
[illegible]
>Syntrophomonas_wolfei_subsp.metylbutyratica_JCM14075T_00909_Fic/DOC_family_protein
[illegible]
>Syntrophomonas_wolfei_subsp.metylbutyratica_JCM14075T_00910_putative_choloylglycine_hydrolase
[illegible]
>Syntrophomonas_wolfei_subsp.metylbutyratica_JCM14075T_00911_Ribonuclease
[illegible]
>Syntrophomonas_wolfei_subsp.metylbutyratica_JCM14075T_00912_hypothetical_protein
[illegible]
>Syntrophomonas_wolfei_subsp.metylbutyratica_JCM14075T_00913_hypothetical_protein
[illegible]
>Syntrophomonas_wolfei_subsp.metylbutyratica_JCM14075T_00914_HTH-type_transcriptional_repressor_YtrA
[illegible]
>Syntrophomonas_wolfei_subsp.metylbutyratica_JCM14075T_00915_ABC_transporter_ATP-binding_protein_YtrB
[illegible]
>Syntrophomonas_wolfei_subsp.metylbutyratica_JCM14075T_00916_ABC-type_transport_system_involved_in_multi-copper_enzyme_maturation,_permease_component
[illegible]
>Syntrophomonas_wolfei_subsp.metylbutyratica_JCM14075T_00917_hypothetical_protein
[illegible]
>Syntrophomonas_wolfei_subsp.metylbutyratica_JCM14075T_00918_putative_sulfoacetate_transporter_SauU
[illegible]
>Syntrophomonas_wolfei_subsp.metylbutyratica_JCM14075T_00919_hypothetical_protein
[illegible]
>Syntrophomonas_wolfei_subsp.metylbutyratica_JCM14075T_00921_Pyruvate_synthase_subunit_PorC
[illegible]
>Syntrophomonas_wolfei_subsp.metylbutyratica_JCM14075T_00922_Pyruvate-ferredoxin_oxidoreductase_subunit_delta
[illegible]
>Syntrophomonas_wolfei_subsp.metylbutyratica_JCM14075T_00923_Pyruvate_synthase_subunit_PorA
[illegible]
>Syntrophomonas_wolfei_subsp.metylbutyratica_JCM14075T_00924_Pyruvate_synthase_subunit_PorB
[illegible]
>Syntrophomonas_wolfei_subsp.metylbutyratica_JCM14075T_00925_hypothetical_protein
[illegible]
>Syntrophomonas_wolfei_subsp.metylbutyratica_JCM14075T_00926_hypothetical_protein
[illegible]
>Syntrophomonas_wolfei_subsp.metylbutyratica_JCM14075T_00927_HTH-type_transcriptional_repressor_Bm3R1
[illegible]
>Syntrophomonas_wolfei_subsp.metylbutyratica_JCM14075T_00928_hypothetical_protein
[illegible]
>Syntrophomonas_wolfei_subsp.metylbutyratica_JCM14075T_00929_Monoterpene_epsilon-lactone_hydrolase
[illegible]
>Syntrophomonas_wolfei_subsp.metylbutyratica_JCM14075T_00930_hypothetical_protein
[illegible]
>Syntrophomonas_wolfei_subsp.metylbutyratica_JCM14075T_00931_hypothetical_protein

MDKYNSEGYPDPTAYEALTAVAKSEAPMKTYRPLVYLASPFAGDTERNIERARGYCRFAVSKGCIPIAPHLHYPQFMDDDDREQRELGMFFALVLLGKCDELWVFGKPSDGMSREIAKAKKRGIPIKYYNHKCEVLEYGT*
>Syntrophomonas_wolfei_subsp.metylbutyratica_JCM14075T_00932_hypothetical_protein
MNNEKKQTIKTALKKVGKVVWSALPWVLMWIGLTCFFSLLFISADKSAERSKQLAALEAENAMYESVNARLDEEIDWLRSLVEQYYQREVPDGQVQ*
>Syntrophomonas_wolfei_subsp.metylbutyratica_JCM14075T_00933_putative_phage-encoded_protein
MQTEANERNAKMRDAELDEELAGMGSIAHFIKKIKCRYAKQPLLSVGSRGVVASRKKLFRRFNMNGLQVFSYEGNEVRTVQKGSEILWVLKDVCGILGIEKYRDAAARLDDDEREPVLVDTLGGRQEMIAVTESGLYSIILLSRKPEAKKFKRWVTHEVLPTIRRHGAYVTPAKLEELMNNPDSWIKVLTALKEERAAKERLQLEATENKPKVIFADAVSVSDGTILIGELAKILKGNGIEIGQNRLFEK
LRQDGYLIKRKGTDYNAPTQRAMELGLFRVKETAITHSDGHVTISKTTKVTGKGQQFFINLFLGKGVNNNE*
>Syntrophomonas_wolfei_subsp.metylbutyratica_JCM14075T_00934_hypothetical_protein
MKKQANQSQNKSFKPTPIPLRTKFGEPMAYYTLSDDGKTHTEVSRFVCLARTEEPENPFQQRCYVDEESGLVVRLPCNQIGEDLARDNMRSIWREQKYQERKFQCVWKGTSKCDQKCETCKSRTSRTVELDKPLGNDSDGSDTFYEPSDPTDITEILEQKAVLDTLHAALATLTQGDLELIKDRVILEQTVRELAPKYGFKSSRSITVHVRRILDILRNDEALKKFFE*
>Syntrophomonas_wolfei_subsp.metylbutyratica_JCM14075T_00935_hypothetical_protein
MAKIVKQRLCGGTFFTLFLRARKPLRGANEYYAGNPEPYSEPIALFALSKVIVPDWQNIFVFADSTVSGNTSQYKTCKNEGGSIYPFGDDAALTAFNARVKENYFSALAEMCKVIELLVDTGSMRKDERLVKELLALIEVDDSINDSQPFFALESGDAITKSDILKATSVCLQSFLLGVWHFAVTRSEKNTFGKETINLWCPSTGGGKREYKGNLANLITRNITLNYFEGFGESVDAEIVDEPFVDEPMIN
DEIPNEEQSTEKEPAASNLTQQVINNNPTFFNFNVSGNNNNFYNHVDTVNIKNGGQKDE*
>Syntrophomonas_wolfei_subsp.metylbutyratica_JCM14075T_00936_hypothetical_protein
MSEKPKLISTAVLPSTGASANINLPGDGNTLIAHANAVNMKSVYIPVVQTSGVALPTRRTVNEDYYSLFVIGDELFTGGHFMVPKECALTESIAPELKAKYAKLDMGAITAIKTFPAIFASRNHQYCRAGENQDAYLGFVDEVVVQDNGIKVCFHLLYAISQRRLNELACELSIDSASAFNEFDRTHWTIKQINLIEKLRKADVINTLF*
>Syntrophomonas_wolfei_subsp.metylbutyratica_JCM14075T_00937_DNA_binding_domain,_excisionase_family
MSNEIDKNITEKWVNLEDIAEYLSVSKDTVRTWMREGKLPVNKAGKRYKFKISEVDEWVRKGKIKE*
>Syntrophomonas_wolfei_subsp.metylbutyratica_JCM14075T_00938_ATP-dependent_helicase_HepA
MQMPEMLDNVNKTVKDDLTVSINKGDKLSIAAACFSIYAYQALKKQLDGIDELRFIFTSPTFLHEKAPKEKREFYIPRLNRERSLYGTEFEVKLRNELTQKAIARECSEWIRKKVKFRSNLTGGNISGFMNVEGNECTTYMPLHGFTTSDIGCERGNNIINPVNKMFAPLSGEYIRMFNQIWNDKSLMQDVTEQVIDGITAAYNENAPEFIYFVAIYNIFNEFLEDISEDVLPNEATGFKESKIWNMLYAF
QKDAVLAIINKLESYNGCILADSVGLGKTFTALAVVKYYENRNRSVLVLCPKKLSDNWNTYKDNYVNNPIAADRLRYDVLYHTDLSRDRGKSNGLDLDRLNWGNYDLVVIDESHNFRNGGEVYGEDRRENRYLRLMNRVIRTGVKTKVLMLSATPVNNRFTDLRHQLELSYEGNPNLINEKLGIKRTIDEIFRSAQKQFNQWNKQEEDERTTTSLLKSLDFDFFEVLDSVTIARSRKHIEKYYNMK
EIGSFPERLKPISLRPRLTDLESAINYSEIYEQLMKLNLSVYIPTDYIFPSMLHKYVDTSRNINRAGREMGIRRLMSINLMKRLESSVESFRLTVGRVKQLIDSTIAEIDAYTSGGYSVIDGRELTGDADFDDDDRNTEYFAAEHSIKIDLGDMDYKTWRDRLSEDSEELALLKMLIDDITPKHDTKLQTLFGLIEGKLMNPINDGNRRILIFSAFSDTAEYLYHHVSSFVKSKFGLDTAMITGSVDGKTTIKG
QRASMNEVLTLFSPISKDKNLLMPNNTSDISVLIATDCISEGQNLQDCDYLVNYDIHWNPVRIIQRFGRIDRIGSRNEVIQLVNFWPDVDLDEYLFLKGRVETRMRISVMTATGDDDLINAEEKGDLEYRKAQLKRLQEEVVDIEDMQSGISIMDLGLNEFRLDLLEYVKNNGDMDKVPFGLHAVVPASEDCPPGIVFVLKNRNNSVNIDMQNRLHPFYMVYIGTDGEVICDHLSPKELLDKFRSL
CRGKTEPTMEACQDFNKETKDGRDMCGVSTLLSDAINSIIEVKEESDIDSLFSPGGTSALNTSISGLDDFELICFLVVR*
>Syntrophomonas_wolfei_subsp.metylbutyratica_JCM14075T_00939_hypothetical_protein
MLGLPRSTQVNRRVAKEKLYQNAALAPQTREMIKDQIDSVFWRNKLADSTMAISAGETVAEIQIFEIQLRQRELDKRVLPAIAKAIPYKILFILVFGDEAQLWIEASGTFYNTDWQPLGGFTLKFEGLNLDALYENLVRQISGGRLGTDGDIEEAVDRDKKRQKLERDIAALEKKLLREKQFNKQVELNGELKRLCAKLERMG*
>Syntrophomonas_wolfei_subsp.metylbutyratica_JCM14075T_00940_hypothetical_protein
MKFTFTAKTYPASQDFLKIIEGGEAYPLDTFIEKHKAEAVSPLDAVLDNCQIEYRGEVKNYKDYISEYLAFTERMRFFEFNSFKTWPEKDDRNAVHFVKKAKECLEIARFFTMKSAEWFESNEGLNWNTGYTPQFLFRCIYFGTAATWYANCYDHILQIVYWSLKLYTATKDRDDKPYDSTWDDKKIISLCVFDLVVAELKARGYKDIRKMLKTCSAQIDEVRQWSNYTKHKGGLDYKYIEADDPFM
VFIQKDGGEKEQIKSFEPPIEIDIDAEMHKLAEAHENLFNCLSAVVSEIDFNSRALQFMKTEEPAHE*
>Syntrophomonas_wolfei_subsp.metylbutyratica_JCM14075T_00941_Adenine_specific_DNA_methylase_Mod
MNKPKFETPNLTAENVAKIAELFPGVVTEGKVNFDLLRSMLGDEVYGDEAYEFTWVGKRAAIAEAGRPIRKTLRPCVEESKDWDMTENLYIEGDNLDVLKLLQESYLGKVKMVYIDPPYNTGSDSFVYNDDNGMDAEEYDDGIDAYDDEGNINFRANLKTNPRFHSGWCSMIYSRLVLTRNLLSKDGVIFISIDDGEVAQLRKLCDEVFGEENFIAIISRVAKTTSFRGNYFAPSKDYLLCFAKSIYD
LPGFSDEVDSSQFKKIETVGERKGEYYRDDIAFYLSTLETRPNQRYFIECPDGEKVIPPGTTFPPEKPVDGDGVWRWSLDNFELKKHLIVFKESTRSPLINEQGKPARWNLYTKSYLSDKQESGNLPRDLFDKFLNRNGSEDLKRLDIPFDFPKPVNLIKHLLKILNDEKDGIILDFFSGSATTAHAVMQLNAEDGGKRKFIMVQLPEVCADGTKAAKAGYKNICEIGKERIRRAGDKIKAEAGLTALNL
DIGFRVLKLDDTNMKDVYYAAGDYTQDMIALMESNIKPDRTDLDLLYGCLLDWGLPLSMPHTHEKIDGFTIHTYNDGDLIACFEERISEKAIREIASRKPLRAVFRDSSFNSSPEKINVFEIFKLLAPNTNVRVI*
>Syntrophomonas_wolfei_subsp.metylbutyratica_JCM14075T_00942_type_III_restriction-modification_system_StyLTI_enzyme_res
MKLQFRHQPFQADAAKAVCDVFAGQPFRTPTYMIDSGLGQLSLSQTHDYTGFSNAPIVVSDDKILEHIRTTQRQSGIKPSDSLVGRFNLTIEMETGVGKTYTYIKTMYELNKRYGWSKFIIVVPSVAIREGVYKSFQMTEEHFAEDYGKKIRYFIYNSSQLTEIDRFASDSAINVMIINSQAFNARGKDARRIYMKLDEFRSRRPIDIIAKTNPIMIIDEPQSVEGKATKERLKEFAPLVTLRYSATHKADSLY
NMIYRLDAIEAYNKRLVKKIAVKGISVSGSTATEGYVYQSINLSKGNPTAIIEFDIKNKTGFSPKTRVVTEGYSLFDNSGELAEYKDGYTVLRIDGRDSSVEFTNGKKLFAGDVIGAVSEEQLRRIQIRETILSHIEREQLFAKGVKVLSLFFIDEVAKYKQYDASGNAQGGTYAEIFEEEYKAIVGSMQLALTDTTEYLTYLDGIEAERTHAGYFSIDKKSGRMVDSKLGDKKERTSDDADAYDLIMKDK
ERLLDRREPVRFIFSHSALREGWDNPNVFQICTLKQSGSDVRKRQEVGRGLRLAVNQGGERMDETLLSREEVHNINVLTVIANESYDSFAKRLQSEIAEAVADRPRKVEAKLFTEKFGEDTALAIYESLIENGYVKRGELTEKYYEDKKNGTIEVPEEAAGQTADVIAVLDSVYDPNANKPENARKNTVEVMLDKGKLDSKAFQELWSRINHKSYYVVGFDEDELVEKAVKELNARLRVSKIYFKVETG
EQAKNIDSKEQLEYGEAFVRKDMVREEAASFMAMRASSSVHYDLVGKIVAETGLTRKAVTKILVRLEKVIFDQFGDNPEEYIIRASNIINEQKATAIIEHITYDKLTAAFGTDIFTEPDLKKGSLGVNAMEAKRHLYDYVIYDSTNERDFASALEAHSQEVEVYVKLPRGFYINTPVGKYNPDWAIAFYEGKVKHIYFVAETKGDMSSMELRKIEEAKAHCAREHFRAISGDNVKYDVVDSYDKLWELVQ
G*
>Syntrophomonas_wolfei_subsp.metylbutyratica_JCM14075T_00943_hypothetical_protein
MGGATIICPNCKKSFEISDAIQHQIEDELLRAKTEQSETLRKKYDAAAEIKLLKQAVENAISEAQQAAQLQIERERQTAKLELDKARQTTELEVEKAKRTTELETERLRREATASKESEKQLRKQLSDLLEELSKANKAREDAELTARKELLEKEKGIRAEAAQKASEDFNTKIREQEETINRLREQLTTAKQVAEQGSQQLQGEILELDIENALRSGFPFDTVEEVKKGERGSDIRQVVNEQFYMNCGLIL
WECKNAKTYQASWLGKLKDEIASEKAQMGVIVFNPTDGGDDFKQLADNIWLVKPRYVIMLATLLREAIVKVFAANRAAEGKDVKELIYNYLTGGEFSNRIRYILESYDEMAKQLDTEKKQAQKRWAAQEKILQKVTNSLYGMSGDLQGIAGREIIALPALGDDVEEVLSIKPRISEPRKRTEDIDDEY*
>Syntrophomonas_wolfei_subsp.metylbutyratica_JCM14075T_00944_hypothetical_protein
MMNTKITSAIEKLMLNAATFHGIPVEPTLINFFYGNNGTGKSTIARAVHANEGLSWQAGKSADDYSVLVYNQEFVEANFRDYGKLKGVFTVGEQNIKIQADIAEKNIQRAEQEKLNGENSIAKDRKESARNTLLDDFQGTCWDKTKAIRDGFDATQSGFKRKAQFAEKVLQIGNPAQHDIGELRTLYETAFDPKAATYKEFQPTGGTERLKGSPGNELLNKSITSSSDTPFAGFIKAIRATDWVRQ
GHEHFAETSEGKCPYCQQELPDDFEKQIADCFDGQYQEDINALRQFQEDYVSDMQGFLDVLNGNLQDAFPKLDLTEYKTKLALLEKTIEINIQRITDKLKEPSSVVTLENVKTLRDEINVLIEGFNKQIQANNDIVGAKRQKQAECTKKVWELIAFTLQSDVSTYRVRRKTFDDEITALIKLVDDGAKASRALGNEIAELNKRVVSTAPTIKSINDLLRDSGFQGFTLREKRGQQNVYEVVRQDGTV
ADKLSEGERNFIAFLYFYHLVRGSHTDADVSKDKIVVIDDPVSSMDSCVLFIVSTLVREMVGVCFNNAEYREQEREVQGDYIKQIFILTHNVYFHREITYNQASRYHCVSFYVVNKASNNSTIKHCVRQCISKPTEQENFNPVQNSYAALWGEYREVDTAIPLMNVIRRILEYYFRQLCGYDGVNIRKRVLDEHRDKFVETPAEGLPDYTKYHLATAMLSYINANSVGFSDGLNYVDDCTDITLYKDVF
KLIFEALEQEQHYKMMMGEEQ*
>Syntrophomonas_wolfei_subsp.metylbutyratica_JCM14075T_00945_hypothetical_protein
MAGKNMNYFLMDSTPNGRIKCTLANWTGVAYKIPRVKLVDCKNIDYLKQSGVYFLFSKGDGDKPLVYVGQSGTRKNGEGILNRLKEHHASPKLGLEDWYEAIAFTTSNNIFGATEISWLENRFFALALTANRYEAKNGNEPNAGNVTEEKESELEEYVDYAKIVMGVLGHLVFEPLVQNRAMQNAACKESDDYLEFSMEQKMQNSGMTVNAKCKRSSEGYIVLQGSVINSKTNEKTCSGVAKK
ARVSAKVDENNVLLEDVLFSSPSAASMFVTGASSNGYTAWKTADGKTLKDVETSEADEL*
>Syntrophomonas_wolfei_subsp.metylbutyratica_JCM14075T_00946_Virulence_protein
MNSEIVMYQTEDGLTKIETTFDNDTVWLSIDQMAELFQRDKSTISRHIKNIFNEGELIRNSVVANFATTAADGKTYQVDYYNLDVIISVGYRVKSLRGTQFRIWASNVLKEYMRKGFAMNDGLLKNNGGIYFDELLERIRDIRSSEKVFWRKVLDIYATSVDYDPRTEASTLFFKTVQNKMHWAVHGKTAAEKIYSSADADKPFMGMLSYKGERPAQSDALVAKNYCTEEELSALNSVVSAYLEFA
EMQARRRIPMYMSDWLETLDGFLKLSKHEILTHAGKSAKTAKQKAKEEYKKFKALHLEDVSNAEKDYIKALEDMEMKLLGEGKEK*
>Syntrophomonas_wolfei_subsp.metylbutyratica_JCM14075T_00947_hypothetical_protein
MNMVNNYIYNLLLALVCGVEGLSNSEIAVVDKDQIKQLNSLGKSSITISRKKNERAYRISVGGGRENAMQIKTNRFEELF*
>Syntrophomonas_wolfei_subsp.metylbutyratica_JCM14075T_00948_hypothetical_protein
MGKAADTETVDALKELNFYDNGVLSSKLIQQMTQLSNANIAKMRFQGQGYRDHVLTANEKANLKTFIELYYCYEH*
>Syntrophomonas_wolfei_subsp.metylbutyratica_JCM14075T_00949_hypothetical_protein
MEKSKISDQELNKVSGGREEYENYFCTQLDKQAFEFHYKRGQNNECPRYIGSGHYPACSNCFHIQPRM*
>Syntrophomonas_wolfei_subsp.metylbutyratica_JCM14075T_00950_hypothetical_protein
MKKDLNHQLLLEEQLKAVQGGRGNSLVTVILRCLYPGCTWSHRCSPSNEQQARQEHTQNNPGHNEFSADVQSPALME*
>Syntrophomonas_wolfei_subsp.metylbutyratica_JCM14075T_00951_Alpha-hemolysin_translocation_ATP-binding_protein_HlyB
MSKGWFDEQIKNRIRYDEEGFQNAFAQLSSVVLGKSLISSALNSDRLKTKNAIEILKPVNTEPVELPEEVEDMNDQLKYLLRPTGIMRRVVKLQGNWWRDAVGPMLGQTKSGDVVALLPVGLAGYEFFDYETGEKLRLSRKTKDKLQEEAFCFYRPLPLKQLGLTDLLQYILNTLSSADILMVALASLGVSLLGLFTPYLTKLIFERVIPAGEPGLLLPVALVLVGVALSSILIEITKTLLIARVQTKMNI
PVEAAMSRLLLLPAAFFKEYNAGELSSRISAIMQLCDMLANTFLSTGLTALFSFIYVFQIISFAPALVFPALMVILAQLAVTVITGLVELNLNRRRIALNARLNGLTFASFSGVQKIKLAGAERRVFAKWADTFKQQADISYNPPLIKVQPVVASAISLGGIIIYYSAAAASVSVADYMAFNASFGLVTGAIMSLAGVTTSFANIKPLMEMVGPILQAVPEVGENKQIITSLSGNIELNNVSFRYHEEMPLVLD
DISLKIKAGQYLAIVGKTGCGKSTLMRLLLGFETPQRGAIYYDGRDIARLDMRSLRQNIGAVTQDGRLFSGDIFSNIVISAPWLSRDDAWQAAELAGIADDIRAMPMGMHTVISEGGSGISGGQRQRLMIARAIVPKPRILMFDEATSALDNITQKQVSEALDSMKSTRIVIAHRLSTIRHCDRILVLDNGKIKEDGSYDELLQKDGYFAELVERQRLDDDSFVGKTTLF*
>Syntrophomonas_wolfei_subsp.metylbutyratica_JCM14075T_00952_Lactococcin-G-processing_and_transport_ATP-binding_protein_LagD
MPKIAKVPVVMQMEALECGAASLAMILAFHGKWVPLERVRSDCGVSRDGSNAVNILRAARAYGMRAAGYRLELDDVKQIEYPVIIHWNFNHFVVLNGFKKNYAVLNDPGRGTVEVPMEEFNKSFTGIVLTFSKTDDFVADGQPRSVLDFAKGRLKGTLVPIIFVILTGILAAVTGVMTPVFARVFMDRILTGANPDWLYPFILAMSGLLAFQIIVSLINTLYMLKIRGKLAIVANTTFFWHVLRLPM
EFFSQRMAGDIAARQSSNELIAETLISQLAPVLLNLAMLVFYLVVMLKYSLLLTAVGLAVTFINMWVARIISKKRVNITRAQMRDSGKLAMTTMSGIEMIETIKASGAENGYFERWAGFQASVNSSSVRFALLNQLYGAVPGALQQIANLAVLVLGVMLIMNGKFTVGMLLAFQGFMASFLAPVNAFIGLGQSMQEMRSSMERIEDVMNYQPDVEYLPQAEDDEREYHKLSGALSMKNVTFGYSRLA
EPLIKNFNLTVQPGQRIALVGTSGCGKSTLSKLISGLYRPWSGEILFDGQAREDICREVFTGSVAVVDQDIIMFEDSICNNIKMWDKSIEDFEMILAARDAGLHTEIMQREDGYNGMVLEGGRNFSGGQRQRIEIARVLAQDPRIVILDEATSALDAKTEYEVIQAIKDRGVTCIIIAHRLSTIRDCDEIIVMNKGKVVERGRHEELYQAGGLYTELIATE*
>Syntrophomonas_wolfei_subsp.metylbutyratica_JCM14075T_00953_NHLM_bacteriocin_system_secretion_protein
MSNTLFRKASLDSLSSPEQLNDYIKVSNPSIWIVLMAMFILLAVVLIWSFTGSLPTSIHTKGVVNSGKAVGYVDITEAGTIKAGQAVKVQAINQNTAINGHIDSVGVVPLSASEIAAELKSDYLAQALAPKGFAVTVDVSLDSSDLPDKTLVNINIVTDTVRPIDFLLK*
>Syntrophomonas_wolfei_subsp.metylbutyratica_JCM14075T_00954_urea_ABC_transporter,_permease_protein_UrtB
VESATIALLAAAIVAGTPILFAALGELMAERSGILNLGVEGMMLVGAVLGFIFASNTGNHWYGLLAAMMGGGLMASIHAFISVTLRGNQVVSGLALTIFGTGISGFMGKSYQGMPAADPFRPIAVPLLGDIPVLGPILFKQDALVYISLILAIGLWYILFRTRWGLRIRSVGENPAAADAVGISVSRVRYICTILGGMFAGLGGAYLSLAYAPSWQENMTAGRGWIAVALVIFALWNPLRGLLGSYLFGGV
EALTFRIQTIGIHVSSFFLAMLPYLLTILVLVLVTIRLRQEAGAPRALGVPYDREER*
>Syntrophomonas_wolfei_subsp.metylbutyratica_JCM14075T_00955_L-arabinose_transporter_permease_protein
MLRLRLIKREQDSLFARVSVPILSIVLAFLFGWVFLALSGFDPGETYLRMYRGAFGSSYAVSETMVKAIPLALCGLGVSLAFRIKLWNIGAEGQFYMGAFAASGAALFAGDLSAWLLLPLMFIAGFAAGGIWGFIPGALKAYWNVNETITTLMLNYVAILWVSFLVFGPWKDPASMGFPLTPRFPAGGYLPTLGTSRVHMGLLIALLLAVIFFIVLQYTRWGYEVRVIGESQEAALYAGMDVTKNIILVMII
SGGIAGAAGMIEVSGITHRLQQGISPGYGYTAIIVAWLARLNPFAILLVAFLFGALLVGGFGIQTFGIPFSIVLILQGAVLFLLAGEIFLNNRIVVMREEVR*
>Syntrophomonas_wolfei_subsp.metylbutyratica_JCM14075T_00956_Galactose/methyl_galactoside_import_ATP-binding_protein_MglA
VRQAPLIEMLDISKSFPGVQANDHISFRVYPGEIHALLGENGAGKSTLMSILAGLYRPDSGYIKVKGEAMRFRSPRMALEAGIGMIYQHFRLIQNFTVAENIVLGSPASSFRVNHRAIEKETAALSAKFGLEVNPAARINQISLGEQQRVEILKMLYRGCDILIMDEPTTVLTPQEVEELFKVLRLMSGQGKAVVLITHKLKEVMEIADYVSVLRKGKVVADGRIENLDEKTLTSIMVAREVEFSQIDRKKSS
EKVVLEISKLQVPGDQGHLAVENVNLTLHAGEIVAIAGVAGNGQKELLEAIAGLRNPSQGEIRLDGKMIQEQSVQEHILAAGIYMVPEDRLGMGLVPNLSVMDNAILRNYTRREVKRGLLFNYKKIIAYTRKLVEDYNIYLSDIHHPINYLSGGNLQKLLLAREIDQEPRVLLASYPVRGLDVAAAEAVYQILLKERDKGTAILLVLKDLDDIFRIADRVAVMYDGQINDVLDLESTDIDKIGALMLGVN
KVGVHYAETQAN*
>Syntrophomonas_wolfei_subsp.metylbutyratica_JCM14075T_00957_Purine-binding_protein_precursor
MRKSKLGILMLVLALLLVFTAGCGGQAEKDQGQADTEKIKVGFIYIGVPGDAGFTYSHDQGRQYLEKEIPEVETAILENVHEGADVERSLEQLIQDGCNIIVANSFGYGDAVLAVAKKYPDVKFLHCSGLETEKNVSTYFGRIYQARYLSGMVAGAMTKNNSLGYVAAYRIPEVVRGINAYTLGAQAVNPNVKVKVVWTNTWFDPTKEKEAAKSLLETGCDVIAQHQDTPSAQQAAEEAGKYSIG
YNSDMSKFAPNANLTSPVWNWGPYYVKTVKAVMDGTWKSEAYWGGLEDGIVDLAPISDKVPADIKKQVEAKKKEMTDKEWDVFTGPIKAQDGTVKVPEGKKMTDAEMLSFDWFVAGVEGNIGK*
>Syntrophomonas_wolfei_subsp.metylbutyratica_JCM14075T_00958_Outer_membrane_protein_P4
MKRSLNKRTLITTLLMIALMTSTVFAKPLLRIIQVSDSDIIIQLNGKIEISFGEDEKPVHNGTTYVPLRTFAEQDNKEVSWDGKNNQVIITDGSKISSSPLLEQNVSGTLWLMSSENRANSYQAFNLARMMFDKAMDENKSKKKLAVIVDIDDTLINSTSYTCQLIGGREWTTQAWEEWIASDAPEAIPGSVEFLNYVVDNGGEAFYITNRWPGIKGSTLESLKQLGFPAKDKKYLMVREDGTPSSKESR
RNLVEKNHNVVLLMGDNLEDFSDAFSPSLGLAGRAKAVDEFKEKWGKKFIVLPNPMYGDWEKTMVNNKKGLSIEETVEKRREILLRTDSSGL*
>Syntrophomonas_wolfei_subsp.metylbutyratica_JCM14075T_00959_phosphodiesterase_family
VRLAQRAEFNSSKLFLLKMLVILLTALLGAILAVWLLGSTAFELEGIEFRAGLTPGRSGITELHFPPFGVVEAKTHQGPVKLVISLEQIRSDTLKSHIDNLPDQKQLVEQLQSSARNKIAVFALRQVAVAFLGAFLLVFLIWRPGLLKSLLYTTASTLILILILAGVFRSYDTAAFREPEYKGVLSMAPVAIKFASDSLSDLQKIRDNTRQIVSNLGLLFSSSNSLPVMANPEEQGQVVKILLVSDIHSNPVGIVLC
KSLAARFKVDFLINAGDLTDMGSQLETGATQDLATVGVPQLFVGGNHDSPGTINFITALPDSQVLNGEMITLKGVKVLGFADPLSASTAVEYESPEAERESLKEQVSRIKEAVAAQGRPDILVVHNYRLGKEIAPLAGLVVAGHDHRIRIEEGPDGVFVDPGTTGASGLRGLYSEKGISYSAAIAYLLPGSGLLAVDMVQYNPVSHQFSLERQVFQSTESDLIPDRDADST*
>Syntrophomonas_wolfei_subsp.metylbutyratica_JCM14075T_00960_23S_rRNA_(uracil-C(5))-methyltransferase_RlmCD
LRCSIERINHQGEGVARVGGKVVFVPQALPGEVVEIVITDNRVRFARAQLVQLLEASPYRLTPLCPHYYECGGCAYQHAKYEEELVLKRQVVKDAIQRIAKIATVVEPVLSMPEPWHYRNKVTWHMAATGKQEPVLGFFREASRQLLPISHCRLISEKMNCLTARVKALLPLIQPAPGSEITLRESSLDHSTMLIWKGLQRWPAPEIWPELSAISDSLVVIEKNTCHYISGPQRLLDKLGQVKFALSPGAF
FQVNHCQAEEMIRLILSLVDFSGKTVLDAFCGVGSIALNLARQAERVIGVESYEPAIHDARCNAIEAGIYNVEFIHDLCERAVARIDEPIDVIVLDPPRSGCKKELIQACSRLSPREIVYVSCNPATLARDLALFAQLGYRPLFIQPIDMFPRTYHIETIVSLQRGNL*
>Syntrophomonas_wolfei_subsp.metylbutyratica_JCM14075T_00961_ubiquinone/menaquinone_biosynthesis_methyltransferase
MGKKLFVFNKIATVYGLFFNWQVGNYRYILNNVKDELEFSTYQSVIDIGCGTGALCKVFQEYGLEVTGLDPAEAMLAIATKKAGKIEPNEPFIRFIRGDALNGLPFRKRSFDLAITSYVAHGLMPKERLILYNEMKRVARHIVVLLDYNEHRSLISDIAEWLEGGDYFNFIGNIRDELMQQFGNLKVINTGKRSAMYICKID*
>Syntrophomonas_wolfei_subsp.metylbutyratica_JCM14075T_00962_addiction_module_toxin,_RelE/StbE_family
MNKLKISPEARNDLVEIKNYISQEPNSSQAAEKLISKITKKIRGLLDYPGIGASLSSVIDIQTEYRFLACANYLIFYRYEDEAVYVIRILYGRRDYVRVLFNDLLEKEEK*
>Syntrophomonas_wolfei_subsp.metylbutyratica_JCM14075T_00963_Hydroxylamine_reductase
VIDAVKAGAIKHFFLVGGCNGAKSGRNYYTEFVRQTPQDSVILTLACGKYRFNDLDLGEIGGLPRIMDMGQCNDAYSAIRVAVALAEAFDCGVNELPLTMVLSWYEQKAVSILLTLLSLGIKNIYLGPTLPAFVSPNVLNYLVDNFNISPISTPDEDLKKILG*
>Syntrophomonas_wolfei_subsp.metylbutyratica_JCM14075T_00965_Oxidoreductase_molybdopterin_binding_domain_protein
MLNKKKGWGKLVALCLMFALLFGCSGLMMPDTVDATGNNNQTNNDIIRIAEASTIDVLFDGEIALTPNETFSLTAYTSNESYTVSKTTPLGALQAAATASGFTFDITDKKYDSSKVLLLDNIGNYKYVKGGSKWYAYVNDVYKDGYDNAADGLNLIELVDGDRVEFYYAAGIATPTDYNAVKAAATAAVKTVARTGVAPTEWTLQLSGAKDEKVSKAYFENGLACASSTHKVSWTDADGNVW
GGVPLWLLVGMVDDNPDVGVKHFNFNDDLAAQKYQVNVISGDGWKATLDSDKIARNNGYIANTLNGEPLPQKLDSGKGSWPLHLKGEAIFGGQQVGNVVKIELSNLPQPAAGWTLELSGEVGDTITQEEFEGLACTGSGHYKEWTDKEGNVWSGVPLWVLLGAVDDIEKGKHWTFNESLAIGYTVKVSAADFSKTFNGADIAKSDNIIANKMNGKPLTDSYPLRLVGAGVTKTDGSG
GLGGKAVGNITKIEIPELQTPAAVEGSWNLTMTGKISDVFSQEEFEAALACPNSGHFVQWTDGNNDVWSGIPLWYLTGWVDDRQPHKYNANLATAGYTILVKAGDGYTKDFSSKDVAWSNDYIIANKLNGKPLTDSWPLRLVGTGVAKADGFLSGTSVSNIAEIALTSFETAVPIPEVRIIKYGEDHTTILQEKTVDYLWMQSNLEVIGDGTTVYRYEGITNNPDDVWDADETYPGGYKVANA
VKGSRIKDLCELVGGMGAGTEIVFVAKDGFETRLPYSSIYTDPSVQARQGDAILAWWGDGKYVPDYADGMRLFFTPEGDNVYGQWDMHETLPEKYWHYYYGDGVQYPSCAGLSAKYISEIRVYSVPQGDWTLALDGQDIGGLQYEVSKTYFESALACQFGANHKASYTDSKGRVWEGMPLWFLLGFVDDADQHSSKAFNDVLAKAGYQVVITADDGHSITIDSRDIDRNNNYIIANSLDGA
LIPESDSSWPLRLVGANASGATSISQIVSIKLLKSFKDWPLKYEPVHSTHDWNIIFSLPVSKNQNLDDHIYVTNVTGKKEPVTCRVNPENPEGIQVLPPQGGYQPGASYTLWINGSLKSEAGKPLKQAYKLQFTIAN*
>Syntrophomonas_wolfei_subsp.metylbutyratica_JCM14075T_00966_Disaggregatase_related_repeat
MNSKDLNSMLATAMEKKSKLLELYQDISSQMPDPNLQEEITRNILSSEKKHAALLQKLMQKLSDKATSVNGEKSDPARTRIADPETKNELAIDVNNDIEGQSSFTAESRREGNQLKVLIITPNQDSYISKYYANHNYRNSLSNYIGRDLSSGDIYHSFLHFDMQPLSKQARIIKANLVLNLCRNEVAYNNPVLTIRPVIEAWNESTITWNTQPQSSTTEHYSTEIKPKHFRFLNIDISDLAREWHQHPEHN
QGFIITGLENNGLLGFRSRRYFEESKRPTLLVEFTLDGH*
>Syntrophomonas_wolfei_subsp.metylbutyratica_JCM14075T_00967_hypothetical_protein
MTCPNFKQMVWEAIADEIGAVAMYAQMANMVSDVELKTLILSIAGDEYGHAKFWLAVYNLDD*
>Syntrophomonas_wolfei_subsp.metylbutyratica_JCM14075T_00968_putative_membrane_protein
MADRIEFVKNYSDLSTNKGFQFEFYCDRCGSGYRTRFKPSITGTVTGVLDTAGSLLGGLFSSAADVSERVRSASWEKSHDDAFEDACNELKPDFIQCPRCSSWVCRQKCWNKKKGLCKECAPDLGVEMAAAQASKSVEEIWAHAAMAEEDKKLGLENWRENVRASCPECEAPLASNAKFCPECGAKLKAAQFCPSCGAKVNPGSKFCAECGEKF*
>Syntrophomonas_wolfei_subsp.metylbutyratica_JCM14075T_00969_Putative_serine_protease_HhoB_precursor
MDEKDYLQSEGEHDDTIDEYLEMPEEEEASAGSKRSRPVFKIGAVVILLAFIAFAYAWLPFLWPPHLDFLQQDQALSEEDLVLRCKPAVVNIQAKKTGDALSSQGTGINLEAEGMIITNRHVVEGAESVKVSFSDEKSYFSRDLEIIDGYDLAMIKLKAKDLPFVAVETEQLVELGQIVTIIGNPRGFQRISSRGEVKEYFESDSGMPAFTINATIAPGSSGSPVLDEKGCLVGIIFAIGTVKLNGEEQERALA
IPATALNYSETR*
>Syntrophomonas_wolfei_subsp.metylbutyratica_JCM14075T_00970_Endoglucanase_precursor
MKRTLSSILALLLVALLSTGVMAKPDNGHGLGNSNSNSNPKAENSQKLSAPGQQKQMESRSQEEKSVKQEQKAIKQEEKVAKQEQKAVKKSLQERVKTSLCEEYRLYNSDNSVQGKAIRFTDSQRHWGARCIEEMAAIGLLRGYPDGTFKPDQKLTQAEALSLAMRVAAGDTARDASDEEVATDEEQSKLADVPAWLRGDADKAAKKGIIKLNRFHSAVQASRVQVVVMIAKALGLEPADTTN
IPFKDGLLISREDVGYILVLYQEGIISGNPNGNFNPNSAITRAEMASILQRLLNNKGEITSVSLPATATVQQGKSITLQATVKYSDGSSDNNVNWSSSDSSLATVENGVVTAAADKTGTLTITATASRGESSKSATCEVTVVEKLPVIAASLKETGKVGGHDGQVYQEYILEVDTRQISLAQDNVKSIILQKDNADALQLTPNTDSSLWFNVQKASGKYCLKVVDKDDKNYEAILDWTAPVKVTAVATG
RSGEHDGNSYSEYKLGNLDLSSFTCMFQIKPDSQVSELTANSDSNLWFKTNAQLTGQHIFLKKSNVWYSSTISI*
>Syntrophomonas_wolfei_subsp.metylbutyratica_JCM14075T_00971_Selenocysteine-containing_peroxiredoxin_PrxU
MWNDHELSKMINRDIPFAMLSDQDGSIGKMYGIYDEDSGVETRGRFIIDPDGIIQGFEVLTPPVGRNVQESIRQIQAFQLVRASGGTEATPSGWRPGKQTLKPGPDLVGNVWKVWKVSEAFEE*
>Syntrophomonas_wolfei_subsp.metylbutyratica_JCM14075T_00972_Selenocysteine-containing_peroxiredoxin_PrxU
MPEEFKAGCQRPPIKKPPIANEGNEQNIIKEAKPSMVKVGKPAPDFTASAYSAGNFFNISLSELKGKWVLLCFYPGDFTFV*
>Syntrophomonas_wolfei_subsp.metylbutyratica_JCM14075T_00973_hypothetical_protein
LRRDTRTAPEQKSLYLFSGFLRCADCGKAMTRSEVKGNVYYYCRTYKDQSKAACTKHTIRHDHLGQGVLYAIRQQVYIAVAFSEMVSRINTAPLQKSQSIRLNELIAAKEKELSKIMCYKQSIYQDWKDGEITHKDYRHMQEDYERQMEAISEVISLYEERQSLKMGLIRKTRS*
>Syntrophomonas_wolfei_subsp.metylbutyratica_JCM14075T_00974_hypothetical_protein
LFKRKWLYLIITVSCAIAMLVGSAYAAQGILIYINGQFTGLNAQMINNSVYVPIRAVSEALGADVSWIQEQQTVKVDSDIKMIASLPEEKTYIYALNEKDGRYDVLILSINGNKKVYDWKTMAHMTFLPDLSYIDEDNNAKLVSF*
>Syntrophomonas_wolfei_subsp.metylbutyratica_JCM14075T_00975_Regulatory_protein_BlaR1
MNTAFIAVLLMSATGILLVLCLALLRPLTTKTLTATWHYCMYILVVLFLLVPVGIVGGNLFSNMPSTTNQGLPNVPVILNDMITVQQTDILLPQTGQALPQTDPTAVTPAAPVTTAFILKTVLSFLPLIWLVGVFVFIILYGIQLVRFRRKIMRTSLQIDDTGTLLALKNSMTEMGIKGKLRLLSNNIIKTPMLAGLFKTFLILPEVELSERELKVILKHELTHFKRRDLWVKSLTLVANAIHWFNPAAYR
LTKNIDTFCELSCDEQVVSDMNMEERQFYGETILNVLCRVVNQHSGIYATLAESKKGIERRLTHMMNVKNTSKRIVIISFVIAATLCFTVFAMASFMNVTGEDTDLNENLGEYSINGINEPKPDDTIRDSDVMQESTANPQDEKSSVSESNTPNDSNIDGKYLWPVTGYNRISAAYGTRMHPVKKEMVFHNGIDIPAPEGTPVLAADKGTVVEIGDIGDNETAGNYVILNHGGDIQTFYSQLFGFAE
GLAEGDTVQQGDVIGYVGSTGKSTGNHLHFSLTIDGEYIDPMSVFTQEN*
>Syntrophomonas_wolfei_subsp.metylbutyratica_JCM14075T_00976_Regulatory_protein_BlaI
MSKLQKMSDAEREVMQFVWAAGRSVSSAKLLDDLKTINREWKPNTVLTFLARLIEKGILTSVRHGRSNEYIPLITESEYKQFETRTFLNSVHDGSVKSFITALYEGNDLTAEEIAELKQWFSQKEAKE*
>Syntrophomonas_wolfei_subsp.metylbutyratica_JCM14075T_00977_addiction_module_antitoxin,_RelB/DinJ_family
VEKTTTLNLRVHPDVKRRAEEVLSQLGIPMSTAIDIYLKQISMTGGIPFAVTLPKAPVSVNADMMTTEEIHAKLKEGYNDIEKGNVQDASAAFRKFRKIRV*
>Syntrophomonas_wolfei_subsp.metylbutyratica_JCM14075T_00978_Plasmid_stabilisation_system_protein
MKQYKIQITDMALSDMEEIYNYIAVQLQAPEVAMGQYNRIADAIETLDTFPERVKLMETEEERVLELRQMPVDNFSVFFHIREDRVFITNVLYSAAI*
>Syntrophomonas_wolfei_subsp.metylbutyratica_JCM14075T_00979_Inner_spore_coat_protein_H
MITSKYLNTLVLILMSLAVLAASFLTLYPHHPDSAAPAEPEYASKLFNKEQIMEINIEMEPEDFDWIIENATREEYRQADITINGNTYHNVGIRPKGNSSLRMVAQDKNSDRFSFKVKFDAYVEGQACMGLNKLALNNIIMDKTYMKEYLAYEIFDYMGVATPKYAYANISINGKPWGLYLAVEAMEESFVKRNYGSLEGHLYRPEGAGSDLKWAGEDAANYTGIRKMAAYNVTDSDLKKIITMIEQL
NNGSELEKYLDIDSILRYFAANSFLVNFDSYAGSLKHNYYLYEENGVCTILPWDFNLAFAGHEISSAQKAVNFPIDTPVTVNLSERPLIGKLLEIPEYRRLYHKYLQQLTEDYINSGRFEDSVEKLDKMINSSVKNDATAFYSHAEYEKSLPVLVEFARLRAQSIQAQLSGEQAATATGQNENSTPNIDASGIDLSALGGMGGGRGGGARPADGGGAAIPGMVKPEGETLKGPGAPPEGPGVFPGGNRP
DPETMMKAFEIMREAEGGQLSTAQIARLQELGLDDNMIDRMRNRPAAMEGRNEWAPPGDRANGPDQSVYNRKLNPSPHMRVKNKI*
>Syntrophomonas_wolfei_subsp.metylbutyratica_JCM14075T_00980_putative_Mg(2+)_transport_ATPase
VNPIEQFNFNDIFKSSFLDKISSFSMLDTAIALGLAFILGLFIFMVYKKTFKGVMYSANFGVSLLAMTLITTLIIMAISSNVILSLGMVGALSIVRFRSAIKEPIDIAFLFWAISIGIVLGAGLIPLAIFGSLFIGIIMLFFVNQKSSDNPYILVINCTDDESEQKALDYVKGKVKKHVVKAKTVAASSGVELTVEIRLKDMSTQFVNEVSRIAGVNNAVLVSYNGDYMS*
>Syntrophomonas_wolfei_subsp.metylbutyratica_JCM14075T_00981_VTC_domain_protein
MEQPKLRHEIKHYINTSDYITLRNRLKHIARRDSYAGSDGSYRVRSLYFDSPDNRALMDKINGISRREKFRLRFYNNDPSFIRLEKKSKINSLCWKEKAPLSREECEKLLGNDINCLLSCHNALLQEFYIRIKQQLLRPKTIVDYRREAYIYEPGNVRITLDSQLQSTLFPRDFLNPELATIAIAPAAMLILEVKYDEFLPELLRDIIQTNQRRSSSISKYAACRALG*
>Syntrophomonas_wolfei_subsp.metylbutyratica_JCM14075T_00982_Response_regulator_ArlR
MRLLLVEDERQLAEALSELLIKNRYAVDVVCDGEAGLDYALTGIYDLVILDIMLPRMNGLEILRQLRLNGISSPVMLLTAKGELEDKVSGLDSGADDYLVKPFAAEELLARIRALSRRKGEMIVDDTLDIADFTLNLSTYELEGKNSSVRLTRKEFDIMRYFVYRPNRVLSKDELIEKVWGFDSEADYNNIEVYISFLRKKLAHIGSQAAISTLRGIGYKLEV*
>Syntrophomonas_wolfei_subsp.metylbutyratica_JCM14075T_00983_Alkaline_phosphatase_synthesis_sensor_protein_PhoR
VFAKLRKKFVIINMSLLTIVFLVIFSVIYLLTAVAGERQTEFALNTIMFAPPRPFPDNPVLASSMVAELDEKGHMVKSFSFMNMSQQIIGQAVEQAVQSRDLSGKIRIEESYYAFLKHDTGNGTRIVFVDRTPQHRMLMNLLLTFLLVGSISLLLLFLISIYLADKSIQPIQEAFEKQRQFIADASHELRTPLAVIKTNLALITANSEESVKSQSRWIDYISSQTDSMANLVDDMLALARIDYMEAKTLFTRFDLS
QTLSSTLLAFEAIFYEKHISLKQEIQEDIFFNGEKENIKKVISILIDNAIKNTGANGSITVRLASDKNKIEMRISNTGEGIPAEDLQKIFERFYRVDKSRSRENRGYGLGLAIAKSIIEQQQGRIYATSTPGADTSFVVEWPRT*
>Syntrophomonas_wolfei_subsp.metylbutyratica_JCM14075T_00984_Phosphoribosylformylglycinamidine_synthase_2
MNKVEKALMHALFVHLHHNTDKAINFGLFKQLNQKVEQIWPGALLVGPDANDDAAVFRIPGTDCFITAKMESHCSPSVPRPYDAAATGAGGAMRDVVAMGARPIFLLDFIGTRPLEQEVIVGPCGFAGECTCGQCEIMSSQDRINLMLEGMAKMCETMGVFVVGGGFSTSFSDIVPAVVVAVIGQQVTEKPLTKPAKSAGDKIIIIGGTGTDGNDTLYRAGLVPVMQPALALFAEEKTTMEA
TLAAFTTGKIKACSDLGAAGIGAAVCESARFGGLGARVELDKVPVREANITPEEIFICETQARMLLQVQPEDVDEVLEAIRSRNGIAAVIGEITDQDYNVFSYQGQTVASIVNKPSPELLQELMV*
>Syntrophomonas_wolfei_subsp.metylbutyratica_JCM14075T_00985_Acetyltransferase_YpeA
MVTGPCRIERAGIKDLPLMLELWRCIPGLGVGSGDEEFSLRLFMERNPSTCLLLKDGERLLGTVLGGFDGRRGYIYHLAVHPDCQGQGYGRELLKEVLYELRKLGAPKIHLFAFNDNQLAAGFYQNQGWEWRRDIQVFSWDMDKWKTEDGKRKTE*
>Syntrophomonas_wolfei_subsp.metylbutyratica_JCM14075T_00986_hypothetical_protein
MKATRVIIMGAAGRDFHNFNSYFRDNPAYEVVAFTATQIPDIEGRQYPPGLAGKLYPQGIPIYAEAELPELIKKYDVDQVIFAYSDVSHEYVMHQASLVMTAGADFRLMGPKTTMLKSKRKVVAVTAVRTGSGKSQTTRYVAELITASGKKVAIIRHPMPYGNLEEQLCMRFASYEDLDKFKCTIEEREEFEPHIDNGMVVYAGVDYEVILRQAEEEADVILWDGGNNDIPFYASDLHIVVVDPHRP
GHETSYHPGETNLRMADIAIINKIDSADPARVEAVKEAIIKNNPQAKIIMANSPITVENPDSVKGKKVLVVEDGPTLTHGEMAYGAGVIAAEKLGAGELIDPRPYALGSIKGTFAKYGHLSRLLPAMGYGKTQIEELEQTINNSPAEVVVIGTPIDLRRVMSIDKPAVRVRYDLEEIGHPQLAELLAKVL*
>Syntrophomonas_wolfei_subsp.metylbutyratica_JCM14075T_00987_multidrug_resistance_protein
VLDIKKSLKNFFGINSSMISMLVMVILIGMGEKMAERFLPLYLIALGGSIYAVGFLNAMDNFLSAIYSFPGGYLAEKLGYKKSLMLFTVIALFGYAIVIIIPRWQAVLVGCIFFISWTAISLPAIMSLVSRVMSKDKRTMGVTLHSLVRRIPMALGPIIGGLLIGAFGKVEGIRIAFCLAFVLGLLSLIVQYYFIEDSEEKQEAHLHARDIFSSFSPGLRSLLLSDILIRFAEQIPYAFVVLWVVEVNKISAFHFGILTTIEMI
TAVLVYIPVAYLADKYGKKPFVLITFVFFTLFPLVLYFSHSFAALAFAFVIRGLKEFGEPTRKALIMDLAPEDLKAATFGTYYLIRDVIVSLAALSSAWLWHISPGTNFITAFFCGLAGMLVFALWGKDLKSEVQQA*
>Syntrophomonas_wolfei_subsp.metylbutyratica_JCM14075T_00988_Acetylpolyamine_aminohydrolase
MKVIFEPRFLSCYCSDPAASEHRLDKALELLREDYPLEQAAPAVDSDVLLVHNQHHLNRVRFNRQVYEMALLSAGAAIKAAELAAAGEPAFALCRPPGHHASPDGFWGFCYFNNIAIALEKQLQKGTINSALVIDFDLHFGDGTNNHLNNRPAFSYYHLDGDNAPNYLQGLKKLLAHLRGVDLLGISAGFDQHSQDWGRLLDTEDYHEIGRLLGEFAREKCAGRVFAVLEGGYNPISLGEAALAF
WQGIEGK*
>Syntrophomonas_wolfei_subsp.metylbutyratica_JCM14075T_00989_NAD-specific_glutamate_dehydrogenase
MGDKTYNPYEHAQAQFDRVAEIIGLEGATRELLRQPMREYHFLIPVKMDNGSTQVFRGFRVQHNDARGPAKGGIRFHPHETADTVRALAMWMTWKCAVVDIPLGGGKGGIVCDPRNLSDNEQERLCRGWVRQVARNVGPNLDVPAPDVMTTSRHMLWMLDEYEAIHGGRFPGFITGKPVGMGGSLGRTEATGYGVVYTLREALKEKGINIAATTASIQGFGNVAQYAARLYSELGGKAI
AVSCWDHVDRKPYTFRKKDGINVDELCGITDMYGGIDKSKAQDLRYEVLEGGAWLEQAADILMPAALENQITIANVEKISPQVRILAEGANGPTAPDADEVIKKRGIFVIPDFLANAGGVTCSYFEQVQSNSNYFWEKDEVLEKLDSKMTAAFKAVSELARQSSLYMRDAAYVISINRVAQAARMRGWV*
>Syntrophomonas_wolfei_subsp.metylbutyratica_JCM14075T_00990_hypothetical_protein
MKIGIDIDNTITHTTTEMIMHYARIFGEERKLNTLPDLNYYYLEDVLGWDESTADLFLNTYLGTIYREMKPKEEAVETIKQLKQEHELILITSRNQNFQKVEQVTIDWLRHYGIPYDDLILNATPNMHHFSKLEVCMQHGVQLMIEDHHDLAWELSRAMPVILFDYPYNRHLSSDSIIRVKHWNEVLDRVKQLPANTRRA*
>Syntrophomonas_wolfei_subsp.metylbutyratica_JCM14075T_00991_McHr
MIKWKDEYSVGVEHIDEQHRRLFEIAGRAYDLLKDELVVDKYDRIVAILGELKDYSVFHFHSEEEYMKSIGYRKFLSHKVIHDDFIEKINGIDLDQVDENQDKYLLDILDFIVRWIDGHILGQDKQYMQS*
>Syntrophomonas_wolfei_subsp.metylbutyratica_JCM14075T_00992_Inner_membrane_protein_YbiR
LTAFYLSIVIFLATYVFIIFEKVHRTIIALVGAGLAIALGLLSQEAAIEYIDFNTLGLLIGMMIIVGITRRSGVFGYLAIKAARVAGGKPLRILVALAVVTAVLSAFLDNVTTVLLIVPVTFVLTDHLEVPAFPFLFAEILASNIGGTATLIGDPPNIMIGSATHLGFLDFIVNLAPIVIVIMIITLLCLVLMFRKDLRADSQKMESIMAMNAREEIRDWAILKKSLLVLGLTISAFFLHGFLHLETATIALLGAAVLMLIC
AEEPEDILLSVEWPTIFFFAGLFIIVGAMEANGVMELLARQAMELTGGSLTATAVVVLWISAVFSAFVDNIPFVATMIPLLKTIGQMSGLTMDPIWWALSLGACLGGNGTLIGASANIIVAGISERHGQHIGFIDYLKYGFPLMLLSIAIANLYICLFLLP*
>Syntrophomonas_wolfei_subsp.metylbutyratica_JCM14075T_00993_hypothetical_protein
MNKNKVLSLFLSAFLILGLLFLPACSQKESKAQKPKESSGQKPEAPPETEKLLKDLKDIIQELDKMIKAQIMPSFQQAEAESSSASSSQKQKEGSSKEGKQKEESASSSKQGGGKKQESAPSSKNAWPSINQGVKSIHQNWNQLEPEAVRAGLPVSSREHWEKTLSSLTMAASKQNAEESLMAAISLYQPFADIAQVFAMTLPPDFFRVKYEVMAAMLESARQDWEKAALRLPRMQENWESLKVQAKD
ADPRLISCCEFALRDLEEAIKNQEMELVLIKGEISLDNLKKLEEKLKKAMTRGKS*
>Syntrophomonas_wolfei_subsp.metylbutyratica_JCM14075T_00994_hypothetical_protein
MESILDELISDINQAESAQVTHYICLGVGISSREV*
>Syntrophomonas_wolfei_subsp.metylbutyratica_JCM14075T_00995_Tyrosine_recombinase_XerC
MVAATKPINQIDTADIREYLSRFDRLKMSSISKKLSVAKSFFGWLTDEELIPKDPARRIKPPKKEQILPKALTIGELEQIRETCSTYRERALVEVLYATGGRLSEIQKLDRSDIDWQAMDTRVIGKGNKERTVFLSYKAVYHLRKYLMTRLDDEPALFITERRPYRRLSQRGIQRVIRVIAKRSGIQKKIHPHTFRHTFAFLTLNNGADLSSVHQALLGHTDPSTTQVYAQVTEEKKREAYKKHLVQ*
>Syntrophomonas_wolfei_subsp.metylbutyratica_JCM14075T_00996_hypothetical_protein
VTMKKNHILIVLIMAFMMMFSPAAYAAIPLDDVLIGSWEYNIAYLTNPTNAAEIQCALDNLGDGPLAYNIEGVTNGWTWVMSGKLLTLEEIAKFPNNITYKYDTTVRY*
>Syntrophomonas_wolfei_subsp.metylbutyratica_JCM14075T_00997_transposase
MKELIKDYCKQLRWGNSIVQNYADIKADTHEEFLAKLLEIEVKGRELNRKNRCLRQAGFDVIKTFNGYSFDHIEIPASIPLDDLKTAVLLKNRENLILYGPVGTGKTHMATAIGVEACNQGKKVGFYRTSTLVNELNDAQNAGNLGKMLKQLAKLDLLICDEWGYIPLDRQGAQLLFQVVADCYEKRSIIITTNLEFSKWNGIFYDEKLTSAIIDRLIHHSHLLVFGGQSYRLTHSTIGK*
>Syntrophomonas_wolfei_subsp.metylbutyratica_JCM14075T_00998_hypothetical_protein
VGYSRRNYFVPEPSFSDIEEFNQGLFAVAEKDHCRKHYRKGRVINELLQDDLTAMLPLPAKPFTIGRMQKLSANKYGKVCVDTNIYSASPQVANKELYMRLGAHQVELLNEQYQPIVKHKRLYGQNQELMDWLPYLITLAKRPNALKYTGFYRELPDPWQDYFNNLDYAEKKKSLNLLVRMITETDMSPATICLLETLDSGKADADSILLSYRRLTEPTFNDFLPLLSASINSPAIYTPDLT*
>Syntrophomonas_wolfei_subsp.metylbutyratica_JCM14075T_00999_Transposase
MIQQYHIKHLNFHKGKSLRSIAEETGHDFRTVKKYAEQTDFNEIRKPKRGRPSKLDPVKPIIDAWLKEDLNRPVKQRHTARRIYDRLCEEHKDIFNACERTVRNYVSARKKELYGEEEGYLPLEHPAGEAQVDFGEIVMIEQGQKVKGYELILSLPYSNAGYPQIFRGQNQECLLTGLKDIFEYLEHVPRIIWFDNMSAAVAVLGKKESGN*
>Syntrophomonas_wolfei_subsp.metylbutyratica_JCM14075T_01000_hypothetical_protein
MESILDELISDINQAESAQVTHYICLGVGISSREGSYRKLRVEKNEEGKIALGVDNDLHYIGDDCEIEKTGSFEDGGIDYEIREDGILLLLLQLQK*
>Syntrophomonas_wolfei_subsp.metylbutyratica_JCM14075T_01001_hypothetical_protein
MKSIGSRLTLYISMVVLFFCAGLGFISYFYASKALMANIEQSVAAKAQDATRLVGSELDNHLSVMETIAEQPEIIGMKWEEQGPILAQANKRLGYLMMGVAGLDGQVLTTAGSTTNLKDRDYFDQALKGQASVSDPIVSRIDGSTIIMMVSPIHGAEGEVIGVLAAALDGLTLSQIVAGVKFCDSGYAYMLNRQGQVIAHPKGEMVIEQYDPIAEAEKDPNLAPLAAIVNNMLKAECGYGEYLWIDG
SNKFMGYAPVENTGWSIAVTAPRDEVLAGLKSMTVGVLVASVFLLIGIALLGRILANPIKNAAGHAAMIADGDLRVEIAAEALARQDEIGHLARSLDIMVQGLRNMMHEVALNSQEVAASSQELAASGENIASTVEELSASTEEIAAGMTNISAATDEVNASGEGISSALLLASKDAEQGVLQSQRIEKRAARMQIGAQDHKQTAEDVYGTIKEKLTAAIEEARVVEQISELAENISGIASQTNLLA
LNAAIEAARAGEQGKGFAVVAEEVRKLAENSASAVEGIQGMTEQVQRSIANLTNYAEELLNFINTQIIVDYGDMVTICTHYKNDSDIFLSLIEKIKRHTEEVLASVEDINHSLDSTAGTIRDSSAGAQKIAEASQTAAIAASEISAASVRMAENAEKLNLLITRFKL*
>Syntrophomonas_wolfei_subsp.metylbutyratica_JCM14075T_01002_hypothetical_protein
MKFSKTLLGFKPGEVINQIENMEKEQQQKVERFKSEIEKIQAALKSAEEKREELQKQLNAYMERERIISDVMVTAQINAQRIEEQARERARHMLENAEEEMKQKLQELDFLRIKVARFKGEFREVLDNYRVSLEKVREEPEDSGFTPTLITKENRRHDVSS*
>Syntrophomonas_wolfei_subsp.metylbutyratica_JCM14075T_01003_6-pyruvoyl_tetrahydropterin_synthase-related_domain_protein
MDKTDRERALIESLKLQMDSLLQKKQAVDLGIEGLKQEIARQAMRQESLERSLEESRRIAAGIEEKAHLSAAKILAEAEALVAVKREKLSAIEQEIALLRAELEEKASGEPIPQKDDDNLHSTAQNSPDGISAPQHHNTDSYPNFSRVAASRASGDDNIYSMKLVGFVNARHFVVFGGATGPVHAHSWQVETEVDVPRGIGDTIEFSRVSQAVSAALGPYENTILNQVHPFDLILPTTENIAMYFFNRLD
EYLAKLGLKLYQLSLWETPTRGIQVNNHNLALDKQIAAELKARDGNAAYREAAAILPDFAKLDQREKELANNKKLLSKPPPVSAGSRRGPYSTRQYLLAAVLILFLSLLAYQHILWTPVEQRFPWGSDSWGHLFKAEMLYEQIQQGNYYPQFTEYWYNGVQPFRYWAPLPYYALAGLRGLSGDIFTAGHLYIYLCALLGALSWLLLSRRMGLWPAVMAALVWLLWQDNVRVAFSEGNLPRVLA
TALLPLLFALFLHIITRRKSYGAIIAVILTVQLTLLCHAMIAAVYCLSLALFVFFLWVFQGCSVKDLARGLMVLAAGVLTSSWWLLPSLSGGITGIDAQAVKQVVQFVPAHISLSPLCRFKNVETFYWGISLIMLLLSSLATWKSKPAWARSLTVCGIILLLITFPLFRAIYITLPLSHLLWPLRFSSFAALAVLASGFTLELEEKRQRWLQSSTVAACLLVVLLVFLLVDCLFSFRLLAHTGTRPFTIQSGEFIKQE
PGWRVATIDLSQLGSAPSFTFSEMAGLEQVFGWAWQGAVTSRNIMLLNTGLEQQYYPFLFRSCVDLGATDLVVKEDVITDLEAFREAAKRAGYQQRNKFADISVWRSISRPYLVQKNPRCLVIGKYGGTIALQFPEVEMALSSRIDDCSLEKLEQYSMLILSGANWHSKKQAEKLIRITRYPAVRFL*
>Syntrophomonas_wolfei_subsp.metylbutyratica_JCM14075T_01004_putative_integral_membrane_protein
MAGMPPNVLAKQPEFLGVYGEAVSIRQEIEVRGEDTRVILPPVWQQEGEWKAYVPMGLDKVELEFSYYGNQAPILGYKMLKGKKIWFLGANLSYHAYQSGQSESARLLKKILDLQTEYSGDTILPLQDYEASEEGFRMSYRLEQDAEVIVPISLLDGLVVKLDGRRIEAGNYENLLKLHLPAGTHRLEMSIEKTAIFTWGKYLSLFALLLVIAALVWLKRAGDKE*
>Syntrophomonas_wolfei_subsp.metylbutyratica_JCM14075T_01005_exosortase_family_protein_XrtG
VNPRLKVLLGVIIMLLLIWLFPPGSWLVPELQLDGKFADWRGRTSLSDPVGDGRGGNDLKKISWATNENDGRLYFMIERYLPEPRSHRMESCLFFDLNSNGKYDDKVDKYAEIFYRPQGLSRGDVSVYLYSMEGDLKGKYTGRWGEGTGTSTTSRLEFAIPMEDLEAYPAQFLRFYLADISGRSDRLPDQGDIQWAPFPVVSKSRPTIAVFFLLWLALTLFLYHHRLWRVFYYIWAAVGFCCLLVLLFH

[illegible]
>Syntrophomonas_wolfei_subsp.metylbutyratica_JCM14075T_01006_Poly-beta-1,6-N-acetyl-D-glucosamine_synthase
[illegible]
>Syntrophomonas_wolfei_subsp.metylbutyratica_JCM14075T_01007_Undecaprenyl_phosphate-aminoarabinose_flippase_subunit_ArnF
VNSNLLYLFVSIVLGASGQVLLKFAVNRMGQVNLAWPHTINTVIHIFTNPWIMTGILCFVSSMILWIKVISSMELSKAYPSVSLSYIIVFLFSIFLFNETVSSGKIVGLLLVSLGVYFLHI*
>Syntrophomonas_wolfei_subsp.metylbutyratica_JCM14075T_01008_Putative_glycosyltransferase_CsbB
[illegible]
GRGLVIS*
>Syntrophomonas_wolfei_subsp.metylbutyratica_JCM14075T_01009_hypothetical_protein
[illegible]
>Syntrophomonas_wolfei_subsp.metylbutyratica_JCM14075T_01010_Cyclopropane-fatty-acyl-phospholipid_synthase
[illegible]
>Syntrophomonas_wolfei_subsp.metylbutyratica_JCM14075T_01011_putative_integral_membrane_protein
[illegible]
>Syntrophomonas_wolfei_subsp.metylbutyratica_JCM14075T_01012_hypothetical_protein
[illegible]
>Syntrophomonas_wolfei_subsp.metylbutyratica_JCM14075T_01013_Phosphomannomutase/phosphoglucomutase
[illegible]
>Syntrophomonas_wolfei_subsp.metylbutyratica_JCM14075T_01014_hypothetical_protein
[illegible]
>Syntrophomonas_wolfei_subsp.metylbutyratica_JCM14075T_01015_hypothetical_protein
[illegible]
>Syntrophomonas_wolfei_subsp.metylbutyratica_JCM14075T_01016_hypothetical_protein
[illegible]
>Syntrophomonas_wolfei_subsp.metylbutyratica_JCM14075T_01017_hypothetical_protein
[illegible]
>Syntrophomonas_wolfei_subsp.metylbutyratica_JCM14075T_01018_Cyclic_di-GMP_phosphodiesterase_response_regulator_RpfG
[illegible]
>Syntrophomonas_wolfei_subsp.metylbutyratica_JCM14075T_01019_hypothetical_protein
[illegible]
>Syntrophomonas_wolfei_subsp.metylbutyratica_JCM14075T_01020_Branched-chain-amino-acid_aminotransferase
[illegible]
>Syntrophomonas_wolfei_subsp.metylbutyratica_JCM14075T_01021_Dihydroxy-acid_dehydratase
[illegible]
>Syntrophomonas_wolfei_subsp.metylbutyratica_JCM14075T_01023_Acetolactate_synthase_large_subunit
[illegible]
>Syntrophomonas_wolfei_subsp.metylbutyratica_JCM14075T_01024_Putative_acetolactate_synthase_small_subunit
[illegible]
>Syntrophomonas_wolfei_subsp.metylbutyratica_JCM14075T_01025_Ketol-acid_reductoisomerase
[illegible]
>Syntrophomonas_wolfei_subsp.metylbutyratica_JCM14075T_01026_2-isopropylmalate_synthase
[illegible]
>Syntrophomonas_wolfei_subsp.metylbutyratica_JCM14075T_01027_2,3-dimethylmalate_dehydratase_large_subunit
[illegible]
>Syntrophomonas_wolfei_subsp.metylbutyratica_JCM14075T_01028_2,3-dimethylmalate_dehydratase_small_subunit
[illegible]
>Syntrophomonas_wolfei_subsp.metylbutyratica_JCM14075T_01029_3-isopropylmalate_dehydrogenase
[illegible]
>Syntrophomonas_wolfei_subsp.metylbutyratica_JCM14075T_01030_2-isopropylmalate_synthase
[illegible]
>Syntrophomonas_wolfei_subsp.metylbutyratica_JCM14075T_01031_hypothetical_protein
[illegible]
>Syntrophomonas_wolfei_subsp.metylbutyratica_JCM14075T_01032_Glucose-1-phosphate_adenylyltransferase
[illegible]
>Syntrophomonas_wolfei_subsp.metylbutyratica_JCM14075T_01033_hypothetical_protein
[illegible]
>Syntrophomonas_wolfei_subsp.metylbutyratica_JCM14075T_01034_tricarballylate_dehydrogenase
[illegible]
>Syntrophomonas_wolfei_subsp.metylbutyratica_JCM14075T_01035_ATPase_family_associated_with_various_cellular_activities_(AAA)
[illegible]
>Syntrophomonas_wolfei_subsp.metylbutyratica_JCM14075T_01036_VWA_domain_containing_CoxE-like_protein
[illegible]
>Syntrophomonas_wolfei_subsp.metylbutyratica_JCM14075T_01037_Phytochrome-like_protein_cph1
[illegible]
>Syntrophomonas_wolfei_subsp.metylbutyratica_JCM14075T_01038_Response_regulator_rcp1
[illegible]
>Syntrophomonas_wolfei_subsp.metylbutyratica_JCM14075T_01039_Cyclic_di-GMP_phosphodiesterase_response_regulator_RpfG
[illegible]
>Syntrophomonas_wolfei_subsp.metylbutyratica_JCM14075T_01040_1,2-epoxyphenylacetyl-CoA_isomerase
[illegible]
>Syntrophomonas_wolfei_subsp.metylbutyratica_JCM14075T_01041_Organic_hydroperoxide_resistance_transcriptional_regulator
[illegible]
>Syntrophomonas_wolfei_subsp.metylbutyratica_JCM14075T_01042_Acyl-CoA_dehydrogenase
[illegible]
>Syntrophomonas_wolfei_subsp.metylbutyratica_JCM14075T_01043_Electron_transfer_flavoprotein-ubiquinone_oxidoreductase
[illegible]
>Syntrophomonas_wolfei_subsp.metylbutyratica_JCM14075T_01044_ferredoxin-like_protein_FixX
[illegible]
>Syntrophomonas_wolfei_subsp.metylbutyratica_JCM14075T_01045_Electron_transfer_flavoprotein_small_subunit
[illegible]
>Syntrophomonas_wolfei_subsp.metylbutyratica_JCM14075T_01046_Electron_transfer_flavoprotein_large_subunit
[illegible]
>Syntrophomonas_wolfei_subsp.metylbutyratica_JCM14075T_01047_hypothetical_protein
[illegible]
>Syntrophomonas_wolfei_subsp.metylbutyratica_JCM14075T_01048_hypothetical_protein
[illegible]
>Syntrophomonas_wolfei_subsp.metylbutyratica_JCM14075T_01049_CAAX_prenyl_protease-related_protein
[illegible]
>Syntrophomonas_wolfei_subsp.metylbutyratica_JCM14075T_01050_putative_regulatory_protein_FmdB_family
[illegible]
>Syntrophomonas_wolfei_subsp.metylbutyratica_JCM14075T_01051_Late_competence_development_protein_ComFB
[illegible]
>Syntrophomonas_wolfei_subsp.metylbutyratica_JCM14075T_01052_Ribonuclease_H
[illegible]
>Syntrophomonas_wolfei_subsp.metylbutyratica_JCM14075T_01053_hypothetical_protein
[illegible]
>Syntrophomonas_wolfei_subsp.metylbutyratica_JCM14075T_01054_Leucine-,_isoleucine-,_valine-,_threonine-,_and_alanine-binding_protein_precursor
[illegible]
>Syntrophomonas_wolfei_subsp.metylbutyratica_JCM14075T_01055_LIV-I_protein_H
[illegible]
>Syntrophomonas_wolfei_subsp.metylbutyratica_JCM14075T_01056_LIV-I_protein_H
[illegible]
>Syntrophomonas_wolfei_subsp.metylbutyratica_JCM14075T_01057_Lipopolysaccharide_export_system_ATP-binding_protein_LptB
[illegible]
>Syntrophomonas_wolfei_subsp.metylbutyratica_JCM14075T_01058_Carboxylate/amino_acid/amine_transporter
[illegible]
>Syntrophomonas_wolfei_subsp.metylbutyratica_JCM14075T_01059_hypothetical_protein
[illegible]
>Syntrophomonas_wolfei_subsp.metylbutyratica_JCM14075T_01060_tRNA_(cytidine(34)-2'-O)-methyltransferase
[illegible]
>Syntrophomonas_wolfei_subsp.metylbutyratica_JCM14075T_01061_Ferredoxin
[illegible]
>Syntrophomonas_wolfei_subsp.metylbutyratica_JCM14075T_01062_Thiamine_pyrophosphokinase
[illegible]
>Syntrophomonas_wolfei_subsp.metylbutyratica_JCM14075T_01063_DNA_integrity_scanning_protein_DisA
[illegible]
>Syntrophomonas_wolfei_subsp.metylbutyratica_JCM14075T_01064_YbbR-like_protein
[illegible]
>Syntrophomonas_wolfei_subsp.metylbutyratica_JCM14075T_01065_flavoprotein,_family
[illegible]
>Syntrophomonas_wolfei_subsp.metylbutyratica_JCM14075T_01066_Phosphoglucosamine_mutase
[illegible]
>Syntrophomonas_wolfei_subsp.metylbutyratica_JCM14075T_01067_hypothetical_protein
[illegible]
>Syntrophomonas_wolfei_subsp.metylbutyratica_JCM14075T_01069_Glutamine--fructose-6-phosphate_aminotransferase_[isomerizing]
[illegible]
>Syntrophomonas_wolfei_subsp.metylbutyratica_JCM14075T_01070_hypothetical_protein
[illegible]
>Syntrophomonas_wolfei_subsp.metylbutyratica_JCM14075T_01071_ferrichrome/ferrioxamine_B_periplasmic_transporter
[illegible]
>Syntrophomonas_wolfei_subsp.metylbutyratica_JCM14075T_01072_hypothetical_protein
[illegible]
>Syntrophomonas_wolfei_subsp.metylbutyratica_JCM14075T_01073_hydrogenase_accessory_protein_HypB
[illegible]
>Syntrophomonas_wolfei_subsp.metylbutyratica_JCM14075T_01074_Bicarbonate_transport_ATP-binding_protein_CmpC
[illegible]
>Syntrophomonas_wolfei_subsp.metylbutyratica_JCM14075T_01075_Glycolate_permease_GlcA
[illegible]
>Syntrophomonas_wolfei_subsp.metylbutyratica_JCM14075T_01076_Aspartate_aminotransferase
[illegible]
>Syntrophomonas_wolfei_subsp.metylbutyratica_JCM14075T_01077_putative_diguanylate_cyclase_YdaM
[illegible]
>Syntrophomonas_wolfei_subsp.metylbutyratica_JCM14075T_01078_Inner_membrane_protein_YbiR

MSNFWLSIVIFLLAYVFIIIEKVHRSVVVWVAAALVIFLGVISQAKAIEAIDFNTLWLLIGMMVIVGITRKSGVFEYIAIKSAKLARGNPLYIMVILALVTALASALLDNVTTVLLMVPVTFAITDRLEISPLPFLFTEILASNIGGTATLIGDPPNIMIGSATGLGFMDFINNLALPAIIILIVTIACLLLIFRRDLAAGEDKIAQVMELDEREFIKEWAILKKSLLVLGLTMLGFILHQYLHLESATIALAGAGLLLLVTK
VEPEEALLSVEWPTIFFFAGLFVIVGGLEVNGVIDLVAREALAITGDSPFRTGMLVLWLSAVGSAFVDNIPFVATMIPLLQSIGEMTAMPMESVWWSLALGSCLGGNGTLIGASANVIVAGLAERYGNTIRFLDFMKLGFPLMILSIILSAIYLYMRFWA*
>Syntrophomonas_wolfei_subsp.metylbutyratica_JCM14075T_01079_hypothetical_protein
MAREPRCRRVEYMPRVECFKPAGIPLSCLEEVQIKVEELEAIRLKDHLRLEQEDCARRMQVSRPTFQRILVEARAKIAYALSSGRAIRIEGGNYCMGAGYCRRRQREIREGEPCDFEGSAIKMESDAPDN*
>Syntrophomonas_wolfei_subsp.metylbutyratica_JCM14075T_01080_putative_selenium_metabolism_protein,_YedE_family
LDRTKWYVLTVGLLIGAIAVTLVKLGNPPNMGFCIACFERDMAGAIGLHRAGVVQYMRPEIIGLVLGALLASLFAGEFKSRGGSATFVRFILGVFMMIGALVFLGCPLRDVLRMAGGDYNAVVGFIGFIFGVGTGVFFLKRGFNLGRAEYSHSQGGGLALPLIMVFFLFLLLKATVFNPDAGGPLFFSAEGPGSMYAPLAFSLGAGLLVGILAQRVRLCLSGGIRDFMLIRDNTLLLGFIGIFAGALLLN
IALGFFQTGFAPQPVAHTMHLWNFLGLYLVGMTATLLGGCPLRQLILSGEGDTDAAAVVVGMIVGAAFCHNFMLASGAMKAAAAGAAASPGGPGIYGQIACIVGIIVVAAIGFAFRED*
>Syntrophomonas_wolfei_subsp.metylbutyratica_JCM14075T_01081_selenium_metabolism_protein_YedF
MEKVDVRGLSCPMPVIKTKKLLDQGVKELLVVGTSSVSKENVSKLARSSGYQVEMKVDAKNEWEMELRK*
>Syntrophomonas_wolfei_subsp.metylbutyratica_JCM14075T_01082_putative_nucleotidyltransferases
MVTIPETLRSGVKKYIERLRQEIPVEKALLFGSYARGTYDSGSDIDLAIFSDYFQGMRRVDGIAFLLSRADEIDLDLEPLAFTCDEYREKIGLVDEIIRNGIEI*
>Syntrophomonas_wolfei_subsp.metylbutyratica_JCM14075T_01083_HEPN_domain_protein
LDKKQKCSYWEDIETARAILSAGRYLYVVFMCQQSIEKLVKGLYVLFCEEEPPRTHSISMVFKKIIESQPDIFEDLEAYRKKREQYGSFFVKLLAYYIAERYPSYKEKLSQTVDRQEATEVLTTTEEVFKWLLSLKHYAPE*
>Syntrophomonas_wolfei_subsp.metylbutyratica_JCM14075T_01084_hypothetical_protein
MTTVGEDCCEVQCIHEDKVNLARRERLAEDTVMALADIFKTLGDATRLRILSALMQQELCVCDLAAVIDMSDSAVSHQLRVLRSQKLIKFRREGKVVYYSLADDHVRTLFAQGLEHVNE*
>Syntrophomonas_wolfei_subsp.metylbutyratica_JCM14075T_01085_putative_cadmium-transporting_ATPase
MPGMAIRMEDIAERGCLRLFAQGELDGSSLVEKMRGWSGILEVHFEAETSRMEYSYNPFLTDSTRIIASLRLLGYEAREEAFEGRAGSQASFAGAFPPAFPNSKSSVIRLEGLDCADCAAKLERTILAMSGVKDARVNFAAAKMKISHQIPLSEILKVIEKMGYRGREEGASGTEEKISLWKSNKYYLSTLLSGLLLLIAFSLQILQMPESWVHASYLLAIVLGGYLPARAGLALLLTSWEADMNLLMILAAS
GAVAIGQLAEAAVVVFLFSLGNALQGYSMDKTRNSVRALMDLAPAEALVRRNDLEQVMPVESIVVGDIFIVRPGEKIAMDGVILKGSSALNEASITGESIPVDKGPGERVYAGTMNSWGALEVEVERLAEDNTINRIIAMIEEAEEQRAPSQQFVDRFARYYTPVVILAAILVALLPPLLFGQPGEKWFYEALAMLLVACPCALIISTPVAIVSAIGAAARNGVLVKGGVHLEEMGRIKVLAFDKTGTLTR
GKPRITDIISSSELGENEVLRRAASLEARSEHPLAETVVEQARKLGLELAEVSNFRAMSGRGAAAEIGGKSCYVGNQRLFSEVGIAIQGQEELIAELEGAGKTLILLGDEKELLGIIALADELRENSREAIRQLKKKGIKKNIILSGDNERAARAMALSANVDEYQAGLLPEDKVQAVRALLEKYEKVAMVGDGVNDAPALAISTVGIAMGVAGSDAALETADIALMADDLSRLPYTVELGRRTLRIIRQNIVF
SLLLKAAILSLVIPGWLTLWLAVAGDMGTSLLVTLNGMRLTRVKA*
>Syntrophomonas_wolfei_subsp.metylbutyratica_JCM14075T_01086_Exodeoxyribonuclease_V_alpha_chain
METIYGYLERISYCNEESNFVVAKLKEKGKRELSSIVGNMVGVNPGESLQLTGRWLHNPKFGLQFQVDSYETIVPATVRGMEKYLGSGLIKGIGPVMAKRIVQRFGVEALDIIEKQPDRLQEVNGIGPKRVEMITQAWEEQREVKEIMIFLQGHGVAASYAAKIYQRYEKEAIQVVKENPYRLAAEVRGIGFLTADRIAQQLGIDPNSLLRAREGIVFVLNQYMNDGHVYCPRALLLEKAEEALQITAEV
VEQALEELLRERRLAAETMMGTGADGEAETRAIYLMPFFIAELQSTRLLLALQEEKSVVRPIDVEKAVDWVGQKLKIKLAARQKEAVATAVSEKIMIITGGPGTGKTTIVNAITRIFAALKLKITMAAPTGRAARRLRESTGLEAMTIHRLLEFSPREGGFARNRENPLNTDVVIVDESSMIDSLLMYNLLKAIPPQAILILVGDVNQLPSVGPGNVLQDLIESGRFPVIELTDIFRQAKGSRIVYNAHRINQ
GKFPFLPEAGEESDFYFMEEKDAEKALQKILGLCRSRIPRYFNFHPIRDIQVLTPMNRGLIGVSNLNVELQQVLNPRQPGISRGGRNFKIGDKVMQLVNNYDKGVYNGDIGWVKRFDVEEQELEVEFEDYPVSYDFSQLDELQLAYACSIHKAQGSEYPAVIIPLLEQHYILLQRNLLYTGVTRARKLVIIIGSKKALHIAINNDKPRSRDTGLKERLMKMGVVEIAQQ*
>Syntrophomonas_wolfei_subsp.metylbutyratica_JCM14075T_01087_2-succinyl-6-hydroxy-2,4-cyclohexadiene-1-carboxylate_synthase
MPDYALLDQPVLLDFLFFPRRELRPAPPGSFDFQVPVETEKEVFVHCRFYPGNKEWPWMLYFHGNGEVVSDYDEISRLYNAQRINLVVADYRGYGGSNGSPTFVHLVEDAHRIFRAVREELSRREFNPELWLMGRSLGSISALELAFHYQQEVRGLVIESGFASLTRLIKGLELPADYRVMEPIEQECLQMLGEIRLPALVIHGEEDNLVYLREGKLVFEQLGSQEKEMLVIPGAGHNDVIIMDLLRYF
GAIAELVHGRGNK*
>Syntrophomonas_wolfei_subsp.metylbutyratica_JCM14075T_01088_Sulfite_exporter_TauE/SafE
MKRKIFLEKYAFVVVLLLVIILALAWVLWNRPPLAANAALPRPDALELLLLAAIGIMAGCFGGLLGIGGGIIMLPLLKFGFNYSTTMAVGTTLFAVIFTAISGGYAHFFRGNAHRPSIAYISLGGVGGILLGSFFFQMLATQMKLLNLVLGLFFVFPGFFMIWESLFSRQNEAVESQNIREKPLGLSVLGMMVGFLTGILGLGGGYLLVPGMTYFFGYPIYLAVGTSLVSVIPITIVGGSIKLLQSYVLLEASLA
LAAGAIIGAQLGAASIKNFKPATLKFLFGLYFSYAAIRLISSFWF*
>Syntrophomonas_wolfei_subsp.metylbutyratica_JCM14075T_01089_homoserine/Threonine_efflux_protein
VAETLKRGFRAAPLTILGHATLELALILAILGGLSVYLKQQAVFHTIALLGGFFLILMGLSLVRDGLQGAFSSFKLKAEEEEKEAGEALAHFDASSKFDARSEIVSPSVMEPSRKYTRGRSLHPVAGGILISLANPYWSLWWLTIGLGYLSLALKSGSAGIVTFFSGHILADLGWYSLVAAAICGGRRVLNDSVFRILILGCGVFLLALGAYFIYLGIRG*
>Syntrophomonas_wolfei_subsp.metylbutyratica_JCM14075T_01090_thiolase
MAPGQRAAISGIGELRPIKNSGGRKSLELLAEAAALAIKDAGLGKKDIDGLLLTPPSEDTSFMWPAQVAEYVQFSPVFLNTVELGGASAVGTVARASMLVAAGICRHCLCLSGGVWDSQVFNTREGKIAVMSQAEIDYELPLGPMGFNSGYALVARRHMFEYGTKAEQLAKIAVDQRTNALANPYALFNEKAITIEEILSSPMIVDPIHLLEIVRPCSGAAAITVSRLEEAYDMPHSPISILGWGEACTH
NSILYAPSLTSSPVKMAAERAFQMAGLIPGDIELLSLYDCYPITVLITLEDAGFCPKGSGGDFVESTDLTYRGSLPCNTHGGQLSCGQASYAGGMSHLTEAVRQLRGEAGKRQVKKNHLAFVNGNGGVFSTECSLILGREG*
>Syntrophomonas_wolfei_subsp.metylbutyratica_JCM14075T_01091_putative_nucleic-acid-binding_protein_containing_a_Zn-ribbon
MEYREFYQKKLDEGELYLQFCPDCQDYIFYPRSLCPKCLKSDWEWRKLSGKAVLYSYTVVHYSALPEFKEDTPYVLALVELAEGPRMLAHLPGLPFDELQVGLALQLSSINREGRRIPAFQVVPN*
>Syntrophomonas_wolfei_subsp.metylbutyratica_JCM14075T_01092_HAD_hydrolase,_family_IB
MKLAIFDFDGTLLSRDTLPCLGREWIRQKRSILRYFLVYLSIAPDLLWYRIGLISRERMKAAAFRKFNYLFSGMSAEEITLFFHEAYPGLRKHFNSRIIEEISLARSQGYHCVLLSGSYFELMKMVAADLGLNTVIAARLAFKDGIFDPKGDTPFINGESKCSLLQEAFGGQEVNWEASRCFADSVADVKVMGMVGEPVAVNPDPGLLSHARREDWRVLSG*
>Syntrophomonas_wolfei_subsp.metylbutyratica_JCM14075T_01094_Acetyltransferase_(GNAT)_family_protein
MGNEYVNLTLDNLENEHLCCAIADKKHQCGVEVKKAWLRERIAEGHVFRKLNEKGKVFIEYAPLDKAWVPVIGDNYVYIYCLWVSGSFKGKGYGKELLEYCIADARKQGRSGVCVISSEKKKPFLSDKKFMIKNGFCTVDTINDEYCLLALSFDGTKPQFADNAKIQGIANKDLTIYYGMQCPYIPNCIEQIESYCKANHIALTLIAIDTLEKAKLVPCIFNNWAVFHSGKFKTVHLLNEGYLKKML*
>Syntrophomonas_wolfei_subsp.metylbutyratica_JCM14075T_01095_hypothetical_protein
MGQKHLGLSAEIIEVISDMDNYFKYDGLLSGLKC*
>Syntrophomonas_wolfei_subsp.metylbutyratica_JCM14075T_01096_D-alanyl-lipoteichoic_acid_biosynthesis_protein_DltB
LQFNSYIFILAFLPFTLIAYYQLHKLGWNLLAKALLLVMSLVFYSYFNFRYLYIICASILLNYFFSKQLLASGRTVPQKKWLLFIVIGLNLLILFYKYFNFFIENMNLLLQASFELKNIILPLGISFLTFQQIAYAVDSYRGETADYSFLDYAVFVAFFPRLIAGPIVLHNEFMPQLNEKKNHSINYENFSYGIMMFAIGLAKKIFIADVFAKAVTWGYASVGSLTSLDAFIVMLSYTFQIYFDFSSYTDMAIGIGLMFN
IKLPINFNSPYKALSIQDFWKRWHITLTRFLTKYIYIPLGGNRKGSFRTYANIMIVFLISGFWHGANWTFVLWGFLHGLASVLTRRFTTQWNKMHKILQWFLTFVFVNIAWVFFRADSITQGFTIIKRIAEFQTLAVSQTLLACFKVPVIIRLESLLHVVNSNAWVNGLDMMLFLAFTFVIILLFKNLHEMEFKPTMVNAVFVTVILLLCSILSMSISAFIYQGF*
>Syntrophomonas_wolfei_subsp.metylbutyratica_JCM14075T_01097_spore_cortex-lytic_enzyme
MNKKKWVVFVLGATSVCLLVLMIVNIIVDPFMQYHSPLSEMKPYYKYTQEYAMYWLIGRVKNTPTEAVIVGSSLSNFISSDKLGEQSGLRVVSLSKSAGRSNIYALFIKESLKRNRLQTIYYELNFPHLHMEYEGDIPLYLYNFNPIDDVKYVLNKDVSKMSAYILISCLSDKFQPGNDKRDKIPAKTFVEVDRSQSSSIYSTLAVARSIYVRHGKVADKSSVNDYHTKAKKNIEQNIIPFLKENPEIKFVFIVPC
ISILNSYDLVCEGTVDDYLAIHKDIMLSILKYDNAEIHMFSDLEGFVNNLDNYKDRVHYAPAGGDLICNGLKTGAYKVTPENIDERLRHLKSMAESFEIPFLKEGFTGDDVLEMQQQLIALGYNCQANKMYDGQTRDMVAEFQADHRLEVTGIATAETRALLQELSGKGDRRTVLFERSNSRI*
>Syntrophomonas_wolfei_subsp.metylbutyratica_JCM14075T_01098_Putative_anti-sigma_factor_antagonist_BtrV
MDIKKTQEDRKLTIALAGRLDSNSAPQLEAELKESLVDINDLVLDFAELVYLSSAGLRVILAAQKQMNKQGRMVIVKVNETIMEIFEITGFAEILTIER*
>Syntrophomonas_wolfei_subsp.metylbutyratica_JCM14075T_01099_multidrug_efflux_pump_VmrA
MHDNDYLVNHSFRRYLIPSILAILGGNISFMVDHIIAGQVLGSDALAAMSLVNPLFFLLTTLGTLVCVGSSTLASVSMGSEDRSTANKLFTLTMLLILLLGTVFSIVGFIFIEPLVKLLSNDPELYDLVYAYCRGLIPGAICVMAVYLPLNYFRIEGRAFLGMIMFLIMAALDIILDLWFTVVMGMGMLGLALATVISALLAVAVMSPFLFIKGGGYRFAPIGQSLRLLYAIAITGSPPALNNLYSVVRTFVLNM
LILAVGGPIAVASFAFVNSVNTLAQAIISGISQTVSPLVGVFYGERDVLSIKKVVFLAFRYGIITMAIFSISVSLLAEPICLAFNLSSPEQLRIAVPALIISSISLLGAVANNIFVYYYMTIGRTSIANRITLCRGFLFIIPAAGAMAQAMGILGIWAAFSISELLTLLMTILTVRKTLLKEPDLRGLLLLNYRDVAEGRFIAFSLDSNNQSVLESSDKISEFCTSCKLNSKQSMVISLAIEEILLLMVQHIFVDKKNKSIDVRIFV
RDDITTMRFRCAGRKFNPLDYYQQAMENADTSEALALGESMGLQMITKMTKQVDYTTSLGLNNIIIRI*
>Syntrophomonas_wolfei_subsp.metylbutyratica_JCM14075T_01100_hypothetical_protein
MSIGKKILFSLLGLSLLVTLLLCAISIYGFNDIKATFQQENKTAIDDLYEKNVAEITKSSSKLAISLANYYAQTINIDFERIMGELNTMKGGIETLYNSGGTTHAIPISRYEEYIISQAAHPSQQEVNRTLNVLGAATPMFDNILKIEPKISLAYLVLENGMVISSTKTFYPAVEKADMRTRDWYKDAVKAGTVNWSDLYTGTDNRSYVTCATPIHDKKGQLVGVLAFDLRVSEISEKTLNTGDNTYIAAFIMN
RQGHVLLSSLDVKATEKQPYFEKFIKNIDLMTTISDYYSDDSSIIGYAQIKSTDWLLVTVLDYEMMMAPVQAVAESVSATGDKMEQVISDETRRVFFVFLIIILVLLPLVFMTATRLSRSITRPVEILADGARVIGEGNLDYEFADLGTDEMGMLATTFNGMIVRLKEYMEAVARSAADKQRLESELEVARDI*
>Syntrophomonas_wolfei_subsp.metylbutyratica_JCM14075T_01101_Phosphoserine_phosphatase_RsbU
MLPQQIDCPGYDITGMMVPAKEVGGDFYDFFMLNEEKLCFVVADVSGKGVGAALFMTVAKMVLKTAAQAHNTTVDELLSTANNLLCQENPAELFVTAYVGIMDINSGQIAYACAGHNPPVIVHPDGSSEFVPVKPKLPLAAMEGIQYPIMYTQLQTGETLVLYTDGVTEATNLDEELYGDARLLAALPSLKRLSAPEMIEGIKSNVDIFVGEAPQFDDITIVAIRKIS*
>Syntrophomonas_wolfei_subsp.metylbutyratica_JCM14075T_01102_Gamma-glutamylcysteine_synthetase
MRNGKPMMRSSAEALIYRLFIDKFAGKGEPCIGVEIEMPVVNLACQAVEPGFIAAMLDLLKQRFAFTPTRFDQNGFPIEAVNPRQDAFSFETTFNTIEFAMAKKPGIDQIAANFYSYVTALQELAHEHHHLICGMGINPYAAYADSKPLNSPVMLAKAEFLKNFTSHHDGEIFHAFSAACQTHLDVELSQLPDLLNLLGKLAFVDAMLFANSLPFAYAGNRKFIARLPASLQREMAHPTLCFRDILWR
LCEAPNIRPYDRDYASLEEVVKHLLDLKIFIISDGSSGFKAIPPVRFADYIARERWSEADLSCFRSLEPVAVSRHGTVEIRQTCTQPLADSFAPAAFYTGIAENITVASELVHDFWQDNQINISNSQLRRQAVSREMAIPADRLREFINNLLAIAAAGLKKRKRGEDKYLGRFLKPDHEVKSPAQRQLDLLQEGWSFQEIMLACSALDESGNSIWDNDMNRGKGEADGLPV*
>Syntrophomonas_wolfei_subsp.metylbutyratica_JCM14075T_01103_Serine/threonine-protein_kinase_BtrW
VKRMDYQYKSSLADFDFEVLNSEIEAYCARDEFLLSRLFSIQLIVEELVTNILKYGVPTTANNMVEIKIHREESRVILAIADNTAAFNPLQIGEPDTSLSVEERQIGGLGLYLVRQKTASLHYEYQNGMNLVSAVIE*
>Syntrophomonas_wolfei_subsp.metylbutyratica_JCM14075T_01104_RNA_polymerase_sigma_factor_SigX
LYDFGRESEFERMYEDYFSKIYNYIFYRLLHREQTEELVSDIFLKVVVHLDSFQPGKASFNTWIFTIARNLLTDYYRKSKIIISIDDEGFPESALAVDINQQCRLIEDEERQELYRALVLLNDQQRNVIALKYYGGFNNREISRITGINESTVSSLCTRGIKKLQTLLNATR*
>Syntrophomonas_wolfei_subsp.metylbutyratica_JCM14075T_01105_hypothetical_protein
LKEIFHDVLCLKKGMDIFMIEDPRYQFSRESAIKEKLLQRLKIEYRKTRMAKVVPLDDDKLILVTAAGDARQQQCPYSCICEECPYFTGSCQQNLVKG*
>Syntrophomonas_wolfei_subsp.metylbutyratica_JCM14075T_01107_Transposase
MAETGVYHIMLRGNERKSILASDGEKQFFLDCIRGKQNETNFSLHAYCLMDNHVHLLINIQDNELASIMKGIATRYAFFYNNRHNRVGHVFQDRFKSEPIEDERYLLAAVRYIHNNPVKAGMVSNPEDYRWSSYWAYIQPELFGEEWIDSASILGIIALDRKSAIEEFIRFSKDVEETFIDIEEKSGIKTLDEGGCILREYLKQEGLPITIKEIKENQELRQKAIVFLRANTQLSQRKIAILLGIDKSIVEKVKL*
>Syntrophomonas_wolfei_subsp.metylbutyratica_JCM14075T_01108_hypothetical_protein
MIEKYRHPVLFYTLATLIPWSFWLAAAYLSHITPGSHIYITVASILGVIGLITPMIISFSMILPDPELREDTLKRFFGFRGIKPVYILATCFLMLASILLAQAVSLLFGYSPEQFKLAGVFSFSAGIFPAWFMLLLAPVLEELAWHTYGTDCLRKRFSLISTSLMFGIYWALWHFPLSFIKDYYNANLVETGWIYSLNFAVSLIPYVVLMNWLYYKTNRNILVAIVFHITAGFFNEIFLTHPDSKIIQTLLLLVLTIVLVIK
ERDFFFKREYPE*
>Syntrophomonas_wolfei_subsp.metylbutyratica_JCM14075T_01109_Transposase
MAETGVYHIMLRGNERKSILASDGEKQFFLDCIRGKQNETNFSLYAYCLMDNHVHLLINIQDNELASIMKGIATRYAFFYNNRHNRVGHVFQDRFKSEPIEDERYLLAAVRYIHNNPVKAGMVSNPEDYRWSSYWAYIQPELFGEEWIDSASILGIIALDRKSAIEEFIRFSKDVEETFIDIEEKSGIKTLDEGGCILREYLKQEGLPITIKEIKENQELRQKAIVFLRANTQLSQRKIAILLGIDKSIVEKVKL*
>Syntrophomonas_wolfei_subsp.metylbutyratica_JCM14075T_01110_5-methyltetrahydropteroyltriglutamate--homocysteine_methyltransferase
MKTATIGYPRVGTLRELKFATEKYFEGVLSKAELQDTAALIRKTHWQMHINNGIDFIPSNDFSFYDNMLDTAVLLNVVPKRYSSLQLDSLEEYFAMARGYQGLNGDVKALAMKKWFNTNYHYIVPEIEDDTEISLTGTKPFDEFLEATNWGIVTKPVIIGAFTFLKLARFTGNKTAIDFTKDIIVAYSKILKKFNDLGAEWIQFDEPALVMDLSADDVALLKKIYGEILLAKDSTKVLLQSYFGDLRDC
YQEVTTLPFDGIGLDFIEGNHSLELVKHFGFPADKTLFAGVVNGKNIWRNNYSQTLLILDELKKTRATIVLNTSCSLLHVPYTIKNEESLTDEFKKHFAFAEEKMGELADLKKITASNTPAEITEYIKNTRLFKESRFCTDASVQAAVSSLTPEDFIRHPAFHEREKIQKAVYNLPLLPTTTIGSFPQTAEVKANRSAFKNGKITSKHYIEFNKKKIAECVALQEKIGLDVLVHGEFERNDMVEYFGECLNGF
LFTQKAWVQSYGTRCVKPPIIWGDISRSQAMTVDYIVYAQSLTKKYMKGMLTGPVTVLNWSFPREDISLQECAFQIALAIRQEVLELEANGIKIIQIDEAALREKLPLRKSDWHCEYLDWAITAFRLVHSGVKAETQIHTHMCYSEFSDIIKDIDEMDADVITFEASRSDLAIIDVLNRCGFKTAVGPGVYDIHSPRIPGTEEIKEAVFKMLKKIPQEKLWVNPDCGLKTRGNAETIPSLKNLVDAAQEVR
RSLQL*
>Syntrophomonas_wolfei_subsp.metylbutyratica_JCM14075T_01111_5,10-methylenetetrahydrofolate_reductase
MRTSEIFNNKTVLSLEIFPPRRTASIGIIYKTLEELQGLNPDFISVTYGAGGSETNTATLEIASAIKNNYGIESVAHLPCINLNKDEVLNMLEGFKQAGVENVLALRGDVNPALVPKQDFRYASDLVSFIKENGDFNIIGACYPEGHSESSSLLDDIRNLKIKVDAGTDQLITQLFFDNSYFYSFRERASVAGINVPIEAGIMPVVNKRQIERMVSLCGVKLPKKFVTMMERYENNPEAMRDAGIAYAVDQIV
DLIAQGVDGIHLYTMNNSYIAKKIYAAISTLLVA*
>Syntrophomonas_wolfei_subsp.metylbutyratica_JCM14075T_01112_Cys_regulon_transcriptional_activator
MTLQQLRYIIKIVECGSITEAAKQLFISQPSLSNAVKELESELGIEIFHRTTKGISLSIDGTEFLSYARQVVEQTELLEQRYLGKKPSKQLCSVSTQHYAFAVNAFVNLLLALDVDEYEFTLRETRTYEIIKDVKNLRSELGIIYLSDFNEKVLNKLLKENHLTFNPLFKADPHVFISSLHPLAYKNSVTLEDLDDFPFLAFEQGEYNSFYFSEEILSTAPHKKTIHVSDRATLFNLLIGLNGYTICTGVLNSDLNGD
NIISVPLETEEKMLVGWIANEKAHLSAIASEYITELKRLISEYGYTVLS*
>Syntrophomonas_wolfei_subsp.metylbutyratica_JCM14075T_01113_Transcriptional_repressor_SdpR
MNSYADYVPVFKALSDETRLKIIDMLSCGEMCACDILEKFSISQSTLSYHMKILTESGLVNGVRDGAWMRYTLKSERTEDAINFINYITNEKDDCICKQASCKVCAEEK*
>Syntrophomonas_wolfei_subsp.metylbutyratica_JCM14075T_01114_Arsenical_resistance_operon_trans-acting_repressor_ArsD
MRKIEIFDPAMCCSTGVCGPSIDPELMRIATVINSLKDKGIIIKRHGLSNEPQDFIANKVISDLLQKEGAEILPVTLVDGAVAKTKEYPTNEEIEKWLEIEIESKPAVIINSSCGPKGCC*
>Syntrophomonas_wolfei_subsp.metylbutyratica_JCM14075T_01115_Arsenical_pump-driving_ATPase
MNTKSFDINKINLTQYLFFTGKGGVGKTSAACAVAVNLADSGKKVLLISTDPASNLQDVFNTELNGKGVQIEGVPGLVVANLDPEEAAREYRESVISPYRGKLPDSVINNMEEQLSGSCTIEIAAFDQFSHFITDNTSENEFDYIIFDTAPTGHTLRMLQLPSAWSNFIAESTHGASCLGQLAGLQDKKDMYKNAVINLADKAKTTLILVSRPEETPLLEAERSSQELSNLGINNQLLIINGVLGEASDDL
SQKILSKQQHALKNLPPGLRGFKAYTIPLRSYNILGLDKIRAFLNSDAYISADAKNKLTDLKPINDLVEDIYAAGKKVIFTMGKGGVGKTSVAATIAVALSKKGVKVHLTSTDPADHLSYVFSGAENISVSHIDEKKELKDYQNEVLAKARETMSEDDVAYIEEDLRSPCTQEIAVFRAFAEIVDKAENEVVIIDTAPTGHTLLLLDSTLSYHREVQRTKGETPVSVQRLLPRLRDEKQTEVVIVTLPEATPV
FEAIRLREDLSRAGINNKWWVVNQSLSVADTNNPMLAARAEAERTWIEKVQQISDDNFVVIPWLQDPSIKNIGNFKD*
>Syntrophomonas_wolfei_subsp.metylbutyratica_JCM14075T_01116_putative_methyltransferase_YcgJ
MNNKEEIREFIRKNYSEVALQGSAGGCCSSGCSCSGPQINIKDASINIGYSESDFEDIPIESNMGLGCGNPIAMAALKKGEIVLDLGSGGGFDCFLARKQVGESGYVIGIDMTPEMVKLARQNAEQSGYKNIEFRLGEIEHLPVADGSVDVIISNCVINLALEKQQVFNEAYRVLKPGGRLSISDIVATAQIPEYMKNDLVSLVGCIAGAEYVGNIENMLKNAGFENIRMIPKDNSKEIIKSWVPGKNIEELVA
SYIIEADKYEYNMP*
>Syntrophomonas_wolfei_subsp.metylbutyratica_JCM14075T_01117_arsenical-resistance_protein
LENEARLSFLDKYLTLWIFIAMGIGVLGGYLFPNLAQSLSQFSTGTISWPIAIGLIIMMYPPLAKVKYEEIGKVAQNKKVFSFSLLQNWVVGPILMFILAIIFFSNKPGFAIGLIIIGLARCIAMVIVWNTLAKGDNEYCAALVALNSIFQILLYTVYIYIFVTLIPKWLGLSWGGQVVDVSMIDVAKSVLIYLGIPFAAGFLTRFSLIRIKTKEWYEKVFIPKISPLALIALLYTIVIMFITKGEQIIQNPFDVINIAIPLLIYF
AVMFFASFFAAYRWGFRYDQTVTLAFTAASNNFELAIAVAVAVFGIASDVAFTAVIGPLVEVPVMISLVNSALFFKRKYFDDKGLPKLYEGT*
>Syntrophomonas_wolfei_subsp.metylbutyratica_JCM14075T_01118_Meso-diaminopimelate_D-dehydrogenase
MNEKIKAAVIGYGNVGRMAVEALQSSIDMELLGVVQQPYCSDSARILAAMDIKMVQSLDQLSGVQVALLCIPTRAVPSMAREILSLGINSIDCYDIHGQLADLRLELDEIAVANHSVAVISAGWDPGTDSMLRCMYEFMVPQGLTYTNFGPGMSMGHTAAVRALDGVEDALSMTIPLGTGIHRRIVYVQLQSGANLAEVKKAIKTDPYFIKDETHVIPVADVKQLIDMGHGVDMVRKGVSGSTH
NQILRFSMQVNNPALTAQIMVAAARASIKQKPGAYTLIEIPIIDFMYGQRDDIIRKLL*
>Syntrophomonas_wolfei_subsp.metylbutyratica_JCM14075T_01119_hypothetical_protein
MGLVQENLSIIVYLIELLKKRREEHKKQY*
>Syntrophomonas_wolfei_subsp.metylbutyratica_JCM14075T_01120_ABC_transporter_ATP-binding_protein_YtrE
VVALDDLSLDILSGEICCILGTSGSGKSTLLNLLAGLEKASRGSILIRGKNIGKMNERQLALFRQKNIGFVFQSYNLLPSLSALENVSLPLVFRGIKKEKREQLARALLQDVGLKTHLKHKPAEMSGGQQQRVGIARAFVGSPPIILADEPTGNLDSKTSLEVIQLMLKLAREKQQTLVIVTHDSEVAGFADKTVFIRDGKIAEVIINNTNVGERRDET*
>Syntrophomonas_wolfei_subsp.metylbutyratica_JCM14075T_01121_hypothetical_protein
MKHRSVFFIVFLSMIISCYLLGWYKPQSLEAADNLSLEEPKLVIGRSNSMPVFKAGEEGNLIIPIQNSRDGEARNINVSLLIDDVAKFPFELEKMSLQTGMSSIGGYRTESAIFFGIKVAKAAESKVYPIGVKIDYTSQNGNEGQSSGTIYVKIENEQKTPGLILAGIQMENEQIDSGESQTIELKIKNDGHLTARDIDIKLAGFTANGLRLDQPLDTFHLKEFKGQEFKYIPFKIYADPSMESGTYPLDLVMKYK
DEFNKDYSKEEKLYLTVEGSGPEGKNKKATPRLIINNYFYGGEYAVAGEVFSLSMSLLNTSQVKMLNNIKLSLSSEENIFSPVNSSNSIFIDSIPAAGSITQSIELKPKVDVANKTYNITVDIEYEDEKGNKYTAKELIGIPVAQPVKLVFSDIEMPPEVFAGTPVGISLDFYNTGRALIRNLIISTEGDFTIQDGNVFVGNLEAGKSDYYDITVTPNKEGKLTGKVLFDYDDDTGKHYKVEKSFELEVMKAAPP
PADIPPPGMEEAGAAKWKKPAIIGSGILVAGIAAFILRRRQLRKKEEVSLDE*
>Syntrophomonas_wolfei_subsp.metylbutyratica_JCM14075T_01122_Macrolide_export_ATP-binding/permease_protein_MacB
MNNLDLFKMSAGSLLRRKLRTILTLLGVVIGTSSIVVMISLGIAMDLSFKESLKQMGSLNVIEVYPSYDMMPSMGSRRSEGIKLDDQAVRTLEQIPGVEAVMPSKNAYMRISAGKMVGDVSLIGINPELMEAFDFKVQKGRLLSSSDKDAIVFGQRVAFNFRNPRSRNQEVHIMFGPGPDMPEEPPLDLINDKMLLTWDMSYGERRSNKPDTDSDYTPPPPRKVKGVGILEGGFSEKAYSAYINLNTL
EELLEENKARHVDKSKQRKEDKYQNIKVKVSDIQQVESVQERIKALGFQAHSLSDMVKSMKETSRKMQAILGGIGAVSLLVAAIGITNTMIMSIYERTREIGVMKVLGARLGDIRDLFLLEAAMIGLGGGCIGLLFSYLVSYILNRVTAGFMGSMGGDVGISVITWELGLAAVVFATMVGIISGYSPARRAMKLSALEAIRTE*
>Syntrophomonas_wolfei_subsp.metylbutyratica_JCM14075T_01123_RNA_polymerase_sigma_factor_SigI
MELGTGVSELDKRLGLVWEKYAAGQKTAVDEIYDDIMPFCLKVCSRVSGKFVNPYDDEASIARLAILEAMARYEPDRGSILLFLAQVVKSRVLDHKRREKRWYSLPFSLLKQVENPVDRQADEDLLEILNDMDRKQEIENLQLILADYEISFADLVTNSPKHRKTREETRRIALLLAQTEELKQDLREKKMLPVKQLIELGRFKRKTLERYRKYIIASAIILAGDFPHLQTFVLSREGMEEPPSSLKL*
>Syntrophomonas_wolfei_subsp.metylbutyratica_JCM14075T_01124_hypothetical_protein
MSGKKGIVMEKGEGWVIVLLDSGEYDQFYTREDWEVGQIYHRKTPIKMLAVAAVFLLVFLATLDFFTVLTYARLSTGVELGFNRWDRVVKVKAQDENAQAIVAGLDLKGATAETAVAEVFKKSLQDNKALRDGQAAATYSLELEGKLQDEVRARLWQKMDRGVNKAIEQQEGQEYSWKTVGKNKKLLVPQTKVLSVKSHRLTGKPPQSEPRARGIIENNGSAKSEAKGRAQSGFEDNANNDI
KKDEISDEIKGQAKDRGNKPEEKKYSEPEKREEGVPSFTDKEKNIKPEVTTESGFNKDNSADSEGTEIKDKDKAEEKTEDKEKDKNKDKAKDEAKDKDEAKDKDKDKKEDKYRGRNQ*
>Syntrophomonas_wolfei_subsp.metylbutyratica_JCM14075T_01125_flagellar_rod_assembly_protein/muramidase_FlgJ
VRIDHTAASSPASMGLGQNQININKEQSQTGKFARELENAQNKKDQAKLYKACQELESVFLNQILQSMRNTIPKSDFTGHSYATELWESLLFEEYAKEISKSNSTGLADIIFRQLSQKL*
>Syntrophomonas_wolfei_subsp.metylbutyratica_JCM14075T_01126_Distal_rod_protein
MMRSLYTASTGMYAQQLNIDTLSHNMANVNTIGFKKQRLEFQDLLYQTIRRPAADEDRNEPVGLLVGLGVRPAAINTIFNTGNLQATENPLDVAINGTAFFGVKVEGNDELLYTRDGAFKIDANGQLVTADGHLLEGAGTFDEGAYDIKIASNGKISYMKPGQENPEEAGQLKLFKFINPAGLQKMGKNLYQATPNSGEAEEWQPDSDNSVSLEAGYLEMSNVQIVEEMVNLITAQRAYEINS
KVIISSDEMLQTAANLRR*
>Syntrophomonas_wolfei_subsp.metylbutyratica_JCM14075T_01127_sporulation_protein,_YlmC/YmxH_family
MIKVSDLRARDVVNILDGKKLGNIIDIDLDLDLDNGKVTSLILPGRNRRYSFFSRREELAVPWNKIIRIGRDVILVEMPVANEIDPYRRDYLLDEEHL*
>Syntrophomonas_wolfei_subsp.metylbutyratica_JCM14075T_01128_Transcriptional_repressor_NrdR
VRCPYCQEFESRVVDSRSGDDGSFIRRRRECNACKRRFTTYERLEERGLLVIKKGGNREAFNRKKILDGIARACEKRPVSMEEMENMVASIERELRDLYEHEVSSKAIGEMVMDRLQRRDEVAYVRFASVYRQFTDLNSFIKTIDQLKQR*
>Syntrophomonas_wolfei_subsp.metylbutyratica_JCM14075T_01129_hypothetical_protein
MMDSPNNNSHEKGLGPLQRLSDKQVKSWITISKVYPPDDIVIESMLKFYGIPVRISRREIPQFPFTTGPLAEVIIAVPEEMVDEARDLLESENAGK*
>Syntrophomonas_wolfei_subsp.metylbutyratica_JCM14075T_01130_Alkaline_phosphatase_synthesis_transcriptional_regulatory_protein_PhoP
MHLLLLFYAKIKKSTNRADGKILMFGMEVSRVQNAKILVVDDETHIVELVKFNLEKEGFRVAVAYDGQCALEMVNDAPPDLILLDIMLPQMDGLEVCRTLKQHSEYKTIPIIMLTAKGEEIDTILGLEMGADDYIKKPFSPREMVARVKARLRALRILKAEKAMGKIAYVFKELTVVPEKYEAFIGEEKLELTPKEFELLRLMASNQGKVFTREVLLEKVWGYEFSGDTRTVDVHIRHLRQKLGDDST
YPDYIETVRGVGYRFVEGQAKS*
>Syntrophomonas_wolfei_subsp.metylbutyratica_JCM14075T_01131_Alkaline_phosphatase_synthesis_sensor_protein_PhoR
MDIAQEKRMMQSILSCMVDGVLVLDIYGCIIHANKAAESLFLVREGVLLGKRLTQISENREISDMIDEVLSTGNESFTETTISPSTLIFRVHAVPRKDHHDRIEGAIVVFHDVTDARNFDQIRSAFVGNVSHELRTPLTSIKGFVETLLDGAMENSDTCRRFLSIIDTETNRLTRLIEDLLTLSSIESQERIVYLKPACLLRSIRNVMNMFGPQISEKSLHVEFIHPYPMPLIQADEDLLGQVFINLLDNAIKYTP
RQGKIIIQCRRRDSRIITTVKDSGMGIPHESLPRVFERFYRVDKARSRQQGGTGLGLSIVKHIVESHGGEVFVDSEIGKGSTFGVSFLVI*
>Syntrophomonas_wolfei_subsp.metylbutyratica_JCM14075T_01132_pyridoxal_phosphate_enzyme,_YggS_family
MEKIKNNWESIKNKIEQAALKSGRKASEVTLVAVSKTVDVETVRRAHSLGINDFGENRVNELLSKRESLPGINWHLIGRLQTNKVKDIVGKTTLIHSLDRWRLAQELDKRGLALEQEVPVLIQLNISGEEQKAGFKPAELGEVLAAAEQFKALSIYGLMTMAPLDPDPESARPVFRELFQLRQKFIDRSYKNVNLKYLSMGMSQDYAVAIEEGANLVRIGSALFH*
>Syntrophomonas_wolfei_subsp.metylbutyratica_JCM14075T_01133_Cell_division_protein_SepF
MGVMDGLWRWLGVEREEVREEIIELPAYSEENQRGPTNVVSIHSNKTFKVVVCEPESFDEVQVLADHLKSRKQLILNFDNTPPEVSQRIIDFISGTTYSLEGHSQQLGKNIFIFTPSNVEIAKDHRSLMRKHGFANPFGGEK*
>Syntrophomonas_wolfei_subsp.metylbutyratica_JCM14075T_01134_Pyrroline-5-carboxylate_reductase
VSRSLGLIGCGKMAYALMKGIGGAEFLGFDSLLVNDIDPERGVLFQEEFNAIPTEAGEVLHSSRVIILAVKPQQVRMVLDANRAWWTTEHLLISLAAGIKTGSYENEIGLHLPVVRVMPNTPCLVGQGVSAISAGRYAADKDLQLVAKMFEQLGTCVMVEEKHMDAVTAISGSGPAYVFLVVESLMNAALQVGLDLKLARQLLVNTVKGSMEMMEQSGEHPAILREQVCSPGGTTIAGIRQLEEN
GLRRAFFQAVEKAYERSIELGKD*
>Syntrophomonas_wolfei_subsp.metylbutyratica_JCM14075T_01135_YGGT_family_protein
LAIYQIIQIVNMAFNVLVWLIIARCILSFVRHNPYQPLIKFVYDVTEPIMAPFRRLIPAAGGLDFSPIIAVLAVTLIQKIVVELLYALV*
>Syntrophomonas_wolfei_subsp.metylbutyratica_JCM14075T_01136_putative_protease_YhbU_precursor
MPDNYVELLAPAGRWDVLEQVAAAGADAVYLGGKRFNMRMLRQDYNFSDQELRDAVDFLHQQEKKIYITLNNLYYDVELNELKDYLFFLQDIAADALIIQDMAVLALCRELKLDLPLHASVQMGIANLQAARFLEDQGFSRAILSKNLSWEEIQEIHDGSKLALEFFAFGDLCIAHAGQCYMSSLVAGESGNRGRCLKPCRWPYRLEGAGQSKIMAHYLAHNDLCLYPQLSALLAAGVTSLKIEG
RMRSAEYLAHIIGIYRQALDKISEAPETYEMNEEDYRKLTEKRVRDFCSGNLFSRPGIESIGLSGEREPLFPTRPVKLKKLAANETSEVPEGPGIGWPEQQPCWSVKIGDFKSLEEAMAALGIKRLILEYAAGNRCPSGWGAEEIKTALSMVAGSDIEIWVETPRIVSQNELGKLEEDLRSISQLPDRVGFIVNEPGSFRLLRSLGQAVWGGHGLNTSNYRAARFWREQGMSGVTASLELEWSRVKSLLSQVE
ETELLVHGPLAGIITDFCLPRAVQPAAVGNAGKDCPLYCLQEDYYLMDEWGQKYRVRADGSCRNQIFYPYDLALIRRLPELLTAGLKYWRIDGQYYPAELLKQTLGLYMETAQDLPAGKWSPERANSLLKLFPAGLGSSPLFAEKG*
>Syntrophomonas_wolfei_subsp.metylbutyratica_JCM14075T_01137_Minicell-associated_protein_DivIVA
MITAMEIRNQQFPKSLRGFKQDEVKKYLLNLAQDYESLYSENASLKEKIQNLEYELKKYRKLEETMNNSLILAQQTAEDLKSSARKEADLMLEESKKRIIEVMMVYQEIIKRMNIFNVELKSQVSAELELLEKNQKKIEELSNFFYSKDFKEVMESLEKVNLKE*
>Syntrophomonas_wolfei_subsp.metylbutyratica_JCM14075T_01138_hypothetical_protein
MNKPLIRTERNGDWLRFGVKVQPRSSRNQIVGEQEGDIKIKVMAPPVEGAANQALQKFLAELFKLPKKDIRIVRGETSRHKIVEIRGIEAENLLAFIPPITK*
>Syntrophomonas_wolfei_subsp.metylbutyratica_JCM14075T_01139_putative_DMT_superfamily_transporter_inner_membrane_protein
MSAAKKRMQAEVSMLLVAAIWGSTFVVVKNALQEIGPFLFLGLRFLLAFLVLLFLSFPCIKKTNRHTLLAGGILGLFLFIGYVFQTIGLKYTTSTNAGFITGISVVLVPIIFSLLYRQRPTVSTTVTVILAATGLFFLSFPRNSFSLAYGDFLVLICAFGFAFHIIFVDRYSHRHNAIAITAVQIFFVGIVSLLIGLISEPWPEHFSANLLTAFFITAVFATSLAFLLQNALQKYSTPTRIAIVLTTEPVFAALAGYLWAQ
EILSQRAMFGAGLILVSMLISILTRKSKNVVEANV*
>Syntrophomonas_wolfei_subsp.metylbutyratica_JCM14075T_01140_Endonuclease_III
LQRSVEIIKCLKKEYPEAGTLLRHSSPFQFMVAVVLSAQSTDEQVNRVTAELFADYGTPEALAAIDLSLLEEKIRGVGLYRNKARHLKKLAQIIVEQHQGEVPSEFDKLLSLPGVGRKSANVIRSVAFKKPGLGVDTHVHRVANRLGLVNSKLPEQTEKALKEQIPEECWSEAHHLLIFHGRRICQARKPQCNNCLIEGLCEKRFGK*
>Syntrophomonas_wolfei_subsp.metylbutyratica_JCM14075T_01141_Isoleucine--tRNA_ligase
MAKGKYDGTLNLPQTGFPMRANLPQREPEILKFWDEIDIYRRIQEKNADRPQFILHDGPPYANGDIHLGHTLNKVLKDIIVKYRSMSGYDSPYIPGWDTHGLPIEQQAIKNLGIDRHKTDVVEFREHCRDYALKYVEIQKEQFKRLGVRGDWEDPYLTLSPGFEAIQIKVFGEMAKKGFIYKGLKPVYWCGDCETALAEAEVEYNEKVSPSIYVKFPVKDGKGVLAEDAFVIIWTTTPWTLPANTGI
CLHPGFDYVLLEVKGEKYLLAQGLLEAVAGELGWDNYQILDQFKGEELERVLCRHPFFARDSLLVLGEHVTLEAGTGCVHTAPGHGEDDFRVGQEYGLAVISPVDDRGRFTAEAEKFQGLYVHDANKAVVEELEKRNMLLKAARIEHQYPYCWRCKQPIIYRATEQWFASIDGFRQDALDAIDTVKWIPSWGRDRIFNMIRDRGDWCISRQRTWGVPIPIFYCESCGEALINDETIQRVSELFAA
NGSDIWFAKTAAELMPPGCSCEHCGGSTFRKESDIMDVWFDSGSSHMAVLEPRKELRWPSDMYLEGSDQHRGWFNSSLSTAVAIRGAAPYREVLTHGFVVDEQGRKMSKSLGNVVDPLRMTREMGADILRLWVSSADYRNDVSVSPNIIKQSAEAYRKIRNTCRFILGNIFDFDPGKERVSYDKLSELDQWALLKLDKLIRRVTKAYEDYEFHVVFHSIHNFCTVDLSNIYFDILKDKLYCSHPQD
AERKAAQTVLYDIINALVVMLAPILAFSSEEIWSHLKKEGQAESVQLLEWPQANELYLNQAIETRMSRVLELREVVTKALEEARSKKVIGHSLGAWITIYAGPGWMEILEATAGLEKIFIVSRAELKPEAPTEALALEGVEGIRVMVQAAEGSKCERCWIIENSVGEDLKHPTLCQRCAEVIAQLQG*
>Syntrophomonas_wolfei_subsp.metylbutyratica_JCM14075T_01142_hypothetical_protein
MPEREPGRDESHYLQELNRKMDELSLNMEKLGIAEYLEMLRNPRRLFFINFWAGLSRGFGMAIGFTILAAIVIYFLQKLIILNVPLIGDFIADLVAIVQNQLRVQ*
>Syntrophomonas_wolfei_subsp.metylbutyratica_JCM14075T_01143_General_stress_protein_16O
MDYQNNLKQRKAEIEQVIKQKRDELIIPLGEWSDELSLYDQHPADIASEVTQRETNLGILEMLEYELEKVNDALSRLQNGQYGVCEACGQQIEPARLERLVNTTLCANCARSQQKGFKRPAEEEVVAAGQMSDLGEGIQVAGYELYE*
>Syntrophomonas_wolfei_subsp.metylbutyratica_JCM14075T_01144_GDP-mannose-dependent_alpha-mannosyltransferase
LRIGIFTDSFIPYTSGVVTSILTFQKELSRLGHDIHIFAPSYPNFQQEEEQVYRFFSLPAPTNPDFSLAIPIYPGMNMLVRRLDLDIIHVQSPFTMGRVGLHYGRKYHIPVVFTYHTLYDQYVHYVPIAQDLAREMAIKYSVSFCNHCSHVVVPSSEVETILKQNEVKTPLSVIPTGVPLDKFESGDKEWLRRNYNIPAENRVLLFVGRLSREKNLEFLIKAFRQVKERVANSTLVLTAQGPLESELKKLVPELG
LSLERDVVFTGALPFDTLVNVYYSADLFVFSSVTETQGLVLIEAMAAGLPVVAVGAYGVQDMVDHEINGLLTPLDIEVFSDAISSLLLDNRRYRQYQLNARQKAESLSAGKMALKLEQLYQTLYDQSPPRYRGFDLGRWLGS*
>Syntrophomonas_wolfei_subsp.metylbutyratica_JCM14075T_01145_UDP-N-acetylmuramate--L-alanine_ligase
MKNLRLTLAIIAGKILIYISRLLGHQGTSFPGRIARKIYPRILRTLSPNIRREIVVITGTNGKTTTSNMLAEILKQQGYSLVHNRLGANMLSGITTAFIEKSDIHGSRKFDYALLETDEATVPLLLRETRPRLVLITNFFQDQLDRYGEMDHTIKLIQEAVKEQEIEMLLNADDPLTANFQAETGLKCWYYGFDDSSYDDSRSMDSREARYCAFCGHQLEYEKYHYAQLGQYSCPRCHSRNPERDFTGQEL
EMSPDIELKVDGIALKSPYQGFHNAYNILAAVSLARLLGVEDENIKKAIASFQPQAGRMEAFFINGKRVVLALVKNPAALNQTLASLIYDPGRKNLFFALNDNAADGRDISWIWDADVELLEYENVMVEQIVCSGQRSGDIALRFKYAGFPQERIRIEEELPRAIELAVQGSAESSYILCSYTALFGCQRILREMQKRSGTGEEAEKAYQAQS*
>Syntrophomonas_wolfei_subsp.metylbutyratica_JCM14075T_01146_hypothetical_protein
MFHPADVTIKVAPAKASRRQGASDRRSRERFTLRVRLVFHPEGVTIKVASHGREISAVPLEPRDKPQVLPMRSALSGTTDAPFGRY*
>Syntrophomonas_wolfei_subsp.metylbutyratica_JCM14075T_01147_cobyric_acid_synthase
MAHFHLAQLYPNLMNLYGDRGNLLCLQKRLEWYGHDSSIENINPGDNLDFKHIDLLFMGGGSEREQLELVLQDLADKTDSLMKAIEEGLPILFICGAYQLLGEYYRSHEGKEMQGLGLFSFFTQGLKERLTGNILIQTTLNGAEESVVGFENHGGRTYFIDKRLQAFGSVIRGFGNNGEDKQEGIRYKNLLGSYLHGPLLPKNPKVADFFIKAMAERKGLKIERILDDSLENYAHEQVKRKLMG*
>Syntrophomonas_wolfei_subsp.metylbutyratica_JCM14075T_01148_Heptaprenyl_diphosphate_synthase_component_2
LSTGNPQVEPIIDYLLESSGKMLRPRLLYLAASPYPHDPLMVRDMCVAVELIHLASLVHDDVIDQALTRRGQESINSRYNNKTSVLTGDLLFATAFNLINQHPFPRVMDQITRTIQLMCQGEIKQLSLAFDLDISEEDYYEKSYSKTACLFACSCRVGAWISSMPEKNASLLEDYGLYLGYAYQIIDDILDLVSDSQALGKPAGNDLKEGNITLPLIYALKTKEYGSLLRKILAADYSQLDTRELKTILLDS
GAIEYARSISRQFLQKALASLELFPVGPAIKEMKNIALFIMEEYYARLSYVNEGELVKVVN*
>Syntrophomonas_wolfei_subsp.metylbutyratica_JCM14075T_01149_Tetraprenyl-beta-curcumene_synthase
MPGLVRKIQEPAHLLKQQGLRGGRVNLLSHYILFTLNQVKSQLKEWEQEARLCPNEELRKQALSSIQDKGFHCQGGAFFAVPYREKEILLLRLIIAYQTLCDYLDNLCDRAGCSDGKAFRQLHRSLEDALSMAEPSILPDYYSYYPSQDDGGYIEKLVRVCQSCVRQLPSYPAVYPDLIHLVRLYIDLQVIKHIDPWQREEELREWAKKQLYAYPGLCWQEFAAAAGSTLAVFALFGLASIQDLPPAES
RKVVENYFPWICGLHILLDYFIDQEEDREGGDLNFTFYYADNRECISRLEDFISQSHAQSARLNDYDFSRVVVEGLLAMYLSDQKIAQRRLQAAARQLLAASGPNALKTFQLCRLVRKIF*
>Syntrophomonas_wolfei_subsp.metylbutyratica_JCM14075T_01150_hypothetical_protein
VDSEKKESPDPGNKKNTAKRSKIDDSDSDDSVLKSAGREGILTEDKGWKKGI*
>Syntrophomonas_wolfei_subsp.metylbutyratica_JCM14075T_01151_hypothetical_protein
LTEGIIKRINELAKKKREQGLNPEELQEQKKLYDVYLNGIRQQVKSQLEAVSAHPPGCNCGKEHHHHHNGECQK*
>Syntrophomonas_wolfei_subsp.metylbutyratica_JCM14075T_01152_hypothetical_protein
MKGTYGDKVEVKYVDTDKTGFDNYPLVRRVLQMGYTFPITLINGEPKFAGGIMEREINNIVDELIK*
>Syntrophomonas_wolfei_subsp.metylbutyratica_JCM14075T_01153_Methyl-accepting_chemotaxis_protein_4
VVQRSLKTRIILMLVIFNLLVFGFFYWNSLMLQHMLVKDFEKEYIAQTSDTVAECVRDAEKEARILSNSLFANAAIKEAFIQQDRAELLRLVLPVYEQWQKDHHISQLNFLAADGTVLLRAQKPEQYGDNVSYRKALARAIQSRQQVLAAEKGITGFGIRCLNPLVVEDRLVGIYEVGISLEEAAGEALSELQQGKYGILALEKQESSVLWQSSVLGVNPEATDLEKLEQGDSLSRKTSDNRVILTLVPIK
DVEGNTIAFVQGEIPRDTFIQAENRAKSRSLLIVIVSLLLVCVIAYLVLNRALRHLQPLRDSMVRVGEGDLTAVIDLSSKDEIGKLAHGFSDLLERFREVMYALFTKSSNLTTNAYFLNDVASSSVVKLGQTAEQLDYVGGQLQGAGQNLREADTGVEEIAGASSMVAEQAQNLQETYMKLSQAAQEGKEEMKRFEEVGQLLKERGESTVYSARELEKISRDIGEITSTIMSVSEQTNLLALNAAIESARA
GEHGRGFAVVAEEIRKLAEQTAGYTRQISSLISGVQGNIGNFVEIESMGTAIEENQTTSRVIARMEHIIRQIVNIQDAVMDISSAMQEQSAASQEISAVVSTVNDNMLLLISNLDQIIEDINQQMDNFSAIVNIAGETNDISQALRDILEQYRLPNKVVLRQVIDDHHGFVRKYQFIVEHNLFAEPDSVPTHMQCRLARWLQSVQDKETLELYQRLAANPHEQIHTLAREAIQANNQGHKDEARARIQ
AMIAASDKIIAALEQLIAFESNEGN*
>Syntrophomonas_wolfei_subsp.metylbutyratica_JCM14075T_01154_(Dimethylallyl)adenosine_tRNA_methylthiotransferase_MiaB
MGDKEKKPLKYRILTYGCQMNVRDSETIAGLLESSGFNQAEDLSEADLIVFNTCSVRHSAENKVYGKLGEIASLKKKRPEILIAFGGCMAQLPEVRQKLKKRGVDVVFGTHNIHELPYLIARAQEERSPVFEVWEKEGRIVEPLPSCRKPGLSAFVNIMFGCNNFCSYCIVPYTRGRERSRKADDIIRELEELAAAGYKEVTLLGQNVNSYGCGLEEKMEFADLLYRANSVAGIERIRFTTSHPKDVSDRLL
QAIAECEKLCEHIHAPLQAGSNRILQRMNRNYSREHYLKLVERMRHYVPGVSITSDLIVGFPGETEEDFLETLDMVERVRFDAAFTFMYSQRSGTRAAEFAEQIPLEEKKQRLERLNRLQYQIATEINQELQGSIQEVLVEGPSKTNPQKLTSRTRSNRIVIFSGEKDLIGRLINVKITEAKTFSLFGEIFNE*
>Syntrophomonas_wolfei_subsp.metylbutyratica_JCM14075T_01155_hypothetical_protein
LTSEEIIKMAFELGNAVAQSEEIEGLKASQVRVSQDHMAYDLIMSYQDAKSKMENKLQDGLSIGQAEQANLKNLEQELKSNSIINELMAAQEKFDNLMQAVYFAMNQALSGGCSSGCDSCGGSCNM*
>Syntrophomonas_wolfei_subsp.metylbutyratica_JCM14075T_01156_DNA_mismatch_repair_protein_MutS
LADYTPMMQQYLQIKEEQQDAILFFRLGDFYEMFFEDARIASRELEIVLTARDGGAGSKIPMCGVPYHSVDNYLARLINRGYKVAICEQVEDPREAKGIVKREVTRIVTPGTIIEEHLLDQAKNNFLAAVEEEPACTGIAYIDISTGEFWLSEIAGENARSRVESEILRISPAECLLAGSGSLTGSWEEEWLLRQNITLSVLDEPPLSLERAENLLLRQLQVASLESFGLKSYSAGIKAARIIAFLEETQKTSLQ
HIKSLRCYSSDNYLEMDFYSRRNLELTATLREGKREGSLLSILDESRTAMGKRLLRRWIEQPLREAGEIEERLDAVDELKNTLSLRTELSPLLSRINDLERLGGKIGASVASPRDLLGLKSSLAVINDIKKALQPCRSEILQRLAAMDALEEVFALIDASINDEAPLGIKEGELIKTGYKQEIDELRELSQEGTNWLVEFENREKQRTGIKNLKVGFNKVFGYYIEITKSNLSLAPADYHRKQTLVNSERFISDELK

[illegible]
>Syntrophomonas_wolfei_subsp.methylbutyratica_JCM14075T_01157_Acetophenone_carboxylase_gamma_subunit
[illegible]
>Syntrophomonas_wolfei_subsp.methylbutyratica_JCM14075T_01158_Histone_deacetylase-like_amidohydrolase
[illegible]
>Syntrophomonas_wolfei_subsp.methylbutyratica_JCM14075T_01159_DNA_mismatch_repair_protein_MutL
[illegible]
>Syntrophomonas_wolfei_subsp.methylbutyratica_JCM14075T_01160_putative_methyltransferase
[illegible]
>Syntrophomonas_wolfei_subsp.methylbutyratica_JCM14075T_01161_tRNA_dimethylallyltransferase
[illegible]
>Syntrophomonas_wolfei_subsp.methylbutyratica_JCM14075T_01162_Host_factor-I_protein
[illegible]
>Syntrophomonas_wolfei_subsp.methylbutyratica_JCM14075T_01163_hypothetical_protein
[illegible]
>Syntrophomonas_wolfei_subsp.methylbutyratica_JCM14075T_01164_hypothetical_protein
[illegible]
>Syntrophomonas_wolfei_subsp.methylbutyratica_JCM14075T_01165_5-methylcytosine-specific_restriction_enzyme_McrC
[illegible]
>Syntrophomonas_wolfei_subsp.methylbutyratica_JCM14075T_01166_5-methylcytosine-specific_restriction_enzyme_B
[illegible]
>Syntrophomonas_wolfei_subsp.methylbutyratica_JCM14075T_01167_5-methylcytosine-specific_restriction_enzyme_B
[illegible]
>Syntrophomonas_wolfei_subsp.methylbutyratica_JCM14075T_01168_nucleoside_triphosphate_pyrophosphohydrolase
[illegible]
>Syntrophomonas_wolfei_subsp.methylbutyratica_JCM14075T_01169_hypothetical_protein
[illegible]
>Syntrophomonas_wolfei_subsp.methylbutyratica_JCM14075T_01170_putative_ATPase_YjoB
[illegible]
>Syntrophomonas_wolfei_subsp.methylbutyratica_JCM14075T_01171_hypothetical_protein
[illegible]
>Syntrophomonas_wolfei_subsp.methylbutyratica_JCM14075T_01172_hypothetical_protein
[illegible]
>Syntrophomonas_wolfei_subsp.methylbutyratica_JCM14075T_01173_hypothetical_protein
[illegible]
>Syntrophomonas_wolfei_subsp.methylbutyratica_JCM14075T_01174_putative_phosphoesterase_or_phosphohydrolase
[illegible]
>Syntrophomonas_wolfei_subsp.methylbutyratica_JCM14075T_01175_Calcineurin-like_phosphoesterase
[illegible]
>Syntrophomonas_wolfei_subsp.methylbutyratica_JCM14075T_01176_hypothetical_protein
[illegible]
>Syntrophomonas_wolfei_subsp.methylbutyratica_JCM14075T_01177_hypothetical_protein
[illegible]
>Syntrophomonas_wolfei_subsp.methylbutyratica_JCM14075T_01178_phosphodiesterase
[illegible]
>Syntrophomonas_wolfei_subsp.methylbutyratica_JCM14075T_01179_Histidine--tRNA_ligase
[illegible]
>Syntrophomonas_wolfei_subsp.methylbutyratica_JCM14075T_01180_histidyl-tRNA_synthetase
[illegible]
>Syntrophomonas_wolfei_subsp.methylbutyratica_JCM14075T_01181_hypothetical_protein
[illegible]
>Syntrophomonas_wolfei_subsp.methylbutyratica_JCM14075T_01182_hypothetical_protein
[illegible]
>Syntrophomonas_wolfei_subsp.methylbutyratica_JCM14075T_01183_hypothetical_protein
[illegible]
>Syntrophomonas_wolfei_subsp.methylbutyratica_JCM14075T_01184_hypothetical_protein
[illegible]
>Syntrophomonas_wolfei_subsp.methylbutyratica_JCM14075T_01185_Histidine--tRNA_ligase
[illegible]
>Syntrophomonas_wolfei_subsp.methylbutyratica_JCM14075T_01186_Stage_V_sporulation_protein_K
[illegible]
>Syntrophomonas_wolfei_subsp.methylbutyratica_JCM14075T_01187_hypothetical_protein
[illegible]
>Syntrophomonas_wolfei_subsp.methylbutyratica_JCM14075T_01188_putative_addiction_module_component
[illegible]
>Syntrophomonas_wolfei_subsp.methylbutyratica_JCM14075T_01189_hypothetical_protein
[illegible]
>Syntrophomonas_wolfei_subsp.methylbutyratica_JCM14075T_01190_hypothetical_protein
[illegible]
>Syntrophomonas_wolfei_subsp.methylbutyratica_JCM14075T_01191_hypothetical_protein
[illegible]
>Syntrophomonas_wolfei_subsp.methylbutyratica_JCM14075T_01192_type_II_restriction_m6_adenine_DNA_methyltransferase,_Alw26I/Eco31I/Esp3I_family
[illegible]
>Syntrophomonas_wolfei_subsp.methylbutyratica_JCM14075T_01193_PglZ_domain_protein
[illegible]
>Syntrophomonas_wolfei_subsp.methylbutyratica_JCM14075T_01194_Lon_protease
[illegible]
>Syntrophomonas_wolfei_subsp.methylbutyratica_JCM14075T_01195_AP-4-A_phosphorylase
[illegible]
>Syntrophomonas_wolfei_subsp.methylbutyratica_JCM14075T_01196_UvsW_helicase
[illegible]
>Syntrophomonas_wolfei_subsp.methylbutyratica_JCM14075T_01197_hypothetical_protein
[illegible]
>Syntrophomonas_wolfei_subsp.methylbutyratica_JCM14075T_01198_hypothetical_protein
[illegible]
>Syntrophomonas_wolfei_subsp.methylbutyratica_JCM14075T_01199_putative_small_protein
[illegible]
>Syntrophomonas_wolfei_subsp.methylbutyratica_JCM14075T_01200_hypothetical_protein
[illegible]
>Syntrophomonas_wolfei_subsp.methylbutyratica_JCM14075T_01201_hypothetical_protein
[illegible]
>Syntrophomonas_wolfei_subsp.methylbutyratica_JCM14075T_01202_CRISPR_type_III-A/MTUBE-associated_RAMP_protein_Csm4
[illegible]
>Syntrophomonas_wolfei_subsp.methylbutyratica_JCM14075T_01203_CRISPR_type_III-A/MTUBE-associated_RAMP_protein_Csm5
[illegible]
>Syntrophomonas_wolfei_subsp.methylbutyratica_JCM14075T_01204_hypothetical_protein
[illegible]
>Syntrophomonas_wolfei_subsp.methylbutyratica_JCM14075T_01205_hypothetical_protein
[illegible]
>Syntrophomonas_wolfei_subsp.methylbutyratica_JCM14075T_01206_CRISPR-associated_protein
[illegible]
>Syntrophomonas_wolfei_subsp.methylbutyratica_JCM14075T_01207_hypothetical_protein
[illegible]
>Syntrophomonas_wolfei_subsp.methylbutyratica_JCM14075T_01208_hypothetical_protein
[illegible]
>Syntrophomonas_wolfei_subsp.methylbutyratica_JCM14075T_01209_Cystathionine_gamma-synthase
[illegible]
>Syntrophomonas_wolfei_subsp.methylbutyratica_JCM14075T_01210_hypothetical_protein
[illegible]
>Syntrophomonas_wolfei_subsp.methylbutyratica_JCM14075T_01211_Glutamine_synthetase
[illegible]
>Syntrophomonas_wolfei_subsp.methylbutyratica_JCM14075T_01212_LexA_repressor
[illegible]
>Syntrophomonas_wolfei_subsp.methylbutyratica_JCM14075T_01213_tRNA_2-thiocytidine_biosynthesis_protein_TtcA
[illegible]
>Syntrophomonas_wolfei_subsp.methylbutyratica_JCM14075T_01214_hypothetical_protein
[illegible]
>Syntrophomonas_wolfei_subsp.methylbutyratica_JCM14075T_01215_Lactate_utilization_protein_A
[illegible]
>Syntrophomonas_wolfei_subsp.methylbutyratica_JCM14075T_01216_putative_FAD-linked_oxidoreductase
[illegible]
>Syntrophomonas_wolfei_subsp.methylbutyratica_JCM14075T_01217_hypothetical_protein
[illegible]
>Syntrophomonas_wolfei_subsp.methylbutyratica_JCM14075T_01218_Succinyl-CoA:coenzyme_A_transferase
[illegible]
>Syntrophomonas_wolfei_subsp.methylbutyratica_JCM14075T_01219_UDP-N-acetylenolpyruvoylglucosamine_reductase
[illegible]
>Syntrophomonas_wolfei_subsp.methylbutyratica_JCM14075T_01220_(dimethylallyl)adenosine_tRNA_methylthiotransferase
[illegible]
>Syntrophomonas_wolfei_subsp.methylbutyratica_JCM14075T_01221_NADP-reducing_hydrogenase_subunit_HndC
[illegible]
>Syntrophomonas_wolfei_subsp.methylbutyratica_JCM14075T_01222_NADP-reducing_hydrogenase_subunit_HndC
[illegible]
>Syntrophomonas_wolfei_subsp.methylbutyratica_JCM14075T_01223_NADP-reducing_hydrogenase_subunit_HndA
[illegible]
>Syntrophomonas_wolfei_subsp.methylbutyratica_JCM14075T_01224_Biotin_synthase
[illegible]
>Syntrophomonas_wolfei_subsp.methylbutyratica_JCM14075T_01225_hypothetical_protein
[illegible]
>Syntrophomonas_wolfei_subsp.methylbutyratica_JCM14075T_01226_2-iminoacetate_synthase
[illegible]
>Syntrophomonas_wolfei_subsp.methylbutyratica_JCM14075T_01227_putative_iron-only_hydrogenase_system_regulator
[illegible]
>Syntrophomonas_wolfei_subsp.methylbutyratica_JCM14075T_01228_NADP-reducing_hydrogenase_subunit_HndC
[illegible]
>Syntrophomonas_wolfei_subsp.methylbutyratica_JCM14075T_01229_NADP-reducing_hydrogenase_subunit_HndA
[illegible]

>Syntrophomonas_wolfei_subsp.metylbutyratica_JCM14075T_01230_Molybdopterin_molybdenumtransferase
MLNSLEEAQQELIKNIEPLVAEPVSLLEAVGRISSTGILADCNMPDELRSAVDGYAVNPDLSGNYDQLLVVGQLTMGEVPNSPLVPGQAVGVLTGGLVPDGAVAVIPHEKVQIKDNYLKSLEFVKPGNNFKQPGEDFHKGDLILGQAARITPGLVALLATFGHGQVQVYRRPRVAILSLGSNVVPCSSQAARGEIRDANGPMLASLVKLDGGLISSVELAGQKDMTQIKIQLLQMLEQSELVITTG
GTYTDGINEIHVLIDEIGARPLFREIPIQPGSHNGGAILGSRLIISLSGNPAACAVGYHLFAAPVLRALQGIEPLLPRVNAICSSSYAKKTGSRRFIRALASPREDGWTVSILPGQKPSMIRSLVDCNALIDLPPGHPPMEAGSEVSILLLDRSAV*
>Syntrophomonas_wolfei_subsp.metylbutyratica_JCM14075T_01231_bifunctional_molybdenum_cofactor_biosynthesis_protein_MoaC/MogA
MRKVPVNEAIGQVLGHDIIKIIPGREKCRAFRRGQIISAADVPLLQNLGKEHIYIWEAGDELIHEDEAALRLAQSASGPGINLSEPSHGRVNLMAAYDGLLKIQVDQLHWINNLDSVIFATLHNNRTVVKEQKLAGTRVVPLAVEHSLVEEAEKLCSNPRPLISVKPFKNLWTAVITTGNEVCYGRIKDGFGRVIRKKITPFGGRFMSQTIVPDSPEIIAAEIKRFIGEGADFIITTGGMSVDPDDVTPLGIKA
SGAEVVFYGAPVLPGSQFMLAYQGHVPICGVPGGALYSKFTTLDLLLPRIFAEERINRSEIVALGHGGFCEECKVCHFPACPFGKATYL*
>Syntrophomonas_wolfei_subsp.metylbutyratica_JCM14075T_01232_Formate_dehydrogenase_H
MTNAISEITTDAELLFLIGTNTTEAHPVTGYKMRQAARRGCKLVVCDPRHIDLVDEADYWLRQKPGTDIPLINGLMHIIKEGLEDKKFIEERTENYEALKATVENYPPEYVAELTGIPVDVLYEVARLYATTDRAMIFYTLGITEHICGTRNVMSLANLAMLTGHMGRAGVGVCPIRGQNNVQGACDMGALPNVYQGYQNVTLPAVKEKFEKAWGRPMPDKIGLKIPEMFEAAHEGKVKAMYI
LGENPVLTDPNSHHIRGGLEALEFLVVQELFLTETAEYADVILPAASFAECDGTFSNTERRVQRVRKAIEPIPGRANWQTICEMVSRMGYPMNYASPREIWDEMASLAPSMAGINYDRLEELGSLQWPCPTTDHPGTQFMHVGKFTRGLGLFQPSDHIPPGEMPDEDYPFLLSTGRILQHYNVTTPYSMGIMSVWDKEMAELNPADAEKMGIKTGDKLKVTSRRGEVVTAVRVTDRVMPGIIW
MSFHYSETPTNALTSHHLDPITGTGEYKVCAVKLEKI*
>Syntrophomonas_wolfei_subsp.metylbutyratica_JCM14075T_01233_Putative_formate_dehydrogenase
VELIKITIDGKEVEVPAGTTIMKAADSVGINIPRLCYDPDLSALGACRLCVVEVEGNRLLPASCVTPIFPGMVVKTNSPAVVEARKTILELLVANHPLDCMTCDKLGNCKLADYCYQYGVKESPFVGEKHEYAIDDTNPFILRDMNKCILCGACVRACEEMTGQDNLSYLNRGFHRKATTAGDVDYIDSDCVFCGQCVAVCPTGALQEKTMVGQGRRWEIDRVRTTCPFCGTGCNFDLAVKNG
KVIGVLSNPDSTVNQRSLCVKGRFGWDFIYNEKRLTTPLIKKNGKFEEASWDEAFDLIAQRFGEIKAQYGSESFAALSSARCTNEENYLVQKFTRAVMGNNNVDHCART*
>Syntrophomonas_wolfei_subsp.metylbutyratica_JCM14075T_01236_formate_dehydrogenase_accessory_protein
LSDALLFEEKDILIYRQGEVRPYRDTLVIEVPLTVYANGQELSTLVCSPQGYKELGVGYLLNEGLISDPADIQDIHHDSNKGILFIEGKIDTPSNGYGHKRYFPEDNETLNSSFYFAKDACQIKPVKSDFRIPASQLLQLISLLEEKSLTFRRTGGVHSAALADNSGLIVRYEDIGRHNAVDKVMGYAFLNGISLDDKCLVLSGRVASEIMFKVARRGIPLILSRSAAMLLAVELAEKLGICIAGFTRGEQFNVY
CHPERIIF*
>Syntrophomonas_wolfei_subsp.metylbutyratica_JCM14075T_01237_CDP-diacylglycerol-serine_O-phosphatidyltransferase
MLLDLITKSFANVLTLMNLVFGSMAIILVQHLHYSWAAIFILFAVFMDGMDGKIARRFGATSELGKELDSLCDLVSFGVAPAVLLYAQVFLESYHLPGLIAALFFIICGALRLARFNVLNIHEYFLGIPITLAGFLLAIISLLADNIYPMVILLLVVFLSLMMISNIKIRKI*
>Syntrophomonas_wolfei_subsp.metylbutyratica_JCM14075T_01238_putative_NUDIX_hydrolase
MMDKILLEEKLKLMANRRPQILGHKEFLKTAVLLPLAEYQNDLCILFEKRAADLNVQPGEICFPGGQIEAIDQGAKEAAIRETCEELGLNTGDIEVVAPLDIFVSPFNLIVNPFVGKIKDYQKIKINSEVEYVFYVPLNYLLKIEPPCAPLGLKLVLPQGYPYDLIPHGRNYPYRDARYPQYFYLWEKEVIWGLTARILNHFLDLLKA*
>Syntrophomonas_wolfei_subsp.metylbutyratica_JCM14075T_01239_NAD-dependent_protein_deacetylase
MEKSIDRVVEILDRSHNTVVVTGAGISTEAGIPDFRGPEGIYRKLGENRVMNIINIDFFRNNPLEFYKFYRQYFIFPPVEPGKTHKVLAEMEKTGIVKAIVTQNIDNLHQKAGSQKVIPIHGNGARFICQERNCRFVHDSNYVNTYPEVIPRCSQCGGILKPDVVLFGEHIKNYPDAMDRILGARVLVVIGSSLTVYPLAGFVKEFSTFTQYLIIINKGPTPLDHAAVVKLQDGNTGELLEEIFEKIKKRRSS*
>Syntrophomonas_wolfei_subsp.metylbutyratica_JCM14075T_01240_NlpC/P60_family_protein
MLKKLFALIMIGCFTLSFPVNPLDVTWAAPIIRAGPDEIDYSEYMLLLDNPRANIPPEEIEKSKPPSSSKATENLSGQAAKPIQPAESKTTTNNKYVSIYKNNYPAYAKEELPIVEEFRSNYNGTLAHAIVGRAIWYMEYGYMIYGHEKYPKSGLIDCSNFVSLVYKDFGYTITSAARRYDTVGNKVSGVYSQKLSSSSSKYKLVGIDKLRPGDIMTFWKKDSQGKKYIAHVAIYMGKINGKPTIIHTRKDNPT
AIGITNSFTYWFGEHFYEARRVLNSTERSSKSFSDSGPVIPQVYKLPPQGPIIQPEDLPGGF*
>Syntrophomonas_wolfei_subsp.metylbutyratica_JCM14075T_01241_Papain_family_cysteine_protease
MKKLKKRRKIKLFKARLAKRNPIFEQQRNKISPTSSKKVLTGQYQPGFIPHPLNLSHLKGQKIFPETRLLRLSSSYDLRKEGRLTPVKDQGPAGTCWAFATYGSLESCLLPDQEFDFSENNMKNLLSENCPAGYDRGPRDGGNQFMSMAYLNRWDGPVLEKEDPYDPFDDGPCTQFSPQKHIQNVIFIPDRKDAADNQNIKEAISKYGAVFSGMYYEDQYYNPDKSSYYFPSYSFSNHAVCLVGWD
DNYDRKNFNPEPPKNGAFIVRNSWGTSWGDDGYFYISYYDTGIGSDNAVYYNAESPTKYNIIYQYDPLGWIASVGYRGSTAWFANIFTASSKQQELLAVGWYVGGVSATYQVLVYTDPAANNPRNGKIAKNVSGTITYPGYFTRSFTFKPVALKAGQKFSVVVKLSTPGYNFPIPLEMPVDGYTSKAVANKGQSFMSKSGTNWEDITNSWENSNACLKAYVANKSSDA*
>Syntrophomonas_wolfei_subsp.metylbutyratica_JCM14075T_01242_hypothetical_protein
MNRLARYFLNGLLFIVPIFLTIYIIYFMFTKIDRLLQIPLPGIGIIPGVGFVVTILIITLTGVLVSNLITRRFMSLMDRLFNRLPLVKILYSSIKDLINAFLGEKKTFNQPVLVTIIPDSNASALGFVTSESLQHLGVDDMVAVYFPQSYNFAGNLLLFPRKQVTPIDASSSEVMTFIVSGGVAKS*
>Syntrophomonas_wolfei_subsp.metylbutyratica_JCM14075T_01243_Sensor_protein_FixL
MNSFIEVIGIKEENCINCHQCIAVCPVKICSDGSGDVVKFHNHLCIGCGRCIEACIKSHGGNVEKSARFPIDDADDFLNDLDSSEVIALVAPSAQANFNLKKLISALKLLGVKAVYDVSLGAEISYISYHEAIESGKAKLPLIAQPCPAIVKYIELNHPALIPYLAPVGSPVHNIAVYAKTLHPQAKLAFISPCLAKKGEFHDSQSVEYNVIFQSLTRILAEKDINIDLLEESDFDNYISAGIATNFSTPGGLKESYLFH
YPETLPSSIKIEGSLVFEKYLKDLETAIKEGQADLPLIIDILACEKGCNIGAGCINQGRSITQIERAVNRRMEISSSEAEKTKELKEFLGKITQDLDFSYIFYQDLSANSDIIIPSELQLQQVYKRMYKEEAKDFRNCLACGYNSCLQMAVAVFNGLNKAENCHLYQEKELLREQEILNNMVDELATLNSKLEEEMAERKQQEQLLVQNSKLAAMGEMIGMIAHQWRQPLSSISTLAGNLKIYLDLDMYEKK
QFLSLLEEINHHAQYLSSTINDFRHFFKPDNPEDTVYLNVVIDNTLGIIGKSLEYKNVQLNINTSFSQPILTYPNELMQVFLNILKNASDAFVDNETSYPRIQINGYEKNNYQIVEIIDNAGGIPERILNKIFDPYFSTKGPGIGTGLGLYMSKTIVEEHCEGELIARNTQEGACFTLKFPLR*
>Syntrophomonas_wolfei_subsp.metylbutyratica_JCM14075T_01244_Cyclic_di-GMP_phosphodiesterase_response_regulator_RpfG
MTIDVKELKERAQGIRVLYVEDDKELRENTARLLATFFSSIDTASNGQEGLEKYNGNCYDLIITDINMPIMNGVKMAAQIKAGNKKEIIIVISAHDEARYLLELINLGVDYFVLKPLDINQFLTALDKAVSLVHFYKMEADYKRALEETVEKRTRELSEALTIVQELTTEVVYRLTAAAELRDSDTGLHNKRLGLYARRLAETLKLPADFVESIAFAAPLHDIGKIGIWDNILLKPGALDAEEFEIMKTHTTT
GASILARSKYEKIRMTESIALTHHERWDGSGYPNGLKGEEIPLEGRIVAICDQYDALRSKRPYKPAFSHFRTMQIITKGDERTPREYFDPKVLQAFIEIADEFDGIFTSNQD*
>Syntrophomonas_wolfei_subsp.metylbutyratica_JCM14075T_01246_Endo-1,4-beta-xylanase_A_precursor
VIRRFNSPLLILLSIICLCCSTLFFPASAEATVDLDTLAKKAVQYNYSSYRQGQALDGFDAWVLGKAGAQVSGWVYDGSNIQTTLLATMEEILTDPDKKTADYFGNPAYSYSSKQIAQLNLLARSWGEDEKADALLRVLQNRQNTTLNGSIDNNPFGDISAFEYLALSGDLTRMNSSSAIDYIINNSDPNTGAWTTSSWNDVMATTQAIRSLKCLEASTGEKTAEINAAINKALNWLSALQKDDGSFQ
DSGGFDDPLIDSVEVLLTLKTLDIDPASWSSQEGKTILDYLHNEALNTDGSLGTSKNKTDATWVLNACLSLGVQIPADTPLGLSITPATASVEKNGTIAFSALLKLFNGEQNDISRSVNWLVADNNIASIDGGLARGLYEGQTTVTATSNDYEASASLTVRPSGGGEGESKSSDLVVNIAVVGKNGTLLFGPSSISISTGDRFGLTAMGALNKTGLRWSFSDRWEGLIVEIAGEQNQGMSGWMYSV
NARVPGVLASEQTVSDGDRIIFWYSQDASASSPEWDRLGSGQIYNPEVKEKLPDKQKVLPEIKPELDKQVPTPAARGFFSDVDDSLAWARDAIEILAGRGIIQGRGEHFDPLAPVTRAEFITLIARALGKNTVDYQGIMPQDVSQSDWFYPSLQYCLQEGIISGYPDGSFRAGDSISRQEMACILYRLMKDKQNNNLPASILGDQDSIAPWAIPAVKFVVAEKLMLGYEDGSFRAGQPCSRAEAAVILFR
YLHSIEAAPLK*
>Syntrophomonas_wolfei_subsp.metylbutyratica_JCM14075T_01247_hypothetical_protein
MKRNFSIYLLLFLLLFLIAGCHSSDGINKQGESSGSSSYKASVEPGQEKLKADDTGSAASTSVKGKGQGNIAPAPLEVSSSTLQETPEAMGGNKTPVLPEPGPADNKAQLRLLVTQDNGEEVIFDQEVELAQDATVMDILKAHLEVSSSYGGEFVEGINGLKSKRAGLGQEPEDWFYYINGTSANVGAASYRPAPGDVVWWDYHSWSSGGR*
>Syntrophomonas_wolfei_subsp.metylbutyratica_JCM14075T_01248_Energy-coupling_factor_transporter_transmembrane_protein_EcfT
MNETMLSYRVKDNLIHSLHPLTTLSYVLVTILSLIFYHPIYLAALLLPAALVIVSSGHYKEWSIYLKLSVSLIILIAVLNVMFSREGETILFTLQLLPGMAPLDLSLEALAFAAGMGLRLLVIITAFCLYSQAIDPDRILRAVAGLGKGKTVLAIILATRLFPQFLRDYRRIMEISQCRGVDPYHGNLWQRTGRLKPVISVLFSSSLEQALELAESMYARGYGSGERSQRQADLWHPRDFLILALIIAFFCGILAYG
QGWTAFNYYPRLSWEFSGSSILTLMTLSLAIPSVLNWGCKRSNSFLSKI*
>Syntrophomonas_wolfei_subsp.metylbutyratica_JCM14075T_01249_Putative_HMP/thiamine_import_ATP-binding_protein_YkoD
LSYSYPDEKEPALKKINLTLNEGEFVLLSGVSGCGKTTLLRAIAGLVPDFHGGHLAGAIYLDRIPLTRIPHRQRLLKTGIVFQEAERQLLMNTVEAEIAFALENLGFPPALIKRRIMEVAAALDLTDCLPQATATLSGGQKQKVVLASILAWEPEILLLDEPTSQLDPVAAEQILNSIRRLNEDSGLTVILAEQRLERCFHLADRVLVMKNGAIACDEQSFDKLSHWAVKNETSCLPPVARLFAGCAFSKIP
ASIKEGRGVLRSLAPQGDFSPSFSAASPGLGNKLDAERSFEQPVVEVEKVWFRYPEGKEVLKDISFNLKAGEFAVLMGPNGAGKSTILKNLNGLLRPGRGKIKICGRDSKGVPVAELANSIAYLSQNPDDYLFLPTVREEVKFSLQYLAGKDDIDSRSDQVLQRFGLCRLAQLNPRDLSSGERQKVALAAVLASEPRVLLLDEPTRGLDYRHKEELGRILLEMQAEGTAILMVSHDVEFVAEYAAKVIFIT
DGSLLCSGSKYDMLSSSTFYSPQVTKVFRGIAQGIVTLEQAQELLQRILYHRQEAIPRLAGNAGTGEK*
>Syntrophomonas_wolfei_subsp.metylbutyratica_JCM14075T_01250_putative_membrane_protein
MQLRISLISLGLLGLLLLLLSIWPGRSLGNWNWGLLAATILTVALAGFFWQFERRRNSIVQLSFIATISSLAALSRVAFVPIAGLQPASFIIMISGYVFGSQTGFLVGAITALVSNFFLGQGPWTPWQMLAWGICGLLAGCLGQALSAFRLLPFVFLCTFSAFLFGWITNISYWSSFVHPLNWSSYLGSCIASFPFDLIHAAGNLAFSLLFGTHFYQLLFRFQRKMSGYSK*
>Syntrophomonas_wolfei_subsp.metylbutyratica_JCM14075T_01251_Formate_acetyltransferase
LQAAWSGFKAGRWMHEIDVRDFIQKNYSPYDGDSGFLTGPGEKTSLLWRKCQDLLQKERESQGVLLVDADTPISITSHPPGYINRELEKIVGLQTDKALKRAINIYGGLRTTLNACRAYGYEPDKKLREFFEKHRRTHNDGVFAVYTPEMRLARKVGIITGLPDSYGRGRIIGDYRRVSLYGVDFLIKAKENDRDWLNRRMTTESIQLREDMAGQIKSLQDLKLMAKSYDYDISRPASTAAETVQHL
YFAYLAAVKEQNGAAMSLGRVSTFLDIYLERDLRSGRITENEAQELIDQLVIKLRLVRHLRTLEYSEIFAGDPNWVTESLGGSGTDGRHLVCKTSYRFLQTLKNLGAAPEPNMTILWSRNLPRKFLEFCAKLSISTSALQYENDDLMRDYYGDDYGIACCVSAMRLGKETQLFGARCNLAKLLLYAINGGKDEISGQQVGVEMSVYDGDKLEFDEVMRRFKIQMEWLAELYVNTMNLIHYMHDKY
AFEYMQMALHNTFVKHFMAFGIAGLSVAADSLSAIKYARVRPIKDERGLVSDFAIEGEFPKFGNDDDRVDLLAVEIIHTFIHQLKRHPAYRNAQHTLSILTITSNVMYGKHTGTTPDGRKKGEPLAPGANPLHGREEKGAIAACNSVAKLPYEDARDGISFTFSTVPEALGKSEEERVNNLLALLEGYFVQDAHHMNVNVLNVETLKEAMEHPEKYPDLTIRVSGYAVHFTRLNREQQEEIIMRTFYR
RM*
>Syntrophomonas_wolfei_subsp.metylbutyratica_JCM14075T_01252_Pyruvate_formate-lyase_1-activating_enzyme
MIGRIHSIDTFSTLDGPGIRTVVFMQGCHLRCKYCHNPDTWELKSLSAQEYSPEELMEVIRRSKPYFIASGGGLTFSGGEPLLHDDFIKAVFLLCREENISTAIDTSLYVKPAALLTVLPLTDLVLADIKHISEEKSRCLTGMSNSLNLSNLKLINSHHIPIWIRYVIIPAWTDAREDLEEMAAFVGRLEQVERIDLLPYHSLGKHKWDLLGLNYELNGVTAPSPEALEQLKNIISAISGKPVYLK*
>Syntrophomonas_wolfei_subsp.metylbutyratica_JCM14075T_01253_DegV_domain-containing_protein
VSVKIFTDSTSYISKEMQEELDIEIIPLTVHFSDESFIETEVDYDYFYQKIEKTGEIPTSSQPPPGQFFAAFKKALSHGDHIVGIFISSTMSGTWATALNVKKQLMEQYPGARMEIIDSHTNCMALGLQVVEAARVAKAGGNIDAVVKAAQYIHDRVHFYFVPETLEYLRKGGRIGTASALLGTLLNIRPILTVDMAKGMTHLLEKARGTSNAVKRMLRLMEEDHKKYGLKEIMVHHINAPERAWQLRQ
TLIDRYALPVSILSIGPVIGLHTGLGTIGMVYVA*
>Syntrophomonas_wolfei_subsp.metylbutyratica_JCM14075T_01254_putative_cation_efflux_system_proteinc/MT2084
LDKFTSSISDFLISKTLKDTYAETNIENPEQRRKIAYLEAWVSIIGNIVLAIIKFIMGILVNSISLIADAVHTASDVLTSLVVIIGFKLAALPADEEHPHGHGRIEFIATLVISLLLVAVGIKFGFDSYKRLMANTPVAGSYFVVAVMLVAGLFKEWMSRFSIDLGQRISSSTLIADAWHHRTDAIASVLVAIAILASKFGYYWVDAVLGFAVSALIVWTGIEIFRESCSKIIGEYDEEQVKRVNQLALKVPGVIAVHDTS
VHDYGANKVVSLHIEVDQGLSVVKAHAIADVVEVSINQYLYTTSTTVHVDWIDNEAQN*
>Syntrophomonas_wolfei_subsp.metylbutyratica_JCM14075T_01255_Spore_germination_protein
MKLLRKAPALILILLLLWLPSGCGQQTGKVPGDKDHKEKPAPSTPVAKTADVAVYYLKMTNDDAYLVREVHQIKKSSNLPQLALNELIKGKPLTPGANRVLPPDTKVLGIKIDKGLATVNFSPEVLKANVGASGEALGIASIVNTLTEFPDIQKVSFMVDGQVEKAIDWWGHIGLSEQPFSRNLAVVYEPVIWVSSPTPGETISSPVEISGSARVFEAMVSIRIKDAQGKTLVQDYTAASEGAPGRGIFQ
KEIAFKAPGPGQGQIEVFWLSMKDGSEQDKVIIPVKWK*
>Syntrophomonas_wolfei_subsp.metylbutyratica_JCM14075T_01256_helix-turn-helix_protein
MLNKGKFSKRLKELRKMQGISTRSLAKAIGLKSHAAITQFEKGTSMPALDTLVALAGFFEVTLDYLVGMTDFPQAIPDKTKKLLEQTENPEACITILNQISNEKFQELQKRTESPQIIAEMIMGLDEKSMLELYMFLRYLHIRQALVETDAISADRI*
>Syntrophomonas_wolfei_subsp.metylbutyratica_JCM14075T_01257_Cyclic_di-GMP_phosphodiesterase_response_regulator_RpfG
MDRTWQPLKLLKRIHYDTYLHSLRVAMLAYNIGKEIALSTSELDLLHMSALLHDIGKVKLGNSILDKPGKLNPEEWNEIERHPLYGANILVHDYEIMSNDVIEGVYSHHESFNGDGYPRGLKGESISIYGRVIAIADALDAMTTRRIYKTHTLGFNEAINEVFLCEGTQFDPYICKKVKGLSHV*
>Syntrophomonas_wolfei_subsp.metylbutyratica_JCM14075T_01258_Sporulation_kinase_E
MNYKNMTKKQLVDELAKLRQKNMELELILKDKECLSTAPESSSPNRWFSTGRAELFVCKSSEQEEIDNSKKKYKFSDLVDIPLLQQFLKSLYEATGLPHALVDIDNNILSGIGWQDICSRFHRACPQSSCRCKESDSYISAHLHDGPYVGYKCLNGLMDYATPIIVEGEHLATVFIGQLLHQPPDEEYFSSQAREYGFDENAYLEALCRVPVIPEEQIETIMKFYIQLGQFLASIGLNRKRQIELAKQAIRK
QEERFRLLWETSNDGFWEWNIEAGKVYYSPRWAEMLGYSFEDCEVDIQNWEKRLHPDDISTAMEAINEHLQERTTVYAAEYRIITESGEEKWILDHGQLVARNAEGQPLTMVGASIDITAFKKVEASLFESQQKFATVFYSSPDIMTISTIKDGRYVEVNDAFVAITGYERNEVIGRCVFDLNIWVVPEERDAVIKQIQENGRVRDFEIELRSKYGETGISLLSGEIVDLGGEEYLITTNRDITERKQMEE
ALRLSEECLFKAFNTSPIIMAITTLEEGMFIKANKAFACIVGYDHEEVIGRTSLEIGFWLNPLERDLLKQSIVAGQSVRDMEIVFGNIYGEQKLGLLSAEGLDIDGKPCLLSVLTDITELRRMEVEMTRLDRLNLVGEMAASIGHEIRNPMTTVRGYLQIMRENKDNIQELEYFNLMIEELDRANAIITDFLSLAKNKMVDMKPGDLNAIINKLLPLIQAKALSRDQYIKLELGEIPYLLLDKNEVHQLILNL
VNNGLESMSLPGYVTIKTYMEDEAVVLAVQDQGQGIDPSLLDKLGTPFLTTKDNGTGLGLAVCYRIAEQHNAKINVETSINGSTFYLKFPGETGEEPMV*
>Syntrophomonas_wolfei_subsp.metylbutyratica_JCM14075T_01259_CRISPR-associated_endoribonuclease_Cas6
LRITLEISSEKEIKLPIHYNHILQGFLYKNLSNKDYREFLHQKGYSLGNKSFKLFTFSRLLGEFRIVPQKKEIIFTPPVKIIVAAGVEQFITDLAETLIKSELNFLGNQPVEVKSIGVHKNFTPSNLLQIKMLSPIVAYSTLTESEKKWTEYYSPWQPRFAELVKANLEAKYQVLYGEIPSQEELKIIPHGSQEQKFKSVIKYKGTVIVGYLGLYWLKGNPDLIKIAYDTGLGSKNAQGFGCWEVVG*
>Syntrophomonas_wolfei_subsp.metylbutyratica_JCM14075T_01260_CRISPR-associated_protein,_family
MLESIIGLGAAVLEEQDALASLIKKVPSIKNNRQQHICKFVFHTKKKELRIDVKEEMDDNSSLRYLHVGSVDGPRSAQWFATSTASSYILSELFPNLLEMQGIIEEDIIRKSRSIVEQFYYPIDPELAGNKKYRYILDIEKLGMGDISLDEIIEEASKSNKPGKKILELLQKQLFKWIQQEYGVGERQLGLYTVVIDGEALVNDPSYRQAVSDSKKPQTARQHEQMELECNVCGASEGVPADLSKMKIKYFTTN
QLIFASQLKDYTKNLNICSSCMEKLQAGEVFLQEQLSTRIAGFTVYIIPHLVYGESVDPSTMKNLAQKLLLTVNMAKSIDALEDFRNRLEELKENIYYLIDFAFVRSSNAATKILRLVKDVRPSFFENLDVSVAQLQELANKHFKNNYHYRGGLEQVYYLTPIRLKQGNASSFRELLALYDALFTNRSIDKKRIIKNLAEALAICFYEKEGFNIRQKQQYINYLTLDGVFLLEFFKRMGLCKGGEGMDYRT
LNLNDSIKGFMADMHYDEEQAALFLLGYLLGQVGNAQYRRASEKGADTTYKPVLNKINFNGIDKSKIIRLRSDVFNKLRQEKVLVYNERIFAECTRLMDKNINDWKMNKDENLFYVLSGYAFGTTLPVKKKGDNSDEQ*
>Syntrophomonas_wolfei_subsp.metylbutyratica_JCM14075T_01261_CRISPR-associated_protein_Cas7/Csh2,_subtype_I-B/HMARI
MNNSEILYLYDAQLTNPNGDPDEENRPRMDYERGINLVSDLRLKRYIRDYLLDKNYELYVQSIDNKPVTAAERVQQIRDTSDENLLNSFIDIRLFGATVPATGDSKSFIGPVQFNWGYSLNKVELLESSITSHFASSSTNRQGAIGKDYRVKYSFIAFSGVVSGKRAEYSRLSESDLELLDEAMKKAIPLLATRSKIGQYPRFYMRVVFNDNETILDDFRNYIQLLPADENLRSINECALECGRLRAYLEARK
GLIKEIRMFQSNELRMLDNGVEKTASDIFSAFTNIPV*
>Syntrophomonas_wolfei_subsp.metylbutyratica_JCM14075T_01262_CRISPR-associated_protein_Cas5,_subtype_I-B/HMARI
MRVLVFDLKGSLAHFRKIYTNSSSLSYSLPPRTCLAGIIAAMLGRERDSYYQEFSREHSFIAVKKLGRTRKIMQTLNYMRATGSRELIVPREHTQIPFELVCGEPELRYRIYFAGNPGAYEELKQRLVNDKYVFPPYLGAAPFSATANFVAEASCQRLQTRGLVEVSTPIKSRNIVEFSFDRMLDNVALVKEKMPVDFTAERYPVASASYVYDENASALPVSLNEDMCWRIAYQWEEQDIKENITFY*
>Syntrophomonas_wolfei_subsp.metylbutyratica_JCM14075T_01263_CRISPR-associated_nuclease/helicase_Cas3
MQFYSHPDKLLSKHLQEVSTMAAAFCPQRYGEAARIAGIAHDFGKFTTFFQEYMLKKKQQGKLKQHGFISALFGAHNAFHRWGEDHFFPLAIYSSIYHHHGNLSDFKEILPSSMKNFGAAFEENVDIARRQVENIETNLKEVRKAYRDMLDEQLINGFIKGFNPHELLRKLLKLEYYYTVRGKYKRDTSYFAHQFIYSSLIAADKLSASGENPTSIRFIQSPGILLARDKIVQSRMEKNNKDSQGEINSIRE
DIFRRVQTNMELFADTQRIFTITSPTGSGKTWTGFCAATKLRDRLGGNRKIVYVLPFTSIIDQNYKSIQELFHNSGEITDKERFYLLKHHHLSGQVKEENEEGYDPASSPVFEQLLTEDWQSAVIVTTFVQFFESMISVRNRMLKKFHNYAGSIFLIDEIQALDIKYYPLVEYVLNILCREYDSRVILMTATRPVIFGSSPNDRLPAMELLENYPDYYSRFNRTCLIPKLDKIGLEDFVQEIAALIKDETSCMV
VCNTIKSSLRVFSLLKKIFGEKRKVYYLSTNLVPAQRADRIDKIKAADLEDGENLILVSTQVVEAGVDLDFDIVVRDFAPLDSIIQCAGRCNRGGIMFSYNRGIVYLFNVVNEKGDDYASFIYGTSNLILTKELLRGRDRIEEREYLSLIEAFYNMIKKEISTQPAHDLIAALENFNMSRDGVSGFSLIKNNSGYIDVLFRYNSRVEEAYQRFWEVIGIKDFQEKRNEYLKIKTILQKYTISLPAQFTDVFEKRPLGES
NVFISLPPEGMADYYDEDTGFKRERNDSFLAF*
>Syntrophomonas_wolfei_subsp.metylbutyratica_JCM14075T_01265_Group_II_intron-encoded_protein_LtrA
MENAYERIAQQVEKHKRAQTLMHYVNKQNLTEEHRQQHKGKATGIDRVGKEAYGENLEANIEDLLTRMKMFSYRPQAVRRTYIPKAGSDKMRPLGIPAYEDKLVQGAMRKVLDQVYEGKFYDFSYGFRKGKNCHQAIREVNQLIMTNKINFIVDADIKGFFDNVDHEWLMRFLEHDIADKNFLRYIKRFLKAGIMEDMKYHESDRGTPQGGLISPVCANVYLHYVLDMWFDKVVKKQCKG
EAHIIRYADDCAPRRRRDATERSHAAQKMREGPSKPPYRWRLQTTLSCFGQKPR*
>Syntrophomonas_wolfei_subsp.metylbutyratica_JCM14075T_01267_Sporulation_protein_YunB
MFGPRINWLPIVLVLILFASIIMTLALLDFELKSSILAIARSKAQIQAVESINQIVAKKIVAQIQYTDIVYVHKDEQGRVVLIQPNTIELNRLMTETMVEVTRAMSSMQENTISIPLGQLTGSRILAGYGPKIKVKTIPVSQINVNILNKFEQAAINQSRHLIYFQIEGRIKVAVPFLDDEVKISTTIPLAETIIVGEVPKTYVNFSGSGSGLNSLLGE*
>Syntrophomonas_wolfei_subsp.metylbutyratica_JCM14075T_01268_Glutamate--tRNA_ligase_2
MRNIRVRFAPSPTGALHIGGARTALYNWLFARQQGGKFILRIEDTDFRRSREDSAQGIVEGLSWLGLDWDEGPDIGGPLGPYRQSERGDIYSHYLQELLDSGRAYYCFCTPEDLQKEREEAAQEKRDYKYGGRCRALKPEEASEMLQAGKPAVIRLKVPLDGNTIVPDLIRGDVSFSNALFDDFIIAKSDGWPTYNFAVVVDDFSMQISHVLRAEEHLSNTPRQLLIYRALGFKEPSFAHLSMILAPDR
SKLSKRHGAISVQEFKNQGYLPEALVNYLALLGWSTGKDIDIWNREEMMREFSLEHISKSPAIYDLEKLAWMNGQYMMRLDIENLMTLLAPQARQRGWLNEDNSDYFQQAVELVRNRAKTRDELLDALGYFFEEVKQYDEKGVKKHFGQQKASTMLSEVLEIVSNMGSFSAAELEEAFRQRAQELEIKAADLIHPTRLALSGRTATPGLFELMEVLGQEKCISRLEKALVFIAQLTTHGPEGPNH*
>Syntrophomonas_wolfei_subsp.metylbutyratica_JCM14075T_01269_monoglyceride_lipase
LENLRIRKYDGQELAALAFIPVEPSMVLLVAHGFRGAKENGGKIYSFASRLQELGIAVYAFDFIGSGASDGSFADITLSRQADDLAVVMDYAYKRHQLPLLLLGRSFGGSTVLARGSTDQRVAGFILWSTPVMLKDCFARIMGSDFNKLKKGQSVRFQDEAGEFALNPGFVQDFDLHDMEHYLAAIASRPVLIVHGKEDEAVDFANAEYAARQLTNAWLYLVDQADHRFTWLTKEREDITINWLRE
TFLTRGR*
>Syntrophomonas_wolfei_subsp.metylbutyratica_JCM14075T_01270_hypothetical_protein
MKQVAARCLLCGKTYDVEEDHKDFKKLSEQAKEVPSFICDFCSNRVRHESDEKRKEKKPI*
>Syntrophomonas_wolfei_subsp.metylbutyratica_JCM14075T_01271_Putative_methyl-accepting_chemotaxis_protein_YoaH
VSLMARVERLLRLLDVRLRLMLAFTLIVAAVTGLMGFYATYVMSDKIVFTAQEKLKSDLALGRQIIELNFPGQWEVIDGKLYKGDILMEENYDVVDKIGRLTGDTVTIFKGDTRVSTNVMKDNQRQVGTQVSEQVADAVLKKGETYIGRANVVGTWNETAYEPIKNAAGQTVGIWYVGVPATPYDEAVGHFRNSMVTYSIIGILIGFLAAFLIAASVHRPLDRIKVAVTEVSGGDLTQKVPVFGK
DEPAQLALMVNSMVEKISELIGKTRRLAESVGEASGILLQTCEMSSTQMQDMTVKANDMNNSANSQADLTDRSKVVIGEMSEVIQQVARNSQEVSSSTMVANSKAQEGEKQVEKAIEQMGVISHTVNSTAQIVQGLGEKSQEINQIVDLITNIANQTNLLALNAAIEAARAGEQGKGFAVVAEEVRKLAEESGEAAKRIAGLIKEVQNEAVRAVKAMEEGTKEVAYGTEAVASAGEAFELIIKGVTEI
SEEIQEMSAASEEMAASAETALESIEKTSAAAEDNLGYTRMIGQLAEEQMASVEEVNASIESLNKIVIEMEEAISYFKV*
>Syntrophomonas_wolfei_subsp.metylbutyratica_JCM14075T_01272_hypothetical_protein
MSKGRILGGILLILLLLFNSGCNFEGKRLFQSSSAPEEQLIKVRIVFTDDQELETYIKNLGIDEKGKVYVGGSSLSYFYDANGKVLGSYNYQRVLYIEIIPEKNKEK*
>Syntrophomonas_wolfei_subsp.metylbutyratica_JCM14075T_01273_Lon_protease
LPGIIGQERAYRSLSMGVDIEKQGYNVYLAGAMGTGKTTLAWEILGEKAKKEPPPPDCCYVHNFKNPDCPRALLLPAGMGSSFKKDLEAGIDRAIKTLLKAFESEEYEYEKNAILGVFVDETNRVFMQLDEEAHSYGFTINRNQNSINSVPLKNGEPLSQEEFISLSEEERAEIMKKSAIIQEKLNQAFRHFKELEKKIKVKVKELENETARSALAPFFAELFEKYRLCKELVEHIEAVQEDLLANYEVFVQP
EEKSPLSLFRLIDRRSSLRRYQVNLIVDNSELKHSPVVFESNPTYANLFGQIEYEGEFGILATDFSKIKAGAIHRANGGYLILRVYDVVKNYYVWDALKRCLINQQIVVENLNRMLGISNTETLQPQPIPARLKVVLIGEPLYYYLLYNWDEEFKRLFRIRADFDIEVGKSRKRIKEYACFISSVCRKEGLKHFDPQAVAAVVDYAARLAEDQNKISTMLNKVVEIVIEADCWANYERAELVGLEHVKKAIREKK
YRSSLLENKIQAMMVEESLIINVKGKKVGELNGLAVYEIGDYAFGKPVRITAKTFMGEKGLVNIEREIRMSGNIHSKGVLTLNGYLGAKYARENPLTLSASLTFEQSYQGIEGDSASSAELFALLSSLADLPIKQGIAVTGSVNQNGEIQPVGGINQKIEGFFRLCQMKGLNGEQGVIIPRKNLSQLMLEEELVEAVKKRQFSIWAIEDIDEGLEILMGRPAGKREENGRFSPDSVHALVDQRLKEWSSRKR
RELVLPDRAINERRRPRKSRRRKGHE*
>Syntrophomonas_wolfei_subsp.metylbutyratica_JCM14075T_01274_universal_archaeal_KH-domain/beta-lactamase-domain_protein
MNALVSILVENTTPSPSLIGEYGFSALLEVDERKILFDTGSNDALFKNAENLGINLEEVEALVISHGHFDHTGAVIPFLQRFGPKKLYLHPDIFSQRFLQMKKGLGPNIGCPFEPEDLVKNGAEIIFIDSFSEIMPGVFISGEIPRRNSFENTGGNFKVATGHGLVEDKLLDDMALLVDHPEGLIIISGCAHSGMINTIEYILERTGRSKILAFIGGTHLITASEDRMAQTIAVLNMMDIEKIAVGHCTGFYAAA
RLLNELGPRVIKADTGMSFSF*
>Syntrophomonas_wolfei_subsp.metylbutyratica_JCM14075T_01275_putative_membrane_protein
MSATSIYLMIMGVSLVSLLPRILPVAILSRFEFPEALKRWLSFVAPAVLGALTALSIIAPRGNIDINTGNIYLWAFIPTFLVAIKSRSLFFTLLTGILSMAFLYNYFSI*
>Syntrophomonas_wolfei_subsp.metylbutyratica_JCM14075T_01276_Inner_membrane_protein_YgaZ
MCNMELREGIKDSIPIVLGYLPLGFAFGVLATEAGMNLQQATAMSVLCFTGAGQYIAIGVMQAGGAVITAILANLLINQRYTLFATSMVPYLKKLPTRWAAFLSYGLTDETYAVAMNRYRQREASIAYMAGLNLTSHLSWIGSTILGAWLGSMISNTERFGLGFALPAMYTCLLVFMVNKKSDALVAIIAALVCILTGYLLPESMKNLSNIIIATLIGASLGMLIDERN*
>Syntrophomonas_wolfei_subsp.metylbutyratica_JCM14075T_01277_hypothetical_protein
MTPLRKPLLLHKGCINIQSERWRSMVATPGEGEQAKGGERQDVARALYDHGWEVSVYPLSRETSRRCCPCEDCERYIYTSQ*
>Syntrophomonas_wolfei_subsp.metylbutyratica_JCM14075T_01278_Penicillin-binding_protein_2D
LRKFILSSIILALITCHTGFTWPLPRIEVPQASVVYDVNGEAIKGLGEQNRINIELSEMPNYFIKAVIAVEDKNFYQHHGIDMAGILRAIYVNIRERKIVEGGSTITQQTAKNLFLSNERTFLRKFKELFYALELERQYSKDEILTMYCNTIYFGQGAYGIEVAARTFFGKSARDLSLAESALLAGIPRWPSNYDPYINPEAAKKRQLVVLQRMQEEGYIDEESKARTAEEKLQYKKTSYIKGEAPYFIALVKDYLS
KKYGEQMIYQGGLKVYTSLDLYMQRAANQALQSGLENYDPNLQAALVAVDPGSGGIRALIGGRDYASSTFNRVFSKRQPGSTFKPFMYSLALYSGFTAADKIMCEEVEYELPNGDIYRPGDYGKEPYHWREFTLKEAVMKSDNVVAVRVNSILGPTAAASYAEKFGFSNIQPVLSLPLGASEVRPIDMALAYAVFANRGIYSNANYILKVTDRNGKVLEENYPQQRRVIGEDNAYIITNILEGVLEAG
GTGAHLRNIIGDRIAAGKTGTTDEFKDAWFVGFTPRISCAVWVGYDKNRDVNISGGVIAGPIWAHFIEGASRRLAEGDFYRPDNIRLLNICLDSGLVASESCPRQVEMAFVAGSEPEDICYEHREEDDWSMDDGTFPWWQSWSP*
>Syntrophomonas_wolfei_subsp.metylbutyratica_JCM14075T_01279_Putative_pit_accessory_protein
LRLFGTKDEQLFMLFSESARVVVRGGDILQNVVNDYNDLDIKMAKLTAMEHEGDRIIQELIRRLNTSFILPFDREDAFQLAQKLSTTLDYITGIIDRMILYKTGRPNKRVKEMVNVLCEALLLQERALKLLQSLEHNKREILECCEEIRQLERKQDNHYRQGLALLFENEEEPIVIIKWREVYEHIEMAQDYVQDVAELISNICVKYS*
>Syntrophomonas_wolfei_subsp.metylbutyratica_JCM14075T_01280_Oxygen-independent_coproporphyrinogen-III_oxidase_1
MRYEGSVYRPPSEARSYILQVTLGCSHNRCTFCSMYKDKKYRVRSLDEVMADIRMAKLYYGDLEKVFLADGDALAVETGDLVKIIAELKKNFYSLRHIGIYASPDSILSKEMEELKRLKEAGLTIAYLGVETGDPELLKDIKKGVTYEEMVEAGKKIREAGILLSVTVLLGLAGRTPQAVDHARKTAQILNEMNPDYVGALTLMLEPRTPLYRQMQRGEFELPGPFEILDEMRIMIENLELEGTEFRSNH
ASNYLPIKGRFPEDKENILALINTIINRNDASYLRPDYLRAL*
>Syntrophomonas_wolfei_subsp.metylbutyratica_JCM14075T_01281_Small,_acid-soluble_spore_proteins,_alpha/beta_type
LSRKRGLMSEQLKYEIARELGVDQLVSTQGWGAVSSRDCGSMVSKAIEMAERSISNQL*
>Syntrophomonas_wolfei_subsp.metylbutyratica_JCM14075T_01282_putative_Mg(2+)_transport_ATPase
MEIWVGRIVLAAIMGFILGVSTDRSISTTRTYAITCMAAALLTIVSNEFFRNLGHPYYSDPGRLSAQIISAIGFIGTGLIWMAEDKPSGSGLSLAASLWITAILGMLIGAGLQQAVVLATFLVIIICFSDRLIRCKDYLWRILSGKHRIDKGE*
>Syntrophomonas_wolfei_subsp.metylbutyratica_JCM14075T_01283_Inosine-5'-monophosphate_dehydrogenase
MSEKFGKEGLTFDDVLLVPGRSEVLPQDVDVSTMLSRNIRLGIPILSAGMDTVTEIRMAIAVAREGGIGIIHKNMSIEEQARMVDRVKRSEHGIITDPFFLSPDNTIRDALDIMEHYHISGVPITEGSKLVGIITNRDIRFETDFTQPIKNVMTSEGLVTAPVGTSMDQAMDLLRKYKIEKLPLVDDNFNLMGLITIKDIEKTSKYPNAAKDQRGRLLVGAAVGIAHDTLERVECLKKAGADVVVVDTAH
GHSLSVIRMVATIKKQFPDIELIAGNVATAEATEELIKVGADAIKVGIGPGSICTTRVVAGIGVPQITAVFDCAQVAKKHNVPIIADGGIKYSGDIAKAIAAGADTVMLGNLLAGTDESPGETQIYQGRSYKVYRGMGSLGAMVQGSSDRYFQEDAHKLVPEGIEGRIPYKGYVSETIFQLIGGLKAGMGYCGVKNIEEMQSNTSFIRITNAGLIESHPHDISITKEAPNYQVI*
>Syntrophomonas_wolfei_subsp.metylbutyratica_JCM14075T_01284_hypothetical_protein
MERNKGQGSFPPWSRIASLEEMSREAGVDFDELLDCIKNGKSKEEMAEKFRVSEETVESLHQHFLHYGISSVMGGD*
>Syntrophomonas_wolfei_subsp.metylbutyratica_JCM14075T_01285_Trk_system_potassium_uptake_protein_TrkA
MKAIIIGAGKVGFSMAEMLSEENHDVVVIEQSPERQQILEDTLDIQVINGSASSFSVLEAAGVREADILLAVTEFDELNMLACMLAKQYGTRVTVARVRNTEYLELKHISLNQLMGIDLIINPERVTAFHIAQIVKNPEALNVDYYAEGKVQLVELRIQPNSPLVGKTLTDADSSLPYNIVSIVRNHKTIVPRGQDTFYAGDHINLMAQSSDIVEVEKLLGFYRQKINQVTILGGGRTGYHLAQILEEQKIN
IKIIEKDLKRAEKISEKLNNTLVIHGDGSDYQLLDEENVGQSDIFVAVTDDDKINMLCSLIVKNLGVKTTICQLKRFEVIPLVEQIDIDIVLSPRILTAGAILKYIRRGDIVSVTILGEEKAEMLELFAQPGSIAVNKKIQDIKFPRGSVVGAVVRGEQVIVPDGNFKINAFDRVIVFTLPQDLPKVEKLFLKGAWLF*
>Syntrophomonas_wolfei_subsp.metylbutyratica_JCM14075T_01286_Trk_system_potassium_uptake_protein_TrkG
LIRTRVILGYLGRIILIIGISMLSSVLCSLYYRESIIPFSLAAGVTIVTGLLLVFSAEKQAIHYKEGFVIVSLGWLLASLFGSLPYIFTGCLPSFADAFFETVSGFTTTGASVISNVEATPKGLLFWRSLTQWLGGMGIIGLFVAIISGMGAQANHLFKAEVPGPVANKISPRVRETAKKLWITYIVLSVCCTLLLYLFGMDLFDSLCHTFATMATGGFSTRNASVGYYNSPLIQWTLTIFMFIAGVNFTLHYLGFKQ
RNLQVYLKNREFLFYTAIVVVASLLVTLSLSLGGSELSTEGKVRAAFFQVVTIVTTTGYSSVDFELWPNMALGIIFLMMFVGGCAGSTGGNIKPGRYLILLHRSIIEIKRIIHPKAVISLHFNDRIISDSLLINVLQFFFLYIMFLALGTIGLSCLDVDLITSITAAASCLGNIGPGLGEVGPMDNYSFIADSGKYLLSLLMLVGRLEIYPLLLLFLPDFWRE*
>Syntrophomonas_wolfei_subsp.metylbutyratica_JCM14075T_01287_sporulation_protein,_YlmC/YmxH_family
VRLNELVGKEMVNIYDGMRMGTVGDSDMLIDEESGLIISIILPNRNNALNFWVDRQKMVIPWEAVKKIGREVIVVDVNNTHMRLKNNSL*
>Syntrophomonas_wolfei_subsp.metylbutyratica_JCM14075T_01288_Deoxyuridine_5'-triphosphate_nucleotidohydrolase
MKVLFTRLHSHDLPLPRYMTPGSSGLDLYAAVDEEILIPSGKIVLVPTGLALAIPEGYEAQIRPRSGLALKYGITLLNTPGTIDADYRGEIKVIVINLGDKDYILKRGERIAQMVFSRVEKAEFMEVKSLDETLRGSGGFGHTGI*
>Syntrophomonas_wolfei_subsp.metylbutyratica_JCM14075T_01289_Putative_zinc_protease_AlbF
VINTWLLDKQARLIVEEIPYLKSAALGVYIKLGSRHEKEEMAGASHFIEHMLFKGTESRSARDIAESFEEIGGQLNAFTSKEFTCVYARTLDENISSAMEIIFDMLFNSTFATRDFATEKEVIIEEINIYEDTPDDLIHDLFARNLWQGHPMGSPILGTLDSVSAFSRDEIFDFYKKCYAPSNMVIAVAGNVDKSHIKEQVEKCLARQPLTQVNWPEPKPTEYSSFVRLLEKETEQVQICLGVPGISYFDQNRYV
QNVMNSILGGGMSSRLFQKIREELGLAYSVFSSPSTYSDTGSYSFYIGTGPGKIATFFEALYNELEFFVSRGVSEREVSRTQQLIKSSMYLGLESVMNRMSRLGKSLLMYDRVIPVEDVIQEILAVDAGKVQGFSSSILQKPAFSLAAIGPAEVLPQVEKEFRKWWG*
>Syntrophomonas_wolfei_subsp.metylbutyratica_JCM14075T_01290_D-alanyl-D-alanine_carboxypeptidase_DacB_precursor
LRAIGREIRFILLLLLMGLIASPVNASPYISSPCYCLMDGESGQLISSNGDETRPVASTVKMMTAILSVEYASMYEEATVSSHAAQTPEYSIGLKAEQKIAVGELLKVALIRSSNDAAMVLAEHIAGDEGFFAHLMSKKAFLIGASNTRFSNASGLPGGEQFSTCNDLAQIGRYAQSHPQIKELVATRQSEFKHPSYSQPLRISNTNSLLGSYQGADGIKTGTANAAGKCLVASATRDGRHLIAVVLKSSDR
AGDCMRLLNYGFKESYYQQIINASTPFKELRVLKGQTPKVKIYPARDLYLWVGDNSPDIEKKVNMKYELQAPLSKGQEVGSLVVYADGKLVESMPLLCGDNITRQANLFQRIIKDFILP*
>Syntrophomonas_wolfei_subsp.metylbutyratica_JCM14075T_01291_Bifunctional_xylanase/deacetylase_precursor
VKIYFISRKKLIVMLLSVLLLGLFTAILLMRGDDSSVASRVLDPIYQGNSGEKAVAIAVNVDWGEEYIPQMLKSFKENQAEVTFFVSGKWAEKNPELLKEMKAAGHSIQNHGYKHLHLNNISAEEASEQIRKAEEIINSTVGEKPRFFAPPYGEYNQQLLNVVAALDYELIMWSIDTIDWQRPDPATIVKRVSNKLHNDAIILMHPTDPTVKALPAILERIKQDGYKMLTIDKIINQSKGDNIKGDNN*
>Syntrophomonas_wolfei_subsp.metylbutyratica_JCM14075T_01292_Vancomycin_B-type_resistance_protein_VanW
MAGEKLEGLLPGELRLLVENMALKEQKLPLEPALDKETGEVIPEQEGCIVDIEGSVSTALQAEEGEKLELLMRAIPSRYSSRDLQKITFSRGYFETWFTGTYQRYTNVSLACSSVNNSVLWPGQEFSFNETVGPRTPERGYMPAPVFLMGASELDYGGGVCQVSTTVFNAAEKAGLIIIERHVHTRKVYYVAEGKDATVSYGDLDLKFSNNTDSPLIIKAGINRGKVWVNILGEED*
>Syntrophomonas_wolfei_subsp.metylbutyratica_JCM14075T_01293_Polyribonucleotide_nucleotidyltransferase
VIVKKYQMEVAGRPLIVEIGQVAQQANGAALMRYGDTVVLVTATAAKEPREGIDFFPLTVDYEEKQYAVGKIPGGFIKREGRATAQATLSARLIDRPIRPLFPKGFRNEIHVVATILSVEKDNAPDVTAITGASVALSISDIPFAGPVAAVIVGLVDGQLIINPTVEQHQKSDLHLAVAGTKEAIMMVEGGANEVPEETMRDAIFFAHEEIKRIVDFQENIIREVGLPKMQVEIPVLDEGIKKAVVEYAAPL
FEEAVKNPDKKTREAQMDSSQKSVLQHFAENYPDEEKMIADVLDKTMKAVVRRMALEEGERVDGRLDEIRQVSCEVGFLPRPHGSGLFTRGQTQVLSVTTLGAISEEQRLDGLGIEERKRYIHHYNFPPYSTGETKPMRGPGRREIGHGALAERALLAVIPSEEEFPYTIRIVSEVLESNGSSSMGSVCGSTLSLMHAGVPIKAPVSGIAMGLVKEGERFAIMSDIQGIEDALGDMDFKLAGTEKGV
TALQMDIKITGVNREIVEAALKQAREGRMFILKKMLEAIEKPNEELSPYAPQMIRMQIDPDKIREVIGPGGKTIHKIVDETGCKIDIEDDGSLFIMATDEEAAKKARFFVESIVADVEVGKTYMGTVKRIMDFGAFVEIIPGVLGTSGKEGLVHISQLAEERVNKVRDVVDIGDQILVKVTEIDRQGRVNLSRKAVLKSAVRK*
>Syntrophomonas_wolfei_subsp.metylbutyratica_JCM14075T_01294_hypothetical_protein
LALSLERKGEIIKTYQIHENDTGSPEVQIAILTERINYLNEHLKINSKDHHSRRGLLKMVGQRRSLLDYLKKKDFERYRSIVTRLGLRR*
>Syntrophomonas_wolfei_subsp.metylbutyratica_JCM14075T_01295_Riboflavin_biosynthesis_protein_RibF
MEVVREIENFTNNECPLFLALGNFDGVHQGHQRLIGDLVEKARASNGIAAAFIFEPHPAMVLNPSRAPKLLVTAERKAELLKKMGLDKLIYNTFDLAISQCSPEEFVKSILVERLKIRAAFVGFNYSFGHKGSGTPQLLKELGEKYGFQVSIIPPVEIKGQVVSSTIIRQALDAGDMKLAREMLGYYPMIEGKVIEGEKRGATIGFPTANLGIGAELNIPGKGVYAALAVLEGENFGAAVNIGSKPTFHQEY
PVSVEAHLIGFNRQVYGQDLRLFFLQKIRDEKRFSSVDELINQIRLDREQAGEIYESLESRGKIDII*
>Syntrophomonas_wolfei_subsp.metylbutyratica_JCM14075T_01296_tRNA_pseudouridine_synthase_B
LHGFLNINKPQAITSFDVIRKLKKALPRKYKLGHLGTLDPMAEGVLPVAVGQGTKIIPFVEDETKEYIVTMTLGASSDTQDAWGAITYSMRKVEPAQIERVIALFRGKSRQVPPMYSAVHHEGKRLYELARQGLEVERKAREIEIFALELLNADWERELPQLSLRVSCSRGTYIRTLCHDIGQELACGAFLSSLCRSRSGCFKIEEAVSLDYLLEKRENLSRSLLPLDYPLNNLPLISLKNTELPAIINGRKISR
TGKLASPRVRLYTPEGQLLAIAEANNYNEDTVLQPCRVFKIGE*
>Syntrophomonas_wolfei_subsp.metylbutyratica_JCM14075T_01297_Bifunctional_oligoribonuclease_and_PAP_phosphatase_NrnA
MNKLKEIARKIMDKDDFLLLGHKAPDGDCIGSMLGLYLGLLAMDKRVRMLLSDPVPPVYHYLSAAEQVKLPGEIGSIPQNLIFLDCSDEQRVGQEVFSLIQERNCSFNIDHHLSNDLFADFNYVDTNAAAAAEIISELLLLMPINISAAMADALLAGIIMDTGCFLNANTTAKTLSIAAQLLNFGANVNLARINLFESKSRKEVLILQHALQHLDFSSDGKIAWMSLPYNELVAIEAEDLYPEGLINHTR
MIEGVEVGILFREMPAGKVKVGLRSRGQVDVAALAAQFGGGGHRQASGAQCEGELEEVKSAIIQAVEDVI*
>Syntrophomonas_wolfei_subsp.metylbutyratica_JCM14075T_01298_Ribosome-binding_factor_A
MSKRRQERMSVEIMRVLSQIIQEEIKDPRIEFKNLSITRIDLSNDYSHARVNISILGDEVQREEAMKALQKAKGYIRSALAQQLKVRHAPELEFRLDRSIEHGIRISSLLEEIREEEEAKGSNE*
>Syntrophomonas_wolfei_subsp.metylbutyratica_JCM14075T_01299_Translation_initiation_factor_IF-2
MAKIRVHELAKELGIASKEMVEVLVELGLDVKNHMSTIEDSQASWVKKRLSKRGEDSKKQPAQPVIRDEAVKKNSGEPAPQTTQKKPDNQLPGSPRLESGTRPQEGSKPGESMGRHDFSGKREQPRKGEESHSAGAKPATQMKSPRNSAPRPTARPEHRSSGATGRTDHRAPGAAGRTDNRGAGSASKPDNRGTRPAAGRPDNKGSRPGDAKRPPQRAVPSNTPRPVSSPERTMEKKTRETG
SKTLPGGDRTAGDKKAFRKNTPALGQYQSKDYSRPGRKSKHKRKKENVEFKTPESIKIEGSIMVRDLAEKLNKNPAEIMKKLMELGIMATINQNIDYENAEIVSSLYDVKVERELSEEKILEELVDIDDDDELKARPPVVTVMGHVDHGKTSLLDRIRQANVVSGEAGGITQHIGAYQVTIKNNKITFIDTPGHEAFTAMRARGANLTDIVILVVAADDGVMPQTVEAINHIRAAKVPFLVAINKVDK
PQADPERIKQQLTEYNIVPEEWGGDTIFVPVSAKSGEGIENLLEMILLVAEMNEIRANPDRAAYGLVVEGELDKGRGAVATVLVLNGTLKIGDYVICGTNWCRVRAMIDDRGKRVEKALPSMPVEIMGWSGVPEAGGKVQVCDEKVAKEIISLRLSEKKIEEQKQSSRVSLDEFFQQMKDAEIKELTLIIKGDVQGSVEALRQSLLRLATSEVKVNVIHSAVGAITETDVMLASASNAIIIGFNIRPDSKA
RKYAEDEKIDVRMYRVIYEAIDDVKKAMSGLLDPEYKEKFLGRAAVRALFKVPNVGVIAGSYVIDGKIQRNASVRVLRDGVIVYEGQLSSLKRFKDDAKEVVENYECGIGIRDFNDVKEGDIIEAYTLEEIPREL*
>Syntrophomonas_wolfei_subsp.metylbutyratica_JCM14075T_01300_putative_ribosomal_protein_YlxQ
VSDSLRKSYAILGFAQKAGKISSGALAAKSSILRRRAFLLIVSNDIAENAKQGLISSCQKQKIPWVVMGNKYEIGACIGKAYRVAVTINDPGMAESLIKSLEGVGVKVNTGVVEWQK*
>Syntrophomonas_wolfei_subsp.metylbutyratica_JCM14075T_01301_hypothetical_protein
MSRIRKQPQRMCVGCREMKNKRELVRIVRSPQGDIDLDTTGKKPGRGAYICPEPECLKQAIKGKRLQKALEQNIPDGVLEKLREQINQDKTRQ*
>Syntrophomonas_wolfei_subsp.metylbutyratica_JCM14075T_01302_hypothetical_protein
MSNDLVQALNDIEKERGIPKRALVEAIKSALSTAYKKNFGIAQNVSVEFNEINGEVKVFSQKKISDEVKDPRLEMSLEEARELYPDCKAEDLVYVEATPSDFGRIAAQTAKQVVIQKLREAERSLIYEEFLEREGEIITGTIQRIESKTVYISLGRTEGIMLASDQIFGERYEVGQRIKAYIYEVKNTSKGPNIFVSRSHPYFLRRLFEMEVPEIYDGVVEIKSIAREAGARSKIAVYSLDDKIDSVGACVGPRGLRVQ
NIVSELGGEKIDIIKYDKDPERYISNALSPSRVVAVNINETDKVARVIVPDYQLSLAIGKEGQNARLAAKLTGWKVDIRSESQMSDYYKDIDVEIDDEVLEQDWIEENPGETEIDEQDS*
>Syntrophomonas_wolfei_subsp.metylbutyratica_JCM14075T_01303_Ribosome_maturation_factor_RimP
MVEERLNELELELVDIEYRKENKEQFLRVFIDKDSGVDLEMCSQANRAIKESLDEQEIPYDYLEVSSPGLERVLKKERDWERFSGYRVRIKTRKSFPGPQRIIGILLGFDSENIAVEVDGELYKVPRDMITIIRLHPEI*
>Syntrophomonas_wolfei_subsp.metylbutyratica_JCM14075T_01304_DNA_polymerase_III_PolC-type
MINGDFYSFISNFNDSLSKYWKDSYVEKLEVFRSNKKWKIYVAIMEPLPAHILQQSIVKLKDSLPFLNELEIIPRLADFGRQMENILRLRQNEVLSEVFLSDQADFASLGWRFNENRIDMLVQDRNVFNKLLEKESCNRLSRWIGQEYALRVVVRAICTENNEDNVPDNYYLLAGQKIEVLAGDTEFKGRKKRYSSRNSGGIKLLENSGIIKSELQEGMKNAVIEGEVWHKEINSLKDNSIVAAYYISDYSNS
IVAKLFLDKVEDDKIKLGDWIKLCGSIRYDNYVKEAVLFIDAFVHLEKPERKDTATEKRIELHAHSKMSAMDGLTEVSKLIKRAAEWQHPAIALTDHGSIQAFPEAYQAAKKYKIKVIYGVEAYLVEEDKKERPYHIVLLALNQNGLKNLYHLVSISYMDYFYRYPKIPRQELIKKREGLLLGSACEAGELFRAMVRGAKEKEIEEIAGFYDYLEIQPLDNNDFLVKEGSVKSREELQEINRRIIRLGQKFQKP
VAATGDVHFLDPHHEIFRRIIQAGQGYEDAESQAPLYFRSTDEMLAEMDYLSEEEARAVVIHNPIALIAEMIEDIRPVPEGFYPPKIESAAEEVTRISWDKAYSLYGDELPTIVESRIMKELNSITHHGFSVLYLIAHKLVEKSNQDGYIVGSRGSVGSSLVAFLTGITEVNPLAPHYLCPLCRFSDFVVDGSANCGADLPERSCPECGGKMHQDGFDIPFETFLGFEGDKVPDIDLNFSGDYQSRAHQYVEE
LFGQENVYRAGTISTIAEKTAYGFVKKYMENKEIDISNAEVNRLVKGITGVRRTTGQHPGGIIVVPQDRDILEFTPLQHPADNKDSGVITTHFEYHAIGEQLVKLDILGHDDPTVLKELEDLTGRKASSIKLNEKETMKLFSGVEPLGLEEADILSTVGTYGIPEFGTRFVRQMLEATRPTTFSELVRISGLSHGTNVWLNNAQNLIKNGTAGLSEVICTRDDIMSYLIQKGLDKKQAFKIMENVRKGN
GLNSGECELMAGQNVPSWYIDSCQKIEYMFPKAHAVAYVTMAFRIAYFKVYYPLPFYASFFSIRAEDFDSQIILEGYEALKKRIQEIEKAGLSASQKDKKLLPVLEVAMEMYARGFTFQPVDIYESDASKFLVVDNALLLPFSALPNVGAAAAHGIIESREGGSFISVEDFQQRSRLNKTAMEVLRKFDCFNHLPETSQVSLFG*
>Syntrophomonas_wolfei_subsp.metylbutyratica_JCM14075T_01305_putative_Mg(2+)_transport_ATPase

MGIYEIVFKLALASILGALIGLERESLNRPAGLRTYTLVCVGSALAMIVSIDIYLQYYHTVNADPGRIAAQVVSGIGFLGAGTIIREGATVRGLTTAAGLWAVACIGLAVGSGLYIPAVAATVLILFVLIYFVKFEEKIMGMRDYKGLVMVVDDRPGQVGSIGSILGDLCVLIKNIQLTRIDEGDSLEIELLLQLPPNLSIEEVIQELSIIKGLRSIDRLG*
>Syntrophomonas_wolfei_subsp.metylbutyratica_JCM14075T_01306_Proline--tRNA_ligase
LKASELFFPTLREVPSEAEVLSHQLLLRAGFIRKATAGVYSYLPLASRVLKKIMNIVREEMDRAGGQEVILPIVQPAELWKKSGRWEVYGDEMFRLKDRHNRDFALGPTHEEIITTLVDADVHSYRHLPLLLYQIQNKYRDEIRPRFGLMRGREFIMKDLYSFDIDEEGLDVSYHKMYEAYNRIFQRLKLQYRVVEADSGAIGGNESHEFMVLAENGEAEIVYCENCDYGANTEKAVCSLEEPKAAEEEQ
LEREKVHTPGQRTIQDLLDYMNIPKEKQIKTLIYYADEELVAALVRGDRELNEIKFKNVLGCNQLFMADEAAVKQLCGAGFGSLGPVGLPLKTYIDLEVSQMKNFACGANEDDYHFINVNLGRDFTPAGINDIRNAVTGDSCPVCSAPLYSMRGIEVGHIFKLGTKYSEVLEANYLDQKGREKPMVMGCYGIGISRTMAAAAEQGADENGIVWPLPIAPFEVIIVPVNSKNEEQMQAAWSLYEEFK
QEGLETIIDDRDERAGVKFKDADLIGIPLRITIGPRSLQEKQVEIKKRTAKDVELVPLDAVSSRVKLMLKEMSK*
>Syntrophomonas_wolfei_subsp.metylbutyratica_JCM14075T_01307_4-hydroxy-3-methylbut-2-en-1-yl_diphosphate_synthase
MRHKSRVIKVGTVPVGGEHPVVVQSMTSTDTRDIERTVKQIQQMEAAGCELVRVAVVDEEAARALARIKKKINIPLIADIHFDHRLALKSLEAGADGLRINPGNIGEEKKLREVVKACSEKKTPIRIGVNAGSLDKRILEKFGGISAPAMVESALENVKLLEDMDFHEIKLSLKASSVMLTVEAYRLLAAEVDYPLHVGITEAGTKDRALIKSALGIGMLLYEGIGDTIRVSLTADPVDEVWAAYEILRSLG
LRRRGIELISCPSCGRCEIDLIETAEEVDRKIRHYQQNLKVAVMGCVVNGPGEAREADLGIAGGRGFGLLFKNGEIIKKVPESELVAVLLREIEDSSKK*
>Syntrophomonas_wolfei_subsp.metylbutyratica_JCM14075T_01308_Zinc_metalloprotease_RasP
MNTILITLLIIALLILAHEWGHFITARRIGIPVYEFAIGFGPKVFSWKRNGVIYSLRLIPLGGFVRMAGEEPGDPEEPNGFSHRTPLEKIRVSFAGPFMNFVLALLIFVFSYSVIGLPHSSNEPVIGTVIKGKPAELAGIKAGDRVVSVNGIAVNSWADFNRQTSRSSGQPLELQLERKQQRLSLEVSPVKLDSSGNMGIGVLNRVVYEKQGIIKSMELGLKQTYELTLLLFSALGILISGGASMGDLAGPVGITHL
VGEFAQMGIIFLLNFTAFLSINLGIMNLLPIPALDGSKIVFAVVEAIRKKPLDPEKEGFLNWIGFLFLIGLMIIVTFNDIVRWIRG*
>Syntrophomonas_wolfei_subsp.metylbutyratica_JCM14075T_01309_1-deoxy-D-xylulose_5-phosphate_reductoisomerase
MNKKKSNVIILGSTGSIGRQALDVIRMHSDSFRVLGLAAKDEVELLREQIELYRPEYISLADKESIKKLGELGGSGVKKALPGVEGLCELASLPEADIVLAALSGAIGILPTLAAIKVRKRVALANKETLVAAGDIVMQEARQQGALIIPVDSEHSAIFQCLAGEARHLQRIWLTASGGPFRDFTREQLKAVNLEMALQHPNWSMGPKITIDSATLMNKGLEVIEAHHLFGVGYNEIEVLLQAESIIHSMVEL
QDGAFLAHLGRPDMRIPIQYALTYPERIESPVERLDFRTLGAIHFGRPDTQAFPALALAYEAGKAGGTMPAVLNAANEVAVNSFRQRRIDFTRIPLLVERVMEMHDIVKQPDLDSILAADAWARVRCQELI*
>Syntrophomonas_wolfei_subsp.metylbutyratica_JCM14075T_01310_sporulation_integral_membrane_protein_YtvI
LDPELQKYLKILSKTTIAVMVLLAIYLLFHYVFPLTGKILAHLPVLFLPFIFAILLAVIIEPVVIFFEKKAHLNRNWSVVISLLLVVGGFFTLLSLLVSRIINDMSRLYPQVLRYSDQITASFMAAISDLKLFYLGLNLSPQAQNALQQNMENGIKLVSSLMEKSINGLIHGLAMLPGILVFLMIATVATFFIIKDRALIRSFVMQWIPGTVRSKTREVFAELFRVLVGFVKAYGILISVTTIVTMVALRVLGFDYVITIG
IIVGLLDILPVLGPGAFFIPWIIWHFVAGDLHLGISLLIVYLLISVVRQFLEPKIVGDNIGLHPLATLISLYAGLQLGGLTGMIMGPVLLVIFIASYRAGLFDSLNWRK*
>Syntrophomonas_wolfei_subsp.metylbutyratica_JCM14075T_01311_hypothetical_protein
LTLLQKPLLLHEGCINIQSERWRSMDATPGEGEQAKGGERQDVARALLTRMGD*
>Syntrophomonas_wolfei_subsp.metylbutyratica_JCM14075T_01312_Phosphatidate_cytidylyltransferase
MFKTRLITSFVGIPLLLAILYQGGWYWKGFFVLLAIMGLFEFNRMIINRGHKVLQLPGYFLLLILLLGADYPQYTLPGIYLFLVMTLLYFVLGYPRFLPLDLAWTLLGSSYIGFLLSYAIQIADLSQPFLIILLAFLLTWASDVGGYLFGSVWGKHKMSPLLSPGKSWEGAAGSLLLSLLVSLAFFKLLDLGQLGISPVVLLGVAASILAQCGDLFISGIKRYSAVKDTGKIIPGHGGVLDRFDAFLLVVPVVYYF
FVYWG*
>Syntrophomonas_wolfei_subsp.metylbutyratica_JCM14075T_01313_Ditrans,polycis-undecaprenyl-diphosphate_synthase_((2E,6E)-farnesyl-diphosphate_specific)
MAVNKKAKLEESIDRNNLPQHIAIIMDGNGRWASKRLMPRTMGHRAGMSSLKEVVRACSDLGVSALTVFAFSTENWKRPLQEVDYLMKLLVEYLHRELDELHANAVRINVLGDYNSLPHQCRLEIAEALENTRYNKGLKFNIALNYGSRAEIIAAVKKLASGLLAGEISLEEISEESFAACLTTAGMPDPDLLIRTAGEMRISNFLLWQIAYTELWVTDCLWPDFSREHLMQAIWDYQHRERRFGGL
SRSQQGEENV*
>Syntrophomonas_wolfei_subsp.metylbutyratica_JCM14075T_01314_Vegetative_protein_12B
MIKDILSDTEERMKKTIEHLRKDLASLRAGRANPAMLEKIMVDYYGQPTPINQLANITVPEARLLVIQPWDKTIIASIEKAIMKSDLGINPSNDGNVIRLAIPQLTEERRKELVKVLRKRAEEARVAVRNIRRDANELLKNGEKEKLISEDENKKGMDDIQKETDRQIKEIDSVLQGKEKEIMEV*
>Syntrophomonas_wolfei_subsp.metylbutyratica_JCM14075T_01315_Uridylate_kinase
LQKPKYKRVVLKLSGEALAGHNTYGIDNEVLNSIARQVVEVVRQGVQVAIVVGGGNIWRGVAGSAKGMDRATADYMGMLATVINALALQDALEQEGMGTRVMSAIEMKEVCEPYIRRRAIRHLEKGRVTIFAAGTGNPYFSTDTAAALRSAEIEAEVILMAKKVDGVYDADPIRNPTAQKFDRLNYIDVLSRGLGVMDSTAASLCMDNGIPIIVFDLTREGNILKAVMGEDIGTYVGR*
>Syntrophomonas_wolfei_subsp.metylbutyratica_JCM14075T_01316_Elongation_factor_Ts
VITAEMVKELRERTGVGMMDCKRALVETNGDIEKAIDELRTKGLAKAAKKAGRVASEGVVTSYIHGGGRIGVLVEVNCETDFVAKTNDFKQLAYDIAMQVAASNPEFLNREKVPQEVIDREKEVLKAQALEEGKPEKVIEKMVEGRVEKFFKERCLLEQAFIKDLDKSVQELINENIARMGENITIRRFARYEVGEGIEKESCDFASEVMSQLK*
>Syntrophomonas_wolfei_subsp.metylbutyratica_JCM14075T_01317_Vegetative_protein_209
MCIFLSVVTMKQLLEAGVHFGHQTRRWNPKMATYIYMERNGIYIIDLQQTVKKFDAAYEFVKSVAAAGKGVLFVGTKKQAQETIREEASRCGMYFVNQRWLGGMLTNYKTIKMRVLRLKELEKQEAEGAFEVLSKKEVARLLNERERLERFLGGIKEMDKLPGAVFVVDPRKERIAVAEARKLNIPVVAIVDTNCDPDEIDYVIPGNDDAIRAVKLISSRIADAVLEGKQGEQITA*
>Syntrophomonas_wolfei_subsp.metylbutyratica_JCM14075T_01318_hypothetical_protein
LKKVNPITSYSLLVALVLAILLIFIFFIQKSHMSNYSQELKQTVLEAGCIRDELEKMMENSLSISELIVERLDTEASGVNYSSSRKDDLLAIQEMEEETISPHLPPGEKPGSASKVRLYELARELEMDSKELIHLLKDMGYTYFHHMNRISRETAEIIKDQILWRDDPISLPDSFMEKALLSEEETTTREELPWLGFSLDELRTAHPYLAVRSLHEKGYNVKEIAQLLERGQGEVELILNLTRKKSVI*
>Syntrophomonas_wolfei_subsp.metylbutyratica_JCM14075T_01319_hypothetical_protein
MTIRSVDMQVLIQKVGDVARIQQSQQLENNSRQQEFAQQISQQTDKNVKTVNQMLRNERTYVHEKQEKEESGKKKQAKKGKSEKMANRDKTDIRPDMRGNELDIIV*
>Syntrophomonas_wolfei_subsp.metylbutyratica_JCM14075T_01320_hypothetical_protein
MAEEPRIDGQVNVSVDKDMMRAFVEVTGPEEGGVPCSLEQVRKVLAEKGVVYGIKEEALQQAVLAENWRRDILIAEGVAPVDGVNASIIFKFPLPKERIGPKVDDKGNVDYHDLGLIYNVKRGTLLVERIPAIEGEPGTNVLGNQIPPKKPKELRLPRGKNTVADNEERFLYAAADGHVTIMDNKVVVDPVLHINGNIDYSTGDIDFIGNVLISGMVNSGFKVKAEGDIEINGFIEGADVTATGSIVV
KGGIAGGLKSLVKAGENIYARFIENSRVEAGKDVIVKDAIMQSFVKAGGSVRVSDRKALIVGGCIQAFQEIESKVLGSQLATQTIVEVGINPLFRSEYQNLVKQKTEKKKLFDNLSNNLQIFQRSGVSPDSLTENKRRTLLKMLEDFKKLRQEIAGIEERIIYLEEEFEKGSMARIKVLETVYPGVRISIGQSMYIVNDIVKFAAFVSDQGEVKLTSLR*
>Syntrophomonas_wolfei_subsp.metylbutyratica_JCM14075T_01321_Sigma-28
MKPDLKPDLATLWIQLKENEDIEARDQLIIHFAHLVKYVASRLAIHLSSLVELDELISYGIEGLIDAIEKFDHKRNIKFETYAITRIRGSMIDGLRSMDWIPVSLRQKSKELEKAYINVEARLGRSASDAEVAEELGLNPGEFALLLRDVAATTIISLDDFLPGEDGEQKKKRMLDLLEDHNSISASEIVEFNEVKDLLAKAISRLPDKEKMVVYLYYYEGLTLKEIGATISLSESRISQLHTKAILRLRGSLSKKKNL
VI*
>Syntrophomonas_wolfei_subsp.metylbutyratica_JCM14075T_01322_Chemoreceptor_glutamine_deamidase_CheD
MSKIIQVGMADYKMATAPDKLITAGLGSCIGICLYDKVIKLGSLTHIMLPSSSQAKNASNEAKFADTGIKLAIREMEKNGAQASRLLAKIAGGAQMFKFSGESDIMKIGERNSVAVKENLQLHRIKLLSSDTGGNYGRTIIFDPETGDLLVKTIGHGERII*
>Syntrophomonas_wolfei_subsp.metylbutyratica_JCM14075T_01323_CheY-P_phosphatase_CheC
LVYDFSELSGMQLDILREIGNIGSGNAATALAQLLNAKIDMNVPQVNILPFAEVPALVGGADLHVVGLFLVATGSAPASILFLLPVDKACLLVDMLMGKEWGKTDPTNLSDMDFSALMELGNIICATYLNALAVFTQLDFRPSVPALGIDMAGAILNAVLAQFGAVADHVLVLETEFKRDEQEIVGHFFLLPEPGSLDVILASLGVSL*
>Syntrophomonas_wolfei_subsp.metylbutyratica_JCM14075T_01324_Chemotaxis_protein_CheW
VDKMVNLPGEEELSGDDGIQVVAFRLGKEEYAVDILHVQEIVRLLSITRVPRSARHIEGVVNLRGNIVPIINLHRRFNIESAGEEEDKRIVVFHFDDFKAGIIVDEVSEVLALNSNNIEETDKVYSSMSSDFIKGIARVDNRLFLLLDLQKIIE*
>Syntrophomonas_wolfei_subsp.metylbutyratica_JCM14075T_01325_Chemotaxis_protein_CheA
MKKDMSQYLDVFLEESKEHLESLNQKLLDWEKNPGDIAALNEIFRAAHTLKGMSSTMGFEDLADLTHHMENILSDLKEGLLETNPQVVDILFQCFDRLQLIIERIESNGSGKIDNSELLMILEGIKSGGLQMAAPTAGNGQDLKGEEILIPAHSNISSFEFNQYDMTVMREAASRNFSSIYIRLCVDPGCLMKSVRAFMVFKALEEDSEIIKSQPPASDLEEGNFEEEIELLVITASPIKELEKRVNAISEIKV
REIKEIDLASLSPGNRTETNDDLLGDSQDAVKESKEMPKGHKVKQTVRVDIDRLDILMNLVGELVMHKGRLEQIGASSKIDELNETIEQIDRISGDLQSVVMKVRMVPVEQVFNRFPRMVRDLAKELNKEVDFLIEGKETELDRTVIDEIGDPLVHLLRNAVDHGIESPQDRLKRGKPQKATVILRARHEGNNVYIEVEDDGAGINIKRTLGKAVEKGILSTKEAEVMSPEEAIQILFNPGFSTTENIT
DVSGRGVGLDVVKTKMESLSGEIFVDSRPDQGTRFRIKLPLTLAIIQALMISVRDEIYAIPLGSVDETTMINNEDIKMVQNQEVIVLRGNVLPLFRLASLLEIPGEAAANEDEMYVVIVRKGERQIGLVVDTLIGQQEIVIKSLGKMLAGIPGIAGAIVAGDGFVRLIIDVATLF*
>Syntrophomonas_wolfei_subsp.metylbutyratica_JCM14075T_01326_Chemotaxis_response_regulator_protein-glutamate_methylesterase
MPRNRVLVVDDSALMRKMIADMINSDDEFEVVGKAGNGQEALEKIKELQPDLVVMDMEMPVLDGLSTLRRIMESQPLPVIMFSGLNSKETEQTLKALQLGAVDFILKPSGQISLDIGNIKEEFIEKLKVAANSNRKLQLYQSPPAPALKTKKSLPDEGGRLNKLVVIAASTGGPKALHQVIPRFPAGIDAAVLVVQHMPPGFTGTLAERLDSLSELKVKEAEQGEKVLPGCVYIAPGDYHLKAQSRL
KGAENELYIELDQSKPRGGLRPAADIMLKSVTEQFWSHIVCVILTGMGNDGSAALPLIKEKKGKIIAEHQSTCVVYGMPKAAIETGLVDKIVPLSEITEEVLSML*
>Syntrophomonas_wolfei_subsp.metylbutyratica_JCM14075T_01327_hypothetical_protein
MKANLIKVNHLLEIEMEKNNNEKEYLPSRLEEINDNYWYISMPMRKGVYITMRVGQKIRIILRYKKTMFAASTVITARRRGHIPVLIVNKPENLTAINQKRTYVRISVAIPVAFRLLLADGEKGELLQGVTGDISAGGMLLLSSQDMEKDQKIEVEIKLPDNDSIFCQAHVVRILDKASEEGSNNKYAIEYDDINEGQRDRIFRFIFDKQRDWLRKGVTE*
>Syntrophomonas_wolfei_subsp.metylbutyratica_JCM14075T_01328_Flagellum_site-determining_protein_YlxH
MRDQADRLREMALNLKQEIEAEIRRDLRHSRVVVISSGKGGVGKSTLALNLSINLCTRGMKIILMDADMGLANLDVMLGLVSKYNIYHLLQEKKTMEDITIFGPEGLKIIPGGSGISELANLKDTELKRILVELRKLDGAYDYMIIDTGAGISKTVINFLLAAEDIIVITTPEPTAITDAYSLVKNAAKNSFIGNIYLVVNKVANDSEGIMVAEKFKLVCQKFLSCEIKPLGHIVNESLIGEGIRRQKAFVQIYPR
STATRNINTIADRLIEASGHKSPGLEAKRGGIRYFIEKVAGLVK*
>Syntrophomonas_wolfei_subsp.metylbutyratica_JCM14075T_01329_Flagella-associated_GTP-binding_protein
MIIKKFIANDFKGALKQAKKEMGSDAIILHTSKVRRGGIFSFLFSPQVEITVAVDESLQVNSDRLRKPVSSTAVSSTAAAAEPNRPVFSPQDANPGAQAIAQSAPGELLQELQTMKNLINDVKVKIYEVEQIKGISGEVQAFYETLINNQVEKELALKIINRVESRLPKDSKGDELWVRDLCLHTVQEYINNIKPIELQREQKGALVFMVGPTGVGKTTTIAKLAANMSFLEGKEVALITLDTYRVSAAQQL
RTFAEIIGIPISVVFNPQDLGEAIKQYQEKDIIFVDTAGRSPYKDEHMEELQDFIEAANPDEIILVLSVTTDSSDLINIYQQFNLIGVDKIIFTKLDESCNYGRILNTVYHIRKPIAYLTTGQSVPDDIEVPDTLELARALLREGGAI*
>Syntrophomonas_wolfei_subsp.metylbutyratica_JCM14075T_01330_Flagellar_biosynthesis_protein_FlhA
MESRVWMNSLFKHSDLMIAILVVSIVMMMIIPMKPVLLDILLSFNISFALIILMVSMFNTEPLEFSVFPSLLLVMTLFRLSLNVSSTRLVLLYAYAGEVIQSFGNFVVGGNPVVGMVIFLILVVIQFIVITRGAERVSEVAARFTLDAMPGKQMAIDADLNAGLINEDMARNRRENIQREADFYGAMDGASKFVKGDAIAGIIIIIINIVGGFIIGMVQKGMSWDEALQVYTLLTVGDGLVTQIPALLISTATGI
VVTRSASKLSLGQEITTQLFAYPKAMGLAAVILLVLGTIGLPRGPMYSLAAFFGMLYLLFRNSTGELADEEHLEEVEEAEAIKKPENVVELLQVEKMEMELGYGLIPLVDAEQGGDLLERIVMIRRQCAVELGFVVPPIRIRDNMQLKPNNYVIKIKGSQIAAGELLLDNYLAMGPGIENDQEFLGIDTIEPAFNLPARWIDTTAKDQAELKGYTVVDPPSVLATHLTSIIKTHAFELLGRQEIQNTINFIR
EHNSAVVEELIPDLMNIGDVQKVLANLLKEQVAIRDMVSILETLADYAKITKDSDVLTEYVRQALKRQISTQYAGEEGRLTVLTLDPSLEGILRDSVQQSDFGSYLAVDPDIAQKMVDKLSLYYEDLSNKGITPVLLCAPVLRLYLKRLLERFIPHLLVLSYNEIDAGISVEVIGMVSP*
>Syntrophomonas_wolfei_subsp.metylbutyratica_JCM14075T_01331_Flagellar_biosynthetic_protein_FlhB
MEEPYRYFLMDLQLFADDGMGEKTEKATPRRREEARKKGQVFKSNDLNSAIILIAGSVASFAAFPYMVNNLKSFTVLYLLERTQQDFSNDYIYFMLLEVVIIMGKMILPIFLTTFIVALLITYLQVGFVFSGESLTPKLERLNPVEGFKRIFSKRALVELGKSLLKVIITGYIVYSVMRKHYILFPRFIDMELIAGLQVLSSIVFEMAMKVGLVFIIIGIADYIFQWYEHEKSLKMSKYEIKQEYKQAEGDPQLKSR
QRRIQRDIAMQRMMAEVPRADVVITNPTHFAVVLKYEASKMEAPAVVARGKDFMALKIKEIARIHGVVIVENPVLARTLYYNTEIGDLVPEELYQAVAEVLAFVYKQKKRVV*
>Syntrophomonas_wolfei_subsp.metylbutyratica_JCM14075T_01332_Flagellar_biosynthetic_protein_FliR
MPYALESFFFLLARLSGLFITAPILSSRMMPGRVRALIVIMLAATLAYFVPVEYSRNINTPALFIAALAVEIFIGYTIGFVIYAVLATIQLAGQLMDMLMGFSIVNVVDPQSGTQIPLMGNFTQALALLSFLAIDGHHYLLQAIAQSYAIVPVMGAHLPGGFLELLVEVGAYIFVVAVKIAAPMLVAILTSDVAMGFIARTVPQMNVFLVGLPLKIFLGLITLLFLLPIYLWVYNVLFAEFFGYLDRIIILIMG*
>Syntrophomonas_wolfei_subsp.metylbutyratica_JCM14075T_01333_Flagellar_biosynthetic_protein_FliQ
LYENIILGIANEALLVVLLLSAPVLGAALITGLLISILQATTQLQEMTLTFVPKIIVVFLVIIIFGPWMLNVITAFTHDLFLAIPSFVSI*
>Syntrophomonas_wolfei_subsp.metylbutyratica_JCM14075T_01334_Flagellar_biosynthetic_protein_FliP_precursor
LAAKEVMKTKTNIWGLSSTKILFLLFLLLLVFPGSVAAEPLQLPNLNLQIGGGAGEPQDLSAVLQLIILITVLTLAPAILIMLTSFTRIMVVLAFIRTSLATQQMPPNQLLIGMALFLTFFVMAPTFQEVNSKALQPYFKGEITQEQAFEKGMSPLRSFMFKQTREKDLALFVKMSKMERPHNFKDIPNYVLIPSFVISELKTAFQIGFIIFIPFLIIDMVVASALMSMGMMMLPPMMISLPFKILLFVLVDGW
NLVIQSLILSFK*
>Syntrophomonas_wolfei_subsp.metylbutyratica_JCM14075T_01335_flagella_biosynthesis_protein_FliZ
MIAWRKRIFPLAILALLVIAMLAGNVWAIDNMSDLNQELDKELPGNDRSSSLWLDFLKLIVVLGLIIAAAWSVIRIFGKQVDRKLQGSWLHVVDEVIMGQNRGIVLCEIGERLYAIGVTDHSISFLFEVDNPKLREEISQIDIPPGDKLTGAGLSQLKKKLGALGLRNSKPDRKDFSLLMGEQVRRLEQLSYKSMEEKRLGSKRSDENKD*
>Syntrophomonas_wolfei_subsp.metylbutyratica_JCM14075T_01336_Chemotaxis_protein_CheY
MGKRVLIVDDAAFMRMMIKDILTKNGYTVVGEAENGTVAIEKYKELKPDLVTMDITMPEMDGIIAVKEIKAVDPAACIIMCSAMGQQAMVIDAIQAGAKDFIVKPFQPERVIEAVSKALV*
>Syntrophomonas_wolfei_subsp.metylbutyratica_JCM14075T_01337_Flagellar_motor_switch_protein_FliN
MNDGILSQEEIDALLTGGSSSGTDTMDSSSEPVSTSSINLSDFEKDTLGEVGNISMGTAATTLSTLLRQKVSITTPDVSVTNPEELQLLYPLPFVVVEVSYTRGLTGTNVLIVKENDAAIIADLMMGGDGLSAQGKSLGDLELSAVGEAMNQMMGSATTSLSSMFNRRIDIAPPQLTVIDMGKESLQLSTNFHDVVKIKFKMEIENLINSEIMQIIPMEAVENIMSILMGSTAESSSAEAVMEEPPAMSLP
ETKAAAVAAPRQAETAIPSPPQEAIYSQAFVQSPREINTRPQFAVQPVQFAPLQESDMIHSPQNLDLIMDVPLDVSVELGKAKKSIREILELNQGAIIQLDKLAGEPVDLLVNGKLIAKGEVVVIDENYGIRITTIISPIDRMNKLQ*
>Syntrophomonas_wolfei_subsp.metylbutyratica_JCM14075T_01338_Flagellar_motor_switch_protein_FliM
MSEVLSQSEIDALLSALSDGSVDADLLKEEELKSKVKVYDFRRPNKFSKDQIHTMQVIYENYARTLGTYLSGQLRVPLQVELLSVEQLTYEEFIRSIPNPTILNIFSLYPLPLHGSAIMEINPNLGFAFLDRLLGGPGYAPSKARALTEIEQTVIERLSQRMLDYLEEPWGSIIQIEPSLERVETNPQFTQLVQPTEIMIIVSLETRMGDVMGMINICIPFLVLESILDKLNVHYYYSTATQERSPANIAAIRTKLENTT
IPIKVVMGKTVITVRDLLALSVGDVIPLNRNISQELDIVIGNRAKFLGKPGVFANRMAIQVTGIVEEESE*
>Syntrophomonas_wolfei_subsp.metylbutyratica_JCM14075T_01339_flagellar_basal_body-associated_protein_FliL
VADEKDSEVKPRLDINIDMKLIIIGLLVFVVAVGGSYFLMKSLMAPLMPKQEGAGKLMTGNLVEVGEFTTNINDVNGQRFLKVKVTVEVGSDDKKAQEKITQYMPVIKDSILSIISAKTVADLDVRNRNNLKAEIKNDLNAKIGANTVLNVYFTDFIMQ*
>Syntrophomonas_wolfei_subsp.metylbutyratica_JCM14075T_01340_Flagellar_protein_(FlbD)
MIYLTRLNGEKFMVNIDMIELMEETPDTVVTLNTGKKFVVRERIWQIREEIIRFRKRSFTNLKK*
>Syntrophomonas_wolfei_subsp.metylbutyratica_JCM14075T_01341_Flagellar_hook_protein_FlgE
MMRSMYSGVSGLRNHQTRMDVIGNNIANINTAGYKKSRVVFQDTLYQTMRGASRPDDSSGRGGTNPMGIGLGMSVSSIDQIHTGAPTTATNKLTDMGIDGNGYFIISDGKQEYYTRAGNFEFDTGGKLVNSNGYNVIGWISTIDPVTGDWSMDTSPGRRIPINIASFKTCSPQATREMIFSGNLNAETPFNEVQTLAFATNPNGGAAGGEFRLEFGGQYTEWIKVGSNPAATAANIQKALENL
GSIGKDNVAVSWNPTYNFYEIKFQGDLASRGVPLIGVEAGPAAAFDGGNPVYGGADTQNQTLAFATIPGGGTQGGGFTITVGNKSTGAILVGADENATAANIANALNAVGATAGVTWNVGTSTYDIAFTSDPGQVIQVHPFAAAFDGGAGTVGNREMGATSAHILTFGVANGGAQGGVFRLSYNGQNTGWIAVGADSAVTAANMRVALENLSNVGAGNIAVNWNDSSQQYEIRLQGTLA
TADVPTITVTAGPNPGFDGGAPTVGGTTQNATLAFATTPGGGTQDGGFTITLGGQTTDMILVGANEAATATNIQNALNALGIATPVAVAWNGGTNTYDIGFNADPGAAMTVTTYNTGFTGGVANIVPPAVDVYYPTDQQSIITAQEVFDSHGNKETIYYRFFKYEIDEGPPRVSRWAVDMSLNNPLFEKAPGYSASTGFESVDLDTTAPVATMSAGSAEIIRLTNLQFDDKGRVVGGTPDL
DPFILTINRAPVAAEDIVFKMDFSVMTQFAGNSFSEISQNGYPEGSLTGYTVGIDGVIRGSYDNGETRDLARVALANFQNPAGLAQLGGTIFQKSVNSGEARINPPIESGMGKIVPSSLEMSNVDLSEEFTDMIVTQRGFQANSRIITTSDEMIQELVNLKR*
>Syntrophomonas_wolfei_subsp.metylbutyratica_JCM14075T_01342_flagellar_operon_protein
LTSKIFIPPSTVIQPAPSKQDKAVKSPAGSSFNQVLTQKLAGELKFSQHARERLQSRNIELSGVDMNNIQNAVNKAREKGARDSLILMQDLALVVSIKNNTVITAVDGQHLKENIFTNIDSAVIV*
>Syntrophomonas_wolfei_subsp.metylbutyratica_JCM14075T_01343_flagellar_basal_body_rod_modification_protein
MDSSQIKWETVPWLNNETQSSGKSSKEINKGLGKDDFLKLLLTELKYQDPIEPVKDKEFISQMANFSALEQMTNLNSSFNNLSASLNSGILPGLVMQQSIAMVGREVVYADPKSGESSTGIISGVVFRDGVPYYLIDDKEITVDKISKLGAVNVGYSDALLEQILAILLKIQNSMSLGEDG*
>Syntrophomonas_wolfei_subsp.metylbutyratica_JCM14075T_01344_Flagellar_hook-length_control_protein_FliK
LTGLAILAAIPEAGKKNTAHIALPGKPSSYRSSISLFQRQLNNAGQRKDDLPTQSEAIQVCKAFTPPLKEPATDASTGSMAPAEISAPESPPLIDSELNKTDAYTTGETGLLSALAVHPDSQIALAMPNEDGLPGNLQATGLILEESREGGLNCKQGKLDGIAMAEDVLFSQAAEAGPVVEEVPAEKSLIALMSSVETTGEAGERAKPLSKAEVLAASQNSPMADTPSVASIYEDGPGSESGEDAAVLRES
FRPERTYQQDGGNKQNLSRDAASSEERGQAQEGAGPERSVHNRGNPPPPVFINRAAGIPEKSALDPAALEVLLANRQGNKAGESLSALESTASPGEDEGENKAAKAPPRMEAVPRPATAAFAREVESMTKQLPQPGEEGEKATPPEHLEEEQLKPEEGKEQGKVLSGLKTESIVAEDNSKPGSRPVQERMQFIPEKFMEQVVKKAELMIRQNSSEMRIQLHPEFLGRMTIKLVLEDGLLTARFIAEN
HQVKHLLESNLNSLRQSLETQGIRVEKTEVNVQLSNGGLFDGSEGSRQDLWEQPRFSSRYNYNGSGVDAYQASSAGDEAEAGLINSSETYGVAADGSMNFLI*
>Syntrophomonas_wolfei_subsp.metylbutyratica_JCM14075T_01345_hypothetical_protein
MTGILKKIKFLLILLSLLLLLGGGYYLLLYLNLLSAPAFLKNAPLMGDFVSRSAEQVSRLQYEEILKQKDRAMAENQKLSQTLEDKAEEMEKTRKSLADLKKKLKLSEQEDDNLKEEIAQLNSEILDLKSKQESRSSAYKDMATYFSEMKAKTAADILSRLDDEDVIGIFSQMESDTVAELMQNMDAARAARISKKMLVAVP*
>Syntrophomonas_wolfei_subsp.metylbutyratica_JCM14075T_01346_flagellar_export_protein_FliJ
MKAFRFRLQTKLEICTRQEQIAWEEMQSRLKERELLLEELLYRERRLLAVEEEIRQDSCKAQEYQALLLKREFVPVIVMQIEQAEQNLSQAEARVEEARVLLLERKRETSTLEKLKERAWQEYLWELQQEEQKIIDEIAINNHFRRDLA*
>Syntrophomonas_wolfei_subsp.metylbutyratica_JCM14075T_01347_putative_ATP_synthase_YscN
MNKAPALPLEKYLDVLDKTTTCQLKGRISQAIGLTIEAAGPRVRMGELCFLQTNYSDRQLVPAEVVGFKDKKILLMPLGNLEGIGPGSELLASGATLRVQVGMELVGRVLNGLGEPIDDRGRIKSETTYPVINQPPSPLKRKRITEVLQVGIKAIDGLVSIGKGQRMGILAGSGVGKSTLIGMIARNTDADINVIALIGERGREVREFLERDLGEEGLAKSVVVVATSDQPALVRLKGAFVATSIAEYFR
DQGKDVLFLMDSVTRFALAQREVGLAIGEPPATRGYTPSVFAMLPKLLERAGTSEKGTITGLYSVLVEGDDLNEPITDAVRGILDGHIALSRSLAAMNLYPAIDVGQSISRVMIDIASPEHIKNANRFRGILATYEEARDLIDIGAYKKGSNPRIDEAVALIDKCRSFIRQDIYEEANITTTLEDLEEAVRL*
>Syntrophomonas_wolfei_subsp.metylbutyratica_JCM14075T_01348_Yop_proteins_translocation_protein_L
LSKVIKGSNMLLFQPKVIDLTAIYELKKQAEAEKPEAAEEFSEEDLLSNDIALKQLELKELQLEAERIVDENEKIVRGLLENVREEARTIVAEALEEAEVIRQQALEEAEELWDSKRKEGYESGLKKAQEEIEADRLSAMQESQRILEEARQSKIQIMNSSEADMVRLVMAVARKVIGGELETNPRVIINILREGLNYLDNPQNVTAYVNPLEVDKVLEVMQSESFTDIGSNEIPLKVTPDKRIQAGGCILESDA
GSVDARLETRIASVEKAIQEVAPDE*
>Syntrophomonas_wolfei_subsp.metylbutyratica_JCM14075T_01349_Flagellar_motor_switch_protein_FliG
MNEEDIEQLTLEIANVRKVPTEKMDEIFREFYEMCLASQFIGQGGIDYAKEVLEKAYGLEKTMEIIGRISSSLQVRPFDFIRKTEPSHLLNFIQSEHPQTIALILAYLDAEKAAVILGALAPERQAEVAKRIAIMDTTSPEIIKEVERILERKLSSIAPQELTAAGGVKAVVEIINRVDRSTEKTIMETLEVQDPELAEEIKKLMFVFEDIVMIDDRSVQRVLREVESQDLALALKGASNEVSEKIYHNMSIRASDML
REDIEFMGPVRLRDVEEAQQRIVNIIRRLEESGEIVVARGGGDEVIV*
>Syntrophomonas_wolfei_subsp.metylbutyratica_JCM14075T_01350_Flagellar_M-ring_protein
MESFVEKLKETAGRVNSFWQNMSLNQKVLAGGGLLLILAAVVFLVISSSQGTAYEVLYTELDKKDASQIMARLDEDKVPYKLEDSGTTILVPPALKDKTRLTLASENLPRGETGFELFQESKFGETQTDKKVKYQVALQGELARSIQSLDKVKAAKVNLALPEPTLFSDNEELPKASVVVNTREEEKLTPKEIQGIINLVANSVDKLTPENVVIVDQHGNLVSENLPTADGDISETVKWQMAMKRQY
EKEKQDAIQSMLDKSLGQNNSVVRVNAELDFDNKEQKDELYSHDDAGPFVRSEEIKKESGTDTTQPVVGVPGTDTNIPQYQEVDAAEGVSTYDKSSKIRNYEINKTEKVTKVAQGDVKYDYLTVAVLVNSKATEPLKLGETEEEKIATIRNIVATACGLRENRKDESVRLEDNISVAFVDFYTEPQAEAPGGTMDQIMRSPLTPWLLAFLVVLIIILLAWWLMQRRERRRAEQEAEEEAGIDTLIQEE
IRVEDLIDTGLSPEEKERRKIREEIDRIINENPETAAQVLKTWLAEE*
>Syntrophomonas_wolfei_subsp.metylbutyratica_JCM14075T_01351_Flagellar_hook-basal_body_complex_protein_FliE
MKLGALDNIPLTGINLGQPPAVQNSVGSKETGFFNYLKEALQEVDELQKEAASSAEKLALGDESYLHNTILAYEKANLALQLTVEVRNKLMEAYQELMRMQM*
>Syntrophomonas_wolfei_subsp.metylbutyratica_JCM14075T_01352_Putative_proximal_rod_protein
LGFLNSIDISASGLTAQRLRMDTIASNMANVETTRVDNRPGPYRRQVVVFEARNATVPKKRFQKLLEEKSQAFNAANSGVRVKQIREINENEAPYRRVYDPSHPEADNEGYVNYPNVNIVEEMINMISAARAYEANAQVIEAAKSMAMRALDIGR*
>Syntrophomonas_wolfei_subsp.metylbutyratica_JCM14075T_01353_Flagellar_basal_body_rod_protein_FlgB
MLEKILESKTHILLNQAMDVGTLRNDVIADNIANVDTPGFKRREVIFEEKMKKALEGTTVQLGLNLTNSRHIQMEGSIPDLEPEVRVLEDLTFRNDENNVDIDNEMAKMKKNSINYDAVSRSMSNEIRLLRMAITGRG*
>Syntrophomonas_wolfei_subsp.metylbutyratica_JCM14075T_01354_Vegetative_protein_286B
MSKSLLEKSRAINKILQKIAGHPVNFFEIADVLCRNLECTVFIVGRRGQIGGYAFTEGAICQEIQQLIKHAERFPESFNQELLNIYETQTNIILKDGRKCLFNPDNVNCSCSARIWSITPVFGGARRIGTLIFMRDKVKFTEEDIVLIEYGAMVMANEIMRIRTERIEEEARKKASVQIALATLSYSEKEAIDHIIAELKGKEGLLVASRIADKVGITRSVIVSALRKFESAGVIESRSLGMKGTYIKVLNDYLFEELRN
HA*
>Syntrophomonas_wolfei_subsp.metylbutyratica_JCM14075T_01355_hypothetical_protein
MQSERWRSMDATPFTLRVRLVFHPTGVTIKVASHGREIGAVPLELQVEPLVLPMRSALSGTTDAPFGRY*
>Syntrophomonas_wolfei_subsp.metylbutyratica_JCM14075T_01356_ATP-dependent_protease_ATPase_subunit_ClpY
LFKLTPREIVEELDKYIIGQQEAKKAVAIALRNRYRRKLLADELREEIYPKNIIMIGSTGVGKTEIARRLARLVKAPFIKVEASKFTEVGYVGRDVDSMVRDLVETSIRLVKQEKMAAVEQKGRQMAEERIVDILLPFDGRKSKSPKNPFELLLGSMQERDEADDDSERRRREIGQRREILRQKINRLELEDEIIEIEVEEKNPSFMEIFSGSGVEEMGINLQDMLGNLMPRNKKKRKVSIAEARRILTYEESQRL
LDMDEIHREGIKRAEEDGIIFLDEIDKIASKESNYGPDVSRGGVQRDILPIVEGSTVITKYGPARTDHVLFIAAGAFHVSKPSDLIPELQGRFPIRVELESLKKEDLKRILTEPNNSLIKQYIALLSTEKLTMDFTPEAIDYIAERAYEVNSRTEDIGARRLHTVMEKLLEDLLFNSPDMAGEELIIDIDYVAERLDRIVEDEDLSRYIL*
>Syntrophomonas_wolfei_subsp.metylbutyratica_JCM14075T_01357_ATP-dependent_protease_subunit_HslV
MFQATTIIAVRKGQQTAIAGDGQVTLGQNTIMKQNATKIRRLYEGKVIAGFAGAVADAFTLFAKFEEKLKQAGGNLSKAAVEIAREWRSDRILRRLEALLIVADAEKIFIVSGSGELIEPDDGIAAIGSGGAYALAAARALNAFSELNAREIAVESLKIASGICVYTNEQISVEVIEK*
>Syntrophomonas_wolfei_subsp.metylbutyratica_JCM14075T_01358_Tyrosine_recombinase_XerC
MRVEKSASNFTLSSYKTDLSQFFAFLSQKKGINPEEVGVELINHNSVRKYLAQMQEKGLSRATMARKLAALRSFIKFLCRENILADNPITAVSTPKQERKLPRFLYTREMELLMNAPDLSMAAGKRDRAILETLYASGLRVSELTNLDKPDIDFGEDYIKVLGKGGKERIVPLGSKAREALLLYLQQGRVYLEAKGQASPALFLNKNGQRLSTRSIRNIINKYVETIAINQKVSPHTLRHSFATHLLNNGAD
LRSVQELLGHVKLSTTQIYTHLSREKIKDIHQQTHPRR*
>Syntrophomonas_wolfei_subsp.metylbutyratica_JCM14075T_01359_Methylenetetrahydrofolate--tRNA-(uracil-5-)-methyltransferase_TrmFO
LQQVNVIGGGLAGCEAAYYLAQKGIRVRLWEMRPQKATAVHRTADLGELVCSNSLKSDQPNTAQGLLKREMRILGSLLLSCAEKARVPAGSALAVDRELFAGLVSQAILACPSIELCREEVNRVPTGELSIVASGPLTSEALAADLRRITGEENLFFYDAVAPSISADSLDMNKIFRASRYGKGSADYLNCPLDREEYESFYENLINADIMAGHSIDKSLFFNACMPVEVLARRGKDALRYGPMRPVGLEI
PGSSKRAYAVLQLRQEDKAGTIYGMVGFQTRMRWGEQDRIIRLIPGLEQAEFVRYGVMHRNTYINSPKLLWPSLQCKKRPEIFFAGQITGVEGYMESAATGIIAGINAARQLQGKALLTPHKATIIGALLDFISSSSCQDFQPMNANFGLLPPMEPPIKDKVQRYLAYVERALNKMGEFSESLLN*
>Syntrophomonas_wolfei_subsp.metylbutyratica_JCM14075T_01360_DNA_topoisomerase_1
LAGNTLLIVESAAKARTLGKFLGKGYKIKASVGHVRDLPKSKLGVDVENDFEPQYITIRGKGDILKEIKDESQKAGRVLLATDPDREGEAIAWHLQNAMKIPPEENCRIEFHEITREAVKKAIKNSRPVDLSRVNAQQARRVLDRLVGYNLSPLLWSKIRKGLSAGRVQSVALKLICDREEEIKNFIPQEYWTIDAIFDAARSKNSQFTARLASCQGKKIEINNEKEVAAIGEYLQGVSFTVLKVERKEKRKN
PNPPFITSTLQQEASRRLNFFSNRTMRVAQELYEGLEIGKEGTVGLITYLRTDSTRVASSAQMEALDFISSTFGAEYVPVKPNEYKSRKSAQDAHEAIRPTSIQRTPESVKAFLSRDQYRLYQLIWNRFVSSQMTPAVYDSLTVDISAGDYGFRASSSQLKFIGYKKVYNDNEEEEPKNNIPELKKGEELALHELKPEQHFTQPPPRFSEASLIKLLEEKNVGRPSTYAPTIDTILKRNYVERQNRQFIPTELGF
IVVDLLKEHFANILDLEFTARMEGELDLVEEGKLDWKKVVRDFYEPFHSDLEKARELAQKIEIKDEEAGKECPQCGRPMLIKHGRFGKFMACSGFPECRHTQSINQELGLKCPLCQGSIVALKSKKGRTFYGCSNYPQCNFRSWDKPTGKICPHCGDAIVEKFNKKKEATQICQNPACKQKVEMGG*
>Syntrophomonas_wolfei_subsp.metylbutyratica_JCM14075T_01361_DNA_protecting_protein_DprA
MIRTEKAALCFLHAIKGIGSRSLWKIKDSFGSFENCLKSDSSRLYALLNSEIAQAIISQRQHSSILFYLERLENEGINPVSFEESSYPSSLGNIYNPPMLLYFQGNMDCIEKICLAVVGSRAASDYGRTTARKLVRQLAARGISVVSGMARGIDTEAHRGALDAGGKTIAVLGSGLNIIYPRSNKRLFHEIIEQGVVISEFPLDTNPEPGNFPMRNRIISGISRGVVVVEARAKSGALITADYALEQGRDVFAVPGPIN
RESSMGANNLIKQGAKLITGVEDIIEEYYDITIPEKELPLQEQLLFLDEQEKVVVNCLAEDPLLFDDIIASTGFSIGELSRLLLKLELAGIVKSLPGNYYVKV*
>Syntrophomonas_wolfei_subsp.metylbutyratica_JCM14075T_01362_hypothetical_protein
MLADLMDRRNSFAKKLMRQDKVDPGTFSFMEPGWWALHAVSIAGVYMLGHNLAKSIQHQHNYDE*
>Syntrophomonas_wolfei_subsp.metylbutyratica_JCM14075T_01363_DNA_adenine_methyltransferase_YhdJ
MESLSGVRLVWEGKENELIERPLEYGSRVEVVYPHPLFQSNNLFGERLDNVPQLEPASLNRMYQGDNLAVLNLLLQQGFAGKIDLIYIDPPYLSNSNYNSRISVEHQGQKYFIERLAFKDRDEDLVSYLQQIYKRLKLMKMLLSEQGSIFVHLDWHSSHYVKILLDEVFSSDNFINEIIWCYGGGSGTRRHFHRKHDQILWYGKGKDYTFNPQYRPYTEGTLQRGLTRVKGKKYKLHKEGALLQDW
WVDINKILSPTARENLKFPTQKPLALLKRIIASASNPGDLAADFYAGSGTTAEACEEMNRSWISCDSSKLAIQSSRYRLLRKKARPFQITELIDEGSEGQKQGILDLIVPEVCSYDEKQAMVKLGISRYDSPLFPFENRNFVSCLDFWELDWDYDGCFSSQLQVIREKPDYNLPLPLQLSILLPKQKEYRMAIKSYDIFGHSIIKSFAFSGCK*
>Syntrophomonas_wolfei_subsp.metylbutyratica_JCM14075T_01364_hypothetical_protein
MRSKILVIFLVAMILSLLVPIAGWYYDAEQAFTPNNLIRINVVSR*
>Syntrophomonas_wolfei_subsp.metylbutyratica_JCM14075T_01365_Stage_II_sporulation_protein_AC
MHQGKTQAREKLIQGNLRLVLSVIQRFTNRGEYVDDLFQVGCIGLIKSIDNFDLSQNVRFSTYAVPMIIGEIRRYLRDNNAVRVSRSLRDIAYKALQVREKLINDNSAEPTVSQIAQHLDVSREEVVFALDAIQEPVSLFEPIYNDGGDPILVMDQLSDERSGDEQWLEEISIKEAMKKLNDREKHIVSLRFFAGKTQMEVAEEIGISQAQVSRLEKAALKQLRKYV*
>Syntrophomonas_wolfei_subsp.metylbutyratica_JCM14075T_01366_hypothetical_protein
MQSERWRSMDASPFTLRVRLVFHPTGVTIKVASYGREIGAAPLEPRDEPVVLLMRSALSGTTDAPCGRY*
>Syntrophomonas_wolfei_subsp.metylbutyratica_JCM14075T_01367_RNA_polymerase_sigma-35_factor_precursor
MDIFEKLRNWRLYFIKLYLKKKLPRFIHYIGSTEVLPAPLREQEEMALISQLDQGIMSVKATLIEHNLRLVVYIAKKFENTGINIEDLVSIGTIGLIKAVNTFEPKKNIKLATYASRCIENEILMYLRRNIKNRVEISLDEPLNVDWDGNELLLSDVLGTENDMIYKQIESEVEKSLLWKAMHKLNNREKTIIQLRFGLADGDEKTQKEVADILGISQSYISRLEKRILKRLQREIRKIE*
>Syntrophomonas_wolfei_subsp.metylbutyratica_JCM14075T_01368_sigma-E_processing_peptidase_SpoIIGA
MAIPEVYADLSFAINFIMDFCILWATARLTGSQVVYKRIVLASFLGGIYALAYLISGMEYWFSLPFKILFSCLMIIIALYPQNWAEFRKAFLYFYAINFIVAGASIAISYLLTDKQSGANLWYLWLPGGILTALAIGIFGQKYLLRQVLPALLRFDVELRFNNYYCQGKGFLDTGNNLRDPITNRPVIVAEYQFLKDCLPEDLREVLEIKQDESAMLDVLGACSWANRLRLIPFSSIGKKNGILLGVRADEIVVNN
GSKAVFHQNMVVGIYREKLSSQGEYQFLIPAEIIENA*
>Syntrophomonas_wolfei_subsp.metylbutyratica_JCM14075T_01369_Cell_division_protein_FtsZ
MPLDFDVEMQQFAKIKVIGAGGGGNNAINRMIEAGLKGVEFIAVNTDAQALFLSRAEKKIQVGEKLTKGLGAGADPEVGMKATEETADEIKKALQGSDMVFVTAGMGGGTGTGGAPIIAKIAKDLGALTVGVVTKPFTFEGRKRNSQAERGIEALREAVDSLITIPNDRLLQVVDKHTAFNDAFKIADDILRQGVQGISDLIAVPGVINCDFADVQTVMQNTGSALMGIGKAKGENRAAEAARE
AISSPLLETSIEGAKGVLFNISGGTDLTLFEINEAAEIIHQAADVEANIIFGANIDEKLNDEVRVTVIATGFNVPRTQGSSTESTRLRGLDSPPSFLDKNDLEIPPFLRRK*
>Syntrophomonas_wolfei_subsp.metylbutyratica_JCM14075T_01370_Cell_division_protein_FtsA
VLGINRNIVVALDVGTSFIKAAMAELSQGGQEINILGVSHVPSLGLRKGNIVDIESTARSIDSCLNDLERLTGVDIASTLLGFSGSSVYAINNHAVVAVGNPSYEITQDDRQRVLQSACNIALPPDKTIVQAVERQYIVDGYDGVKDPIAMVGSRLELETTIIVAATAAIQNMHRSMQRINLQTEKIIYNPLLVAEAVLLPTEKEMGVVLLDIGGGTTEISFFEAGSLLYTSVLPIGGEYITRDLAIVLRTSLEEAG
RIKERNGVASLEVARNDVIVNVKNVQGKEIRQVSQEVVAEIISARVMEIVEMIYAELKQFACLDRIPGGIVVTGGEAELTGLVKTIEEYTNIPTRLGIPENLRGIPVDFNRPQNAVILGGLIYSSRYLNINCDNKRGIAALFDQLSRWFKELFR*
>Syntrophomonas_wolfei_subsp.metylbutyratica_JCM14075T_01371_hypothetical_protein
MWLLVLFCLMIGLLIGMQVPVFLSVVYAKYMSIAVIAALDAVFGGIRAYMEDNFDTTIFVSGFVVNTLLAAGMAYLGDRLGVQLYLAAVVVFGVRIFQNLGIIRRYLLKKY*
>Syntrophomonas_wolfei_subsp.metylbutyratica_JCM14075T_01372_hypothetical_protein
LRSKGAQISIAIVCMVLGIMLAIQFKTTESYQANLVPARVEDLTQQVQSVSEERDALVEEVLSLREKLKNVRETDQAMADLQADLQDANMSAGLIAVEGPGVIITVNDNPRELQPGDNPNFSLVHDSDLLMLVNELKTSGAEAISINGERITAKSEIRCAGTLILVNWNRIGPPFVIKAIGNPDMLESGMLIKNGYMESLKVLGIQATIEKAEKITIPAYSGSVKFTFAQPVKYEEKAE*
>Syntrophomonas_wolfei_subsp.metylbutyratica_JCM14075T_01373_Cell_division_protein_DivIB
MNKAISRKFFGISILILLVLVSLYLFLHSSFFNIEKITITGLKIVSEDEVLRLSGLSRGQNIFEINDEFVSKAIEIHPVIKKAALVRHFPRQIEVQVQERKIWALVPYHDVLLCIDKDSICIDKLQKYSLVDYPLITMDNLPPRVNLGQAVEPEGVKMIKVLYDALSIKSRKAISDFHYINKSKEIVFYTQKGTEVNFGNLERLEEKTKFVEQVFAIETELDEKGTDVLEYVDLRFKGQPVLKTRNVQE*
>Syntrophomonas_wolfei_subsp.metylbutyratica_JCM14075T_01374_UDP-N-acetylglucosamine_1-carboxyvinyltransferase_1
LNSLRLRGGRRLKGQVEISGSKNATLPIMAASLMSMGEVVLSGVPDLEDINVMSMALEILGAKVKREASVLVIDARTINNYELPESISRKMRASNLVMGALLGRFQRARVAYPGGCAIGVRPMDLHLKGLRNLGYELTEEYGFMEGKAQGIMGKEILLDFPSVGATENIIMAAALTPGITTIRNAAREPEITDLQNFLNGMGARITGAGLDTVHIEGVERLNGVEYRVIPDRIEAGTFMVAAAISRGEVF
LENVVLEHLQPLIAKLREIGVDVIPKSSGVRVIGNHKYRPTDIKTMPYPGFPTDMQPQMMALFAAIPGTSIIVETIFENRYMHVQELRRMGADIKLEGRIAIVKGKTALEGATVEATDLRAGAALILAALFAHEETIISKVEHIDRGYEKIHEKLEKLGADIRRV*
>Syntrophomonas_wolfei_subsp.metylbutyratica_JCM14075T_01375_UDP-N-acetylmuramate--L-alanine_ligase
MAFTPGKWIHMVGIAGAGMSGIARVLAQQGYKVSGSDLQVNDTTRRLEEIGIEIFKGHSSSNLKEGVDLLVASSAVPQDNIELRLAREKKIPILKRGQMLAHLANAKKAVAVAGAHGKTTTTSMLYMVLANCGTDPSFIVGGELQGSELNAKLGRGDYFVVEADESDASFLDLRPYIALITNVEDDHLDYYKSVDNIRKAFRQFVEQLRPEGFAMLYGGDAFNRSLVKDLTLNKRLLFYGEDLSND
YYFLNWESIGLGSRFDVYKRDLGFLARFELAVPGKHNALNALAAIASALELGLEMEPIKSALKNFHGARRRFQIQGQKALVTVVDDYAHHPTEIRATIDAARNFHSGRVIVVYQPHRYSRTQLLGRQLGEALINADLAIITEVYSAGEEAIPGISGEVVCQAAQNIGCHSVYIPQREEIIPYLLQISQENDLIITMGAGDIWKLGLQLLEVLPESVLKV*
>Syntrophomonas_wolfei_subsp.metylbutyratica_JCM14075T_01376_UDP-N-acetylglucosamine--N-acetylmuramyl-(pentapeptide)_pyrophosphoryl-undecaprenol_N-acetylglucosamine_transferase
MKFIITGGGTGGHIYPALAIASGLKGRQGEAEILYVGTKKGLESNIVPRAGLRFTTIDISGIDRSSMLKASRSLVKFPRSFFQAWDIIKDFQPDIVIGTGGYVSFPVVLAATFFPSKTVIHEQNAIPGLANRNLARRVDYALLNFAEAAPYMKAKSMKVTGLPVRSEILDVQRAESIKKLQLEPNLFTLVVFGGSRGAMTINQAMLEAVERWQDSQMQIIWITGESSYNEIKPQVEECLSLGKRRFLHLYPYM
FNIEEALAAADLAVCRAGAGTLSELAILGLPAILVPYPYAAENHQEKNARALLAKKAVEMVIDEFLDGDTLYKKVNELRKTPVCLKEMARNMAKEGRPNALNEILDVILTLPDN*
>Syntrophomonas_wolfei_subsp.metylbutyratica_JCM14075T_01377_Cell_division_protein_FtsW
LRAKKGPPDFILFITTMALLGIGLVMVFSSSAVTSNIRYDDAYHFFKRQLYWAILGIMAMLVIMKINYSKLKDLALPLMVISLICLVLVITPLGIEVNESKRWLGVGFLRFSPSELAKLGMIMLLARTMDQNLSSIRSFSKGVLPYLLLVALVGGLIMLQPDLGTAFAIAATVFFMLLAAGAKWSHLGAVFMAGLGAILAAIAVAPYRLERLVAFLNPWKYAGDEGYQTIQSLYALGSGGLFGMGLGRSRQ
KFFYLPEQHTDFIFAILGEELGFVGACLVLLLFLLFAWRGFRTAIKAPDTFGSLLAVGITLMIVFQALVNIAVVAGALPVTGITLPFISYGGSSLLFTLCGVGLLLNISRYCN*
>Syntrophomonas_wolfei_subsp.metylbutyratica_JCM14075T_01378_UDP-N-acetylmuramoylalanine--D-glutamate_ligase
LDFSGKRILVVGLARSGMAAIKALEKRGAQLSACDEKEARTLAPILDELGKQGIRYYAGGYPEVKRQDFDLLVVSPGVPLETAAIRQAREQGIPVIGEVELAYILKPKTVEFLAISGTNGKTTTTSLLHYILAQAGRNSLAGGNIGIPLTTLLDSISAGIIVVELSSFQLESSSSFRPYICGLLNITPDHLNRHKTMERYIEAKARIFAQQKPDDYAVFNYEDSILREMARSCPARTLFFSCERELQEGAFIDNGVI
TIKRAGSVQEIARIDEVSLRGKHNLENILAATVMASIAGVSPDDIRQALATFPGVRHRMEELGLYDDVFYVNDSKATNPESVMKALDSFNQPIILIAGGRNKGSSFSQLAGLIKQKVRELVLLGEAREEIKAAVIDAGFRNIHEVEDLQAAVSKAHELARKGEVVLLSPACASWDMFASYEQRGDLFCEMVKSISK*
>Syntrophomonas_wolfei_subsp.metylbutyratica_JCM14075T_01379_Phospho-N-acetylmuramoyl-pentapeptide-transferase
LQNYLINAFIAAAIAIVVSIIMGPIMIPFLKRLKVGQNIREEGPKGHYAKAGTPTMGGIIIITAVMLASFIMAGSSTEALAAVLLMLAFGSVGFGDDYIKVVLKRSLGLRAREKLGLQLLIAFLFGLLLILYFKRGTAILIPFTGHAVDPGWWYLPFIVFVVVSTANAVNLTDGLDGLASGVSFFVSLALAVICVMTGHYSLSVFCGALAGACLGFLIFNRHPARVFMGDTGSMALGGAVAAVAALTRSEIALIII
GGVYVIETLSVILQVISYQTTRKRIFRMAPLHHHYELKGWPETRVVRYFWGLSFVFMLLGIWSFWGVG*
>Syntrophomonas_wolfei_subsp.metylbutyratica_JCM14075T_01380_UDP-N-acetylmuramoyl-tripeptide--D-alanyl-D-alanine_ligase
MYLELGFVVDSIKGKLLAGNEKTIVSGVEIDSRRVNPGYLFFAFKGESSDGHDFLEDAWRNGAAVVVVSRGDCLTKAGPGKALILVNDTLDALQDLAAAYRRSFNIPVVAVTGSVGKTSTKDVLALCLQSRFKTLKSSGNYNNEIGLPLTLLQLDETHQVAVLEMAMRGRGELLQLAAIAQPSAALISNVEKVHLETLGSLENIARAKAEVLATLRTGDFALINGDSPLLEEAVRPYAGKKYRFGYSKS
CDFRIEETTIRNQGIEISLICLGQPEKLFLPLPSRRWAMNAVAAAAMAFLLGVSMEEIKSSLLNFNPGSQRLEIIPLPAGGKIINDTYNANTLSMINALETSRELRGKGKLAAVLGDMLELGDYEQEGHREVGRHAVKNQVDLLICIGERAAWMAEAARAAGMAGEQVKHYLSKEESIEFLKQSLSSSDTILFKASRGMQLESLAEEIRSYFK*
>Syntrophomonas_wolfei_subsp.metylbutyratica_JCM14075T_01381_UDP-N-acetylmuramoyl-L-alanyl-D-glutamate--LD-lysine_ligase
LKLVELIENLTIELQDGPLDREISGIAYDSRRVKPGDIFICISGLKSDGHLFAGQAIENGAGAVLAERQLDTEGKATLLTTPDTRSALALLAANYYRRPSKSMRVVAVTGTNGKTTTTHLIKAILEETGKKTAIMGTLYAQVGKMQREMQHTTPEALEIEKFMALCREEKADYIVMEVSSHALQLQRVAEIDFNVAVFTNLTQDHLDFHQNMDNYRAAKLQLFQMIKAEEQNFAIINIDDPWAEEIFQ
AAAIPCRSYGIKKRADYQAGELKIDLNGSSFRLQYGDKSLIINMKLIGLFSIYNALAAISFALQEGIDPGLIQSALARVEGVPGRFEKVDCGQDFAVIVDYAHTPDGLENVLRTARELGEKRLISVFGCGGDRDRGKRPLMGEIAAKYSDFCVVTSDNPRSEAPHAIIAEIIPGMDKVEKSRYAIIADRREAIRHAIHLARTGDLVVIAGKGHENYQLVKDQVLEFDDRKVAAEFLRGKVK*

>Syntrophomonas_wolfei_subsp.metylbutyratica_JCM14075T_01382_Sporulation-specific_penicillin-binding_protein
MQTTGFIVRKRIATLFFFFVIIFLLLGSRVFWVQFVKGAELSEKALQNRMRDVPVESKRGIIYDRNGNELAISVSADSVYAIPAEVCSKGQQKEIAAKLAQVLEMDEESLLRRISRNSSFEWVKRQIDPEKSKVIRKMDLPGIGLTEESRRYYPKGTLASHVLGIAGVDNNGLEGVDFYYDKLVGGTKGRIIIEHDAVNRPIPEATHKFIPPVDGANLILTIDETIQYIAEREVDKVFNERKAKSAAAIIMDVSTG
EILALASRPTFDPNDFSAYAVSNRRNYGINDSYEPGSTMKITTAAMALQEGVVNAESRFYCPGYIKVGKEVIGCPDRKPHGSQSFAQTIEKSCNVAFIQIGLELGLENYYKYLKAFGFGQKTGIDLPGEAEGILIPRASAQQIELATMAMGQANAVTPIQLLTAVGAVANEGKLMKPHLLKQVIDDKGNVIKKVEPQVVRQVISPETARELCGMLEGEVVNGTGRNAYLEGYKVAGKTGTAQKVGPD
GRYMSNEHVASFVGFAPADQPRLLGIVVVDAPKGYPYYGGWVAAPAFREILNDSFKYLEIPRYNSGKDGQSIEQKEQLIVPDVVNLPVSEASSLIKARGLRVEIDGSGTVVWQQTPRAHTKVERDSEVIIHLSPFEGGKEGAEVTIPDLQGKSMKEVARILADLGLHLVPEGYGLAYQQLPEAGKVVSSGSSIKVKFQPVGE*
>Syntrophomonas_wolfei_subsp.metylbutyratica_JCM14075T_01383_Septum_formation_initiator
MLQAGHSLARVLDTPGAIQQELQSRQSRKIRRTYKKIDCRKTWLFKGGLFFFAWALLIVFLCITISTMGYQIVGLQKDIEKLQASNQRIEYEIAQKSSLDRIELLATKDLGMVKAEEDLNFAVVIPSERRGETAVKQDSVNVANRDQEKALKKIYDNLIRLAARSN*
>Syntrophomonas_wolfei_subsp.metylbutyratica_JCM14075T_01384_Ribosomal_RNA_small_subunit_methyltransferase_H
LHIPVLLDEVISSLLGDPKGCYVDCTLGGGGHLEKLLQHLKAGARVLAIDKDMAILEQSRQRLPLPNVTFVHGDFRNLKQMLEHEGISAVDGVLLDLGVSSFQLDTPERGFSFHEDAGLDMRMNQEQDLKAWEIINFYPEEKIAQILFDFGEERYSRRIAAAITKHRKTGSIDSTLQLVEIIKAAVPAAYRRGKHPARKSFQALRMAVNSELDALREVLPQALEALKTGGRLCVITFHSLEDRMVKEF
MKNRARTCICPSQSPICTCGHQAELKIISRKPIVASKEESEANPRARSAKLRVAEKI*
>Syntrophomonas_wolfei_subsp.metylbutyratica_JCM14075T_01385_cell_division_protein_MraZ
MFLGEYQHSLDEKGRITIPAKFREEIGYKFVATKGLDNCIFLYPQDEWQLIEKKLRSLPFTRADVRSFVRFFFSGAAELDLDRQGRSVLPLNLREYAGIDRDVIIIGVGTRVEIWSIEKWTDYNENAQSSYEEIAESLVDLGI*
>Syntrophomonas_wolfei_subsp.metylbutyratica_JCM14075T_01386_acetolactate_synthase_3_regulatory_subunit
VAKQISVFLENKAGRLSHVTRVLGTAGINIRALSIADTSDFGILRLIVNEPDRAYQVLKENGFTVSETEVLAVQVADSPGGLATVLEQMHEENLNIEYLYAFIGTSGNDALVVLKVEDSEKTSEIFRQKGIKFLDESQLYRL*
>Syntrophomonas_wolfei_subsp.metylbutyratica_JCM14075T_01387_Phenylacetate-coenzyme_A_ligase
MLYWDKEKECMPREELEKLQLRRLKETVFRTYAFVPTYKEKLSQVGITAEDIRSLDDLKQLPFTTKQDLRDNYPFNLFAVPMSEVVRIHSSSGTTGKPTVVGYTKKDIEIWAELMARALTSAGATKNAVIQNSYGYGLFTGGLGIHYGAERIGASVIPTSGGNTRRQVMLMQDFGTTLLTCTPSYALFMHEAMLEGGIEPNDLKLKAGVFGAEPWSESMRREIENKLGINAFDIYGLSEIIGPGVAIE
CSCKNGLHIAEDHFLAEIIDPLTEEVLPDGCPGELVITSITKEALPLIRYRTRDLTTLERTRCDCGRTHVRMQKVLGRSDDMVIIRGVNVFPSMVESVLLNIPGVEPHYLLIVDRKGNLDQLEVQVEVSERLFSDEVRKLEELGGLIRKDLESNLGIGVKVRLVEPKSIPRSEGKAQRVIDKRNV*
>Syntrophomonas_wolfei_subsp.metylbutyratica_JCM14075T_01388_UV-endonuclease
MKQQRLMEIYHKLLERFGPRGWWPGESCLEIMLGAVLTQAVAWKNVEKAIAALKEAGLLDFSALLAITEEELADFIKPALYHRQKARRLKILLDFIAENYGGDIDLMFSEPLSQIRARLLALWGIGPETADSILLYAGNYPVFVVDAYTIRIFSRLGWVEDKCSYEKMQGLMQRHLPADTQIYNEYHALLVALGANYCKKRKAFCQECPLAEYCSAKRPAGHN*
>Syntrophomonas_wolfei_subsp.metylbutyratica_JCM14075T_01389_Molybdopterin-synthase_adenylyltransferase
MMNERFFQRTELLIGQEAMNILRASCMLVIGLGGVGSYAAEAIARCGVGRIILCDPDRVEASNINRQLVALNSTRGELKAEVMGRRLRDINPGLQLEEYPFSYSEESSAEILDEEIHFVLDAIDSLPDKIHLIKTCLHRKIPVISSMGTANRLDPLLLTIADIKDSSICPVARKLRRELRRENILQGVPVVYSRETPVKTAYSGETRLGSISFVPAAAGLLLASYAVKQLISKKSEG*
>Syntrophomonas_wolfei_subsp.metylbutyratica_JCM14075T_01391_Aspartate--tRNA_ligase
MKRTHNASELDTHLVGREVVLNGWVDTRRDHGGLIFVDLRDRSGIIQLVFSPEVKEEAFHLAEQIRSEYVIAVRGKLSLRPEATENPNLKTGKVEVYVDDMEVLSPAKTPPFYIENDIDVDENLRLKYRYLDLRRPEMRDNLLLRHRVVKCMRDFLDSRGFIEIETPILTKSTPEGARDYLVPSRVHPGEFYALPQSPQIFKQILMVAGMEKYFQIARCFRDEDLRADRQPEFTQLDMEMSFVDEEDII
VLVEEMMAEIFFKAAGKVIKTPFPRLAYDDAMIKYGSDKPDLRFGLEIVELSEMLRNTQFKVFASALQSGGVVRALNAKGCGSFTRREIDALGAMAVESGAKGMAWILVQENELRSPITKFLSEEEIKQILAATGAESGDLILFGADQAEIVARVMGSLRLELGRKMGLIAEEELNFVWVTDFPLLEYDEEEKRYQAKHHPFTSPRLEDIEIMAREPGRVKARAYDLVLNGTELGGGSIRIHRREWQEKM
FSVLGMSQEEARDKFGFMLEAFEYGTPPHGGIAFGVDRLLMLLAGRNSVRDVMAFPKTQSASCPMTEAPSTVSARQLRELALRIREK*
>Syntrophomonas_wolfei_subsp.metylbutyratica_JCM14075T_01392_Histidine--tRNA_ligase
MQVKAPRGTYDILPQDSVKWQYIEKIMRDSAESFGFREIRTPLFEHSELFQRGVGQGTDIVSKEMYSFNDRSRRSLTLRPEGTAPCARALIEHGIYSGVMPVKWYYLGPMFRYDRPQTGRYRQFHQFGVEAFGSNSPYLDAEIIILLVEILRRLGLDDYELHLNSLGCSVCRDDYRQKLLQHISPFKEQLCQDCQQRYMQNPLRVLDCKMETCHQAIRGYPIFYDHLCPYCQEHYSRVQAALQANG
VSYIHDNNLVRGLDYYSNTAFEIHLPGIGAQSAVGGGGRYDGLVKELGGPDIPGIGFALGMERLLLALDMLGKREFSKPSLDVFVVVMNDAYEMQGLQLVSELRRRGIRADKDYGGRSGKAQMKYAGKTAARFVVMVGEEEVEKHFWTLRDMQSKEQRQLGQEELIDSIRSVIETK*
>Syntrophomonas_wolfei_subsp.metylbutyratica_JCM14075T_01393_P-protein
MACAILGPAGTFSEEAAQLYWSDKEDYVLARSIPEIFVLLNTKQVAEALIPMENSRAGSISLTMEALEANNVYIKGEISIPVRQHLMAGKKLDLADIELLVSQPTALMQCQHFINSYLPETRKEITTSTTRAAEIVRSEERRAACIGNQLACNLYGLEMIQNNIQDEDNYTRFVHVRSGQAPKVEKEKSSIIFTLPDRPGALYHTLEIFNRRNLNLSKIESRPGKLAKGSYSFYIEVENGSNRVGIEELLKELQKR
CTLVKYLGSYSSCTRGKTRQE*
>Syntrophomonas_wolfei_subsp.metylbutyratica_JCM14075T_01394_Phospho-2-dehydro-3-deoxyheptonate_aldolase
MKNLLAQKDGELLVSRICQSESTQIDVKGCLIGGEQVMVIAGPCAVESEELLKETAFKVRAAGAAMLRGGAFKPRTSPYSFQGLGEKGLKLLARVGEDMNMPVISEIMDSEDIGLLADYADILQVGARNMQNFALLRKLGQLKKPVVLKRGMSASIEEWLLAAEYILAGGNSQVILCERGIRSSEPWTRNTLDLSAVALVKELSHLPVMVDPSHATGVRSLVSPMARAAIAAGADALMVEVHPQPE
KALSDGDQSLTPRGFSTMMRELGPVARAVGRRLR*
>Syntrophomonas_wolfei_subsp.metylbutyratica_JCM14075T_01396_Beta-lactamase_hydrolase-like_protein
MILKGLELSSMMVNCYIIGCEETKEVAVIDPGGNARAILNLLEEDGLKTQYIINTHGHIDHIGANQAIKDATGAKILIHENDAKMLVNPVSNFSFMMGSKVVSPPADQFIREGDIIKIGNTVELEVIHTPGHSPGGVCLKTGNIIFVGDTLFQGSIGRTDFPGGSYKQLIQNIKEKLFRYNDDTICYPGHGPATTIGFERMHNPFLT*
>Syntrophomonas_wolfei_subsp.metylbutyratica_JCM14075T_01397_D-tyrosyl-tRNA(Tyr)_deacylase
LRAVVQRVSFSEVRVEGKLRGRIKQGLMVLLGIKKGDSKADGDYLLDKIVNLRIFPDEEGKMNRSLLDIEGEILLVSQFTLYGDARKGRRPSFSEAELPELAEPLFDYCVDGLRRRGVQVETGIFGAEMVLSIENDGPCTILLDSEKLF*
>Syntrophomonas_wolfei_subsp.metylbutyratica_JCM14075T_01398_GTP_pyrophosphokinase
MNMRCEALIKRVSEYDPDTDFALIEKAFRVAEQAHQGQKRISGEPYIIHPCEVALILADIEMDTPTICAALLHDVIEDTQVSYLDILKEFGEEIALLVDGVTKLTRIDFKSREDAQAENLRRMFIAMARDIRVIIIKLADRLHNMRTLSYHGEAKQKVIARETMEIFAPLAHRLGIFRFKWELEDLAFYYMEPEMYYEVAKRLKQKRRERENYVNDLVEQIEVGLHQIDIDADIKGRPKNIFSIYKKMIKQGK
DVDEIYDKIAIRVLVEDVRDCYGVLGIIHTMWKPLPGRFKDYIATPKANMYQSLHTTLIGRGGEPFEVQIRTAEMHRTAEYGIAAHWRYKEGYNPRERKFDEKLSWLRQILEWQQEVKDTSEFMESLKIDLFDDTVFVFSPKGAVFELTKGACPVDFAYRVHTEVGHRCTGARVNGRIVPLDLPLENGDIVEILTSKSSIGPSRDWLNFVKTSSAKSRIRQWFNREKRQENIIRGHDLLEKELKKQHL
NPKELLKEDKLLEVVKKLGFNAVEDLLAAIGDSALSALQVVNKQKELYFPDLLLEDIAAVPEESKAAVKDRVTTALRVKGVDDVAIRLARCCNPLPGDQVLGYITRGRGVSVHRKDCPNMQNYLQNEKDRILEVEWADTSGIYYVELEVKAFDRARLTTDVMNEIAEVRIHINSVFSRATKNNQAVINLKLEIKDMSRLQAVIQRILNVRDVLEVRRVLPGEMRGESLAGRSSKG*
>Syntrophomonas_wolfei_subsp.metylbutyratica_JCM14075T_01399_Single-stranded-DNA-specific_exonuclease_RecJ
MRSWINGKYDETIVEKIAGEMGISHLLAKILVKRGMGGTEEASRFLYGKLEDLSDPFQLKDMDLAVARIEQAIAAGERIVVYGDYDVDGVCSIVILLECLQLLGANCSYYVPDRFLEGYGLNGDAIEKLASEGCQLLITVDCGISSLAEGQLAGKLGMDLIITDHHTPLDELPLALAIINPKLGGGAGSSDLAGAGVAFKLAAALCQQKIEQEELYKWLELVALATVADIVPLRGDNRIMVKYGLKKIEQ
TSRKGLRALLRETALEGKTLRTWHIAFVLGPRLNSAGRLNSARNSIELLLARDEERAASMAAFLCRVNEERKRIEGTIFQEAMAEIEQNIDLEKEAILLLGGENWHDGVIGIVASRLSEHYRRPCILISWEGDCGKGSARSIAGIDIFAALDYAACFLQQFGGHKMAAGLRMERRNFSAFKEKILEWAAAQELSSLKPSERKIDAEIKPVDINEALLQELELLQPCGEGNPVPLFLLRRVAINSAVLIGKGREHF
KLKTEPDDLEGIAFNQAQYMDLPWRNSCQDLLFELAENEYQGQKRIQLKVKDMKCSFMPDRVGTVQNKLQRAAELLTASMEELKEGRPVLFIYPTSRILKRSRVVLENYFHRSALQALHGLMNTGERRETENMFRQGRPRIYLMTRAYCRYYLRRNTFPQNLRLVLELWAGKMKELEYDQELRQEALYPPTAELCWQRTERVADSPSPTWIYTNRKKRTVESLRRRWPQAVVQTGIRELEELRSL
YQSGLKAGASVFLSDGVFAGIFPQKRLNARLFFADVPFSIYEAFSVRGQMAADSEEPVLALFDSQGIKSNWDYLNQAYPDLPEVKAWFRQLIELRQNPIILDLNNWSAAAKKFSRLKLLSVLLTLSDLDLCRWRKEGSIIEIKLLKPDSSTINLANSPYYLEGQAEKKHYQNLVSQIEKSLKW*
>Syntrophomonas_wolfei_subsp.metylbutyratica_JCM14075T_01400_hypothetical_protein
LTKLRHMYPGANSCRGFYSFYQYMLWPEGERKIILKGGPGVGKSTFMEKIAEAFSSHGLDMEYHCCSSDNQSLDGIVIGQKICILDGTAPHVVDPLFPGAMDEILNLGDFWDSRIIKEHKNEVIKLGQEISRCFSRAYLRLQEAAAAYEEWQSYYKEARDPAAVKRNILALSQEILQDCSVSPYELRHLFAAAITPAGPVTRIESLVDHDYSLYALKGSPGSGSKELLKHVAYMLKLQQYYGEVYHSPFEP
QEIDLIILPEQKTALLDFSSYIINYGDKISAKPKRLLDFDELIHKSLIDPHAGRVFSARNRFDESLQGAVEFIRKAKQFHDELESFYIPAMDFTALENLRTELLQQLMAEL*
>Syntrophomonas_wolfei_subsp.metylbutyratica_JCM14075T_01401_LexA_repressor
MKFKERLQYLRLEKELSEIQLSKKVGISESVIRLYERGKLRIDLEHLHKLAAFFDVTPGLLLGTAFPHLPPEEALESHRVVNELLEIRNEERKDDEDSLPYYIDSLTQTAAQSNKISANNKAPNWQAALRAGVVAVYNRIVSARSEANQEFIEDYWPVNTTVMQIYGQDISNYFYLRVNGDFMEPTIKNQSIVLVKRQAQVDNNDLAVVLYEQSPAAVKRLNRYEDKIILLCDNNAYPAQICEQEEYIILGK
VLWKPK*
>Syntrophomonas_wolfei_subsp.metylbutyratica_JCM14075T_01402_Molybdopterin_molybdenumtransferase
VAEFLKVVSFQEARQKIIEGFPRQGKERVYLTEAQRRVLASDVKSPEDLPAFHRTVVDGYAVLAEDTFGSSESLPAYLDYVGEVKMGQEAGLSLQKGQCCWIPTGGMLPAGSNAAVMVEYTEKLGEDTVLVYRPVGPWENIMQKGEDIKKEEVIFSEGSFLRAQDIGLLASLGISKLLVFKPWRVGLISTGDEIVPIEETPSTGKVRDVNSHALAAAIRDCGAIPHNYPLVMDDFKVFKNAVELGLRE
NDLLIMSGGSSVGLMDVTLDVLLSFPGAEMLFHGIAVKPGKPTLAVKIGDKMVIGLPGHPVSALMMFHIICAPALSPYPPLSIDACLELNLSSQAGRDDFVPVELREEKGEKLALPLLGKSGLMNILSRASGFIHIPYEKQGLVKGENTRVYLFM*
>Syntrophomonas_wolfei_subsp.metylbutyratica_JCM14075T_01403_Molybdopterin_molybdenumtransferase
MQRKIYIENIPREQALQKFLQALEQENYFKIESEEVDVLSSHGRITSQAIYAHRSSPHYVASAMDGIAVKASSTFAANEVNPVSLKKYQDYVEVDTGNYVPRQFDAVIMIEEVNFLDGYAQIIKPAVPWQHIRSVGEDLVTHDMMVPVFTRIGPYELASIHTAGVEKIPVVKKPRVAIIPTGTELEERASDDMEAGKIVESNSRMLSAFCEEWGAIPLRHDIVVDDPDLLRQAVRKTKDNADILVICSGSSA
GREDYTSTIIEEFGQVLIHGVATRPGKPAILGIIDGKPVIGVPGYPVSAQLIFQLFARPVVFKKQGLVLPDADKVQAHVSRKIASSMGVDEYIYVNLARIKDKYMAYPLNRGAGISSILVKADGVICIERGNEGLQAGESCPVSLLRPREKVDHSLIAIGSHDMTIDILGNLLQKQYAIRLVSSNVGSMGGIMALRRQETHFAGIHLLDAKNGEYNRSYLKKYLPRESWILLNLVKRQQGLIIKKGNPLQILGL
QDLLRSDVRYINRQKGAGTRILFDYLLAREGINNEAINGYNREEYTHLAVAAAVKNDACDTGMGIYASARVLDLDFIPLSEERFDLCILPDLIGERELDLLLNTINSPEFRQQVSDFGGYNLEDSGQIIHYNNQF*
>Syntrophomonas_wolfei_subsp.metylbutyratica_JCM14075T_01404_Formate_dehydrogenase_subunit_alpha_precursor
MKVTRRQFLKVTGATAAGFALVDLGFDLQATASAAKELRIKNVTPTPTVCPYCASGCGLVIYAERDANGKFVKLLSVHGDPDNPINQGGACSKGAAMFNLREIYDERTGEQMINPKRVQKPLYRAPGSDKWEEKDWDWMLARIAERVKETRDSSFIHKEKLEDGNEIIVNRTERIASIGGSGLDNEECYILSKMMRALGLVYLETQARI*
>Syntrophomonas_wolfei_subsp.metylbutyratica_JCM14075T_01405_Formate_dehydrogenase_subunit_alpha_precursor
MTNHIVDLKNTDCALIMGSNAAENHPISMRWLLKARENRGAKIIHVDPRFTRTSARSDIYCPMRSGTDIAFLGGMIKYIIDNELYHKDYVVNYTNAAFLVKEEYGFEDGLFTGYDAEKRKYDVSSWDYQVDEAGLPLKDLSLQDPRCVFQLMKKHYERYDIETVCKITGTPKNKYLEVLKTFCATGAPDKTGTIMYAMGITQHTVGSQNVRSFAMVQLLLGNMGRPGGGVNALRGENNVQGA
TDMALLFHIIPGYIDSPISNAANKDLLSFIRAQTPGAAKLPFTNLEEFRALVKAGFPNSGFKINTSKWVISLLKAWWGKAATLDNDFGYHYLPKRDGNKNYSHIALFEAMHRGEIDGLFVNGTNPVVGGPNANKEQEALTKLKWMVSVDLWLNETADYWTYQGWERTVKNPDVPTYTPADIQTEVFFLPAAAVYEKEGTAAQTGRWVQFRWKGADPVGESKADLWIYNELGKRIKKLYAGST
KPQDEPIVNLDWNYDDEHGHDDIMKVALELCGYNVADGTPVEGFAKLSDDGSTACGCWVYCGAMTYKDGKIIYKTQNRDNKDNSKTGLGLYSNWAWAWPMNRRIVYNRCSVQPDGMTPWPGDEERQLIRWDPTIPADPKKPEALGSWVGDDVPDFLKPTSPDKPAFTGPFIMRPELKGCLFAPKSAMKDGPFPEHYEPWESPTENIMNKQNLNPATKIWEPDKQGTTDKYPIIGTTYR
VVEHWQTGALTRNLPWLAELMPDMMVELSEQLAKEKGIKNGHKVVISTTRGDIEAVACVTKRFKPFIVNGKTVHEIGLLWHYGFKGYATGDPANRLTPHIGDANTMIPEYKAWLCDIRRA*
>Syntrophomonas_wolfei_subsp.metylbutyratica_JCM14075T_01406_Formate_dehydrogenase-N_subunit_beta
MANLTNLYDVSKCTACRGCQVACKDWNQLPAVIEPFKGNYQTHKDTNGDTFTIVKFMEYEDPVDGVQWNFLKYQCMHCFDPACMKVCPRQAYSKTEWGATFHDPDKCIGCQYCTYACPFEVPKYREREDKITKCTLCVDRVEAGLTPACARTCPTGALTFGPRDELLQVAEERVKFLRVNGFPNATIYGKLELGGLNKLYVLTDTPDKFDLPVNPKVSGNTVFWQDWIQPYFGWLIPL
ALAGSATSFITTRILANKHGAHGEGGHE*
>Syntrophomonas_wolfei_subsp.metylbutyratica_JCM14075T_01407_Formate_dehydrogenase-N_subunit_gamma
MALIPEYRADVPRVERFNFLARFSHWGHTVTFLLCLFTGLVLFLDGVNWLAAIFGGYAGAGLVHRIAAVGMTIVIVIGGVFGFKGVIEWIKDLLRFGKNDIIFVMKFPLEFLGFPVKMPPQTRFNGGEKGNSMLTPTSVMLLVLSGYIMWFPAIFPGGLVRVAYWTHDIAMVLATFMVCMHGYLGSFHPGSGESFWGMWKGTVRADWAQHHHAIWYEEAYGDKDQDKTE*
>Syntrophomonas_wolfei_subsp.metylbutyratica_JCM14075T_01408_formate_dehydrogenase_accessory_protein_FdhE
LFEHKVPVTLPDGYVDFFKSLESWENEELIKLRTVYAPSQLDILRMLDETKKPLILQVNPHIEADTYREVFERLLDFLNSTRPEIGPQLALIKNNLDKVNFENIISSFIAMQTTEIEKAAETAAIPGELFFFVADHALRPFLRIFAEPYRNDLTGERFYWDFSSTCPVCGSKSHFSRLQSENGQRFMFCDRCFSEWKVRYLFCVHCGHDRPGDIRYLNVENDEAYKLYLCDNCKGYLKTFDERPGGDMVD
LFIANIETIYLDLLAQEKGYTSHDE*
>Syntrophomonas_wolfei_subsp.metylbutyratica_JCM14075T_01409_Spo0E_like_sporulation_regulatory_protein
MAAPCAGKGNNIAGEDNRELIEWIELLRRRLETLAVLRQNYTDPEVLATSQALDEALNEFYYRSEAEGAQRVQSQVWQVKKAQKSAIG*
>Syntrophomonas_wolfei_subsp.metylbutyratica_JCM14075T_01410_hypothetical_protein
LLVNNNGAVAMKANLSALQQLAKTKNWTTPELARRLKVDYSYLFRIMRGEKRGGAKLWSGIYFLCKKEGLSIEDYIFLDEE*
>Syntrophomonas_wolfei_subsp.metylbutyratica_JCM14075T_01411_hypothetical_protein
LLRGLVDNAPSATIIKRLAEVAWNDIAIQDLLAAAGYLEPDNKRAPDGLSYSPQLQQWEKVIEEAVRYDIPPEAAVDLIRSLGKSMKKIKNE*
>Syntrophomonas_wolfei_subsp.metylbutyratica_JCM14075T_01412_Transcriptional_regulatory_protein_ZraR
VLEINLEAINGALVESPYMALVTVNKDGYITLMNETFLDLLGLKKEQAIGKHVLEVLPHSELPEILQTGRIDKADIWPINGNDTVVTRFPLIKDGEIVGALGQSFFLDMSGARILMQRLQETEEEFQAFSEALIENPYLVYVAVNKEGLITLMNQTCLDALGMEKKEVIGRYILDIIPESRLPEVLQSGRIHEADIWSVNGRDTIVTRLPIIKNGKIIGAIAKSLFLDVSGAKMMLQKLQETQQEFQALFEALI
ESPYMVYVIVDKDGFITNMNKTYLDALALEREQVVGKHILEIIPESKLPEILNTGRIDKADIWPINGRDTIVTRLPIIKDDKIIGAIAKSLFLDMSGARILMHRLQETEKEFRAISEALIECPYMVYVIVDKDGIITSINQTYLDALGMEKSEVVGKHISKITPTSLLPEVLKTRQIDLADIYSINGQDTIVTRLPIIKDGEIIGAIARSLFMDISGAKILMNKLQETKKELNIYREELRQGYQAKWQFKDLIGQSPAFL
KVKSVAEQVSHSVSTLLITGESGTGKELFAHAIHNSSDRSSYPFVMINCAALPDSLLESELFGYEEGAFTGARKGGKPGKFELAMGGTIFLDEIGDMPLNMQTRLLTVLQDRMVERIGGTRPIYIDVRVIAATNRDLEELVANNKFRQDLYYRLNVVRLDIPPLRERSDDIPLLAKNLIKKINKKLGTKINKISYKTIELMQNYAWPGNVRELENLLERALNLADMNRENSITIKHFPLMVENAYFRDEPN
PSTLPDAIEQLEKQLIIQALEKTNGNKMQAAKVLGIYTSALYRKISKYGLDEL*
>Syntrophomonas_wolfei_subsp.metylbutyratica_JCM14075T_01413_putative_3-hydroxybutyryl-CoA_dehydrogenase
MAINKIAMLGAGTMGQGIAWALAAVGKKVVLYDIKQEFVDRAIAAIEKSLAKAEERGKAAPGTKDTVVGNITGTVNFDDLKDVDFVIEAVFENMEVKKDIYTKLDALLAPEVIIATNTSSLSITEIAAITKRADKVVGMHFFNPANVMKLVEVIPALQTSEETVQTVIDLSVEIGKKPVKVKEGPGFVVNRILVPTINEGCFILYEGLATAEDIDTAMKLGAGWPMGPLTLCDLVGGDIALAVMNTLY
DETGDPKYRPSGLLKQYVRAGWLGMKTKKGFFNY*
>Syntrophomonas_wolfei_subsp.metylbutyratica_JCM14075T_01414_putative_enoyl-CoA_hydratase_echA8
MSYENIILEKQDYLAILTINRPKALNALNKDTLSEMRKALEDIAADQAIKVVIVTGSGDKAFVAGADIAYMQPLSASEGREFSDYGEKTMRMIELIEKPFIAAIKGFALGGGCELAMACDIRLAAENALFAQPEVGLGVIPGFGGTQRLPRLIGEGRAKELTCTADTINAAEAYRIGLVNHVYPVDELMEAAKKMAARIASKGTLAVGFAKYAIGKGLQTDLDTAMGIESDMFGMCCSTADQKEGMGA
FLEKRKPVFKGK*
>Syntrophomonas_wolfei_subsp.metylbutyratica_JCM14075T_01415_Acetyl-CoA_acetyltransferase
MMKEIQDVVIVSACRTPIAKFLGSLKDVQAIDLGIIVAKEAIKRAGIQPDIIDEIVMGEVYPHMQGSLPARQVGMAAGLPVRSNACNVNQNCASGMRALDIALNHIQLGKTDVALVVGVESMTNAPYMIPKARMGYRMGPGSIEDAMLHDGLIDRLVPGHMGVTAENIAEKYGITREECDQLALTSHQRATRATQDGTFQREIVPVELKSKKGVKIYDKDEHMIPDANLEAMAKLPSAFRKGGV
VTAANASGLNDAAAAVIIMSKAKALELGVKPLMKLINICGEGVAPEVMGLGPAVAIPKCLKEAGMKYEDVEYWEINEAFAPQFIGVGRMLKEEFGIEMDLSKVNANGSGIGLGHPVGCTALRIVVSLYYELEKQGKTVGGASLCVGGGPAYCSLWTRDI*
>Syntrophomonas_wolfei_subsp.metylbutyratica_JCM14075T_01416_Acyl-CoA_dehydrogenase
MDFRLNEEQEMMKKMAHDFATNEIAPYASEWDNNHIYPEDCIKKMHEVGLMTIGVPAEYGGAGLDHTAQNIVAEEICWGDAGVGTVMVASTLLASDPVLVAANHEQKKEFYGRLLDGELAAFCLTEPGAGSDAGGIATRCVKSGDEYILNGSKQFISNGGTAGVYTVLATLDKKMGTKGMCAFIVDRNTPGITVGKTEDKLGIRTSNTTEVIFEDVRVPAKNLLGTEGQGFYIIMKTLDLSR
ASIAAMATGVSQACLDASLEYSKLRTQFGKPICSFQAIQFKLADMAMRIEASRLLYLEASSLQDMNVPRFSKAASLAKAYAGDTAVFCATEAVQVFGGYGYTKDYPVEKYYRDAKIFQIYEGTGEVQRLVIAGDLLRG*
>Syntrophomonas_wolfei_subsp.metylbutyratica_JCM14075T_01417_Potential_acrAB_operon_repressor
MEANKKSRTERKKEETRKKILAVAMDLFKRQGFDKTTVDQIAMEADVAKGTVFNHFPEKEAIIYEHIQSMIREQTPAMLEYLDKLPDTRTRLIAALQKTLEWMHIDLSNDLFERHFIYKMSQWVHAIKHLDRNVSSGFSTVMEHIFKLGIESGEIRPDWHPFVVGGLEVSHFLSALLWVNHPELISINESIENNVDCFLNGVKNELHDS*
>Syntrophomonas_wolfei_subsp.metylbutyratica_JCM14075T_01418_Phosphoenolpyruvate_synthase
VRSSATAEDLPYASFAGQQDTYLNVKGREAILQSVKKCWASLFTDRAVIYRLRNGFDHRQVYLSVIIQRMVFPEASGVMFTADPATSNRKVLSINSCFGLGEALVSGLVNADLFTVRDARITDKVVAHKKLGIYALEEGGTETRGIEPEQQHIQTLTDEQVLRLEQIGRKIETYFACPQDIEWCLSGDKFYIVQSRPITTLYPVPEVSDGKNHVFASMGHQQMMTDAIKPLGISFFQLLSDNPMPQAG
GRIFMDISAMLSSAIGRKTMVSMRGDGDILMENAMFKVMERKDFLKTLPRKLPGNNAMVKGALPLLFHIVKTSRANDAARVPKEIGRSDALIRSLQEKIASVSGDELFDLILQEQKEFQALSSDPENFAVFMAGQQALKWLNKNMAKWLGDVKEGDALSRSAPHNITSEMGLALLDVADVVRPYPAVIAYLEQADDNTFFEGFDGLPGGGVVRDSIQAFLEQYGMRCPGEIDITRPRWSEQPTAL
VPAILSNVKNLQPGAHKTRVEQGRLEAEQKERDLLARLERLPGGKRKAKKTRKKISILHNFIGYREYPKYFIIRRWQVFKEALMREAAVLVQKGVIQNREDVYYLSFEEFRDAVHSGHLDYSIIAARKEEYAAFEKMTPPRVMTSEGEVIFGEYEAVDIPPQALVGAPVSSGVIEGPARIVLRMEDANLEDGDILVTTFTDPSWTPLFLSVRGLVTEVGGLMSHGSTIAREYGLPAVAGVENATRLIEDGQ
RIRVNGTRGYVEIL*
>Syntrophomonas_wolfei_subsp.metylbutyratica_JCM14075T_01419_hypothetical_protein
MGEKGFFFNWSEAFTAGVEAVGGKGWNLARLERYGFTIPLAVF*
>Syntrophomonas_wolfei_subsp.metylbutyratica_JCM14075T_01420_Phosphoenolpyruvate_synthase
LYIQEELIAQLKGMGILSKPLAVRSSATCEDSARASFAGMHDSFLNVRGTDNIILAVKECYASLWTPRAVAYRRKMKVGDDEVFQAVVILELVEAVAAGVGFTCDPRSGREDVVLVNANFGLGESVVSGAVEPDEYCLDTKLEILERRMGRKENQTMARPDGGTKLIASPGSSEKQVLSDENGGDVLVAPTTDPGWTPLFLRASAIVVEPGGIGSHGAIVAREYGIPAVLNVLGAMSLIKDGQNVVV
DGDEGKIYMQ*
>Syntrophomonas_wolfei_subsp.metylbutyratica_JCM14075T_01421_Tyrosine_recombinase_XerC
LLTSLYSNFVDRFSYAMCIKSCIIPYFKEFKLALKDVTPKHIQDYYQYELNEKGLSASTVIHRHANIRKALQYAYKVGLIAFNPADRIERPKTTKFVGSIYDAGELEALFAVVKNKPIELAVILGAFYGLRRSEIVGLKWNAINFEKKTLTIKHTVTEVRVDGKAIIVEKDRAKTKSSHRTLPLVEPFEKLLRQLKEEQERDQKVCGKDYCREYFGYIYVNELGERVKPGYVTQNFALTLKNHGLKKIRFHDLR
HSCASLLYANGVSLKEIQEWLGHSDISTTSNIYTHLDFSSKVASANAIIGVYPS*
>Syntrophomonas_wolfei_subsp.metylbutyratica_JCM14075T_01423_Sigma-W_factor
MQSEQELVKKSMEGDTQAFEKLVLQYQNKVYALSFRYMGNEDDAYDMSQEVFLKAFRSLKTFKGNSSFGTWIFRITTNVCLDELRRRKRRIVPLSLDEAAVTSRGIEVEREIVDPSPPVDRVYEQKELSQYIQGLLDQMKPEHKAVIILRDSMDLSYEQIAYVLNCSVGTVKSRLSRARSILRKQWVERELLP*
>Syntrophomonas_wolfei_subsp.metylbutyratica_JCM14075T_01424_mycothiol_system_anti-sigma-R_factor
MKCEQVSELLSAYIDEMTSEKETKALEAHLAQCADCRKELDGYRLLRGIMQQLESPLPPDDFSTVVQKRLRENKNKIFSATTIERPKKTGWIAAVLAGLALASGIYASSYLSIGDFLVSWQDKKEQKKPRIAIEDIIERFQKWNKEEDNSSREAAEPEAKIASNPSAKVSVGVGAENTREEVELKTAGDDSYSSHIMVKELSTALAQVIGIADEREIKYSMTSGSYGSSEARRVVLEVEPQEVPQLLMELEAIG
RKLDVADLTEASGQDEALQASDLDEKPLLPEKSEFSDNEKLSMVKIENNEEAGEDRKEELAKEKAASAPDDKMLFNSEKGEDSKDIESIKEENKPEGTKVNLDILIIQEK*
>Syntrophomonas_wolfei_subsp.metylbutyratica_JCM14075T_01425_DNA_polymerase_I
MADKFMLIDGNSLLYRAFYALPLLHNSAGVYTNGVYGFLTMFNRVLAEIQPTHIAVAFDKDRSTFRNELYSEYKANRSAPPEELGGQFQLLRDVLAALNIEYLEMQGYEADDIIGTLSKKAEEQGLKTVILTGDGDALQLVSEQVDVLLTKKGISEMELYDPQGVKDKWGVEPEKMAEIKGLMGDASDNIPGVPGVGPKTAIKLVQEFASLENLYENLPQVKGKKLVEKLEQNREQAFLSRQLATI
NRDMDLDFPIEDYVMREPHYPELLELYRSLEFNNFLKILQEKELKKGMAPVKQEIEVLFLEREEDVTALLEEIKQGESVALYFQSNYHHPMWASLQGVFLEYGERVYQLKLEAGDDYKLQWLKTLLESAAIKKYLHNAKFVQVLLLRHGIELGGVAGDTMLLAYVDDPAFEGDELDACLKKHLQLNIAENNLAALVSRIKELYFQLEEQVTEELRELLQQVEMPMSEVLARMEFCGVKVERSTLTRI
SEELASGIAVHEERIFELAGSSFNINSPKQLGKVLFEDLGLRVIKKGKTGYSTGAEVLEELYQEHEIIAHILNYRQLSKIKSTYADALQELIHPETGRVHTIFKQAQTATGRLSSVEPNLQNIPIRMEEGRRIRQAFVAPGPDWLIMAADYSQIDLRSLAHISGDERLIEAFKSGVDIHTRTAVEIFHVPVEWVDSELRRRAKAVNFGIIYGISDFGLARDTGVSRKEARKYIDNYLNSYPGVKKYMEDIVEFGRR
HGYVETLLKRRRYLPELNARNKMVQAFGRRMALNTPVQGTSADIIKLAMIKVDEEIRKRRLQARLLLQVHDELVLELPRTELEEVSGLLRDCMENACSLKVPLEVSLKTGPNWYEME*
>Syntrophomonas_wolfei_subsp.metylbutyratica_JCM14075T_01426_Formamidopyrimidine-DNA_glycosylase
MPELPEVETIKNNLQEILPLRIKELELRREDILRCQDYALKELAGQSIEEASRRGKYLILAVNNGLFLVFHLGMSGRLYIQEEENTILEPHVHVIIHLDKRLKLLYQDARRFGGLWLLKDIDSFFSRLGKEPLSEEFCPPYLEQVLQGRQTAIKNLLLNQNLISGLGNIYADEALFMAGIRPDRQAASLSVREIEGLCCGIKEVLTKSIKYRGTTFRDYRDGKRQPGEFQNHLQVYGRFNQACPNCGQPLKR
TRIGGRSSHYCEKCQH*
>Syntrophomonas_wolfei_subsp.metylbutyratica_JCM14075T_01427_putative_sporulation_protein_YtaF
VEYFHILLFALAVSSDGFFAGMAYGLKKIKVPLLSLLVIALASALAVSFSMLCGKGLATIFPPDFAGRLGAIMLMLIGVYFLLSACRDRIESMDEIGEEPLFSLNIKPLGIIIHILKEPARADFDLSGEISTREAFFLGLALAMDALGAGIGVALAGFNILLTALAVGVLKFILVNCGLQLGIKIKAERWQLISSLIPGLMLISLGILEIM*
>Syntrophomonas_wolfei_subsp.metylbutyratica_JCM14075T_01428_Dephospho-CoA_kinase
MKTIGLTGGIASGKSMVARALQEMGAVLLSADEIGHQVIEPGKTAYYDLINAFGKEIVNADGTINRKELGGIVFEDPQKLQLLNQLTHPSIMQEIRLKLAQIEQEQPAAIVVMEIPLLYETRMERLFDQVWVVWVDRETQIKRLMARDAIDRSDAISRIDSQMPLDEKARRADLVIDNCGSIEETIAKATRYFNNI*
>Syntrophomonas_wolfei_subsp.metylbutyratica_JCM14075T_01429_Soluble_lytic_murein_transglycosylase_precursor
LNKKKKKRFSISLWVSILLFLLVVLTFPQWISYFYPQPHRDIVFATAYDNQVDPYLVFAIIRAESKYQPTARSPVGARGLMQIMPKTAAWIAEQKGISGFEAEQLNEPALNISFGCWYLNSLNQEFEGELPLVIAAYNAGRGKVKEWQVQGIWDGDTGKLDKIPFEETRNYVKIVLKNYEAYRAIYQ*
>Syntrophomonas_wolfei_subsp.metylbutyratica_JCM14075T_01430_Hemin-binding_lipoprotein
LIIRCKRIVLFIVFCGLIVALASIIHWRQVAVYTASLDTRPFHLGLVGEINSIEPAKLSNHPESLLASAIYEGLLCFDEEKGEIKPALARKWKYSSDGKSLTLYLRGDVQFHNGKKLTAADVKASWERNLGSLKDWSNVSLFLPIAGCAERLQGKNIDIYGIKVLNEHTLKITLLQPNRVFIYSLGHPLFWVFDSEDNNDPLAGTGPFIFQENKENKKFFLIKNEKYYRGNPHLAGINVQVFPDEQQAMDDYK
SGKLDFLDTVPLEEIKKIRKASEYKGLYIEKPILETYSLVFNLNKKPFAGNYYLRRALNYAIDRKKINDEVFGGSCLPAKGAIPTRIPGYSEEMQGYSYKPEKAKELLEEAGYPGGHDLPPLLLTYNSGEAHKMIAEAVAAQLAQLGITVQTQALDWNYYRKQMSSMNMSFFRLGWKADYPHADSFLYSLYHSSQIGNSNFSGYNNPQVNKILESSRAATNEKERNALLRKAEQIVVDDAPCLWLFQKK
AAKLLGKDVDSLTIDAMEMIDWYQVELFKPGVNQGGDAVKKKV*
>Syntrophomonas_wolfei_subsp.metylbutyratica_JCM14075T_01432_hypothetical_protein
LSDTQIEVLKALEDIALSRKDIFAAIGMNGDSRSFKRHIEPLLADGFIEMTVPDKPNSRLQKYRLTDSGKAFIGDNGK*
>Syntrophomonas_wolfei_subsp.metylbutyratica_JCM14075T_01433_putative_zinc_finger/helix-turn-helix_protein,_YgjT_family
MNKVYCYLCDQTNKVAIKKEEEIHTIKGTPVKCKVENAYCRQCGSKVYIPALNDANLDRMDYTYRRHNGIITVQEIEEILRQYNIGAKPLAVILGWGEVTILRYLKGQIPDRAHSNTLLSLKDPYVFFRYFKEKQERLSPVACNKVVSALEGLNAKLAGDELHERSQHLRQAYRRQPDIYNGFVPFNLEKMVQVILFFLKSGDRVYETGMNKLLWYVDMLCYKRSERQALTGLCYKHNIYGPVPLWY
DYLYGSLDDVYITLVEDEYGTQIGALADLSADVFSNDELDVLETVANNFKGWSAKKISQYSHRETAYRQTTNGQFISFEYARELSLS*
>Syntrophomonas_wolfei_subsp.metylbutyratica_JCM14075T_01434_hypothetical_protein
MSDFSIKEQLESLKSDELIEIIMDLVQRGEQSRLHVLEWLDNYRKPQNTVNPKKNSKTQKIHDELLFEYWNNAQNIISEFNSYGGGPEEDEEEAYEWLEKIWDLIDEAKITFESKRKLMDAVFIEYDEGNSGFDDSLMELCFQLCQEKEEWEYLVKKLGNKPSNWNKGLAISIYKNYLHDDSKYLEVRLKNLHYGSDYWDLVQYYIDKQETELAIETAQKGIEKGEGRLTELYDYLIDYYSEVQDDNA
LESIAQICLKRKNDVKYVFDKLFMYYQNRNYEKAKEKLLKAYKHTKRKNYFEDYKRIKSFLSESDWEKIEPQIINEAHDKSIQDYMNICLDKGMKDAVINMIISPPKNQWGYSIFSDFDSFAEKLEKDYPQEIINYYYQKAYSRIANGNRNTYKEAVRYLTKVKTIYLEYLKDEDKWTQTLNNLQIQFKKRPAFIDELKKIKI*
>Syntrophomonas_wolfei_subsp.metylbutyratica_JCM14075T_01435_hypothetical_protein
MNGHLNDCLICDGKLEYLDAAEEMECVICHKKHLNNVRCINGHYICDECHSKSGAKIILEVCRTTDSKNPIEIMQKLMAKPFIHMHGPEHHILAGAALLAAYHNSGGQLDLNKALNEIERRAKQVPGGACGFWGCCGAAISTGMFISIATGATPLSEREWSLSNLITSKVLRRISALGGPRCCKRNCFTAAKVAVEFTREHLAVNMELPVDICCFFSPNNHECRGVLCPYSPQQSNSQ*
>Syntrophomonas_wolfei_subsp.metylbutyratica_JCM14075T_01436_serine-protein_kinase_RsbW
MGKYQDICITIPNNLRYIPLIQDSTAAFARIIGLDEKSTNEVVLGVEEAVVNVINHAFAEGELASFNIIFQQTALGMQILIREQGMPFDPEELTSLAQKDRTEAEDTRGLGLHLMYRFMDEVSFVNLGKAGKETRLVKKYSGIRMRDVMDLPQPDSHKQADEVPVYQIHLMKPEQAVNVSRCAYMSYGYSYSSDYIYYPELLRQLNEEGKMVSLVASSENNEIIGHVALIFQGDDPHVPVVDDAFVNPK
YRGAGCLNDLGAAVIEWAQNNGLTGLYTWSVTTHPYSQRVASKWGFSDTAILISGDLPFDFKALKEGAGQRESETILFTYLNAKHAIQIYPPAQHADIIQQIYHSLGVEAKILPIGSSLDLLEEDAVLELKYDDPSIIAFVTVKKTYGKNVVHEVNRMLRGLCLERLETTYLKLPLTSPYTAILTEEFENLGFFFSGIMPGSEGCDQLILQYLNNQVIDYDFIKVHTEMGQKILAYIKNHDPNQI*
>Syntrophomonas_wolfei_subsp.metylbutyratica_JCM14075T_01437_putative_oxidoreductase/HEAT_repeat-containing_protein
LFALGKQLLVRPDDTHRLRLMIPVFFSLGVGEVLGISSSTAIFNVRYGVEYLPLMYVLEAAGLLLLSLLIADLSGRMDRSRFLRLTYGMMAGIVLLNGLVLVLSKFSAFSLWPVFYPILLVTSMVVFFQLTPLIWLIALDVCSTQQAKRLFPLMAGASTIGCIAAGVIGKLLTPIGVEIIYLFWSLFLLGGGYYLFQTIEYYISPLKALTVEEPPNLKNSIVSIYRSRFLLGMLSLLTLIMTLYFLMDYQFNTVARIA
YNDEAQLAGFLAIFLAISNVVAVVIELGFLSRIMSLLGVGNVLLLVVAGLACSFIITIIFTSGPWALGAVFISYLITKIMVNVLGEPSYQLLFKVIPPSGRDGIRFLVEALIILGGMIGGAAVSGLYSAGIITMQSMLVSALILAIIAAFVAWKTRGMYLNELLRSIGNGVKDLSENGAAFLGRYVPSTFLTQLFSFLHHPDDRKRILALGIAEQLDSHALEPRLGDLLNDSCADVRRSALKYLRLDNTSYHQESIM
ACCMDEEPEVRAAAIDLWPALNLDRHQLYQALNDSDSLVVAHAVAAICSLEQAINYPKVIDAVKRCLDKGETSAATICQAISSAGLDEFSPRLISMLDSSPQLRTAACEALGKLQICNSIPRIIDIYSKGDRDFHKVADQALIDMGEDAVPILLDEINKQSDLRSWLGIIKALSAFNREDRHSRLLIDSCLQRLEQLAVFKEVPSLIKSLNYADLANVAFLRYQEIYSLQIEGCWAVLGSIYDPLVVSRVRTASQ
DEDIELREISLEALSEGLVDRRLARAMLEAMVSPATIPKPSADIVKAKLKKAQSLDDYWFSEISTAALSRLKGGNTMKDREMLSLLDKVMLLKSVELFSYLQLEELGLLARAASLEIYDEGTVLVQEGQANSNLYVIIDGHIELSAHSAGTGVNATIAVRGTGETLGDSSVFDQSASSLTAQIILSEATLLIIDGKDMHRLCSLYPNIAMGFIKAISGRNRKLEKMLITMA*
>Syntrophomonas_wolfei_subsp.metylbutyratica_JCM14075T_01438_secondary_thiamine-phosphate_synthase_enzyme
MLHQLKLTTGQHEQMIDITAQVQNVVEQEGVREGMVLVYCPHTTAAITINENADPTVVQDILMRLDELFPWKHSAYRHREGNSAAHLKASTIGLSQTIPVTAGRLLLGTWQGIYFCEFDGPRQRSFYVKVSAI*
>Syntrophomonas_wolfei_subsp.metylbutyratica_JCM14075T_01439_hypothetical_protein
MCGRYTLAVQLDEVAERFLCPKTNQQMKAHYNVLSLSIN*
>Syntrophomonas_wolfei_subsp.metylbutyratica_JCM14075T_01440_Endoglucanase_precursor
VQSMGLGFKPDVKIRGILLVIFIVLFSFMGMVNCDWAAAKTESCDPASSLTVVLNDNGVAFELAQYTIDQLKNMPQVEREYSSIDRMPAPVFTYGRGLDLENFLTSQGIDINSISYIRFYATDNVVKKLDRNILLDRTGYYYPKIVECWDSDGVDGETYIYPDVQRASEGAAPVKPMLAITSSQGRFLPAPDRNNLDGSTCLRLCLGQASPEECITMNFVRWIYKIEVFGKLRAGGGGSIAPRVALSSPA
ANASYQAGDKVEIRGTVEKLSSLSLTITDPDGHSIYTVFDLEAKDGSFTEEYTLEADAVSGMYTIEAGPVPGSSLGCKQTFQVTAAPTSAANITPSNTPQVAESEKKPPVEAAKALPISSLNDISNHWAAERIKDLVARGAIKGYPDGTFQPDATITRAEFITVVVKAFNLINPGGIVFTDTSGHWAQDYIATATAAGIVGGYNDSSFGPNDPVTREQMAVIVVKAAQLTPIAAENQFADSSNIAAWARE
AVATAVNKQIIKGYPDNTFKPGSKANRAEAAAVIVNALNLK*
>Syntrophomonas_wolfei_subsp.metylbutyratica_JCM14075T_01441_Oxidoreductase_molybdopterin_binding_domain_protein
MRETIENNKSKKLLIAGILLLVAVIAVFSGLNRNQDNLQEGQLLIKADDTRLAVLTLAELQQLPAVEKKMAINSSSGRSEHQFTCIELGQVLASVDPGLSSRYTRIITRGIDNYTSGLEMSEVLQPDNVYIAYADAGKPLKSKTGQDGSMRIIIVRDEFGQRFTNFLVSLELE*
>Syntrophomonas_wolfei_subsp.metylbutyratica_JCM14075T_01442_Oxidoreductase_molybdopterin_binding_domain_protein
MGAVVKMRSWIRNLVLLGLVLSMIFSLIACAEETEVTGKTAAANDGQEILITGLQDSDYKITVGELKKLPAVAKKAQSTRANGETVKIKATGCLLEDVLQQKGKSVKDYSTIRFTARDAYSVAVPPDVLKNRDIILAYQIDNKALDAENEPLRVVIPGERAMYWVRMLDRIDLETGAKRTAIKKVVFLETAVKALPQEDYQYFDSSDKAIKTKDLVETYAGGDKPKNIFIKAGDGLYKNETPANFLGA
YIKISGQEAPKFLAPHLPQGMHVRDLLFINYGETSFLAYSQGQKVLSLQTLEGQSGIALSDLIKQTGLSRAAKYKFNSLNAESLELAVNELGSGLIYQNDQGTLVFACPGATDKSLENLLSMECMNP*
>Syntrophomonas_wolfei_subsp.metylbutyratica_JCM14075T_01443_hypothetical_protein
VNRRNWLPAIGLGFLGFVLGGLAGSFLQPTPGYIPPLKVVGDVARVVKLEDPEQLGKLQHISYDGHEYQAIKLMDIITAAQPVAAPKQIYLVGSDGFTSSFPAEGLEKSYIAFTAQNGWEAINLNHPVNSNAKMLKEIVVVSDGSSPNGLTLINSEKELVRVTPGQLYTRTLLEYPYAEGQAAIEKQGKTYSTSVYTQRKVFRLADLTPVPDGEKMLVMGANGEQRFLENRGYFEVKDNYINYLDFD
ERRQLEKIKGVIVNPPAASIMDIYYDARHYLENGEKVLVLVLDGLTYSLYKDALERGQMPFLKKAAPAVKAVGVYPLESNVWLAAMITGAAPEENGVVSEKQRDLKMPSLIALARQWQKQALLLHAGPKLLKSEMEAEVTDKNANKTADKELYAITLDKLEQDYDLLIVRFQDIASNSELYGDEAEATISAMAITDQYLQEIVKNWAGKVIITGSPRSAAQEFTCDAMFVPYLSLK*
>Syntrophomonas_wolfei_subsp.metylbutyratica_JCM14075T_01444_hypothetical_protein
MNRVDKGTGLLTTSSFSLKNYFLTKMNSGRLSFCQNKALTTIYIFALAVLILSGCSLLTPPIVQAEETSLYIIDANNQLIMTEPPPVTRDGWVLVPISTLADTLQGKVVWDAEQQTITLTKNRDTIRLRVDSDQAKMNGQELSLDAPVCNIDGRVYVPLVSVAEAMGAKLEWDEEIRTVKLLPGMGTVSHTSFSPTRDGTPSETLTVKVGYGFGTPYNTLKVFTFSELEAMADLYQAYTFIDSMPAVV
LDSAQGVKLVDILESCAIDINSIDKLYFYTTDVNKGWYQSLSKSFLLDTERYYFPNLPTHWDYDEEKALPGAEKDAVAVEPIIAVRDYWKRFATTPDFTQMDESNGFRLVFGQTDTRTNTAMRSAKWIREISVMLIGKPPSEVSLNQNTINARVGSSVHLEATVGPSDAADKRVTWSSSNPAAATVDASGRVSVIAPGTAVITVTTVIGGYTAGCIINGPGQVSGNPEGGVGGSAVSNEEEQAGADA
QQRLAQKGSAVVSRLPGRIYQMSADVVPLQPHSEYGNVNMPTAAAALVLFLLGAGRKYSEFKKEVAL*
>Syntrophomonas_wolfei_subsp.metylbutyratica_JCM14075T_01445_MotA/TolQ/ExbB_proton_channel_family_protein
VIFAVLDPLRETMHTVSYGLLAPTIIILLLLAAFMVVELGGLLVEIFTERRREKTKLPELLESFQGKDTQGIIAEIDKSSLFRRQKAALHELLQHGGLPAVSLQALARRLLSNEELHYFKITNRTDVVARLGPMLGLMATLIPLGPGLIALGQGDTKTLADCLLTAFDATVTGLAAAAVAFAISRLRKRWYEDYLSSLEALMESLLEVLAGEPGITE*
>Syntrophomonas_wolfei_subsp.metylbutyratica_JCM14075T_01446_hypothetical_protein
VNRGLRSRRRLKTLDEEVSPLEGAINIVDAMLVFACGLMLALVIRWNVDLAQFGERVNVTQGEEVVDDADIRSDLVESEGEGQLYQKMGTVYKDPATGKMFMLTDE*
>Syntrophomonas_wolfei_subsp.metylbutyratica_JCM14075T_01447_Bacterial_Ig-like_domain_(group_2)
MHLMNWGLKPRGRKSIACLVALIMLLSIIGLGSPGTAQAKTDTSPAATEFVVNINDNGEIIPVHSYTIAEMEALTTGYAGYYSSIDSMPAPNITIARGVTLNALVADINQKYNANVTISPSTLKSIRLYATDNWSTSYTYDYLYAATRYYYPELVNKWDKENGVTGPGSDANPIEVEPIFAIYSYQDRFLTDLNHAMVGPSDEGSTTFRFCFGQTANDITNNVITNSRFGRWVNRMDIVLPDRPAAPALT
ADSSYNKVGKPIDITFTDNEAWRNAITEIKVGDTVLESSHYTKEAGKITIAAGVFATANTYTITVKATDYSDATVIQIINKDYESTALSIKVDNGNPITVTAKQINALNPNNEIRYFSHYKDGQMNYFTGLGAPLAAILSQYASLNSANIASMTVRAADGYYVTFADPQNELFNERYFYPAVGERVQVDTIISTKAAENFAGDISQLDNTYTMRLMMGQTSSEERTISRMVKWVSEIDITTTATINIPVTGV
TLNKTATTLVLNHSEQLTALLTPANASKQTVTWTSDKEAVAIVDNTGKVTAVGAGQAVITATTDDGSFTASCTVTVAANPLYKLTPAKDAVYIIGATADGIPTLTVNQGQSGQKYFAVSVEPVISHAGNETVIFTHWRNESELELNAIMADFDVANTAMAGFNVQPGDVIKVYIVDRLSNDESNNPVIFH*
>Syntrophomonas_wolfei_subsp.metylbutyratica_JCM14075T_01448_Endoglucanase_precursor
MVKKRIAIILAALLILSMLTGLALGAPQVTLNISKSSAAVGDSITASGSSNPDEWVSIKLLDSTKSIVFYDAVKPNLSGNYSCTFKVPQISSGTLTVVAGYESNVATQLLAIPVSGGGGGGGGFVSPTGNTVGSGGGTIEEAGVRVSFPANAVSSDIKVSINKLNASSVPAIASGFKLLGEAYEISSDRNTTFAKPVTITLPFDWNQADKDKYGIGLYTWNNSQWVILEQVKVDSTAGKISGEVKHFSKFAV
LAREREKEKEPAKDVIAPVKATLKDITGHWAEKPIYELVALGAVSGYPDGSFKPDSSIMRAEFATILIQAFKLEARSGQTFADTAQHWAKDSISTAAAHGIVSGYSADSFGPDDLITREQMAVMISKAANLPAAQGKSFSDSAQIADWAQAAVAAASSKNIISGYPDNSFRPKANAKRTEAVSVIIKAIN*
>Syntrophomonas_wolfei_subsp.metylbutyratica_JCM14075T_01449_Cellulosome-anchoring_protein_precursor
MNLMNWGLKTRSRKSITCLLALFVFLSMLLISHPGLAYAKTEESDPADHFEVWLVDPANKDNKIELLNYTIPELEQMPQVERAYSSIDSLPAPVFTAARGIDLIDFLESLKLDMGSISSFKFRTTDFDTDGYPPNFGKVLQASVLLDSERYYFPQIQECWDEYWDEDLENPSNSAVFGYWKDGSDPFTRKGKILLRPMLGIVSFQARAIGSDPGKPVYLEPRFDLMDGETTLRLCYGQKNPGECITMD
YLKWTYKMEVTGRLLPIALNPDTSANRVGTPIDITFAENIAWQNGIAAVSIGDTPLGKGQYTLTPGKITIAPTVFSAAGTYTVKVTSGFNESSVEQEVLEASASDPHIPPTLIADSSQNIIGNSMTITFSDDESWRKAISEIKVDGNAVKSSNYSTTEAGKITIDGSIFSAAGSYSIVIKATGYQDAGLQQIVFPLPGPVTLKSIEISKTAAKLLYSVDEPLDISGLEVTGHYSDGSSKVENITMVNISGFDSSKPAS
RQTLTITVGNKTITYTVSIFQAKTAADNSNISVTPDIPLKITMPEGIINSSMKVAQNMPLPPVIIESSQAILSIPEATLITGSDIINLPQVKPGSTVKAPGIKTIGLVIEVGSDSNIINFSKPVRLLLKGQASKSAGFIDQAGNLRKISKPASLKALTGDDAAETVAALFEREKMQDAAVDFGKDLVIWTKHLTKFIAYIPLTDTPVIPPKKIADEFIIIPFEQIARTQTIGIRGGIVKTAGAVLNFPTNSLESDIKVTIKKI
TQDIPAISSGFKLLGEVYEITSDKDNKFRKPVTITLLFDPSEVEQEKHDVGIYCWCNQQWTSLEQVKADMASGKVSGEVGHFSKFAILLSKKSQKPIILEKKAPEVPLPKPILSDIAGHWAQANIIKLVNAGVISGYPDATFQADKGITRAEFVTAVVKAFKLESRSGQVFNDTTQHWAKESISTAAAYGMTSGYDAGSFCPDDAITREQMAVMISKAAKLPAGEGKTFIDSAQIEDWAQAAVAAASSKN
IISGYPDNSFRPKANANRAEAVSVIVNALNNLPPAGEGRMAE*
>Syntrophomonas_wolfei_subsp.metylbutyratica_JCM14075T_01450_hypothetical_protein
MDINNGLLAPPNVRYRQVRTGPKSPRGPKTTAGARTIKKGRSENLPVLQDLPAESVASWGYVFQLNTQTVAVGSNITFSNNGPLNGINHTPGTAGIRVTLDGTYNITFSVYTTQNNPQHWGIVVNGVVKSEFNSAGQSLTASTTLTLSALDNVTIRNINTIPDPATLRIGNFTTAYVLIYKVD*
>Syntrophomonas_wolfei_subsp.metylbutyratica_JCM14075T_01451_hypothetical_protein
MAKKRIAFIVAALLILSIFPGLALGASVSLNLSNSSALIGDSITASGSADPNQWVSIKIVDSLGNIVFFDAVKSNAGGNYSYTFKIPQVADGTLTVVAGYGSNVANKSLQVSIYKVLDPKTCDPDKKWKVVFNKPANPNTLKEQIKVMNTTDNSQVGIQEVLPENDGLWAVIYPAQDYQSGQTYVIYINPDIATTDGEPLKSGYQMSFTVN*
>Syntrophomonas_wolfei_subsp.metylbutyratica_JCM14075T_01452_hypothetical_protein
MSREAMVLIYYACNRYLLMENYGHPLQFDMKKK*
>Syntrophomonas_wolfei_subsp.metylbutyratica_JCM14075T_01453_Cupin_domain_protein
MEIKEKKDYPQGKLKLDTAIEYQTAAVVSKTLINRPSGTITLFAFDEGQGLSEHTAPFDAVVQVVDGEAEIIISGESHILTSGETIIMPAGEPHALKAVKCFKMLLTMIKS*
>Syntrophomonas_wolfei_subsp.metylbutyratica_JCM14075T_01454_hypothetical_protein
MNKKFSWIFVMVALTLLTAFLIIPEYRSAKSTVGTIQIHKEGNQLHREGMTSGDGAQEGKKLIAEKPKDASVDSVNDKKVDLPVDKKEAMANKTSNDSNKKEDMVQKISNDRIEVSRAGSSAAGNKQNSRMGKAAEASTGKSQYPQVSKAVDNSEDYQAGKVAAGNYADSSYPQLSKVQGSFGQFRYKDISGGRIEIDPRWIAENIVTITLPGLNREVQVHKEAQDNFIQAFNYIKNGKAWINGKE
VPLLSLVKTMDGTFVPRHVNWNPAKGLSRHSWGIAIDINAPNHFRYVDPEQEANDPNLILWGKAFKPAGFTWGNSYGDSMHYELLE*
>Syntrophomonas_wolfei_subsp.metylbutyratica_JCM14075T_01455_Aminomethyltransferase
LNLKRTALFPAHQKYNGRMVDFGGWELPIQYESIIKEHHTVRKKAGLFDVSHMGEIEVRGEKAEAFLQYLLSNDIKKIAPGQVQYNIMCYPDGGVVDDLLVYKYTTEHYILVVNAANTDKDFEWIKKNAFPGVEIKNLSDDYAQMAIQGPLAEQILQKLTDLDLRSIKYYWFQSNVEIAGKTAIISRTGYTGEDGFEIYLSPADAIDVWEAILAAGGEDIAPIGLGARDSLRFEAKLPLYGQELGPDISP

LEARLGIFVKFDCGDFIGREALLRQKESQPPRVQAELLMLERGIPRSHYEVCQEGKVIGQLSSGGLAPSLEKNLALALVDREYYQPGAEVEVMIRNKPVKAQIVTGAFYRKKTQAS*
>Syntrophomonas_wolfei_subsp.metylbutyratica_JCM14075T_01456_Octanoyl/lipoyl_carrier_protein
VKIPADLYYSPSHEWVRVEGDQAWIGISDYAQHELGDIVFVEMPEPDDELEAGGQLGVIESVKAASTIYSPVGGTVVAINEELEDAPQLINEDPYANYIVVVAMNNPGDVDSLLSAAAYEELCQKEQGGE*
>Syntrophomonas_wolfei_subsp.metylbutyratica_JCM14075T_01457_putative_glycine_dehydrogenase_[decarboxylating]_subunit_1
MRYSPHSPTEVEEMLFSIGMKNKAELFADIPDRLKLGRDLELGPGLTEIELGRHLKELAGKNMNLDDYPCFLGAGAYDHYIPAALDQLLLRSEFYTAYTPYQPEISQGILQAIFEYQTMICALTGMDVANASLYDGASALAEACQMACEGSRRRKVILPATLHPEYLEVVKSYAVSGKMEIIMAPEREGIIDKETTLALLKQDSACLVIQQPNFFGCIEEIAAWEKAVHAAKALLIMVVNPISLGLLKSPG
EWGADIVVGEGQPLGNPLSFGGPYLGFMACSKKYMRKMPGRLVGQSVDSNGETCYVLTLQAREQHIRREQASSNICSNEALNALAASIYLSLVGRQGLVDIAVRCHQLAVYARRQMESCGLSLKYPQAFFNEFAVKVDDPARINRLLLEQGIIGGYELPGALLLAFTEKRTRAEIDRLAVLIGGE*
>Syntrophomonas_wolfei_subsp.metylbutyratica_JCM14075T_01458_putative_glycine_dehydrogenase_[decarboxylating]_subunit_2
MSKVIFARSVAGGGGFSLPPCDVAEIEPAAEINREFLRAEAPLLPELSEVEVVRHYVELSQRAYGVDNGFYPLGSCTMKYNPKINEWAARLPGFAFLHPYQVEESVQGALELLYRAEKLCCEIGGMDRFTLQPAAGAHGELTGLMIIKAYHRHRQDLARSKILVPDSAHGTNPATAHVLGFEVVEVKSNEEGLVDLQDLKACMSSEVAALMLTNPNTLGLFEKEIEEIAAVVHAGGGLLYYDGANLN
GIVGVARPGDMGFDVLHFNLHKTFATPHGGGGPGSGPVGVKEFLSDFLPRPLVEKKEEGYCLDYDRPLSIGKVRSFYGNFGVIVKAYAYMMALGGPGLREACELAVLNANYLRHHLQEDYHIAFKRLCKHEFVASGQKQLAENGVSTLDIAKRLMDYGFHPPTVYFPLIVKEALMIEPVETESRERLDEFIAALREIAREARENPDILHQAPHSTVIKRVDEVRAARQPILRWPG*
>Syntrophomonas_wolfei_subsp.metylbutyratica_JCM14075T_01459_hypothetical_protein
MRSRIAPSELEHSRNDSAAKNLLLHKGCINIQSERWRSMDATPGEGEQAKGGERQDVARALSPMDGRLARYPLSRETSRRCCPCEDCERYIYASQ*
>Syntrophomonas_wolfei_subsp.metylbutyratica_JCM14075T_01460_Dihydrolipoyl_dehydrogenase
MKDLLVIGGGPGGYVAAIRARQLGMKAALVEKDKLGGTCLNRGCIPTKTYYRHAEIMRSLQRLDEFCIQLDADSARLDMAGTRARKDAVVEQMAGGVAALLQAHGVEVIRGEAVVEEPGRVRVGEESIRAERLLIATGSESIRPAALPGAALPGVLTCEELLERSEIPSRLLIIGGGVIGMEFACIFQAFGSQVTVLESLPRPLAFLDQEIARRMSVLFKRQGIEIKTGAKVESINTQDAHLLVTASDKK
GNMQYEAELVLLAVGRSPATAGLNLDKLGVKTEQGFIKVNQDYESSVGGIYAIGDVIGPPMLAHVASEEGRAAVERMAGMDSRLDYEAIPHCIFTFPEIAAVGLTQEEAATRGIDCRTGKFQFAANGKAVAMGESEGLIKVICGPDDTILGVHIMGPHASDLILEASLLVNLGLKVEEALHTVHPHPTLGETLYEALLDAQGRAIHLKPPRK*
>Syntrophomonas_wolfei_subsp.metylbutyratica_JCM14075T_01461_Lipoate-protein_ligase_LplJ
VLYVKNDSCDPYFNLALEEYLFMQRQDLGPLFMLWQDIPVVVVGRNQNTREEINMEFTREKGIAVVRRLSGGGAVYHDLGNLNFTFILEQAERELDFARYTVPVIKALERMGITASFTGRNDLTIDGRKFSGNAQFRQRERVLHHGTLLFEVNLENMEQALAVAEDKISSHGVKSVRSRVTNIAEHLTAPFSLKEFKELLREMVREYYGLDGEYELKLDDIRAVEKLRLEKYCSWEWNFGHSPAYNI
KRSRRFDWGKVDIFLEVQAGVIREARIFGDFFSPGDIDELCRSLSGLPYRREAISRCLAGNRLEQYFPFISEEDFMDLMGVRHLP*
>Syntrophomonas_wolfei_subsp.metylbutyratica_JCM14075T_01462_hypothetical_protein
VEFLFQRFWHSFVERLDEKFIIARTTYLDTRMEREIVLRIEAESLDIKEAWLLRLGRPGMMADSREELQALKGVKAYLGSGPELRQALRSVEDELERSLVNDTVIAVVQAESFLYRERGYNDAAQYTRAWEEFYRGSCRYYSHLERVKVSWGDFIGAFSRETRLFDRCKTQQLFREDNGTYRINGSIIDSFHHMAACLELDQDMKLSQAWADMPRVPDEVCRESTGQLANVIGHTLAGKPKKELARL
LGAGQGCVHLIDLLYDSARMLELYMLDVG*
>Syntrophomonas_wolfei_subsp.metylbutyratica_JCM14075T_01464_hypothetical_protein
MTTRERTWKTLSAIALSLALIILSSVLLMPEVVGAEEISLTVTGDGVSTPTTFTQVQLEAMSQLEAKYSTISTYPTKKLLVARGVKLAELLNLAGIKDEASLIIVTATDGFKVTYTRKELLDDTRYYYPGLKDNHAYAGYITGSPEGAVRVDTILALASAEGDNFNNINSINAPVLMMGQRWVSEQTNHAHVKKVMTIEVSTASPAKWENPVATPAAGSVIAGSKVELTTPDMDGDSIYYTIDGSDPTF
RSPMYNWIKKRWWNNHSDQLATINQAIEINEDTVIKAVAIGFGKFDSDIVTFNYQVSNPKPPQYILTPVAQNTYTVQTTADGVPTMTVNSGISGLKYFTVNITPITAHQGEETVVFTHWRKGRQININSTRADFDLLKTAQAGFNVMPGDLVKVYLLDDLSNTHDSNPLIWQ*
>Syntrophomonas_wolfei_subsp.metylbutyratica_JCM14075T_01465_Endo-1,4-beta-xylanase_A_precursor
MKRGFHNKLGNISSTTGGSIVLAIFLLLSFCCLFSPAALAEEVAKPEQIILSWTSDPLTSQTITWLGADDSLGQLQFQAKSSFNGSFDSAQQVEAEATKFDSRYYRYSINIRHLTPDTDYIYRLGKEGCWTEPYFFSTADDTDKFSFLYMGDVQSGYLGWGRMLNALYQENPRLRFSLLGGDLTNNDADEMEWGEFLDAASGVFSRIPLMPTLGNHDGTMYKNFFALPDDGPAGLEQEFYSFDYGN
AHFVVLNSNNNRNEKAKQWLHTDLQNSKQTWKFALFHHPAYPASPDYKGIDQSIIANWVPILEQNRVDMVFVGHQHQYMRTHPIFQGEIQSDLGRYGIIYVMGNAGAKTYIPGQGFPYIAREDSGSNYQLIDIEGKSLTLTAKRADGGLIESYTINKTLKSAPRLVPDVAGKIVGQPVQLNFIDDADWRLAINEIKLDGVALTPGQYSLDLPGKITIAGDLFSSAKEYNIEIKATGYEDAATTQNILA
TVTLNSPHDGQEFQQGQQITVKGTVNAPWNSVRIQVKNPRGESIYGPLELQVTAGQFESSFTLNAKAEVGSYNIILEGGLPATILGKFKVKAGDAAKPVGEVILTISGSGVRNELKLTQDDLLAKNKSQVVFSAINTWPSKKWYIGEGPLLKDLLNEAGLRDTAGMIRFSSVDGYSILLTTRELIQEQRYVFPQFMDGDSSQGHIRGSANGARAVDAIIALRSAEGTDNPAYMSEVDSLLLMIGQRA
VHEQNAQLFVKKINKIEVINTSPSKWDNPQADPSSGEVSLGTRVRLSSAIMDDDKIYYTTDGSSPTTIDSLMYNPIASRWQSSRADVLDTINHPIEITRDTTIKAITIGPGKMDSDVVTFSYKLKKADIETIGKVIPSNGGVVNLGTEASIRIPAYALKESGAVEVKIERVINPPSVPAGLKLLGGVYEFSVGGQNSYSFAKKVLVKLSFDSSKLESKENPAIYYYDESKSQWQKIGGTISANSIELEVDHFTKFAV
MVADKGKKEEQKKADIVIPMVKLSDIQGHWAQKDIEKLVAAGAIAGYPDGSFQPDKTISRAEFTTVLVKTLKLQNKGSEVFADTESHWAKDYIAIAAAYGLISGYNAASFGPDDPITREQMAVIISRISELNSGFELIPFSDQARISVWARAAVAIMAEKEIMKGYPDNTFRPQDFATRAEAVTVIVKGGGVNASH*
>Syntrophomonas_wolfei_subsp.metylbutyratica_JCM14075T_01466_Bacterial_Ig-like_domain_(group_2)
MGFALLLLSISFNLLPLLSPAEAASGLEITGDGVANPLSLTLSDLQEMEQYQHVYSTINTWPSKKLYVAQGVKLKDLLALAGLKENAQLIVFTSSDGYSVTLTVKELLKDKRYYFPYFQDNSVNDGTIPGSPRDAVEVQPILALLSAESSKNPADMNDMDALLLVCGQRAVSEQTNSIFLKYISKIEVLTSKPSKWDAPRANIGSGKVAEGTLIELSNKRNDTDKIYYTTDGSTPTINSPIFNWSASRWWS
QRPDSLNEINKPIEIKKDTIIKAITIGPGKEDSDVVTFTYQPGLKDEDAPVKSASPSGLSLDEKLVNLKIDSVIELTAILDSDNNTEQGLIWSSSDTRVAAVDNHGWVTVVGQGTATITAKTKDGRFTASCMVNASAGVSSPSLPPQSSNSESQVGTQQNPEVLPVPQDDPLYQPEKERIAASVDSSSDTPEPPIAATTDANWEYLAKKDDTAGTDIAVQPQDPGGQIYELKVDTVALQLQMETKIWS
IYILLVYLSLFIFGAINKYIEYTKEVAG*
>Syntrophomonas_wolfei_subsp.metylbutyratica_JCM14075T_01467_hypothetical_protein
MTESFLIPLKGSMHIIAYGLLVPTLIILLLFVALVIIEMGSLLVEALGERRRSKVNIPELLNAFKEKDFEGIRDEIMNSSLLRRQKAALSELLKYNDLPGVSRQALARRLLSDEELYYARITSRTDLVARLGPMFGLMATLIPLGPGLIALGQGDTKTLADSLLIAFDATVTGLAAAGVAYAISQLRKRWYEDYLAGLEALMESLLEVLERE*
>Syntrophomonas_wolfei_subsp.metylbutyratica_JCM14075T_01468_hypothetical_protein
VNEGLRRRRRYRTLNEEVSPLEGAINIVDAMLVFACGLMLSLVIRWNVDLVQPGNRIDVQKGQEVTENTDIREDLTKSEGKGQLYEKMGTVYKDPHTGKLFLLTE*
>Syntrophomonas_wolfei_subsp.metylbutyratica_JCM14075T_01469_Cellulosome-anchoring_protein_precursor
MQVIKNKAVYHSLISLLALLLLFVLFECLGTSAVLAAPSNSLEICGDGVKNPTTFTRAQLEEMPQYQQVYSSINTWPSKKWYVGKGVHLSELLSLVGIKDDARLLKFTSQDGYTMTLTVKELLEDKRYYFPHFNDNQNRDGEGNIVGSSQGAELVEPIIALISVEGSNNPNYMSDLNTLVLMMGQRAVTEQTGNAFVKYLNKIEVLTSEVPKWDSPQANPGSGEVSAGTMIALSTARMDDDKIYYT
TDGSTPNLNSPIYNWIAKRWWSARADILGTYNHPIGPINENTTIKAITIGPGKENSDVAAFTYTVPVSKPASETSIVAPNEETNYPDQALEKPLQSEPKATLTDVQNHWAQKNINELIASGIAGGYPDGSFRPDNTITRAEFATMLVKAFNLQKQGGKVFADSEQHWAKEYISQATASSVVSGYDAENFGPDKLITREQMAVMISKASKLLLKAEAMHFADSASISEWAKEAVLAANQSGIMKGYPDN
TIRPQGSATRAEAVTVIVNALTLK*
>Syntrophomonas_wolfei_subsp.metylbutyratica_JCM14075T_01470_hypothetical_protein
MKQKSFFQAVGIILGMIIIGCILSIFGCSSGGHTPSGQAGNTTREEVVYFTGNGLMKESRMSLSQMLELPNAHFEHRYSLINNWPNEKILVARGVKISEILKAAGIKDEAKCILVKGEDGYECSYTREQLLETKRFYFPGVREREASRAELAEPIIAYEYMEDSEQLSEARADRLCLIMPQAYVNEQNQHSFVKAVSEIHVTVDDPGKWEAATVYPPAGNIKKGDSVKLQHKHMGKVKMYYTLDGSMPG
ENATLYNPSTYQPELNQPIVINQDTCIKVVVKGFGKYDSDMAEYRYQIK*
>Syntrophomonas_wolfei_subsp.metylbutyratica_JCM14075T_01471_hypothetical_protein
LMKGQIKTRKVLIFALLFLSLNILAACQGQKPESGGKIEKPDITPGFLTISGNGVKAPSRFTLAELKDMEEAQTSQCYSIVNNWPSKKYMLGRGIELSYLLKKAGIKDEAQTISFQAVDGYFAVFTRQQLEEKRFYYPKLMEGSEEEAQEVPAILAWEHREGSKDLSKASGGKLTLLLGQKGLNDVQAQVCVKDVAVIEVLTSPPGQWSTVRATPAPGRIKPGTEIVLHHSEQDQVKIYYTADGSSPSEK
SLLYNPSTTYYQPELNKPLVVKQSVTIKAIAVGYGKNDSQIASFAYDVEGY*
>Syntrophomonas_wolfei_subsp.metylbutyratica_JCM14075T_01472_Oxidoreductase_molybdopterin_binding_domain_protein
MKLNKTSYFFLIALLFCIILTGCAEKQVISSKSSGNQEYEQQKIIEGDIEESKDITVAEMRQLPQKESKCSFQRATGEMEEFHAAGPDFSDVMAHAGIDVKDFKGFGIIGTDGYYCLVTPEIINNREMILALAIDEQLELPKNMRPARLCIIDEFGPYWVRMVDKIILYKDIPKKDITSVWIFNNLAQGIEPYPYEYYGSKDNAIELAQIFSRFDQVEGRAFFTMKSADGFTKSEALSMVNKHYYIKVAGEGAP
MNICPNIKLGMNVKHIAWFSTNADAAIFPEQMAELMGEKDFSGIKGIVLKDILEEVQLKGIEEKEFELVGMDGESVTVSGKDLSQGLLHIEDDGNYLVLWQEGTGLQAVDNLLCIRSIK*
>Syntrophomonas_wolfei_subsp.metylbutyratica_JCM14075T_01473_electron_transport_complex_protein_RnfG
MLKRAIAYIVLVAIIIGCWGITGCSREKTIPKQQLTILNQLLSTSVSDKQVETIEIQDETARKFPAIDKMFKVVADEEQENYVFLVSPIGYRAPINIMVAIDGEKDEIAGIKVLQQDETPGWGEWLAETWFTDRFKGKSVDIYLEKAVLEAKAPNEIIQITSATVTTQAVLNGVNAAMGAYRELVLGSEAEAVPLNVEGFITESE*
>Syntrophomonas_wolfei_subsp.metylbutyratica_JCM14075T_01474_Oxidoreductase_molybdopterin_binding_domain_protein
MNKHPKEIVTLPQYRISNKPVFIIIIILLFAIILFSYFNLQGEKLEEGRIRVKAGDHIIGEITIDEVKKLPAVNKKLVINSTAGLSKHNFTGVALYEVFNQMDPAIVKNYNRVITRGVDNYVSGLNMEEVLESNNVFLVYADNGKALKSKSGREGTMRIIILNDPYGQRFTNYLAEIELE*
>Syntrophomonas_wolfei_subsp.metylbutyratica_JCM14075T_01475_Putative_electron_transport_protein_YccM
LRKIRRVVQLISAIGINSYYIGFFKGSIYLGNLKKICCPGLNCSSCPGALLSCPLALLQTSIAGIGTAHYSFFILGFLLLVGVTLGRFICGWFCPFGLIQELLFKIPLPKISWEERLKRLESLKYVVLVIFVLILPGFGTYGMSTAFCKYLCPVEVLEAHIPIMMVRPSLMSTVGPWFQWKLLLLILLILALFMFKPFCRYLCPLGAIYSCFNSFSLYRLEVDADKCIDCKQCQAACKFKIPVHQIPNHRECMRCNDCL
LCPSQALQVKTCRKRNISAGHGLNFKDSGR*
>Syntrophomonas_wolfei_subsp.metylbutyratica_JCM14075T_01476_Thiamine_biosynthesis_lipoprotein_ApbE_precursor
LIYQLYTRLGVILLLLICLSMLGCEDSSLPPEQSDLREYSRENFIMNTLVTIRVYSSDPALGKRALAEAFAEFQRIDKLSNRFAEKNLPHPELSDVYRVNKNAGLKPVKVSNDTLAMLDRSQYFAGLSEGAFDVTVGPLMDLWGFGKERYQVPSATQLESALALVGYKRILINETEKTVFLREKGMEIDLGGVAKGYATDMAVQKLRQIGIKSAIINAGGSIYALGLKPDGSPWRTGIRDPRDVDKIIAVLE
VRDAAVVSSGDYERYFIRDGVRYHHILDPVTGQPARKLISSTIVAPQATDADLLSTAIFVLGSSEGKKLIEEMPNTSAVLVDASGVINCSTELAEQIEFIEDTGYQVLGNI*
>Syntrophomonas_wolfei_subsp.metylbutyratica_JCM14075T_01477_Heptaprenyl_diphosphate_synthase_component_I
LSLRKQTIIAVLLSLAIILHLFEALIPIPFPIPGVKLGLANIITLLALVLFDFKTAFLIAFLRTMLGSLLSGTIFNIAFFLSFAGAISSTCFMAILLFLLPPIFHHDDRGFSLVGISIGGAAAHNLGQLAMAALLIKHSGIFYYLPIMLLSSIPTGFLTGLILRELIKYMKLSGQLEIQQWNQL*
>Syntrophomonas_wolfei_subsp.metylbutyratica_JCM14075T_01478_hypothetical_protein
MNKANKILIAVVLALVLFSYWGFFPAYRENVPLTAVISVNGDIVVQLKLDESISKHFIKVPGPLGTSVVEVKPGAIRMKSSPCPEHYCTKTGWIDRPGQVVACIPNRVIIKISPDKESLDAISH*
>Syntrophomonas_wolfei_subsp.metylbutyratica_JCM14075T_01479_hypothetical_protein
VSLMQKFLDRFSLYDLIIIALLAALGIATKSIIVPLAHIITGPFFIPSGTVAGGFYMMWLVLGFGITGRRGTMTLMGLVQAILVIATGIVGSHGVISLLTYTMPGILADLGLFLIGHRVCCLPCSFLAGLLCNITGTIMVNLVYFRLPLIPLVLSLSIAALSGGLGGMLAYKILEQIRTFQNRDQNVGSNQ*
>Syntrophomonas_wolfei_subsp.metylbutyratica_JCM14075T_01480_Putative_HMP/thiamine_import_ATP-binding_protein_YkoD
VESCNEAVVVQALTYQYHASGPFILRDIGFRIDRGEVVVITGLSGCGKSTLCLCISGLIHHHRNGVISGKIILNGKDTRGMRGPALALEVGMVFQDPDTQLFSPAVEDEIAFAPENLCLPPEQIQERVNNVIDTLGLAALRHANPYYLSGGEKHLVALAAVLALDPPVLILDEVLSQLDSLGKKRVIATLEILRDKGKTIIAVEHNLEALAFADRFMVLEAGKLIRFDRTEKILADQDFLMTRRLV*
>Syntrophomonas_wolfei_subsp.metylbutyratica_JCM14075T_01481_Energy-coupling_factor_transporter_ATP-binding_protein_EcfA1
VFLELRQVGFSYPQKTSPAVIGINLSLKKSGITAIVGPNGSGKSTLTKLMLGILPASEGQIYLEGRNITEYSLAEAGRRIGYVSQNPDLQLFCASVAEEIAFGLVNQGCDVATLEERVNFYLDYFELAAYRKSFPLHLSQGEKQRLAIAAVLANEPEYLILDEPTNGLDAYRKRLLEEYLHKVAELGRGILLVSHDIAFVDRMAERIIALANGQIQSDSNQRGEGYLCY*
>Syntrophomonas_wolfei_subsp.metylbutyratica_JCM14075T_01482_Putative_HMP/thiamine_permease_protein_YkoC
MLLDPRSKMIMVICISSLALIYNTPARLLQLLLVTLVLLFIFSFNLRVIAGYLKPFVFLMFFLFVVQSIFSPGDRVILALGPVSLISIEGVLAGATVVLRLLVITAAAMLFTTFSSRDFILGLVQWKVPYEIAFMVSTALRFLPLFRDELKNVLTAIQLRGVDLKKVPWGKKMVMYRRLTFPVVYRNMLKAEQLAIAMEARAFRAYPQRTYLRQLNLQRVDYVVILLSLLVTFILIMRYIG*
>Syntrophomonas_wolfei_subsp.metylbutyratica_JCM14075T_01483_Energy-coupling_factor_transporter_ATP-binding_protein_EcfA1
MDGRIELIDATKEYKHAGSPPVSALNCVNLSIRQGEFVGMVGLNASGKTTLARMLNGLILPSRGKVLVNGLDTADLAQKKEIRRLVGMVFQNPDNQIINPIVEEEVAFAAENLNLSPAEVRRRVDWALEVSGLADLRHHAPQMLSGGQKQRLAVAAVLVMQPSYLVLDEPTSMLDEDGRYELIKTLQDLNRQQRLSIIMISHRMEEVAPAERIIVLHQGTVYLDGPPAEVFRSPRFALAGLKPPPIA
RLTNSLLRQGHPIDDRIVTLEQMVEFLCR*
>Syntrophomonas_wolfei_subsp.metylbutyratica_JCM14075T_01484_Energy-coupling_factor_transporter_ATP-binding_protein_EcfA2
MPVIETKELTHIYLAGTPYEKKALNQVSLSINKGELFGIIGANGSGKSTLIQHFNGLLTPSCGTVRVYGKDTANRSCRNELWKKVGLMFQFPEQQLFASTVFDELAFGLKNMGLEGWEIENRVEEALLQVGLNPKEVIKVPPLGLSGGARRQVALACILAMRPTILVLDEPTAGVDPSASEHILKAIKKMQREQQATVVMVSHYVNDLIFLADKLAFLKEGRLLSYGDKKTVFRDMAQWDVNDLILP
EHLKLMHRLAAYGLPVNTEILTLEEAAAEISRVMEEWARCER*
>Syntrophomonas_wolfei_subsp.metylbutyratica_JCM14075T_01485_Energy-coupling_factor_transporter_transmembrane_protein_EcfT
VREMITGQYIMGDSIIHCLDPRTKLIGCLVVIVAVWPRQTITILLFNLAFIIIVIYFAEVGVGRVLRGLRSLKILFLLTFICQVFLTRGDPLWSWGSIVITRQGIDLGINTFLRLLILFLSASLLTMTTSTLKISAGLESLLSPLKYLRFPVQQFAMIINIALRFIPTITEEAEHIVRAQKSRGAQFDSGSLSIRLRTVMALLIPLLAASLQRATDLAWAMESRCYTSDGQIYSRVYKLHFAQRDLIAMGVISIVLLLAIL*
>Syntrophomonas_wolfei_subsp.metylbutyratica_JCM14075T_01486_CxxC-x17-CxxC_domain_protein
MFEDKYLVCKDCGSEFLFSASEQEFYAEKGFENEPGRCPDCRRARKQQRNDFGRSRSREDRPMFKTVCAACGQQTTVPFEPSGDRPVYCRECFQARQSRNNYSYR*
>Syntrophomonas_wolfei_subsp.metylbutyratica_JCM14075T_01487_2-hydroxyglutaryl-CoA_dehydratase_component_A
VAQMNAVLHMGLDIGSTTVKVVILDDKDRMVYACYRRHYARVKTTISNLIRDAYKRVGNRPLTVMVTGSAGISLARWLELGFLQEVIACHQAIERFIPESDVAIELGGEDAKITFLTGGLEQRMNGTCAGGTGSFIDQMAVLLETDAAGLNDLASRHKVIYPIAARCGVFAKSDIQPLLNQGAAREDIAVSVFQSVVNQTISGLACGKSIRGRVALLGGPLYFLPELRKRFQETLQLKDEDIIFPDEAQ
FFVAKGAALASKKETEIYFSELLERLPGLEQAQAQEIWRLEPLFASEGELQAFRQRHARYRASRQELEYFRGDCFLGIDAGSTTTKAALIDNEGNLLYSYYGGNQGSPVQSTVKILEDLYRRLPAGANIVISAVTGYGEGLIKAAFSVDLGEVETIAHFTAAEFFQPGVNFILDIGGQDMKCLHIENGVIESIMLNEACSSGCGSFIETFAHSLNMGVEDFAVQALRAESPVDLGSRCTVFMNSRVKQAQKE
GATVGDISAGLSYSVIKNALFKVIKIKNSAELGEKIVVQGGTFYNEAVLRSLEVLTGREVVRPDIAGLMGAFGAALIARNKYHKAYATTLLNRDKLLDFSIRTSTRRCSGCENQCLLTINRFQDGKRFISGNRCEKMLDNKKNKDIPNLYDYKLQRLFHYEPLAEEQAKRGTIGLPRVLNIYENYPFWFTFFTQLGFRVELSPLSSRGIYELGIDTIPSESACYPAKLVHGHIAALVKQGIKNIFYPCISRERPE
KGEADNHFNCPMVISYPDAIKNNMDVLREKHVRFMNPFLPYDDQDRLITRLFEELGDFGISKREIARAVRAAWKEDECFKEDIRKKGEEVLAYLEQTGGRGIVLVGRPYHLDPEINHGLPNMITSFGIAVLSEDAVAHLGSVQRPLRVVDQWMYHSRLYAAASFVAEQRNLELVQLNSFGCGLDAITTDQVQEILQASGKIYTLLKIDEVNNLGAARIRIRSLLAALEERDKNGFIPDRISPATPRIAFT
RRMKKKHHILCPQMSPIHFEFFEEALRAEGYNFEVLPEVDQITVDEGLKYVNNDACYPSIMVIGQLLQALQSGKYDLKHTSLMISQTGGGCRATNYIGLLRKALRDAGLGHIPVISLNLAGMEKNPGFKINRRLMQKLAWSLVYGDLLMRVLHRVRPYEKIPGSANMLYRKWNKRCREQIARGDRGAFSENIRGIVREFDHIEIADIVKPRVGVVGEILVKYHPEANNNIINVLEREGAEVIVPDMLNF
FLYCAYDDIFDYQYLSGKKRQELSGRMMINYLEKQRSEMISCLDESARFSAPQSIYHMAKSARKIISLGHHTGEGWFLTAEMVELIESEVPNIVCVQPFACLPNHVTGKAMIKELRRNYPQANIVAVDYDPGASEVNQLNRIKLMLTVAFKNLQSAQGDGSLDNTLSASELNMQLS*
>Syntrophomonas_wolfei_subsp.metylbutyratica_JCM14075T_01488_hypothetical_protein
MESISFNRPRRIRVEPGKEKNANISRVDSSPLWLNSSQLMDEVLKSLEKRRPCSIVSLGATEAFVMAQYTLLTEEEFMHHPEALVANRGARGGHGHRGISFPNLRARDDTVEAVRMAHIVGYNSLVEPAREMAERVLEAYAIQPRYLFEANIRRVLLFSQREKFEAMLRGRKVLLISSLAPRLKVLRQQEYKDRLDCDLVAALSIYDYGEIPEVKRQLDHYDFDLCLLAAGVNAIILATHIAGYLGKVAFDI
GWCMETMISGEIFVDPWLGNIIGLEMLLQM*
>Syntrophomonas_wolfei_subsp.metylbutyratica_JCM14075T_01489_hypothetical_protein
VDSFIIYKDDFAEISEIISRVNYCLRNQLGFSLVRVGDAENQVMAQGTIIAEDKIAEIWWAEDENWTGVTLPNYSARDRLLDAVQKADIVGVLHQDEVFIWKPLTEAVFSHYKIKPRQLCYAFINTYLPKSDQFISLLQYYRLLLIGKAASSLALLLQERYGIEVAGTISISNYSELERVMEESSKLDFDLALISAGSNAVILAVELAAQGKVAIDLGRGMHLEFWE*
>Syntrophomonas_wolfei_subsp.metylbutyratica_JCM14075T_01490_hypothetical_protein
MTREALYLSYNMHMPAIVINVQGESLVVRE*
>Syntrophomonas_wolfei_subsp.metylbutyratica_JCM14075T_01491_UDP-glucose_4-epimerase
VKILLTGGAGFIASHICDRLISEGHEVAVIDNLSTGRLEFLNPRAYFYQLDLCDPAIALVFQIEKPELVIHHAAQVSVNNSWENPVLDAAVNVVGSLNLYENCRQYKVKKIIYASSAAIYGEPLYLGIDEEHPVKPLSFYGLSKYLAEIYLKYYAGLFGLKFTILRYANVYGPRQRSDGEGGVIAIFLSSLLKGEAPMIFGQGKQSRDFIYVADIVEANIQALTRAENEVLNLGTGIELNINQLYAGIEALLCSQLQ
PVYVPERPGDIMHSYFDQRKAAKLLDWEASYPLQDGLRKTIEYMS*
>Syntrophomonas_wolfei_subsp.metylbutyratica_JCM14075T_01492_hypothetical_protein
MGDSATVRIRVGRSSRVSLIPGSSPYSIREAPIVSFQTPWLNSAQLMHKIWLALEKKQGLSVVSIGQTEAFVMAQYVLFSEEEFLSHREAEIANQGSREGFMHRGIRFPNVAARDEAVEAVRKADIVGYNTIEPVARDLTERVFALHQIHPSFYFEAHLRRVFMFSQKERFERILAGRRILLIGALAPQVSLVLNTKLKRRLGFEIAAAIPLYEFEEIPWVKEQIARYNFDLCLLGAGVNAVILSSYIARHLGKVA
VDLGSGMESLITGKIVTDSFINEVIGLDRLLQM*
>Syntrophomonas_wolfei_subsp.metylbutyratica_JCM14075T_01493_hypothetical_protein
MRVLVTTGDFSHYLSPNFHYLLTELGKMVDLQLWHWPGDINEIIRSLDAKPDFVFINEFAETNSPIITGLAGLSLPWAVLLHDLHYQIEARTRMLREINAPGIFSIYRDRFSEWYPEFWPKFRWLPHHANTDVFYDYALNRDIDYLMLGAVHESIYPLRCRILEKMSDKPGFVYHEHPGYRNFSGEDGALVGTRYAWEINRAKIFFSCDSIFRYPLAKYYEVLACKTLLLAPASQELADLGFLPGVHFVDI
NEYDFEEKAEYYLQHEEERLQIAEQGYEMVRTHHYTGRRALDLKIAIEEICRDWSGAQLSTGKQPFSSRIRVRKGVRL*
>Syntrophomonas_wolfei_subsp.metylbutyratica_JCM14075T_01494_hybrid_cluster_protein-associated_redox_disulfide_domain_protein
LITKDMVIQEIVTKYPQTLPVFGQFNMGCLGCSGALFETLEQGALAHGIDVDAMLKALNDLIKK*
>Syntrophomonas_wolfei_subsp.metylbutyratica_JCM14075T_01495_hypothetical_protein
MDKPILLGNFIFCSLQQIPLLGNDCQACEYIRSLENEYFCVYEAEEENYPEDSYPVRTVIEVLLDMLAAGQKDLRLDELESLLLKRK*
>Syntrophomonas_wolfei_subsp.metylbutyratica_JCM14075T_01496_putative_enoyl-CoA_hydratase_echA8
MAYENIILEREEKLAVLYINRPKAMNALNKDTLLEIKDAVTAVNEDPAVELLIITGSGDKSFVAGADIAFMQNLSAMEAREFGALGQKVFRLIEAVEKPVIAAVNGFALGGGCELAMCCDFRIAASNAKFGQPEVGLGITPGFGGTQRLPRLVGPGMAKQLLYTADVINADEAFRIGLVNKVVQPEELLPEVKKIAGRILSKGQLAVRLSKAAANEGMQTDIDRAMSIEADAFGLCFATQDQKEGMT
AFLEKRKANFISK*
>Syntrophomonas_wolfei_subsp.metylbutyratica_JCM14075T_01497_putative_3-hydroxybutyryl-CoA_dehydrogenase
MKIMVLGAGTMGAGIVQTAAQAGFEVVVRDIKQEFVDRGIAGIDKLLSKNVDKGRMTAEDKAAVMGRISGTVDMGAAADCDLVIEAALEVMDIKKAIFKELDSICKPECILASNTSALSVTEIAAATGRADKVIGMHFFNPVPAMKLVEVIRGASTSQATYDAIKDLSVKMGKSPVEINEAPGFVVNRLLIPMLNEGMYCLMEGVANAADIDTSMRFGAGHPMGPLALADMIGLDICLKIMETLYK
EFGDPKYRPCPLLAKMVRANKLGRKTGEGFFVY*
>Syntrophomonas_wolfei_subsp.metylbutyratica_JCM14075T_01498_Acetyl-CoA_acetyltransferase
MTREVVLVGACRTPVGTFGGTLKDVGAAQLGAIVMGEAIKRAGIKPEQIDEVIFGCVLQAGLGQNVARQSMIHAGIPKEVTAFTINKVCGSGLRAVSLAAQVIKAGDADIILAGGTENMDKAPFILPNARWGYRMSMPKGDLIDEMVWGGLTDVFNGYHMGITAENINDMYGITREEQDAFGFRSQTLAAEAIESGRFKDEIVPVVIKGKKGDIVFDTDEHPRKSSPEAMAKLAPAFKKGGSVTA
GNASGINDAAAAIIVMSKEKADELGIKPMAKVVSYASGGVDPSVMGLGPIPASRKALEKAGLTIDDIDLIEANEAFAAQSIAVARDLGWADKMEKVNVNGGAIAIGHPIGSSGARILVTLLYEMQKRGSKKGLATLCIGGGMGTALIVEAL*
>Syntrophomonas_wolfei_subsp.metylbutyratica_JCM14075T_01499_Acyl-CoA_dehydrogenase,_short-chain_specific
LAHENYLYQMRDIKFAVKEWLDMNKLLSCDAYKDYYGIDDIDAFLDVNFKVCRDVLCPANKEADDPGCKFVGGDTQAVITPEVFKNAYNTVCEAGLGPQFSDRSAEGRMPLVWEAPILEMQSGASPGIVMFWCLTAGACTVIQHNASEVLKERFLPKMYSGEWGGTMGLTEPGAGSEVGAVATKCFPTDTPGLYKIKGQKCFITSGDHDLASNIIHLVLAKTPDAKPGTSGINCLIVPKFWV
NEDGTQGAWNDVTSTGIEHKMGIHGSSTLSLSFGENDNCYGWMIGDGPVDGRGKGMAQMFQMMNEERLNTGTFAQGCIGSAYYAALDYCKMRVQSPKFTDPKGPSVRIIEHEDVRRMLLFQKSIMEACRALLYTTYFYQDLSHDAADPAEREYYDDMTMIQIPLCKAYVSDMAWISTEQAIQCLGGYGFIEEYAPAELARDCKIYSLWEGTNFIQAQDFNNRKTTMKKGEPMKKWVAQIA
DFLATKKDPAFADEFAMMDDAFSAYNEILSTKEAWRASNPQLVQLFATRMLHAASMMICGKLMLDQALLAAKKLAELGEDHFDAMFYKGKIATAKFYIMNVVPGVFGTLKAMKVADTSAIDMPEEAFM*
>Syntrophomonas_wolfei_subsp.metylbutyratica_JCM14075T_01500_Succinyl-CoA:coenzyme_A_transferase
MYQKLLEEYKSKLVTADEAAKQVKSGDWVEYGFGINCARDFDEALAKRKDELEDVKIRCDLGAYQHFTAEVDPDNKHFTWNSWHVAGHDRKFINKNLFYIPMKFHENPMMTRKDCVPTNVAVIQCTAMDKHGYFNFGGSSVNCCAMMETARVTILEVNEKMPRCLGGNQECLHISQVDYIIQSKNEPIATIGSAEPSPVEIAMAQHIIERLYDGNCIQLGIGGTPNAVGSMVAASDLKDLGV
HTEMYVDAYLLMAKAGKITGARKSIDKYKQVYSFAMGSQELYDYIDDNPGLASYSVDYTNNPWVVAQIDDFVSINACIEVDLYGQVCAESVGTRHISGTGGQLDFVEGAYKSKNGQSFICLPSTIEIKGEVTSRIKPILTPGAIVTDPRTATHMMVTEFGIATLKGRSTWERAEELIKIAHPDFQDELVKEAQKMNIWRKSNKIG*
>Syntrophomonas_wolfei_subsp.metylbutyratica_JCM14075T_01501_Transcriptional_regulatory_protein_ZraR
MNSKKTSVNPYLVRYDTSLQDISLIFEQDDDIEFLLVCDAEKQIIGRILPQNIVEANEIGLAPDTPVQQVIPFEQQFIDFINNLQFVLGDSMSSLHSMITNETVAEYYFRLIWRLCRIWHKNATEWEACFDSVYNPVISIDEDGRINLFNRALERVSGIAASEARGQKITDIFSNSKLIEVLTTGQTQPTRKIQYQDKILLTNRTPLYKNGKIAGAVAVLQDISELEHIAEQLARTRELNRQLDAIFESSFDGLYL
TDGEGNTLRLNQGFVRITGISMEECVGSNMRELVDTGVFSDSGTLLALEKQERATITLVARTGKEVLVTSTPIFDEQGNIAFIITNVRDITELNELERRLKHIGLRDRELDTILDSSFDGIFITDGEANTLRLNQAFERITGVTAQECVGRNMKELVDEGYFSGSGTLLVLEKGERATITLVTRTGKEVLVTSTPIFDEQGNIMLVVTNVRDITELNKLEREFKKMVGLRERELDAVFESSYDGLYITDGE
ANTLRLNEAFERILGVKAEECIGRNMRELVAEGVYSRSGTLLALEKQDQVTITLQAKTGKTALVTSTPIRDEHGNIILVVTNVRDISELNELNEQEKLGHAEGLSRFFQSELQEMKLRTQCVIHSSKMRELISMVVRIAAVDSTVLIQGESGVGKELVANTIHSNSNRGEQPMIKINCGAIPENLLESELFGYEPGAFTGASKQGKVGMFEVADQGILFLDEIGDLPLGLQVKLLRVLQDKEILRVGGTKPIK
VDVRIIAGTNYNLKEMVEKKLFRKDLFYRLNVIPITVPPLRERREDIPILANHFLDNFNQKHGMNKKLSGSILAYFMEYDWPGNVRELENLVERLVVTSLNQTITAHDLSAWSELKPENKSDNEHGIVRLQDALENTERRILENAFSVCKSTYEVARALGISQPTVVRKAAKYGIRG*
>Syntrophomonas_wolfei_subsp.metylbutyratica_JCM14075T_01502_hypothetical_protein
MNKNDERVREEWVNSYLDIMEQLQGPVMQQEAMNYEEMNEVLRQNCMQEEEVGEYSMIDRWVQLYLGLMDLLHGLFKQPSLDIEAINQEWIRCFQPAAIPVGEQLTAKWVNTYLDFVEQYQGWSEPEKTAAIEDINREWIRCFLPQEAEQDGQLIKKWEAVFLQLNHELRGISEKKADFTIEEINDEWRRLFLQGEVKAVALKKKVA*
>Syntrophomonas_wolfei_subsp.metylbutyratica_JCM14075T_01503_hypothetical_protein
MIYRLTLLRNLPEAEAGFSFYLKGGLASVDGEDWFSWITRDSIYTRMYHLFLEERHDWVLVEDSSIPDNSYDLQDCFPDHRLLGFKEALDSQGFIYDESSYGINNIRCCGEQLKMLGATKADCIYCSNCGKGVQDAVNYLVWGLKDVPPPRTYLYEFRYDPFKKYLAGIIKDENLKKD*
>Syntrophomonas_wolfei_subsp.metylbutyratica_JCM14075T_01504_hypothetical_protein
MTFDAAIITENGVTFVIIIVKPEILKHDFTINEARKIFSKFFPTLPIILMAQESATTPTYQGRQDLVKFLNTVDYMLLPWKSYTVKEG*
>Syntrophomonas_wolfei_subsp.metylbutyratica_JCM14075T_01505_Formate_dehydrogenase-O_subunit_gamma
MSNENGKVLKHGTQTRTMHWIHLLAFIILGLTGVGFYWDIGGIASIFGGEANASLVHRWTGVVFVVGPLLYTLLNFDRFSRFMDTISHFSKDDFAWLKVMGGYLPFIKADDVPPQDKYNAGQKILGWLIIIGCLLIILTGFPMWLWRHSVPAVFLSFCYDVHFWTAMVLILLVAGHFFLAAIHPKSRVEFSSMMLDGYIDAEITAHHNAKWFAELKESS*
>Syntrophomonas_wolfei_subsp.metylbutyratica_JCM14075T_01506_Periplasmic_[Fe]_hydrogenase_small_subunit_precursor
MKLFHESEGITRRQFFKGAGMLTVAAVISGVFAKFGFDVFAASDDYITQRVAGLYTLDESMAIRKSHENPEIIQIYKDFLSPGKVECVSPKAHHLLHTKYGKDIPGFIAELKNPHHVPEVESAANEETSTEEPAA*
>Syntrophomonas_wolfei_subsp.metylbutyratica_JCM14075T_01507_Periplasmic_[Fe]_hydrogenase_large_subunit
LSTVLSEGVIHVSNDCKACDHCTFICPTGAISGELGQKHHISPKKCINCGQCLINCPFNAIHDLSRIEELKKALADPQKFVVVQEAPAVRVALGEEFGQAAGTNVKNKMYASLRKLGFDKVYDTEFTADLTIMEEGTELIHRVYGALGVPGFEEVGPLPQFTSCCPAWIKYAEDNYPLVLPHISSAKSPQQMWGAVAKTYLAEKLNVDPANMFSVSVMPCTAKKYECERPEFISSGYQDVDLVITTRE
LAQMIKDAGIDFMALPDEEADRFVGLSTGAATIFGATGGVMEAALRTAYEILSGESLGKVEFDAVRGLDPVREATVEVPVKALGTSLPVSVCIVTGTKHVASVIEDVLAGRSKYHFIEVMNCPGGCINGGGQPIRRDTY*
>Syntrophomonas_wolfei_subsp.metylbutyratica_JCM14075T_01508_hypothetical_protein
MSPASIIVSEANISGARRVLSGSLFTKQAAGSNPSPRCSGGLKLLKQFLPMLKNLSST*
>Syntrophomonas_wolfei_subsp.metylbutyratica_JCM14075T_01511_putative_ABC_transporter_ATP-binding_protein
MSTERKTQTSPMGAHGGGPRGGPLGPMGRPAEKAKDFKGSFKRLLGYLKPRRFRLLVVLLFAVLSTVFSIASPKIMGKATTKLFEGLMMKLKHVPEATVDFNYILNILFILAGLYIISAIFMYIQQYIMAGVAQQTVYDMRRDVNLKLSRLPLKFFDSRTHGDILSRVSNDIDNIATTLQQSLTQLITAVVTLLGILMMLAISPWLTLIFLAVLPLSALVTKAVASRSQKFFAGQQKVLGELNGHVEEMY
TGHQIIKAFGREEESIARFKQINEELYEVGWKAQFMSGIIVPLLSLINNIGYVLVTVVGGIFVINRTITIGDVQAFIQYSRQFTQPIVQTANIVNIIQSTIASAERVFEILDETEEVADSDDAQLIAFPRGNVNLQNVKFRYQENVPLIEDLSIEVKAGQTVAVVGPTGAGKTTLVNLLMRFYEIDGGKITVDGVDIRDIKRTALRSIFGMVLQDTWLFNGSIRDNIAYGREGASEAEIIRAAKTAHADHFIRTLPE
GYNTILNEEASNISQGQKQLLTIARAILADPAILILDEATSSVDSRTEVYIQKAMIALMKKRTSFVIAHRLSTIRDADLILVMDNGSVIEKGTHNELLAQGGFYADLYKSQFSGGNIDEQAV*
>Syntrophomonas_wolfei_subsp.metylbutyratica_JCM14075T_01512_putative_ABC_transporter_ATP-binding_protein
MIKLYRFIKPFTVSILAILLLVFLQTLAELYLPTLMSDIVDIGIVNGDTGYIMKIGGLMLLITALGTVCAITASFLSARTSVGFGRILRSKVFSRVEDFSLQEFNEIGTATLITRTTNDINQIQTVTFMILRMMVSAPMMCIGGIIMAVSKDRPLSLCIVVVIPVIALIAIIISKAMPLFKLMQIKLDKINLVLRENLTGIRVIRAFNRIEREQQRFAEANYDLCQNAIRVNKLMASLMPSMMLVMNFTIIAIIWFGSIRI
NNGDMQVGSLMAFIQYAMQIMMSLVMVAMMFIMLPRASASAIRINEVLETNPQIKDPEETLSADKAQGILEFKNVSFSFPGAEKPAISNISFTARPGETTALIGGTGSGKSTLINLIPRFYDVDEGSVLVDGVDVREMSQKSLRAKIALVPQKSVLFSGSISDNIRYGKQDASDEEVRRAAEISQAIEFISGMPGGFDAVIEQGGSNVSGGQKQRLSIARALVRRPEIYIFDDSFSALDFKTDARLRLALKKET
ADATVIVIAQRVTTLMDAGQIIVLEDGRIVGMGTHKELLNSCDVYREIVYSQLSEEELA*
>Syntrophomonas_wolfei_subsp.metylbutyratica_JCM14075T_01513_Staphylococcal_accessory_regulator_Z
LSEKHELLVELLREVNRGVGKYVKNVLAEHDISVPAVIICKQLKGEAGITISELARRTGMAKSHISNIIRELEQKGWVRKKTDSSDHRVLRLYLNEAASAHLELLRIDIRQNINALVADITEQKAEDLIQGLREIIEALDKVRTEENGCEGKRRQ*
>Syntrophomonas_wolfei_subsp.metylbutyratica_JCM14075T_01514_(R)-2-hydroxyglutaryl-CoA_dehydratase_subunit_beta
MADYKQMWANLGMDLEKHDVLCEVLPEAFGGVYLTQQDRPKGMDFYDFVVSEIHGVRPAEMLQHQKEGGKVFGTFCVYVPDEVVFAAGGIAAGLCGGSQFWVSGGEKVLPANTCPLIKASVGARLDKTCPFFLIPDMYVAENTCDGKKKAWEILAGEVPMHIIDIPQMKRDRDVAAFEAEIRDFVGVVEKVTGKKITAESLGEAIKLINNKRRALQRVYNARKASPVPISGKDALLLTQINFY
DDPARAAQMANNLADELEQRINNGTGVFPANTLRIMISGTPMAIPNWKLHHIVESLGAAVVVEESCTGTRYFENLVDETATTLDGQIRALAERYMKTNCACFTPNSARIDDIIRLADEYKVDGIIDTNLQFCNLYSTESYLIKQLMTERNLPIMHIETDYSEQDFEQLRTRVEAFLEMLRK*
>Syntrophomonas_wolfei_subsp.metylbutyratica_JCM14075T_01515_2-hydroxyglutaryl-CoA_dehydratase_component_A
MIVIGIDIGSVAAKGYIISNGSHHRAMIPSGWSPREAGQALIDQLLDASGLERDQVERIYVTGYGRVAFEHADKTVTEIKCHARGVAELYPEVRTIIDIGGQDSKVISTGEKGQVLDFAMNDKCAAGTGRFLQVMATALGLDVSELGDAEDPRQMQTISSMCTVFAESEIIGSLARGNPKGGIIAGLHQSVGKRVAAMVRRMGIKEQVAFTGGVAINPGVRRALEEEIGTKIIVPGACQYTGALGAALL
ALETLRRD*
>Syntrophomonas_wolfei_subsp.metylbutyratica_JCM14075T_01516_(R)-2-hydroxyglutaryl-CoA_dehydratase_subunit_beta
MLDKYIQGFASAREVNLMRIKEAKENGAKVVGIYCTYCPQELILAAGAIPVGLCGMSENPIAAAEETLPRNLCPLVKSSYGFAATDTCPFFHFSDLIVGETTCDGKKKMFEIMQVLKPVHVMQLPHVNNTKASFELWVEELRCLKKRLEKELQVTISDQDIWRAVEVINQEKQAIKAMFDLNRADPPPLSGMEMLTVAWSRSFSSDKGEMIENLNLVVREVANQPEDRRVKAPRVLLTGCPVGLGT
EKVIRLTEELGAYIVAMENCSGYKTLELQTDTNSADPIVALAEKYMAIPCSCMSPNPYRLKLIENMINEFRVDAVIDLTWQACHTYNIEAFEVGKLVKSKGLPYLHLESDYSNSDLESLKVRIEAMLEMVEK*
>Syntrophomonas_wolfei_subsp.metylbutyratica_JCM14075T_01517_Antitoxin_DinJ
MGKNTSNVSFRIDTDIKNQADRLFARLGLNMTTAFNIFLRQSIREGCIPFNITLNTPNAVTVAALLEAERIGSDPTVKCYSDVEEALRELKL*
>Syntrophomonas_wolfei_subsp.metylbutyratica_JCM14075T_01518_hypothetical_protein
MSRKIVWTTQFKKDYKLAEKRGLDIALLDDCIRILAAGEELSTKFRATIILPADGAVIASAISNPTGF*
>Syntrophomonas_wolfei_subsp.metylbutyratica_JCM14075T_01520_Endoglucanase_precursor
MQRKFWAGLLFLSLIFNSFIFSGLPLSAATSFPDSSSHWAKDYIQQLSSLNYLSGYPDGTFKPDRDMSKAEFITVLTLCLGVEASDKTTVNYKDTGKHWALGRINEAVKRGILIPAEDPDGLRPDESIKRSQAAAMLVRALGKTADNGTLPFNDRAAVEKSDYRGYIKTAYDLGLISGFPNGNFEPFRNMTRAQVCTVMSKFLTARGISTPVTPPSSSVTGNINTLALGERLFNPQTTPIYIKINFTDLRIS
SIVAAGSSVLINGNQKLELESTSDNPDLIIDNNRYAIRRYSLSGNKLVAYPGCRKFNRISPGTYTYNSDYIKLYINSVNSERYLSDLEIVDEYTVKLGEKNYNLSQDKITIQLDKDFYDIKRVIMGDSDTSPQLNKTDPVVFEGLSMSDILAIFTGTSTLNLQSSNRIDFIIDGKRYTMSQVKLDAKGNFTVNSTNYPSANVIMIIDGTQYKINHIDINNSRFIFYCDAGSTHEWVIINNEYHNPDDVRILWDGG
VYELNEIITVKRNILRIKGRQYDLDSSFKVRFDNKVYDIDRIDYDASMQATVIETGKTSTGYLASQPQKFVFFKGSRKLQEGANNVTIYVNYTWVDFNQILIVDPSRLTYKNSNYELIGSRIKIDRIEYKIVDSSWHGLNQVLDLYLEET*
>Syntrophomonas_wolfei_subsp.metylbutyratica_JCM14075T_01521_hypothetical_protein
MDYYSYAQALKYFLELYRRPVAVRFLSDEEVPPDYRSDLDLSFCQFVMLAQRGDKLLATADNLACANGAAALGLRPLPEKIRSGEMHSRLNLFASPELASQVSALTPRIPLGAYPRILLAALDEADFEPQVVILQGKPATLMWVLIADNYHKGSRYSFTTAVSQGVCVDTTVIPFLEGNINLSLSCFGGRGSTDQKEEEVLLGVPFKDLEEIVEVLPELKNTIMKRSIEKTAYKRLQAKLEN*
>Syntrophomonas_wolfei_subsp.metylbutyratica_JCM14075T_01522_GTP-binding_protein_HflX
LSKRNTWEKAILVAVKTGTAKEAEFNSSMQELAGLAEAAGAKVVATLTQARDKPHAATYIGKGKLQELEHLLAEKEPELLIFDSELSPTQLRKLEDKLQIKIVDRTLLILDIFGQRARSREGLLQVELASLEYRLPRLTGIGKELSRLGAGIGTRGAGEQKLELDRRHIRNRIQEIKRQMEKLEKTRELHKKQRRRAGYPVISLVGYTNAGKSSLFNALCQVAHSSGQPQVEANRRLFQTLDTTTRKIKLD
SHYEVLITDTVGFIQNLPHHLVAAFRSTLEEAVAADLLLHVVDLADPAYLDKIKVVEGVLEELGAEKERIMPVFNKVDLLEGISPIQAPPNYVSARTGQGIEELLGQIKNRLEAIS*
>Syntrophomonas_wolfei_subsp.metylbutyratica_JCM14075T_01523_Regulatory_protein_MsrR
MRIKDVFRKKIVLFGLIFLAAFGLGKIIGDQFTEQSVQTRISGGKQVNILLMGIDARSNEVNSRSDTMILASIDGKNKKVALVWIPRDTRVEVSPKRYDKINSVNYIKGPEAACKVVSDLLGIPVNYYVVSNFNGFARIIDILGGVDINVEHDMYHWDPDPKLQINLSQGQQRLDGQQALSFVRFRGVPTADIGRTGLQQTFVKALAEEVLQGSTILKLPRLLPEIAKNVKTNLPADDMIYLAKTARDFK
TEDMITQTLPGYSYTDPASGASYWQADEKIANGLVAALFAGKTFEVAQARPAWVKQKPAPSSSPGDSVEASELEAGQEEQAVETEEDLSSPEGYLEPVIPGEKENISGEEHSPLAPGPVEGSNSPENSGGSGTTPAPVPPDEGQKQPDTTQSDPLL*
>Syntrophomonas_wolfei_subsp.metylbutyratica_JCM14075T_01524_NYN_domain_protein
MGVAILVDLANIHLGLKFNYLNPPPYERLAQIINDYGATQGDIRIRKVYTDWNDGRLERIARQFASAGFRAEQVTRKASGRDRTDICLSLDALELFYSEDDLDTLVLAAGDADYVEVMQRLRSKGKRLFVITLEKNFASELLPYLTGHAIIEKELTSIGYDLIDYSPLLKFLYNLQQFHHSKTGGFVAASLYMRKLEEYNIVTQNTAKSLLSQMIENNVIAKYTVENPSTPEHPTSAIKLNESSEVVKNFIRN
YLD*
>Syntrophomonas_wolfei_subsp.metylbutyratica_JCM14075T_01525_Oligoendopeptidase_F,_plasmid
MEGQSKKREEISPEYKWKLEDIYADENSWEEDYKKLEEQIPELEKYRGVLGSSSARLLQVLEFSHELERKAEKLYVYARMRRDEDNGNSHYQALFDRVQRLSIELGSASSYIVPEIISLAPEKIRAFLDENQDLQLYSRLLDELMRQKEHILSPEEERILAMSADLSLAAKNIFGMLNNADIKFPVIQDEEGKAVELTKGRYGSFMESNERRVRKEAFTALYGSYGKVKNTLAMSLASNVKNDIFYSRVRKYPS
ALEASLDQDNISPEVYERLVETLHNNMEPMYRYLRLRKKALGLDELHMYDIYAPLVKEFKSKVSYEKSQEMVLKALQPLGDEYLAILRGGLKGGWIDVYENEGKTSGAYSWGCYDSHPYVLMNYDDKLDDVFTLAHEMGHALHSYYSNKNQPYVYSQYSIFVAEVASTVNELLLIDYLLAQSREPREKAYLINHYLEQFRGTVYRQTMFAEFEKKIHEQVEQGQTLTAEGLCHNYRELNRLYYGP
DTLLDEEIDMEWARIPHFYSAFYVYKYATGFSAAVALKEQILHEGKPAVDRYLEFLKSGSSDYPLNLLRKAGVDLTTPEPVEKALHLFARLVTEMEAVLNV*
>Syntrophomonas_wolfei_subsp.metylbutyratica_JCM14075T_01527_Putative_formate_dehydrogenase
VVDLVKLIINGKEVEAPAGSTILQAAELAGIEIPRLCYDKELSPLGACRLCVVEVKGNRLLPASCVTPVFQGMEVETESPAVMEARKTILELLIANHPLDCLTCEKAGACKLQDYCYRYGIKGSPFVGQKHAYAIDDSNPFIVRDLNKCILCGACVRACEELTGADNLSYLKRGFHRKATTAGDVDYIDSDCVFCGQCVAVCPTGALTEKTMVGQGRRWDIERVRTTCPFCGTGCNFDLAVKDGKVI
GVLSNPDAPVNQRSLCVKGRFGWDFIYNENRLTTPLIKKNGQFEPASWDEAFSLIATRFKEIKEKYGPDSFAALSSARCTNEENFLVGKFSRAVMGTNNVDHCART*
>Syntrophomonas_wolfei_subsp.metylbutyratica_JCM14075T_01528_Formate_dehydrogenase_H
MFLIGTNPTEAHPVIGYKMRQAARRGAKLVVCDPRHIDLVDEADYWLRLKPGTDIPLLNGLMHIIIKEGLEDKTFIEERTENYEELKAIVESYTPERVSELTGISVDDLYAVARLYATTDKAMLFYTLGITEHICGTRNVMSCANLAMLTGHLGRPGTGVCPQRGQNNVQGACDMGALPNVYSGYQAVGVDAVREKHEKAWEASLSPKVGLKIPEMFEAAHEGKVKAMYILGENPVLTDPDANH
IRGALEKLEFLVVHELFLTETAAYADVILPAASFAECDGTFTNTERRVQRVRKAIEPIPGQANWQTICQMFVAMGYPLNYNSPKEIWDEMASLSPSMAGINFERIDQENSLQWPCPSLDHPGTPVLHVGKFTRGLGLFQPSEHIPPGEIPDAEYPFLLSTGRILQHYNVTTPYSKGISSIWNKEMSELNPVDADKLGVVTGGQVKVTSRRGEAITQIKVTDRVLPGVIWMSFHYNATPTNALTSHHLD
PITGTGEYKVCAVKLEKV*
>Syntrophomonas_wolfei_subsp.metylbutyratica_JCM14075T_01529_NADP-reducing_hydrogenase_subunit_HndA
MADYREIISAYKEVPGGIIEAYHAVQKEYSYIPEDAVVYAAQVFDMPKAKAYGVATFYSYLQVGPRGKNVIRICESAPCHIAGADKVVAALEKELGIKMGETTPDGKFTLEFAECVGQCQATPVITINSQPYGDVTADKVAAILAEYK*
>Syntrophomonas_wolfei_subsp.metylbutyratica_JCM14075T_01530_NADP-reducing_hydrogenase_subunit_HndC
MAEEMRIVLRNYGKVDPLNIDSYTQVGGYQALAKAKSMSQPDLIEEVKKSGLRGRGGAGFNTGMKWGFSYGVKADQKYVICNADEGEPGTYKDRIIMENDPQSVLEGMAICGYAIGANKGYIYCRGEYPYVVEILNKAIAQASEKGLLGDFNIEVRMGAGAYVCGEESALIESIEGHRGEPRFKPPFPPVIGLWGKPTIVNNVETFANIPAIVDKGADWYKGIGAAGYPGTKVMTLTGDIVNRTVIE
VPTNTTIRQVLNDFGGGIAGGKKFKAVQIGGTSGGFIPESLLDTPIDFDSMSAIGATLGSGAFFVMDETRDIVDVIDRISKFFAHESCGKCTPCREGTQRMHEMLHRVKNGGAVAGDLDFMERLGSVMSKACLCGLGQAAPAPVLTTLKHFRNDYTAKFN*
>Syntrophomonas_wolfei_subsp.metylbutyratica_JCM14075T_01531_Molybdopterin_molybdenumtransferase
MSRLQLVNDKILEAVSLDKAVDISCESIQALDTEYLHIDNAYMRVAADNIASFIDIPNFDRSAMDGYTIGKTDLKRLQTGRSLRLRVASIIGAGSIENRPTRPGETCRIMTGALLPEGSVAVIKQEDVEIINENYIIVSIRPRRGENIRRAGHELMAGDRVAAKGQVLKAEILERIAACGIERVPVYKVPRVYIIDTGNELLLPGSPIKTGCIYCSNRSLLACKINSSRALPSLSPSVINDDLHTIIAAIKKAARVSDMII
ISGGTGNGLFDLVRNAFEYLNAQLLFKGINVFPGKNTSAALLDGKLIFNLSGSPSAAGILFEALVKPALLKLKGELLYKGDWFNIELASPIEHLKSGRSLHRGEMIIEQGHAYALPVNRKDINTGNIPLLLDVHKRSGEEPVMVKAKLIVD*
>Syntrophomonas_wolfei_subsp.metylbutyratica_JCM14075T_01533_Formate_dehydrogenase_subunit_alpha_precursor

MKFTRRGFLKLSGLSVAAVASGLGFDLEATAHGHALKLAGCTKIPSICHFCSGGCGLLLHIKDGKLVHLDGNPDNPINEGTLCPKAASLAQVAYSPERPKNPLYRAPGSDEFVDITWEEAYDRIAKKIKEVRDKTWTSTEEITNAKTGAKETVTVNRAEGICMIGSAEVDNEESYLLSKLARLIGTPFHEHQARI*
>Syntrophomonas_wolfei_subsp.methylbutyratica_JCM14075T_01535_Formate_dehydrogenase,_nitrate-inducible,_major_subunit_precursor
[illegible]
>Syntrophomonas_wolfei_subsp.methylbutyratica_JCM14075T_01536_Formate_dehydrogenase-N_subunit_beta
[illegible]
>Syntrophomonas_wolfei_subsp.methylbutyratica_JCM14075T_01537_Formate_dehydrogenase-O_subunit_gamma
[illegible]
>Syntrophomonas_wolfei_subsp.methylbutyratica_JCM14075T_01538_formate_dehydrogenase_accessory_protein_FdhE
[illegible]
>Syntrophomonas_wolfei_subsp.methylbutyratica_JCM14075T_01539_putative_molybdopterin_biosynthesis_protein_MoeA/LysR_substrate_binding-domain-containing_protein
[illegible]
>Syntrophomonas_wolfei_subsp.methylbutyratica_JCM14075T_01540_hypothetical_protein
[illegible]
>Syntrophomonas_wolfei_subsp.methylbutyratica_JCM14075T_01541_hypothetical_protein
[illegible]
>Syntrophomonas_wolfei_subsp.methylbutyratica_JCM14075T_01542_Staphylococcal_virulence_regulator_protein_A
[illegible]
>Syntrophomonas_wolfei_subsp.methylbutyratica_JCM14075T_01543_Nicotinate_dehydrogenase_FAD-subunit
[illegible]
>Syntrophomonas_wolfei_subsp.methylbutyratica_JCM14075T_01544_4-hydroxybenzoyl-CoA_reductase_subunit_gamma
[illegible]
>Syntrophomonas_wolfei_subsp.methylbutyratica_JCM14075T_01545_putative_xanthine_dehydrogenase_subunit_D
[illegible]
>Syntrophomonas_wolfei_subsp.methylbutyratica_JCM14075T_01546_Molybdenum_cofactor_cytidylyltransferase
[illegible]
>Syntrophomonas_wolfei_subsp.methylbutyratica_JCM14075T_01547_putative_xanthine_dehydrogenase_subunit_A
[illegible]
>Syntrophomonas_wolfei_subsp.methylbutyratica_JCM14075T_01548_hypothetical_protein
[illegible]
>Syntrophomonas_wolfei_subsp.methylbutyratica_JCM14075T_01549_hypothetical_protein
MRVFAEMNRWGSITTSISRKWQRLLSDNP*
>Syntrophomonas_wolfei_subsp.methylbutyratica_JCM14075T_01550_Histidinol-phosphate_aminotransferase_2
[illegible]
>Syntrophomonas_wolfei_subsp.methylbutyratica_JCM14075T_01551_hypothetical_protein
[illegible]
>Syntrophomonas_wolfei_subsp.methylbutyratica_JCM14075T_01552_CRISPR-associated_endoribonuclease_Cas6
[illegible]
>Syntrophomonas_wolfei_subsp.methylbutyratica_JCM14075T_01553_hypothetical_protein
[illegible]
>Syntrophomonas_wolfei_subsp.methylbutyratica_JCM14075T_01554_hypothetical_protein
[illegible]
>Syntrophomonas_wolfei_subsp.methylbutyratica_JCM14075T_01555_CRISPR-associated_protein_Cas5,_subtype_I-B/HMARI
[illegible]
>Syntrophomonas_wolfei_subsp.methylbutyratica_JCM14075T_01556_Helicase_Cas3
[illegible]
>Syntrophomonas_wolfei_subsp.methylbutyratica_JCM14075T_01557_CRISPR-associated_protein_Cas4
[illegible]
>Syntrophomonas_wolfei_subsp.methylbutyratica_JCM14075T_01558_CRISPR-associated_endonuclease_Cas1
[illegible]
>Syntrophomonas_wolfei_subsp.methylbutyratica_JCM14075T_01559_CRISPR-associated_endoribonuclease_Cas2
[illegible]
>Syntrophomonas_wolfei_subsp.methylbutyratica_JCM14075T_01560_hypothetical_protein
[illegible]
>Syntrophomonas_wolfei_subsp.methylbutyratica_JCM14075T_01561_CCA-adding_enzyme
[illegible]
>Syntrophomonas_wolfei_subsp.methylbutyratica_JCM14075T_01562_mRNA_interferase_PemK
[illegible]
>Syntrophomonas_wolfei_subsp.methylbutyratica_JCM14075T_01563_Antitoxin_ChpS
[illegible]
>Syntrophomonas_wolfei_subsp.methylbutyratica_JCM14075T_01564_Cellulosome-anchoring_protein_precursor
[illegible]
>Syntrophomonas_wolfei_subsp.methylbutyratica_JCM14075T_01565_hypothetical_protein
[illegible]
>Syntrophomonas_wolfei_subsp.methylbutyratica_JCM14075T_01566_ABC_transporter_ATP-binding_protein_YtrB
[illegible]
>Syntrophomonas_wolfei_subsp.methylbutyratica_JCM14075T_01567_HTH-type_transcriptional_repressor_YtrA
[illegible]
>Syntrophomonas_wolfei_subsp.methylbutyratica_JCM14075T_01568_hypothetical_protein
[illegible]
>Syntrophomonas_wolfei_subsp.methylbutyratica_JCM14075T_01569_putative_protein_encoded_in_hypervariable_junctions_of_pilus_gene_clusters
[illegible]
>Syntrophomonas_wolfei_subsp.methylbutyratica_JCM14075T_01570_hypothetical_protein
[illegible]
>Syntrophomonas_wolfei_subsp.methylbutyratica_JCM14075T_01571_Putative_anti-sigma_factor_antagonist_BtrV
[illegible]
>Syntrophomonas_wolfei_subsp.methylbutyratica_JCM14075T_01572_Serine/threonine-protein_kinase_BtrW
[illegible]
>Syntrophomonas_wolfei_subsp.methylbutyratica_JCM14075T_01573_Low-molecular_weight_cobalt-containing_nitrile_hydratase_subunit_alpha
[illegible]
>Syntrophomonas_wolfei_subsp.methylbutyratica_JCM14075T_01574_RNA_polymerase_sigma_factor_SigM
[illegible]
>Syntrophomonas_wolfei_subsp.methylbutyratica_JCM14075T_01575_hypothetical_protein
[illegible]
>Syntrophomonas_wolfei_subsp.methylbutyratica_JCM14075T_01576_SagB-type_dehydrogenase_domain_protein
[illegible]
>Syntrophomonas_wolfei_subsp.methylbutyratica_JCM14075T_01577_hypothetical_protein
[illegible]
>Syntrophomonas_wolfei_subsp.methylbutyratica_JCM14075T_01578_bacteriocin_biosynthesis_docking_scaffold,_SagD_family
[illegible]
>Syntrophomonas_wolfei_subsp.methylbutyratica_JCM14075T_01579_Glutamine_synthetase_1
[illegible]
>Syntrophomonas_wolfei_subsp.methylbutyratica_JCM14075T_01580_hypothetical_protein
[illegible]
>Syntrophomonas_wolfei_subsp.methylbutyratica_JCM14075T_01581_Hypoxanthine-guanine_phosphoribosyltransferase
[illegible]
>Syntrophomonas_wolfei_subsp.methylbutyratica_JCM14075T_01582_GMP_synthase_[glutamine-hydrolyzing]
[illegible]
>Syntrophomonas_wolfei_subsp.methylbutyratica_JCM14075T_01583_hypothetical_protein
[illegible]
>Syntrophomonas_wolfei_subsp.methylbutyratica_JCM14075T_01584_long-chain_fatty_aldehyde_decarbonylase
[illegible]
>Syntrophomonas_wolfei_subsp.methylbutyratica_JCM14075T_01585_hypothetical_protein
[illegible]
>Syntrophomonas_wolfei_subsp.methylbutyratica_JCM14075T_01586_Extracellular_esterase_EstB_precursor
[illegible]
>Syntrophomonas_wolfei_subsp.methylbutyratica_JCM14075T_01588_N5-carboxyaminoimidazole_ribonucleotide_mutase
[illegible]
>Syntrophomonas_wolfei_subsp.methylbutyratica_JCM14075T_01589_Adenylosuccinate_lyase
[illegible]
>Syntrophomonas_wolfei_subsp.methylbutyratica_JCM14075T_01590_Amidophosphoribosyltransferase_precursor
[illegible]
>Syntrophomonas_wolfei_subsp.methylbutyratica_JCM14075T_01591_Phosphoribosylformylglycinamidine_cyclo-ligase
[illegible]
>Syntrophomonas_wolfei_subsp.methylbutyratica_JCM14075T_01592_Phosphoribosylglycinamide_formyltransferase
[illegible]
>Syntrophomonas_wolfei_subsp.methylbutyratica_JCM14075T_01593_Bifunctional_purine_biosynthesis_protein_PurH
[illegible]
>Syntrophomonas_wolfei_subsp.methylbutyratica_JCM14075T_01594_Phosphoribosylamine--glycine_ligase
[illegible]
>Syntrophomonas_wolfei_subsp.methylbutyratica_JCM14075T_01595_hypothetical_protein
[illegible]
>Syntrophomonas_wolfei_subsp.methylbutyratica_JCM14075T_01596_hypothetical_protein
[illegible]
>Syntrophomonas_wolfei_subsp.methylbutyratica_JCM14075T_01597_hypothetical_protein
[illegible]
>Syntrophomonas_wolfei_subsp.methylbutyratica_JCM14075T_01598_ATP_phosphoribosyltransferase_regulatory_subunit
[illegible]
>Syntrophomonas_wolfei_subsp.methylbutyratica_JCM14075T_01599_ATP_phosphoribosyltransferase
[illegible]
>Syntrophomonas_wolfei_subsp.methylbutyratica_JCM14075T_01600_Histidinol_dehydrogenase
[illegible]
>Syntrophomonas_wolfei_subsp.methylbutyratica_JCM14075T_01601_Imidazoleglycerol-phosphate_dehydratase
[illegible]
>Syntrophomonas_wolfei_subsp.methylbutyratica_JCM14075T_01602_1-(5-phosphoribosyl)-5-[(5-phosphoribosylamino)methylideneamino]_imidazole-4-carboxamide_isomerase
[illegible]
>Syntrophomonas_wolfei_subsp.methylbutyratica_JCM14075T_01603_Imidazole_glycerol_phosphate_synthase_subunit_HisF
[illegible]
>Syntrophomonas_wolfei_subsp.methylbutyratica_JCM14075T_01604_Phosphoribosyl-ATP_pyrophosphatase
[illegible]
>Syntrophomonas_wolfei_subsp.methylbutyratica_JCM14075T_01605_Hemolysin_C
[illegible]
>Syntrophomonas_wolfei_subsp.methylbutyratica_JCM14075T_01606_hypothetical_protein
[illegible]
>Syntrophomonas_wolfei_subsp.methylbutyratica_JCM14075T_01607_Anti-sigma-V_factor_RsiV
[illegible]
>Syntrophomonas_wolfei_subsp.methylbutyratica_JCM14075T_01608_hypothetical_protein
[illegible]
>Syntrophomonas_wolfei_subsp.methylbutyratica_JCM14075T_01609_phosphodiesterase
[illegible]
>Syntrophomonas_wolfei_subsp.methylbutyratica_JCM14075T_01610_hypothetical_protein
[illegible]
>Syntrophomonas_wolfei_subsp.methylbutyratica_JCM14075T_01611_hypothetical_protein
MARVKEITVGVYQVGGDGFSRSEDCCVYLVDGGESALIDAEKIVSRGINPPAILA*

>Syntrophomonas_wolfei_subsp.metylbutyratica_JCM14075T_01612_hypothetical_protein
MLKVYPVILTPSDSGYVVTVPDLDINTQGKDLAEAIYMARDAIGLWGICEQDDGRIIPEPSTSEPPHEDNELVSWVDIDFEKYRKANDLITMRINVSVPKYLKVLGDEAGINFSQTLQKALKQQLGIQD*
>Syntrophomonas_wolfei_subsp.metylbutyratica_JCM14075T_01613_YcfA-like_protein
MKRKDLIKLLEKNGWWLKREGHDHTIYTNGTANEPISRQTEIDDILAKKIIKRRGLG*
>Syntrophomonas_wolfei_subsp.metylbutyratica_JCM14075T_01615_Metallo-beta-lactamase_L1_precursor
MARVKEITAGVYQVGGDGLSRAEDCCVYLLDGGGESALIDTGAGRSASMIWNNIIAAAVDMSGLKYIVVTHGHIDHIGGLAFLKEKLQAQVIAHSLELPAIEEGLPQLTAASWYGVTYEKVKVEQVLKGEEQRLKLGQLELICLHTPGHTPGGISPYVDIAGKRVLFGQDIHGPFNQQWGSDIEQWRISMQKLLELEADILCEGHFGIYSPAAAVRKYIEGYLRQYARK*
>Syntrophomonas_wolfei_subsp.metylbutyratica_JCM14075T_01616_Manganese_transport_regulator
MHVTQSITPALEDYLEVIYRLSEENGRAKISDIARRMDIAKPSVTQAITALRNEGMVYQERYGPVYLTAKGEKKAREVWHRHQVIRQFLREVLQVNPATADRDACLMEHSISAETFSRMEKWLQLPEEAPEKKHPVEKALLPLSELLPGQKARLVKIVGTESWLRQRLLEMGLVPGVKLELERVAPLGDPIEIGLKGFHLSLRREEASMLLVKVLEREIN*
>Syntrophomonas_wolfei_subsp.metylbutyratica_JCM14075T_01617_hypothetical_protein
MTVCILVFSLCVLLIVLILIKRVKRLLRTRFGPTFEDAGKESVMMSYAASGLKQPANGKRVLEEKDDYENKNQ*
>Syntrophomonas_wolfei_subsp.metylbutyratica_JCM14075T_01618_hypothetical_protein
MIMKTKINSCSAGLLMPERKKIWELRPNLHCSICGTCLSIEEQRQILKKMKVPEKDYRDYEIHAIVANNLFRENMVSCMINAYLDKKYRVEIARFGFLEEAKLMMIWRDKMAEGDICGLYWAVLTNPLLPEESINRVVGEVHMLSHLNGGLCRQERMKLKRLAEEKQKYVVRLRQCRSREKELAAELDAARICIAKMERQLQEQNTRSRSSEDGQDYRQMLNSLKIENNELRLKLEELNRKCQDYKE
ESRQLLRDNDELEKQVRQQKEAIIQLCRESKMMARCQALDSGISAGGEAGEIKKRRLLLVGGNSGLNNHYRDLINGMGWEFRHHDGCLNGGRQSLTEMLKWSDLILCSLDANSHGAVHCVKELAPKLHKEYRLLGNSSLSGISRALAEQSDTCTA*
>Syntrophomonas_wolfei_subsp.metylbutyratica_JCM14075T_01619_hypothetical_protein
LGLKRMENVLDYLIALDRKRPLEEMSCAETRDLLQALGWKDNRDYEFARHKLLRLVKFITR*
>Syntrophomonas_wolfei_subsp.metylbutyratica_JCM14075T_01620_Cyclic_di-GMP_phosphodiesterase_response_regulator_RpfG
VIKMLAEKLDNKTIENRNTVPVSLNKLIAALSAAVDLAGSSDRKHSLRTAYIATRLASNMSMEKAEVDNIYYAALLHDLIPAQSYFNQSLIFEILGNLPLSSQVAKQVVALWQYKQGNGIASRLGHLSLSERIIYLAECFENQYCNGESEEYLLRRSLSNWSNRVAAAADKKIAAALADCLREEAFWQDLKENRVQRALEQMAPDWRQQLNIDDLEKVSRSFSLLIDRKNPYTGQHSQRVGMIAGCCAR
LYGLDSAMSRRVSIAGYLHDLGKLSIPEVILDKAGSLTEREMQAIRAHPYDSYAVLSEIEDFTDIARWAGNHHERMDGSGYPWGRSDLTVVDQIVAVADIYEALTSNRPYRRALSQGDALSLIKKQVAKGKIRLEAYELLEMTVESKNSCKEADNLANARGSSCWFRVYSGFDAKQIAAEGLKRIDRI*
>Syntrophomonas_wolfei_subsp.metylbutyratica_JCM14075T_01621_hypothetical_protein
MAVIGVQRLRAILADLEKENDDLHYMSPEQSEVLFNRYLAHLQGISKETEKNRQKALRFEDSRSV*
>Syntrophomonas_wolfei_subsp.metylbutyratica_JCM14075T_01622_Ferric-citrate-binding_protein
MHIMKSLTSKMAAMIISLLLLAIILPACDSGTEKEAKSSRTIIDQRGKQVKVPGEINRIVTLPIPLPSMIVAVDGNGKRIVGMHPESKSAVEKSTLKNISPELMNVSTAFVQEGFAVNIEELVKLQPDVVFQWKHQPEEIEKIENAGIPVIALQYGTQQDLEAWLKILGELLDKKQQVNKIINYHHEVINDIRNRTSTINEKDKPRVMYLNGPEPSTNGGDSYNHFWITASGGCNVAADIEGTGKYVNMEQ
VLRWNPEIIYIGNFCNLQADDLFKNKIQGQDWSHVAAVKNRRVYKIPLAGYRWDPPSVESPLMLKWLAQKHYPEIFADYSMAAELKEFYRNIYNYELSDEEVARVLKD*
>Syntrophomonas_wolfei_subsp.metylbutyratica_JCM14075T_01623_Nitrogenase_iron_protein_1
VRRIAIYGKGGIGKSTTTSTLSAALSTMGYRVMQVGCDPKADSTTTLTAGKTLTPIMDYLKEHGKCDSLEEITVQGFNGIVCVETGGPTPGIGCAGRGIITSFNVLDELGAYQVFNPDFVFYDVLGDVVCGGFALPLRQGYADEVIIVTSGEPMALFAAGNIKKALDNFAERDYAQLRGLILNLRNIEREEEIVYDFAERMGVPVFGIIPRDKNIQLYESRQQTVIEGDPALNISRSIMKLAEQIIDLGEAS*
>Syntrophomonas_wolfei_subsp.metylbutyratica_JCM14075T_01624_light-independent_protochlorophyllide_reductase_subunit_B
MACAPYSLSITELKTAVESGTRKLLDGGSLRYCPPSAGGWGIIRVGLLVPESIMLFVSPAGCGRHGAIAGLQLGFRQRLFFLYVDEVDLVTGRHLERAKEAVEEILASISRPRAIMICATICIDDLLVSDYEKLAEEVENDYRIPVRACHMNPIAMDGKKPPQLTIQKAIHEFIPAVKQKEQAINMLGSFAAINRGSEFYELMAGAGVSPVRHIAACSTFDDLCSMGRSFYNVLIKPGGKLAAEYMAKALNIPY
CYAPVSYGLEAIAASYRKLEEQLGISLDTAAYYEKTEKAINYYRKILGSISIAVGENINACPFELARALLSYGFEVPYIFTDQVLDIDRENINWLAERRPHIKVFTNAHPSMANFLDEKLKVDLAIGFDAGYFCSGAKTAALSMDCQAYGFEAANSLLKEMTLAMNNNPHSHREQMYAAGLVL*
>Syntrophomonas_wolfei_subsp.metylbutyratica_JCM14075T_01625_Nitrogenase_molybdenum-iron_protein_alpha_chain
MKGLYKYLPPFAPDYSGVCSVLFNLNGIVIIHDASGCTGNFTGYDEPRWYGSNAAIFSSGLREIEAVLGDDERLINRIMEAVERMPRNFVALLGSPAPAVIGTDMQALARILKARSGLPVLSFDTTGIKYYDTGASQAYLEIARNFVKPWNGKKQALVNILGAIPLDMGKGKRVRDIGLLLRQCGFSKVYAWGQNAGLKEISQAGAASLNLVVAYSGLAVARYLHEHFATPYMVGIPIGQGSTNQFMR
TASSVLGLETPPRTTPYLLPPNTYAQALVIGEQVMAGSIRSFLRLDWGIRKVNVASFFAMDKELWEEGDKFLEEEDDLSSLCQKENYDLIIGDPLYENLLGANQGRFIHFPHVAVSSRLYWDNEPACVSKQLEGLSSKLKTPARRT*
>Syntrophomonas_wolfei_subsp.metylbutyratica_JCM14075T_01626_putative_ABC_transporter_permease_protein
MRAKRSSSDESKKSTLIMTILILMPLLAFFLSLCIGRYHIEAGRTAGILFSAIAHIEQAGLELEQSIVLDIRLPRALLAMMVGAGLAVSGAAFQGLFGNPLVSPHILGVSSGAGFGAAMGILLSGHLAVVQLLALLFGILAVMATFMISRLKGSTQLFMLVLAGVIVGAFFQALISLIKYVADPQDKLPTIVYWLMGSLTGASFHDLLIAIPLIGAGIIIIFLLRWRINILSLDEDEARSMGINVQLTRWIVIAAATITT
ATSVSLCGIIGWVGLVIPHIGRMLVETDHRKLIPACISIGAFYLLIDDIARTATAAEIPLSILTAVIGAPFFAYLLRKTGGKWS*
>Syntrophomonas_wolfei_subsp.metylbutyratica_JCM14075T_01627_putative_ABC_transporter_ATP-binding_protein
MKISVEQGSFGYPGSRTLFENLCFEVERGEILAILGPNGVGKTTLLKCMTCMLPWNRGAAYLDGQNIKYFDSVDIWRHLAYVPQVHASVFSYSVLDMVLMGRSAHINWFSMPGKNDRRVARNALESVGIGHLARRICSELSGGERQLVMIARALAAEPEIIILDEPESHLDFRNQLMILDILESISQQREISCIINTHYPEHALRISDKTLILGKECDYVVGETEEVISEDNLQKFFAVRTRLLSYTDRGVA
RKTVVAVAI*
>Syntrophomonas_wolfei_subsp.metylbutyratica_JCM14075T_01628_hypothetical_protein
MKRAMMSVGLCALCLLLVSVYMLGCSDKSSNNIEKELPETRTITDQYGRQVEIPAEVNRV*
>Syntrophomonas_wolfei_subsp.metylbutyratica_JCM14075T_01629_hypothetical_protein
MGRLIDDIQHMKMDVEHVKNDVEHMLRDLENLGDGPNAEDIKTHLRDMENHMEDLYSHVSHVENHE*
>Syntrophomonas_wolfei_subsp.metylbutyratica_JCM14075T_01630_hypothetical_protein
VGTGSIPDYAYVKKNPFDVVADSDKFSIAFVFPEAFEELLESDVEQELCYSYRLNGHITDNRLKDLIAEMDIDEAMVQNCYMSEIIDEVEGKAG*
>Syntrophomonas_wolfei_subsp.metylbutyratica_JCM14075T_01631_Regulatory_protein_PchR
MNNHLIMKDIDEYYQVMNSELGIFEQIGSTCCYQIHPRWGKGLAERIILGNGVEINIWNMELVQDLKLVWQLEERCFEIMHCVEGHACYGGCECKNEKQIKTGEYGCWTNDGKQNWVHFSAELPWKSISICYAEGFIKSFLEMAPSLTDRQEVKSVLDYYNKGNDSCYLVSPDTELVFSEIMNCSQKGIARLLYIKSKVLEIFSLFIQNELLLRKHKNYKIFLSREDRLKLEQAKRIIVDNMLDPLSVAE
LALQIDLNVYKLKVGFKEMWGTTIFGYLRDMRMEKARFLLSGTHKNVAEVAQEVGYSNPSHFSTAFRKKYGINPHEYANHYRSL*
>Syntrophomonas_wolfei_subsp.metylbutyratica_JCM14075T_01632_Antitoxin_HicB
VQLVYPACFYPCEEGGYTVIFPDLPGCVTEGDDLLEAMHNAIDAASGWLLFLIEDEKQQIPEAADIKAIIPDEYENGFVSLINIDLNEYSKKYGHKAIKKTLTIPAWLNSLAEENNLNFSQILQRALIDHLGIKHP*
>Syntrophomonas_wolfei_subsp.metylbutyratica_JCM14075T_01633_Plasmid_stabilisation_system_protein
MSKWKVVYTEQAERDLCSIFEYIAFSLLEPETAKNQSRRIMDTVAKLNEMPLRYPLYQKEPWHSKGLRVLPVDNYLAFYLPVETKTTVAVIRIMYGGCDIEEQLRQTEAGG*
>Syntrophomonas_wolfei_subsp.metylbutyratica_JCM14075T_01634_addiction_module_antitoxin,_RelB/DinJ_family
MARTSNIFARVEPEVKEQAELVLEQLGIPMSNAIGLFLRQVVLQRGIPFELKLPQSKPLSVSSLTEEQFNAEIEKGLADLTAGRVISAESITDRMRQDYGI*
>Syntrophomonas_wolfei_subsp.metylbutyratica_JCM14075T_01635_hypothetical_protein
VPLEVNETLLDDMPDSAGARMGYIEKIDLGCSKNACGNCYMKAGGDSHKPLMWAITKKGIDFMGKQTLI*
>Syntrophomonas_wolfei_subsp.metylbutyratica_JCM14075T_01636_cysteine/glutathione_ABC_transporter_membrane/ATP-binding_component
MNNAALVRKKTGIPRLLEIAGEKRGLLVLSGVLSVISTVLMFFPPVAAYFIVAELLQHAASPALSDMDSIRQWGLWALYALLGALLFLYASTMASHIAAFRILYSLRVRLTEHLARLPMGYHTRQSSGTIKKLLELSVEKIENFIAHQLPDLVGAVALPVIMLTVMFILDMAPGGCLCRPYYSGLFPAGAGVLWRGGDGQYQRYA*
>Syntrophomonas_wolfei_subsp.metylbutyratica_JCM14075T_01637_Iron_import_ATP-binding/permease_protein_IrtA
MANIRDMHDTQERMNAAGVEYVRGMPAVKVFGLTVSTFLQFHNSITGYRDWAVQYVRFCKRPYMVFITILASLIVFILPVGVFILSQKPDNQAFALTLMLFLVVAPGFVTPMMKLMNLGGNMRIIAEGVERIDEIFAQEPVAEPINPQKAEGYNIEFDHVVFSYDDPAVSTRTEALAGVSFTAREGELTALVGPSGGGKSTIAYLIPRFWDVGTGAIRIGGVDIREMGSEQLMDMVSFVFQDVHLFY
DTIEENIRMGRSDAGYEEVVAAAKAASCHEFIECLPQGYQTRIGEGGTYLSGGEAQRVAIARAILKNSPILVLDEATAFADPENESKIQAGLSELIKGKTVIIIAHRLSSIREADQILVVDEGRIVERGRHNELIDRDGLYRRMWRAHIDADSWTIAAEQASKAVNE*
>Syntrophomonas_wolfei_subsp.metylbutyratica_JCM14075T_01638_Putative_multidrug_export_ATP-binding/permease_protein
MRNFFYNITSGNPTALLVPSLASFFDGLGKIIPAALIFDVVNTMYLFFAHPGTPLNTGRLWTVCAILLAWMLLQYLVSGWAYSQTFTAAYAVSAQGRIALAEHLRRLSLGFLGSRDPGDLTTMMLGDYAAVELTISHYVPQLVSATALPVIAFIGFSFINWKMALAMFIALPLAILIVWLSAGLQARLGLNHVKAKVDSASRLQEYLLGMREIKSHNLSGERFERLREAFARLMRESIKIEGVVGPIMMAA
IALVRAGLTLMIFVGSYLLVGGELTLPVFLMFLVLGNMVFEPLTGVLINYAEIRYSVLSAERIMQIRREEPLAGAKNPPPGNHIEFDHVTFAYEDALVLKNISFSIPPGSITALVGPSGSGKSTITRLIARFWDVQGGEVLLDGQNIKEIDPEKLLTRISMVFQDVYLFKDSIRNNIKVGKMDASQEEIEWAARQACCHEFIMKLPQGYDTPVGEGGCTLSGGEKQRVSIARALLKDAPIVLLDEATASLDPE
NENQVQRAINALVSNKTVVIIAHRLKTVKKADNIIVLDDGRVAEQGRHEELLANQGLYHHLWTLQQESAGWSMKAS*
>Syntrophomonas_wolfei_subsp.metylbutyratica_JCM14075T_01639_Endo-1,4-beta-xylanase_A_precursor
MKGKSRIVSNGRTLRKIIGMLLAVAMVVALLPITAYATFSDMQGHWGQTAIEKWSDMEIVQGWDGAFRPNAPITRGEMAVIIDRVMKYQTKSKNSFSDLGSAFYTDAILKANAAGVIKGDGTTVRPMDNITREEAAVMLGRALGLSENPTGGTAFSDYADISGWAVGGVNAMANKGYVKGFNAAFHPKRSITRAEVVAILDNSIKQLYNGNKEYTGNVSGTAIVNTPGAVLKNMKISGDLIIAEG
VGDGDITLNNVTVAGNTLLRGGGSDSIHIQGGSQISNLIIEKTDNGAIRVVTSGGAVVETVWVDDGRDDIILTGSFESITVAADVTVKAVDAKIERVEVTVKNAVLDVDKESAIDALNVTGTAANTEIKVFGKVGTLTADAKISVNNQGIIARAQINANDVSINGNAPATMNVAATVTTRPATGSAPSSDGSSGGGGSSSPSFAGGSGTESSPYQIATAAQLDQVRNYLDKHFVLTADIDLSGYENW
TPIGAFRPLSDAPEHVETPDPEAAFSGTFNGDGHTISNVRINQPEGMFVGLFGCTVGQEDNPTSIYNLTVENVDVTGFHLVGGVVGMQYSGCILENVNLISENTGDNTVCGNQGVGGITGISFEDMKNCRAEADIVVLGDDGAAAGVLAGGMEGCSTIECRVTGGTVRAEGKNCWGLGGLAGCIFKGIAATNCHVANVSITASGANTRLVGGLLGFTGTYDNDPPTQITECSADTTITVSDS
TTRVGGLVGGSYDDETKWPVPSVYAIANCETHGSITGGSEALGSIAGYAYRSTVENCRSDMTINGDSNPPQVVQAEGFENLSAWANRGSGSFESLVNYVNDDALNDVWNAAASSEMAQMMAGMFGATVTDGASLKAMFANMASTPANNGTQIYQIKFAYDYKTIYFCDNDGHVIASHPYTFVERNDNLGMLAGETTYVFKADDDAGVLTYMAMLKPHFDEGMDETTMPAHIHFYYAAT
KDTLGTYKPDSVTLNGWMPTMLQMGDEFSTADKTALLKCLFGINNAPEEPVAATGILVVAHGSDENWNQRVRESVTQVEVPYPVELGFLDVETENIAMAVQALEQKGVKRIVAVPIFVCSASSHMEEIKYMLGLPSSLNDEAAQKKGLERIEKTATVELTPALDDHLLVAEIINERLKNLSQNPEKEIVVLVAHGTSDPENLAVWKEKMASLGDKLQQIQGFKQVCCGFVGAGTPKIRTVVEEV
KNSNPDCTVIVMPVMLSAGVYTDSKIPTSLEGITYLYPEAGQRALLPHANIAKYIAFRANDAIMGDIEMQESGQLYVVQYSDVALEEGGKVCVCGSLAYRAMQEALEVLSPGTVAERSRFTVLGPKSKGTEAALQAILGDGRFQLEERPHNEAYYSYQITDCERGTILTVKARPEVFSDNFFALKTKIKNNTATSEEKKAFQVLRAQVVEKVRWEEADNLFAIEASTAFAGGSGTQSDPYQIATAA*
>Syntrophomonas_wolfei_subsp.metylbutyratica_JCM14075T_01640_Demethylmenaquinone_methyltransferase
MNSSDISTYEKSLLLSQPLREPGLKNIIQYLPLPSSGIGLDIGCGTGFTTFMLAEALGPAGSVIGFDIEERFLQSARLLRQNKPQIRERVEFVQGNAGDLPFSGKSFDWAVSIDCVGVLDIDPVVMLKEIGRVLKPGGRVFIIMWSSQMLLPGYPLLEAQLNATAAGIAPFKTGMAPDRHIMRAPEWFRSAGFQDIQTHTCAMDVCSPLSAEIIVAVSDLFIMRWGSCEKEVAPEIWHDYQRLCQPDSTE
FILNVPGYYAFFTYSVFSARNS*
>Syntrophomonas_wolfei_subsp.metylbutyratica_JCM14075T_01641_Cation_efflux_system_protein_CusA
VSVVIVCYNTDAGSDEIDKANRELREKIGEIKDDLPDGCFEPEVNTKVAEVAGVLLSLSGGGYSYEELSAYAEEIEDALSEIDGIYKTKLVGKVEKQVTVEVDSDRLNQLGFSLSEVNQVLYAQNLEIPNGALENEAGKIYVKTEALYQSLDDMKNIIVGVSRQTGAMVRLKDIASVYMESKDDAYKCKQGDKNAVLVAAYFKDDRNIIPVGKKVRETLENLRESLPSDLDLSETAFQPDDVDKSTDNF
AGHLIAGIILARSPASILFSIHK*
>Syntrophomonas_wolfei_subsp.metylbutyratica_JCM14075T_01642_hypothetical_protein
LEANSFASYFLVPDDALRYYLNLRLQNSSRKPNMRDLIYLENYFAVSHALMLLRLINMGVISEAEAEQMKAGVISAAIRLGYSPALYRDTADDQGRVIYSNYAELAEELLEAGQITYGKYEELLLEGGYGEILFGDGEENADSDAGNL*
>Syntrophomonas_wolfei_subsp.metylbutyratica_JCM14075T_01643_mRNA_interferase_YafQ
MYIVKRTRKFDKDLKLLVKRGYNIDLLTEVVKKLANGDKLAEKYQDHALSGNWIKYRECHIIPDWLLIYRIDKDTIVLILTRTGTHSDLF*
>Syntrophomonas_wolfei_subsp.metylbutyratica_JCM14075T_01644_bifunctional_antitoxin/transcriptional_repressor_RelB
MAQTSITMRIDENLKKQAEELFEEFGMNMTTALTVFIKAAVRQGKIPFEISVDIPNAETIEAMKEADRIIHDDSVKGFYDVDEMMKEIIG*
>Syntrophomonas_wolfei_subsp.metylbutyratica_JCM14075T_01645_transposase
VVIDEIGFVPITRPEANLFFQLISSLYEQTALIITSNKGFEDWAELMGDPVIATAILDRIAHHSEIFNLTGDSYRLKHRDSILSP*
>Syntrophomonas_wolfei_subsp.metylbutyratica_JCM14075T_01646_YcfA-like_protein
VAGVDKIIEKMKRQPNGIRPEEAEKVLKANGYEYKRQKGSHRHYLHQNGDLITVKIETPLKAVYVKDILTRIGE*
>Syntrophomonas_wolfei_subsp.metylbutyratica_JCM14075T_01647_putative_protein_encoded_in_hypervariable_junctions_of_pilus_gene_clusters
MRTEEYVKLPYTRIIQEINDESGHYFYGRIMELDGCQSTGDTLEELYKNLNEALEGYIETKLENNLDISLPVNQEDYSGKFVVRLPKSLHMRLVLEAEKEGVSLNQYALYKLAR*
>Syntrophomonas_wolfei_subsp.metylbutyratica_JCM14075T_01648_Demethylrebeccamycin-D-glucose_O-methyltransferase
LKPPPVWRLRGALPHLLCSMAALCNLLSCISAPSWRTIIKRIALLLQPEDRVLEIAAGTGIIALGLADRMQNIDAIDFSPSMVAKAQKKAQHLGLANVHFSAQNACDLRFAPGSFDAVIICNTLHIMPEPEKALAESHRVLKPDGRLIAPTFVHAGSKKALLFFALMSLSGLRAYHKWTQQSFHAFLEENAFTVLDSVLLKASFPLAYVVARPKQ*
>Syntrophomonas_wolfei_subsp.metylbutyratica_JCM14075T_01650_RNA_polymerase_sigma-28_factor_precursor
LSFLLWALAITGILKGSLCLFAYLNNQVFPQPLSEEDERKCLQEMQRGGEEARNILIEHNLRLVAHVVKKYESSGEDLEDLISIGTIGLIKAIRTYNFERGIKLATYAARCIDNEVSMSAPGGKNWYTVKMIKACY*
>Syntrophomonas_wolfei_subsp.metylbutyratica_JCM14075T_01651_type_I_restriction_enzyme_EcoKI_subunit_R
MWLFWSLDESGIRRIYTWAQNTEFKCKLVVVVYPACPTGYLDLEELLQLRQNTEGKLDFRLYVTNSTRGEKEPYIAISVDMLDTGIDVPELVNIVFSQEPPSLPLPKIFMSD*
>Syntrophomonas_wolfei_subsp.metylbutyratica_JCM14075T_01652_hypothetical_protein
VDRVKSNFLAVILFIPVMRATLGNLYLTVFNTVNKAVTIINSSTPKPLEFISQRLWLADAIIAVAVNVL*
>Syntrophomonas_wolfei_subsp.metylbutyratica_JCM14075T_01653_addiction_module_antitoxin,_RelB/DinJ_family
MTQINVNIRMDENLKKQFEAFCSEIGMNMTTAFCVFAKTAVRQNRIPFEISAESDPFYSEANQARLRKAIADLNAGKGKAHEIIEVTDE*
>Syntrophomonas_wolfei_subsp.metylbutyratica_JCM14075T_01654_DNA_topoisomerase_3
MKSLVLAEKPSVAREIARVLQCSKKSKGYIEGPRYVVTWALGHLVTLAEPDDYDKRLKQWRMEDLPMLPEKMKLKVIKQTSHQFQVVKELMKRNDIEELVIATDAGREGELVARWIMEIVRWKKPFKRLWISSQTDNAIREGFARLQPGTQYNSLYAAACRAEADWLIGLNVTRSLSCKFNAQLNAGRVQTPTLAMIVKREKEIKDFVPRDYWTIRADFGDYYGDWRGKDGGRLFDYARAEEI
LSRIKGRMAAIKEVKTEARSEAPPLAYDLTELQREANRRLGWSAQKTLSVLQELYEVHKLVTYPRTDSRYITEDIVPTLPLRLKSMGVGPYADLARPLLQKTLKPGKRLVDNSKVSDHHAIIPTEQPLQLNSLNEEERRLYDLIARRFLAVLYPPYRYEQTTITVVEGENFHSRGKVEIDRGWRAVQAVPGDKDKDEEQLPEQTLARHKKGETLEVKSGKASKHKTKAPARYTEATLLTAMENCGK
LVEDKKLRESIKGSGLGTPATRAEIIEKLIRTSYIERQRKELVPTSKGIQLMDLAPPDLRSPELTAQWEQRLAEIARGQGKRPDFMADIRRDTVQLVQSIKSDSSTYRADNVSKSKCPQCGRFMLLVNGRKGKILACPERSCGYRQPEAEDFSGRGGNSRVNRRLMAKYSDQEELETNLGELLKAALAKKEQG*
>Syntrophomonas_wolfei_subsp.metylbutyratica_JCM14075T_01655_hypothetical_protein
VNTAKKDLLELVNRISEIEAKEVLDFALFLEMKRRKTEFKDLEMASQTSCDFWDNAIDDEVWNNA*
>Syntrophomonas_wolfei_subsp.metylbutyratica_JCM14075T_01656_PemK-like_protein
MPKQGEIVLIPIPFSNLQSIKKRPVLIISNDYYNELTGDIIVLAITSNLNEKKYSIEITDDNLDEGQLKIKSCIRCDKIYTLSQEIIVSKFGIVSHDIMEKVRSKISELIK*
>Syntrophomonas_wolfei_subsp.metylbutyratica_JCM14075T_01657_Chaperone_protein_ClpB_1
MERLTQKSRQALLAAQNLALEKRQQEVQGKHLMMALLEQEGGLVPRLLQQAGISIPAFTQQLGQRLDKIPSVYGYEGNVYLSGSLGRVLNRAEKEAQDLKDDYISVEHLLLALLEEGEPDLKDLLQKAGLNREGIFQVLHGIRGSQRVSSDNPEDSYEALERYSRDLTRLAREGKLDPVIGRDEEIRRTIEILSRRTKNNPVLIGEPGVGKTAIAEGLARRIVARDVPEGLKDKRLLALDMGSLVAGA
KYRGEFEERLKAVLKEVEDSAGQVILFIDELHTVVGAGAAEGAMDASNLLKPMLARGELRAIGATTISEYRKHIEKDAALERRFQPIMVNPPSVEDTISILRGLKERYEVHHGVRIQDSALVAAAVLSDRYISDRFLPDKAIDLMDEAAARLRTEIDSMPAELDEITRRIMQLEIEAAALGKEKDEASRERLEDLLKELQDLKSEAEVMQAQWQAEKEAISRVGQLKRDIEDCRYEMEKAEREYDLNRIAEL
KYGRLNELERKLQAEEELLAGKEKHSALIKEEVDEEDIARVVSRWTGIQVSRLMEGEKEKLMNLEEILHQRVVGQEEAVNAVADAVIRSRSGLKDPNRPAGSFIFMGPTGVGKTELSKALAEALFDDEKNIIRLDMSEYMEKHSVARLIGAPPGYVGYEEGGQLTEAIRRKPYSVILFDEIEKAHSDVFNILLQILDDGRLTDGKGRVVDFRNSIVIMTSNIGSQEILNFQQAGGDYEDMKNRVLGLLRL
NFRPEFLNRLDEIIVFHALGVEQIKEIAEILLQKLALRFRQNTRAGLEWDEKALDYLAEKGYDPAYGARPLKRLIQHEIETALSRAMVKGEIRNEGEVIISAEGGKIALKIG*
>Syntrophomonas_wolfei_subsp.metylbutyratica_JCM14075T_01658_hypothetical_protein
MRLVKVYYHSKEEKIPLSWLNLHPDLLKIMEEMGIIELAGDSIKADGLQKIYKMMRLKNLLGVNFNGAAIIVDLLERIEELEDEIEELKRKR*
>Syntrophomonas_wolfei_subsp.metylbutyratica_JCM14075T_01659_Heat_shock_protein_J
MAVNYLDYYKILGVERDADAKKIKAAYRKLARKWHPDLHSGKKREEAEEKIKEINEAYEVLSDPEKKALYDRLGPNWKNGERVDSSGFGDGVFYRSSNIDPEDLQGFSEFFSSLFGAEPGRQSRRASYRSAPVRGQDIESTIELSLEEAYHGLSKSITLSTAAACPECQGSGLAGRGFCRRCGGTGSVSEARTLQVKIPPGVHEGSSIRLKGQGGEGLGGAERGDLYLQVRILPHPVFTLRGRDLETEITL
RPEQAVLGDRVTVPTLDRKLTLKVPAGSRSGQKLRLKGKGMPARQGENGDLYARIKIDLPHDLSEEEKELYRQLAELRAPRQAERGVAE*
>Syntrophomonas_wolfei_subsp.metylbutyratica_JCM14075T_01660_Heat_shock_protein_70-2
MAKAVGIDLGTTNSVVAIIEAGDPVVIVNAEGNRITPSVVAFKDNNERLVGQIARRQGALNPENTFYSVKRFMGRRYSEVESERKLLPYTVVSGPNDAVRFKSRDKLLAPEEISAMVLKKLVEDASKYLGEKVKDAVITVPAYFNDAQRQATKDAGRIAGLNVLRIINEPTAAALAYGLDKKANQTILVFDLGGGTFDVSILEVGDGVFEVKSTNGDTHLGGDNFDKAIVDWLAEEFKKDYGIDLRQ
DKQALQRLVETAEKAKIELSSVLETSISLPFITADASGAKHLDMKLSRAKFEDLIHEDLERLRGPVKAALDDARLSARDIDEVILVGGSTRVPAVQKLVRELNGKEPNLGVNPDEVVAVGAAIQAGVLAGEVRDVLLLDVTPLSLGVETMGGIMTRIIERNTTIPARRSEIFSTAEDKQPAVDIHVLQGEREMARDNRSLGQFKLEGISPAPRGVPQIEVSFDIDANGILTVSARDQATGKEQSVTISGSSN
LDKAEVDRMVKDAQTYSEEDKKRRQEVEVMNQGDSLCYTVERKLKAVGEQLPLHEKARIEELLQELKKALEEKAGLDRLKELIDELGQAAYSLDNIEPAAAGPDRSGPAAEGNAEVVDAEFEEN*
>Syntrophomonas_wolfei_subsp.metylbutyratica_JCM14075T_01661_Sporulation-specific_penicillin-binding_protein
LPLRRLYFLMSFILLLYMILALRIAYFQVIKGQELAAQATAMRSQGIRLSEYSRGEILDRNFQPLTGSYTSTALYGLPAEMRSSRSVSNEIYGRDTLQMTAEILGRILNKQDSRKIQEQFRQAAQSGAAFVRIASDLSEAEIQRVLSRNLAGIAVAPLIKRYRQDGFCCHVLGYTGGYHNNEGQAGIEKRYDALLKKGNSPQELLSVYDARGAVIKGLNFKFYEEEDRARGSVVLTIDKRIQEIVEKEISAKVKS
GAVVVMDVDSREVLAMASRPSFNPYHIEYLLQHDQNSSLINRALSRYHPGSLFKILVATAALEENLVSERDKFHCSGKYFFNETQSTSCWKEEGHGELSFAQAFACSCNPSFIEVGQKLGREKLLTYVDKLHLCDETLLGYAYHPGSYVRINPGKIALSNACLGQEGVMLSPLQITSLLATVADDGLWQAPSLLRYTINSKGERSRIAASRKERVISPETAGKVQELMELVVSEGTGKNAALAEIKVAGK
TASSETGNWKEDGEQSLDTWFGGYFPADNPRWVVLVLVQEGESGAVDAAPVFKNIAREMLKIF*
>Syntrophomonas_wolfei_subsp.metylbutyratica_JCM14075T_01662_Antitoxin_HicB
LDKYLFMAVFTPAPEGGYEVRFPDLPGCFSQGDSLEEAMTMAKDALELHLWGMENDNDPIPPATLPENIELKKGCFTVPVSVYMPPVREEMDNKTIKKTLTIPAWLNRVSEEADINFSQTLQKALKQQLGIQD*
>Syntrophomonas_wolfei_subsp.metylbutyratica_JCM14075T_01663_YcfA-like_protein
MKPKELLKLLYTDGWVKKAQRGSHIQLIHPTKTNKITVPYHNKDIKPGILHDIIKQAGLK*
>Syntrophomonas_wolfei_subsp.metylbutyratica_JCM14075T_01664_putative_protease_YhbU_precursor
MKEPELVLPAGTLEKLKTAVLFGADAVYFGAKEFGLRAYAGNLSRAEIEEGLAFAHSRKKKAYLAVNILAHNRDMHKLPAYLEEMASLQPDGFIISDLGVLRLAQKYAPSIPLTISTQANVSNYEAVCFFQDLGARRIVLARELNLGEIEEIKKKSSVEIEIFVHGAMCVSYSGRCLLSCYMTGRSANQGACAHPCRYRYALVEEKRPGEYFGIEEDERGSYILNSRDLCLLEYIPRLLEAGVDAFKVEGRMK
SPLYLASVASVYRQAIDRYRERATPFTREELSAWMAELHKTATRPFTSAFIGGEEDRAQDIDKEEMPLRAEFCAIVTGYREQQGLIELQQRANFGCGDYLEFLLPGGELLGFVLEKLYDEDLAPLDRARHPQQKVLIPFDHALPEFSILRKGGKINQG*
>Syntrophomonas_wolfei_subsp.metylbutyratica_JCM14075T_01665_putative_aminodeoxychorismate_lyase
MSEIKLRLEKPKVVAIILAVLFIILAGVFFTGQMIARQYQPVDPADKSMVDIVIPEKTGARQVADLLYNERLIHKQSVFLAYCQKNGYDSSLKAGHYRFSRSQSLKEIVDDIVQGRIVNISFTIPEGYTLEQIGQLLIKKQLCSQEEWNMSIKQDYDFAFLKALPLENENRLEGFLYPETYFVPEDYNCRQAIELMLNNFEQVWQKKFAEQAQAKNWSVYHTITLASLIEKEAQVPEERAMIAGVILNRLNA
GMLLQIDASVLYALQRHKELVTYADLEVNSPYNTYKYAGLPPGPIACPGEASINAALNPKEHSYYYYVSKGDGSHFFSRTYSEHLRAQKKYGQ*
>Syntrophomonas_wolfei_subsp.metylbutyratica_JCM14075T_01666_Vancomycin_B-type_resistance_protein_VanW
LANERVKTSIFFSLILLLGIALAGAMYFFQVFYKSPYFMPGVKIASTTVGGYTSKQAASRLERDLEEYKKTPVIFHYQGYSFESKLEDLSYPVNYEETVNKVWQQEQERDWPSKLTNLDGSREVIYPLKLEYKPEIKARMIDEWNKVLGAAAEDARLEIEKQKGLVLIPARKGLKVDPEQTFADLPQEWGQMEEIKIPIAMQEEWPQVKDEDLSNMGELSSFSTWFNVNEINRSHNLYLASSAINTSMLP
PGEIFSFNRRVGERVIEAGYRDAMVIVNGKFEPGLGGGVCQVSSTLYNTVLLAGLEIVERHNHALAVAYVPVPGRDATVVYGLQDFRFKNNSPYPIYIRAWAQAGKLTMTIYGNLTEKRDIHLSTIVDRVIPFKEIRENDPSLNPGQEKLDHAGIQGYVVRSFRNYMDEEGRLLKSEQLASDYYKPLDRLIYVGPGVKVESPPENVDNPENPPNGIEPDPLGDSTGEAAPLVEPGPSEIPSPQPENGDEG
TPPITI*
>Syntrophomonas_wolfei_subsp.metylbutyratica_JCM14075T_01667_hypothetical_protein
MTEEFLEEDFPILVLVDEDGIEHLFELLAELEIENDKYRVLVPLDEEDEEYDEEDGEVVILKVIIDEEGNELLSDIDDETEWEMVVEAWQELVDSEDL*
>Syntrophomonas_wolfei_subsp.metylbutyratica_JCM14075T_01668_Putative_Holliday_junction_resolvase
MRIMGLDLGEKRIGIAFSDPMGWTAQGHSILQRKGLKKDISYLQELCQEFQVEKIVLGLPLNMNGTMGPKALESQEFARALQEVLKIPVDLWDERLSSKSAERVLLEADLSRKRRKELIDKIAAVHILQAYLDGGCSGKDY*
>Syntrophomonas_wolfei_subsp.metylbutyratica_JCM14075T_01669_hypothetical_protein
MPQLPGETVSFKLERDDENKVRAILHTVYQALQEKGYNPINQLVGYMISGEPAYITSHSEARNLICKVDRDEIMEVLLKSYLQNSREKGE*
>Syntrophomonas_wolfei_subsp.metylbutyratica_JCM14075T_01670_Alanine--tRNA_ligase
VWSSAEIRKTFLEFFKERGHSVVESSSLVPVNDPTLLFTNAGMNQFKDVFLGLDKRKYVRATSSQKCVRAGGKHNDLDTVGRTPRHHTFFEMLGNFSFGDYFKREAIGYAWEFLTEVVGLPEEKLWVTIYRDDDEAADLWPEISGIDPARIVRLGEKDNFWSMGDTGPCGPCSEILYDRGIEYSCGAPDCGIGVCDCDRWLEIWNLVFMQYNRDESGEMTPLPRPSIDTGMGLERLSSILQGVDS
NFDTDLFIPIIKRIEELTGKAYEQGERGFPFRVIADHSRACSFLIADGVLPSNDGRGYVLRRILRRALRFGRLLGIEGSFLYKNVDVVCDIMKEAYPELLEKQDFIKEVIRLEEERFLLTLNDGLKKAEEIMERARQRGDNVIPGEEAFMLYDTYGFPLDLTEDMAEENQFTLDKAGFDRSMEEQRQRARQANKGEDLLGQERLLSEKLAGIAPSSFTGYENSRDESVLLAIIKGSELMDKALNAEEVILITSR
TPFYAESGGQVADRGIIKGQEGLLRVQDVKKLSAWILHYGIVEGVLTTGEGVSMQLDDRARMDTARNHTATHLLHRALREVLGEHAQQKGSLVEPARLRFDFSHLKALSNEELSRIEQMVNEAIWKLYPVTTTETALNQAREMGAMALFGEKYGEEVRVVQVDTYSSELCGGTHVQNTGQIGLFKITGEGSIGSGLRRIEAITGSYALEYLQQLEGEIKKAASALRSSPLELTKRIENLHNSLKEKE
KEIESLQQRLSRSSSDELVNKAFQLNDAWILIEEADIEDPGSLRQNAEMLKDKLGRAIVMLASIKGEKVSFVCFVSKDLLEQGLHAGKIVAAAAQVAGGGGGGRPDMAQAGGRDKSKISEALAEVRKMVKKTLS*
>Syntrophomonas_wolfei_subsp.metylbutyratica_JCM14075T_01671_Transport_of_quorum-sensing_signal_protein
MLPANSKIYRYFFFIVVVLATIYFLFLVREVLLTFFLGAVLAYLLFRPISFLERKGLKRVWAILLLYLLLLAVFSLFFSLAVPAMVVELNQMAGILPDYADQARDMAKSIDGMELGKLTPVLKENLNQIENKLFKGLKDFLGGFYSFLGKFLALAFSPILAFYIINDWEKIRDAFLGLFSPSFRREIKAVLEKIDEALIEFLKGHLIIATFVGIFTGIAAALLGVKFPLLIGLVAGVADLVPYFGAFLGGFFALLIALSES
LQLALYMGLAVLLIQQVESNIITPRIMGGKLGMHPLLIVFALLAGGKLMGIWGMLLAVPLAAALKVVIQWAFLKMVES*
>Syntrophomonas_wolfei_subsp.metylbutyratica_JCM14075T_01672_hypothetical_protein
MIIKCPVCGGLQVGRVGSDQYYCWNCFLEFNFRKGMLNLYEVAEDGTLVAMENSSQLL*
>Syntrophomonas_wolfei_subsp.metylbutyratica_JCM14075T_01673_PRC-barrel_domain_protein
LKTRNIQHLPVFLATRAEVVAMVQKAVIGDDFGLLYLVVKGEDGKAGLIFRDDFDLTPDAVKIWDLHCIKSYAYGEELSIYEKKLGDTVFDRQGKELGIVSDFILSPAEKQVQGLEISSGTINDILKGRMELSLQEVSWKSVKGGVLAGEGSQEE*
>Syntrophomonas_wolfei_subsp.metylbutyratica_JCM14075T_01674_hypothetical_protein
MWIDRLTQAVVLLLLVFATQGNNPYDHQAKTKKSCPGNHRHHLPSRESDQPPNGNSYTNFSITFIIQAEKNGSADSFIRFTRRNRQGNIGGRVKPFFFILLV*
>Syntrophomonas_wolfei_subsp.metylbutyratica_JCM14075T_01675_tRNA-specific_2-thiouridylase_MnmA
MLNCLKSMEKGIIVEMIQGDGRVKVAILMSGGIDSTVSALLLREQGYEVTGLTMVHLEREAGEKAARIANELKIEHKVVDLREAFQSAVADYFCTEYEQGHTPNPCVKCNEEIKFGLLLKAALDLGADKVASGHYARIDYDDKSQCYRLMKGLDPRKDQSYFLYRLKQQQLSRLIFPLGGYRKEEVREIARCYNLQLAEEKESQEVCFIPGDYREFIRPQVSYKEGSFLDRTGKVLGRHRGIPFYTIGQ
RRGLAVNAGRPLYVLKIDPEKNQILLGENEELFSKLLRFKQNHFICPRDFELPIRVKAKIRYAAREAEAILHYIEDDIWELVFEEAQRAATPGQSVVYYQGDYVLGGGTIMA*
>Syntrophomonas_wolfei_subsp.metylbutyratica_JCM14075T_01676_Replication-associated_recombination_protein_A
MDLFSLANEEKFNAHAPLAWRMRPRSLDEVVGQEHIIGPDSPLRRAIENDRLQSFVLYGPPGSGKTTIAHIIARTTSSHYAAIKAVSSGVGEIRKIAADAAERLRYYQQSSIIFVDEIHRFNKSQQDVLLPYVEDGTLILIGATTENPLYEMNNALLSRMKLYVMEALNTDSLRRIVEKALADPERGLGKHNISIDEQSMAMIVQAAQGDARTALNILDTLHNSYSSKNGSLRITPELLEKVTGRLILKYDRS
GDYHYDTISAFIKSIRGSDPDAALFWLAVMLEGGEDPKFIARRLIVHAAEDIGLADPYALTLAASAARALEFVGMPEARIPLAEATIYLAAAPKSNSSKMAIDKAINTVQNTPRIIVPPHIADSSHSQSASLGKGKGYKYPHDYGGYVKQEYLPQEIKGITLYEPGKNGYEQNIRDFLQKIKNSHPHK*
>Syntrophomonas_wolfei_subsp.metylbutyratica_JCM14075T_01677_Thioredoxin-M
MADIKVFNQDNWDKEVMQSDLPVLVDFWAPWCGPCKMVGPVVESLAAENAGKINIGKVNVDENQDLAARFGIRGIPTLAFFRGGSEVKRIVGAQNKAQLQKAIDEVSGS*
>Syntrophomonas_wolfei_subsp.metylbutyratica_JCM14075T_01678_Copper-sensitive_operon_repressor
MTKNNEKTRKNILVRLRRIEGQVRGIQRMMEEEADCMDILTQISAVKSAINQVGIIVFEKHAQECIVKAMDEEKQEENLKEIVNMMGRLIK*
>Syntrophomonas_wolfei_subsp.metylbutyratica_JCM14075T_01679_hypothetical_protein
MEELGQQVKMELLRTLIWVLLSLAVGMSIFYFVL*
>Syntrophomonas_wolfei_subsp.metylbutyratica_JCM14075T_01680_putative_vancomycin_resistance_protein
MTDAGKLWLKVISMAVIAISASLLIVSFALAARDQDYYPSNLWLGGISLADMSKEEAYALLQSELASSRGDKLILVVGKQEISLPMEEIGINYDIAASLDKIEEELFPGQGVATLVKHSVARGQRQEIKPVLSWNKEAVLEKVLQIKRDYDQPAVDARIVYNHDFLEYRAHQNGYLINVNASMNHISDALEKGILGPMEIIATEVHPRVKIEDIETVKDVIAVSARRINHLSPETSALLEKLNGLIIMHGEKLD
LPAIMDSGCEAAMDVGSETKAQIKGAIVQACLQAGMQEKDKQSVMENKLQYPLLLTTSMSEHTLLVKIYGCQTELGKEITLLSEREDVYPEVQIKLNHRLSPQQRIIQQEGKKGFILRNYRVVTSGGKVVEKSLLSEELTPASDTIIIVGPGNIRK*
>Syntrophomonas_wolfei_subsp.metylbutyratica_JCM14075T_01681_PRC-barrel_domain_protein
MKKTQEIIGLPVFSVLDGKKIGQVKDLVINPEEGKVDFILVSNRNWYDGARVLGYKSVMGIGEHAVTTESENLLTTINETANANKLLERHIELKGNRVLTNRGNLIGVISEYIVDEDTGAIACLEFRTAEDESKIEIIDAKQVMTYGVDVIVIKQEESPEVSLQQKAVEAEKLVSPVAVTSVTSPEPTSLVAPASSPPAGSSDGAAFFKQKQRQFLLGKRLIQDIKDAIGNVLIAQETVITEEILDLAERNNRFI
ELTQCAR*
>Syntrophomonas_wolfei_subsp.metylbutyratica_JCM14075T_01682_hypothetical_protein
MAYFLRREGLGKRNMAIILSLSLLIIITFPITVVRLGIFCSFLSYILLIVVITRYLLGNEGDFVPQSKGRLVTGPELEKFMETIETRLDSDLSGLRVEAFDNLEGDSIPAGLIEEVPEAVPILESTVTTEETREKELFAAELLEQDSGELAVKPAVSEEESEAETLQLAIDESRSPDAEIEVETTALEEKALDDAGSIEEEATEATLEEIDIMEELELEDTGREGIDLPAEELEEKNEEVEESLEAEEGAEEKEEKTEEEIEEI
AEGIAVKLEPAEYDNDLSDDGAEKIIEKLDEDINIEYVENLAGKTIEELPEEGEAILDEEVAGVLEAAAEAELLEEQDLPAAVHDKESIKIEQADTFKEEDTFILDNINTRIFDLIEQGFLHKEAANLPEAATCFEEAWDITMDDELKRNLSIELLNIYQFIDENQL*
>Syntrophomonas_wolfei_subsp.metylbutyratica_JCM14075T_01683_hypothetical_protein
MIDNKGFAGMNLIVDDSYLQELEKLDFPVVKRQLLSMEDFNEEIRQGLIASKGKNDLAAIYLEELELMEFPIIKTHRLSLEEFNEELRKGIVEDGNAIQAHFAAMGIKVKNLALVS*
>Syntrophomonas_wolfei_subsp.metylbutyratica_JCM14075T_01684_Arginine_utilization_regulatory_protein_RocR
MAVSDLTVNEITSLLEGLIENPYMFYVIVDSDGLISSINQTYLDILEMKKEDVVGQHILDITPNSGLPEVLRTGRMDKADIWSIKGRDTIVTRVPIFKNGKIIGAIASSLFLDMSGAKILMKKLQETEKEFSAILEGLIESPHMAYVIVDKEGYITVMNQTFLDILEMKKEEVIGKYVLEILPHSKMIEILKTGRVDNADIWPIRGRDTIVTRTPIKKHGEIIGAIGHSLFLDMSGAKILAKKLQETEKELSLYRDEV
SQIYSARWTFDDLVGCSQEFLVVKSMARQLSYSISTILITGESGTGKELFAQAIHNGSERKNWPFVRINCAALPENLLESELFGYEEGAFTGARKGGKPGKFELAKGGTIFLDEIGDMPLTMQTKLLTVLQEKVVERVGGTTPIAINVRVIAATNRDLEEMVEKQEFREDLYYRLNVVGLNIPPLRRRMDDIPLLVNDLIHRINQCLKTDISGIEPEAIDLLREYSWPGNVRELENLLERAINLAVMRREQ
VIRAEHFPSLDQKNKDKKFAKIEILDDGQSSLPQIIEKIEKQMIIQYLQESGGNKQQTAKMLGIHSSALYRKLGKYGLE*
>Syntrophomonas_wolfei_subsp.metylbutyratica_JCM14075T_01685_hypothetical_protein
LKIAVIGDTHGRIEKICRELKLVKADQIFFTGDFLSDAKRIAHHLGGVMLHAVAGNCDFYESGPAERILDLEEKRFYMVHGHQYGVKGSVNSLYYRGLELGADVVLFGHTHIPFCEQIEGIWLINPGSPSRPRLDKKGSYALLYLEKGKIEVAIEYI*
>Syntrophomonas_wolfei_subsp.metylbutyratica_JCM14075T_01686_Non-canonical_purine_NTP_pyrophosphatase
MPRELLLATRNRKKKLELQEILRELDLNIITLEELPSLAEVEEDGNSFAENAIKKAVFTARASGKVCLADDSGLVVDALGGQPGIYSARFAGESAGDEENNQKLLKLMEMIEEDQRTARFVCVIALSDAQGNVQTVEGRCEGKIALVPAGKGGFGYDPLFIPRGYTQSFALLPSATKNLISHRGKALEQARPLIERLFQGST*
>Syntrophomonas_wolfei_subsp.metylbutyratica_JCM14075T_01687_Ribonuclease_PH
MERPQNRANDQMRKVRIIPGFQKYPDGSVLIEAGDTRVMCSAMMEEKVPPFLRGKGSGWVTAEYSLLPSSTETRTQREASKGKISGRTSEIQRLIGRSLRAVVDMQAMGERTLWIDCDVLQADGGTRTAAITGSFVALYLAFKKFKEQGIIETIPVKDFVAAISVGIVDAMPVLDLEYIEDSQADVDMNVVMTGNGDFIEIQGTAEGTVFSRAELDQLLELAGKGIQELIALQKSILGV*
>Syntrophomonas_wolfei_subsp.metylbutyratica_JCM14075T_01688_2-hydroxyglutaryl-CoA_dehydratase_component_A
MSITGIDLGSRTIKIIEMDGQEIINHSLYDTVSFYRNCGRIADGEMTVDLAGLGFSRERIIATGYGKISVRIAGAEQIPEIQAHVYGALWQSGLQDFTLLDIGGQDTKVVQVRQGQMLDFLTNDRCAASSGRYLENMAAVLGMSLEELSSFSEEAVELNATCAIFGETELIARIVEGHPVSHLAAGVNYSLYRRVSAMLNRLRSKKIILAGGGALNSALAKIIARETGSAIYQLPRPQYNGAIGCCAWAWRH
ETA*
>Syntrophomonas_wolfei_subsp.metylbutyratica_JCM14075T_01689_2-hydroxyglutaryl-CoA_dehydratase,_D-component
MSRVGFTTSIPVEVILAAGKTPVDLNNVFINSSSPQSKVDLAEEEGYPRNCCGWIKGLYATGLDYDMEALIAVMHGDCSNTQALMETLQLRNKKIIPFAFPFDRERAQLEAQVDKLMQFFEVDMEEVLKARARLEKIRSLCREIDRMTWRDNLVTGRENHYYLLCSSDFNGNPDLFEAELQSFVEEAGKRRPIQEEVRLAYIGVPPIFTDLHQLLEHMGARVVFNEVQRQFSMPVESQDIIEQYLHY
TYPYGAFARLEDIEEQLAIRDVQGVIHYTQTFCFRQIEDLIFRERLKIPFLTMEGDRPGKVDARSRMRLESFVSMLKK*
>Syntrophomonas_wolfei_subsp.metylbutyratica_JCM14075T_01690_Glutamate_racemase_1
LGKNKPIGIFDSGIGGLTVVKSLMEKMPDESFIYFGDTAHVPYGNKSETQLFNYAHKIISFLISKQVKAIIVACGTHSSVTLPSVSQEYSLPMLGVVKAGARSAARLSCNGKIGVAATQATVNKKAYTREIQELEPEFQVFETACPRFVPLVEAGKLNNSESREAVAEYLGPLLAQGIDTLVLGCTHYPFLATAISEFAGEKLKLVDTSCETIDELQEMFGDRDLFNNSGKRPRHEFYVSGQDDSFFNVGRL
LMGDIIKEIHKLDWD*
>Syntrophomonas_wolfei_subsp.metylbutyratica_JCM14075T_01691_Spore_germination_protein_GerM
MTSKVISILAVILLVLCLLSGGCSSIAPEQNLKAWKDLIKLAPEDNTKKEVKKNPEELIPAREKSESISIKLYFAQADKEELVMERREITRTEGIARSTMQELLKGSDNPAYRNVFPEGTRLLDINLKPDGTCILDFSSELRRLENGTEEKQMIDAVCQTLAQFPAVKQLVFMIDGREIDIIKGEVDFSSPIKVSQ*
>Syntrophomonas_wolfei_subsp.metylbutyratica_JCM14075T_01692_Oxygen-independent_coproporphyrinogen-III_oxidase_1

LRYEGMVYRPPSEAQSLIIQATIGCPHNRCTFCSLYKNTKFRIRPVKEIKEDLQMARDYYGEYIESVFFADGNTIIMKTEQLLEIFDYTHNLFPHLKRITVYGSSRFVNKKKLEDLKRLRAAGLKRIHTGMESGDDIILERIKKGTNSQEIINAGLKLKEAGISVSEYYLVGIGGRERSREHALNSALTLSAFSPDFIRLRTYVPMPNTPLYDDYQNGVFALLSPHEALREVRLLIENLNCDNSQLLSDHINNYW
NVYGVLPQDKNKMLAELDRALAIDESRFRRPERGRL*
>Syntrophomonas_wolfei_subsp.metylbutyratica_JCM14075T_01693_DegV_domain-containing_protein
VGIKIITDTSCDLPQELLEQYNIEMIPLKVTFANGETYLDRFEISPRTFAEKMSSSATLPKTSAPDPLTFVKYFEQGIREKGAVLFVSLSSGLSSTSQIAQMACNMMNSSKVKVFDSLTASLGTGIMAIRAAQMAARGMSLEAVLDKLAAIRAAREVIFVLDTLENVVKGGRLSKFEGMAGTILNIKPILRGNQLGVPEIVEKVRGRKKAMKRLLAMVGDLAGASLLQRIVGISHVNCLEDADKLAWEINSQ
YQQEAVLISDMSATIGTYAGEGGLMINF*
>Syntrophomonas_wolfei_subsp.metylbutyratica_JCM14075T_01694_hypothetical_protein
LDLKEIVIRKNGKWYFGQAEMFRRNILNILAAHIQKEDGAYVIRLGNDVNPITVEDCPFLAMGYIEEEDGTIKLRFHDLQEMKLDHELKITFKGDVPYIDFKWEADTRLSRGVYWKLSNYFDFRGEEVYIVPPGAGLEQ*
>Syntrophomonas_wolfei_subsp.metylbutyratica_JCM14075T_01695_Putrescine_aminotransferase
MNQRKDLLNPTLYKIFQSFQLDKNYLSGDGSYLVDEKGIRYLDFIAQFGAIPFGYNPDFIWAKLEEVRKKALPSLVQPSLPGEALKLANTLAAVSPGKLAYCTFCQSGTEAVEAAIKLARSTTGREIILSTYNSFHGKSLGSLSATGKVSYQSPFRAPAPGFIYIPYDDAAALQAVLDEQSDRIAAFIVEPVQGEGGIIVPRPGYLKAAEQLCRQYGVLFIVDEIQTGLGRSGALFACEHEKVEPDIMLLAKAL
GGGIFPLGVCLSSEEVWNDDFGFLHSSTFANNNVSCAIGLAVLDKLLEDDRRLIKEVAAKGDYLLEKLQELAAKYPEVIKEVRGKGLMLALEFQELDDCGSYDMAFMVDQGAFTIILAGFLLNVFKIRLAPYLNNSMTLRLEPSLNIGREEMDYVLAALDKLCQILKYRDYALLYGYLLGDYSPPARITDYRAVSRTVKASPLSPEEKISHKFAFIIHYPAPEDVIANNPSFKVFERPDLYRFLEWQSSFRG
AGICCHMPALRSRTGAVVEGWLIGVPFGAREMMNLPREETLGVIMKAVELGRELGAEIVGLGALSSVVSRGGRAVTGQGAAITSGNSFTTLMAMEALLAGARKMRINFSSARGAVLGATGSIGRACALLLSQDITNIALLGNPRHPRSSRNRLNSLTLEMLALAYQRRCQGILSGLGEWLDSLINICRKKDELRSLELEKALRRADGSSLELIKGVCRDLAVEFPLEISMDIDASLPQCDMIVAASNSPEF
VVYPRHLQPGAVICDVARPADVAPEVRERDDVLVLEGGLVSYPEPVAFGPNLGYRDGVNLGCLSETILLALEGDCRDFSIGSRLSLDTIEYLRRLGEKHGFALAGLMTGNREIGDKEIEEIYRKSLSFKQANNL*
>Syntrophomonas_wolfei_subsp.metylbutyratica_JCM14075T_01696_hydroxyacylglutathione_hydrolase
MIEEVLPGIFCLQVPLPRNPLRALNAYLIKGQDRHLLIDTGFDWPECKKALLTGLNSLGVTLSQVDFFITHVHGDHSGLVCDLLTGDSKVYASETDAQILRKTTTREYWQEIDAFFEMNGFPRNKSKNQGSRIKGYISGSEMDITYIREGQMLEIGPYRLRCIMTPGHTPGHVCLYEPENKFFISGDHILGDISPNITAWPEMEDALGSYLDSLEKVEKMDITLILPGHRSLIYHYRQRIAELRKHHEAR
LQEVRDILKKGAMSAYQVASFMSWDLDYESWEDFPMFQRWFATGEAVAHLEHLLHLKQVQVNKGEVLLFSI*
>Syntrophomonas_wolfei_subsp.metylbutyratica_JCM14075T_01697_hypothetical_protein
LAENEVESREKYELGSFEFLKTGWWVWHIVAIVGIFYLGYLFGGSIF*
>Syntrophomonas_wolfei_subsp.metylbutyratica_JCM14075T_01698_Transcriptional_regulatory_protein_ZraR
VNIGSVLEGLFTQNQFMAAIIVDSNGKVLSVSETYLQVLNLTKEEVVGKPIKEVTPHTRVFTILATGKAMVGYNWKVNGHNMIASTIPIIKDGETVGAFAYSVFPDIWDAKNLVENLLSELNMYRDEVDSLYTAKYTFDDIVSKDKGMEMLKSFARQIANHPFTTVLITGESGTGKDLFANAIHNASPRSRFPFLRINCAAIPENLLEAELFGYEEGAYTGAKKGGKPGKFELAHGGTIFLDEIGEMSLS
MQSKLLIVLQEQEIERLGGSFPIEINVRVIAATNRSLEQLIQEKKFREDLYYRLNVVRLEVPPLRQRKQDIPVLIRHFIDKLNPRLRTMIANISPKSLKLLEQYHWPGNVREMENMLERAIIMADMENGRSLDCRHFKFLQNRCSSEAPLELQSLKAASQKFEEELIARALEISNFNLAKAAEQLEIDLSTLYKKIKKYGIET*
>Syntrophomonas_wolfei_subsp.metylbutyratica_JCM14075T_01699_Succinyl-CoA:coenzyme_A_transferase
MNYANEYRKKLVSADEAVKVVNSGDWIDYGSMCGQVVMLDEALAGRKEELKDVKIWTLLTLRRPRVVDVDPEEESFVWHSWHLSAMDRKIAGERAVYYSPIRYSELPRYVREHIEPLAVAMLQVAPMDKHGYFNFGPQNSHSRAVCERARIVIVEVNNRLPRCLGGYEESIHISEVDYIVEGNNPPLPQLPDAASSEVDKKAAEFIIEEMKDECCVQLGIGGMPNAVGKMIAESDLKDLGCHTEM
LVDAYVHMYEAGRLTGRKKNIDPGKMVYGFAVGSQRLYDFIDNNPLCASYPVNYTNRPSIAALNDNLITINNAIEIDLYGQVCAESVGTRIISGTGGQLDFVLAAYESRGGKSFICLPSCSEREGKLESKIVPILKPGAIVTDPRSVAHYIVTEYGKFAMKGMSVWQRAEGLINLAHPQLRDELIQAAQAQGIWRRSNKIS*
>Syntrophomonas_wolfei_subsp.metylbutyratica_JCM14075T_01700_Propionyl-CoA:succinate_CoA_transferase
MFESEYKAKFRTPEEAVKVVKSGDWVDYMYFNGYPKALDRALAKRKDELYDVHIRSGISLPPFEPVEPQSDPTMKHFQWDSWHYSAFDAKLADQGICSFSPLLYHEVPGYYRRREVEMDVAMMRVCPMDKYGYFNFGINASHCEAVLENAKIAIVEENENMPYVYGGNGNQIHISEIDFVVRGENELMANPVPLEPTEAEMKIAEYILEDIHDGCCIQLGIGGLPNYLGKKIAEAGLRDLGVHTEM
MADAYYEMWKNGCISGAKKELDRKKMVCSFALGSQELYEFMDHNPMIATYSVDYTNNPYVIAQLSNVISINGCLEIDFYGQVASEMQGPRQITGTGGQWDFVLGAYHSPGGKSFLCTSSTYKDKSGQLQSKIKPFLTPGAACTVSRQMTHYVVTEYGKALLKCQSIWKRTERLIEIAHPQFWDELIKEAEKLGFWKRSNKIEP*
>Syntrophomonas_wolfei_subsp.metylbutyratica_JCM14075T_01701_Acyl-CoA_dehydrogenase
MDFRLSEEQEMIRKMARDFADNEILPYCQEWDEHHTFPWDTVKKLQELGLMTCGVPAEYGGPGIDHLGQCIISEEICRGDAGLGTTLAASTLLGPDPVYVAASDEQKKWFYGRMMEGALTAFCLTEPGAGSDAGGISTRCVKDGDYYILNGTKQFISNGGVANLYTVFATLDKKMGYKGMCAFMVDRDSPGISTSPPENKLGIRSSNTTQVIFEDVRVPAQNLLGKEGDGFKIAMKTLDISRA
VVGAMATGVAQGAFECAVKYAQERQQFGKPIASFQGIQFIIAEMAVLCETARLLYQKAASNQDSGLPYTKMASLAKYWGGEAAMKVSTDAVQVYGGYGYTKDYLAEKYFRDAKIFQIFEGTAQIQKIVIAGDVFRN*
>Syntrophomonas_wolfei_subsp.metylbutyratica_JCM14075T_01702_Acetyl-CoA_acetyltransferase
MELNEVVMVSACRTAIGRFMGSLKDVSSRELSLTVGREAIARAGITADIIDEICVGQVFPHGCGSLPARQVSIRLGLPARSGAVNVNQNCTSAMRALEIACHNIMLGKTEIALVVGAESMTNCPYLVPKGRMGYRLGPGSLEDVMLYDGLIDEQVPGHMGLTAENVAEKYGITREECDQLALLSHQRATRAVKDGTFKREIVPVEIKSRKGSKFYETDEHMIPDASIETMAKLPPAFKKGGVVTAAN
ASGINDGAAAAIVMSRAKAKELGLKPLMKLVNICGEGVEPPVMGLGPAVAIPKCLKQAGIRFEDVDYWEINEAFAAQFLGVGRMLKEESGMELDMNKCNLNGSGIALGHPVGCTGLRIIVSMYYELEKQGKTLGGASLCVGGGMAMASLWSRDV*
>Syntrophomonas_wolfei_subsp.metylbutyratica_JCM14075T_01703_putative_enoyl-CoA_hydratase_echA8
MELKTLLFQKEAGVGIITLNRPETMNGINDQMMLELSVLMDEIAVDDEVRALIVTGGEKVFAAGGDIAYISGLNTLQAESFVALVHQAFDKIANCDKPVVAAIAGLALGGGCELALACDIRLAAEGSRFGLPEINLGIMPGAGASQRLTRIVGMGWARHLLFTGSPIDSDTAFKIGLVTMVLPREKLMEEAQKMARTFASRSPVALRTAKKCLNYAENVDLPSGLVFEQKLWSFLFSTEDQKEGMN
AFLQKRKPEFKGK*
>Syntrophomonas_wolfei_subsp.metylbutyratica_JCM14075T_01704_putative_3-hydroxybutyryl-CoA_dehydrogenase
MEIKKIGVLGAGTMGAGIAQVAAEAGFPVILVDIETAIIEKALKAVKKAWKKAMEKGKLDESEMQKREGYLSTGNSMADFKDCDVVIEAIVEKIEVKKAVFKELDEICPKNTILASNTSALSITEIAAATGRPDRVVGMHFFNPVPVMKLVEVIPGAESSEAVSSAIVELCKKLKKEPVLAREFPGFIVNRILVPYINEAAMAFQDGVASAEEIDKAMKLGANMPMGPLALCDLVGIDVLLMVCEYFWQEF
GDSKYRPSLALKQKVRAGHLGVKSGKGFYDYK*
>Syntrophomonas_wolfei_subsp.metylbutyratica_JCM14075T_01705_YcfA-like_protein
MGSKYPLLPPNDIIKVLTGLGFQKVSQKGSHAKYVKQGNPIRTTIIPMHREVARGTLKSILEQADLTLDVFLEKLYRNNSTQSR*
>Syntrophomonas_wolfei_subsp.metylbutyratica_JCM14075T_01706_hypothetical_protein
MNIKVTVVVQKEDSWYVAKCLENNVASQGKTIDESLQNLREALELYYEDEQPDIDSRQTFITTLEVAI*
>Syntrophomonas_wolfei_subsp.metylbutyratica_JCM14075T_01707_hypothetical_protein
MDDDLKKFIQFFRQAGAAVNIQFLKYRPQRGMHRAFRCHPVKGKQSK*
>Syntrophomonas_wolfei_subsp.metylbutyratica_JCM14075T_01708_Regulatory_protein_BlaR1
MDIFQMSFSAAFLIIAVIIIRAFALHRLPKKTFLVLWGVVICRLLIPFSIPSRLSFYTGVDMLRRAITAETAYIPAPAVLTGTPAMEIVDMGKLAATGASTISISMSPLISIWIAGICICTIFFLLSYIKCRREFQTALPVENDFINRWQQRHPLRRTVQIRQSDRIKAPLTYGVRHPVVLLPKAMDWTDETILQYILTHEYVHIRRFDALAKLLITSAVCIHWFNPLVWVMYMLANRDIELACDESVVRAFGDTAKS
TYALALIGLEEKKSRLIPLCNNFSKIAIEERITSIMKIKKYSLPVIIVAVLLIAGVTVGFACSKATVGQNSYLPEKSEELNPASPSPASEKTPVNDGETIAEPASNNDGHSSSSEIRKVYYSDGQHKTEIDGVPVHPVHCVVIGKYYYKRIDRETGKDQVSFDDGSTWLWTRNNPKSKKIEVSADGVTWEDLIATIQEPKYSEFCNHKYFYMRSDPDTGQELASFDDGVTWLFYRENVVTGRAEISSDGVKWR
EWIR*
>Syntrophomonas_wolfei_subsp.metylbutyratica_JCM14075T_01709_Methicillin_resistance_regulatory_protein_MecI
MEIKLFDSELKIMDVLWREGDTTAKKISDILNKQVGWNVNTTYTLIKRCIAKGAIKRSEPNFMCHALIAKEEVQEQETTELINKVFDGSADLLFASLLSRKNLSPEEIERLKQIVNNLK*
>Syntrophomonas_wolfei_subsp.metylbutyratica_JCM14075T_01710_Ribose_transport_system_permease_protein_RbsC
LRIKEEASRLLDSEYMVPAIFIILSYFAWQFSGLTGSFVFNQVVTRFIRDGILVLSLIIPVVAGMGLNFAITVGAMAIQTALLIVIAYQVEGIKGILLAIIIGVLLSLVLGYVIGRILNRVKGKEMITTIIIGFLFNSIYQLIFLVGFGTFIPVANKEIILTRGIGVRNTVDLGMYRNTIDKLGLFKIGELEIPLFMIIVLLFAAIIFYILHTRFGQEVKAVGSNMQTSEILGINTDMIRIKVMILSTILAAFGQLAYLQNIGMFD
VYTAHMNADIISCALLAGGASIKKARIRHALMGIFLFHTLFIVSPQAGQNLFNNAALGEYFRSFVAYGTIALALIINIKNENKFS*
>Syntrophomonas_wolfei_subsp.metylbutyratica_JCM14075T_01711_ribose_ABC_transporter_permease_protein
VKNYRLKPPQIISAFLILLIGLSYILHMDLVVLMNESMVKLVMNGVLVLSLIPMLNVGAGMNFGLPVGIIGGLVGMCLAVNFRMTGFYGFFMAILFTLMICTLLGWIYGLILNRVKGREEIAGIFIGFSFIPLMNYFWTLAPFQNREMLYPIGGQGLRPKISLENYFNHILDNYGLISIGNIEIPVGLIVIFAIICLFLYLYFRTKIGRATIAVGENEAFAKLSGINISQTRLIAIISTIIAGFGICIYAQSYGFIQLYDEP
LSMAFPAVSAILIGGSTGKKTFIFEAILGTYLLQSMYLLSVPIANEILVPELTEILRTFITYGIILYALLVRERRGIYS*
>Syntrophomonas_wolfei_subsp.metylbutyratica_JCM14075T_01712_Xylose_import_ATP-binding_protein_XylG
MENILEIVNLNKSYNGIPALSNINLSVKAGEIHGIVGANGSGKTTLMNILFGNPIIRETGGYQGQISFRGNKVNFKNCAEAIRQGIGMVHQEFALIPDMTVGENIKLGRENTHSLTDRLFGRKYSFIDRKQDYENAGQTLRRLGISVDVGIKIINLSVNIRQFIEIAREIDRTDLKLLILDEPTAVLNKNDGLKLIEILKDMSDHKGIIFISHRLEEVQYVCDKVSVLRDGKLVSQYAKGNIYVNNLAQDMIG
HTINKAAAEKREMENKPVITFKDFSVKMPGEEVKNLNLSVYKGEILGLTSLSGHGKLALGYGMMDMYPVDGEVYFEDKKLVKMNARTNIIKGMYVLPDDRQGLGILADQSLESNIIFTGNQIKNMFIRDSAFNFLGFIDHRKTNLHVYKMMLDLDIKCTSIKQKVKELSGGNQQKVCIARALTVDPKLLFVAEPTRGIDISAKEKILNMLIEINRSRNTTIIIASSELDELKRVCDRIAVMVEGKIFKILSP
DSSDVDFGLVLSGEEGVDCEKL*
>Syntrophomonas_wolfei_subsp.metylbutyratica_JCM14075T_01713_hypothetical_protein
LKKITSLLLVVLLMTTLITGCTKQTTTTNKEVKKLADYKIGVVTPTLSTSEDEFRAAEQMAKKYPDMVKHITLPENFSTEIETGLSQITSLADEPKMKAIIVISGQAGLLPALQKVEEKRPDIITMTAPIWDDPALMSKYVDVNLDTDWVRRGVTIAQKAHEIGDKTMIHYSFPTHLAKEVISQRKDAMEKTCKELGMQWVEVATPDPQTGNGTGPMLQFLREDIPRQIAKYGPDTNIFGSNCPMY
DVIIDEAFKLKYTVVEQCCPTPLQAYPTVLGLQISEADAWNFSKVNSMISEKAAAAGMTGRLGGWRVPATVFMPQFAVELAKKMIEDPSFNYKDVNNLNQFAKEVMGSEVSFSKFKAKADSPELDNYFVMIMKTILY*
>Syntrophomonas_wolfei_subsp.metylbutyratica_JCM14075T_01714_Anaerobic_sulfite_reductase_subunit_B
VHNCNCENPLVPQIVEIVKIIDETPDVKTFHVRNENGVPFDVKPGQLAMVSLLPVGEGMFSVSWQEEKEHLQFAIRRVGLMTDELHSVGVGHKIGVRGPYGNGFPVEACQGKDMLFIAGGIGLAPLRSFIKYCLKHRADYGKIQVLYGARSYADLCFKDELFELWPKEKDTEVFTTLDRPEEGWDGHVGLIPSYLEELKPAPEGKVAVICGPPIMIKFALKSLEKMGYNDEQVITTLEMRMKCGIG
KCGRCNIGSEYICLDGPVFNLAQLRKLPPEW*
>Syntrophomonas_wolfei_subsp.metylbutyratica_JCM14075T_01715_Anaerobic_sulfite_reductase_iron-sulfur_subunit
VKVLNKNKLPAVFDKLAQQSELFVPLQKKEQSGFYSWKSFDQASDQLALDLLNVYLPPKQVLLPQTEKMYSFRQEEGGVAISEVYEESQARVIFGIRACDLQAINCLDQAFLTRGFVDEFYKARRDNTTIVASACYKPGANCFCSSMGVNPLEPAGADAIIRDTGTEYFIWEARTPAGEELSRQLAEFLEEKELPLPQSQAFERQVDMEGVAEKLSQVFSSPLWEELSDACQTCGICTYLCPSCYCFDI
QVKNQGEAGYRFRCWDSCMYGEYTLMAGGHNPRASATERFRNRFLHKLEFFNERYGMPLCTGCGRCIIACPAGIDISKIITAAKEAEWSA*
>Syntrophomonas_wolfei_subsp.metylbutyratica_JCM14075T_01716_putative_NADH:ubiquinone_oxidoreductase,_subunit_RnfC
MDITGKIREIAARLLQNGEVDLFMAWEKGDLPFQSKPFFARRVEDVERIIFDEYSIHNLSNALLKFRDRQEKIGLVVKGCDSRGVVRLLEDGQIERDRLYIVGVCCPGMKDPLQAMRDYSGFNRNPEAAPLAAKCENCIQPNPVIYDELLGEEQPPRVQGERFARVRELEQLSPDERYRFFEDILSRCIRCYACRQACVACNCRTCIFDETKPQWVGRETSISDNMMYHLVRASHMAGRCIECGECER
VCPVNIPLMLINQKLIKDVDNFFGPYEAGMQYVEGAKPPLSVYQENDPDDF*
>Syntrophomonas_wolfei_subsp.metylbutyratica_JCM14075T_01717_Coenzyme_F420-reducing_hydrogenase,_delta_subunit
VSIDKSQWQPKIIVFACNWCSYAAADLAGLNHLEYPADVRIIRTPCSGRMDPLLVLRAFNRGADGVLLSG*
>Syntrophomonas_wolfei_subsp.metylbutyratica_JCM14075T_01718_NADH_dehydrogenase_subunit_I
MVAVAQHPNIEMITYAEVTQVKGYIGNFKATIKKKPKYVDWDLCTGCGTCAEKCPTKKLKDEFDLGLGLRTAIHKVYPQAVPGKFYIDAQNCTKLTSGKCGVCEKLCPAQAIRFDDKEEYQEVEVGAIVMATGYDLFKWEEAYGEYGYGKYPDVISSLHFERLVSAGGPTGGKVQRPSDGKEPKSVAFIKCVGSRDDTKGKSYCSRACCMYTAKHAYQVKDKIEDSEAYVFYMDVRTAGKNYE
EFYQRSLNAGAKYIRGRVSKIYPRGERLVLKSEDTLLGRPLEVEADLVVLATAMVPSEGAGALAKAIGFSSDKDGFYQEAHPKLQPVETFAAGVYLAGCCQGPKDIPDTVAQASAAAVKICGLFSKNELATEPMVTQVDVSKCSGCGFCVPICPYNAISLEPIQERGHHGPFTRNAAQVNSSLCQGCGACLPACRTLALNLKGFTNDQLVAEVDALCL*
>Syntrophomonas_wolfei_subsp.metylbutyratica_JCM14075T_01719_Dehydrosqualene_desaturase
VKKRVGVFVCDCGTNIASVVDTEKVAAAARQFPGVVFATTYKYMCSDPGQELVRNAIKEQELEQVVIASCSPRMHEKTFRKAVEDGGINAYMFEMVNIREQDSWVHHDREQATQKAIALVRMAVSKVLRNQPLQVSTIPVTRKALVIGGGIAGMQAALDIAEAGHQVILVEKEASIGGKMTQLDKTFPTMDCAA*
>Syntrophomonas_wolfei_subsp.metylbutyratica_JCM14075T_01720_Lactate_utilization_protein_A
MKLAYYPGCSLDGSAIEYGLSTKRTAELLGVELLEIEDWNCCGATSGHNTNKLLSLALPARNLALAEKTGLDVLAPCAACYNRFRNTEHVVRHDRKMQEKITAVIEMDYAASNQTLSILELIVNKVGLEKLREKVSQPLKAMKAACYYGCLLVRPQEHTGFDDNEDPRSMDLIMQALGAESVEWSFKTECCGAALATSRPDVGYRMIYEVIRNAREAGAECLVTACPLCMMNLDMRRRGVEKAF
NQRLDIPVYYITELLAIACGDSPRDVGVHRHFVEASYLASLPEKAARIEAEEAARLEAEQAKKAARAAQAKKAAQDKAKKKEASEAEAMAKAGDEKKAAPAAEAAAKAETKEKAAPAVEAAATLAEVGDKGSKAAPTQAQAAAPAAAEAVDDGALQKKITAMIKAWEKSPEKVAARLIEDEERAILAQVVSGDEKKTARLAELMITDKDKAAKAAEAFVTGELKKRQKAQES*
>Syntrophomonas_wolfei_subsp.metylbutyratica_JCM14075T_01721_sn-glycerol-3-phosphate_dehydrogenase_subunit_C
MGNALQLLPATQSDRALLKHLEEEAGTEVNLCYQCGKCSAGCPVGFAMDQTPRQVIRLLQLGLLEEALSTESIWICASCETCSARCPRGVDIASLMDALRREALKQGRITDRKVAVFNRAFLSGVKNFGRTYEAGLLLQHNLFTGQLFKDAELGLPMMQKGKLSFFPEKIKGREAIKKIFERAENPGGE*
>Syntrophomonas_wolfei_subsp.metylbutyratica_JCM14075T_01722_addiction_module_antitoxin,_RelB/DinJ_family
MAKTTNISIRMDKDLKERADAVFNEMGMNMTTAFNIFVRQTLRQGKIPFEIYADPFYSERNMNALHQSIQEAEEGKFVTKTLDELLAMEK*
>Syntrophomonas_wolfei_subsp.metylbutyratica_JCM14075T_01723_Toxin_YoeB
MKINFTEIGWADYLYWQMQGKKTMKRINQLIKDIERNGGLNGIGRPEPLKGNLTGWCSRRIDDTNRLVYRLRDDSWEIGQCRGHYSNK*
>Syntrophomonas_wolfei_subsp.metylbutyratica_JCM14075T_01724_putative_distant_relative_of_cell_wall-associated_hydrolases
VSKKIRFQSWISLLIFPSLTIILVLSIVAQNQAVFSNSDAVMYTYLKNACQGHAGYAYMSNCGNNISFAELEPGDILLGGYPDCAYGRFSHAGIYLGKGRVAEGYVDLGITIQTLDHYNNYSDICLLKVKAPQDVKLKAVDYVLEQEGKIFYPLAFKPGDRWWNCSKIMWKAYYEQGINLTPEADFWIAPDAFYQSPLLDVIAEEGWFK*
>Syntrophomonas_wolfei_subsp.metylbutyratica_JCM14075T_01725_hypothetical_protein
MSPASLIVSEANISGARRVLSGRLLTKQAAGSNSPPRCSAVLKMLKQFLPMLKKRNGRLK*
>Syntrophomonas_wolfei_subsp.metylbutyratica_JCM14075T_01726_hypothetical_protein
MRGILRKLDQGQKLGEDEYHRLMEYIEELRDKSPESYGLFYERYALLLYQDYSTYLPRFVQGIDHLLNLLMEKPELLPKLKEHQLAMELFPPELHPYLQYSFTQPADSLSLSILFNFLDNNQELVNQLPAARKNEVVCKFEEGNPYKEVGLKTHFDRLSRYSFITRLQSYRYLSAAKAASDRIEFLAADRLGGIFTNREKSIYYFIFLSEADEIKARNACRLLNMVFYGGK*
>Syntrophomonas_wolfei_subsp.metylbutyratica_JCM14075T_01727_hypothetical_protein
MSLMAYNDWPLILDNYRSVQDSPDLFFFWQEELRLRQLGRNLEKSPQKVLVKQAEELDFLLRSMYFSGQQPQFFNILLQNMHLTFVLQWLLDSPRYVLEAFLDYLPWYLSSSRINKRNLLFLIQIYQESFKDKFRAIINTLDAEACGYIAARTASPELRELIKLREEELEQSRKENYYAIKREAYKNNLYPSIFGDKIELFVQAIDSIEATFPEHFTEPYGAPRFLSLLESAELVFQCGWPEDSLAILLDVYEDY
QQKNRLVKILDDENIYRQFYKVLRRVIPVYSLLFGLPDCLNRAKSIYQHAFPRILPDSASLQYLAVYESVLAGLNAVLQHPQWEIIIKISPIQKQRPSEPPLLLPSEVGSGLSPRRWIELQELIEQKMASLPHEAFITLEYLRFLQLHFTPDREQQLAQRLMAGYLALWKWLPSPLFINPSLLEQLGPLLPASERTEAQKILTFLDDHDKQSLNNELDSRPELFHNKAKSTLREILIGQFAGVW*
>Syntrophomonas_wolfei_subsp.metylbutyratica_JCM14075T_01728_hypothetical_protein
MKELEINLDFFLDKLESFLQQLFQEINTVSGARTWKEGELEKMENHLYEINSFYYRSFNEHFFPAYRSELKEELAGLLKLKRQQDSIKRDIMANLQRYARLYNQVKNLNFSLQQAQENCLLARESIDEKEKERGIELYYRLQKTRDAISYVNELLYQIKQLEQDKEAMAALNRESSSIILINILKLDRDNPEELDYFYQWMKSIHHWNNVLLDLLENQESLPRKAAKFIAKFSKEYRELKERALPSFSFMEPY
PAYWHTQELEFFDLYLTSLKNGGVLRVVKALQLFLSDTLLFMTKALASEGENKNIGTGALAMMDYCHIKTLNPNLKELEEIISKNWQNLDHLIKEFPARGEMDMDYFLERFQEIISDSYPAVSTAKSSLGSRQNTALASALYRATLELSSSKAHIELLKEKQHYGKAIQQSYLQLAALLSQHKEWIANFRSDLERLLAPRNLSRSWKDLSLRVEKINLQKGREFPEEFLYLLLDKYQIASRVSPERPNLILEEE
GDIFIFKVDDLQEELIPFFVVSAGPVDSGSE*
>Syntrophomonas_wolfei_subsp.metylbutyratica_JCM14075T_01729_Heat_shock_protein_70
MQLGLDFGTSYTKLGLQHNGEFINLLGNEDEGQIPTVAAYLPSQRRLCFGPLALRIDEAGAETAFFFKLALKRQASLTLGSYSLPDLLLAFFRFLQQEYLESRGIKAESLNISVPNYFGLNSRRILLEAARQAFGVKKVFLLPEPLAAALGYNLLHPLFPLQGDILSIDIGGGTSDFSFLTLAHDKREMLVETQFQIGHDAFSGSEIDQAILQRLLFPAFTMQTGLRLPREFYSGKFTSSRNKFQYNRLRQLA
EKIKIELSREEYYHLDFPDFHAGYSLNLELTAEMLAARLEPVFERLKSYIEEQVKLRAKALQLFSADKWHIDAVLLLGGASQTRGLKDLIAGIFPDLKVICPEDPSFYVLRGLCHWENDGNSGATSIKSIYPFNFYIERISPDGSSHLLEKIPFDTANLELDLQKRYPIFSLPLDSVYNLAPDPDSILFRIYEVEEEDNQVNLERFSGQEMVWQWEGPRQELHNYLELYLDLKKSRIESGEALSATRTRPLDSNIL
SGLLARQKEIQKLMADSHSNPRLAEDFGEHLSRLEKVNKPYSNHSETTLYKLLYLLDFFTRPGN*
>Syntrophomonas_wolfei_subsp.metylbutyratica_JCM14075T_01730_hypothetical_protein
MDATPGEGEQAKGGERQEVARALPPMDGRLARYPLSRETSRRCCPCEAPCRAQLMLPAVAIKDAGAVYLYKPMNINFL*
>Syntrophomonas_wolfei_subsp.metylbutyratica_JCM14075T_01731_HTH-type_transcriptional_repressor_YcgE
VQEIYYKISEISEILGVEQYTLRYLENSLGLKIKRNERGDRLYSESDLETLKLVLQLKNKGLNTTAIKMALENIEESEEKAISPLENKGQGMMEVVSMAKKIVEQNQTLLEQNRKLEQRMEKLEQKIDKRNLERERKIDEFLELWKAEQARGKSWLSRILSK*
>Syntrophomonas_wolfei_subsp.metylbutyratica_JCM14075T_01732_Acyl-CoA_dehydrogenase,_short-chain_specific
MAANFAINNIRDFEFIIGEWLPTERVFNYPQFADYYSKDDIKSVLGPILKMCKEVIEPTNDDGENHPIKFENGKVTTPPTFGPLFHQLQSEGWGTSNIDKAPDAMVMPEMLHMAVWELIGAANPTFMPYVLLTTGAADLIQSFGDEKLKQMFLPKMMDGTWSGTMCLTEPSAGSDVGDILSRAYPTDDPRIFKIKGNKIFITAGDNDFTENIIHLYLARIEGARPGTGGISLFVVPKYWVNEDGSF
SDNDFQTTGVEHKMGQAGSVTAALAAGENDGCRGWLLGNDPREHEGKGQGMAQMFQMMNLARMETGHMALSCIINAFCNARDNAKERVQGRLLTNPKAGRVAIINHEDIKRTLLMGKAHIEAIRAMMYRVYLAFDQRERDPDPEVRKAASDLIEMTTPLCKAYPSDEGWWLISEAIQSYGGYGYCEEYPVAQIARDMKIYSIWEGTNYIQSMDLVGRKMQMKGGSLFGAWVKEMVDFC
EANKGNEALAKEFAILDKALKAYQEMLKVYAGYAKTNFSLVPLYSRRVLTITSQLYCGRQILDQAVLADKKAKELGADHYDYNFYSGKVASAKYYLHNVVPNVWSVCEIIADGDSSALEVPLEAFEYM*
>Syntrophomonas_wolfei_subsp.metylbutyratica_JCM14075T_01733_Inner_membrane_protein_YaaH
MSNSNEMHVSGHIQELVANPSPLGLLGLAMVTLVASSQKLGITTGTAFIIPWAIFLGATAQFVAGLMDYKHNNTFGATAFCGYGFFWFGVAMTWMMANGTIGMSADITPDIHQLGFAFLGYFIFTVYMTVGAAGANKVLFIIFFLIDLLFLGLFMNTLGLGGEAWHELAAYSEMGIAIMSFYGSAATVLNSHYGFTVLPVGAPFGSLARKAAQLKK*
>Syntrophomonas_wolfei_subsp.metylbutyratica_JCM14075T_01734_hypothetical_protein
MKSKSFLFTLLLALLFGLAGSGIILPALAAETQPVKIFVDGRQVDPGEAAPFIKNDRVMVPLRVISEGLGLKVDWDAENYRVLISSSSSANLSSLPADGKGQFVQIYFDGREIKPDDVSPLIVNDRTMVPLRSTFEGMGMKVDWDDTNYQVIVMTPQARPVDSSITPVTPVNDPPAPLADGSPQPAAVNNDYEISIMGDSSATPEQLKALLQENNPLAPDLVELYLQMGREYGVRGDIAFCQAAKET
GWWKFGGLALPEQNNYCGLSVTGKAATATEDLRGANPQKVRFIEGKHGAFFDSPANGVEAHIQHLYAYATAAPLPAGKELLDPRFILVNRGCAPRWIDLGGKWATPGFDRNKYSGFAEAFAAEDTYGHSILANYYWKALPPATI*
>Syntrophomonas_wolfei_subsp.metylbutyratica_JCM14075T_01735_Autoinducer_2_sensor_kinase/phosphatase_LuxQ
MSGRIWWAESSIVQGNGNPADMVKSQFLANMSHEIRTPIIGILGSLDLLEQSDLNHQQAANISVIRECGEQLLQLIDNILDVSKIESDLVAPNLEPCNLGELIMATVDRVDALLKRKGLESKLNIDSRIPAMIMLDRTKLQQVMLNLLYNSIKYTPEGRVELKAKIHPGNSSRLLFSISDTGIGIPSGFLEKVFEPFTQADNSNSRQYGGAGLGLFICKKLLEIMEGKISLNSMPGVGTTVSFSIPLIEVDETEI
EAPHELDREQLGNRDDSLLDFIPIRVLLVEDNPLNQKIVGQMLYNYGFEVIMAPNGLEALKILQDKPVNIVLMDMQMPVMDGYEATQFIRQHSKWRDIPIIAITAHSLPGDREKCLACGCTSYIAKPFKAHELVQLMKEYIKRDLKESVSSQRLIAELLPEFLILLDEMIDDLEAAINSRNIDKIQAISHDIKGTAGMYGFMDFSKLAAAIEQASRIKDLTKVKQIFSELYQEVEKVKIQVS*
>Syntrophomonas_wolfei_subsp.metylbutyratica_JCM14075T_01736_5,10-methylenetetrahydrofolate_reductase
LSLEIFPPRMAYPLESVFDTVQELKKLNPAYISVTYGAGGSNRERTVEIAARVKKEYGIEAQAHLTCIGHRQEDIKNILARLNREGICNIMALRGDIPEDEPEFDSSRQEYHYAFELIRDIRAAYGDSFGIAAAAHPEGHPECPRLKDDIFHLKNKADNGVDFLITQLFFDNRIFFDFWERCQATGINCPVIPGIMPVLNAGQIKRMIYLCGASMPARLLKLVDKYEKEPADMEKAGIDYASQQIKELLENAV
AGVHLYSMNKPRQISEIVYNIGLAYPGKD*
>Syntrophomonas_wolfei_subsp.metylbutyratica_JCM14075T_01737_hypothetical_protein
MVPLELRDKPQVLSMRSALRAQLMLPTVVIKGAKRYIYTSQ*
>Syntrophomonas_wolfei_subsp.metylbutyratica_JCM14075T_01738_Isocitrate_dehydrogenase_[NADP]
MPVGEKIRVEDGVLKVPDHPIIPFIEGDGTGPDIWAASRVLVAAVEKAYKGQKSIVWKEVLAGEKAFQQTGEWLPQETIDTIREYIIAIKGPLTTPIGGGIRSLNVALRQELDLYTCLRPVRYFEGVPSPVKRPEDTDMVIFRENTEDIYAGIEYPRGSEESDKLLKFLQEELGVKNIRFPGSSGIGIKPVSEEGSKRLVRAAINYALSEGRRNVTLVHKGNIMKYTEGAFKQWGYDLAESEFGNKVFTWD
EYDRIKEEKGTAAADQAQKEAEEDGKLIIKDAIADIFLQQILTRPNEFDVVATLNLNGDYISDALAAQVGGIGIAPGANINYVTGHAIFEATHGTAPKYAGLDKVNPSSVILSGELMLRHLGWNEAADLIIAAMGKTIASKVVTYDFARLMEDAQEVKCSGFADALIANM*
>Syntrophomonas_wolfei_subsp.metylbutyratica_JCM14075T_01739_2,3-dimethylmalate_dehydratase_small_subunit
MDLKGQVHKFGDDVNTDYIISGRHKFKTLDMKELAKYVMEDLDPEFYSKVKAGDFIVAGSNFGCGSSREQAPLAIINANISAVVAKSFARIFYRNCINTGLPLIECDTEQIDEGDELEVDLQKGVLYNRSKGIEIAVTPLPDVMLKILSDGGLVEHFKKYGTFNFD*
>Syntrophomonas_wolfei_subsp.metylbutyratica_JCM14075T_01740_2,3-dimethylmalate_dehydratase_large_subunit
LGKTIAEKILSTRSGRDVKANDIVVAELDFIMGQDGTSPLAIQAFQEMQARRVFDPARIAMVIDHSAPSPLEGVSELHQQMRSFAGEQGICFYDIGDGVCHQLLPEQGHVVPGDLVIGADSHTCTYGAINVFSTGVGSTDLAAGLISGKLWFKVPETFKFVLNGQLPAGVYSKDLILHLIGDVTADGATYMAAEYVGEAIAGLSVEARFTISNMAIEMGAKAGLMEADQKTLDWVKQHSTKSFTPV
KADADAVYARIKEYDVSRLEPQVARPHTVDNVAPIGEVAGTPIQQGVIGTCTNGRIEDLRIAASILKGRKVKSRLIVAPSSRQVMLQAIREGLIEIFLESGAAVVTPGCGPCVGTHNGVPSDGENVISTANRNFKGRMGNNRAFIYLGSPATVAASVIKGEIGDPREFLR*
>Syntrophomonas_wolfei_subsp.metylbutyratica_JCM14075T_01741_2-isopropylmalate_synthase
MTRSSMNKDWFLEEGARVNIVDTTLRDGEQTAGVVFTNDEKVQIARYLDQIGVDQIEAGIPVMGGFEKDCIKEIVALGLKSSIMAWNRAVIADIKESLDCGVDAVAISISTSDIHIEHKLQTTRQDVLNRMSDAVKFAKDKGLYISVNAEDASRSDIDFLTEFALLAKRSGADRLRFCDTVGTLTPLSAFRYIKTLIDAVGINIEMHTHNDFGMATANALAGVYAGANYVGVTINGLGERAGNACLQ
ETIMGLKYLMNVNLPYNTTLFREVAEYVAQASGRALSVSKPIVGSGIFAHESGIHGDGVLKNPLTYEVFSPEEVGLERQIVIGKHSGTAAVRSKFTREYSIELDDTEASQILARVREMSIELKRSLFDKELMYIYEELHGKTR*
>Syntrophomonas_wolfei_subsp.metylbutyratica_JCM14075T_01742_Aspartyl/glutamyl-tRNA(Asn/Gln)_amidotransferase_subunit_B
MSKEFETVIGLEVHAELKTESKIFCSCSTAFGAEPNSQVCPFCAGFPGALPVLNEKVLELAIRTGIALNCEIPEYCKFDRKNYFYPDLPAAYQISQFDQPVCKNGYLEIDLDGEIKRIRINRAHMEEDAGKLVHQGDITTTPFSLVDLNRCGIGLLEIVSEPDMRSAREARAYLEKLRSILLFAGVSDCKMQEGSLRCDANISVRPAGQEELGTRTEIKNLNSFRSLERAIEYEARRHVEAIEDGEELLQETRT
WDEEKQLTRSMRSKEEADDYRYFPDPDLPPLRISRERIEEIRRQMPELPDAAQERLVETYGLPHYDAAILTTTPDYLEFFDRVIKIYPDAKVVSNWMMSELNKYLNQSNLEISDCLLKPEKLAEMLQLIDDGTISGKMAKTVFAEMFNSGKSAPEVVKEKGMVQISDEKALSAIIESIVNGNPKVVEDYRNGKEKAFGFFVGQVMKATRGQANPAVVNKLLKEKLK*
>Syntrophomonas_wolfei_subsp.metylbutyratica_JCM14075T_01743_Glutamyl-tRNA(Gln)_amidotransferase_subunit_A
VDLYKLTVHELQDKLLAGEISSEDIVKSLFSRIAVVEEKAQAFITLCEETAWESARRIDQQGEYGGIKGIPYGLKDLFCTRGIKTTCASRMLENFVPSYESTASKLLNEKGGILLGKLNLDEFAMGSSTEQSAFFPSRNPWDWERVPGGSSGGCAAAVAAGEIPFALASDTGGSIRQPASYCGIVGLKPTYGRVSRWGVAAFASSLDQVGILSRNVRDCALILKIIAGKDLLDASSADIEVPDYSAFLDGEVK
GMRIAYPREYFRQGVTESIRTAVMKALRKFEELGAIVEEVSLPHSEYALPAYFIVAPAEASTNLARFDGVRYGWRDGEAANVADMFSLSRAQGFGPEVKRRIMLGTYALSSGYYDAYYLKAMKVRRLIADFAEVFRDFDLIVSPTTPTTAFQLGEQIDDTLTLYMNDALTVPVNMAGLPGISIPCALDDGLPVGMQLIGRAFDEATLLKAAYAFEQNTDYHRLTPVPGGGINE*
>Syntrophomonas_wolfei_subsp.metylbutyratica_JCM14075T_01744_Aspartyl/glutamyl-tRNA(Asn/Gln)_amidotransferase_subunit_C
MALSPEEVNHVAMLARLALSEDEKTAFAEQLSLILDYVERLNELDTGEVEPLIHILPVFNVLRPDEALPGSSQEEILSNAPLVEDGQYKVPRII*
>Syntrophomonas_wolfei_subsp.metylbutyratica_JCM14075T_01745_putative_membrane_protein_YdfK
VTGGIAGEEAVTGTLINAGAIVAAGAVGLLLRRGIPENISRSLQDAMGLLILIIGIQYGLKTENLVVVGLSLALGTVFGEWRNWEGKLENTGKRLERLLGQEENLFVKGFVSATLVFCVGAMAILGALQDGLTHNYDILLVKSMLDGIMAMIFAASMGVGVLFSALPLLLYQGAISLGAGFLKPLLTDPVINNLTGLGGIIIAGIGLNTLGLARIRLANLLPGLLLVPLLMFLFK*
>Syntrophomonas_wolfei_subsp.metylbutyratica_JCM14075T_01746_DNA_ligase
MEVSSRIKELREEIQKHDYHYYILDAPLISDASYDRLMQELKKLEEECPQYITADSPTQRVAGKASEKFSPVRHRFPLLSLDNAFSYQDLLEFDRRVGRVTRTLSYMAELKIDGVSIALVYENGVLLNAATRGDGLVGEDVTANIRTIKTIPLRLRYSLPRLEVRGEIFMPKQEFIRLNEEKEEKGERVFANPRNAAAGSLRQLDPRITAGRALSAFVYDIIYMEGQNLAEQQEAWHFMQELGLPVNPEVRF
CADINAVLAFTEEYAEKRHELPYEIDGVVVKLNTLAEREELGATARSPRWAMAYKFPAEEKETRLLGVEINVGRTGIIAPTALLEPVFLAGTTVSRASMHNFDLVKEKDLRIGDMVLLHKAGDIIPEIIASLPEKRSGEERVIMPPENCPACDSKVARFAGEVAYRCENINCPARLKESLIFFASRGAMDIDGLGSAVIEQLVDKEMVKRIDDLYRLREEEITALERMGPKSAANLIKAIDESKSRPPSRLLTAL
GIRHIGARSAKILSRHIHDIDDFYKLGVENLTSIPEIGPKMAESIVNFFAEPRNRETIENLKALGVNTREAIEAGEQLLQGKTFVLTGTLPSLTRQQASEMIESRGGKVSNSVSKKTSYVVAGDDPGSKLDKAQQLGLTILDEAGFLNLLGLS*
>Syntrophomonas_wolfei_subsp.metylbutyratica_JCM14075T_01747_ATP-dependent_DNA_helicase_PcrA
MQNIFADLNEQQKEAVAHVDGPCMVLAGAGSGKTRVLTRRIVHLVKQGIPPHRIMAITFTNKAAQEMRSRVISMLPDFNSQWIQTFHSTCNRILRMDIQELGFDKYFSIIDDTEAKSLIKTLLREENDYETKPEELLYIFKQAKNSLEKPEKYFYSFAVPDFIKERNCRVFSLYNSRLKNLNVLDFEDLIVLCIRLFQQNPAILEKYQGWFQYIMVDEYQDTNYSQYFWARLLASRHHNIFVVGDPDQSI
YSWRGAEPYNIKRFLGDYPDSRLIKLEMNYRSSGNILSAANAVICNNEEREEKQLYTQKGQGEKLVHFCAGDSFQESRFIADEINQLLRSENMNFRDFAVFYRTHAQSRVLEEALLYRNIPYHIVGARKFYERKEIKDILAYLRLVSNNNDLLSFERVINVPRRGIGEKTLGRIKAYSEERGISLLDVLAEAESIPGIGRKMLAAMEDFHGILKYLTDLVSSRASLMELLEQVIEVTGYIEELKKSKAADAEARIE
NLQELRSLVVEFEREGGQGLEEFLSQTALVQESDELDNSDHVLLMTLHGAKGLEFPVVFITGLEEGVFPSYRSQTVDEMEEERRLCYVGITRAQERLYLSHALNRLLYGYERSNPPSRFLYEIPEELLLSPQQKKVAKPVFGAGDRVLHAKFGMGIISELNEEEQIAVVEFVQAGTRMLRLDIAPLEKID*
>Syntrophomonas_wolfei_subsp.metylbutyratica_JCM14075T_01748_Smr_domain_protein
MKVQSLNLHGLSLEEARQKTRQNLIWCMQNGVDVLDLNHGKGYHSSRNFSVLKQELRRMLKSEPSLKEFGYKVVYGESNLPVALAYDEGHTLIVARGKEKEYIGGWKEQEKKQRIFSPEGRQERKQRKKRSK*
>Syntrophomonas_wolfei_subsp.metylbutyratica_JCM14075T_01749_putative_membrane_protein
MLKRFLLYGTLGWTTEILFTGMGSLLNASWRLGAHTYLWMFPIYGLAVFLEPFHDRIRSAPWPVRGFIWASLIIGIEYLCGWLLKISLGFCPWDYSGASFFSLDGFVRLDYTPFWFIAGLAFEQIHDYMDRIGIS*
>Syntrophomonas_wolfei_subsp.metylbutyratica_JCM14075T_01750_Anthranilate_synthase_component_1
MNNISQHLAGFRSLRYSSEGGVDIERRVRSIELNEATEPIIQQIDRQKGALFSSSYDYPGRYSQWDIGFLNPCLELRAQGRDFCIEALKSSGLKLLFGLYAELRGREEFTALLLKQNKIEGNIKKSPELCWEEERSRQASIFSLIRVMRRYLFYPDDCFLGLYGAFGYDLVYQFEPMELKRPRPEGQCDLLLYLPDELTVVDHRMARAYQLSYSFRSSGESINIPQYSREGIYPYEVLSRQQERSLPGHYTALVK
KAKQSFQRGDLFEVVPSYSLLEPCPIAPSEVFKNLKSINPSPYGFIINLGDEQLVGASPEMYLRVEGKRVETCPISGTIKRGKDAIEDALQIRKLLNSGKDEAELTMCTDVDRNDKSRICEPGSVKVIGRRQIELYSHLIHTVDHVEGILRPDFDALDAFLTHMWAVTVTGAPKRAAIKWLEENEGSPRGWYGGAVGYLSFNGDLNTGLTLRSIRIKNKLAEIRVGATLLMDSIPEEEEAETLVKAAAMLK
AIQGASADRRPLIVPDLQLEKQAPRVLLVDHEDSFVHTLSNYLKQLGAEVWVTRAPLARERLRQGQDFDLLFLSPGPGRPDDFQLPETIDLAIKRSIPVFGVCLGLQGVVEYFGGRVEELPYPCHGHASLLTVSQSSPLWRSLPRQFRVGRYHSLYAAVLPDCLQVSAVSEDGVAMAVEHREMPVAAIQFHPESILTADNGNGLQLKNVLNYLALPQKQAVAGRVFLTDGEELKGRLPSIAP*
>Syntrophomonas_wolfei_subsp.metylbutyratica_JCM14075T_01751_Phospho-2-dehydro-3-deoxyheptonate_aldolase
MLACKLASRSPDSNNNTIIKIKTAGKEISIGDGNCTIIAGPCSVESREQYLEIALILKEMGVDILRGGAFKPRSSPYSFAGLGTEGLKILAQARELTGLPVVSEVMDPRDLDQVAAHCDLIQIGSRNMQNFSLLKEAGRVDKPVLLKRGLAATIEEWLLACEYIMDAGNEKVILCERGIRTFEPLTRNTVDIGAVALVKELSHLPVIVDPSHATGRRSLVAPVARAAMAAGADGLMIEVHQHPEEALSDGE
QSLTPEQFAALTERIRAMA*
>Syntrophomonas_wolfei_subsp.metylbutyratica_JCM14075T_01752_Tryptophan_synthase_alpha_chain
MWKKWLVCWEVLGVKIRNRWDELKKKEEMALIAFIMAAVPDEDLCLECIRALEQGGCDLLELGVPFTDPLADGEVIERFHHRGVRRGLNLKRGLDFAARVRDACQLPLILFSYYNPILQMGLDKFAGDCRSAGVEAVIVPDLPLDELGRLAGQGLELIPMLAPSSTLSRVQMAAELDPAFIYCVSVRGVTGLRSLPEMEIKDYLQKVRRVSTAPLALGFGISQPEQVRAFREQADGVVIGSALAQIIE
EYESRPALLPGILEKRCQALKLLS*
>Syntrophomonas_wolfei_subsp.metylbutyratica_JCM14075T_01753_Tryptophan_synthase_beta_chain
MYPDERGYFGRYGGRFVPETLIPALMETEQAYRELAGDEKFRQELQLYLKDYAGRESPLYFASRLSENAGKARIFLKREDLNHTGSHKINNTLGQALLARKMGKKRLIAETGAGQHGVATATAAAVFGLECTVYMGSKDMERQRLNVFRMQMLGAEVRGVKKGSETLKDATNEAFRDFIRSFEYSHMVIGSVVGPHPYPMMVRDFQAVIGRELRRQFQEQEGGLPDCIIACVGGGSNAMGIFY
PFLEDPVKLLGVEAGGCGTAPGQHCATLSRGNDGVLHGAFSRLLQDEYGQVMDTHSISAGLDYPGVGPEHASLRERGRVDYVQVSDREALDAFMQLCRMEGIIPALESAHAVAYALKIAAALEEEKSLVINLSGRGDKDVEEVARLLGGVGSEN*
>Syntrophomonas_wolfei_subsp.metylbutyratica_JCM14075T_01754_N-(5'-phosphoribosyl)anthranilate_isomerase
MTRVKICGIRCLEEAIAAREAGAWAIGQVFAPSPRRLEVDVAAAINRKLGRTILKIGVFVNEEVEDLRRIAASCRLDMVQLHGDEEPAYLEEVSVPVIKSFRMRGSLELEQLRRWRPWAYLFDSYRPGVYGGTGESFDWSFLQEIARQERIILAGGLNTENVGRAIHQLRPMAVDVSSGVEYPGGGKDPAKIREFINIVQEQAAEYL*
>Syntrophomonas_wolfei_subsp.metylbutyratica_JCM14075T_01755_Indole-3-glycerol_phosphate_synthase
MLKTIAEAKLKEVKRLRESLDIAQVEARCETSRQISLSRDPGGKLQLIAEIKKTSPLKGLLCREFSPLEIARSYEANGAAVISVITEQEFFDGRKEYLPLVKTGVKLPLLRKDFIIDEVQVYESWQMGADLLLLIAALHDYDSLLALVEKCSALDMEPLLETHGRDEIKMALDLPVKIIGINNRNLRDFSVDIRSSLDLLPLIPDSYLKVSESGIKEAGDLRLLESAGCDFALVGEALVTAADPGAKLRELLSYRQE
QGL*
>Syntrophomonas_wolfei_subsp.metylbutyratica_JCM14075T_01756_Anthranilate_phosphoribosyltransferase_2
MFDRYLKSVVEGDYLNSEEAYLTARMLLHEDIPEIKAAAFLSALRTRKESQEELTGFVQALYEEAKIVDCEMEVLDTCGTGGDGLGTFNISTASALVVASCGVAVAKHGNRAVTGKVGSADVLEALGVEVQLEPDEARQLLDKAGITFLFAPHYHPILKQVGGLRRGLGIATIFNFLGPLLNPCRPPYQVLGISDSNLQEAVAGTLQRLGRKRALVLHAFNGMDEISPEGKTRVFDVGEHGLEVFDI
DPDELGIGGFSLDAIQGGDAAASAHLLMRVLQGEPGPYRDTVILNTAAALLTAGRAKNMQEGMQMAAEAIDAGKSMETLQKMISFSRDRVLAC*
>Syntrophomonas_wolfei_subsp.metylbutyratica_JCM14075T_01757_Cob(I)yrinic_acid_a,c-diamide_adenosyltransferase
VLQIYTGEGKGKSTAAFGLAMRAAGHGCRIRVIQFLKGNSFAGELRSAKKLGIEVFSFGRSCPHATLINGGFMECDECAECWVNPEKLTDSDRKKASMAWQLAQNTVREGSIDILILDEVFAALELGLLPFEDFLKWLGNASSGMEIVLTGRNAPPEVIAMAHLVSRIEKIKHPFDINGHPRRGIEY*
>Syntrophomonas_wolfei_subsp.metylbutyratica_JCM14075T_01758_Chemotaxis_protein_CheY
MAKILIVDDSKLIRRNLRIILSRAGHEIIAEAENGLQAFQEYEKNQPDLITMDISMPLMDGVAAVKKIISQYPNAIIIMISAMDQKNMVMAAIQSGARNYIIKPFSPDKVVAVVNAALTTGGKAVAASPQHSALEDKQVLYPFYIEAHDANVRVTIKQSFASGHVKPLLDALQSFFNKKPLQVTFDFASVQIYEYSLIDQLLEIMQIIKDNEGKLRVMAEDKKITRYLKSKGIM*
>Syntrophomonas_wolfei_subsp.metylbutyratica_JCM14075T_01759_Chemotaxis_protein_CheA
MRNSLKSRFIIVVFTVCFCCLLVVSLVSYYNSYILVREETNQVAHEAVSRYASELNNWFMQQAGVLHAMSQDIEINRGFSDEYLSLYLLEKFQRCDSCVIDYYIGFADSNRKMVCATGWVPPEDYEASKREWYKEAVINNKLVYITPYLDADTKEMVITLAEPIRDGDKLLGVLAADILLTDVMKLAQDVHIDKKHYSFLLDDRYNFIVHPHPDFQPLASESRNASQVMQGLYRPLTERIQEGRYPIIEL
KDYDNSKRCFALSEIKETGWTFGIAIPHSEYFGPLKKLWYGFALALAISLFIGFIIILPVVRSLLRPIEDLHQAVGRFADKDFSARSPVTSTDEVGELSNNFNRMADIIQEYNVGLERRVEERTVAIRNLLDNAGQGFLSFGSNLQVNQEYSSECKRIFAYDVAGQYFPMVIFPGDEAERQFMENALQTTLEEKDEYRRGVYLSLLPEELMINGRHIRLEYKVISDQHQQENRSMMLIITDITEKRCLEKQME
EERLLEMVVKVVANRDDFLEYRNDFRRFYVHGIEELLDSGLPRDEIIAEIYRQIHTFKGGFAQMRMSHLFNQLHDCESEIAASRDELREMSAVEIKDFFDSFKLLEAMAKDISLLEEVLGEDFLERDNKLLIDEQRLLEIKQKITCLAPWECKMLLPYIKRLRFRPFKELLRSYPENVASLAESLGKSLLPLRITGGDMLVDSDYYHDFCKSLSHVFRNAIDHGLESPEDRLELGKNEYGIISCDIKEEDGGI
AIVISDDGRGIDIELLKQQALDRGLCDEEYLEGLSFEEALQLIFADSLSSKEKVSSISGRGMGLPAVKAELEKLGGIVEISSEKEWGTRFCFWLPAHDKIDVEDFNIEGIINPLLETAREFFSREIGITLLHSSEVKRKKRQELLRITAFITIKGVIRSDFILSADEELGRYLVQAMTMDEIESGAEEDFVEDVLAECANIILGNSIKLFPGISELVMMEVPVTLYSDQTAIKHLQADIWTCDFLFEEGRLRLGLISQKD
IYSCY*
>Syntrophomonas_wolfei_subsp.metylbutyratica_JCM14075T_01762_PhoU-like_phosphate_uptake_regulator
MINETLACEMEKLKEEILKMGRLLEEQLYKAVKSLVEKNLELAREVIAHDDLIDQMEMDIEKKSLGILALKQPMASDLRLIGTALRIIVDIERMADHAEDIAHLTIDLYDQVYIKPLIDIPRMAAIVQKMLEKALKAFVNHDVNLAMSLIPLEEEIDNLYDQIFRELLSYMMQDPKNIPQATSLLLVAGHIERIGDHATNLAEMVVYLVEGKRIDLNRMARKSRSASKK*
>Syntrophomonas_wolfei_subsp.metylbutyratica_JCM14075T_01763_Phosphate_import_ATP-binding_protein_PstB_3
VEKAIKMKSRELNLFYGNFQALYNINMDIEENRVTALIGPSGCGKSSFLRSLNRLNELIEGSRISGEVFLDDSAIYEKGINVVELRKKVGMVFQKPAVFPMSIYENVAYGPRIHGIKKRSELDEIVCRSLQSANLWDEVKDRLVAPANQMSGGQQQRLVIARVLAVQPEVILMDEPASALDPISTARLEELIAILKTRYTIVIVTHNMQQAARVSDYTGFFLHGCMLEFNRTEELFLNPRHNKTEDYITGRF
G*
>Syntrophomonas_wolfei_subsp.metylbutyratica_JCM14075T_01764_Phosphate_transport_system_permease_protein_PstA
MPGTRIGEKALTCLLYLSALIIGILLAIVGYIVYKGLAMINLDFIMQAPRRAGKEGGISSTIVGTLYLTVLSLAIAAPLGVGTAIHLEEYAQKESYFAYLVTLTAETLAAIPSIIYGLFGFVFFVIYLKLGWSIWSGSLTLAIMVWPTITRTAQEAILSVPREYRENSLALGASKWQTIRKIVIPAAIPGIVTGMILALGRAIGETAAVLLTAGTSLGMPLSPADPARSMSVHLYMLAMEGLSMERAFGTAFLIIILLLFI
NYLANRSMSLLSHNGG*
>Syntrophomonas_wolfei_subsp.metylbutyratica_JCM14075T_01765_Phosphate_transport_system_permease_protein_PstC
MNGIIEGKITAVKQHNNREEKSQILQAKAGRWERILVILRKSKARFIPEILVEKLLMLSAVVSIMALLLISVFIFSRGVPLIASVGLGDFLFSLEWKPIQGKFGIGAMFVGSLLLTSASLAWAVPLGLLTAIFMAEIAPPPVGRIMTRMVSLLAGIPSVVYGFFGLVIIVPLIRNYLGGTGMSVLAGAIILGIMILPTIINISRDAIQAVPLVYKESSLALGASHYQTIGRLILPAASSGIITAVVLAMGRAIGETMAVVMV
TGNAAIIPDSLLAPLRTMTSNIVLEMGYAAGDHQAALFATGAILFIFILLLNLLLNFIRRAGKRYAGN*
>Syntrophomonas_wolfei_subsp.metylbutyratica_JCM14075T_01766_Phosphate-binding_protein_PstS_1_precursor
MYSEKLRRVGSFIILGLLLLALVFTSACSKNLSEPESGVKNGGNDELQGSITIAGSTSVQPFTEVLAEEFQSKNPGVKINVQGGGSSQGIETAISGGADIGASSRELKDEEKKAGLKETTIALDAIAIVVHPSNRLGELKMEDVRNIYLGNIANWKELGGADAPISIVCREEGSGTRGAFEEIVMNKQDIFAKSLIQNSTGAVRTTVAGDKNAIGFISLAAVNQDVKALKIDGVLPAVDNVKNGSYKISRPFIYL

TRTASDNKIADSFIEFVLSDEGQNILGNEGAISIK*
>Syntrophomonas_wolfei_subsp.metylbutyratica_JCM14075T_01767_Alkaline_phosphatase_synthesis_sensor_protein_PhoR
MPNSFRWKLTISYLLIIMLVLAVLGVFLSFSFKDFYLDNLKANLVYEGLLVAEMTRSYDGQEETGRFMQQIAELAGRDTDNRVTIVDQEGKVLGDSQFDPRQMEEHKNRPEIFRALKGETAVEMRYSETAAVQMLYVAVPFGNGEQKGVVRIAKALLEVESLYRQILHIILLVLVMTGILAFLLSLGIARWFSGPVAELTATVQDMARGNLKKRISFPARDELGLLAVAVNEMAENLEQNINQISAVN
ERLEALLANTVNGILMVDNKGIINYVNPVAINLLGFQDNVVGCRHLEVISNYYLLALIDQVREEGKTCSQEIILHKLGEKRVETTAIPLFIGVDAVAGGVLLVMNDISELKRLEAIRKDFVANVSHELKTPVASISGFAETLLNERELEEKRVREFSGIIYEEAQRLSRLIKRILELSRLESQSEELRKEWMDLNQTIENAIGLMEKRKKNSQVKLCFDKPENPVIIKGNAESIVQVMLNLLENAINFSPDGEMV
KISIEEEENSIRVKVADKGEGIPEKELPRVFERFYRVDKARSKKTGGTGLGLAIVKHLVENHGGQVGVESVPGKGSCFYFSLLRE*
>Syntrophomonas_wolfei_subsp.metylbutyratica_JCM14075T_01768_Alkaline_phosphatase_synthesis_transcriptional_regulatory_protein_PhoP
MRGKILVVDDEEALVRLLTYNLKKEGFITIPAYDGVEALQKITEEKPDLIILDLMLPGKDGLEICREVRAQGVKTPIIMLTARDEEIDKVVGLEMGADDYVTKPFSVRELSARVKAVLRRNRQMEEKADASSIITVGEFALKSESYEVYYKDKLLELTLKEYELLELLIKNRGRVLKRDYLLQVLWDYAEPVNTRVLDVHISKLREKIEPDSKNPRYIKTIRGVGYKFEVSGNA*
>Syntrophomonas_wolfei_subsp.metylbutyratica_JCM14075T_01769_hypothetical_protein
MQNNRNAYVLRRIISYCNDISEAIHRFGDDYTVFKQDSVYKNATALCVLQIGELTTHLTNDFKQSYTGIPWTQIKALRNVVAHNYGKIDDESLWETITSDIPKLKDYCSSIIKQFDSDA*
>Syntrophomonas_wolfei_subsp.metylbutyratica_JCM14075T_01770_putative_nucleotidyltransferases
MPVKKYTIEEIKAIVEPIARKYGVARVYLFGSYARGDIGENSDVDFRVDKGSLKGMFALCGLYTEIEEALQMKVDVLTTGSLEDDFLQKIKNEEILLYAE*
>Syntrophomonas_wolfei_subsp.metylbutyratica_JCM14075T_01771_hypothetical_protein
MKKKVLKPRIPVALNQHAGTHKDKKKEAKKYACRKGRQR*
>Syntrophomonas_wolfei_subsp.metylbutyratica_JCM14075T_01772_Threonine--tRNA_ligase_2
MAIARVETAGVEVRIKGIGSYNVLEGTSVAELLKRLEKKLLYPVMAIIIDNRLRDLNYRFTRSCAIELVDMNSEIGVRIYRRSVSFLLMKAARDLYPERVLTVKHSLSNGLLCEFLNEDSRADEIEALEKRMREMVEDNLPIRRMRINKERAQEIFREQGEEDKVKLLAYRDVDKVQVSELDGFYENFYIFMLPETGMLKKFRLFEYPPGMILQTPELSAPNSLRPYVEQKKLASIFQEAKKWAEMLETPHVA
ALNDIIEQGDINDIIRVNEALHEKKIAFIADQICSDEDIRLVLISGPSSSGKTTFAQRLLIQLRVNGKKPVVLSLDNYFVDRDLTPRDENNDYDFEALEALKVDLFNEHLGRLIKGDEVEIPIYSFTRGSCEERGMSMKVPAGEPIIIEGIHGLNERLTWSIPPEQKFKIYISALTQLNIDYSNRIPTTDSRLVRRIVRDARTRGYNALDTIKRWPSVRRGEERNIFSFQENADIMFNSSLVYELSVLKPYIEPLLRQI
GPEHPEYVVARYLLQFASYFKPIAPDAVPANSILREFIGGSCFKV*
>Syntrophomonas_wolfei_subsp.metylbutyratica_JCM14075T_01773_Soluble_lytic_murein_transglycosylase_precursor
MNVFTNLFKEISFMKLLKALEDGLASTGRALSGSKTPDNSTPPASSKSRPFSEIIKEASQAYGIQEEVISAVIKQESSYNPRAVSSCGAQGLMQLMPGTARSLGVRDAFNPEENIMAGTRYLKEKLNEFNGSLPLALAAYNAGSGAVKKHGGIPPYKETQAYVNKIIKSIDHLA*
>Syntrophomonas_wolfei_subsp.metylbutyratica_JCM14075T_01774_Poly-beta-hydroxybutyrate_polymerase
MERIFQAHSPGEAGEILQQNWEHWLMTTTRWREILTYDPRPATGCSPKETLWTKNKAQLYHYQSSSERRYRTPVLMIYALINRPYVLDLTPGSSLVEYLVNEGFDVYLLDWGEFEWEDRDISYADLVYDYIATAARKVARSAGSKEISIIGYCMGGTMSTLYAALFDRPVLKNLVYLAAPIDFNRAGTYDVWLKAPGYDPDRIADTMELVPKSFIDWGTRMLNPLANYWGTYTRLWKTLEEGKPV
HFWKVLNKWSSDNINFPGGAYRDWIRDFYQGNKLIKNQVVLRGKRVQLQRIKANILAMVGLRDHIVMPHQTAAALTYLGGNDKTYMEFPVGHGGLVLGETARMRVFPEIAYWLGERSEILSED*
>Syntrophomonas_wolfei_subsp.metylbutyratica_JCM14075T_01775_polyhydroxyalkanoic_acid_synthase,_PhaR_subunit
MSDEIKQDDSKVVMFNPFNVWKNVYFSSEAALTAITRKAVETTMFANSVDVILNSYLQYLKLQNEVSNYIAETLPFSSHRDTARVAKMLTALENKIDRLDEELFTDIYDLKEDTAAILERFAPVPASISDEADAGRVEQIDQHIKNTEALVEKITALELAIEEIKASLLELKAKPKPQATTRKSQKTATE*
>Syntrophomonas_wolfei_subsp.metylbutyratica_JCM14075T_01776_Inosine-5'-monophosphate_dehydrogenase
VKVKDRMTPNPYATTPDTCVGELWHLMQEKSLQRVPVLDRGKLIGIVTRRDFNARPELDLKRSSLATRFFPEEMEQKLSKLRVRDIIPLNQQLITIHQDAFIEQAAKLLRDNRISGLPVIDDEGRMVGIITQSDLSDAFLYILGVNCRGTRLCLRVDDDAQVLLRLGEVLSHFDIKIESMVRMEVKGEKSLLIIRLDTVDSRSIIEEIKAAGFKVESVIIKQ*
>Syntrophomonas_wolfei_subsp.metylbutyratica_JCM14075T_01777_Endoglucanase_H_precursor
MRKNSCLFALFLLAIFLPPLFYPLDASAATTLIKNGAIAYQPEYNSYDDIKAIATLEPGINRLLDYSQAYSIDYPSHMNIDASLSQVKTSLWDDKTCIEIYRDDFSQTVHNSTGYQYYSNQFLNNRKDHKIQENRSINIAGAKAHLLRWQRSKLSRVVGDKNYYMSVEIPRNYKEVYTILIKSSSPLESFLPLVKSFTFIDKKGASQINTRFKKKDKAWNEETKAFYEQYFDPDASLQWGIFENSAPASMDYLN
KLEENLEYRFNFLVLYKTFDAPFPLEELQKAYEEQKYVELTLQTMWYNRDNSSVTYDILNGHYDNFFQDYAEKARQFGHPILFRLNNEMNGDWCVYSSYYSSKDSELFKEVWRYIYNIFQQNQVDNVIWVWNPHDLSFPGFMWNHYLNYFPGEEYVDIIGLTGYNTGTYHKGEIWRGFRQIYDPLYEEYCRYFDYPFMISEFACSSVGGNKALWIEEMFAQMGKYDRIKVAIWFNGVDFDLVGR
AARKYRLDEGPAIIEAFQNGLPSYKPDPKANKTQ*
>Syntrophomonas_wolfei_subsp.metylbutyratica_JCM14075T_01778_Phenylalanine--tRNA_ligase_beta_subunit
MGVSLNWLKKYVDIGWSPEELAQRLTMAGIAVEGWQEQGGDTVLELDLTPNRGDCLGLINLAREVSALNGQELRIPEIQIKPAAQKVEDYIKVEIAARDLCPRYAARLVKNVNIKESPEWMQEALINSGIRPINNIVDITNYVMLETNQPLHAFDYNLLGDEKRILVRRAGDGEKFTTLDGVERVLDSEMLVITDGRKAVALAGIMGGENSEIQDDTCDVLLESANFQDTSIRKTSKRLGLRSDSSIR
FEKGADIEGLIYALNRAAALIQDLAGGQVLEGICDIYPEPRTPITLTLRPERVNYLLGTDINPDEIKQYLQRLKFNLKEQDKQFLVDIPFYRPDIQMEVDLIEEVARLYGYGNIPAGLPGGESSGGLNPYQKFRQRLNEFMAQSLSEVVNYSFINPKDFDRLLLPADSPLRNVVKIANPLSEEQSVMRSLLLPGLLETIKRNLARKNQDLAFFEMGQVFIPTNELLPLEKLYLGAIVTGKSEPNWLKQQVEL
DFFFLKGIMEDLLQELGISDYEFKETQNPSYHPGRTASLEVKGQEIAVIGELHPLVLENYDIKERASAFELDVEKLFTLLGAASKMESISRYPAVERDIAILLDQEIKASRVLSVIKGMNNPLLYDLKLFDLYSGTQVTKGQKSVAFKAIFQSAERTLTDAEVNHSMDSILEQLKKQLGAVLR*
>Syntrophomonas_wolfei_subsp.metylbutyratica_JCM14075T_01779_Phenylalanine--tRNA_ligase_alpha_subunit
MLRKIEEIKNECQARLSQVSTMEELNEFRIKTLGRKGEVTLLLRGLKDLSGEEKAKAGRVANELKQNLEQMLREKIEAFKRDELEQRLLQEKIDVTLPGLPRQRGRQHPLTQITREIQGIFIGMGFTVAEGPEIELDYYNFEALNVPRDHPARDMQDSFYISEELLLRTHTSPVQVRTMEQMAPQIPIKIIVPGKVYRKDDDATHSPMFHQVEGLVIGQRITFAHLKGTLVRFAHQVFGEDVTVRYRP
SFFPFTEPSAEMDISCVMCQGEGCRVCSHTGWLEILGAGMVNPRVLQYGGYDPEEVTGFAFGMGIERIAMLKYGINDLRLFFENDKRFLAQF*
>Syntrophomonas_wolfei_subsp.metylbutyratica_JCM14075T_01781_23S_rRNA_(uridine(2479)-2'-O)-methyltransferase
LLKICSKDNKKIKYAARLKQKSFRNQEACFLIEGKKMLEEALKHPIKLLRQIFIEAELAPYETELSQQHPEWECYVVDKRLMKHISDTETPPGIVALCQIPSWSREEFLSKNAFLIFLDRIADPGNLGTIIRSGWALGADGILLSPDSVDPFSPKVVRASMGGIFNIPLLLDVSLDELKRLKQIGYQFFCTSMQARQEVFSLDFTGPSLLAIGSESQGIRSVIEEICDINCKIPIKPGVDSLNAAVACAIIINEAWKQR
QGFILS*
>Syntrophomonas_wolfei_subsp.metylbutyratica_JCM14075T_01782_50S_ribosomal_protein_L20
MPRVKGGVTSHKRHKKILKLAKGYRGSKSKLFRVANEQVMKSGQYAYIHRRLKKRDFRKLWIARINAAARNNGTTYSRMVHGLKVAGVDINRKLLAELAISDPQGFTQLAELAKRSVQ*
>Syntrophomonas_wolfei_subsp.metylbutyratica_JCM14075T_01783_Ribosomal_protein_A
MPKIKTHRGAAKRFKKTGTGKIKRSKAYASHLLGGKSPKRKRNLRKAGLVSEAETRGISRLIPY*
>Syntrophomonas_wolfei_subsp.metylbutyratica_JCM14075T_01784_Translation_initiation_factor_IF-3
LRLNEEIRVKEVRLISESGEQLGIVSIREAMDLAQEKELDLVEVAPSAKPPVCRLMDYGKYRFEQSKREKEARKKQRIISIKEVKMRPNIEEHDFQVKAKNARKFLSGGDKVKLTIMFRGREITHPDIGERLSLKLTEQLSDISTIEKMPKVEGRNMVTILVPKMEQEKNEKKDKKEEA*
>Syntrophomonas_wolfei_subsp.metylbutyratica_JCM14075T_01785_Threonine--tRNA_ligase_2
MISIRLKDGSQREYPEGISLLQVAEDISPKLAKNAMAAVFNGKISDLNSQVKEDGCLELLDFEDERAREVYRHSSAHVMAQAVKRLWPESKLAIGPAIDKGFYYDFDSGHTFTPEDFATIEEEMKRIIKADYPIVRKELSREEALQFFSERSEDYKLELIEDLPEDAVISTYQQGEFVDLCAGPHLPSTGRLKAIKVMSLAGAYWRGSEKNPMLQRVYATSFPKKSMLDDYLQKLEEAKKRDHRRLGRELG
LFVVLDEGPGFPFFLPKGMVLRNELENFWREEHRKAGYQEIRTPIILSRELWERSGHWDHYKDNMYFTTIDEGDYAIKPMNCPGGLLVYKQNLHSYKELPLRMGEMGLVHRHEMSGVLHGLMRVRAFTQDDAHIFMLPEQIIDEIKGVMDLVDRFYSLFGFPYHVELSTKPEKAMGSDEIWEATNALIKALEERGMEYKVNEGDGAFYGPKIDFHLQDSLDRTWQCGTIQLDFQMPEKFDL
TYVGEDGGKHRPVMIHRVIYGSIERFIGILTEHFGGAFPLWLAPVQVRVLPITQRSQDYARQVVTELEQAGIRVEADFRSEKIGYKIREGQLQKIPYLLVLGDREAEEGTVSVRHRKEGDLGAKLLEEFKQTIVKEIKDKVL*
>Syntrophomonas_wolfei_subsp.metylbutyratica_JCM14075T_01786_hypothetical_protein
MIWQIIAIPLISAGIGYMTNVVAIRLLFWPKKPIRIVYFELYGLLPKRQAEIASKLGQLVEEQLLSIDDLFERIDTPETYSRLSSELSHMVRGKLYALLPRIIPGKVSALIVDNLDRILQQETPAIFKQLLATGRDYLNEEVQIKQIVENRVMALDLDELEMMIRGVSGTEIRFIELLGGILGFIIGLVQLLIISLTA*
>Syntrophomonas_wolfei_subsp.metylbutyratica_JCM14075T_01787_putative_sporulation_protein_YtxC
MNYGFRIVTDSEIDNLLIKLEERLKWIKQKDFSFQVQSSYKMEHSTYIVDINLEGNKRDKLFRDEDIIHIFKHQIAEVLAEHIVYEWESKLLWKAINRKYRSALREDKTIIHNKASEFLCKCHYNESLNLLMAYGRKNRIAHRLFDQIENNKTLVIEGFINFCLPEYLAEIRFAVELASEELKSEKEYNEFVKLLRYFVETQMPRVLEVNLIITDKGRFYLWDENGIKIEDKYINYYLDDILQNEISLDDVLISILVTI
APRKIILHNTDELSCNEPVKMIKNVFQERIIACPGCKFCRHDENHLVPGT*
>Syntrophomonas_wolfei_subsp.metylbutyratica_JCM14075T_01788_Intracellular_serine_protease
MNRFSLYPYKIQEAQSQRGIPWNIKQVNAASLWPRSQGDGVVVAVVDSGLELEHPEIAGRVVSPRNFTAAGNSSNLHDEIGHGTHVAGIVAGKNCGVAPEARIMPLKVFGDREVDKNILEAFKFILQYNQNAGEKDRVLVVNCSFGSPLYNPLMAYYIRSLTNSGVAVVVAAGNEGDGTPDTQEIFSYPAYIYEVITTGATNQNGKAAGYSNSFDGIDLAAPGTNIYSAWPGGGYRLLSGTSMAAP
HVSGAYALLAALFRKREGRWPSTDEGENILFRHICQVPIDPLLVGRGILDLSWETTRWPLYRVELAAFYQREGAEEMARKAREKGFNVNIAKY*
>Syntrophomonas_wolfei_subsp.metylbutyratica_JCM14075T_01789_hypothetical_protein
VKGRLRAEDGRWNGRQMSGERGCSSVAGHQLSENERMKAEGRGRWQKGGIFIQANEY*
>Syntrophomonas_wolfei_subsp.metylbutyratica_JCM14075T_01790_4'-phosphopantetheinyl_transferase_sfp
MIKLYLMKISGLEPDDIKWKKHVSPKRYEKMLSYRQKKDRALSLGVELLLNAALQELYPGISCPVTWGEDENGKPYLPLDYPAIYFNLSHAEEFAVCALSDAALGVDIEYCADIDGDIARSYFLSQEYEYILGKPESECLQAFYDLWVLKESYMKACGAGFRLALDAFCLQMGDRITLIQDGQIQPCSFFLTQFENYKLAVCYQGNEDASITPLFCELQNLYLAPLQKVINIHWLV*
>Syntrophomonas_wolfei_subsp.metylbutyratica_JCM14075T_01791_Tyrocidine_synthase_III
MKTFKLSNAQARIIATELGNPGTTAYIVPISVSFAARDEAYIMDAIKAVLVGNINIRFTRDNNLDVVQYIAEADYSTIKYIEADTEDVEMIVKDSVAKGFFTIFDVPLYRFTIIKTPRKLILLIVLHHLICDGSSVYMLADRLQEAVRCLRSGGEYLFNEADYRLYIEREQQYLNSSEGKNDKAFWLEQFQDLSGYVQAIAVSEELSIGTRTIDLPEELSWGIKAACEKCPVQISPFVLASALLAVYLSRVNKAEGM
VLSTGYSGRNFGEDLNEAMGMFVNLLPQKYPYNPEMTFIQALENAKGILKGGLTHGRYPFNLYSEELKQNNIDAGMLLNYSIVSNSWKSDYIDVLELSHAEFPLLFRINPGRDDRDGLQRIRIEYRYDSFSDEKIDSLVDNLLTMLRDMVNNPEIACDEISIWGATEKRLIAAGAATPENSFAYQEAQKYFEKIFAGNEIDSNLIPDLPGEKAPPLLASYTLSGELPHIDQTTCLAAFAYTLAKYSNQEEVI
FCSVIEGKTLPVYTNIDEEQAAPEYLQQIKKYLARTQKYSFYPYTEIKKNFALSDSVDLLLFGESELNEPFKFSVKLSENGITGVYNANLYTKEESIERFLTSLQAVILAISEGQRLSEIEIISGKDLDLIKSFNDNNVPLDRELTIVDMLRAQAARTPERTAVVYGDNSYTYRELDEITDRIARFLTAKGMGRGQAVGVLIHRSELMAICSIGVLKSAAAYLPLDPNYPSERLEFMLNDAAAKILIVDDDLYDRVPN
YQGEIILSSSIWDLEDSKLAITAPRAQDLFILLYTSGSTGTPKGCMIEHRNLVNFCLWHQDYYAVTEEDKSAAYASYGFDACMMDLYPFLTRGACVHIIPEEMRLDLPALNDYFEKNGISIAFMTTQLGRQFALSMDNKSLRHLSTGGEKLVPCAPPAYNFYNLYGPTECTIISTAFLMDKEYANVPIGKPLSNTDLYILDKQGRPMPVGVPGELCISGYQVSRGYLGRDDLTAEQYVANPYKRAEGY
ERYYKTGDVCRWLPDGNIEFVGRRDFQVKIRGFRVELSEIEGKIREYPGILDATVVAYDEAGGGKYVAAYIVADHQVKIAELNDFIAQDLPAYMVPAVTMQLASIPLNQNGKVNKRALPEPKVTFAEAIPPQNTLQQRIFNCIKSITGNDEFGINTDIYLAGLTSIGAVKLNVVLAKEFGINIKSRDIKQHKTVEKLEQFILGNSQGEKSYEKLAEYPLTQTQIGIYLECLRDEKSTIYNIPMLFKLGEAVNA
ERLAAAIEKTIDAHPYMKCYLKTDANGDIKQLRNDDFEYEVKPIIIKTCAFEEMKNTLVQAFDFASAPLFRIVIYQGESGQYLFMDIHHIICDGSSTAILLNDINRAYKDEILAVEEYSSFDLSLDEIEARSGKEYSEARAYYDSLFKDVEINSLPARDVFNDSEQCQSWERVEKLPRQIDAFCQENNITPNALFTGAFAYLLGQFTNQDEALFTTIYNGRNEARKAHIVGMLVKTLPVYANLDQRQNVAVY
LQGMKEHLMELMSNDIYSFAEISRAYGIKADILFAYQGEEFTDFQIAGEACELMYLEAKDAKAPLSVDVFVEGEQYRFLSEYRSDWYSEEMIASFTDAYIETIKSFLEAETLADVNMCSEQALAFLEGFNQTAWPVRDIPAAHLFEEQAALHPDKVAVIAGDEQLTFSELNERANRVANSLIEKGIQSEQMVGIMLPRTVNVYVAIQGVVKSAAAFLPIDPDYPDDRIQYILEDSAAPYIITSEAIKSKGCSIC
SQGNYEVLSIEQLLDNENNKNPLVDIKPEHICYCIYTSGSTGKPKGVMIEHSNLTHYCNANPLNPEIMSYVNNASVSLALAAITFDVSILEQFVPLLNGITVCLANEEEIHNPLALSDLILKNKVDMMTCTPSFISNIVDMPEMRRALRQIKAFNVGAESFPAALYEQIKALGSNAAVFNGYGPTEATIGCTFCEVLGEKITIGKPMSNVQIYMINENHKILPAGAPGELVIAGAGVGRGYVNKPEMTAEKYI
SINGRRAYRSGDLARWNFNGEIEFHGRIDDQVKLRGLRVELGEIEKVINDYEGILSSIVVVKENQGGQFLCAYFTAQGIVDKAALTRHLADTLTYYMVPSILIQLDKLPLTNNGKVDKKALPEPDFIMVEREYVAPANKTEADLCEIFKSVLGLPQVGAVDNFFEIGGTSLSASRVAMQCMSRGLKIVYADIFKFPTPRDLAAICADTGAAEIEADKEDIISAYDYKKLAAALAPNDLRYIDEVEYTPIGNIL
LTGATGFLGTHVLKEFLDNYSGKVYCFIRQGQRKSLEEHLKTMLMYYFENTYEDLFGDRIMVIDGDITDLDNVLALEQYNFDLLINCAANVKHFVADDSLEKVNVEGVKNLIKLGQKTKKRLIQVSTASIAGEGRDGRPPVDKKLLENEVYFGQALENAYIKSKFLAERAVLEAISEGLDAKIMRVGNLMSRYSDGEFQINFLTNSFMQQLRGYKIIGKFPITAMDAPAEFSPVDSTAAAILKLSGTNK
AFSVFHPYNNHLIYMSDVIYAINQYGFKIEVVSEDEFQKSLLEAMGDENRNEAISRLIAYLSNDADSKIYWLDASNKFTTEILYRLAYKWPIISDEYMKQSIKALDGLNFFDQ*
>Syntrophomonas_wolfei_subsp.metylbutyratica_JCM14075T_01792_hypothetical_protein
LTGLAKPIFFFEELEKPLKTNLAFKPGKCVRLMYVSTDENYLRRGIARKLGESMIKRAIDLSYELMIADATNINSAGMFEKLGFRREKVLPYKLVEQDGLKVFGNVEGECILYCRYLQGVKGTVLLTM*
>Syntrophomonas_wolfei_subsp.metylbutyratica_JCM14075T_01793_hypothetical_protein
VYIKIITSTIDMLEEIVAIQTAAFSEREPMTMSLNFTKEEMYDYFLKIIPESINAGLTMAAVDEDTGAVAAVVICFDAFAVFPNIEWTPKEIDGISKTNIFF*
>Syntrophomonas_wolfei_subsp.metylbutyratica_JCM14075T_01794_transcriptional_regulator_MalT
LLTAIARASIPRLHILIISRSYPEIPVDELELKGLCKEISQNLFEFTQEETVELFAQNGFQLSAKEQILLCNNTDGWVAAIYLASTIKTKGNCCRW*
>Syntrophomonas_wolfei_subsp.metylbutyratica_JCM14075T_01795_ATP-dependent_transcriptional_regulator
VANGCGTGFEHTARAEYCLETGDLSNAELFAYKAIFKAKTKNQLCLVICGNLCLVRLALLNGKPHKAFSLLNKLRTEVETAAHPVLFNSVDIAAAYIYGILDKLEQIPYWLQDEDFSRCNLFYQGIGINYTAVGQAAVLRKSYAELEVIVETIREIYKPNNHIFGLIYVGIYDAIAKKHLYGLEKAGQALLPAITMAAADAIITPFAENLPELYPVLQSMPPQEHRSWINQVLKLGERFMQSWKIINEQEHIP
PLTEREREVLTLLVKAAPKKKLP*
>Syntrophomonas_wolfei_subsp.metylbutyratica_JCM14075T_01796_mRNA_interferase_YafQ
MLKIRYSSRFKKDFKAIVKRGYDVKLLEEVLNLLVQEKALPQKYLDHPLTGNYAGHRECHITPDWLLIYKIEKDILILSLTRTGTHSDLF*
>Syntrophomonas_wolfei_subsp.metylbutyratica_JCM14075T_01797_Antitoxin_DinJ
MAKTTNLNIRVDEELKREAEAIFNELGFNMSTAMNIFLRYSVRYGGIPFDLRIEKPNKETMAAIDDVNNNRNMSKTFDSVSALMEDLNA*
>Syntrophomonas_wolfei_subsp.metylbutyratica_JCM14075T_01799_Magnesium_transporter_MgtE
LPAKEVCLPLRPILPYSRFFNLHFHYCLSIAATGEMEVYMEELREQISNLINQQLWNQLRQLAWDDYLIPDVASLLIGLNKADRVILFRLLPRPVATAVFSYLEKEDRNALLKDLTNEETRYLLTNLPPDDRTTLFEELPGQVTQRLLNLLNPDDLKEARLLLGYPEESVGRLMTPDYVAVRPQWTIQEAINHIRLKARNSETLHTIYVIDESWKLLDSLELSLLILANPQETVEAIMSKSFISLSAFDDRE
MAVKVMQKYDVFSLPVVDSDGILLGLVTFDDVMDVAEEEATEDFHKTAAVTPLKASYQESSIKDLVGKRMGWLIALVFVSLLSSGVIALYEQTLQTVIVLTVFIPLLLGSGGNAGAQASTLMVRALATSDVTADEWIRVFFKELMVGLLLGVFMGIISSIFGSMRGGLQIGLIVGLTLMLTIIIAANLIGVMLPFILTKMKLDPAVASNPLITSITDVMGLLIYFAIATMVLGPL*
>Syntrophomonas_wolfei_subsp.metylbutyratica_JCM14075T_01800_hypothetical_protein
MRLIWKHLSSGLIYLRSFRLIPGLVGTIIPFFWQLVNLYGTLPAVIIIAGVFQMLIVTMAAIIYPFFFFKWSFIEIYSLAAIFMLLAVISWQFINTGVNRRAGFKLVKLQFSSRAALLLLGLLLGNRWLSLSISPRTTFWDLHLKPQLAGQLRYWSREQIVAAISHDYRQALNLMEDAIFFGCSPGSFKKLINAAGLKESQYIIMETIPPKHAEIFGLRRPFYFYVISIRNQTGQ*
>Syntrophomonas_wolfei_subsp.metylbutyratica_JCM14075T_01801_transcriptional_regulator_MalT
MATGKSNSMELHYYSDRLKRKLEQMRLVPVTIVEAPSGYGKTTAIRDFLQNSLPQGTPVCWFTAMDEKTSAGFRRLCREIEKIDSRASERLLKIDFPNAANIGEASEALRSVQCKHETYLVIDNFQFLQDALPPAFFLALLEHGGQGLHVIIATQMLKRDTLAIVASHGVLHITTADMRLSAEDISRYYALANVKITPEDANEIERYTEGWIIAIYLQLSTFLETGTFSDTPGVLALMEHLVWDKLTEAQ
QTFLLYLSPFEMVSIQQACSLIGCDTLPEYALESFASPFIRYDPVERQYELHSILTELLIQKCGERGPAFERQCLLQAGDFCRDEGQTARALGFYMQIRDYERMLSLDLSEMNLEIIGGVPFTELASDIAQNCPAEIKKKYILNMLRIAWALFMVGMKEQFEILMEELHTIPELLENSNLLREWLLLSSYRSFPYLAEMTAIIRQAAPLFKNQCSQVILPTALWCFGNYSPLTEFYTTSGEADHEADALEEYISI
YSRLTNGHGSGVDALFRAELAYQRGNLNDAEILAYKAAFLAESKQQSVVLLGAAILLAHVALHKADIVGWERAIDSMERAASFDRQDTLVIRSLLDIIRGVLLNEFQDHMSIADWLQNADFAERRLPPAMIQIGLFVHLGFLMHQGEFARVIGTAQAIRPEDGLLSPYRDLFACFLMAISHMALDNRTQAAALVEDAARKVLPDGLVSPFASYSQLLGNLVDEVIARDYPALLEKFKTVRKRFSEGWF
TLREAFFSGELPPDLTEREYEVAKLAAAGLRNSEIAEKLMITESTVRAHMRAIFQKLQIDRRTRLAEKLKLNIQR*
>Syntrophomonas_wolfei_subsp.metylbutyratica_JCM14075T_01802_hypothetical_protein
VNYIKTVLSMKDYRLFMEMESGSTVTVDLSGKLHTMKYGELADEAFFKTATTDGDYVIWGGGRLRLTVNELMEVVLLG*
>Syntrophomonas_wolfei_subsp.metylbutyratica_JCM14075T_01803_hypothetical_protein
MKDQLECTRVLERTKHINVEDTRTRLALAGFKSILGILVFVLLLLPGFSQVQPETVGQTVYSVCSGNTLLGYNGPVSSLNSQGGNTGAIVVPIALGQTINGNIDKSDYFYPTAGGGIWQDEYQLVLHQDTKIALELHAPNGGYTRTLLYCDHATLPNGDKGTYSAFSGGNLVGNNIYRRPAAGFGDLSLGSNIYFPGLYRIVVFSDTPGPYTLKVLSPVAGTHINFTVENGNANAPQPVISDSNGH
WTRDFDPASTYRITAEKTGYSVSPSSQVFDGPSDELDFELFSLASIPEITYLTLNKNTGVAGEIQTVNATIHATGAVDGTPVSICLLNAQGSNPLAPLFYPSPGDLDNKDLKKFWENLKEDPYQEITYKSIAGNGTIINGQATVTMVIPDKLAPGYYQVRAQLAGSASIMSKNYNIATATDVPLISSISLTPAQHNTGQSQYIDVSVETYNVKNGTSVTATLVNNDGSPLATPVDSVTGVISDNQVQLGL
YLPSGVPAGHYSVKVTISLAEILTGFKPYHIVNYPDAEAVDAAIEALTWESIKGGNTSPDNITTNLKSPLPTTGPNGTSISWSANPVDWINTTTGAVTRPTTGQGHQTIVLTATISKGMFSGIKNFTLTIIDPTMVVTPSASLASGTYGGTQNITLSTASEGATIYYTTDNSKPTTASTPYTNMIEVAYNMTIKAIAIKEGMENSPVSTFEYVIFAGGDGSIDNPYQVATAEQLNNVRYHLDKHFQQTADI
DLSSYYTGSGWEPIGSDGSPFTSIFNGNGKIISNLTINRSTENYTGLFGFTGTTAKIRNVKLENNNVIGQQNTGALVGYNQGTIFNSYATGNVTSTDDCVGGLIGCNQQNSSITDSYAIGNVVGGQCTGGLVGYNQGTITRSYATGEITGRGKVAGLVGSNSSGTITNSYATGAATGTENYVGGLVGWTYCGTITNAYSTGAVTGTGGVGGLVGWYDSKFSAPYVTNSYWDINTSGITSSAGGNG
KTTADMTGGNYGAHIYFQWNFGGIWKYGGNNGYPSLINNPPVGPDKTLISIITPSAITGVANGTAKEAAALGLPKDVKRMITDRGNVQADVTWYVDACSYEPAVTTEQTFTVNGTVTLPTGVINPGNKPLTTTIRVTVKAQAGTVTNITAVSNVEVAYGTAQAAALAALAATTTITDSNNNTHTVSLSWSIASYDSNTAGNYTATGTFTLPTGVDQSDPQTELKVTAIVTVKTQGVEPNPTITG
ITSVSNVEVAYGTAEAIALAALAATTTITDSNNNTHTVSLSWSIASYDSNNAGNYTATGTFTLPAGVDQSDPQTELKITATVRVNAAVSTDKTLVSITAPSAITGIANGTANTAEALGLPTKVTLITNDGNVQANVTWYVAACSYDPAVTTRQTFTVNGNVTLPAGVVNPNSVELRTSISVTVNTAASPPPPWYPVSGVTLNKSSLTLEVNGPAQKLAATVQPSYASYPYVYWNSSDTAVARVDNT
GLVTPVAPGQAVITATTAEGGKTASCTVTVMAS*
>Syntrophomonas_wolfei_subsp.metylbutyratica_JCM14075T_01804_hypothetical_protein
LHDVPVSISIPPEVEATIEVTPGSTMPQVNINSSTALGIVEIQIPEGTIASGPDGWDGTFKLPTISNQPSVNINGASQVNTVIVLGLEDEQISFSQAVRILIPGQAQQQVGYIRNGVFTPITRILSADRQDVADSEIPADGDGKIDVDSNLAVWTKHFTEFVIYTPISNPKPASYTVSLSSNYTKAGTVSGDGTFYGGSLITVRAKAQSGYVFDYWSENGSMLSRNSVYSFNLGESSRQLQANFVREFQELSIT
DATKPRIITFSHPVLPDEQNLGNIYVATDINGDNKVEGVIVAGVPGNNCQISVKPPDGQWLSGASYYLILEPGLQSALNKTLGIRTRMKFTVE*
>Syntrophomonas_wolfei_subsp.metylbutyratica_JCM14075T_01805_hypothetical_protein
MRGNENSRKWLSSIVIGIYMLSFILAQAPVMYAWISSAVDGLKVSGRD*
>Syntrophomonas_wolfei_subsp.metylbutyratica_JCM14075T_01806_Endoglucanase_precursor
MSLLKVRHGEKWERGITYCLLVCMVFSCIIAMPSASYAADDNAWEQNGIGLSELTPEKVVVFAGNTMNASKMGFLKNYYYLSEENLNDLRQYDNGTRAYGYFGEEAWISNASYSSSDDHGAREWRYSCLTGIDLEKAFTGLGVTPSTGLSLKVKSTDGTSFVLENAFDQSRYYFNPQGEQSTTAVAPILALKKSTVSSTIAPSSEATVVVPTTAADTYDPLFAYGQKEVNEDNNCHFVKYVNSIVA
GTEDIALTVIEDGKSENQISISEIIRKGVYKTNYSYSSGGSLHNHSVTGIPLAALLNAMGVCVADGQSIQPATSDNYAANAISQADIDKWFVAYDALENGSQVTNNTILRLYGPGQFGNQVLVKNLSTLTITGQSAAVSKTALTDAIKAANTKYMNAAEGIEAGQYAVGSKDAFKTAIDTAQAVADNAEATQAQVNQAVTDLKTAEGVFDAAKVTAVSKTALTDAIKAANTKYTNAVEGSAAGQYA
LGSKAALKTAIDTAQAVADNTEATQAQVNQAVTDLKAAEGIFDAAKVTDGKAAGGDTENAVFYIAVKESADSTAKYYYYTKAELEAYETTEDFLYNDHSVIKTVTAKGALLSNLLLYNLNGVTITGDMIIQYAESDGYHADQATSIDNSSYKDKIAWLTAPHYNGSNTIEAARTVIAYGIHEEYLNPDENNINDAPGVFKDADKGSGYLRAYRETGSGKNDANIGGANATVIKYLMGVVVSYDGAQ
FTGKDGYTLKAVSAKNPDLAIVADQKVTGLMPGMEFAVKAPRVVNSVLASGEPAFQKITVGTGASQTVSFKYTENPYFYVTNGSSTTNYTYTDLVKTSVQLPSKDENQAPYGYSRPMYYRYNGAWLADIIGSALMNDTAVTGFNIIGKDGKKTFVSKADVAQYFVAYNNTQSKTSTNIPEGKRVTRTYEDAKVIIPGQGENITGSAADDYTTAGKDVGVLVAAAEGVEASSAGGSGGSGGGGGSSG
GGGSVSTSSGGQQVQSSGATVTKNGVVVVIPSGAVSSGIYVHVAIVSSSGLTVDSGSKLISDVYEITKDKEGNFAKIDITVTLPFTKSKADQSKDELTICFWNGSKWVELDNVKVDWSTGTVSGTINHFTKFAVLAKKKESAAVPMISQPAANQTVLTDINGHWAAESIRNMLGIGILSGYPDGTFKPDQTITRAEFASMLAKAFKLSASGNKVFDDTAGHWAQDYIAAASDAGIVSGFNNNNFKPDEPI
TREQMAVMVTKAAKAGKPTQAISFKDKNDISVWAQEAVASAVSAQLLSGYPDQTFRPKNNASRAEAASVLTKALNLK*
>Syntrophomonas_wolfei_subsp.metylbutyratica_JCM14075T_01807_putative_polysaccharide_deacetylase_PdaA_precursor
MFFVLRKRSVGVFLGGILFLGISLGAYNLALHPNMHNWKAQKLVITDVKTTEKAVALTFDDGPDPVNTPVVLDVLKKHQAQATFFVVGIRAERQPELLQRMASEGHEIGNHSYSHADFNHKKKEFYQEEIRKTNASIESISGQKPRLFRPPGGYLSYDMVEVTQKEKVTIAYWTYQQDSKDWQGNSSSQISAHIIKNIKPGQIIILHDGYKNGLETARALDKLIPELKQQGYRFLTMSDLMLLEKQE
*
>Syntrophomonas_wolfei_subsp.metylbutyratica_JCM14075T_01808_putative_polysaccharide_deacetylase_PdaA_precursor
MPKVFGFYKIRARTRLTLTALITLIILVSGALYVQAYNKNKPVYEVPTEKKMAALTFDISWGNKTPLPVIEILKEADITCTFFLSGPWVKENPEAVQRIKKDGHELASHGYRHINYSTLSKSEIKEEVMKAHKNIHEVGGVNANLIRTPNGDYNDHVIEAIHEINYEAIQWSVDSLDWMNPGTATIIERVKKKVHPGAIILMHASDTCKQTTDALPVVIKNLKEQGYELVTVSTLLKEMPPENN*
>Syntrophomonas_wolfei_subsp.metylbutyratica_JCM14075T_01809_hypothetical_protein
MLSRDGVIARYILAFCFFFSSGSEMLTGWLATLSGILGCVELATALLKYSPLYELWAVIKNHYQQNKALE*
>Syntrophomonas_wolfei_subsp.metylbutyratica_JCM14075T_01810_hydroxyacylglutathione_hydrolase
MIIALTSVIKLIRPEARAVFPYSNSLYIDDEMQTLIDAGSGGRAFAEVPVDNIELLLLSHNHFDHVNGISFFPQARIMAGREEALTYQDQELYLKFSGFYDWERLMGEMDRRLLSGTMPLPEDVPAQRGFQEVQLDGLIKDGDVFELGVTSLRAIHLPGHSPGHYAFYCEKEGILFSADLDLAPDGPWYGGGLSDFDELEKSIKKLIEINPRILVTSHRRVFDAAQDNIPALLQTYLDVPLKKEEQSLVYL
AEPRTIDDIARQDFIEGFKGKTPFAVFFTKMMIDKHLKRLEKAGKVKKIDDTHYIRIG*
>Syntrophomonas_wolfei_subsp.metylbutyratica_JCM14075T_01811_hypothetical_protein
MVYCITHNSNPGKAYVDPSANIYGGDMVKKRWGKGEPVNLSDLLKITPVANEILQLSEEMDMTCTDVMWIVAQIIDNNEEINLHHIRHNHDDDELVLDEEWSEADLESLEEEIDQALYTVSVKVNSQSRFPEVAFKAGINNREDINYFFDMVLEMLEKLE*
>Syntrophomonas_wolfei_subsp.metylbutyratica_JCM14075T_01812_hypothetical_protein
LKFIGFENLRLTDRNRAGGDAVFELNGEKAKAEFSFYLQLNECLSIRLNRHDKKLSTAELEKFISDNRLELKKMVQPDVERLRRQKRKELYGVEEFNNPHKNLP*
>Syntrophomonas_wolfei_subsp.metylbutyratica_JCM14075T_01813_hypothetical_protein
MKHTAIGSGDVSCFLFILSILALLRNRKIYNNNDFYILAMHFAGIMLLDVETNIYNEAKYFSASLSPGY*
>Syntrophomonas_wolfei_subsp.metylbutyratica_JCM14075T_01814_Putative_K(+)-stimulated_pyrophosphate-energized_sodium_pump
VTLDSVTLFPIGKQVKAAKAMFSALLSLLMLLLWPAAAFAGEANLVIPTLSPAQNNLLIVGIVVCVLGMLFGLYQFTQVKKLPAHQSMLDVASIIYETCKTYLIQQGKFLILLFTFIGVCIAFYFGFLQKTPLSGVLLILAWTVIGILGSYSVAWYGIRMNTLANSRTAFAGLEKKPLKVLNIALDAGMSIGVLLVCVELIMMLIILLFVPRELAGASFIGFAIGESLGASALRIAGGIFTKIADIGSDLMKIVFGIK
EDDPRNPGVIADCTGDNAGDSVGPTADGFETYGVTGVALVTFIVLAVAPALQTALLTWIFVMRVLMVITSIASFFINRAFSQAKYAGKEEFDFEQPLTSLVWITSLLSIAVTFAVSYFLLGPSSDAPANLQSLWFILAAIISVGTLGAALIPEFTKIFTSPKSDHVAEVVKASREGGPSLNILSGLVAGNFSAFWIGMVFLALMFTAYYFSGFGLAEIMIYPSIFAFGLVAFGMLGMGPVTIAVDSYGPVTDNA
QSIYELSLIETIPNISKQIQKDFGFKPDFDKAKYYLEANDGAGNTFKATAKPVLIGTAVVGATTMIFSLILVIQSVLGVEPESILTILNPYTILGFICGGAVIYWFTGASTQAVTTGAYRAVEYIKKNIQLDEKASQKASTEKSKEVVKICTQYAQAGMINIFIAIFSFALAFACLSAPRTYEAPVALFVSYLISIALFGLFQAVFMANAGGCWDNAKKVVEVDMQEKGTPLHDATVVGDTVGDPFKDTSSVAL
NPIIKFTTLFGLLAMEIAISESFRDIAPVVGGIFLIIALFFVWRSFYGMRIPTE*
>Syntrophomonas_wolfei_subsp.metylbutyratica_JCM14075T_01815_Phd_YefM
MPKIIPIRDLKNTSEISQMCHASDEPIFVTKNGYGDMVIMSIKLYEEKMFMLDVYQKLRDAEQQLAEGEILEGDTSLKKIREKYNV*
>Syntrophomonas_wolfei_subsp.metylbutyratica_JCM14075T_01816_MutS2_protein
VNQSTLEVLEFDKILEQVKTYALTEIARNRLQQLQPSARCEQVQNWMDETTEARAMLEVNASVPIPIMDRMSEVMAKLGKGFVLTPAELEACQNLLEGVKRLGKYMEALETTAPRLASYASSMFVLQELQEEIAGAIVNQQVADRASAALLKIRKRRLIIEERIRQKMQEVLRSPAYASMLQDTVIQERNGRLVVPVRRQYRKAFAGTVVDTSSTGSTVFIEPAAVTRWLDELGQLKAEEKKEVYRIL
THLTGQVEKHARELSVNIETIAHYDFVFAKAKYSRALDMRSVKLNTRGYCLLKQGRHPLLGAGAVPLDFYIGESNRALLITGPNTGGKTVALKTVGLLTLMAQAGLHVPLEAGSEIAVFVDILADIGDGQSMEQSLSTFSAHVLNILTMLNCADSRTLVLLDELGAGTDPAEGRGFAIAVLEEIFARGATIVATTHFGEIKEFAAYTEGFVNGCMEFDPDSLQPLYRLKIGTWGDSHAFLIALRLGM
DPDLIARAHEISYGEQISYRPEPLPRTEKLMDSKAVEEHRETARALEKAEKDQERLQRQRRYEKKNLKIGDRVYISTMQRTGVVCEEEDEKGDLVVLVLGKKVRINQKRLTLHIDRAELYPENYDLDIVLESKEDRKKRKIMRKRHVEGLVIEHHDE*
>Syntrophomonas_wolfei_subsp.metylbutyratica_JCM14075T_01817_HTH-type_transcriptional_activator_TipA
VLNAAFQAGDPAGDLAQEAVKLHRQWLSYYWHSYSKEAHIGLAQMYVDDERFRAYYDQKQPGTAAFLRDAVLVFAGKKK*
>Syntrophomonas_wolfei_subsp.metylbutyratica_JCM14075T_01818_Multidrug_transporter_activation_protein
LWEHHAKLLAKKEQLDLLIANVEKTIALKEGRATMTDQEKFVGFKQKLIEENEEKYGQEIRNKYGDQTVDQANRKLMNMSPEQYEEVARWARSC*
>Syntrophomonas_wolfei_subsp.metylbutyratica_JCM14075T_01819_(S)-limonene_6-monooxygenase
MANSIQIESTNLRNEWVSFIKNGTMPKAVRAPVLRSWIRCKKLGIDPEGGKGNNVLSIVELNRCLKDNALLIGIVRDLLQGVSWAAKSSGYIFYLTDKYANLLYIAGDTRVSREYEELINFGVGVNWGEAAVGTTAVSLALDENSPIFWISEEKYCVVLKDKACASVPIQAKDGSVVGILGTATDTDGLLKSACPVFDFLLMAAAAVQNYYQRFEVENVYYRIEKSLSLVLGEIPEGVLITDQGGMIVEVN
KSFGALLGLDISEAIGKPVDSVFPQVSLPLSRDLTNGVQLFKSSYGENKHLNTEGLVSRKLGFKDASGLKEYSIHILQKKPEKMKNVSGSRPFQAKIFDDIIGESREIKQTKSQALIMAKAPYEVLIEGKTGTGKEMFAQAIHNSSDRAEAPFVSINCGAIPDELMESELFGYEGGAFTGARSHGNPGKLELAEGGTLFLDEIGEMPSNLQVKLLRVVQEKQYMRLGGLRPVSTNVRIICATNRDMESMVE
AGAFREDLFWRLNVLALHVPSLYERKTDIPILIDYFLKQHSAEQGLPYTLSDEARNLMMTYSWPGNVRELQNALKCAVVYAEDGLILPCHLPQHIVNDRNIHHEEDSNMPLQNAEKQVINQALSSCGGNLSQAARMLGISRVTLYKKIKQYCLVR*
>Syntrophomonas_wolfei_subsp.metylbutyratica_JCM14075T_01820_hypothetical_protein
MKEEQSKPAPEYLLGSPEQQSWMKKYFFSPRGLGFLEPGIVGLDDAKMSAYNVAKSSYQIRPDVFNMNYLSKTVGLGKEEIIQRMQRMYEQRTIVFVMNPAVSVYGWGLYYWMVKLNENTPPEAKAELSEWFQNKDDICTGYECQGDFDFYNGNHMRVIDNLLYDVLEPWKNRPEVKYVHLCPIRRDLRESHVNQFDCPGDGYREVLWGPGQIEKLAQVQDKLDLPDLKIIQALNAKRPME
DFFDFKVLAEVSGLDEKAMLDGIKELVEVKRIIVPLFHLNFMKLNLTNRMFVIRLFQNIPCCRKAEISDELSAIKEFNTVKEFSDSFYDIILWAYNEITDIPALRAKLNSYSEIEEIKEADMTRMYRRWVCRLDDQNGYWEECVFTDDFLQDRTQPGGPECTIRKGAK*
>Syntrophomonas_wolfei_subsp.metylbutyratica_JCM14075T_01821_succinyl-CoA_synthetase_subunit_alpha
MKIDAPRKYVIDNLKHFLNPSSIAVVGASRYPAKVGYKVIQGLKDWGYRGEIYPVNPRADKVHGLTAYKTITDIPGPVDLVFVAVPAHLVKMTLEHAVAKKAKIVVIATSAFKEIGRGDLQDRLTQYCRDNKLPLFGPNLVGMGSPYLNFNCGFIPYLPIKGPVAMISQSGANLLAALGNSTTMHFGMSFFVGLGNKADVDFSELIEYGNTDPHTKSLAIYIEGLDSPTAFVKACTSVVPNKPIVVIKV
GGSEIGVKAAFAHTASENEGTSDAFYDQLFEQCGVIRATTFQEFLDISLALGLQPELKGDCVAMITNGGGSGLLSCDHFERLGQPMQELSSISPDLGLRIRTYMPMFGSPLNPVDISGTASPVHYKGALRQAMRDPNVDMVYCSICPTAVTDVPGVVDVAIEMYEAYKPLGKPFVMECQGGAECLEAIIRLRNHGIPAYATPEQAVNAIVALRKYSKIKEKFSKKK*
>Syntrophomonas_wolfei_subsp.metylbutyratica_JCM14075T_01822_Putative_K(+)-stimulated_pyrophosphate-energized_sodium_pump
MEVVSRNKIRTMLGSLLSFVLLLLSPAMAVASEANLKIPNLSADQNSLLYAGIVVCALGMLFGLYQFMKVKKLVAHQCMLDVAQTIYETCKTYLIQQGKFIAILFAFISVCIAFYFGFLQEMPIASVLFILMWTVIGILGSYSVAWYGIRMNTLANSRVAFTSLERKPLKLLNIALDAGMSIGVLLVCVELIMMLIILCFVPRELAGASFIGFAVGESLGASALRIAGGIFTKIADIGADLMKIVFGIKEDDPRNP
GVIADCTGDNAGDSVGPTADGFETYGVTGVALVTFIVLAVAPALQTTLLTWIFAIRVLMVITSIFSFYINRVFSQAKYGSKDDFDFEAPLSSLVWITSIISIAVTFGVSYYILGPGAGTETALTPIWSVLALIISFGTLAAALIPEFTKIFTSPKSLHVAEVVKASREGGPSLNILSGLVAGNFSSFWIGLVFVGIMFGAYYASIPISHYIMSYSSIFAFGLVAFGMLGMGPVTIAVDSYGPVTDNAQSIYELSLIEEIKDI
DKEIQKEFGFKPDFDKGKYYLEANDGAGNTFKATAKPVLIGTAVVGATTMIFSLILVIKNVLGVEPATILTILLNPYTILGFLCGGAVIYWFTGSSTQAVTTGAYRAVEYIKNNINLEEAANVKANTEKSKEVVKICTQYAQSGMFNIFIAIFSFALAFACLSAPQAGNSQPVAFFVAYLISIAVFGLFQAVFMANAGGCWDNAKKVVEVDLKEKGTPLHEASVVGDTVGDPFKDTSSVALNPIIKFTTLFG
LLAMEIAISETFRDIAPYVGGAFLTIAIIFVWRTFYGMRIPSGK*
>Syntrophomonas_wolfei_subsp.metylbutyratica_JCM14075T_01823_Hydrogenase_expression/formation_protein_HypE
MKIDRVLLAHGSGGLMSQQLVEEIFKKEWNNSYLEPMLDGALLDLPAGRVVMTTDSFVITPLFFPGGDIGKLSICGTVNDLVACGAKPLFLSTALIIEEGLPMSDLIQIAQSMARTAREAGVQLVTGDTKVVEKGSADGIFINTTGIGVIAEGIDYRPQRIQPGDLVVVSGTMGDHGLAIMARREGLSFSTPVNSDCAPLFRIGQVLEAFGGNVRCMRDPTRGGLATVLNELARQSGRGIKVVEEDIP
VNPAVAGACDMLGMDPLYMANEGKMVIVLAPERAAEILAELQKIDIAAEAAIIGEVRAEPAGLVLIETELGAERILGILEGEHVPRIC*
>Syntrophomonas_wolfei_subsp.metylbutyratica_JCM14075T_01824_Hydrogenase_expression/formation_protein_HypD
MNDKTYMKELLQEIQSYKQEVSLMEVCGTHTMAIARSGIREMVPANIKLLSGPGCPVCVTSQGDIDAVIKLCREPGIILCTFGDMMRVPGSESSLQEERSRGADIRVVYSPLDALEIARKNPEREVVFLGIGFETTAPTIAVSLEQAERERINNFSLLSYHKLVPPALEVIFSDPDIRVDGLICPGHVSAVIGLKPYELLAAKYHKPCVVTGFETVDILEGIIMLLHQMSQGEARADIQYRRVVRKEGNPVAV
ELLERIFQPEEARWRGLGPIPGSGLKLRSAYEFLDAAKKFQLPQIADLPIKGCSCGEVLTGKISPRECLLFGRGCTPLKPVGPCMVSHEGACAAYYRYTPAKERN*
>Syntrophomonas_wolfei_subsp.metylbutyratica_JCM14075T_01825_Hydrogenase_isoenzymes_formation_protein_HypC
MCLGVPGKILEIKEHDVAQVDVDGNQLDISIRLTPEVQKGQFVLVHAGFAMEIIDESRAVETMDLLKELQKYRELGI*
>Syntrophomonas_wolfei_subsp.metylbutyratica_JCM14075T_01826_Carbamoyltransferase_HypF
MEGTRQEASAVLAYEMKIFGIVQGVGFRPHVFRLAAELGIRGWILNSSAGVFLEVEAEEESLREFTRRLLAEPPPLALIRDYQLREIEAQGFSDFQIRESESQEELLVMVSPDIAVCHDCRREVMDPVDRRYAYPFINCTNCGPRFTIIKDLPYDRAQTTMAEFTMCPECDREYHNPLHRRFHAQPNACPLCGPHISMLDSNGNEVAQGAVELLKKGCILAVKGLGGFHLAVDARNREAVSCLRQRKH
RDAKPFALMARDLETARKYCHISQLEEQHLTSGRAPIVVMESRTELDLPQDLIHPGLNTLGIMLPYTPLHYLLFDDELELLVMTSANISDEPLIYENETAQKKLAGIADYFLLHNRDIFNPCDDSVMRVTALNTPQLFRRARGYVPLGIRIPSSSSPVLAVGGEMKNTFCITRQGEAYLSQHWGDLNHYLNFRNFQDGIERFKNMLAVEPQVIAHDLHPNYQTTRWAREQEHMEKIAIQHHWAHM
ASAMAENQLQGEVLGLICDGTGWGTDGAVWGGEILQGDYRQFSRLGHLQYLPLPGGDLTARRPYRMALLYLLAAIGEKAAALSRDLIPAFPEEERELMLQRWQKGTRELQTSSCGRLFDAVAAVLGICIVNRYEGQAAIELEARADYTQRGSYSYALNDENGMLIMNLLPMWPELLADRKKGISVGGIAQKFHLTLVDVFTAALDRSREKSGLSRVVLSGGAFHNQILLFKLSEALKKAGFEVYH
QQQVPPGDGGIALGQAIIASEVKANVSGSTG*
>Syntrophomonas_wolfei_subsp.metylbutyratica_JCM14075T_01827_Hydrogenase_2_maturation_protease
LKKIMVVGIGNLLMQDEGVGVHAINQLAQMSFPQNVEIIDGGTHSYDLVEFFCQADNLIIIDAMKAGGEPGTIYRAPFDELGLKPDENITSLHEMHFVEAMYLVRLLGYDPEVMVFGVEPQNIGLSLDLTPQVAEKVPRLVELVKEEVERLLQ*
>Syntrophomonas_wolfei_subsp.metylbutyratica_JCM14075T_01828_putative_Ni/Fe-hydrogenase_B-type_cytochrome_subunit
MVKQMEDRHSIWIRIFHWTNMIAITMLCLTGFYIHAPESFRLFANMDNARMIHFGMAYLLCVGVLGRVYYAIVADDAKNIVFSVTKDTPKFPSMMKYYLFMADSHPYYGKYNPGQKMMYTGWLFMALIQIITGFVLYKPDMFMAVSGWLGGYMAVRVIHYIVTWLFVLSVLAHVYLDISEGVPVLKSMFTGKIPADFDHGTHEEHAPAPGESIGA*
>Syntrophomonas_wolfei_subsp.metylbutyratica_JCM14075T_01829_Periplasmic_[NiFeSe]_hydrogenase_large_subunit
LAALDYVKGPDIPPFIPRYEADYRLPPDVNQKAVETYLEALKMRRKAHEMTALWGGKMPHQQAITPGGVTEIPDAQKVIEFKYRLKEITAFIDNVYIPIVQAVAGAYSDLFDVGRGSMGALAYGAFPLEEGMDHVNKQKFFPSGVYIRGQYKELKPDYITEEVKYSWYEDDTGGNKPVEAVVTPNAEKKDAYSWIKAPRYNGEVVEVGPLARQWILKQKDVVALGEKAFSVMGRHFARAIEASAV
AHALDEWVGQVEPGKPVSTPHKIPGSAVGMGLTEAARGALGHWNVIKHQRTSVYNAIVPSTWNMSPRDAKGVMGTMEQALIGTAVKDPKNPVEIVRIVRSYDPCLACAIHIMTPDKKTVSKFAIN*
>Syntrophomonas_wolfei_subsp.metylbutyratica_JCM14075T_01830_Periplasmic_[NiFe]_hydrogenase_large_subunit_precursor
MADKIMIDPITRIEGHLAVEVEVEGGLVVDAKSMGMMFRGMELIMRGRDPRDAQQIMQRICGV*
>Syntrophomonas_wolfei_subsp.metylbutyratica_JCM14075T_01831_Periplasmic_[NiFeSe]_hydrogenase_small_subunit_precursor
MPTKLSRRDFIRLCAGGAAAISLSSYLAPFMAEAVAAGAPPVLWLQGGSCTGCSISILNTVSPDIEKVLTEVISLRYHPNISAAAGDLAIQEALYKTAEEFKGQFFLVVEGAVPVAADGMYCIIGEKDGQHLTFERAVKEIGSQAAAILNFGTCSAFGGIPATPPNPTKCKPVSEIIKTVPIVNVPGCPPHPDWMIGTIAHVLLYGDVPEVDSFGRPKMFFGGIIHDNCPRRQYFDNAIFAKNFGDPGCLLEI
GCKGPIAHCDSTTRLWNGGVNWCIKAGAPCIACTEPQFPGWPMYERIPEMPIAPAMTATIDQIGGVLGAAAVLGIGGHLAGNIATGRIGPKKNDAEKEGDN*
>Syntrophomonas_wolfei_subsp.metylbutyratica_JCM14075T_01832_Hydrogenase_isoenzymes_nickel_incorporation_protein_HypB
MVQIALGSNILGANELLARKNRKLFEQDGTLVINLMSSPGAGKTTILERSLEGITGKLKIGVIEGDLYTDQDAQRIGKKGVEVVQINTEGTCHLDAGMVNKALERLPASDYQLVFIENVGNLVCPAEFDLGEDFRVVVFSVAEGNDKLLKYPLIFREARVILLNKIDLLAYTDFSLEKFQEDLAQINPSAIVFLVSGRTGEGVKEWTEWLLEEVSKKQNRF*
>Syntrophomonas_wolfei_subsp.metylbutyratica_JCM14075T_01833_hydrogenase_nickel_incorporation_protein
VHEVTLIEGVMNTVRASALEHDIVRITRVKLVIGKLSMALPDSLQFAFQVLGEQDDLFRGAQLEIEEQEIQCRCSDCQQEFVVDKDYRFVCNFCNGVNVEIIRGRELYIDHYEGDKN*
>Syntrophomonas_wolfei_subsp.metylbutyratica_JCM14075T_01835_Nickel_import_ATP-binding_protein_NikO
LDKELFYLDRVSFKYQDFQPVFNNLSIIIQKGESVSLLGANGSGKSTLLKILCALVFPSEGIFRAFGQVINEEAMENEAFSKEYHRRLGFIFQNSDVQLFTTRVWDEIAFGPLQLGWEQDRVREMVDDVINLLGIKHLKERSPYRLSGGEKKKVAVASVLVLNPQVLILDEPTSGLDPKTQRWLVDLLLELKRGGRTIVTSTHNLALAHSISERALVLSEQHCLVYDGPASDVLENRDLLMEVNLIDEYC
HIHGEGEHIHCYTHE*
>Syntrophomonas_wolfei_subsp.metylbutyratica_JCM14075T_01836_Energy-coupling_factor_transporter_transmembrane_protein_NikQ
MLNQMDSGNRIKLPLWMEQGNDPGTFAPCSGRKTHRFLGRTINEMKKTLADDLTAEYYAGQDGLLQRFDPRSKLIMAIIVILLAGLTRSITVLLALWLLSLVLMYCSHLPVLSLQKRIWLFIPLLTLLISLPATLNIFNPGDILLKIHSFGEGAQFLGIKLPAVLYISWQGACSAAFLFLRVGISLTFGILLAISTPVARLLRSLQVLGVPQLFIMIVEMSFRYLLLLLNISIEMYEARSLRTVGKLPPKTQRAM
VASSIAALFVRSMTLSEEVYQAMASRCYTGEAVSIEEMKMGRLDFAAILIIIVLTFSITFGGAFFG*
>Syntrophomonas_wolfei_subsp.metylbutyratica_JCM14075T_01837_Energy-coupling_factor_transporter_probable_substrate-capture_protein_NikMN
MFRNYKALWLALGTLIILSPLSPLGLLATGTAFGEWGLDELVEEVGFIPAGLEKLAEFWQHSLLPDYGIPGMDATFSQAAFGYIFSAMVGVILVSAAILILSKIAKD*
>Syntrophomonas_wolfei_subsp.metylbutyratica_JCM14075T_01838_Energy-coupling_factor_transporter_probable_substrate-capture_protein_NikMN

MHIPDGYLAPQTAIPLLVAAVPLLAVGVKKVGETVKKREVPVLAVGAAFSFVIMMFNLPIPGGSSAHAVGAVLLAILMGPWAAFVGVSVALIIQALVFGDGGVMAIGANCINIALVMSFVGYYVYRMIRGNADSTSGRAVVAAFLAGYVALNAAAFITALEFGSQYHLFQAADGTPLYFMYPLKVAIAAMMSEHLLLAGPVEGIVTSLGMLFVSRNYPEMLSRNMIAGGKNWGVDSHV*
>Syntrophomonas_wolfei_subsp.metylbutyratica_JCM14075T_01839_putative_RNA-binding_protein
MCEANVYINREGREEILMEKVDRIIPGEDNNIFLESIFGERKIVQARIREMELVHHRVVLEEVKQEIITPDKEIWLEPETDHGHFHEGEEVRLKVFQGYNMRAEGKPACPELKAFAVVDGEARQIDIAEKEDCGEINLGSEVDGLVQVYVRESGKIDNYAKVVVEVGHHHHRGLQAIGLPLEIIPCDYSHARMGESYEVQVLKEGKPLPGVLVRATFAGTHNRDYPHKLNTNDEGKVKIFLTARGNYLF
SVNDGEIRSTFTLMKSF*
>Syntrophomonas_wolfei_subsp.metylbutyratica_JCM14075T_01840_FmdE,_Molybdenum_formylmethanofuran_dehydrogenase_operon
VCKDMPPLELAIQFHGHICPGLLMGVRVAEFAQEYLGVGRDYDEELVAIVETDSCAVDAIQAILGCTFGKGNLIFKDYGKSVFTIGSREKSKAVRFSQNIDALAKPQFKRFRELNQKTSLSSDEVAEKENLVGELFEKIMTASFEELFKWREVPYEIPAKARIHKTIPCALCGEGVMETRVIISERGKLCPSCQAEFGGELNV*
>Syntrophomonas_wolfei_subsp.metylbutyratica_JCM14075T_01841_hypothetical_protein
MVFAPMFFRKKHIAVLCFYVSKVMLYLGEKYRKGGESLRIAVIDGQGGGIGRVIVEKLRHELGQSCSIIALGTNAVASSMMLKAGANEGASGENAVTYSLKRADIIAGSVAILAANSYSGELTPRMAEAIASSEAIKILIPLNRCGIEISGVYDEPLPIQVDHLVNRVKKLYDEKKS*
>Syntrophomonas_wolfei_subsp.metylbutyratica_JCM14075T_01842_hydroxyacylglutathione_hydrolase
LLKQIGPRIFVVTPEDSPRYPYGNCLYVEDDVPAVLDLGAGGRAFQEIPCEKVQLALISHSHFDHLHGDSFFPRAGLYAGKEESKTYSDENEYLKFHGYELWAEMMPGIKRVAFSEVSPLPDDVPVKPGFRYIPLAGIFKDMERFQLGKTEITAIHLPGHTIGHYGFWIEKEGILFSADMDLVVAGPWYSSNSADIGQLVSSVQRIREISPGLIVPSHRRPILDKIEKRLEQYIGVVFKRQERILDLLKEAHTI
DELLEYQMVFHGRRDIYLIFWEKMTLRNHIRFLQREGMVEEVEKGRYKRC*
>Syntrophomonas_wolfei_subsp.metylbutyratica_JCM14075T_01843_hypothetical_protein
MNWIGLYFGLYVLATIGIYHILVVKLELRFGTWPWVGFLLLGLVCLYFSLRVQDVGWSMWWGYNAFINLWSVKEMFDQGKRRA*
>Syntrophomonas_wolfei_subsp.metylbutyratica_JCM14075T_01844_Glutamine_transport_ATP-binding_protein_GlnQ
MEKIIKINHLQKSFGAIEVLKDINFSVSKGEVVCIIGSSGSGKSTLLRCLNLLEIPSSGEVVYHGQNILEQKMSNAVYRAKMGMVFQQFNLFNNLNVLDNCVIGQVKVLKKNTAAAKKTALDYLELVGMSQFINAKPHQISGGQKQRVAIARALSMEPEALLFDEPTSALDPEMVGEVLKVMKNLAKSGLTMLIVTHEMAFARDVANRVVFMDSGVIVEDDVPDVVFNNPNNERTRIFLKRILEN*
>Syntrophomonas_wolfei_subsp.metylbutyratica_JCM14075T_01845_Inner_membrane_amino-acid_ABC_transporter_permease_protein_YecS
MAVDANSSFITWVLYILQSSWPLFLRGAQVTLIISLSGTIIGFLIGVLIGIIRTIPIDHNTNLFKRIVLKIIDYFLLCYIEVFRGTPMMVQAMVIFYGSAMIYGIRMSPIFAGVFIVSINTGAYMAEIVRGGIISIDKGQFEASHAIGMTHAQTMISIVLPQAIRNILPALSNEFVINIKDTSVLNVIGVTELYFQSNSAAGNNFRYFEVFLITCIIYFIMTFTVTRILRYFEAKLEGPSVYTIHGSQTVPEAEIKISTGSKKNGK
NH*
>Syntrophomonas_wolfei_subsp.metylbutyratica_JCM14075T_01846_Arginine-binding_extracellular_protein_ArtP_precursor
MKKFVKLGICAVLMMALCISVVGCGKSGDKAEKFKVGMECGYPPFNWTQLDNSNGAVPIAGSKEFAGGYDVEIAKKLAEGMGKELVIVKTKWDGLTPAVQSGTIDGIIAGMSPMADRRLTLDFSDYYYSSDLVIVVKKGSKYEKATSLKDFTGAKITAQQNTFHYTVIDQIPNVKKQTAMADFPAMRVSLESGIIDGYVSEKPEGVSATSANAKFGMAEFAKGQGFEFTADDIAISVGIKKGNTEL
LNNINKVLAGISQEQRDELMKNAILNQPASQ*
>Syntrophomonas_wolfei_subsp.metylbutyratica_JCM14075T_01847_Putative_sporulation-specific_glycosylase_YdhD
VNSTQKLIKLKKIISIIMLILLLAGCNGCNAAKKPLHIPRQKKLQVMAFYEKGWNDLTYGFPSLKTHYTDISILMPYWYTLNIDGSIKVSAVGLEPDVIVFMRQHPEIKLLPLINKDLVNQSVLTDPTIRTTAINNIVKIIADNNYDGINIDFELLPAQNRDDLTIFVRDLAKQLHAQNKMISVSVMPKLDG*
>Syntrophomonas_wolfei_subsp.metylbutyratica_JCM14075T_01848_Spore_germination_protein_YaaH
LADIITIMTYDKHSANTKPGSVSPYKWVEKNIKEALKSIPANKLVVCIGAYGYDWPVPKTAEIKYIGQREAQQRADSHKAKVQWDNDSQSAFFFYWDRGLKHEVWFENGFSITRKVALAKKYSLRGVAIWRMGFEDEQYWVKLRKAISK*
>Syntrophomonas_wolfei_subsp.metylbutyratica_JCM14075T_01849_CRISPR-associated_endoribonuclease_Cas2
MFVILVYDVGEKRVTKVLKTARKHLYWVQNSVLEGNISESSFKQLKVELEKIIDEDYDSVIFYTFRTTKYSDREIMGVKKGGEDNFL*
>Syntrophomonas_wolfei_subsp.metylbutyratica_JCM14075T_01850_CRISPR-associated_endonuclease_Cas1
MKKTIYVFNDGEFKRKDNTLYFETEEGKKYIPVEDVKEIIVFGEVTLNKRFLEFLTQKEIILHFLNNYGYYTGSYYPREHLNSGYMILRQAEHYLDGVKRLVLAKCFVRGASANMLEVISYYANRGKEVQKQVEAISNLTERLEDSKAVDELMAIEGNIRDHYYQSFNAIIGKAEFDFQGRNRRPPRDRINALISFGNTLAYMAVLSEIYKTHLDPRIGYLHTTNFRRFTLNLDVAEIFKPILVDRVIFSLLGR
NMLRAGHFENKTQSIIMNDKGRKIFVEEWENKLRTTIKHRQLGREVSYRRIIRMDLYKLEKHLMGEKGFKPFVSRW*
>Syntrophomonas_wolfei_subsp.metylbutyratica_JCM14075T_01851_CRISPR-associated_protein_Cas4
MVIKFKTKKQAWGRVDMDFSVTGTLIWYYYICKREVWLMAHQLEPDQEHENLQIGKIIGESTYPRDKKEIDLGNAKIDLIRTEHGEMVVGEVKKTSRFKESAGKQLLFYLLQLKEMGIKARGELLFPEERDKLEVLLDDDGEKEIRQAMQDIEAIVRMDRPPEVAKTKYCRNCAYSEFCWS*
>Syntrophomonas_wolfei_subsp.metylbutyratica_JCM14075T_01852_hypothetical_protein
MIAVINASPLISLALINQLNIIREVFSEVYVPSQVYHEVLGNETERPGSEVLKAANWLKVVRVSNQITVDILSYSLDPGEAEVLALAYQLKPDFVIIDELKGRKMANYLGIDVIGTLGLLLMSKELKIIAEVKPLMQQLKKKGIWISNKLYDEVLRLAQETD*
>Syntrophomonas_wolfei_subsp.metylbutyratica_JCM14075T_01853_putative_small_protein
MQLVKINIEIPEEVLSNMGKERKEFEAETKLWLATKLFLDKEVSLGKAAMVAGLARLDFMKYLQEQKIDIYRYEEGELNKEFEKIKKAVKK*
>Syntrophomonas_wolfei_subsp.metylbutyratica_JCM14075T_01854_Retron-type_reverse_transcriptase
VRFPRATYLICTFQHENEAKAFYEMLEARLKKFNLELSKDKSKIIRFGRFAKQHSADGKTDTFDFLGFTHINGKTLTGKYTVLHRTSKKKPKAKKQNVKEWLKENIHSKPSDTIERLNKKLVGHYRYYGISGNYEGLLKFYRYIRTALYKTLTRRSQRTYLTWRRYRMLLEKHPIAKPRIYVNIWQAV*
>Syntrophomonas_wolfei_subsp.metylbutyratica_JCM14075T_01856_ABC-type_thiamine_transport_system,_periplasmic_component
LEKVILHCPMNISRAFSGLISEFIQREYSGNEFEVYDEPHRLGSEDSLLSSVREGGPPALYIGHATDFGRLSAPEITAAFETIPGLPLAESLQRLGFKHEHAYFHPFTIIPFGVIYNKELVGEKRPVSWQDFQDPVYSGKIRIPDRQRTISRVLVGTMKTLYPDGYEKFVANCVFQGSPIEVVNAVDQGEYHYGMVNIAFSRFSRLNNTAIMWIKEGSFCMPLVIAVGKGKGELVRKIVDYILSPAVQEFFTLQG
FIPAISGEIPDVLQKDELHLIWKGWDEFLKATA*
>Syntrophomonas_wolfei_subsp.metylbutyratica_JCM14075T_01857_Dimethylglycine_N-methyltransferase
MLKSSEGKSSYNALLPIYPLLAQQCVDDYALDSGICLDIGTGNGYVGTEIAKITRMDIYYIDIEAEALSFARRSVAEADIDNEVFFIEADICQGLTLADNFADFIISRGSLWFWKDRARGLAEVYRLLKVGGIALVGGGLGRYVPWTMRKRLSGERKNTLERKGGKRLSLEEMEALAIEAGISSFRVMSDAPGDKGSWIEIHKHL*
>Syntrophomonas_wolfei_subsp.metylbutyratica_JCM14075T_01858_vitamin_B12-transporter_protein_BtuF
LRRSRSYIIFLSILALLLSSCLAGCNKEKPQETAAPAEPGMRTIVDMAGREVSVPEQINKVFSTNPPGTILLYTMDPDLLIAWNYYLGEAEKKFILPQYHELPNLGGWMAKTTCNTEELLKIGPDLILYMGDINDMSKSQADNIQKQVNIPVVMVNGDLTKMDETYGFTGKLLNMEKQAELLGSYCRETVKQVQTKSKNIAEDKRVRVYYAEGASGLETDPCGSRHTEVLDMVGGVNVADVIAKG
ERGMTQVSHEQVLLWNPDLIICWGQCFKEIFSDTKWQNIKAAQDKKVYMVPSGPFNWFDRPPSVNRILGLKWLGNLLYPEVYAYDIAKETREFYQKFYHYLTDEDITFLLKDSGGK*
>Syntrophomonas_wolfei_subsp.metylbutyratica_JCM14075T_01859_ubiquinone/menaquinone_biosynthesis_methyltransferase
MKVEINALEFDRMAREVFAPVYPVLAEQIINRMGISEGCCLDIGCGGGYLGLALARISELETILFDESQDMLNLAQDYIGQSRLESRVKTLLGDVHEIPLANESVNLVVSRGSMFFWENRVRAYQEIYRVLAVGGAAMIGGGFGNVELLREIEAKMLEKDSEWKEKRQQRIGKGKVEVYREELKKAGISSFEILQSEAGLWIVTRKTA*
>Syntrophomonas_wolfei_subsp.metylbutyratica_JCM14075T_01860_iron-dicitrate_transporter_substrate-binding_subunit
LKRNRSFIALLTILALLLSCCLAGCGNEKPSETTASKESGIRTVVDMAGREVEIPAKIEKAFPTSPVGVVFIYTLEPELLIGWNNELRQGESKYLPLPKYQNLPHLGGWYAKTTCNTEELLKIHPDIILAVGNTDQMAISQADSIQQQLDIPVVILSSDLNKLDKSYELAGDILNLKEQANKLGAYCCESVKDVQSKAQSITEEQRLKVYYAEGPAGLQTEPKGSAHTRVLDMVGGVNVADVAMKGGKGL
TPVSLEQLLSWNPDVILSWNMGQGGYYEKMLSDSKWESISAVKNGKVYAVPSGPFNWFERPPSVNQVLGLKWLGNLLYPDVYKYDMAEETREFYKTFYHYNLTDEDLTSLLKDSGGK*
>Syntrophomonas_wolfei_subsp.metylbutyratica_JCM14075T_01861_glycine_radical_enzyme_activase,_YjjW_family
VKCMICERFCKIPEGKSGACGLYLNQGNEIVERYPNRYLITTPISIETMPMLHYYPGSKFLQVSTAGCNFSCSGCISTVIVKELEAGSQALRCLQPDEVVALARKQECLGIAFLINDPLASFFTFSRLARTAKEAGLLVGCSSNSYFSEQSLSSIIPYLDFINIGSKGLSDESYRRCGGSSAAPVLRNLNTLHDNGVHVEVSCILERDNQEELLQLARIIAGISTDIPLQLMRFIPFEDADPEREISIQEAEEFMPEVK
QQLPHVYLFNSPGTRYLDTPCPQCNSTLYRRDFYGPMGAKLKNLKEIHEAAGVCPHCGQLSPISNGAPADPYQEGGFQGGYPFTRALEMIQAILIALGVENKQEVVKVWEEVLKEQHLPELHHQMQNPQSYLVLLRRYGQMVGRSLQAEALAGYIEEKIALIEEAGYPRIKRLPRVYYCMGKPLFCIKGERFENQMVELAGGYSVNKEIDIDGRPGETLTPAELMALNPEVVFISAFISAPVKSFFQSC
AENAIDIPAVRDSRVFLHPSPGWDFGNPRWILGLMNMANLLHPEIYHFDIEEESRRFYQEFYRSDYNPAVINRSFSKPTMHWSWK*
>Syntrophomonas_wolfei_subsp.metylbutyratica_JCM14075T_01862_ferrichrome/ferrioxamine_B_periplasmic_transporter
LKRTRNIVVLLTILALVLSCCLVGCSKEKAPETTPPTEPTVRTIVDDAQREHTIPTEIKKVFSTSPVGTILLYTLDPELLIGWNYELREGEKEYILPELHNLPNLGGWYAKATCNTEELLKIHPDIISVGVIDDMAISQANEIQEQIGIPVILINGELANFDKTYEFAGKLLNREEKAQVLAAYCQKAISDAQNKSKTIAPEERLKVYYAEGAAGLETDPRMSRHTEVLEKVGGVNVAEVAMKGGMGMTPVS
LEQVLSWNPDVIISWNRTQGGYFDKIMTDPKWEGIAAVQNKKVYAVPSGPFNWFDRPPSANRILGIKWLGNLLYPDIYKYDMAKETKEFYKKFYHYDLTNEELTSLLKDAGGK*
>Syntrophomonas_wolfei_subsp.metylbutyratica_JCM14075T_01863_hypothetical_protein
MNVIVKRQESILEEVLEQLKVLLGESINELTIERVVIGVFFTGVKLSNGQGGICATPVKVMPDAVCCSSSTGRMPRAGMLKGRRALEIAEEAIREGDTALKKALAIAILSALSATSWVNNPPQDYVIKRNTDPLDMVFSQDKNVAVVGALGPLLKKLKTGKVPYRVLEMDISTLKPEEIPYFCPPEKAAEVLPWADIIVSTGTTLINDSLEELLITARPDAQFIVVGPSASILPGPLFARGVSSIGAVLVTDAD
QLLDILGEAGAGYHFYSRCADKITLYPRKDN*
>Syntrophomonas_wolfei_subsp.metylbutyratica_JCM14075T_01864_hypothetical_protein
MLSLEAANELIWKKFRELPLEYVNFCDGITFRKVEANECDR*
>Syntrophomonas_wolfei_subsp.metylbutyratica_JCM14075T_01865_putative_ABC_transporter_ATP-binding_protein
MEMEFRNLSCGYGQRRVVDGISLKLKVGEVVGLLGANGCGKTTLIKTILGLLPVQGGEVLLDGKNIRNWTSAQIARVIGYIPQMHTPPFPFEVLDVVLMGRTAHIKPFSSPSPQDVKIAEQALDMLNISYLRDRVYTEISGGERQLVLIARALAQEPRILIMDEPTASLDFGNQIKVLSHVKQLAGMNIAVLMSCHFPEHAFIYSNRAVLLNEGKILHSGTPGETITAERLKTLYGVDVDIVSVKNRMN
QELRVCIPSRNFALQPMQSMDRRKEYAFA*
>Syntrophomonas_wolfei_subsp.metylbutyratica_JCM14075T_01866_putative_ABC_transporter_permease_protein
VKTVIKERIAEVKTKRDAGWKILILLSTLILIFILSFALGRYPVPPGQLLEVLASRVFPIEAHWNATVETVIFQVRLPRIFAAILVGAALSTAGAAYQGMFKNPLVSPDILGASAGAGFGAALAIYFSFSMLGIQMMAFVGGLLAVGLTYGISLRIRHDPMLALVLSGIMIGSLFSAAISCIKYIADPYDKLPAITFWLMGSLASITTRDVYMALTPILFGCILLYLLRWRLNVLAMGEEEARVLGINTGVMKTIVIV
CCTLMTAASVSISGMIGWVGLVIPHLARMIVGPNYRVLLPASILLGGGYLLLVDDLARLLASMEIPLGILTALIGVPFFLYLLLLNSKWGWK*
>Syntrophomonas_wolfei_subsp.metylbutyratica_JCM14075T_01868_Copper-sensitive_operon_repressor
MENESKNSCANCIKTNNKNRSWSDENAVKELTVRLNRIEGQVRGIKRMIEEGVYCDDVLNQITSAQSALYGVARLLLQKHMKSCVKEQLASGDEQVIDEVLATIFRMMR*
>Syntrophomonas_wolfei_subsp.metylbutyratica_JCM14075T_01869_Copper_chaperone_CopZ
MEEVILNVEGMSCNHCKMAVEKSLQSLFGVKKADVDLAAKTVRVAYETGKLTLKDFEKAVAEAGFELLK*
>Syntrophomonas_wolfei_subsp.metylbutyratica_JCM14075T_01870_Copper-exporting_P-type_ATPase_A
MEEIKSTIRIGGMSCAACSARVEKKLNNLPGVKKAQVNLLSNKATTFYDPGIIKLSDLEEAIRQIGYEVLPEEDGNYINATLAIEGMSCAACSARIDKKLNSTPGVVNASVNLLTNLAKVKYDPQLISIDEVEKVVDKLGYPTHWIEQREHPLDSPDKNTEIKNLKLLLVASAVLAFPLILNMLLMIFDIQVTLLHNPYWQLALATPVQFIIGYRFYRSAFLALRSGGSNMDVLVVLGTTAAYFYSLYNIYQG
EMHNIYFEASATITLILLGKYLEERAKNKTSEAIRVLGSLQPRSARVVRQGEEMDLPIEEVRTGDLVVIRPGERIPVDGIVEEGHSAVDESMLTGESLPVEKRPGDPVVGASINKNGSLKFVVTRTGQDTTLAQIIRIVEEAQGSKAPVQKIADQVSGIFVPAVIGIALLTFILQYWYNANISIAVTTAVAVLVIACPCALGLATPTAIMVGTGKGAENGLLIKGGEFLELLHKVDVVVLDKTGTITRGKPAL
TDIISLDGYVESEILHWAGILEKHSEHPLGEAIYASAREQYGDLPDPEDFKNYPGQGVTGKSTNLALAIGNRSFMHSRAIDTTEAEQQARQLEEAGKTAMYLAIDGKLAGLLAVADTIKENALLAIQELKDMGLEAYMISGDNQHTAQAIARQVGIETVLAEVLPERKAEEIEKIRQSGKIAAMVGDGINDAPALATADIGIAIGSGTDVAMETAGIVLMSGDLSGISAAIKLSRQTMRIIKQNLFWAFFY
NSIGIPFAALGFLSPVIAGAAMAFSSVSVVSNSLRLRRFEP*
>Syntrophomonas_wolfei_subsp.metylbutyratica_JCM14075T_01871_prevent-host-death_family_protein
VNDTTIIITATELKTHLGKYIEYVIDDHEVVITKNGKKAIRITPYITEIERYFLVKENALDYQYGGKKVSYEEFMEIYEKSELRMEYINGEIVLLASPSTYHQDISGNLIHIHLRSYLKGKNCKVFYAPFDVHFYKKDLKTPDVMQPDLLIACDLENNVNENGRYMGTPTLCIEILSKSTRSKDMVDKLNTYMLSGVREFWIVDPFKHSIMVYSFKDFEIDEYTTCKLGDTVTSYFFEGLEIPINEIF*
>Syntrophomonas_wolfei_subsp.metylbutyratica_JCM14075T_01872_Acetoin_dehydrogenase_operon_transcriptional_activator_AcoR
MISRMQKEFTPLESCWEEFVGNGSLDRDALSTEIADAWERCARLGTNPYVGNSKRRLAESEVQEMLQRHKELIDVARPFMAKLHRFVAGSGFVVLLADERGYLMETIGDQDTVREAENIDLLPGACWREEEIGNNGVGTAVVLKKPVQVSGKEHYCRKHHSWTCSGAPILNEDKQVIGILEMSGPLEKTHLHTLGMVVAAVEAISDQLRIQQKNRELVLLSNRLGYIYNTVSDGIMVIDRQGIIT
QINPAVERILGQNSRKIMGIPVKELFEPGQRVEEMLDSGRDFSYSELTLGIGEAVQCLVSGKAIMDEQGKVNGGVIFINPINHIKSLVNRFSGAQASLHFSDIIGQSQELSRVVRIASLAASSESNILLEGESGTGKEVFAQAIHNASSRRDAPFIAVNCGAIPRELLGSELFGYVDGAFTGARRGGRPGKFELASGGSIFLDEIGEMPLGKQVSLLRVLQERTVTRIGGDKVIPVDVRVICASNRKLHEEVEKG
NFRRDLFYRLNVIAITLPPLRQRKEDIPLLFHHFLQDISQKLGVKTPQVEAEVIHSLQDYDWPGNVRELQNVVERMVNIAEESRISQEHLPQEISHPAARSLKELENGEDSQLLSLSTERRKRKEMEQERERERREEIMALLVLHGGNVSQVAKSLGVSRNTIYRKIKQYRIKI*
>Syntrophomonas_wolfei_subsp.metylbutyratica_JCM14075T_01873_NADP-reducing_hydrogenase_subunit_HndC
MKLIIDGKAFNAYPGDSILDVARREGIAIPTLCYHEAFGGQGSCRLCTVEVTAGGRSRLVASCTYPVSEGIEVKTSTPAVEKIRRNIIMVLYKRASGSVLMQKLYREYGCSENSLGEKQEERCIVCRLCVLACEKMGTSAISTVMRGIDKRVGTPYDQAATACIGCAACAHICPTGAIEVLDSGNIRTIWNKNFNLINCERCGQPFATREEIDYVSRRSGIEDAELCFCEECRRKKLVGKMQGFSPC*
>Syntrophomonas_wolfei_subsp.metylbutyratica_JCM14075T_01874_NADP-reducing_hydrogenase_subunit_HndC
MTTMNKSLIHLLLVCCGTGCVANGAREVYQALKDNLTSDDKAVLAATTSARATGCHGLCAQGPFVRILPEDITYCRVKAADIPEIVEKTLKQGEVVKRLLFRDRQKKALVQGQNDIPFYRRQTRVALRNVGLIDPSSLQDYLERGGYRALEKSLLDMRPEQVIEEIIKSGLRGRGGGGFPAGLKWKACAATPAQPRYIICNGDEGDPGAFMDESIMEGDPHSVLEGMIIAAYAVGASTGFIYVRDE
YAQSVASLQKAIEEAENHGFLGENILGSGLDFDIEIVRGGGAFVCGEETALIASIEGHVGEPWDKYVFPSEKGLWGQPTIINNVETWANIPVIMERGASWFTSLGTEGSKGTKVFSLVGKVANTGLVEVPMGTTLREIIFDIGGGVPQGRKFKAVQTGGPSGGCIPAELLDLKLDFDSLSQAGSMMGSGGLIVMDDRTCMVEVARYYLSFLAGESCGKCVPCREGIKCLLEILTRICHGQGEIEDLELM
EDIAETMQQASLCALGRTASNPVLSTLRYFREEYLEHIEGQRCPAGICRELSEFWIDPAICTGCGLCLPRCPVEGIKGEKKQAHRIESDKCICCGECIDSCSFQAIKVK*
>Syntrophomonas_wolfei_subsp.metylbutyratica_JCM14075T_01875_NADP-reducing_hydrogenase_subunit_HndA
MSEQECEENIEGFRRIIYGFPGEKRYIIAIMHELSRCYRYLPRAALELTAEYVGVPFSQVYSMATFYRAFSLQPRGRVNFKVCDGTTCHIKGSNILLDEIYKNLGIGPGETTADREFSLETVNCIGACAIAPALLVNERVYPRVNAAALKEIIKEYRGGDNDHHE*
>Syntrophomonas_wolfei_subsp.metylbutyratica_JCM14075T_01876_putative_oxidoreductase_YdhV
MYAWIGQILRVNLTDATIKKEKLDPVLRRNYIGARGLGSKLFMDEVDPQVDPLSPENKIVFMTGPLTGTLAASGGRYNVVTRGPLNGTIAASNSGGSFGPELKYAGYDGIIFEGKAEKPVYLWINNAEVELRSAEELWGRNVPETTDMIKQATHEEAKVACIGPAGEKLVKFACIMNEYNRAAGRSGVGAVMGSKNLKALAVRGTGGIKVADRQGFMQAITDARNKLKAHPVTGAGLAAYGTN
VLVNILNEHGGLPVKNFSEAAVFAKAENVSGEYQAEHCLVRNKGCFGCSIGCGRVSRNRGKYKGIGEGPEYEATWGLGPNLFIDDFEAISKANFLCNELGLDPISLAGTLACATEMMEKGFIPWEKADLRWGDADMLVEMTIKTAYREGFGDELAEGSYRLAEKYGHPEFSMSVKKQELPAYDPRGQQGIGLNYATSNRGGCHVRGYMTSPEVLGIPEKVDPDSTEGKAALLKIFQDLTALVDSA
GICLFTTFGQGLPEIAEQLRQATGLDLSDEEFLLAGERIWNLERSFNLQAGISSKDDTLPPRLLREPMKGGPHQGNVVRLELMLPEYYTLRGWDQAGVPTPEKLQELSL*
>Syntrophomonas_wolfei_subsp.metylbutyratica_JCM14075T_01877_NADH-dependent_phenylglyoxylate_dehydrogenase_subunit_beta
MGSQSEMEKIIVVQPEKCTGCRTCELVCSFTHTGEFNPARSRISVFTFEKVGFSSPVLCQQCSTAACMQVCPVGAVSRDNKTGAMVVNSSRCLVCKACTIACPLGATFYDHVSDSILKCDLCGGDPECVKFCPSGAITYQEAGEAGLARRKAVAAKLKDVYEEVS*
>Syntrophomonas_wolfei_subsp.metylbutyratica_JCM14075T_01878_Molybdopterin-synthase_adenylyltransferase
MDKMDVLLRQKAPSGLIGWKEEEMALALGLSLHEAEGRLLAQGVLPQRYQRNAKSITREEQCRLFQSRVAVIGCGGLGGYLLEELARLGIASISAWDYDSFAEHNLNRQLLSDMDTIGLSKVETAAKRVQAINPAVKFQAFNRPFAAGEGEDLLAGHQVVVDALDSIPVRKMLAAVCQRLSLPLVHGAIAGWCGQVCTQFPGEDSLETIYRNSNQEQGVESKQGVLAFAPAVVASLQVAEVVKI
LLGRGELLRKRILLFNLLDMEFEVLDL*
>Syntrophomonas_wolfei_subsp.metylbutyratica_JCM14075T_01879_MoaD_family_protein
VKIVVKLFATLRQGRFKIQSGIYPEAAKVQDIVEGLAISPTDLGIIFVNGKSADLNRVLQEGDTLSIFPPVGGG*
>Syntrophomonas_wolfei_subsp.metylbutyratica_JCM14075T_01880_hypothetical_protein
MKAVIERFEGDFALLEIRDKGQVIKVRRAKLPPPAREGDVVFCQGEEWLIDQKATAALKKEVEDLAAELWQD*
>Syntrophomonas_wolfei_subsp.metylbutyratica_JCM14075T_01881_ComEC_family_competence_protein
LGGCAAPREEASPPAHNELPVQSKAVSPAPKAEPAGSSEDILKLHFIDVGQGDAILLQCPGGENILVDGGDKVQGEALCAYLQECGIQEIAAIVGTHPHADHIGGLIKVVQEFPVKAVYMPRVTHSSDTFRDLLLAIKNKGLKISTAKAGVELPLQAIKARFIAPRADHYEDLNNYSAVLKIDYGAKSFLFMGDAEKESEEQILSSGLNIRADVLKLGHHGSASSSGEDFIKAVAPSWAVIMCAPNNDYG
HPHRETLQVLGKYGAELLRTDEDGTLIITSNGQKIEAYKKGKKEASAGLSPVPVSTAVQGEEGVFYIGNLKSRVFHRPDCPNLPAAKNQIRLPSREEALEKGYRPCKNCQP*
>Syntrophomonas_wolfei_subsp.metylbutyratica_JCM14075T_01882_Superoxide_dismutase_[Mn/Fe]
MAHTLPDLPYAYDALEPFYDEQTVRLHHDMHHKAYVDGLNNAESKLAEALEKGDFALIKHIERELSFHGSGHILHTMFWENMKPGGGGPADGAVAELIDRDFGSFDNFKKLFSASALAVEGSGWAILACNPVFEKLVVLQAEKHQDLTQWGAVPLLIVDVWEHAYYLKYQNKRAAWIEAWWNLINWDDVNRRVALMQQK*
>Syntrophomonas_wolfei_subsp.metylbutyratica_JCM14075T_01883_cryptic_autophosphorylating_protein_tyrosine_kinase_Etk
MQGNDPSYYNDYEEIDLRDIFKTINKWKHTIIAVTLICMLLSGIFSFFILEPVYEASTVVSIYQAQLSRENESTNDIADTVNELGEIPYMSALSCEQQVKSPAIMEAVIKKLQLPYTRNGLKSSIKTQQLQNTNLIIITVSNNNPELAATIANTLREEFILHINKINKKKMAQSLEIMEKEWLQKEEAELDAASEKLKEHKLQSRIEFLNSQLGKKREDLINYQSWLAKAEIDRASQQEGIKQLQETIAHTPHTL
TTVNTANGILPADLQGIDIKDGTVSSEQINMNYTSLMDAYNSKTTDLAELNVQINKTRQEITRQEKEILDLEAELIRNQIGEKKLQNEVSRRESVVTLLSSKIAELKMTESINLADNNIITVSEAMVPEAPIKPNKMLNIAIAAVLGLMLATFSIFLIEYLRENGTNKSESEELKGFTGNV*
>Syntrophomonas_wolfei_subsp.metylbutyratica_JCM14075T_01884_Macrolide-specific_efflux_protein_MacA_precursor
MKSKKNWIIGGLILLALIAIAAAMMAISAEPVEVVQASRGTFTLTLEETAYVQAADDRELQAVSTAQVRELLVASGDKVMPGQLLMKLSSPELTAETASARSQLARTEAELQAAGLKMQSLRLELKQAENDLARKKTLLQSRALAQAEYEEAELEVDKLRKTVSQQEAGVSGLSQQVASLSEMFNSLEEKREQLQVTSPVSGIILDLPVKVGQVVAPGTLLAQLGSAGQLEVKAELLSDEIRRVRIGQS
TQITAPVLGEEVLSAQVSKIYPRAYEKTSALGVIQRRVPVIITLEQTANLQPGYEVRVAIETLRKEGVILLPRESVRLDEKGHYRVWLVKEGRIEQRSIQVGEKNQQWVEVIKGIQVGETVVRDGSRELKKGSRVKALKQDESV*
>Syntrophomonas_wolfei_subsp.metylbutyratica_JCM14075T_01885_lipoprotein_releasing_system,_transmembrane_protein,_LolC/E_family
MTILTRRLFRTIQAGRGQFLALVLIVTLGVIIYIGMSSSYNNLSRSQQQFYQDYAFADYTFQVVKAPESIVARVEALPGVLNASGRIQKDVPIIKEGQERITGRLTGYSLPMENELNRLHLLSGRQLEPTASGKVAVLLDPQYARANLLAPGQDMEIIADGKKVSLAVIGTATSPEFIYPMKDAGSLFPEPERFGILMLSQLQAQQILGLPGQINQLLVDLAPGVDEEKIKEQVEKILAPYGNITSYPRKDQS
SHLVLQAELDGIKLFALTLPFIFFLVAAGIQFIILTRLIRSQRLPIGVMKALGFDNCRIMWHYTSYGLAVGLVAAILGTGLGIALTAVLNNLYAQFFNLPNMTNEINFRVVLYSFLITSLVGIASGFFASRSVIRINPAEAMRTQPPSSGRRTPLESWYWFWQQLSSSWKMSLRSIFRNRGRFAVSVLGIISSVVLLVFSCFSLDATDYLMNQNFEQINRYDYMIRFTEPVKYSEILDWTRWNEVQRMEPMMEV
PVKLHAGGESKDEVLLGMTPSNRLKRVYDKLGQERQIPEEGILLGKRVADKLGLQVGDEVVVETTLGIGPSRSSRLTIMGINEPMTGSGSYVSLATANSLLEEQELASAVLLKLDAARMPSVENRLQEMSRVASVSSPARERETFEQLMGTTTTSIAIMILFAGLLGLAIIYNSSVMTFNERQRELASLRVLGYSRGEVSGLLRKETWMQALLGIALGLPAGKAAGAAYIASASTELYSFPVIIYPRTYFIVA
GLALFFVWIGQQLAIRKVERLDMVEALKNQD*
>Syntrophomonas_wolfei_subsp.metylbutyratica_JCM14075T_01886_Lipoprotein-releasing_system_ATP-binding_protein_LolD
LPNEVLLSLSDISKTYTMGEVKVEALKSTSLEIYRGELLVILGPSGSGKSTLLNLIGGIDTPSSGQMLFGQQLLSNGREKALTRYRRQEVGFVFQFYNLIADLTAAENVALAAELVAEPLAVDDVLRDVNLWERQDHFPSQLSGGEQQRVAIARAMVKSPPLLLCDEPTGALDYETGKMVLRLLRDIQKNHASTVIIVTHNTAIADMADRVLRMRSGLVAEIVINENPLPPERIEW*
>Syntrophomonas_wolfei_subsp.metylbutyratica_JCM14075T_01887_marine_proteobacterial_sortase_target_protein
VAINFSHPLLLLIIPLAFFPLYLWYRDSGHLPAFRRNLALLLRSLLLSLILALAGLELRFPLERQSVVFAVDLSASCENSREAAENFIREALKHKKADDQAGVVVFGGNALVDQSLSDSSRLHPIESLVDRNYSKPEQALKLADALMPDSFSRRVVLLSDGRQNDGDALKEAAYLAEKKIRLDLLPLESSPTRDARVKSLEVPQSLFAGENFPLKVGIESNFATTARLRLYQDQELIGEEEVKLRQGENMLLY
SSVLDEAGFHSFQASIEVEQDGVADNNEAYAFSMVQGPPRLLLVEGKKGEARGLEAVLSSLNMDYDLLSPRELPASLTELQRYGLMILCNVPAEDLREAAMQAVHTAVRDLGKGLLMVGGEDSFGPGGYFNTPVEKALPVYMDLRGKKEIPSLGLVLVIDKSGSMSESSGGYSKVELAKEAAIQATSILGPLDMAGVVAFDDTAQMVVEFQAVKDKDAIQDDIATIRADGGTSIYPALAYAALK
DAHTKFKHIILLTDGQSATGDYYFLSRRMARAGITMSTVAVGEGADTLLEQLAAWGQGRYYFSDEISNIPRIFTKETMKAIKSYLVEESFFPVLTAESPLMDGISAVPVLEGYVATTPKKAAQVVLSSHRQDPVLAQWQYGLGRSIAFTTDGGWRWSSAWTAWGDYNHFWGNLISWTLPRSQQDARLELKTSREGRQGLIQVDSRELSLSLPSEAVVVDPELKAQKIKLEPVAPGRYEGRFSTGE
PGVYFINVTQQGSEGELRSASGGITLAYSPEYAESGSDKKFLQALAQAGGGSIISEPREAFAPNLPVAHGVRELWPWFLMLAALLLPLDIANRRLAYNLSDLQAWWQRWKKPEGTALTSTPAAVTFNRLRQRKDDLNEQERQRSSGRQLIGDEGRGKPEKASIPIREGSEKDKALEKDKVQKTMQDKDGMSRLLAAKQRARK*
>Syntrophomonas_wolfei_subsp.metylbutyratica_JCM14075T_01888_hypothetical_protein
LSLLNPAGLGFAAIIPLIILLYLLKRRFQEKVISSTYLWEEVLKDMEATTPWQRLKKNLLLFLQLLAAVLLVMALSRPYLPVAGGLEKQLLVVLDASASMMASDVTPSRWGMAQKEVEKLIDRLGPGEKMTLIKMGEQPEVLQAASGDKNELRRALRDAQLETGRADLEAALSLIASLSRDSRDIMLLIVSDGKTLPMEHDPGIKCPVEFIQIGRGSDNLAITTLATRRDSGQVLALARVQNFGEKAVV
SDIELRVDGELFDIREIKLEAEGAREIIWEDLPAGAESIQAILQRPDFLPADNEAWAVVEELTENRALLVSQGNLFLEKALNLLPGLELFKTSPAEYDPALADYQLFIFDSWLPAGLPQASIIVNPPPGNPWLAINGEAKNLSGIKAAGEDSNLRYIDSSDWQVARSQKLLCPDWARTLLESEGVPLLLAGQLSGRVAIFSFDLHESNIPLQTGFPILVHNLVGWLLPGNAGASFDLNRSRISFDPHPEAEAI
YLQRPGEKEERYLPPFPRSFSASHPGLYQINQVLGDRIESNFLARNQGDPLESDIKPATLPWQAFEAESRGGGERLGKKELWPWLCWAVLLLLLLEWEVYRRGY*
>Syntrophomonas_wolfei_subsp.metylbutyratica_JCM14075T_01889_hypothetical_protein
MKNIFDEDFLKRLEHLRLVSRKMRAGKNSGERRSPLKGQSVEFADFRNYSSGDDFRYIDWNSYARTEKLFVKLFMEEQDLLLNIFIDVSKSMSWGEPPKERLALQLAAAFSYLALAAYDRVAIAACSEQLAAYQAPLRGKTGIDQAWTFLGQLSFAGSTNLNQALREYGRFSRGPGVALLISDLLSPHGFHDGVKFLQYLRQEVIVLQLLAPEEINPTLHGDWRLLDSESGESREISASSRLLKAYRKKL
DDFTEANRDFCFRRGIGFLQVSSATPFEELILQLLPRAGILA*
>Syntrophomonas_wolfei_subsp.metylbutyratica_JCM14075T_01890_ATPase_RavA
VDIDEFVDKARQIEAEIARVMVGQKQIIRHSLIALLAGGNVLLEGVPGLGKTMLVKTLSQVLSLDFKRVQFTPDLMPADIVGTSIISEDAEGRRAFQFQPGPVFANIVLADEINRATPKTQSALLEAMQERTVTAGGKTRKLPHPFFVLATQNPLEMEGTYPLPEAQTDRFLFKLLVEFPAEEELAAIISRDMASDPGDLERIASGEEVLAMRDIAAGVPVAERVNRRIVKLVLASHPDSPYAPEIVRKYV
RYGASPRAGQALLAGARVRALMEGRYNVAFADIEELAYPVLRHRIILNFEGEAEGIKIDDLIGELLAATNDER*
>Syntrophomonas_wolfei_subsp.metylbutyratica_JCM14075T_01891_hypothetical_protein
MKWGDYEKIYDPQRIGRSGEISQVRGQAGEGPQQLIETDDPAIMPGSMRPYREVLAEYSQAARESLDRQPIPSGMTKVVKNYFSSLEE*
>Syntrophomonas_wolfei_subsp.metylbutyratica_JCM14075T_01892_hypothetical_protein
MSFKDYGDKRIIAALRPVLRPVLRIQLQHYLHWLLRGLAGALLLSAFLLLLSFFFPWVGVKQLCLYTLGASLLLAWGWAFWRRPDLWTAACMSDAKGLQERVSTALELSAEDSEMIRRQRRDARKQLQAFDFRTAFPFIFPAREGKIITVLLLVGLFCFLPNPQQAEVDRAIAVKKEIVRQEKKEIEAVKKEIQAKSGPIPVKELEKTVSSLEKLQKDLQEAGDMKKALKSLAQAEETLEQLRLDNLRQGRL
NAPALASFKPGQERPEAATEDSKDRDNGRDEPKAPGSEGEESPGKEGKGDSSSDTGANAGSNSLEPVNHGGSASNIKDSAATRQEGRAAAMAELNRALRAVQNSRQALLAAGSASPSQWASSQGYGSNTADSSGGSGNNTAGPSAGSSGSGAQGGANSAAPDSCSSGSGAGGGT*
>Syntrophomonas_wolfei_subsp.metylbutyratica_JCM14075T_01893_gliding_motility-associated_ABC_transporter_permease_protein_GldF
MKSLVLNPILAKEFCSRMRNWKSPLMISLYLALLGLITLAYYWMQQKRGFYSGFGPDVGPQIFIILSVFQLLLLSFVTPALTAGVINGERERQTFDLLLCTRLSSASIVLNKLLSSISYILLLVIASLPVFAIVYFFGGVLLSDMGRVILVSLATAITFGVIGIFCSTLFKRTQVSMVVSYVVVLLFLVGTLAVTVFLQGIGTGPNSQVTAQYFSYANPLVALFSIFPNQAAGVGIMSSFLGGYRTYGYNAAASQALS
PWRYNFIFDGVLIIVLLLLTVFLLDPVGRFKRLRKNPAVVETEAESAPVQG*
>Syntrophomonas_wolfei_subsp.metylbutyratica_JCM14075T_01894_putative_ABC_transporter_ATP-binding_protein_YbhF
MLQVEELSKSYGKFLAVDRLSFNVEEGAIFGFVGPNGAGKTTTMKILATLLRPSSGKAWIAGEDVSQNPRRIRQLVGYMPDFFGVYDDLKVDEYLDFYSASYRIPTRQREKIISDLLELMDLEHKREAYVDSLSRGMKQRLCLARCLVHDPALLILDEPASGLDPRARVEVREILKELKNMGKTIIISSHILSELTELCTHLGIIEAGKMVACGSVDEIMHRSQSAQAIEIRVLSQIDEALLLINNELGYSAIKL
DEQMLELPFNGDEKEMNRILTHLVQAGIPLVSFAESQSNLENIFMQLTKGVVQ*
>Syntrophomonas_wolfei_subsp.metylbutyratica_JCM14075T_01895_hypothetical_protein
MKKMMQLSFSILLGLFLLLGSLPPATAEAAGQVEMTAEAGFKGFARWGQAFPLQVTLVNPGPEIRGRLVFDTNNNELRIQQQKEVVLPTGSKKRVSMFIPSNSATNYEIKLMAGDQEMAKCKSRVTIMQPQEMLVGVLASGPSTLDYLGGIKFPAAGQRISVTHLEAKDIPEKSLLLDNLDILALNDFSSSSLSKGQLKAMEDWVQRGGMLVLGGGPNWKKTFFDLPGTLWPVGISGTRQLSAL
SGMEKLAGERLEVKKPFIISEGQLRSGKVLLSSGEIPVVVEDKLGRGNILYLAYDLALQPFGNWTGNNAFWSNLILRINPHQMAGSSGRYVSSVQDMGWILRNFPASDLPSAGWLALVLLAYIVLLGPGIYLFLKKYDRRDWAWALIPALAVLLFSMTYLGAFKAKGRDVFTNVITLVKLQPDLDRAEASTYAGVFAPTRKEFSFELQGQQVVDVIGGDMGHMMRRTVSVMGGNNSSQFPLLATVS
QDERTRVSFDDASRWSMRCIALDENIEQAGRIEAQLETSRGKIKGIVKNKSDRTLSNCIVLNRYGYQRISKILPQGSVEVEFAPRFSPNRGPAFYRVFERYPVYRPRGYQEEANWERQWSSQILENASRNGQILDDAIMFIGFSKAAASQVFVPENRGKVNYNSIYLAPLELKLASEKALSIPPGIINGRFTATDGRNFNQDPWGIGCEGGFLEFELDLPFSREEVKIDEFKLYVGSNDYRRAYLMKLMVYNF
HKGRWEDLSYQPGGMLLADWKPYVSEKGSLQVKVGSGDKGPGYINISGVTLSLEGEYLSPQAVPAAPGGNNMEGGE*
>Syntrophomonas_wolfei_subsp.metylbutyratica_JCM14075T_01896_hypothetical_protein
VKKRLGIALGGIVLLLIAWLAFDHYYYFSPGTDHSRIKQEAEKILQKEGPKLLKNNSLGMRPDMEKEMKWVQVLDIRELGSKSYLLFKLEYGLKGANMDRMIYEYGGGNGYILGLRQVEKKLGRLSLEKGMEFETNYLSAPVSCGLDADGIFYGYCKNPQVAKVRLETGDGKIVECEAKDRIFLAAVPGKNQDISPFFFNAQGEEMEPSYGFKVAFVSNDEKYYQQYTNSPMEWWNMSAKDIGA
GSLPAGSMDAVWVFADQQQEALQGQTLKKLKELAEGGVPVIFIEMKDSNKLLRAFQVKEAPGKVAASEVEALYLGKDKSQELQVGFIVLDEDELSPILQKSINLRYQLDEDQQKVAGKKAAASKATTGVKQGTTAVPDAPAAPAPVGRVKP*
>Syntrophomonas_wolfei_subsp.metylbutyratica_JCM14075T_01897_Antitoxin_HigA-1
MSRIKNIHPGEVLNEEFLKPLGITPYALAKEIKVDQTRISEIIRGKRRISPDTAIRLGKYFKTSPQLWLGLQNDFDLEEEILKKRKEFMDIKPHRSAQNRAKKYSMH*
>Syntrophomonas_wolfei_subsp.metylbutyratica_JCM14075T_01898_hypothetical_protein
MPFAPDKNGQSHNAINIDAQHKLDERQRKKLQHEFKIYMEQYFKEKLGKGVDYTKIIIWEDMVIVRGERFLTEPEQFIVKTAAGKEVVRAARMQVARQHSIDNVPHFEEKLQARVIHQTYDIEPENDFWIHVMVYDRILTK*
>Syntrophomonas_wolfei_subsp.metylbutyratica_JCM14075T_01899_putative_zinc-type_alcohol_dehydrogenase-like_protein_YjmD
MADTMKALVYRGPEKIGLEDVPMPKIIEPDDAIVKVTTSTICGTDIHIWHGGMPEVEPGRIIGHEFVGEVVEVGPAVRNNKVGDKVAVSCVTQCGECFYCVRGVYSHCTTGSWIFGYMIDGCQAEYVRVPHANLGMFTIPDGLTEEDVLFVGDILSTGYFGSEQGRIEPGDTVAVMGGGPVGMCAMTTARLWGPAEIIAVDINQERLDFAVKNGIADVGINPQKVGNVGDAIRERTGGRGAD
VTIEANGFKPTFDMAVDSVRPGGTVSLIGVFEKPQELAMNTLWIKNIRISMGLVNANRIPELIKLIQKGKIDTNFLITHRAPLNDILKGYDIFGGKKDGCLKWVVTPYKK*
>Syntrophomonas_wolfei_subsp.metylbutyratica_JCM14075T_01900_YcfA-like_protein
MKPKELMKILAEDGWAIVRKQGSHYIHKHPTKPGTIPVALHNKDMAPGTLNKILKAAGLK*
>Syntrophomonas_wolfei_subsp.metylbutyratica_JCM14075T_01901_hypothetical_protein
MVYIYPAIFTPDEIEGRGDVYTVDIPDISGCITEGESIEEAISMAREAIAGCILAMKQAGEHIPAPSNYNDMKMKIMLDREFVTLIDVDLNDYLRKKETKSVIKTVSLPQWLAIMAEEAGISYSQALQETLKDRLGL*
>Syntrophomonas_wolfei_subsp.metylbutyratica_JCM14075T_01902_Nuclease_SbcCD_subunit_C
MKIKQIRFQNLNSLVGEWIIDFSHPAYLSDGIFAITGPTGAGKTTVLDAICLALYGRTPRLERVNKSSNEIMSRHTGSCYAEISFENREGYYRCHWSQQRARKNPQGELQPPRHEIADARSGQIMENKIRDVAARVERITGMNFTRFTRSIMLAQGGFAAFLQAPADERAPILEQITGTEIYSEISMKVHERQREEKNRLEILQAEISGIKLLSTEEEKALRQDMQTKIARGKERQEELKGLQATLDWLDR
MIGLENEIKDIEAQWQQYLKRQQAFQGEAWRLERAWQATVLDTPYIRLKDLRDLQKSDLSEIERIREELPRYKEAAGKAAERWENTRKELIQLNLLRKSEGETAKKVREMDILLKQQGQVIKGMEMVIGAGESECAALQGEITAGVEKAKEMGQALLAIEEYRRGNARDAALLANLTGISRAFKRLEELLSAWQQKGLERQRAAVALQSLLDAGEEIAATHESNKELQRQQYDVLQAEQDKLEEA
LQGYEIAWWRDELEQFKEQDDKLQNLGQVWDRIVQLEDNIRDGRESVQHLDKQKQLVLEIEQGEKGAGHLAARIDDLETRVLLLNRIRDLEEERAQLEDGKACPLCGATEHPYAQGNIPQMDESQKELNQARKQHKKLSEDLKKKEIQRGKLDKELEHASAAVEQAKKQLEKESKMRQEGMNDLGLDISVVKTRDELERLANQSRQNSINHARQLKVIEKWQKNIGTLTLHYEKSRTAFI
QSDKELQAAILQQKQALQEDERLQKEYLSKSELEQLQEETLQEISPYGMVNLPLEKLAAIQKALDERKKRWEAKEKEKLDLEKTVDKLQNEVGQKRILLEDKASALQIEQKRLKQQREQYRRQQETRFALYGERDPDEEEARLDEEIHLGEEMVNRAHEDLQELQRRQLRLEEREKTLAVAIQNRAPELQSSEENFTRKLQQAGFEQEEDYRRASLSPEEQQALAQKAEALKKEATELQARLLDR
QDKLQREKEQKLSGEAREVLEEKQRLGEQELVDLREEIGAARNMLENSEKSRRQMQAQLQRVEEQKKELQRWQLLHDLIGSADGKKYRNFAQGLSFDIMIKHANRQLQKMSDRYILLRSQEQPLELNIIDNYQAGEIRSTANLSGGESFLVSLALALGLSQMASRKVSVDSLFLDEGFGALDEDVLDTALNTLAGLQQDGKLIGVISHVPALKERISTQIEVIAQFGGRSILQGPGCRLQ*
>Syntrophomonas_wolfei_subsp.metylbutyratica_JCM14075T_01903_Nuclease_SbcCD_subunit_D
MKIIHTSDWHLGCSLRGRKRYEESAEFLDWLGELLEKESIDILLVAGDIFDTTTPSNRAQELYYRFLCRVAESSCRHVVITAGNHDSPTFLNAPRELLRHLQVYIVGKIGESLDDEVLLLKDAQGEAELIVAAVPYLRDRDIRSAEAGESLEDKARKLVEGIRSHYELVGRRAEEIRSALPGPVPVVAMGHLFAAGGSTVEGDGVRELYLGSLAHVGHDFPPVFDYVALGHLHKSQLVGGRENCRYSGAP
IPMGFTEALREKQVIMVDFCAGQMEVTPLKVPIFKALHSIRGDREEIERQLEELKSKAQKAWLEIIYEGKEIISDLREQMEAATAGTELDLLRVSDNRSWQQVINLMEEGEVLSELDHSEVFRRCLEAYEVEAEQRESLLQMYNEILDCLLTEDVMAE*
>Syntrophomonas_wolfei_subsp.metylbutyratica_JCM14075T_01904_putative_CtpA-like_serine_protease
MKNRNSAVLIILSLFMLLLTPLGSAIPANAADAVDNSYLQEMMRFIKDRYYFEVNEEQITQGALKGMFQGLDDYSDFFTREEAQAMLESVEGNYEGIGVALSKVDNYIVITRVFPSSPAESAGLLSGDRIVTVDKKDVMGYSVEQVSTLIRGQKDSLVNLGIVRSGIKNVMEFKVKREDIKLNPVRYENRGGIGYIAIDSFNSNTAEFLEALLEAAEKDGISRLVLDLRDNTGGEVTQALAVARHFVPAG
PITIKFKAAGMKDKVYTSELPSPPYKLVVLVNGMTASASEIVAGAIQDSQAGVLLGNKTYGKARVQAVVPLLSPEAYQKYARLYPDSSLDSNEMLIRHGIQATDREIMGWAKITTGIYTTPSGKYIDGQGLLPNIKVSDPTPINGLEISNLMPLEKGGKPGLDSSSLEVYNAEKILFASGYDLDKPDMTLDIKTFNAIKKFQQEQRLYPSGILDFTTQEAMNRKRQELINRYDQQYAQAVEYLQGR*
>Syntrophomonas_wolfei_subsp.metylbutyratica_JCM14075T_01905_Methionine_aminopeptidase
MEQDLYLEDFKRRGILVPDRKLIKTEAQIEGIRRSCQLTKSILNLVEERIEAGISTAEIDRWVNDALTAAGAYPATLNYQGFPASVCTSINEVICHGIPSERKLKEGDIVNVDITSILDGYYGDASRMFMIGEVSAFAQKLVKIARECLYLGIEQVKPFNRIGDIAFAIEQYANKHGFSVVRDFGGHGVGLAFHEDPFVAHYGPRESGMLLVPNMTFTVEPMVNVGTYRCRKLDDGWTTVTADNSLSAQ
WEHTVRVSETGVEVMTE*
>Syntrophomonas_wolfei_subsp.metylbutyratica_JCM14075T_01906_hypothetical_protein
LKKNLSLTLCLLALFLLTAVIAGCGPAKESSAPADKEASAPSSDKSSGSEPVLKDLLGKGKAVNEMSFDCETQVGEQKMLVKTWIKGKKVRSEMESPEAGGKIINIIDSAAGVVYVYQPEQKVATKMDISLAQQDGSSSPQEDLEAMNPDKMKYLGKDTIDGKKCLVYEITEDNIGSSKVWLWEENGIPLRMEATADGEKMVIEYRNIKVGGIPDSMFELPKGTQIMEFNMPTMPTP*
>Syntrophomonas_wolfei_subsp.metylbutyratica_JCM14075T_01907_Succinyl-CoA:coenzyme_A_transferase
MNYKELYQRKLVPVLEALEAVRSKQEIVCSLAACEPVTLLSNLHHIKDKVENVSVVNAMMMGEYEFFMNPELAGKFLLNSWFYSKAPRQAHPQGNVSYVPLQLHQFSSKRTFHRRPNVFFGCASPMDKHGYLSLSLSTVLEKDFVEQADVVIMEVNPHLPRTFGDTHVHISQIDYIVESEREIPTMDPPVLTEKDHLIGAYAADLIEDGSTIQIGFGSIPGAVAYSLRNKKDLGVHSEMFSDCILDLY
EAGAISNRKKTLWKDKFITDVGLGSRRLYDFLDENMAVEFHRGSVVNDPAIIGRNHKMVSINSMLEIDLTGQCCAESVGSRLYSGTGGHKEFVTGAQESPGGKSILAFYSTARDDKVSRIVPFFDPGTVVTTSRVDVDYVITEFGVACLRGRSIRERVKELVNIAHPNFRDYLKSEAERHMIW*
>Syntrophomonas_wolfei_subsp.metylbutyratica_JCM14075T_01908_Glycine_betaine_transport_system_permease_protein_OpuAB
LFPSDIEFHVAPYISDFVKWITVAWEPFFTAFSNMVLGMLLQIEHILVAIPVWWLWIIAVTVIAWRQTHEVVKTIMPGLLLFTVGIFGFWGIAMETLGIVIVSVIISLLLGIPLGIAMAVSDRCNTLVTPILDAMQTMPSFVYLIPALMLFGLGKVPGVVATVIYALPPVVRLTNLGIRQVSSSVQEAALAFGATSWQLMKEVRIPLAMPSILAGINQTTMMALAMVVIASMIGAGGLGEVVLICTNRIDVGG
GFEAGWAIVVLAIVIDRLTQGLVRRWDTPGV*
>Syntrophomonas_wolfei_subsp.metylbutyratica_JCM14075T_01909_Glycine_betaine_transport_ATP-binding_protein_OpuAA
MGMQDFNLLEVERLWKVYHKTEKALDLSDPVAIEKAEAAGAVIAVRDVSFKVKRGEVFVIMGLSGSGKSTLIRCLTRLIEPSTGVIRVKGKEVTSMDAEELTAFRRGEAAMVFQHYGLLPHRNVLENVAFGLKLRGLARELRESRAQEALRQVGLAGWEKKYPHQLSGGMQQRVGIARALVQDTDLLLMDEPFSGLDPLIRREMQDELIKLQKEWQKTIIFVTHDLGEALRLGDRMAVMGAGV
FVQVGRPQEIVMNPADEYVQRFVQDEQRIAQLLDKGRVSRVRSIEAHASARAGR*
>Syntrophomonas_wolfei_subsp.metylbutyratica_JCM14075T_01910_glycine_betaine_transporter_periplasmic_subunit
MQQLQILLRRSRSRCLLLAFLLLFCFSFLMGCGSKTGGDSGTQEGSDKPRLVLVDSWDSIKVHNRIVGYILQHGYGYPEPEYLFAETLPALQGMVKGNIDIYMEVWADNIEEAWKEALASGKVKNLGPNFPDAPQGWYVPTYMIEGDAERGLEATAPDLKKISDLPRYWELFKDPEVPSKGRFHNSPPGWNCTSINGVKIKSYGLDKNYNNFVTGSDPALVASMVSAYEKGEPWFGYYWEPTW
VMGKLKMTRLEEPDFDDAVWQDESNRACSYPSAQVLIGINSELEKRAPEVVEFLQNYETSLEQNNDFLAYMSDHNNNEKAAVYFLQKYKDEWHGWVPADVAAKLDQALEEVK*
>Syntrophomonas_wolfei_subsp.metylbutyratica_JCM14075T_01912_hypothetical_protein
LDNLETKVDKVDASQVRMENELTEKVRALFDAREVQNDINERIIMALGRIEAKVDVLQLETAHLRRVK*
>Syntrophomonas_wolfei_subsp.metylbutyratica_JCM14075T_01918_Arsenic_efflux_pump_protein
MEWQIWFAGILFLIIYTVIISEKIHRTIIALWGAALLIILGIILTQQEAINYIDFNTIGLLFGMMVIVGITRKSGIFEYVAIKAAHSTGGDPVKIMITLSIVTAVASAFLDNVTTVLLVVPVTISICSVLEVNPIPVFVAEIFSSNIGGTATLIGDPPNIMIGSAAGLGFMDFIKNLTPVIIVIFIVTILLLKLIFKKDLQSSKKNMTKLMAMNPDDAIKDQVLLKKSLLVIAVVIIGFVTHQYLGLESATIAITGATLLMLINR
QDPQEALLTVEWATLFFFVGLFILVGGLEKVGIIEGMARETIKTIRGDVTLSSIFILWFSAIASAFIDNIPFVATFIPLIKEMGQIGHMNIMPLWWALSLGACLGGNGTIIGASANVIVSGIAAQEGYPMTFIYYMKIAFPLMLVSILISMVYLLIFFV*
>Syntrophomonas_wolfei_subsp.metylbutyratica_JCM14075T_01919_Ktr_system_potassium_uptake_protein_B

MNNHNLTAPQLLSVSFLCVIILGSLLLSLPCSSQVPTSYLDALFTSTSAVCVTGLVVVDTGTHWTCLGQTIIMLLIQIGGLGVMSFAGLFALLLGRKIFLRERLLMQKSINVSALGGIVKIFKYLLGFSFGVEAVGAVILAIHWTPLMGLKKALWFGLFHSISAFNNAGFDLFGNFKNLTAFTSDITVNLVISSLIIIGGLGFYVCYEIFHIRKLGKLSLHSRVVLYTTIFLILSGMVLLFLSEYHHALKDMSLGTK
FLAAYFQSVSPRTAGFNTVDLNSLLLSSQLLMIVLMFIGASPGSTGGGVKTTTLALIGATVLSQLKGKRESEIFERRIESNDIYQAITVVVLAAFVLVIITYLLALTNPGSLIKIAFEVASALGTVGLSLGITASLSGFGKILIIFTMFLGRVGPLTLGFALAYKPEQPKIQYARGKIMIG*
>Syntrophomonas_wolfei_subsp.metylbutyratica_JCM14075T_01920_Ktr_system_potassium_uptake_protein_A
VKNKQFAVIGIGRFGESLIKELTRLGYEVLAIDVDENRINEAVGIVTHAIQADSMDENTLKAIGIRNFDVVIVAIGDNIQSNILTSIILKEMGVRTIVAKAQNALHGKVLEKIGVDHVIYPERDMAIKLARSLVSHNFLEQINLSSTYSIIELFTPHIFINKNLTELDLRKKMRISILAIRRGEDIIVAPSPDEKVLPGDILVVLGNNADLEAISKLD*
>Syntrophomonas_wolfei_subsp.metylbutyratica_JCM14075T_01921_Cellulosome-anchoring_protein_precursor
MIIFKQKPKWERIIVANLLVLFLVIQPVSSVMAFSDVEWHWSKPVVTRAATLDFIKGYPDNSFQPEKEISQMEALVLFMRAVGLGNGKTTAKKSKKEAPRPLKSPEVPWGQNYMDTAVENELLDDKWLSSFQADRPATRLQVVSLLCRLLQLPLPDSVSSGQNAFSDLDSTSSEYIPYIIALSQAGIMKGYEDGTFRPERGLKRSEAAALLSSLIEQNWVKTDASRILEGWVEKIIVDKNKGEIELRSLSG
VKKVKLDPGLQCFAGVGECQLRDTLDYRVEVLLNQKKQAACISIIEKRKFIAAEDKITGTVKSVLIGLDSILVLSDLNCEERRLPLSWGALIDSEGKARVKDFTSLKPGTYVTVQLAEGQVQKVTVLETKTISGTIKDLSGRRLTLAKKGSSSKSGKPEWFNYWDRARMVDKDGRVIGRVRSRGDAIKVTYLDPNPGEIDDEIPLEIMLSSRPGLKKVKAEVEKLTILGDYYKLTVKKNKDYEVDKSVQLY
SSSGGEIQFNNLNAGDKVEMEVDGAGVVIKITVL*
>Syntrophomonas_wolfei_subsp.metylbutyratica_JCM14075T_01922_outer_membrane_biogenesis_protein_BamB
MMQLMKLGVINNSRKNFYPGKALGCFAAGNRELWEYVDHNENFCFREVDYINDPRVIAFRGGNKELIRGFSNRMKIALFMVLFLLLAFPSSSEAASKLEQFPAAKEAEQPGQLLWKLSGLGKLSSDIIFAPNGNLLFPSNNKLNCVNPEGKLIWEMQGKGKGSLGSPILTEAGAVFFASGSVLQEGKLNGVLGWDFTVYGEGKAQKTPLLARGPGNILYLPLPDALYAVDTVGHYVWMLSPWESS
DGKTSKLINPRTFLACTADEQAFYVVFGDKAGKYKLAAINAKGEYLWKYWLGDISSAFLLTDNEGKLYASVNFKKAAQGSKGGKLNQSKVYCFNNSSGNSPLWSNSFSAANQITAPVLGNSKTLYFTGGNKLYALDTEKGKVLWETPLLKLVSTPTVSPKGYIYGGSSEGYLFAVKPSGRMAWFRQLDGAVERAPLIGPDNFIYIHTAKGSLYKIKDNFKES*
>Syntrophomonas_wolfei_subsp.metylbutyratica_JCM14075T_01923_butyryl-CoA:acetate_CoA-transferase
VIVVKKLSHAGSWREDYRQKLCSAEDAADLIKNNDIITMAGGTSIPKGFATALSKRARELFNITLLQGFSLCNYEFMQPQFKEHIHIETSFVGLMERKCIQMGLATYVPIHLHKLPAWLDNRGPNVAAHAVTPPDENGYMNRSCYAGLCYRRSIENAKTVIVEVNPNTPLVMW*
>Syntrophomonas_wolfei_subsp.metylbutyratica_JCM14075T_01925_PAP2_superfamily_protein
MCLSGNYSAFSRLFYFFSNLDLRVQDYVQTINSPCLLKIMIIITYLGSPWVFVILSALTSSYLWFRGKKLETIFLNVTLFSAWGIMEGLKHLFKCGRPAGQALTMARGYSFPSGHAMLSLAFYGFLAALLLKYGGRGNKQAWLGAVALYILVLLIGLSRIYLNVHYLSDVMAGCLFGILILILSLKAMKLVQNRL*
>Syntrophomonas_wolfei_subsp.metylbutyratica_JCM14075T_01926_hypothetical_protein
MPGYQVKLKENQTSQWGVWLNIMLDNIDIIFRNLIAVGILFLVTLVIGKKLISQLNFFDFIVGITIGSIAAALSVDKTITYSHGIISLLIWGLIPLVVAKIALADIRARRRLDGVPTLLVQNGKILEDNLKKEKYHVNDLLEELRLKGVFNIADLNFAILETSGQISVQLKPEKQPVTVSDLNLSATRQGLCANLIIDGKILYQHLKLVNRDEIWLREELKKQNIEDVGQVLLASMDGSGNLYIDVKDDQVKEF
NVLK*
>Syntrophomonas_wolfei_subsp.metylbutyratica_JCM14075T_01927_Ferrous_iron_transport_protein_B
MDEKRIVIALAGNPNSGKTTIFNNLTGARQHVGNYPGVTVEKKEGWLKHRGFEIEVIDLPGTYGLSAYSPDEVVARNVIVNDKPDLVVNIADASNLERSLYLNTQLKELEVPIVLVLNMADVAEKNGITINYELMSQLLGMTVVRAVGTKNEGTVTILDAVVNTFAGEDKPSERMLRYRPEIEEEINSLLELPFAGRQSGSPSSEAAAAAGNLDGILNYPLRWTIIKLLERDRELTKKLENNSEGKVLLK
QIEQSRQRITDKTGDDPEMALVEDRYAFIRGVCREASQITVISKQSLTEKIDNVLLNRALGIPLFLGMMWLLFQLTFTLGTPPMEWIEAGFTALGGWLNEVMAEGLLRSLLVDGIIAGVGGVIVFLPNILLLFLGIAFLESTGYMARAAFVMDKVMHQAGLHGKSFVPMLIGFGCTVPALMASRTLENPRDRLVTMLVTPLMSCGARLPVYTLLIGAFFAPQIAGNILFSLYIIGIVLAIIMARVFRTWLL
PGESEPFVMELPIYRLPTLKSVLIHMWERAWLYLKKAGTIILALSIVMWGLFTFPTVDKEGYEFESAVEQVENSYAGRMGKVIEPVLRPLGFDWKTGVALVAGLGAKEIVVSTLGTLYSIEDEEGLAEEEEPVVKSFAQRAREQSGYSPLVAYVLMLFTLIYVPCLAVVAVMKRETNGWKWPLFTVGYTIVLAWVVCFLVYRGGLLLGIG*
>Syntrophomonas_wolfei_subsp.metylbutyratica_JCM14075T_01928_ferrous_iron_transport_protein_A
MMSKTLKDLAPGEKARIKAVSGNGSVRRRIIEMGLVPGVELTMERYAPLGDPVEVSNPGFFLSLRKEEADTIVLEENAEEEQ*
>Syntrophomonas_wolfei_subsp.metylbutyratica_JCM14075T_01929_hypothetical_protein
MNLPTEIQILKLVITKLEELNIPYMLSGSLAGSFYAQPRMTRDIDIVIELAAEQIAKITEIFSGDFYIDEDDVNEAVRLQSMFNIIHYQAVVKIDFIIRKDSLYRKLEFERRIRKIIAGFALWVVSPEDLIISKIFWARDSKSELQLNDVSSIMKYQAKKLDWEYIEYWVKELGLGQLWEEIK*
>Syntrophomonas_wolfei_subsp.metylbutyratica_JCM14075T_01930_hypothetical_protein
MNDTSSTVENKLFEMMQQKTESERFFMAASMFDMARLVSKAAILEDKPDISPDEARVELFRYWYWEDFDTDDRENILQAIRLINSPYD*
>Syntrophomonas_wolfei_subsp.metylbutyratica_JCM14075T_01931_YibE/F-like_protein
MHKYRQKTFIRSTLLLMFSFFFTVSILAGPAHGQLHPEFDEEQYQERYEKALVLEIKDKSVKEIEALQGNIVEQEVKVRMLDGADKGKILTAINTLSGSAGFDMHLSPGDKVVIYIAEFTPADGGEAVKTIYVSDRVRSPALLWIVGVFAGLLLLIGRWQGAKALFGLGITILGIYKVLLPGLIAGKSPLPLTVIVLIVVTLITMICIAGFSRKALAATLGTLGGLIVAGWLAYIFGDLAQLSGLATEEERMLV
YVENLNLDMRGLLFSGILIGAVGAIMDVTMSVASAISEIKRANPEMNTAELVAAGMNVGRDIMGTMANTLILAYTGGAMPFMLLFMVYEMEPLRIVNWELIVTEIIRALVGSIALIISVPITAVIAGWLTATRKV*
>Syntrophomonas_wolfei_subsp.metylbutyratica_JCM14075T_01932_hypothetical_protein
LSNENIVEALKDTNKKIADLKSFNIPIILKTIEEYEKSGVEECFIEQQRLQLQKVYARINELEAKAERLFNRLE*
>Syntrophomonas_wolfei_subsp.metylbutyratica_JCM14075T_01933_hypothetical_protein
MELSVECLPCLVNQAVRLVKIHLDNEKAQRILVKKIMLEIGASDDTASTPYIAHKMQQVLKDVLQNPDPYRDVKLYYNSEMLKLEKEFLAVIRSAEDQLGTALKLAAAGNIIDFGPGHDLSRAEVIKVLNQTLEKEFNHEVFTSLRSKLQAARKLLYLGDNAGEIVFDKMFIRTIKAEYPDLEVDFATRGAPVLNDVTEEDAYFIGMNAYAHIINNGTDIPGTVREYCSASFNLAFDNADLVIAKGQGN
FESLYGTGRVNLYYIFLCKCNLLMERCGVLQNDIILMKE*
>Syntrophomonas_wolfei_subsp.metylbutyratica_JCM14075T_01934_Flagellum_site-determining_protein_YlxH
MSENCDQDCSICGDDCTERKEPKTDFSEKPHELSNIKKVIGVVSGKGGVGKSLVTSMLAVTMNRKGYNTAVLDADITGPSIPKAFGVKEKATANALGLLPIKSKTGIDIMSINMLLEDDTNPVVWRGPLLAGAVKQFWTDVIWYDVDFMFIDMPPGTGDVPLTVFQSLPVDGIIIVTSPQELVSMIVSKAVKMAEKMNIPVIGLVENMAYFKCPDNNKDYWIFGESHIEEVAEKHKLKLLARLPIDP
KISEACDKGMIEFYDGVWLNGIAEILEKTEEKEMLKIAVANENGMVTEHFGHCESFMIFDTEKNQIIKSETIANPGHKPGFLPIFLNDKGVKVIISGGIGSGAIDIFNEKGIEVIAGARGDAKAAVEQYLQGMLKSTGSVCHEHQYHDECGH*
>Syntrophomonas_wolfei_subsp.metylbutyratica_JCM14075T_01935_Ferredoxin
MYIDDTCIGCGLCLESCPVEAISLVENKAVINKEICIECALCADVCPLEAIQQED*
>Syntrophomonas_wolfei_subsp.metylbutyratica_JCM14075T_01936_hypothetical_protein
MPRPRKRRKVCCLPGNSRFGPLDSPANTKNFVNMTIDEYETIRLIDLERFTQEECANRMNIARTTVQGIYNEARKKVAESLVNGKVLKIEGGDYRLCDGSGDFCGSEGCHRHRHGWGK*
>Syntrophomonas_wolfei_subsp.metylbutyratica_JCM14075T_01937_hypothetical_protein
MPRRNGTGPLGMGAMTGRGLGACTGVNAPFYGSEFGRGCGRGFGRGFGRGRGFGFGRGFGFGAYNNYNYSQTASKEVLQAQKEQLQITLDAIDKRLGSL*
>Syntrophomonas_wolfei_subsp.metylbutyratica_JCM14075T_01939_putative_secretion_ATPase,_PEP-CTERM_locus_subfamily
MFESFYGLERTPFSRDIPTDQLYQSHMLEETLGRLEYTAQRQMFAVLTGDCGTGKTTTIRKLKEALDTSRFMVMYLADSKLTPRHFYKGLLEQLGCEAKFYRGDAKRQLHKEIELMQGIHHLQPVVIVDEAHLLDKEMLEEVRFLLNFKMDAQSPMALILVGQLSQKSDNCPYPSNYFILNIIVKA*
>Syntrophomonas_wolfei_subsp.metylbutyratica_JCM14075T_01940_Integrase_core_domain_protein
MRDQKKADEIAEQRVQILSPLLAEGLDSAQARQIKARICEQTGISERTLRRYLAQYRKDGFSGLKPKGKGERRSDEAIPVNILEQAILLRRQVPSRSIAQIIQILEWEGLVEPGQIKRSTLQEKLAERGYSARHMRMYADTGTAARRYQKKYRNQLWHSDIKYGPYLPIGKDGKKKQVYLVTFVDDATRYILHGQFYPTLDQVIVEDAFRQAILKYGVPEKVYFDNGSQYRTKWMKRACAKMGIRLLFA
KPYSPEATGKSERFNRVVDSFLAEAVLEKPQTLDKLNQLFEVWLEECYQNKPHSGLEGNISPQTAYRSDRKSLKFLDPETIASAFLHCEERKVDKAGCISFAGKKYEVGLTFIGCKVDVIYDPADISELTIEYEGHKPFKAKELVIGERSAPRPKLPEHLQKAPADSSRLLNAAARKNQERKETQIPAVSYRKVRKEGESHV*
>Syntrophomonas_wolfei_subsp.metylbutyratica_JCM14075T_01941_hypothetical_protein
LEQLPCPCCSGQLKVIGSRQRKCINASGEKIILIIRRLRCIECNRIHHELPDMLVPYKLHVSESIEAVVSNDSELSVISDESTLNRWRKWFNEKADYFQGCMESITIRYGSESVEDKSDLPESKLQRIWHYVGDAPGWLARVVRPIANLNLWIHTRLAFCP*
>Syntrophomonas_wolfei_subsp.metylbutyratica_JCM14075T_01942_Acyl-CoA_dehydrogenase
MAFGFTYNEDQIALQKTVRKFVENEIVPVRASYDESEEFPWPEVKKMFELGLNCMNAPEKFNGMVFGNVGLCMVVEELCRGCLGIAISPIANMLASEPVLYGGTEEQWDYWYPSICEEGKLAAYAVTEPGAGSDVAGLSTTCKKDGDYYILNGTKIFITNGKHADKLTVLATHDKSKGNRAQSFFMVDTKTEGVSFGKSEHKMGMRCSETSEVILDNVKVHKKYRLGEEGDGFKLAMKAFNSS
RPFIASASTGVASAAFEFARDYAKERKAFGKSIADFQAIQFMLADMAMDIEASKLLWQKAAWLLDEKLMAADLSAMSKCFAADAANRVVTNAVQILGGYGFCREYPVEKMMRDAKILQIFEGTAQIQRVIIGKSVLA*
>Syntrophomonas_wolfei_subsp.metylbutyratica_JCM14075T_01943_Glutaconate_CoA-transferase_subunit_A
MNNLFDKRPRLARSNKVVSLSEAIDEIRNGDVLMMGSFVDTRRPMAAAYEIVRQQKHNLILLAQCSLAEDIMVGAGCAIAWRGCYTGIATFGLSPATLRKTEAGKFFPDEIGHLDIIYGALAAMVGSPFVATRATLGSDVLNDQLDRNRILIDKAGNKAMIPKKKFELIEDPFYNQGLVKLLPAMPADAAIIHTQMAGEKGTARISGSLAFDHYFVHAARTVIVTTEQIVPEEYLRRDPNRNQIPCTA
VDMVVEIPWGGHPSQVMGYYDTDIPFIRDYVLSARTDEGFERWVGEWILDVKSRDQYLDKLGASRLEGLRAADPQGYRSRS*
>Syntrophomonas_wolfei_subsp.metylbutyratica_JCM14075T_01944_Glutaconate_CoA-transferase_subunit_A
MKTSKVVSLKEAIQEVRDGDVILTGGIVENRRPVAASMEMVRQKKKNLVLIGTCSLSDDILVGGGCVEAYRGCYTSNGAFGISPNMKRRTEEGTIITDEIGHIDYCLGLMAGQAGLPFVASKTDIGTDVLNPAYDNLSKTAEVARDKTMLPNKKYVLMEDPFFGMGTIQLLPAIKLDVCICHVQMAGEEGTVRIDGSLTFDHLGALCAKKVIITAEQIVPESYIRQDPNRNCIPCTSVDMVVEVPWG
AHPGQVYNCYDMDIPFLQEYAKMCATQEGFDKWAAQWIFNQNGHWDYLDKLGVSRLEKLRALPPYGYVPRNKGGVK*
>Syntrophomonas_wolfei_subsp.metylbutyratica_JCM14075T_01945_Glutaconate_CoA-transferase_subunit_B
MTKEYAKIGEYSAVEAMVCHSARFLANFDGKKGLIGTGLPLVASQLAKKLYSPNMIIFIETGQADANPQELAISIMDSRLTYGSSWQMGDPEGLYPLSRNEIDFGFIGGAQIDKYGNVNSTFIGGDYNKPAKRLAGTAGAVDIGVFAKTTVILMAHQKRRVCEFVDYLTTPGWMVKKWPEGTLVRREELGLQGGPSCFISSMGICKFNDDDKVMYLDKYFPGITLEQIKENTGFDIDVSRADEADHI
LVDEIRLLREEIDPLNIYKMR*
>Syntrophomonas_wolfei_subsp.metylbutyratica_JCM14075T_01946_Acetyl-CoA_acetyltransferase
MTNKEVVMVSACRTPIARFLGSLKDVQAKELAITVGKEAIRRANVDPSMIDEITMGEVYPHMQGSLPARQVGLACGLTVESSAVNINQNCTSAMRALEIAAHNIILGKTEIALVVGTESMTNVPYMLAKARMGYRLNAGILEDALIQDALFCGFTGGHMAITAENVAEKYGITREECDELGLISHQRATAAVQNGTFKREVVPVEIKGKKGKVTYYENDEHMIPDANLEAMSKLPPAFKKGGVVTA
ANASGINDGAAGAVIMSKEKAEKLGIKPLMKLINICGAGMEPTLMGLGPAVAIPKCLKQANMKFEDVEYWEINEAFAAQWLGVGRMLKEDYGMNLSLDKVNHNGSGIALGHPVGATGLRIIVSMYYEMERLGLTIGGASLCVGGGPAMCSLWTRNI*
>Syntrophomonas_wolfei_subsp.metylbutyratica_JCM14075T_01947_putative_3-hydroxybutyryl-CoA_dehydrogenase
MAIKKLGVLGAGTMGAGIAQVGAQSGLQVILVDLEQAFVDKAIKGMIKNWDKDLSKGKITDQDKTNFLKNLKVSTNNNDFKDCDLVIEVIVENIDVKKKVFRELNDICAPRTVIASNTSGFSITEIAAASGRPDKVVGMHFFNPVPVMKLVEVIPGAETSQETIDLAMEACKTINKKAIPAKESPGFIVNRILVPYLNDAAVAYGEGVASAAEIDEAMKLGANMPIGPLALCDLIGIDVLLMVVDYFK
EFGDPKFRPALAFKQKVRAGHYGVKSGKGFFDYSK*
>Syntrophomonas_wolfei_subsp.metylbutyratica_JCM14075T_01948_3-oxoadipate_CoA-transferase_subunit_A
MKAPKIITLQEAAALIKNGNCLAFSGFTIWRRPVAMCYELLRQSKKDLHLFEVQGGFHSEVLIGGGAVKLWEGSWIGQEILGKTGVNMSRKQVAKEILVDDYSHGHAVGRIMAGSVGIPFFPTALAMVQIS*
>Syntrophomonas_wolfei_subsp.metylbutyratica_JCM14075T_01949_hypothetical_protein
MPAMHADKLDWQMFYAAYAKTYIEHDVRALAQVGDQIKFIKFMTAMAARTGQMLNLSAVSRETGISVPTADRWLSILISSNIVYLLPPYYSNITKRMVKTPKLYFLDTGLAAYLTKWNTPQVLEAGAMAGAFFKTFVVSEILKSYYNTGILEPALYYYRDKDAKEIDILIKQNGMIYPVEIKKTANPGKEHIEHFSVLNKLKGVKIGNGGVICSALEHERFPQSTMGA*
>Syntrophomonas_wolfei_subsp.metylbutyratica_JCM14075T_01950_hypothetical_protein
MTYISRHAETTADKAAKMFPVVLITGARQVGKTTLLKHMTSDIPYLTLDDPILLQNAIEEAGSFFKMTPLPVMLDEIQYAPSLFSYIKMSVDESGKKGQFFFDRFAAI*
>Syntrophomonas_wolfei_subsp.metylbutyratica_JCM14075T_01951_Anti-sigma-V_factor_RsiV
LLKRIICYAMVLSLIALSAAPALAKPAPGDFADLLEHWAQRNVSAVCNLGLMSGMGENEQGSQVFSPDGLVNRAQLALVLQRTFELDYGEKSFIKQPQAGDYYLDVDNGAWYAEAVKFCAINQVFDSAEKFYPEQAVTRIEVARAIHRSIKAKGLNIPMIMLMPYYQDMEGLSQEDSNALVFASNTSLMKGDGQNWRPQEQITRAELATVLNSLLRLLAVDESYDGQEYRLAPGHSFTLMLDSN
PTTGYSWTASYDEKVLALDARHYQQAGEGNIMGQGGKDTWRFKALQAGTTEIKMVYSRSWESVEPIKTFTLKIVIAPGQAETGKVKVSSRMLKEKSDTMDVDLEIPVISGLEAVLQSAINQRFEGDAMELKQSLETGLKAYLAECKAEGYPIRSYQLFTRYQQCRLNDKVLSLYVDYYQYTGGAHGITERRAYNIDLKSGELLPLAAMFKSGYDYKAVIEQEIKRQIALNSDVYFKGDQGFKGLNQ
EQGYYLEDENLVIYFGQYEIAPGVSGIPEFKIPLKLLSI*
>Syntrophomonas_wolfei_subsp.metylbutyratica_JCM14075T_01952_Membrane-bound_metallopeptidase
MMVRSFKLRMLLVIFLVAAAGLAMQSGESSRRIAEPVIRFMLKDYGVEQKIASFIDNLRDQGRLDLVPARSNSTLTLPCEYIAVKKNYGWHWNSKTHKQEFSPGIYLEVNRNTAVNPILDGRVIEISSNKEGRQVLLEHNENFYSLYGGLDEVFVEEGKEVAADSLIGKTGKTLYFEIRNQDGPQDPTPLFE*
>Syntrophomonas_wolfei_subsp.metylbutyratica_JCM14075T_01953_Putative_zinc_metalloproteasec/MT2700
LGIPIRVNPLFLLLAILYSCLGLGREIIIIILAAVFLHEIAHTLVALMLGIKVAEIEILPFGGQAQIEDFTGLDPSKEICLALAGPAVSLLLAGLFYFLQPYLNVDLRFFVNINLLLGLFNLLPALPLDGGRVFRALISPLLGFKRSTKIAAFLGQLIALLLFAYGFYLSYTRFTGANYIAIGVLLFWAARREGKLLLYSFMRYLINKKAELARKGFLSCNQLVAYTYTPVKEILNAAGPSYYLVVVVLDEKHHPRGMISE
VELIECYLEKGPRVSLEDCQ*
>Syntrophomonas_wolfei_subsp.metylbutyratica_JCM14075T_01954_Ribosomal_protein_S12_methylthiotransferase_RimO
LKDRLFRDILPRVAKPARYTGSELNMIKKDWEQAGVKMVFAFPDVYEVGMSHVGGKILYGLVNEKSRHLLERVFAPWPDMEAIMREEQIPLFSLESFRPVLDFEVLGFSLQYELSITNVLNMLDLAGIPLWSRERDEAHPLVIAGGPVVFNPEPFAEFFDIFIIGDGEEVLLEFLDQVEASRGLERLEKLKRLAAIEGVYAPALYSVEYREDGTLKSFAPMFPGVPARVRKRVVKDLDQAYYPDKPLVPY
MEITHDRAVLEVMRGCQRGCRFCHAGIVYRPVRERSLEILENQARRQLQNTGYEEISLASLSSLDYSGIDPLLKALVGNYGEKGIGVSLPSLRVDAFSIDLANEVQKVRKTTLTLAPEAGTQRLRDIINKNVSEEQLLEAVEAAFKSGWLSLKLYFMIGLPGEREEDLEGIIEILQKVKYIGDKYSRRPVEIRASLASFVPKAHTPFQWRPQNTMAELDSKQQFLSRRRRKRIKLSFHDSRTSYLEGAIARGDR
RLAPVIYLAWQKGCKFDAWSEYFNFDCWQDAFRECAVDPEFYTGRQRPYEEDFPWDFIDIGVSKDFLQEEDQKAEEGLTTADCRQEGCSDCGICPAMEVSLNIKGGNVDAIEKQI*
>Syntrophomonas_wolfei_subsp.metylbutyratica_JCM14075T_01955_radical_SAM-linked_protein
MRLRSRYRVGPELKFLGNLDMMHLMERSLRRAVIPYALSEGFNPHIKLSMGTVLPVGLWGENEYFDLELSQRMDTIDFRQKLSAALPSCLQLEECQEIADGAPSLMKIINAASYVFLLEPPFPSLDKWKEELLARPSLTVRSRGKKKGLDKELRTGIYRIEVEQSLNFGRVEIWVAVGEPVNVRYDELGELLLQTGFTQKSIAEVFRSGNYIREGSSYLSPLEVLLDKHTSPCGHSWCSTLRCHY*
>Syntrophomonas_wolfei_subsp.metylbutyratica_JCM14075T_01956_Ribonuclease_G
LIKEIIAEVYPWESRVAIVEDGRLAEVFWADQGENVGKIYKGKIKDILPGLSCVFIDIGLARNAFLYAGDVVRVGKKKGLNVYELLKSGQDIMVQVKKEAFSEKGARVTGDLSIPGRLLVLLPFQNEVSISRKITGDERRKHLRSLLEESKPENGGIIVRTACLEAEDQEIIQELNRLLKTWEEIKQRYQNRKAPSLIYEDMDVLERTMRDYLDGNLSRIVINNQRLKDKILEYISGKKIFNRLTVDYEEGALFE
KNNLEKDIKRSLRRKIWLKNGGYLIFDRTEAMIVIDVNSGKFTGKDNFEETVFKINMEAAIEIPRQLRLRSLGGIILIDFIDMKEKSNQDELVRVLKTELEKDKANTRIIGITGLGFLEMTRKKSRYGVVEFFTDECSNCSGRGHIINLQALASEAKRKMAHMGYLENSQIICKAEPRLLKILKDDEKDIGYIESRSGKSVLLLSDPDLAPGEYKIFAGSE*
>Syntrophomonas_wolfei_subsp.metylbutyratica_JCM14075T_01958_50S_ribosomal_protein_L21
LYAIIESGGKQYKVAEGSVIKVEKLDIAAGEKLTLDQVLFLNDGNGNVKIGNPLVAGVRVIAEVMEQGKHKKITVFKYKRRKNYRKKQGHRQPYTKIKVEKIEA*
>Syntrophomonas_wolfei_subsp.metylbutyratica_JCM14075T_01959_putative_ribosomal_protein
MLALTINYKNNKIISFKIEGHAGFAPEGEDIYCAGVSAVAQTALLGLIKQLTVEPKYKMEKGFLECELPPSLNEEELEKAQIILSTMEAGLLSMQEAYPGRIRVNIRRC*
>Syntrophomonas_wolfei_subsp.metylbutyratica_JCM14075T_01960_50S_ribosomal_protein_L27
MFTFDLQLFAHKKGVGSSKNGRDSQSKRLGVKRYDGQIVTAGNILVRQRGTKIHPGQNVMIGGDDTLFAVIDGRVKFERKGRDKKQVSVYPLETMIM*
>Syntrophomonas_wolfei_subsp.metylbutyratica_JCM14075T_01961_sensory_histidine_kinase_DcuS
LEAKHSMEILRRVRHDFANHLQVVSGYLELGRCQQVKEYIAEIVKDMAEEKKIFEAGLSAKEALYFFEKTLTARELGIILRYKDLEVNSLDLAKLNHKLDEVLNELSDQLKGREEEPVVYLSVHNNEEEITMLFSCTMLAQSLVRISIKE*
>Syntrophomonas_wolfei_subsp.metylbutyratica_JCM14075T_01962_Spo0B-associated_GTP-binding_protein
MFVDQARIFVKGGDGGNGIVAFRREKYVPMGGPSGGDGGRGANVILMADEGLKTLMDFKYRRHFKAERGVHGQGKNMHGAWGHDLRVKVPVGTVIKDDESGEVLADLLLHGQEAVVARGGRGGRGNARFSSAINKAPSFSENGEPGEEKWIRLELKLLADVGLVGFPNAGKSTLISRVSAARPKIADYPFTTLVPNLGVVMTRERDTFVLADIPGLIEGAHQGLGLGHEFLRHIERTRVILFI
LDAAQTEGRDVVEDYRILYRELELHNPDLLKRPQLIVANKMDIPDARGNARRLESELGKTVYCISAVTGQGVEELMEKTYALLQAAPQDIPRGEEPVVRRFEEELPFQIDKVEGVFEVSGPRIEKLVVMTNFNSDEGLQRFQRTVIKMGLEEALKEQGIKEGDSVRIKDFEFEFTE*
>Syntrophomonas_wolfei_subsp.metylbutyratica_JCM14075T_01963_Glutamate_5-kinase
MGWRDKFNKCRRVVIKVGTSTLTYNNGQLNLLRIEHLVREMADLHNRDMEVLLVTSGAIGVGANRMGYKKVPKTMPEKQALAAVGQGALLHLYEKFFGEYGKTVAQVLLTRDDLDDRMRYLNATNALLAILAMDVIPIINENDTVVVDEIKFGDNDTLSALVAGIVNADLLILLSDVDGLYDCDPRTNKEAVLQHQVSEITRDMEEKSASRGSSFSSGGMITKLKAAQVCMAAGVPMVIANS
DEDNVIRRVVGGEELGTLFIPREEKMHAREKWIAFGTVCQGKLLVDAGARQALLNRGKSLLPSGVTGVEGDFDRGTVVAVIEPDGREIARGLVNYAAREIALIAGKRSGEIEAILGEKDYDEVIHRNNLWVRG*
>Syntrophomonas_wolfei_subsp.metylbutyratica_JCM14075T_01964_Gamma-glutamyl_phosphate_reductase
MDKSLEEKLMEQGKKAKAASRFLATASTTLKNEALLKTADALEAQGAGIVAANAIDLELGEKQGLTSALLERLALNESRIKDMAQGLREIAALPDPVGEVLGMWRRPNGLEVGRIRTPIGVIGIIYESRPNVTADAAGLCLKAGNAILLRGGEEALNSNRIIARIIAQSAKDCGIPEGAVQLVDSEDRQAAVYMMKMNEYLDVLIPRGGKGLKQAVLEHASVPVIMTGMGNCHVYVDALADLSKAQK
IVFNAKVQRPSVCNAAETLLVNEKIAGEFLPAMIAELKGAGVEIRGCERTREIVPDILPAREEDWDEEYLDLILAVKVLDSVEEAIEHINCHGTGHSEAIISEEYSSVRRFMSAVDAAAVYANASTRFTDGNVFGFGAEMGISTQKLHARGPMGLQELTTTKFIIYGDGQIR*
>Syntrophomonas_wolfei_subsp.metylbutyratica_JCM14075T_01965_SNARE_associated_Golgi_protein
MLEHLLPVLQAYGVWGLLILAFAESSFFPVFPDFILIPLCLANPEQAAFYALLATVGSATGAYFGYVLGNVVGQPLLRRLASGEKIALIEKTYQKYGAWAVCIAGLAPLPYKLFTISSGIFRLSLPGFLTATLLGRGLRFFAEAILIMSVGEQALVFISQNSGWLSTIVISGIIILLMLFFKRQSVSNFLLGLRDKAGLTRISKGRILGPRLQLLGEWLVALGSGAFLSFFLAGSIKDILNNRLFNTDQVLNQLFAPVI
GPAWKTVDWLFNGWVLFLLILISSYLFLRREKWESVAFLFLSLMGSSTVLWGFYRIIMENYSLNPFSSHLFTHPTRVLINTLWACYIIWLSASRKTLWRKEKLVKFLSGLLVLLLLGVSRVSAGFRPSEVVVTLAIAGLWSVVVWIIFLYRHSLREN*
>Syntrophomonas_wolfei_subsp.metylbutyratica_JCM14075T_01966_Cysteine_synthase
MEWDANNKIFDNVLEVIGRTPMIRLNKIGKGLKPDICAKLEYVNPSGSLKDRVVHKIVEEAEKRGDLKPGMILLEGTTGNTGIAAAMVGAAKGYRVIIVMPEGMSEERKKTIAAYGAELLLTPGAETDVDLVLEEVKRIKQEYGEQIFEVGQFYREDNLLAHIDNTAPEIWEQTAGEMDVFIMCQGTGGTVTGTAKYLKEKNPAIKVYVSEPEDSPILACGRIGQHKIQGIADGLIPEILDLQYIDGIIL
VSSDEALETARQMARKEGIFCGSSSGINVAAAIKAAQKYPEKKCIVTIINDNGLRYLSTELCGTMPERQLLDREYRLSQEDLDKLARYNFEII*
>Syntrophomonas_wolfei_subsp.metylbutyratica_JCM14075T_01967_hypothetical_protein
MLKKLELTFHNQGLRSYIELDLCTECPRQDDKGCCGYYSPVFYPCDFAYLLQYKPEVLDYIFSLDNLTILDASVTVNSRPEGQSYRCSFHSNTGGCLLPQALRESVCRHFVCPGVAWEKEDKLAHWKDFFTALADYEIELNNRIAGILKEKGLSLREEKRREEFLAELLILFEQETAITAPFLAECPPLEKFSLFRELQYGTDWPL*
>Syntrophomonas_wolfei_subsp.metylbutyratica_JCM14075T_01968_Nicotinate-nucleotide_adenylyltransferase
MNWDKIGQVVKVEKIKPWGILGGTFDPIHYGHLIAAEYACHNFKLDKVLLIPAASPPHKELGQVLPAMHRYRMAELAVKSNPHLEVSPVEMEREGLSYTVDTLAYFQAKFPEVEFFFIVGADSLFIMHSWKEPERLAELCRFIVVTRPGYKIERSEAALGRLPDIIWDRMLQMEIPGLDISSSDIRQRAAAGKPIKYLLPPEVEEYIFEQGLYRGDKYAEG*
>Syntrophomonas_wolfei_subsp.metylbutyratica_JCM14075T_01969_putative_nicotinate-nucleotide_adenylyltransferase
LSRDYTGGINMLRDRQQLMDIIKRRLSPNRLQHSLQVAETAVQMAGQYELELEQVYLTALLHDYAKGLSGQELLRLAEENNLIEDEVDREVPDLLHAPVGAFMIKRDLGIEDEEVLTAISRHTLGSTKMSELDKIIYLADMIEPGRDFPGLERLKCLSFRDLDEAMLFGLESTIKYCLDRGRILHPRTIEARNHFLRIRS*
>Syntrophomonas_wolfei_subsp.metylbutyratica_JCM14075T_01970_Ribosomal_silencing_factor_RsfS
LLNEEELAQAVAAACLDEKAVDVVILDVNELTITDYFVIATARSRVQVQNIVEMVQERLKEFNILPSRVEGVEQGNWAVMDYSSTILHVFQPEEREYYDLENLWGDAKEVDVNESYA*
>Syntrophomonas_wolfei_subsp.metylbutyratica_JCM14075T_01971_hypothetical_protein
MSMSAPKPDREYTYGDYLQWPDEERWEIIGGVPYDMSPVPSGKHQEISGELFFQLRSFFQDRSCRVFAAPFDVRLPQGDEKDEDITNVVQPDLSIIRDLDKLDERGCKGTPTVVIEIISPSTAKKELNEKFNLYEKSAIPEYWVVFPGLNVIVVYSLDDEGKYQKTGEYAANQVLTSPLFPGLEVKLEEVFQIK*
>Syntrophomonas_wolfei_subsp.metylbutyratica_JCM14075T_01972_Methylated-DNA--protein-cysteine_methyltransferase,_inducible
MKWLVFDTSWGWAAVLSDNRLIKRTILPFAVGLNEFKTGLESSGIESRLFGENDVTLPLINNIRRYFQGQLIDNWEAQWDINSLPAFTQKVLQHISQIPYGVTKTYGEVARELGKPRAARAVGRALSLNPLPLLIPCHRVLAQNGGLGGFTAPGGVETKKALLALENEVISCHNSDRRVHQ*
>Syntrophomonas_wolfei_subsp.metylbutyratica_JCM14075T_01973_Leucine--tRNA_ligase
MLDRKYDFKSIEAKWQAYWEKNQVFKVEEDPEKPKYYNLEMFPYPSGHLHMGHVRNYAIGDVVARFKSMNGFNVLHPMGWDAFGLPAENAAIKNAIHPATWTYSNIENMRSQLKTMGISYDWDRELATCKPNYYKFTQWMFLKLYENKLAYKKKSAVNWCPSCQTVLANEQVVDGACERCEAVIEKKQLEQWFFKITDYAQRLLDDLVLLPGWPEKVKTMQKNWIGRSEGCEFSLHIEDS
DEVLTVFTTRPDTVFGVTYMVLAPEHPLVEKICRPEQETEVKAFVNKMKYLNEMARTSTEAEKEGVFTGAYAINPMDGSRIPIWLANYVLMDYGTGAIMAVPAHDQRDFEFARKYDIPIKVVIKGEDIPLDGNLLQESYPGDGHMVNSGEFDGLIVEEGQKAVIKFMEEKGIGRGTINYRLRDWLISRQRYWGAPIPIVYCPECGPVAVPEEELPVYLPEDIEFKPYGESPLNFVESFYKATCPSCGKE
ARRETDTMDTFVCSSWYFDRFCSPHESKQPFSREAVDYWMPVDQYIGGVEHAILHLMYARFFTKFLFDINVLSCQEPFTRLLTQGMVLKDNAKMSKSKGNVVSPEEIIDSYGADTARLFILFASPPERDLEWSEQGVEGAFRFLNRVWRLVSELAEGIKDLPAAGHFPELDNDAKNLRFKTHATIKKVTDDIGERFNFNTAISAIMELSNTLGAYKGVKKPDWPVVKEAVDNLLTLLSPFSPHICEEL
WQLTGHAESIYLQKWPKYDPEALLQEEIEIVLQISGKVRDRIMVPVDVGREELEKIALDNPKIRELTRDKEIVKLIVVPGKLVNVVAK*
>Syntrophomonas_wolfei_subsp.metylbutyratica_JCM14075T_01976_hypothetical_protein
LRGSQTIMCDVSSCRFFENNMCSLDAIQVAPCSDISSGSPEDETLCASYEQS*
>Syntrophomonas_wolfei_subsp.metylbutyratica_JCM14075T_01977_Cyclic_AMP_receptor-like_protein
MLDIAALAKLGGVKKYNADEIFFNAGETGHEMFIILKGRVGVYITSIDGFPIMITELVSGDFFGEMSLLEGQPRSATIGAIEDTIVLVINESNFEQVIAQQPSLAYRIMKGMSNRVRQLNEDLGQLKRGKTEERKAHKLAPPTAKKPSTEVLDNSLFPPGHKPYPLEAPLSDDNLLFDREVLCPVCEEKFKTKNVRSSKLRLDKVDNDLRQRFVDFEPLWYLVWTCPNCYYSNFNFEFKQVAEAFKKKLL
EQGKQLKAKVRLQFSEPRKLDEVFVTYYLMLQSLQAAGKPNPTEAAKVWLRLSWLYNDAGDEEMFLYASQQALDMFKDTYYNTRRDTSVEQDQRLSLLLGELSMRIGENREALKFFRGAIVRKGGNNTINRQAEDRIRDLKDMILVQEKKEEKV*
>Syntrophomonas_wolfei_subsp.metylbutyratica_JCM14075T_01978_ComE_operon_protein_1
MDNIDKRYLTAGSLILLVLVFMAGFKYAEFRYRDSAEKLELIEVNPAEKPEHLEDSLIQVYVCGEVKKPGVYQLKENERVYQAVEMAGPEDKADLRLIDMARPLVDGETIVVPGEGELAGEMAGEQGASQLRVSSPTSPVGSAKVNINKAPASELADKLNGIGPALSQRIVDYRESNGAFQKIEDIKNVSGIGEKKFEAIKDHITVR*
>Syntrophomonas_wolfei_subsp.metylbutyratica_JCM14075T_01979_ComEC_family_competence_protein
MPLSWFEVFIVLAASACFSYYAFWPLFILPFLFLLAGGIWGGKYSLRSYSALALFLMAVLFFNWMQMPVPLDLRIQDGVVLTGRVETVPEFDGNRSSFVLKSKEPERAMKKIQVFCNFEAAVQKGEEISLRGDLKLPRPPGNPGEFDYPRYLSYSGIYYLMSVKEKNAIAVISAPGRSECFFAAIRNSGEKIIKDRLPAPEAAILLGMLLGKTDDMAPEQYRDFQKSGIVHIFSVSGLHVGFFLLLMACLTSL
CRMSPGMKLVVTGIFLLLYASVVAWPVSVIRAVIMAIVGLLAYYSGRENALLNGLGLAGTIIIVSDPFALFKISFQLSFMATWGLLYLFPLLKKRLQYTNRLWDLLLIPLSAQLAVLPLTAYHFNLFSPVSLLSNVLTSYLSGACVILGLLALMLAAVWPWLAGIFLYPAGFFIELIMGLNSAVLKLPLAFWWVATPCFILIIVYYLGLLLLALSWSKNYRKRGFGYTGLCLILLFILLISLPASLYQQGVMELVLI
DVGQGDSILLKSPGGKFVLVDGGGSHYSDPGSKKLLPYLHHRGIRKIDVIINTHPDIDHLQGLEKASKGLSVAHIALPYCFKEAEEYEELKGIAARKNIPLIFLKSGQRLSLEKGCELTVLYPEEELYHGNHYNNKSLVLYCQYGAFSFLLTGDVEKEGMQELLQQGNLGTSTVLKVPHHGSKGSLLPEFYRELNPRWAIISAGANNSFGHPHASVLKELQEQGIEVLRTDEDGAIVFQSNGRWVKIKTG
AKGQSI*
>Syntrophomonas_wolfei_subsp.metylbutyratica_JCM14075T_01980_5-methylthioadenosine/S-adenosylhomocysteine_deaminase
VSTIIRNGTLVTMNSRRDILRADILIEGDRIKEIGELKEYEAROFIDAAGMLVIPGLIQSHVHLCQTLFRGLADDMELMDWLKQRIWPLEAAHDEESLYDSALLASAELISGATTSIIDMGTVRHSNSLFEAVKQSGLRYLGGKCMMDCGDEVPEELIDSREKSLQESMDLFERWNGQEEGRIHYAFCPRFAVSCSEELLREVSQLSDKFNIPVHSHASENRGEIRIVKKQQGLRNILYFEQLGLCHEKAIL
AHCIHINEEEMKILAEKQVNVVHCPASNLKLASGLAPVPPLLDMGARVSLGADGAPCNNNLSQFMEMRLAALIHKPFYGPTSMPAEKVFEMATLGGAQAMGQEEEIGSLEPGKKADLAIVNLGHWHNQPATHSSVYSQLVYQALSSDVRATMVDGRFLMQNAALLTIDAEAVKINSEKALNRILQRIGVK*
>Syntrophomonas_wolfei_subsp.metylbutyratica_JCM14075T_01981_potassium/proton_antiporter
MLFFFAVLLLIATFSSKMSARFGVPGLVIFLALGMIFGSDGFKLIQFDDPVLAQQIAIACMIIILFEGGFSTKKELLRLAFRPAFSLATLGIMVTAVTLGLLSHLLIGLPRESAILIGAIVSSTDAAAVFAIFRNKRIEPKTAATIEVESASNDPMAIILTITIISFMQGEITSWQLFLGQLLWQILAGLSIGYLLGKTAPHLINRIKLDSGGFYYVLILSLCFLSYGLADELNTNGFLAVFIAGCYIGNAEFVYKQSITRFIE
GLSTFSQVVLFLMLGLLVFPSNLLQNWQHGIIIAVILTFIARPVAVFLCTIFWKYSLKEKLFICWGGIKGAVPIVLATYPYVEGLEGGSYYFNVVFFVVLLSALIQGSSIDLVAKKLGLLAGSKTRNPFSFELIALQETQSELLEYSVDKGSWLVNHSLQDIPWPQDSLVTAIVRQEEIITPRGDTVIKAQDLLFILLKNDYQEELLDLLDGTDGQNEREGYSCQP*
>Syntrophomonas_wolfei_subsp.metylbutyratica_JCM14075T_01982_Putative_esterase
MSEESKIFDNGFFKNTVMEVLGIEISEFSQERVVAAMPVTPRTHQPFGFLHGGVSLVVAETVASAGSYLFIDVEKQRAVGLEINANHIRSKRDGMIKAVGIPIHVGRSTIVWDVRIYDEAGDMLCISRCTVAIIELRDEKQ*
>Syntrophomonas_wolfei_subsp.metylbutyratica_JCM14075T_01983_DNA_polymerase_III_subunit_delta
MSKNRIYFLWGEEGYLIDRELERIAALMSRESGEEVETAFLDADELNAYELLEALEFSPLFSSQRLVIIKRPWWLGKAKRRGGKTAEIEKIFLDYLQHDMEGQVLVLTANEHSATNPLVKILDREATVIACKSPGPQQLSEWINKEFASYGLKVEKSAANLMAKSGQDMYYLQNLIDKTALLVKGRPVKIADIEGELETKAEIKVFKLSDALLKRNLKASFQAYNQLLVQGEHPILFLYIIVRQFMALARVK
YYSEESYGSREIVAQTGLHEFMVKKMLDNARNFSWDELRQLFQIFLQTDVKFKSSSLDDKMLMEALIVEICSKR*
>Syntrophomonas_wolfei_subsp.metylbutyratica_JCM14075T_01984_30S_ribosomal_protein_S20
VAKSKTPAKRARRAEANRLRNKAYKSKLKTTIKQYENAIIAEDLDTASNKLLQVTSLLDRSITKGILHKNNVARKKSALSKKLNSISQ*
>Syntrophomonas_wolfei_subsp.metylbutyratica_JCM14075T_01985_stage_II_sporulation_protein_P
VHKNYFGTFIKSIFLMQLFLLVLITGVSAEISSPYLEKMPAFNIQNILLPFKGIELNKKQAASLFREVNVVFAGEKTSFPMTRYYFKEKMPEDLMISSIQALAYGRSEEDLRENEGEFEVQLPEVEPEPLEESRDIDYSSFFKGCKVVFYCTHSAESYIPDSGRARCDGQRGLVNEVARSISESLSKRGLPAEFEDRVHDFPDYNKSYTNSRETVQKILQSNNKLLALFDIHRDSIPGLEKAETVEIDGKKSARILIIV
GTDERKSHPEWRKNLEFAQEIYRQGEKKYPGLIKGIRTKAGTYNQEFFPRALLLEFGGDLNSLAEAHYAGMLFSDILIEVLKEDL*
>Syntrophomonas_wolfei_subsp.metylbutyratica_JCM14075T_01986_hypothetical_protein
MLKLKRFFLIFILLVLFISGLNISNQGINHLTLDNRGPVFACNYEQGSLNLQLLSRDYKVSPELLNCEWQLLRQSAKNHGQQAVQYLNKSWLIFKAVFL*
>Syntrophomonas_wolfei_subsp.metylbutyratica_JCM14075T_01987_putative_peptidoglycan_biosynthesis_protein_MurJ
LSERGRNVARAAVLLMITVILSRILGYGREVALYTLFGQDYITDAYRAAFSIPDFIYMLLVGGALSSAFIPVISTFVARDQEEDAWRSASIVLNYVLLLMLFIMALAYLYTPLLMKILVPGLPAQYSDLAVYLTRIMFIQTFFMALNGMAMGILNSFHHFWAPAWGSLLYNLMIIVVGVGLVEQLGIAAFSLGVVLGAVANFVVQIPTLRRLGMKYYFSFDYKDQGFQDIIRLMVPVLAGLGAVQVSLFVTQ
NLASSLAAGTVSALSLAQRIVNLPLGIFAVSLAVAIFPTLTHLAARGEIAQFKRSSSLALRAVFLLTIPASLGLMALGEPIIRLLFEQGRFSATAVAITNEALSFYCFGLFAYSSLQVLNRSFYALHDSFTPVAAAFITIAANILSLNLVVTMGHKGLALAYSLSGLAGFIFLMLALRKKLGQLGGGRIVGSFLISLGASLVMWFLLRLFTGRMLVFWPLMGKLQLLMLVGGGVFFGAIVYGLIIYFFKLEESQLLL
NMLRQRLSKNS*
>Syntrophomonas_wolfei_subsp.metylbutyratica_JCM14075T_01988_Elongation_factor_4
LQKNIRNFSIIAHIDHGKSTLADRLLHLTGALSDREMKEQFLDKMELEREKGITIKLKPVRLNYRARDGQEYILNLIDTPGHVDFSYEVSRSLAACEGALLVVDASQGIEAQTLANVYMAMDLDLEIIGVVNKIDLPGAEPEVVKQEMENVLGLDQDEVLPISAKLGTGVEDVLEAIVERIPPPSGDKEAALKALIFDSYFDPYKGAISYIRVVEGELRQGDMIRMMSSGKEYEVDEIGVLSPYMTEVEVLS
SGEVGYVAAGMKNVNDTRVGDTITGARRAAPEALSGYQEAKPMVYCGLYPLENSEFERLRDALDRLKLNDASLFYEPDNSDALGFGFRCGFLGLLHLEIVKERLEREYDLSLLATAPSVIYRVLLTDGSEVMVQNPTHWPPPQKIDRVMEPYVKASIMVPNDYVGTIMELCQEKRGSFITMEYLTSSRVVINYRLPLAEILYDFFDALKSRTRGYASLDYEFHGFDTSDLIKLDIMLNGEVVDALSFIV
HEDKAYYRARAIVNKLRKIIPRQMYEVAIQAAIGNRIIARENIKAMRKDVLAKCYGGDITRKKKLLEKQKEGKKRMKQIGRVEVPKDAFMTVLDIENE*
>Syntrophomonas_wolfei_subsp.metylbutyratica_JCM14075T_01989_Oxygen-independent_coproporphyrinogen-III_oxidase_1
MKKKPAGLYIHIPFCLKKCGYCDFYSLPLQSPAQLQRYTAAIRLEIEKRAILFPDSIILSIYLGGGTPSLLGLEQLQSIVEAVVHNFNVSSNAEISLEMNPATRSEGFLRGILAAGVNRISLGVQSFSPEELRALGRVHGYCEIMQSIEALHKEGCQNFNLDLIYGIPGQSLESWQRNLELAVSCGAQHISAYLLQLDESTAMARAIESGEMELPGEESENAMYHLALDYLPEKGYKHYELSNFCQAGFECRHN
LNYWCGGDYIGIGAGAVSCIGRQRYRNRPFLEKYLISMEAGRVAPVEILEQMSVEEKMREEIILALRLCDGIDLAQFAARYQLDLRRRFAVEIEDAEQKGLLKLGNNRLCLTRRGYFLSNQVFCRFI*
>Syntrophomonas_wolfei_subsp.metylbutyratica_JCM14075T_01990_Heat-inducible_transcription_repressor_HrcA
MTLDERKKRILESIIRDYVETAEPVGSRAMVKKHDLKISAATVRNEMADLEDMGYLEQPHTSAGRIPSESGFRYYVDCMMEKEPPGEREYALLQQLFEQNIEDWEQVILGIAHFLSQMTRYASFVIVPAIKFTEFRSLQLFPLDKGKALLLLVCDMGVIMHRIISIPELISSQDLQAITGMFNQAFRGKRLEEVKRSDLQLIRESLSQRRKIIDNALEAIDNLLQNSRDEKVMVSGALNILNEPEFKDMEKL
KRILNVLEEDDSLRQIIPGELGEAVDIKIGRENPLEEIKEMSLVVAGYKSFGELGKIGVIGPVRMEYWKAVGTVETVRDIIEDYLKGLG*
>Syntrophomonas_wolfei_subsp.metylbutyratica_JCM14075T_01991_Stress_protein_H5
MSENKLARQEKSDDRFQALLSNANASRAISQAVEGTIGPKGLDIMMVDRFGDVVISNDGVTILKLIEVNHPAARMIINTARAQQSEVGDGTTTATIIAAALVSEGMEQVLKGVPVTRVIEGIEQGIAALNLVKKQSRAVESLDDKVLFNIAEVAGRGKRDLARLVIKGAQLVGKKKLLDRDYKFADAVVARELAPSQVFRGVLLNKEPVNKDMPAVLENPLILVIDDALGPEEIEREAMKSESGFQYY
LQARANYEENLRKIPELGVKMIVADRGIDDMAEEFLTDAGIMAVQRVSSREIEKLCQHTAARKIKRNGLNRDLDSLKGYLGKARMVKVDEKLQHIYVYDGGGESWTTVLIGAATEEVVEERERMAKDAAAAVQAAVQGGVVPGGGATEVWLAAHMEEVSRELEGMASFGVNCVKEALLKPFSCLISNAGFNPLEKLGDVVVAQRKNGSDSIGIDCEKGKLMDMMRQGIVDPTPVKIHAIKAAG
EVAVAILRINTIIKMKNDPEQPESTENWD*
>Syntrophomonas_wolfei_subsp.metylbutyratica_JCM14075T_01992_HSP-70_cofactor
MTKEELELEKEQEQEIIESVEASTIVEGGEIQALKEELARLQEEKQENYDLYLRALAEQENIKKRAGREREEYIKFAALPLIKRLLPVIDDLDRALDVSHANQDLEALNKGVEMIARKLHELIKNEGVEAIEAVGKPFDPMYHQPLMVEGSSEEQENMVIEEFQKGYIMHGRVIRPSLVKVSNS*
>Syntrophomonas_wolfei_subsp.metylbutyratica_JCM14075T_01993_Heat_shock_protein_70
MGKVVGIDLGTTNSVIAVMEGNEAKVISNAEGERITPSVVGFSKSNERLVGRIAKRQAITNPDRTVLSIKRKMGTDYKVEIDNKKYTPQEISAMVLQKLKTDAEAYLGEKITQAVITVPAYFTDSQRQATKDAGKIAGLEVLRIINEPTAAALAYGLDKDENQTILVFDLGGGTFDVSILEIGDGVFEVKATSGNNRLGGDDFDERIINWLVEEFKKEQGVDLRNDKMAMHRLKEAAEKAKHELSGV
MSTDINIPYITATQEGPLHLEISLTRARFEEMTADLVEKTMGPARQAMKDAGLKTNELDKVILVGGSTRIPAVQEAIKNITGREPFKGINPDEVVAVGAAIQAGVLTGEVTDVVLLDVTPLSLGIETLGGVFTRIIDRNTTIPTAKSQIFTTAADNQTSVDIHVLQGERKMAGNNVTLGRFQLSGIPSAPRGIPQIEVKFDIDVNGIVNVTAKDTATGKEQRITITSSSGLNDEEIDRMVKDAEKFAEEDE
KRLEEIEARNAADSMIYQAEKTVKEYSEKIDEESKKEIEEARDELKTALEGGDIEDIKKKTESLTEVIFKFTTKMYEEAAKNAEAGAGSSDAGDGEVFDADYNVADDEKKED*
>Syntrophomonas_wolfei_subsp.metylbutyratica_JCM14075T_01994_Chaperone_protein_DnaJ
MSAKRDYYEVLDISQNASAEEIKKAYRQKARKLHPDVNRDDPQAEEKFKEVSDAYEVLSDPQKRELYDRFGHDAFDPSRNGAGGFGGFGGFGGFDDMGGGFGDIFDIFFGGAGTRRRTGPQRGADRELRLEINFEDAVFGIEKEIEISRMERCEKCKGSGAEPGSEVKTCPTCRGTGQQRTVQNTPFGRFETSRPCSQCRGEGKIIEKACSACKGNGEQRKNRRIDIRIPAGIDSGSRLRVQGEGEQGIR
GGPSGDLYITILVRPHASFKREGYNLLCKLPIDFVQAALGTEVEVPLLGGAVHLLHIPEGTQPGDIITVKGKGIPHLHSHRNGDLKVQIEVKIPRKLSRRQREILLQFAEESDEKGGKKGLFEKFKDAMG*
>Syntrophomonas_wolfei_subsp.metylbutyratica_JCM14075T_01995_Ribosomal_protein_L11_methyltransferase
MKKEERKACSKNSKMLWDRGCSGMKWKEIAVLTEGICIEAIAGIFHRLGSGGVVIEDPQAARKYENQEEWNPNLVSPDFLDHEFVLIKAYFMEEREVMEELQSCLEAVKENFNVECKVFIDEVRDEDWESSWKKYYHRFKIGERLVIKPSWEEYQPQSGEVVIDIDPGMAFGTGIHASTRFCMKFIDHYVKGGEKLIDAGCGSGILSIAAAKLGAARVLAMDVEELSVKIARENVELNGLSDIITVKLG
NIVEEIQTFEADMVAANITAEVVTCLIPEAAKVLKSGGYFFGSGIVDSRWPGVEKQLKTHGFVIEQVLQDVDWIGVAARKE*
>Syntrophomonas_wolfei_subsp.metylbutyratica_JCM14075T_01996_Ribosomal_RNA_small_subunit_methyltransferase_E
LPRFFVSADNIKGNSVLIDEEQTRHIEKVLRLKSGDIVKVFDGNGNEYQVRLQEKKQKILQAEILAQETTAEDLAINLTLVQGIARSEKMDFIIQKAVELGVRSIIPLQSEHSVARFDKEKEGKKVKRWQKIAQEACKQCGRNHIPEVKPILDFPALMAQIGDKPALLCYEQEREAYFRTLLREQRENFCTRGLFLIIGPEGGFSPAEVETARRQGCFIAGLGPRTLRTETASLLACGIIMYEYGELG*
>Syntrophomonas_wolfei_subsp.metylbutyratica_JCM14075T_01997_Threonylcarbamoyladenosine_tRNA_methylthiotransferase_MtaB
MKKVAFHTLGCKVNQVETEQLKEKFIQRGYQLVDFNESADLYIVNTCTVTHSSDRKSRAMLRRAARRNPGALVVATGCLAQVDAAQLAAIPGLNLIIAGSQQKEDILDLIEGQVISRSESEPLVVCPPLVAEKKLPPVIYSKRHERSRAFVKIQDGCQSYCSYCIVPFARGPSRSKLPEDVAAELQQLVDLGYHEIVLTGIHTGLYGNDLEDWNLSRLLRLLFARVKGDYRIRLSSLEPVEIDEELLEMAAG
GERLCRHFHIPLQSGSSRILRAMNRRYDREFYYKLVQDIVQRVNGVAITADVMVGFPGEEKKDYQDTLALLQDLPLLDLHVFKYSKRPGTAAAKMEPQVSDREKEMRSTELIQLAEAKKRSFINRQLGQELEVLVEQRVGEDSFRGLSDNYLELEFSSPRELTGELTGELVMVELEAMENSIARGRLLYGSFLIEPYCHDTHQEG*
>Syntrophomonas_wolfei_subsp.metylbutyratica_JCM14075T_01998_30S_ribosomal_protein_S21
MSEVKVGKNESLDSALKRFKRSCQKAGVLQDIRKHEHYEKPSIKKKKKSEAARKKKRF*
>Syntrophomonas_wolfei_subsp.metylbutyratica_JCM14075T_01999_HIT-like_protein
LNKSDCLFCKIVNREIPAELVYEDERIVAIKDINPAAPVHILLIPREHIVSLDQASNENVELLGCIQTTAAKLARELGIADKGYRLVNNCGEWGGQSVLHLHYHLLGGRQLAWPPG*

>Syntrophomonas_wolfei_subsp_methylbutyratica_JCM14075T_02000_hypothetical_protein
MFCDMMFLQNDYNKIRYGYNEYSYTGELSSGRYFFIIMKHSPWQQEERELLKWETIMSLLVGRGVIL*
>Syntrophomonas_wolfei_subsp_methylbutyratica_JCM14075T_02001_hypothetical_protein
MGNHNVFIGWKGCYSIMTALNDTFEIIVNGQVISFPIDKPLIANKIRDEVYIYVPLDFCSETLGARVEWEEIGKTLAIHRDGQSVKMRFGDNLAEINDEKIWIGDNLAEIEGVAFLNYPLPWLRIEEGNIYVLLQMFSRFQGMTIKWEPGGKLLISDATVLALESRPASIINGSIKERAGQEKDILYEEVSRLIDNSGLDRVKEDIKGYLMESVRIRPKRCEEGNRIGESKIAGIPDLPFTMEWPSVKGRPLSFI
AQLNMEELADILPQNELLPAKGWLYFFYDNEEQPWGYQPEHREQWEVLSFDGDPALLERRAFPAMLPDYCRFMPYRLQLAREICIPSIWSLYYQTLKLGWQEQELYRKLEQEIVRLYGDRDEFAPVYRVMGHPGIIQHDMQLECQQVCNGFEPYNFNDPKVAKEYGKAWDWILLMQVDSDESNNGMMWGDCGRLYFWIPRQALREKDFQQVWTILQCY*
>Syntrophomonas_wolfei_subsp_methylbutyratica_JCM14075T_02002_hypothetical_protein
MSIVIPCNTTIFIGGCGPRPWGVSKKIYFLMLKDEVVNNPVPMTIVYIHGVQCLKLHFINIIFAKGKKGAG*
>Syntrophomonas_wolfei_subsp_methylbutyratica_JCM14075T_02003_sporulation_protein_YqfC
MERRREILLNKAMAEFLELPGDLVLDLPKITVVGRNELYLENHRGIIEYALNRLRINLSRGFLEIEGEELQIKTLMPEEMGISGEIHSIRFLD*
>Syntrophomonas_wolfei_subsp_methylbutyratica_JCM14075T_02004_sporulation_protein_YqfD
MSNRFLNQLAGIKVKLRGERPEKINLALSRGIHMHKIKRTDDGMHMVKISIGLPEDLKVVSEEVGKPFLKGLFVRRLGFWAGAVIFIAALYMASFIWFIDVSGNQKVDKSRIIMTAARYGVYPGAPRWTFSRSEVEGAMLRDLNELAYVKIDIKGVKATIEVVEKIPREEISGPCHIVAAKEGVVEEVLVLAGEAQVEKGKAVSKGDILISGVYPQKNPFMEGVEEELEEKPSLVRARGQ
VKARVWYQGYGECRLRTEKILISGKKSSEIKLEIGGKTIPLKKAKIKNYDSISEMKQKEIKTPLGSVRVYKTCWREQLKKITVNEEEALSIAQEKAMKSLLKKMGKDKKIVNQKKILSSPSDSIIRVQLSVETIEDIAREPINVSPNGN*
>Syntrophomonas_wolfei_subsp_methylbutyratica_JCM14075T_02005_PhoH-like_protein
LLRVQILLDGYFDRGTECILLEKESRIALSDNQQAAIFGPGDENFRHLRSISPAEIYARGAELFIKGQEDDVKQSFDLIQNLLEVVDREQQLNINDIDYRYTLIKKGEKSRHQDIVSSPILNTARGKAIKATLGQKKYIKSILKDDVVFAIGPAGTGKTYLAVVMAVRALKHKEVERIILVRPAVEAGEKLGFLPGDFIEKVNPYLRPLYDGLFDILGVEITQRYLEKNIIEVAPLAYMRGRTLNDAFIILDEAQ
NTTPQQMKMFLTRLGFGSKAVITGDITQVDLPKEYSGLKEIQKILGHVSGVSFEYLTKEDVIRHPLVQKIIDAYETYEAQLF*
>Syntrophomonas_wolfei_subsp_methylbutyratica_JCM14075T_02006_putative_domain_HDIG
MKLSTILNFVWKRIYGFLSQSTTRKVLGVLLFFSVTIFIFSKFTPTQVRLKPDEVAQRNIQSNITAVIDEKQSDELRKQAASKVQKVYQGDQYALSNTETEINNFFSTLMEIMNSDKQEKLLDLLDSSSKRKDYLNLKYNRQQLASYLLDTPISDIETMRQTSIMAVRGIMSKPLTDEAIKGASEQVDKQVDALAFAPQAQEIMKLVISNSLRPNLIFNEEATNKAIEEAMASVQPVQKTIKAGEIIVRE
GDRVTEEQISTLEQLIQRSKSYPLTILGAGLFVFLSVLIIFLRRYIYRDIFENDRLMLLGLVFILLILLTRFLTIIKISNQPDINNLMAYLAPVAAGSMISAILLDNRLAYFLTMIMALYYVLLAEGNQLFQFYAITAFVGGSVGVFQVYKLNQTSDLARSGLYIAILANMVSIITLLFGNNWSLNILAITGVLIGAINGIFSAVLMIGALPLYLESAFSITSMNKLLEFSNPNHYLLRRLLLEAPGTYHHSLMVGNLAEA
SAESIGANPLMVRVGAYYHDIGKIKRPNYFVENQQGFDNPHEKIAPALSALIITSHVKDGVEMAREARLPQTIIDFIEQHHGTSLTKYFYNRAMEEDRDGKILGEESFRYEGPKPKRKEVALVMLADSVEAAVRSLSDPRPEKIRNMVRLLIKDKLNDGQLEQLCDLTFKDLDTIGNSFCKVLEGMYHKRIEYPETIVREFEKRRESHGNHDNKPAEQD*
>Syntrophomonas_wolfei_subsp_methylbutyratica_JCM14075T_02007_Endoribonuclease_YbeY
METMIINQQNKINYTKEMQQVILNVANAVAKMVKLSPNTELSVMITDNSYIKELNLIYRGENNPTDVLSFAMNELSEEMDLDLPGEVNVLGDIVVSLEKAVSQSEEYGHSVEREILYLAIHGMLHLLGYDHENEEERNLMRNLEERIMHKVKLER*
>Syntrophomonas_wolfei_subsp_methylbutyratica_JCM14075T_02008_Undecaprenol_kinase
MKGTGLKRSFACALAGIAYVFQQERNMKIHGVAAIMVMGLGLFLRVTRWEWGLLLITIFMVLVAETINSAIEKAVDLTSEYHQLAKAKDLAAGAVLLTAIMAVIMAFIFGPYLLSLAGGG*
>Syntrophomonas_wolfei_subsp_methylbutyratica_JCM14075T_02009_Cytidine_deaminase
VSKREELLKWAREAQKNAYAPYSNFQVGAALLSAGGKVYTGSNVENSSYGLSICAERAAVFKAVNAGERDFTLLAVVSGQGYVFPCGACLQVLAEFSPEMKLIITDEKDHYKEYALQYMLPQIFALGKQEE*
>Syntrophomonas_wolfei_subsp_methylbutyratica_JCM14075T_02010_Bex_protein
MKSGFVSIVGRPNVGKSTLLNALIGEKIAIVSEKPQTTRTRIQGIYTCERGQVIYVDTPGIHKPKHLLGEYMVKVSARSLEEVDIIYMTDVTRPFGGGESFILDQLKDARVPVFLLVNKIDLVSEQEVKDYIQIFNQQRSFSEVVSISAACGTNLQLLLDKTLDALPEGPLYYPEDDLTDQPIYLIAELIREKALMLTHDEVPHSLAVEVEEFKKQSNGKVYLRAAIYMERDSQKGIVIGKKGQMLKTIGEQ
ARQDIESLLDTAVYLDLWVKVKKNWRDNENTLNQLGYRM*
>Syntrophomonas_wolfei_subsp_methylbutyratica_JCM14075T_02011_Deoxyribose-phosphate_aldolase
MLAKYLDSTNLQAEARASDIESLCQEARQFQVAAVCVNPFRLELACHLLAGTPVKACTVIAFPLGADHWKNKVDSARRALQMGAAELDMVMNIGAFKDRDYRLVEREIKELLRLREEYAFLLKVIVETALLSKGELQDISALLSNSEADFIKTSTGFSSRGVTLEDIEIIRESRQRKDLKIKASGGIKSLDFALELLKAGVDRIGSSSALALLEEYRQRGGQ*
>Syntrophomonas_wolfei_subsp_methylbutyratica_JCM14075T_02012_hypothetical_protein
MRIKRISLFIILLAFYTSIMINYPSECLKNLGYNRVLDFYGRWVSSSCNLDFLYNPGLRQTAIPLHTSRVAAVIPGGSNQGIKRQMEKLLAEKYQVIECSAIDTWHSSKDGQEYLSRIAAQAYRVVVFDGGHHLPTLGMAPDIILVPELAGFAVHTYMLDGMRVETIRDLAEEAGCPAVIVRIPRALVKNQRSLSIITRRIMAASHYSDRESSTGKIMLQSRMSKFNGIIFAYVNYEYAKKPELFCQCLNALG
VGDARKLYLAFDYGCEPAEEGMKKVSKSSGLPAQIVNEAVKVSSVFWGGK*
>Syntrophomonas_wolfei_subsp_methylbutyratica_JCM14075T_02013_Recombination_protein_O
MYLRSRAIVIKNMDYRESDKLVTIFCEHEGKMKAVARGIKKPRSSLRACVQPFCHSSLFFSRGKGMNLITQGKLLEFYGNTREDREFSLYALYLMELLDKSLMEGIAIPRLYTSTLEVLSHLNHSGYNPMLIRYFEMNLLVNLGYSPLLHHCVLCGKKDDLKVFSLPDGGMLCRDCSYQASGAVSLSKETLALIGLLGSSRLVTVERVRVSNKAQKELEYFLEKYLEYLLERRFNLKKAMSILKRSMPKDT
HLI*
>Syntrophomonas_wolfei_subsp_methylbutyratica_JCM14075T_02014_hypothetical_protein
MNGFTNKILSAFSTLVDKQGIKFVYYNEPINKQAACSNPRFVAAVWLKLLHSFFRCLKIKIQIPSKSNYNA*
>Syntrophomonas_wolfei_subsp_methylbutyratica_JCM14075T_02015_Magnesium_transport_protein_CorA
VIKTYFYNEQEDRMFHDVDLANAPDLLVSPKNLLWIDIYDCSERELQYIGDIFNFHPLTLEDCLQEENPRAKIDRYDDYYFFVFHALNYFEEAEEIDEISIELDVFLGPNYIVTIHPIALSAVGKIARICLKDSRFMTRGPDYLLHAIVDNIVDDSFPILQRLGERIDKLEDNIFLQKGQVITEEILTLKRTLILLRKVLIPQRHIFANISGRYSFFARNENIPYYLDLADNLNSILDTVNTFRDLVNSSTEIYYSVL
SGRTSEITILTVTIVIMMPLTVITGFYGMNVKLPEANPYSVWYISLAMFSLALGMLGFFRFRKWL*
>Syntrophomonas_wolfei_subsp_methylbutyratica_JCM14075T_02016_putative_molybdopterin_biosynthesis_protein_MoeA/LysR_substrate_binding-domain-containing_protein
MKEDRNLTPVQAVLDLIKTKVTPLKSEEVMVKEGLPAYRKQKIEIFLGRDVLDAYIKQGKKKYKYKEIDTLEASSKPQAARQDYLSNAPGVLIQEQGDLLDLVSREILESHSNQRSVIAHAYHNGGSIEVQELAVQTGNKGQTLPHEKISDAVTDETGLQPMIPVRIPVTGSSLFKILKVVAKLMLQVFNDENHHLGLRLEIRLQYQSSEDMAGIHLWDSDSDSYNIPYVRRMVPGIPTLIIHLAMRQQGFYVARGNPLEIKGWEDLTLPDLRFINREPGCGTRVLLDEHLYQMLDRRKINGYKREETSHLAVASAVARGTADLALG
NEKAAMQVRGIEFIPLQKERYELVIRKEDHDIQAIRAIDIEESIISSAKFSELEGLGDYCLEETGRIVAEF*
>Syntrophomonas_wolfei_subsp_methylbutyratica_JCM14075T_02017_Molybdate-binding_periplasmic_protein_precursor
MRHNKKAKARISLVALIFVLCMAGSAFAANPAATGNLVWKAGDQCSVNASALNFRTGPGMNYKVIKSYTRGSLMTIAEKSGDWYKVTAADGKAGYVFGRYLLTATEAPTPVKAAPSVTTSKAEVTAEPQAKAVSGELIISAAASLTDAMNEMKGLYEKQNKNVKLTFNFGSSGALQQQIEQGAPADVFISAATKQMKALQDKKLIVDESCKNLLKNRLVLVGPKDSAKVKGFSTLKEASLIAIGE
PDSVPAGKYAKETLTKMGLWDAVKDKVVYAKDVRQVLAYVESGNVEVGMVYLTDAKISDKVQISIAEIENTHTPIVYPAAVVKASKNPEAAQSFVDFLSSDAAKDVFAKYGFSLVK*
>Syntrophomonas_wolfei_subsp_methylbutyratica_JCM14075T_02018_Molybdenum_transport_system_permease_protein_ModB
MTEAELIPIWISLKTAGTATLITFILGIAAARWMAYGDFKGKNLLDGLFILPLVLPPTVLGFGLLLIFGKNGPLGQLLLYFDKTVIFSWSATVIASTVVAFPLMYQATRAAFEQIQPNLEDAARTLGAKEWTVFWKISMPLAWPGIAAGTILAFARSLGEFGATLMLAGNIPGRTQTAPLAIYFAVEAGRSDQALKWLAILLISFSIIALNYWKSRQYSSWRNGQPVVKEVIDAGYRN*
>Syntrophomonas_wolfei_subsp_methylbutyratica_JCM14075T_02019_Sulfate/thiosulfate_import_ATP-binding_protein_CysA
MLDIEIKKSLPGFKLEVSFAVEREILALIGPSGCGKTMTLKCIAGLQADEGRIAIDGRILFDSGRGVNIKSRERNMGFVFQNYALFPHMDVFNNIAFGIRQLPVEDIKEKVRLWQKMRLSGLEKRFPGQLSAGQQQRVALARALVSEPEVLLLDEPFSALDSIVKERLEEELLEIHEFFPGHILFVTHNLAEAYKLSSKIAVYEAGRLLQFGEKEKVVNRPSNRVAARLTGVKNFLPAVVQEVESSSLNLEL
SGLGTRLKAGKVPGEKWSGQVVNLGIRPEYIRIINDGENLIAVEDIVDKVEIEISSYIIRCSLAGSGGEKIELEARIPKTEAVNLSQGDSCQFCLPADKIFMLE*
>Syntrophomonas_wolfei_subsp_methylbutyratica_JCM14075T_02020_CobQ/CobB/MinD/ParA_nucleotide_binding_domain_protein
LIKVIMIISNRPACGQTTVAVNLASGLSRKGYRVLIGDTGNNKELNAYLPLIVGNNLDRQETGTNAAKPLEDKAQAKIFSSRLGIDLLQLLLKPEDSATADYSPHLEKLTYDYLLLTPASSEDCQWLSAMADAIVCSDLTAANELEELKNLEEWLRASRINAGGIDLIVPNKINTKEWEHNIEQLSILADYFGYEHIADPIPT*
>Syntrophomonas_wolfei_subsp_methylbutyratica_JCM14075T_02021_putative_addiction_module_component
MGTDEILKQALKLIEERFIILVESLIKSIDEPDRKLDELWAEEAEKRLAAYREGRLEGIPMEEVFKDER*
>Syntrophomonas_wolfei_subsp_methylbutyratica_JCM14075T_02022_Plasmid_stabilisation_system_protein
MRVVFSNYAKLELEDAARFYNLQHAGLGNRFKVEVKNAALRIAKYPTAWSNESGEVRKYLLHKFPYKLLYSIESDHIFIIAVAHQHRKPNYWVERDEL*
>Syntrophomonas_wolfei_subsp_methylbutyratica_JCM14075T_02023_hypothetical_protein
VLCKNYLGYDSRCFPIKQILDNRRRGNVGDYLVDNIDKKAKLSIVSAYFTIYAYNELMTQLNKIEQLRFLFGEPTFVRDENLKENPPLRL*
>Syntrophomonas_wolfei_subsp_methylbutyratica_JCM14075T_02024_putative_methyltransferase
MNKFDGKTMDLVQQNIDELKKLFPEALTEGKIDFQALQDLLGDEVDTGKEKYEFTWPGKSDSKWDGTTEQDMMAKPANSGRYHSDWLNMMHPRLNLAKNLLRDEGVIFISIDDNEVDNLKKICNEIFYEYNFVEQIIWKRRSTPPNDRVIGDEHNYILVYAKDIEKME
LYLQPRSEGINSRYSNPDTDPRGDWVASDLSANGKGGRLTSECIYPIKNPITREEYYPPQNKCWLYNEEKMTGLINEGRIGFRANSGAPFLKRYLSEVRQGATLPTIIDNGGFSSDSAKEIKDIFSKDVFEFPKTTKLLKILVQTGSVNNDIILDFFSGSATTAHSVMQLNAEDNGNRKFIMVQLPEPCSEDSEAYKAGFKNISDISKERIRRAGEKIKEENKDKEGIENLDIGFKVFKLDTSNLKAWDPDVE
ELEETLKGMVDPIKEGRTQEDMLYEIMLKYGIDPTMPLKEIQIKGKTAYSVGLYGMIVCLEDGLTLDVIEAIGDMKQSGEIARVVFRDSGFADDNVKTNAVQLLKKFGVEDFRTI*
>Syntrophomonas_wolfei_subsp_methylbutyratica_JCM14075T_02025_type_III_restriction-modification_system_StyLTI_enzyme_res
MKLKPVSDQQRQLDVASITDIQQAAVKQTNPSVASTMGSGAQRLGYQELGYKELGYANKLDLLDELLENINRQLRNGLPKSTDIQGRNFTIEMETGTGKTYVYIRTIYELYKYGFTKFIIVVPSVAIREGVNKSLEIMEEHFKSLYEGVNCDYHYDSSKLGQLRTFATSSNIQIMVINIDAFRKSFVDPNKPDKANIIHREHDRMNGRRPIEFIQQTNPIVIIDEPQSVDRTDKAKEAIASLNPLCTLRYS
ATHVETYNLMYRLGPVDAYQKKLVKEIVVDSIEKAGNFNRPYIRLVSVKNSNGFQAVLELDYEIKGELIRKKGFVVRQKQKTVRVGQDLFDISGEREIYSGYIINEICYPGNEMVEFTNGSHLHLGMEMGNISEDDIKRGQIRSEIRQTIKKHLQKELRLRPQGIKVLSLFFIDRVDNYRIYDSSGNPNHKGKYAMFEEEYSKLIAKPKYRTLFEDHSYMLNDDTSMVHDGYFSIDKKGRKIDTRGNTLADHDTYNLI
MKEKEKLLSFETPLRFIFSHSALKEGWDNPNVFQICTLIEARETLTRRQKIGRGLRLCVNQEGERIFDPQINTLSVMANESFAEFAENLQSEMEEAETGVKFGFHHAFAGISYTDDSGKIENLGYEASENLIEAVQKNNKLIAKNGKLTDKLRPLTKEDVPPEFEFHVEHGIKQVIRQSLNKLPIRNHNNEVTAQLNKRVLGPEFESLNWRIKYRTTYSVAMDIEKLARECVDAINKMPAIPKIRLIQES
ARIDQQSGVSGELTSVKKLVDQIDIKHSLPDILRYLQDETRLTRRTIVNILIEARRLEDFKNNPQEFMEAASDIIKQKMKLMAIDGIKYERVGDLEFYRQELFEMQELKGYLSKNAVEVKKSVYDLIDSNTEKEFAQMLEQDDEVKVYTKLPGFFKIIETPIGNYNPDWAIVWDKDGTEKLYFVVETKGSTDSQQLRDIEYNKIECGRKHFEAALETGVEYEVAEDYVNKLGI*
>Syntrophomonas_wolfei_subsp_methylbutyratica_JCM14075T_02026_hypothetical_protein
MSFPTIPSITPSISNRTQVINLLLASIALEELGLAHIINAEGEKLQAVLGALPGLSVKATSIKSLLSVNREVRRTLTTIIKNQMLLQFKLEDIMDIFSTPTRPPSPPIFVDAGSAWGVGIEYGTGNAQYFTSSDTEKTVNLVGLGATNIPVGTVHILLPGTVNLLVTFTTDFPYVMNQVHLYVGATAPVNSNPGSFPYKYPPAPDGYFTTYTFTVDVSGIPGTIYVAAHAHILQQV*
>Syntrophomonas_wolfei_subsp_methylbutyratica_JCM14075T_02027_Glycosyl_transferase_family_2
MPEGKPRVLLGSPIRQKTEILSNFLSSKLSMSTVNLDFYFIDDNSEEESQLLAAFAARNPNTSLTKVQDERPYICNESTHIWEEELIWKVAELKNSINKAREDDYDYLFLVDSDLVLHPQTLEHLVSLKKDIVSEIFWTRWQPDFPELPQVWVSDQYNLFKCFRGENLSESQVLQRVLQFLNQVKVPGVYKVGGLGACTLISKRAIQAGVNFKEIYNISFVGEDRHFCIRAVALGFELFVDTHYPAFHI
YRLSELGGLAEFKKMRINQTT*
>Syntrophomonas_wolfei_subsp_methylbutyratica_JCM14075T_02028_Spore_coat_protein_SA
LTRALTTDSKPRDKKFSAEMLEIVRVLNSQKHKAAILYLIPENENPRKEMLQLLREMSYKGYLCFLCKPSDSGGIETLEEKLILVNQGFSILPVIKISLSAIVLSTQNIHPDWAEVLHHKLLWFHIDMDTPNDISEDISKQANLISYFPSTSIMEKFSSNSKGLCLKEGQENQANVALIEARIKSLPLGWLPYANLNLLGKVAVMTATFLDFCGEYFYNGGAERYLLDLAEICTELNNQLIVFQYGDYSWMRRF
KNIDIVSLSRRGIAEGWILKCARDFNQVFYEQVQGRTALNIYSAFFEAWPYAIAPAANIGISHGVSWDNPYNDYENAVEYWLMNQRYIDGAKACEDLVSVDTNTANWLQTVDFDLGQKTKLIPNYVNLSVFKPAEDYLEKGDKTVILYPRQLSSARGLYMVLEILDDIIEQQPEVEFHFVGRGGEQDINQVIEKQKKWSERVRHYPMSMEEMPRAYQQADISIPSVHSEGTSLSCLEAMASGNAVIAT
RIGGLSDLIINNYNGFLIDPRPEELKQAIMNLLDNPEQLNLFKRRNLEVALAFSQDLWRKRWLLLLKNINEIPANKITNSRTVEIKLLSNPVDYGKLGELITTLANGDLVYVRSSEPVEPELSFARLQWLAPETPLFAPCDLTIEW*
>Syntrophomonas_wolfei_subsp_methylbutyratica_JCM14075T_02029_bifunctional_3-demethylubiquinone-9_3-methyltransferase/_2-octaprenyl-6-hydroxy_phenol_methylase
VAGCSDLSFYDQDYFDRGCQSGKSNYVDYSWERIGGEVEKTAAHIIKHYAPEIKTLDVGCAKGFMVKALYERGVDAYGIDASDYAVSKVPAEVNDRVKLGLASDEL*STLNRSEFDLVTIFDVLEHIPEEELYYTLKCELLRRVSSKWVLVYVVTKYEPDDIDPTHINIKPREWWEAKFVELGGKICSTNVIYNPDIWWFNLAERLILVKK*
>Syntrophomonas_wolfei_subsp_methylbutyratica_JCM14075T_02030_SPBc2_prophage-derived_glycosyltransferase_SunS
VGQNKKSLSLAMIVKDEENCIKRCLDSVINLVDEIIIDTGSTDKTIEICRSYNAQVFPYWNNNFAAERNFGLDKVTQDWVLWLDADEEVAPENRNQLNNQTLFEDYDALSVPLINFYGAEEVDHDQVVIAQPRFFRNHMGFRENKIHEWLNLSSAYEQDRVGFLDLKIYHYGYMNSQVENKQKFKRNVSLLLRELAEEKNHSWTHYYLAAEYYSNEFNKAFEHVNQSIELFINEGIIPPPSMLY
SLYTLNLSIGTMEGSWEGAWPGIKSAVKMYPDYVDLKFYMGVILYYKKMYQEALDCFKELCLGESNLNYLTIGLGSFSAWFYAGLIKEKFNEEEAFLAYLKAALISNNYIPAREAITKLINMHPSLADRIKYLKPEFMRLLESTK*
>Syntrophomonas_wolfei_subsp_methylbutyratica_JCM14075T_02031_hypothetical_protein
MSLPTIPTITPSINLNRTQVINLLLASVALEELGLAHIINAEGEKLQAVLGTLPGLTTATSIKSLLSVNREVRRSLQTVLKNQMLLEFKLEDILDIPPG*
>Syntrophomonas_wolfei_subsp_methylbutyratica_JCM14075T_02032_hypothetical_protein
MPEFPPQKHARIPDLYHTIDLLDSIALEELALSHLINAEKIQYFGKIHGSCTPADVLIDFNAVVNETLETVIMKEWLLLRKLEKVTRFFHQRFPAPIPPRPPIPPCPPVPHPPCPPCPPRPYQHIYPCHPKSTCKISCKPRHKVKKYYRYCCEE*
>Syntrophomonas_wolfei_subsp_methylbutyratica_JCM14075T_02033_Putative_anti-sigma_factor_antagonist_BtrV
MLLDVSSNIEDNIPRITLDGRFDGLGAQLLEQEVERRISSEKQWLLLDFANVIYLASAGIRFLIKYGKKLARIDGQLILAGSVRDRAGGVLVSLEVIPRRQHRRPRPRHKVHVYCIQGERGHHNSYTRQAEAAQASNHEYAMRYRRHAAEHHHGGGGGRSGAPLHVHIQGGMGDVTVTDVGPDLSRTLAHGGGVGDGGPGTGGAGTR*
DLRSLIDLIKQELDGKTTGLLSVIGMVAIGQTRPGSGSYYQNYEIDNRQTLENNGGFRQIVWLGGNNGWQADEQLSMLMAQQIGPRGRNGSGSTGQGLLGQDWENMQEPGRILEYCSRAELQNKQMDVIMVHEHTALSQGRVWFVPGQIRSGEEKQLQLEVQGEIPFPEEWQRIITTRRLYADRRVVLEPLHGGSPGKPFVTVTGKQIALDSITSKIDLNKRYVCQGIYLNNST
TIMGQTTCGASTGMRYNFVGISGPDSKLDWLTHRFRERPAEELIPLFDEIFTHILKPWYGQPRWEMIKPYGEHNPLGLFPMVFAAAEAEFGISAQEKTIACPELGLLLPNPYHFLQNGNILLDERDNVYIIDFSETGFRNIVSDFARLEPIIPKFEMTRMSGEADMVAFLEMEQALARANSLDEVPTLVYRGDDPAVDKVYQVMCRIRKYAKTVVLFESDLIPLL
AMLEWTLPIVAFVNVTPWAKKAATYSAALLVEQIMRLESGDQPPG*
>Syntrophomonas_wolfei_subsp_methylbutyratica_JCM14075T_02034_Inner_membrane_amino-acid_ABC_transporter_permease_protein_YecS
LPKKLLMVGITILLITLAGCRENPAGGKLKTLDDLKDKRIGVLLGSIYDEYANKTYPDATILQYKSQSDLILAVKTGKADAAFFNHESSLELMRKDQELAYVGEKYVKPVGTGFNQENDELREEFNQFLQQLKASGVYDDMVKRWISNGISDMPKIDNPKNNGRLVVGVVSDKGLPFAIVKNNRIVGFDIELSERFAAYLGKELLLQDMEFAGLVAAAASSKVDMIASTIVITEERKCQVDFSDPYY
ELGVSVFGLKKNIARYENVQENKALTLEAELRKGVLTGSAGDNAARQRFPQAQVYDFDNIINATAALGTNKIDAVVFERNSLQRIAQQNPDLTVIEEAVKSVDVAIAVKKNNQLNQVNQVITDLEKSGTLAEMKKRWIGVEDAYSQKMIPDELMPVTETALRVGTEALMPPFEFVDSNNECSGFDIELANRIGQALGKKVAVSNMNFPALIPALQSGKIDLAISCFNITEERQQKVDFTRPYHTSNI
AAMVKKKPSDLGFFNNIKTSFYRNIIMEGRYLLIVDGLKVTVIILSFSVFGTLLGALVCWLRMSKNRGLQALARVYISLRGIPVLVLLMVFYVVFASVNINPVMVAVIAFGMNFAAYVAEMFRTSIGSVEKGQAEAGIASGFTPWQSFGYIIMPQAVRRVLPVYKGEFISLVKMTSVVGYIAVQDLTKASDIIRSRTFDAFFPLMVALLYFAISWLLALVLDYIAFKSDPIRNSKGGIA*
>Syntrophomonas_wolfei_subsp_methylbutyratica_JCM14075T_02035_Arginine_transport_ATP-binding_protein_ArtM
MIKTEQLICKRYGSLTVLKDVSTEISKGEIISIIGPSGTGKSTFLRCLNLLERPSKGTIVINNDLLLAKDTDIPKIRQKTGMVFQSFNLYAHLSVLENTTLGPVKLLGKSKAEANQKSLELLKKVGLAEKAHSFPDELSGGQKQRVAIARCLAMEPEVILFDEPTSALDPTMVSEVLAVIRRLAKAGMTMLVVTHEMEFARNISTRVFYMDEGIIYEEGTPEQIFENPQREKTRAFINRVRSFNYHIDNPNYDL
YAMNAEIEAFCEKHLLPPRVCDHILLLVEETLLLQTDFSDISLNLAYFEKTGHLEFRCEAAGEPVNPLAEGVQEDSDIGLKLQRIEDSQYRENHKNMLFFKVKSE*
>Syntrophomonas_wolfei_subsp_methylbutyratica_JCM14075T_02036_Transposase_DDE_domain_protein
MIPDNHILKRIDSAIDLSFVNELLADRYCKNFGRPAKEPEMMLRMQILKYLYNLSDEQIIQELSVNLAYKWFVGLNPEDPLPETSLLTKFRTQRLKDISMDIAIIEIVRQCIESGIIKGSNGIVIDTTHIEANTIKKVPERIMKQLARNIFKAMGQEEYEIPDYTQIEDHVAAKQIMKDYLEDLIEQVSKESSEEVIQAVQEAQEILESDIFLEQKGIRSLVDKDARVGHKTKTQNFYGYKAEICQTTNGRLSITSIT
IEPGSYVDGSNFKELHEETQKSGLTVTGVYGDKAYFRPDILNLLKEKQVTAYIPVSASAYKIDEKLFSYNKDSDQWFCVMGNETVKVKSKTRERNGKKKQKYLDYTFEKEICRNCPRHSECIMAIKAAKLMITSINTPELYEYSQRAKTPDFVEEYRKRAKIEPKNAELKRFHGLDRAKGYGLRSIRIQAKLTALSVNLKRIAKLVSALKGLHVLILKLLSPLVVNRPDCDWVAAQAA*
>Syntrophomonas_wolfei_subsp_methylbutyratica_JCM14075T_02037_hypothetical_protein
MLMVALFRHTCGSIAKYYPSSFWNKYREHIRNHDFQYPLDAFEENVYKENYSEIKTGRSYHQNGTIVYLYHICIKMP*
>Syntrophomonas_wolfei_subsp_methylbutyratica_JCM14075T_02038_hypothetical_protein
MKQPNIDFLMNMTKEYLDGKIDSIAYSLDFPHELEQRYKKAHREDDDYKDICELIYECLYEEGIGRFNELSDGEFKKLIRKQYNYIKKIAREGFY*
>Syntrophomonas_wolfei_subsp_methylbutyratica_JCM14075T_02039_Transposase
MRHHHEETTWQVNPRWGKKASSKYIWIHRSGSEVSIPYLYETRTRAGDHAKKFLAGFHGFHVSDAYEGYEKVEGITRCFCYSHLRRYYLEAIPLDSGKKEIPGSGGAIGRAYCDKLFRIERKWKELSPAERKKNRLMYSVPVLEAFFAWAETTRTRPENLPRKALVNTLNKKHYFTNFLLDGRIPLSNNLSEIAVKPVAITRKNSLFSDSVEGAEASAIIFSIVGTASANNLDAYKYLEYFRRLPNLDFK
PNHPVLGEYLPWSEKVQLECRMQNAGTQAEEKDEPRDETA*
>Syntrophomonas_wolfei_subsp_methylbutyratica_JCM14075T_02040_hypothetical_protein
VRQYRSRYRAVYGIGIRLALPAFHTFVHLLDVFHNQLGRDIPQLRAYFFLANNLQFTGADPTHSFFRVQNTLHGLSGKSCRQFLTTTFFAYGSFVFLDGNDARIGQREVICVFFTEQGSLSLCWRRFFTSAEPLLGGL*
>Syntrophomonas_wolfei_subsp_methylbutyratica_JCM14075T_02041_hypothetical_protein
MFQPVNLLLQFLVFVFKLSDALIFFVDSLHGGLLPGGFVTSIIPENWHFCQHFRAFSVNDAGSVNPVGVRFFLVYL*
>Syntrophomonas_wolfei_subsp_methylbutyratica_JCM14075T_02042_IS66_Orf2_like_protein
MQHIADKADHIYALGATDFRKQQNGLASLVALKFNLDPHAGTSVFIFCNKRRNALRALRWDGNGFILVTKALADEMKFQWPKNQGEVRDITKRQLEWLLAGLQVDQKKAYPDRIDTAGIIY*
>Syntrophomonas_wolfei_subsp_methylbutyratica_JCM14075T_02043_hypothetical_protein
VKSTAEKLQLWEQRIRERIQTGMTIGEWCKKNGVSKSQYNYWNRRVREKQKTGEETSFADITPISLSPDDAARQNPVFTSDFQIFKSIRVTVPGSFNPAALAGLMKVLQEL*
>Syntrophomonas_wolfei_subsp_methylbutyratica_JCM14075T_02044_Sporulation_initiation_inhibitor_protein_soj
VPKVIAIANQKGGTGKTTTTVNLGIGLVMQGKKVLLVDADAQGNLTDSLGFHEPDNLPASLSVLTKSMTEEPYEPNEGILHHEEGIELMPGNIELSAIEVSLVNTMSRETVLRSYINTVKDKYDYVLIDCMPSLGMMTINALAAADSVIIPVQAHYLPAKGMTQLLQTIARVRRQINPKLTIDGVLVTMVDNRTNFAKDISFILRRDYGDKLRIFQTEIPLSIRAAETSAKGKSIYLHDPHGLAAKAYE
AFTKEVMDK*GKIEH*
>Syntrophomonas_wolfei_subsp_methylbutyratica_JCM14075T_02045_Nucleoid_occlusion_protein
LAKSSARDSIKLASVDDLFTTTESRAFEQREKVLDIPLSEISDFPNHPFKVKADEAMLEMADSVKQYGVLVPGLVRPKADGGYEMVAGHRRKKASEFAGKETMPCIVRELDDDAATIIMVDSNLQRESISPSEKAFAYKMKLEAMKRQAGRPSKENASQVGTQKRSDQLIAEQSGDSRNQIQRYIRLTELIPPVLEMVDEKQIAFNPAVEISYLKEEQQELYDTMQSEECTPSLAQAQRMKKLSQDG
RLNMDVIFSILTEEKPNQKEKFNIQRERIDRFFSSFTEKQKEDLIVQLLESWYKRQGHEQ*
>Syntrophomonas_wolfei_subsp_methylbutyratica_JCM14075T_02046_hypothetical_protein
MHFTEGPLRAYERIMQEKPKHDSEPAKTVQDRDCKHCLHFDEHHHKCSKKKCVIFNE*
>Syntrophomonas_wolfei_subsp_methylbutyratica_JCM14075T_02047_SAF_domain_protein
LKLLKNRIFLSALCLILAAGVSFFLLPRFYESKSATIMVPAAQDIPAGTAIQDKHLVAAEVGSYGLPEDVINDKSLIIGKIAQTDITKGGLWLAETICTLHRPSVAAQMWHQSVSDFVAERGRLHIAEKQIHILQAPGGEFDDKVRDMTGLSFPFELPYVKRASELQNAGTPLYFHRDRACRHYGKVLEQIADRICGGGKP*
>Syntrophomonas_wolfei_subsp_methylbutyratica_JCM14075T_02048_Pertussis_toxin_liberation_protein_H
MNSRFSHRDLFRANSKSNIRGSENELEARYDVGEILDKLQRRIAQMPEEKFRRIQAFQEKEEKLSEAMQEALGIRLMYLNSFEQLVKSLNSRIDSIRLNSSSVEVSEEIALVLKRGSGVQRVQLQFSRLNKNRYDPISRDFDKGVKNNLEVLKSGEKFPKVMLQESLRDIMTSEKTPYSLMGKGGRVTEQRGKQAVKSIGYFGKQRPGKIQSKFLRRLAPRIQTALGKQHSLSEGRLYTPELAAITASWIFLRLAAAWLSERFQ
PEKRVVTIEDTREILNSEKFDNEGVMLNDVVHLLTKEAPNKTITNLLLDRLLSRLHPTVLVPAEMRGKEAMVVEAGRTGHTVISSLHANSARSAYDRLTTMCLEAGTSLSEGRLMKNIVEAFPVMVHLPKVSDLPDHSRKYMEIFEATGVQDGEVTGNTLFKYVVDHYKRDKAGRITKVGNLSRGRLGNISPALAEKLLVGGVPQKEIRRFAEGGLA*
>Syntrophomonas_wolfei_subsp_methylbutyratica_JCM14075T_02049_hypothetical_protein
MNPIHLSLMLVFVLVSLALFLWLGLNPFAVERNPLKKRRLYLAGGKLKTAERIAIRFATLFRQTRCTRKKFLMMFASAAGGFLAGMLLFGSGDLAVMAVCVLSAPYYLTIQSAAAAREEIEGLENTMSVISNAYAGCDDIVKAMELYVEEKNRYIPPSPENPYPP*
>Syntrophomonas_wolfei_subsp_methylbutyratica_JCM14075T_02050_hypothetical_protein
MNDAKSMQVESDGMMVRVWRDYLLTAGLMFSVIPMMRFSSNAEWFSILTTTAIGKLLVVLMILTALATAFYVLRATKPSNR*
>Syntrophomonas_wolfei_subsp_methylbutyratica_JCM14075T_02051_hypothetical_protein
LFYQTVIFILLLAAGFLICRELLKIPQASMKKNLKNLQEKEKPVIGRKGIRLNRFVMPLVRPVSRLIRDLPVQEARMASMLRRGGLNLTPREYQARALICAAFTLPLLPILLLLLGATNVAPVILFAVVVYFHFTSDLKDQLKAKKNRIEMEMPSFIRSILYKLDDIRGGNQTVVQVDLIQIFKDYLRVASDVFYSDISLVMEMKAKDIETALRSFNARLGLTDISDLTNALIGLIRGEHQREALAYLARDMDKARE
LKLALKNLKLPGRVKIASIPLVAVTLATLLYVIGSHMTQAMGGLF*
>Syntrophomonas_wolfei_subsp_methylbutyratica_JCM14075T_02052_hypothetical_protein
MQTIKNAASITEERRTDPMRKRMDNTLVKAGNVLAKRIGGDMTGWLLIVVLVVVVGAIFMTLYQGSITDIWNSIVDKITGLLN*
>Syntrophomonas_wolfei_subsp_methylbutyratica_JCM14075T_02053_hypothetical_protein
MMTALKNKIRMMGLKAGNVLARRDGDQMTGWLIVVLIVVVVGAVFMTLYQGSITTWNSIVSKITGLLN*
>Syntrophomonas_wolfei_subsp_methylbutyratica_JCM14075T_02054_hypothetical_protein
MNSNHRGRGRASLRPRPQKLLNKEGSSYFDLIKTLVVTLMAVVISFLFGFTTYLNLNHMCRRVVRVVLEGQVSNAAYDVFYRLKQQTGLSPEMTVEDVAYCDVQDQKIQLRETFTVTMKYSHAFAIFKPSFAPPVEIHIPMKVSIGGMSEKYWKLPE*
>Syntrophomonas_wolfei_subsp_methylbutyratica_JCM14075T_02055_hypothetical_protein
MRAPERGKAVGHLKRIMNNRKGDVFLFILLVFFILTSLVMLIEYFRMESLYQQVEYVLQRGVNVSVEYAIRDDYRRDGYSRLDSALAEEMLYAYLYENMGLDSELNKYAGNQWVYQLEVERIDATDSPPGLTLTGTLKTRSAFNFLSGTIRLPFTISSVNRIEEGGSP*
>Syntrophomonas_wolfei_subsp_methylbutyratica_JCM14075T_02056_hypothetical_protein
MIKKLIFTIPLIAVICLLLSAVAFALSSSSASNPEKRSAVELNYVAVTQPFTQENATAKTGVLTEVRLLEPGGYKIVPQEIKVEVCALVQTQDQGAVLVVTANVVFQVRATYDTVTRGTMNVKVDLQVDGSNEYIGLVYRVAANSIQVDFIEVNEKLKNSPQLRIDLVNANTVADPHEQARIAGWKGKHDGGTYTLKGSVLVEIHNISTPDNGKEKCKRGTSLVGKHHHVPDVHNCAAGGTTKYLSDAVTLLQLETRNEVCSKKTSIVSKRLGGRSLFARR
CDESKQEIFGKLNAQDKGVPELLPYPQTAAVTFHKDDIKLSRNYQYMGLEWDYTPETKKFTDGESKTQTEITQKIHYQIPAAHFYFKFKACDGNDLSVAIRAPATVNRGDDPFSTYIYMNSGKSPAYDVKPLKGTTDQVEIEIPAVDFQPNTSQTYVVKRTANTAAGEIHLWANIGVPEGFHIDGNLSNNTATAVIKIVDPAPEATPEPGDQPDQPDNPDNPSNPGNPPDDPPGMKESCDLSAS
ILAPPTVYEHEGYSFTVSFANHSDMDLKAVPLKGKSNENTLVSIPKTADFKPQETKSFTVTGQVTASAGRIGTNNTFSTVSVVGGSAVRKQKPYNGTTVSPRKEIRLRVVPVDVVEKPADVSLGDNLVTGKSLTSLSSPTAYEEEKYSSAVGVDVNSGWLTPTNGEVTGGIANGIASSGGIVQGGGTVLNPMQGMKASMAVGKAVIAPAVNGNDNNTKTTLALSRTALDKFASLTRTQYTSWLGGGGKGTVLGGSNTFT
LERPYDLDVQRITPDRYKENQTVISTIRVSNKGSMDFTPGQRVSVLFQIPELSKKRVDIVSGDAVVMEQNTNVSVRWDTPSVQTDKNITLIATINPDRKIDENETSTANNTYVTVEVRPWQDLMSQAIQPQPQRNEQPRVTWWERYENGQVFWRQYYAELRVNAALDYDTKAKGYLKSGYGYAIRVTATVNTNDYRPELITAPQTAEVYLPEHPRHYETAIPLVKEGGQFVRENPASPFRYRKQ
YIPVWFPDFRDCVQVLLVTDAHTPGGTLSKWITGGELKIYVDSMYDDDVTTGN*
>Syntrophomonas_wolfei_subsp_methylbutyratica_JCM14075T_02057_Flp_pilus_assembly_protein_CpaA
MWWTACTMMMSPREIDMLLCAIPLLWAALMDWKKRIIPDWTWSILLIGGVSAFLLPYPTPAQRIAGFLLPGICLLLLAVKYGGVGGGDVKLAAALGFCFGLNALAAILFFALPPACVYAKATRQRSVPLAVFLCIGFFIYAGVLIYGLTC*
>Syntrophomonas_wolfei_subsp_methylbutyratica_JCM14075T_02058_hypothetical_protein
MSEKRMVDTYEITHAIHVGDKEVLFAVDEKSDCPYMVCDCTWDNPLGIDHYFNAAGSADYLEMMAEFADRVQAQLEAVKAERDKITVPLSPFTTEHCTPNDYGENIENKVVVIRTERLRPEYRTADKQLVLAVSGFGTYANTRGRAVYTVNLYSGKESRWNREDILGVLKPEYMPDWAKDRLKQITQERQAKRKKQGQER*
>Syntrophomonas_wolfei_subsp_methylbutyratica_JCM14075T_02059_hypothetical_protein
MEKEINAGYVITDRALVGNAEFVIGQSDTAPAKFVTWKCRKGEDYFWGHYLGDRLTALEDLCNRALDEIHHLKLLQQDQENEVKPERPVKKRCEPER*
>Syntrophomonas_wolfei_subsp_methylbutyratica_JCM14075T_02060_hypothetical_protein
MAFKINAATPEEAAIFYSNAEKDKELGCIGHLRGDFGCGGREFWSTWWEHQGDLKTQAFKDEFDVFNSLMREQHHIISSPKIPGAWHLDVFGRFVDTDKHRYYIRCFPHGGDNFYIYCYINEKERLREQAQSKPIHKKKHEPER*
>Syntrophomonas_wolfei_subsp_methylbutyratica_JCM14075T_02061_hypothetical_protein
MSDLTRYRLCAGLPAPASAKLLYCYLLDMAGGRHNSVISKNLSKVGLSRSATSRNLNRLDMIGIVPRYSEDGGRLSSRNQYILK*
>Syntrophomonas_wolfei_subsp_methylbutyratica_JCM14075T_02062_DNA_methylase
LATKLQLITELSERTARSVTRSHANWTSFLKTSAWSKYKPFQDLIQFRQPDATACAPIEVWNGKLGRWVNKGAKGIALIDDSGSRLRLRHVFDVSDTNSRYNRPVPLWEMRDDYAEAVTESLENTFGELDEKNSLPYALLSAAANAVDNFTDYLPELLSFREGSFLAELDDLNAELFRKDFALKSSVAYMLLTRCGYDADQYLRFEDFQSMLHFNTLDTVSRLGTATSDISEMLLREIEATVKDL
QKAEQKQSRTFAKDRGTFAHNKSVKQSNKRSGNEEQSKDTLDHAAGRLSIDTPEGPAGGTNANQTPQQRDVAQNIPEKPPERDVRQPQDAIRGPVPDPGSGTPAPQAPQPGRPSIGGKRTGRTGEGTDLDQLTLPTTEEHQIETSFEEEKEAFSPQAEIDAVHRQGQYSRSGSVQDGKYRIYLHFQQNASKENADFLKNEYGTGGRTPVTGEEPLDNIDEWHDKGGITL
TSGKLTGPDTQLRLPWTKVQKRIGELINEGRLHVVRQKMESRQQQLAEEAYARELINEERPPPAAPEPPARENQAYAELSGLTLDTVQLGADEYEFSHEISFFDDSVTNLRDISFPLFGKEIAKSFERMLRETPLNDHLITAREAPEQPAEEKTEKPLARALYREYLPQVIDRYNVLREPLDRDTDPDAKQELDSALDRILLSMREEHPDVFEAYATLPQFREWLHEDVFERTYQDYLTEPRDLV
THADDPDAPEWVRQTGDTFTFDNEDGGDEKSDVELDRELPEAQAGETEKQPAEKEMAVGLELSIDGRRFSIDTIEEEAGTVSLRDTFQNGVGFPIFRERVGFGMEEDAQEAEVAEEKAEQYLASGISLTSPETEIEQQEPQPEAAEEFDVETPTAERYYSLTLQEDSEKEQIVSEVEATQASGHTKVTEKTTKKKPEPVVTTSKDQRRNVNTEMRIR
KAIYTCLENRNLLLKHITEAPVDEVMNRNYRDESDWGLPPGTFFRMPIKSQATPAHNTLRISLVESYEDEVQPLLGDRQWGGDVAMEYLAMHLSGMQVVIGQARLVAVQGLRPIEEHSLITQLHRDPFSVLQNAGRAEKQWLPHEQHTGRVAVNTNGLRRINWHIEALRDSWGEDIHKALRLAVQRLHTAEEKRRKWRLEAEKKETDIDPVERRQQNPRDLVRKIRTRTEHWHRHEKDWT
AETEYELDIGEDADSSIPADPSVRNFSYCLVDGEIYFRENSRMNRVETSVTAQGKRMGIELRDCVRDLIEYQTEDYGDEAILQQQRKMLRLYDAFTAKYGLINSRGNSMAFADDSAYCLLCSLEVLDENGEMERKADMFTKRTIRQRTSVAHVDTAAEALASIAIEKACVDIGLMQSLTGLSEERLADDLQGVIFRDFGKAEPEHAPRAFFQVESFPYVTADVYLSGNVREKLKQVRELAKILPE
QAEKIAVNIKALEAVQPQDLSASEIDVRLGATWIPPEDYEDFMFELLETPYMYQRIDVYLNYSLLSFIKFHLLEKLIRR WKYHGYRTDTEKRAAHGQTLESAKTVLEGLVHRHSCRREGYIPRQEFTFKGGSVRGTDPEQTPALNREMETEGQHPSGVRPLTEEIPEPVAPPETIGEEETSDEETTPTKVALKWAFDSDGFIIARNKVHLSKDSL
HVKAGGETLGTVKVRWPEGVEGYVSLLRLGSFGEIVDGLLLNGTEAEEVWKAYLDFVLLRYDRRYEPQLAFDKGAHPTIEEMLSTFGGNLQYLQFAGREYAQKNLITRRAQANGNQ
QAVVKFMPLAGGKGGRAVLTVFCTGSAAREPTWRDLVSEDRVINQQRGYLDRHYEEGSEHARERYSRAAHIIRQESCEEWRVADDEDRPETNQQFKATSGRSFLSSLSAERKDKKTLQTRRKAIETRAAHSDNASQYAETLYRCLLIHLRQRYIGVVNSQREMKRTPRNMFTASKQGDSSE
...

>Syntrophomonas_wolfei_subsp.metylbutyratica_JCM14075T_02075_Glyoxalase-like_domain_protein
MNIKFSSSVILVKNIVTSREFYEELLGQTVMMDNGPNVIYEGGFSIWQRDAALGIIFEDTPKPNIENALPAFELYFESSSLEQAWKRCSINPERVIHPIMEHPWGQQGFRIYDPDGFIIEVSEPLPVMVQRFIKEGYSMAQIAEKSGMPESLVCFMAEQSD*
>Syntrophomonas_wolfei_subsp.metylbutyratica_JCM14075T_02076_Bacillibactin_exporter
VNKWLKVTILSISLLTVMSGAAVAPALAEIARAFPHIGSNSIKMVLTVPCLLIILGGLISGRLVSVMKKRSILLIGLSLYLVGGLGAGMVDSFSALLVLRGILGLGVGLIMPLSISLIADYYTGDERGRLMGMSTAVSSLGGIIATVLSGIVAVISWRWSFSIYALGFAVLFLVLCFLPKLEKESAADSEFKAQPLPSSIYYTAILTFGLLIVFFAIVTSALFIQNQGWGNANTTGALGLTNIFVFGAGVKFQTVYSRFKSYTVPGGLLLLVAGFALLSLSHNIYTVLAGLAVFGLGFGILMPLIYLQTTSVVSEEMRPRATALVGGCFYLGQFVSPLVLSLVGIISGNYSIRMIFTVLTFALLAAFIVSAVMTIKKSHTLYHESNETM*
>Syntrophomonas_wolfei_subsp.metylbutyratica_JCM14075T_02077_hypothetical_protein
MNNSDEKFKILYLSKRLICVVPLLICNRLYKEMIL*
>Syntrophomonas_wolfei_subsp.metylbutyratica_JCM14075T_02078_putative_HTH-type_transcriptional_regulator/GBAA_1941/BAS1801
MLIKDSKNKNKISFTLLRIVWKLLELDKKTRNYGTDEPLFEAEIHMIKSIKENEGIHVTGLADLLEVTKGAVSQIVMRLEKKGMVVKDKDDSNQSKLVLKLTPKGETAYVNHEKLHQEFDDLIHEILKEASPEHIAFLTDFLDLLDNRIDDYEKGKSE*
>Syntrophomonas_wolfei_subsp.metylbutyratica_JCM14075T_02079_Multidrug-efflux_transporter_1_regulator
MLTIGEFSKISRVSTKTLRYYDQIGLLKPGFVSKDSGYRYYEVSQLRDMLLITRLKRYRFSLPEISAVLATRDNSRLGELLQAKKEEFLLHINDQQHILLQMEQDIAKIERCENIMQADYLVKTVEMHPMTIFSHRKNMGLEDLDAAFAAMFAEMERKKINPSGPYIIIYHDEVFDHDNSDMEIAVQIAGGAGEHIRDLSPGFCCFATHIGPYDNLGECYAALSEWIEREGYVIAGPPFDLYVKDFSHGVSLSEYVTEVYFPIKK*
>Syntrophomonas_wolfei_subsp.metylbutyratica_JCM14075T_02080_Putative_peptidoglycan_binding_domain_protein
MRKGYFFFMILVLASAVILGSTIGLISNVSYIVQHTDLLKTQSAEPSSEGNINDATTPVVPQPAGDASSQDLNQANSSSSFASSSSSSSEYMVISQEEKEQIQRMLFSLGMDEKDDLNQFVREFQKKNALEATGILDSQTLDTIIKQTTLQEVSRSLNRGINV*
>Syntrophomonas_wolfei_subsp.metylbutyratica_JCM14075T_02081_Arginine_utilization_regulatory_protein_RocR
MSLNLELHSHDLLLYPILQNIDEGILITDTSLNIIFCNENAGKILGLNPNQILGQNVDCLNPHLTFDNILKGPHRIVKDIKVINEDEVNVTRGLLEIRPKCYAAYAILRTIDSFKEYQLNSLLQTPYEGIAVFDKNLQVMMLNREAEKILEVPRKEVLGKHILDVLPLSHRVMVSEDFGKNMRSLLDMLGVIIDREYYQGGRLTITKYEIVGHDLKENVGKLQ
QKALAARAARSVSTVLITGESGTGKEIFAHAIHNMSPRRKGPFVKVNCAAVPETLLESELFGYNEGAFTGALKEGKPGKFELANHGTIFLDEIADMSLSMQAKLLRVLQEREVERIGSLSSTKVNIRVIAATNQNLAELVSENKFREDLFYRLNVIVLNLPLRQRVDDIPLLSHHLLHRLNQELGTRVKINPEVIQSFCSYDWPGNIRELENTIERAVNLCEGNTITIDNDIPQIRQKNHYSSTTGSHSSL
GNILGKQEKALIEEVLKNCGGNKSKAAKTLNIHRSVLYRKLHKYDIKL*
>Syntrophomonas_wolfei_subsp.metylbutyratica_JCM14075T_02082_putative_3-hydroxybutyryl-CoA_dehydrogenase
MGFNNVAILGAGTMGQGIAWAIAAGKNVILYDIDMKFVDKAIGAIKKGLTKAEEKGKAAPGTADAVVGRIKGTTNFDDLAAGDLVIEVVPEVMSLKKEIYTKLDALMGPDVILATNTSSLSITEIAATTKRSDKVIGVHFFNPANVMRLIEVIPAVQTADATVDAVVEFSKEIGKTPVKVKEGPGFVVNRILLPGMNEAAFIYYEGLASAADIDTAMKLGAGWPMGPLTLCDLVGVDIALAVCNTLFAETGDSKYRPAPPLKQLVRAGWLGMKTGKGFYDYKK*
>Syntrophomonas_wolfei_subsp.metylbutyratica_JCM14075T_02083_putative_enoyl-CoA_hydratase_echA8
MAFENILEHGGVILIINRPKALNALNAETLYEMEEAIKDIKADSNIKVVIVTGVGDKSFVAGADITFMLPLSPHEGRFFSDVGERVMRQFELMEKPVIAAINGFALGGGCELAMACDIRLASEKAVFAQPEVGLGVIPGFGGTQRLPRLVGEGRAKELTYTADVINAQEAFRIGLVNHVYPAEQLMDEARKMAKKIAAKAPLAVGYAKFAIGKGMQVDIDTAMSIESDMFGMCCATEDKTEGMKAFVEKRKPTFKGK*
>Syntrophomonas_wolfei_subsp.metylbutyratica_JCM14075T_02084_hypothetical_protein
MKKLVIILLLFMTIILNACSDVRESNNFEDSQQPLTKTQIDEITAATESVIVDVDFSQISKPNPVQQTEINERMHCLGEKLKGKVTIENRNIVVARVNGETTTASNWYWEKINKILQAEFNNKSIPSDAEILSDLIETKVISSAARSLGIYPPEAQIKAYIADQQKYIEILKPEEITVLIQAWGISEEDYFLLMENRFADSLAKINWGVYLQKYEDEAQEDEQGYVAKSFRSIFGDDRIKPLVEKAKVEITSEGRQLGISYPAPD*
>Syntrophomonas_wolfei_subsp.metylbutyratica_JCM14075T_02085_DNA_alkylation_repair_enzyme
MSDIEKEIQHELFELQDLKYKEFSCKLMPTVNPETVIGVRTPDLRKLAREISKMPYSEEFLKLPHAYFEENNLHGFLIETITDYDAVVAALDEFLPYIDNWATCDLISPKVFKKHLHKLYGKIKAWLKSDRIYTVRFGIGMLMRFYLDDDFRSEMIEFVACIRSKEYYVNMMIAWYFATALAKQYEAALPYIQEQRLEKWTHNKAIQKAIESYRIGTEEKAYLRTLKVK*
>Syntrophomonas_wolfei_subsp.metylbutyratica_JCM14075T_02086_Helix-turn-helix_domain_protein
MSMDKELITAQIALAEALHLSVETIWRYTREKKIPYIELGNKQYRYKLSDVIDALVKASNGSIQESASPYEETEEPRKLTYQDYLLPEEPGYHFEILDGMLIKEPAPNVMHQRVSRRLQRILEDYFWKVDPKGEVFNAPLDVTLIHDVNVVQPDLLYVASEQEVIVKDTRIDGPPMLVVEVMSPSSTRKDRLQKRRIYQEARVEHYWLVNPQEKTLECFAWHDGVYALVAGGMEEELVEHPDFPGLSIALKALWNREI*
>Syntrophomonas_wolfei_subsp.metylbutyratica_JCM14075T_02087_ABC-type_transporter_ATP-binding_protein_EcsA
MEICRVSKSYNRGQVKAVDDVSLTVQGGEIFGLLGPNGAGKTTLIKMITGLLNADRGSIKINGRDIKDSPLEAKMQFGFVPDDPNIFSRLTGIEYLNFMADVYQVSSEIRVERIQKLLERFEMSTAADLIQSYSHGMKQKIVVGIALLHNPPVWILDEPMTGLDPRSSYDLKEMMREHADAGKTVFFSTHVLEVAEKVCDRLAIINKGRMIFCGSLEEIRTHARENRSLENLFLELTENA*
>Syntrophomonas_wolfei_subsp.metylbutyratica_JCM14075T_02088_hypothetical_protein
MHRFWALLKILLINYFGISFQMKTSKNRKPYFKKIGLVLLIIVALAPTIALYVVNLYLKIFGALAPLGQEGLSIGLTLGIVLVFSLVFSIFGFIYPFILFYGGKDLKILLPLPLVPKYGIKSGAIRTIIYLVGALVFGLLLSIYYALVALVGFALVPFVGFSILIFVILAVLAFLIAVLIGLVLLFLASVSSLFAAVNYLAVAGHTGRVAAIALALPFFILLLLGIVLCTLFFLGLASPVFGFLKAFFSFDLYGL
SGLVNLLVFAGLSLLTVVALAWLTGKDKLYFGLVGSTETTVKRKKLSEADYRRAGKGLPPLLSYWAKEMRLLFRTPTYFINSVMTVILLPLMIIGMTWLPMLLLRVFSGDVLVVIPLLGLLVLLFFTSLHTLLNLILTLTLGSWVIVGMGLALGHTLLPAALLLLPILLILPSLKFSVLGILKVNFPLTLLAVLLGGFSFAVGM
IIYIIYIIYHTPQVAALFILLGFGLALFLYYFLMYLFMTWGVRRCHLELEG*
>Syntrophomonas_wolfei_subsp.metylbutyratica_JCM14075T_02089_Pyruvate_kinase
MRKTKIICTIGPASEQIENLLLMIEAGMNITRLNFSHGSYEEHQKRIANIRFARKKAGVPVAIMLDTKGPEIRTGLLKAGKVKLEAGQRFVLTTRQVDGDEHLVQITYDRLPQEVEKGNSILLADGLINLQVLETSDTDIICQVVNGGELGERKGINVPGARINMPFLSEKDKEDINFGIEQEVDFIAASFVRTADDVLEIRRLLERRDADIDIIAKIESQQGVENLEDIIKVADGIMVARGDLGVEIPAEEVPLVQKIIIEKCSPRGKMVIIATQMLDSMIVNPRPTRAEVSDVANAIFDGADAIMLSGETAAGKYPVESVKTMARIAREEVLPYQERLQRRLQREGLSITDAISFATCATAMNLNASAIITPTVTGLTARMVAKYRPQASIVAVTTHEKTRILNKLALVWGVYPLLIPETSGTDELFEESIKAALESSYINNGDLVIITAGVPIGSPGGTNLLKVHVVGRIMAQGMGIGKDPVTGRVRIVIYPEDMEKIKDGDIVVTTSADCALAPHISRIAALVAEEGGLTSNAAMLGLNANIPVIVGARNATGLLEDDMLVTLDTPHGRVYQGIARVR*
>Syntrophomonas_wolfei_subsp.metylbutyratica_JCM14075T_02090_6-phosphofructokinase
LQKIGILTSGGDASGMNAAIRAVVRTALYHQMEAYGIHLGFEGLMAGNFDPMSRGSVADIIHRGGTILQTSRSQIFMTGNGREKARDELQKRDIQNLVIIGGNGSLRGGYELSKLGINVIGIPATIDNDIVYTRSIGFDTAVNTALEAINRIRDTATSHGRIFIIEVMGRHCGEIALAAGVAGGAESILIPEIETDLDEVTRKIKQGTQRGKLHSIILLAEGVYPVMELAQEIEKRTGKDTRVSILGHTQRGGTPTAVDRIMASRMGMAAVDFIVEGKRNVMVAEEGDRILPVPLQEVIGRSKTPEIAMFEIARILSI*
>Syntrophomonas_wolfei_subsp.metylbutyratica_JCM14075T_02091_Tryptophan_RNA-binding_attenuator_protein
MNDSGYIGEQYIVVKALENGVTIGLTRGKDTKFHHTEKLDKGEVMVLQFTEHTSAVKIRGHAEILHRYGMSTSESTISSKNP*
>Syntrophomonas_wolfei_subsp.metylbutyratica_JCM14075T_02092_DNA_polymerase_III_subunit_alpha
MPGSFVHLHNHSEYSLLDGACRIKDLVNRVEELEMPAVAITDHGVLYGVIDFYREARKYGIKPLIGCELYVAPRSRLDKEARIDDNLYHLTLLCKNELGYRNLVQLVSRAFLEGFYYKPRVDQDLLEKYHEGLIALSGCVAGEIPRLLYQEQLDKALRLAEDYLNIFGKDNFYLELQDHGLQEEKAQSLPGLLRSLRDRIADEQLGEVQQLLKNYSEHEDLE
EEMAALFPDCPEALENTLKIADECNLDFTGEYHLPVFEVLPPGHSAEFSLEMVHEKFRRKYPDAQEKIIERLEYELRVINEMGFAAYFLIVQDLVNWSRQNGIPVGPGRGSAAGSLVSYVLDITTIDPLRYDLLFERFLNPERVSMPDIDIDFCEKRDRVEDIVEYIISKYGADRVAQIITFGTMAARAAIRDVGRALDVPYGQVDRIAKMVPAELGVSLERALEISPDLIFTAYQSDYNTRNILDIAQAIEGMPR
HASIHAAGVVIGRQSLSELLPLQKTPEGHVLTQFTKETVEEIGLLKMDILGLRTLTVIDRALEIIKKTRGEDIDISSIPLDDEAVYKLLSAGDTAGVFQLESDGLRDILKETVPNLEDIIAVIALYRPGPLGSGMVEDFINRKNGRQEIEYIDPLLEDILKETYGVILYQEQVMRVASELFTMGEADVLRRAMGKKKAEELMAQERDKFIAGAARKGIDPHIAAKIFE
QTAYLKAHYPVEYLAALLTSSVDNQDKVVSYLRCRELGIKVLPPDVNESFYTGMSPEENNIRFGLAAIKNVGQNAVKNIVEARKQGYFTSLFDFCRRVDLGQLNKRMLENLILAGCFDSLDFSRKQLIFMYMLEEENRRYVAQQATAQKMLFGDDSSGEVSVEEQGGETFEEVQ-VKILKGEYEPEAIREAFQEGSRSLAFQQAMAREWAELLIEALKRFQ
VFPSAYRKNGDRLEADRAVIVEGYFDRREQPKIALRKTGPIPESLREMHLRLAGNGNGEMDRNKLVKLMQKYPGDIEVFLFLPGRRIVLLNEKFNVSPSQDLKQELAAIYGPQNVVFN*
>Syntrophomonas_wolfei_subsp.metylbutyratica_JCM14075T_02093_Cys-tRNA(Pro)/Cys-tRNA(Cys)_deacylase_YbaK
MDEIERVRLYLSEKNPELKIIVLEEDTSTAPLAAQALGTEVGQIAKSILFKSNGDKYFMIVAAGDVKMDNKALKQLLGSRPRMANAEEVLAVTGFNVGGVCPFALPNPIPILDESLKRYDVVYAAAGTANTALPISYEELMRLTGGSPCRVARDN*
>Syntrophomonas_wolfei_subsp.metylbutyratica_JCM14075T_02094_Phosphoglycolate_phosphatase
VFKAVLFDLDGTLINIDMDFFLQKYFARMQKMALEAYSDSKGLVEQVFKSTGVMIKNRPECNNEIFMRDFFSNWRYPEKDFREFFECFYREFYLPPCKPFAGVPEMMEKLFDQDLKVVVATNAVFPLTALQQRLNWAGVGHFDYDLITSYEIMHFCKPHQEYYQEVAEVIGVKPEECLMIGNDVGEDLPAGKIGMKTFLVEDMLIDRGVGLSPDWRGKLSGLFSFMEKI*
>Syntrophomonas_wolfei_subsp.metylbutyratica_JCM14075T_02095_Carboxymuconolactone_decarboxylase_family_protein
MATALPWFVEQYKEKDPEYFALLEGSLSGAVNTQRLDARTYLLILALDVSRKQKGTAEGVKKALSRQAREAGASEEEIRAILAYFVNSMDLLTKTAVNAF*
>Syntrophomonas_wolfei_subsp.metylbutyratica_JCM14075T_02096_hypothetical_protein
MPFIFNKKEKAELSRSALIRVSPREVLYYWPVSLGPWEQDVIKRLAAESGKANPIKEWLELFEQELNADADLSSLNDNEFIDSGPYYYLPGNTRFYFDKNFRNPVDMVSSENLASLAALATILPLNNEIQANCKIKKAKRKATKSKDELLKDINLCLTSLREIERLNKQINYWEKILEQRYFLREREDLFPAEPDSLPQKPEKPAEIEVSDNVLPFSRLLSRQKKQHNLDLNHYNHEIKVYFIRYREYEK
ACDRYKEALENWPEYHQIFLDNCLNDIKKAEEKLNTARQNRQTYSEVIQKSMVHSAYQDIRTLELFKYYLKTGRANELQDCMNIFEEERNWTEIKASQERIENTIHFLQSANPDTHFADEHINLFLNHFQEKTGDLAKVGV*
>Syntrophomonas_wolfei_subsp.metylbutyratica_JCM14075T_02097_rhomboid_family_protease_GlpG
MIPLRDSTPSNTFPLITVTIALNLAIFFYESSLGLEGMAKLFSVFGLTPLNYSQGYVPLLAPLFTLSMFLHGSWMHVIGNMWILWLFGDNLEDRMGRGHFILFYLSCGILAALTHYFVNPLSSVPVVGASGAVAGVMGAYFLLFFKARVLTFIPPFFLFNLPAWIYLGFWALTQIWCGTASIFFSDACGAIAFWAHIGGFLAGMVLYKLFLKKELPLYTV*
>Syntrophomonas_wolfei_subsp.metylbutyratica_JCM14075T_02098_hypothetical_protein
MIMLGDEDLIFEEVKANLIKQTNIWLVWVLLLLVNTEIIIIIVTTSLVLIFFLLALVGIKVKSWANVEDIQVELQVSKEAQELMQGVKHIGEFIFDQEVQKFSRFVNSIYQDFSRSLSWLWEEKDVFIERTEGMILELKSIPALIVSLNEEKGKKLIKNLLNNIEISSLLNDLKITRGISLKEMEQFLKKLEDLRSTMLKEKDIFYDVYKLLLAQIRNREEIENEEFNLEEYFNVNSLKEGQFALNLEKSL
ETRTMAFQDGIERDMEDISADVVGKMQNCTIQLMNSFSEIHQILSRLLDESENEDQMLLQRLNQCCIKIAKLKDEAEEVMLTLAWQDILVEKRWGEIVQLLEAEEIPSFAGEMQNRDKAVFAKALVDAELIYQLYSGNKLSEVIRDGSYPLLMFIEPVDYLVRRNIRVTQEGLNRRISDLRNVSDEYEKLFQRVIEALESQNRELVQYIKDLFPRSFYSYCNNPYLKQ
RPENLNQAAWALFIDLLDDEYDNSGFYLVGILLVIHQIRNKYIHPFKALPRDLEKETELDYMRNCAIKATDILSNQQAVSKADSA*
>Syntrophomonas_wolfei_subsp.metylbutyratica_JCM14075T_02099_Acetyl-CoA_acetyltransferase
MAREVVIVGCRTPVGTFGGTIKDVSAIDLGALVMKETIKRAGIKPEQIDEVIMGNVIQAGLGQNVARQCMIKAGIPKEVTAFTINKVCGSGLRAVSLAAQIIKAGDADIIMAGGTENMDKAPFLLPNARWGYRMSMPGKGDLVDEMVFGGLFDIFNGYHMGITAENVNEMYGITREEQDAFGFRSQELAAKAIESGRFKDEIVPVVIKGKKGDIVFDTDEHPRKSTPEAMAKLAPAFKKGGSVTA
GNASGINDGAAAVIVMSKEKADELGIKPMAKVVSYASGGVDPSVMGLGPIPASRKALEKAGLTVDDIDLIEANEAFAAQSIAVGRDLGWSDKMEKVNVNGGAIALGHPIGASGARILVTLLYEMQKRGSKKGLATLCIGGGQGTALIVEAI*
>Syntrophomonas_wolfei_subsp.metylbutyratica_JCM14075T_02100_Acyl-CoA_dehydrogenase
LPHDNYLYQTRDIKFQIKEWLDIDKILSLDAYKDYYGADDFDAICDVNYKICRDVICPANKDSDEIGMKHVGGNEKAVISPDVKTVYNTVIEAGMGPGFGDRQVEGRMPLYWYAPILEMQTGASPAIVMLWCLTQGATTVLQYNLSQELQERFLPKMYSGEWGGSMCLTEPGAGSEVGAVTTRCFPTDTPGLWKIKGQKCFITTGDWDGVENIIHLVLAKDPDAKPGTAGISCLVVPKFWV
NEDGSLGAWNDVVTTGIEHKLGIHGSATCSLAFGENDKCYGWLVGGVGNGMAQMFQMMNEERINTGISALGAFAAYYNALDYAKERVQSKKSTDPKGPSVRIIEHEDVRRMLLLQKSYVEGMRALLYSSYYIDLSKKAEEERELAEDFFMGNQYLAKAYSDMAWQMCAEAIQVHGGYGFMEEYPASLARDCKIYSLWEGTNFIQSQDFTGRKFTMKKGEPFKKWLAEIGDFIA
NKKTPEFAAEFAMMEIRAFASNSVDMNAAAVTTTNKQLKQLFMRIVNHAARVICGNMLMLDQLLAASKLAELGDSHFDANFYKGKIASVKFYIMNVVPEIFGTEAMKAADTSALDCPEDAFM*
>Syntrophomonas_wolfei_subsp.metylbutyratica_JCM14075T_02101_Nif-specific_regulatory_protein
MKVYEVNMPRLPLTRRTQTIGEVSRLFDFSLDASPVLNEELQVGLLITKNNLFLVQSLSESPAIEVQDMLTDPITIAYIDSDVYEVLRIMETGRLPVVRNGQLVGMLYYSDLVRAVLTKELQKSKGDLEAMLDAAEFPVHMYKGKFTCRPVMRNGQLVGMLYYSDLVRAVLTKELQKSKGDLEAMLDAAEFPV
DGLYVTDGQATLRLNKQFERITGVTAAEVCRHMDDLVKEGKSRSGTLLAEKKRVTISLVSMNDNELLVTSNPIFDEEGNIILVVTNVRDITELNELQRKLEQVEGLTQLYRSQLEQIKLQTSRQLVFRSNTKMKELLDLVLRVTGVKPNVLISGESGVGKELIAEIIHSSSNRKDGPFIKVNCGAIPENLLESELFGYEAGAFTGASKSGKVGLFELAQGGILFLDEIGELPLSLQVKLLRFLQ
MLRVGGEQPITVDVRIVAATNRNLVEMVAHGRFRLDLYYRLNVIPIVMPPLRERREDVIPVAYFFINRLNSTYHTNKRLYPAALEILGQYNWPGNVRELENVIERMVVTCSEGMIDSFALPHPSINNILSMQDLPASFKVSRTIRGEQIYRQLLTQKYREFSGSKVSPPIALALLKNMSAQKSQYAAALQVGRLTLYYKKIRELHSTRRRRQYEV
>Syntrophomonas_wolfei_subsp.metylbutyratica_JCM14075T_02102_H(2):CoM-forming_NADH_oxidase
MKALEIKENVFYVGVQDYGLRVFDIVMRTEYGTSYNAYLVKGREKTVLIETVKDKFFEEYIKDLQEIVKLSEIDYLIMNHTEPDHSGCVEKLLEIIPGLTILGSQTAIRFLKEISNKNFSREILNHGDELDLGGKTLRFISAPFLHWPDSMYSYLPEDKILFTCDSFGSHYADERVFNDDIFDFTDAYKYYFDMIMGPFKPYVLEALEKIKDLEFDIICPGHGPVLRQNLDYIIELYRQWSTPVEVADGEKK
PSIVLAYVTAYGYTEMIADSLVEGIKESIGSIMMGDFDIKRYNLSELDYYQEAKSLMKAIAGHTHGPIWDEISGGYKGVSSPAPLTDVDPRITEAPKIYYGVSPEAALRDMSQPLNDKSPIRPDAGSFSSLGMEFAGRAMVPGDAVAFLVGEKAPGADPRMIGPPNPGFKAVPLADEGRGLVPEAYNELNKLGRMTLKSESLGFKLGRYISQKAAAGDPFAVSEENYPLGCYSMRAFKLKKTVS
KRLVVFDAAWYQENAIEVKTGFKVNSIDTAARTVSGDNGENLTYNKLIVASGARSNIPPFPGVEKEGVFAIRNLKDAIKLKEAIKTSKKAVVIGGGLLGLEAVWEMVSAGKEVTAVEFGERLMPRQLDDSSSARLADIIRSKGVQLYLGKATEEILGEARASGRVEAMKLLDGRQVLEADLVLLSTGVKPNVELAIEAGLEIGKQGIVVDEKMRSSAPDVYAAGDVAQHGDERMIGLWPVAMEMGRIAGSAAAG
DWLEYKEPRISTNLKIFEIEIFSIGEVNLPEEKREYEVKDPIEDYYKKSFKGVILVGGEIISRVDSESMQNLGRDKSGKQRHNRWKCRICGYIHEGPEPPDICPVCGAPKDMFDPID*
>Syntrophomonas_wolfei_subsp.metylbutyratica_JCM14075T_02103_hypothetical_protein
MPYIGIIGERRIFLNLIHFRAFFPIMYIGSLNGRKLFKGGYDNEGTGN*
>Syntrophomonas_wolfei_subsp.metylbutyratica_JCM14075T_02104_Calcium-transporting_ATPase
LIPYTANNEELLNHLQVNPEKGLSSTEASKRLAEYGENLLQAAKKKSMLQRFWEQFKDVMIILLIAAAISFVVACYEGEGFYEPVLILLIVVLNAILGVVQESRAEKALESLQKLSAPNARVMRDGKESIIEASFLVPGDIIELEAGDFVPADGRLIESASLKIEESALTGESVPSEKDAAEVAANAPLGDRRNMVFMGSTVVYGRGVAIVTATGMNTEIGKIARLLDAANDRQTPLQRKLAVLGKYLGLLAL
ALGICAVIFIIGLIDGMKVMEIFMIAVALAVSAIPEGLPAIVTIVLAIGVQRMVKKNAIIRRLPAVETLGSASVICSDKTGTLTQNQMTVLKALADGASVLEDIGESNNSAVIKKLLQYAALCCDVTEFEDGKERHIGDPTETSIVLAAHKNHLPKDELNRYPRLAELPFDSERKLMTTVNRIDGKYMVIVKGAFDVLAARCIAGDLEAARRFNENMSRQALRVIAVAYKEIETLPETASSEELDKLIFMGL
LGMIDPPRPEAREAVAVCQRAGIKPVMITGDHVETASAIAKDLGDLQEGDQAITGSELANLSASELDEQVRQISVYARVSPEDKIRIVRAWQHQGEIVSMTGDGVNDAPALKAADIGCAMGITGTDVAKGAADMILTDDNFATIVDAVKEGRGIYDNIKKTVGFLLGTNVGEILAVFMAMILWKASPFLAMQLLLINLVTDGLPAIALGLEPVESDVMERKPKPKEESIFAHGMGTRIILQGAMFAL
LTLIGFFMVWQTSGDIVAGRSMAFLVMALSQVIHSFNMRSSHSLFKTGIFTNRYLNGAAFISLALLAVLFLPPVAFAFGLTLQSAVMYFYALGLALVPLVVLEFSKAFGLVKHQA*
>Syntrophomonas_wolfei_subsp.metylbutyratica_JCM14075T_02105_hypothetical_protein
MFIGYNINQLFIAFIAPCSAPRAEVAGWRWDIIKMHGVLFTRDILGALLRCSAGNNSL*
>Syntrophomonas_wolfei_subsp.metylbutyratica_JCM14075T_02106_Glycosyl_transferase_family_2
LQMHYSTNTVRLNRDKLHMRQQGVSIFTCTNRPECRDNIANFFRQDFSPKELLIILNSNSMNLREWRMWTAGFPAVRVFQVDESQSQGACLNYALNYINMGYAAKFDDDDYYAPGYLEQMEVFPVSGAHVVGKSSWFIYFENSQILAIFNPNHENSHEEYLTGASLVIKREVFQEVKFRDFSVGEDLWFLKDCMKGIVIYSSSRFNFVGIRRPNPESHTWQESEEQIMPRCEFAAHTADYRPLIR*
>Syntrophomonas_wolfei_subsp.metylbutyratica_JCM14075T_02107_hypothetical_protein
LQVKVSEFVGESRRSWQDAVENAVSEASRDVNNISGVEVVNWTANCADNKIVEYKANIRIAYLG*
>Syntrophomonas_wolfei_subsp.metylbutyratica_JCM14075T_02108_stage_V_sporulation_protein_AC
VNTPDPKRWKKIEVEEYENLVQQTKPKPLLKHCIWAFIVGGLICLLAQLIMNYLLNQGIGKVDASSYTSVIMIFLGAFLTGIGIYDELGKKAGAGSIVPITGFANAIVASAMEFKREGYIFGVGARIFTVAGPTLLFGFVVAVLIGLIKVFLSS*
>Syntrophomonas_wolfei_subsp.metylbutyratica_JCM14075T_02109_Stage_V_sporulation_protein_AD
MPAKKKGRKAGLVFDKPVTSWASIGPKEGQGPWGSEFDKEIEGGLVTDLEQMPEDRKSVAMLTGEMSRLLEVFHAKLAENKMLRETVKKALNKRNFDEADAEILMAGDLLNQLVSSNYAARELGIPFLGLYGACSTMAEALLLAALLVDGEFFERVIAAACSHHYTAERQFRFPTEQGVQKTPSSQWTATAAGAVLLEPVGTGPRITAVSIGKVIDMDQKDISDMGSAMAPAAHTIKTHFNDLNRSSDYYDLIVTGDLGRVGM
AITRELFIKDGIQPEPNYSDCGVILYDDFQEVNAGGSGCGCSACILCGPILKKMTAGEINKLLFVATGALMSPITSFQGESIPAIAHAVAIEV*
>Syntrophomonas_wolfei_subsp.metylbutyratica_JCM14075T_02110_stage_V_sporulation_protein_AE
MVPLYMAFLIGGLLCLLGQLIMDLTRASITPGHILVGYISGGAVLSGIGLYEPLVKNIGGAGGTGTLKAVEQKGLAIAGALFGSFGVGAFGSGGVEAAAAGITAAVVFGYLAAVLFNPKG*
>Syntrophomonas_wolfei_subsp.metylbutyratica_JCM14075T_02111_Spore_germination_protein_B1
LFKKGFHLTLIGKDYIDNLIMKKELAEGDNFDLIVREVRVGLREAAAKLFCIFKDMTTECMKSLLALKKEVFLSSTVKSILEKGVPYVELTTENKIDELLYSVLSGSVLFIIDGSEEAFIIDPRTYPARNPEEPDIERVVRGSRDGFTETIVFNTALLRRRVRDPKLRIELLQAGKRSKTDICVCYLKDVADPALVEDIKSKISGIAIDALPMAEKSVEELIMPGNWWNPFPKVRYTERPDVAAVHLLEGHVL
VMVDTSPSVMILPVTFFHHLQHAEEYRQSPSVGVYIRWVRFIAVLASIITPLLWVLLLALSPEILPASLKFIGPKEGNVPIFLQFVIAEISLDMMRMAAIHTPSPLATALGVAIALIGDVAVRGNILVPEVLYTAMVAVGSVTTPSYEMAMANRLARFFLLGTGFFRLPGFIVASILVILLAAFTSLKVPVLWPLIPFNYRALKGLVRSPVPIQNLRPEILNTQDQSRQPSPAFKRRREK*
>Syntrophomonas_wolfei_subsp.metylbutyratica_JCM14075T_02112_hypothetical_protein
MSRVGIPISLFYYTYPLMKSFFLDLGVEVLTSNPNSDKTYEEGYGAAEVDETCFPIKVYLGHVAELCREEPDYLFVPRLVSVEARSYICPKFMGLPDMVRALIKDTTPPLIDITVDLSRDEKCFKKDIIRVGRLFTPYVQRPKIQLAYQHGCEELRLCRSLAAGDGGFTMEEAIKIWAEEGGGAMEAERETDLNIGVLGHGYSIYDERISMNLVRKLRAMNCKVIYSPEHLGSQEVEAEATVRKRVFWTLGRKMLGSAL
HLSQREDVDGIYLACFGCGPDSLVGEIIERKIRQNKNPYLTLTVDEHSGEAGLITRIEAFCDMLERRRRNCGSESKLSPYGASLHSFENTV*
>Syntrophomonas_wolfei_subsp.metylbutyratica_JCM14075T_02113_2-hydroxyglutaryl-CoA_dehydratase_D-component
VKVSFPHMGHLYIPLKTLFKGLKEVIAPPIISQKTMELGVKYSPEFACLPLKINMGNFIEALEQGADTIVMAGGWGPCRFGYYAQVERRILQELGYDFKMVVLEAPDSRISELLLQLKAIGAESVREAYKAIRFAWKKLVAIEKLEKTLEYCLPRAIDKNYMETVFERALQAIDEAESQEVEKIAQESLREMEKSPCKGAEVLKIGLVGEIYTILEPASNCHVARCLGRLDAQVSRSIYVSDWVNDHLF
GGYLRKSNKQIIQSSRPYLNYEVGGHGRETVGSTVDFARRDYNGGIIQAPLTCPMEIVAQSILEGKGKVSEELEIPCMTLYFDEHSGAAGVYTRLEAFVDMLQRKKNLSSSPLLLEARA*
>Syntrophomonas_wolfei_subsp.metylbutyratica_JCM14075T_02114_2-hydroxyglutaryl-CoA_dehydratase_component_A
LNLYLGVDIGSVSSNFILLDQNGRLYKRLYLRTLGNPVKALKDGYASLSDKQHGKIRAVGSTGSGRHLAAVLLGADVVKNEITAHAVAASHLFPGAKTIIEIGGQDSKIIILNNNEHVTDFAMNTVCAAGTGSFLDQQSARLNIPIQEFGDYAVKSGHPVRIAGRCGVFAESDMIHKQQMGFPVEDIIMGLCEALVRNYLNNVGKGKKIEEPVVFQGGVAANRGLAKAFEKILGKNVMIPAHYEVMG
AIGAAILAREQIELSHSPTRFRGDNFVLEGNFTSNSHECSGCPNLCEVVCIKRDNEVLAYWGDRCKKWESSKQPKREKLKVV*
>Syntrophomonas_wolfei_subsp.metylbutyratica_JCM14075T_02115_UDP-N-acetyl-D-glucosamine_6-dehydrogenase
MNKLIYGKNRFCVRQRRDVKQVIVMGLGYIGLPTAVILANAHVEVVGVDRIKLLQELEQGKIHIEEPNLNLLFQQVRSEKLRFSPQPENADIFIIAVPTPARADKSCDMTNVLQAVESILPQLQTGNLVIIESTIPPLSCEEEIIKPLLEEAGHKVGKGENLFLAFCPERVLPGDIVRELLVNNRVIGGFTRRCAQEAAKFYGSFLQGEISLSDLKTAEMTKLLENTFRDVNIALVNEMTRICSLGIDVLEVI
RLANKHPRVKLLQPGPGVGGHCLAVDPYFIVEKAPEKLPLIKTARRVNASMPAFVEHKAGQWLNQVRGDLSMGKVGGIHAHADVRESMFVAVMEKTIVCLEKGVEQLVKSGGVVDGGKSRKLLGLFAWSLKILGQHGEAVYLEEWKKLDMDFLKKVFAPEKMMFWNRGFDANRIHHANKLAALRA
>Syntrophomonas_wolfei_subsp.metylbutyratica_JCM14075T_02116_dTDP-glucose_4,6-dehydratase
MKILVTGGKGVVGSSLVRELRAQGEQVWACDLFHSHENNYIRTDISKFRQLQILFEQEEFDLVYHLAAEFGRWNGEDYYEQMWTNSVAGTKNLLRLQEQFGCRMIFSSSSEVYGDYDGIMAEEVMNEVAIKQMNDYAISKWANEMQILNSALMAGTQTVRVRLFNTYGPGEFYAPYRSALCVFIYRALHSLPYTVYRQHQRTSLYIDDCIRALASIPDNFQAGEVVNIAGIELHDMKSVSDMILD
YLGQDDSLVTYKDEENFTTRVKKPDISKAVKELNYQPKTLLEQGIAEWIRENYPTAK*
>Syntrophomonas_wolfei_subsp.metylbutyratica_JCM14075T_02117_Chondroitin_hypothetical_protein
LKALVSIIIPSLRADLLKWNLFSLAQQKIPYPFEIIVLNDGIPDDTERVCREYEQSLNLKYFFSGKRNLDNGNIKWRGQGFAMNIGARIAAGSILILCDAEMYHLNDTVFYLAEPLLQKPNLIAIPGLGKNDWSGEFRHIEERQGKYEPQALELCDDLRIRLVPESLRFLPMAVSRSEFYSIGGFDEDFVGWYEDDDLVGRLQMNACSYFETGAETVHLFHPRDKGKGVEERIALNEKLYFEKMSQIVRNQGREWGKLE*
>Syntrophomonas_wolfei_subsp.metylbutyratica_JCM14075T_02118_hypothetical_protein
VQMKLLVISGDFRQYLDESFHSLMTALADLLEYLVHHSGQKRLSLPLQLLFSPTDPVLNEPGEINSPVIDELSSLSIPYAVYLHDLHHDSCKEALQDKKLCWVFTRYRDRFDRWYPEWQNRMSWLPHHIDSNIFKDYHLPKTIDYLMMGALHEYIYSLRYKIREKMTEKPGFVYHEHPGYKGHFSPAEMKKLFVGPNYAREINRAKLIFSCDSVYKYPLLKYFEVLACNTLLLAPSSPELLDLGFVPGVHF
VEIDLNFEEKAKYLYEYSKKIARQEGYEMVRESKEYMLKKSRTRRAAQLLEMVEGILESYSSFPHSRPWLRT*
>Syntrophomonas_wolfei_subsp.metylbutyratica_JCM14075T_02120_Hydroxymethylpyrimidine/phosphomethylpyrimidine_kinase
LRKILTIAGSDCSAGAGIQADIKTIQALGAYALSVITAVTAQNTRGVTAVQEIEAQVIEKQLEAIFSDIRIDGIKIGMLGSAGTVQVVSKFLASFFQAGAFREKQLKNSLPLVVDPVMIAKSGDRLLEEEAVESLKKNLLPLASVITPNLMEAGALLGREVSSLEGMKEAGRELLEFGCEWAIVKGGHLEGEPVDVLANKDSLYWLRGRRVDTDCNHGTGCTFSAALATLMGQGWEVPAAKRAKAYIE
TCLEHGFAIGEGVGPHHFAAQRPWEPEALNDSGDASIKEEV*
>Syntrophomonas_wolfei_subsp.metylbutyratica_JCM14075T_02121_Hydroxyethylthiazole_kinase
MLKYCAIIWDEVRSKRPLVHCITNYVTVNDVANIILASGASPAMVEYPAEASGFAPLASALYFNLGTLTGEQEAAMLEASWAAASRGVPIILDPVACGVIGRKVDAAEIKSLIREKIKSCKIQVLKGNAAEIKSLAGLAGQAQGVDSLDTGEGLAEACLQLAAEDGTAAKQNPTQVYISGAVSALARRGNKIPVVKVDAIAMTLGRQRIEIGR
FVLRGEELVKEGRLE*
>Syntrophomonas_wolfei_subsp.metylbutyratica_JCM14075T_02122_Thiamine-phosphate_synthase
MINSDYSLYLVTDRGLLQGRSLLEEVRKAVKGGVSMVQLREKEAGSREFYELAQALQTELIQQLLPGKIRLDQGLLPLPLIFNDRLDIALAVDADGLHLGQEDLPLPVARSLLGREKIIGLSINKREEAWEGEKMGADYLGVSPVFATPTKADAPPPATGLEFLASLRQKEIPLVAIGGINKENLKMIKETGADGAAVISALMGASDIEGEARKLRQIWERS*
>Syntrophomonas_wolfei_subsp.metylbutyratica_JCM14075T_02123_2-hydroxy-3-keto-5-methylthiopentenyl-1-phosphate_phosphatase
MGEKLIIFMDFDGTISREDVCNKMAARYAGRDWEEINRLWEKGVITTGECARILSSMEVGAAELEAFFQAQEVDPGFSSFDWVRKKQHLPIIISDGYDRYISRLRGQGWEIFYANKLYWNDGWRMESPYLDEVCPQCGSCKKIIRRSLLPGYLTVYIGDGYSDRCPSEFAACCDIVFAKNELAGYCRKEGLTYYPYRDFHDILQLPGIIRNKV*
>Syntrophomonas_wolfei_subsp.metylbutyratica_JCM14075T_02124_Phosphomethylpyrimidine_synthase
MNYKTQKEAAKRGEITPEMKKVAEKEGVEIKYLQEGLAKGRIAIPANRNHLSLQAAIGAGLRTKVNVNIGISRDCSNMAEEMEKMQADAIQLMDLSCHGKTQEFRKALLSPAMIGTVPVYDAMGFLEKELSELTTVAEFLQVVETHAREGVDFVTIHAGITRRIRSTLENTRRAPLTSIVSRGGALLCTWMERNKAENPFYQYFDEVLDICREYDVALSLGDACRPGSINDASDACQLEELIT
LGDLTLRAWEKEVQVIIEGPGHMPLNEIEANVTLQKKLCHSAPFYVLGPLVSDIAPGYDHITSAIGGAIAAAAGADFLCYVTPAEHLRLPSLEDMAEGIIARIAAHAADIAKGIPGASEWDWQMSKARAELDWESMFKLAIDPEKARRYRAESSPELEDSCTMCGQSCALRNMKNL*
>Syntrophomonas_wolfei_subsp.metylbutyratica_JCM14075T_02125_Tryptophan_synthase_beta_chain
MSRDELTKILPDEKGFFGDYGGRFVPETLMSALEEILDAYQEAKKDPAFQAELEDLLRNYVGRPSPLYHAKRLSEHLGGAKIYLKREDLNHTGAHKINNALGQALLAKRMGKKRIIAETGAGQHGVATATVCALLGLECTIYMGEVDVARQALNVQRMRMLGAKVIPVTSGSRTLKDAVNEAMRDWVTNVSDTHYIIGSVVGPHPFPMMVRDFQRVIGEETKAQIMEQEGRLPDAVIACVGGGSNAMG
MEMVDDYPDIIIGCCGGGSNFAGIAFPFIPDKLAGKNRRIIAVEPTACPTLTQGVYAYDYGDVAGFTPLMKMYTLGHDFIPSGIHAGGLRYHGDSPLVSRLYHDKLIEAVAVKQNPTFEAAVLFAQTESIVPAPESAHAIRAAIDEAL KAREEGVSRTIVFNLSGHGHLDLFSYDKYLSGETEDVEFSDAELQESLRNLPQI*
>Syntrophomonas_wolfei_subsp.metylbutyratica_JCM14075T_02126_tRNA_(adenine(22)-N(1))-methyltransferase
MPNKRLLAISDRVIGKAAADLMGADHDLIAACPIFLEKDNVPRVIASEKGPFERLDLRLAVEKSPFGERMELRLLNNDLREIKGSNRLLNLGVEFLRGPEVGATVIIAGMGGDNIAEILSNKNKLSLSVEYIASLHLVLQPNNNYEELKQWLLENGFSIIKETAVDEGRRLRLLCYSNEQFRSQQGWPILLLLLNTITGTYYKRGVVSNISRLEQKLEEKLRRKRSLA
>Syntrophomonas_wolfei_subsp.metylbutyratica_JCM14075T_02127_metal-binding_protein
MQARVKDIISMEEHFPPWLAEAWDNSGLQLGSTNKAVKKVAVALDLDEISLNQALELQVAMIITHHPLIFKPLKQINYESPVGCLIQRIVKAGISVYAAHSNLDAAEKGLNQILAELLGLQDIDTLGNSQKEELYKLVVFVPVSHCEEVRQAINGAGAGFIGNYSDCSFRVRGTGTFRPGAGTQPFLGQEGELEEADEFRLETVVRKRELAQVLQAMQEAHPYEEAAFDIFRLEQAGKGYSMGRCG
LLPEETKLGDYARQVKETLGLEAVRVVGDLNKVIRKVAIVSGSGASLIPGISRQGVEVLVTGDLKYHEARDAEALGLSIIDAGHQGTEEIISAHLSRFLRQECQRGMKVEFIYSGCPFKYL*
>Syntrophomonas_wolfei_subsp.metylbutyratica_JCM14075T_02128_Cyclic_di-GMP_phosphodiesterase_response_regulator_RpfG
MLKEKNNIKDVNPALNNIRSEMLLVDDNSQNLLLTNNILMPIIGAVFSDAKLILESNLVETLFLGIPLELGDLPFQEIDKTVDFLREQAFDAIDLFRQMRLMDGKEYLFTHSINVSIICMLLGRKLKLCKDQNTIREIGLLHDLGKVFIENDILNKPGKLTEQEYEIKKHTIRGYNILFSGNPALNIEVANAALMHHERLDGSGYPSGLKGQEINFYAAVVAVADVDAITSAH
SYADKRSPYSAAEILWQESFGKLDARITKVFYDKITNFYVGNKVLLSNKKEGIVIYVDPSQPTRPIVMTDDEFYNLAIDRSITIQDIID*
>Syntrophomonas_wolfei_subsp.metylbutyratica_JCM14075T_02129_hypothetical_protein
VNKSELVKSLAEKAETTQKDAAKVLDALVDSIQQALAGGDKVQIIGFGSFEVRDRKERKVISPATGKEITVPATKVPAFKPGKSLKEAVAVKKKAAPAKAGGKKKTAKKK*
>Syntrophomonas_wolfei_subsp.metylbutyratica_JCM14075T_02132_hypothetical_protein
VLAIFGSAGAGHGIWANPLVVLAIVVSIYIFLKFCSWAKSKQLSAGVKKWFIITGLLVFVNVLYARGNAMVFNVIFFIAYALMTETKAE*
>Syntrophomonas_wolfei_subsp.metylbutyratica_JCM14075T_02133_Na(+)-translocating_NADH-quinone_reductase_subunit_F
MEFWTLTGKNLWDSIMTSGMAATGAGGGNGCGQCKVRLEGEINEISDESRQHLLPEELRTGTRLACFCRVKAPLTVYLDPLALAIQDQAWLKSDSYPASVAFSAPVKNRQILIPGMEKDSPVPLLTLRARALPGIKLELSDVNLNRLAQIDIRGIRPALELQALVFKEQAVKYIGRKKEKACGLALDIGSTSLFAAMLNLEDGDELAISTKPNMQRVYGADILSRVDYCLKENPDGLEELQQILLNNSSMI
EEMLEETFSPEHIYVLVLVGNPVMLHFLLGLNPAGFAQAPYAGFCPEFTYPAAALGLPVNPEAFILLPQVGGFVGADTVAAVLSLPDWRSSSFLLVDIGTNGEVVVGNRGKLWAASAAAGPAFEGGSISGMIAGDGAIDKICLNPEGGLEYNLIGDSPARGLCGSAIIDITACLQGAYIDGSGITELAREKLRVRQGERGAFIILVEENESFRGAPVVFNQEDIRQVLQAKAALRTAIDVLMEEA
GLEYKDLERVYLAGAFGNYLNPHNAVDIGMIPPDRKDKIITIRINGAVAGAYLALLSPDNKQRAEEIAKKIEYKSLVNEQIFVEVMHRGVLYFRLLFH*
>Syntrophomonas_wolfei_subsp.metylbutyratica_JCM14075T_02134_putative_enoyl-CoA_hydratase_echA8
MKNTIKNHIEKQQIIANWRAGGNEVEVIRLTKPDDDYNALVSLTLEHDNAYNAYLKQWFTVGVEAAFNLGDEYVGTSNCAERANFIQKMAAFFTPEVTREHIEGVEHFPGKVLPNVLAGGNEGNDGFHVFDAWQDFFIGAPLNTYDNNMKLLKAAMYLASRPVLDQLATNDAAEKIRQAIPNVLFRRGKERKSIPAENIF
KG*
>Syntrophomonas_wolfei_subsp.metylbutyratica_JCM14075T_02135_(S)-1-Phenylethanol_dehydrogenase
MKLKGKVAVITGGGTGIGRAAAIALAGEGARVAVMGRRPGPLNETVDIIREAGGEGLAIPSDVTVIEDIEKARDKVRETYGRLDILVNNAGSALFKPFMKTSLEDFDRIYQVDLRSVFAVSQQMLPLMLEGGEGSIINISSIGLRVGLPNMSAYCAMKGGVVQLTRALAEELGPEVRVNCLCPSHIIITPMMEDELSRIIETKGMRKLDLTKMFPMKRLAYPEDMTGSIFLYASDDSKWLTGNILMIDGGLS
CYI*
>Syntrophomonas_wolfei_subsp.metylbutyratica_JCM14075T_02136_hypothetical_protein
MNMNRQGDGSFACQRPLSREVTVLNYRVIVES*
>Syntrophomonas_wolfei_subsp.metylbutyratica_JCM14075T_02137_hypothetical_protein
MADIIPFRGLRYNPEKISNLASVVTPPYDIIDESAQARYYAENPANVIRLELGIFPQDSSSNNRYTRAAQYLEKWQEDQTLLAEEKAALYLYQQEFNFRGEKVVRTGFVCGLKLEPYEKGNILPHEETLSKPKADRLQLMRATNSNFSSIFGLYSDEQKEAEQLLAEVKEQAPDINIIDEAGETHKIWVVKNEELIERIVALLADRPVYIADGHHRYETALEYYHEMKEQGLQGYDYVMATLVNVFDE
LVVLPTHRLVGNIPGKFLDAFMNRLADLFEIELYKDKNIGAFMQEVKSEGKKHRVFGMYSKDESFLLKLNPEQASKLLPDNKSAAWEKLDVALLHHLLIDKVLGIAWQEGKEQLRYTRDGQEALAVKGIASKQASLLLIGCLNIHK*
>Syntrophomonas_wolfei_subsp.metylbutyratica_JCM14075T_02138_Histidinol-phosphatase
MLLDYHIHALAHGEYDYSEWLRLFAENARRRKLAEIGFSEHDEYLPRIKPELYRRLQEDFPELKIKLGLEVDYHPGREGEIQRMLVNSNFDYDYIIGSVHFIDGWGFDHPDFKDQFATRDIDDIYADYFTLLNQAVKSRLFDVIGHLDLIKIWGHRPKLPKKISAYVEPLLNTIKAAGMVVEINSSGLRKAVGEIYPARQILELLMEANIPITLGSDAHHPSQLGEEFAGLAQEVGYRQLVLFSGRK
QLVEI*
>Syntrophomonas_wolfei_subsp.metylbutyratica_JCM14075T_02139_putative_calcium/sodium:proton_antiporter
MSSIGILILSLGIILLAEFVNGVEWLGEKLNLSEGAVGSVLAVAGTALPETIIPIVAIVFGDRGGSIGVGAIIGAPLMLSTLAMFVTGLAVVFFSRRRKGKVQADYSSMTRDLSFFIIVFAAIITGTMPPAGNLIRLALAVVLLIIGFMVLSYLFYVVMNSEERLDDNISDKLSPNSCYFARKKSPRLSLVIGQVLFALLIIIGGADMFVGAVKEVAPMVQVPAFVLALIITPIATELPEKFNSVIWISREKDSLALGNITGAMVFQSSLIPALGIFLTDWQLSSGAFLSALLALAAGILYTELLRKKHITAGTLLLMGVFYGGIFLVGFVEGIIR*
>Syntrophomonas_wolfei_subsp.metylbutyratica_JCM14075T_02140_hypothetical_protein
MSNVNSYDKAHELARVITKSEPYLNYLKVEKKKPEWTKEVLNELVKEIMDEQAFRAEFAEMISLEFARINQIKEIAEFLRAETAFVQMFNDIQIINKAIKSGLEA*
>Syntrophomonas_wolfei_subsp.metylbutyratica_JCM14075T_02141_NADP-reducing_hydrogenase_subunit_HndA
VHFCSKNKRPEEKSMLDAQLDLSPVLIEIIEKHHEGSLIAILQQAQEVYGYLPKEVLSYIAGITGIPKAKVYGIATFYTQFRLQPTGKYQIMLCQGTACHVNGSERIISSTICQEIKLKRGETTPDGLFSLESAACLGCCSLAPVMMINGQAYGPLSAEKAVAIIRKIKAAESLSLVGEAQ*
>Syntrophomonas_wolfei_subsp.metylbutyratica_JCM14075T_02142_NADP-reducing_hydrogenase_subunit_HndC
MKVRLGLGSCGIAAGGLQVLEALQKEMESRGLKLEIQKSGCMGMCYREPLLDWIDSQGQVFTYGAVNAGRSVEILEAHLAGRGPLEAYLVSSSDEPAEFLQQRLRIVLKNCGRIDPESIDDYRRQDGYSALQKALAGMSPEEVIKEIEVSALRGRGGAGFPTGLKWKATREAAGFYRIICNADEGDPGAFMDRSILEGDPHAVLEGMIIAAYAIGAFQGIIYCRAEYPLAVKRLEIAIAQDRENGLLGR
RILGSDFSFDIHVRQGAGAFVCGEETALIASIEGERGMPRLKPPYPSESGLWGYPSSINNVETLANVPWIIRRGGEWFAGIGSSRSKGTKVFALAGKINRGGLVEVPMGTTLREIIYGIGGGVKAGRRIKAVQMGGPSGGCIPAELLDIPVDYDSLNTGAIMGSGGMVVMDESSCMVDLARFFLDFTQKESCGKCVQCRIGTKRMLEILERICRGEGEIGDLELLEELSAHISENSLCALGQSAPNPVLTT
IRYFRDEYEAHIREKRCPAKQCQELLKYTIDPDRCMGCGLCVYACPADCIKGGENNQPYYIEQENCSKCGACLDICPERFAAVRVPGT*
>Syntrophomonas_wolfei_subsp.metylbutyratica_JCM14075T_02143_Glutamate_synthase_[NADPH]_small_chain
MITLTINDREIKAAAGQTILQAARGNGIEIPTLCYDERVKPFASCGICVVEVEGSKKLLRSCATEVQEGMQVYTESPRVRQSRKLTLEMLLSNHPGDCLAPCRQACPADTDCQAYVGLIANGQFHEAVKLLKERIPLPASIGLICPHPCESACRRGLVEEPVSMAALKAFVGRYDLQRGQEYVPEIAESTGRKVAVIGAGPAGLTVAYFLARQGHQVTVFEAMPAAGGMLRYGIPDY
RLPKKLLEQEIDIARLGVKFFTNTRLGQDIFLEQLRENYDAVFLGIGAWVSSKIGCAGEETRGVTLGGIEFLRAVASREPVLMGERIAIIGGGNTAMDAARTAIRLGAAEVMVLYRRSRAEMPAEDAEIEEAVEEGIEFHFLAAPKEIIEDGRVAAIRMQKMQLGEADASGRRRPEPTGEEIEIAVDTVIAAIGQRQVDLGGLEKLELSRWNTLDIPASLPSFMSSMEGIFAGGDVTGPGIAIEAIAQGRKAAQA
MNAYLQGEKLPDNNSPYRNSRVREAEDLAGIKKEARIEPPTSSPERRLRSFAEVKAVFTPEEALREAGRCLECGCTAFYECKLLSAYGRYEVKPQRARGEIFSAVAGETHHHSYEYIFLDWDTDKCILCGLCVRICDEVMGVTALGLVNRGFGTVVMPEWEGNDLENSCLNCCGECAACVPTGALSQRKRCLY*
>Syntrophomonas_wolfei_subsp.metylbutyratica_JCM14075T_02144_Putative_peptidyl-prolyl_cis-trans_isomerase_Cbf2_precursor
MKKTKLAMLVILGLLLLLGCGNDKKASPIAEVINSEIITQEQYDKACQQNIAFEKQNFIQQGLDKADSSGKIDPEMEKRLQAFDSLVMQALLRQDAEKRGIKISKEEIDTISDFSKANLAAEKGDAYQKTLQENNLSEKEFRARMQLLVNQLRMKQKISFSIAEEGQYYQENIASYQEAAGMGIVMLIYETEDKAKEVQEQIEKGQGFAELAAKYSADGKQIGQGEIGNKSDIQMGYVYQKINEIQTKYIAYAAARIGMWQEGKKAQGFSIGG
ALKLKAGEITQEPVKSDFGYHIIKAGQLQPTTTKSFDEVKNQIQNQLLQDQLSEELTQEIGQ*
>Syntrophomonas_wolfei_subsp.metylbutyratica_JCM14075T_02145_Ferric_uptake_regulation_protein
VFGKSINNKLKDNDYRLTGQRTAILEVMMDNQGRHLSAEEVYDLVSKNNPEVGRATVYRTLDLLAHIDLITKIQLDDNKRYYIKDDHHHIHHHHIVCLACGKIAELEDLLCSLEGQLETQGFEVVDHELKFFVYCPVCREKKNK*
>Syntrophomonas_wolfei_subsp.metylbutyratica_JCM14075T_02146_Ferrous_iron_transport_protein_B
MAIVALLVNDVIRNGSTKTVAIVGQPNSGKSTLFNALTGARKRVGNWPGVTVEKKIGTIQIEGQKAIRLDVIPGTYSLSPHVGNKNSEQRLSIIEALLNAMEVLILNIVDASNLENTVLLTIKKAMNNWKAEEGRKKLALRLIDAVPLVMLLLNQIDRAKALSHLRLADVKLMVPLQEVLGKSLAMRTGDVLLEEQKLDFTNGYEKIIFGNALGLPPGIISQIQASEDFQCGTRNKREIRGKMLLGNE
TPVLLLLYAFVLLLKVRAASQSPALTFLTQPIQPTESFFLSGRLGGQAIMFTEYSQMGGVAPILSLLILPEVLLFVSVLVTLLLMSLFAVIPLILKFIINYSLCARYIKPQGAGVFRSLWANEIGEEMLYRVLPLLGGVLLFAPWFCLALVKQMAEFLTATGLSSIIVVVVCLPNGNTVRLQLVPFIMAAPIPLLAVIAWLAIILLIPLNNTLLLRAVLTFSMWLRLFVLSGQLRIAAKAGFLAAVIMLVLIPQLF
FIAVLVTIFQRYLAPLVLLDTPREATIAISMIALSLPCLPAMVMTVREIGWSLLFLLLGLGFLTSCSVGIMLNLL*
>Syntrophomonas_wolfei_subsp.metylbutyratica_JCM14075T_02147_FeoA_domain_protein
MEVTAMTINDMKPGQSGIIRSLRGLQGRRLFEMGLVPGARVKLVRSHPFKGPLVLQIANTQIALGRKMANSVEIQE*
>Syntrophomonas_wolfei_subsp.metylbutyratica_JCM14075T_02148_antiporter_inner_membrane_protein
MLIKFKKKKESKPKKEGAVTETPTATATTEDNSDAGPSAQIQGRNDIKRVIAVISGKGGVGKSTVSSLLASALLAHGYKVGLLDADITGPSIPRVFGVSGGMGKNDYGIIPRRSRKGLKIMSLNLFLADELPVIWRGPRIGGAVKEFYSQVDWGTLDFLILDMPPGTGDIAITVLQSIELDAAVVVSTPQDLALIMTAKAVKMHLMNNIPILGIVVENLTSGVCPHCQHEVELFSGGGIRQWCEEK

QVNYLGSIPWDAALSYCADRGQIDNYYPEAVGQMVEAFLAALPAIEDN*
>Syntrophomonas_wolfei_subsp.metylbutyratica_JCM14075T_02149_putative_ABC_transporter_ATP-binding_protein
[illegible]
>Syntrophomonas_wolfei_subsp.metylbutyratica_JCM14075T_02150_FeS_cluster_assembly_protein_SufB
[illegible]
MDKARIKSIPQLEAEDANAVLTHEAAIGKIAGEQLIKLMSLGLSEQEAIDTILEGFLR*
>Syntrophomonas_wolfei_subsp.metylbutyratica_JCM14075T_02151_CysJI_operon_transcriptional_activator
[illegible]
DPHRRIRSLKIDDPGATRFFYIIYPRQKSRRKAVESFLDFIKEE*
>Syntrophomonas_wolfei_subsp.metylbutyratica_JCM14075T_02152_pyruvoyl-dependent_arginine_decarboxylase
[illegible]
>Syntrophomonas_wolfei_subsp.metylbutyratica_JCM14075T_02153_Spermidine_synthase
[illegible]
CKYYTPEIHQAAFQLPAFVSRIIE*
>Syntrophomonas_wolfei_subsp.metylbutyratica_JCM14075T_02154_Agmatinase
[illegible]
DVVGFDLVEISPPCEQGDNTSILGAKILREALLAY*
>Syntrophomonas_wolfei_subsp.metylbutyratica_JCM14075T_02155_Rubrerythrin
[illegible]
>Syntrophomonas_wolfei_subsp.metylbutyratica_JCM14075T_02156_hypothetical_protein
[illegible]
>Syntrophomonas_wolfei_subsp.metylbutyratica_JCM14075T_02157_hypothetical_protein
[illegible]
>Syntrophomonas_wolfei_subsp.metylbutyratica_JCM14075T_02158_Tryptophan--tRNA_ligase_2
[illegible]
>Syntrophomonas_wolfei_subsp.metylbutyratica_JCM14075T_02159_hypothetical_protein
[illegible]
>Syntrophomonas_wolfei_subsp.metylbutyratica_JCM14075T_02160_S-adenosylmethionine_decarboxylase_proenzyme_precursor
[illegible]
>Syntrophomonas_wolfei_subsp.metylbutyratica_JCM14075T_02161_Acetyl-CoA_acetyltransferase
[illegible]
>Syntrophomonas_wolfei_subsp.metylbutyratica_JCM14075T_02162_hypothetical_protein
[illegible]
>Syntrophomonas_wolfei_subsp.metylbutyratica_JCM14075T_02163_3-octaprenyl-4-hydroxybenzoate_carboxy-lyase
[illegible]
>Syntrophomonas_wolfei_subsp.metylbutyratica_JCM14075T_02164_4-hydroxybenzoate_octaprenyltransferase
[illegible]
>Syntrophomonas_wolfei_subsp.metylbutyratica_JCM14075T_02165_putative_aromatic_acid_decarboxylase
[illegible]
>Syntrophomonas_wolfei_subsp.metylbutyratica_JCM14075T_02166_Heptaprenyl_diphosphate_synthase_component_2
[illegible]
>Syntrophomonas_wolfei_subsp.metylbutyratica_JCM14075T_02167_Sec-independent_protein_translocase_protein_TatCd
[illegible]
>Syntrophomonas_wolfei_subsp.metylbutyratica_JCM14075T_02168_Sec-independent_protein_translocase_protein_TatAy
[illegible]
>Syntrophomonas_wolfei_subsp.metylbutyratica_JCM14075T_02169_Siroheme_synthase
[illegible]
>Syntrophomonas_wolfei_subsp.metylbutyratica_JCM14075T_02170_Glutamyl-tRNA_reductase
[illegible]
>Syntrophomonas_wolfei_subsp.metylbutyratica_JCM14075T_02171_Porphobilinogen_deaminase
[illegible]
>Syntrophomonas_wolfei_subsp.metylbutyratica_JCM14075T_02172_Uroporphyrinogen-III_C-methyltransferase
[illegible]
>Syntrophomonas_wolfei_subsp.metylbutyratica_JCM14075T_02173_Antilisterial_bacteriocin_subtilosin_biosynthesis_protein_AlbA
[illegible]
>Syntrophomonas_wolfei_subsp.metylbutyratica_JCM14075T_02174_Delta-aminolevulinic_acid_dehydratase
[illegible]
>Syntrophomonas_wolfei_subsp.metylbutyratica_JCM14075T_02175_Antilisterial_bacteriocin_subtilosin_biosynthesis_protein_AlbA
[illegible]
>Syntrophomonas_wolfei_subsp.metylbutyratica_JCM14075T_02176_hypothetical_protein
[illegible]
>Syntrophomonas_wolfei_subsp.metylbutyratica_JCM14075T_02177_hypothetical_protein
[illegible]
>Syntrophomonas_wolfei_subsp.metylbutyratica_JCM14075T_02178_Glutamate-1-semialdehyde_2,1-aminomutase_1
[illegible]
>Syntrophomonas_wolfei_subsp.metylbutyratica_JCM14075T_02179_Chemotaxis_protein_methyltransferase
[illegible]
>Syntrophomonas_wolfei_subsp.metylbutyratica_JCM14075T_02180_Electron_transfer_flavoprotein_small_subunit
[illegible]
>Syntrophomonas_wolfei_subsp.metylbutyratica_JCM14075T_02181_Electron_transfer_flavoprotein_large_subunit
[illegible]
>Syntrophomonas_wolfei_subsp.metylbutyratica_JCM14075T_02182_Anaerobic_glycerol-3-phosphate_dehydrogenase_subunit_C
[illegible]
>Syntrophomonas_wolfei_subsp.metylbutyratica_JCM14075T_02183_Phosphopantetheine_adenylyltransferase
[illegible]
>Syntrophomonas_wolfei_subsp.metylbutyratica_JCM14075T_02184_hypothetical_protein
[illegible]
>Syntrophomonas_wolfei_subsp.metylbutyratica_JCM14075T_02185_ABC-2_family_transporter_protein
[illegible]
>Syntrophomonas_wolfei_subsp.metylbutyratica_JCM14075T_02186_SkfA_peptide_export_ATP-binding_protein_SkfE
[illegible]
>Syntrophomonas_wolfei_subsp.metylbutyratica_JCM14075T_02187_HTH-type_transcriptional_repressor_YtrA
[illegible]
>Syntrophomonas_wolfei_subsp.metylbutyratica_JCM14075T_02188_hypothetical_protein
[illegible]
>Syntrophomonas_wolfei_subsp.metylbutyratica_JCM14075T_02189_Inner_membrane_protein_YhhQ
[illegible]
>Syntrophomonas_wolfei_subsp.metylbutyratica_JCM14075T_02190_hypothetical_protein
[illegible]
>Syntrophomonas_wolfei_subsp.metylbutyratica_JCM14075T_02191_hypothetical_protein
[illegible]
>Syntrophomonas_wolfei_subsp.metylbutyratica_JCM14075T_02192_NADH-dependent_butanol_dehydrogenase_A
[illegible]
>Syntrophomonas_wolfei_subsp.metylbutyratica_JCM14075T_02193_L(+)-tartrate_dehydratase_subunit_beta
[illegible]
>Syntrophomonas_wolfei_subsp.metylbutyratica_JCM14075T_02194_L(+)-tartrate_dehydratase_subunit_alpha
[illegible]
>Syntrophomonas_wolfei_subsp.metylbutyratica_JCM14075T_02195_SH3_domain_protein
[illegible]
>Syntrophomonas_wolfei_subsp.metylbutyratica_JCM14075T_02196_Methionine_import_ATP-binding_protein_MetN
[illegible]
>Syntrophomonas_wolfei_subsp.metylbutyratica_JCM14075T_02197_Sulfate_transport_system_permease_protein_CysW
[illegible]
>Syntrophomonas_wolfei_subsp.metylbutyratica_JCM14075T_02199_hypothetical_protein
[illegible]
>Syntrophomonas_wolfei_subsp.metylbutyratica_JCM14075T_02200_Cell_division_protein_FtsW
[illegible]
>Syntrophomonas_wolfei_subsp.metylbutyratica_JCM14075T_02201_Cell_division_topological_specificity_factor_MinE
[illegible]
>Syntrophomonas_wolfei_subsp.metylbutyratica_JCM14075T_02202_Cell_division_inhibitor_MinD
[illegible]
>Syntrophomonas_wolfei_subsp.metylbutyratica_JCM14075T_02203_Septum_site-determining_protein_MinC
[illegible]
>Syntrophomonas_wolfei_subsp.metylbutyratica_JCM14075T_02204_Sporulation-specific_penicillin-binding_protein
[illegible]
>Syntrophomonas_wolfei_subsp.metylbutyratica_JCM14075T_02205_rod_shape-determining_protein_MreD
[illegible]
>Syntrophomonas_wolfei_subsp.metylbutyratica_JCM14075T_02206_Rod_shape-determining_protein_MreC
[illegible]
>Syntrophomonas_wolfei_subsp.metylbutyratica_JCM14075T_02207_Rod_shape-determining_protein_MreB
[illegible]
>Syntrophomonas_wolfei_subsp.metylbutyratica_JCM14075T_02208_DNA_repair_protein_RadC
[illegible]
>Syntrophomonas_wolfei_subsp.metylbutyratica_JCM14075T_02209_Septum_formation_protein_Maf
[illegible]
>Syntrophomonas_wolfei_subsp.metylbutyratica_JCM14075T_02210_hypothetical_protein
[illegible]
>Syntrophomonas_wolfei_subsp.metylbutyratica_JCM14075T_02211_Redox-sensing_transcriptional_repressor_rex
[illegible]
>Syntrophomonas_wolfei_subsp.metylbutyratica_JCM14075T_02212_Low-affinity_inorganic_phosphate_transporter_1
[illegible]
>Syntrophomonas_wolfei_subsp.metylbutyratica_JCM14075T_02213_Putative_pit_accessory_protein
[illegible]
>Syntrophomonas_wolfei_subsp.metylbutyratica_JCM14075T_02214_Folylpolyglutamate_synthase
[illegible]
>Syntrophomonas_wolfei_subsp.metylbutyratica_JCM14075T_02215_Valine--tRNA_ligase
[illegible]
>Syntrophomonas_wolfei_subsp.metylbutyratica_JCM14075T_02216_phosphatidylserine_decarboxylase
[illegible]
>Syntrophomonas_wolfei_subsp.metylbutyratica_JCM14075T_02217_SWIM/SEC-C_metal-binding_motif_protein_family
[illegible]
>Syntrophomonas_wolfei_subsp.metylbutyratica_JCM14075T_02218_Type_I_restriction_modification_DNA_specificity_domain_protein
[illegible]
>Syntrophomonas_wolfei_subsp.metylbutyratica_JCM14075T_02219_Restriction_enzyme_BgcI_subunit_alpha
[illegible]
>Syntrophomonas_wolfei_subsp.metylbutyratica_JCM14075T_02220_putative_transcriptional_regulator
[illegible]
>Syntrophomonas_wolfei_subsp.metylbutyratica_JCM14075T_02221_hypothetical_protein
[illegible]
>Syntrophomonas_wolfei_subsp.metylbutyratica_JCM14075T_02222_hypothetical_protein
[illegible]
>Syntrophomonas_wolfei_subsp.metylbutyratica_JCM14075T_02223_DNA_binding_domain,_excisionase_family
[illegible]
>Syntrophomonas_wolfei_subsp.metylbutyratica_JCM14075T_02224_hypothetical_protein
[illegible]

>Syntrophomonas_wolfei_subsp.metylbutyratica_JCM14075T_02225_hypothetical_protein
MARNYPLTKMNKEGTLLRPQHSYFSNEYTETMCDLFLRDCVLSDEHGKLHKYYRLHAKQDHNMEMAFAYDIHCPECQNGMLKQISRPLSYNELGLYRCPVCDKK*
>Syntrophomonas_wolfei_subsp.metylbutyratica_JCM14075T_02226_phage_major_capsid_protein,_HK97_family
MNTYTERLLYTGSPEYDKHFWNAMRGKDSSYDELSKGRSTGTGLYAMPNSSDNKYMAALEKASLFRNIGTVIKAYGSGYRIFAKDSNDLAEWVAENEAIPIYEGIEDFTINTVDSWKLAALVKMDEAFIHDASFDIENYLIKRFAKNFGRAEDNAFINGNGTEMPTGILNATGGAEVAATIDEIIYDDVIGLYFSVKPEYRKNAVWLMNDVTALTLRTLKDSAGNYLWRDSDDTILGKKVIMTEFMP
DAESGSKPIAFGDFSYYWVIGRRPIGVRTLTEKFAALDQLGYLAYEFLDGKLIRPESVKVIQISEIIG*
>Syntrophomonas_wolfei_subsp.metylbutyratica_JCM14075T_02227_hypothetical_protein
MDEKTISITEKAIGDTLYIIESAVSSAAKETAYDKLKRMILSDVKSLEMKAS*
>Syntrophomonas_wolfei_subsp.metylbutyratica_JCM14075T_02228_hypothetical_protein
MNNRQSNNTVKDNKITALYCRLSRDDESQGDSNSIKNQKTILQKYADDNGFTNTEFFVDDGYSGTNFDRPDWQRLLSQVEEGNIGTIIVKDMSRLGRDYLKVGYYTEVLFPGSDIRFIAINNNVDSSNQQDSDFTPFLNIINEWYAKDTSKKIRSVFKSKGQSGKPLCTNPPYGYIKDPEDKLHWIVDEDAAKVVKEAFRLCMQGYGPTQIAKEFSERHIMNPTAHAKASGINIPDNRGHDDDYI
WRGSTIVHMLSRQEYLGHTVNFKTYRKSYKQKKQMKNDPSEWQIFENTHEAIIEEPVFEVVQKIREGRRRITPMGEMPILSGMLFCADCGAKLYQVRHRGWEHDKEHFVCATYRKIKGGCSSHQIRNVVVDELLLDGIRRVTAFAREQEDEFVEMVTKKTRIDLDRSIRDGKRELEQSQTRINKLDDIIQRLYEDNIEGKISDERFAKMTANYEAEQHTLKSRVAELKSAMTSDKESALGVDHFLAL
VRKYTDIKELTAEIIREFVDKIEVYKAARIDGRRVQRIKIVYNCIGEFDPSVSTSTTEQEKSA*
>Syntrophomonas_wolfei_subsp.metylbutyratica_JCM14075T_02229_putative_GTP-binding_protein_EngB
MLNGGRKAEGGGRKPQNEATHGDNTKGWQKDCPLAPLAGYKIKKAELEGIYVDINQLPAGNIPEIALAGRSNVGKSSLINKICNRKNLAKSSSTPGKTRTINYYLINDQWFMVDLPGYGYARVSQKEKARWKKMVEAYIGKREQLRGVIQLLDIRHEPGENDILMKDWLLHLQIPLLIVATKADKLSRGARMKQIAIIRKTLDLPEMPLVFSAQSGDGVEELTAALAELL*
>Syntrophomonas_wolfei_subsp.metylbutyratica_JCM14075T_02230_Lon_protease_1
MPTEEHQTYRELPMLPLRGVLVFPYTVIHLDVGRKKSINAIEDAMLGSKEIFLATQKEAQTDEPDEEDIYEVGTVAEIRQILKMPGGTMRVLVEGLFRAEINAYLTNEPYMKVRVEELKDKKLKSPELEALMRNLVGQFEQYVRMSKKIPPETVVSVVAIEEGGRLADVIASHLNLRINEKQRILELSDVNKRLNYLCELLAKEMEVLELERKINIRVRKQMEKTQKEYYLREQIKAIQKELGEKDERSSE
VEEFRERIKKANMPKDAEEKAFKELERLEKMPPMVAEAVVVRNYLDWILSLPWSFETRDRLDLKAAEAILEEDHYGLEKPKERILEYLAIRKLAKKMKGPILCLVGPPGVGKTSLGKSVARSLGRKFIRMSLGGIRDEAEIRGHRRTYVGSMPGRILQGMKTAGSKNPVFLLDEIDKMTMDFRGDPASALLEVLDPEQNYIFSDHYLEIPFDLSKVMFITTANSVFNIPRPLLDRMEIIEITGYTEEDKVHI
ATDYLVPKQIKEHGLKESNISFSEGTLRRIIREYTREAGVRSLERQIASICRKVARQVVEDKDTFVHVASNSLNRFLGAGRYRYGLAESENQVGVATGLAWTESGGDILSIEVALLKGKGNLTLTGKLGEVMKESAQAALTYVRSKADELGISDEVRDKYDVHIHIPEGAIPKDGPSAGITLATALASAMSGLPVRSDVAMTGEITLRGRILPIGGLKEKILAAHRAGIAKVLLPVENKKDLAEIPAPVKRKIK
LVLVSHMDEVLEETLLRLEPIQPGEDFPGVLLNPELVGENLNQDRVEPEANKDLC*
>Syntrophomonas_wolfei_subsp.metylbutyratica_JCM14075T_02231_Lon_protease_2
MNEFFAQYSGILYLIQLFFAIVIGLYFWNLLKNQQSHKTVMVKESEKESSRLRQMRNIKLSEPLSSLTRPAQMDDIIGQKEAVSILRSALCGPNPQHVIIFGPPGVGKTAAARLVLEEAKKNPLSPFGKDANFVEVDATISRFDERNIADPLIGSVHDPIYQGAGALGSAGIPQPKPGACTRAHGGILFIDEIGELHPLQMNKLLKVLEDRKVMLESAYYTECNRTIPVYIHEIFNHGLPADFRLVGATTRSP
DELSPALRSRCLEIFFNPLAKEDIERITLNAAQKINFPLETAALEEISRYAGNGREAVNMVQLSAGIAQVEKESKISLSIVEKVLNNGRFSPRREIKIKEEAMVGVVNGLAVFGANMGTLLEIEAAALPVAKGQGNWTVTGVVDEEQVGGQERQFRRKSMARGAVENVLTVLRSIYDLEIGDYNIHLNFPGGIPVDGPSAGISMATAIYSSITGKVVDNLLAMTGEISVYGKVKPVGGVIAKIEAARRAGVQ
KVIIPAENWLNIFDDFPDLKVIPVRDLDEVFTWAFLEQEVFPATTDKPLPTINEPGLGVST*
>Syntrophomonas_wolfei_subsp.metylbutyratica_JCM14075T_02232_ATP-dependent_Clp_protease_ATP-binding_subunit_ClpX
MHRYGDEKGQLKCSFCGKTQDQVKKLVAGPGVYICDECIELCNEIIEEELADDLGFELGDIPKPQDIREILDDYVIGQDRAKKSLAVAVYNHYKRINLGMKLDDVELQKSNIMMLGPTGSGKTLLAQTLARILNVPFAIADATSLTEAGYVGEDVENILLKLIQSADYDIEKAEKGIIYIDEIDKIARKTDNPSITRDVSGEGVQQALLKILEGTVASVPPQGGRKHPHQEFIQIDTSNILFICGGAFEGID
KIIENRIGQKVMGFGADIKSREEKKIGEILNMILPQDLLKYGLIPEFVGRVPIIVTLEALDIDALISILTEPKNALVKQYKKLFELDGVDLEFKEDALQAIADEALRRNTGARGLRAIIEDIMLEVMYEIPSRLDVTKVAITRDVIEKKEQPLLVTMEARRKVN*
>Syntrophomonas_wolfei_subsp.metylbutyratica_JCM14075T_02233_ATP-dependent_Clp_protease_proteolytic_subunit
MSYLVPMVVEQTNRGERSYDIYSRLLKDRIIFLGSGIDDTVANLVIAQLLFLEAEDPDKDISLYINSPGGSITAGMAIYDTLQYIRPDVSTICVGLAASMGAFLLAAGKKGKRYALPNAEIMIHQPAGGTQGQATDIEIHARRIMNMKESLNKILAERTGQPLERIQKDTDRDYFMTAQEAKEYGIIDEVISHPKKTSKK*
>Syntrophomonas_wolfei_subsp.metylbutyratica_JCM14075T_02234_Trigger_factor
MTAKLEKIENSEAYIEIEVSAEQVEEGLQYAYRKVVKQVAIPGFRKGKAPRELLELQFGKEVLFQDALEYIVPEAYEKALEELNIKPIAQPEFDINDPESGQPFKFNARVPVKPEVKLGEIEGIEVEIPDFQVKEEDVIQRLEDMRRHYAQVAEKIEEPAAMGDKLNIDFEGFIDGEAFAGGRGEDYSLELGSNTFIPGFEEQLVGLKAGESKDVLVTFPESYHAEDLAGKEAVFQVSVKRIETTEARELNDEF
AQEVSQFNTIDELRQDIRKNLEEMAESRRKESIKTELMEKALENCDIPVPDAVINMQVERMLQDFEQRMAYQGLTLEQYFQFTNSNREDFSQKIWPEAEKSVKGDFMLEKLAEEKGMEVSEEELNEHIMKLADSFGMEVDKIKEELGDAIENIRTGLKIDKAIDFLIDKAVIKEAAEIATAAE*
>Syntrophomonas_wolfei_subsp.metylbutyratica_JCM14075T_02235_Putative_peptidoglycan_binding_domain_protein
MAIKRNLILLITSALLLGVLGGCGGQVAQYPQRSPDLPPPADKQMSALDEVITDPNIKKDTNLAHDENIQELKHLKYGVRPGEGSELSSPDSKDSSEPGAAPLRKPRASYREEPYTRVNIQLLTVEELDAVVEELIKQDYLQQKPATEEELGNALSRFQSDHNLTPTGQVDAATLELIKGK*
>Syntrophomonas_wolfei_subsp.metylbutyratica_JCM14075T_02236_FtsH_protease_regulator_HflK
MANNGDYREPDWTKNRGDRLNAPAILLFIIGAFSIYAYIIMQQIWLTVILGIAAILAAWSLKVAREWDRVVVLRLGKFRRMAGPGLFFIIPIIDEAPIWIDMRIRTTFFAAEKTLTKDNVPVNVDAVMFWVVDDPMKAALEVEEYQKAVFWAAQTTLRDMIGKTELYAMLAGREHIDEELKVMIDARTHSWGVSVRSVEIRDVMIPDELQDAMSREAQAERERRARVILGTAELEIADKFAQAATRYH
NNPEAFSLRAMNILYEGIKEKASLVIVPSNMVHSLNPGSILGYVSKMEDKENGD*
>Syntrophomonas_wolfei_subsp.metylbutyratica_JCM14075T_02237_tol-pal_system_protein_YbgF
MKANEYEGNFFHDVLYLKKGMVNMKESMETNINENTDSSITSLSEQAAEKILKHDITGAYEILVEAIKADVENIEILNLVARCYFLWGDFERAELCWKKVLELDTANPEASLSLEDLQDPPFQFWLKRYYEALNQVENRNYEAARNSLWELLQEKDCFVGIYQLLGLCYLAEADRYSARRVWSKGLGWDSSNQPLLNYLNIYEEESSQIVELEEASEKWIHWPALPRIRLAWLLSGVLCTVLLVQGVSY
LVGQRDNDEKQSSKNKQEVEYFQQKIEKGNKAQMVLSEKPAVRFLPEEINSRGEENSMAGADYDLEQEKWYYSEGYQAYLAGDFKKSASNLGMVVSMQSKTYLHREALYYLARVYYINSDFKNAEKYFLDYTRDFPSTNYYDESLFYLACIYYFDQQEDKAREKLEKLCEAVPDSGYLTSKMALTLLGAQ*
>Syntrophomonas_wolfei_subsp.metylbutyratica_JCM14075T_02238_Putative_L,D-transpeptidase_YkuD
MVYASRFLRLEKIRMSGPDIHLMQKRLKELGYNPGAEDGIFGEQSEAALKAFQASRGLVVDGIVDPNTWLQLEAGSSIRLQSKREGQTWEQPRISIDVVKRKLTYTSGSFKKTYPVAVGKPQTPTPLGNWTIVQKTVNPGGAFGARWMRLSVPWGGYGIHGTNRPSSIGKAASHGCVRMYNADVIELYDRTPLGTPVNIFGKANTGRVLSRGDEGSDVKEVQRILRTLGYYRAKPDGKFGPKTE
AAVTAFQRDNQLIADGIVGPYTHHALQKAYDLAINDVEP*
>Syntrophomonas_wolfei_subsp.metylbutyratica_JCM14075T_02240_Adenosine_monophosphate-protein_transferase_SoFic
LREFDYTAVPNELMNHELMNLVSAIHEYKGKQELFIEAKPDVLEAMLEIAIIQSTGASNRIEGIYTSDDRLDALVKFKAEPRNRSEREIAGYREVLSTIHESYDYVVPRTNVILHLHRDLYQFSPVSSGGKFKNSDNIISEIDSSGRSKVRFQPLSAFETPEAVNRLTSTFIEAINAEKYDPLLLIPMFILDFLCIHPFNDGNGRMSRLLTLLLLYRSGYVVGKYISIEMIVEKTKETYYEVLLESSSGWYEGGNDYF
PFVKYCLEVILSAYKEFSSRVELMQNRSLSKPERIRVLFDNTLQKLSKRMILEKCPDISTSTVEITLASLLREGYILKTGAGKNTAYIRNMEQPDKDK*
>Syntrophomonas_wolfei_subsp.metylbutyratica_JCM14075T_02241_Mg-protoporphyrin_IX_methyl_transferase
MDEQSIINWDALWKAAQRNYRNIRPNQWDSMAPALKRWMSNDEYFRQFMANIRLCPEWTVLDVGCGPGDITIWAAKRSKHVTALDFSSEMLRLLKETATKECLTNIDCIQCSWEDDRLDSELDKHDVVIASRSLATMANLKEGLARIDDKTKRYAYVTMGAGFSSPLKKMVYKVMGKEISDPPGYIYAYNLLYQMGIRANVKFIEGRSRYIDLNDALEQCRWIAEDLRPSEEKEIAGLLNDCLIK
RQDGILEFPYNDICWALMWWEKF*
>Syntrophomonas_wolfei_subsp.metylbutyratica_JCM14075T_02242_corrinoid_ABC_transporter_substrate-binding_protein
MRKSMALLIMFFLTTVFCTGCGQQSATPAPEAVPAEKTITDMAGNTVVVPANPERIAFHWANQYEIMMILGAADDIVAIHPNVKKFVWLVKYKPEVLNLPTPFADPVNFEELLKTDPDLVFEFEHCTDTIEKCKEIGLTVAVLSKAKSIKNIRENIMFIGQTLGNEHYEKAKKYDTYFSEKLKMVNSIIAGIPDGQKPTVACIITDASLRVGGTDTIMDEWIDIAGGKNIAHEIPGYKVVDNEQLLKW
NPDVIITPSNRAKNLIVNSPVFKELTAVKNDRVYVNPHGFFDWQYPSAEEALQIQWAAKTLHPDLFPNIDMRKEVKYYHQQFLGFSLSEEEIDTILFPEIYEAT*
>Syntrophomonas_wolfei_subsp.metylbutyratica_JCM14075T_02243_biotin_synthase
VARFERILEKSKNEAINEEEAWYIFKETEQEEKATELLQTARFVREKVMGNTFKWSGGIARVLRCNLKPLCQYCPYWRKKGDEPLGIEEILKGVDYIARHGLQEFHLSGGTTLRSDGSDVLEIVTAIRAAGYNDMELDINCGAAMSFDTLKELKQLGVKLVRSVFETINPQVFNKVKPGDDLEEKKKFARLIGEAGLGLGTGIMAGLSPNETKYQDYVDFIFHIKHYEHLKSVYVSKFFPFNGIPMKDH
PSCSDWEAARLIAVMRLVLRDKDIGWAAGWGRDNYPAPLMAGAGNRVGGIHINRTPHYHVYQNEEYLYEDNMEFHNHMESTSKKLKELGIEITL*
>Syntrophomonas_wolfei_subsp.metylbutyratica_JCM14075T_02244_corrinoid_ABC_transporter_substrate-binding_protein
MFSQNKNTFKLIALGLIMVLTLALVGGCSKAAGDKSEQAMQQKNEPPAQVNIQDMAGRTVAIPGEVKSILALHPIPTYMLWQLAPDKMVSKDKVFDGRYLAAESVKAYPDTEIDKLKKLPVTGVFFKGTSPEQILQLNPDVIITLTKDPQIDKLAGQVNLPVIAVSKDTLADYEESMRLLGKIVGNEAQANKLADWWRDNIRTMSQETGKIPQDEKPRVYFTGASDILTTPGESTIMASILKLAGG
NNVATELSGKPTDESIPLSMEQILKWDPQVIITWKQTTKDQIMNGAEWKNVSAVKNGRVYVQPRYANMDGITALVGMIWAHGKLYHDNDANYDKLFEDKMIEFYSLFHNNLIKPEQIVEVAE*
>Syntrophomonas_wolfei_subsp.metylbutyratica_JCM14075T_02245_Staphylococcal_iron-regulated_protein_F
MKKKRFGLLLVMVFFLALSVMFIAGCGKEKAADMPDATRKIVDMAGREVTLPLEINRIVTLGPVPVINSLIFTVGEGDKIVNGLPEFARNERYKYQHKFAPNITNQPMLQMSTGKPNVEELLKLKPDVVFTMSTKKAWDEQTAKVLTEAGLSTVYLVWTNPEEVKQAINLVGQALNQEKCAREYNKYFDDTVVRINDVVKSIPRNEQPRVLHCNTQNMTAPHAISDWWIKQAGGISVTEDFRK
ETHVAENIELSVEQLLKWNPQVIIVGSPEQVDSLFNDTRFKDISAVKNRRVFASPMGAQEWCYRTSEQPLMLLWAAKTFHPEVFKDLNLEEELIQFYKHFFNYSMSVEEAQEILGGGPKVDTGKKE*
>Syntrophomonas_wolfei_subsp.metylbutyratica_JCM14075T_02246_Ferric-citrate-binding_protein
MRKSIALFLSMLLLITVFCVGCNKQSEKTEPDIAMNKVTDESFKRTITDANDQVVEIPQNVRKIAITCHGGATHELVVLGAEDMIVAQPTMERFPLLMKIKPQFNGVVDAGSFDNVNVEELLKLEPDVVFAFCGSEKGNTKIKEVGIPVVTMLCGKANIQALKNEFLTTGIILDKNDEAKKLVAYWDEKLGLINNRLSRISEEQRKKVYYAGGQLLKTEGGQWWINELVTNVGGVLVSKDMVSNG
VSMECSIEQLIGWDPDVIVCSRETGKSGLAEEIKKDPRLKDLTAIKNNAVYDYPLGAFWWNRPSPEAPLGFIWLAKTLYPEETRDIDLKAETKKFYKEFYKYELSDKEYESFF*
>Syntrophomonas_wolfei_subsp.metylbutyratica_JCM14075T_02247_Hemin_import_ATP-binding_protein_HmuV
VGMGNKEWLTAQEPRILIMDEPTASLDFGNQIKVLSHVKQLAGLNIAVLMSCHFPEHAFIYSNRAVLLNEGRILHSGTPGETITAHRLKTLYGVDVDIVTVKNRMNQELQVCIPSRSLAEPDKSLELIESRKEYALA*
>Syntrophomonas_wolfei_subsp.metylbutyratica_JCM14075T_02248_putative_ABC_transporter_permease_protein
VEAVTKHKIVEMETKRGSGWKITILLITLALIFILSFALGRYPVPPGQLLEVLASRVFPIEAHWNATVETVIFQVRLPRIFAAILVGAALSTAGAAYQGMFKNPLVSPDILGASAGAGFGAALAIYFSFSMMGIQMMAFVGGLLAVGLTYGISLRIRHDPMLALVLSGVMIGSLFTAAISCIKYIADPYDKLPAITFWLMGSLASITPRDVYMVLTPILLGGILLYLLRWRLNVLAMGEEEARVLGINTTLMKT
VVIVCCTLMTAASVSISGLIGWVGLVIPHLARMIVGPNYRVLLPASILLGGAYLLLVDDLARLLASMEIPLGILTALIGVPFFLYLLLNTKWGWK*
>Syntrophomonas_wolfei_subsp.metylbutyratica_JCM14075T_02249_biotin_synthase
LFTTLLHTVLNEGINKRLALKILEESRWPQNALELFAVASRLRDENLGKELYFSAGFGGVLPCRIVPRCAYCTYFVAEPFPLPDLLACVRKVEELGIRQMHLSGGSNLEGYDSEILEMVQAIQETSDIQVEVNLGPSLSADTVKALKTMKVVSITSSLETFTPELFNRTKPGDSLEKRKQLLEICEQEGVPIRSMMMIGLGEKYADRIEQLFYLSRFSHLYHLRFSRFYPYPRTALHNQLRCSPWELARTLAV
ARLIMPRINLGLAAGNEPDDLPLWYAAGGGNQLLGVMVSNRKVENKTNPEDELIIINERCSVYNRMPLMQRYLQEMGASVISKEKIQ*
>Syntrophomonas_wolfei_subsp.metylbutyratica_JCM14075T_02250_pyrroloquinoline_quinone_biosynthesis_protein_PqqE
MIRKHFKGYNFVCIPETGVTFRWGDSIAEDPYMAPWPELADISISNYCTNGCEYCYRASNEEGIFMTVADYKFILGQLTSKEYGSIFQVALGGGEPLLHPDFNEILHVTREEYGIIPNYTTCGKYFNQKNMEASKKYCGAVAVSWDPYRDNLSLEELRELGVQLKEEKIRTNIHYVISERTLIEAIKLLKGKYDEYLKDFNSVIFLTYKAAGRADKSDVIKSQKNLREFLDMVDAPLTKLKLGFDACFVPVL
MKATGVDTDLVDSCECGFFSVYVSESMEVMPCSFCNNSNFTYNLKDLCFKDIWQKHFSAYRSYVNDNCKTGCAECDERSGCRGKCPFYNDIYLCNLV*
>Syntrophomonas_wolfei_subsp.metylbutyratica_JCM14075T_02251_hypothetical_protein
VKIRCDFVTNSSSASFVLTVKEEIIDAKIKRFTEIGKTGWVQLLDFLKKKMGEEGYKTIIGSQEYSFTIKEFPLGKSQFLADDSDPEEISKSDFSELSDDEMWAVINWVVVKGESNDILGVGATQTTKPQCERGGCKV*
>Syntrophomonas_wolfei_subsp.metylbutyratica_JCM14075T_02252_corrinoid_ABC_transporter_substrate-binding_protein
MKKRGLVILAMLALLIISGMLIAGCGKEKAADTTATPRTIVDMAGREVTLPAEIHSLATIGPGPVLNSLIFAVGEGGKIVNGLPDFARSERWKYQHKFAPNISEQPMVQTSDFQPNVEEMLKLKPDVVFTMEARGSCEQIAEVLTNAGLPTVCLVWTEPDEVKQAINMIGEVLHQETRAQEYSKYFDDTIARVSKVVDSIPEDKRPRVLHCNIQSMTAPHAITEWWVGQAGGKSVTKEVRVAENIEF
SVEQLLKWDPQVIIVSSPDQVNLLCNDSRFKEVSAVKNKQVFVPTPVGAHIWGNRTSEQPLMLLWAAKTFHPEAFKNLNLEEELIQFYKHFFNYSMNVEEAQEILGGGPKVDPGKKK*
>Syntrophomonas_wolfei_subsp.metylbutyratica_JCM14075T_02253_hypothetical_protein
MNNGEIKNQHRKVDTGFILIFRQETALPAYVNLLSLLANTANPQKGCSISSFQIREAQPFTAIPWGAIDAPFVQLSMA*
>Syntrophomonas_wolfei_subsp.metylbutyratica_JCM14075T_02255_Signal_transduction_histidine-protein_kinase_BaeS
MAKSGLSNKITTSFIIVSVLAVIATSLIMLLMTRQQFSTYIDNYNQVMLDQWAPIISDYYSQYGSDGLQEYLQANTIGNGKAMGQRRHHGSMPMGMGIRQGQRLIVTDANNMVVADTQGLLIGQPAKLDSLHSSRELRVDNQPRATLYIISPLGSGLSSLENQFVARLTINTAILAIVGIIALILGLALGKRISSPLAALSSAIHQLANGKLDERLSLQGDREFMELGRDFNLMAQKLDDVEKNRRRLT
ADISHELRTPLTFLRGQLEGMQTGSIPLDIESIALLQDEVIRLSHLVKELDNLSLVENRAVSLNFSRFPLSELLEHLIPVNLAMQDKGISYSIDLDSTIKEIYADHDRLLQILLNLLSNAMQHVEQGGQVTLSIQRYRNHLQFAVADNGPGIPPENLPHVFERFYRIDDSRNRREGGMGLGLAIARGYVEAQHGKIWVESSPGCGTVFYFTLPQE*
>Syntrophomonas_wolfei_subsp.metylbutyratica_JCM14075T_02256_Alkaline_phosphatase_synthesis_transcriptional_regulatory_protein_PhoP
MDSLILLVEDEKKIRDMLSQNLRQEGFGVALAADGLEALRVWQDIKPDLIVLDLMLPGLSGWEVLKRIRQKDNTPIIVLTARADEVDKLLGLELGADDYITKPFSPRELALRIKAVLRRSRPHEQVTNISFPNLSINPERLEALIEDTPLALTPTEFKILLLLAENPGQVFSRLHILDRVFGDIYEGYERTIDTHISNLRHKIEATGKSKVSIKTVYGIGYKLVAEE*
>Syntrophomonas_wolfei_subsp.metylbutyratica_JCM14075T_02257_hypothetical_protein
MKKRSSKVAILALSLLLLVGMVASAMAANGTNGQAGLRQGNAAGQQKAQTTLQAVSDLTGLSIADIRSQRAEGKSLAAIAESKGVSEQAVIDKVIAERTAILDELKADKKISGEQYQACLNNMAERIKANVERTVLGPKNGNQRGQGRGHMQGFGQGQGRGAGCGQNQANCPYAAPANQ*
>Syntrophomonas_wolfei_subsp.metylbutyratica_JCM14075T_02258_Macrolide_export_ATP-binding/permease_protein_MacB
MIDSISVIGGKDKDGAAEAIPLLDIRAGEILAVVGTTGSGKSMLIADIEQWADGETPSQRRILINQMPAGEFADDRVLRGMAAEVSQNMNFVMDMSVKDFLNLHACSRHLEQAEMLAEQVLNYANRLSGEPIGSQDKLTVLSGGQSRALMVADVALISDAPVVLLDELENAGIDRLAALKGLALQNKIVVLVTHDPMLALLADRRVVMKNGGMFKLHSTTPEEKLLLKKLEQSNRQISFWREQL
RLGFTVNEEALKQY*
>Syntrophomonas_wolfei_subsp.metylbutyratica_JCM14075T_02259_hydrogenase_accessory_protein_HypB
LRFIIVSGPPSAGKTAVMIHTIRHLLKDGIKVAACKIDCLKTSDDDNYRALGIPVAVGLSEYLCPDHFFAANLEEIYAWGSYHGAEILILETAGLCHRCAPAVQGCQSICVIDNLSGIDTPQKVGPMLSTADVVLISKGDVVSQAEREVFRQRVEMANPDAAVLSVNGLNGQGSLKLKKIIMESASVNRIEGQELRCSMPAAICSYCTGETMIGSSYQMGNVKKVDFGKFEKMRSKEPLPSSDV*
>Syntrophomonas_wolfei_subsp.metylbutyratica_JCM14075T_02260_putative_secretion_ATPase,_PEP-CTERM_locus_subfamily
LLGYGQPLIRSKLALNVNNPLRQRIVVKHMMSNMQKDELGGYLKTRLAFGGTHEELFTAPSMEAIYGVTNGVPRLVNSLATNCLLYACEKKQRQVDEETVYQAQSELNF*
>Syntrophomonas_wolfei_subsp.metylbutyratica_JCM14075T_02261_Putative_transposase
MIGFKGIMVIGSLMYWTSYINTWTVATFDVHGWTSAAVPWMARSCRLGFARVKCDDCQHEYLLPFSCKRRYFCPSCHQKRVLEFGEFLYTEVLKQVPHRQWVFSIPKRLRRYFMYDRSLLSQLSRCAWKVSSLYLKQGVSLDDPEPGAVIAVQTFGDFLNFNPHLHIATNGCFATDGGFMVGTVPDASNLIDLFRLEVFRMLEKAGKINEDIIENMMNWHHSGFNVYCGPAINPGDQEGLEKL
AQYIIRALLSQERLLYIPSSAVPDGIARVIYKGKNSSIRESFTALDWLARLVSNIMKFRPHFSSQACLNHYLLPCGILFWKKQIPIQ*
>Syntrophomonas_wolfei_subsp.metylbutyratica_JCM14075T_02262_Chitinase_A1_precursor
MKRTKTKWTLVTTLLIISLFTTTCFADEIIKKEIKKDQFKVVGYYSGDLFNEPVEKLQTDKLTHVIYAFLIPQEDGTLVDLEKPEQLKQLVSQAHNDGAEVFIALGGWSYQGKPLAPVFEAAASQEKTTLLIDNICALVEEYDLDGVELDWEHPNVNSIADYERLAVELKTALDLQNKELTAALNGAWSATEGPEVSKLITNACLESFSFVNVMAYDMNNEDHSPAWFAETSVDYWLNRGIPADKI
VLGMPLYARPSWLQYRHLVEQNPQFAYSDYAPTVPLESYYNGLNTLREKTLIALKQAGGVMLFDVNADSGDKTSVILMINDILSRTKHLSKDELNKYITVIIDNKELVFLEEEGFGVPFIDENNRTLIPLRKPLEAIGATVSYDEKNHMVKVEKAGTNVTLPIGENIITVNGQEVKIDTKSIIKDGRTYIPLRVVLAAFGYDIEWHGNSATILILNNSKE*
>Syntrophomonas_wolfei_subsp.metylbutyratica_JCM14075T_02263_zinc/cadmium/mercury/lead-transporting_ATPase
LFESPVARAILKAAHEANGTFTEPENFRQIVGKGIEAMVDGKQVFVGAVNEGGPDYEKIKNEDHFGTILIVKQSEEMVGYLAISDSSQCRFRFCGGQLGIANKEKFLILSKGCINKSFNPKYLPYSLLYLITAAQTHIYKLLS*
>Syntrophomonas_wolfei_subsp.metylbutyratica_JCM14075T_02264_hypothetical_protein
MWNGEKSIILSKLCVLLFMGLLVAAVVTAPWLTQWYVQFSQAGLEEEAAYFMATIYVGSVPAAYLLYSLLKLLRRIETGQVFTAKNVELLRRISWSCFVGAGIALISLLYYYPWFFMGVAAAFMGLIVRVVKNVVAQAVELKNEADYTV*
>Syntrophomonas_wolfei_subsp.metylbutyratica_JCM14075T_02265_putative_transcriptional_regulator
MPIVVNLDVMMAKRKISSGELAEKIGISPANLSILKTGKAKAIRFSTMEAICKSLDCQPGDILEYKPDQD*
>Syntrophomonas_wolfei_subsp.metylbutyratica_JCM14075T_02266_hypothetical_protein
MDEISINKNEADLISGSLVLGESEESVPEVPILKKGKAPFAADRRDSYFALFAFVLGFLFARWVLFAWQGWGVTLFTLLFCGSVTLYMLKKGVQIPKTSWLWLVVVLLIGLSFSLWTNNGLAPLHGLLLFGSAVYWILCATGLLIQGKTSDLLLLDSYNALLVIPFSNFGCQYKGLALLGNNKLTRGRQVLSIALGLFLSFIVAAMVLPLLMEADSGGFAKLINGILDGFRGLGDKFWETFWQLILAVPIA
AYIFGLVAGSAHKRGTGLFEKDSTLQQISGLRILPMTTVYILMGLLCTLYIVFIGSQFPYFFSAFVGQRPEGWQIYSEYARSGFFELCRIAVINLFVLTAANIMSQKHSRDSMFLKLLNVLLTILTLVLIATAFSKMTLYIGAYGLSMRRLLPCVFMTFMAVICGGVIALQKWSFSITRLAVGAGVVIFCMLCIVNPDSLVARYNADRYLSGTLENFDVEILYRSGPAGVDSALKVYEQTNDPALQFKLKEYLL
AEQQQADEVAGEPGDSLERAHARYRISEFYCGQSLLSE*
>Syntrophomonas_wolfei_subsp.metylbutyratica_JCM14075T_02267_hypothetical_protein
MKRINLRDYYPSYTSDFFMEVADEIALSLKRFELD*
>Syntrophomonas_wolfei_subsp.metylbutyratica_JCM14075T_02268_Transposase_DDE_domain_protein
MLVVKDTQQCLWESILPPEILVLNTELSKVDTILDDDRFIRPFIKRFHTTTGRPTTPVDTYLRLMYLKFRYQLGYETLVQEVDDSIKWRKFCYIPLDKKVPHSTTLIKLTNLYGSEIVEELNHLLVQKAAEEKIIRGRSLRVDTTAVPSDIHYPTDASLLSDGIRVITRTVKQIKKAGAAIRTDFHNRQRTVKKKILSMTKILKRRTGQAYNEVRQVTDQVMETAQQVVEEANRVLKNARHYIWRKGKD
VSTKARKLAEKLEDAITITEQVIDQTTQVQSGNTHIKDRVVSLFDRNARPIRKGKLSAPTEFGRKVLIQDTEEHVVTGYQVYEGNPSDDSLLTDAVDLHTKNVGRVPVNVATDRGFGTAKNEQTLTTLGIKRCILPRKGKIGTERKQYQSQPWFKRLQRWRAGGEAIISLLKRKYGLNRCRSRKPHGTQTWVGYGILAYNLKRIATLMA*
>Syntrophomonas_wolfei_subsp.metylbutyratica_JCM14075T_02269_Regulatory_protein_BlaI
MNNFQKLSETEMEVMQVIWASGRPITSSELLDFFARNKGKAWKGQTMATFLARLVEKGVLISIKQQGRTNIYEPCMSPEEYRSQEAKSLLETLYEGSVKNFLATLYDGKELTKDEISELRHWFAEKAGEKNG*
>Syntrophomonas_wolfei_subsp.metylbutyratica_JCM14075T_02270_Regulatory_protein_BlaR1
MGNLLSNLLAMSVTGSVVTGLMLILRPVTAKIFPAKWQYGIGKMAIAFFLFPVFLFAGKLHLVQPVVESYPAEPYIIQKALPSNGFVDAMDTLMEKHLTVELMEAVLFIWLVGAMVFAGWHFYCYRRFSKQLRTDSFSVADDTATVVLLSSCKAALGIHGEVKLMLNPKIKSPMLVGVRRPMILLPTSNMQELDLKLVLAHELMHLKRKDLWVKMLALLAGTVHWFNPLVHVLRKDVSTWGELS
CDEALACDMSNEERKHYGEAILNTLDNHSGINTAFCSSLCESKKHIERRLTMLLNVKKMKKHVALFAVVVIVAIAGIGTAVSALAADNTLKADSLTRNPFAPIMNSEVNVAFTTDTPVIIGSIDGLKEVELKGDGKEYTFEEFKQWLEAARLEAAELVKIGIWTQEQADKKLALYENTLADIEQKGGKTFGKMIDKGDEIFPETVDVFYTADELVFELPAKLADTANGEVGFGLSVQPKK*
>Syntrophomonas_wolfei_subsp.metylbutyratica_JCM14075T_02272_hypothetical_protein
MISLEEKQQIILRHYHDGESQRKIGYLYSFV*
>Syntrophomonas_wolfei_subsp.metylbutyratica_JCM14075T_02273_hypothetical_protein
MSNFDISALISSDNNPNFLVSSFIDVLTRQIERFRGEEQCCRLLVGMQRPEEELLRNALARIDEGLMDNPRALALKKEIALSFRVP*
>Syntrophomonas_wolfei_subsp.metylbutyratica_JCM14075T_02274_hypothetical_protein
MKASLGNEELLNLPKTAFLCSRRVLASAVLKCYDWANAQREAGRCVIGGFHSQVEKDVLHYLLKGTQPIILALARGLKTRLEPELKTPLEQGRLLIITPFDHSVKWVTAENAAIRNRMMIELADEIVVGYSSPEGQLDMILHYYKERMIVRL*
>Syntrophomonas_wolfei_subsp.metylbutyratica_JCM14075T_02275_Toxin_ParE3
MARLKIRYTRDAVDDLDSIFDYIAEDNRKAAARLLERIENSILKLADKPRLGTVLPTNDLSLVKAGYRRIIVQPYIIFYRIAKDEIFIARILHSKQDWMNLLLENTYEDNS*
>Syntrophomonas_wolfei_subsp.metylbutyratica_JCM14075T_02276_hypothetical_protein
MLNRIRRPGLMIVKPSSVIRSDYKGFSKLCHETDDPILITNNGENDLVVMSHEAFCRREAWIHLQAKLSAADKQIAAGELFDHEEVFSHLKERIHGQA*
>Syntrophomonas_wolfei_subsp.metylbutyratica_JCM14075T_02277_antitoxin_HipB
MKNYVKVYRAMHNLTQAQLAEKLEVSRATINAIEKERYDPSLKLAFKMARLFKVKIEELFEDVETQH*
>Syntrophomonas_wolfei_subsp.metylbutyratica_JCM14075T_02278_hypothetical_protein
MWSFIVYLMAALAGTTAILIIDYATDWVFRTGSILSILSGWFLAYIILAWSRKQKHTGTAWAMFMFFFLLIPMVFFTFIMTLNNILHWQLPFGVLMFIYAAALIVIFLLSRRVLGQLLMDIPPPWDKTPKPDERDIHLLVQAGHPGFIILAGAIILALLQPWLHYNDLRLWLGTLAVGELFWFGSFIFYSYTKQRNKLSNFFSLSIIIGFLLCTIVMLFDYSKNNSIDAEATLAVLIGFFLGYIVLRYLKSKPNYY
WGTTIIMMLASFILPAMVILAAIVMRVDVPGWAVGLVWVLGLGIESISTYSATTRLAGTDNAIENGIDERQEQHVLWSGLFGFLALMIMLIFSLLQPWVSLEDTRLWLSVLTVGITIWLLSYVVLETGR*
>Syntrophomonas_wolfei_subsp.metylbutyratica_JCM14075T_02279_YcfA-like_protein
MPSLPVISGQQAISTFSKDGWIFVSQKGSHIKMTKEGSSVKLIIPNHKTLDRATLRSLIRKSGLTVDEFIKLL*
>Syntrophomonas_wolfei_subsp.metylbutyratica_JCM14075T_02280_hypothetical_protein
MKLPVILTPGEDGWIIAECPVLPGCISQGKTKEEVLDNIREAAELWLEEEPAGFEISAEYTEIEVVI*
>Syntrophomonas_wolfei_subsp.metylbutyratica_JCM14075T_02281_hypothetical_protein
VLKKQVLGVILIIIMALGLAGCNSAVTKGGQSEGKDVIPLIMLTSEPAGENDAGSMEREARLASWDLKSGKLSWEQEACFRQEMGEEYNPRLVWDGGSRLFACSFGSQETGRLIKGHASNLSVQEVRTDSMPMVAAIKASSEQDELLLAYPRSPSGESPDSSIPEVIVIESHRGDLVVKRELPLPDYAAKGDIMPLLISGNSSQFKLLLLNNDVEAGKNGLLLAKVAEQQEDWQKIKGEALGLIVGGGT
DLAQVGSNIYRTIPEPAKLSLQQREPVWEEFSPLSDLQAGLSKNIEKAMEKYGDAYCNLRLGSFKDILLVAADLPEEHWIWAFRGDKCIGRLYTNNETQSMESSPGNEKMQATAFSARADWFILPTDGYGQYK*
>Syntrophomonas_wolfei_subsp.metylbutyratica_JCM14075T_02282_Ferrous_iron_transport_protein_B
MSTINNPGLSVLQEIEPLLPTNTANIKDRIVSSIYQQAESVSAQVVRSKNSQYTGLEARLDNILTSRWLGFPIMLLLLTLILWITITGANYPSQLLATGLFWLEEQLTALFLAVNAPVWLSGLLIQGMYRSLAWVVSVMLPPMAIFFPLFTLLEDLGYLPRVAFNMDKLFRRAGAHGKQCLTMCMGFGCNAAGVISCRIIDSPRERLIAILTNTFSICNGRFPTLIALSTIFMGTLVSQAYSSVLATLAVVAII
MTGIATTLLVSSLLSRTLFRGMPSFFVLELPPFRRPQLGQILIRSLLDRTLFVLWRAVVIAAPAGALTWILANSFVGGTSVFALIVGWLNPLGQLMGMDGIILIAFLLGLPANEIVLPILIMGYISAGSMLELDSLNALRILLLDHGWTWITALCVMLFSLLHFPCGTTLYTIFKETRSARWTLLSALLPLLLGIVVTLLINQGAQIIMR*
>Syntrophomonas_wolfei_subsp.metylbutyratica_JCM14075T_02283_Ferrous_iron_transport_protein_B
MAINTTLLSESTENSPDKQNNFVIALAGNPNVGKSTVFNRLTGLKQHTGNWPGKTVIKAEGTFSHRNRHYTLVDLPGTYSLHTSSSDEEVARDFICFSRPDITLVVCDATCLERNLNLVLQILEITDRVLVCVNLLDEAERKGIHVDIAMLARQLGIPAVGTAARSGRGLESLKNVLDDMLAGKIAIRPRQLVYDPELERSINKIEKRLAPYLPAWINSRWVALRVLERDFSILEHISNHKNNDFPRQVL
KEEVALCLP*
>Syntrophomonas_wolfei_subsp.metylbutyratica_JCM14075T_02284_FeoA_domain_protein
MPHTLSLADIPIGASVRIAEILTPGNNRRRLLDLGLVPGARVEIIRRSPSGNPTAYLIKGAVIALRNENAAQIMVDTN*
>Syntrophomonas_wolfei_subsp.metylbutyratica_JCM14075T_02285_hypothetical_protein
VLHPCFANACFAYLCNPMQQKGVFAVESKIDYLTLLSSGCNLMQQKEFLHRITTLIIPDYGLLSHSPQKCAASNLPLIQL*
>Syntrophomonas_wolfei_subsp.metylbutyratica_JCM14075T_02286_hypothetical_protein
MDNTVLSFLQASKKKTILLSELEQLVGGQASYEDFALLINDLEQKGILTAVKQQGYNQKPIPLANAYRIIQSKIPADFLQEIERFHFILHPLIKLEAYYSLAASEWENDLPYIQHINRFLKEQGLPDDEATAPERSYALVGDEKWIDEKGGKKLLERVGIWSAMNIVYLPDPLMLAVNQLQHAQRDETGLHLVVENKATFHALLEYLPDTMFYSLIYGAGWKITADIIMLDKQLSLQNREHCIYYFGDLD
YEGISIWHSLNMRHLALPAIAFYRALLSKPAARGKETQVCNEEALAHFLTYFSFAEQETIRDILGNGGYYPQEALSRAELGAIWRDASWS*
>Syntrophomonas_wolfei_subsp.metylbutyratica_JCM14075T_02287_hypothetical_protein
VELDARNITANYRERVQRLAIFDPLFRLENKKTTDNSNRPIDYFGLGLLTLLFFFENMLLRNHKTGVKELAQFFQSINQGELDLDSEGYEKLARDIIDVFRPPGGKRNSRSFYDWHTRQEDTIFISILKADRRDIKSSTQYYSLADQGLELVFATREYYSEFQVSINQLLLRKQLERGQFWGALRQIDEMRVAVETLEERIIRIRHEVQRNIVSESTYQRYRDIIEEINLRLSREDKEFEELQVFVGETRERLSY
ERKTPKDEQTYELMVRIDVELFEVHNQHGNLLRESIELKTTALQAAQESLYFAGIDSFNFQKEITEQLLAAPLPLTAARTLVEPFLFLERREQWSPLTVFARQRVERRKENVETEGFLQARDEELQAREKAVRRKNYRQVMELVLEALGEGEEIYLQEVVNYCYNSGQEHLLKQRFFYDFWILLHQRSPINLDKDLESIQESTLGEAVAILTGKARSLTVVERGEVLKVTARYSIRNMQLRLEKEKNAL*
>Syntrophomonas_wolfei_subsp.metylbutyratica_JCM14075T_02288_hypothetical_protein
MLYEEQQVMEAFRLYTRLALKGYGDKEALRLYLADDVVRGLVEEFAQEVECTVISAGDQLYLIPLALSSPFHVSNQSLKDQYLPAYAVNADIYLMYVAIIILFGEFYDSYQTMEATREFLTLTDHWLTRLNERLLALKEINPKELEKMESELEYNWQQVIDKWDALDDLKEKVKARDARTRSRLGFLNTVKKFLEAQDLIRDIGEDEDEVTLTEKAQTIIQRYYMEVQHNQNILEFIYRNSPDKERK*
>Syntrophomonas_wolfei_subsp.metylbutyratica_JCM14075T_02289_hypothetical_protein
MPAISRIRFTNLVFENGAKRYNDVIFQFDSYNGVILLENGGGKTVLVQAVLQTVLPHTLLGERKAKDTFALENSPAHLAVEWLISDRPRRYALTAVTLFHSKEGMDSYKYVYEYGPGDEHSLEGLPFVREGQNGPSRPSSKEEIADYYQYMSRNHLNAHYFSSNREYQSYIEDNFKIINSEWKSVARINAAEGAVEGFFAGCSTTTQLVNQLLIPTVEEALAGGGTKDFVETFKRQREHFKKHRQLRD
KILESQQVDRKINSYVNRYALYDEARQALLKVKGDTRSLYQLATQQQEQVAQRLEEQRQATTRLQEKRQEWERKQASYQLASRRQELDYTGQIYEERRKEYEEQHEEYDQKNSRLQNLEISQLKARIRSSDEEIKYYTEQIRNLAADPDVRNLEEKIKNNAGYLRYCYLQEEEILNGQKQESQAELDNVNRELREIERNHKEQQNHYYKLREQKGQAEQQRIQAENDMQSIASQILANPVQEEVIKE
KAKWEQRVAELERNRLENQGLIRKLQEDKRRLLEELAQLRPQLQNYAREEQSLKDLNERIQEEQESLLLRMKELKTELYSVHSLYTQQTTVINHFEDTVENLRLHKERAILEESQALALHTFYESSIYYSADPLVETWIDDWREQFTYLEAGAVYVEKAAQQLGYSVSEYFSLYPFWAISLVCGTSEANNLLARLRRQAMMLTHPVFILTQEEARSILDGRGEPSHEAPEVKRRVFPANWEANLSSEAF
QNWKQELEKKAHDASSERRQQEVLLQEMEEFRKQLLQFWEKHPYEETCQVQQSWQELREQLVELDQGIRSREERLQEIDTRLDSLSKMNNEISEEHAYLSNRIMKAQDYIRKTGERDKAELDLQQCQELLLAREQELERLNRQKERGERRKQELQQELLMIKQELRYLLEQPLYQEVNSAIPQESKLSRDLLEIERRDLVDVLHQKQKGRQQLEDRLNQARTARNDSEKNLERKRRQCDTEIDE
DFIFPPGGEEEINVIIQQTKVLKQQLKRLEPGFKQAEKDYAIAQDRFNQQEGDFYKKFTQAVVFSQSLIVVKDELDQEYQEIQSKQNYLDEQAIILKAEEDMFKQALVELERYHERYEYLQDDIAYVDLPSSVAQDFSYRPMPIIERYIKELEDKQAHLNQLEDGLEQERSRLGQFCREELQDIKLRRMVMNGLKYLDNYQEVGEWKNRLSSRIALAIRHAEDDMREHDRQLQQFINHIHSYLLTMAG
ELRAIPRKTRVKVQDSWKEIFQFDVPQWEEKEGKDELRRHVDWMLNQIEENRYRDENGKENYALMHKDIEKWLQAPQLLSNVMQGKNIKVKCRKVTADGKVSSYPSAWESSNQWSGGEKWSKNMALFLGIQNYLAEKRQAVNPRVKRSRTVILDNPFGKASSEHILDPVFFIAEQLGFQIVALTALAEGKFISDYFPIVYSCRLRPSTSPDKYLIAADKEIRHAYFQDHDPEALRRLGQQKQMELF
*
>Syntrophomonas_wolfei_subsp.metylbutyratica_JCM14075T_02290_hypothetical_protein
MSMQEEFYSELMKRFRKIVEDNNLLEQEIIITGRPLTAEEAIGKPKRQDFPIIKGKEKLMQAEFKGAKGQAFTDMAGNFKGTLQQVLNMLLTNNFDRAVLIASMNAVCRYLGLIDRTVHCHDEQPEQCAAELVEQIKRDFGIPRVALIGYQPAMLEKLAAFFPLRVVDLDPDNIGKIKSGVQVEGYEATDEVLEWCDVIAATGSTVVNSTITAYCKQKPVYFFGTTGAAACHLMGLHRFCALGG*
>Syntrophomonas_wolfei_subsp.metylbutyratica_JCM14075T_02291_Manganese_transport_regulator
MGEQGREFRTVRGYQIKQQATKTITASMEDYLEMIYRLAGKKGYTRMGDLASALNVQPPSASKMVQKLADMGYLQFEKYGVIELSKKGQEHGQYLLKRHETLERFLSIIGVKEQLLEETEKIEHYISPETIGKIILLVEFMEDNPEWKQAFYDFKKHEG*
>Syntrophomonas_wolfei_subsp.metylbutyratica_JCM14075T_02292_hydroxyacylglutathione_hydrolase
MFDVFNYEDIICARGSREMPWKPSFQMNVYYYLIDGLLIDSGPYSLAEEAIEFFNAHKIEQIFLSHIHEDHAGMAYWLQEKMQVPIYLHESSLEEARQEPELAKYRLDIWGRRKPFNAEPINDTIKTGSYVFDVIDSPGHYPYHKVLHEKNKGWLFSGDLLNNLKPKSVMFDENMNETILSIKKILDLDFTTVFCTHSGIRKNAKVLFQYKLNYLLELQKDIQTLRNRGFTNTEIAQQLFPGPDPIGSLSG
GEFSPYYLVSTL*
>Syntrophomonas_wolfei_subsp.metylbutyratica_JCM14075T_02293_hypothetical_protein
VALLGDPFVANLPRPLSNEELIQALRVDIAGELEAIIGYEAHIMATTDERVKKVLTHIANEERRHVGQLQQLLFVLNPGEQQYFDQGLQAIQQQQSQNFQMPMQ*
>Syntrophomonas_wolfei_subsp.metylbutyratica_JCM14075T_02294_Dinitrogenase_iron-molybdenum_cofactor
MPKKIAVCSADSSPSSPVDGRFGRCKCFMLWDPETRQYESLANTGPEAEHGAGTGAVQALVRNNVGMVISQRVGPKAYAALKEAGIKVFAGAGGKTVEAARQSYEAGELHELLTPNA*
>Syntrophomonas_wolfei_subsp.metylbutyratica_JCM14075T_02295_Dinitrogenase_iron-molybdenum_cofactor
MLVAIPKEGDMVCAHFGHCEEFTLYDTNAKTLKSVTNPGHQPGFLPGFLKELGTELVIAGGMGGRAQDLFAAQGIHLIVGISGKIDDVIVRFEKGELVSSGEVCSEHAHAGDCHS*
>Syntrophomonas_wolfei_subsp.metylbutyratica_JCM14075T_02296_ferredoxin
MQIAVASGKGGTGKTLVSTCLLRILGDKNSALIDTDVEEPNAGLVLMASAPKTADVSRLVPEIDYNYCNFCGRCAEVCNFNALLVLNEKVLLFHELCHSCGACAYLCPLAAIKETEHPIGVIEEAHLPEHGRVLTGRLNIGEAQSPPLIKAVKKQGERAALRIIDCPPGTTCAMIESIRDSDYCLLVTEPTPFGLHDLELSLEAVAMLGIPGGLVINRWQGEDGELLALSGQTGVPILGRIPFSVELAQAY
MQGRDPLEAMPVLLDILSDVFRQIKGALS*
>Syntrophomonas_wolfei_subsp.metylbutyratica_JCM14075T_02297_ferredoxin
MKEILFISGKGGTGKTALTSSFIQLAGNCIACDYDVDASNLPILLKPQNVRAYHFTAGYSAHIDEESCISCGLCTELCRFDAISSEYQVLPLFCEGCGFCEKICPVEAIAMQARSSGHCFEGVFNQKKQNLFFADLRPGEENSGKLVAQVKSMARKMADMNKAELIIADGPPGIGCAVISSMVGVDLIVLVAEPSLSGFHDLQRVHQLMQLRGIKGVLIINKWDLNPPISRELENWALKNSLPLAGKIPFSA
IIAESIAKAQIPATNPELHKMLFPLWENIIGQLNAV*
>Syntrophomonas_wolfei_subsp.metylbutyratica_JCM14075T_02298_hypothetical_protein
MPNRDGTGPLGRGTGTGRLGGKCSVGRGYNCPNPGRGRGRRNGSGNGPRGIGGGGIRELNTPEDTSGK*
>Syntrophomonas_wolfei_subsp.metylbutyratica_JCM14075T_02299_hypothetical_protein
MSMASPKPDKKYSYADYLQWPDEQRWEIIDGVPYDMSPAPSTKHQSISMELGRQIANYLRDKECQVFAAPFDVRLPLNEKKDEEIYNIVQPDLSVICDPSKLDEQGCKGAPDLVIEIISPFTAKKELNEKFNLYERSGIPEYWVVFPKFNVVVVYSLDDEERYQKSGEFTSDQLLSTKLFPGLEIKLEDVFR*
>Syntrophomonas_wolfei_subsp.metylbutyratica_JCM14075T_02300_hypothetical_protein
MKDPAYTVECARILDIVPMLNLERKYFDTCWHSEAALICKLIEQEPMMFRVCKVNGKVKGYYWVFPLEHSIWNKIITGEMPEGELVKHIRSFDEPELYLYISSVIVDQTDKLRKNYTKALVYDFGRRFVLGKSSDAPDVRAIGAFTISEGGRRLMERAKFSYQGNFRVHGKEMKSWAIDRRTLAQQSVSERQLRLTRKIA*
>Syntrophomonas_wolfei_subsp.metylbutyratica_JCM14075T_02301_Thiosulfate_sulfurtransferase
MSNLKRPPLKRSMKLVTTTVVFLLLFSLAGCSSSKQGVNSSSDSAPRLTDQAMEGYYVDARWLQENLDQVTIVDARADKEYKKGHIPGAVNISWQALSNMQPKQGEPGWGVVLSNEQLSKKLGEFGINGKKDIVVYNDPAGLGEDGRVVWMLRVAGLQNSRMLFGGWPAWQKAGGEETKSIPQIKAVSFSITNPDTSLLATTEYIKANKDKVKLLDTRSPKEFTGKTDHGERVRGHIPGAIHL
HYSDALNADGTVKTVDELKDMFAQAGLKPGDEVIAYCTVGIRSGFMTVLLRMCGYENAKNYNASFSEWAGDPNNTIEK*
>Syntrophomonas_wolfei_subsp.metylbutyratica_JCM14075T_02302_hypothetical_protein
MYCIQWKQYKTLFKAIISLGLLIWLGYSIQWRELGQILLDVKLYWLMTALGWIILSMIISTCKWQVVLKAQSLNLDWVELWKAYWAGIFMNNFLPSSIGGDALRIYRVNEITGNVSAATASVLMERVLAISGIALVGMVGAIVTGKTRDGIWLLFIVLLLITIILTALLLSRYSCKWPRARRLGQFLEGIGDQGKNFRANWPKLGVVVALSLLFQITVVAVNYNIFLAFNLSGLGWWDACYLIPVTSAVAM
IPVSINGYGVREGAYVALLAGYQVSPTVAFSSSLLFAFMVSLCSLYGGIVILRHNKQGDINNGYQKGFANSR*
>Syntrophomonas_wolfei_subsp.metylbutyratica_JCM14075T_02303_C_GCAxxG_C_C_family_protein
MDTKRASQIAGEEMKKGRNCCQAVLLAAKEVWSIPVDENILAAAALMGEGMGAGCVCGALSGIIMASGIRQHYQAHPAGNNLPVILHDAFKNEFGVTCCRAIKNKRSLVEKIGRKGCIQLTERAAAIMLEEWEKGSNANKDICNSTGTKRGRMSS*
>Syntrophomonas_wolfei_subsp.metylbutyratica_JCM14075T_02304_putative_glycosyl_transferase
MLTKISVIVPVLNEEECLPELLNSLQRFSGIEIIISDGGSSDRSLEICSRHPVKTVTGSAGRGMQLNRGAQLATAPILLFLHADSQLEPIVVEQVLEAIKEGYHWGCATMDFDERTPFYRVLAFFSNLRARCWKSCYGDQAIWCRKDVFFNNGGFPDIPLMEDLALSHHLRSKYRCRVVSGRVTSSVRRFIAGGLWATLIKMQVLKMLYFLGTSPERLADYYQQWRRVSL*

>Syntrophomonas_wolfei_subsp.metylbutyratica_JCM14075T_02305_transferase_1,_rSAM/selenodomain-associated
MKCALIVMSKIPIGGFTKTRLMGKLSKEECVALHSACLYDICSLLTELGWPAYFSYIGVSSTGEVINKEILEQKCNLPQGMLKPFEVLEQKGSDLGERILYSLDQVLKHYTAAIILGSDLPQLKVKTLQKAAQLLDENDIVLGPCNDGGYYLLGVKHPYPFLFKNMPWGSKQVLRLTSERAKIHKLKLAFLDRDRDLDRWEDLLYFMAQGVIDKRLSSLHSYRYISNLRVRKEALKRGIYNENTDKANL
RQSSGWSSS*
>Syntrophomonas_wolfei_subsp.metylbutyratica_JCM14075T_02306_biotin_synthase
MKTPIKQICDKALGGALLSANEIRGLFAIPPLSEESCHIQWASRSISEKAGLAEIHGQVGVNTAACPCNCRFCSFAAANLIFKENQVSDLEYIIEQCLHLQEQGANAIYLMATANMNFADFLNIAGEVRLKLQPESIMIANVRDIDYYEGLALKEAGFAGIYHAIRLGEGEVTRISPRKRLETVQAAKKAGLKVGTCVEPVGPEHSLNELVEKTIIAREIEPVFSGSARRISIPGSSLEQYGMVSEASMAHILAVV
HLAMGPGISGNCTHEPNTPGAIAGANLFWAEAGFNPRDTFEKTEASRGFDIKKCQDIFKEAEFPVLQGSSVFFARD*
>Syntrophomonas_wolfei_subsp.metylbutyratica_JCM14075T_02307_TVP38/TMEM64_family_inner_membrane_protein_YdjZ
LNKKVWKQWWFKIALLVIPVLLYYLVPAFQEIIQNAIKVLSRVDVDAARDYILSFGIWAPVVSFFLMVFQSVAAPLPAFVITFANAGLFGWVKGAALSWSSAMVGAILCFAIARLYGRSVVEKFTTRRALKEVDVFFEKYGKWAVLIARLLPFISFDIVSYAAGLTAMGWWEFIWATGLGQLPATLVYSYVGDMLVGSVRNVVFGLLILFALTTVIIMARQIWRDRNAMQTTSEEI*
>Syntrophomonas_wolfei_subsp.metylbutyratica_JCM14075T_02308_4-oxalocrotonate_tautomerase
MPIINVKMIAGRSQDKKQELVTVLTSETARILEIEPDWVTVVIDEYTRDNWASGGQLHSIKLGEGFGRQGTSK*
>Syntrophomonas_wolfei_subsp.metylbutyratica_JCM14075T_02309_hypothetical_protein
MSMASPQPDKKYSYADYLQWPDEERWEIINGVPYDMSPAPSTKHQAISMGLAGQLWSFFRDKECSVFAAPFDVRLPLNDEKDEEIYNIVQPDLSIICDPSKLDEQGCKGAPDLVIEIISPFTAKKELNEKFNLYERSGIPEYWVVFPKFNVVVVYSLDDEGRYQKSGEFTSDQVLSTKLFPGLEIKLEDVFR*
>Syntrophomonas_wolfei_subsp.metylbutyratica_JCM14075T_02310_nif11_domain/cupin_domain_protein
MNLFDIPGHLRDKEFFESLLPDRGILIERIISTGQASPTGFWYDQDRDELVMLLQGEAQLAWPEGRTLSMGRGDWLLIPAHERHRVERTSSDPPCIWLAIHGQLT*
>Syntrophomonas_wolfei_subsp.metylbutyratica_JCM14075T_02311_Ribonuclease_VapC11
MILVDTSVLIGFLKGQINEKIQLFDQVLSRNIDFGFSSYTFQEVLQGARNEKEYKQLRDYLSTQIIYFLPEKTATYEKAARLYFDLRRKGITPRSTIDVLIALTAIENNLLLLHNDRDFDLMAGQLETLNILKMI*
>Syntrophomonas_wolfei_subsp.metylbutyratica_JCM14075T_02312_hypothetical_protein
MRTNIIIDDTLMQRAMEVSGLKTKKDVVEKAIIEFVERRTRKDLKELRGKIQFAGNYDYRAMREGRK*
>Syntrophomonas_wolfei_subsp.metylbutyratica_JCM14075T_02313_hypothetical_protein
VLYKCHVIVYVIILIFITIVLNIRRRCCHLPFLSQGIDINMDFDYDFKVRTNNRNKTGDKPFASPEARALRDLVIEEAPDIIIDFHGWINCASGDKEIADIFCQHLGLTYEPMEKAIYRGFFLDGQQKYARSVLVEYPDPITGQGACDLKSDQKYDYSQDKLDELQYSINTLKSIKDIIDHEL*
>Syntrophomonas_wolfei_subsp.metylbutyratica_JCM14075T_02314_Antitoxin_DinJ
MAQTMVNFRMDEDLKKSMEKVCSDMGLSMTAAFTIFAKKVAREKRIPFEVSADPFYSESNMKYLEQVIADINEGRAKLFEHDLIEAD*
>Syntrophomonas_wolfei_subsp.metylbutyratica_JCM14075T_02315_Toxin_YoeB
MQLLWEERAWEEYGYWQTQNKKVLKRINILIKDIQRNTYEGIGKPEPLKGNLSGWWSRRIDEEHRIVYCEKDGSIIIASCKGHYEE*
>Syntrophomonas_wolfei_subsp.metylbutyratica_JCM14075T_02316_NADH_dehydrogenase
METREAIFGRRSIRKYLDKPIEPEVLQDILEGAVMAPSAVNNQPWYFLAIQSQEQMEKLRGIFQEVSVKTRAVLEKRFPNNPEVVSETLGFLTGLGNARVCVLVFLLKPSKDYDIIAVESTSAAIQNLCLMAYDKGIGSCWMTAPLSTGFSDILEKTFAPGKGKFIAAVTLGYPAVSPQAPKRKPGRYEII*
>Syntrophomonas_wolfei_subsp.metylbutyratica_JCM14075T_02317_hypothetical_protein
MCAIAFKESEPRIPNILPVTEIDRVAYLLKYGYYNMQIQAVITFDQHLDTEVLKAAVMLSLDAEPVLGCRFVEDEKRPYWQRFLLAAPPKTKP*
>Syntrophomonas_wolfei_subsp.metylbutyratica_JCM14075T_02318_Acyl-coenzyme_A:6-aminopenicillanic_acid_acyl-transferase
MLGYESIHNDFEIIRPEKDLLLHSNHYLTERFKEGDTAPQYQPDSYHSLDRIRSFMNQHYGHINVETVMEILADHEHHPYSICRHIDPAAPISSVTLASFIAVPAEGAIYIAAGNPCEQDYVRYSF*
>Syntrophomonas_wolfei_subsp.metylbutyratica_JCM14075T_02319_Antitoxin_VapB
LDTAKIFINGSQAVRLPKDYRFDGNEVYVKKIDDIVVLIPMDSVWTILESGTHYFTEDFMADRKQPEIQNREEL*
>Syntrophomonas_wolfei_subsp.metylbutyratica_JCM14075T_02320_Regulatory_protein_BlaI
VQEVPQISDAEWQVMKVIWADSPVKASEVIDELANTSYWKPKTIKTMLRRLVDKKVLGYYQEGKSYLYYPIVSERDSIKAESDFFLKKVFNGSLNLMLASFIEEEKLSQQEIEQLRRTLDDKE*
>Syntrophomonas_wolfei_subsp.metylbutyratica_JCM14075T_02321_Regulatory_protein_BlaR1
MVNLPVISSLFDWIMLSSLMGTILICLILSVKFVFKHKLGANWHYYIWFLLLLRLIIPFTPQSPVSIYNLLNQNSQNMHSDNIYDYLGEAKHPAIGITDATGISARDADYRIQNNQGVAGSLNEYRKSVDMKDSDRRNIFIIIWLAGVFALATYTLIINLKLLYKIKSEPIINNSSIIAELEECKSSMNISKRFAIVTTRQVSSPALFGLINPILLPDSMVKSLGAGDFNYIALHELAHWKRKDVLINWITVILQILH
WFNPVIWYGFYRMRHDCELSCDALVLSRLAPEKHKEYGHTIIRLLEMTIKTQWIPGTTGILTRKPHIMRRVSMIANYKKESHNLTLAALVIFIVMGVLGCTNAQDSKNPDARANIDESTYNQLLGDLKNNQIAAGTADVEGPGIIVNLSDQAADNTTKGIPEHGLLVHDTDILLTVNELIKAGAEAISINDERLIASSEIECSGDKIRINGTPCNNPYVIKAIGNSEKMLETLESKGGYIEFFRKLGFGANIE
KSDNLFIAKYSGELNFKYARVKN*
>Syntrophomonas_wolfei_subsp.metylbutyratica_JCM14075T_02322_Germination-specific_amidase
LTTYITLGSRVLRRGDEGTDVELLQNLLKILPDPIGSGIKEKGVFGKETENAVKKFQKYFNLAIDGIVGANTFLFLGVPTASYLPPGGNLFGARTLKKGSYGYDVWVLQNRLATTAKKFAETLGQPANRNFDSKTESAVKIFQRDVHLEDDGIVGPQTVYQLYNYATMGARILQKGRWDRNQGYDVYWLQRNLQELGYYQGKLDGKFGSQTEAAVKKLQEASGIKVDGIVGANTFYHLASC*
>Syntrophomonas_wolfei_subsp.metylbutyratica_JCM14075T_02324_Glycine--tRNA_ligase_alpha_subunit
MNFQELILRLHNFWGQQGCIIQQPYDVEKGAGTMNPATFLRALGPEPWKVAYVEPSRRPTDGRYGENPNRLQHYYQYQVILKPSPDNVIDLYLASLRDLGIEPEKHDIRLVEDNWESPTLGAWGLGWEIWLDGMEITQFTYFQQCGGFDCLPVSAEITYGIERIAMYIQNKESVFDIEWVDGISYADVHHQAEVDYSHYNFEAADVGALTSMFNICEEEAVRVAERQLVQPAYDYVLKCSHLFN
LLDARGAISVTERTAYIGRVRNLARMVAREYLEQRKRLNYPLLKDKNLREQLIIEEEVL*
>Syntrophomonas_wolfei_subsp.metylbutyratica_JCM14075T_02325_Glycine--tRNA_ligase_beta_subunit
LAQDLLLEIGVEEMPSAYMLRALKDLENLARKNLAEARLSCGEVLTWGTPRRLCLWVKEISEEQEDSLLENRGPKKSIAIDANGNPSKAGLGFARSQGVDFRKLQIREVGGVEYLFAIKKEKGQAAEQILPGLLLKVIHSLSFPKSMTWSYYQTRFARPIRWLLAIFGDKNVEFNIENIKSANYTYGHRFLSTGALPVTSIDDYFRVLREHYVILDQAERKTMIWQQVQKVAGAAGGKAMENEELLEEV
AFLVEFPTAFYGEFSPSYLAVPPEVLTTSMIEHQRYFPVYNNEGRLLPGFVGVRNGTDYCLDLVRAGNERVLKARLEDALFFWNEDSRKSLEEMSAKLKDVLFHERLGTLADKVLRLQKLALFIGQETGLSQPEKLQRSALLCKADLMSNMVYEFPELQGIMGRYYASGSAEEPEVAEAIFEHYLPRFAGDKLPSSAAGIVLSLTEKTDNLMGCFSIGIKPSGSQDPYALRRQALGLVNIILDKKLSIDLK
LVFEQAYLGYEDLVLENSREDSVKELLDFVYQRMRGVLLDSGVSYDVIDAALSRPSFDLFETYNRVLKLQEFKGSPVFEDFMVVYNRVHNLSRKWESEEIDPDVLLDESEKNLYRKLPELQQEIKTSLKAQDYTRALELLAALRADVDQFFTAVMVMVEDEKLKAARLGILKSIANLFNSMADFSKIV*
>Syntrophomonas_wolfei_subsp.metylbutyratica_JCM14075T_02326_Control_catabolite_protein_of_gluconeogenesis
VVNPIQLNDRQEKILEIVKKHEPITGENIAEMLNVTRAALRPDLAVLTMSGYLEAKPRVGYTFKSEGVESAMRRVLNQYRVKDIQSLPVIMKETCSIYDAIVTLFIEDTGSIFVVDENKYLSGIVSRKDFLKTTIGQADIHKVPVSVIMTRMPNIVTATLEETVVAAIKKIVEHEVDSLPVVKNRVEDEEEKLEVVGKISKTKNIARLFLDLAANKTKEV*
>Syntrophomonas_wolfei_subsp.metylbutyratica_JCM14075T_02327_Putative_pyruvate,_phosphate_dikinase_regulatory_protein
LNNHLPGVFIVSDSIGETAEMVVRAAASQFNSGSMEIRQVPNISDTETLEEIINQAAASNFIIAYTLVINELADFLQMEARKKGVICVDVLGPVIEALKSASDIEPRREPGLLRKIDEMYYRRVEAVEFAVRYDDGKDPRGVTQADIVLVGVSRTSKTPLSMYLAHKRIKVANVPLVPEVAPPEEIFSVEKGKVIGLIINPEQLNQIRTERLKTLGLKGQASYANLERILEEMEYAQEIMKRLGCPVIDVTNRAV
EETASKILEIYYRRLSHV*
>Syntrophomonas_wolfei_subsp.metylbutyratica_JCM14075T_02328_Pyruvate,_phosphate_dikinase
MSKKYVYLFEEGNSNMRQLLGGKGANLAEMTLIGLPVPAGFTITTEACNEYLASGQEFPDGVLEQVFTALERLESNSGKSFGEKNNPLLVSVRSGAAVSMPGMMDTILNLGLNDESVQGLASLTGDSRFAYDCYRRFIQMFSDVVLGVEHARFDEVMEKHKRKLALIFDYEIPAGELQNIIEEYKEIVQQEKGFAFPQDVREQLTMAIQAVFDSWNNQRAIVYRRLNKIDDELGTAVNVQCMAFG
NMGLDCGTGVAFTRNPSTGERELYGEFLVNAQGEDVVAGIRTPTPIDRLKEELPGVFQQFLDTCQKLETHYRDMQDIEFTVEKGKLYMLQTRSGKRTARASVKIAVEMVNEGLISVEEALLRVDAEQINQLLHRQIDQSVKLDAIAKGLPASPGAACGKVLFDADLAEKMGGAGEKVVLVRSETTPDDIHGIIYAQGVLTARGGMTSHAAVVARGMGKPCVCGCEAIKIDNEDRQFMVNGIVV
KEGDVITIDGASGNVMLGEVPMIEPTLSDEFALLLQWANQVKKLAIRANADTPQDAARAREFGAEGIGLCRTEHMFMAAERLPIVQQMILAENLEDRKEALSQLLPFQRDDFYGILKAMAPQPVTIRLLDPPLHEFLPHPETLLLEIAEMKHQQVKGKELLEKEELLKKIHSLSEANPMLGHRGCRLGLTYPEIYRMQARAIFEAMVQLQKEGIGCFTEVEIPLVMDMAELFLLKAEIMDVYEQVKK
ENNCELDFAVGTMLELPRACIMADEIAREAEFFSFGTNDLTQTTFGFSRDDAEGKFIPIYLEKKIIKDNPFAVLDRKGVGSLMRLAVEKARSVKKDILMGICGEHGGEPSSIEFCHIVGLDYVSCSPYRIPVARLAAAQASLLNK*
>Syntrophomonas_wolfei_subsp.metylbutyratica_JCM14075T_02329_hypothetical_protein
MSNQSPFIQINTRNDGWIKLLQPAVGKDLLEYLADMGKLLQSQMMDKILAAPAGTGFHIKRGSDILTEIRTWAENLPFEITVSSDKMKVFLTLNPRLPIKPRRQDLLAALNNMGINYGIDESSIDHAINNTGQTMVVAQGKYPVPGKNGSVQYLYHKPIIKPVLDESGQVDYYELGFIIPIEAGTLLAKRKAATEGAGINVFGEPIPAKAGRNYKFNVGKGIITTKDKAIAEFDGALAWINDKIVVT
KMLAIKGDIDFSIGNICFPGKVLIEGSVKDGFAVEAEDDIEVRGGIEATRVISRHGSVFVKNGIIGGGKAQVKAGKNVEARFIQEATIEAGQNIVINEYTVRSNLKAGDAVLIQGRRGIILGKNIITARTRIKASKVINCPALDLRVEGIERKQFYEQIKELNSRIDQQDTELKKIAEQIRYLRDKSSEQNSLQQLQELLPQYMDMNEELDCLYEERRSLVNMLKSTRGEGMIEIGGGLETGMTFGIKEDFIKLK
QQAKNLRMYYDPDKKRIIILD*
>Syntrophomonas_wolfei_subsp.metylbutyratica_JCM14075T_02330_hypothetical_protein
MKYIPASLRSGRVPINKTGVGSNPPNRCSTVLKLLMQFLPMLKILEIK*
>Syntrophomonas_wolfei_subsp.metylbutyratica_JCM14075T_02331_NAD(P)H_dehydrogenase_(quinone)
MSRPLIVGINGSPHKNGNTAFLLKEALDTCKKMGASTRILHCQQIMKDQKNPFCIACSSPCAGKCLEGKELARAFTMISQAEGLIVGSPVYFGTASAQLKAFWDKGRSLRADKKLLNVVGGAIAVGASRFGGQETTLRAIQDMMLVQGMIVVGDGFHEDDCGHMGGAGQRPANEDDNAINRSRILGKRIWEVAMATRDLRIR*
>Syntrophomonas_wolfei_subsp.metylbutyratica_JCM14075T_02332_Deoxyguanosinetriphosphate_triphosphohydrolase
MSIREEIARENAMLDKAASLSSKSRGREIEEEPDPVRTCFMLDRDRVVHSKSFRRLKHKTQVFIAPPGDHYRSRLTHTLEVNQIAKTIGAGLNLNLDLIEAMALAHDLGHTPFAHAGEQVLDQLLAGGFRHNENSIRVLTRVEQHKGRNGLNLSHEVLDGVLHHSGYGQSESRSYTLEGQTIRLSDKIAYVQHDIDDSIRAGLLRIEDIPADYLNILGYTHSQRIATLVTDTIKYSRQLILGGGHPQV
MPSPRIENALRGLRDFMFKEIYRGPVCQAERSRAIFIIELLFKYYCQHPEIMTPLYLEIADREGIERAVADYISGMSDAYCISLFQDIYIPQSLVPDAGKLLKVF*
>Syntrophomonas_wolfei_subsp.metylbutyratica_JCM14075T_02333_DNA_primase
LATYDDQVLEEILNRLDIVEIVSESVNLSRKGNRYWGLCPFHQEKTASFCVTPDKNMFYCFGCHAGGDIFSFVMKRDGLEFREALEYLAEKAGIKLIESRSGKQTDHRKPVIELNQAAVEFYQQVLAGKAGQEAREYLSRRGIEAETIRKYRLGYAPDGWTRLEEHLLKKGYSQEYLKLSGLIKRSENRNSFYDLLRRRLVFPITHYNGDIVGLGGRVLDDSLPKYLNTPETELFSKRKVLYGLFQARDSI
RQANEAIIVEGYMDCIKLQQLGINNVVATMGTALTSEQARLLKRYCEKVLLLYDGDEAGQRETIRALEIMGNEGLKMSVVSLPMGEDPDDYIEKHGKEELLRYIQNNKLSNIEFKINRYLSQKKELNLESQIGAINYLREDIGQLNSELEKDYYIKVLARKLKIEENIVYRELKKRERDISGIKRNKSKIIRDNIKYVNYGINERILAAMFKKPELFEYIKSRIGLEFLEEGDLRVLAGIYEKLKDNNHDGRLERL
GLLANQQGFGSAFARMALLMEEESLPGDMEIKRFIRQVERKEEELAWKNQFYRLKDLDSEGNFYSFLRFILNLNSFLNDIQEGGKR*
>Syntrophomonas_wolfei_subsp.metylbutyratica_JCM14075T_02334_Sigma-A
MKLDRKLAEAVVNLAEKGKKKKSLSYEEITEELQSFNLEADQIDDIFEHLQTLGIVIGSEQDAELDEENESIETEEVVIPDGIEIDDPVRMYLKEIGRVPLLIAAQEIDLAQRIEEGDEEAKRQLVEANLRLVVSIAKRYVGRGMLFLDLIQEGNLGLMKAVEKFDYRKGYKFSTYATWWIRQAITRAIADQARTIRIPVHMVETINKLSRVQRSLLQILGREPTPAEVAEEMDIAVDRVIEIMKVAQEPVSLE
TPIGEEDDSHLGDFITDEEAESPEESASFVLLREHLDGILNTLTEREEKVLRLRFGLDDGRPRTLEEVGQEFGVTRERIRQIEAKALRKLRHPSRSKKLKDYIE*
>Syntrophomonas_wolfei_subsp.metylbutyratica_JCM14075T_02339_hypothetical_protein
MEKVIAVEDNLTPIKEYLEARGCLVVDIGSGWNIPVDAVVISGLDKNLMGMQDIVTVAPVIEARGKTPEEVWDSISR*
>Syntrophomonas_wolfei_subsp.metylbutyratica_JCM14075T_02340_hypothetical_protein
MKIIVDADACPRPVLQSCLELGCKYDFPVWTVASFNHHIDSDHHIVVGNASQEADLKVINLTQRGDIVITQDWGLAAMVLGKGARCLSPIGREYRNESMDFLLEEREIKAKLRRAGGRTKGPRKRSVEDNLRFQRSLENLLLTIPPHPHP*
>Syntrophomonas_wolfei_subsp.metylbutyratica_JCM14075T_02341_Cyclic_di-GMP_phosphodiesterase_response_regulator_RpfG
MKRVFAEELESGMVIARTVGADGRALLTENTRLNEAYIQRLQQLGISSVYIKDGLADIEIPEIISAQVLTAVSSNLNSIFKTFSSRKTLDINQLRKMISLLLDDIVTNRNVIIHLEDIRAYDDHLLFHSINVAVLSMMTGLSMEYPEAKLVDLGLGALLHDIGMIMIDSAIFNSARERELTAEEREEIQKHAEIGFNILRTYREVPTKVAHIAYQHHERFDGSGYPRKLDKKQILENASIVAVADVFDAVVSDRPF
RRGYSSTEAMMIIRRLVNTYFDSEVVEAFATNIAMYPIGCLVSLNTGHVAVVTSVSRINSLSPVVYVTLDHELNFIEKPFQLDLQEIRGFKIQRRLSYEETEVVRAKIAGQKIECQNKSPKINSEKSYAVL*
>Syntrophomonas_wolfei_subsp.metylbutyratica_JCM14075T_02342_Cyclic_di-GMP_phosphodiesterase_response_regulator_RpfG
MKRVSTDELEPGMVIARTVIGSDGRALLTENSQLSESYIERLQSLGFKSVYIKDGLADIDIPQVISNQVLSAVSSTLSNSIKTYSTKKSLDLKSLRMVVTMLLDDILSNHNLLIQLEDIRAHDDYLFFHSINVAIFSMMTAITVGYTEGKVIDIGLGALLHDIGMIMIDPAIINKSGQLTESESAQIRKHPEIGFNILRTYQEVPTKVAHIAFQHHERIDGSGYPRMLERKQILEYALIVAVADTFDAVVSDRPFRK
GYSTTEALIILRKLSNSYYDPEIVEAFASNIAIYPVGCLLSLNTGHIALVTSVTRINSTRPTVGNILR*
>Syntrophomonas_wolfei_subsp.metylbutyratica_JCM14075T_02343_thiamine_biosynthesis_protein_ThiI
VKAVCLFSGGLDSQLAAALVKKQGVTVIGVNFSSPFFGANRATHMAAKELGIQLFVLDTGKEYIEEVLKNPVYGYGKNFNPCIDCHGYMLRKAGEFMQKLDASFLISGEVLGQRPMSQNRSALNAVDKLSGFRGLVLRPLSARLLPATIPETEGWVDREQLMDISGRGRTRQIELAEKLGIYDYPTPAGGCLLTEASFSRRLKKLLEVKPELEAGEMEILKLGRHFYLGERTLLAVGRNHSENENIQKI
ARGNDYLFKVLDRPGPLGLLRGDDDPQADEINLAAAIVARYSDARKENMAQVKIFKPGHEAFETINITPLPPDEVPASL*
>Syntrophomonas_wolfei_subsp.metylbutyratica_JCM14075T_02344_Arylesterase_precursor
MNHSLRIVCLGDSITYGFPWGPQASWVTMLDEVLDGEVINRGINGNTTANMLARFERAVLKYNPSHVIIMGGTNDVICGESFDRITWNIREMVERAEKAGIKVVLGIPTAVDEPYWEKLLIRLRSWIKDLAKQEDLQIIDFSRPFYDDKGRVRSDLLLADGGHPSRAGYQAMFAQIDLKIFTD*
>Syntrophomonas_wolfei_subsp.metylbutyratica_JCM14075T_02345_hypothetical_protein
MQKTSSTTKLVIFLVVLGLISYLASLYVDWLWFKSVNFQSVFSTILLNKIGLYVFVFLATALLFAFNLRLTRSHMGPVKEPVYDDEEGRVIYLDDEKTPWKDFLRGKSSRWLYLGISLFAAYLVSSVAVDNWIVVQQYINRVPFGTTDPIFSKDLGFYFFNLTFYKFAYGTLMMALILLFITLGSIYLINASTDLLFGNWRQFSFAKGHLATLLAAIFLLKAWGYKLAAYDILFSPAGIVYGATYSDIFARLLSY
RILLLSIIIAAVILANIFVKKFKWILISLGTWVLVAFLMGSVYPSLIQKLVVQPNEFNKEKPYIENAIKYTRQAYSLDRAENREFNIDYNLDISDPSHQSTIDNIRLWDWQPLRTTYKNLQQLRSYYVFDDVDVDRYTVDGKYRQLMLSVREIDQEELPQQAKTWINQRLMYTHGYGLVVSPVTEVAEEGFPQFFVKDIPPRFSTDLKIKRPEIYFGEKTDNYVIVNTEQKEFDYPAGEKNVYTRYEGKDG
IKIKSFARRLLFGWVLKDYKMLLSSDISNESQVLMNRNILERVQKVAPYLSYDGDPYVVINDDGKLYWILDAYTVSDKYPYSEPFDKQRNNYLRNSVKVICDAYTGKMNFYIADESDPIIKTYSRIFPQVYQPLTAMPESLRSHLRYPVDMFSIQAQMYRTFHMTDPSVFYNKEDPWLIPNETVEDKQVKMDPYYLVMRLPEEEKPEYILMMPFTPKSRANMVGWMCARMDGDNYGKMLVYNFPK
QETIYGPEQIESRINQNTEIAQQLSLWDQRGSRVYRGNLLVIPMGNSVLYVEPLYLQAENNRLPELKRVIAAFENKVVMEPSLDTALLKLFGQDQRVESQRTGVVLTPGELPGATVAELAKSAREYYDRANQLLRDGDWAGYGENIKKLNETISRLEEAVKP*
>Syntrophomonas_wolfei_subsp.metylbutyratica_JCM14075T_02346_S-methyl-5'-thioinosine_phosphorylase
VEIPGCELAFIGGSSSLSIEVPESLGLDYVQVLEKSLVFSTPYGLSPEFKLMNIQGEQEERKVLSCRMHGWRSGVSRADASRQVFWVLQQAGVKRVLAEGGVGAINHLLKPRDVIIPHDYMDFSLRKDVGLEERHLLVMREALCRVMRKQLLELLEKSWKFRVFDRGVYVNTDGRHFESPAEVAFFKMAGGDIVGQSICPEVYLAREIGACYVGLYLVVNYAEGIVSPWEHEELAEIFYGESQTLARMV
FEFFRTLPPGRECNCAELRKETLLKEVYK*
>Syntrophomonas_wolfei_subsp.metylbutyratica_JCM14075T_02347_FO_synthase_subunit_2
VPITIRQRLKEIMKGRRMSDEDFQLLYEQGELLEIGQAANRKREERFDNNQATFVIDRNINYSNICSCKCRFCAFYRDKEDADAYVISPESLFAKIDEAMELGATQLMIQGGLNDDLDIRYFEEMFSSIKARYDICIHSLTAPEIAFIARTSGLNIRETLMRLKAAGLDSLPGGGAEVLHDEVRQKISPKKISSSQWLEVMKTAHEIGMETTATMMMGSVDQLEHRVAHLRLIRDLQDETGGFRAFIPWT
YQPGNNELMGRKMYSAQYLKFLALSRLYLDNFAHIQGSWVTQGDRVGQISLLFGADDLGSIMIEENVVRSAGLEYKMKKEEMIELIRGAGRIPALRDTEYRILEVY*
>Syntrophomonas_wolfei_subsp.metylbutyratica_JCM14075T_02348_putative_periplasmic_solute-binding_protein
MRPRVGHIQFINCFPLFYGLIEKKFLLEIDLIKGNPSDLNRMLQENLLDLAPIPSIAYARNYRDYVLMPDISVSCDGEVKSIFFFSKVPINELHQKKVALTNTSATSQALLRVILARYYNIMPEYFSSAPELGAMLMEADAALLIGDDALRARYKNQDRLYIYDLGEEWKKFTGLPMVFALWAIKKDFASKNVEQLRIIKETFTESIKFSLENVLDVAKKASQWEDFGADYLVDYFRCLRFDFDSGKQEGLM
EYYRQAKNQGLLDEVPPLEILDI*
>Syntrophomonas_wolfei_subsp.metylbutyratica_JCM14075T_02349_FO_synthase_subunit_2
MDNRLEEIKNKVNAGERLSREDGIYLYQSNDLLAIGEMARNKKLSVSGRRVYFNINRHINLTNICVSRCRFCAFGVDEDGNSGPYLMSPDEAFAYGAEAVDYGITEFHVVSAMHPHMGLDYYIEVISRLHKAYPDIHIQAFTAVEIFHFSKISGLPVREVLQQLKDAGLGSIPGGGAEILNDDIRQELCPRKASSDEWLNVHRIAHQMGIKTNCTMLYGHIEEIEDRVEHLIRLRDLQDEAPGFQAFIPLP
FLPENTKISHLKRTGAVDDLKTIAISRLMLDNMEHIKAFWIMLGLPVAQIALEFGADDIDGTVVEERIMHAAGASTEKGISKEELVHLIKESGYIPTERDTLYSIIREF*
>Syntrophomonas_wolfei_subsp.metylbutyratica_JCM14075T_02350_D-alanine--D-alanine_ligase_B
VNNVLVLMGGTSEEREVSLRSGKAVYEGLREAGYEVEALDFTPETIHRIKEISPDVVFITLHGKNGEDGTVQGYLELLGIPYTGSGVLASAVCMNKVITKKLCSFEGLPTADFQVIKKRGFNRDSFNPALLMEDFGLPLVVKPATQGSSVGTSIVRESKNIVPALELAFSLDEEILVEKFIAGTELTVTVLGKDSLKTLPVIEIVPKNEYYDYESKYMPGMSDHIIPARISEPEREKVEEISCKAYEAVGCRGYA
RIDLILDREGNPYILEINTLPGMTGTSLVPDAARAAGIKFPELLDFFVKMALEK*
>Syntrophomonas_wolfei_subsp.metylbutyratica_JCM14075T_02352_MscS_family_inner_membrane_protein_YnaI
MKSLSNYLMEQLLTFISQPALKEILIAVLVFLIFWLLGKLFYRKVLSWLLQMSNKSKSKWDETIFTALGKPMQVLIILIGLYLALDILLNSVSQEEMLLKLFRSSLVATLGWALYRLSASSSILSQEARERWNIDSILIPLLSKVLHFLIIALVIIIIAGEWGFDVNGFIAGLGLGGLALALAAKDMLANLFGGIVIILEKPFIIGDRISIPGVEGTVESISFRSTGIRTSEQSLVTVPNSALATQAITNYSRMGKRKISINLG
ISYHTSKAQIESVINKLKQMLTEHPAVDPETILVNFESFGESSLNIFLYFFTKTTVWAEYLLIKEELNLKIIEILEDEGVEIPFPSRNLYLLKTEETKS*
>Syntrophomonas_wolfei_subsp.metylbutyratica_JCM14075T_02353_hypothetical_protein
LKENKLLFVQILLAAIILCFSFWALLATGNALYFMAILLSLAAIVFIVYILYELRNEQKPIYSKDYYFYFIDRQNSADESFFFPFREMRR*
>Syntrophomonas_wolfei_subsp.metylbutyratica_JCM14075T_02354_Elongation_factor_G
MKVYPSSKIRNIAFVGHGGTGKTSLAEAMIYNTGATKRLGKVDDGNTVADFYPEEIKKKITISTALVPCEYKDHKINILDTPGYSDFYFEVAGTMRVADSMLVVLSATAGVEVQTEIVWEDYPTVPKLAFINKMDRENADFYKVLDDMKNSFTDNIVPVQIPIGAAESFKGIVDLLKMKALIFEAGSGKISEEEIPADMMDEVESFKETLIEAAAEANDDLLGKYLEGEELSDAEIMDGIKEAASNGSAV
FVFCGSALNNMGVSPLMDFIVNCGAAPDKNPLVAGKDMENEANLAQVFKTIADPYIGRLTMFRLFTGKLKADSVVYNANKEKEEKIAQLLIMTGKEQASVPEIYAGDIAAVAKLSVTSTGDSLTLKAQPVIMDPIEFPEPTLGIAIEPRSKGDEDKLSGALQRLLEEDPTIRVEKNAETRQTILRAMGDTHVDIILEKLSRKFGVDVISREMRIPYRETIKTSVEAEGKHKKQSGGHGQYGHVFVKFEPL
YEGEFEFAENVFGGSVPRQYFPAVEKGLIEAMEEGVLAGYPVTNIKATLYDGSYHSVDSSEMAFKMAARIAFRKGVESAKPVLLEPIMNVQIEVPEAYMGDIIGDLNSRRGRVQGMEPAGKKQLIKAQVPLAEMARYTIDLKSMTQGRGKFKMEFSNYEEVPGQNAEKIIEKAKQEKEEKEK*
>Syntrophomonas_wolfei_subsp.metylbutyratica_JCM14075T_02355_hypothetical_protein
MIFFSLILNTAIFFIVLNFSYIKKKRENPAYPDKPVSQLILFPLALGVVFTLIVDVFRGFMLYQLLIFLLAALLLYWIFYVLKKS*
>Syntrophomonas_wolfei_subsp.metylbutyratica_JCM14075T_02356_5'-nucleotidase_SurE
MRILLTNDDGIHARGIQALIGELGSIAELFVVAPDRERSGTGHSITVFDPIKVIKAKLAGIKAGWVIGGTPVDCVKLASSKLVGDNIDLVVSGINHGPNLGTDVLYSGTVSAAVEGVIMGSPSIAVSLNSFAADTDFSFAARFTRRVIQNLFKNGMEKKTLLNINIPYLCPQDIKGIRITRLGVRNYENLFEERQDPRGNTYFWMGGGVLEEPQEEDSDVNAVQHSYISITPIHFDLTDYRLVEQYRKSFSQ
FSHLLGDANDIF*
>Syntrophomonas_wolfei_subsp.metylbutyratica_JCM14075T_02357_Signal_peptidase_I_P
LSKELRDFIKETISIIVIAFILSLILRAFVIEGREIPSGSMLQTLQIDDRVMVNKFIYHFKEPVRGDIVIFDPPEELNSSKYFIKRVIGLPGEKVEMKEGRVFINDKPLSEPYLPEEINYQFGPVVVPSDALLVLGDNRNFSFDSHMWNTWLTRDRIKGKAFMIYWPLSHFCLLEREVSFE*
>Syntrophomonas_wolfei_subsp.metylbutyratica_JCM14075T_02358_hypothetical_protein
LQQDKGKLDIIASKAFSASPELVRIVDFLNKSLKGKKIMFGLSKNKEKEQMTISIYEFE*
>Syntrophomonas_wolfei_subsp.metylbutyratica_JCM14075T_02359_Succinyl-CoA:coenzyme_A_transferase
VGKTYLDLYKEKLRTADEAVKIVKSGDWVEYGEFMMQAKDLDAALARRKDELKDVKVRVVTATFMPEIVKCDPERDHFIYNDWHFSGGSRQLAMKDLCNYIPLTYHEAGRFVEDFAHIDVAFIPVAPMDKHGFFNLGTSNSISSYVINKAKIKIVEVNTSIPVCFGGSKEAVHISDIDYIVESQTNPKLLSLPELPVTDVDRKIASLVMEEMEDGSCIQLGIGAMPNTVGTMIAQSDLKDLGVHTEM
LVDAYVDMYDAGRVNNKRKSIDKGKMVYTFAMGSEKLYNFMDRNPAIASYPVGYTNDPAVIGQNDKVVGINNALEVDLFGQVASESAGWRHISGTGGQFDFILGSYRSKGGKGFICLSSTTTLKDGSRVSRIRPTLSPGTIVTVPRSCVYLVTTEYGTVNLKARSTWERAEALINIAHPDFRDELVKEAQKMRIWTKTCKINP*
>Syntrophomonas_wolfei_subsp.metylbutyratica_JCM14075T_02360_hypothetical_protein
LCPTGRYAWATPTACLAAQGVPRQSPVHGRWRSRDILSLAPFRLLVFAGATLIVTSEGWNIRSTRRVKHPCFANARFAYLCNPMQQKGFFAVESLMNYRNNKSRCHDRHLLFNSRINLFYGLILQVLVQIRIF*
>Syntrophomonas_wolfei_subsp.metylbutyratica_JCM14075T_02361_ATP-dependent_helicase/nuclease_subunit_A
MAHWTIEQEEAINARNSNLLVAAAAGSGKTTVLVERIIQLVLRDRIDIDRMLIVTFTQAAAGEMRERINAAFFKELEKGREDGHLRRQLYLLNRSSISTIHAFCSDVVRQHFHLVNIDPHFRIADSTETELIKMEALEELLDGEYEKGNDGFGDLVEVFGGNKDDKPLEALILRLHSFIQSHPQPFSWLEEKIDNLALGEDNWAENPWASELSQQIKIELSAAQDILNQALKLCNKADGPWGYLEAIEND
QKWVALLLKAADQGLPALYSCLQQLSFTRLGRVYRDVDENLKNQVKILRDESKKILNAINSLLGRDPLEYLQDLNEIYPIMKYLGEIIQSFAEIYQEKKREKGIVDFNDLEHLALQILSNEAVAREYRDYYSYIFIDEYQDSNLVQETILNYIKKEDNLFMVGDVKQSIYRFRLADPSLFLEKQNSYPLQDGSVNRRVDLNKNFRSHPEILNAVNYLFRHLMSQELGEIDYDEKSLYLYPGLNTKLKYEEIDDP
SSQEGKEKENTASRVEIYLIENNPDLIARLEENETEEREIPGEAENFIERIIEMDNTEMEALLIAQKIRQLLEKDIYDPKLQCRRKIEYRDMVILLRATRNSAGIFMEQLSSEGIPVYADASSGYFDTIELNLFINLLRLIDNKRQDIALLSVMRSPIGGFSIEDFIKIRTRLIPRREKQSCSFYEAMEIYMEENNDDLKDRLVFFIQRLKEWELESRIMALDEFMGKLLMDTAYYYYAGAMPGGVQRQANLRILLH
RAREFQKSSFKGLFSFIKYIEKIKSSGSDMGNACIIGENDNVVRIMSIHKSKGLEFPVVILGGLGKNFNIRDSNENVLLHRKLGIGPRYINTQLRTYHDTIARLAIKNRIKLENLAEEMRILYVACTRPQEKLIMVGTTRQLESAWRRWSQAVNSYNQSRARSFLDWLIPIIMRHKEEGQYLREIAGGDWDREILWEDESRWKVQLISPWQLSAEEKRKKEEKHEWERLLEQGGYPCPSAENEEIRKRLNWK
YPYQEAEKIPAKISVSQVSQISSGYLEDGADTFLTRVPAFLSGEEGQKTKFSPVDKGIIVHLVMQNIDYRRVSREDSIKQQLGEMVEREILTPEQLAVVEVNKIWRFFQAKLGQRVLQAKRLFREAPFNLLTPANEVFPATESQEELLIQGIIDLYFYEGEDIVLLDFKSDIVHHKSEEEILAPYQVQLKLYKKALERITGHRVKESYLYFFDLNRAIRV*
>Syntrophomonas_wolfei_subsp.metylbutyratica_JCM14075T_02362_Poly-beta-hydroxybutyrate_polymerase
MNWMEEIDRYSRYNVFLKILQSKEYISLFDPRLKRYMDILIELEKPFTNWSTPNNIILVHNTLCLRHFPNKYESSAKRPVLILPPQAGHHSNLADYSPAQSLVRVFHRYGYDVYVTQWLSATTEYKDLGIEDYIRLTDEAVEEVRKRTGVYKIHLVGQCQGGWQASIYTSLFPDKISTLVSAAAPIDVNAKSSPIVEYAQKLSIDVFRFMVNSGGGVMQGKYILQGFKSMQPEEHYVRKYFRLWQMIKEG
DEDGIKRFIHFENWYQYTQNLPGRFYLEIVKNIFKENNLTKPDSFQLDGRPVDLRNISCPVIIMAGKKDHITPPEQAFALKNYISTPAADVIEILTEGGHIGTLMGTESLREDWTAVNEVLKLAI*
>Syntrophomonas_wolfei_subsp.metylbutyratica_JCM14075T_02363_Long-chain-fatty-acid--CoA_ligase
LNHNDFEAMHLNIIESLKKPQALSLGWQLENWAAQQGEKTALIYGDRYISYEQFNQMANRYAHFFQQEGFKKGDVVSLLMDNRPEYLMAASGLNKLGVIVNLVNTVIRGERLAHAINVSESRALIVGHEFLELYQSVSNGIRLRTPGRILVETGEQNITLPPAAEDLNQLLSGCPAHNPESTGKSSSEDIIIYMETAGSSGLRKTVMLSQKRWLLMGQQFTMLTNMNQNSIIYLVIPFFYNMGFNICF
SSMLAAGASMVIKPRFSLSNFWPDIRRYKVTHFMAVGEMLRYLCNQPEEADDLDNPLEYIVGNVTRGDLLQQLQQRFGIKKIVEAYGTSEGVGTYINEDEIPGMCGNLNLRAMRQGEVVKYDYDSERITRDDKGLAVICKPGEIGLVLSEINANNPFGGYVNDSEMSEARIVRDVLQIGDEYFNTGDLVKLHDGDYISFVDRLGDTYRWKSKTVSANQVADVINKFFGSIEEAFVYGVKVPGMEG
NCGMAALQLLDDVPLDWDNLVDHINRRMPDHARPVFIRICAHVDPRLFRKKRRQLQEEGFNPTLVRDPLYYFDLKRNAYLTLTTEKYQDIKDGKIRL*
>Syntrophomonas_wolfei_subsp.metylbutyratica_JCM14075T_02364_hypothetical_protein
MKPAKHDKIVSFSGISDSSFKGFKVIIDLFTDELYLRAPAAKLSGISYKFDSLTMLRLDRKEKGHQDILDINIVYPDGEQTETVIINREDAYRVLIRYKFFKDGASELRKRKTEFEEISELKNVALIEFTRNRDVRNYNYGTLLLAQKEMNFISYNGKNFILHLDELDSGYYYHTVVLPTDILGYSRWISVHRVSDLKRESWEIVTFMSNEAQAGQVEKLFEEHYAKYLENKMKSVQQLWQVFNQQIEEHYQ
ALKTDSEVKELAGKFIKEKGEAVLLNPDSYWDLEYLMEKKRSNAYVKILAPYEEEIEVSPQVKTFFEDSFLPFYESLVQKPEIKNEVAFLAVWKLLKASSLECYGDMFSAQFPNFSEQDSDWQACVNNFYSLFKPASPAEKDLALFSYFMMANYWKNEDNFTYYYQIIKESQNNA*
>Syntrophomonas_wolfei_subsp.metylbutyratica_JCM14075T_02365_ATP-dependent_helicase/deoxyribonuclease_subunit_B
MAIRYILGRAGKGKSHLVYREMETALRSDKEQALILLVPEQYTLQAERDVISQLRLPGIMRLEVLSFTRLAQNVFSRVGGLTRVFLNEQGKIMVLRRIVDECGPELGLYKKAIRQEGFIKQFSQLMADFKQRNIRPEDLQLEAAFEEIPDQIIKQKLKDISIIYQRFNQYMEGRYIDSEDYMELLMEKLEHYTLLKNARVWIDGFTTFAPQTLRIIEKIMVMAYDTTICFTLDPDTKKADLDLFQLPARSYR
QIRDMVRKNNLQEELIWVDSRPAEMLKSPEINHLEAELFAYPGRIFAKKPQNIELFAATNINSEVEALAAELLLLARQGYRWKDMAVVCHDMDRYGSLIRRTFRDYGIPCFMDRKRDIMSHPIIELILAALEILSRGYRYEDVFAYLKSGFSDLSVDEVEILENYSWEYGIKGGKWKEDFSYGDAESLIELNQKRLLFIKPLEDLGVMLKGNNTIREMAQALFVFLERENIYSKLEKWTNDLMARGFLEEAS
ESSQLWNAMIEILKQMAEIMGEQKVNLKEFKRILEAGFLSLEIKIIPTTIDQVLLGDIQRSKSHDIKCLYVIGVNDGILPSLGREEGILSETEKEALQEKGLDLGYEREMNVEEERFLIYTALAKPAERIYFSYALADEEGRALRPSLLIDQLRMIFPSLSLKTNLLEDRQWQLQMIATPESSFKHMVEKLRLGLDGKELEDIWWDVYRWYFEQERWQGSRELLLQGLFHHNQPEPLEPQLSRRLFGTPLRSS
VSRLEQFVSCPFAHFVRYGLQPQERKNYELNSPDLGLLFHDSLLRFSHKLRENKQKWPELSKEDCHAIIDAVVDEIAPSFGQGIFNSHPRYQYLLNRLKRISRRAVWILTEHIQQGDFKPWDYEVAFGRGGMLPAVEIDLENGEKIILEGRIDRVDLLELEESCYVRILDYKSGQQDFHLSEVFYGLSLQLMVYLKALLTAWEQMGKKPAVPAGVFYFKIDDPMINSEEKIVEVVEKEIAKKLKLKGLVLA
DAKIVRQMDREVNGYSTVLPLGLSTKDEFYRFSSVLPLKEFLALLKHVERLLKRIGQEMVNGQIRIEPVYTRRRLACTYCSYPSICQFDRLFSDNNWRYLKPMSREEVLTYLMEENNQG*
>Syntrophomonas_wolfei_subsp.metylbutyratica_JCM14075T_02366_hypothetical_protein
VNTGLWLLLIGVLLVGLAYFSYKRAESSLLTLKKEDLVSYYLELALRLLPFPFWTLLLGILLILAGLVVLLVNLSRGFFY*
>Syntrophomonas_wolfei_subsp.metylbutyratica_JCM14075T_02367_hypothetical_protein
LAKWLYLSLAALSGAAMALQGTFNAALGKVVGIWESTLIVHLVGTMTVLLIMLGLSTGFSNLGKLNSVPWYAFLGGFLNVLIIYALVRAIPQIGVGNATTAIIITQVLTAVCIDNLGAFGMKKYEFHYLDLLGVALLAAGARILLME*
>Syntrophomonas_wolfei_subsp.metylbutyratica_JCM14075T_02368_Zinc_transporter_ZupT
VIAVKALYLMSILAGGCTLLGSMALFAKRKWSNSSLGLFLGLAAGVMIAVVCMDMLPSALLVNAIEAGLGFVVGLLVLALLHIMLLSRQAENEGLLRLGYLIMLGIALHDLPEGMAIALGSEMKVRTGMVIALAIGIHNIPEGMAIAAPLLMGGMKRLKIFLRVFLVSLITPLGTLTGQGLVKIVPEVLAFLLGLASGVMVFLVLFYLWPQAGSKDKKSRVQGFLLGLLIIAAATFL*
>Syntrophomonas_wolfei_subsp.metylbutyratica_JCM14075T_02369_putative_membrane_protein
MNRILSVELRAITKAVLFSLVLGLVSAFVIYFSSLSEELLPALGKGILILSIFLAACQVSKHYGSKGLIRGLSIGMVFFIIMLIATFIFNPSLIALKTFVYTLLLCLLAGGLGGILGIGLNGN*
>Syntrophomonas_wolfei_subsp.metylbutyratica_JCM14075T_02370_hypothetical_protein
MPLYPLDEIKKYFEPSGPMEQLLTDYRFRQEQVNLAEAVSAALSDQEFLLAEAGTGVGKTFAYLLPAVLWSLQENEKVVIATRTKALQQQLIERDLPDVKRVIKADFTCAEAKGRENFLCWNKYINILAGKKRLQEGEIEFLQPVLPWAEESETGDRKELRLSSALMKHWDILAADRKSCRKEKCLYHEKCFRLKMIKRLEKSQIIITNHALLLSDVQVDNRLLPEYHHLVIDEAHSLDREAFDKLSMR
FSFGDSMELLEYLQKAYLPHIRELSFADRELLEECLTHISMVVKLLKEYFSAIRNSKNDYEATLVIKAADLEKTWFEVLEAELIEHRDWQDSINLLLRLQKKLEEGMGGVEESEELSRIIISLQEFADHAFQINEEHSFQSDMLVWLDCERGKVKAVSSSAVCIAGELQNRLYHKLESLVLVSATLAVEERFDYFLNKFGLSLQLQEEERLKTLLEKSPFDYEKQAGYILLDDMPARAEPDYQEKLAGILSQIIDI
TGGRTLILFTSRQQLKDIAGILRPYCEQRQVKLLVQNEDGGFETLREQYTAHPRSVIMGLDTFWEGIDLKGDLLKCLVIIKLPFRSPDEPFCQAWEKYYQQLNQSGFSRFLLPDAVIRLKQGVGRLIRGENDRGAIVILDARLGKSSYSKVFLNSLPFKNPRVLSSRQFTQELKKWV*
>Syntrophomonas_wolfei_subsp.metylbutyratica_JCM14075T_02371_hypothetical_protein
LSNELLRSNNYALLTFASTSHALKAEKLLKSRQADFLVIPTLREISTSCGLSIKIAPTNLPDYYNYLSSKKVHIEGTYQVAKEGKKNRVEQIQVFKAE*
>Syntrophomonas_wolfei_subsp.metylbutyratica_JCM14075T_02372_Acyl-coenzyme_A_thioesterase_PaaI
MQNQGIDEELFRYIADSIGDTPYYKLLGIQVIKIGPGLAELSVKVDLKHTNPLGVTHGGLMMSLADAAMGNAIRSLGIKAVTVDCSTGFIASAQQGETVIARGEVLRAGKNMLFAQAEVRAGNRLLSNSKASYFKTGEMDLNN*
>Syntrophomonas_wolfei_subsp.metylbutyratica_JCM14075T_02373_hypothetical_protein
MLNNNKRMLIITALLIFIVMIITIVYANINGNKSSPQKPQSKTPIQQKDNSPAEKWYVQFTAKQQTTAYTEEINAPRPANGQYYFTGGIAVHPRYPGYDPRVPIIPFGTVVYPSPPLEINGNKYHSLVVMDTGDIHYGLWGNYPYWFDVYFGNTEYYNIKNAKKYGAKKHDYHWYEEWK*
>Syntrophomonas_wolfei_subsp.metylbutyratica_JCM14075T_02374_Adenosylhomocysteinase
MNALLKTDASSLSIIKDCSLAPDGFKKLEWVSNNMPVLNKLREEFAEKKPLAGKTIACCLHLEAKTGYLLQTLQVAGARVIACASNPLSTQDDVVAALVESGITVFALHGESPEEYQRYLELTLDTCPDALIDDGADMVTTLIKQRPEQAQKLIGGCEETTTGIIRLKSMEKDKALPFPMMAVNDAYMKYLFDNRYGTGQSVWDGINRSTNLVVAGKNVVVAGYGWCGKGVSMRALGMGARIII
TEIDPIKANEAIMDGFEVMPMSTAATLGDIFITVTGNINVIRKEHIAVMKDKAIMANAGHFDVEINKNDLAALAVSRRIVRNNIEEFKLADGRRLYLLAEGRLVNLAAGDGHPAEVMDLSFSLQALSLLYVIENRSRLENKVYNVPDSIDQRVACLRLEAEGVRIDELSSEQEKYLASWDPY*
>Syntrophomonas_wolfei_subsp.metylbutyratica_JCM14075T_02375_stage_II_sporulation_protein_P
MDTITKRYIWLFFAVLMVLMVGAYVIHTAYSPQSKPQPGNDLNSQRASYVTIRDSEGKVILETGIPVYEQDEYISEDNLHYVIISISGSNAVARKKDIDKSELNSVSQKLNAASITYPHTVEAQALLNPHHVVIYHTHSDESYLPDSGRPGKPGNGDIFQVGAALAESLTKAGISVHHSYNPHDPHDINAYHRSRRTVTQLLKERPDASFDIHRDSAPLSSYATTINGIDTARVMIVIGRSNPNLNPNLNFA
RSIKAAADDLHPGLMRGIYLGQGDYNQDLYPTSLIFEVGTESNSLDAAQKAVRYLADAIIAVIGS*
>Syntrophomonas_wolfei_subsp.metylbutyratica_JCM14075T_02376_hypothetical_protein
MTLTQLALAAILTEKLWESLKLIWEKKKLSIDRVGALVLGITIALGTGIDLFATVGLPINIPYLGLILTGIIISRGANFIHDLFKITEEVKVKRKAENGKFKNLSLQ*
>Syntrophomonas_wolfei_subsp.metylbutyratica_JCM14075T_02377_Spectinomycin_tetracycline_efflux_pump
MKLEEKWRVLIIICIGIFMSTVDGSILNIANPTIARELSVSMREIQWVVTAYMLVITTSLLFFGKLGDMLGSSKIYSYGFLIFTIGSLLCSLSPSLAYLVAARVIQAIGASMMMATGIGIVSNAFPPGERGKALGITGSIVGVGNMTGPSLGGILIANFTWPVIFLINVPIGILAFYLGMRYLPQQAKSLAPKKHDLGGLLLFALSTIILLLSFSGSHGIEWKLFSLGLLIMLLFYLYEKRISDAILDLELFKIKNFSHGNL
LAFAAYSAQTSVFFLMPFFLENLLHYPPAFSGLIMTIPPVTMAITAPLAGALSDRIGNRRITPLSFLFMTSAYLLLSTLEQESGLLKVAGSLLLMGVGMGSFGSPNSSSILGSIPREKAGYAGGFMATVRNFSFSLGVAASVSAFAYFLSLYQQKTSYIAAYAGAAHSVYRIAAIICILAFLSLLEREPARSTESPE*
>Syntrophomonas_wolfei_subsp.metylbutyratica_JCM14075T_02379_Hom_end-associated_Hint
MQDYFASLYKTTQAHNLQRNRSMPLNFAREEDYDCFCAMCGGVDHFQQEYPQLYALAVNTRQHYVDHYRDSSAAKGFVDSCMVNDITYDQAGQKLTSFGTVCLTQFALRMFVTLTLYDGNNIIGCNTAFGNGHGHKNLTITCTVEQVNLPNHNIKAILHATWEFANSNLLNSYITESTLLTSVAEVIDHIDITDPVHKKSGPADDIGVCYARTPIAAEKIDYSYPEARTPERHQKLYLDNRGQVV
LAGGYTFDRVKPNSVGIILNCEGFGGLFYNGDAPQFQAQAGGFNWTFNQYWNNYITESVIAGNRAYKFDMEAIFYVHEDNQEHLLKVSSYPISQPSPHYKQISKIHLYWGCLAEDTLVRMADGIARKIQEVRIGDQVMANTQGMPATVTNVWSGTDETLYCLRADNDAEIYATRDHPFISRNGSKPLILLNDRDELLMEDGSYSRIKYAYPLAFGKRIFNLDLSGEAHTMICNGFVVGDNYAQGL
AIDYLNQIENIDVPAEVIAESEKLSRNWQAAIKEVLNHEKY*
>Syntrophomonas_wolfei_subsp.metylbutyratica_JCM14075T_02380_hypothetical_protein
MKNINRKNINGLRTYNDSYMQHNPIPDFTLGGSRPFTLQVFFSLLSDSSRGVLYRQEGVFELGIEEGMIYFNAPGFCSLKTDYPNEVKLVPQYFYYAAISYDGTDTALYLQGYPVLKQTKTGTAAVNNNNNYDIGCGHNGYVTAIQVHDQVLSPEQILAAAANPLTSSEHCVAWLDFSQRQPLDRSKSQLNIWIAGDNTKHRSAIVNLVACTAWDGSGCALLTNNLSTEPTAYSIIGKVFPQRTQLA
QICLYSNRRDDRSSGLEILLQKTASNNFKLVFANGTASIVSERELLPDQWGDFAVTVAHNHVTLYVDGTADNSGTATRPLAGRETAVIGARTIDGRPDYTVGYKGYIDYMAEFDEALSPEKINSYIDNPPFIFDPHIVSNMFMAQQYPQEIVYNKPIALVRNVRFVLAENTNPLDCEKGVACFIPTEEDPLWGTLTPEQQWQMETDSKTSEEVTHSVYGVQAAGSAGMWMLGSTGLRRRRIRGVE
YQEVPMEEVAEARPVQIAAQTGRAGAMSFFSCGGITAGFGTLLQSPVFWGVVAASATVAVVAALIIAQDKKRPAADGELQIVSLKFDHDADPSLGSIHFRSNQSNAPVSMEFSKGSNEQINMSGVFIPALLTNPRLTITVKFVSSTAESLRCKLSAQELDSAHPLLGNVSTNEINIAKNETKTIDVNLSLSDLIKNASISKNSNTWKWSFQDLDNKQSKFIINSNHLVYTLPNTPKHPWNSAAGAYNAGD
ITYVWTDVIDVLIKMVPPASTDVIGALTTSHNNSSSFRYDTTRGASFYTDDQRRVKMSKYLNDRSSPTRKPLNCTDCANLVATLAASAGVHAYTNIMFSNFNLNKIIPIGCDNENPNWIVPFGGGFSYHEITLSEPTARQDIGIYDACLKVDQSQYPGQSDVSGKTAYLPANYAYAQTSSPKVNVPVNTPYKGAFYRERLVKDQQVVNMYLMALEPVGISSEVTSMERKSIDNSSLAKFKKMIRAQFG
LDDLPISNRPARQDFTPRFSAVNLIKDAKLIEDIASQKIWQITAADGRDYTVDINVADDEHTAREVLVERLSKISNPDIIRNDPPVAGDISFTVAGQSVLFVRNNIAVTVFFGPQSKPLATAIDQQIMT*
>Syntrophomonas_wolfei_subsp.metylbutyratica_JCM14075T_02381_hypothetical_protein
MKYVYPAVFTIDKKNSYTVAVPDLPGCFTFGDSLVEAIEMARDAISMWLVDAENKDEIIPTPGKIKDIPCEANSFINLIDIDTVEYRRENDNHAVKKTLSIPNWLNTKAEKAGINFSQTLQKALKNELGLE*
>Syntrophomonas_wolfei_subsp.metylbutyratica_JCM14075T_02382_putative_ATP-dependent_DNA_ligase_YkoU
MGESATIAIAGREIKLTNLDRVLWPEDGYCKRDLVEYYAAVFPYMLPHLSERPLVFTRYPRGIGEKSFYQKNAPEGLPQWIKTFTWTGSDGDSKNYVLIQQTADLMCLANLACIEIHPWLSQISSIEYPDFIVFDLDPSEQNTFGQIVSVTRLLQELMESLGLRVYPKTSGAKGLHLYLPIAKGYTYSQIRRVAQAMAEMVCQVVPDIATTERAMKRRGPRIYLDYLQNGLGKTVCAAYSVRPHKGAP
CSTPIEWQELESVRPDQFTIKTLPERLQQVGDLFADVLYDRQDLKKAMATLGVY*
>Syntrophomonas_wolfei_subsp.metylbutyratica_JCM14075T_02383_Putative_DNA_ligase-like_protein/MT0965
MEIFLREIRSMDPINCGQAFDSEDFLYQVKWDGVRMLTAVAGEQVTLLNKRGNLRSRQYPELQNLIKASTAVLDGEIVVLREGKPSFPAVMQRDNCRNPMKIQHLSKTLAISYMVFDLLYLNGRDLRSASLVDRLSQLAELFDNQGCLYLVENFSQGTILFNSVQRAGLEGIVAKRKNSFYRPGKQHDDWFKIKCRRSQNCLVGGYTLRGKQVNALLLGVMRDRSLSFAGKASSGLDAEQL
QILSETLPRLEVKDSPFPEPTPAGSHYITPQLAVEVEYLEWTDSLHLRFPVIKSFMKNSEADCNV*
>Syntrophomonas_wolfei_subsp.metylbutyratica_JCM14075T_02384_putative_DNA_repair_protein_YkoV

LKTLWKGAVSFGLVNIPVGMYSATENKDLKFHYLHQECMAPVNYKKVCSKCNKEIKAEEIVKGYEFQKGNYVIISEEDLARIPQENTKTIDILDFIKLEQLDPIYFDKSYYLEPVSGGDKAYTLIVEAMK[illegible]
AEENKNEGKKKGSPKKAAKTGS*
>Syntrophomonas_wolfei_subsp.metylbutyratica_JCM14075T_02386_Glutamate_2,3-aminomutase
[illegible]
>Syntrophomonas_wolfei_subsp.metylbutyratica_JCM14075T_02387_hypothetical_protein
[illegible]
>Syntrophomonas_wolfei_subsp.metylbutyratica_JCM14075T_02388_molybdenum-pterin_binding_domain_protein
MRISDPNKLAGKIMEIKEGSDFTEVIVDIGDFAVTASLTSSAYQDLRLKKGDEVFTMFNSTDVTLIKDSKKNED*
>Syntrophomonas_wolfei_subsp.metylbutyratica_JCM14075T_02389_hypothetical_protein
MCSTLAIDFLLESMHNIISENVIHIKSEVVK*
>Syntrophomonas_wolfei_subsp.metylbutyratica_JCM14075T_02390_hypothetical_protein
MRRQNVTLSLPEDVLRKAKHLAVDRNMSLSALLGTYILDIVAREEQYSKAKEFSIRLMEEGFDMGSAGESTWNREELHERN*
>Syntrophomonas_wolfei_subsp.metylbutyratica_JCM14075T_02391_putative_nucleic-acid-binding_protein,_contains_PIN_domain
MSEIKGRQFIDTNILVYAYDCSAGNKYEKARNLLINLWEEKNGCLSIQVLQEFYVIVTRKLKNPISAQQAANVIENYDQWPLHSPNVEDLLEAINIQKQEHISFWDSLIIRSAQRLGCTCIWTEDLNNG[illegible]
>Syntrophomonas_wolfei_subsp.metylbutyratica_JCM14075T_02392_Cation_efflux_system_protein_CzcA
[illegible]
>Syntrophomonas_wolfei_subsp.metylbutyratica_JCM14075T_02393_Thymidine_kinase
[illegible]
>Syntrophomonas_wolfei_subsp.metylbutyratica_JCM14075T_02394_acid-soluble_spore_protein_H
[illegible]
>Syntrophomonas_wolfei_subsp.metylbutyratica_JCM14075T_02397_hypothetical_protein
[illegible]
>Syntrophomonas_wolfei_subsp.metylbutyratica_JCM14075T_02398_Tyrosine--tRNA_ligase
[illegible]
>Syntrophomonas_wolfei_subsp.metylbutyratica_JCM14075T_02399_Penicillin-binding_protein_F
[illegible]
>Syntrophomonas_wolfei_subsp.metylbutyratica_JCM14075T_02400_MutS2_protein
[illegible]
>Syntrophomonas_wolfei_subsp.metylbutyratica_JCM14075T_02401_putative_protease_YhbU_precursor
[illegible]
>Syntrophomonas_wolfei_subsp.metylbutyratica_JCM14075T_02402_hypothetical_protein
[illegible]
>Syntrophomonas_wolfei_subsp.metylbutyratica_JCM14075T_02403_DNA_polymerase/3'-5'_exonuclease_PolX
[illegible]
>Syntrophomonas_wolfei_subsp.metylbutyratica_JCM14075T_02404_Z_ring-associated_protein_ZapA
[illegible]
>Syntrophomonas_wolfei_subsp.metylbutyratica_JCM14075T_02405_Plasmid_stabilisation_system_protein
[illegible]
>Syntrophomonas_wolfei_subsp.metylbutyratica_JCM14075T_02406_prevent-host-death_family_protein
[illegible]
>Syntrophomonas_wolfei_subsp.metylbutyratica_JCM14075T_02407_Na(+)/drug_antiporter
[illegible]
>Syntrophomonas_wolfei_subsp.metylbutyratica_JCM14075T_02408_hypothetical_protein
[illegible]
>Syntrophomonas_wolfei_subsp.metylbutyratica_JCM14075T_02409_Farnesyl_diphosphate_synthase
[illegible]
>Syntrophomonas_wolfei_subsp.metylbutyratica_JCM14075T_02410_Exodeoxyribonuclease_7_small_subunit
[illegible]
>Syntrophomonas_wolfei_subsp.metylbutyratica_JCM14075T_02411_Exodeoxyribonuclease_7_large_subunit
[illegible]
>Syntrophomonas_wolfei_subsp.metylbutyratica_JCM14075T_02412_Bifunctional_protein_FolD_protein
[illegible]
>Syntrophomonas_wolfei_subsp.metylbutyratica_JCM14075T_02413_hypothetical_protein
[illegible]
>Syntrophomonas_wolfei_subsp.metylbutyratica_JCM14075T_02414_t(6)A37_threonylcarbamoyladenosine_biosynthesis_protein
[illegible]
>Syntrophomonas_wolfei_subsp.metylbutyratica_JCM14075T_02415_hypothetical_protein
[illegible]
>Syntrophomonas_wolfei_subsp.metylbutyratica_JCM14075T_02416_hypothetical_protein
[illegible]
>Syntrophomonas_wolfei_subsp.metylbutyratica_JCM14075T_02417_hypothetical_protein
[illegible]
>Syntrophomonas_wolfei_subsp.metylbutyratica_JCM14075T_02418_Alkaline_shock_protein_23
[illegible]
>Syntrophomonas_wolfei_subsp.metylbutyratica_JCM14075T_02419_Biotin_carboxylase
[illegible]
>Syntrophomonas_wolfei_subsp.metylbutyratica_JCM14075T_02420_Methylmalonyl-CoA_carboxyltransferase_5S_subunit
[illegible]
>Syntrophomonas_wolfei_subsp.metylbutyratica_JCM14075T_02421_hypothetical_protein
[illegible]
>Syntrophomonas_wolfei_subsp.metylbutyratica_JCM14075T_02422_Stage_III_sporulation_protein_AH
[illegible]
>Syntrophomonas_wolfei_subsp.metylbutyratica_JCM14075T_02423_stage_III_sporulation_protein_AG
[illegible]
>Syntrophomonas_wolfei_subsp.metylbutyratica_JCM14075T_02424_stage_III_sporulation_protein_AF
[illegible]
>Syntrophomonas_wolfei_subsp.metylbutyratica_JCM14075T_02425_Stage_III_sporulation_protein_AE_precursor
[illegible]
>Syntrophomonas_wolfei_subsp.metylbutyratica_JCM14075T_02426_stage_III_sporulation_protein_AD
[illegible]
>Syntrophomonas_wolfei_subsp.metylbutyratica_JCM14075T_02427_stage_III_sporulation_protein_AC
[illegible]
>Syntrophomonas_wolfei_subsp.metylbutyratica_JCM14075T_02428_stage_III_sporulation_protein_SpoAB
[illegible]
>Syntrophomonas_wolfei_subsp.metylbutyratica_JCM14075T_02429_hypothetical_protein
[illegible]
>Syntrophomonas_wolfei_subsp.metylbutyratica_JCM14075T_02430_hypothetical_protein
[illegible]
>Syntrophomonas_wolfei_subsp.metylbutyratica_JCM14075T_02431_Nucleotidyltransferase_domain_protein
[illegible]
>Syntrophomonas_wolfei_subsp.metylbutyratica_JCM14075T_02432_hypothetical_protein
[illegible]
>Syntrophomonas_wolfei_subsp.metylbutyratica_JCM14075T_02433_transcriptional_regulator,_AbrB_family
[illegible]
>Syntrophomonas_wolfei_subsp.metylbutyratica_JCM14075T_02434_putative_nucleic-acid-binding_protein,_contains_PIN_domain
[illegible]
>Syntrophomonas_wolfei_subsp.metylbutyratica_JCM14075T_02435_Elongation_factor_P
[illegible]
>Syntrophomonas_wolfei_subsp.metylbutyratica_JCM14075T_02436_Xaa-Pro_dipeptidase
[illegible]
>Syntrophomonas_wolfei_subsp.metylbutyratica_JCM14075T_02437_3-dehydroquinate_dehydratase
[illegible]
>Syntrophomonas_wolfei_subsp.metylbutyratica_JCM14075T_02438_Peptidase_PmbA
[illegible]
>Syntrophomonas_wolfei_subsp.metylbutyratica_JCM14075T_02439_protease_TldD
[illegible]
>Syntrophomonas_wolfei_subsp.metylbutyratica_JCM14075T_02440_hypothetical_protein
[illegible]
>Syntrophomonas_wolfei_subsp.metylbutyratica_JCM14075T_02441_Fimbrial_assembly_protein_(PilN)
[illegible]
>Syntrophomonas_wolfei_subsp.metylbutyratica_JCM14075T_02442_type_IV_pilus_assembly_protein_PilM
[illegible]
>Syntrophomonas_wolfei_subsp.metylbutyratica_JCM14075T_02443_Late_competence_protein_ComC
[illegible]
>Syntrophomonas_wolfei_subsp.metylbutyratica_JCM14075T_02444_Tfp_pilus_assembly_protein_PilX
[illegible]
>Syntrophomonas_wolfei_subsp.metylbutyratica_JCM14075T_02445_Type_II_secretory_pathway,_component_PulJ
[illegible]
>Syntrophomonas_wolfei_subsp.metylbutyratica_JCM14075T_02446_hypothetical_protein
[illegible]
>Syntrophomonas_wolfei_subsp.metylbutyratica_JCM14075T_02447_putative_major_pilin_subunit
[illegible]
>Syntrophomonas_wolfei_subsp.metylbutyratica_JCM14075T_02448_General_secretion_pathway_protein_F
[illegible]
>Syntrophomonas_wolfei_subsp.metylbutyratica_JCM14075T_02449_Twitching_mobility_protein
[illegible]
>Syntrophomonas_wolfei_subsp.metylbutyratica_JCM14075T_02450_Type_II_traffic_warden_ATPase
[illegible]
>Syntrophomonas_wolfei_subsp.metylbutyratica_JCM14075T_02451_hypothetical_protein
[illegible]
>Syntrophomonas_wolfei_subsp.metylbutyratica_JCM14075T_02452_hypothetical_protein
[illegible]
>Syntrophomonas_wolfei_subsp.metylbutyratica_JCM14075T_02453_hypothetical_protein
[illegible]
>Syntrophomonas_wolfei_subsp.metylbutyratica_JCM14075T_02454_3-dehydroquinate_synthase
[illegible]
>Syntrophomonas_wolfei_subsp.metylbutyratica_JCM14075T_02455_Shikimate_kinase
[illegible]
>Syntrophomonas_wolfei_subsp.metylbutyratica_JCM14075T_02456_Chorismate_synthase
[illegible]
>Syntrophomonas_wolfei_subsp.metylbutyratica_JCM14075T_02457_hypothetical_protein
[illegible]
>Syntrophomonas_wolfei_subsp.metylbutyratica_JCM14075T_02458_[illegible]
[illegible]
>Syntrophomonas_wolfei_subsp.metylbutyratica_JCM14075T_02459_Shikimate_dehydrogenase
[illegible]
>Syntrophomonas_wolfei_subsp.metylbutyratica_JCM14075T_02460_UMP_phosphatase
[illegible]
>Syntrophomonas_wolfei_subsp.metylbutyratica_JCM14075T_02461_Galactose-1-phosphate_uridylyltransferase
[illegible]
>Syntrophomonas_wolfei_subsp.metylbutyratica_JCM14075T_02462_hypothetical_protein
[illegible]
>Syntrophomonas_wolfei_subsp.metylbutyratica_JCM14075T_02463_2-oxoglutarate_carboxylase_small_subunit
[illegible]
>Syntrophomonas_wolfei_subsp.metylbutyratica_JCM14075T_02464_RNA_polymerase_sigma-28_factor_precursor
[illegible]
>Syntrophomonas_wolfei_subsp.metylbutyratica_JCM14075T_02465_Type-1_restriction_enzyme_R_protein

[illegible]
>Syntrophomonas_wolfei_subsp.metylbutyratica_JCM14075T_02466_hypothetical_protein
[illegible]
>Syntrophomonas_wolfei_subsp.metylbutyratica_JCM14075T_02467_Type_I_restriction_enzyme_EcoKI_specificity_protein
[illegible]
>Syntrophomonas_wolfei_subsp.metylbutyratica_JCM14075T_02468_putative_type_I_restriction_enzymeP_M_protein
[illegible]
>Syntrophomonas_wolfei_subsp.metylbutyratica_JCM14075T_02469_hypothetical_protein
[illegible]
>Syntrophomonas_wolfei_subsp.metylbutyratica_JCM14075T_02470_PglZ_domain_protein
[illegible]
>Syntrophomonas_wolfei_subsp.metylbutyratica_JCM14075T_02471_RNA_polymerase-associated_protein_RapA
[illegible]
>Syntrophomonas_wolfei_subsp.metylbutyratica_JCM14075T_02472_putative_methylase
[illegible]
>Syntrophomonas_wolfei_subsp.metylbutyratica_JCM14075T_02473_hypothetical_protein
[illegible]
>Syntrophomonas_wolfei_subsp.metylbutyratica_JCM14075T_02474_hypothetical_protein
[illegible]
>Syntrophomonas_wolfei_subsp.metylbutyratica_JCM14075T_02475_DNA_binding_domain,_excisionase_family
MTNEPEKWSSLEEIAEHLGVSKDTIRAWIKKEVIPYRRIGKQYKFKISEVDAWVDSGESAKIE*
>Syntrophomonas_wolfei_subsp.metylbutyratica_JCM14075T_02476_hypothetical_protein
[illegible]
>Syntrophomonas_wolfei_subsp.metylbutyratica_JCM14075T_02477_putative_secretion_ATPase,_PEP-CTERM_locus_subfamily
LIRSKLALNVNNPLRQRIVVKHMMSNMQKDELGGYLKTRLAFGGTHEELFTAPSMEAIYGVTNGVPRLVNSLATNCLLYACEKKQRQVDEETVYQAQSELNF*
>Syntrophomonas_wolfei_subsp.metylbutyratica_JCM14075T_02478_hypothetical_protein
[illegible]
>Syntrophomonas_wolfei_subsp.metylbutyratica_JCM14075T_02479_putative_ABC-type_exoprotein_transport_system,_permease_component
[illegible]
>Syntrophomonas_wolfei_subsp.metylbutyratica_JCM14075T_02480_ABC-type_transporter_ATP-binding_protein_EcsA
[illegible]
>Syntrophomonas_wolfei_subsp.metylbutyratica_JCM14075T_02481_DNA_mismatch_repair_protein_MutS
[illegible]
>Syntrophomonas_wolfei_subsp.metylbutyratica_JCM14075T_02482_Formate_dehydrogenase_H
[illegible]
>Syntrophomonas_wolfei_subsp.metylbutyratica_JCM14075T_02483_Putative_formate_dehydrogenase
[illegible]
>Syntrophomonas_wolfei_subsp.metylbutyratica_JCM14075T_02484_NADP-reducing_hydrogenase_subunit_HndC
[illegible]
>Syntrophomonas_wolfei_subsp.metylbutyratica_JCM14075T_02485_4-O-beta-D-mannosyl-D-glucose_phosphorylase
[illegible]
>Syntrophomonas_wolfei_subsp.metylbutyratica_JCM14075T_02486_putative_zinc_finger/helix-turn-helix_protein,_YgiT_family
[illegible]
>Syntrophomonas_wolfei_subsp.metylbutyratica_JCM14075T_02487_hypothetical_protein
MAITTQEKVVLYLMKVKQLLVKEQYDFVPRKVNIGALTELGWTIEYFFLRIFNATVFVKLCKRSRN*
>Syntrophomonas_wolfei_subsp.metylbutyratica_JCM14075T_02488_hypothetical_protein
LLAFLGKKMNRLLNSKRGQDLPSKDSYYRFLNNPKYAWRIFLLQLSLQVTDAFDRLTSDKRVRVFILGDSILSRSRTKKAELLARIFDHTSQKL*
>Syntrophomonas_wolfei_subsp.metylbutyratica_JCM14075T_02489_Glutamine_transport_ATP-binding_protein_GlnQ
[illegible]
>Syntrophomonas_wolfei_subsp.metylbutyratica_JCM14075T_02490_Inner_membrane_amino-acid_ABC_transporter_permease_protein_YecS
[illegible]
>Syntrophomonas_wolfei_subsp.metylbutyratica_JCM14075T_02491_ABC_transporter_arginine-binding_protein_1_precursor
[illegible]
>Syntrophomonas_wolfei_subsp.metylbutyratica_JCM14075T_02492_hypothetical_protein
[illegible]
>Syntrophomonas_wolfei_subsp.metylbutyratica_JCM14075T_02493_hypothetical_protein
[illegible]
>Syntrophomonas_wolfei_subsp.metylbutyratica_JCM14075T_02494_ABC-type_transport_system_involved_in_multi-copper_enzyme_maturation,_permease_component
[illegible]
>Syntrophomonas_wolfei_subsp.metylbutyratica_JCM14075T_02495_putative_ABC_transporter_ATP-binding_protein_YxlF
[illegible]
>Syntrophomonas_wolfei_subsp.metylbutyratica_JCM14075T_02496_Alginate_biosynthesis_sensor_protein_KinB
[illegible]
>Syntrophomonas_wolfei_subsp.metylbutyratica_JCM14075T_02497_Alkaline_phosphatase_synthesis_transcriptional_regulatory_protein_PhoP
[illegible]
>Syntrophomonas_wolfei_subsp.metylbutyratica_JCM14075T_02498_RNA_polymerase_sigma_factor_SigM
[illegible]
>Syntrophomonas_wolfei_subsp.metylbutyratica_JCM14075T_02499_hypothetical_protein
[illegible]
>Syntrophomonas_wolfei_subsp.metylbutyratica_JCM14075T_02500_conjugal_transfer_protein_TrbA
[illegible]
>Syntrophomonas_wolfei_subsp.metylbutyratica_JCM14075T_02501_hypothetical_protein
[illegible]
>Syntrophomonas_wolfei_subsp.metylbutyratica_JCM14075T_02502_ribosomal-protein-alanine_acetyltransferase
[illegible]
>Syntrophomonas_wolfei_subsp.metylbutyratica_JCM14075T_02503_hypothetical_protein
[illegible]
>Syntrophomonas_wolfei_subsp.metylbutyratica_JCM14075T_02504_hypothetical_protein
[illegible]
>Syntrophomonas_wolfei_subsp.metylbutyratica_JCM14075T_02505_Succinyl-CoA:coenzyme_A_transferase
[illegible]
>Syntrophomonas_wolfei_subsp.metylbutyratica_JCM14075T_02506_Transcriptional_regulatory_protein_ZraR
[illegible]
>Syntrophomonas_wolfei_subsp.metylbutyratica_JCM14075T_02507_putative_3-hydroxybutyryl-CoA_dehydrogenase
[illegible]
>Syntrophomonas_wolfei_subsp.metylbutyratica_JCM14075T_02508_putative_enoyl-CoA_hydratase_echA8
[illegible]
>Syntrophomonas_wolfei_subsp.metylbutyratica_JCM14075T_02509_Acetyl-CoA_acetyltransferase
[illegible]
>Syntrophomonas_wolfei_subsp.metylbutyratica_JCM14075T_02510_Acyl-CoA_dehydrogenase
[illegible]
>Syntrophomonas_wolfei_subsp.metylbutyratica_JCM14075T_02511_Methyl-accepting_chemotaxis_protein
[illegible]
>Syntrophomonas_wolfei_subsp.metylbutyratica_JCM14075T_02512_HTH_domain_protein
[illegible]
>Syntrophomonas_wolfei_subsp.metylbutyratica_JCM14075T_02513_5-amino-6-(5-phosphoribosylamino)uracil_reductase
[illegible]
>Syntrophomonas_wolfei_subsp.metylbutyratica_JCM14075T_02514_hypothetical_protein
[illegible]
>Syntrophomonas_wolfei_subsp.metylbutyratica_JCM14075T_02515_Phd_YefM
[illegible]
>Syntrophomonas_wolfei_subsp.metylbutyratica_JCM14075T_02516_Plasmid_stabilisation_system_protein
[illegible]
>Syntrophomonas_wolfei_subsp.metylbutyratica_JCM14075T_02517_hypothetical_protein
[illegible]
>Syntrophomonas_wolfei_subsp.metylbutyratica_JCM14075T_02518_Cation_efflux_system_protein_CzcA
[illegible]
>Syntrophomonas_wolfei_subsp.metylbutyratica_JCM14075T_02519_Multidrug_transporter_MdtC
[illegible]
>Syntrophomonas_wolfei_subsp.metylbutyratica_JCM14075T_02520_RNA_polymerase_sigma_factor_SigW
[illegible]
>Syntrophomonas_wolfei_subsp.metylbutyratica_JCM14075T_02521_Helix-turn-helix_domain_protein
[illegible]
>Syntrophomonas_wolfei_subsp.metylbutyratica_JCM14075T_02522_hypothetical_protein
[illegible]
>Syntrophomonas_wolfei_subsp.metylbutyratica_JCM14075T_02523_DNA_mismatch_repair_protein
[illegible]
>Syntrophomonas_wolfei_subsp.metylbutyratica_JCM14075T_02524_Z1_domain_protein
[illegible]
>Syntrophomonas_wolfei_subsp.metylbutyratica_JCM14075T_02525_hypothetical_protein
[illegible]
>Syntrophomonas_wolfei_subsp.metylbutyratica_JCM14075T_02526_AIPR_protein
[illegible]
>Syntrophomonas_wolfei_subsp.metylbutyratica_JCM14075T_02527_Modification_methylase_HaeIII
[illegible]
>Syntrophomonas_wolfei_subsp.metylbutyratica_JCM14075T_02528_Very_short_patch_repair_protein
[illegible]
>Syntrophomonas_wolfei_subsp.metylbutyratica_JCM14075T_02529_hypothetical_protein
[illegible]
>Syntrophomonas_wolfei_subsp.metylbutyratica_JCM14075T_02530_hypothetical_protein
[illegible]
>Syntrophomonas_wolfei_subsp.metylbutyratica_JCM14075T_02531_hypothetical_protein
[illegible]
>Syntrophomonas_wolfei_subsp.metylbutyratica_JCM14075T_02532_Sporulation_initiation_inhibitor_protein_soj
[illegible]
>Syntrophomonas_wolfei_subsp.metylbutyratica_JCM14075T_02533_Nucleoid_occlusion_protein
[illegible]
>Syntrophomonas_wolfei_subsp.metylbutyratica_JCM14075T_02534_hypothetical_protein
[illegible]
>Syntrophomonas_wolfei_subsp.metylbutyratica_JCM14075T_02535_Murein_hydrolase_activator_NlpD_precursor
[illegible]
>Syntrophomonas_wolfei_subsp.metylbutyratica_JCM14075T_02536_HTH-type_transcriptional_regulator_YodT
[illegible]
>Syntrophomonas_wolfei_subsp.metylbutyratica_JCM14075T_02537_Putative_S-adenosyl-L-methionine-dependent_methyltransferase
[illegible]
>Syntrophomonas_wolfei_subsp.metylbutyratica_JCM14075T_02538_Phosphoenolpyruvate_synthase
[illegible]

EVIMGEYDTGNIPKGALAGTPVSNGVIEGRARVVLKLEDAIIEEGDILVTKFADPGWTPLFVSVKGLVTEVGGMMTHGAVIAREYSLPAVVGVDDATKLIKDGQRIRLNGSEGYVEIL*
>Syntrophomonas_wolfei_subsp.metylbutyratica_JCM14075T_02539_Glycosyltransferase_family_10_(fucosyltransferase)
VKTLEGSIAKGETVANPGINDLFELTPRFFYLKGEAEMLKVKISTRVPHEPIIRQTPGSQGIWRNCRFFINEEVEECDYWVVYDGLQHEESTLCPPENTILLTVEPPSVFYYEPDFVQQFSTVITCPGHDFSHPRIIYSPAFNWHAGRVIIDIKAVQSKVNLDYDDFKRMGAFKKSQQISLVCSNKDFTEGHRKRLAFVDRLMEHFGSRIEHFGFNINDIEDKWDAIREYKYHIAIENSFFSHYWTEKIADCY
LAGAYPIYYGCPNLAEYFPQGAYTSIDIEDVETAISSIENCLAENRYEKNLDKIWAARELVLDYYNLFAVIERCILRLEAATHSRQTPKIPTTIRPCQEAWLIT*
>Syntrophomonas_wolfei_subsp.metylbutyratica_JCM14075T_02540_hypothetical_protein
MLSSGDIVITPFIYSDLKGLKVRPALVLFLYVVHIIFLTVIEAIK*
>Syntrophomonas_wolfei_subsp.metylbutyratica_JCM14075T_02541_hypothetical_protein
MSVKIEQLIQEVQQLPHEELIVLCKALIIQLATPLRDPQEMYDDWDDLEVDKAYAQLW*
>Syntrophomonas_wolfei_subsp.metylbutyratica_JCM14075T_02542_Antitoxin_YefM
MIATNYSDVRQNFKEYCDKATYDFETIIVTRERGENVVIISESEYNNLLENLYVRSNPEYYNELLQSIEQLKKGRGKKRELLDE*
>Syntrophomonas_wolfei_subsp.metylbutyratica_JCM14075T_02543_Toxin_YoeB
MNKIFSDISWEHYLYWQVNDKKILRKINDLIKDIERNGNDGLGKPEPLKHELSGYWSRRITSEHRLIYSIDGDNIYIASCKGHY*
>Syntrophomonas_wolfei_subsp.metylbutyratica_JCM14075T_02544_GDP-L-fucose_synthase
MDKNAKIYLAGHQGLVGLALGRYLQARGYNNIITRSFDELDLRRQNAVEEFFRSFQPEYVFLAAAKVGGMWANYLRPGEFIYDNILIQSNVLEAARKYAVKKLLFLGSSCIYPKFAPQPIKEENLLQGELEPSNQAYAIAKIAGIAMCRAYRAQYGSNFIAIMPNNLYGPGDNFHPENGHVLPAMINKFHQAMLSRSPQVYLWGTGTPRREFLYVDDLADACCFLMKHYDEADIINVGSGEEYSISELA
AMVAAIAGYQGEIIWDSSKPDGTPRKLLDASRINAMGWHSRVSLWEGLKLTYDWYIKNP*
>Syntrophomonas_wolfei_subsp.metylbutyratica_JCM14075T_02545_dTDP-4-dehydro-6-deoxyglucose_reductase
MARKYLITGGTGFIGTALARRLLSAGHELKIYDNNSRGRRERLQGLAGEVQIVEGDIRDRERLIGAAQGMDSLIHLAYINGTEFFYNQPEMVLDIAIRGMLNVIDACRAEGIGELILASSSEVYQSPPQIPSSEAVPLSIPDPFNPRFSYGGGKIACELMAINYGRKDFERVLIFRPHNVYGPDMGWEHVLPQFILRAIKAIESKPQGPVPFPIQGDGSQTRAFIYIDDFIDALISLIQSGQHLNIYHIGNPEEISI
NTVADKLFQYLGREVEIIKGSLPPGSPPRRCPDISKLLELGFRPRISFEQGLAATIEWYTRAIPEGKNPKY*
>Syntrophomonas_wolfei_subsp.metylbutyratica_JCM14075T_02546_UDP-glucose_6-dehydrogenase_TuaD
MSKVPLLYPDRNICIMGLGYVGLTLATVMAECGFQVLGVEIRKDIVARLQKGESHFYEPGLSARLKNVIKTGRLQVTASIADNFAGSVFIITVGTPLDEKGKVRLDMVENVCREVAAHLKENDLVIMRSTVKLETTRKIVRAILEEAGVNFELAFCPERSLEGKALQELRSLPQIVGGLTPGAAIRAAHLFLFITPTVIKVSDMETAEMIKLIDNAQRDVFFAYANEVARLCDAIGVSAAEVIQAGNLAYPR
SRLPQPGPVGGPCLEKDPHILAEKILELGLRPEITLAARKINEEQPEEVISYLHRVCSGLASFPACPQISLLGLAFKGQPPTDDLRGTMARPVLNSLQKHFPCAHFRGFDPVVRAEEIEAFGLIPCVSLEDAVRGANLILILNNHPRFAHMQIEDLSELLARPALIYDFWNNFVAKELHLPSGIGYMALGSHSRAVLP*
>Syntrophomonas_wolfei_subsp.metylbutyratica_JCM14075T_02547_Disaggregatase_related_repeat
VATITLLPFHDSYIAEWYARDNFAGESCLNLSQFRQTGDNYRSLLKFRLDAIPGGCSIEKAELELTMYRNEVKDAIDITVQRLLSRWHEKSVNWDTQPLHSPEIAGKIRVDANTPMGALRMDLSGLVKGWHDGSIINNGLLLKGNEVENNLVAFYNSKHINGDEWPKLHISFVDGVLNAFDKEEIIIPSYPEVPVATSAPIALGPRALATFLIANCSDSCKLRARIQVGYGKCHCSTYYDVGEQISLKAR
GYPGEAVALSSWEAAEFARILLIGAGGEKVSIYARSKEL*
>Syntrophomonas_wolfei_subsp.metylbutyratica_JCM14075T_02548_hypothetical_protein
MSDYKLENPGCCPPCPPYPPRLNQTILSCGTGSGVNLPSHKESRSQLLNPYVVASLSIDTSNLSSPSVKLDFSSIITYKELHDYWLCDLRLTFQLSRVCGGAKAVLGSWNFERNVGFGWYGNSGRATEGSEAELRANGIGLTVETVDAFGFTFCDCHACPGCCTYMVEVINAETEGVDFANVSNPMFNALAVGPLAWGD*
>Syntrophomonas_wolfei_subsp.metylbutyratica_JCM14075T_02549_Stage_II_sporulation_protein_AC
MSSGDSYQNNEALLRLAQEGNQEAKAQLYEANIGLIYLVLERFKNTSYDQEDLFQVGAMGLLKAIERFDFSFDVRFSTYAVPMIIGEIKKFLRDDGMVKVQRGIKELYARIRWAQDKLRGDLGREPGVNEIASLLEMEKEDIVVAMEACQTPAYIHDVVPSEEKEQLSLLDKLVHEEGNVAILEKLALREALAKLDERERVVIIRRFFQDETQTVIAEDLGVSQVQISRIEKSAILKLRSLLQ*
>Syntrophomonas_wolfei_subsp.metylbutyratica_JCM14075T_02550_Anti-sigma_F_factor
MNVKNYVRFEFKSLAENVSFARSCVAAFAAQLDCTLEDIEEIKVVVSEAVSNSIIHAYENRCNEKIVVLVSIMDDRSLEIVVEDHGKGIEDIEKAIEPSFSSDSSRTGLGFTFMKSYMDEMEVFSQPGEGTTVRLKRCFGKKEQEAYA*
>Syntrophomonas_wolfei_subsp.metylbutyratica_JCM14075T_02551_Stage_II_sporulation_protein_AA
VEGIRPLLVEACFSSLQGGDFAVEIDLKVIRNTLVVKISGELDLLVAEKLRREIDSKIQSQQVKNLVLNLEKVSFIDSSGLGVIIGRYKRMNAENGRMYIVGASPAVEKILIFSGINKLVPLYRSEQDIINI*
>Syntrophomonas_wolfei_subsp.metylbutyratica_JCM14075T_02552_D-alanyl-D-alanine_carboxypeptidase_DacF_precursor
MCCRRSIAWLVLWVMVFIIAAPARLEAEVVEAKAESYVLMDADSGKVLLAKNEHKQLAPASMTKLMTLILAMEALEEGKATLKDKVVTSESAWKMGGSQIYLEPGEQMSYEDMLIAIAVGSANDACVAVAEHLEGTHQNFVDKMNHKAKMLGLKNTHFINSYGLTAPKHYTSAYDMAVIGREALAHPKILEYSSIKEYDLRQGEFKLFNTNKLLWWYQGADGFKTGWTDEAKYCLVGTAKRD
GLRLISVVMASPEKHGNFRDSMQLLNYGFARYAFKSLIGQGKSCGMVKIGKGMEEQVGVVAEEEVGCTLIKGEEKKINSKTTLLPYVDAPVSKGQKLGEISLYKDGDLIKEINLVAASNVGRGGLLKEIGKIFAEIYLL*
>Syntrophomonas_wolfei_subsp.metylbutyratica_JCM14075T_02553_hypothetical_protein
MLENWQSIAEQIYMNELTFPHLRERLQHGGELRAWYKYYRGGNQWAGKHFMEENVFLMEEVLIWVQIAEDGKMGLYSFKLDDISKIDRSYTFADKSQEKLVLSQATATFKTMKDNRKRDTLVFKRPLPAEEGDVEGFEQLIALLD*
>Syntrophomonas_wolfei_subsp.metylbutyratica_JCM14075T_02554_Hef_nuclease
MSLLYEGNSEGPDRTGPAVGGQLKKKLFAWQEECLDIWMQHQGRGIVNVVTGAGKTILALGAIARLEKQLLRENAAGLKVIIVVPQVFLLQQWAQTLQDELGIARDQIGIYSGAHKDLPTRKYMIYVVNSARHCLARHLLEDYQRGAAVMLIADECHHYSSPENSRIFDFISHISPGSSSRYYALGLSATPQAPRFDEKLVPALGVQIYKYGFAEALNANIISSFSIFNLKLKFTPDEEQEYLELSEQLSR
VLDVLMRRCPYLNGLSSQHFFACLEEMARESDDSALSGLSRAVLILAHQRKDVVYKARARISCVQNLLQRIPAAAKVIIFNERIETAEAIYALLQRLFSGQVGRYHSEMDERSKKRVLRRYSNSEIRILVTCRALDEGLNVPATDVGIIACSTRSSRQRIQRLGRILRRSGENSHACLYYLYIGASNEEQELMADISQELAGLIPVLDLEYDHESQSFLHPSYQLLVDRVLAYARRKAWDAEIVGELERNLERGK
LGCEWWFSQQECLRRIQSARSRAERNYWISMRLLAQASRQGLPNG*
>Syntrophomonas_wolfei_subsp.metylbutyratica_JCM14075T_02555_hypothetical_protein
MADYESLLKQQLLTERELKEKISSLQKCFKSLCKNMEKGDLKSWNRELSTMRSLLQEQEALLTGMQAQVDEFGIRPYMENGDFVEQMLAYCDSLGVDVKGDFPSYEMFPFRVKIDAENLELFLDRKKVQCLRPLAFVQEVKSSRDKMLRASFDPRFFVRELAAAYDLLLLKQNQGKAHPVLEGDVYLKNIYTILTPMRRFRRDYDQQSFAFDLARLYASDLRSTEDGRRFQFGPSRNISKAIRILD
QEGREQYLATIRFFASPEEEMTLPL*
>Syntrophomonas_wolfei_subsp.metylbutyratica_JCM14075T_02556_hypothetical_protein
LDNDIKSALACLKKGHSPGKREFLQEISVGTAFMLDFWREKYLAEYLARGGSKIKFLTGRSGSGKTHFLEMLALEAENMGYIPVKLSAREVWIHDFKEIYSAVLHQVNLLERLKLCSHEIIRQVGYDPEAIPQDMTLVDYLASQGKLDPLTKLEIRQQLNQLFLSNPLIDNNFALACALLTGSIMGHPTLEESSCRLLQLWLEGNREARLPSIRRLGLSPSKVTKYNARHMLRSLVEICRLAGYKGLIISIDE
LDILVRKDDMPELRYTRMKREDAYESIRELIDEIDTLKCVMFVFSFDRRLMDDDRWGLKSYPALWMRIQNEIEGERFNRFADIVDLDRLVDEVYTPDKIVEMSTRLARLINRYDEGARPLSLSTAEELYARARFGKVSLPRRVILSTLQGGVEEQ*
>Syntrophomonas_wolfei_subsp.metylbutyratica_JCM14075T_02557_hypothetical_protein
MNFEAQRVIEALRSGVSSRAVGHYFSSARPELMRQLSRELDRVMENGDSSGMVITGRYGEGKTHLLNTVVNLAHNNNMVVSLSLSKETPFDKLYLYLVYQKMVSNTYLPGRLQPGFEQLLQDLTPNSQAAQDILSFTSKHLETDKLFFVFRSYLHVDDPDDKYLLLADLEGDFISTSRLRQVYKRIFGQAARFATPFTKSRHSTDYFAMLSHLFKLLGFNGWVILFDEGELVGRLGKKARLKAYSNM
ASFLFPEQYSHLESTFCIFALTASFIEDVIEGRHEYENLNSVYVERSEREPVEKVLNSIKTAAQLQALSPAEIKVVLEQLRDFHAQAYDWPANLDIEQVLKATETRGYLLRTRIRAAVELLDQLYQYGQAGDIRINELGEPQYLEDELPALDDCLLES*
>Syntrophomonas_wolfei_subsp.metylbutyratica_JCM14075T_02558_Pyrimidine-nucleoside_phosphorylase
MKMSYIIEKKRDGKDLSRDEIEFLVRGIAVGTIPDYQISAWAMAVYFQGMSPGETRDLALAMAYSGEVIELSAVEGVKVDKHSTGGVGDKTTLVVIPLVAAAGVPVAKMSGRGLGHTGGTIDKFESITGFKVEMAHPAFLQQVSQIKAAVVSQSGNLVPADKRLYAIRDVTATIDSLPLIASSIMSKKIASGAEGIVLDVKVGSGAFMKSYEKAAQLARIMIDIGRGAGRKVVALLSNMDQPLGNAVG
NALEVKEAIDTLKGQGPPDLEELSLELAAQMIFLGEKASSINEAREIARSALKSGLGLVSWKQMVEAQGGKLNYSLPHYGLPEAAIKEKITAGQRAYVEHLNALEVGQAAMLLGAGREQVRDIIDHAVGIELLKKPGDLVEAGDTLALIHANDPGRLETARRQLLRAYRFSEQAPEKSPLIIQLIS*
>Syntrophomonas_wolfei_subsp.metylbutyratica_JCM14075T_02559_Phosphopentomutase
LTRRAILIVLDSAGIGEMEDSSLYGDQGSNTIVNTARAVGGLELPRMQSLGLGNLDEIPGVKPVQQALGAYGKMKEKSPGKDSTTGHWEMMGQVLKYPFPTYPHAFPPDLIEEFERRIQRKTLGNVVASGTEIIERLGPEHMKTACPIVYTSADSVFQVAAHEELIPLAELYAICHTAREMLAGENAVGRVIARPFTGHPGAFQRTPNRHDFSLAPERSILDAIIEAGQQVIAIGKIYDLFAGRGVSESR
PSKSNRQGIELIIAAIKEESSGLIFANLLDFDQVYGHRNDVEGYARALEEFDAELSNIMSALKPGDLLFISADHGCDPTTPSTDHSREMVPVLVYGRECRPGVDLGIRESFADLGQSIAEHIGVPASGLAGKSFYPLIRGLV*
>Syntrophomonas_wolfei_subsp.metylbutyratica_JCM14075T_02560_Tyrosine_recombinase_XerD
MQNSIEDFMAYLNFEKGLAANSRIAYKRDLLKFQEFLRENSRALCPQNISKHDIMAFLSWQLDQGAAHSSIARSLSSIKSYYKFLILEGELEKNPSSDLETPKIKRKLPQVLSIEEVDKLMEQPNPVVPLGIRDRAMLELMYGTGIRVSELLSLQVEDLNPTAGFLRCMGKGRKERIIPVNQTSIEWVNRYLARVRNSLVKNSLERTLFLNARGRPLSRQGFFKILENYVGKAELEKEVTPHTLRHSFATHLL
ENGADLRAVQEMLGHADISTTQIYTHLTKSRLREVYQQYHPRA*
>Syntrophomonas_wolfei_subsp.metylbutyratica_JCM14075T_02561_hypothetical_protein
VIVVINNWKKKILNFLILLVFILAFALAVPTMTGILHKQVPALGNWFKEEHPSGNPMRVEKNKQSTRFEKVMDQFVFKLQNFYYEEKE*
>Syntrophomonas_wolfei_subsp.metylbutyratica_JCM14075T_02562_Stage_II_sporulation_protein_M
MYLKQRIKQHIRDNRWQYLLILLIFLSGIVVGSYKVSSLDGGVRSHLLQMIDKYLKGGMEGNLDGASIFCSAFLHQSKTILVIWFLGLTVIGFPLILAVIFLRGISLGFTLSFLFQEKAGAGIILSFIAVLPQNIVYIPFLLIWAVVAVNASLYILKGRNNSYLPLGTALIAYSILMLLFILVFLLGAFIEAYLSPWLLQLLV*
>Syntrophomonas_wolfei_subsp.metylbutyratica_JCM14075T_02563_Cobalt-dependent_inorganic_pyrophosphatase
VKAIYVIGHKNPDIDAVAAIAYREYKEATDQGLYLAAMAGEMSDEIDFVLEAFDFAPPLHIKNVKTTVEDLLDEKEPFCVCRDMNLMELSNILRRQELKTVPVVDEKERLLGLITIGDMAMLFMNSLLDMGDLAETPGILGKLLDRKVTDIMKTRDLILFEKDEAAEEARKLMLSTRFRNYPVVDEENRFLGIISRYHLLNMRRKKLILVDHNEKKQAVDGVEEAEILEIVDHHRVGDLQTISPIYFH
NEPVGSTSTLVAEKFISMKLALSKNLAGLLLSGILSDTMLFKSPTTTGKDLSIAAKLQDISGLEPLSWGKAIFENSHQLDKRDDEELIYEDLKEYSSEELIFAISQIETVDLASFSRRKASLLKSMQEICERKAYAFICLMVTGILEEGTELLFAGEKSSLVERAFGLHSEEGEIFLQGVMSRKKQVVPVIYEALRQQNLL*
>Syntrophomonas_wolfei_subsp.metylbutyratica_JCM14075T_02564_Signal_transduction_histidine-protein_kinase_BarA
MTDGFSINEMKANFDTLFNTLEDYLFIIDENGKTVYANKAVIEKLGYSPEELEGMRLLLLHPPERRDEAANIFASMIAGKRDHCPIPLYTKGGSCIPVETKVVRGQWQGTPVFFTISKDISQISQANARFSKAFSINPALMAISKIDSGELIDVNESYLRRLEYSRDEVIGKTSSELGIIPFAERDLLIKELYQQGYIHNKELTLSTKNASELYVIFSVEYIETDDQVYLLTVMVDITGRKIAEELLKESEDRWSSAL
ECSGNGVWDWDAVTNQVFFSREWKSMLGYEEYEIGDSLQEWQSRVHPEDWERIQVELQKHFDGQTPVYGSEHRLRTRDGSYKWILDRGKVIKRDEEGQVLRVIGTHTDITEIKKVQEDLSRTRGQLKAILDNLPFLAWFKDKEGHHIEVNRFFEISCGLTRDEIIGKTDLDIWPREFALKYMEDDREVMASKKQINKEERLQDKAGGIWFSTFKTPVFDQKGNVIGTTGITRDITESSRLEHELIE
QRTFLKSLIDAIPDLIFYKDINNIYLGCNHAFAHRFIGLAEEEIVGRTDLDFVKDKELARFFMQKDREMLLSGETRMNEETITLVDGSIMELETVKTPFYKDGTIAGLIGVSRDITKRKIAQNQLLIKQKILSNIAAATNELLINSDYYEAINKCLALLGEATGVDRVYMFENHYEDNKACTSYKMEWNSGIFPAQINNPRLRNIPFEQIANFLEPLMRNEAIIGRVQEFKDGLDKELLLEQGIISILALPIFVGG
VLWGFVGFDECKEERNWSEDEFSILKAFSNSIAEAIERSQMEQKLAQAKEAAESANHAKSLFLANMSHEIRTPMNGILGFLELLGETELSTEQQDYVQEAHRASEILLYLINDILDFSKIEAGKLTMEEIPFRVRTVVEDAVSLQAPKAREKGLEMHTLIKSNVPDEVVGDPARLRQILNNLLSNAVKFTHTGEILVIAETITETAEQVEIAFEVCDSGIGIAAEDMEKLFQPFTQADASTTRKYGGTGLGL
AISYELVRLMKGTMQVKSKLGHGSKFYFTACFKPGLHKDSLDFEPAELTGARVLVVDDNSSNRKIIRTYLEDAQCLVEESDSAEKAITILLSTAAKKSFDVVIVDFQMPVMNGYELAAALKAIPSTRAIHLIMLTSAAQRGDANRAKEYGFSAYLTKPVKRDELLKCVSIVLGLKAETSLGDSIITRYTIKENPVSAHPRFLLVEDNEMNQKIIKMLEKKGMYCDLASSGAEALKALQQKDYDMVFMDC
QMPEMDGYETTARIRQMEGEERHTVIVAMTANAMEGDREKCLQAGMDDYISKPVDFQLLFNIIEHYAAGQQESVSEIAAMLEESLSSFMAETGLSERDSRELYDKLWSTLPGVLEELQNAVTNDDYKKVRSLAHQLKGSSGTLRIQKLYELFLDLEQKAIAEDTAASVDTLQFIKRLVKS*
>Syntrophomonas_wolfei_subsp.metylbutyratica_JCM14075T_02565_Stalked_cell_differentiation-controlling_protein
MRVLLADDDLISRAVVKALLSKWGYEVLEVGDGIQAWDILKEKDSPQLVLLDWMMPGIDGLELCRRLRQLDNNTYHYIILLTGRDSKEDIIGGLNAGADDYITKPFMPEELEVRLRVGKRILDLQQSLNEALEVQRYQAQHDLLTGIFNHAKILNILEKELYRARRQNSNLAIIMGDLDHFKKVNDTYGHVAGDAVLAEVARRMKNNIRLYDSVGRYGGEEFLLVLPGCSTEEAIIIANRILESISQEA
VMFNSTPIAVTISLGVAMKAVGDEKTTTAELVQLADAALYKAKQNGRNRVEQAAEPSP*
>Syntrophomonas_wolfei_subsp.metylbutyratica_JCM14075T_02566_hypothetical_protein
MVKSKRIIVSSDASGAVQAAREGLLLIIVDVIDMSTTLESALDAGAAAVLGCSPDFTRAPVKVAPEEIGQEASRLAREKGRGIILVAEPRVGTEEERRGRCQRVISGIEKGGGVIEAVVPNIGAETPRLVDMKDRVVVAVTDTGGVAFDAAFQESRRLTVGTVARTLKQKGMEPALTAVSRALKMLQETDQGIAVVAASRNSLEDVLAAQFIANLFL*
>Syntrophomonas_wolfei_subsp.metylbutyratica_JCM14075T_02567_hypothetical_protein
LREIFADLHIHIGRSQGRAIKITASRELTVKNILFQEAPHKGLDMVGIVDAGSTLVAAEIEEMLLHGELEEQPQGGFLAANGVLMIAGAEIESREGVHWIIFLPFLHSIKSLQKYLRSRVRNMTLSTQKADATAIDLLNLSYLLEGIFCPAHAFTPHKGAYGFWTDSLADKMGRDLTQVKVLELGLSADSLMADMIAETSGFTFLSNSDAHSLANIGREYNLLRMKQKNFAEFRYCLENYEGRRVLANYG
MDPLMGKYHRSFCLKCERIAAEAPPVQACANCGSENLVNGVYDRIVSIRDYSEPRQPLGRAPYYYRVPLQKLPGIGPKTLEKLRAGLGSEIDICENANQDDISKIAGERVAALIIEMRKGRLDISPGGGGRYGKVQKNNRKQ*
>Syntrophomonas_wolfei_subsp.metylbutyratica_JCM14075T_02568_ADP-ribose_pyrophosphatase
MDLEEKTLASREIFQGRIIKVRVDQVLLPDGSESSREIVEHSGAVGIVAIDKENNLWMVRQYRKALERVLLEIPAGTLEENEEPLECARRELEEETGLQAAEWQEILSYHSAPGFCDEKLFLFMAQGLSLGESSLDRDEFLEVKKVPLKEAYEMIFSGEIIDGKSIIGIQYACRLLGC*
>Syntrophomonas_wolfei_subsp.metylbutyratica_JCM14075T_02569_hypothetical_protein
MSISVGKNTIVFSGKFGELLALLKAYPPDTRLQDFIRLHLH*
>Syntrophomonas_wolfei_subsp.metylbutyratica_JCM14075T_02570_hypothetical_protein
MIAIKRGKILKELFTRPGIKGYIISIDGEEARSIVYPELVGEVLPGDEVLVNTTAVELELGSGGYHYVIASLPGRNRELAPGGHIMKLRYTPMQLKVLSVEEEASPFHQVMQEADSLENTPVLVSSLHSMLLPLCLQLKEKDKELRLAYIMTDGAALPLAFSQVVDYLKREGLLQAALSVGHAFGGDLEAVNIYSGLLAARHILQADIIIVGMGPGIVGTGTKWGFTGIEQGEILNAVEALEGRPVAVPRISF
ADKRKRHQGISHHTLTVLSRVCRVKALVPLPLLEEEKMDFLWTQVREAGLLDKYHFTVENEPGILDLLNNGSFKASTMGRGVEEEKEFFLALGAAAQAALRLYRQE*
>Syntrophomonas_wolfei_subsp.metylbutyratica_JCM14075T_02571_Peptidase_T
MVKVKRLVDIFIRLVETASVSGRESAVRDLLQQWFQQRGLTLVEDEAGQELGGDSGNLLLQIPGTMEGPTLLFAAHMDTVEPGIGVKAVVDEDGFIRSKGDTILGSDDKAAIAVLLEVFDILKEKGISHPPLEFLFTVSEEQGLKGAKAFDFKQLKSKIAYVLDAGDKPGAIIIQSPCQNEMEYTAFGRAAHAGINPEDGINAIQLVAHALAAMPCGRIDDETTCNFGGIEGGTARNIVAEFCRVKGE
ARSLRREKLDLLSTELKDIFIAEVEKRGGKAEVAVRSLYPEIRLEESEEVVQLAARAARNIGLKPRLLSTGGGSDASIINGQGIRCANLGVGMRAVHTCNEHISIEDLVNDARWVLGIIEEAARIGE*
>Syntrophomonas_wolfei_subsp.metylbutyratica_JCM14075T_02572_Glycosyl_transferase_family_4
LNIWLVAGLAAVAGVLAEGLILHYLLRLLQESAATRKNYQGLDIPVSAGISFPLALLLIYTILLFAPLYAQPVKQEVYFLFLMGIFAISLLGFADDMLGQRDTLGFKGHLGALLHGRMTTGGLKAAGGALIALFLALSLASGWPDIIVNTLIMALFANMLNLLDLRPGRAVKGFFFFFILLAFLASWKINWILIAPLLGATLYYLHFDLKALAMMGDSGSNVLGLALGFMAAGSLPLSFKVGVLFFLIFMH
LITEKYSLTRIIEKVAVLRKIDQWGRGKSDGKSEKTG*
>Syntrophomonas_wolfei_subsp.metylbutyratica_JCM14075T_02573_Glucosyl-3-phosphoglycerate_synthase
MKNITVVIPAYNEEERIGETLQAVKSIPGISRVIVVSDGSTDATASRAREEGVEVLELYPNRGKGGAMNAVLPFLNIEQREPSLSAEAMELGFVPQLSTREHTADSSANLLPPQLEGKAADIVVFLDADLGASASQASRLIEPVAKGMADLSIASFPPPKKKGGFGLVKGTAAWLIRKVGKMEARAPLSGQRAMTREVLQAVTPFNEGYGVELGMSVNALLQGFKLLEVPTTMSHNESGRDWKGFLH
RGRQFWDVLRVIGDLRRKGI*
>Syntrophomonas_wolfei_subsp.metylbutyratica_JCM14075T_02574_Mycobacterial_copper_transport_protein_B
LIDLRYHIASIVAVFLALGLGILIGSTIVGDNLLVDQQKKMIDRLEGQFYSLRERESELSASNQYKDQIISNYENYSQALLPPLVKEHLTGYQVALVVTGGSDIPAGMLNALSIAGANVVSKSVILSNISLDEANLRQRVQEFYGLDKEVSPDVLRQYVAASVAAVITDKADSGVISFLQENDLLKFSGSNTMPLNGIVLLGGTNNLAGFFAASFDHTLIESLAQQGLKIFGVENSKVTYSYMEEYQKDKISTI
DNVDLSPGQISLVYAMEGEPGDYGIKVTAKKFMPSLPVESVNKQQR*
>Syntrophomonas_wolfei_subsp.metylbutyratica_JCM14075T_02575_thiamine_pyrophosphokinase
MLAKGRARMDRRTKNLVKRLKKSDIAIIDHDDIDEVAANSLIDARPQAIINTQASITGRYPAKGVYKILKAGIPVLDEVGENIFSQLREGVLIEIRGNQVFWGKTVIAEGKELSLEDVRLKLKEAHQNIQLELDHFVDNTMEYAKKEKDLLIGNIDVPVLDTYMLNRHVLIVVRGSSYKEDLRTISSYIQEEQPVLIGVDGGADALLEFGLKPDLIVGDMDSVSDRALSSGAEIVVHAYADGTAPGLDRIES
MGLKAKTFPMPGTSEDVAMILAWEYGASLIVAVGSHSNMIDFLEKGRKGMGSTFLVRLKVGSILVDARGVSQLYKQHLPSKYLLQLLVAALIPITVILAVSPATRPFLRLILLQLKILFNI*
>Syntrophomonas_wolfei_subsp.metylbutyratica_JCM14075T_02576_Stage_0_sporulation_protein_A
MFSEANRIKILIADDNREFCGILRDYFNNDSDFEIVGICNNGTEVLEIIEKTPIEVLILDLIMPYMDGIGVLEKINELNIEPRPRIIILTAFGQENITQKAVQLGADYYILKPFNLQVLGDRVKQLVRDVPPRVENRNAGSSVVSSRSGAGKDMEIEVTKVIHEIGVPAHVKGYQYLRDAIMLVVAEINYLGAVTKELYPTIAQKYDTTPSRVERAIRHAIELAWDRGDLDKINKFFGYTVSGEKGKPTNSEFIAII
ADRLRLENKVS*
>Syntrophomonas_wolfei_subsp.metylbutyratica_JCM14075T_02577_SpoIVB_peptidase_precursor
LQRIRPWLGIVLSLLIALCITPQAQSLLNLPQYQRLVVGESSTISLPLPEKLKDKIEMQVISPSRSVFAAPQDPPVTISSNSSGYEILALKPGKVNVKLKLLGYIPLKSMAVETLPTKRVVAGGHSIGVILQSKGIMVVGYAPVSDSEGKKAYPAREQGVEIGDIIYQVDGQPLHSESELARIIDEKKSVLLGIKRGSKKLSITVRSTYCPETNRCRIGLYVRDGVVGVGTLTFWEPDSKIFAALGHVIIDADTKQ
QIDVLQGKIVSASIQGIKPGKPGQPGEKIGVFNGEGEIEGKIIKNSYSGIFGKTGDEITNPLAIYTMEVAYAHQVNEGKAQILTVVNGDDIESFDVVVEKVFPERQDGKSMIVRVVDQRLLNLTGGIIQGMSGSPIIQNGKIIGAITHVFLNDPTRGYGIFMDNMLSEIPSNLNPWSKVSTNSRDTWGIIAH*
>Syntrophomonas_wolfei_subsp.metylbutyratica_JCM14075T_02578_hypothetical_protein
MFELTPFRKRRDMMNSLLGGDMLKEFNDFWYGAALNMKADIKENNKEYIVEAELPGVKKENINVELKDHTLTIAATQDEETKDEGINYVRRERRTGSVSRSFYVENVDQEGVKADYKDGILKIMLPKLKETPPDKYQVKID*
>Syntrophomonas_wolfei_subsp.metylbutyratica_JCM14075T_02579_Recombination_protein_N
MLQEIYIKNFVLIEELRLSFEQGLNVLTGETGAGKSIIIDALGLVLGERVRNDLLRDSQKKAIVEAVFAVENNSAAHSVLLENGLLEEEEDCIVVSREIATSGRSLARINGRNVNAGFLKSLARHLIDMHLQDESSGFLNPQNYLQFVDGFIEEGQDIGKEMESLFLQIRDEERQLENYKNNEQNRLQKVDFIRYQIEEIEKAGLRSGEEEELLALNQRIKNAQALEEGTGRILELLYHRQHADESALDLISAS
LENLGRLKEDEFLSRLETPLEEIYYSLQDIASQLSSFRDSLDFEPGLLEESEERLYQLQRLKGKYGQSIDEILDYLGKARLELEYLENSGEKQQELEESIAALKLRYHHQAAQLSQLRQSAAQKLQESVDMELQELNMPQVKFAVQMEPRETPGIKGMDQVDFLFSPNPGEEMHSLSRIASGGERSRFILALKKALAGIYMIPTLIFDEIDVGVGGTALAAMASKLYELSLEHQLILVTHSPQIASLAHQHFL
IEKASSEDCTLTTVRELEAEARVEEIARMLEGEDFSPLALEHAREMLTRRELTPAAVKRE*
>Syntrophomonas_wolfei_subsp.metylbutyratica_JCM14075T_02580_Arginine_hydroxamate_resistance_protein
MKLRRQLCIIDIISQKEVGTQEELCETLKNQGFDVTQATVSRDIKELKLIKVADKDGYHYALPDTPGVKGSYERMKRVIEDSVLGLDYSENLIVIKTLPGSAHAVASLIDSAEWPTIIGTVAGDDTILAVVKPKEAAPGILEEFEQLMLKSNR*
>Syntrophomonas_wolfei_subsp.metylbutyratica_JCM14075T_02581_Inorganic_polyphosphate/ATP-NAD_kinase
VKIILVNNRFKENTEKKAQEIAGQLSALNIEVIIDNGLDEPYAGTVDFIMVLGGDGTMLRAARRYGQRAIPVLGVNMGTVGFLSNIEINELAEYLPLILREEYSLEARMMLELAVFYQQSLLTRVFCLNELLLRSNSPRMLSFALEISGQKLEPYRGDGLIVSTTTGSTAYSLSAGGPVADPQLDAFIITPVASHIINKRPLVVSPEREISLRLLEEREAIIGIDGQIKMDFGDENRVLIKRAPHPLLMVNLKAKP
FFTSIDRSLQRQ*
>Syntrophomonas_wolfei_subsp.metylbutyratica_JCM14075T_02582_16S/23S_rRNA_(cytidine-2'-O)-methyltransferase_TlyA
LARIRLDSLLHDKALAPSREKARAMILAGEVRVNGQMVDKPGTTVDEEARIELKSHLSRYVSRGGFKLEKAIEDFRLDFSQRVVLDIGASTGGYTDCALQHGAIKVFALDVGYGQLDWKLRNDPRVINLERRNIRYFSREELGEAVDIITMDVSFISTTLLFPVIKELLKEDGVIVSLIKPQFEAGRDKVGKHGVVRDPAVHREVLLNCINSAQQENLYCTAVSFSPITGPKGNIEFFIQLKKEAKPCERIEE
VVEKVVEEAQQKLGGKK*
>Syntrophomonas_wolfei_subsp.metylbutyratica_JCM14075T_02583_1-deoxy-D-xylulose-5-phosphate_synthase
MDEILNTINSPRDLKKLSLPEMTQLANEIRQLLVKSVAKCGGHLASNLGVVELSLALHTVFDSPEDKIIWDVGHQAYVHKILTGRREQMSTLRQYGGISGFPKVEESEHDAFNTGHSSTSISAALGMALARDLQGQSNSVVAVIGDGALTAGMAFEALNHAGQEDSDLIVVLNDNEMSISKNVGAMSAYLNRLRTDPSYSRTKEEIESVLNRIPGIGPNLARAAGKFKDTVKYLMVPGIIFEELGFTYI
GPVNGHDLAELKAVLSNIKKMKGPILLHTITQKGKGYEPAFQKPDIFHGVGPFDVDTGTQLKKSLKTYTEIFGDFMLNRAQRDNKLVAITAAMTSGTGLFEFSRQFPERFFDVGICEQHAVTLAAGMASSGLRPVVAVYSTFLQRAYDQIVHDVALQKLPVIFAIDRAGLVGEDGPTHHGAFDFSYLRHIPNLIIMAPADENELVDMLHSAFSMEGPVAIRYPRGVGEGVRIKSERQLLEPGQSRLIV
EGQDLAIIAIGRGVSIGRDVVDILAGKGVNPLLVDARFVKPLDRRVIAGAAQKYRRLLTIEDNSRAGGFGSAIGEMLAEEGIDAELLHIALPDEFVEHGRVELLFEQLNMNPDSILERIARKWPELFSAGSRWELLKFGQN*
>Syntrophomonas_wolfei_subsp.metylbutyratica_JCM14075T_02584_hypothetical_protein
LMGYIKKDNLISGLLVAFFTFFIALLVSLGSEALVRAVNNVIVALILLLTVIFLGIFFDIIGTAATAAQMPPFNARAAKKIFGAKQAVKLVGNAHLVANFCNDVIGDIAGTLSGAIGAGIVISLVNELVFLDIILAGAMMTSFIAALTVGGKAVGKSLAVNQANNIIFRVAIILAWWEKMSGMELFKNRR*
>Syntrophomonas_wolfei_subsp.metylbutyratica_JCM14075T_02586_hypothetical_protein
MKANMEKSKIANNIPAELENSLEKIIHFGGISRNSAEQLKQGLDSFGHLQQQYANDKKIRKVREQVSPVFFELYTAVLKKVAAEGYSDPLLKMFLCFGYLDERLLLPEQINSLYQLAESIDFGRKDAIYTIDNWLVNIYKQDEEPSVNQFGHDYNEVFREKKKRGELRDTDKPQYDNDASGKLQHEIENLFKLGQRICHGSSASYFPVLYSAILPKDFTKSLLTPQKIRESVERILQVDYSVFHREIVYHHP
EIGINKHIIMKQVWPDYILVPTPGSRGVMWQELTGRVRNSSARFVLPLFCNDNLDTILLEIMANFRWELSRSTSSYVRTSSVSDSLIGEYSDYIQFFKKNRNLTEEAKEKLRLQIEKHRNNTREIFASDYKTWINFEARGLLRLNKVARQILFQHCPFSLSIRESLEKHPLYNAQISRMNNLRNREIKILTANYARLTKNGAPLDPDLEQNLLYYQG*
>Syntrophomonas_wolfei_subsp.metylbutyratica_JCM14075T_02587_hypothetical_protein
LDSRNGIGGVSPSIRIPMTMENAIRVANGEDVELEEAIRILSSLAENS*
>Syntrophomonas_wolfei_subsp.metylbutyratica_JCM14075T_02588_hypothetical_protein
MTWYVQQKIEPSKNAIVRKGIELYLEILKQETLQKTMEEAINDTMFMNDLTESMRDFEAIDREGDSLW*
>Syntrophomonas_wolfei_subsp.metylbutyratica_JCM14075T_02589_mRNA_interferase_MazF9
MVKEYQWNIFWVNLDPAKGSEQSGTRPVLVVSTEPVNRALPVVTVLSITSMKPGRKIYPTEIRLPQRESGLDMDSIAMAHQIRSISKERLGTVCGKVNNPELREQIHKTIMLYLGIS*
>Syntrophomonas_wolfei_subsp.metylbutyratica_JCM14075T_02590_hypothetical_protein
MFKSDNSINTNKKITTYIDNLFSEVGASPAYICENFTCR*
>Syntrophomonas_wolfei_subsp.metylbutyratica_JCM14075T_02591_lineage-specific_thermal_regulator_protein
MSSTISNDLIRGHTDTIILNVLRQGDSYGYEIYKKIITLSGHQYELKEATLYTAFRRMEQDHLIASYWGDESQGGRRKYYRITTRGKEVYQQNIVDWNFAKEVLDILIKGGLEDVQER*
>Syntrophomonas_wolfei_subsp.metylbutyratica_JCM14075T_02592_putative_membrane_protein
MKLNKAGMRWIRILHIFSVSIWFGTVVCILGLVIMCFFQLNKNDFLIIAPLVPKLYQKIVMPAAIFTIIQGIIYGFFSSWGFFKYKWVLLKWIFVLLIILCTGLGGISQMVSVLAKIEATHFLGGFADGGLVLLFVSLQILFMLIMIVLSVFKPRTFKPIIFPEDPGCNLKASI*
>Syntrophomonas_wolfei_subsp.metylbutyratica_JCM14075T_02593_hypothetical_protein
MQNYWNNDPGVVNDLIAQNVAMIENMEFNNDT*
>Syntrophomonas_wolfei_subsp.metylbutyratica_JCM14075T_02594_hypothetical_protein
MILDEPGKSLDAIHTHKLMIQLARLSADEKPSIILVTHEFHQDCLPRAVSRRLKASGTKLIMNY*
>Syntrophomonas_wolfei_subsp.metylbutyratica_JCM14075T_02595_hypothetical_protein
MTEYKQAELFNQSVNEIMEGKTPSQPGDIVEQQLFQLIARLKDTDFSRDNLAQARMRNRLLSRCREKDSKDYKKEGIMKKLFEQHRTVTVFGSLALIFMLTLTLVFPGTVTAVADNVGDNIIKMLKLGKYSTVIQMDDPAPAEKKELTEEQKEQLRKEEAASAKEDENYKVKYASIEEAQKIASFKVQAPDYLPNGYELKEVVGYKGSGDYINLYFKGKGRDIIIRERVMNEHTQFTYGTSGQVDEV
DISGVTGALEESNVMWEKDGVSYSLICKGFEPEEALKIARSIK*
>Syntrophomonas_wolfei_subsp.metylbutyratica_JCM14075T_02596_Beta-hexosaminidase_A_precursor
MRINRCLAGLLLIFTIFSIIVGGVARADSDPNTTAQLLAIVEKMSIEEKVGQMFMPDFRQWNGKNVCEINEEIVQVIQQHHIGGVILFRENLVDTEQTVRLVDQLQRAAGSIPLLLSTDQEGGVVNRLQSGTVMPGNMALGATGSTESCHRVGQAIGAELHALGININFAPVVDVNVNPDNPVIGVRSFGSDPQLVSDLGVSYIQGLHDAGVAATAKHFPGHGDTAVDSHLGLPSVPYDRSRLDE
VELKPFQTAINAGVDLIMTAHVTFPAIDSSMLISRLDNTPIYVPATLSAPVLTGLIRNTLGFEGVVVTDSLQMKAITDHFGPEDTVIRAVQAGTDIILMPSDLNRAYQAMLAAVKNGDIPETTIDKSVTRILALKLKLGVVEINNGKLQPGMGSRNTCRCGTD*
>Syntrophomonas_wolfei_subsp.metylbutyratica_JCM14075T_02597_putative_glycosyl_hydrolase
MAHESFADQAVADLLNQNFISIKVDREERPDIDHIYMMACQALTGSGGWPLTVIMDADKKPFYVATYLPRSSRGSLWGLLELLPRVVELWNKERESLRQAGEEISRHIIKRDAKQAGQPISEELLNRAFKQYARAFDAEWGGFGSAPKFPIPHNLLFLLRYYHFRHEEQALEMVEKTLQSMYRGGIYDHIGFGFARYSTDRQWLVPHFEKMLYDNALLAMAYLECYQLTTKEFYAGVARDIFSYILR
DMSSPEGGFYSAEDADSEGEEGRYYLWDKDEITEILGEQIQSLARTYDISQKGNFQGRNIPNLTRTPNFEEPRQEFAAARQKLFNYREKRIKPLKDDKVLIGWNGLMIAALAMGARVLGDESYLQAARRAADFVKENLRRPDGRLLARYREGESEYLAYAADYAYFIWGLIELYEAGFDSHYLALALELSEDLNKYFGDQKEGGFFFYGLDAEQLLARPKEGYDGAMPSYNAVAALNFMRLARLSSD
LSWQEKAQQAMDYFAAEIQEYPTAHSFWLLAAIFQIYPGSEVVVAAESENQATSEMLKLLNSRYDPHRLIILNESKTSEILGSRAPYLQHMPGIEGKLTAYICENFTCREPLVGMDELLESGYI*
>Syntrophomonas_wolfei_subsp.metylbutyratica_JCM14075T_02598_Phosphoenolpyruvate_synthase
MSTYIILAFQDIDKTKRMVVGGKGANLGELFRIKGIHVPDGFCISTEAFKRIIGETSSINELLDQLSLLKVEDRDKIGELGGEIRRVIEGMAIPEDIREEIARHILSRLGEKNAYAVRSSATAEDLPTVSFAGQQDTYLNIIGKEAILKHISKCWASLFTERAVIYRLQNNFDHCKVYLSVVIQKMVFPQAAGIMFTADPVTSNRKVLSIDASFGLGEALVSGLVNADIYKVRNGKVIDKKISAKKLAIYALKDGGTK
EQETPPEQQNRQVLTDEHILQLEGLGRKIEAHFGCPQDIEWCLADDTSKWDIPPGEACPLTLFIVQSRPITTLYPIPEANDQENHVYVSVGHQQMMTDPIKPLGMSLFQLTSYGPRFKAGGRLFVDVTPMLASPDSRKNLLDTMGQHDPRIKDALMTIVERGDFKKSLPKGKKEQGSIKSKAGMSWDFLGQFENNPTIVADLVKSSQTSIEELKQNIQTKSGADLFDFILEDIQRLKKIIFDPQNM
GVIITTMNASSWINEKMKEWLGEKNVADTLSQSAPNNITSAMGLALLNVADVIRPYPEVIDYLQQVNDDNFLDKLVKLDGGQVTLDAIYAYLNKYGMRCAGEIDITKTRWSEKPIALIPIILSNIKNFAPNASQRIFAQGRQEALKKEQEIIERLKQLPDGEQKAYETKRMISLMRNFIGYREYPKYGMINRYFVYKQALLKEAEQLVQAGVIHDKEDIYYLTFEELREVVRTNKLDYQIISLRKDDYKLY
EKLIPPRVITSDGEIITGEYKRENLPAEAIAGLPVSSGVIEGRARVILNMEDAVWEDGDILVTSFTDPGWTPLFVSIKGLVTEVGGLNTHGAVIAREYGLPAVAGVDNATKLIKDGQRIRVHGTEGYVEIL*
>Syntrophomonas_wolfei_subsp.metylbutyratica_JCM14075T_02599_Daunorubicin/doxorubicin_resistance_ABC_transporter_permease_protein_DrrB
MESANKHFFGDMGVMLGRSLCHISRSMDTIITVTIMPIAFMLLFVYVFGGAIQTGTDNYVNYLLPGILLIAIASGIAYTAFRLFMDKQSGIFERFHSMPIARSAVLWGHVLTSLVSNAISVVVIILVALIMGFRPSAGILSWLAVAGILALFTLALTWIAAIAGLSAKSVDGAYAFSYPIIFLPFISSAFVPTDRCRR*
>Syntrophomonas_wolfei_subsp.metylbutyratica_JCM14075T_02600_Daunorubicin/doxorubicin_resistance_ATP-binding_protein_DrrA
MKDIAVQVKRLQKSYKKIHVLKSVDFEVEKGSIFALLGSNGAGKTTIVKILTTLLKQDSGAATVNGFDVSSKPDQVRLSISLTGQFAAVDEILTGRENLIMIARLRHLKNPRQIAEDLLNRFGLIDAADRRVSTYSGGMRRRLDIAMSLIGKPQLIFLDEPTTGLDPEARIEVWKVVRELAGGGTTVFLTTQYLEEAEQLADRIAILHKGIIASGTLAELKKLFPPAKVEYIEKQPSLEEIFLAIVGKKEEK*
>Syntrophomonas_wolfei_subsp.metylbutyratica_JCM14075T_02601_hypothetical_protein
MSEFFDNYFNIKKMTESKREYRQQMARVEALPKDYQYVFKKIQGHMWMFAAGSGYDMMEIHSGLIELFEAGAADGKHVLEITGEDVAAFCDELLRSARTYTEDWREALNRDILKKLGKENDSK*
>Syntrophomonas_wolfei_subsp.metylbutyratica_JCM14075T_02602_lineage-specific_thermal_regulator_protein
VLENLTEMLKGVLEGCVLEIISRKETYGYEITRQLNALGFADVVEGTVYTILVRLEKNKLVDIEKKPSDMGPPRKFFTLNNAGRDELQKFWEKWEFVASKINQLKEEESNV*
>Syntrophomonas_wolfei_subsp.metylbutyratica_JCM14075T_02603_N5-glutamine_S-adenosyl-L-methionine-dependent_methyltransferase
LNKDIQLWETREIAAKYLSGVRGAIPLAAEQIEVMLMLVKGAKINVNSFLDIGCGDGVLAAILEHFPNAKTVLLDISEPMIESAKEKLSIYNNTDFIVCDYGRKDWLNRVSNSTPFDLIVSGFSIHHQPDVRKHELYEEFFNMLNVNGLFINIEQVLSPTKWIESIFDELFIDSLYKMHLKQGNSISRDEVAEFWFNRPDINDNILALVETQCQWLRQIGYRDVDCYFKILELAVFGGRK*
>Syntrophomonas_wolfei_subsp.metylbutyratica_JCM14075T_02604_hypothetical_protein
MELIDRLAEEDPAIGKAKRVLEQMASNPRERELYELRRKAILDRNSALYEAKMEGIAEGKAEGKVEGKVEGIAEGIAEGQASLLLRQLKKKFGYLPVEMENRVMSMPVEKLQELAEAIFDLETIDDVNSFISK*
>Syntrophomonas_wolfei_subsp.metylbutyratica_JCM14075T_02605_Putative_transposase_YhgA-like
VETRLKGESGIVLVHVENQASAEKDFAERMFIYFSRLYQKHRCRVVPIAIFTYPEKRNEPDRFKLLFPFMPVLDFHFLTLELNKHKWQDYIQSNNPVAAALLSKMGYQKKERVQVKLEFLRMLVRMELDPARMTLLTGFFETYLILNEEEERKLEEEIGKVDEKEAKRMIEITTSWEEKGRVKGRMEGRMEGRVEGKAEGQASLLLRQLKKKFGYLPVEMENRVMSMPVEKLQELAEAIFDLETIDD
VNSFISK*
>Syntrophomonas_wolfei_subsp.metylbutyratica_JCM14075T_02606_flagellar_assembly_protein_H
VLIDVPEILDPKVDLAFKKVFGSQENKSILLSFLNAVLNWTAEQKIADVKILNPYLEPENIDDSVGILDIKLQLENDDLVDVEMQIGNLGNMERRSSYYACRMFGDQNISSGRYQDLNRVIAINILDFTRIKLTKRYHTIFRLRETQENIDLTDAIEIHFIELPKLKQEIASYTNPLDRWVVFLKGGWDMELIDRLAKEDPAIGKAKRVLEQMASNPRERELYELRRKAILDRNSALYEAKMEGIAEGKAEGK
VEGKVEGIAEGKAEGKAEGKVEGQASLLLRQLKKKFGYLPVEMENRVMSMPEEKLQELAEAIFDLETIDDVNSFISK*
>Syntrophomonas_wolfei_subsp.metylbutyratica_JCM14075T_02607_Antitoxin_DinJ
MAKTPTQIRIDEDIKRQAVALFSDLGLDISSAVNIFLRQVVLHGGLPFDVKMPQYKQEVIEAMNEAREISRDTTVKGYTDIEEIFRELDS*
>Syntrophomonas_wolfei_subsp.metylbutyratica_JCM14075T_02608_mRNA_interferase_YafQ
MEYQVKWTSKFKNSYKLAKKRGLDISLFKSVIRKLQHGEQLDERYRDHELKGEYKGLRECHIKPDWLLIYLIEDEMLTLTLIDTGSRFI*
>Syntrophomonas_wolfei_subsp.metylbutyratica_JCM14075T_02609_hypothetical_protein
LLESLITSKTRLKLILKFFLNPETTAHLRGLATEFGESTNGVRVELNRLTEAGLLESKQKGRSINYRVNKKHTLFPDIQNIVKKYLGIDEIVESIIKELGNVEVALITGDYAQGIDSGLIDLVLVGDIDKPYLQQLVEKAEELINRKIRTLVLDQEEFAKLRDKFEKEKTLLLWRSEE*
>Syntrophomonas_wolfei_subsp.metylbutyratica_JCM14075T_02610_hypothetical_protein
MITLKSVKSLKSNITKKNAELAMSNSLDTPNASMQHLEEQT*
>Syntrophomonas_wolfei_subsp.metylbutyratica_JCM14075T_02611_hypothetical_protein
MMSIRKKIGNDGSIVIEEALKIAKIRPGDWVKITPTDNKIIIKLTTHKKQKGVVRAAAGILKDSNLVEDMLRTREVEDDRQGSII*
>Syntrophomonas_wolfei_subsp.metylbutyratica_JCM14075T_02612_PIN_domain_protein
MIDREVSYKKKYLVDTNIVIYTLKGEKEAVEAMESFEDEMIEIFYSTIIEAELFSFHQLTKNQKFKIGQLLDLGEIIDVDSEIALKAAELRALSSKQYNRKLKLPDALIAATFPRR*
>Syntrophomonas_wolfei_subsp.metylbutyratica_JCM14075T_02613_dTDP-glucose_4,6-dehydratase
MNLKNKKILITGSDGFIGSHLTEALVRMGCNVRAVVYYNSFNSWGWLDDSPEEIKKELNVFAGDVRDPHGMKNAMQGCDVVFHLAALIAIPYSYHSPDTYVDTNIKGTLNILQAARELGIEKVIHTSTSEVYGTARYVPIDEEHPLQGQSPYSATKIGADQIALSFYRSFDTPVSIIRPFNTYGPRQSARAVIPTIITQIAAGQRELRLGSLHPTRDFSYINDTVKGFIAVAESENSLGEVINIGSNYEVSIAD
LVQTITAIMKADIQINTDDNRIRPEKSEVERLWADNKKAAHLTGWQPQYAGMEGLKRGLQETIDWFSDPNNLKRYKTGVYNI*
>Syntrophomonas_wolfei_subsp.metylbutyratica_JCM14075T_02614_Putative_pyridoxal_phosphate-dependent_aminotransferase_EpsN
MNGAVKEFVHSIINTLESVLPSKEYIPLHEPEFSGREWEYVKDCLDSGWVSSVGAYVDRFEQMLCDYTGSPYAVAAVNGTAALHICLLLAGVKAGDEVLVPAATFIATANTVTYCNAIPHFVDVEERTLGLDPHKLNDYLKEISEFRRGICYNRLTGRPIRAVVPMHTFGHPVDLDPLQELCERYHLVMVEDAAESLGSLYKGQHTGTRGLVSSLSFNGNKTITTGGGGAILTNSKSIAKVAKHMTT
QAKTPHRWEFNHDLIGYNYRMPNINAALGCAQLEQLPGFIEKKRNLARCYQVAFQDIPGIRFFTEPDFARSNYWLNVLILDEEFARERDNLLESTNNAGIMTRPLWTLMHKLSMYHDCPRMDLSVAEDLESRVINIPSSAYLGK*
>Syntrophomonas_wolfei_subsp.metylbutyratica_JCM14075T_02615_Demethylmenaquinone_methyltransferase
MTKNLSYKERENALRIRIEAHKKYSTNPLDKWLEINLNLKEGGYLLDLGCGTGNFFPIYADKLQNNGLIVGVEKELSLIKGAQSLNLLTPKILLNMDMDGYWPFTEGTFDYVISTFAIYYVDSSKKVLENIYKILKKRGEILLIGPSVNNAIELYDFNHDLFGIKHTEEAMERSKRLKDEFYPLIKQLFGEVTYSLIDNKVVFPNKFEFIEYYRATPLFEGSCEINKYKPQYKEMLEAVQAESPIITKEMSVVW
GRK*
>Syntrophomonas_wolfei_subsp.metylbutyratica_JCM14075T_02616_Polysialic_acid_biosynthesis_protein_P7

MGEKINILVFTGNRAEYGLLYPILYNLKLDKTYRVSLMISGSHTTELFGSTLDEIDKNGFESIEIIDLGDFTSFNNINTIKVISKIIETSSAVFNNIKPDIVLVAGDRYETYAVVTSAFYNNIPIAHFFGGDLSQGGHLDDSARHAITKLSHLHFATNEDSYNRIIKLGEEEWRVFNVGSTFVENILRNDYSTPEELKNDLNIDLEKKIVLFTQHPLTTEPQKAYDQIKISLQVLADLGIQSIITYPCNDNGSEQMIK
AINEFSNVSHFRIVKSLGMKKYLGCMSIASCVAGNSSSALTESIPFGIPCVNIGKRQEGRLQSENVLNVEQDYIQIKKAIQKALYDEEFISIARNCHNPYGDGKTSIKIVELIKILMSDKKQSLLKKRMTY*
>Syntrophomonas_wolfei_subsp.metylbutyratica_JCM14075T_02617_bacillithiol_biosynthesis_deacetylase_BshB1
MQEIRAKKCLVVAPHPDDETLGCGGTLLKHKASGDKIHWLIITGIDKEAGYSAERISARSQEIKAVSEMYGFELVVNLNLPTTKLDIIPMQQLIEMIGDVIIKIKPEILYVPFPGDIHTDHKVVFQAVAACSKWFRFESIKRILAYETLSETDFAISPDINDFTPNVFVDISEFLQEKLQIMQTYKSEMGLFPFPRSEKTIIALSQIRGAAAGCEAAEAFMLLKEII*
>Syntrophomonas_wolfei_subsp.metylbutyratica_JCM14075T_02618_Pseudaminic_acid_synthase
MNNVFIIAEAGVNHNGSLDMALQLIDAAVNAGADAVKFQTFKGEKLVSCFASKAEYQTRNTGTAESQLEMIKKLELDEEQHMILAERCMESGIEFMSTPFDSDSVDFLANVVNVSRLKISSGEVTNGLLLRFAQTGKSLILSTGMSTLDEVETALGVLAFGFINSKEKPSIGAFTEAYSSREGQSFLKEKVSLLHCTTEYPAPFEEVNLKAMDTLRDKFSLPVGYSDHTMGIAVPIAAVARGAVIIEKHF
TLDRNLPGPDHKASLEPGELKQMISSIRQIELALGNGVKQPTTSERKNMVVARKSLVATRDIKSGEMFTEANLGVKRPGTGLSPFEYWSLLGKTADKDYKADEVIK*
>Syntrophomonas_wolfei_subsp.metylbutyratica_JCM14075T_02619_Bifunctional_polymyxin_resistance_protein_ArnA
VKIGYFADGPWSHYALEKIIKDPRLKIVFIVARYPQPDEKLRLYSEQLKVPFLVDLNVNSVEFIDIIKGCAPDINVSMSFNQILKGDILKVAPLGFINCHAGQLPFYRGRNVLNWALINGESHFGVTVHYIDIGIDTGDIILQKLEPIENTDDYQSLLNKAYRLCGETLYDALLMLQQGDNQVIKQSSIHPVGFYCGRRIIGDEYIDWNWNSRLIYNFVRAIVPPAPGARTFLNDSEIVILKTELIDNAPTYIGV
PGEVIGTNDKGVVIKTGDSTIQITQIGELDQNGELINIRIPLMKIGTRLGFFNPWIELRNLRQKVNQLQIMLNTEE*
>Syntrophomonas_wolfei_subsp.metylbutyratica_JCM14075T_02620_Putative_acetyltransferase_EpsM
MAGAVIQALVTIGVNTIINTRASVDHECLIGDHVHIAPGTTISGGVKIGDNVLIGAGATIIQGIHIGASSIVGAGAAVTKDVREGVIVMGIPARVVKYS*
>Syntrophomonas_wolfei_subsp.metylbutyratica_JCM14075T_02621_N-acetyl_sugar_amidotransferase
MRYCTKCILPETHQTIKFNQEGVCSVCEQIDYRDTEIDWDERRCMLQELVNEYRDKGEYDCIVPFSGGKDSTFQLWYVVKELGLKPLVVRFDHWGYRPKVAENNTRTFKLLGVDVLQFTPSWHVVKELMLEALKRTGDFCWHCHTGVYAHTMQAIRYNTPLIIWGETLAEYASWYSYDEFEEVDETRFNRVMNLGITADDMYEFLNGKVSKRDLYPFAYPPRDELMKIKCRSICLGNYIKWD
IKKQVEIIKREIGWEGQEVEGIPREYEYEKIECQWQGIRDYCKYIKRGYGRTSHLVSIDIRNKRLTREEGWALAEKYDGKRPASLDIFLEKLGLSEEEFVNILSRHRIEPWQFDQEQVEPGQPLPDMGQWTRG*
>Syntrophomonas_wolfei_subsp.metylbutyratica_JCM14075T_02622_serine_O-acetyltransferase
MRMSLTRERLLSYLCAQLNSFFPDGSLVKPEHMENSFDHILKRVEYCFSQVNNKYFRSDGKAVFNHLNGDQYAMFLYFAANTVYKDNNHVELATKIFLLNKYLHGIDVFYEVELPNIFIFVHPLSTVLGRGNYSNYFVVYQRCNIGSNHDIYPCMGENLIMHPGSAILGNCKVGDNCRLATGSLLLDMDLEENSIYIGNPSNYFKKAEALPNIWL*
>Syntrophomonas_wolfei_subsp.metylbutyratica_JCM14075T_02623_Glucose-1-phosphate_cytidylyltransferase
MSKKEYGKFIIPKGTTLKAMEQLENAASRNLYVVDENNLQLGSLSDGDIRRALLNGANMDASIDAYMNKTPRFIEYIDREKTKHIKALMLENIETIPVLDNNRVVVGIITWIDLFSSHKAVAYQPKSNLVFILAGGTGSRLDPFTRILPKPLIPIDDQPIIEKIMDKFSFYGFDSFILSLFYKSDMIRAYFSDKDVIRKYQTVNFIQEEFPLGTIGSIDFARSKLTDSFFISNADILIEADLNSILHYHQDSGAIL
TAVGCVKNSVYPFGVQTDERGELIVNIEEKPQYQLFVNTGVYLIEPETLSLIPKNSFIDMTDVLKSMLTAGERIGVYPIRECSWLDVGQWDDYLHHGIEYLERTRKYFELRK*
>Syntrophomonas_wolfei_subsp.metylbutyratica_JCM14075T_02624_N-acylneuraminate_cytidylyltransferase
MNTLFLITARGGSRGVPKKNICKIGGLPLLAYKAIAARKSNYCTRLIISTDSEDIAAVAREYGVEVPFTRPAELATDSANSMDVILHAMDWIEKNDPKVDAICLLEPSTPFSTPDDVNQAILLYQNKNALGLVGVKQVKDHSIFVSPIGDDLNMARHYEKMRGNYHTPRQVLIHEYTMNGAIYLAAWDYLLKEYHTFHSPRTYAYIMPEERSIEIDEMNDLYYAIFLVEKGLIDINWK*
>Syntrophomonas_wolfei_subsp.metylbutyratica_JCM14075T_02625_Glutamate-1-semialdehyde_2,1-aminomutase
MSINRYYQEMAHRYIPAGAHTYSRADDQYPVNAPPVLERGKGAYWDLEGNRYLDYGMALRAVTVGYDYERISNAIEQIHNGNNLTRASKIEIEAARDICELIPWVDMVKFAKNGSTVTSAALKLARAYTGKKYVARCPQHPFFSYDDWFIGDTVMNAGIPEEYKSLTLNFNFNDLASVREALEQFKPQGIAAVILEPATTEEPKDNYLNRLKDLCHRNGAVFIFDEMITGFRWHLQGASAYYGVQ
PDMSVFGKAIGNGFAVSALAGKREYLEAGSIQHHGERVFLLSTTHGAETHSLAAAIEAMKIYREQPVIQHLYQQGERLIKLGLEIIKEMNLEDYFHILGKTPCSLIFGTKDQEKNPSLPFRTLFMQEMIKRGVLTPSFVVSYSHTEEDIEKTIEAVEHALYVYRKALEEGVEGYLLGRPTKPVFRKWN*
>Syntrophomonas_wolfei_subsp.metylbutyratica_JCM14075T_02626_3-oxoacyl-[acyl-carrier-protein]_reductase_FabG
VNKYISRFSLDNKTIIITGACGLLGREICKALAELHARIVLADIDIMAAEKLQEELRDVYGASVTAFPLDISSEDSVNKLVDELSKQEVSIDGLVNNAYPRNITYGTIFENITMDSWRENIDMHLNGYFNVSQKIARVMMQQRQGNIVNITSIYGMLGPDFSIYEGTTMTMPAEYAAIKGGIINFTRYLATYLAAYNIRVNCLSPGGIFNEQLVSFVEKYKQRTPMGRMGKPEDIAGGVLFLLSNLSSYMTG
QNLVIDGGWSAW*
>Syntrophomonas_wolfei_subsp.metylbutyratica_JCM14075T_02627_hypothetical_protein
VIDKNSRMNKINKIIFFISSPFNQRDYSRFGIETLKKNGFDVEVWDFTPIVYPQVEGVVIPNDPVNYDKHHKFEYKKDVILAVGKLKNVFVINFCPYTPAALFIYRALSRFKIPYSVFMANAIPFGQKRKLNIAWFYSKIKKFTFRKLFGFIFSLIPSSYLGVHPATFILAGGEKSLIYKYPVSKKTEIIWLHTLDYDIYLEEKEYVETSNNTAVFLDEYLPFHEDYFFLSNTPYSTADKYPALCNFFDYVEDKCDV
ELIIAAHPRSHYEQLPDYFNSRSVIRGDTINLVKKSRFVIAHASTSINFPVLFRKPIVFITTNELNNSPEGYAIRSFAGLLKKTPINISEKFPWEIVAERLYNYDRT*
>Syntrophomonas_wolfei_subsp.metylbutyratica_JCM14075T_02628_Polysaccharide_biosynthesis_protein
MTEHNDSLGKRKYKLLTNLLGLLNITGIAIPRGLLPGKAYGDFNLTNFPFNIQIGHLDMGTSIAFYTKLSQRPKENGLVVFYLYFSALASGITLIFVLITHLSNSYFSIWPGGIFYIYLAAIWGLLTWFIQVLNKMTDAYGLTVPSIARMIQKLLGLILVILYFIHRLNLVNFFYYNFAILIFLVIGFIWIEKSNYSIAENWRINSEQVRKYFYEFYEYSAPLIPTFALVGLIVGILDRWLLQQFAGSEQQGFFSLSF
QIGTVCFLFTSAMTPLLMREFSIAYANRDIILMAVHYFRRYIPMLYSIAAYFSCFIAVQASTVVHFFGGEYQGAIIAVMIMAFYPIHQTYGQLSGSVFYATGQTRLYRNIGILFMVLGLPLTYFLIAPEGYLGLNAGATGLAIKMVALQLVGVNVQLYYNAKFLGLHLNKRYLGHQIVSVGSMLVIAWGTVVSINQISWMNQHMLVSFLTTGFIYSLMVMGLVYKYPILFGLNREDIVALVSVREQLNN*
>Syntrophomonas_wolfei_subsp.metylbutyratica_JCM14075T_02629_2-keto-3-deoxy-L-rhamnonate_aldolase
LLKERLSKNQFTIGSWILTLAHTAIAEIMCQAGFDWLTIDMEHSVIELAQAQELIRTIESYGVTPLVRVGENNPTIIKRVMDAGATGIIVPMVNSRADAERAIESIHYPPSGKRGVGLARAQKYGLGFEEYKLWLAKESIVVVQIEHIKAIENLIEILSTPGIDASIIGPYDLSGSLGCPGEFGNERVVEAIQKVCHYLGKPMGFHIVEPDICQANVMKKQGYNFLAVGLDTLFLGLKCRETLHGIYE*
>Syntrophomonas_wolfei_subsp.metylbutyratica_JCM14075T_02630_3-deoxy-manno-octulosonate_cytidylyltransferase
MYMMNLKTYILLVPMKNLSQYGIPARMASSRFPGKPMADLQGLPMIAHVYQRSKMSSTLEYVYVATCDEEIVDYIESIGGQAIMTSDTLHERASDRCAEAMLKIEEQTGRKIDIVVMIQGDEPMLFPEMIDEAVSPLTENKDIQVVNLMAPLSNREEHEDPNEVKVVVDLYNNALYFSREPIPSWKKGASSLPMFKQVCIIPFRRDFLLKFNELTPTPLETAESVDMLRVLEHGYKVRMVPSNFETYSVDT
NEDLLKVQELKNDPLLKLYIRNYGKG*
>Syntrophomonas_wolfei_subsp.metylbutyratica_JCM14075T_02631_3-demethylubiquinone-9_3-methyltransferase
MREEDIRRKDVFNRYLDLSRKDAENFFANHNRFIDIQCPACNSGNYQYEFDKYGFTYVSCPVCHTLFVNPRPAFDDLIRFYADSPSTHYWVEEFFKPVAEVRREKIFRPRAEYVTEVLPNLNNGVIGDIGAGFGIFLEEIRRLWPSRLIAIEPSPQAEICLGKGIEVQCCALEELQGFENEFDLLTCFELFEHLHTPVDFVQRVFKLLKPGGHFLMTTLNGQGFDIQLLWDKSKSISPPQHINFFNTTSITL
LRQCGFQIVDVSTPGKLDWDIVEGMIKNEETSLERFWHLLSRQGNETLCKNEFQDWIARNKFSSHMRVLARK*
>Syntrophomonas_wolfei_subsp.metylbutyratica_JCM14075T_02632_(S)-sulfolactate_dehydrogenase
LAEEIEEMINGQYDALVGTKEVRKSISYKCPSLKVISRVGVGYNNIDVEYFKKHGVTITYTPFGPTDSTAEMALAFILAGTRKLRNYDSLMRQGIWRREFGLRLKDATVGIVGFGRIGKRLATLLYAFGCTILLHDIKPDLATAHAMGWEFVGKEEILEKSDIISFHVPLKDNTRDWISYDELQLLKHPVTLVNTARGGIVNEQAIYEYLEKEQPDSYYCCDVFEDEPYKGNLIELGNTLLTPHASSFTE
GSRRQTELLAIDNCIRVLRGEPCENIVERNE*
>Syntrophomonas_wolfei_subsp.metylbutyratica_JCM14075T_02633_Kynurenine_formamidase
MRRIEYLSYPLEQNNPVYGKPDQPMITNPVKSIESGQSCNVSYFAMENHWGTHVDQPAHFFSNAPGIADYPAEFWLFSNPQVINIKVKAGQIISPGDLLEIVAETTDLLILKTGWYKYRKNSCYCLENPGISPELGTLLRKKRKNIRAIGFDFISLSSYVNRMIGRQAHQVFLNPDSEGNPILIIEDMCLGADLKGLKEVWVAPLRLSSADSSPCTVIGVFND*
>Syntrophomonas_wolfei_subsp.metylbutyratica_JCM14075T_02634_Phosphorylated_carbohydrates_phosphatase
MINTIIFDFDGVIIESAEIKTVAFRKLFENDYPEEVEEIVNYHINNMGISRYVKFRHIFRNIIMQPLTLKQEEELGQRFSTIVFEEVLKAPIVSGLLEFLEEYYKTIQLFIASGTPEDELHTIVKHRGLGKYFQGIFGTPKTKAEIIFHIMNTCDISMADIVFIGDAMSDKQAAEETGVHFIARISGGNELDSPCYKIRDYFAFSEILKHL*
>Syntrophomonas_wolfei_subsp.metylbutyratica_JCM14075T_02635_CDP-paratose_2-epimerase
MKAIVFGGSGFLGSHVADVLSGRGYEVIVFDLQSSPYLRDGQKMVGDILDEEAVKAATTGCDYIFNLAGIADLDDATTRPIETVKLNVKGNLNMIEAALQVPAKRYVYASTIYVYSQKGGFYRCSKQASEIYIEEYNNRYGLDYTILRYGTLYGPRADNRNSIYRYLKQALEDNVVRCRDTGDEMRDYIHVRDAARLSVDVLGEEYRNQHIIISGTMRFRDMLLMIKEITGKDKMEIRFSNKKDD
HYAYTPYSAPYKGSKLVNHLYVDMGQGLLECMLLDMDPNGKVGIT*
>Syntrophomonas_wolfei_subsp.metylbutyratica_JCM14075T_02636_hypothetical_protein
MKVLLVFDSHKLYPWDKLECRHLDWFGCEMVEPLTSNSDINQVVSLEGQLNPIINLLSGEIVNYKAQNLRDEYIEWAAEIGNCKIGKSKLREILLMSLSDGNVSAWWFSLLADKDPAKSNAFRVIQTAVIDQAQNGHYDLVILALKDGNFGEKLKLVLSSECIKCQLWRVNKERLFYRVKDKVKQNGFIRAAWALLTICGRLHLRIKLPSLKQRNLETENALLFSYFPYVLPKEAQEGIFRRNYILPLQDIYH
EHGKPIVSGLIYTYIDNYSFADAVLAKRFIHGEPLFYEEFLSKLIANAFIEYMRIRAWSKVSNKLSEVLSEKKPGNDFALPFLLELLNQSFTGFYLIQGILQYYAFNNMFRKLNNQTCLYYSEMMNWEYALIAAKRYSDSGITTIGYMHSSLSRNYLQMYHSQKELEDKNTPAGIPMPDIFACNGKGPKRMLSQYYQDIVELAYLRIYHIYEYLQFDHSKQQPQRKDKFILLVCTTIDAKESTIVDL
VVATFWGNQDGVEKFWGKGHPLSVDSFMPQTGAQYGDTPNYEIKYEAIADLMPECDAVLGASTVAIEALAFGVAVVIHGTNLQNSPLIGYNECYHEIYDQASFREAIDNIRTQKEHDTLIQKLEFVKEWSYSDHPLNAWKQLLGFKMIDS*
>Syntrophomonas_wolfei_subsp.metylbutyratica_JCM14075T_02637_UDP-glucose_4-epimerase
LGKVLVTGGAGYIGSHVVKALGEKGYDIIVYDNLSTGNQWAVLYGELVIGDILDIMMRKVFINNKIDIVVIHLAACIIAPESVEQPLKYYMNNVCGTTLNLLQVMAETKVNKFIFSSTAAVYGIPQEIPVTENSAIAPISPYGKSKAIIEKIINIDAKADLQYTTLRCFNVAGADLYGRIGEGKPQSTHLITSCVRTALNPISCLNVYGNDYPTTDGTGIRDYIHVDDLASAFTMSLQYLLDGGKSLVCNCGY
GRGYSVLEVMEATKKLTGVNINVAFKERRLGDPPILIADSQKLKQSLNWQPRYNDLHCIIESAIRWEQKIMKNGNFVKC*
>Syntrophomonas_wolfei_subsp.metylbutyratica_JCM14075T_02638_UGMP_family_protein
VIVLGVNFETTHDSVALIKDNEILFAAIERFSRIKNDFQYPLEAKNFVIGKIKTQGLIDIPIDFKINNIVAISGFPPMRNWVVYAKSFIVRKTFCKGKDFKIFVRRIKGEKLVFKRGGALIVNSLFCTGVPQFLFLYLTKRIRVGHLLKGFKGKFVYTPHHYGHAASACFTSGFSDALSIVIEGYDWENSIVIDEFENGNFKQLTKTSWPHSPGMFYELATTILGFDYLKHAGKITGLAAFGDPNVAYDKVAALIWSNGV
TLEVNPLIYGFIQDYLSNQIPGYFANCLKEDIAAFQKRLEDVLIDLLKNALKITNKRKIVLSGGVAANVLLNQKIHEIGEVEEIYIHPAMSDAGQALGAAQYAYNQELKKQGKYLPKSCLKNVYFGSGYSDEEIKYCLDKNIKYCYYDDIERIARLLADKKIVARYNGRMEYGPRALGNRSILFHAGDKTANDWLNSRLKRTEFMPFAPSVLMEDREYFLNGEYTAEFMTITFNCTNKMKQE
CPAVVHIDGTARPNFVSKENNPSYYRIIKEYKNITGIGVILNTSFNMHEEPIVCTPSEAIRAFKQSGLDYLAISRYLVQAQ*
>Syntrophomonas_wolfei_subsp.metylbutyratica_JCM14075T_02639_Methionyl-tRNA_formyltransferase
MNLNLKFLLIVQDESELVPIMLSRVLDEFRENVIGVNMISPKHLPHPLRTIRHARNKLGVFEPVRTAVKQIFSSKSVKSICLKNNCNITSYIPNSPKFREIIRNYEVDILISLACPIILCRETIEAPNLCSINVHGSYLPDFRGVSTVFWAMLKGADRTGTTVHKMTTKIDSGDYFYRESIPITQESTLFGLYKNVADKGGELLVRTLHDACNDKMKTYPIPHGGQYVSFPQWTDGLEFRRKGLKYR*
>Syntrophomonas_wolfei_subsp.metylbutyratica_JCM14075T_02640_putative_polysaccharide_deacetylase_PdaA_precursor
MINALSIDFEDWYQASFVNIPSKEWVNCRPAIEATDLILDLFAIHAIKATFFVSGYIAQRHADLIKTIHRAGHEIESHGFWHQLAYKQTPEQFAEDISMSSTEITSSVIEVGKPIGYRSPEWSITTHIPDQAAKILLENDFIYDSSLFPAKNFFGYSNNNFKTDIHYYKDTLNLEVPPSTLSLAGLRIPVTGGFYLRFYPIPVMRKLIELVNTSKPVQLYFHPWEVLNSYPKRSMPLTQRFIQYYRCGSMLVILNELSKR
CQFGSLRQAYPEINQAIINRHN*
>Syntrophomonas_wolfei_subsp.metylbutyratica_JCM14075T_02641_Methyltransferase_domain_protein
MGYTPRSKQQLKIKDFTFIPRAVHIFEDKWGLISKAEHFDYVNLHKKGEEVQQVLSCEYLDVCGGCRYNRFINKYLEHGIGIDVFKYEGLTEENLVEIDKKFPFNETFDTVTFIANINHIPPSMRDNELSEAYRCLKYGGNVLVTIANPVAGIAVHKVVRFHDIVRLFGTNYDMDHARGMAEGEEYYLKDKEIVSRLAKAGFINIRRKYFWTQWFLNHMFIGYK*
>Syntrophomonas_wolfei_subsp.metylbutyratica_JCM14075T_02642_Poly-beta-1,6-N-acetyl-D-glucosamine_synthase
MDDIIIIIPAYNEAKNIFELVKTIKEYGLNLVVIDDCSSDKTASLALMAGAMIVKHPTNMGYGVALQTGYKYALSCKYKYLVQLDGDGQHNPQDILTVLQPIIDGEADLCIGSRFLDSQEYKPPYLRRLGMKLFKKILKVLTKSITDPTSGYQAMNSKIFRYLSLDTFPDDYPDADVIYML...

MIALVLIGFGMVALIQIPGLVRKQWWRELLCFAVLLFMGLILSVMISMGIEIPPVTTIINETITGMLGM*
>Syntrophomonas_wolfei_subsp.metylbutyratica_JCM14075T_02691_HTH-type_transcriptional_activator_CmpR
LTIRHLRILVAVYDELNMTVAARKLFMTQPSISQAIKELENYYGVVLFERLSRKLYVTEAGEKAYQYATHIIKLFDELQDNLKENASQKKLVIGANYTVGAGLIHKYIDNFKGLYPNSEIMVIVNNASILIE[illegible]
LPVKGMDLSRKLTIVYHKNKFISRAMQDFIRICHEP*
>Syntrophomonas_wolfei_subsp.metylbutyratica_JCM14075T_02692_Molybdenum-pterin-binding_protein_MopA
MNISARNQLKGKIVSIQEGTVNGMVALKTDGGDTFTSTISKEAVKELGLAPGKEAIAVIKATEVMIGLGEMKLSARNQLAGEIVNVQEGAVNAIVTVKTDGGSTLYATISMLGIKELGIAPGVKAKAV[illegible]
>Syntrophomonas_wolfei_subsp.metylbutyratica_JCM14075T_02693_hypothetical_protein
MRKAFFILAAAMTLLLSTASAIAAMDLPQPDRNFYCLDQADMLSEESENAIINTSKNLQQKTSAQIVVVTVPTIGEDAVLEDYSLELFRQWGIGDKEKNNGVLLLIAQKERKSRIEVGYGLEGVLPDG[illegible]
AGGGGSSRGW*
>Syntrophomonas_wolfei_subsp.metylbutyratica_JCM14075T_02694_LemA_family_protein
VKNRTLLIVLAVLLIMLVAGISTYNGMITANERIDNKWGDIDTQLQRRSDLIPNLVSTTKAYAAQEKEIFTQVAEARSKLAGANSVGEKAQADAELSTALSRLLVVVERYPALKSDANFRQLADELAG[illegible]
>Syntrophomonas_wolfei_subsp.metylbutyratica_JCM14075T_02695_Cellulosome-anchoring_protein_precursor
MAIRRKWIVFITMLIAALAIMFFSPTQVLADTDIKVRICDPDRINDEGDAQEGNTINQVLSAPTVSYGENRELGSVQITGKAGVAVPLSEGQKIMVILPWGISYMQVPDESNYRNYVEWPEQFEGKKN[illegible]
AGGVLQPEQSISRAEAAHLAGTLFGVAASEPAPFKDSLPEWAQKGINKAYAARIIQGYPDGSFQAGNPLSKAEAIIILQNCLERYSKFPLLN*
>Syntrophomonas_wolfei_subsp.metylbutyratica_JCM14075T_02696_putative_nucleotidyltransferases
VEKSILVQLQKDRDLIKQIAGKHGARAIRIFGSVARKEEQADSDIDLLVEFDENASLFDLINLKLDLEEIYNRKVDIVTPDSLHKKIAVSIMNEAITL*
>Syntrophomonas_wolfei_subsp.metylbutyratica_JCM14075T_02697_hypothetical_protein
MKNDLLYIEHILTSINKIEQYSSSGKMLL*
>Syntrophomonas_wolfei_subsp.metylbutyratica_JCM14075T_02698_hypothetical_protein
[illegible]
>Syntrophomonas_wolfei_subsp.metylbutyratica_JCM14075T_02699_hypothetical_protein
[illegible]
>Syntrophomonas_wolfei_subsp.metylbutyratica_JCM14075T_02700_hypothetical_protein
[illegible]
>Syntrophomonas_wolfei_subsp.metylbutyratica_JCM14075T_02701_hypothetical_protein
[illegible]
>Syntrophomonas_wolfei_subsp.metylbutyratica_JCM14075T_02702_hypothetical_protein
[illegible]
>Syntrophomonas_wolfei_subsp.metylbutyratica_JCM14075T_02703_hypothetical_protein
[illegible]
>Syntrophomonas_wolfei_subsp.metylbutyratica_JCM14075T_02704_type_II_restriction_m6_adenine_DNA_methyltransferase,_Alw26I/Eco31I/Esp3I_family
[illegible]
>Syntrophomonas_wolfei_subsp.metylbutyratica_JCM14075T_02705_PglZ_domain_protein
[illegible]
>Syntrophomonas_wolfei_subsp.metylbutyratica_JCM14075T_02706_hypothetical_protein
[illegible]
>Syntrophomonas_wolfei_subsp.metylbutyratica_JCM14075T_02707_PD-(D/E)XK_nuclease_family_transposase
[illegible]
>Syntrophomonas_wolfei_subsp.metylbutyratica_JCM14075T_02708_hypothetical_protein
[illegible]
>Syntrophomonas_wolfei_subsp.metylbutyratica_JCM14075T_02709_2-dehydropantoate_2-reductase
[illegible]
>Syntrophomonas_wolfei_subsp.metylbutyratica_JCM14075T_02710_Long-chain-fatty-acid--CoA_ligase_FadD13
[illegible]
>Syntrophomonas_wolfei_subsp.metylbutyratica_JCM14075T_02711_Inner_membrane_protein_YbaL
[illegible]
>Syntrophomonas_wolfei_subsp.metylbutyratica_JCM14075T_02712_hypothetical_protein
[illegible]
>Syntrophomonas_wolfei_subsp.metylbutyratica_JCM14075T_02713_aspartokinase
[illegible]
>Syntrophomonas_wolfei_subsp.metylbutyratica_JCM14075T_02714_hypothetical_protein
[illegible]
>Syntrophomonas_wolfei_subsp.metylbutyratica_JCM14075T_02715_Endospore_coat-associated_protein_YheD
[illegible]
>Syntrophomonas_wolfei_subsp.metylbutyratica_JCM14075T_02716_Ribosome-binding_ATPase_YchF
[illegible]
>Syntrophomonas_wolfei_subsp.metylbutyratica_JCM14075T_02717_hypothetical_protein
[illegible]
>Syntrophomonas_wolfei_subsp.metylbutyratica_JCM14075T_02718_putative_3-hydroxybutyryl-CoA_dehydrogenase
[illegible]
>Syntrophomonas_wolfei_subsp.metylbutyratica_JCM14075T_02719_6-phosphofructokinase_1
[illegible]
>Syntrophomonas_wolfei_subsp.metylbutyratica_JCM14075T_02720_Putative_serine_protease_HtrA
[illegible]
>Syntrophomonas_wolfei_subsp.metylbutyratica_JCM14075T_02721_Endospore_coat-associated_protein_YheD
[illegible]
>Syntrophomonas_wolfei_subsp.metylbutyratica_JCM14075T_02722_Endospore_coat-associated_protein_YheD
[illegible]
>Syntrophomonas_wolfei_subsp.metylbutyratica_JCM14075T_02723_hypothetical_protein
[illegible]
>Syntrophomonas_wolfei_subsp.metylbutyratica_JCM14075T_02724_Modification_methylase_DpnIIA
[illegible]
>Syntrophomonas_wolfei_subsp.metylbutyratica_JCM14075T_02725_Type-2_restriction_enzyme_DpnII
[illegible]
>Syntrophomonas_wolfei_subsp.metylbutyratica_JCM14075T_02726_Modification_methylase_DpnIIB
[illegible]
>Syntrophomonas_wolfei_subsp.metylbutyratica_JCM14075T_02728_Phosphoribosylaminoimidazole-succinocarboxamide_synthase
[illegible]
>Syntrophomonas_wolfei_subsp.metylbutyratica_JCM14075T_02729_hypothetical_protein
[illegible]
>Syntrophomonas_wolfei_subsp.metylbutyratica_JCM14075T_02730_Exodeoxyribonuclease
[illegible]
>Syntrophomonas_wolfei_subsp.metylbutyratica_JCM14075T_02731_antiporter_inner_membrane_protein
[illegible]
>Syntrophomonas_wolfei_subsp.metylbutyratica_JCM14075T_02732_Sigma-W_factor
[illegible]
>Syntrophomonas_wolfei_subsp.metylbutyratica_JCM14075T_02733_hypothetical_protein
[illegible]
>Syntrophomonas_wolfei_subsp.metylbutyratica_JCM14075T_02734_Elongation_factor_G
[illegible]
>Syntrophomonas_wolfei_subsp.metylbutyratica_JCM14075T_02735_putative_hydrolase
[illegible]
>Syntrophomonas_wolfei_subsp.metylbutyratica_JCM14075T_02736_Mg-protoporphyrin_IX_methyl_transferase
[illegible]
>Syntrophomonas_wolfei_subsp.metylbutyratica_JCM14075T_02738_hypothetical_protein
[illegible]
>Syntrophomonas_wolfei_subsp.metylbutyratica_JCM14075T_02739_6,7-dimethyl-8-ribityllumazine_synthase
[illegible]
>Syntrophomonas_wolfei_subsp.metylbutyratica_JCM14075T_02740_Riboflavin_biosynthesis_protein_RibBA
[illegible]
>Syntrophomonas_wolfei_subsp.metylbutyratica_JCM14075T_02741_Riboflavin_synthase
[illegible]
>Syntrophomonas_wolfei_subsp.metylbutyratica_JCM14075T_02742_Riboflavin_biosynthesis_protein_RibD
[illegible]
>Syntrophomonas_wolfei_subsp.metylbutyratica_JCM14075T_02744_Ribulose-phosphate_3-epimerase
[illegible]
>Syntrophomonas_wolfei_subsp.metylbutyratica_JCM14075T_02745_Putative_ribosome_biogenesis_GTPase_RsgA
[illegible]
>Syntrophomonas_wolfei_subsp.metylbutyratica_JCM14075T_02746_Serine/threonine-protein_kinase_PrkC
[illegible]
>Syntrophomonas_wolfei_subsp.metylbutyratica_JCM14075T_02747_Serine/threonine_phosphatase_stp
[illegible]
>Syntrophomonas_wolfei_subsp.metylbutyratica_JCM14075T_02748_Dual-specificity_RNA_methyltransferase_RlmN
[illegible]
>Syntrophomonas_wolfei_subsp.metylbutyratica_JCM14075T_02749_Ribosomal_RNA_small_subunit_methyltransferase_B
[illegible]
>Syntrophomonas_wolfei_subsp.metylbutyratica_JCM14075T_02750_Putative_neutral_zinc_metallopeptidase
[illegible]
>Syntrophomonas_wolfei_subsp.metylbutyratica_JCM14075T_02751_hypothetical_protein
[illegible]
>Syntrophomonas_wolfei_subsp.metylbutyratica_JCM14075T_02752_Methionyl-tRNA_formyltransferase
[illegible]
>Syntrophomonas_wolfei_subsp.metylbutyratica_JCM14075T_02753_Peptide_deformylase_1
[illegible]
>Syntrophomonas_wolfei_subsp.metylbutyratica_JCM14075T_02754_Primosomal_protein_N'
[illegible]
>Syntrophomonas_wolfei_subsp.metylbutyratica_JCM14075T_02755_S-adenosylmethionine_synthase
[illegible]
>Syntrophomonas_wolfei_subsp.metylbutyratica_JCM14075T_02756_DNA/pantothenate_metabolism_flavoprotein
[illegible]
>Syntrophomonas_wolfei_subsp.metylbutyratica_JCM14075T_02757_DNA-directed_RNA_polymerase_subunit_omega
[illegible]
>Syntrophomonas_wolfei_subsp.metylbutyratica_JCM14075T_02758_Guanylate_kinase
[illegible]
>Syntrophomonas_wolfei_subsp.metylbutyratica_JCM14075T_02759_hypothetical_protein
[illegible]
>Syntrophomonas_wolfei_subsp.metylbutyratica_JCM14075T_02760_hypothetical_protein
[illegible]
>Syntrophomonas_wolfei_subsp.metylbutyratica_JCM14075T_02761_Calcium-transporting_ATPase
[illegible]
>Syntrophomonas_wolfei_subsp.metylbutyratica_JCM14075T_02762_acyl-CoA_synthetase
[illegible]
>Syntrophomonas_wolfei_subsp.metylbutyratica_JCM14075T_02763_Poly-beta-hydroxybutyrate_polymerase
[illegible]
>Syntrophomonas_wolfei_subsp.metylbutyratica_JCM14075T_02764_(R)-specific_enoyl-CoA_hydratase
[illegible]
>Syntrophomonas_wolfei_subsp.metylbutyratica_JCM14075T_02765_polyhydroxyalkanoic_acid_synthase,_PhaR_subunit

MSQESDKAKNDIQIPDMAELWKEMYFTTEGDWAKTMKSFISTETFVAMLDKTLEQYLAMSKISRQQMDKFSEKGVVPSKKDIARVAELVISLEEKIDMMEFQFFDNFNKMTDSVIKMVDFQNHFKQELSLIKTELQEINRNIESLKPAHKNQPNEQIEPVAEAPKSKKRGRKKAVEKTTLPDNIKGS*
>Syntrophomonas_wolfei_subsp.metylbutyratica_JCM14075T_02766_poly(hydroxyalkanoate)_granule-associated_protein
MATKESLKKAFVISESSAEKLWDMWLVTLGSMSWTQEQIENMIRKYLDQRKVAREETTKLVEELMNQAKKNNQQLQKMMQEAVNATMENVDFPSFTYFEELNKKVEDLSKKVENL*
>Syntrophomonas_wolfei_subsp.metylbutyratica_JCM14075T_02767_uracil-DNA_glycosylase,_family_4
MPSSREEIIRRIIGLEDRISRCFRCKSLIKCTGRPSLGKGELEPELMLVFESENRLTRDSKKLIELRRFIQDEFKLKDIYHSFLVRCHPKACTLQKSIAAYTDYRLPSERYIDNYNICLLKQEPCSGILVRPGYEEILFCLPFLLEEIDILHPRYIILFGQRTAEFVLRSFGIFGDIANNQHFEKDGMAFIFVKYPQEDFALQDLKALQRIFHKASS*
>Syntrophomonas_wolfei_subsp.metylbutyratica_JCM14075T_02768_hypothetical_protein
MEENIFDSFGIPPTIFGTKEWQDIEKKENTLGADMLLAEIIEKKIWSNEEILWVMKRLIFFYGKKDKLLKKTPIERLFVNMVDILRAFYVILDISNPELDDNMRSYISTKLADATWGINLRTREYLEKLKDN*
>Syntrophomonas_wolfei_subsp.metylbutyratica_JCM14075T_02769_D-gamma-glutamyl-meso-diaminopimelic_acid_endopeptidase_CwlS_precursor
MRRIWAIIFMLPILLLFNGTSLDASEAVYIVKSGDSLWKISSQQGITVKEIKELNNLTSDSLKIGQKLLLKGSIGVETGDSLGQESYTVQAGDSLELIAQKFAMTVARLKELNNLNSDILNAGAQLRVENRNLANPSRAGFPLDGRLIIEKAAQYLGTAYRYGGEGPAGFDCSGFVRYIFSNFGYNLPHNAAAQFKSAYELDSSEMMIGDLVFFACGGKGIDHVGIYSGDNKFIHSSSPRSGGVIYSSLTEGY
YAGKYVGARRILR*
>Syntrophomonas_wolfei_subsp.metylbutyratica_JCM14075T_02770_Histidinol_phosphatase_of_the_PHP_family_protein
MLYDLHIHTSASDGLLSPVEVISQARDIGLPGLAITDHDTVDGLEEARCFMEENSPALDFIPGIEMNTELAEKEIHILGYFIDYHNRLLLKRLQEIKEARLDRARKMIMRLANMGFNIDMEQVERIARGDLIARPHIAQVLMENAYVSSVKEAFDKYIGKGCPAYVNRYKFLPTEAIELIKGAGGIAVLAHPGLIRDDSFVVTIISLGIEGIEVFYPEHNHGQVLKYSQLSRENRLLITGGSDFHGYNQDENR
GRIGSCGVNPEIMRNIFDYQQRKTKNQQEEH*
>Syntrophomonas_wolfei_subsp.metylbutyratica_JCM14075T_02771_Membrane_dipeptidase_(Peptidase_family_M19)
MQIVDLHCDTISEITASDEALLSNGRHFDLLRAKEAGVSLQVFALFSSSREADSSLREILMQIDKFYQELEQNSSHVFQILGSNELQRVGQTDKIACLLHLEGADAVGSDLAVFRQLCHMGLRSIGLTWNYSNQLAGGIAEGGNAGGLTCQGKIILKEMERNGILLDLSHISEEAFFESLECYNKPAMVTHANARALCNHPRNLSDKQLKLLAENGGVIGLNQVAHFIKEGKATLEDFLNHAVYISDLI
GVKHLALGSDFDGTDSLVLPGVEAYATLEQSLYNRGFLPQEVEMIFQQNALRVMKRVLNAL*
>Syntrophomonas_wolfei_subsp.metylbutyratica_JCM14075T_02772_Stage_V_sporulation_protein_S
LIQNIDKYKGAIYLFLYFLKGVILVEVLKVSAKSSPNSVAGALAGVLREKGAAEIQAIGAGAINQAVKAIAIARGFVAPSGMDLICIPAFTDIKIDGEERTAIKLIIEPR*
>Syntrophomonas_wolfei_subsp.metylbutyratica_JCM14075T_02773_hypothetical_protein
MSRERLTLRVRLVFHPEGVTNKVASHGREIGAVLLEPRNKPLVLPMGSALSGTTDAPCGRY*
>Syntrophomonas_wolfei_subsp.metylbutyratica_JCM14075T_02774_metallophosphoesterase,_MG_246/_family
LNILVIGDIVGRPGRKAIRELLPQIQKDYKIVFTIANAENAAGGRGLSREVMKELLSHNIDVLTMGNHVWDNKEIFNFIDDEYRLIRPANYPPYCPGQGYHVYSAAFNQKITVINASGRVFLPSLDCPFRVVDEIVSELQEESDYIIIDFHAEATSEKLAFAHFFDGRVAAVLGTHTHVQTADEKVLPGGTAYITDLGMTGPVDSILGMQKEQVIEKIIFQRPVRLEVAKGACQLQAVVLNFDEASRKCISI
TRISQQLPAF*
>Syntrophomonas_wolfei_subsp.metylbutyratica_JCM14075T_02775_hypothetical_protein
MDRDLGLDVFLTVSLDHPEINAVKYLADKEQIILEVALNTVIEPMKRKSFLKKTRDCLDLFHRLADQKPVLMKVDLIDKSELSFLRFYRDVGTLSQDEISLLILILREEFANLLVKDNNDIVAKDAFKRKLKRTLMQKLDRNSSKSTDFLAYRKEGRVFVFNR*
>Syntrophomonas_wolfei_subsp.metylbutyratica_JCM14075T_02776_Ribonuclease_Y
VIEIDLTSFVIITLSLAVGLIGGYYGRRFLAESRIRTAEEEVAKMLDEAAKEVEAKKKEILLEAKDEIHRNRQDAEKDIRERRRELDRVERRIIQKEEMIDKKTENMEKKEQVLLEKEKETEKTQQDLQMILSQQLKELERLSGLSSEEAKELLLYNVSQQIRQETALLIRGIENEAKEEADKKAKNIVSLAIQRCAADVVSESTVSVVPLPNDEMKGRIIGREGRNIRTFEALSGVDLIIDDTPEAVILSSFDPIRRE
VARVALGNLVSDGRIHPARIEEMVEKARKEIEQEIREVGEQAAFEVGVHGLHPELIKLLGRLKYRTSYGQNVLRHSVEVAHLAGIMAAELDVDIMLAKRSGLLHDIGKAVDHEVSGPHVEIGVDLAKKYRENKDVIHGIEAHHGDIEPETVEAVLVQAADAISASRPGARRETLETYIKRLEKLENVAESFSGVDRTFAIQAGREIRIIVKPEEIDDLHAINLARDIAARIEQDLDYPGQIKVVVIRETRSVEY
AK*
>Syntrophomonas_wolfei_subsp.metylbutyratica_JCM14075T_02777_Recombinase_A
VEINEVNKGKMEALNSTIKNIEKQFGKGSIMKLGEAVNMNVEVISTGCISLDLALGVGGIPRGRVVEIFGPESSGKTTVALHAIAQAQKDGGMAAFIDAEHALDPLYARKLGVDINNLLVSQPDSGEQALEIAEALVRSGAVDIIVIDSVAALVPRAELDGEMGDIHVGLQARLMSQALRKLTAHIGKSNACVLFINQIREKVGVVYGNPETTTGGRALKFYSSVRIEVRKGEAIKQGTDLIGNKTKVKV
VKNKVAPPFKTAEFDIMYGIGISREGDLLDIAVTTDIIQKSGSWYAYQGERLGQGRENAKEYLKNHPEFMTLLESRIKDWKQEGNAASNSLEEA*
>Syntrophomonas_wolfei_subsp.metylbutyratica_JCM14075T_02778_hypothetical_protein
LFWRVVRVCVYGKDTPYYYQGKAYKRSDTSIVEVDRSELRRLAIEGINMDYEERKASSQDLSFTILESGLKEKAGIEKINLDILKTLNLFNKDGYYNITGNYWRIGMISLFQELMC*
>Syntrophomonas_wolfei_subsp.metylbutyratica_JCM14075T_02779_Response_regulator_ArlR
MRQVLIVEDDRNLSRGLCLALRNQELQIVPCFDLKSAREQLACGMTELVLLDINLPDGSGLELLGEIKGGSKSIPVILLTANDTEMDVVMGLEHGADDYITKPFSLEILRARVKTQLRRLAVNEQKSVVIDSYRFDFAHMEFFKADTAVELSKTEQKLLRLLVENRGVTISRAVLMDRIWTDGADFVDENALSVTVKRLRDKLDAAQYIKTIYGLGYIWVVKQDE*
>Syntrophomonas_wolfei_subsp.metylbutyratica_JCM14075T_02780_Alkaline_phosphatase_synthesis_sensor_protein_PhoR
MNECNLGLFVIALAAILLCLAAVLLQQHKTRNILKTLNRMLDSALDGSFTEHSFNESLLSAVESRLNRYLTASAVSARNLTAEKEKIKELLADISHQTKTPIANILLYAQLLEEQEPPKESIECVTALRGQAEKLSFLIASLVKLSRLETGVFTLHPISNAIAPMLREMAEQFAAKTAQKEIQLTINTTDAYAVFDYKWTVEALGNLVDNSIKYTPADGRIQISAKEYDLFCRIDVADTGIGISEQEQAKVFSRFY
RSPSVSEQDGVGIGLYLTRQILAEQGGYIKVASSLGGGSVFSMFLPRKA*
>Syntrophomonas_wolfei_subsp.metylbutyratica_JCM14075T_02781_Macrolide_export_ATP-binding/permease_protein_MacB
MTILQTRNLKKIYGAGENAVHALAGVNLQVEKGEFVAVVGTSGSGKSTLLHMLGGLDRPTNGTVTVDGKDIFSLKDEELTIFRRRKIGFVFQNYNLVPVLNVYENIVLPIQLDGNSPDAAYVESIIETLGLKSKLQNLPNNLSGGQQQRVAIARALAAKPAIILADEPTGNLDSKTSQDVLGLLKITSQKFVQTIVMITHNEEIAQLADRIVRIEDGLIVAR*
>Syntrophomonas_wolfei_subsp.metylbutyratica_JCM14075T_02782_ABC_exporter_transmembrane_subunit,_DevC_protein
MNVANKKYIRQLSMKSMKAARTRNLIAILAIALTALLFTSLFTVLLSINHSVQQANFRQTGGWAHGTFKYLTHEQFDEIKTDPLINQYGLRRFVGTPDGVPFNKSHVEIGYSDAMQAHWMYCNPGEGRLPAEGTNEAATDTRVLELLGIEPVLGNKFTMTFDVDGIEITETFVLSGWWKYDEAIVANHVLIPHSRAQAIFDQVGIGSGIGKDGMTGSWNMDVMLGSSLHIEQDLNEILTNYGYQ
NESRSLGDNYIQIGVNWGYTGSQLADNIDPITVIAIIGLLLIIVFTGYLIIYNVFQISVSNDIRFYGLLKTIGTTGRQLRRIIRQQALVLSIIGIPLGLLTGYWIGIKLTPVILSRLNGVVEDAVSASPLIFIGSALFALFTVMISCRRPGRMAARVSPIEAVRYSEGTAYNKTIRKAQSGASLPGMAWANLGRNRSKTAITVASLSLAVLLLNMTVTFTNGFDMDKYLANVVSDFIVADSSYFQTGNFWGGDRALSE
EVLSSLESQSGVEAGGRVYGKSSPVEEFVTEDYYRALYGKWNDKEALDRAVTFTEKNEEGLLAERVQLYGMERYVLDKLRVLQGDLSKLHEPGGRYVAAVYSDDDYGNAKMDSHWAKLGDTVTLRYVEKYEYYDPKSGKILDPDNIPDDQPYRHRAKNYQDIDYQVAALIIIPHSLSYRYYGTDEFIMNDQRFIQDSGTSNIMYYACDVSDERTGDMEAFLYDLTNKQMPQLDYESKSTYAAEF
DSFRNMFLMLGGVLSFIIGLVGVLNFLNAILTGIITRRRELAMLQSIGMTGKQLKAMLTWEGLYYALGAVAVSLLLSIAAGPLLSNVLASMFWFFTYRFTVLPILSVAPIFTVLGIVVPLTVYRVVSKRSVVERLRETE*
>Syntrophomonas_wolfei_subsp.metylbutyratica_JCM14075T_02783_YvrJ_protein_family_protein
MSEFLEVVGNVGFPIAVSVYLLIRVENKLGELTAAIGELREAIILLPHEKYWPATPTMPSFPSSGQPAKQEYL*
>Syntrophomonas_wolfei_subsp.metylbutyratica_JCM14075T_02784_hypothetical_protein
MAAQRILEMAFNTQLGKTQRIRVYDAKDPITGAEVAAAMEDIIAKNIFTSTSGNLTGKIDARLLTTDSSDLSLA*
>Syntrophomonas_wolfei_subsp.metylbutyratica_JCM14075T_02785_hypothetical_protein
MAVTTTSLSSDLVLVMDAGTGASGQPLSKNRIFKNVKTSATNEDVYAVAQTLVGLQSKTNNAIQRRDIVELEEV*
>Syntrophomonas_wolfei_subsp.metylbutyratica_JCM14075T_02786_Cellulosome-anchoring_protein_precursor
MNVRKVSLIPELMVFMVISFFSVFLLTAPVSAADIMGNGSEDKVYSDLSNSNPNFNYIKYLSQRGIIAGFPDGSFQPEESLNRAQAAVILVKAMGLEISPASEMSFKDVGKDHWAATYIDSCVKAGYIKGYPDGSFQPEEKLSRAQGISLVLRLSKQDMNRASLPQIEDVNHEHWAARHIATGLAAEMVGLDSSGKKFLPDSPFSRQEMCRALGMLLTQDPDHYACELKGKLVVESGEIILKRAGQSRKI
EKNEIIVKGDIIESGSGGSAVINYPDGSSILMKENSVISIIDSRGRSYIMKDGSPSVAVDWLNYKIDKGLVFTALATIAAEPGQEKAEENKTTGLTTLLAAGDSSRYIAAAEQSIPPWYKTAQKKVKVKVDMPYGVAAVRGTIIMISIGSDGRCNVSCLSGNAELSGGGGTVNLTGGQSSGFNQSGAPPQPPGPMTLQQQQLFQNVQQWIQQSNSGGSGSSGGVPGPGPNVPGPGPGGSGGGGGG
GGSGGGSGGNPPSIQSIANISANVIKGQSYQPPATVTAVMTNGTSQQVAISWDNNSPDTSNTGVIILNGTVAGYAATVTLTLTVESSSPIARIDNLNATVEEEQPFELPASVTAILEDGNSRQVEINWDIDNIDTGQPGIVIINGTVAGYSGTVTLTVKLTINVKPRVSIKKIDDIKVEIKVGDSFTLPAQVEALMSDDSTQEVAISWTPSSVDSSKAGNYEFEGTVEGYSKTVKLKLKIEPGVSIKKIDDIKVDIKV
GDSFTLPTQVEALMSDDSTQEVDISWTPSGVDSSKAGNYEFEGTVEGYSKTVKLKLKIEPRVSIKKIDDIKVQIKVGDSFTLPTQVEALMSDDSTKEVDISWTPSTVNTDVAGIFSFSGQVEGYTSEVSLLLIVKAIPPPPPPPLSIKKINNIEVEINVGESFTLPTQVAALMSDDSTKQVAISWTPSTVNTDVAGNFSFSGQVQGYASAVCLLLTVKAISLPQLDPPIANPPAGTYSTAQSVSLSSETSGAIIF
CTIDGSDPRTSGTRMIFNGAITISTNTTIMAYAEKEGMADSDVVSFSYTIEAPSVGAYVYSVSPTILYEGPNSPGILDPDTAFINIEIANGTFIDDISVSDIVYENMPFGLFVSGIEHSDNCLYIYIDGQATNHGANDSIDNLYFTIGKDAVTGATADLTTGPISITFYD*
>Syntrophomonas_wolfei_subsp.metylbutyratica_JCM14075T_02787_Cell_wall-binding_protein_YocH_precursor
MHLVKRVLIMSVILSGVLLLYSNDAMAAEPNLQWGSRGNAVVELQKTLNNQGFWCGAADGIFGAKTYNAVINFQKSKKLQVDGIVGPQSRQALGLSTATPSYTQVSRGGERTLSMVTTGYCPCAKCNYPYGGQPSYLGYPLARGIIAVDPHVIPMGTRLYVEGYGSGIAADQGNAIKGNRLDLFFDTHQEALKWGIRTVKVTIL*
>Syntrophomonas_wolfei_subsp.metylbutyratica_JCM14075T_02788_50S_ribosomal_protein_L28
MAKCDICGKGVGFGKKYSHSHIRTNRQWKPNIQPVKAIVDGTPKKIYVCTRCLRSGKVERAI*
>Syntrophomonas_wolfei_subsp.metylbutyratica_JCM14075T_02789_hypothetical_protein
MIKIKTSELGDIDMEKEVIETIAGLATVDCYGLVGMVPIDWQSGITSILGLESIRKGVLVRSSEDGLIIDVYVIVGYGIKISEVASNVIQKVAYVVQNNTGLKVAAVNVNVKGIKVVTGK*
>Syntrophomonas_wolfei_subsp.metylbutyratica_JCM14075T_02790_dihydroxyacetone_kinase
LSLLKIDGNDLIRAVKSGCIKLEHERDRVDALNVFPVPDGDTGTNMYLTLLSAVKEGEKARGQSLATVAKAISTGSLMGARGNSGVILSQVFRGFARLMEGKEQAAATDLAQALKAGANTAYEAVMKPVEGTILTVIRELARAAEAEAKKDSDIVRTLLLAIKAGHQTLQKTPMMLPILKEAGVVDAGGQGLMIFFEGVLEGLVQNQDIILESYDKQVELGWQDKRVEEVLSLDFQYCTETLIKGK
ELEPAEIKEHLAPLGDSMLVVGGDELVKVHIHSNHPGKVLESCLQWGSLSDIKINNMLEEVHEHRDNWTRFNEELAAASKNLKNIGLVAVGAGSGVVQILQNLGVDVVVEGGQTMNPSIEDLLNACKEVPAPNVIVLPNNSNIIMAAEQAKQLSDKPVAVVPSRSVMQAITALIAFNPELALEDVAQAMIEEMKQVKYGEVTYAVRDSQLNGLEIKSGDTIGLIEDEISVTADNAGEAVLKLLERMV
DEDSELITFFYGEDVSEEEANGLKEQIIKNYPDCEVDLHYGGQPHYRYLLSVE*
>Syntrophomonas_wolfei_subsp.metylbutyratica_JCM14075T_02791_hypothetical_protein
VESKKDTKEAMERIKFEVSQEMGLSKPVEKKQKNIKK*
>Syntrophomonas_wolfei_subsp.metylbutyratica_JCM14075T_02792_hypothetical_protein
MARNNQLLVPQARAAMDTFKMEAAGEVGVSLKQGYNGDLTSKQAGSIGGQMVKKMIYDYQQRASGKV*
>Syntrophomonas_wolfei_subsp.metylbutyratica_JCM14075T_02793_Germination_protease_precursor
MINREFNIYIDMAIEARDLVRGAGDQEIPGVKEDIKQLKNIKITTITVLDQNGAEKLGRPIGSYVTIESPPLKINDPYVRDEIVEAMEESMHTLIGEKLKPQDTVLLVGLGNWRATADALGPRFIEYSPITRHYHNYAPDALVEGMRPTCGISPGVLGITGLETFDVIKGIVEKVQPALLVAVDALAAQDVKRIGTSIQMCNTGIQPGSGVGNARHAITEQELGVPVIAIGCPTIVNAAIIANEAVKNFCYNI
GAPFNETTSLDAIKEILSPFGGSLGVTPKEIDEIIENTTRIISMGVARSLFPGISEEQLLLYAT*
>Syntrophomonas_wolfei_subsp.metylbutyratica_JCM14075T_02794_Ribosomal_RNA_small_subunit_methyltransferase_D
MRVIGGMARGRPLKAPPGMNTRPVTDRIKESLFNIIALGIEGATLLDLFAGSGSVGIEALSRKAKWVCFVDNNREAVKTIKANLDRCGFSGDYQVLRNDVFKAVEALRRRGLRFDYIYVDPPFTQEKIFDLIMDALDKAELLNEKGIVIIRAPRKKSLPESLDGLERYRLNHYGESSLHYYRLDKEEQGHDGDIQDTGRNGTNGKEQ*
>Syntrophomonas_wolfei_subsp.metylbutyratica_JCM14075T_02795_hypothetical_protein
MMEIFRILDEMEQMVKNSKKVPLMNDKVLIESPRFLDRLDRIRAILPEDLETARLLLSEKERIVKDACAEAETVMEQSRDRVARMVDDNEITRNAMKVAEDIIAKAEQVAQDIRRDADDYADGLLSHMEIVLQKGLDAVQQGKEALRQE*
>Syntrophomonas_wolfei_subsp.metylbutyratica_JCM14075T_02796_Sporulation_integral_membrane_protein_YlbJ
MGSYSRLFFLIIILFLAVFMLLNPQETVNAASSGFKLWFSIIVPALLPFFILAELLVNLGVPRILGILLEPVMRPLFNLPGCSSLVVVMGFTSGFPVGAILSKKLYDEKMISGEEMSRLVSFTNNCSPLFIIGAVGVGMFGSPFLGYILALSHYLSNLIVGMFWGQRTKKPLRNISRLPLSQELSQALAEARENYCGPGKLLSDAIKNSLNNILAIAGFIILFSVITQMFSFWGIIDLLALFLLKILSIFNLSYELIYGWLM
GLFEITIGARAITATSPANILPQLLAVSSTLAWSGLSIIAQVMSIVVGTPVKLSFYLYSRLLQMSLSILITAIAYKLLATGQQSVLSFSLPYNKALYSFDAWGISIACLWVCFLLLAFMLASSLYLRRP*
>Syntrophomonas_wolfei_subsp.metylbutyratica_JCM14075T_02797_NTE_family_protein_RssA
MLDDKFRPRIALVLGAGSARGLAHIGVIQVLREHHIPFDFIVGSSMGAMVGGIYAAGADIKMLDRMLENMDTSVFYDVHVPRLGFMSGKKIKIFLELMTKKKSFAELDLPLLTVATDLLSGGRVVLEEGPVAEAIRASISIPGIFTPVQQEGMILVDGAVVDRLPIEVARSRGADLIIAVDVTFGPGKNVSIANTMDVIMASLDIMGKQHFDLVHPGADILIQPAVGSFQPGDFGKSRQIVDQGRIATEE
KIDEILEKIMTEAAKKIEGI*
>Syntrophomonas_wolfei_subsp.metylbutyratica_JCM14075T_02798_hypothetical_protein
LPVEYVLGLLLIFYIGVLIKGDDSTKKRMGGVLGVIGFAGIVYSSTQFGNYTLDESKLWFLLLVSIALAILGVVAFFPKNKDKS*
>Syntrophomonas_wolfei_subsp.metylbutyratica_JCM14075T_02799_hypothetical_protein
VKKVEVFCVIEKADDEYLAYCDELRATASGDTEEEAIVNLKAAIKELIKYQEPQIFAPNKKRVLVEVV*
>Syntrophomonas_wolfei_subsp.metylbutyratica_JCM14075T_02800_cytidyltransferase-like_domain_protein
MAILGIIAEYNPFHNGHLYLLEKSRQQGDFSATVAVMSGNFLQRGEPAFCDKWARAEMALNAGVDLLIELPFCFATRSAYYFAKGAVQLLQRSGVVTHLAFGSESGQLSRLQEIAGILAHEPESYKTALKKGLSQGLSFPLARSSALQEYMGGEKKQLQEILPGPNNILALEYLRVIEEEEIPLLPLTIPRQGSSFHSSDLSPYSSARAIRQALYQNSNWEKITSSIPPATKKILQREIALGRAPIGPDNLEQAI
MVNLRLVSTDYLKEIYEVSEGLEFRIKEAANSCGTLEELRQFIKSKRYSLTRINRTLLYTLFALSKNQVELYDQHGPQYLHILGFSAKGQEILQKIKIKSELKIFSRGSEMKKARDENQGTALAEMIKLDCRATDIYSLLFPDPATRRAGRDFTTSPVPGT*
>Syntrophomonas_wolfei_subsp.metylbutyratica_JCM14075T_02801_Phosphate_acetyltransferase
MDLIAQIKEKAAKNVKTIVLPEGTEERTIQAIKGIVDSKVARPILLGDEGAIKGLASKLGADISGAEIINPEKAPFFDDFVQAFYEMRKSKGVDLDKAKATMLDPLFFASMMVKMDKADGEVAGAENTTGNVLRPALQIIKTSPGISAVSGAFIMIVPNCPYGDNGVFVFADCAVTIDPTAAQLAEFAKASVGTAVDLCGMKDPKIGMLSFSTKGSASHEMVDKVVQATAIAKEKFPELKVDGEFQLD
AAIVPSVGSSKAPGSPVAGDCNVLIFPDIQAGNIGYKLVQRTAKAEAIGPILQGIAKPVNDLSRGCSVEDIVNVVAMTAVRS*
>Syntrophomonas_wolfei_subsp.metylbutyratica_JCM14075T_02802_Acetate_kinase
MKVLVVNAGSSSIKYQVVDMTDESVLAVGLVDRVGIPGTTLDHSPLGKDKVTIKKDLPDHTAGMELVLQVLVNEEYGCIKSMDEIGAVGHRVVHGGEAFAASVVIDDEVKRVIKECFEIAPLHNPPNMMGIEACQRLMPNVKHVAVFDTAFHQTMGPANFMYALPYDVYEKFRVRRYGFHGTSHFYVAHRAAEMLGKPYEECKIITLHLGNGASMAAIKGGKVIDTSMGFTPLEGLVMGTRS
GDIDPAIVFFLMDKLGMNSSEANNYFNKKSGMLGLSGVSNDLRDILEAAGSGNERAKIALDVYYNKVKGYIGNYIAKLNGVDCLVFTAGVGENAIDIRENVCSNLDYLGIKMDVEKNKVRGKEVDVATADSKVRIFLIPTNEELVIARDTFNLAK*
>Syntrophomonas_wolfei_subsp.metylbutyratica_JCM14075T_02803_hypothetical_protein
MKIDLRRLKLRPQQSEEFYLEAVGRNDFLKEVGGKFAAPLQVKLLVDNTGKIFVARGHVRTALQLPCSRCLGEAIVPVDTELACNLLEARYSEHADWEEDIIIQDPEQVDISPCVEEALFMSIPINPLCKLECRGICPQCGVNRNLEECQCESEEIDPRWEKLKNIIK*
>Syntrophomonas_wolfei_subsp.metylbutyratica_JCM14075T_02804_50S_ribosomal_protein_L32
MGVPQRRQSHARKNKRRSEWRKIDKPGLVECPQCHELKMPHRACLNCGYYKSNKVM*
>Syntrophomonas_wolfei_subsp.metylbutyratica_JCM14075T_02805_Phosphate_acyltransferase
MRIAVDAMGGDHAPVEIVAGAVKWVAEEEGRQAFLVGQEELLKQELKPYSYDPSRLSVINASEIIAMEESPATAIRRKKDASIVVANRMLKEKKADAILSCGSTGAQMAAAIFILGRMEGIERPPIVASIPNMAGAYTLLIDVGANVDCKPRQLLQFALLGKTYASIIYGVEQPRVALLNNGEEESKGNSAIVETYALLRQQPGINFTGNIEGRDIFTGKSDVIVCDGFTGNVLLKTMEGMALFIAKGILDA
GGPLPAFFQRLDYTQTGGAPLLGVNGLSIVCHGSSKREAVYNGLKIAEDCYNKNIIEMQQLELSKAVETEA*
>Syntrophomonas_wolfei_subsp.metylbutyratica_JCM14075T_02806_Acyl_carrier_protein
VQEFFERARKILAEQLDIEVEKIRMETTFDEIEADSIDIVEMIMALEDIYDVEFPEEEQKDYQTIGSLINDLYEFLKQSKNAE*
>Syntrophomonas_wolfei_subsp.metylbutyratica_JCM14075T_02807_Ribonuclease_3
MQNERRIARRFLEDKQLPYSSNELITMALTHPSYAQEKNTAANNQRLEFLGDAVLNLVVAEYLYNHYGRKAEGELTKIRAKVVCEDALAIFARNINLGQYLLLGRGEEMSGGRKRKSILADAVEAVIGAIYLDQGLETVQLFIITQLEELISETASGDYYDYKSKLQELVQARDKENVSYAIIEESGPAHAKVFMAGVYYRERLLATGEGKSKKEAEQKAAQKVLQNNLVPGT*
>Syntrophomonas_wolfei_subsp.metylbutyratica_JCM14075T_02808_Oxygen-independent_coproporphyrinogen-III_oxidase_1
MKHINIPIFIPHLGCPFTCVFCDQNRISATRDIPGEMEVINIVENHLATIPAGSEVEIAFFGGNFTMVEKTRQEMYLATVKRYLQDGRVRGIRISTRPDGIDAQILDFLQRWGVRTIELGVQSLSDEVLKASWRGYQAADVFKACQLIKEYGFRLGIQLMIGLPGDKLDLDMKTAAKVIELRPDMVRIYPTLVIAGTHLEKLYLKGKYIPLTLAEALQNSLAVYLQFQKENIPVIRMGLQPGEELRREGCV
AAGPFHPSFGELVEQEAFKAQAGKAIEQFFTAHGRHPQLLLFVHPRDISKVTGQRRSNLNYLKEQFGLELIKVRGIENLARDSVGAGTIDASAPALLLSRLEFIEQVTWDTGTGSLSHVNVKKYQR*
>Syntrophomonas_wolfei_subsp.metylbutyratica_JCM14075T_02809_Bifunctional_protein_BirA
VRQRILGELCQNPGEYVSGKELAEKLGISRVAVWKHIENLKGEGYAILGVSGKGYCIEESYNVLLLDSIKAALSTARLDWKLHLFPELDSTNEMAKKLIREGQAAEGTVIISRQQSRGKGRRGRSWQAPLGGLWFSIILRPELALSEVVVLSLVFALAISRALESFRLFDPQIKWPNDVYINGKKVVGILLELSGELEQADYLVAGIGINVNIDRASLGRGVAKTATSLWEESGTYYQLGEVLLRVLQSIEEYY
YEFLENGFEAIRKEYKSRCFHLGKMIKLDTPRGSLEGLNLDIDEQGNLIIDSGDSIECLTTGDVALLAEE*
>Syntrophomonas_wolfei_subsp.metylbutyratica_JCM14075T_02810_Chromosome_partition_protein_Smc
LYLKRLDIKGFKSFADNTELQLNPGLNIVVGPNGCGKSNIVDAIRWVLGETSIRQLRGQKNEDVIFNGSDKKKALGMALVELLIDNSDHCLPLDFSEITLGRKVHRSGESEFYLNKSRVRLKDISELLSGSGVGKKGYAIISQGELEEVLNGQALDRRLMLEEAAGVIKYRQQRDEVKKRILNSSNDLLRLGDILEELGLRRQELFKKAEKARLYMALNSECQELEKSVLGFELARTEKDWQQKTGDLIK
KQDEIKAQAAQVALLEAKLREEEGLARQQLSLGELGEQRHLLESRLNLLQGEIRLGEERIKNNNKRIDDAVTDEKKQLILLDNIQRDLHLCSRDYDKEQQNMAQRVAEYNELEHEVQELESIIAGFKGDFDKKALVFDKMKQESQHKNELAEKAELLGQAREKKERLKIHIEELAAKLLKGRKGHLQCEEEIRALQAEIGQKQSFIEELSREKDELAEVLQNLNNSCRELNQRSIEINNQLLAIKDL
QRNLTGYSPAVKSIIKQARSGRLKGIMGVLGEILEVPAGMELAIDVAAGNGLQNIVVASEKEVREAIDYLKVQELGRATFLPLDVLKVKPLPGSVVEKILVQDGVLGLASRLVHYDNKFAKAVEYLLGRVLLVEDLTRGIRIFRESSLPCRIVSLDGELINVSGAITGGKKASPTQTPLQRRAEEKKLLRWQQENEQAREDKRREQKQVESRWQELEQVISRARNDLLESQFRCDMMMKQKEDLALELL
EDERERDGYLEQLTRLDGEVAENERETSLLHRKLGDIEQENQKLMIDLENFKDKMEVKRRDYEVFQERLLSYRKQLDMKGRELNIKKNNLSQFKELRASYRQSLDDSRQLMQSLQLEIGREESRMEQFKKELENKTEDLARLSAESREMQEKEKGNWALIEKYRQEIIPARESLARLETGARNLEVATARLEVELEGLKNRWREKFAAEAGLLAEEPLTPGEIRFARKRIAILQQQLEAIASVDIDSIREY
EETSGRYDFIKQQYDDLSSARDSLDELLQKTEKLMLQDFSRFLLLANESFKNTFSEIFGGGEASLRIEAEVERLEAGVDIEVKMPGKRSQSLNLLSGGERALTCIAFIFSLLRLRPVPFCVLDEIDASLDEVNLQRFSGFITAMSRNLQFIVITHRQATIESGENIYGITMPEEGVSAVLTLNVFEAESLAG*
>Syntrophomonas_wolfei_subsp.metylbutyratica_JCM14075T_02811_Signal_recognition_particle_receptor_FtsY
LAFFDRFKFKKKDKENEEIPWEKEAPGEESPEEEPRETLGAEENGSEEAASPSLAGENGHSSIFDRFKTKKENGRAEEKEQAQPADDVEAVEREEDAYEKEVNGKKTKWGFLDKLKKGLSKTRENTIGKIEALVKSSKKLDDDFWEELEEILIQADVGVNTTIELVDNIKKAAKKQKMHDSSEVLELIKDEIRKLLEYNNVPLNIPQGELAVILVVGVNGAGKTTSIAKLAYRFHEEGKKVLLGAADTF
RAAAIDQLNIWADRVGVEIIKHQEGSDPGAVVFDAIHASRARHADILIIDTAGRLQNKANLMKEISKIKNIIQREIPEAPHEVLLVLDATTGQNAISQAKIFEEATGVTGLILSKLDGTAKGGIILAVEKELKIPVKLVGIGEGLEDLRDFSPQIFTEALFACNGGD*
>Syntrophomonas_wolfei_subsp.metylbutyratica_JCM14075T_02812_S-methyl-5'-thioinosine_phosphorylase
LLKTAIIGGTGVYDPCLLEDVREMVETTPYGSVRVSLGKYAGMEVAFIPRHGLEHSIPPHLINYRANIKALQQLGIKNILATAAVGSLHFEFKPGQFVLADQFLDFTKSRRNTFYEGGEAGVVHCDMTVPYCPELREALVKAGKELGIQVQNGGTYVCTEGPRFETAAEIMMFKQLGGQLVGMTSVPEVCLARELGICYATLAIVTNFAAGITTGILTHSEVLELMKRNIELARSLMMSSLKYIGEEKS
CNCGKILDEPGAIK*
>Syntrophomonas_wolfei_subsp.metylbutyratica_JCM14075T_02813_Methylthioribose-1-phosphate_isomerase
MIRNLYCEDQALMVLNQNCLPEELVYEKCESPEEVVAAIKGLKVRGAPLIGVTAAFGLAMAFSSYSGTGKLRDAYFSRMKELFAAARPTAVNLFWALERMERVYRENRHLSPGELASCLRQEAQNLYEEDIRVNEAIGEQGQELLGEGSRVMTICNAGALATCGYGTALGVIRSAARRNKIEMVWACETRPVLQGSRLTVWELWQDKIPVTLLTDNMAGYTMSLGKVDAVITGADRIAANGDTA
NKIGTYSLAVLAKYHGIPFYVAAPLSTFDWNIEQGGEIPIEERSAEEVRTIKGSCLTVPAVNVFNPAFDITPEHLITAIICEKGIIRPPFGAAIKIWGREDE*
>Syntrophomonas_wolfei_subsp.metylbutyratica_JCM14075T_02814_L-fuculose_phosphate_aldolase
LQYQQERKELLQIAQEIFSSGLVSGTWGNVSLRVSGPGLMLITPSGMDYNQIEAEDMVLLDGEEKVLAGKYKPSVETPLHLGIYKKRPEINAIVHVHSLYATAFAVARQNIPVILEEAAQVIGHEVRVAPYALCGSQQLAENVLKVLGKDKRAVLLANHGLLGLGGNLPEALRVCLVAERTARIAIYSRVLGPLHSLRAEDILSLRQAFRNYGQKKD*
>Syntrophomonas_wolfei_subsp.metylbutyratica_JCM14075T_02815_5-methylthioadenosine/S-adenosylhomocysteine_deaminase
VRILLDNISIIPMSESNSFIEKGYLLIEDAFIKELGTGKAPEGVFDRIIDGENQVLLPGFINAHTHAAMTLLRGYADDLPLMEWLENKIWPLEAKLTPEDIYWGTMLAIVEMIKSGTTTFNDMYFCMDEVARAVALSGMRAVLARGMVGVGPESEQAIENSRELIGKWQGQADGRISFRLGPHAPYTCPPAYLERVMQLSDELQAGIHIHVAETRVEYEDILKQYGKTPVSHLESLGLFQGRQVLAAHC
VHLNEEEIGILHQYQVGVAHNPESNMKLASGIAPVPRMLESGIAVALGTDGASSNNNMDMLQEMRSSAFLHKVNTMNPTVLPAYQALEMATANGAISLGMGNELGRLEPGYRADMIIMNLKEAHMIPRYDLLANIVYSAQASDVNSVIIDGKIVMENREIKTFDEQEVLAKARETARRLVGK*
>Syntrophomonas_wolfei_subsp.metylbutyratica_JCM14075T_02816_Molybdopterin-guanine_dinucleotide_biosynthesis_protein_B
MPLDYSVKGMLIKVKIISFIGRHNSGKTTLLRRVIEKLRQTGYKVAVIKHTSHVLDTDGEHDSDLLFSAGAEMVCAVSPALSIHYLRHEREPDLQEILAQIPTVVDLIITEGYKKETYPKIEVLRQATGIEFIDAENIIARVSDFSIDDDLPCFNFEEIEELCRFIISL*
>Syntrophomonas_wolfei_subsp.metylbutyratica_JCM14075T_02817_hypothetical_protein
MGEGRIDFIAISAFLEQLYLPKTKIRLAKIIQAIKYGYNLKMLEGAGLKALNIY*
>Syntrophomonas_wolfei_subsp.metylbutyratica_JCM14075T_02818_NADH-quinone_oxidoreductase_subunit_E
MANYQDIIASCKDKKGGIIEAYHALQREFNYIPREAVAEAARVFGVSEAQAYGVATFYSYLSVEKRGKYIVRMCESAPCHVAGADKVLKAMEDYLGIKVGETTADGKFTLELTECVGQCQATPVITVNSQPVFNVSSEKIPEILSAYK*
>Syntrophomonas_wolfei_subsp.metylbutyratica_JCM14075T_02819_NADP-reducing_hydrogenase_subunit_HndC
MAEEIRILLAHADQIDPAQAEDYIKVGGYKGLEKARGMTSKDLIEEVKKSGLRGRGGAGFNAGMKWSFVPPAEVKYVVCNLDEGEPGTYKDRIICEKNAQALIEGMAICGVAINAQQGYIYCRGEYPFVVDLLRKAIQSAKDVGALGDFDIEVRMGAGAYVCGEETALIESIEGHRGEPRFKPPFPGVAGLWGVPTVVNNVETFACLPYILTNGADCFASIGAPKYPGTKVLTLTGDVNNPTYFEVP
TNYILSDVVYKLGGGVKNGRKFKAVQVGGTSGAFIPEQNLNTPIDFDSMSAIGAALGSGAVLVMDETRDIVDIVTRISKFFEHESCGKCNPCREGTFRCREIMEKINSGRATEADIDNLLLLARVMKRAALCGLGQAAPVPITSTIEHFGHEYRRKLLQYA*
>Syntrophomonas_wolfei_subsp.metylbutyratica_JCM14075T_02820_Putative_formate_dehydrogenase
MIKLTIDGKEIEVARGTMLLDACRQAGADIPTLCYDPDLRLAGSCRMCVVDVKGRPALVASCVAPAENGMVVQTESDDVVESRKIILELLAARHKFECPTCEKDGECKLQDYMYRYGVKESRYDGEGPHFGMEDPNPFIERDYDKCIMCTRCVRACDGITGAQAINVKDRGHHAKIAAPFDGKLADSTCVFCGQCIMVCPVGALSSKVAAGQGKPAQNKKVLTTCTYCGTGCTLELNVNND
RVVGVTSNRDENWSPVNKGALCVKGRFGWDFIHSPDRLTTPMIRENGGLRPASWDEALDFAAGKIKEIKEKYGPDSLAMFSSARVTNEENYLAQKLMRAVIGTNNVDHCARL*
>Syntrophomonas_wolfei_subsp.metylbutyratica_JCM14075T_02821_Formate_dehydrogenase_H
MTNAIGELEKDATAIFLIGTNTTENHPVIGYKIRKNVRENGAKLIVADPREIELAKMADVYMQFRPGTDVALLNGMMNVIINEGLLDKEFVETRTEAYEAFAQVVEKYTPEYVSPITGVAAEDIRKAARIYAEAERASICYAMGLTQHSTGTDNVKSCCNLALLTGNLGKPGTGVNPLRGQNNVQGACDMGALPVVFTAYQGVGIEANREKFEKAWGVKLSGQNGLTLTAAINKAHHGELKALL
VLGENPMVSDPDLDHVEDALDNLEFLMVQDIFLTETAQRADVVFPGVTFAEKEGTFTNTERRVQRVRKAISNRGDARQDYEILCELSTRLGYPMSYSSPEEIFEEIRTLTPSYAGITYQRLEGKGLHWPCPTEDHPGTPILHKEKFNRGLGLFHAIEFREAAELPDAEYPLILSTGRSLFHYHTGTMTRRAVALDLHQPENEIQVNPATAAKLGIIDGEMARVVTRRGNIELKARLTDIVKENVIFTFF
HFSEAAANVLTNASVLDPVAGIPEYKVSAARLEKIS*
>Syntrophomonas_wolfei_subsp.metylbutyratica_JCM14075T_02822_hypothetical_protein
LKNQRSFSISFGEKVTIKVYSLTETGYRGYMAKVRLHSIDALEMVLAGNKGETTREAAKRTNAVLAINAGGALIHITGQC*
>Syntrophomonas_wolfei_subsp.metylbutyratica_JCM14075T_02823_hypothetical_protein
LTLLQKPLLLHEGCINIQSERWRSMDATPGEGEQAKGGERQDVARALLTRMGD*
>Syntrophomonas_wolfei_subsp.metylbutyratica_JCM14075T_02824_putative_nucleic_acid-binding_protein,_contains_PIN_domain
MNRVLVDTSAILALINRNDSRHHAASEIARFMLQERMQPFLTNY*
>Syntrophomonas_wolfei_subsp.metylbutyratica_JCM14075T_02825_transcriptional_regulator,_AbrB_family
MLQAKSTLSSKGQLTIPLEIRKKLGLRQGDQIEISVIDDSIRLRKTNEFNIPPLAEDDPIFTFIGMGEGPEDLSEMHDKYIYGKNDE*
>Syntrophomonas_wolfei_subsp.metylbutyratica_JCM14075T_02826_hypothetical_protein
MSSLQWILIAVVFGAIEIFTVGLWFLWLALSALLVAAAAAFKLLPGLEGQLLLFAFFTLLFILFTRPLVLKFFKTNDTASNVKALVGQHGICLSSITPLEYGQVKVNGEVWTAVSQQEIEEDTRIVVAGIDGVKLLVEKAEPEASK*
>Syntrophomonas_wolfei_subsp.metylbutyratica_JCM14075T_02828_hypothetical_protein
VTEQKELAVKLKELFPQGRITCTEAHDLAREWDMDPRELGEACDEAGIKISGCELGCF*
>Syntrophomonas_wolfei_subsp.metylbutyratica_JCM14075T_02829_Cyclic_pyranopterin_monophosphate_synthase
MLDKHERNINYMRISLTDRCNLRCRYCMPETGVDNLSHYSILSLEEMAKLVRIASELGIQKIRLTGGEPLVRRNVPQLISYIAQIPGIDDIALTTNGTLFAALAEELKTAGLNRVNFSLDSLVPEKFKYITRRGDLSKVKEAIFEALELDMHPVKINMVVIRGFNDDEIIDFVELARKYPLHVRFIEFMPVGDLLFWKQDRMMPSQDIKAYIEESYALTPQKNILGNGPARYYSLEGGEGSLGFISPMSNHFC
GECNRIRLTAEGGLRGCLYDKREVNLKTALKNGSSDEEIKELFIRAIKAKPSRHHMNNGWGEENKRKMYQIGG*
>Syntrophomonas_wolfei_subsp.metylbutyratica_JCM14075T_02830_Molybdenum_cofactor_biosynthesis_protein_C
MNFTHINEEGYARMVDVSSKNDSERVAVAQAIVKMQPETLEKIRDGAIKKGDVLGVAQIAGIMGAKQTSSLIPMCHPLNLTSVNLEFTFDEINASIIVEAEVKTTGKTGVEMEAITAVSVAALTIYDMSKAVDRWMEITEIKLLEKSGGKSGHLVRD*
>Syntrophomonas_wolfei_subsp.metylbutyratica_JCM14075T_02831_molybdenum_cofactor_biosynthesis_protein_MoaC/MOSC-domain-containing_protein
MKKGKLYSICISPERGQLKTEVEQARLIENYGIENDGHAGEWHRQVTCLNWASVLKANKEHNLQVGPGAFAENLLIEGLELVEVGVGGKLKIGDNAILQVSQIGKEDHPSVVTRTLGVTLLPYEGLFCKVIQGGTIKKGDPVEVL*
>Syntrophomonas_wolfei_subsp.metylbutyratica_JCM14075T_02832_Molybdopterin_adenylyltransferase
VYRVGILIISDKGSRGEREDRSGQQIQDLLGQGYLLEYYEVIPDEKEEIVKRLIHACDVLSLNLLLTSGGTGFSSRDVTPDATLEVIEKAVPGIPEAMRLYGMQKTPKAMLSRAVAGIRGRSLIINLPGSVKGVKESLEAVLPALDHALDILLGSAAECGQG*
>Syntrophomonas_wolfei_subsp.metylbutyratica_JCM14075T_02833_co-chaperonin_GroES
LKIQPLGDRLVVKVAEVAAEKTKSGLYVPDTAKEKPQEGEVLAVGPGAFNDKGERMPMDVAVGDKVIFSKYGGTEIKIDGEEYLVMSQRDILAKIN*
>Syntrophomonas_wolfei_subsp.metylbutyratica_JCM14075T_02834_Stress_protein_H5
MLSKEIKFGEEARRALERGVDTLANAVKVTLGPKGRNVVLDRKFGSPTITNDGVTIARDIDLKDPWENLGCQLVKEVAIKTNDVAGDGTTTATLLAQAMIKEGLKNVAAGANPMIMRRGIDQAVKAIVDEIKALSKPVESRDSIAQVAAISADDPEIGQLIADAMEKVGRDGVITVEESQSVGTSLEVVEGMNFDRGYISPYMITNTEKMEAVLEDPYILISDKKISSIGEVLPVLEKVVQTGKPMLLI
AEEVEGEALATLVVNKLRGTFNCVAVKAPAFGERRKAMLEDIAILTNGQVASEELGVKMENVSLDMLGKARQIVIKKDETIIVDGAGSEQAIRDRSANIKRQLEETESEYDREKLQERLAKLSGGVAVIQVGAATETELKERKHRIEDALAATKAAVEEGIVSGGGVTLINAIKAIDKIEAQGDELTGINLVKKAMEEPLRQIANNAGIEGSVVVEKVKEAETGIGYNALTGVYESMIGAGIIDPAKVTRSA
VQNAASISAMVLTTEAVVSELPGEDEMKGMGAGGMGGMGGMGGMM*
>Syntrophomonas_wolfei_subsp.metylbutyratica_JCM14075T_02835_hypothetical_protein
MDSWDRRIKLEAKRFISGWDWDLVYHTWSRRFHDFNVNLTPLFKEIRVYDPNIPLVRRKFFHGAQWILLLNIILIFNVILNVIRVLLGQHITGFKGNLKP*
>Syntrophomonas_wolfei_subsp.metylbutyratica_JCM14075T_02836_Transposase
MPWFFILQRNMLKYFSIIRLMRRICGSFVYWYNLKYQRIGNLFQDRFKSEPIENDKYFLTVLRYIHQNPLKAGLVKSIEDYQWSSIGEYSNSSKFVFTDFALQTRNNHNNDSVDY*
>Syntrophomonas_wolfei_subsp.metylbutyratica_JCM14075T_02837_hypothetical_protein
LDIKDKRQLSDAEATDLIKNVCKVINVSDIKNLDKTTRNEYLRQLKKEYHLSIRQIERLTGINRGVVLNA*
>Syntrophomonas_wolfei_subsp.metylbutyratica_JCM14075T_02838_hypothetical_protein
MDVTMNVNGKYTSNNLQGSARLKNDSSDNTIQNSDKEVLRLCK*
>Syntrophomonas_wolfei_subsp.metylbutyratica_JCM14075T_02839_hypothetical_protein
MQINGYSSATMLAYRSSDMQRKVGLPQTNSFAQTIENTAATEKATKTTETTGVSAADEMATFKKEIYDELEQINGMNSSSILSNSVHITEDGFQRMKDDPEYRKEIMDWLRADARASHGVPFDVHVTTNITGSGATCYGANVYHDDNVMTKSSKKQLADKKAEGAFYRSERKSHREDNSKLWIKEREKRDRLQKMLLEKSINQKIQRQAMNEEYINNYIDNL*
>Syntrophomonas_wolfei_subsp.metylbutyratica_JCM14075T_02840_Nitronate_monooxygenase
LQLPTLRIADYIPRFPIIQGGMSVRVSTASLASAVARAGGIGVIGATGISLAELKDEIRQARSRAEGGILGVNIMFAARQFAELVKTAIDEKIDIIFTGAGFSRDIFRWIQDSTTAVVPIVSSVKAARLAEKLGAPAVVAEGSEAGGHLGTDRSIKDILPEIVESVNIPVIAAGGIVDGKDMAEMVNLGASGIQMATRFVLSEECSVADEYKQLYLEAREEDVIIIKSPVGMPGRALKNQFARLIAEDDAPEPEDCD
ACLKNCSADYCILQALNNAREGQVDDGVVFAGQNVFKIKDILPVSCIFEQILAEFAAAK*
>Syntrophomonas_wolfei_subsp.metylbutyratica_JCM14075T_02841_3-oxoacyl-[acyl-carrier-protein]_synthase_3_protein_1
LKIQILGMGHSLPEGILNNQELEQMVDTSNEWIVERTGIRERRIADKDTATSDLCWQAAKIALERSATEAKDLDLIIVGTSSPDMLFPSTACIVQERLGARNAAAFDVEAGCTGFVYALGIAEKFLLSPEYKKILVIGADLCSRFTDYTDRNTCVLFGDGAGAAVVGKGDLGPGILSSYLGADGSGGKHLYMPAGGSALPPSKETVEQRLHFIRMDGNEIFRFATKIVVAVSEKLLSQAGLSYQDVDFFI
PHQANLRIIKTAMKRMRIPAEKTLINLDHFGNMSAACIPVGLSMAEEAGKLKNGDLVLMVAFGAGLSYGGILLRWGRDQDVF*
>Syntrophomonas_wolfei_subsp.metylbutyratica_JCM14075T_02842_Nitronate_monooxygenase
MFFKTLLCDLLNIEYPLIQGAMAWIAGGNLAAAVSQAGGLGVIGASGAEPAWIKQEIEQVRKLTGKPFGVNLMLAAPGIEKVIELIIQEKVPVVTTGGGNPGPYMERLKAAGIKVIPVVASVALARRLSRLGADAVIAEGTESGGHVGEMTTMCLVPMVVDAVDVPVIAAGGIADGRGFMAALALGAQGVQMGTRFICAEECNVHPAYQQRVIKAKDRDTVICGQSTGHPVRAIRNQFTREYLKS
EKMGAGQEELERLGQGRYPAAAVEGRIDEGSVLAGQICGLVNQVQPAAGIVADIISDACQVKNILGGIECQE*
>Syntrophomonas_wolfei_subsp.metylbutyratica_JCM14075T_02843_Malonyl_CoA-acyl_carrier_protein_transacylase
MPRIAFVFPGQGAQYPGMGKELAEKYEEAAQVFTAADRISPYSISSLCFTGPAEQLNQTEFAQPALLTSSLAALVVAKKLGICADILAGLSLGEYTALVAAGALSLEEALPLVQKRAILMQEAVPPGEGAMAAVLGMEASRLEDFCAMARGQVGIANYNCPGQLVISGEKEAVQAVTASIKEAGGRVIPLAVSVPSHSPLMYEAAMKLKAELAQVKWQEPLIPVVSNVNARENGAPDFEELLAQQLFK
PVLWEQSIRYMMDKADYFVEIGPGSTLSGLIKKIDRHRVLGQVENLSSLEKLIKKVESL*
>Syntrophomonas_wolfei_subsp.metylbutyratica_JCM14075T_02844_3-oxoacyl-[acyl-carrier-protein]_reductase_FabG
MSAKVALVTGSSRGIGRAIALQLAREGYRVVVNYYDNPQESPEIIKENQFQARQVLQEIEAAGSTGIMVGADVSDPAMARELVEAAIKEYGQIDVLVNNAGINKDQLILRINDEEWDKLINTNLSSAFYCCRAALKQMVRKRYGRIINISSVVGLSGNAGQAHYSASKAGLLGLTFSIAREYGARGITANVIAPGYIQSDMTAGFSPELKEKMLSTIAVGRLGTPEDIAGVVAFLASDAAAYITAQVIKVDG
GLS*

>Syntrophomonas_wolfei_subsp.metylbutyratica_JCM14075T_02845_Acyl_carrier_protein
VNVFEELKKIIVEIKDIPEDEIQLEAKFAEDLEADSLDIVEMLMLLEEKFDIQIPEEEAAKLKTVKNVVDYVEAKLQ*
>Syntrophomonas_wolfei_subsp.metylbutyratica_JCM14075T_02846_3-oxoacyl-[acyl-carrier-protein]_synthase_2
MTARRVVITGLGMISPIGNNKKDFWDNLIEGRGGIGQIDRFDASDFPTRIAAQVKDFNVQDYIPRKDARRMDLFVQYACSAARIAVEDAGLQLDAAEAPRVGVWIGSGIGGIQTFEEQHAKMLQKGVNGLSPFFIPMMISNMAAGQVAIMLGARGPNGCTVTACASGSNSIGEALQLIRLGKADVMIAGGTEASITPVAVGGFCAMKALSTANEDPSRACRPFDIKRDGFVMGEGAAILVLEEME
HALRRGARIYAELAGYGASADAVHMVQPDIEGRGAALAFSMALADAGIKPEEVDYINAHGTGTELNDQVETRAIKSVFAEHSYQMLVSSTKPYTGHMLGAAGAIELIASVLSLVNNSVPPTLNLEEPDPLCDLDYVPGKARQKELKVVLSDSLGFGGHNAALVIRKI*
>Syntrophomonas_wolfei_subsp.metylbutyratica_JCM14075T_02847_3-hydroxyacyl-[acyl-carrier-protein]_dehydratase_FabZ
MKKLELNIEEIMKILPHRYPFLLVDRIIELLPGERAVGIKNLSVNEPFFPGHFPGHPVMPGVLMIEAMAQVGACAILCDEKYQGRLGYLAGVDRIRFKRMAAPGDSLLITTEFTAIKGNIGKGKGQIKINEEMVCGGEFLFALG*
>Syntrophomonas_wolfei_subsp.metylbutyratica_JCM14075T_02848_Acetyl-coenzyme_A_carboxylase_carboxyl_transferase_subunit_beta
MEKEEEKKVALPINCPSCSARIPAEALQRNLKVCPKCQHHFSLNARERIDLMVDNGSFQEFDADLSSFNLLDFPGYQEKLEKAQELSGSREAIITGLATVDGHKLVLGVMESSFMMASMGSVVGEKVCRAVEQAMELSCPLLICSCSGGARMQEGMVALMQMAKTSAVLGKFNQAGLLYISLLTHPTTGGVSASFASLADIILAEPGALIGFAGPRVIKQTIGQQLPPRFQRSEFLLEHGMIDLLVE
RSQLKETLSSLLGLHQEG*
>Syntrophomonas_wolfei_subsp.metylbutyratica_JCM14075T_02849_Acetyl-coenzyme_A_carboxylase_carboxyl_transferase_subunit_alpha
MVKRQFDYEQKVREIRDKLEELNNLSSNMEFDLSQEIESLEEKIENNRERRYKNLSPWEKVLLSRHPERPNSNDYIRYFCEEWIELHGDRHFGDDSSVIGGIGRFNGQAVTILGYRKGKDTRENLQYNFGMPHPEGYRKIQRLLLQAEKFQRPVITLIDTPGAYPGIGAEERGQAWAISQVLMTLSALEVPVIAVVSGEGGSGGALALAVADRLLMLSNAVFSVASPEACASILWKDLERVEDMARAMK
ITAHDLQRLGIVDEIIEEPLGGAHLNFPEMAEKLKKALQKHLGELTSQDSGELSEERYQKLRKIGEFRG*
>Syntrophomonas_wolfei_subsp.metylbutyratica_JCM14075T_02850_putative_metallophosphoesterase
LHGEMAGNMRIAVIALFLLLWGSLNFYIAWRGWQYFGSHILNYRILYWVMLVFLSASYFLGRWGNHHYPGQVSNCLIWIGSYWMAFFLYALLITVLLDLIKYLDKLWGFLPAFIHNSSSIIALLIFFLIAGLFYGSCNANRPVLRQYQVNIPKEAGVRKDLHIVMLSDIHLGNIVGRNRLSRLVDRINLIQPEMVLLVGDIIDGDIRPFEEQKMAGLLRSIKAPQGVFAVLGNHEYLGGQYQELVAALESC
GVTVLRDQYHLLENFYLVGRDDKMSRLRKPLKELMLGVNCHYPVILMDHNPIDIEESRLNRVDLHLSGHSHAGQLFPLNFITRNIFVLDRGYLRQDNMQIIVSNGFGTWGPPIRIGNRPEIVEIFIHFGEAERFPL*
>Syntrophomonas_wolfei_subsp.metylbutyratica_JCM14075T_02851_hypothetical_protein
MKNKLLKDVIVPKNRGIIVLCLIIILVITSAGIIGCNKTVPNQTTQPALDETGDEINTELAGKTTNKTETTKEKSIDAYNDSKGYKAVMNSGVGFDIYIPQKERENNQSFFDNLNKQSRLNGYDFSAYLDKPLRYTAMAINSDDSTKDITFLCHDEQIVGVWIDASNKEHLEVRRKLLQYNFVYDAEELLQYKSNYAGDNSNVVHLMYSLPYVNGIKPVGCELQTDSEPYGLIINCQGYGTKERASLPYF
KNAAVIFSLIENVGIVTFNIETNDGKIAFTYTRAQIDNYFKKDIRKHSKNKADFAGFIMEVQDASEIGL*
>Syntrophomonas_wolfei_subsp.metylbutyratica_JCM14075T_02852_hypothetical_protein
LRRIFISLLVLGLFLIPLAAELEKTWAATVTGQALSSDIHAYVNGQMLPCLIINDRTAIVAEDLRSCGFDVIWEPVERKLEISENTSHPTGKTGSRTTTDYAAFAGKDRMILASDIKTFLQQKEIASFNIDGYTAIYLSELAAYGEVKWDPLQRVARFDSSRRSGDTILAESSEEANLIAEEIDRREGIIEFHGEKLLYQNQQVGYAHNGKAMVSLDWMAGWLGYQVQSKQNSYLVKRGSHSFQVKAGER
QVQVFYDGSPAKIVELYEVPSLISTRLYLYSLDLENLFGLDSQWNNEKRQWKVRYADYRIKELGNYLGGSSCTIKAQYSELLSGFNTPPELSVKNITLISEGSDKGGYAYVSGDGKNYAATVSLKVLSDNEIQICLGKKNRVLFYKQMTLRGEVQNHQLERQKIIGPFTEYSLVKPEQGFIQVKQNSFMVEGQVGFTNEAVIHILPAKIDQASGIITELPEQTLSLQEQKFSGPVALNAGPGLYRIFFQVQ
NNGLHGVVATTRFGEFYLDYH*
>Syntrophomonas_wolfei_subsp.metylbutyratica_JCM14075T_02853_Methyl-accepting_chemotaxis_protein
MTSIGIIGGGKGGTTILGAFHEIEEFRVLGLCDVNAKAPGIKLARELGVPAFHDLGELLRIPGLEVIIEATGSEKVRDMALAMKPASALLMDSHMANMMMTFVEGHERILKKARSKKEAFRTSAPFLTPTYGKDGVIYFTTNTERYDFVENHNMDIAGIRMGEPLVKNGVVQRCIQTRQAISETVNEKIYGISLNLWAAPIFEDDDEHNAVVGTYGVFAPRLHPVVKAFDIFAPIIIDSQPEGAWVGAS
DTEKVLCRMGSEKFDTNVVVVGLPISEVKTAMQTIQARKRVQVDINTRKLGNIRMISIPLFDEESGELVGTFGITTPRNLASNLQNAADKLRSHTDDMARVMQQIATSAGEINITESKLADIIKSIQENLENIVRVLSFTKDVANQTKMLGMNAAIEAARAGDLGKGFGVVAGEIRKLSDQSKQTADEIGQLTRAIEIRIKDAVEASQGTVMQSQEQAAATQEVTATVMEMSEVAEKLATLAKSL*
>Syntrophomonas_wolfei_subsp.metylbutyratica_JCM14075T_02854_Bacteriophytochrome_cph2
LIIVMFSFFAINIGMNEEFNIYSILGWLGLIAAIILLLAYLRHHKFVRQSTPEDANLKKILDELNIATWALDPYSGSYSFSNSIESITCCKARDLMLNPTLWSKMVHPYDILLLREADKELMAGRKSMLEYRIMLTDGRVRWIQRLGIPVMDQAGKVKRLTGLIIDVSEQKQEEENIKQKAYYDTLTGLANRSMLEYYFAHCQASSRHFAQNLAIVFIDLDSFKAVNDQFGHNTGDILLKMVAARLKNLLRG
NDLLSRLGGDEFVVLLTRVSRESLIGVAERIMGTFSEPFEVEGYRLMIGASLGISIYPDDGEDLEILIHNADQAMYQAKNQGKNNYRFYS*
>Syntrophomonas_wolfei_subsp.metylbutyratica_JCM14075T_02855_Penicillin-binding_protein_E
LNKFRVVAACLIALLLAIMIPASFNAAENPALSNTRLTARSEIWKAINAGNAGSGTVAVMENGKIVYAEGFGMADRERSISVDKNTLFNIGSISKVYCATAIMLLVDEGKVELDQPVTAYLPEFTMEDERYKDITVRMLLNHSSGLPGTIGSNSFGFEYHKEYYQDVLDTLAKSHLKHRPGEMAPYCNDGFTLAEMIVARISGKSYLQFLSERVFEPLGLNHTGPGVGQRQGLKEGSVARYYTTDGKSE
PLEVVSLLGSGGLSATPEDLCKFADTFSASGPQILSPASLAEMRKGQPAESYMQLKQPELSFGLGWDLTEIAHYKAKGINVLGKSGGTGNYSSMLYTIPDKRISVAVIFTGPQSNAMVIAASIMEAFLCENGLFDKTPAVLKLPLKAQSLPSELANYEGYYSNGSVLKRLKVDRAENKLTVYMLEGGQETEILSAIYNDGYFHNQRDKYYFITLGGRQYFVQHSATLAVDMIASQKLEPVAKASELKISV
DKRKWLRRNAKAYEARMLVSGHIITSRQFSELPGYIDFDGIKRVISPTSAGMAINSMRDLTELGLSEKDGISWAWISGMLYTPADAVQSLASGDTNITTGREGYNEWRKVAEDSILDFQIPNKGRVIVFDPDGQAIYDSAVDSGEVLATTGSFVEVAGNPGATFKLSASSPL*
>Syntrophomonas_wolfei_subsp.metylbutyratica_JCM14075T_02856_prevent-host-death_family_protein
MFNVTEVRQNISKIITQVIETKEPIVLLKRSKPVAYIIEAKVFEELQAKAKKAESYEKTEKAKDTLQRLAKLRGEMKPQPDSTPLIRRLREGEERSE*
>Syntrophomonas_wolfei_subsp.metylbutyratica_JCM14075T_02857_putative_nucleic_acid-binding_protein,_contains_PIN_domain
VSSYICLDSSVLIKVLVNEEDSDKAVTLLERVINERMTVVLPDFAWAEVGSVLRKKVNRRLINPKQAETAWEAFNSFGVITFINGVSVMRAAWQIADKEELPTLYDAAYLAVAEIMAKQNGKECEFWTADNKLASSVKNKSYVKVLQSCIIF*
>Syntrophomonas_wolfei_subsp.metylbutyratica_JCM14075T_02858_Spore_photoproduct_lyase
LFPEKIFYEPPVLHYELGKQLQEKFAHIPWIAIENHNNIEELRKNPNKEFVHMKRYLILGVRKSLKYTPNHKVSDFLVPYTSSGCSAMCLYCYLVCNYNKCSYLRLFVNREQMLDKLMKTANRSPGDLTFEIGSNSDLLLENSISGNLEWTIENFAGSKKGFITFPTKFDMVEALLPLNHGGRTIMRMSVNPQPIIQRLEFGTSSLTGRIRALEQMHDAGYKVGILIAPVVLLDNWQELYSQLIEQLADEL
PDKLKKELFIEIIFMTYSYIHRAINAEAFPNAVKLYDKSLMTGRGRGKYCYRSDIRLQAEEYLKELLEQKLNGIPVIYIV*
>Syntrophomonas_wolfei_subsp.metylbutyratica_JCM14075T_02859_hypothetical_protein
MRSTLRALLLCFLAALALGNEVLWNMIREKERAGGQGKRDSIYRFWEEGLKKKSTHPQKGILVVAKTLVGSGGGAVGISDKGKRLSEYILESIKEQLLLETGKNLPAL*
>Syntrophomonas_wolfei_subsp.metylbutyratica_JCM14075T_02860_hypothetical_protein
MQKGLATRLKDLKPYIDSEVKLKTELIGSESGGGVAPDSITALIAAYSTAYIALHDAVTECCDNSRTGINVLLQGREFSNYRFSYIYNVIYDTVY*
>Syntrophomonas_wolfei_subsp.metylbutyratica_JCM14075T_02861_hypothetical_protein
LEKIAVIDVGEWQTSLDFRTGKKLDPIPVEPGVQALQKLLRSYPYPGDLSPDSLAWVSNLGLELIHLFEPRFIFLSYATPFFLSTFALSESYNWKEVAHECIEQACNFARVAGFTPVIVGTGSTRPLEGEIDLTYLDGLAIGTGMGVSYAGLYNPSVADLKMLAGDSRVKKIIPQVTIQAEWPCQPDFARRLPDYLLVAQPGFQFRGMGTMSRKIYRISSHDPFIPVLSPRPLDDITGIAPLVRELLKTERVA
LIILEGIGSEDFPTEQQLINNQYSWYTYSPGDAQYLAITSGIHLPGQQYPPGNRCYDEDGAEKPYPFSGIYHSMPEKVIGSDPGLQSVAVGSRSILTHLASGARVCVECFARTLYNYGIMGIIEER*
>Syntrophomonas_wolfei_subsp.metylbutyratica_JCM14075T_02862_molybdenum_cofactor_biosynthesis_protein_A
MYREEQGKVVEIYPFAWNSFGVTHIESLPFFHAYPGSRSLLLGSAGCNFDCQYCSNAHVARVNPKSVNLWHLQPEKLLTKARQSGCHNIVFAINEPTVSWPSLRALGETAREQGIEMGCMSNGYFTPRVANEMAETFSFINISLKAMNRDFYQRYIRVTDIAPVLRNIAYLAEHTHLEITTPIIQGLNDGDIPSAASFIASQDPEIPWHVFRLLPEYQMKDMLAPSIEAIEYALQEQENKLAYIYFGNFVGS
EWVNTVCPDCGKVLVERINLGGCGAKSLACHLQGDCCPSCGRRLRLLGEWTSWNKGGAQIGKNSSY*
>Syntrophomonas_wolfei_subsp.metylbutyratica_JCM14075T_02863_putative_ABC_transporter_permease_protein
MKAQRLVLLGLIVLAATALFTMLVGRYSADLSTVLQIFKLHLYHIAIPEDLVSADIVIWSVRLPRLLMAAMVGAALSASGAVLQGIFRNPLVSPDILGVSSGASFGAGLAILLIGTSTLAVSFFAFVGGLAAVTLAYQIGRRGQNTITTLVLAGVIISALFSAALSFLKYVADPYEQLPAIVFWTMGGFNNIDWADLGRAFPLVAGGLLLLYLFRWRLNLLALGEEEAMGLGVNVKRALPLFVLLATLVVAAS
ISACGTIGWVGLVVPHMARIMIGPDHKHMLPFSMIVGAVFMLLMDTLARTITGAEIPISIMTSLVGAPFLGYLLLREQDKEWNG*
>Syntrophomonas_wolfei_subsp.metylbutyratica_JCM14075T_02864_Ferric-citrate-binding_protein
MKAFRNSVMLILMVALLALPGCGKPATAPQNEGAKAVSSIVVKDGLNRDVKIDGNVESIVSSYGIALHMVVALGAADLIQAIDVPSMKSDFFKATIPQNAQGKSVGSPRELNIEEVAALDPDLVLVPGRNQEMVESLEKRGITVFGVVAETPQELNLTMLNLGKALGREQVAQEFVDYYDQTIKDVKDRLAGLEKSQRPRVYLVGPMGILSTCSSDMYQHELIAMCGGENVAAQLQGKLDGHG
WLEIPAEQLITWDPEVILVVQYSGAKPADILNDSRFRNIQAVKDKKVYWFPSDLNPWDYPSPQAVLGVKWLAATLHPDCFENVNMQVEADNFFNRFYGKSFTGMGGKL*
>Syntrophomonas_wolfei_subsp.metylbutyratica_JCM14075T_02865_Iron(3+)-hydroxamate_import_ATP-binding_protein_FhuC
MDNTILRVENINFAYKKRQVLNGISFSVKAGCFCALLGPNGSGKTTLLKCINQILFQDSGNILLDGQTASSLGRRDIARLVAMVPQQTHLVFGFSVMELVVMGRSSSLGTTGLPGRQDYHRAQTALEELGISHLAQRKFNELSGGERQLVLLARALCQEPRILLLDEPTSHLDFKNQFLILNKVRGLTRDRGLASLISIHDPNQAARYCDSVVMLKDGAVLKQGLVQEVFQPLSLAELYGMEVVVQ
RSSTGSDFVLPRYY*
>Syntrophomonas_wolfei_subsp.metylbutyratica_JCM14075T_02867_Neu5Ac_permease
VLPLVEMLTIVILTRRNPDLGPAGPKANWQERLVALKGGLGEVLIIFAISMGRLFAGWFTPTEAGAVGAAGVLLVSLLGRSLNWEGFKKSLLDTTKTTAMIMLLIAGAVIFGRFMAISRIPFALASWAGDLALPAAAIMAILLIYFVLGCFIDALALALVLLTIPIFYPIVVNNLGYDPIWFGVIIVLVVAMRVITPPVGMNVYIIKGVAPDVPLETIFKGIWPFLLAIVVCLAILITFPAISTVLPNLMK*
>Syntrophomonas_wolfei_subsp.metylbutyratica_JCM14075T_02868_L-dehydroascorbate_transporter_large_permease_subunit
MGFLAYYSGIGTRLYVFAYKMVGQRPGGLAIATQAACAVFGAICGSNTATAATMGAIALPEMKKYQYEDAMATASVAAGGDVNHSYTYPP*
>Syntrophomonas_wolfei_subsp.metylbutyratica_JCM14075T_02869_hypothetical_protein
MVERQPDSTPEENTYLKLIQDNSGNESEDARTIINWVAEQLKADMGKIVRIIRSAYGRGHFDARDNSQMNFNPGGELTAIVTPLVDRVLSGIYESRDIKFDGPSSFKKEDGVKIINGIVKTGSIPRRAKPNQNISAVQNFATGLKIVKKSA*
>Syntrophomonas_wolfei_subsp.metylbutyratica_JCM14075T_02870_hypothetical_protein
LRPYAERLLNATLEETPDDALISQYRQQLRQHFAEEREASRIKRLAQDLFNMLELSNPYQEEVMQVKALYQTEIESGNDINLILYALKEVFTYRAFDESQVFEEEVADLALRMNRYRHIKTFLRYEREIQTAYAYCKQEIPDLPQLKEIRKMQKGLATRLKDLKPYIDSEVKLKTELIGSESGGGVAPDSGWASIR*
>Syntrophomonas_wolfei_subsp.metylbutyratica_JCM14075T_02871_Lon_protease
LRLIPDGQVLDEELEEVLLLSCELRQRVREQLHLMAQGEYERVKLGVKILSSGKVLAPNLPDAERIQRISLPVEPRVGEVLGLAVAGDHGCTLHFEMQASKGHGRIVPLGSIQKIMRESIEAAAQYIKTHHDELGIGAQWRENYDVAVLATYMGVPKEGPSAGIAMVVGIVSALKRVPVRNDLAMTGEITIMGKVLPVGGIQQKIRAAYDAGIKEVLLPADNLRETENLPAYVLKSLKITPIISIAEALDK
ALTKFDITAIF*
>Syntrophomonas_wolfei_subsp.metylbutyratica_JCM14075T_02872_hypothetical_protein
MVIVNTIREKTIVKPGGLVEARSGELPVGVTVEATIVVERDNTQSVTRSLVNLIGSAKGCFLSPQEADEFIRGERDAWD*
>Syntrophomonas_wolfei_subsp.metylbutyratica_JCM14075T_02873_hypothetical_protein
MLHKKKVIIGIILSLAVITIGAVAVLINANPKPTLIAPDTKKPSKIQNTSKDSQMNQQELSSEKLKLRTEYYYLLKDLDNMRFFSDFPLSENIPVEELLLFGYMKALEAGWQDDSGAVKLANVNTFTEKHFNRTLMSNIDTSYFTYDANGDSYIPTGWDFHGSDPHYLARISNNPDGSVTAIFDVYLFGESDILDNKSPEEYIGDTSLIPPELKHLRAESSFYRKADNGKTYLQMLSYKRLYDEAKNPIASK
ADASKSVPYCPDEYLSDDEFVFANGVRLGMSYEKVIEILGGYDEAFDNAPDVKSIIKDGCHYGFYQINDKYTNQSDLKTDGVFRLLNLSSESYQGEFPRGIRIGDSIEDVLNKFPGKDKQLRKWAHQTIYGKDEIGSPRAYLQFTMYNECYRFLATTSRQVLIINFDRKNQVRSVELHKEDS*
>Syntrophomonas_wolfei_subsp.metylbutyratica_JCM14075T_02874_putative_oxidoreductase_YdhV
MFGYAGKMLEVYLSTQKIKTRKLEPDLARDYIGGIGFNARILYDEIPAGADPLGPDNVLVFSVGCLVGTPFPTASRMEVSAKSPASNGFGSSNSGAFLGLRLKCAGYDGMIIKGQAQKPVYLLIEEDTIEIKEASFIWGKDAWESIDLLRARHYGAEIILIGQAGENLVRFASIENGYYDGFGRTGMGAVMGSKKLKAVVVRGSKPIVPADPQGVLELSAKGQKLIKSASSYQAFCAYGTMNATIPYGGFN
ALSVHNYSRGTLPDWKQKAGRQIVDIYGSRHIACQSCIIACGHLAEINEGKYAGTLVKDMEITPTVSYSSNVGLSTEASIKSSELCQRYGIDMSSSGSVIAFAMELYQKGIINKDDVGYELAFGDDDAAFALLRDISLRQGIGDILAEGVKRAADHWPGADDYAIHVKGVEVPMIDPRGRWSTWTLGMLTNIRGGDHLRCRNPVENLRYNENLYHYQKERFGFKKPMYDRLDMPENLKSAAIDLESD
TVDIAIMSKWAEDLINLYNSVGICIRPPVMETIGPTLLAEIYTCMTGIPMSPDELMMGSERAWNLMKLFNIRHGEVAGDSKFPRRFYRELQSGNIVDEDKVQAVLEQYWQARGWDPGTGHPLPETEKRLGI*
>Syntrophomonas_wolfei_subsp.metylbutyratica_JCM14075T_02875_putative_ferredoxin-like_protein_YdhX_precursor
MKQKRLYHNTSLCDGCGLCQLVCALEHCGQWNHYQSRIKIVNDMEREAFYAVCCTQCEEAPCILTCLMNTIYKQPGSDITVRDLYHCIGCRACELACPFDAAVFDVIQEKVVNCDLCGGRPLCVEYCPNHALTYDYPGDQADKKRNQAALQRFGTSLLEVFKFSGKEA*
>Syntrophomonas_wolfei_subsp.metylbutyratica_JCM14075T_02876_Nitrite_reductase_[NAD(P)H]
LRIIIIGNGAAGNQAAQTIQDYDPEAEITILGAESIPFYSACALPDYLAGWIKRDQLFLRSSLERHRVEIRLGQAVEDIKPDRQAVSAGGELLFYDRLIIASGSRPVLPNIPGTGLPGNFVIKSVYDIDRILSHNPRRIVVIGSGNIGIEAAEALASRGCQVSIIEMMPRIMPRLFDEQPAMLIRNILEENGIEIHTGEKAREVIGKNKVEGLITDCRRISCDAVIWAAGVRQNTELARAAGIEIGGLGGIKVDSQMKT
SHPAIYACGDCIETSDLLTGKPALCLLWSCAKAQAEVAARNCLGEEIVYPGALKLIAEEAYGKPCMAAGLLEEELDRTGLKIIEKETENTYHRILIKNERIMGIQAVGSMEAMGPLLYFMKKMIAPSEIKRIINSPALLKNMPWMLAIDKYWQ*
>Syntrophomonas_wolfei_subsp.metylbutyratica_JCM14075T_02877_Bicarbonate_transporter_BicA
MKIGMFSEIHGEISGAIADLGTFLPYVLGAIIIGGLDASSIFITFGLMYIFTGYFYRIPIPVQPMKIIGAAILVHHLTAGEVAAAGIMMGLTLFILAITGLASRLAGLTPDSVTLGIQAGLGVSLAMLGIKYIKTDLLLGLIIMLFMLFLFQNRRFPASIAGVVGGTLLAFVLHPELHWPGLQLGFYWPHLILPAWTDFARGFTLAYLPQLPLTLTNSVLVTAILAHELFPKQSEKVNEKNLCLTLGIGNLLAAPL
GGFAMCHGSGGLAAHYRFGGRTGFTPALMGIILLFTGIFLGPAGVDLLQIIPQAVLGGLLFFSGVDLVRGVQDFGDKKALFCFAVVLIISIAVNPALAFLVGLILHFLFNKGWVKI*
>Syntrophomonas_wolfei_subsp.metylbutyratica_JCM14075T_02878_hypothetical_protein
MNFYHYIRFPFRDEDWIKKILLGCVISIVPVLNLLSLCYFVECMKFGIAGRQSLPEWERWEDFVREGIIAFLISLGYLLIPLLLAFPLLHIPLVGVFLLSIIILAVGMIFPLAIANFLKDHYLMDAINVKEIFYMMNRVASNYIPAYFIAVFLMALGLTIVFLLPYLSFLGVLLIFYCGMIFFHFTGSLINSRI*
>Syntrophomonas_wolfei_subsp.metylbutyratica_JCM14075T_02879_hypothetical_protein
MSKVEAYETPIMSLEEFNVGLLEKDRIPGLLIRSDEQGFYNIGVQINDREVVKVASAMEDDAMYKIQFWADKVDQIKDQYKERQPQLK*
>Syntrophomonas_wolfei_subsp.metylbutyratica_JCM14075T_02880_hypothetical_protein
MHNKKGKIMSKDDFLQNSLYAKEEYEGLLVSSDTNKGVYNIGIELGDNQILLIDQVKDSEVHERVHMWVPQIQEIQRRYGVEGDLGNYSRS*
>Syntrophomonas_wolfei_subsp.metylbutyratica_JCM14075T_02881_Putative_pyruvate,_phosphate_dikinase_regulatory_protein
MADKLHIFAVSDSVGETAEKIAVASVLQFKLERNITRFSRVTKEEQVEKIIEQAVENDAIIIYTIVKPELSRFMDETARVKQVIAVNVMAPLFDAIQFKTGQKPEYITGLTHKMDDQYFNRMKAIEYTIEHDNGQNLDSVHEADLIVLGLPRTSKTPLSMHLANLGIKVANYPILMDTEIPQEIVDLKGRIPMVGLTIDIDTLMELRKERFRSFELPDDIETLDDLVAEELDNAYRIYAKLKCLVIDVTLDDI
EEVGNTITHKFNLPLRITHHRF*
>Syntrophomonas_wolfei_subsp.metylbutyratica_JCM14075T_02882_Cell_division_inhibitor_MinD
MGKKTEESSVIVVTSGKGGVGKTTTVANVASALARKGYKIAVMDLDIGLKKLDLILGLENRVIYDIIQVIEGECTLKQALVKDKRFPELYMLPAAQTRNKDDIKPEQVQEICRQLRPEFDFIFIDCPAGIEQGFRNAIAAADKAIVVTNPEVSAVRDADRIIGMLESAQFQDIQLVINRLQASMVKSGDMLSIDDLLEHLCITLLGIVPEDKKVLISTNKGEPIVLNERSEAGLAFNNIANRLLGQQVDFPSF
EEDSLWGKIRNLGRNLFSKE*
>Syntrophomonas_wolfei_subsp.metylbutyratica_JCM14075T_02883_cell_division_topological_specificity_factor_MinE
VLDFLKRIFADSSSRKQANDRLRVVLTHDRTGTSSRLMETLKEEILEVISRHVEIEGRPEVKIIREGRHSALDINIPLKGR*
>Syntrophomonas_wolfei_subsp.metylbutyratica_JCM14075T_02884_Septum_site-determining_protein_MinC
MVKIKGLNGNLVFIFGPGSFDEYLSFLEEKLTANKQLFSGSRVIFKGEELKKLSHSQIASLQELCLRHGVIMNNNAVTVNKGSSKDLVIYRNLRSGQKLRSEGSVIIWGDVHESAEILAAGDIIVLGKLDGIAHAGCYGDMNRLIFALSLSPGQIRIGDRLSRSPEDPDKSPHPEIAFWDGENICIKAYSSRSGLRG*
>Syntrophomonas_wolfei_subsp.metylbutyratica_JCM14075T_02885_Adenylate_cyclase_2
VNLSYESHPVEEKKPRRIFSGISHNRFFIPILLFLLLQGLVMLGFFTRSDLGLYDTFFRLKGASTPGEKVVIVAIDDYSISRLGPPAWPRSVHARLLEKLEEARAVSFDLTFNSTQGAREDQAFASAIQKHGKVILAAKFTFEKDESGDLVQGFEPPLQELMSGAAGLGFVNTPTDIDQRVRRSTLVDVNSFEVPFPSLAIATAMVAQDIGYEELKLEAGRLLLGKKEIPLSPLNQAMVKFWGPKATFKTISY
ADVIEEKIPPAYFQDKIVLIGTITPEDHDFVTTPYTTSNMVKGGALDTPGVEMHASIIQTLLEDSWYREAEKPFNIAFLFLAGLLTSLVVRGRGPWKGLIGTVLVTLASIALVYGLWEYQRIWLSLAAPLALILLSYVVVTATEFIQAELGRRKTKAMFSRYVSADVVDELLKNPDEISLGGKKQVVTIMFADIRGFTAFSENKDPVDVINRLNEYLTAMTDSIQSHGGTLDKYLGDGLMAFFGAPVYFE
DHVERAIKVAREIQVKVADLNQKWAEQGAVPLLVAVGINTGPVVVGNVGSPERMDYTLIGEDANLASRVEALTKLFETLVLVSERSYDLLPEGKTKASLQYVGEELVKGFTHPIKVYSFSDLDLHFEKSSDKGFK*
>Syntrophomonas_wolfei_subsp.metylbutyratica_JCM14075T_02886_putative_ubiquinone_biosynthesis_protein_UbiB
LGLLQRIRHIRRRQQIVGVLVKNGFGYMIQRLGLDSLTPLAERPTLVAERPGDRLLAQKLRQSLVELGPTFVKLGQVLSTRPDLLPPVYIEELERLQDKVEAMPNVELMKQLLSELGHPDEVFAEFNPEPLAAASIGQVHLARLKSGEEVIVKVQRPGIEKVVQNDLEIVLGLAHLSELRSSEARRLGIKDMVEEFSRMFMRELDYAREARSTERVYQNFAGDQRVVIPRVYWEYTTGKVLTEEYLSGV
KLSDLEEIDKRGWNRRKISQLGTETFLSQIILHGFYQADPHPGNILLLDENRIAFIDFGQIGSLSETRLIHLGELLLAISKQNMDKAMSSLQDMGIVGELDDIEDFQEDFADLIKSVSGSIGKLDINRLRNELMDLSYRHQLKMPAYLTGLMKALITVEGVGKKLDPGYDFMETARPLANKVLEERLKAESVYKYIRRKYYQDIKPLAALPSNFNKLVRRTSEGQLKVRMELDLSDKAYHGISRLISRLSASL
IITGALIGSSFILQANHAAWVEEYSFLGVLGFGVAIVSLVVFLISSLRP*
>Syntrophomonas_wolfei_subsp.metylbutyratica_JCM14075T_02887_Putative_aliphatic_sulfonates-binding_protein_precursor
MKKGVWLLILVLLLVGGMLFWKHGGLGPEPPAQITLINPLGPTVIPVAGISSQQVKSKMPITIQYWKDTDEAVALLASKKADFAVLPISAAANIYARGIDIKMLGVHEWKVFYLLAAKDTPFEGWRSLVGKRVYTAHGRGQTADVIMRAALSKEGIEPDQDVKILYAPPQEIVALFKAGKVDFAALPEPFVTLAMSDDSGRIVLDFQKYWGESTGKPERIPVAGLFVAGDFQEKYPRETKELAQIFAD
STRWSNENVDQALAVAAETLPTIPQPVMKKALSRIEFEFIDSSKSRPEVEFYLQKMKELYPQGTPKLPDKDFYYS*
>Syntrophomonas_wolfei_subsp.metylbutyratica_JCM14075T_02888_Putative_aliphatic_sulfonates_transport_permease_protein_SsuC
MRTGLRSLFGIIVFILLWQLLAVLLDKSIIVPTPGETFGLFFQLLLSPVTLLAGLQTAWKVLMALGLVLLLGLPLGLIIGLFEPLYDMSRPIIMVIQAVPVISWLSLVLFTWGRNWQGPVLIAFLSILPIAILTTVSGVRNLDKKLLEMARLYQVPARKIMHDIYLGSLLPFIIAIIDVSLGQAWKVILVAEYLCGDSGLGLKIFDARSFVDTPSVYAYTLLAVLLGIITERLIKMGTGRLSKLWVPV*
>Syntrophomonas_wolfei_subsp.metylbutyratica_JCM14075T_02889_Aliphatic_sulfonates_import_ATP-binding_protein_SsuB
MGARLEVRSLHKSFGALSVLDNWNLSIRESERIVLLGPSGSGKTTFLRLIAGLERPSSGEIKLSSQQMGFVFQEPRLIPWRTVEDNLLFVNEKGDISGILEKLRLSGFAGFFPAQLSGGMQQRVNLARALLVEPDLLILDEAFSSLDLPVKTSIMKDINLHWQEKRFTIIAVTHDLKEALFLADRIIILSPAPSVIAREEPVTLGKGRNFSSPEFLHLEGQLLDIICSASN*
>Syntrophomonas_wolfei_subsp.metylbutyratica_JCM14075T_02890_Ribonudease_Z
MAKRNFEVTFWGVRGSLPVPGPHTIKYGGNTSCVQVQIDQRLFILDAGTGIYHLGQHLLKSQSPISGEIFISHTHWDHIQGFPFFAPAFIKGNRFILYGQSKVNSTFADLMKGQMIYQHFPVSLEGMGARIEFHELDNGSEMDLGEQIRLRTVHTNHPNGSLAYRLDYDGRSCCYITDTEHYSCVDPHLKAFCEETDLMIYDSNYTDEEYPRFLGYGHSTWQEGIKLAQAAGAKKLVLFHHDKARS
DEEMEKIEKEAQRCFPNCIAAREGLVIAL*
>Syntrophomonas_wolfei_subsp.metylbutyratica_JCM14075T_02891_Cyclic_di-GMP_phosphodiesterase_response_regulator_RpfG
LRSYTPEILEQIEDIINIGIALSAEKDKNRLLEMIVSEARRITNADAGTLYLCENDCLSFKIMQNESMNSFLGGRGEKINIPPVPLKMENVSAYAAISHKSVNIPNVYQAEEFDFSGPKKYDESTGYRTLSMLVIPLENHEAEVIGVLQLINARDENGKLIPFAPYFENVITSLASQAAIAISNMQLVADIEELFNSFVQVMATAIDARTPYNANHTRRVALLAGEMARAVNRQSQEYWAGVSFSEEQIAQLEMA
GWLHDIGKITTPLEVMNKSTRIEHQLELVLLRFDYILSSSEAEYLKEKQALLAINSNNSREQLEQLERAWEDKQCYIKESREFIIKVDHPSTFVDRKIVEKLKEIAVQSYRDGSGKLQPWLRENELKCLCVAKGTLTEQERKIMEEHVVVTGKMLEKIPFTKKLQDVPFFASIHHEHLNGKGYPRGLSGEQIPLEGRILALVDVFDALTASDRPYKKALPLEQALKILGFMVKDGELDGDLLQLLVEQRV
WEKI*
>Syntrophomonas_wolfei_subsp.metylbutyratica_JCM14075T_02892_Cyclic_di-GMP_phosphodiesterase_Gmr
MFKLAYLALDKVNQGIIILDKDLKIFFWNAWLEQYTGKSQDETIGQKLPQLFPDLSRKPCLDAFQGALFHQQSRFLSGALHHCFIAPAGDIRKDVRQNLLVEPLYCDDEEYILLQISDLTGHYSRVKQLKHMIKEIDNEYEQVRAAEKINRFKAFHDALTGLPNRYLFYDRLDNAISMAQRNKEMLAVAFLDLDGFKEVNDSYGHEAGDLLLQAVAERLRELLRESDTLARLGGDEFLLIFPQIKGEA
DAATIAEKILAALAYPFMFWGLEIYLTASIGISLYPKDAKDSRNLINKADLAMYQVKNSEKNDYHFYNSSI*
>Syntrophomonas_wolfei_subsp.metylbutyratica_JCM14075T_02893_flagellar_motor_switch_protein
MLNPLQKDVLTELVNVYVGYAANLLSEMVGLRILLSVPRVELIPEAEMEQGGLPLPEIFSPGHIVSSYISFGQDFRGKAFLVFPVQQAKLLVNACTGEQLLLEEGDAVPPRFLDTDFDVLKEISNLILNAVIGEFGNLLETRVVFTVPEVELIDVTESDQRLFLKDGVYILILHTTFTVAEIQVQGVIMIALNLNSVSCLLQKIDALLEGQDV*
>Syntrophomonas_wolfei_subsp.metylbutyratica_JCM14075T_02894_Chemotaxis_protein_CheY
MGVVVVKILIVDDSQFFQGFLKKLFHNYLPEAVLLTANNGRDAYALYEQERPEFVITDLLMPEMNGQEFLQVLKESNPQVKVIVISADIQKATRREIEKMGALAFFNKPLNNEKAGELLALLKEGSYA*
>Syntrophomonas_wolfei_subsp.metylbutyratica_JCM14075T_02895_O-acetyl-ADP-ribose_deacetylase
MKISSVEIQVVQGDITRQEDLAVIVNAANSSLRGGGGVDGAIHRAAGPELKKESSALAPIGPGQAVITGAYRLPNRFVIHCVGPVYGVHKPEEELLASCYRNALRLAEKRQVDSIAFPAISTGVYAYPMREAAQVMFKTIIEAIPALKHIKKIRIVLFDRPAYELHLQVLEACQAE*
>Syntrophomonas_wolfei_subsp.metylbutyratica_JCM14075T_02896_Extracytoplasmic_solute_receptor_protein_YiaO
VKKARFVGGKRSWRPGKILLSLLLVIALLAVGGCKTSPTSTTSKEAPKKSQTQTIELRLAHFWPSTHPAEVELVQPWAKAIEEATGGQVKIVSYPNQTLLQADSIYDGVLNGIADIGISCFSYTRNGFPVLEVFELPGITYESSRVASTVAWEGIKELNPKEVQDTKLMMVLTTGPGDIYSKVPVRNLQDLKGLEIRATGLSAKTLQALGATPVAMPQSDAYESLSKGVVKANLGPVEVLKGWRQAE
VTQYLTQTPFLYNTLFFVTMNLDKWNSLDPETQKAIEKVNEKFFKEVAAGLWDKQNDEALKWAVDEKGMELIQLPADEAQRWIDLVKPIQDDFVKRMDKQGFEGAKILNTAKTLADKYNKEFK*
>Syntrophomonas_wolfei_subsp.metylbutyratica_JCM14075T_02897_TRAP-type_C4-dicarboxylate_transport_system,_small_permease_component
MAKKYNGFVAGLSQFLDQIAGLSLVAVMLVVVGNVLMRALFKHPILGTYDYVGFLTATAIGLALAHCALQNAHIAVDFVVERLPHKTRALIDTATNSVAITFWGFALWNLAIYAGTMKTNGIVAATSQLPVSPFIYLVAFGLFSLCLVLLSHLGESLRRVAR*
>Syntrophomonas_wolfei_subsp.metylbutyratica_JCM14075T_02898_Neu5Ac_permease
MNPFGLGVLGIIVFLVLLMLRMPIAYAMALVGFTGFALLSSASASFSMVAKEIFNTFSSYSLSVIAMFVWMGFLAFYSGIGTRLYVFAYKLMGHLPGGLAVATQLACGIFGAICGSNTATAATMGAIALPEMKKYQYDTSLATASIAAGGVLGVLIPPSVIFIVYGMATEQSIGELFMAGIIPGILLMLAYIAAILFLTLRNPALGPAGPRASWKERASSLRGGLFEVLVVFALSIGGLFAGWFTPTEAGAVGA
AGVLAVSLLGRHLDWTGFKKSLYDTTRTTAMIMLMIAGAMIFGRFMAISRIPFELANWAGSLPLPPFAIVGVILLIYLVLGCFIDALALILLTIPIFYPVVVKTLGYDPIWFGVIVVMVVAMGVITPPVGMNVYIIKGVAPDVPLEDIFRGIWPFLLAIIFSLIILIAFPSIATFLPQLLHGV*
>Syntrophomonas_wolfei_subsp.metylbutyratica_JCM14075T_02899_putative_domain_1
MNYQQHFMRLERMYLSAPYNRQLYHNNTIKISREQAIITTEVMEKHFHGAGAMHGSVYFKLLDDAAFFAVNSIIKDYMVYTVSFNVNLLRPVGAGVIKSIGIVKHQARNRLVADANLYDPEGKLAACGTGNFMRSPLKLGEMPEYTRELSDEG*
>Syntrophomonas_wolfei_subsp.metylbutyratica_JCM14075T_02901_ABC-type_uncharacterized_transport_system,_periplasmic_component
VRKSLPRLLLLPVLLLLVFSLAGCAQEKEKTPPKTAEETEMKTIGIVQIVEHPSLNTIRESLVKQLAEKMALPLSLLTPM*
>Syntrophomonas_wolfei_subsp.metylbutyratica_JCM14075T_02902_hypothetical_protein
MSRERLTLRVRLVFHPTGVTIKVASYGREIGALPLEPRDKPLVLPMRSALSGTTDAPCGRY*
>Syntrophomonas_wolfei_subsp.metylbutyratica_JCM14075T_02903_ABC-type_uncharacterized_transport_system,_periplasmic_component
LIVAIATPSAQAAVGETREIPIVFSACTDPVGSGLVSKMEKPGGNVTGTSDAVSAEKIMELAKRITPNIKKVGALYNSSETNSIAVVNNLKDYAAQNGMTVVDATVTNSSEVQQAVSSLIGKVDAVFSPIDNTVASAMPLVAQVANKAKMPVYVGADSMVKDGGLATYGINYPVLGQETANMAVDILNGKKAGDIPVKSMTDMDIYLNQATAKAIGIDIPADVMKEAVQVFDK*
>Syntrophomonas_wolfei_subsp.metylbutyratica_JCM14075T_02904_Autoinducer_2_import_system_permease_protein_LsrC
LVGLALAFAAGCLAGSVTALLHTKLNIQPLLSGILVMLGLYSINLRVMDGRANIPLLNKATVFSLLQGTPLEESAALLIPTIILLSMLVLLFLFLKTQFGFVLRATGDNDQMVRALGVNTDRVILIGLALSNALVALSGSLIAQYQSFADISMGIGMVIIGLASVIIGEVIFGTTPLLRRLVAVILGAVLYRLVIALALELGMAPTDLKLISALIVTLALSMPSIKAYLIAWKKKHSSPYSRKGGQA*
>Syntrophomonas_wolfei_subsp.metylbutyratica_JCM14075T_02905_Spermidine/putrescine_import_ATP-binding_protein_PotA
MLIIESLSKTFNPGSINEKLALDNITLSLQPGEFLSIIGGNGAGKSTLLNCISGVYPMDQGRIILDSMDISYRPEYQRSRYIGRVFQDPMLGTAFDMTIEENLAIAYARGKSRGLKPGIRKAEAKLFQEHLARLELGLEDRMKQKVGLLSGGQRQALTLLMATIVEPRILLLDEHTAALDPAIAQRVLQLTREIVSRYHLTTIMVTHNMKAALEYGNRTIMMHEGRIILNLQGAEREEMTVEKLVQLFGLK
SGAELDNDRLLLI*
>Syntrophomonas_wolfei_subsp.metylbutyratica_JCM14075T_02906_hypothetical_protein
MVLVIIRTLILFTVTVIFLRLMGKRQIGQLQPFELVIIMISELAAIPMENTGIPLLSGLIPIFVLFTAQVTLSYLSLRSEKARGIICGKPSILIENSQIVEDELRRIRYNINDLLEQLRSKNVADIADVEYAILETSGQLSVILKSEKRPVKPEDMDLPVKYDGLPITLVIDGQVINENIKKMNYNEEWLQQKLNESGIKSIKDVLFASLDPDGTLYFQLKKGV*
>Syntrophomonas_wolfei_subsp.metylbutyratica_JCM14075T_02907_hypothetical_protein
MRLLASLLAILLVVLAAGFWTNHSLQQSTKDLSRQIERVSQEIKQEQWEAAHKQNRQLERTWQEKARWWPIILDHQEIDNIEFSLAKIKEYVASHDLPLALGQLSELKLMIEHIPRKEAVNLENIL*
>Syntrophomonas_wolfei_subsp.metylbutyratica_JCM14075T_02908_hypothetical_protein
VLGITSINRNHRGEIVNIELSDGRRISLEELFQEVEQDNVGGAYIATDANGEKHLHLSRDGDTDEDRDCLSLI*
>Syntrophomonas_wolfei_subsp.metylbutyratica_JCM14075T_02909_hypothetical_protein
LQRIFLTLISPAEYARQGVNFKFPVPDACPNPECLIPIPPKKHGFYGRNCLDVTYRGRVLIRRYYCPFCGKTVSYLPSFCLPYYQYSLAVIYMIIVNHIESAMSYASIIRNLRKHHPNLHLGLQHVEFYILRFLMNLNSIQIGLRQFLPDLALPDFGLDIKKGAKKILHIVNGGFGTIQTFSQRFFEQCKRSFLSPLHNSLA*
>Syntrophomonas_wolfei_subsp.metylbutyratica_JCM14075T_02910_hypothetical_protein
LTSFNMKIESVFKDNLDDAAPSLKFTTVWQQHQKTGKNRFSFKKMVAVPVAALVSLVVLLMVGFSFAAIKDKTDYPFVNDTSVIGLWESVDLLDQPGQFVPGKNHSEDPLFLKQLVFYKDGGMLMAVNKGNAKLSPTQYTWTKGMVLDKQAKTNSKYDIKEINGTTYMFFEWKCGDYTYFHMEPPYFVLKKVNDKDYSDYQMTAKSDKIDYPFVNDPQLLGSWKSVDFVPAIEEFKPGTK
LTPGELFLTRFDIAENGKIGGMTSQKEIPDDLLTWTKGLIIEKKGKVASKYEIKEIDGDTYLFYEWKNGNYIYSGMQPDYYVLKKVK*
>Syntrophomonas_wolfei_subsp.metylbutyratica_JCM14075T_02911_Sigma-W_factor
MKDISQEQAEAIFRDYSSAVYRLALFLSKSPILADDITQDTFLQVFKKYGSYDSRRPIYPWIYKIALNITRNTLRRQKWLVLFGEPPESGDSTTIEDRILKNELAAELWQEINKLTLKSREVIVLHYYLDLKLQETAAVLGIPLGTCKSRLNSALNTLEKQLQENSLLILKKGDDLLDII*
>Syntrophomonas_wolfei_subsp.metylbutyratica_JCM14075T_02912_HTH-type_transcriptional_regulator_MtrR
MIMTTKEKIKQAALAAFAQKGYEGATMQEIAEAVGINKASIYNHYKGKEDLFLAVYQDVARKYEKLLKRMIRDSENMEIRDKLLHMFKEYILFYYKDLHISLFWTQCLVFAPPELRENLYVDIVKREEPFEKKMLEILGEGMQQGIIRRDIPTKMLLSYRAMRDGLLLWMRIVPELSGEWIEVFWKDFWLGIQEGKGGGEQQDETRNELKLKGSPDSQPADKGALSLYTATCLICHREFGIPFTDYRY
KDIKYNRCAYHVCDDCGKKIQEECQKITGLSPESIDFWERLENRRK*
>Syntrophomonas_wolfei_subsp.metylbutyratica_JCM14075T_02913_hypothetical_protein
MGDYNYDTYCGLYCGACSIIKAYQTGIKDPLACCLGDELGMELKCHGCKTDSVFGNCAVCQIRNCARGKGVERCLNCSDFPCPNFTSMEFLLEMLPHWNTAVTNQEYIGNHGAARWLEQQATQWKCPDCHTDYSWYATHCSNCGKELSTLRPYRNSFDKRIFQLMAMPSPDEMFKKEVVFKLSGMDQVKVQKEISYNHGEGKFLLDLYFPPGAVRGQKLPLVVLVHGEAEARNMKDSG
PYTSMGRVIAASGLAAATFNHRTLLQGFQIKDVIADIENLIAFLINNADAYGIDKNRIAVWALSSGVPYGLYAGIHNHPAYVKCIVAYYGFGEFEFASLCALLNIPFNAGGWASIQAEQYSPLQFLSQNPDRIAPMFITRAGRDQIPHLLESLDGFISTALANNIHIDLYNHPNGVHAFDLFNDEPRTHEIIEKTLEFLKRHLLIL*
>Syntrophomonas_wolfei_subsp.metylbutyratica_JCM14075T_02914_CarD-like/TRCF_domain_protein
MFKVKDYVVYGLNGVCQIADIRKDNYDNSNETEYYILKPVYNTSITSIMVPVNNSNIMMRAISTKNDVLSLIAKMPDIETSSWIDNDTQRTNQYKAALRTGKTEEWVKIIKTLYQEKKARSAVGRKLTTTDEGLLNTAEKHLNEEFAIALDISPDEVVSYISKHIS*
>Syntrophomonas_wolfei_subsp.metylbutyratica_JCM14075T_02915_hypothetical_protein
VSFRISSQHQNIQVLDICVKYLYIIIILYKYMGCCLLNKIETQLVNNILGAFQCAKDTVALLPQLPPNIKPVYFRILNAIYKLNHYTGRPRISDISKESGLLLPNTTKVINEMVELNILEKFTPDFDKRVVLVRATELGEQYIQKYIVNFIESLEKQVSRINEIECIIMIDTVYKVHQAIKTVYDEQEPEKDAIN*
>Syntrophomonas_wolfei_subsp.metylbutyratica_JCM14075T_02916_Putative_glutamine_amidotransferasec
LNVLLENEPGDTGERKGEAMKAVIGISANHNAAESLYCLKEAYVVSVREGGGVPLILPALDDVALVDEYLDICDAFIFSGGGDIDPFYWGEVPETGLGEINPLRDSFELKLARRLLLQNIPVLGICRGCQLLSVASGGKLVQDLATGMSHEQNAPRYYPFHDILVYKETLLARIMDNHEARVNSFHHQAVREAGVGMRVSARAPDGTIEAIESIDKNFWLGVQWHPEFLRDEYSRRLFRTLIEAAYMT
K*
>Syntrophomonas_wolfei_subsp.metylbutyratica_JCM14075T_02917_mRNA_interferase_EndoA
MIKRGEIYFAQLNPVIGSEQGGVRPVVVVQNDIGNQYSPTTIILAITAQINKAKLPTHVELKAAVYGLERDSVILAEQIRTIDKTRLKQRIAVLNDDTMNKVDQALNISLGLTEV*
>Syntrophomonas_wolfei_subsp.metylbutyratica_JCM14075T_02918_EndoA_inhibitor
LPASRRIIITVSESLLSEVDHITLEESKNRSQIVREAIKFYLGERKRLMAIEQMKKGYLEMAEINLNIACKNSGVEEEALVNSIENLLE*
>Syntrophomonas_wolfei_subsp.metylbutyratica_JCM14075T_02919_Alanine_racemase
MIMAVVKADAYGHGMLEVSRACLQEGVEWLGVASLDEAIHLREEGIKAPVLVLGFIPEEFAEATVEYDISASVFSLSFARALSQAASKLSRTARVHIKIDSGMGRLGFAPIPSSLALIQEIAALPGIEAEGIFTHFADADAKDKSFTYEQLRAFSNFVSNLEKRGLSIPLKHCSNSAALLDIPEAHLNMVRAGIVIYGLYPSEDMKRLGLDIIPSMTLKSRISYLKVLEPGHYVSYGRTFCCQRATRVATVPIGYA
DGYHRLLSNRSTAVIRGEKAPSIGMVCMDQCMFDVSGLERVEEGDEVILFGKPSDGVTADDLARILGTINYEIVCAVSARVPRLYITR*
>Syntrophomonas_wolfei_subsp.metylbutyratica_JCM14075T_02920_putative_manganese-dependent_inorganic_pyrophosphatase
MLAKDIMTREVIAVNKNDSLEEVARILLKEKISGVPVVDADRHVIGIVTEKDLIVKASELKMPFYVTLFDSIIFLENPIRFNNNLKKFTASQVNDAMTSRVIMVEEDTEVSRIVEIMQEKQVNRVPVVRHGKLVGIISRNDILKSLVKKNG*
>Syntrophomonas_wolfei_subsp.metylbutyratica_JCM14075T_02921_Nicotinamide_nucleotide_repair_protein
MVKILTAREMKAIDSKATSEYGIPSLILMENAGIRTVEVIEDLLESSQSKKVVVLAGKGNNGGDGLVVARHLINAGIHVDVFLLAGSDEMTQDSYTNYQILSRMSRNIFPLQLEEDLDRLMLALLSCDLIVDAIYGIGFQGQMNDFDSQIVRMVNWSKALVVAVDIPSGVEADSGRVHGEAIQASHTVSFALPKIGLLLEPGKDYVGTLSVADISIPAPLLQDKNLKTGLISEAMVKSWLGPRHPESHKG
TYGHVLIIGASPGLSGAVIMAAGAALKSGAGLVTAAVPESLLGVVDAGWMEIMSSPLAESKQGTMALEALPLIEGLLGRASACAIGPGMSRFPEASAILHSLLKKAGIPILIDADGLNALAEDLNILKDHQVPVVLTPHPGEMARLTGKNIEEIQSNRVAVARKFAQQWGVTLVLKGNKTLIASAAGEIFLNITGNPGMATAGSGDVLCGLITGLMAQGLRPLDASIAGVYLHGMAGDRAAEIKGQRG
LLAGDILNSLPDILQQFER*
>Syntrophomonas_wolfei_subsp.metylbutyratica_JCM14075T_02922_Holo-[acyl-carrier-protein]_synthase
LLVGIDIIEIERVKEALTRTPRLLQRLFTEQERLYCFSKKNPYPSLAVRFAAREALRKLHPAFTAGAAFHDVEVLSLEDGRPELLLYGSAAEICREQGIGKISLSLSHSQEQAIAVVIAEKG*
>Syntrophomonas_wolfei_subsp.metylbutyratica_JCM14075T_02923_Chemotaxis_protein_CheW
MADEIQLVVFTLKRDNVVCEYGVPITKVQEIITMPVPTKLPQAPDFVEGIINLRGKIIPIIDLKKRFQMGASETTSESRSVVVVEVEGQTVGIIVDEVSEVLRLSTESIEPPPAVIGGIAADYLTGVGKLDDRLLILLDMDRILNEKEKTELVDMSEAVV*
>Syntrophomonas_wolfei_subsp.metylbutyratica_JCM14075T_02924_selenium_metabolism_protein_YedF
MRKTIDARGLTCPQPVVLTKKALEEEISEVLTIVDNSNALENVSRLAQTLKLECKVDEKEGNYYIDIWKGESSDNLSLAENSYSNTVVLISGNVLGRGDDKLGALLMKNFIYTLTQVEGAVKSLIFMNGGVLLSTEGSEVLEQIRLLEESGVEVLSCGTCLDSFQLSDKLRVGITTNMYTIVERLNGAARVITL*
>Syntrophomonas_wolfei_subsp.metylbutyratica_JCM14075T_02925_Putative_L,D-transpeptidase_YkuD
LLKSFTLYFFIFTFICIGICIGTAPLMAESTIMNPQKANSQKIPVNKIDRTDFPSLFLAEPQLEGESVWLVQARLKELGYDIQPSGIYNLPTSQIVRLFQVASGLEENGVVNRPVWEALLLSDAGEPCLQGDSQDKRGRLLIEIDVSSRKLVLTEDGKPVKEYPVAVGKSKTPTPLGEWKVVHKGLNWGNGFGTRWMGLNVPWGIFGIHGTNKPGSIGSYASHGCIRMFNRHVEELYPLVPPGTRVRIVE
NGKMFPQDLKPVLLKKNSSGQRVVYVQSRLKELGLEFDRADGRYGNMTELAVKYFQAWHGLEVTGEMDEATYRAMGMIK*

>Syntrophomonas_wolfei_subsp.metylbutyratica_JCM14075T_02926_High-affinity_zinc_uptake_system_membrane_protein_ZnuB
MELLQFDFLRHALAAGILASIACGVVGSYVVIKRIVFISGGISHTAYGGIGLGYFLGINPILGAVFFTVISALGMGVLAERTRQREDTLIGIIWAVGMALGVIFVKLSPGYTSDLMSYLFGNILAVPFQDLILISILDAIIVAVVWFYFEELKAVAFDEEFAQVAGISTRLFNLLLLLLIALTVVVMIRAVGIILVIALMSIPPAIAGQSSKSLKGMMVLAIILGIVFNSGGIFLSYFFDLPSGATIILLAAAGFIISTASRQRF
G*
>Syntrophomonas_wolfei_subsp.metylbutyratica_JCM14075T_02927_putative_zinc_transport_system_ATP-binding_protein_AdcC
MDRMDREERKKVIEIENLLVKLDGRTVLEEVNLSVYEKDFLGIIGPNGGGKTTLLKTILGLVTAEAGTIKIMGEKMKKNRGQVAYVPQYAAFDRSFPVNVWEVVLMGRLGSKRGRSYSELDQEIAREVLKRVEMYELRHRPIGELSGGEIQRVLVARALAGRPEILLLDEPTASIDSRFQAGFYELLEELNREMTLILVSHDISAISVYVKTLACLNCKLYYQHSKELSPEMIEKGYQCPVELIAHGVPHR
VLAAHDGVVGS*
>Syntrophomonas_wolfei_subsp.metylbutyratica_JCM14075T_02928_putative_zinc_transport_system_zinc-binding_lipoprotein_AdcA_precursor
MRKKLWPVLLLLGILILPGCKPASQSNPVLDAEEHTGKIKAFVSILPQAYFVECIGGDKVDVSVMIPPGANPENYEISPAQLKELSTADVYIMSGKLPSEEIWLPRLIAANKKMLVLDSSQSIDFDEHNPHIWLSPRLVKKQAANIAETLIQLDPDNKAFYSRNLEQLLLALDELDKEIEEILSGVKQKSFIVYHPAWGYFARDYGLEEIAIEEHGKEPGARELARLVEEARAKKINEVFASPQHSSRSAEVFAQ
EIGGKVVFLDPLPRNYISDMQAAAKTLADALSD*
>Syntrophomonas_wolfei_subsp.metylbutyratica_JCM14075T_02929_Peroxide-responsive_repressor_PerR
MNSRLQELSNCGFKITPQRRLILDILQKSDRPLTAEETAERIKKKEPRISVATIYRNLNLLVEIGVLSKLEIADAAARFQINQGHNHHLLCLGCGAAIKIGICPLQGRVEELIQEHGFSVDSHYFEITGYCRECQLKETGK*
>Syntrophomonas_wolfei_subsp.metylbutyratica_JCM14075T_02930_hypothetical_protein
MIANKFSSKTERQTEEPVTLKDIGGILLGCFILAAAIQGILIPAQLLTGGVTGIAIILNYLTSLELWLWYMLLNIPIFIAGYRLVSHRFAIYSLIGTFALTAFLAWLKPFNLGIEETILAAIFGGAMGGIGTGIIFHSKGSSGGLDIIAVIIKRYWGYNIGQTFFAANLLVISLFLLLSSLELALFSVIGIFVSSRMVDLVESGLNVSRTAMIISKQHEEIVYGILHNLQRGCTYLSGTGAYSSKEEKIIMTTVGKTQLPRLKE
IVFQIDSQAFLIINETIEVFGKGFKRSGREY*
>Syntrophomonas_wolfei_subsp.metylbutyratica_JCM14075T_02931_putative_cysteine_desulfurase
MKSIYLDNAATSFPKPEGVYRAVDFCQRNLGGNPGRGSSREALKAGSLLLDAREALGALFNIQDSAQIAFCSNVTEALNIGFKGILKPGDHIITTSMEHNAVARPLFQMSKEGVEWTAVKCEPDGSLDPEELRRAIKPQTRMICVLHASNLTGTMMPVKTIGQIARENGILFMLDTAQTAGVLKIDVEEFKIDILAFTGHKGLLGPQGTGGIYLRPGIDIKPLKEGGTGSFSEYLEHPDFMPDHLESG
TLNTPGIAGLLAGVNFIREIGMDDIRKHEQELTQLLIDGLKEIPGISLYGPDDINQRTAVLAFNVVGMDCGELSMALDYEYGVVTRSGLHCAPLAHASIGTIHNGACRFSPGFFNTKEEIEAVVKAVYKLARRN*
>Syntrophomonas_wolfei_subsp.metylbutyratica_JCM14075T_02932_GPR1/FUN34/yaaH_family_protein
MSGNGGWANPAPAGLVALAIACFVFYAVLSGTVDHSCIPLMGIWLLGGFVVQFTVAVIELREGATTGGNVFLFFSAFFMFVGGFEFMLKYFAAVNGWAMDAHIDGWAWLVLWLSLILWTPAYLKQSPLVMSLIVLLLDVAVFFVAFMDIGWLGHSYAPVAAIFLLASGVLAIYLAAAIILNTAFGRAVLPLPGPILK*
>Syntrophomonas_wolfei_subsp.metylbutyratica_JCM14075T_02933_3-oxoacyl-[acyl-carrier-protein]_reductase_FabG
LFPQSNELEAYTCLIAGGSRGIGAAIARAVAARGASVIINYRSSELEALRLARDLREKGAGVLAIRADLSRENEVERMFTRIEAEMGNVNALVYNAGVSQRGLLIECSEEQWDRVMNANLKGAFLCCRRALPYMIRERFGRIVNIASVLGITGASYESIYSSAKGGLIALTRSLAQEVGPSGITVNAIAPGPIFTDMLREELDEDEMADLTREIPVGRLGCPEDVAAACVFLLGREASFINGHILSLDGGWKP*
>Syntrophomonas_wolfei_subsp.metylbutyratica_JCM14075T_02934_Poly-beta-1,6-N-acetyl-D-glucosamine_synthase
LLLQLFRFAVTGLLNTLVDFGVFNLLIWLWGATDTLTISFINLLSVSLAIINSFFLNRHWTFASSGGKGRLQFQRFLLASLAGAAINTTTVFLLSSLLSQIHTTSYLYLNAAKVLGTIFSAGWNFWAYRNWVFSRDKSAQVSFSEDYVPGLTSVVIPAFNESKRLPARLQELAALLPGLFPVEIIVVDDGSNDHTGQIARSLAEKNPCIRCLGYDCNQGKGKAVQTGMLAARGEYLLYTDADHTFTPQHIE
QILQRLRSGSQVVIARRNFSAGTRLEGESPLRGLMGRGFNRLVQFLLLPGIADSQCGLKGFQREAARKLFTRQHLKGFAFDVELLALARVLNLEICEQVVEAIDCPGSSVNRILTPLQMARDVLRLKIALMTNRYDLPTFQWPWKPVLLAMALFLLALAIRLPWLWEFPRFIDELKEVQLAYLIYYGQSWPLHNAAHDIGAMHNYILAAIFKIAGPNIYWPRLYVAITSSLSVYLVYRLGKHLFGESSGLI
AALFLLTNGMHILLSHMAWANCTTPFFFTLALLVTLKSEQKKSGRLLVLAAFLWAATLQTHSSVIIYLLVVVAYLAVTFRNSAIPFRYYLAAAFSFLFGYFNMIYYNLLSYGGSFKWINNKSYALEQNLSINSYLHNMAQLLVELIRSVTSIYRNYGELWHYSLHPLFILSLFLIGTGAYFSIKEKKGLPLWLLLGGVLIMPLFNKRYVFYLATRYIMPLLICALILLAYGLVQIIRRIQSRTSQKKAVNIITATLILL
LATAQVFPYYSYCSARLNTNESNRLALEVVKLTRQMSQGHESIVLIDDKLPLENQPLPYLLTFLEHPYQMLDSKKSWDNDINRYSGISTLYPGHGQQLIMVMNEENFRNWKFLLHERINRVERLSCQISFPSPASRPRSIFVVEINDKYSQPVFQGKASLPGEEIR*
>Syntrophomonas_wolfei_subsp.metylbutyratica_JCM14075T_02935_Deoxyuridine_5'-triphosphate_nucleotidohydrolase
VTAINVKFKKLVDWAKIPEYETRGAAGCDLAVAIQEEIILYPSEIRILPTGLAMMIPPGYEAQIRSRAGMAGKGIVVANAPGTIDSDHNGEVKILLLNVTDKPIRVLPGQKVAQIVFAPVVQAIFSSDI*
>Syntrophomonas_wolfei_subsp.metylbutyratica_JCM14075T_02936_hemolysin
LACSHNSEQMPESKSRISLPFKDPVSGFTHLLGALLSIWGLVVLIRHAANYGGARYLLAAAIFGASLILLYSASAIYHLVLAPEKTTMILRRIDHMMIYVLIAGTYTPICLIALQGKWGTGLLIAIWILAFIGILLKLLWFNAPRWLSTLFYLLMGWLVIIAIFPLARVIPAIGISWLVIGGVLYTLGAIIYATKWPHISSRIFGFHEIFHLFVMGGSLSHFWLVFRYVMVL*
>Syntrophomonas_wolfei_subsp.metylbutyratica_JCM14075T_02937_Major_Facilitator_Superfamily_protein
MSQAKKAFANQLLLFAVSVFMMGLYSGLYDPSFNNYLSQVHQLGEVARGGLEFPRELPGFLVVFVFTALAFFADSRIAMLAALIVGISLWGQGFLAPNMRMVVLWMLLWSTGAHLFMVLKSSISLRLAVQGQEGRLLGQLGALEAAGALCGMLVVYLGVSQFHFSFGVIFGIAGTFALLAALCLYLLDPKPIKRPPHYLLFKKKYSLYYLLSILFGARKQVFLTFAPWVLIKVFNTQVQTFALLGLIG
TVVSLGFRPLLGRAIDAWGERLVIFLESAILVVICILYGFSLYWFSARIALVIIMICYIVDQLLFAVTMARSTYLNRIADSRDDVAPTLSMGTTLDHGVSMTIPFVGGIIWALFGFHWVFVAAAVIALLNMAASLFIPGIEEERLNNRGV*
>Syntrophomonas_wolfei_subsp.metylbutyratica_JCM14075T_02938_Coenzyme_A_disulfide_reductase
MSAASKARRIDSELKITAITDDQYVSYAGCGLPYYIGGKIPEQSQLIARRPEDFAQQNISVKTRVRARLIRPEQKYVVTENLETGQLSEESYDRLLVCTGARPHIPSLEAINLEGIFTLRTVHDSLAIKAYLNQHKPKRALIAGGGYIGLEMVENLLEYGCEVILLERSSHLLPNMDEDMALILTAYLQSRGVEVRTSENLSGFAGDFRVREAFTDKGSLPVDFVLLSMGVVPNSELAAAAGVELGIRNAIRVN
DRMESNLPGIYAAGDCATTKHLVSGQEVYIPMGTTANKQGKVAGENAAGGNARFAGVLGTGIVRAMEMELSRTGLCENECRQLGIDFISRRVKSRTAAHYCPVSGEIHVKILVEEPNGRILGAQIVGFAGAAKRIDMLATAITMRATVESLIDMDLAYSPPFSPVWDPVLVALNQF*
>Syntrophomonas_wolfei_subsp.metylbutyratica_JCM14075T_02939_Fatty_acid-binding_protein
MPGIRIYADNACDLDKEYLEELGVKLFYMPVTIKEQTYRDRLTISPPDFYKLISEPGVLPTTAQITPQEFKEEFEKALQVSNDQIIYIAFSSGLSGTYQSACVARDMLDSQRIMVFDSKSASVGYALTVIRAARAVAAGENLEQVLAAIEDNIRRIEHIFIVGNFDMLKRGGRISSTSATLGNLLNIKLIANFVDGRILPLEKVHGLKKAKKRMVEIMAERGVNLKEQLIGISHSNDYQGALELKELIEERFACRE
FVISEIGAAIGAHVGAGSYSVFFLRQG*
>Syntrophomonas_wolfei_subsp.metylbutyratica_JCM14075T_02940_hypothetical_protein
VLMWEQDLIRFSFCARASLEFSILALNLYTCLTQDKIYMSARSPLRLKVEDMGLISYFHLLDKEGVRIDNPLTICL*
>Syntrophomonas_wolfei_subsp.metylbutyratica_JCM14075T_02941_D-inositol_3-phosphate_glycosyltransferase
MKVALLSPIAWRTPPRHYGPWERVVSLIAEGLVKKGIDVTLFATGDSKTAGHLDYVCPIPYEENPNMDPKVWEAMHIAHLFEQAHKFDIIHNNYDFLPLSYSRLVDTPVVTTIHGFSSPKILPIYREYNRDNYYVSISNADRSPDLDYFATVYHGIDLNELTLNLDGDSYLLYFGRIHPDKGTAQAIEIARKSDMQLLIAGIIQDKQYFNREIYPRIDNKNIIYLGSVGPARRDELLGNALALLHPIHFNEPF
GLSVIEAMACGTPVIAFNKGSMPEVIKDGETGFLVDNVEMACEKLRSINKIKRANCRYWVEENFSQERMVSNYLSLYKQIVKGLSIRTPSLSSRWK*
>Syntrophomonas_wolfei_subsp.metylbutyratica_JCM14075T_02942_DEAD-box_ATP-dependent_RNA_helicase_CshA
LSFENFYSMGLKTDLLQMIDEKGFEKPTPIQIKSIPVAMAGLDLMGQAQTGTGKTASFGIPILNQIIKGEGLQALVLCPTRELAVQVAEEISSLSRRMRIQVLAIYGGQSIELQLRSLRRNPEIIAGTPGRLMDHMNRGTISLSPLKYVVLDEADEMLDMGFIPDIQKILSQCPQERQTFLFSATLPDEVRELGTKFMKQPEIILIESPERTVPEIEQYYYQVNSRRKIETLCRIIDAQQPPISLIFCRTKRNADE
LARVLTSRGYNADALHGDMSQRERDHVMHGFRQGNTKILVATDLAARGLDIELVTHVFNFDIPEDLDSYIHRVGRTGRAGRSGIAITLVEPTQIRLLRMIERHTGKRIERAILPTLAEAVEKRQDLLLERVRQASSEPGDICLSLAEKLMQQGDPKKMLAAALKLLIDEEPDFEMADLPESDDNMAHLELPAGRLQGMHPRQLLEFIAAHTSLSPQRVGDIEINRNSTFVEVPMDYIHEVYEAFEKYR
RTQKNKTRPARFSKENRKIMVNHGGR*
>Syntrophomonas_wolfei_subsp.metylbutyratica_JCM14075T_02943_Spore_coat_protein
MNKHMRDKEMVNDVLSMTKGGLATYAHCISETANQQLRQTLQQIRNSDEQFQYQLYQMAEQKGFYAPAAQVDTQDVQQVKSQLRS*
>Syntrophomonas_wolfei_subsp.metylbutyratica_JCM14075T_02944_hypothetical_protein
MNLNHQELQNLRHLIGSHQVTERKLNFYADECQDPQCKQMFSQSAKSASQTIQKLMSFLG*
>Syntrophomonas_wolfei_subsp.metylbutyratica_JCM14075T_02945_Cyclic_di-GMP_phosphodiesterase_response_regulator_RpfG
MFIRADISGLPLGRRLAQDLYAFDGHLLVAKGIVLQESHLQQLRQRGYEYVYIQEEDEVNENSLNQGIDYLIDKQLPKVFSISVESMRHMMARVSSGHMIKAVEVEECIDLVYDHVLRTYNVFKYLQNLRNKDEYTLQHSVSVGILSIKIGQSLGLDTDKLRNLGIAGLLHDIGKSMISPDIINKPGPLDENEFKEIQKHPVYGYRIVNGIKLQDYDIAQAVLQHHEHQDGNGYPLGVQGDKINDFARI
IAVADVFDALTSDRAYRPRMPLLQAVDIIIKSSCGQLDPLASRRLLSYVLDITTGEKVILSTGEEATVILKNEVEPARPLVRIGDNFLDLKTNRDVFIKDFAR*
>Syntrophomonas_wolfei_subsp.metylbutyratica_JCM14075T_02946_TVP38/TMEM64_family_inner_membrane_protein_YdjZ
MRLPGKQNLLHLALLLVFLLLFCYCTAVYAPGISRIISNPEQFRDYILGFGSPNILVFILFQVVQVIIAAIPGELLQIAGGFVYGTWWGSIYSLAGIFLGSIVVFFISRLLGYPLVCYMVSPQKLERFSRILRQQKSEISIFILFLIPGLPKDILTYVGGLTPLNPIRFLLIAIIARFPGILGSAYLGASVEQQAYGQALLFSLAAFLLFLAGFLLKDRLFKPY*
>Syntrophomonas_wolfei_subsp.metylbutyratica_JCM14075T_02947_Aerobic_C4-dicarboxylate_transport_protein
VQLKMKDKRDFRLEIASIESASDYVRDILKQYKCTDRDMVRAQLFTEETMVYWTETANEGDILQINVRKRFKTISLSLSYRGAQSNPLAISDEEEDETEFSFIGQNILIGLSTVTYTSYENGYNVITFTLKEKGTNPVILIIFALAAAIICGLIVNRFAPSLGPGLSASILIPLSKAFFGLLNAIVIPFLFLSVIASIFNMENIAQMKRIFRILFSWFLGLTAMSAVIAVLAGMIYFPLQNGASTGVNEVKIWTQIAKMLF
DIIPSNIFQSFLDGNTLQTIFLAVITGITMLVYKGRFPVISKVVSESNLIFSTLLDAICSLMPWVIFICIFDMLLSGDGRVLLSSIAVVVLICICFLLIVLLCLLSVALIEKQNPLQYIKTIGPVLLIALSTASSSATFAPHSMIASNKQGIRDYLANFSIPVGALFAKPFVVPVLLLMTLFVGNFYSLTFTAADVVSMVLLCVILSVAVPPVQGMGIFLFTIIFKRFTIPLEGLAMAASLFMLFDYLMTTGNVFSINISMLH
TEQRLREKDKRVIVYNNQKC*
>Syntrophomonas_wolfei_subsp.metylbutyratica_JCM14075T_02948_hypothetical_protein
MAYWLLCQSCKEWSKFTTPLSNDKVCPFCSNSFIMVKSHLSSIPYERTIEKAQDLPENPATVENEPAPSIEEKESLELQETEIAEITETAEMPKMKIENTAEIEEMPETSTAPDKPETEDKAELQENAEMPDQPEMTEISEIPEDTAIQETSLEEDQTNEISELQETEKVLTGNEKQNILRQKKKRSKYYR*
>Syntrophomonas_wolfei_subsp.metylbutyratica_JCM14075T_02949_Multidrug_export_protein_MepA
MDRSARMRNQHIGKLLWNFSLPTIVGMLVSSLYNVIARIFVGRGIGSLAIAATTVAFPIMILLMAVSLLIGVGATALISLKLGEQKKDEAEKVAGTAMTMLILLPAALALLFLLFAEPILISFGASAAVLPYARDFTQIIMLGSVFGSISMGMNNFIRAEGNPAMAMYTQILGAVVSVALNYVFIFIFHWGIKGSALATILAQLISAIWVLSYFFTGRSLVKIHWKNLKPAWPVLSSIMAIGFAPFAMQTAACIQQ
LILNHTLMFYGGDLALAAIGIVMSISMLLVMPVLGLGQGAQPIIGYNYGARQYQRVRETWKTAVLAGTGIAVAGYLALHIWPLQLVGLFSKGDTALTTMTVHAMLTFFALLPVVGFQVVSSMYFQAVGKARQAAILSLSRQFLIFIPLLLILPRFWGIDGVWRTAPIADGLSVALTAGFILFEMKKIRQEEAQEPRGGGGETTPSGTFENLA*
>Syntrophomonas_wolfei_subsp.metylbutyratica_JCM14075T_02950_Glutamine_transport_ATP-binding_protein_GlnQ
MYILQAIDIHKNFGSLGVLHGVTLQVQKGEVIAIIGPSGSGKSTLLRCLNHLERIDSGRIEIEGETIAQNNSEGQAVYSSEAKITQARRKMGMVFQNFNLFPHKSVMENLIMAPTRVKNMDKAEAEESARLLLAKVGLSQKADNYPFELSGGQQQRVAIARALAMNPDIMCFDEPTSALDPELTGEVLMVMKDLALEDMTMVVVTHEIGFAREVADRVIFMDEGVIMEEGQAEEVLLNPRQERTSA
FLSKVLMAR*
>Syntrophomonas_wolfei_subsp.metylbutyratica_JCM14075T_02951_Arginine_transport_system_permease_protein_ArtQ
MDYIIMLLPSMLKGLTVTLKLFSITLVMALPLGVIMGMARISSFSPLRWFMEFYVWLFRGTPLLLQLLFMYFGLMTLGLRPEREMAAYLAFVLNYSAYLAEIFRAGIQSIDRGQYEAADVLGLSRYQTMKNIILPQVFKRVLPPIGNEVINLVKDTALVYYVLAISELSRVARTHVMRDDTFIPFLIAAIFYLMMTGMVQRFFKWLELRYDYFH*
>Syntrophomonas_wolfei_subsp.metylbutyratica_JCM14075T_02952_Sulfate_starvation-induced_protein_7
LKRKVAWLILLCFSLGMLLMSGCAAKTPAPESSGDDSWTKIKDKGYFVMGLDDAFPPMGFRDEANEIVGFDIDLAKEVARRLGVEVQFQSIVWDTKKEELNSGNIDVVWNGFTINEERKKDFLFSKPYISDRQIIVVRADSDIAGKADLAGKKIGIQAASSANEAVEADKETFEVIKDNLLQFESNDLALRDLKGGGVEAVVVDEVVGRYYLSKHPGDYKVLKDNFGLEDFGVGFRLDDKAFHAEV
EKALDEMKADGTAAKISEKWFGEDIINK*
>Syntrophomonas_wolfei_subsp.metylbutyratica_JCM14075T_02953_Endo-1,4-beta-xylanase_A_precursor
LRLTGLKDLRMVVGVGWLFVLSLLFTVFPVSWGEAMDIQNSIFQVQASEQLSLAASEELDTGGWVAEIIPEITNKGDISLTFDKVMADPSGTKEQFTVVVNGNTAVIANVVKTNTPEKIKLELAAKASGAQNLIIDYVKSSDPAKQLKSIDGGTVESFSIQIGEPSQAPSLIADSSDNLRGNVIDITFVDDSLWRDKVSNVKVNDSSIRGKYRIEEGRLSIDADVFLTSGDYSIVVKAPGYTDATVVQTILE
AVTQIDPPVLTADLTNNTIGQAIDITFSDDIAWRTAISDVSIDGQSANAKYSINEGKITINAEVFNEARSYIIVVKAAGYNDIQLEQKIMQPSANGGGGSGDGNNSGGGSGDGSGGGAGSGTPGGLTVLSFNQAKLAPSDTLLQFDFGNGMDKTLSSNLNKIRVYEKASGTEVKHSNYNYIKQGSGNDAVKLRRLELMFNNLKPGTAYVVELEAGIEANNGSTLGSKQSFEFSTSGTTAGGGAAVPA
DEKSYGQEAATINEQGARIDIPAAAFDQDFKVKVRKVADVSQLPLAEKSKLISDVVEIEKDKSGEFKKAVSITLSFDKTKVDSNKYDIRICYLDEKANKWLPLSNIKVDLATGQVSGQTKHFTKFAVIAAEKATVPEQKAIENSKVVKLSDIQGHWAQKAIEELLASAAIGGYPDGSFKPEQNITRAEFTSILVKALKLNVKDGKVFDDTAQHWAKEAIAAAAAGGIVSGCSESIFAPDESITREQMAVMVV
NAGQLAGLGDNKIFADADQVSAWARKAVAVASAHSIISGYPDNSFRPRANASRAEAAMVIAKSLKLAG*
>Syntrophomonas_wolfei_subsp.metylbutyratica_JCM14075T_02954_tRNA_modification_GTPase_MnmE
MQDTPKANRLHIAIFGRRNAGKSSLINALTRQEIALVSEVPGTTTDPVFKAMEILPVGPCMIIDTAGIDDEGYLGELRIKKSREVLNQTDLALLVIDPALGISEFEQELKQEIQSRKIALIGVLNKIDQGGVDQKSLEKQLGLSLVAVSSRSGEGIELLKERIIERAPAHFEEAHIVGDLIEPGDICLLVVPLDMAAPKGRLILPQVQTIRDILDKNATAMVVKEDKLREALEGLAKKPRLLITDSQVFGKVAPQ
TPDDVMLTSFSILFARFKGDLLELVRGAKAIEKLRPGDEVLIAEACTHHAQDDDIGRVQIPRWLNRYVGGELNFHWYSGSGYPENLEQFKLVVHCGACMINRRQMLHRIDMARDSGVPIVNYGVLLAFVNGILERALQPFPQARAVL*
>Syntrophomonas_wolfei_subsp.metylbutyratica_JCM14075T_02955_Aldehyde_dehydrogenase
MTMTPNEYRSVSTEVIKKQQAFFQSGAGRELAFRMQQLRQLERAIDKCENELLRALELDLGKCRQEAYMTEVGLLRAELAYIMKHLPVWVQARKVRTPCLYPGARSLIYPEPLGQVLIISPWNYPVYLALAPLLGAMAAGNCTVIKPSELAPHSSAVLQALIQDSFPEEYIAVLEGGAEMSSELLEQPFDHIFFTGGSAVGKLVMQAAARRLTPVTLELGGKSPCLVDQDINIEYTARRIIWGKFLNAG
QTCVAPDYLLLQREIKEPLLKKMGEVLLKFYGANPRHSPDYARIISAGHFQRLEALLADGRILYGGEAVREERYIAPTIMDRIASDSPLLQEEIFGPLLPVLEFGDLEEAIQFVNERPKPLALYFFSRNRKRQEQVLKQSSSGGVCINDTLSHISSPYLPFGGVGESGMGSYHGQASFELFSHKKSVLQQSLSFDIPLKYPPYRIPLQALKKLLRILQA*
>Syntrophomonas_wolfei_subsp.metylbutyratica_JCM14075T_02956_50S_ribosomal_protein_L13
LKTYVAKPAEVDRKWYVIDASDKALGRLSSEVASILRGKHKPIFTPHVDTGDFVIVINAAKIKLTGDKLNQKKFRHHTGYPGGLREMDYRTLLQKRPEKAIEAAVQGMLPHNRLGRSMIKKLKVYSGSEHPHQAQKPELRELKG*
>Syntrophomonas_wolfei_subsp.metylbutyratica_JCM14075T_02957_hypothetical_protein
MAKVQYWGTGRRKEAVARVRLVPGEGNIIINNRSFEDFFPNQTSRLIVQQPLVLSAMQGRFDVICRVNGGGVSGQAGAIQLGIARALLKANPDIKAMLRRSGFLTRDPRMKERKKYGLHKARKAPQYSKR*
>Syntrophomonas_wolfei_subsp.metylbutyratica_JCM14075T_02958_MORN_repeat
VLSLKAQPHGQGKLYYANLNLWYEGEWKEGKREGLGTSYYEDGKPAFAGEWKGGKPLK*
>Syntrophomonas_wolfei_subsp.metylbutyratica_JCM14075T_02959_N-acetylmuramoyl-L-alanine_amidase_LytC_precursor
MYIRKPYIFVAKKTSILLPMFLFIILSYGVVANEYQPAEPVLSYSLANQLIVIDPGHGGFDPGAWRGELMEKNITLSISKKLQQHLSQAGAIVVMLREEDKDLAGEQFKGSLKERKRQDLKARVDEANRLKADLYISIHTNADPSPRWFGAQTFYNARSEESKIMAECVQDELTRILGNTKRKAKPGSYYIIDKTKMPAVIVEVGFLSHPTEAKLLNDPAYQNKVAYAVFSGVVNYQASQMEPADKSDKK
IVTGSDIAK*
>Syntrophomonas_wolfei_subsp.metylbutyratica_JCM14075T_02960_protocatechuate_4,5-dioxygenase_subunit_beta
MIGYAALSPHPPLIIPVIGGSSLEDVKATVSGMKEMARELVDSKPDSLVFLTPHGNVFADCLSALSEEELQGDFRNFGSKLGTSRRNDKVLLDEIETRARKAGISFITLSAETLRLHHLNPRLDHGILVPLHYLEEAGLPQIPILAISVGFLSPLELYTFGVVIRDAALAQGKKLAVVASGDMSHRLKNEGPYDYHPDGAVFDQQVQQILRAGDSEALIKMPEKLRNNAGECGYASLLIALGSLDGSIYESKI
FSYEGPFGVGYLCAGLRPKASTPSRLEKLVKERKESINTSRNEESTPVRWARMILESYIREGKRAKLPPEMEELRQQRAGAFVSLKKNGQLRGCIGTIAPSYGDLADEIAGNAISAGTADPRFLPVDENELDDLVYSVDILGDAEACRIEDLDPHQYGVIVSSGRKRGLLLPDLEGVDSVEEQLSIALQKAGISPGERYEIERFEVKRYY*
>Syntrophomonas_wolfei_subsp.metylbutyratica_JCM14075T_02961_Pyruvate_formate-lyase_1-activating_enzyme
VQGVRCQKDRPLDTGQWKRVIVMPEVEASFYEKMEEQRVKCWLCPHHCMLKEGQSGLCRVRVNKEGTLFSLNYAELASLALDPIEKKPLYHFFPGSMILSAGTYGCNLFCPYCQNYSLAHEKPSTREISPLLLTEIARQSIDDGSIGFAFTYNEPTIWYEYVRDAAYSLKEAGLKSVLVTNGYIERAPLEELLPLVDAMNIDVKAFNDDFYRKHCKGRLEAVKATVERAVEMTHVEITTLLIPGENDE
PEEIKALASWLAGLNPQIPLHLSRYHPAFKFSREATPAATLERAREIAREDLDFVYIGNVLQEENNTFCQTCGELLIERNIYQVENRGLREGKCKACGSSIMVPGT*
>Syntrophomonas_wolfei_subsp.metylbutyratica_JCM14075T_02962_S-adenosyl-L-methionine-binding_protein
MEEIVLRPVGQVLSSTEEPEEMPLGGLEAVIEIFPEYQEALKGIEENSHIWVLSWFHKAPRDVLRVKPVRINPELPEYGVFALRTYTRPNTIGLSLARLERVEGCRLYLKGLDAIGGTPVIDIKPYFENDTIFSPLTSYIKGKDREMRQGILSKRALVHHQEECSYLHLAVRMAIIAEDYLGKLNSPDLFVRVRGPLCLVDCVQGISRARLANPPRFSFQPAEAEIESEWKRGNSTLLLRLKPGAELEGLESKED
QELFHIEYWE*
>Syntrophomonas_wolfei_subsp.metylbutyratica_JCM14075T_02963_N-acetyl-gamma-glutamyl-phosphate_reductase
LFRVGIVGDGYTAADLLRLLAHHEQAEAVRIFSTENIGRPIDEVYPHLIGFSELICEESNWDLLKEECDLVFLALPHGLSVSIAMELLPAGVKCVDLGADFRLKDAGLYEEYYELTHPAPELLEEAVYGLPEIYREEIKQASLIANPGCFPTGAILPLVPLLEAGVLEPSNLVIDSKSGVSGAGRSPKASSHFCEVNEGVKAYGVGTHRHGPEIAQELSRAAGKTVEMVFTPHLIPMNRGILTTIYARLRTTAD
PGLVRLVLEEKYRNENFVRVLPAGVMPHTKWVYGSNLLDIGIYADEKSRHVILVSALDNLVKGASGQAIQNMNLLLGLEEDSGLKLAGIHP*
>Syntrophomonas_wolfei_subsp.metylbutyratica_JCM14075T_02964_Arginine_biosynthesis_bifunctional_protein_ArgJ
MKIINGSVCAPRGFLAQGAEAGIKKIDKKEVAIIYSTEPCTAAGVFTSNKVRAACIDYNKACLADGTARAIVANSGNANACTGEQGIKDSQRMADLTGELLGIEGSDVLVGSTGVIGVFMPMEKVEKGIRKAVEALSYDGDHNAAQAIMTTDLASKELAIEIEIKGNPVRIGGIAKGSGMIHPNMATMLAFITTDAAIDSKCLQKMVKTAADRSFNMISVDRDTSTNDMLVVLANALAGNPLIDEVDS
EDYLLLQEALDYLCVSLAKKIARDGEGATRLIEVQVVNAADYESARQIARSITASNLTKAAVFGEDANWGRILAAAGYSGAEFDPGRVDIYLGQEKMAENGMGLLFDEERARQELKQDPVLIKVDLKSGKQQATAWGCDLSYDYVRINAAYRT*
>Syntrophomonas_wolfei_subsp.metylbutyratica_JCM14075T_02965_Acetylglutamate_kinase
MYVSAMEKAEILVEALPYIKDFYGRRVVIKYGGAAMTDCELKQKVMQDIVLMKFVGMHPIVVHGGGPEINQMLSRLGIKSEFVNGFRVTDAATMEIVEMVLGGKVNKEIVAGIHASGGKAVGISGKDGALIQARPADGSGRMGFVGEVQCVNPELIEKVIENGYIPVIAPIGLGEEQQSYNINADLVAAAIAVSMKADKLVLLTDVPGLMMNPADSNSLISVLKVSEVENYVKEGIIAGGMVPKVQC
CVEAVTGGVGRTHIIDGRVPHSILLEVFTDEGIGTMVVNE*
>Syntrophomonas_wolfei_subsp.metylbutyratica_JCM14075T_02966_Acetylornithine_aminotransferase
MSSQDIMDKGQEYVMNTYGRFPIAPVKGKGCYLWDANGKQYLDFVGGIAVCLLGHCPDILNQTLREQSEQLWHVSNLYWIKPQVELAEKLVQASGMGKAFFCNSGAEANEAAIKLARKYFYRQKEGKRNTIIVFKNSFHGRTLATLTATGQPKYQEGFAPLPPGFVYAEFNQLDTVEALLDDSICAIMLEPIQGEGGIQPADLSFLRGLREICDREGILLIFDEVQCGMGRSGKLLAYQAYGVQP
DIVTLAKGLGGGFPIGAMLASEKAAGGFAPGDHASTFGGNPLATALACKVVDTVASQEFLAQVEKIAEYLYEALLSISDPRITAVRGRGLMLGIEFNTEVRELVEICMEKGLLLLGAGPKVLRFVPPLVVSEMEINQAMGILKGALMEWSA*
>Syntrophomonas_wolfei_subsp.metylbutyratica_JCM14075T_02967_Ornithine_carbamoyltransferase
MLKLKDNLRGRDFISIHDFSREEIAYILEVGAELKKMQKSAIAHPYLAGKTLAMIFQKASTRTRVAFEVGMYQLGGHALFLSPRDIQLGRGETIKDTAQVLTRMVDGIMIRTFGHEEVLELAQWSTVPVINGLTDLLHPTQVIGDLMTIKENKGTLQGLKCAFIGDGNNVAHSLLFGGAKVGMDVAIASPGGFEPEEAIMEMAREDAQESGARLQVLSDPVEAITDADVVYTDVWASMGQEEEAD
DKEQHFLQYQVNRKLLSLARPDAIVLHCLPAKRGKEITDEVMDGPQSRVFEEAENRLHAHKAIMALLM*
>Syntrophomonas_wolfei_subsp.metylbutyratica_JCM14075T_02968_Beta-1,4-mannooligosaccharide_phosphorylase
LSNLNTESLFDEVLREIAPELEQYSNYDLLIGIPFHNEVEALPLILQSVDQVLQSWIGRRQLIVCVGDSSGQAVIDSIKKLELKHPHIEFLLPREISGRGMSIRAIIEISARLDADLLVFSANMATERGPGIDGAWLESLLTPIQGHYDLVLGSLRRHLGIDSIAHMLAAPVLEAFYGSRVGDPLGGTYAISHDFCEELAHEARFWGENIRGYGVDFWLLTRALCWGKGICEVNMGGVIKPHSLETRNRIFHDN
LGTIFEALRRDSAIWLQDRLVIKVADILARSEVKQPDFESYSFPDLWQSFQEGCHKYRSLLHSCDHEKEIEKQLQLPPEKFHILSDELWVSALFSLLLAYTFDEQADRQEILAALTTLYNGRVASYVLEMHSFSEQLGFLTTREKDAILVGKMDNIRQCLSNRFWLLKPELSRQWLHRREQVKPAIIPLGYMEYVPGKPLVVPKKILGKDQCLINTDCIFRELRKRYENSFNAFLEQGLAVDVNAPPPTIVA
AIKDFMEQLEQSLEELFPGDLYNEQGLEKFVEALFAVFPQHSMFTVGEDLLREMLVRFPPVNVMIPLACYKPADLLKKMEARDALTYANLVEGRMYSDRTLLWLLDNLKPESFHWVGIKPIILKEELHPGELAQIRLSYLNRVTGRVTIKYLEEGKGGKYPRLRYFTSIIRRLAVAENFSQLFQVIVRERKNLGLKVGYSLLKLQKGDELSALNIFENQHHRKLVEKLRYLAKKLSEQGNEEKSRIFTLM
ADSYGLSQVLEDETFLTCTAWSWASYSFKGGIKIPTPMTTSVENRWFNHDFLELLYQELGYDPKEITSIIFRLIQANKGGQNLLDTLLPARPRDVAVVVQEITNEPSKYLKRYEGNPILEPIEEHDWESKYVLNPGALRIKDKVYLFYRAVGSDGISHIGLAISDGYKVLERLPHPIFSPSIAEEKRGCEDPRLIIIEDRIIMLYTAYDGNIAQIAAASIKVRDFLAGKYENWKRDGLAFKNIWDKDAILFPEKIN
DKYVIYHRIEPSIWATYMKEISFPLKENHAIIIGPRPGRMWDSLKIGAGAQPLKTKYGWLLIYHGVDYNYVYRLGVILVDLNDPAKVIYRSPNPVLEPEEDYEIGFSGAWVPNVVFSCGAVAAADKEVLEDNDEILVYYGAADTSIGMARATLADLVPEVFRVEVHT*
>Syntrophomonas_wolfei_subsp.metylbutyratica_JCM14075T_02969_Sporulation_kinase_E
MASKTQNPEKSSKNTNSEIFFYTDDESRETPGDSLCFMLRALKHSSQAFLAVNQNAFFKAVNQALCNTLGYSRQELRLLNEREDLKAIITAIIAEYESSKPGKKKNKLKIAGKNGEALVLKYVVNRSSDGRYYYIFVDDISEQEQAGIRLRESEERYRLIAENAFDLITLIDPRTHCYRYISPSHQRMLGYSPEELIGKNCFEHMHPDDRVRVSKKMEAGLRAGHGRAQYRSAKKDGSYIWLEAIGRIIWNQ
HGEEELLLVTRDISERKKAEEALKASEERYRYIVDSAADGITVIDCSSFRAKYINPALEKILGYSSKEYRHVNLFERIHPEDRDSIERLVREGKKSGYNSAEFRFRKKDGNYIWLDTNGKMLYRDGQAEWLCISRDISNRKQAEEELRYSEERFRMITDSSFLMIDISCPQTKKYLYVNSSNPKILGFTEEEMLGRNIFDWIHPEDYEKALHTFQDIIENGEGSCECRHQRKDGSYIWIESQGKLIRNPAGKLEVL
FFSRDVSELRKARDKLQCQLEEQQELINNLNELFYIYGPDFRLRFVNRKTIECLGYSREEMLGKSIFDFVPAEDLELVKAHVQESIVMGEVKTCEHQALCQDGRIILLRVKGSLIIKEGNLEGVQILAEDITEQRRMEKEMLRLAQLQTVGEMAAGIGHEVRNPMTTVKGFLQLMSQNTELTRYQAYFDTMLEELDRANSIISEFLSLAKNKMVDLHNLNLNAIIRQLYPLLLADAMVSDKYILLELGKI
PNLLLDDKEMRQLIINLVRNGLEAMQAGQSLEISTRLEAEEVILSIKDQGKGIDKSYWEKLGTPFFTTKDSGTGLGLAICYSIARRHNASIRVDSSNRGTTFEVCFKVELLEGILELQF*
>Syntrophomonas_wolfei_subsp.metylbutyratica_JCM14075T_02970_hypothetical_protein
MPDFRHMQEMSRENGILQFSHLDTADPASGFTLDDNARALILAMEMPGGREYAACYTSFLYQSQQKDESWSNFMLDGKYSSLFNSDDSVGRALLACSLGSSSPWPEISQKCQEMLLKNLSRAFSFQSPRAIAYALIGLCKCQWSGLGTRQQDSIKKLGEFLINIYQVQHSRKWLWFEDYLTYCNGILPQAMFALYQLNADKKALKIGYDSLNFLNSILFRQGYLNIIGNDGWYFHNQKIALFDQQP
VDAASTAFACIEAYESIGEIEFLELAILAHKWYRGHNIHGLSLYNAETGGCYDALTPEGVNLNQGAEAVLSLLLSDKLMENFIGKKVDIDQSS*
>Syntrophomonas_wolfei_subsp.metylbutyratica_JCM14075T_02971_2-deoxystreptamine_glucosyltransferase
MILLNLGTYPPKQCGIATFSMDLRNSLLINGNEVKVMAVSDNSYQYHYTDEVLFNLKQNHKQSYIRAANYLNKAPLELIIIQHEYGIYGGSEGQYIMELVRLLHKPFVLITHTVLPRPSKRQKQVLNYLCSRASAIVCMTRRSEHLLSDLYEAPPELIQVIPHGVPEFKEQTQDSLKEKYGLQGRDLISTFGLIGPGKGLELGIQAMAQIVKEHPPTTYLILGQTHPMLKKQEGEKYRHMLEDMVVNLG
LEHNVVFVNKFLSDEELGEYLYMSDIYLSPYPNKDQAVSGTMAFAIGCGRAIVSTSYAYACEFLSGGRGLLAAEADPEELAGLMKKILTNPVLKQSLQDNALKLGKSWSWPSVGQQYTQLFEQLLGETPLTKEPRISYARL*
>Syntrophomonas_wolfei_subsp.metylbutyratica_JCM14075T_02972_Phosphomannomutase/phosphoglucomutase
MLDIARAFATHAIIKGHSKILLGRDNRLSSPQLRELALEAILDSGCDVIDLGMVVTPVFYFASRHLDIKAGIMITASHNPGEYNGCKLLLGDHTIFGEEIQNIRQRIERRDFFTGPRGKLETIDIIPDYIEMIKSKIKMGPNIAHLVVDCGNGTASTLAPQVFRSLGCEVSELYCDSDPSFPHHHPDPVNPKNMVDLVAEVKKTGADLGIGIDGDGDRLGVVDARGNMIWGDMLMILFWRDILPRYPGC
ECIVEVKCSQALIDEIERLGGKPLIYKTGHSLIKSKMKEINAVFTGEMSGHMFFADNYYGFDDALYAGARLLELLSHSEKNISEMLSDVPRYYSTPEIRIPVGDEEKFFIVDKVREHFKLKYPIIEIDGARILFPQGWGLVRASNTGPELIVRCEGRSPEALEQIKAELFGCLQSLGMEEEPGPAEFPFTPVYNYPKSEFQDQV*
>Syntrophomonas_wolfei_subsp.metylbutyratica_JCM14075T_02973_Alginate_biosynthesis_protein_AlgA
MIAVVLAGGKGLRLWPESRQTRPKQLCKFVDGKTMLDHTIDRLMTAGSSRVIITNDSLLPGINELVQQRGDKEKIEILSEPEGRNTAPAVGLVLTRYFGEGEDEILGFFPADHHVSDTEAFVASVQRACMAAQNGHLATIGISPSRPETGYGYIEKTKWEIGEIPGVFSVNSFCEKPDPETANSYLASGSHMWNAGIYISQAKTLCDEFKTHLPEIYEKLILGFDKYIDSYPLLPEISLDYGIAEKSDSMAV
VPANFGWCDLGNWNALAEIYLPDLKSNICNGNDVLLIDSNNCIIKQNEKTIVLFGVDNLLLVESEDLVLVADRNRAQDIREIVEFLQEKKRFDLL*
>Syntrophomonas_wolfei_subsp.metylbutyratica_JCM14075T_02974_hypothetical_protein
LLYQKHRCRVVPIAVFTYPEKRNEPDRFKLVFPFMSVLDFHFLTLELNKHKWQDYIQSNNPVAAALLSKMGYQKKERVQLKLEFLRMLVRMELDPARMTLLTGFFETYLILNEEEERKLEEEMGKVDEKEAKRMIEITTSWEEKGKMKGRMEGKAEGKAEGQASILLRLLKKKFGYMPVEMENRVMSMPVEKLQELAEAIFDLETIDDVNSFISK*
>Syntrophomonas_wolfei_subsp.metylbutyratica_JCM14075T_02975_putative_nucleotidyltransferases
MSGKRSAVNFSEFRAMRKHLIKLDKLDERRYYQQALQKAKDVAAMLKEHYGVKQVYLYGSLAWGGFDRHSDIDLYLVGFQGNYWRAYSEAEDIVAPIEISLACEEDCIISLKDKVLEKGVLL*
>Syntrophomonas_wolfei_subsp.metylbutyratica_JCM14075T_02976_lysozyme
VDELLNPPSASAREGRAISIIKTGLSFGRLLGRRSTRRIIMHHSASSDVAAATIHGWHLKRGWSGIGYHFVIRQNGDIEEGRPLDMIGAHAGSLGNGDSIGICLTGNFMEVKPSEQQLYSLVELVQYLRELYQDNLEIMRHKDVVASECPGDFFPWPEDNWRLNKPSCNVREACNSMELWKNELVNEALRKNLISERHNPDEAASKWFVLALGLNILKEIDRKLT*
>Syntrophomonas_wolfei_subsp.metylbutyratica_JCM14075T_02977_putative_nucleic_acid-binding_protein,_contains_PIN_domain
MEIESFLHNNKCLALDTNVFIALFAQEVLGVKVVPLIDAAANQGKLELIASVMAFAECAVKPYQEGNWMALDQIKLMFQMPHLTVYPVDDQVAEEAARIRAVYNFKMPDAIHLATAIIQRADVFLTNDYHLATIKEIPVIKLEDL*
>Syntrophomonas_wolfei_subsp.metylbutyratica_JCM14075T_02978_transcriptional_regulator,_AbrB_family
MFGVVEEVKVGVKSQIVIPARIREDAQVRPGDILIMSSDRNGRISIMKKPENWASAAYGCCKDAWGDKPLEYLQKERETAWK*
>Syntrophomonas_wolfei_subsp.metylbutyratica_JCM14075T_02979_hypothetical_protein
MVNGKVEQVHDQVIGLRPVYTDRGNATELWLASGEILLDRRGVKSVVTALARSYALDLKAQRKQLGQLLNRSMILPFYLGQRVFVPLKMRKALAGNDMVYGYFDGEYIEDIQGGKFRECRVLLKTGDELECFSSQTTAQKSLHLGLNLQKALEGNDDDDRDEQQVMEAVRALYRSFREILLRLQSIEGYIAEAESIYQHEKGP*
>Syntrophomonas_wolfei_subsp.metylbutyratica_JCM14075T_02980_hypothetical_protein
VLMGSGPDRILGAVDLYEQGYAPRIVMVDNWQPGYELLESRGLELPRDAELAASVGVQLGVPEEAFIILPGNARSTQDEALIITQYLKEQQAGVDKVLLVSSKFHSKRSAKLFRWALGGLNREVKVLSCPTPYDDFNAAAWWQSREDAKRVVSEYTKLANFYLVDRWR*
>Syntrophomonas_wolfei_subsp.metylbutyratica_JCM14075T_02981_hypothetical_protein
MGFTPEETYGYIVQVILETESLLLNPVLGKVYTEEAGEYAGFMRLVVRKFRFYIETVENDAVVVAVKYPGEK*
>Syntrophomonas_wolfei_subsp.metylbutyratica_JCM14075T_02982_hypothetical_protein
MAINSINPAQLSYTEFLEDIQLELNNNPKQTIIDLTQRVVQEQQAAPWSTAFREAWQDSNHHTVILESLLQLCNNQDSTQALGAILLLSAIDKTPSSILDIG*
>Syntrophomonas_wolfei_subsp.metylbutyratica_JCM14075T_02983_recombination_protein_F
LKILELEVSGYRSLKKVNWKPGDLNILIGPNGSGKSNLLRLLQIISISARGELSSYIQNAGGSFINC*
>Syntrophomonas_wolfei_subsp.metylbutyratica_JCM14075T_02984_putative_transposase
LKIQNPHDRFFKETFGKVEVAKDFLNNYLPENIIKVIDVDTLEPQKDSFINEELQEGFSDLLFKASINKREGYIYFLFEHKSYSSKDIALQLLRYMVEIWETKIKKEKSDKLPVIIPLVVYHGKDRWKIGTTLGEIITGYEELPEDIKKFTPNYEYLLYDLSRYTDDEIKGEAIDRIAMTIMRDIFTEDNKGLLDSVFRAVSYLLELDDKQTGIEYFETLMRYIFSARADLTRADFNAVVKKIDTTHPEGSETVMT
LAEIFKEEGREAGMKAGEAKALARTAIKLLTKKFGLLPEEFKTGILKLDSPTLDVMIDGILDYKSLEEVKRYLQ*
>Syntrophomonas_wolfei_subsp.metylbutyratica_JCM14075T_02985_hypothetical_protein
MINGNKAVLLNDRFIKRVKGKAIKMLKHLYMRDLDRIKNPKNQEWCNCQVKMNKK*
>Syntrophomonas_wolfei_subsp.metylbutyratica_JCM14075T_02986_hypothetical_protein
MDKLSFKGIITSIQPRIRLTRSFDEASHTYLGYAIKIAGTIDNQETISSNQNIFSIGIGKAAQAKFNFKVNDIISGECLPVPDPDMEPVDYYKVSKLAKVEEGEQGSISAPWELVPPELEVYRERGHRRLAARTYDTKCYSCM*
>Syntrophomonas_wolfei_subsp.metylbutyratica_JCM14075T_02987_glycerol-3-phosphate_dehydrogenase
MWPLIPINREDICGTFLGIRPLVIDKHDETDATSMSRDYKIDVIRKEGTRLIHVYGGKLTTCLSMAEKVAARL*
>Syntrophomonas_wolfei_subsp.metylbutyratica_JCM14075T_02988_hypothetical_protein
VADCKVISVINWKGGVGKTTLTHHLGTGFYHLSDEERIKYLGTTELPRILLIDNDAQCSLSVSCLGEKEYEDLFFIKKMGTISDLYLPFLQMEKPNINVASYTY*
>Syntrophomonas_wolfei_subsp.metylbutyratica_JCM14075T_02989_hypothetical_protein
LLALLAVFFRYAGVGIVTVDFPEKADLIIVLMGSGPDRILGAVDLYEQGYAPCIMMVENWQPGYELLESRDVDLPRDAELAASVGIQLGVPEEAFIILPGNARSTQDEALIITQYLKEQQPEVGKVLLVSSKFHSKRSAKLFRWALGDLDREVKVLSCPTTYDDFNAAAWWSSREDAKRVVSEYVKLANFYLVDRWR*
>Syntrophomonas_wolfei_subsp.metylbutyratica_JCM14075T_02990_hypothetical_protein
MPVLDFRFLILELKKHNWRNYIRSNNPAAAALLSKMGYSAKERVQVKLEFLHMLVRMELDPARNTLLTGFFETYLKLNEEEEKQLEAEIGKIDTKEAEKMMEITTSWERKGRVEGRVEGQADLLIRLLKKKFTNVPTEIENQVKALPAEKLQQLAEAIFDLETFEDVKAFL*
>Syntrophomonas_wolfei_subsp.metylbutyratica_JCM14075T_02991_Modulator_of_FtsH_protease_HflK
MDLVLGLFVNFILVIFVIMLAFSSIKIIKQATVGIVERLGKYHKSAEQGINVIIPFIDRFRAIVDLREQVVDFPPQPVITKDNVTMMIDTVVYYQVTDAFKYTYEIARPILAIENLTATTLRNIVGDLELDETLTSRDLVNTKLRTILDEATDKWGIKVNRVELKNILPPQDIQTAMEKQMRAEREKREAILRAEGQKTAAILEAEGQKQAAILNAEATREAAIKEAEGMRQAQILRAEGEAQAILNVQKAVA
DSLVMIKEAGADNKVLAIKSLEALKEIGNGQSTKLIIPSDLAGLGGVFAALKEAVESKE*
>Syntrophomonas_wolfei_subsp.metylbutyratica_JCM14075T_02992_putative_5-formyltetrahydrofolate_cyclo-ligase
MEDRWLVRQSLRKQMLEARSAMAASEAGELSRRIGHRLEELEPLRRAKNIMGFASVKNEVDLRPILEQQRGQGKRIFLPRVESAAEMVAVEFRGWEQTAGGPFGIREPLGPAVPAAEIDAILVPGLVFDARGYRLGYGKGYYDRFLAGMEKRVFLCGVCYEFQIIDDSFPDSLDIPVHWIVTEKSELAINWEFF*
>Syntrophomonas_wolfei_subsp.metylbutyratica_JCM14075T_02993_Prolipoprotein_diacylglyceryl_transferase
LYPVLIKIGPVAVYSWGFMLAVAVIVAIIGARALMRKEGWDEEMVLDLVLVMVLAGLLGARLLYVFIYERELFLKPLLIFLPGPNGFAGLVWYGALFGGFLGLVIYVWKKRLPFWKLADIFSPFAALGYAIVRIGCFLAGCCYGKVCTSVPGVVFPLVDHLPRHPTQLYSSGINMLIFLFLIWFYPRKKFPGQVFLFYLLLYSLYRFILEFFRANLVMYGPFSTSQTYSLILFALAAVLYYLQFRKHRKVKEE*
>Syntrophomonas_wolfei_subsp.metylbutyratica_JCM14075T_02994_t(6)A37_threonylcarbamoyladenosine_biosynthesis_protein
VRESLILGIETSCDETAAAIVRNGGEILSNIVNSQIDIHQQFGGVVPEVASRKHIENIAGVVHRAFSEAQLAYSAIDAVAVTNRPGLVGALLVGVSFAKAFAYALEKPLIAVNHLHGHIYANFLEHKDIEFPAICLVVSGGHSSLLLMSNQNKMEVLGETRDDAAGEAFDKVARFLGLGYPGGPAIQEAATKGKAGQLQLPRVFLDRNDFEFSFSGLKTAAMNQWNKLQRRGQANVYDMAAEFQAALV
EVLVEKSIKAAARYQVRTIMMAGGVAANQELRNLMLKKTNEAGLKLFYPSLNLCTDNAAMIAANAHYHYGNRSFAPLSLNAYPSLLSL*
>Syntrophomonas_wolfei_subsp.metylbutyratica_JCM14075T_02995_Protease_synthase_and_sporulation_negative_regulatory_protein_PAI_1
MLNTRFLIRKMTIKDIDRVMIIENKSFTLPWSRDSYLKEFDNEYASYLVCDVRGEVAAYAGIWVVFEEAHITNVAVDRRFRQQGMGRALMERLEQEARNKKAASIMLEVRPSNLAALTMYRKLGYIQTGLRREYYSDNLEDALLMTKYLF*
>Syntrophomonas_wolfei_subsp.metylbutyratica_JCM14075T_02996_t(6)A37_threonylcarbamoyladenosine_biosynthesis_protein
LLILAIDSATPVAGVALLDGNKLLKEEFSNYKKTHSQTLMPMIDRVLRECECSIDDLAAVAISAGPGSFTGLRIGMATAKGLCLASGKPLVTVATLDALAYNVLRSQDLVCPLLDARKQEVYTCFYDVSAALPRRLVEMSACSPVEFVEQALHLAHKFGREKILLLGDAYYPYEEYFQKTLGEKLLVAAPHLMYPRAASIASLAILKLEAGEWDNLFSLRPHYIRLSEAEYRLGKGAQ*
>Syntrophomonas_wolfei_subsp.metylbutyratica_JCM14075T_02997_ADP-binding_protein
LHISVKSDDEMRKLGYNLARVLEKGDIVYLRGVLGAGKTTLVRGISRGLGFNGRVNSPSFTLLNIYPATIEIYHFDLYRLGNCDLNDLGWEEYLEGDGISLIEWPEAGEGQFPREAMFIDIKLCDDDYERERVVEICAQGEKYQHKLEELKAFVDTGY*
>Syntrophomonas_wolfei_subsp.metylbutyratica_JCM14075T_02998_uracil-DNA_glycosylase,_family_4
MSSEQPSLFELLPGEELYRQEKQVAAKEIHSPFLPGIKVEEAFPGIKEAAPLEELARNCNNCRLRSGCRQVVFSDGDVKSRIMFIGEGPGKDEDEQGRPFVGKAGQLLDKILVAAEIPRSQVYISNVVKCRPPGNRLPNPDEVRECRNYLEAQIRIMQPAIIVCLGAMASQVIIDPKASVSKIRGKWFNRQGVKIMATYHPAALLRNPSYKRPAWQDFQMIRDEYNKVK*
>Syntrophomonas_wolfei_subsp.metylbutyratica_JCM14075T_02999_Putative_sporulation-specific_glycosylase_YdhD
LRFDKYILFVMLLAFTYLPAFPTLTMASSPAIRINGELRSFSPGAQIVNNRTMVPIRFVMEDEALKGEVFWDASLHKVAMNCSGKYIEMFIGSNEAWVDGEKRFLDSPPYISSDRTFIPLRFLSENLGAMVGWDAKNSEVLINFKYQPRVFAYYYRSFTEYQENIDLFSDVAFRWFETNGRGEVFYEYQDRFAEALDLARSKGVKTHASVVLMGKDALHQLLSNPENRARLIANLLDVVKKDHYDGVNI
DFELLDAGDAGNLTLFLQELKTSLGTDKELSVAVFARTGKEKWPVGYEYKKIGQIADSVVVMAYDYSYSTTAPGPVAPFWWVRDVCSYMRSVIPQDKILLGLPTYGYDWGAGKNAVTITAPKLEALEAKYGLQKDFNYQSMSPRYSYYDEEGKYHEIWLENEASLREKLNLVQENRLGGVSFWRIGNGFTDLYRLLEGWRGK*
>Syntrophomonas_wolfei_subsp.metylbutyratica_JCM14075T_03000_hypothetical_protein
VNKGDLFTVYLNGVMMTVCVLGFYSEEYSGEDMVILAVVNQDNMVYLPLGDLQALFPRLKQLH*
>Syntrophomonas_wolfei_subsp.metylbutyratica_JCM14075T_03001_D-hydantoinase/dihydropyrimidinase
MDEAGSIENGVILVEGEFIKSVGENEGLPSDCQVIDAKGKYILPGFIDAHSHIGLEEIYQSEGDDVNESSDPITPYLQAADGINFHDLAFSDALRGGVTRSLSMPGSANIIGGQAVFLKHLAGSPGEMIYKNPWGLKAALGENPKRVYGEQKKTPLTRMANASLLREALYVASKEMTKVERKAREEFKQGALLRVLKKEMPLLLHAHRADDILTALRIRDEFDLNLIIQHGTEAFLIADELRKRNVAVFL
GPLLVNRAKVEMKEVSFKTASALAQAGVNFAFITDHPVIPVEHLRVCAALAVREGLDEDMALQAITTSPARLLGVEDELGSVRQGKRADLVIFDGHPFDFRSRVVLAMVDGKIWGNRRE*
>Syntrophomonas_wolfei_subsp.metylbutyratica_JCM14075T_03002_putative_NTPase_(NACHT_family)

MRLCFGTFARVLQLCKLDVITDPRLVGTMTRTIDPQCQYIMSGNATSVHRLLNCTGNLSPGNITEGGVGVIRRPGKSISNVLLMAPRANRDDLIRKFGEDVISLLNEDKKLQAVLALLDIIEKDTVLDDDSSEGKKMSFEKYVGTTKNALLSQSAYVLSDLLAGIFLYTVAAGVVNTVGREAVQTITPEYIGGIENPRGITVIDCSPQLEQIVQSGTMVAATASPAFGQETFLTYLTNVRNKYSTIKTLL
YNEQPRPFYDFYVCNNITWRTRIEGDGPPFKREFVSDANIKKLAEISHFIFITGAGGLGKSMMMRHLLLNAIDNYKDFKLVPVFIPLKEFDDASSKLFDYIFSKIDDFGGGITKTDFENILTSGSCLLLLLDGLDEIGMNNIEYFERELDSFTDKYPNCIYVISSRPYQSFVSFERFSVLRLLPFNLEQAVRLIDNLDFRPDDPSIKEKFREELRARLYRTHRDFTENPLLLTIMLLTFEMFAEVPSQMHIFYREAF
NALSRTHDASKGAYKRELKTKLSVDAFADVFAEICFRTYWDEKFEFSMPEFEAYFKKLTNPYVGPVTADDFMYDLCFSMCLMYQESGKYYFTHRSFQEYFSAVFLSKQKDTVISKLGDFFEKRDRRMRGDQTFLLIRKLMAPEGGLFYRIK*
>Syntrophomonas_wolfei_subsp.metylbutyratica_JCM14075T_03003_hypothetical_protein
MKNRKAKQENRITYTYWRWDEESKKNLPITITAGQDSVTEEHIIMLNELDHAADLGDRYEQVNRDFTTENKKSKYESDPDDCFGDPIENLGTRKTDPAFFLEEKSDELNPLVEHLLILMEKLTPQQIDLIYDLFGSRRQLTEIAKEEGTSVTAIHNRKSKIIARLRKLFAEQGIL*
>Syntrophomonas_wolfei_subsp.metylbutyratica_JCM14075T_03004_hypothetical_protein
MNLQHKVQINVARKDGSQKKAFIKSGISKIPQRLLNFLFGEFTEILVLTPGQSVQSVEIHEIGKGGTAHEPNQTAS*
>Syntrophomonas_wolfei_subsp.metylbutyratica_JCM14075T_03005_hypothetical_protein
MSRIKLLLDVVNDMESLTKSLRTLANAIASDEPSIESEEKPSTQETQEVKPVAKTISVEDGRAVLTPISQSGKTAQVKALLFKHGANRLSDIDPSEYESLLADAEVLANG*
>Syntrophomonas_wolfei_subsp.metylbutyratica_JCM14075T_03006_PD-(D/E)XK_nuclease_superfamily_protein
MGRHAVLSASSSHRWTKCTPAARLELEFDDNESSAAAEGTAAHALAEHKLRKALKMRSKKPISPFDCDEMDEYTDAYVNFVLEQLELAKQTCSDPLVLIEQHLDFSKYVPDGFGTGDCILISDKALHIIDLKYGMGILVNAEHNSQMMLYSLGALEIYDSLYDIDEVSMTVFQPRRDNVSTWTIPVDELRDWAENEIRLKAELAFKGEGEYCPGDWCTFCRAAVKCRARAEEKLKLAQSEFRLPPLL
SDAEIEDVLGKLNDITKWANDLLAYATDAAVNHVKEWSGYKVVAGRSVRKFKDEDAVAKVAKVNGYKDIYRQSLITLTEFEKLMGKAKFNEVLGDLVYKPPGKPTLVPISDKRPAINVSSATNDFNEIMED*
>Syntrophomonas_wolfei_subsp.metylbutyratica_JCM14075T_03007_hypothetical_protein
MANKNTSKTKVITGVNTRLSYFHGWEPVSINGGTEKYSVSVLIPKSDKETIKAIEAAVDAAIEEGIAKFGGKKPNKAAIKLPLRDGDIERDDEAYKGHYFVNANSTTAPQIVDKAVKPILDRDEVYSGCYGKVSLNFYAFNSNGNKGVACGLGNIQKIKDGEPLGNRSSASDDFTSLEDDDFLA*
>Syntrophomonas_wolfei_subsp.metylbutyratica_JCM14075T_03008_Recombinase
MTISSPRQNVTRGGWGRVFRPHPISLERKWRVFGCLKHQERYTGNILLQKTYVENHITQKSKINRGELPMYYAENTHPAIIDLETFEKVQAEIARRREMGVFANWSINTTCFTSKIKCGNCGVSYRRSGKRQQTDSNQVYYIWTCQTKDRNSVSACNAKNVPEKMLQKVCAQVLGLGEFDEGLLLEQIDKIVVNGQDKLIFHFYDGRVITQHWESTARKDWWTPQARAAKSVYSKKNPRSSGTIT
CFTGKIYCGKCGQNLRRNTSTRVSGEKTHHWRCPPRNDCAHNGLEENLLKTISADVLGINEFDEAVFVDKVDRITVVSNEQLIFHLKDGSDVAHQWQFKRRQPAWSEERKQRHSKKMLEVWRDKHEQRENS*
>Syntrophomonas_wolfei_subsp.metylbutyratica_JCM14075T_03009_Transposon_resolvase
MSSARTAKKVTAIPATISRFTAAPINEQRKRRTAGYARVSTDSEEQFTSYEAQVDYYTNYIKSRDDWEFVEVYTDEGITATNTKKREGFKRMVEDALAGKIDLIVTKSVSRFARNTVDSLTTVRQLKEKGIEIYFEKENIWTLDSKGELLITIMSSLAQEESRSISENVTWGQRKRFSDGKVTVPFGHFLGYDRGEDGNLVLNEEEAVIVRRIFVLFLEGYSPYKIAKTLTAEGILSPGKKSKWNAATVRRML
ENEKYKGDALLQKSYTVDFLTKKKKLNEGEIPQYYVKNNHEAIIDPAVFDIVQIELENRSPGPNRRSGVSIFSSRIKCGECGSWYGSKVWHSTSKYRRTIYQCNHKYDDDNHCRTPHLDEERIKKLFISAVNKLLSGRDEILGNFELIKSTVFDSTDLEKEQAELQNEIEVVAGMIQQAIGENAHLALDQVEYQTRYNALVERFELAKARHTAVTEEITDKQTRLSTMNAFLYTLRKQDNLLTEFDEKL
WCSLVDYATVYDKGDVRFTFKDGTVIN*
>Syntrophomonas_wolfei_subsp.metylbutyratica_JCM14075T_03010_SWIM/SEC-C_metal-binding_motif_protein,_family
LFCNLCMYFELLKEKRYKSSWDKLQDCIDLAKMVGSYIEIENRLEISKVLHLLSSYETLYPYSVFASSEYIISKSHCSICGQSMLSLSCQHIKGKLYWGEVAVEMIDEIKTFQAVALVSHPEDKRCVIELSDDNRTEVKKFKRIDQFLELNQPFLQDFSIKKIIENRMRDDIKIVGRNDPCSCGTGVKFKKCCGKDLYYKHERNIITPLQTVSMIKL*
>Syntrophomonas_wolfei_subsp.metylbutyratica_JCM14075T_03011_hypothetical_protein
MLNDLQSSGIPVLRVSLSHHPDKFSEGSQELNITKKVNFIFGRNGTGKTTISDQIMSQLSTEYDVCLFKDFDGVVENDRLNAVALGTENAKIQKEIDIVDNDIAEIRKQIEQPEDVNIENLLTKATKSKKAYDTQYNKINAFFVDSARKLKNLSNPQIAKTSYDKPSFKDEIAKASLLSEDSIVAHKNTIKADKKADVPNILFPDIDLSLYLESTNEVLQSSVAQTQDIPELKGKTDKQNFARQGMIIHQHK
IGEICAFCGNEISDERWQLLGNYFNDEVKKLESRIASGITKIELELDAIKGIEEIKEVVFYDRFKQDIRKLNLQIVAKRNEYKEFLSSLQAALEEKKKKLFIESTPLEIAIPANFNYIEKECEDLVKGHNELSLNLKTEQEKAKDALRYHEINKMLDEFQYDDESISLAKLSAINDEAQKVLGDKKNELQIKQDARKDLILQTKDEEKIAEKINKLLLSMGVVSFSLKLVKDDDENQKGQYQIKSHDENIRPITKLS
KGEKNIIAFLYFIFSLESVDSRSKPKVIVLDDPMTSNDDTMQYLMISEIQKLYRGLRDGNYLIILTHNCHFYLNVRPNTGRTYKENGEEISFYEKYGVYHLFSDGKRTTIKSISKGKQDFKTSYETLWKELVFLHNTEDASPDLMLSPCRKICETYMNFTKKGIEAFYGDNISAKKLFDVNQHSIDDFEAEANGKTKCEILAILQKLFTDNDAVDHINSYWRGGET*
>Syntrophomonas_wolfei_subsp.metylbutyratica_JCM14075T_03012_hypothetical_protein
MNIRQGYVFSFEDAINLQPRSRLEVILATLDFYDIITVLDKENKQHRGPTGYPFESKLNALIAMRVYNMATFTELVERLTHDPVLRYNCGFDVFGKVPSIATFSRFYERLTQSEVLRELFKK*
>Syntrophomonas_wolfei_subsp.metylbutyratica_JCM14075T_03013_Transposase
MGLIDTSSIAIDASKVDANEKSVPRKNIKDDGQSANWGSKLDTNGNQITWFGYKLHIATDVKSELPVALSITPASTNDGIMAESILEECSNNLNSKPQYYLMDAGYDQKSIYELIRKDYKAQAIIPLNHRGAKEPPEGLDWDATPICSAGYRMAYWGGSNGVNKFRCPHVMGKCDCPFGSSWCSDSNYGMVVKTRARQDSRLFTVPHRGTSNWKLLYNKRTSVERCFGRLKEHLGLETGLNVRGI
KKVKTHAYLSVITMIASVIAINKDKSSTDIAA*
>Syntrophomonas_wolfei_subsp.metylbutyratica_JCM14075T_03014_Serine/threonine-protein_kinase_PrkC
MKNNGDIILFLKQKDYIMVNNDLGGGSFGKTVLLQDPFIDELFVAKKYEPEYEDIKERFYKNFLDEIKILYKLNHRNIVRIYNYYAYENIFTGYILMEYVNGKNIGEFITDYLGFIKDVSLDDVFSQLIDGFCYIEEQGIIHRDIREGNILIDKIGTVKIIDFGIGKIFEKKTDDSDSLVAEINRSGSDTLPQEYFDGIYTSKTDMFYLAELFNRLLMEAHEQDLLDFSYQDILYKMMEKQPQNRYETFADIKEAI
GKHDFVNMVISQEDKDIYQAFTNLVFQALISFFDEQKFNYDIDIFVARLEKALRDNLFENIIQKNADIIGSIVFGAYRCSSNVVIPCSVVNDFLNWFTASTPQSQKLILTNIISKLSTIAVVEKEPEIPF*
>Syntrophomonas_wolfei_subsp.metylbutyratica_JCM14075T_03015_hypothetical_protein
MDRIQVLIQIGKLGGKDIHYSEVADEIIKSDEYVLKAYEILEKDGYIHRGGLSTGLITEATLTVSGKNFLRNK*
>Syntrophomonas_wolfei_subsp.metylbutyratica_JCM14075T_03016_Type_I_restriction-modification_system_methyltransferase_subunit
MITGEIRNKIDKIWTDMWAGGITNPLTVIEQLTYLMFIRSLDEKELEMERVEALSGEEMPKIFPQDKAGQDMRWSKFKTKDSRAIYDIVGSKVFPFIKAMNGKNTSAFSRYMQDAMFLISTPQVLQKIITGLDELYEHDIKDLDMQGDVYEYMLGKLSTAGQNGQFRTPKQIRDMMVRLLDPTPEDKICDIITAKTIQFNYPILMAS*
>Syntrophomonas_wolfei_subsp.metylbutyratica_JCM14075T_03017_hypothetical_protein
MKKDDLIITESTNPLNKYFVEKEVFEQTKEKLNTTDILAVPIRYDDGEYYYAQETISFLKYCRQTDPEHSADVLSDGDIKLRSLHSFDIWMPVIWIGSSIVLPLVINLVSNYIWEKMRGREQEDAKVNITIMVQQGKNTKELHYNGDARTFSETFEKIDITKL*
>Syntrophomonas_wolfei_subsp.metylbutyratica_JCM14075T_03018_putative_secretion_ATPase,_PEP-CTERM_locus_subfamily
LLGYGQNELWERLKLQSYAAIRQRIDLQCKLPYLDRAEVGEYIKTHLAYSGADHDIFSDNAIDEIFKFSSGAARMVNKVCTHCLIYGAQNNRNIIDDHMVKFVVQGELS*
>Syntrophomonas_wolfei_subsp.metylbutyratica_JCM14075T_03019_butyryl-CoA:acetate_CoA-transferase
MELKIALNYLKDYDRKFTFADEAVKCIKEGSIVRYAFGVCHTNELDFALARRRDKLNNVYILYDPDAYQYAAMISNSKKNISTLYHINGRCGNKKIFHLSIVY*
>Syntrophomonas_wolfei_subsp.metylbutyratica_JCM14075T_03020_Arginine_utilization_regulatory_protein_RocR
LIPDLLVSDLLDTSAMTAIIVCNNGTVVFINKTYLDVLGKKKEEVVGKNVGDITPESRTLTVLRTGKAIVGYNWSVNGYNMIACALPIIKDGKVVACFAYSLFMDIWDAKDLVNNLMSELNMYRDEVRNLYSARHGFSEIIGQARNIQDMKFMAQKAALHPSITVLVTGESGTGKELFAHAIHKASSRSRMPFIRVNCAAIPENLLEAELFGYEEGAFTGSRKGGNPGKFELANGGTIFLDEIGEMSTS
MQSKLLVFLQEKEFERLGSNHPIRVNVRIIAATNRNLEEMITQQKFREDLYYRLNVLTLDIPPLRNRIEDIPLLVNYLITKLNIDLKTNVVSMSDEALDLLSKYNWPGNIRELVNALQRAMLLADMGNYNIINAKHLYFLKINLDNVNLSLSSTKTLKMQLRCYEKQIILSKVLEETNYNKRKTASILDVDLSSLYKKIKQYGLQPDDA*
>Syntrophomonas_wolfei_subsp.metylbutyratica_JCM14075T_03021_hypothetical_protein
MQAEYATDIVFKKQADLGLLYETLVRTAVHSVKPEDIASFLGQKLHWNYQGEMGNNFNTRIRSTRIKHQMGAVSIPALPGVYLYL*
>Syntrophomonas_wolfei_subsp.metylbutyratica_JCM14075T_03022_hypothetical_protein
MIKFKSCSDDGFPEFRKIVFVGMRNFFDQAVKQKALQDPGDGSRAQVGDKFADHLVLKTLDVKLSPGQDFKKLTVGMAEGIEAFVASLILHDGIGDFGKLLNTVAGIVKGRDKLQVAPVLRPHPSGA*
>Syntrophomonas_wolfei_subsp.metylbutyratica_JCM14075T_03023_putative_enoyl-CoA_hydratase_echA8
MELKTILYEKDAGVGIITLNRPKALNALNDELINELTILMDDIGSDNEVQAVIITGGERAFAAGGDIAFMANADSLQAEKFVEAIRVAFDKIDRLDKPVIAAISGLALGGGSELALACDIRIAAEGSSIGQPEINLGIIPGAGGTQRLTRTVGPGWAKYMVMTGLPISADTALKIGFITKVVPKEQLMDEAKKIANIMAVKSPLAMKAAKRCVNYGLSVDLSSALSYEIKTWAALFSTEDQKEGMKAFLEKR
NPVYKSR*
>Syntrophomonas_wolfei_subsp.metylbutyratica_JCM14075T_03026_hypothetical_protein
MEDIIRLYKDRIRIFLIIYFFSEEFHDSTNLNLVRVLYTETRIQKIDFLLRNPDYFALELLNLLPTKKHDRNQIKNVIIDIFKNREPEIRRLEMEKFFFGAYEDIDDVIAFLESYGFIVFKSKRNIELNVIDKQYYVTKYAKEKTDKVLSEMDSLKWYIDRCEQIKQYLGDYTGTQLKNMQYEEETYRNTSYKEFIANVQEKVKTIFKREFGENL*
>Syntrophomonas_wolfei_subsp.metylbutyratica_JCM14075T_03027_ATPase_involved_in_DNA_repair
MKKLISELKKELEIRDLVIGDTELNEILERNEYVAELPIATGKRILLEELRFTGTKNNGDKIDYIQKFASGINIIIADNLTGKSSVFKVVKFALTGRDSLKSDISSWIKTIMLGFSIAAKSYTICIDKTSSRMSSTLYSTAIDQVNIEYDDNVIFQSNTKSDYEVQIQDFFFNQFSYYPLSWTQKDSAKDSIELRETKASWSTYFKSIYLESKDTNSFYGNQGSKTFQILLGLKYTKTINKLMIKRDFIKNSLAKKDGGD
NISLVKKEDLLNELKKIEVGLDAIRTNSDYVKFQSLKELQNSLFMDIQASSMIFSSSNNRHYQLLAELDNSRSARDSLTKEKDGLEKEVTKITRKINDLKEYLESGYFFSNLTIKMCPSCNHEIDMSPTNEEHECNLCHKPVVSEIENKEQFSERLKQLTETKIILEIKINEIEEKISTLQKSISGLEKEKLDIEKTIQNNDYAELSQKSAQINKQLNELNDKLKQSTERENELISQKAILNYRLSLQEVDSDDNVG
KLNKKLEVLEISIDYFINKRFEESKPVLTSLEDLMLNEIHAFGLPSITKIHIDKLFNIEYTQNGTRIKFEDIAEGEQLRVKLAFYLSIIQLDIQKNYGKHPRLLMIDSPAKEEGDNKYLEGLKAVLADIENRYADKLQILIGTAERNLSDIVRSQFVYEADQYVF*
>Syntrophomonas_wolfei_subsp.metylbutyratica_JCM14075T_03028_hypothetical_protein
MCFDIAILDTYKTKNQAEKLNFWEGQLPLCNFEKQNSRQLVSFIHQIIADSQEAIYSKCQAIDFSIRMFLADILKDRNILDILLDSWEPTSDIHLETLRIKRLALLYPYDTDIQSVLNELAQSDSLVSTESNYQLGLINLFVAREKRSIDEVVEVLRIAYVHFDVASKEENRIDASLFKVITNSIIYIFCNNWLEFDKCIKELERLIFIQRIYSWNPDVVTLHRAIYSKLYNAKLIIKKDTKNWLNYKAEFDQLCMEFF
NIQNQSVKSDLFSMEIEHGISDNLITKVIEPVFKTNFHSVLCKIDCLLNDDSIEENQAEFLIYLKDVIQQQSETEISEYDFFNQSIIRTFPHLPESKSRIFYDAIKDSNLQIISNILDQLTNSNGYDKLFERIMYACVTLQGTRHYYGSEENIRNKFIAQLLEASGFRTKDQTQWGLSNKGISDGEIDVQIVDDKGTAYSIIEGLILKSLDTTYLNLHLDKIFKYDTTGLEYIFIVSYVAVADFSSLFTAIQKSQQCDV
LEPHCA*
>Syntrophomonas_wolfei_subsp.metylbutyratica_JCM14075T_03029_hypothetical_protein
MILRNVQLVRKGHILADNIGIKRRRRFNINIDDMSIQGVFYIKQYLEKLQLEDQFCRLFAPEDINQLLYIYGRVYRINYSEALINVFEIVLNNAIYSVLSGNSEVKLRISQEQFEHLQQKFMGLDHSHTIALIEDAIEILIKQLGIDQPRPQDYIRKFKSVFLSRFINGLTYNCLENVILIDKEQTQNFDIVFDEGNRLDDESFRLVVDDIMDCTFTKRKTDIVRSSIHSLGDFIDILEADCFFGDEFYDLFNSLGDM
ELSVLARIVFIEEIRSNLMSFSLASPGEKAEAMEWQVEFCNFLQSLSADRIESIETYIKSSLQMTDAWDFLG*
>Syntrophomonas_wolfei_subsp.metylbutyratica_JCM14075T_03030_putative_ABC_transporter_ATP-binding_protein_YheS
MKIAMGLAIMGQYDLLILDEPTNHLDVHSREALEESLRQFPGSILLITHDRYFLEQICDGMLVFEQQRIMRIEGNSAKKQFAETRADSVKTSNDEETLVLETRISWVLGQLNRYKPGDPKYTALDKEYSELIQRKNALSKQL*
>Syntrophomonas_wolfei_subsp.metylbutyratica_JCM14075T_03031_Regulatory_protein_BlaR1
MASTGTKVGAYGFIAGWHFYCYRRFTMQLRADSLPVADDTAAVILSSCKAALGVHGDVKLMQNHKITGPMLVGLLRPMILLPASSISGMDLNLVLAHELMHLKRKDLWVKMLALLAGTLHWFNPLAHILCKDVSIWGELSGDEALVYEMSHEERKLYGEAILNTLDNHSSINTALIQITASQCIS*
>Syntrophomonas_wolfei_subsp.metylbutyratica_JCM14075T_03032_Competence_protein_ComM
MEDKKVTISRVKYTNSYPANFMLVAAMNPCQCGYYGTDKCRCTDYEVLKYRQKISGPISDRIDIQKYVHPVDFMNLSNFTAKTSSSELRERVEAARIIQNKRFEKYENIISRAGFTFMSQTHLLRGRILLLSATDPIYSL*
>Syntrophomonas_wolfei_subsp.metylbutyratica_JCM14075T_03033_(R)-stereoselective_amidase
MICYDCRFPELARAAALEGAELLVIPAAFNTTTGPAHWQLLMRCRAVDNQLFVAAASPARNLSASYQAWGHSLVVDPWGDILQEAGSGEEIIHARLDFSRLEQVRQELPLLRQRRKDLYRLDYKAPGNGIV*
>Syntrophomonas_wolfei_subsp.metylbutyratica_JCM14075T_03034_HutP
MALSAYRQEETRLKKELLENWEVKAVAVDYGGEFISSVGKIVERAVVAASPFA*
>Syntrophomonas_wolfei_subsp.metylbutyratica_JCM14075T_03035_Tyrosine_recombinase_XerD
MDISYWRELNDGFTDFLVEKGYTHDITRHYRGTINLLIRFANANGYKAYTQEIGEKFLKSEERLMYLQQAGYQRQRLVIRRFNEYLDDGKYSYTYLRVNYECPEAFEEVLEHYLKSLETANLKQCTIDKHRVFTIKLCRDFARNGIESWDTVDAKALAGAFDRATSKAQFAACIRKLFGFLVKEGIVKYNYAGIVPQMHYWKRIPSVYSSDEIEAILGSVDRSNEVGKRNYAILLLAARLGMRSSDISFLRF
ENIDFVKAIIEFKQRKTGVANQLTLLPEIAEALQDYINNARGDSVEPYIFLTCRESLRKGVLPQLSSGAICRVAAKHFRQSGIEFGDRHHSAHALRMSLASGLVAENVPYEAVRTILGHEDPNAITHYVKFDTESLRSCALDVPMASGRFAEYLASGKGAL*
>Syntrophomonas_wolfei_subsp.metylbutyratica_JCM14075T_03036_Tyrosine_recombinase_XerC
MPKQYGSCLAAEMNDYLKLLENADKDTSKYRTTFNSLDAYLLNSNISQKALTEAVVSGWLKTLACKPKSKRNYAGNLRRFARYLCALEIPAYEPELFRTQSDYVAYTFSDEEFAAIIDAADNFAAIKSKENETSYVFPMLLRILYGCGLRLGEALSLRWRDIDLDTEIITIRKAKNHKQRIVPISSSLAEVLRLYNRRMIGDDSDASLLFESVRIKGRPYLQNTFRLWFLQILEQAGIPNVRSVPSERIISPHTLR
HYFTFKSFLKSEAARRPLEESGPRLAAYLGHESLIGTEKYITTNYVLYKNSHARMEESIGNLFPEVNFE*
>Syntrophomonas_wolfei_subsp.metylbutyratica_JCM14075T_03037_Tyrosine_recombinase_XerD
VKSKPGELTRLLSEFFTEYLPTTKGLSANTITAYQYAFRLLFGYLDDVKGIMPTKVSFETLSNGVIDEFLVHLEQERQCTAKTRNVRRSAIIAFAKYAAKKSFSASLPFYSEAITSAKKKEPKNNIIKYFTKEEIQLLLKLPDTSKAIGQRDVAMMAALYATGARAQELCDITLKDITLATPSFPTKVRLTGKGNKSRIITIPMSCTAIITEYLRVRKYDLNNKSTYDRHLFSSQANEQMSISCVEEVVRKYVVRA
KELYPKLFRQNDYTPHSFRHSIAVHMLEAGESLIAIKAFLGHASISSTVVYAKLTPELANKYLDELGKPLENTMMQSIPQSLPNAMPFLYR*
>Syntrophomonas_wolfei_subsp.metylbutyratica_JCM14075T_03038_Transposase
MNVAEGWNMYLEIQDLKKMHFNVSQISRRLDLSRTTVYKYLDMPPEEMEQILENRKTRTKKLDKHRELIKSWLIQYQDMTSAQVLDWLQERKLDGGVSEGTVRNYVRQLREECVIPQVKYVRQYEAVDELPAGQQMQMDFGQIALRSSIARMVTLYFATFVLAHSRHKYVEWLDRPFTTRDLINVHENAFEYYGGMTKEIVYDQDHLILVSENGGDLLLTKEFGEYQKTRKFKIYMCRKADP
ETKGKVENVVKYVKRNFAKNRTFHNLEKLNEDCLAWLERTGNGKVHNTTKKIPVEVFALEKQHLIPVPHKINISTTKIITRVVRKDNTIVYKGNRYALPLGTYAPERRVVVKEDEGILIIANLDDELEIIRYPVCLEKGRLIKSSDCRREKDKGIDTYLQEVAQILNDSQQAYDFLGLVRQEKPRYIRDQLKIIKDHSLDKSPEVISKALNYCLKNRLYGATDFVDSLNHFNHFHNVAEEKLSIKNDIQPLY
EVDRSVLKTSVEVRDINVYIQELQGVRECQQNLNNSKTI*
>Syntrophomonas_wolfei_subsp.metylbutyratica_JCM14075T_03039_Chromosomal_replication_initiator_protein_DnaA
VSAELEQLKNDMKRLKITEAAAVVEERLLEAQVTEPSYQVFLTNLVQHEIKKREEKQLGRLYKLAAFPEYKPLDNFDLSEQQSLSKKQLKQLQELIWLEQAYNLILLGPPGVGKTCLSIGLGIEAISRGYRVTFVQMDELIRLLKTEEISRNSRAKVKRMVGSDLVIIDDLMFMAIDRHEANLFFQLINKLYGQTSIIITSNKGPEDWGDILGDPAITTAILDRLIHKSEVIHLSGDSYRLKHRQTIFGNN*
>Syntrophomonas_wolfei_subsp.metylbutyratica_JCM14075T_03040_hypothetical_protein
MLVETTVKQGSFYNVLYQMIPDNHILKRIDSAIDLSFVNELLADRYCKNFGRPAKEPEMMLRIQILKYLYNLSDEQLIQDLSVNLAYKWDVRGIGIFTAPPGMGKAYALRCFAKDLNPNLYQMVYTFLSTVSVSEFYRQFCTMLGIDAGFRKTDMFKAIQDTSIMPCYYCTQVFTNKPYWYHSAAIGDYCSGIPPLTARLIVLTSG*
>Syntrophomonas_wolfei_subsp.metylbutyratica_JCM14075T_03041_hypothetical_protein
MIEITTSWEEKGRVKGIAEGIAEGQASILLRQLKKKFGHMPIEMENRVMTMPIEKLQNLAEAIFDLGTLNEVDNFINK*
>Syntrophomonas_wolfei_subsp.metylbutyratica_JCM14075T_03042_Transposase
MIKYREILRLHSQDVSGRSIASSCQCSRNTVSKVLERAEGCGITWPFEKDMADADLQKLLFPEKSIKSSRKLPDCANIHKEMAKSGVTLSLLWHEYCETCRLSGEIPLMYSQFCRYYREYANKTKATMRIKRKPGEMMELDWAGKTAFLKDNITGEPIPAPIFVAVLSCSQYAYVEAFLNMNMESWINAHIHAFNYFGGVTRILVPDNLKTGVEQVSKPDPKINKTYHEMAEHYGTAVIPARVRHPKD
KPHAEGTVGIISTWIIASLRHQQFFSLRELNGAIQEKLAEFNEKPFQKKPGSRKSAFLEEEKALLLPLPASPYELATWSKASVQYDYHIYVDKNQYSVPYEYIRHQVDVRLTSKIVEVFYHNHRIASHLRVLGKEGQVITVPEHMPEKHQKYLEINGDFFRKWAADVGANTVIVVNAILTRHKVEKQGYRACTSLMKLADTYSLSRLEEACHRALSYTPSPSFKTIQTILKTGQDKMSVKESRPVTRSNTET
HGFVRGADYYGGKEHD*
>Syntrophomonas_wolfei_subsp.metylbutyratica_JCM14075T_03043_YcfA-like_protein
MNPKELMQILAQDGWKVVRIQGSHHIHKHPAKSGTIPVALHNKDMAPGTLNKILKIAGLK*
>Syntrophomonas_wolfei_subsp.metylbutyratica_JCM14075T_03044_hypothetical_protein
MIYIYPAIFSHCEVEERGTVYTVEIPDIPGCITEGESIQEAMAMGREALAGCILAIKQDNEEVPAPSEYRDITTESPDNFVTLLDVDLNEYLRKRETKSIIKTVSLPQWLAIMAEESGISYSQALQQALKQHLGIQD*
>Syntrophomonas_wolfei_subsp.metylbutyratica_JCM14075T_03045_Putative_zinc_ribbon_domain_protein
MKICIACGMPMNKPADFAMGDESKDYCLYCCNPDGSMHSYEEKLNGLTAFIIRTQGLDEQAACSAAQSMMAKLPAWKDRNNQDA*
>Syntrophomonas_wolfei_subsp.metylbutyratica_JCM14075T_03046_hypothetical_protein
LSKKEKLMARLLSKPKDFTFEELTALLGYFDYEQTKLGKTSGSRVVFTNGDGDYIRLHKPHPRNILKPYQVEDVITALQERGLL*
>Syntrophomonas_wolfei_subsp.metylbutyratica_JCM14075T_03047_putative_protein_encoded_in_hypervariable_junctions_of_pilus_gene_clusters
MSNYIHYRDYTGSIEFSEEDAVFHGRVIGIKNMLSFEGDSVKTLTEDFHNAVDEYLEFCENSGKQPEKPFKGSFNVRIQPELHRKAALAASARGVSLNAFVEDAIRHTVD*
>Syntrophomonas_wolfei_subsp.metylbutyratica_JCM14075T_03049_Group_II_intron-encoded_protein_LtrA
MDKTKSYEISKHVVMEAYQRVKANKGAAGIDDESLEAFETNLRNNLYKIWNRMSSGSYFPPPVKAVEIPKKTGGKRILGVPTVADRVAQMVAKIYFEPQVEPYFHPDSYGYRPGKSATDALAVTRQRCWRYDWVLEFDIKGLFDNIDHDLLIKAVRKHTDNPWVILYIQRWLKAPFQMPDGTVKERTKGTPQGGVISPVLANLFLHYAFDMWMARNHSDKPFARYADDAVTHCRSKENAEKL
HDSLKERFAECGLELHPTKTRIVYCKDDDRREEYPETKFDFLGYTFRPRRSKNKYGKYFVNFTPAVSNKAKKAMRQTIHDWRMHLKPDKTIEDLSHMFNPVIRGWINYYGRFYKSELYSVLRHMDWALVRWVQRKYKKFATHKRRATYWLGQIARREPKLFAHWQMGIRPAAG*
>Syntrophomonas_wolfei_subsp.metylbutyratica_JCM14075T_03051_hypothetical_protein
LSTEKIAELKEIPYGTNPNIKMGDLSKEDEKLAREKILEHLTLKPGQSFASSSELVYEDSLSYWVVRGVLTITNSDGTITTENVEASAFNGGSSEQAEYGWHPAIILSIDHEKRFN*
>Syntrophomonas_wolfei_subsp.metylbutyratica_JCM14075T_03052_Succinyl-CoA:coenzyme_A_transferase
MDYSMEYKRKLLPVEEAMKVVKSGDLVQYGEFVMNSSYLDAALAQRVDELENVNIRTTTCPFLPKTVLADPGRKHFIYNDYHMSAASRKLHDKDLCNYVPLTYHEGPSFYERDYVAADVALIKVAPMDKHGFFNLGTALSLTPLFCDTAKTVIVEVNENVPICLGGCRESIHISEVDYIVEGDNQPMIQLPELPISDTDKKIAAIVLEEIEDGACLQLGIGAMPNAVGAMIAQSDLKDLGVHTEML
VDSFVDMYEAGRITGRRKQLDKCKMAYTFAMGTNKLYNFLDGNPACAIYPVDYTNDPFIIGRNDKVLGINNAIEVDLYGQVASESSGTRHISGTGGQLDFIIGSYYSKGGKGLICLTSTFTDKEGRLQSRIRPTLTPGAVVTVPRSINFYVVTEYGITMLKAKSTWQRAEALINLAHPNLRDELIKEAEKMRIWVRSNKLV*
>Syntrophomonas_wolfei_subsp.metylbutyratica_JCM14075T_03053_hypothetical_protein
MQMVCAQMGCSLIHSPPFQANTRGKIERFFRTVRLRFLSQINPAEIKDIDELNLLYWQWLEEDYHRKVHSELKMSPLDFFMAQSERIKIFPNPALLDEYFLLRVKRKVKHYHWITSCMKQMSTWLIPG*
>Syntrophomonas_wolfei_subsp.metylbutyratica_JCM14075T_03057_Transposase_IS116/IS110/IS902_family_protein
LNRIQKVLEGANIKLSSVVSEIDGKSSRDMLTMLAAGCNDAKAMANKARRQMRKKIPQLEEALHGFMGEHQRLMLDSMLKHIDFLESHILLLDKEIHEKMEPVSEQITLIDSIPGIGERSAQVIIAEIGTNMNQFPSAAHLASWAGICPGNNESAGKRKSGKTRKGSDILKATLIECAKVAGHLKNTYFSAQYSRIAARRGKNRATVAVAHTILTIVYQMLKADVLYQELGPDFFEQRRKNEIVKKSVKR
LEAFGYTVTIDQQIGISTA*
>Syntrophomonas_wolfei_subsp.metylbutyratica_JCM14075T_03058_putative_secretion_ATPase,_PEP-CTERM_locus_subfamily
LLGYGQPLIRSKLALNVNNPLRQRIVVKHMMSNMQKDELGGYLKTRLAFGGTHEELFTAPSMEAIYGVTNGVPRLVNSLATNCLLYACEKKQRQVDEETVYQAQSELNF*
>Syntrophomonas_wolfei_subsp.metylbutyratica_JCM14075T_03059_Aminoglycoside_3'-phosphotransferase
VGKWGMLAVDDLVVPHGEYVLSYIMVENVQFSGFIDCGRLGVADRYQDIALATRDIAEELGGEWADRFLVLYGIAAPDSQRIAFYRLLDEFF*
>Syntrophomonas_wolfei_subsp.metylbutyratica_JCM14075T_03060_DMSO_reductase_iron-sulfur_subunit
MVCSLVHENACSPALSRIRIVRYRREALNVPVTCAFCEKPPCLDVCPVGAITKNHETGDVSIRAELCIGCRQCAQACPFGHMNFNAAKGIAFKCDLCGGDPQCVKFCWTGAIEYVDSEIAVDIKRSGFADSVLQQHNK*
>Syntrophomonas_wolfei_subsp.metylbutyratica_JCM14075T_03062_hypothetical_protein
MNLYCISQVYILLCFSQPEYCVITFSKFYPGKIQYVYGDYPVKVLISSAAYVLVAKHATGIIAAASLHVAQVANLHFYIHQVIVF*
>Syntrophomonas_wolfei_subsp.metylbutyratica_JCM14075T_03064_hypothetical_protein
LAKEDPAIGKAKRVLEQMASNPRERELYELRRKAILDRNSALYEAKMEGIAEGIAEGKVEGQASILLRQLKKKFGYLPVEMENRVMTMPTEKLQNLAEEIFDLGTLDEVDNFINR*
>Syntrophomonas_wolfei_subsp.metylbutyratica_JCM14075T_03065_hypothetical_protein
MGSFNKGRCHKGHPIPGQAMRHLVRIPMGLCHLHAMRADDSPADSHRPHRSRSASLNPATEGLDAFTAERPDANPTPSPVTGKNVDERHGGAIMLDVDIDERLGKGVENLI*
>Syntrophomonas_wolfei_subsp.metylbutyratica_JCM14075T_03066_ferrous_iron_transport_protein_B
VATLLTWFLQTFDAKLNVVTDSAKSLLAMIGTAIAPALNPVGFGDWRIATALITGFIAKESVVPPLQILLGGGAPSSLFTVRRSLTFLSFTLLYTPCVAAVSAVRRELGSGLRALGVVIAQTAVAWIVAFAVYQLLGIWL*
>Syntrophomonas_wolfei_subsp.metylbutyratica_JCM14075T_03067_putative_FAD-linked_oxidoreductase
LVTRGRVTGGAGVCDALKGGIVLDLSTLDEIVEIDDPSIQVIVRPGVIHAQLNQEVSKYKLFFPPDPGSSQMCTIVGMVANNASGLRAVKYGCTEQYILGMEVVLPNGEVITTGGVKDRCIKNVSGLNLTKLMVGSERVLGILTRSRLR*
>Syntrophomonas_wolfei_subsp.metylbutyratica_JCM14075T_03068_hypothetical_protein
VTFSEAFERLNTICNHTFSDDFKEKIDLLEKYRNRLTHAEISIDDQDIILLFDNLLDELDMYLFSNIGVATACFLRRVLRIGRAGVHQRPRQHRVGSLWSQSGYERGLQGDKFKDHVVNNGVHFFRHKKHGI*
>Syntrophomonas_wolfei_subsp.metylbutyratica_JCM14075T_03069_hypothetical_protein
LKLVELIENLTIELQDGPVAREISGIAYDSRRGQSGHICL*
>Syntrophomonas_wolfei_subsp.metylbutyratica_JCM14075T_03071_hypothetical_protein
MSRERLTLRVRLVFHPTGVTIKVASYGREIGAVPLEPRDKPLVLPMRSALSGTTDAPCGRY*
